# Predictive, Data‐Driven Design of Red‐Light Photoredox Catalysts for C─Heteroatom Bond Formation

**DOI:** 10.1002/anie.202526086

**Published:** 2026-01-19

**Authors:** Amir Gizatullin, Tingting Yuan, Sascha Grotjahn, Luigi Cavallo, Burkhard König, Chen Zhu, Magnus Rueping

**Affiliations:** ^1^ KAUST Catalysis Center (KCC) King Abdullah University of Science and Technology (KAUST) Thuwal 23955‐6900 Saudi Arabia; ^2^ Ningbo Institute of Digital Twin Eastern Institute of Technology Ningbo, Ningbo 315200 China; ^3^ Fakultät für Chemie und Pharmazie Universität Regensburg Regensburg 93053 Germany

**Keywords:** C─heteroatom bonds, Cyanoarenes, Nickel, Photocatalysts, Red light

## Abstract

Photocatalysis is a powerful tool for the synthesis of organic molecules, yet its widespread application is hindered by the dependence on high‐energy light sources and expensive metal‐based catalysts, which can limit scalability and environmental sustainability. In this study, we present a modular design strategy for organic dyes engineered for efficient red‐light absorption, enabling photocatalytic reactions under low‐energy irradiation. Our findings establish a clear relationship between the oxidation potential of the photocatalyst and the nature of its donor moiety, as well as between the reduction potential and the electronic characteristics of its core structure. Moreover, we demonstrate that the *E*_0‐0_ energy of a photocatalyst can be predicted via multivariate linear regression using the donor's oxidation potential and the core's reduction potential as descriptors. Utilizing this strategy, we synthesized red‐light‐absorbing photocatalysts that efficiently promote C─heteroatom cross‐coupling reactions under mild conditions. This approach overcomes the limitations of blue‐light photocatalysis by offering broad substrate compatibility, including π‐conjugated aryl bromides and photolabile functional groups, while minimizing undesirable hydrodehalogenation. By reducing reliance on precious metals and improving energy efficiency, our approach provides a scalable alternative to traditional photocatalysis and advances the development of metal‐free photocatalysts for sustainable chemistry.

## Graphical abstract

**From redox numbers to red light**: Donor oxidation and core reduction potentials predict *E*_0‐0_ in cyanoarene dyes, guiding selection of an efficient red‐light catalyst, 4MeODPATPN (PC13). Under 620 nm light, PC13 facilitates Ni‐catalyzed C─heteroatom cross‐couplings (C─N, C─O, C─S) with high functional‐group tolerance and reduced hydrodehalogenation.

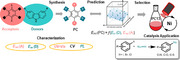

## Introduction

**Figure 1:**
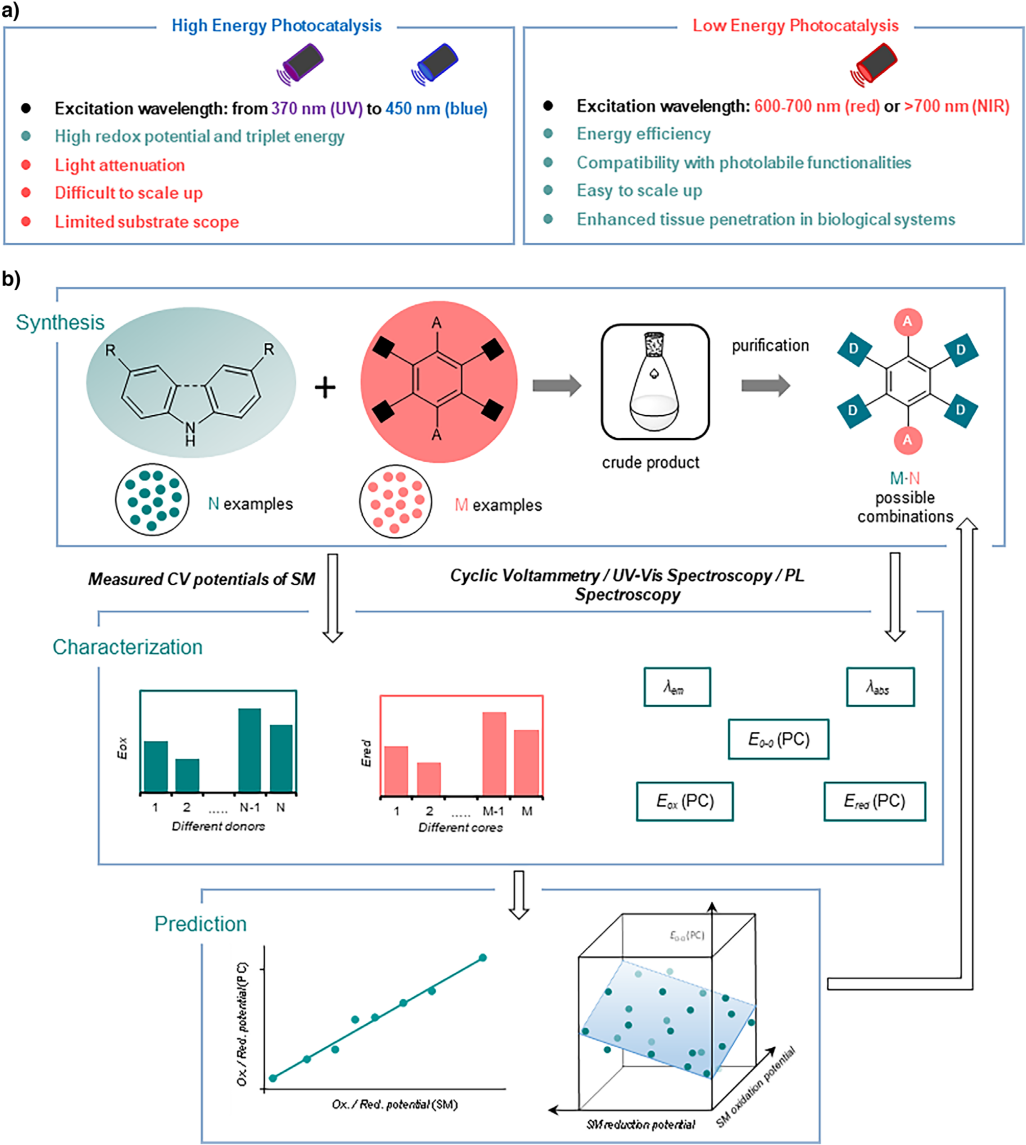
Overview of various light wavelengths and photocatalysts. a) Advantages and disadvantages of high and low energy photocatalysis. b) Our modular design strategy aimed at developing red‐light TADF photocatalysts.

In recent years, photocatalysis has emerged as a powerful platform for constructing a wide range of organic molecules, commonly employing iridium (Ir) and ruthenium (Ru)‐based complexes as photocatalysts. Despite their advantages, the need for precious metals and multi‐step syntheses has stimulated the use of organic dyes, such as inexpensive phenoxazines, acridinium salts, and thermally activated delayed fluorescence (TADF)‐photocatalysts (TADF‐PCs).^[^ ^1^, ^2^, ^3^, ^4^, ^5^, ^6^, ^7^, ^8^, ^9^ ^]^ However, existing photocatalysts that primarily utilize high‐energy light, such as near‐UV and blue light, may have significant limitations.^[^ ^10^ ^]^ One major challenge is the scalability of reactions in batch reactors, constrained by poor light penetration as described by the Beer–Lambert law.^[^ ^11^ ^]^ While flow chemistry improves light exposure, challenges remain with the compatibility of photocatalysts and reagents within flow reactors, as well as the costs associated with commercial reactors.^[^ ^10^, ^12^, ^13^ ^]^ Moreover, the use of high‐energy light in photoredox catalysis can lead to photosensitized substrate decomposition or side reactions.^[^ ^14^, ^15^ ^]^ Red‐light (RL) and near‐infrared (NIR) catalysis offer promising alternatives to traditional photocatalysis, presenting advantages not achievable through conventional methods (Figure 1a).^[^ ^16^, ^17^, ^18^, ^19^, ^20^ ^]^ A primary advantage of RL/NIR catalysis is its enhanced energy efficiency, which could lead to reduced operating costs and a smaller carbon footprint. Due to their lower triplet energy or HOMO–LUMO gap, these systems possess narrower redox ranges, which help to fine‐tune reactivity and facilitate the selective activation of specific substrates under controlled conditions. Additionally, RL/NIR catalysis offers improved compatibility with photolabile functionalities, which are prone to unwanted photodecomposition reactions in conventional photocatalytic processes. This methodology extends the range of substrates, potentially facilitating the synthesis of novel classes of compounds. Furthermore, the superior light penetration associated with RL/NIR catalysis simplifies the scale‐up of reactions (Figure 1a). In this context, Rovis and other researchers have made significant contributions to the development of Os/Ir/Ru‐based complexes, which can be directly activated from the ground state singlet (S_0_) to the excited triplet state (T_1_) under NIR or RL irradiation via strong spin−orbit coupling (SOC).^[^ ^21^, ^22^, ^23^ ^]^ Recent work has also shown that direct red‐light excitation of a bifunctional Ni(0) precatalyst enables C–N and C–O cross‐couplings, albeit at elevated temperature.^[^ ^24^ ^]^

Although appealing, these RL/NIR photocatalysts are typically based on precious metals. Alternatively, organic RL/NIR photoredox catalysts such as methylene blue, dimethoxyquinacridinium, bridged eosin Y, porphyrins, cyanine dyes, squaraines, and aza‐dipyrromethenes have been investigated.^[^ ^24^, ^25^, ^26^, ^27^, ^28^, ^29^, ^30^ ^]^ However, the structural diversity of this class of compounds is relatively limited due to the multi‐step synthesis required for their derivatization.

Thus, the development of inexpensive and tunable organic RL/NIR photocatalysts would expand the scope and application of this new field of RL/NIR catalysis. TADF‐PCs, composed of commercially available acceptor cores and carbazole (Cz)/diphenylamine (DPA) donors, have demonstrated excellent performance in photocatalysis.^[^ ^31^, ^32^, ^33^, ^34^, ^35^, ^36^, ^37^ ^]^ Furthermore, they are highly modular and straightforward to synthesize, making them ideal candidates for the development of RL/NIR photocatalysts.

## Results and Discussion

**Figure 2:**
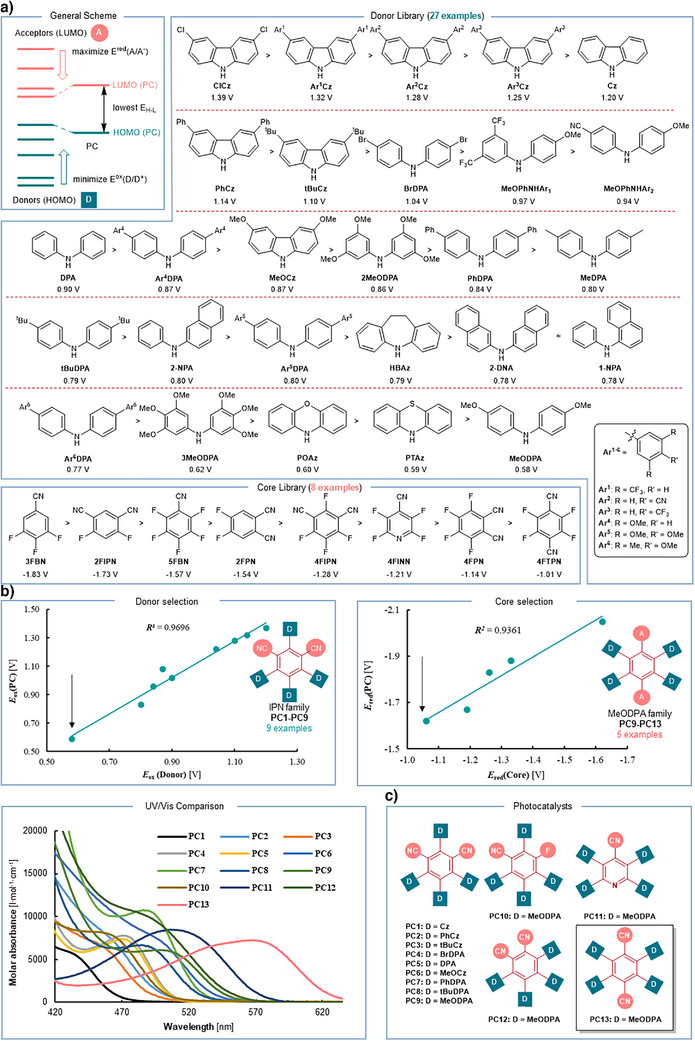
Modular design of red‐light‐absorbing TDAF dyes. a) General concept and the studied library of cores and donors. b) Core and donor selection. c) The list of synthesized photocatalysts. The donor and acceptor building blocks employed in the synthesis of the studied photocatalysts are highlighted in their respective colors.

To develop a red‐light‐absorbing photocatalyst, it is crucial to narrow the highest occupied molecular orbital (HOMO) and lowest unoccupied molecular orbital (LUMO) gap of the molecule (Figure 2a), which involves elevating the HOMO energy while minimizing the LUMO energy. Previous studies have shown that in thermally activated delayed fluorescence photocatalysts, the HOMO and LUMO energy levels are predominantly determined by the donor groups and the core units, respectively.^[^ ^31^ ^]^ The range of assessed donors and cores has been limited, and it remained unclear whether this relationship can be quantitatively expressed.

These energy levels can in principle be assessed by determining the oxidation and reduction potentials of the donor and acceptor moieties, which can be measured using cyclic voltammetry (CV). To test this hypothesis, we first constructed a library of 27 donor molecules, focusing on diarylamines and carbazoles with a variety of substituents, including methoxy, alkyl, halide, aryl groups, and others. Most of these donors were commercially available; however, we also synthesized 10 additional diarylamines and diarylcarbazoles (2MeODPA, 3MeODPA, Ar^1–3^Cz, MeOPhNHAr^1^ **^–^** ^2^, Ar^4–6^DPA) with diverse substituents. The oxidation potentials of this donor library were then measured via CV (Figure 2a, Table S2). In parallel, we selected eight polyfluorocyanoarenes as core units and evaluated their reduction potentials using CV (Figure 2a, Table S3).

To validate the hypothesis correlating the donor's oxidation potential with the photocatalyst's HOMO energy, we synthesized and characterized nine photocatalysts (**PC1**‐**PC9**) by fixing the 4FIPN as the core and varying the donor groups (Figure 2c). A clear correlation was observed between the oxidation potentials of the photocatalysts and their respective donors (Figure 2b, left). From this analysis, 4‐methoxydiphenylamine (MeODPA) was identified as the most promising donor candidate. Subsequently, we synthesized and characterized five additional photocatalysts (**PC9‐PC13**) by fixing MeODPA as the donor and varying the core units (Figure 2c). Again, a clear relationship was observed between the reduction potentials of the cores and the resulting photocatalysts (Figure 2b, right). In addition, we also tested hydrogen‐substituted cyanoarene cores; however, the correlation was notably weaker. In contrast, fluorinated precursors offer better control over substitution patterns and enable systematic variation in both the number and position of donor groups (Figures S12–S15). Based on these findings, we predicted that the photocatalyst 4MeODPATPN would exhibit the most significant redshift in its absorption spectrum. UV–vis absorption spectra of all thirteen synthesized photocatalysts (Figure S4) confirmed this prediction (Figure 2b, bottom), with 4MeODPATPN (**PC13**) showing absorption in the 600–650 nm region. With the established correlation between the HOMO/LUMO energy levels of the photocatalysts and their respective donor and core components, we further hypothesized that the ***E*_0‐0_** energy of a photocatalyst might also correlate with the combination of the donor's oxidation potential and the core's reduction potential. To test this hypothesis, we applied multivariate linear regression to the ***E*_0‐0_** energy of the photocatalysts (**PC1‐PC13**) as a function of the oxidation potential of the donor and the reduction potential of the core.

**Figure 3:**
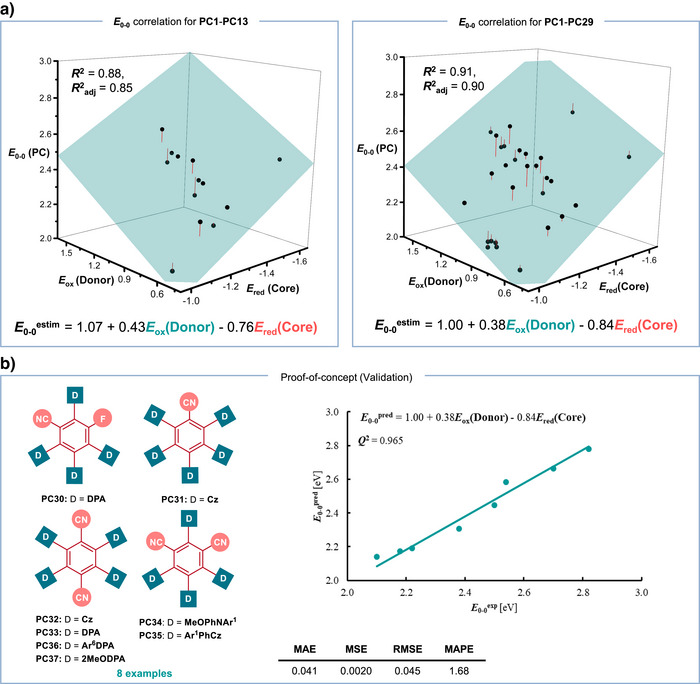
Establishing photocatalyst–starting material properties relationship. a) ***E*_0‐0_** correlation of the PCs with redox potentials of the respective cores and donors b) Prediction of ***E*_0‐0_** energy.

This analysis yielded a strong correlation (***R* ^2^** = 0.88; Figures 3a and S16). The correlation matrix (Table S8) confirmed that a trivariate model, considering both donor and core contributions, provided a better fit than models using either factor alone. To further expand the chemical space and improve the even distribution of data, we synthesized and characterized an additional 16 photocatalysts (Figure S5). These new photocatalysts included previously studied cores paired with simple donors like diphenylamines and carbazoles (**PC14‐PC23**), as well as novel donors. Specifically, we tested two methoxy‐substituted diphenylamines for the TPN‐family (**PC24‐PC25**), electron‐deficient carbazoles (**PC26‐PC28**), and a diarylamine (MeOPhNHAr^2^) containing one electron‐rich and one electron‐deficient aryl group (**PC29**) for the IPN core. Analysis of all 29 compounds further strengthened the correlation (Figure S17), with a resulting ***R* ^2^** of 0.91, supporting a predictive equation similar to the one previously obtained (Figure 3a). This supports the view that ***E*_0‐0_** in these donor–acceptor cyanoarenes is largely governed by the donor oxidation potential and core reduction potential, which serve as experimental proxies for the underlying HOMO/LUMO energetics. Notably, the regression coefficients (eV·V^−1^) quantify the sensitivity of the optical gap to changes in the electrochemical donor and core descriptors. Accordingly, gap narrowing (lower ***E*_0‐0_**) is driven mainly by cores with more positive *E*_red_ (−0.84*E*_red_) and secondarily by donors with lower *E*_ox_ (+0.38*E*_ox_) indicating a stronger acceptor contribution within the explored chemical space. Within the explored fluorinated cyanoarene TADF scaffold space (diarylamine/carbazole donors), the model provides reliable ***E*_0‐0_** estimates. Outside this domain—particularly for hydrogen‐substituted cores—predictive performance diminishes (see SI, Section 2.1) (Table 1).

**Table 1:** Electrochemical and photophysical properties of the photocatalysts.

Name	***E*** (Pc^●+^/Pc) (V vs SCE)	***E*** (Pc/Pc^●−^) (V vs SCE)	***E*** (Pc^●+^/Pc^*^) (V vs SCE)	***E*** (Pc^*^/Pc^●−^) (V vs SCE)	***E***_0‐0_ (eV)	***λ***_em_ (nm)	***E***_em_ (eV)	***λ***_abs_ (nm)	***E***_abs_ (eV)	Solvent
**PC1**	1.37	−1.22	−1.26	1.41	2.63	541	2.29	428	2.90	MeCN
**PC2**	1.32	−1.17	−1.13	1.28	2.45	588	2.11	462	2.68	MeCN
**PC3**	1.28	−1.32	−1.23	1.19	2.51	570	2.18	440	2.82	MeCN
**PC4**	1.22	−1.43	−1.28	1.07	2.50	526	2.36	470	2.64	MeCN
**PC5**	1.02	−1.64	−1.48	0.86	2.50	529	2.34	470	2.64	MeCN
**PC6**	1.08	−1.38	−1.23	0.93	2.31	611	2.03	506	2.45	DCM
**PC7**	0.96	−1.56	−1.44	0.84	2.40	554	2.24	486	2.55	MeCN
**PC8**	0.83	−1.67	−1.56	0.72	2.39	564	2.20	485	2.56	MeCN
**PC9**	0.59	−1.88	−1.71	0.42	2.30	580	2.14	498	2.49	DCM
**PC10**	0.58	−2.05	−1.92	0.45	2.50	538	2.30	462	2.68	DCM
**PC11**	0.43	−1.83	−1.79	0.39	2.22	598	2.07	512	2.42	DCM
**PC12**	0.72	−1.67	−1.54	0.59	2.26	633	1.96	485	2.56	DCM
**PC13**	0.61	−1.62	−1.43	0.42	2.04	643	1.93	569	2.18	DCM
**PC13‘**	0.76	−1.41	−1.33	0.68	2.09	632	1.96	557	2.23	DMA
**PC14**	1.18	−1.61	−1.5	1.07	2.68	542	2.29	409	3.03	MeCN
**PC15**	1.24	−1.52	−1.17	0.89	2.41	552	2.25	477	2.60	DCM
**PC16**	0.8	−1.64	−1.57	0.73	2.37	553	2.24	485	2.56	MeCN
**PC17**	1.47	−1.14	−1.12	1.45	2.55	577	2.15	436	2.84	MeCN
**PC18**	1.27	−1.24	−1.17	1.2	2.44	611	2.03	447	2.77	MeCN
**PC19**	1.11	−1.49	−1.36	0.98	2.47	575	2.16	434	2.86	MeCN
**PC20**	1.34	−1.20	−0.94	1.08	2.28	578	2.15	513	2.42	DCM
**PC21**	1.03	−1.08	−1.06	1.01	2.13	640	1.94	520	2.38	DCM
**PC22**	0.85	−1.41	−1.28	0.72	2.13	617	2.01	554	2.24	DCM
**PC23**	0.80	−1.67	−1.33	0.46	2.13	612	2.03	557	2.23	DCM
**PC24**	0.91	−1.36	−1.21	0.76	2.12	617	2.01	546	2.27	DCM
**PC25**	0.81	−1.36	−1.30	0.75	2.11	625	1.98	553	2.24	DCM
**PC26**	1.60	−1.11	−0.97	1.46	2.57	530	2.34	458	2.71	DCM
**PC27**	1.50	−1.11	−1.01	1.40	2.51	570	2.18	450	2.76	MeCN
**PC28**	1.43	−1.10	−1.07	1.40	2.50	543	2.28	470	2.64	DCM
**PC29**	1.10	−1.39	−1.35	1.06	2.45	565	2.19	476	2.61	MeCN
**PC30**	0.99	−1.78	−1.71	0.92	2.70	492	2.52	433	2.86	MeCN
**PC31**	1.41	−1.52	−1.41	1.30	2.82	513	2.42	388	3.20	MeCN
**PC32**	1.15	−1.05	−1.23	1.33	2.38	520	2.22	486	2.55	DCM
**PC33**	0.94	−1.50	−1.28	0.72	2.22	585	2.12	530	2.34	DCM
**PC34**	1.18	−1.26	−1.32	1.24	2.50	565	2.19	459	2.70	MeCN
**PC35**	1.60	−1.08	−0.94	1.46	2.54	555	2.23	465	2.67	MeCN
**PC36**	0.81	−1.43	−1.29	0.67	2.10	624	1.99	555	2.23	DCM
**PC37**	1.00	−1.46	−1.18	0.72	2.18	584	2.12	517	2.40	DCM

**Figure 4:**
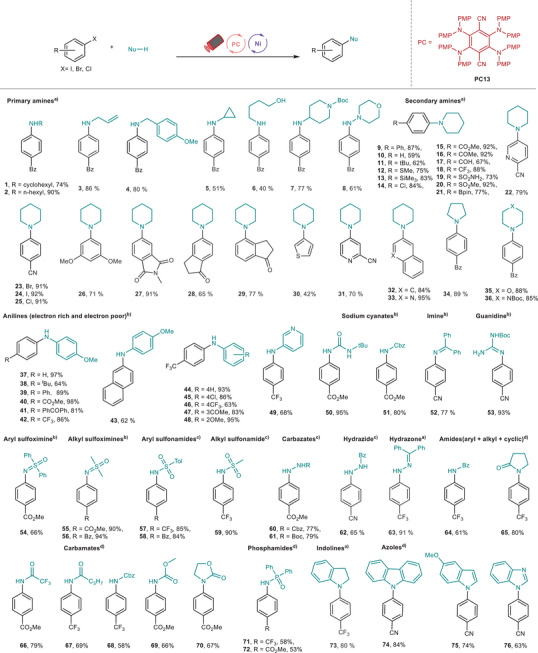
The scope for C–N cross‐coupling reactions. All yields are isolated yields. See Supporting Information for full reaction conditions for each substrate. 3 mol% 4MeODPATPN photocatalyst, 10 mol% NiCl_2_·glyme, 1 mL DMA, 620 nm LEDs, 48 h. ^a)^2.0 equiv. DABCO. ^b)^2.0 equiv. DABCO, 0.5 equiv. Et_3_N. ^c)^1.0 equiv. cyclohexylamine, 1.0 equiv. TMG. ^d)^10 mol% dtbbpy, 1.5 equiv. BTMG, 0.5 equiv. Et_3_N. DABCO, 1,4‐diazabicyclo[2.2.2]octane; Et_3_N, triethylamine; TMG, 1,1,3,3‐tetramethylguanidine; BTMG, 2‐*ter*t‐butyl‐1,1,3,3‐tetramethylguanidine; dtbbpy, 4,4′‐di‐*tert*‐butyl‐2,2′‐bipyridine; PMP, *p*‐methoxyphenyl group, Bz, benzoyl; Bpin, boronic acid pinacol ester; *^t^* Bu, *tert*‐butyl, Boc, *tert*‐butylcarbonyl; Cbz, carbobenzyloxy.

To validate this computational model, we synthesized and characterized eight (Figure S6) additional photocatalysts (**PC30–PC37**) and predicted their ***E*_0‐0_** energies using the derived multivariate linear regression equation. The predictions were highly accurate, yielding a ***Q* ^2^** value of 0.965, a mean absolute error (MAE) of 0.04, and a mean absolute percentage error (MAPE) of 1.68% (Figure 3b). Thus, we developed a straightforward approach for predicting the ***E*_0‐0_** energy of TADF photocatalysts using only the measured oxidation and reduction potentials of their constituent donor and core units. We conducted additional validation on previously unexplored cores and obtained acceptable predictions for the resulting carbazole photocatalysts **PC38**–**PC41** (Table S12). Furthermore, based on this analysis, we confirmed that **PC13** remained the most redshifted photocatalyst among all 37 synthesized (see Supporting Information, Section 2.2), as even more conjugated diphenylamines such as Ar^4–6^DPA or 2MeODPA did not result in further redshifting (Table 1). Finally, we evaluated the catalytic activity of our best candidate, **PC13**, under red‐light irradiation. Motivated by our ongoing efforts in catalytic C─heteroatom bond formation and the widespread use of heteroatom‐containing products, preliminary studies were conducted using a photoredox and nickel dual‐catalyzed cross‐coupling of aryl bromides and piperidine.^[^ ^38^, ^39^, ^40^, ^41^, ^42^, ^43^, ^44^, ^45^, ^46^, ^47^, ^48^ ^]^ After thorough optimization (see Supporting Information, Section 3), the reaction conditions were finalized as follows: **PC13** (3 mol%) as the photocatalyst, NiCl_2_·glyme (10 mol%) as the nickel catalyst, and 1,4‐diazabicyclo[2.2.2]octane (DABCO) (2 equiv) as the base, in dimethylacetamide (DMA) (1 mL), with irradiation at 620 nm. Under these conditions, the desired product was obtained in 89% yield (Table S13). Additionally, photostability and mechanistic experiments (see Supporting Information, Section 5) confirmed that **PC13** remained stable under the reaction conditions. This is significant because compounds in the 4CzIPN family are known to undergo photosubstitution, which can generate catalytically active species with blueshifted absorption, potentially affecting red‐light absorption efficiency.^[^ ^49^, ^50^ ^]^ Encouraged by the excellent activity of our photocatalyst, we explored the scope of diverse N‐containing nucleophiles for the reaction.^[^ ^51^, ^52^ ^]^ As shown in Figure 4, various N‐containing nucleophiles (**1**–**76**) successfully provided the coupling products in good to nearly quantitative isolated yields, demonstrating the versatility of the present red‐light photoredox conditions. For example, aliphatic primary amines (**1**–**8**), aliphatic secondary amines (**9**–**36**), and anilines (aryl and heterocyclic) (**37**–**49**) all participated and afforded the coupling products in good to excellent yields. Notably, high chemoselectivities of C–N coupling were obtained for the amino alcohol substrate (**6**). Aryl chlorides and aryl iodides also proceeded smoothly, furnishing the coupling products in excellent yields (**24, 25**). A wide range of N‐nucleophiles, including weaker ones such as sodium cyanate (**50, 51**), imines (**52**), *N*‐Boc‐guanidine (**53**), sulfonimines (**54**–**56**), sulfonamides (**57**–**59**), carbazates (**60, 61**), hydrazide (**62**), hydrazone (**63**), benzamide (**64**), cyclic amide (**65**), alkyl amides (**66, 67**), carbamates (**68**–**70**), and phosphamides (**71, 72**), all worked efficiently, giving the desired products that are vital in the pharmaceutical industry in good to excellent yields.^[^ ^53^ ^]^ Carbamates and ureas are common scaffolds in a series of biologically active compounds.^[^ ^54^, ^55^ ^]^ Notably, urea (**50**), in situ generated from sodium cyanate, was successfully applied for the first time under red‐light‐mediated conditions.

**Figure 5:**
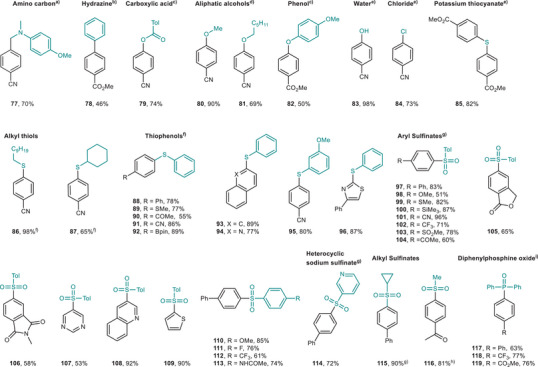
The scope for C–C, C–O, C–Cl, C–S, C–P cross‐coupling reactions. All yields are isolated yields. See Supporting Information for full reaction conditions for each substrate. 2.5–3 mol% 4MeODPATPN photocatalyst, 10 mol% NiCl_2_·glyme or 5–10 mol% NiBr_2_·glyme, 0.4–1.2 mL DMA, 620 nm LEDs, 48 h. ^a)^10 mol% dtbbpy, 1.5 equiv. BTMG. ^b)^3.6 equiv. TMG. ^c)^10 mol% dtbbpy, 1.5 equiv. BTMG, 0.5 equiv. Et_3_N. ^d)^1.5–2.0 equiv. TMG, 0.5 equiv. Et_3_N. ^e)^2.0 equiv. DABCO, 0.5 equiv. Et_3_N. ^f)^1.2 equiv. pyridine. ^g)^2.2 equiv. DABCO, 0.5 equiv. Et_3_N. ^h)^5 mol% 4MeODPATPN photocatalyst, 2.2 equiv. DABCO, 0.5 equiv. Et_3_N. ^i^1.2 equiv. Cs_2_CO_3_, 640 nm LEDs, the compounds were isolated as their corresponding oxides. DABCO, 1,4‐diazabicyclo[2.2.2]octane; Et_3_N, triethylamine; TMG, 1,1,3,3‐tetramethylguanidine; BTMG, 2‐*tert*‐butyl‐1,1,3,3‐tetramethylguanidine; dtbbpy, 4,4′‐di‐*tert*‐butyl‐2,2′‐bipyridine; Bpin, boronic acid pinacol ester; Tol, toly.

Carbamate (**51**) also showed excellent reactivity. The use of OCN^−^ (**50, 51**) to install carbamates was realized for the first time via red‐light‐mediated methods. Importantly, various heterocycles such as amides (**65**), indoline (**73**), and azoles (**74**–**76**) proved to be suitable coupling partners, delivering the desired products in good to excellent yields. Furthermore, an array of O‐, S‐, and P‐containing weak nucleophiles were investigated to assess the generality of this transformation (Figure 5). Gratifyingly, these nucleophiles, including α‐amino carbon, carboxylic acids, aliphatic alcohols, phenol, water, alkyl thiols, thiophenols, aryl sulfinates, heterocyclic sodium sulfinates, alkyl sulfinates, and diphenyl phosphine oxides, were viable, affording the corresponding products (**77**–**119**) in moderate to nearly quantitative yields. Pleasingly, hydrazine (**78**) underwent Suzuki cross‐coupling effectively, producing the corresponding products in good yield. Additionally, lithium chloride (**84**) coupled with aryl bromide smoothly, resulting in the halogen exchange product in good yield. Potassium thiocyanate (**85**), an inexpensive and readily available sulfur source, was employed for synthesizing symmetrical sulfides via this red‐light‐mediated protocol.

**Figure 6:**
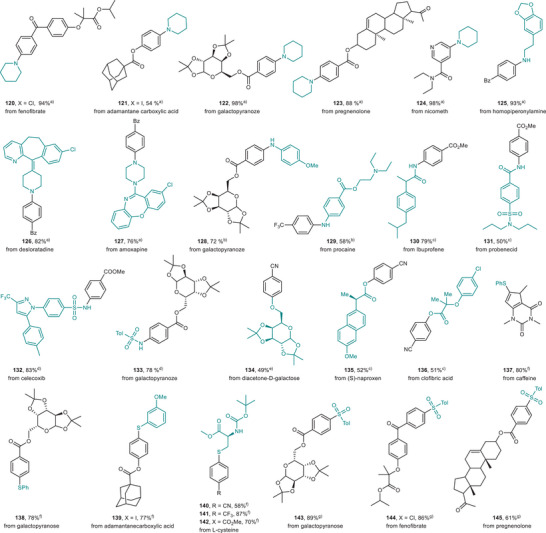
The scope for late‐stage diversification of pharmaceutical‐related molecules. All yields are isolated yields. See Supporting Information for full reaction conditions for each substrate. 2.5–3 mol% 4MeODPATPN photocatalyst, 10 mol% NiCl_2_·glyme or 5–10 mol% NiBr_2_·glyme, 0.4–3 mL DMA, 620 nm LEDs, 48 h. ^a)^2.0 equiv. DABCO. ^b)^2.0 equiv. DABCO, 0.5 equiv. Et_3_N. ^c)^10 mol% dtbbpy, 1.5 equiv. BTMG, 0.5 equiv. Et_3_N. ^d)^1.0 equiv. cyclohexylamine, 1.0 equiv. TMG. ^e)^2.0 equiv. TMG, 0.5 equiv. Et_3_N. ^f)^1.2 equiv. pyridine. ^g)^2.2 equiv. DABCO, 0.5 equiv. Et_3_N. DABCO, 1,4‐diazabicyclo[2.2.2]octane; Et_3_N, triethylamine; TMG, 1,1,3,3‐tetramethylguanidine; BTMG, 2‐*tert*‐butyl‐1,1,3,3‐tetramethylguanidine; dtbbpy, 4,4′‐di‐*tert*‐butyl‐2,2′‐bipyridine.

We then examined the potential of our red‐light‐mediated system for late‐stage diversification of pharmaceutical‐related molecules (Figure 6). The fenofibrate and pregnenolone derivatives underwent reactions smoothly, yielding the corresponding amines (**120, 123**) and sulfones (**144, 145**) in good to excellent yields. Adamantane carboxylic acid derivatives were also suitable coupling partners (**121, 139**). Both the nicometh and caffeine derivatives delivered the coupling products in excellent yields (**124, 137**). Furthermore, the galactopyranose derivative coupled with various C‐heteroatom partners, resulting in amines (**122, 128**), sulfonamide (**133**), thioether (**138**), and sulfone (**143**) in satisfactory yields. Our method was also successfully applied to the preparation of homopiperonylamine derivative (**125**), antihistamine drug desloratadine derivative (**126**), and antidepressant drug amoxapine derivative (**127**). Additionally, the protocol worked well in preparing derivatives of the anesthetic drug procaine (**129**), antigout drug probenecid (**131**), nonsteroidal anti‐inflammatory drug ibuprofen (**130**), and anti‐inflammatory drug celecoxib (**132**). The carboxylic acids derived from the anti‐inflammatory drug naproxen and fibrate drug clofibric acid also performed well, yielding the corresponding esters (**135, 136**) in good yields. Notably, the natural α‐amino acid derivative from *N*‐Boc‐L‐cysteine served as an effective coupling partner, producing the desired products (**140**–**142**) with good efficiency.

Encouraged by the success in late‐stage diversification, we briefly considered factors underlying the observed reactivity. Although base and ligand effects are pronounced, the reaction remains strictly light‐ and photocatalyst‐dependent (see Supporting Information, Sections 3 and 5). In line with this picture, excited **PC13** can undergo SET with common amine bases or S‐based nucleophiles to form the radical anion, which provides an entry point for reduction of Ni(II) to Ni(I) and maintaining the Ni(I)/Ni(III) catalytic cycle.^[^ ^51^ ^]^

**Figure 7:**
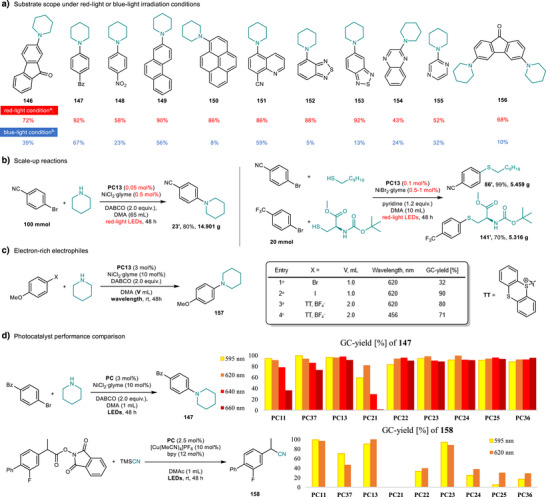
Applications. a) The scope for selected aryl bromides under red‐light or blue‐light irradiation conditions. b) Scale‐up reactions. c) Electron‐rich electrophiles. d) Photocatalyst performance comparison. ^a)^Red: 3 mol% 4MeODPATPN photocatalyst, 10 mol% NiCl_2_·glyme, 2.0 equiv. DABCO, 1 mL DMA, 620 nm LEDs, 48 h, isolated yield. ^b)^Blue: 1 mol% 4CzIPN photocatalyst, 10 mol% NiCl_2_·glyme, 2.0 equiv. DABCO, 1 mL DMA, 440 nm LEDs, 16 h, yields were determined by GC‐FID using dodecane as the internal standard.^c)^10 mol% NiCl_2_·glyme, 2 mL DMA, 2 x 456 nm Kessil lamps, rt, 24 h under Ar atmosphere. DABCO, 1,4‐diazabicyclo[2.2.2]octane; Bz, benzoyl.

To compare light sources, we evaluated amination of a panel of aryl bromides under red‐light (RL) and blue‐light (BL) irradiation (Figure 7a). Under RL, substrates that strongly absorb blue light (**146**, **147**, **149**, **150**, **154**, **155**), photosensitive substrates (**148**), and those prone to hydrodehalogenation (**146**, **147**, **151**, **155**) all delivered moderate to excellent yields. Across all cases (**146–156**), RL consistently outperformed BL. For carboxylic acids, cross‐coupling products were obtained exclusively under RL, with no product observed under BL (Supporting Information, Section 5.2), underscoring the advantage of the RL platform. To assess scalability, we conducted gram‐scale reactions up to 100 mmol using low catalyst loadings (0.05 mol% 4MeODPATPN and 0.1 mol% NiBr_2_·glyme) without loss of yield (Figure 7b). Electron‐rich aryl halides, which are challenging for low‐valent nickel catalysis due to higher oxidative‐addition barriers, were also examined: para‐methoxyphenyl iodide afforded **157** in 90% yield, whereas the corresponding bromide gave 32% under identical conditions. Aryl thianthrenium salts were evaluated in an excited‐state Ni‐catalyzed system under blue‐light irradiation and showed comparable reactivity (71%), whereas **PC13** under red‐light afforded 80% (Figure 7c).

We further benchmarked **PC11**, **PC21–PC25**, **PC36**, and **PC37** (in addition to **PC13**) in dual photoredox/Ni‐catalyzed C–N coupling (to **147**) and dual photoredox/Cu‐catalyzed cyanation of an activated ester (to **158**) (Figure 7d). Despite weak long‐wavelength absorbance, most catalysts remained active, likely due to the broad emission bandwidth of the LEDs (Supporting Information, Section 6). Yields tracked three factors: spectral overlap (set by *E_0‐0_*), ground‐ and excited‐state redox matching, and solubility. Across both benchmarks, **PC13** remains the most practical choice, combining a red‐shifted absorption profile with suitable redox potentials, good solubility in aprotic polar solvents, and straightforward synthesis from commercial precursors. These results further support the versatility of our system and its potential to address known challenges in photocatalytic cross‐coupling.

## Conclusion

In this study, we successfully employed a modular approach to design and synthesize organic dyes capable of absorbing red light. This methodology, based on the initial measurement of the redox potentials of both core and donor components, was validated through the synthesis and comprehensive characterization of distinct photocatalysts, culminating in the creation of efficient red‐light‐absorbing catalysts. Furthermore, we expanded and refined this approach by incorporating additional photocatalysts, each extensively characterized, to demonstrate that the *E*_0‐0_ energy of these photocatalysts can be quantitatively predicted using the measured redox properties of the donor and acceptor building blocks. Additionally, we developed protocols for C─heteroatom cross‐coupling reactions using dual photoredox and nickel catalysis under low‐energy light, with PC13 as the photocatalyst. These mild reaction conditions, which are free of precious transition metals, proved to be highly effective, showing broad substrate scope and compatibility with photolabile functional groups and extended π‐systems. In addition, some synthesized photocatalysts also exhibited catalytic activity under red light providing additional options for future studies where red‐absorbing catalysts are desired. We anticipate that our approach will significantly advance the development of novel organic photocatalysts optimized for targeted applications, thus enhancing their versatility and potential utility.

## Conflict of Interests

The authors declare no conflict of interest.

## Supporting information



Supporting Information

Supporting Information

## Data Availability

The data that support the findings of this study are available in the Supporting Information of this article.
