# Supplementary material for: Predictive, Data‐Driven Design of Red‐Light Photoredox Catalysts for C─Heteroatom Bond Formation
Source: Angew Chem Int Ed Engl. 2026 Jan 19;65(9):e26086. doi: 10.1002/anie.202526086 (PMC12929924; doi:10.1002/anie.202526086)
Supplement: Supplementary file 1 — Supporting Information [file ANIE-65-e26086-s002.pdf]

Predictive, Data-Driven Design of Red-Light Photoredox Catalysts for C–Heteroatom Bond Formation

Amir Gizatullin,[a†] Tingting Yuan,[a†] Sascha Grotjahn,[c] Luigi Cavallo[a],

Burkhard König*[c], Chen Zhu*[b] and Magnus Rueping*[a]

[a] M.Sc. Amir Gizatullin, Dr. Tingting Yuan, Prof. Dr. Luigi Cavallo, Prof. Dr. Magnus Rueping

KAUST Catalysis Center (KCC) King Abdullah University of Science and Technology (KAUST)

Thuwal, 23955-6900 (Saudi Arabia) E-mail: magnus.rueping@kaust.edu.sa

[b] Prof. Dr. Chen Zhu

Ningbo Institute of Digital Twin

Eastern Institute of Technology

Ningbo, 315200 (China)

E-mail: chen.zhu@eias.ac.cn

[c] Dr. Sascha Grotjahn, Prof. Dr. Burkhard König

Fakultät für Chemie und Pharmazie

Universität Regensburg

Regensburg, 93053 (Germany)

E-mail: burkhard.koenig@ur.de

[†]These authors contributed equally to this work.

Table of Contents

1. General information 1
2. Design, synthesis and photophysical measurements of photocatalysts 3
2.1. Photocatalysts design and screen 3
2.2. Synthesis of the donors and the photocatalysts 18
2.2.1. Synthesis of the donors 18
2.2.2. Synthesis of the photocatalysts 21
2.2.3. Cyclic voltammograms of the donors and cores 36
2.2.4. Photophysical data of the photocatalysts PC1-PC37 56
3. Optimization of the catalytic reaction conditions 140
4. Experimental procedures for catalytic reactions 144
4.1. General procedures 144
4.2. Scale-up reactions 147
5. Mechanistic studies 154
5.1. Radical-trapping experiments 154
5.2. Reactivity comparison of the red and blue light protocols by using carboxylic acids as a cross-coupling partner 156
5.3. Reaction progress monitoring 158
5.4. Light on-off studies 159
5.5. Decomposition pathways of the photocatalysts, isolation procedures and characterization. 159
5.5.1. Synthesis and spectroscopic data of PC1 and PC13 decompositon products 162
5.5.2. Crystal structure determination for photocatalysts 164
5.5.3. Photophysical data of the photocatalysts decomposition products 168
5.5.3. Cyclic voltammetry of organic base and substrates 176
6. Low E_{0-0} photocatalysts performance comparison 181
7. Spectroscopic data of the products 185
8. Copies of NMR spectra 227
9. Supplementary references 479

1. General information

Unless otherwise noted, all commercially available compounds were used directly without further purification. Solvents for chromatography were HPLC grade. Anhydrous and degassed *N*,*N*-Dimethylacetamide (DMA, 99.5%, AcroSeal®) used in reactions were purchased from Acros, and stored in glovebox at room temperature. Analytical thin layer chromatography (TLC) was conducted on Merck silica gel aluminium plates with F-254 indicator, visualized by irradiation with UV light. Column chromatography was carried on silica gel (particle size 0.043–0.063 mm) by using Interchim PuriFlash®430 automatic purification system.

NMR spectra (^{1}H NMR, ^{13}C NMR ^{19}F NMR and ^{31}P NMR spectra) were recorded on Bruker DRX-500 and AMX-400 instrument in $CDCl_3$, CD_2Cl_2, CD_3CN, $(CD_3)_2CO$ and $(CD_3)_2SO$. Spectra were calibrated relative to residual proton signal of the deuterated solvents: δH = 7.26 ppm and δC = 77.16 ppm for $CDCl_3$; δH = 5.32 ppm and δC = 53.84 ppm for CD_2Cl_2; δH = 1.94 ppm and δC =11.32 ppm, δC = 118.26 ppm for CD_3CN; : δH = 2.05 ppm and δC = 29.84 ppm, δC = 206.26 ppm, for $(CD_3)_2CO$; δH = 2.50 ppm and δC = 39.52 ppm for $(CD_3)_2SO$. Coupling constants (*J*) are reported in hertz (Hz). The multiplicities of signals are designated by the following abbreviations: s (singlet), d (doublet), t (triplet), q (quartet), dd (doublet of doublet), m (multiplet). Mass spectra (EI-MS, 70 eV) were performed on an Agilent 7890 gas chromatograph equipped with 5975C EI-MSD Triple-Axis Detector using DB5MS and HP5MS columns. HRMS analysis was recorded using a Thermo LTQ Velos Orbitrap mass spectrometer (Thermo Scientific, Pittsburgh, PA, USA) equipped with an ESI source.

UV/Vis spectra were recorded using Agilent Cary 60 UV/Vis. Luminescence intensities were recorded using a Fluoromax-4 spectrophotometer from Horiba Scientific. The photoluminescence signals were obtained using an automated motorized monochromator. Photoluminescence decay traces were acquired based on time-correlated single-photon-counting (TCSPC) techniques using a Fluoromax 4 spectrophotometer from Horiba Scientific. A 394 nm and 457 nm nanoLED were used as excitation sources. Time-resolved emission data were fit to a single or double exponential decay to extract the lifetimes. In all the time-resolved experiments only prompt luminescence was measured. The samples for UV/Vis/PL measurements were prepared in 2 mL quartz cuvettes (1 cm), equipped with screw cap PTFE stoppers, and sealed with parafilm inside the argon-filled glove-box.

The zero-zero vibrational state excitation energy $\boldsymbol{E}_{\mathbf{0\text{-}0}}$ was estimated by the corresponding energy of the wavelength at which normalized emission and absorption spectra overlap. Normalization was done by setting the intensity of emission maximum at $\lambda_{em,\ max}$ and the lowest energy absorption maximum at $\lambda_{abs,\ max*}$ as 1. Excited state oxidation and reduction potentials were calculated by the following approximating formulas: $\boldsymbol{E}(PC^{*}/PC^{\bullet-}) = \boldsymbol{E}(PC/PC^{\bullet-}) + \boldsymbol{E}_{\mathbf{0\text{-}0}}$ and $\boldsymbol{E}(PC^{\bullet+}/PC^{*}) = \boldsymbol{E}_{\mathbf{1/2}}(PC^{\bullet+}/PC) - \boldsymbol{E}_{\mathbf{0\text{-}0}}$.

Cyclic voltammetry (CV) was conducted on BioLogic Potentiostat SP-50. All CV measurements were performed under anhydrous conditions inside argon-filled glovebox. All supporting electrolytes were dried under dynamic vacuum (less than 0.1 mbar) over 24 h at 100 °C and stored inside the glovebox. The cell for the analysis was equipped with a glass

vial (working volume is 10 mL) and Teflon cap, equipped with O-ring for tight sealing. Glassy carbon was used as 2 working electrodes (circle, d = 3 mm), platinum wire as a counter electrode, and saturated calomel electrode (SCE) (CHI150 from CH Instruments, Inc.) as a reference electrode. All measurements were conducted in 0.1 M solutions of $^{n}Bu_4NPF_6$ in DMA/DCM/MeCN. All analyte concentration was 0.4/1/10 mM. The scan rate was typically 100 mV/s unless otherwise noted. In case of reversible/quasi-reversible peaks the potentials are obtained as $E_{1/2} = (E_{pc} + E_{pa})/2$. If the peak is irreversible the redox potential is calculated as half-peak potential $E_{p/2}$.

All catalytic reactions were carried out under irradiation from AL3 parallel photoreactor (purchased from 3STECH) 15W LED lamps, and PR160-640nm Kessil 34W LED lamps. For AL3 parallel photoreactor, the built-in fan provides cooling to maintain the temperature. For PR160-640nm Kessil, with a cooling fan to ensure the reaction remained near room temperature.

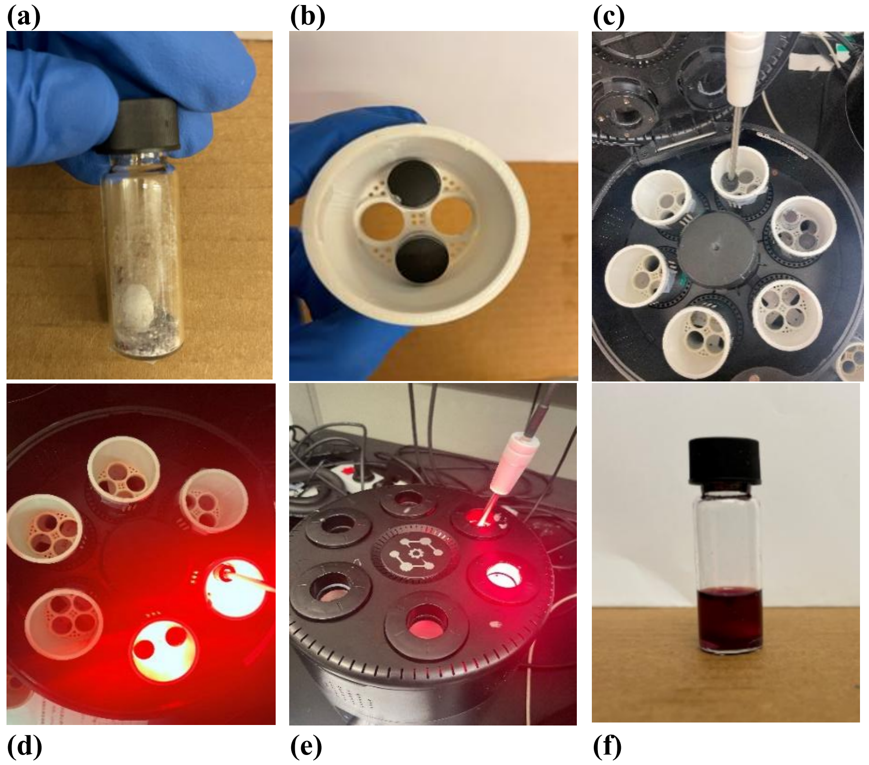

Figure S1. Photographs of the photochemical reaction set-ups. (**a**) The reaction mixture. (**b**) Modular hold for vials. (**c**) The reaction mixture before photo-irradiation. (**d**)The reaction mixture photo-irradiation ongoing (the cover of the photoreactor was opened). (**e**) The reaction mixture photo-irradiation ongoing (top side view). **(f)** The reaction mixture after completion.

2. Design, synthesis and photophysical measurements of photocatalysts

2.1. Photocatalysts design and screen

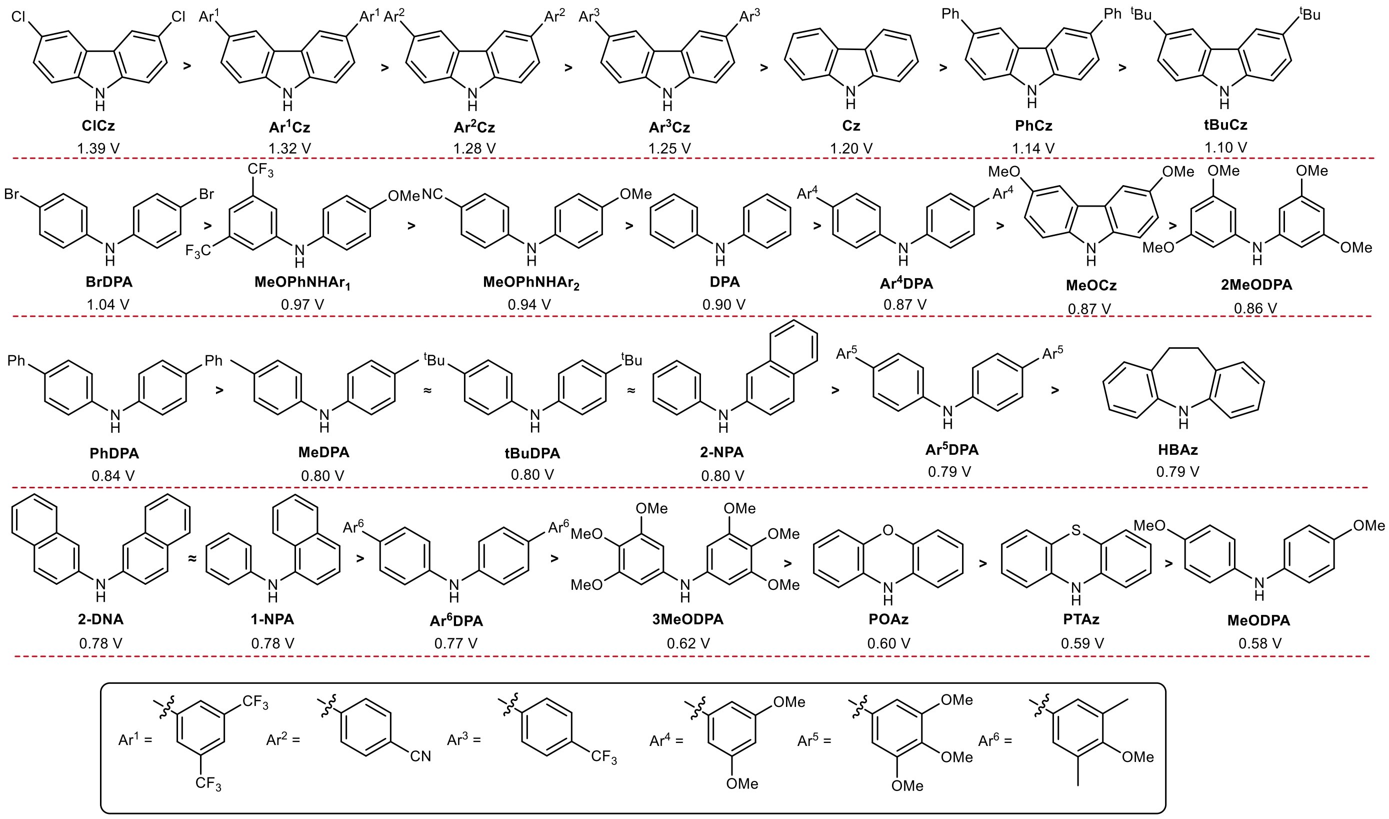

Figure S2. CV measured redox potentials of different donors.

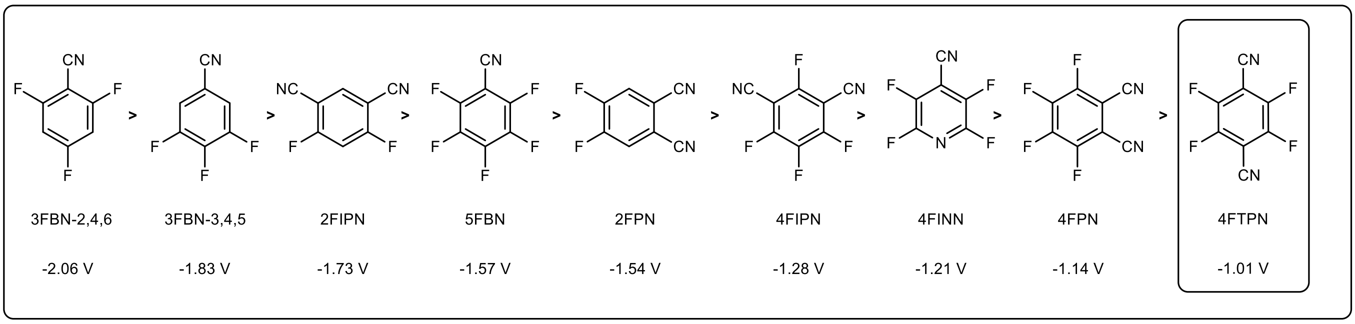

Figure S3. CV measured the redox potentials of different cores.

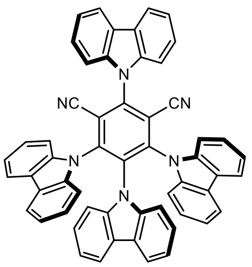

4CzIPN
PC1

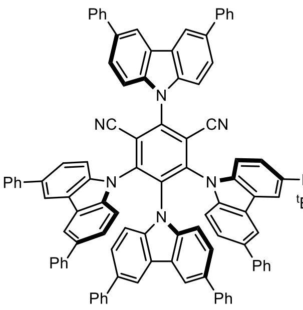

4PhCzIPN
PC2

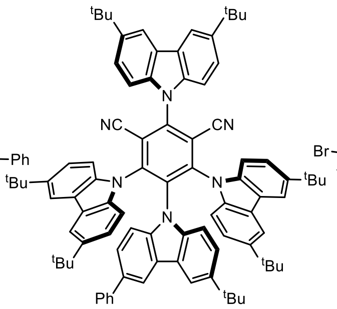

4tBuCzIPN
PC3

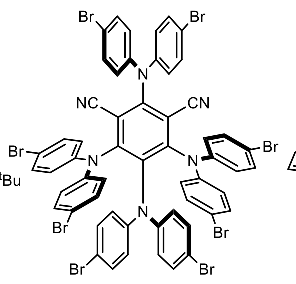

4BrDPAIPN
PC4

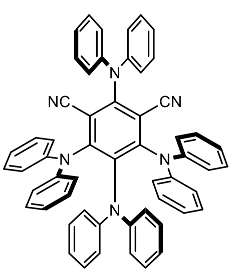

4DPAIPN
PC5

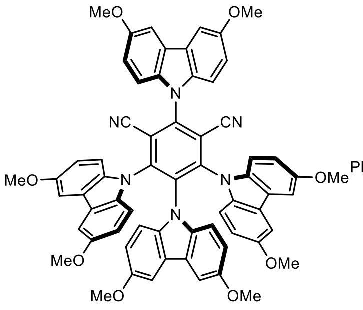

4MeOCzIPN
PC6

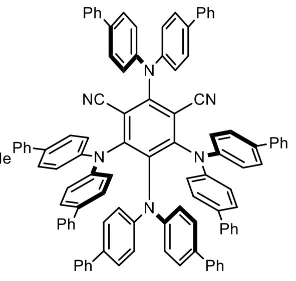

4PhDPAIPN
PC7

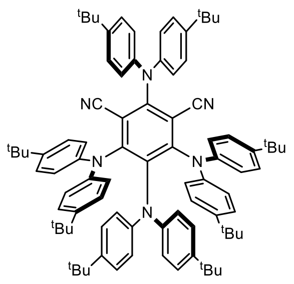

4tBuDPAIPN
PC8

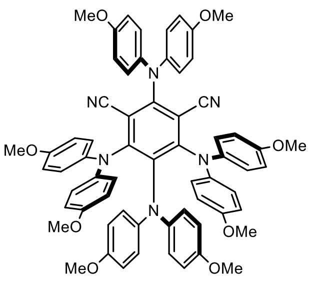

4MeODPAIPN
PC9

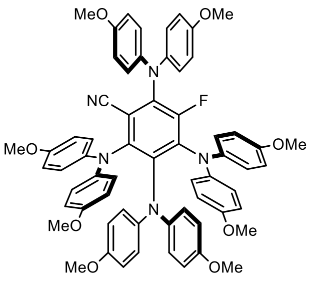

4MeODPAFBN
PC10

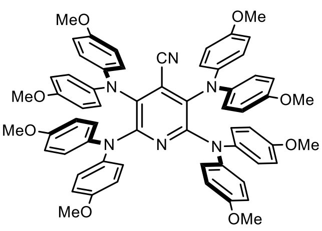

4MeODPAINN
PC11

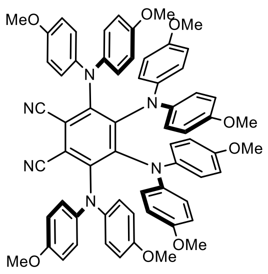

4MeODPAPN
PC12

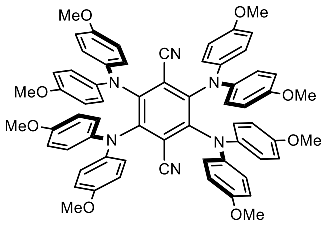

4MeODPATPN
PC13

Figure S4. Structures of **PC1-PC13**.

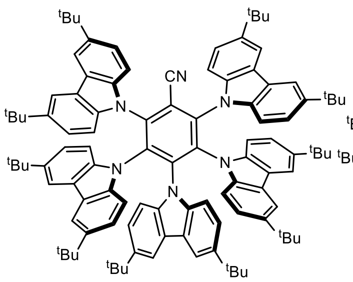

5tBuCzBN
PC14

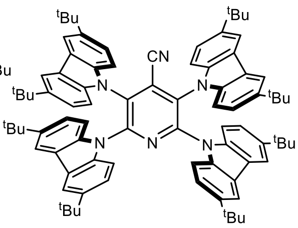

4tBuCzINN
PC15

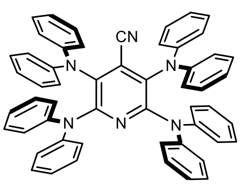

4DPAINN
PC16

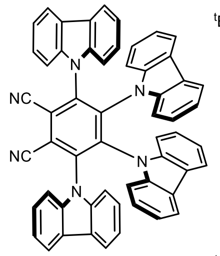

4CzPN
PC17

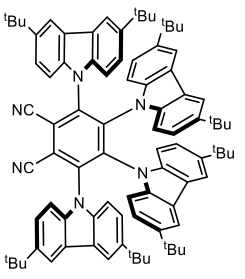

4tBuCzPN
PC18

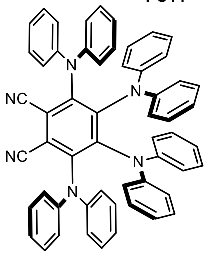

4DPAPN
PC19

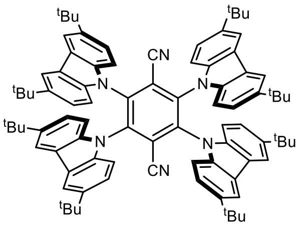

4tBuCzTPN
PC20

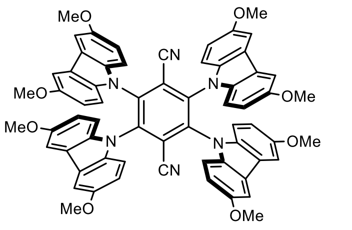

4MeOCzTPN
PC21

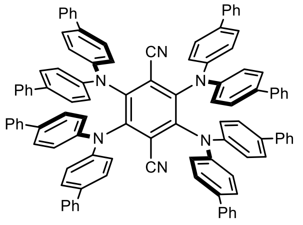

4PhDPATPN
PC22

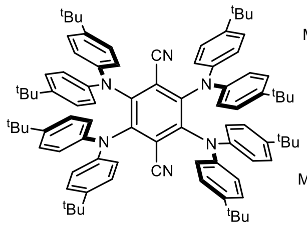

4tBuDPATPN
PC23

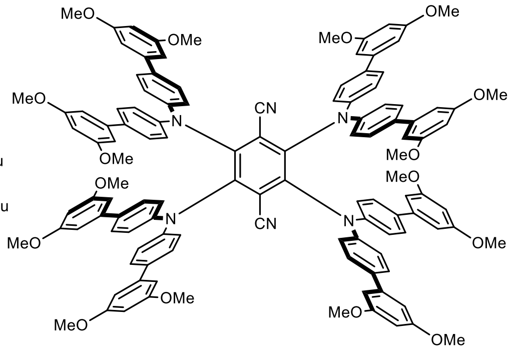

4Ar^{4}DPATPN
PC24

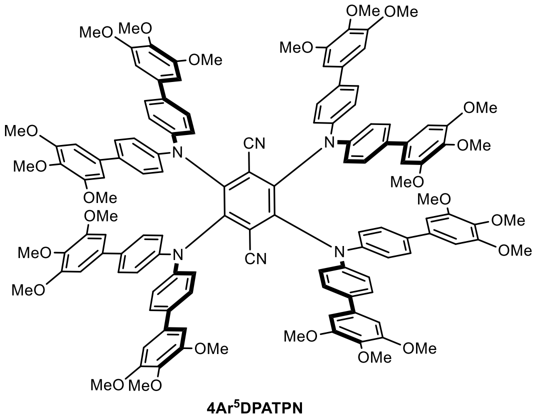

4Ar^{5}DPATPN
PC25

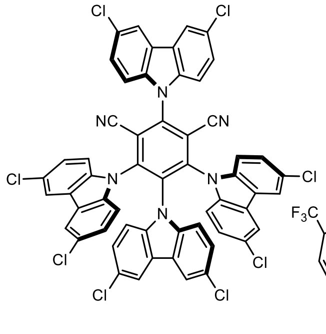

4ClCzIPN
PC26

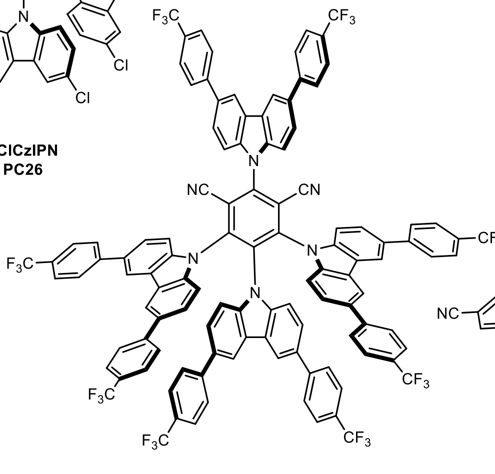

4Ar^{3}CzIPN
PC27

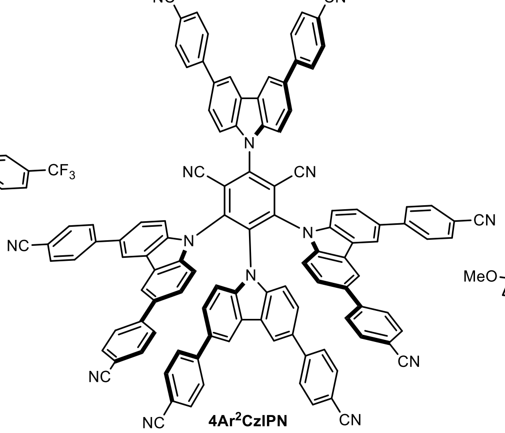

4Ar^{2}CzIPN
PC28

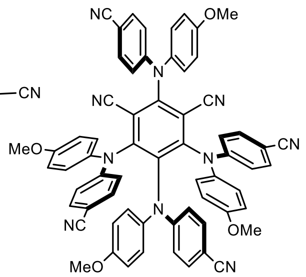

4(MeOPhNHAr^2)IPN
PC29

Figure S5. Structures of **PC14-PC29**.

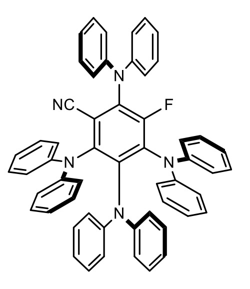

4DPAFBN
PC30

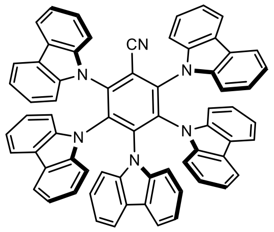

5CzBN
PC31

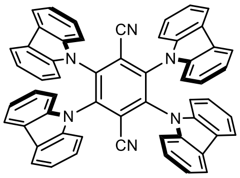

4CzTPN
PC32

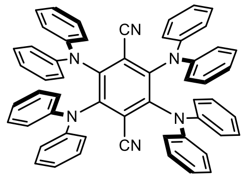

4DPATPN
PC33

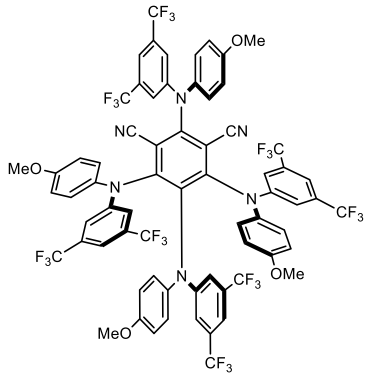

4(MeOPhNHAr[1])IPN
PC34

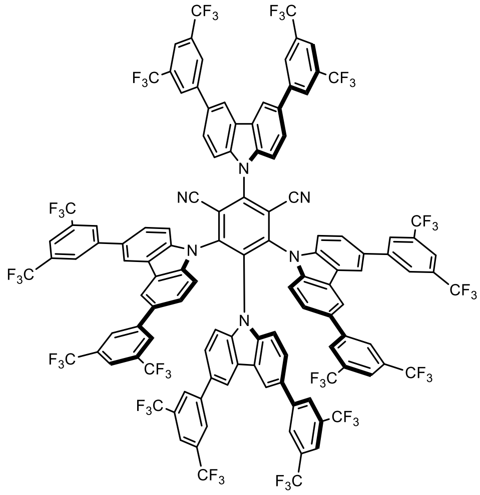

4Ar[1]CzIPN
PC35

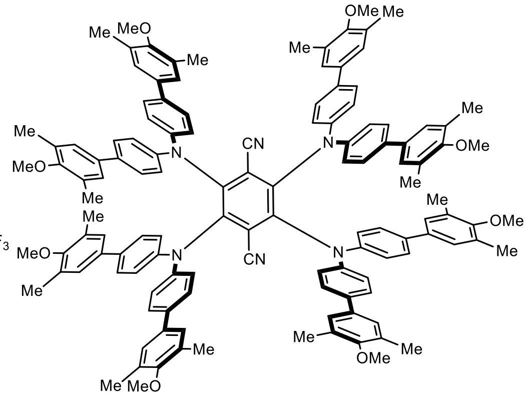

4Ar[6]DPATPN
PC36

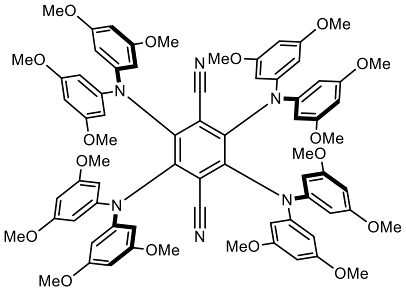

4(2MeODPA)TPN
PC37

Figure S6. Structures of **PC30-PC37**.

Table S1. Photophysical properties of photocatalysts.

Name	***E*** ($Pc^{\bullet+}/Pc$) (V. vs. SCE)	***E*** ($Pc/Pc^{\bullet-}$) (V. vs. SCE)	***E*** ($Pc^{\bullet+}/Pc^{*}$) (V. vs. SCE)	***E*** ($Pc^{*}/Pc^{\bullet-}$) (V. vs. SCE)	$E_{0\text{-}0}$, eV	λ_{em}, nm	E_{em}, eV	λ_{abs}, nm	E_{abs}, eV	Solvent	Lifetime, ns
**PC1**	1.37	-1.22	-1.26	1.41	2.63	541	2.29	428	2.90	MeCN	19.7
**PC2**	1.32	-1.17	-1.13	1.28	2.45	588	2.11	462	2.68	MeCN	20.1
**PC3**	1.28	-1.32	-1.23	1.19	2.51	570	2.18	440	2.82	MeCN	9.6
**PC4**	1.22	-1.43	-1.28	1.07	2.50	526	2.36	470	2.64	MeCN	1.0
**PC5**	1.02	-1.64	-1.48	0.86	2.50	529	2.34	470	2.64	MeCN	3.7
**PC6**	1.08	-1.38	-1.23	0.93	2.31	611	2.03	506	2.45	DCM	1.3
**PC7**	0.96	-1.56	-1.44	0.84	2.40	554	2.24	486	2.55	MeCN	4.3
**PC8**	0.83	-1.67	-1.56	0.72	2.39	564	2.20	485	2.56	MeCN	5.3
**PC9**	0.59	-1.88	-1.71	0.42	2.30	580	2.14	498	2.49	DCM	2.4
**PC10**	0.58	-2.05	-1.92	0.45	2.5	538	2.30	462	2.68	DCM	4.0
**PC11**	0.43	-1.83	-1.79	0.39	2.22	598	2.07	512	2.42	DCM	2.7
**PC12**	0.72	-1.67	-1.54	0.59	2.26	633	1.96	485	2.56	DCM	2.0 [53%] + 0.5 [47%]*
**PC13**	0.61	-1.62	-1.43	0.42	2.04	643	1.93	569	2.18	DCM	0.7 [66%] + 2.1 [33%]*
**PC13'**	0.76	-1.41	-1.33	0.68	2.09	632	1.96	557	2.23	DMA	11.8 [85%] + 0.8 [15%]*
**PC13-1**	0.55	-1.99	-2.03	0.59	2.58	514	2.41	443	2.80	DMA	3.8 [75%] + 9.4 [25%]*
**PC13-2**	0.70	-1.88	-1.79	0.61	2.49	543	2.28	461	2.69	DMA	2.6 [58%] + 9.2 [42%]*
**PC13-3**	0.58	-2.06	-1.94	0.46	2.52	523	2.52	464	2.67	DMA	2.6
**PC1-1**	1.36	-1.6	-1.52	1.28	2.88	506	2.45	381	3.25	MeCN	26.4
**PC14**	1.18	-1.61	-1.5	1.07	2.68	542	2.29	409	3.03	MeCN	12.2
**PC15**	1.24	-1.52	-1.17	0.89	2.41	552	2.25	477	2.60	DCM	4.4
**PC16**	0.8	-1.64	-1.57	0.73	2.37	553	2.24	485	2.56	MeCN	8.2
**PC17**	1.47	-1.14	-1.12	1.45	2.55	577	2.15	436	2.84	MeCN	5.9
**PC18**	1.27	-1.24	-1.17	1.2	2.44	611	2.03	447	2.77	MeCN	1.7
**PC19**	1.11	-1.49	-1.36	0.98	2.47	575	2.16	434	2.86	MeCN	4.9
**PC20**	1.34	-1.2	-0.94	1.08	2.28	578	2.15	513	2.42	DCM	4
**PC21**	1.03	-1.08	-1.1	1.05	2.13	640	1.94	520	2.38	DCM	0.7
**PC22**	0.85	-1.41	-1.28	0.72	2.13	617	2.01	554	2.24	DCM	2.5
**PC23**	0.8	-1.67	-1.33	0.46	2.13	612	2.03	557	2.23	DCM	3
**PC24**	0.91	-1.36	-1.21	0.76	2.12	617	2.01	546	2.27	DCM	2.6
**PC25**	0.81	-1.36	-1.3	0.75	2.11	625	1.98	553	2.24	DCM	1.3
**PC26**	1.6	-1.11	-0.97	1.46	2.57	530	2.34	458	2.71	DCM	15.3
**PC27**	1.5	-1.11	-1.01	1.4	2.51	570	2.18	450	2.76	MeCN	10.5
**PC28**	1.43	-1.1	-1.07 0	1.4	2.5	543	2.28	470	2.64	DCM	20.8
**PC29**	1.1	-1.39	-1.35	1.06	2.45	565	2.19	476	2.61	MeCN	1
**PC30**	0.99	-1.78	-1.71	0.92	2.7	492	2.52	433	2.86	MeCN	3
**PC31**	1.41	-1.52	-1.41	1.30	2.82	513	2.42	388	3.20	MeCN	24.6
**PC32**	1.15	-1.05	-1.23	1.33	2.38	520	2.22	486	2.55	DCM	6.6
**PC33**	0.94	-1.5	-1.28	0.72	2.22	585	2.12	530	2.34	DCM	2.4
**PC34**	1.18	-1.26	-1.32	1.24	2.5	565	2.19	459	2.70	MeCN	0.8
**PC35**	1.6	-1.08	-0.94	1.46	2.54	555	2.23	465	2.67	MeCN	15.9
**PC36**	0.81	-1.43	-1.29	0.67	2.1	624	1.99	555	2.23	DCM	1.7
**PC37**	1	-1.46	-1.18	0.72	2.18	584	2.12	517	2.40	DCM	2.7
**PC38**	1.33	-1.71	-1.96	1.58	3.29	460	2.70	336	3.69	MeCN	27.2
**PC39**	1.38	-1.81	-1.77	1.34	3.15	463	2.68	345	3.59	MeCN	16.1
**PC40**	1.50	-1.52	-1.45	1.43	2.95	503	2.47	370	3.35	MeCN	19.0
**PC41**	1.46	-1.44	-1.37	1.39	2.83	536	2.31	363	3.42	MeCN	12.2

*Values correspond to biexponential fits; numbers in brackets indicate the relative amplitudes of the decay components.

Table S2. CV measured redox potentials of different donors.

Donor	E (D$^{\cdot+}$/D) (V. vs SCE)	Donor	E (D$^{\cdot+}$/D) (V. vs SCE)
**ClCz**	1.39	**PhDPA**	0.84
**Ar^{1}Cz**	1.32	**MeDPA**	0.80
**Ar^{2}Cz**	1.28	**^{t}BuDPA**	0.80
**Ar^{3}Cz**	1.25	**2NPA**	0.80
**Cz**	1.20	**Ar^{5}PhDPA**	0.79
**PhCz**	1.14	**HBAz**	0.79
**^{t}BuCz**	1.10	**2DNA**	0.78
**BrDPA**	1.04	**1NPA**	0.78
**MeOPhNHAr^1**	0.97	**Ar^{6}PhDPA**	0.77
**MeOPhNHAr^2**	0.94	**3MeODPA**	0.62
**DPA**	0.90	**POAz**	0.60
**Ar^{4}PhDPA**	0.87	**PTAz**	0.59
**MeOCz**	0.87	**MeODPA**	0.58
**2MeODPA**	0.86		

Table S3. CV measured the redox potentials of different cores.

**Core**	**E (C$^{\cdot-}$/C) (V. vs SCE)**
**3FBN-2,4,6**	-2.08
**3FBN-3,4,5**	-1.83
**2FIPN**	-1.73
**5FBN**	-1.57
**2FPN**	-1.54
**4FIPN**	-1.28
**4FINN**	-1.21
**4FPN**	-1.14
**4FTPN**	-1.01

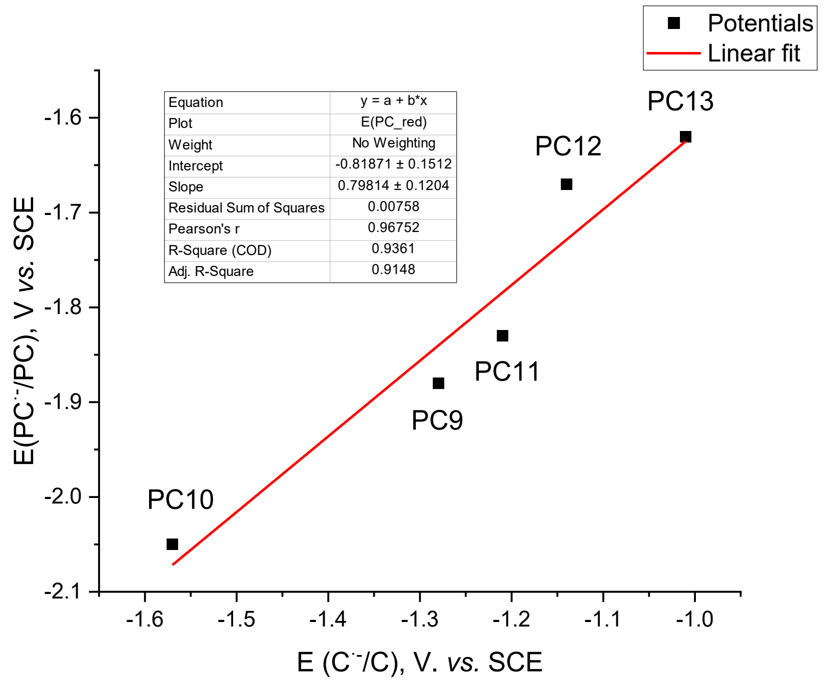

Figure S7. Correlation of reduction potential of photocatalysts and the respective cores.

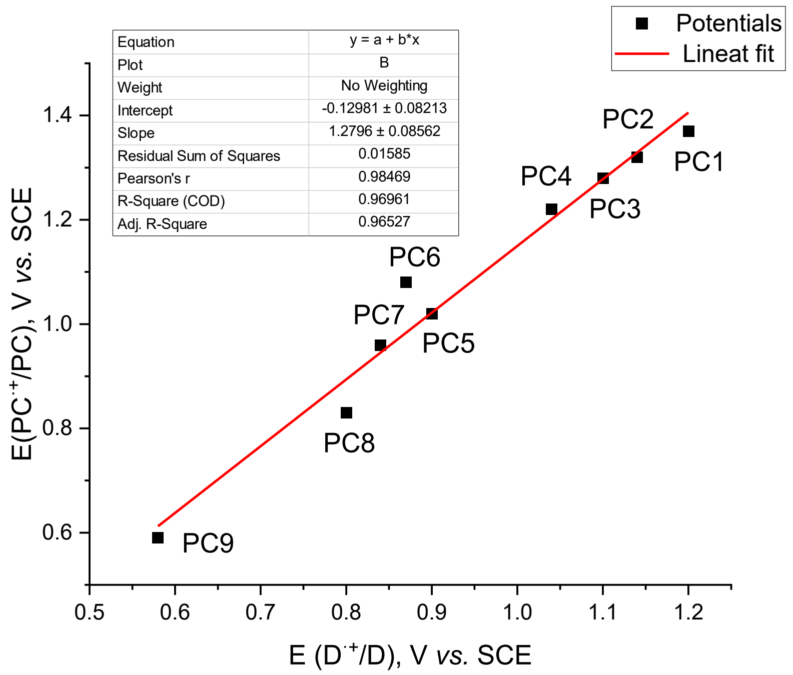

Figure S8. Correlation of oxidation potential of photocatalysts and the respective donors.

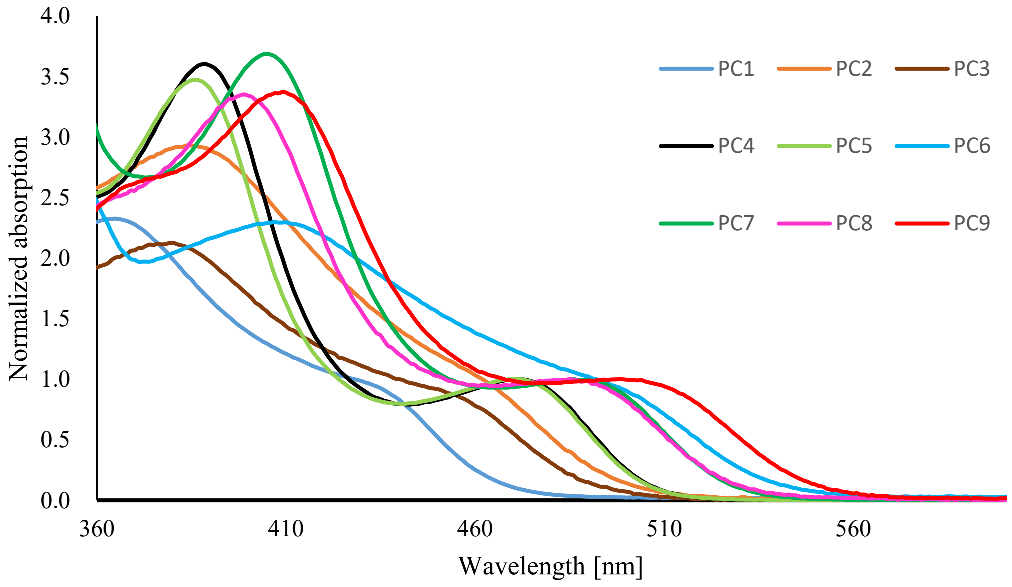

Figure S9. Comparison of different donors. Normalized absorption overlaps of **PC1-PC9.**

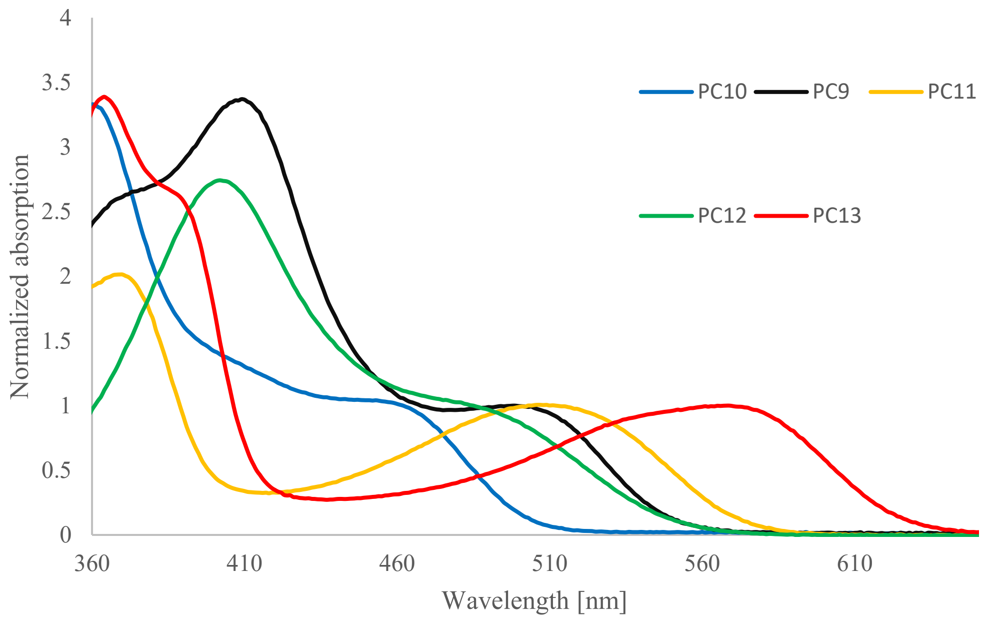

Figure S10. Comparison of different cores. Normalized absorption overlaps of **PC9-PC13.**

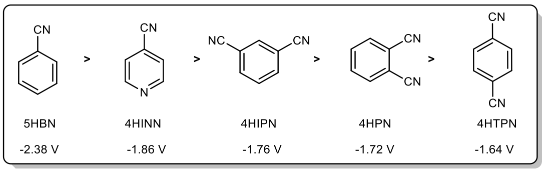

Figure S11. CV measured the redox potentials of H-cores.

Table S4. CV measured the redox potentials of different cores.

Core	*E* ($C^{\cdot -}/C$) (V. vs SCE)
**5HBN**	-2.38
**4HINN**	-1.86
**4HIPN**	-1.75
**4HPN**	-1.72
**4HTPN**	-1.64

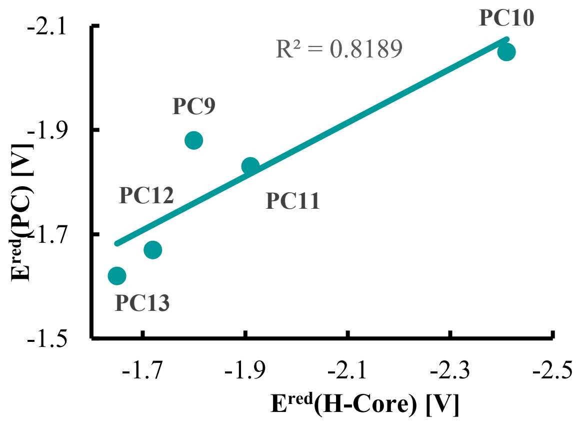

Figure S12. Correlation of reduction potential of photocatalysts and the respective H-cores.

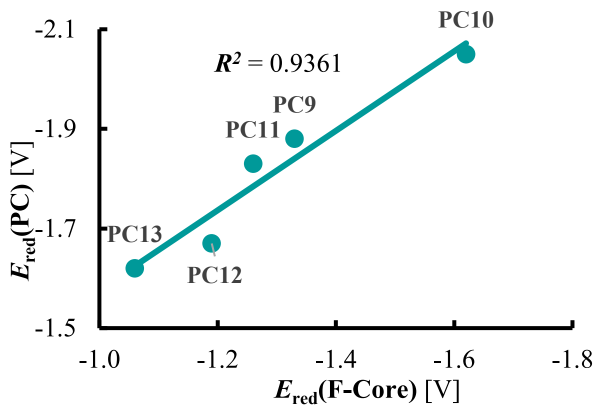

Figure S13. Correlation of reduction potential of photocatalysts and the respective F-cores.

Table S5. The reduction potentials of different Cz-based dyes.

	E (C$^-$/C) [F-core] (V. vs SCE)	E (Pc/Pc$^{\bullet -}$) (V. vs. SCE)
**3CzBN-2,4,6 (PC39)**	-2.08	-1.81
**3CzBN-3,4,5 (PC38)**	-1.83	-1.71
**2CzIPN (PC40)**	-1.73	-1.52
**5CzBN (PC31)**	-1.57	-1.52
**2CzPN (PC41)**	-1.54	-1.45
**4CzIPN (PC1)**	-1.28	-1.22
**4CzINN**	-1.21	
**4CzPN (PC17)**	-1.14	-1.14
**4CzTPN (PC32)**	-1.01	-1.05

Table S6. The reduction potentials of different Cz-based dyes.

	E (C$^-$/C) [H-core] (V. vs SCE)	E (Pc/Pc$^{\bullet -}$) (V. vs. SCE)
**3CzBN-2,4,6 (PC39)**	-2.38	-1.81
**3CzBN-3,4,5 (PC38)**	-2.38	-1.71
**2CzIPN (PC40)**	-1.81	-1.52
**5CzBN (PC31)**	-2.38	-1.52
**2CzPN (PC41)**	-1.72	-1.45
**4CzIPN (PC1)**	-1.81	-1.22
**4CzINN (PC1)**	-1.86	
**4CzPN (PC17)**	-1.72	-1.14
**4CzTPN (PC32)**	-1.64	-1.05

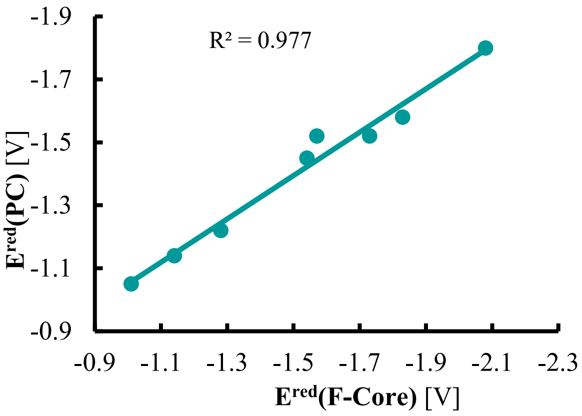

Figure S14. Correlation of reduction potential of photocatalysts and the respective F-cores.

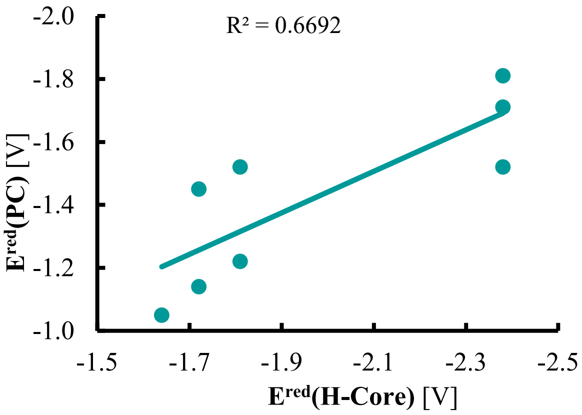

Figure S15. Correlation of reduction potential of photocatalysts and the respective H-cores.

Table S7. The data for the initial study and the results of the fitting. ***E*** ($D^{\cdot+}/D$)

PC	***E*** ($D^{\cdot+}/D$) (V. vs SCE)	***E*** ($C^{\cdot-}/C$) (V. vs SCE)	$E_{0\text{-}0}^{exp}$, eV	$E_{0\text{-}0}^{pred}$, eV	Residue	Rel. Error
**PC1**	1.20	-1.28	2.63	2.56	0.07	0.03
**PC2**	1.14	-1.28	2.45	2.53	-0.08	0.03
**PC3**	1.10	-1.28	2.51	2.51	0.00	0.00
**PC4**	1.04	-1.28	2.5	2.49	0.01	0.01
**PC5**	0.90	-1.28	2.5	2.43	0.07	0.03
**PC6**	0.87	-1.28	2.31	2.41	-0.10	0.04
**PC7**	0.84	-1.28	2.4	2.40	0.00	0.00
**PC8**	0.80	-1.28	2.39	2.38	0.01	0.00
**PC9**	0.58	-1.28	2.3	2.29	0.01	0.00
**PC10**	0.58	-1.57	2.5	2.51	-0.01	0.00
**PC11**	0.58	-1.21	2.22	2.24	-0.02	0.01
**PC12**	0.58	-1.14	2.26	2.18	0.08	0.04
**PC13**	0.58	-1.01	2.04	2.08	-0.04	0.02

MAE	MSE	RMSE	MAPE
0.0393	0.00282	0.0531	0.0165

Table S8. Correlation matrix for the initial study.

	$E_{0\text{-}0}^{exp}$	*E* (D·+/D)	*E* (C·-/C)
$E_{0\text{-}0}^{exp}$	1	0.73	-0.67
E (D·+/D)	0.73	1	-0.13
E (C·-/C)	-0.67	-0.13	1

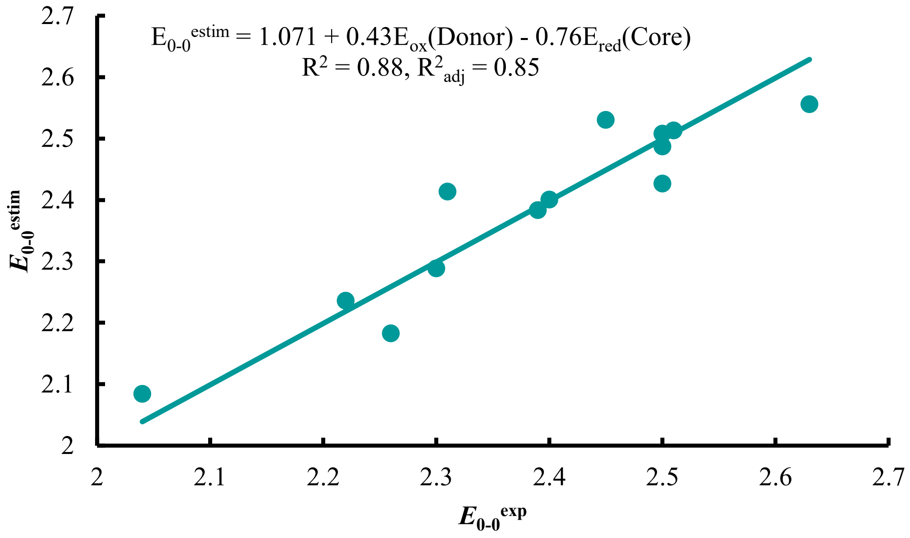

Figure S16. Estimated values of $\boldsymbol{E_{0\text{-}0}}$ vs. experimental for **PC1-PC13**.

Table S9. The expanded data for the study and the results of the fitting.

PC	*E* (D^{+}/D) (V. vs SCE)	*E* (C^{-}/C) (V. vs SCE)	E_{0-0}^{exp}, eV	E_{0-0}^{pred}, eV	*Residue*	*Rel. Error*
**PC1**	1.20	-1.28	2.63	2.53	0.10	0.04
**PC2**	1.14	-1.28	2.45	2.51	-0.06	0.02
**PC3**	1.10	-1.28	2.51	2.49	0.02	0.01
**PC4**	1.04	-1.28	2.50	2.47	0.03	0.01
**PC5**	0.90	-1.28	2.50	2.42	0.08	0.03
**PC6**	0.87	-1.28	2.31	2.41	-0.10	0.04
**PC7**	0.84	-1.28	2.40	2.39	0.01	0.00
**PC8**	0.80	-1.28	2.39	2.38	0.01	0.00
**PC9**	0.58	-1.28	2.30	2.29	0.01	0.00
**PC10**	0.58	-1.57	2.50	2.54	-0.04	0.01
**PC11**	0.58	-1.14	2.22	2.18	0.04	0.02
**PC12**	0.58	-1.21	2.26	2.24	0.02	0.01
**PC13**	0.58	-1.01	2.04	2.07	-0.03	0.01
**PC14**	1.1	-1.57	2.68	2.74	-0.06	0.02
**PC15**	1.1	-1.14	2.41	2.38	0.03	0.01
**PC16**	0.9	-1.14	2.37	2.3	0.07	0.03
**PC17**	1.2	-1.21	2.55	2.47	0.08	0.03
**PC18**	1.1	-1.21	2.44	2.44	0	0
**PC19**	0.9	-1.21	2.47	2.36	0.11	0.04
**PC20**	1.1	-1.01	2.28	2.27	0.01	0
**PC21**	0.87	-1.01	2.13	2.18	-0.05	0.02
**PC22**	0.84	-1.01	2.13	2.17	-0.04	0.02
**PC23**	0.8	-1.01	2.13	2.15	-0.02	0.01
**PC24**	0.87	-1.01	2.12	2.18	-0.06	0.03
**PC25**	0.79	-1.01	2.11	2.15	-0.04	0.02
**PC26**	1.39	-1.28	2.57	2.61	-0.04	0.01
**PC27**	1.25	-1.28	2.51	2.55	-0.04	0.02
**PC28**	1.28	-1.28	2.5	2.56	-0.06	0.03
**PC29**	0.94	-1.28	2.45	2.43	0.02	0.01

MAE	MSE	RMSE	MAPE
0.0436	0.0027	0.0523	1.83

Table S10. Correlation matrix for the initial study.

	E_{0-0}^{exp}	*E* (D·+/D)	*E* (C^{-}/C)
E_{0-0}^{exp}	1	0.63	-0.81
***E* (D·+/D)**	0.63	1	-0.18
***E* (C^{-}/C)**	-0.81	-0.18	1

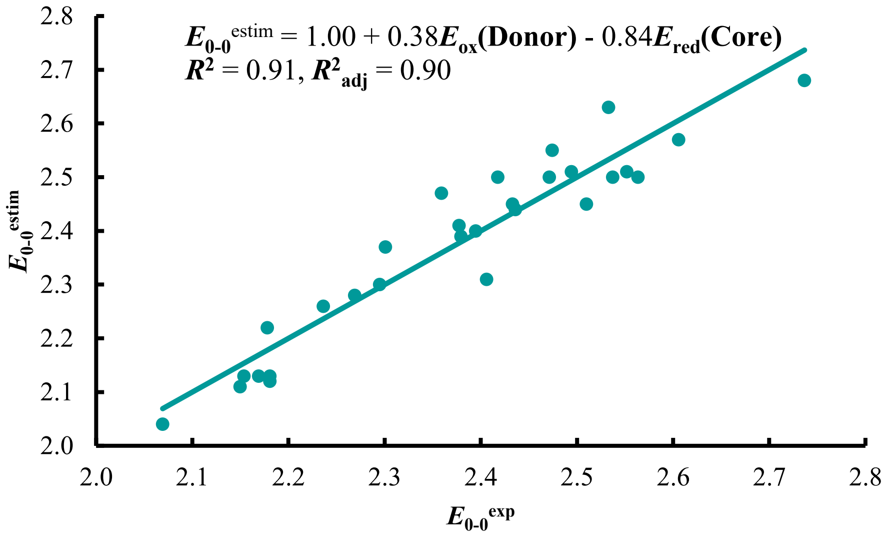

Figure S17. Estimated values of $E_{0\text{-}0}$ vs. experimental for **PC1-PC29**.

Table S11. The results of the prediction.

PC	E (D^{+}/D) (V. vs SCE)	E (C^{-}/C) (V. vs SCE)	$E_{0\text{-}0}^{exp}$, eV	$E_{0\text{-}0}^{pred}$, eV	*Residue*	*Rel. Error*
**PC30**	0.90	-1.57	2.70	2.66	0.04	0.02
**PC31**	1.20	-1.57	2.82	2.77	0.05	0.02
**PC32**	1.20	-1.01	2.38	2.31	0.07	0.03
**PC33**	0.90	-1.01	2.22	2.19	0.03	0.01
**PC34**	0.97	-1.28	2.50	2.44	0.06	0.02
**PC35**	1.32	-1.28	2.54	2.58	-0.04	0.02
**PC36**	0.77	-1.01	2.10	2.14	-0.04	0.02
**PC37**	0.86	-1.01	2.18	2.18	0.00	0.00
MAE	MSE	RMSE	MAPE			
0.0407	0.0020	0.0449	1.68			

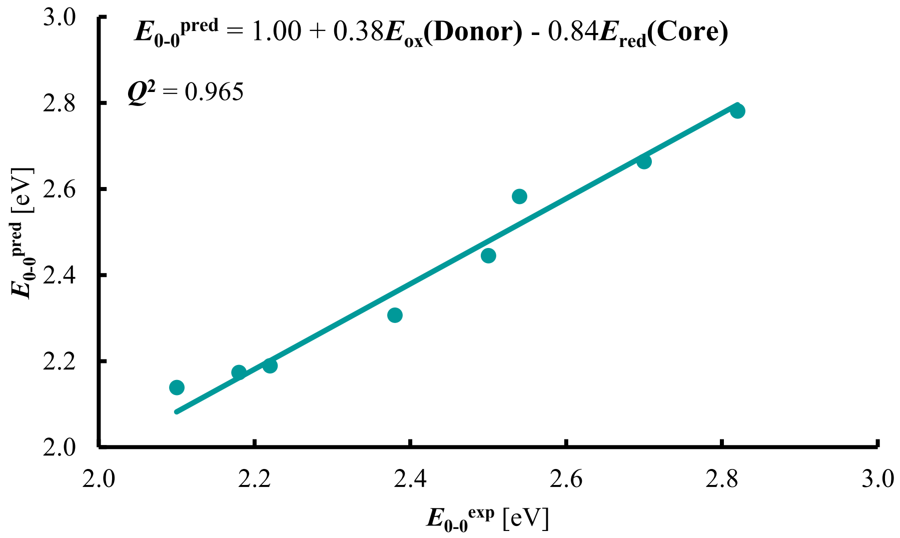

Figure S18. Predicted $E_{0\text{-}0}$ vs actual for **PC30-PC37**.

Utilizing the python script (The data and code are available in GitHub repository https://github.com/lolikbolik123/k_means_CV_data.git) of **PC1-PC37** to split the data into training and validation sets with n = 5 and 100 repeats we obtained the average mean R^2 value of 0.876 and median value of 0.90 for validation.

We synthesized carbazole-derived **PC38-PC41** from the corresponding precursors (3FBN-2,4,6; 3FBN-3,4,5; 2FIPN; 2FPN), measured $E_{0\text{–}0}$, and prospectively tested the equation obtained before (Table S12) . Agreement is good for three of four cases; the outlier **3CzBN-3,4,5** likely reflects diminished conjugation of the *meta*-3,5-carbazoles with the nitrile whereas in all other examples there are *ortho*- or *para*-conjugation.

Table S12. Additional validation of unexplored cores.

PC	$E_{0\text{–}0}^{exp}$ (eV)	$E_{0\text{–}0}^{pred}$ (eV)	*Rel. error, %*
**3CzBN-3,4,5 (PC38)**	3.29	2.99	9.0
**3CzBN-2,4,6 (PC39)**	3.15	3.20	1.7
**2CzIPN (PC40)**	2.83	2.75	2.8
**2CzPN (PC41)**	2.95	2.91	1.4

We have now extended our analysis to ground-state redox properties (Fig. S19-S20). E_{ox}**(PC)** is well correlated from the donor's E_{ox}**(Donor)** ($R^2 \approx 0.93$ across our set), while E_{red}**(PC)** shows moderate correlation to the core's E_{red}**(Core)** ($R^2 \approx 0.80$). We attribute the lower accuracy for E_{red}**(Core)** to donor–core torsion and solvent-specific stabilization of the core-localized LUMO, which are not captured by the current two-descriptor model.

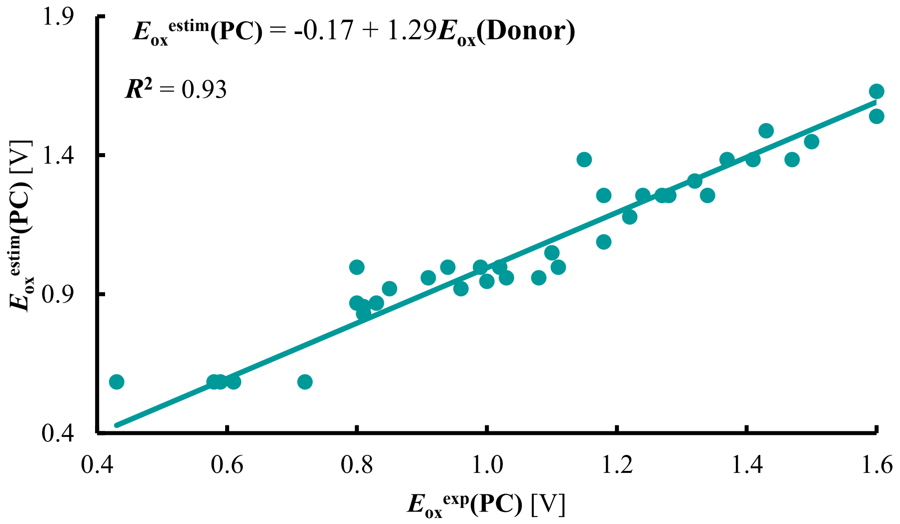

Figure S19. Estimated values of E_{ox}^{estim}**(PC)** vs. experimental for **PC1-PC37**.

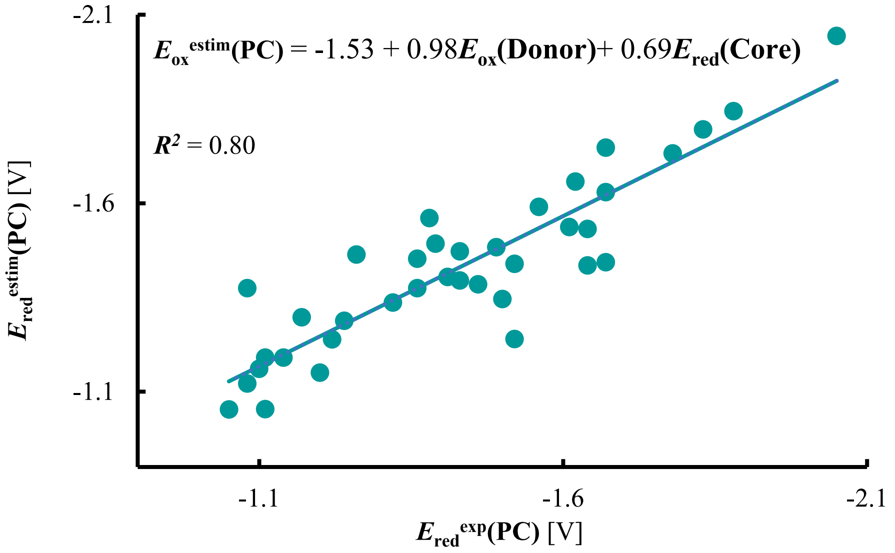

Figure S20. Estimated values of E_{red}^{estim}**(PC)** vs. experimental for **PC1-PC37**.

2.2. Synthesis of the donors and the photocatalysts

2.2.1. Synthesis of the donors

2MeODPA---bis(3,5-dimethoxyphenyl)amine

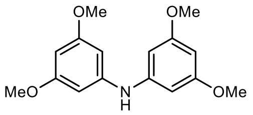

A dried (under reduced pressure) Schlenk tube was charged with 1-bromo-3,5-dimethoxybenzene (3.26 g, 15 mmol), 3,5-dimethoxyaniline (2.99 g, 19.5 mmol), NaO^{t}Bu (2.16 g, 22.5 mmol), $Pd_2(dba)_3{\cdot}CHCl_3$ (69.9 mg, 0.0675 mmol), and (2-biphenyl)-di-*tert*-butylphosphine (89.5 mg, 0.02 mmol). The reagents were dried under reduced pressure and the vessel was backfilled with argon. Anhydrous toluene was added (30 mL), and the reaction mixture was heated at 80 °C for 24 hours. The mixture was cooled to room temperature, diluted with ethyl acetate, filtered through Celite, and concentrated in vacuo. The crude product was purified as a white solid by flash chromatography on silica gel using hexane/EtOAc as eluent. Yield: 90% (3.9 g). The spectral data are consistent with those of the literature.

^{1}H NMR (500 MHz, CDCl$_3$) δ 6.33 – 6.27 (m, 4H), 6.14 – 6.09 (m, 2H), 3.76 (s, 12H).
^{13}C NMR (126 MHz, CDCl$_3$) δ 161.6, 144.7, 96.6, 93.5, 55.2.
HRMS (ESI): calculated for $[M+Na]^+$ $C_{16}H_{19}NO_4Na^+$ 312.1206; found 312.1214.

3MeODPA---bis(3,4,5-trimethoxyphenyl)amine

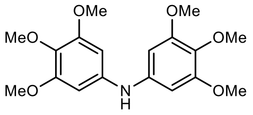

A dried (under reduced pressure) Schlenk tube was charged with 1-bromo-3,4,5-trimethoxybenzene (3.71 g, 15 mmol), 3,4,5-trimethoxyaniline (3.57 g, 19.5 mmol), NaO^{t}Bu (2.16 g, 22.5 mmol), $Pd_2(dba)_3{\cdot}CHCl_3$ (69.9 mg, 0.0675 mmol), and (2-biphenyl)-di-*tert*-butylphosphine (89.5 mg, 0.02 mmol). The reagents were dried under reduced pressure and the vessel was backfilled with argon. Anhydrous toluene was added (30 mL), and the reaction mixture was heated at 80 °C for 24 hours. The mixture was cooled to room temperature, diluted with ethyl acetate, filtered through Celite, and concentrated in vacuo. The crude product was purified as a white solid by flash chromatography on silica gel using hexane/EtOAc as eluent. Yield: 73% (3.85 g). The spectral data are consistent with those of the literature.

^{1}H NMR (500 MHz, CDCl$_3$) δ 6.33 – 6.23 (m, 4H), 3.76 (s, 6H), 3.72 (s, 12H).
^{13}C NMR (126 MHz, CDCl$_3$) δ153.7, 139.7, 132.1, 95.4, 60.9, 55.8.
HRMS (ESI): calculated for $[M+Na]^+$ $C_{18}H_{23}NO_6Na^+$ 372.1418; found 372.1432.

MeOPhNHAr^1--- N-(4-methoxyphenyl)-3,5-bis(trifluoromethyl)aniline

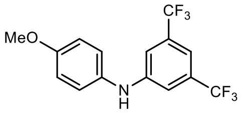

A dried (under reduced pressure) Schlenk tube was charged with 1-bromo-3,5-bis(trifluoromethyl)benzene (2.59 ml, 15 mmol), *p*-anisidine (2.4 g, 19.5 mmol), NaOt-Bu (2.16 g, 22.5 mmol), $Pd_2(dba)_3{\cdot}CHCl_3$ (69.9 mg, 0.0675 mmol), and (2-biphenyl)-di-*tert*-butylphosphine (89.5 mg, 0.02 mmol). The reagents were dried under reduced pressure and the vessel was backfilled with argon. Anhydrous toluene was added (30 mL), and the reaction mixture was heated at 80 °C for 24 hours. The mixture was cooled to room temperature, diluted with ethyl acetate, filtered through Celite, and concentrated in vacuo. The crude product was purified as a white solid by flash chromatography on silica gel using hexane/EtOAc as eluent. Yield: 60% (3.0 g). The spectral data are consistent with those of the literature.[3]

^{1}H NMR (500 MHz, CDCl$_3$) δ 7.22 (s, 1H), 7.17 (s, 2H), 7.11 (d, *J* = 8.8 Hz, 2H), 6.96 – 6.91 (m, 2H), 5.88 (s, 1H), 3.83 (s, 3H).

^{13}C NMR (126 MHz, CDCl$_3$) δ 157.2, 147.3, 132.9, 132.7 (q, *J* = 32.9 Hz), 124.8, 123.6 (q, *J* = 272.6 Hz), 115.2, 113.7 (q, *J* = 4.1 Hz), 111.8 (hept, *J* = 3.7 Hz), 55. 7.

^{19}F NMR (471 MHz, CDCl$_3$) δ -63.24.

MeOPhNHAr^2--- 4-((4-methoxyphenyl)amino)benzonitrile

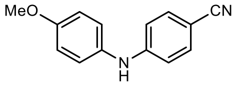

A dried (under reduced pressure) Schlenk tube was charged with 4-brombenzonitrile (2.73 g, 15 mmol), *p*-anisidine (2.4 g, 19.5 mmol), NaO^{t}Bu (2.16 g, 22.5 mmol), Pd$_2$(dba)$_3$·CHCl$_3$ (69.9 mg, 0.0675 mmol), and (2-biphenyl)-di-*tert*-butylphosphine (89.5 mg, 0.02 mmol). The reagents were dried under reduced pressure and the vessel was backfilled with argon. Anhydrous toluene was added (30 mL), and the reaction mixture was heated at 80 °C for 24 hours. The mixture was cooled to room temperature, diluted with ethyl acetate, filtered through Celite, and concentrated in vacuo. The crude product was purified as a white solid by flash chromatography on silica gel using hexane/EtOAc as eluent. Yield: 76% (2.55 g). The spectral data are consistent with those of the literature.[4]

^{1}H NMR (500 MHz, CDCl$_3$) δ 7.43 (d, *J* = 8.6 Hz, 2H), 7.12 (d, *J* = 7.2 Hz, 2H), 6.91 (d, *J* = 8.8 Hz, 2H), 6.79 (d, *J* = 8.4 Hz, 2H), 3.82 (s, 3H).

^{13}C NMR (126 MHz, CDCl$_3$) δ 157.2, 149.7, 133. 9, 132.6, 125.3, 120.2, 115.0, 113.9, 100.6, 55.7.

Ar^{1}Cz--- 3,6-bis(3,5-bis(trifluoromethyl)phenyl)-9*H*-carbazole

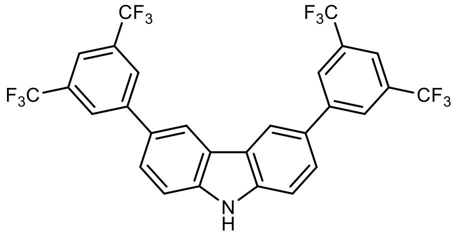

3,6-dibromo-9*H*-carbazole (3.25 g, 10 mmol), (3,5-bis(trifluoromethyl)phenyl)boronic acid (6.19 g, 24 mmol), K$_2$CO$_3$ (5.53 g, 40 mmol), and Pd(PPh$_3$)$_4$ (347 mg, 0.3 mmol) were dissolved in a 150 mL mixture of 1,4-dioxane and water (10/1, v/v) under argon atmosphere. The mixture was stirred for 24 h at 90 °C and cooled down to room temperature. Afterward, the resulting mixture was mixed with 200 mL water, and then DCM was used to extract the desired product, which would be collected and evaporated under reduced pressure. The crude product was purified by flash chromatography on silica gel (PE/DCM) followed by recrystallization from EtOAc/PE as white solid. Yield: 73% (4.32 g).

^{1}H NMR (500 MHz, CDCl$_3$) δ 8.40 (d, *J* = 1.5 Hz, 2H), 8.15 (s, 4H), 7.86 (s, 2H), 7.73 (dd, *J* = 8.4, 1.8 Hz, 2H), 7.61 (d, *J* = 8.4 Hz, 2H).

^{13}C NMR (126 MHz, CDCl$_3$) δ 144.0, 140.3, 132.3 (q, *J* = 33.2 Hz), 130.66, 127.4 (q, *J* = 3.3 Hz), 126.0, 124.1, 123.7 (q, *J* = 272.7 Hz), 120.4 (q, *J* = 3.8 Hz), 119. 6, 111.8.

^{19}F NMR (471 MHz, CDCl$_3$) δ -62.7.

HRMS (ESI): calculated for $[M+H]^+$ $C_{28}H_{14}F_{12}N^+$ 592.0929; found 592.0936.

Ar²Cz---4,4'-(9*H*-carbazole-3,6-diyl)dibenzonitrile

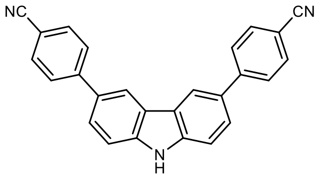

3,6-dibromo-9*H*-carbazole (3.25 g, 10 mmol), 4-cyanophenylboronic acid (3.97 g, 27 mmol), K_2CO_3 (5.53 g, 40 mmol), and $Pd(PPh_3)_4$ (347 mg, 0.3 mmol) were dissolved in a 150 mL mixture of 1,4-dioxane and water (10/1, v/v) under argon atmosphere. The mixture was stirred for 24 h at 90 °C and cooled down to room temperature. Afterward, the resulting mixture was evaporated under reduced pressure. The product has low solubility in EtOAc. The crude product was washed with 20% (EtOAc/PE) then dissolved in acetone and recrystallized from hot acetone. Yield: 81% (3.0 g).

^{1}H NMR (400 MHz, DMSO) δ 11.64 (s, 1H), 8.77 – 8.71 (m, 2H), 8.01 (d, *J* = 8.4 Hz, 4H), 7.94 (d, *J* = 8.4 Hz, 4H), 7.85 (dd, *J* = 8.5, 1.6 Hz, 2H), 7.63 (d, *J* = 8.5 Hz, 2H).

^{13}C NMR (101 MHz, DMSO) δ 145.7, 140.6, 132.9, 129.2, 127.2, 125.2, 123.5, 119.6, 119.2, 111.9, 108.8.

HRMS (ESI): calculated for $[M+Na]^+$ $C_{26}H_{15}N_3Na^+$ 392.1158; found 392.1167.

Ar³Cz--- 3,6-bis(4-(trifluoromethyl)phenyl)-9*H*-carbazole

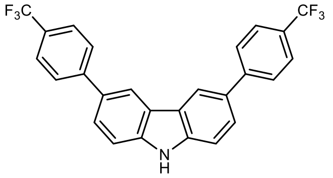

3,6-dibromo-9*H*-carbazole (3.25 g, 10 mmol), 4-(trifluoromethyl)phenylboronic acid (4.56 g, 24 mmol), K_2CO_3 (5.53 g, 40 mmol), and $Pd(PPh_3)_4$ (347 mg, 0.3 mmol) were dissolved in a 150 mL mixture of 1,4-dioxane and water (10/1, v/v) under argon atmosphere. The mixture was stirred for 24 h at 90 °C and cooled down to room temperature. Afterward, the resulting mixture was mixed with 200 mL water, and then DCM was used to extract the desired product, which would be collected and evaporated under reduced pressure. The crude product was purified by flash chromatography on silica gel (PE/DCM) as white solid. Yield: 69% (3.16 g).

^{1}H NMR (500 MHz, $CDCl_3$) δ 8.36 (d, *J* = 1.1 Hz, 2H), 8.21 (s, 1H), 7.82 (d, *J* = 8.1 Hz, 4H), 7.76 – 7.68 (m, 6H), 7.55 (d, *J* = 8.4 Hz, 2H).

^{13}C NMR (126 MHz, $CDCl_3$) δ 145.5, 140.0, 132.0, 128.8 (q, *J* = 32.4 Hz), 126.0, 125.9 (q, *J* = 3.9 Hz), 124.6 (q, *J* = 271.9 Hz), 124.1, 119.3, 111.4.

^{19}F NMR (471 MHz, $CDCl_3$) δ -62.2.

HRMS (ESI): calculated for $[M+H]^+$ $C_{26}H_{16}N_3^+$ 456.1181; found 456.1173.

Ar⁴DPA---bis(3',5'-dimethoxy-[1,1'-biphenyl]-4-yl)amine

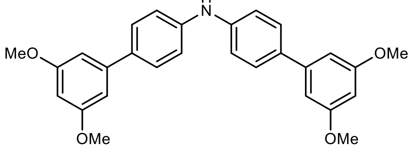

Bis(4-bromophenyl)amine (4.9 g, 15 mmol), (3,5-trimethoxyphenyl)boronic acid (6.5 g, 36 mmol), K_2CO_3 (8.3 g, 24.6 mmol), and $Pd(PPh_3)_4$ (520 mg, 0.45 mmol) were dissolved in a 150 mL mixture of 1,4-dioxane and water (10/1, v/v) under argon atmosphere. The mixture was stirred for 24 h at 90 °C and cooled down to room temperature. Afterward, the resulting mixture was mixed with 200 mL water, and then DCM was used to extract the desired product, which would be collected and evaporated under reduced pressure. The crude product was purified by flash chromatography on silica gel (PE/DCM) as white solid. Yield: 48% (3.2 g).

^{1}H NMR (500 MHz, DMSO) δ 8.49 (s, 1H), 7.59 (d, *J* = 8.5 Hz, 4H), 7.18 (d, *J* = 8.6 Hz, 4H), 6.75 (d, *J* = 2.2 Hz, 4H), 6.44 (t, *J* = 2.2 Hz, 2H), 3.80 (s, 12H).

^{13}C NMR (126 MHz, DMSO) δ 160.8, 142.9, 142.2, 131.4, 127.6, 116.9, 104.0, 98.5, 55.2.

HRMS (ESI): calculated for $[M+Na]^+$ $C_{28}H_{27}NO_4Na^+$ 464.1832; found 464.1833.

Ar^{5}DPA---bis(3',4',5'-trimethoxy-[1,1'-biphenyl]-4-yl)amine

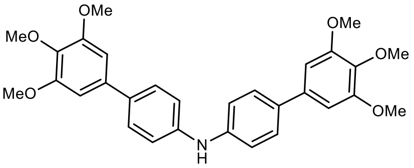

Bis(4-bromophenyl)amine (4.9 g, 15 mmol), (3,4,5-trimethoxyphenyl)boronic acid (7.6 g, 36 mmol), K_2CO_3 (8.3 g, 24.6 mmol), and $Pd(PPh_3)_4$ (520 mg, 0.45 mmol) were dissolved in a 150 mL mixture of 1,4-dioxane and water (10/1, v/v) under argon atmosphere. The mixture was stirred for 24 h at 90 °C and cooled down to room temperature. Afterward, the resulting mixture was mixed with 200 mL water, and then DCM was used to extract the desired product, which would be collected and evaporated under reduced pressure. The crude product was purified by flash chromatography on silica gel (PE/EtOAc) as white solid. Yield: 40% (3.0 g).

^{1}H NMR (500 MHz, DMSO) δ 8.44 (s, 1H), 7.61 – 7.56 (m, 4H), 7.22 – 7.14 (m, 4H), 6.87 (m, 4H), 3.86 (s, 12H), 3.69 (s, 6H).

^{13}C NMR (126 MHz, DMSO) δ 153.2, 142.6, 136.5, 136.0, 131.8, 127.5, 116.9, 103.4, 60.1, 55.9, 54.9.

HRMS (ESI): calculated for $[M+Na]^+$ $C_{30}H_{31}NO_6Na^+$ 524.2044; found 524.2046.

Ar^{6}DPA---bis(4'-methoxy-3',5'-dimethyl-[1,1'-biphenyl]-4-yl)amine

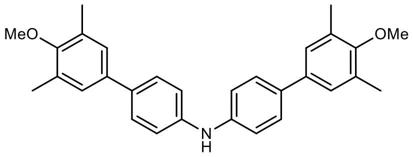

Bis(4-bromophenyl)amine (4.9 g, 15 mmol), (4-methoxy-3,5-dimethylphenyl)boronic acid (6.5 g, 36 mmol), K_2CO_3 (8.3 g, 24.6 mmol), and $Pd(PPh_3)_4$ (520 mg, 0.45 mmol) were dissolved in a 150 mL mixture of 1,4-dioxane and water (10/1, v/v) under argon atmosphere. The mixture was stirred for 24 h at 90 °C and cooled down to room temperature. Afterward, the resulting mixture was mixed with 200 mL water, and then DCM was used to extract the desired product, which would be collected and evaporated under reduced pressure. The crude product was purified by flash chromatography on silica gel (PE/DCM) as white solid. Yield: 85% (5.6 g).

^{1}H NMR (500 MHz, DMSO) δ 8.36 (s, 1H), 7.50 (d, *J* = 7.5 Hz, 4H), 7.28 – 7.24 (m, 4H), 7.15 (d, *J* = 7.8 Hz, 4H), 3.66 (s, 6H), 2.27 (s, 12H).

^{13}C NMR (126 MHz, DMSO) δ 155.6, 142.4, 135.5, 131.4, 130.5, 127.2, 126.2, 117.0, 59.3, 16.0.

HRMS (ESI): calculated for $[M+Na]^+$ $C_{30}H_{31}NO_2Na^+$ 460.2247; found 460.2261.

2.2.2. Synthesis of the photocatalysts

General Procedure A-PC for the synthesis of diphenylamine derived photocatalysts (0.5 mmol scale)

In a Schlenk flask equipped with a magnetic stir bar 5 equiv (2.5 mmol) of appropriate diphenyl amine (diphenyl amine, bis(4-methoxyphenyl)amine, 4,4'-di-*tert*-butyldiphenylamine, di([1,1'-biphenyl]-4-yl)amine or bis(4-bromophenyl)amine) was dissolved in 20 ml of dry DMF under argon. 8 equiv (4 mmol) of NaH (60% in oil) was added to the Schlenk flask in portions. The mixture was stirred at 50 °C until hydrogen evolution had stopped (usually around

1 hour). Then 1 eqiuv (0.5 mmol) of corresponding tetrafluoroterephthalonitrile/tetrafluorophthalonitrile/ tetrafluoroisophthalonitrile/pentafluorobezonitrile/tetrafluoroisonicotinonitrile was added to the mixture and it was stirred at 50 °C for 16 hours. The mixture was poured into 150 ml of brine. The resulting precipitate was filtered, washed with water, EtOH and sometimes with Et_2O. The remaining solid was either recrystallized from DCM/EtOH solution or dissolved in DCM and purified by column chromatography on silica gel.

General Procedure B-PC for the synthesis of carbazole-derived photocatalysts (0.5 mmol scale)

In a Schlenk flask equipped with a magnetic stir bar 5 equiv (2.5 mmol) of appropriate carbazole (9*H*-carbazole, 3,6-diphenyl-9*H*-carbazole, 3,6-di-*tert*-butyl-9*H*-carbazole) was dissolved in 20 ml of dry THF under argon. 8 equiv (4 mmol) of NaH (60% in oil) was added to the Schlenk flask in portions. The mixture was stirred at room temperature until hydrogen evolution had stopped (usually around 30 mins). Then 1 eqiuv (0.5 mmol) of corresponding tetrafluoroisophthalonitrile was added to the mixture, and it was stirred at room temperature for 16 hours. The reaction mixture was quenched by the addition of water. After the removal of THF, the residue was dissolved in DCM and washed with brine and water. The organic phase was dried over Na_2SO_4 and the solvent was removed under reduced pressure. The crude product was purified by flash chromatography on silica gel.

4CzIPN---2,4,5,6-tetra(9H-carbazol-9-yl)isophthalonitrile (PC1)

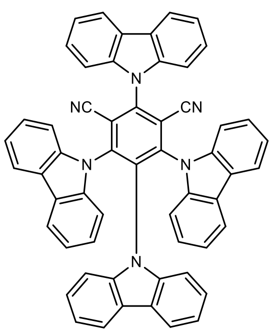

It was synthesized according to modified **General Procedure B-PC** in a 5 mmol scale. 150 ml of THF was used. After the synthesis, THF was removed, and then water was added to form a slurry, which was filtered and washed with water and EtOH. The resulting solid was recrystallized from DCM/EtOH. Yellow solid. Yield: 91% (3.6 g).

^{1}H NMR (500 MHz, $CDCl_3$) δ 8.25 (d, J = 7.7 Hz, 2H), 7.77 – 7.68 (m, 8H), 7.55 – 7.48 (m, 2H), 7.36 (d, J = 7.6 Hz, 2H), 7.26 – 7.22 (m, 4H), 7.11 (tt, J = 7.4, 5.8 Hz, 8H), 6.89 – 6.82 (m, 4H), 6.66 (td, J = 7.6, 7.2, 1.2 Hz, 2H).

^{13}C NMR (126 MHz, $CDCl_3$) δ 145.4, 144.8, 140.1, 138.3, 137.1, 134.9, 127.1, 125.9, 125.1, 124.9, 124.7, 124.0, 122.5, 122.1, 121.5, 121.1, 120.6, 119.8, 116.5, 111.8, 110.1, 109.6 (2C).

HRMS (ESI): calculated for $[M+Na]^+$ $C_{56}H_{32}N_6Na^+$ 811.2581; found 811.2542.

4PhCzIPN---2,4,5,6-tetrakis(3,6-diphenyl-9*H*-carbazol-9-yl)isophthalonitrile (PC2)

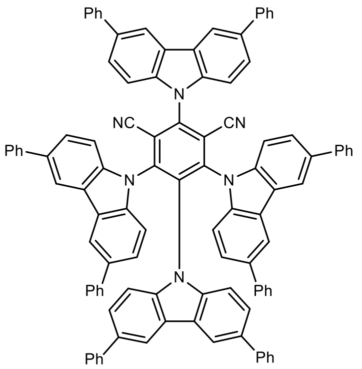

It was synthesized according to **General Procedure B-PC** in 0.5 mmol scale and purified by column chromatography (Hexanes/DCM). Yellow solid. Yield: 58% (0.41 g).

^{1}H NMR (500 MHz, Acetone) δ 8.83 (d, J = 1.8 Hz, 2H), 8.32 – 8.25 (m, 6H), 8.11 (dd, J = 8.5, 1.8 Hz, 2H), 7.96 – 7.88 (m, 8H), 7.84 (d, J = 1.8 Hz, 2H), 7.70 – 7.59 (m, 10H), 7.58 (s, 8H), 7.48 – 7.36 (m, 14H), 7.36 – 7.24 (m, 10H), 7.13 (dd, J = 8.5, 1.8 Hz, 2H).

^{13}C NMR (126 MHz, Acetone) δ 141.9, 141.2, 140.2, 135.9, 135.2, 129.8, 129.6, 129.5, 128.2, 127.9, 127.8, 127.7, 127.5, 127.3, 126.1, 125.9, 124.8, 120.6, 119.7, 119.1, 118.8, 112.7, 112.6, 112.0.

HRMS (ESI): calculated for $[M+Na]^+$ $C_{104}H_{64}N_6Na^+$ 1419.5085; found 1419.5130.

4*ᵗ*BuCzIPN---2,4,5,6-tetrakis(3,6-di-*tert*-butyl-9*H*-carbazol-9-yl)isophthalonitrile (PC3)

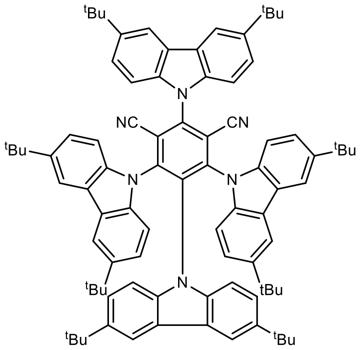

It was synthesized according to **General Procedure B-PC** in 0.5 mmol scale and purified by column chromatography (Hexanes/DCM). Yellow solid. Yield: 66% (0.41 g).

^{1}H NMR (500 MHz, CDCl$_3$) δ 8.24 – 8.19 (m, 2H), 7.79 – 7.72 (m, 2H), 7.66 – 7.58 (m, 6H), 7.22 – 7.15 (m, 2H), 7.10 – 6.98 (m, 8H), 6.55 – 6.40 (m, 4H), 1.54 (s, 18H), 1.30 (s, 36H), 1.23 (s, 18H).

^{13}C NMR (126 MHz, CDCl$_3$) δ 144.8, 137.1, 125.0, 124.6, 123.4, 122.1, 117.6, 116.2, 115.0, 109.9, 109.1, 109.0, 35.1, 34.7, 34.4, 32.1, 31.9, 31.8.

HRMS (ESI): calculated for $[M+Na]^+$ $C_{88}H_{96}N_6Na^+$ 1259.7589; found 1259.7544.

4BrDPAIPN---2,4,5,6-tetrakis(bis(4-bromophenyl)amino)isophthalonitrile (PC4)

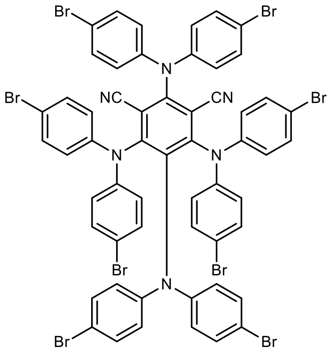

It was synthesized according to **General Procedure A-PC** in 0.5 mmol scale and purified by column chromatography (Hexanes/DCM). Orange solid. Yield: 51% (0.36 g).

^{1}H NMR (400 MHz, CD$_2$Cl$_2$) δ 6.28 – 6.20 (m, 4H), 6.12 – 6.03 (m, 8H), 5.95 – 5.87 (m, 4H), 5.74 – 5.69 (m, 4H), 5.37 – 5.29 (m, 8H), 5.22 – 5.15 (m, 4H).

^{13}C NMR (101 MHz, CD$_2$Cl$_2$) δ 153.6, 151.7, 144.5, 143.6, 142.2, 139.6, 133.2, 132.6, 131.6, 125.0, 124.4, 122.7, 118.3, 118.0, 116.3, 113.2, 112.9.

HRMS (ESI): calculated for $[M+Na]^+$ $C_{56}H_{32}Br_8N_6Na^+$ 1450.5966; found 1450.5939.

4DPAIPN---2,4,5,6-tetrakis(diphenylamino)isophthalonitrile (PC5)

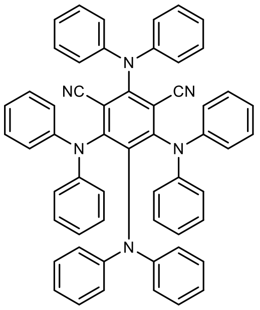

It was synthesized according to **General Procedure A-PC** in 0.5 mmol scale and purified by column chromatography (Hexanes/DCM). Orange solid. Yield: 61% (0.24 g).

^{1}H NMR (500 MHz, CDCl$_3$) δ 7.30 – 7.25 (m, 4H), 7.14 – 7.00 (m, 14H), 6.95 – 6.82 (m, 8H), 6.73 – 6.66 (m, 10H), 6.56 (d, *J* = 8.0 Hz, 4H).

^{13}C NMR (126 MHz, CDCl$_3$) δ 154.3, 151.9, 145.7, 144.8, 143.3, 140.4, 129.5, 128.7, 127.7, 124.3, 124.1, 123.1, 122.8, 122.7, 121.2, 113.3, 113.2.

HRMS (ESI): calculated for $[M+Na]^+$ $C_{56}H_{40}N_6Na^+$ 819.3207; found 819.3169.

4MeOCzIPN---2,4,5,6-tetrakis(3,6-dimethoxy-9*H*-carbazol-9-yl)isophthalonitrile (PC6)

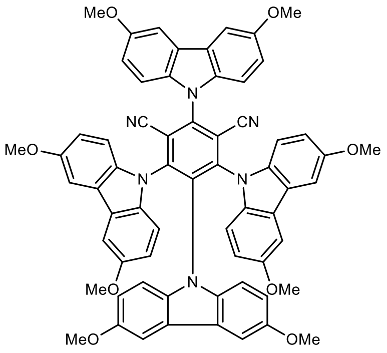

It was synthesized according to **General Procedure B-PC** in 0.5 mmol scale and purified by column chromatography (Hexanes/DCM). Orange solid. Yield: 52% (0.27 g).

^{1}H NMR (500 MHz, $CDCl_3$) δ 7.59 (d, *J* = 2.4 Hz, 2H), 7.52 (d, *J* = 8.8 Hz, 2H), 7.31 – 7.25 (m, 3H), 7.15 (d, *J* = 2.4 Hz, 4H), 7.04 (d, *J* = 8.9 Hz, 4H), 6.82 (d, *J* = 2.4 Hz, 2H), 6.73 – 6.64 (m, 6H), 3.98 (s, 6H), 3.79 (s, 12H), 3.69 (s, 6H).

^{13}C NMR (126 MHz, $CDCl_3$) δ 155.7, 155.3, 154.5, 145.2, 135.4, 133.9, 133.7, 132.6, 125.8, 125.4, 124.7, 115.9, 115.1, 114.6, 113.7, 112.1, 110.9, 110.7, 110.2, 104.2, 103.6, 102.9, 56.2, 56.0, 55.9.

HRMS (ESI): calculated for $[M+Na]^+$ $C_{64}H_{48}N_6O_8Na^+$ 1051.3426; found 1051.3383.

4PhDPAIPN---2,4,5,6-tetrakis(di([1,1'-biphenyl]-4-yl)amino) isophthalonitrile (PC7)

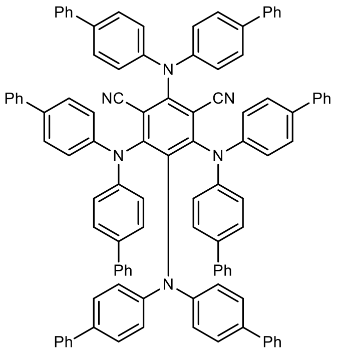

It was synthesized according to **General Procedure A-PC** in 0.5 mmol scale and recrystallized from DCM/EtOH Orange solid. Yield: 47% (0.33 g).

^{1}H NMR (500 MHz, CD_2Cl_2) δ 7.68 – 7.62 (m, 4H), 7.57 – 7.52 (m, 4H), 7.45 – 7.21 (m, 52H), 6.99 – 6.93 (m, 8H), 6.85 – 6.79 (m, 4H).

^{13}C NMR (126 MHz, CD_2Cl_2) δ 154.4, 145.2, 144.3, 142.8, 140.7, 140.6, 140.5, 137.4, 137.3, 135.8, 129.2, 129.1, 129.0, 128.5, 127.8, 127.6, 127.4, 127.2, 127.1, 126.8, 126.8, 123.7, 123.4, 121.7, 113.8, 113.5.

HRMS (ESI): calculated for $[M+Na]^+$ $C_{104}H_{72}N_6Na^+$ 1427.5711; found 1427.5687.

4^{t}BuDPAIPN---2,4,5,6-tetrakis(bis(4-(tert-butyl)phenyl)amino)isophthalonitrile (PC8)

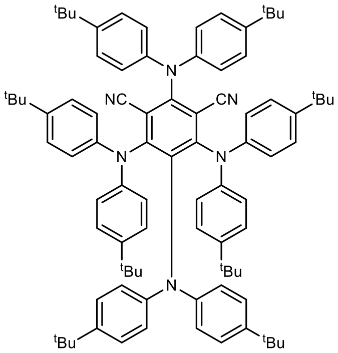

It was synthesized according to **General Procedure A-PC** in 0.5 mmol scale. The reaction was carried out for 72 h in 50 ml of DMF and purified by column chromatography (Hexanes/DCM). Yellow solid. Yield: 31% (0.19 g).

^{1}H NMR (500 MHz, $CDCl_3$) δ 7.32 – 7.28 (m, 4H), 7.06 (t, *J* = 9.1 Hz, 12H), 6.86 – 6.82 (m, 4H), 6.65 – 6.60 (m, 8H), 6.43 – 6.38 (m, 4H), 1.29 (s, 18H), 1.16 (s, 36H), 1.04 (s, 18H).

^{13}C NMR (126 MHz, $CDCl_3$) δ 154.7, 151.4, 146.3, 146.0, 144.8, 143.2, 141.9, 140.9, 126.1, 125.2, 124.1, 122.2, 121.7, 120.6, 114.2, 113.4, 34.4, 34.2, 34.0, 31.5, 31.4, 31.3.

HRMS (ESI): calculated for $[M+Na]^+$ $C_{88}H_{104}N_6Na^+$ 1267.8215; found 1267.8172.

4MeODPAIPN---2,4,5,6-tetrakis(bis(4-methoxyphenyl)amino)isophthalonitrile (PC9)

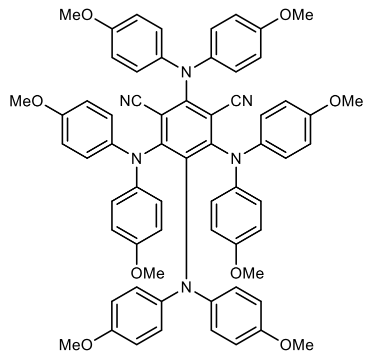

It was synthesized according to **General Procedure A-PC** in 0.5 mmol scale and purified by column chromatography (Hexanes/DCM). Orange solid. Yield: 44% (0.22 g).

^{1}H NMR (500 MHz, CDCl$_3$) δ 6.98 – 6.93 (m, 4H), 6.79 – 6.74 (m, 4H), 6.60 (s, 16H), 6.46 (s, 8H), 3.75 (s, 6H), 3.68 (s, 12H), 3.63 (s, 6H).

^{13}C NMR (126 MHz, CDCl$_3$) δ 156.0, 155.9, 154.8, 154.2, 152.1, 139.7, 139.2, 138.9, 137.7, 124.3, 124.0, 122.1, 114.7, 114.0, 113.0, 110.9, 55.6, 55.5 (2C).

HRMS (ESI): calculated for $[M+Na]^+$ $C_{64}H_{56}N_6O_8Na^+$ 1059.4052; found 1059.4008.

4MeODPAFBN---2,3,4,6-tetrakis(bis(4-methoxyphenyl)amino)-5-fluorobenzonitrile (PC10)

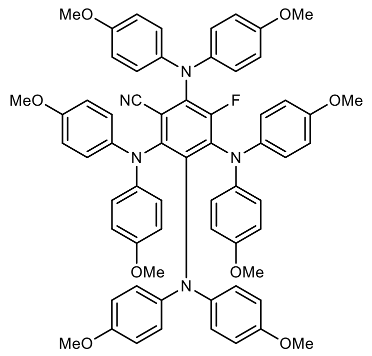

It was synthesized according to modified **procedure A-PC** in 0.8 mmol scale. It was used 10 equiv (8 mmol) of amine, 15 equv (12 mmol) of sodium hydride and reaction was carried out for 72 h. Purified by column chromatography (Hexanes/DCM) followed by recrystallization from DCM/EtOH. Orange solid. Yield: 25% (0.20 g).

^{1}H NMR (500 MHz, CDCl$_3$) δ 6.95 – 6.90 (m, 4H), 6.81 – 6.76 (m, 4H), 6.65 – 6.55 (m, 16H), 6.43 (s, 8H), 3.76 (s, 6H), 3.70 (d, J = 6.1 Hz, 12H), 3.61 (s, 6H).

^{13}C NMR (126 MHz, CDCl$_3$) δ 157.2, 155.6 (d, J = 3.3 Hz), 155.1, 154.9, 145.9, 141.7, 140.0, 139.7 (d, J = 9.1 Hz), 139.6, 139.1, 123.0 (d, J = 5.4 Hz), 122.8 (d, J = 5.6 Hz), 114.7, 114.4 (d, J = 4.1 Hz), 114.0, 113.9, 113.2, 55.6 (2C), 55.5.

^{19}F NMR (471 MHz, CDCl$_3$) δ -119.7.

HRMS (ESI): calculated for $[M]^+$ $C_{63}H_{56}N_5O_8F^+$ 1029.4107; found 1029.4059.

4MeODPAINN---2,3,5,6-tetrakis(bis(4-methoxyphenyl)amino)isonicotinonitrile (PC11)

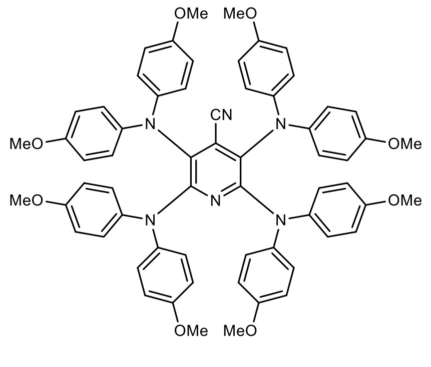

It was synthesized according to **General Procedure A-PC** in 0.5 mmol scale and purified by column chromatography (Hexanes/DCM) followed by recrystallization from DCM/EtOH. Red solid. Yield: 38% (0.19 g).). X-ray quality crystal **PC11** was grown from DCM/EtOH solution by slow evaporation. CCDC deposit number is 2370576.

^{1}H NMR (500 MHz, CD$_2$Cl$_2$) δ 6.68 (s, 16H), 6.57 – 6.51 (m, 16H), 3.72 (s, 12H), 3.67 (s, 12H).

^{13}C NMR (126 MHz, CD$_2$Cl$_2$) δ 156.3, 155.5, 151.4, 139.8, 139.3, 133.0, 125.6, 122.7, 114.2, 114.0, 55.7, 55.6.

HRMS (ESI): calculated for $[M+Na]^+$ $C_{62}H_{56}N_6O_8Na^+$ 1035.4052; found 1035.4005.

4MeODPAPN---3,4,5,6-tetrakis(bis(4-methoxyphenyl)amino)phthalonitrile (PC12)

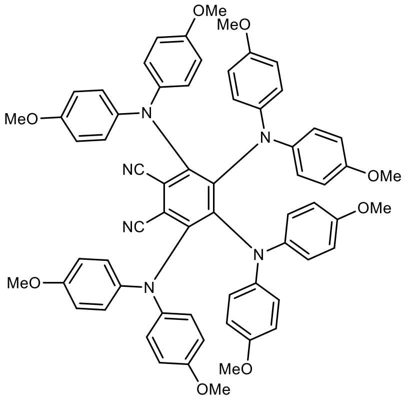

It was synthesized according to **General Procedure A-PC** in 0.5 mmol scale and purified by column chromatography (Hexanes/DCM) followed by recrystallization from DCM/EtOH. Red solid. Yield: 34% (0.18 g).

^{1}H NMR (500 MHz, CDCl$_3$) δ 6.74 – 6.62 (m, 16H), 6.44 – 6.31 (m, 16H), 3.73 (s, 12H), 3.60 (s, 12H).

^{13}C NMR (126 MHz, CDCl$_3$) δ 155.8, 155.5, 149.6, 147.5, 139.3, 138.3, 123.5, 123.4, 115.9, 114.1, 114.0, 113.0, 55.6 (2C).

HRMS (ESI): calculated for $[M+Na]^+$ $C_{64}H_{56}N_6O_8Na^+$ 1059.4052; found 1059.4018.

4MeODPATPN---2,3,5,6-tetrakis(bis(4-methoxyphenyl)amino)terephthalonitrile (PC13)

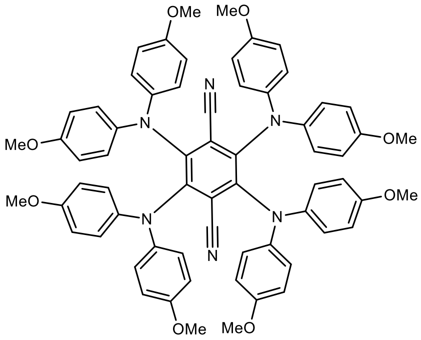

It was synthesized according to **General Procedure A-PC** in 0.5 mmol scale and purified by recrystallization from DCM/EtOH. Purple solid. Yield: 57% (0.30 g). X-ray quality crystal **PC13** was grown from DCM solution by slow evaporation. CCDC deposit number is 2353170.

^{1}H NMR (400 MHz, CD$_2$Cl$_2$) δ 6.69 – 6.66 (m, 28H), 3.70 (s, 24H).

^{13}C NMR (101 MHz, CD$_2$Cl$_2$) δ 155.9, 145.9, 139.2, 123.4, 121.9, 114.3, 55.7.

HRMS (ESI): calculated for $[M+Na]^+$ $C_{64}H_{56}N_6O_8Na^+$ 1059.4052; found 1059.4005.

5^{t}BuBN--- 2,3,4,5,6-pentakis(3,6-di-*tert*-butyl-9*H*-carbazol-9-yl)benzonitrile (PC14)

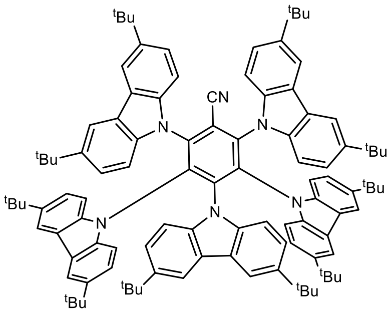

It was synthesized according to **General Procedure B-PC** in 0.5 mmol scale and purified by column chromatography (Hexanes/DCM). Yellow solid. Yield: 35% (0.26 g)

^{1}H NMR (500 MHz, CDCl$_3$) δ 7.68 – 7.60 (m, 4H), 7.32 – 7.21 (m, 6H), 7.16 – 7.07 (m, 2H), 7.07 – 6.95 (m, 8H), 6.79 – 6.68 (m, 4H), 6.68 – 6.52 (m, 6H), 1.39 (s, 36H), 1.28 (s, 36H), 1.18 (s, 18H).

^{13}C NMR (126 MHz, CDCl$_3$) δ 143.6, 143.3, 142.9, 142.8, 140.1, 137.7, 137.4, 136.6, 135.9, 124.7, 124.4, 124.1, 122.9, 122.4, 121.8, 116.4, 115.8, 115.1, 114.9, 113.5, 110.7, 110.0, 109.8, 34.6, 34.3 (2C), 32.0, 31.9, 31.7.

HRMS (ESI): calculated for $[M+Na]^+$ $C_{107}H_{120}N_6Na^+$ 1511.9467; found 1511.9465.

4tBuCzINN--- 2,3,5,6-tetrakis(3,6-di-*tert*-butyl-9*H*-carbazol-9-yl)isonicotinonitrile (PC15)

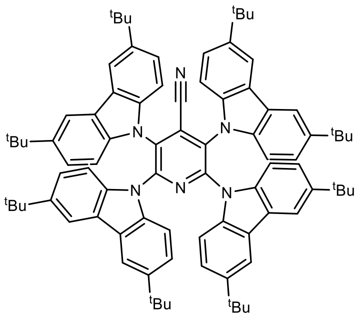

It was synthesized according to **General Procedure B-PC** in 0.5 mmol scale and purified by column chromatography (Hexanes/DCM). Orange solid. Yield: 33% (0.20 g)

^{1}H NMR (500 MHz, CD_2Cl_2) δ 7.75 (d, *J* = 1.9 Hz, 4H), 7.67 (d, *J* = 1.9 Hz, 4H), 7.25 (d, *J* = 8.6 Hz, 4H), 7.14 (dd, *J* = 8.6, 1.9 Hz, 4H), 7.08 (d, *J* = 8.5 Hz, 4H), 6.99 (dd, *J* = 8.6, 1.9 Hz, 4H), 1.41 (s, 36H), 1.38 (s, 36H).

^{13}C NMR (126 MHz, CD_2Cl_2) δ 147.2, 144.5 (2C) 137.0 (2C), 130.0, 126.1, 124.6, 124.5, 123.3, 123.1, 116.2, 115.6, 112.9, 110.0, 109.4, 34.6, 34.5, 31.6 (2C).

HRMS (ESI): calculated for $[M+Na]^+$ $C_{86}H_{96}N_6Na^+$ 1235.7589; found 1235.7602.

4DPAINN--- 2,3,5,6-tetrakis(diphenylamino)isonicotinonitrile (PC16)

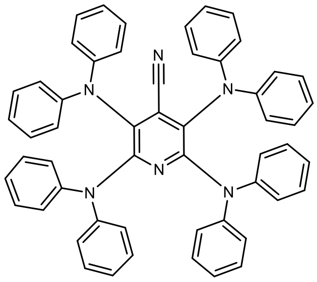

It was synthesized according to **General Procedure A-PC** in 0.5 mmol scale and purified by column chromatography (Hexanes/DCM) followed by recrystallization from DCM/EtOH. Orange solid. Yield: 33% (0.20 g)

^{1}H NMR (500 MHz, $CDCl_3$) δ 7.14 (t, *J* = 7.8 Hz, 8H), 6.99 (t, *J* = 7.7 Hz, 8H), 6.91 (t, *J* = 7.3 Hz, 4H), 6.85 (t, *J* = 7.3 Hz, 4H), 6.80 (d, *J* = 8.2 Hz, 8H), 6.62 (d, *J* = 7.9 Hz, 8H).

^{13}C NMR (126 MHz, $CDCl_3$) δ 151.1, 145.7, 144.9, 133.7, 128.8, 128.5, 126.1, 124.3, 123.8, 123.2, 121.6, 113.0.

HRMS (ESI): calculated for $[M+Na]^+$ $C_{54}H_{40}N_6Na^+$ 795.3207; found 795.3203.

4CzPN---3,4,5,6-tetra(9*H*-carbazol-9-yl)phthalonitrile (PC17)

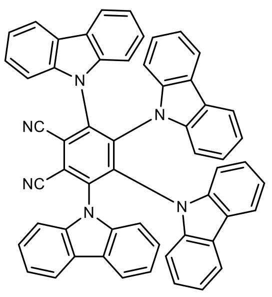

It was synthesized according to **General Procedure B-PC** in 0.5 mmol scale and purified by column chromatography (Hexanes/DCM). Yellow solid. Yield: 32% (0.13 g)

^{1}H NMR (500 MHz, DMSO) δ 7.91 – 7.83 (m, 4H), 7.74 – 7.66 (m, 4H), 7.42 – 7.34 (m, 8H), 7.17 – 7.07 (m, 8H), 6.77 – 6.68 (m, 4H), 6.63 – 6.55 (m, 4H).

^{13}C NMR (126 MHz, DMSO) δ 141.6, 141.1, 138.8, 137.5, 125.2, 124.1, 123.1, 122.8, 121.1, 120.5, 120.2, 119.3, 119.0, 113.5, 111.2, 111.1.

HRMS (ESI): calculated for $[M+Na]^+$ $C_{56}H_{32}N_6Na^+$ 811.2581; found 811.2576.

4^{t}BuCzPN---3,4,5,6-tetrakis(3,6-di-*tert*-butyl-9*H*-carbazol-9-yl)phthalonitrile (PC18)

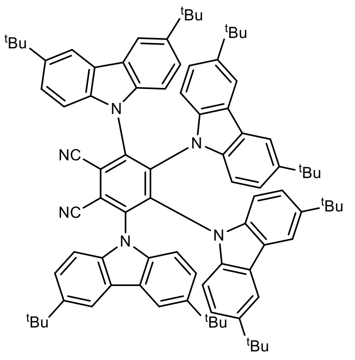

It was synthesized according to **General Procedure B-PC** in 0.5 mmol scale and purified by column chromatography (Hexanes/DCM). Yellow solid. Yield: 52% (0.32 g)

^{1}H NMR (500 MHz, $CDCl_3$) δ 7.64 – 7.56 (m, 4H), 7.01 – 6.89 (m, 4H), 6.81 (d, J = 8.6 Hz, 4H), 6.77 – 6.68 (m, 4H), 6.58 (d, J = 8.5 Hz, 4H), 1.38 (s, 36H), 1.21 (s, 36H).

^{13}C NMR (126 MHz, $CDCl_3$) δ 144.3, 143.9, 141.4, 139.8, 136.9, 136.3, 124.8, 124.5, 123.1, 122.7, 117.7, 116.0, 115.4, 113.4, 109.8, 109.5, 34.7, 34.5, 32.0, 31.8.

HRMS (ESI): calculated for $[M+Na]^+$ $C_{88}H_{96}N_6Na^+$ 1259.7589; found 1259.7553.

4DPAPN---3,4,5,6-tetrakis(diphenylamino)phthalonitrile (PC19)

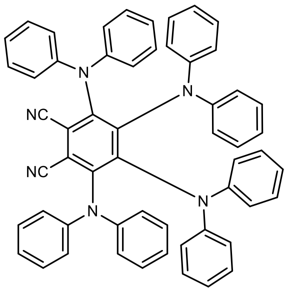

It was synthesized according to **General Procedure A-PC** in 0.5 mmol scale and by recrystallization from DCM/EtOH. Orange solid. Yield: 87% (0.35 g).

^{1}H NMR (400 MHz, $CDCl_3$) δ 7.19 (t, J = 7.7 Hz, 8H), 6.96 (t, J = 7.4 Hz, 4H), 6.85 (t, J = 7.7 Hz, 8H), 6.77 (d, J = 8.0 Hz, 8H), 6.69 (t, J = 7.3 Hz, 4H), 6.43 (d, J = 8.0 Hz, 8H).

^{13}C NMR (101 MHz, $CDCl_3$) δ 150.4, 148.0, 144.9, 143.9, 129.0, 127.7, 123.9, 123.6, 122.4, 122.3, 116.9, 113.6.

HRMS (ESI): calculated for $[M+Na]^+$ $C_{56}H_{40}N_6Na^+$ 819.3207; found 819.3207.

4^{t}BuCzTPN--- 2,3,5,6-tetrakis(3,6-di-*tert*-butyl-9*H*-carbazol-9-yl)terephthalonitrile (PC20)

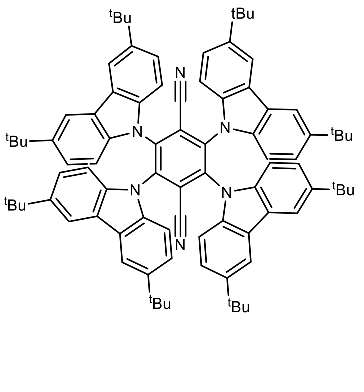

It was synthesized according to **General Procedure B-PC** in 0.5 mmol scale and purified by recrystallization from DCM/EtOH. Orange solid. Yield: 26% (0.16 g)

^{1}H NMR (500 MHz, CD_2Cl_2) δ 7.67 (d, J = 1.9 Hz, 8H), 7.12 (dd, J = 8.6, 1.9 Hz, 8H), 7.04 (d, J = 8.5 Hz, 8H), 1.40 (s, 72H).

^{13}C NMR: too low solubility

HRMS (ESI): calculated for $[M+Na]^+$ $C_{88}H_{96}N_6Na^+$ 1259.7589; found 1259.7573.

4MeOCzTPN---2,3,5,6-tetrakis(3,6-dimethoxy-9*H*-carbazol-9-yl)terephthalonitrile (PC21)

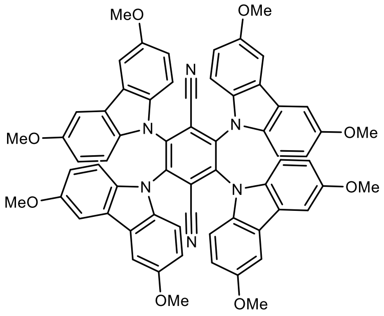

It was synthesized according to **General Procedure B-PC** in 0.5 mmol scale. Due to low solubility the solid was washed with ethanol, diethyl ether and acetone without futher purification. Purple solid. Yield: 68% (0.35 g)

^{1}H NMR (500 MHz, CD_2Cl_2) δ 7.27 (d, *J* = 2.5 Hz, 2H), 7.15 (d, *J* = 8.8 Hz, 2H), 6.83 (dd, *J* = 8.9, 2.5 Hz, 2H), 3.84 (s, 6H).

^{13}C NMR: too low solubility

HRMS (ESI): calculated for $[M+Na]^+$ $C_{64}H_{48}N_6O_8Na^+$ 1051.3426; found 1051.3381.

4PhDPATPN---2,3,5,6-tetrakis(di([1,1'-biphenyl]-4-yl)amino)terephthalonitrile (PC22)

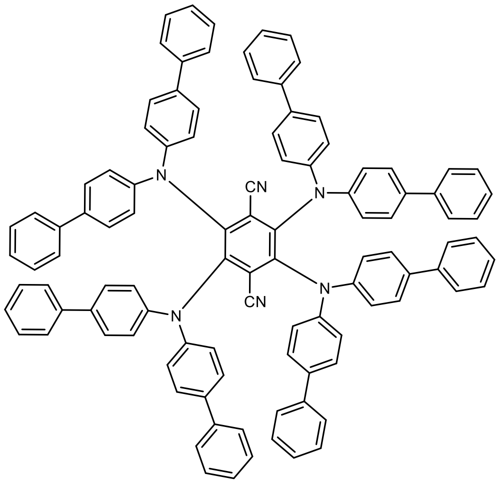

It was synthesized according to **General Procedure A-PC** in 0.5 mmol scale and purified by recrystallization from DCM/EtOH. Red solid. Yield: 85% (0.60 g)

^{1}H NMR (500 MHz, CD_2Cl_2) δ 7.46 (dd, *J* = 7.8, 5.1 Hz, 32H), 7.34 (t, *J* = 7.5 Hz, 16H), 7.28 (t, *J* = 7.3 Hz, 8H), 7.03 (d, *J* = 8.5 Hz, 16H).

^{13}C NMR (126 MHz, CD_2Cl_2) δ 146.1, 144.3, 140.6, 136.9, 129.1, 127.9, 127.4, 127.0, 122.7.

HRMS (ESI): calculated for $[M+Na]^+$ $C_{104}H_{72}N_6Na^+$ 1427.5711; found 1427.5683.

4^{t}BuDPATPN---2,3,5,6-tetrakis(bis(4-(*tert*-butyl)phenyl)amino)terephthalonitrile (PC23)

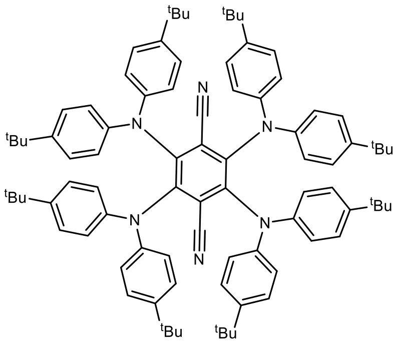

It was synthesized according to **General Procedure A-PC** in 0.5 mmol scale and purified by column chromatography (Hexanes/DCM) followed by recrystallization from DCM/EtOH. Red solid. Yield: 25% (0.16 g).

^{1}H NMR (500 MHz, $CDCl_3$) δ 7.09 (d, *J* = 8.8 Hz, 16H), 6.70 (d, *J* = 8.8 Hz, 16H), 1.17 (s, 72H).

^{13}C NMR (126 MHz, $CDCl_3$) δ 146.6, 145.7, 142.2, 125.4, 123.7, 121.3, 113.5, 34.2, 31.4.

HRMS (ESI): calculated for $[M+Na]^+$ $C_{88}H_{104}N_6Na^+$ 1267.8215; found 1267.8200.

4(Ar⁴DPA)TPN---2,3,5,6-tetrakis(bis(3',5'-dimethoxy-[1,1'-biphenyl]-4-yl)amino)terephthalonitrile (PC24)

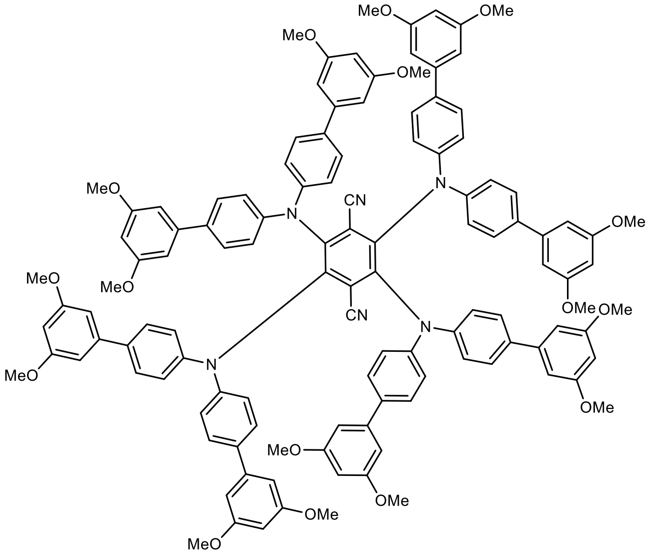

It was synthesized according to **General Procedure A-PC** in 0.5 mmol scale and purified by recrystallization from DCM/EtOH. Purple solid. Yield: 72% (0.69 g)

^{1}H NMR (500 MHz, CD_2Cl_2) δ 7.47 – 7.40 (m, 16H), 7.02 – 6.94 (m, 16H), 6.60 – 6.53 (m, 16H), 6.41 – 6.35 (m, 8H), 3.74 (s, 48H).

^{13}C NMR (126 MHz, CD_2Cl_2) δ 161.5, 146.5, 144.4, 142.7, 136.9, 128.0, 122.5, 105.2, 99.4, 55.6.

HRMS (ESI): calculated for $[M+Na]^+$ $C_{120}H_{104}N_6O_{16}Na^+$ 1907.7401; found 1907.7337.

4(Ar⁵DPA)TPN---2,3,5,6-tetrakis(bis(3',4',5'-trimethoxy-[1,1'-biphenyl]-4-yl)amino)terephthalonitrile (PC25)

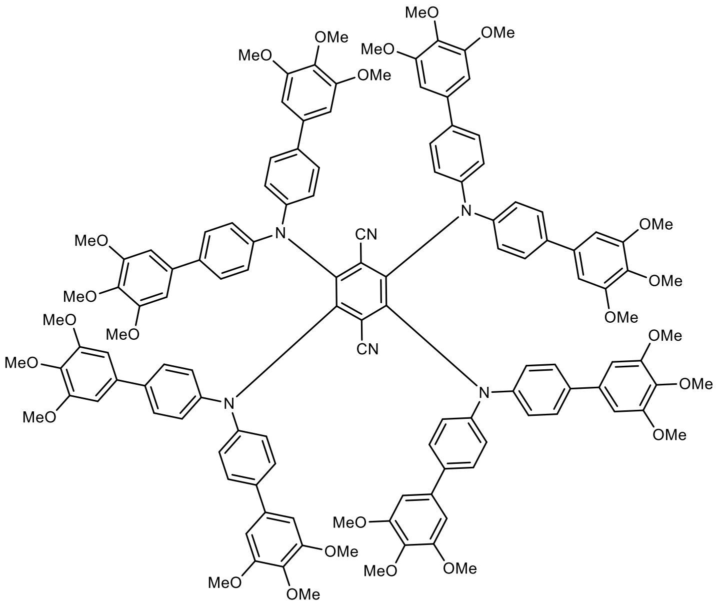

It was synthesized according to **General Procedure A-PC** in 0.5 mmol scale and purified by recrystallization from DCM/EtOH. Red solid. Yield: 79% (0.84 g)

^{1}H NMR (500 MHz, CD_2Cl_2) δ 7.45 – 7.39 (m, 16H), 7.04 – 6.98 (m, 16H), 6.62 (s, 16H), 3.78 (s, 24H), 3.75 (s, 48H).

^{13}C NMR (126 MHz, CD_2Cl_2) δ 153.6, 146.4, 143.8, 137.8, 136.7, 135.9, 127.5, 123.7, 122.1, 113.5, 104.1, 60.5, 56.0

HRMS (ESI): calculated for $[M+Na]^+$ $C_{128}H_{120}N_6O_{24}Na^+$ 2147.8246; found 2147.8317.

4ClCzIPN---2,4,5,6-tetrakis(3,6-dichloro-9*H*-carbazol-9-yl)isophthalonitrile (PC26)

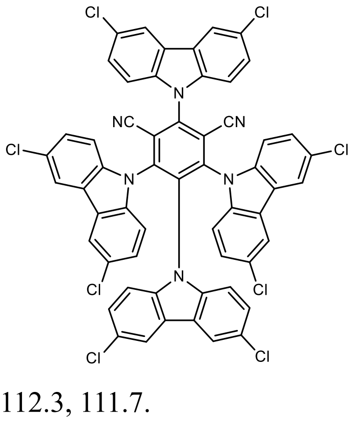

It was synthesized according to **General Procedure B-PC** in 0.5 mmol scale and purified by recrystallization from acetone. Yellow solid. Yield: 62% (0.32 g)

^{1}H NMR (500 MHz, DMSO) δ 8.58 (d, J = 2.0 Hz, 2H), 8.15 – 8.04 (m, 6H), 7.87 (dd, J = 8.7, 2.0 Hz, 2H), 7.78 (d, J = 2.0 Hz, 2H), 7.68 (d, J = 8.8 Hz, 4H), 7.46 (d, J = 8.8 Hz, 2H), 7.31 (dd, J = 8.8, 2.0 Hz, 4H), 6.92 (dd, J = 8.8, 2.0 Hz, 2H).

^{13}C NMR (126 MHz, DMSO) δ 145.0, 144.6, 138.6, 137.4, 136.5, 135.9, 127.8, 127.1, 126.5 (2C), 125.8, 125.3, 124.3, 123.9, 123.4, 121.7, 120.9, 120.3, 116.9, 112.6 (2C), 112.3, 111.7.

HRMS (ESI): calculated for $[M+Na]^+$ $C_{56}H_{24}Cl_8N_6Na^+$ 1082.9463; found 1082.9474.

4(Ar^{3}DPA)IPN---2,4,5,6-tetrakis(3,6-bis(4-(trifluoromethyl)phenyl)-9*H*-carbazol-9-yl)isophthalonitrile (PC27)

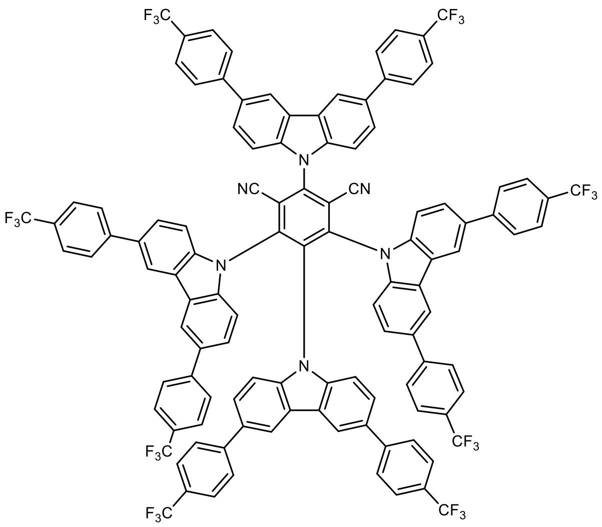

It was synthesized according to **General Procedure B-PC** in 0.5 mmol scale and purified by recrystallization from 1,2-dichloroethane/EtOH. Yellow solid. Yield: 46% (0.45 g)

^{1}H NMR (500 MHz, CD_2Cl_2) δ 8.64 (d, *J* = 1.8 Hz, 2H), 8.15 – 8.08 (m, 6H), 7.99 (d, *J* = 8.0 Hz, 4H), 7.94 (d, *J* = 8.5 Hz, 2H), 7.84 (d, *J* = 8.1 Hz, 4H), 7.67 (s, 18H), 7.60 (d, *J* = 8.1 Hz, 4H), 7.56 – 7.46 (m, 12H), 7.15 – 7.05 (m, 4H).

^{13}C NMR (126 MHz, CD_2Cl_2) δ 146.2, 145.0, 144.6, 144.5, 140.5, 139.5, 138.7, 136.5, 135.5, 134.9, 134.1, 129.6, 129.3, 128.2, 127.8, 127.6, 127.4, 126.4, 126.3, 126.2 (2C), 126.1 (2C), 126.0, 125.9 (2C), 125.4, 125.2, 120.9, 119.9, 119.2, 117.5, 112.0, 111.4, 111.2, 110.8.

^{19}F NMR (471 MHz, CD_2Cl_2) δ -62.6, -62.8, -62.8.

HRMS (ESI): calculated for $[M+Na]^+$ $C_{112}H_{56}F_{24}N_6Na^+$ 1963.4011; found 1963.4075.

4(Ar^{2}DPA)IPN---2,4,5,6-tetrakis(3,6-bis(4-cyanophenyl)-9*H*-carbazol-9-yl)isophthalonitrile (PC28)

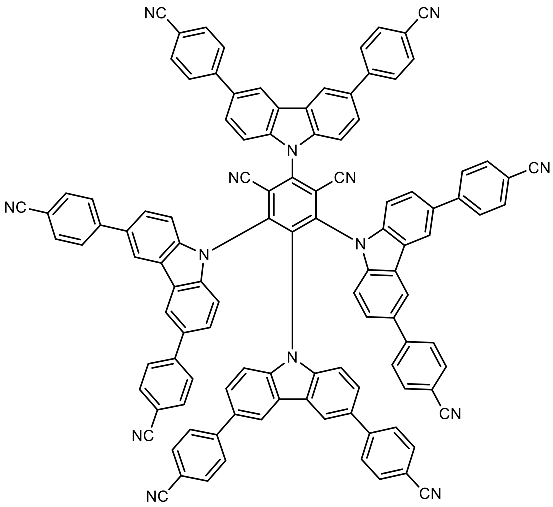

It was synthesized according to **General Procedure B-PC** in 0.5 mmol scale. Due to low solubility the solid was washed with ethanol, diethyl ether and acetone without futher purification. Yellow solid. Yield: 46% (0.37 g)

^{1}H NMR (500 MHz, DMSO) δ 9.05 – 9.02 (m, 2H), 8.58 – 8.44 (m, 4H), 8.44 – 8.33 (m, 2H), 8.30 – 8.22 (m, 2H), 8.20 – 8.11 (m, 4H), 8.09 – 7.98 (m, 10H), 7.92 – 7.83 (m, 16H), 7.83 – 7.79 (m, 4H), 7.78 – 7.73 (m, 2H), 7.73 – 7.67 (m, 4H), 7.67 – 7.62 (m, 4H), 7.29 – 7.22 (m, 2H).

^{13}C NMR (126 MHz, DMSO) δ 146.0, 144.9, 144.8, 144.6, 144.5, 140.5, 139.7, 139.2, 137.3, 133.0, 132.8 (2C), 132.6, 132.1, 131.5, 127.8, 127.7, 127.4, 127.3, 127.2 (2C), 126.5, 125.1, 124.6, 124.4, 124.2, 124.0, 120.6, 119.7, 119.0, 117.2, 112.3, 112.2, 111.8, 109.8, 109.5, 109.3, 109.2.

HRMS (ESI): calculated for $[M+Na]^+$ $C_{112}H_{56}N_{14}Na^+$ 1619.4705; found 1619.4652.

4(MeOPhNAr²)IPN---2,4,5,6-tetrakis((4-cyanophenyl)(4-methoxyphenyl)amino)isophthalonitrile (PC29)

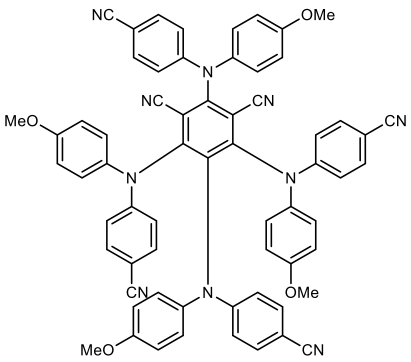

It was synthesized according to **General Procedure A-PC** in 0.5 mmol scale and purified by column chromatography (Hexanes/DCM). Orange solid. Yield: 39% (0.20 g)

^{1}H NMR (500 MHz, CD_2Cl_2) δ 7.59 – 7.52 (m, 2H), 7.36 – 7.31 (m, 1H), 7.20 – 7.12 (m, 4H), 7.02 – 6.77 (m, 12H), 6.67 – 6.15 (m, 10H), 3.84 (s, 3H), 3.75 (s, 6H), 3.64 (s, 3H).

^{13}C NMR (126 MHz, CD_2Cl_2) δ 158.8, 151.5, 150.2, 147.8, 136.3, 134.1, 133.4, 132.6, 127.7, 120.2, 119.2, 119.1, 119.0, 115.7, 115.5, 114.3, 113.1, 56.1, 56.0, 55.9.

HRMS (ESI): calculated for $[M+Na]^+$ $C_{64}H_{44}N_{10}O_4Na^+$ 1039.3439; found 1039.3426.

4DPAFBN---2,3,4,6-tetrakis(diphenylamino)-5-fluorobenzonitrile (PC30)

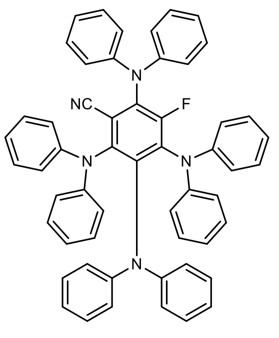

It was synthesized according to **General Procedure A-PC** in 0.5 mmol scale and purified by column chromatography (Hexanes/DCM). Yellow solid. Yield: 41% (0.16 g)

^{1}H NMR (500 MHz, $CDCl_3$) δ 7.33 – 7.22 (m, 4H), 7.15 – 6.97 (m, 14H), 6.94 – 6.80 (m, 8H), 6.77 – 6.63 (m, 10H), 6.57 – 6.47 (m, 4H).

^{13}C NMR (126 MHz, $CDCl_3$) δ 157.7, 155.7, 146.1 (d, J = 3.1 Hz), 145.9, 145.3, 145.2, 144.6, 139.9 (d, J = 10.4 Hz), 135.7 (d, J = 13.4 Hz), 129.4, 128.8, 128.6, 127.9, 123.6, 123.4, 123.0, 122. 7, 121.9, 121.9, 121.7, 121.6, 114.5 (d, J = 4.9 Hz), 113.8 (d, J = 4.2 Hz).

^{19}F NMR (471 MHz, $CDCl_3$) δ -117.56.

HRMS (ESI): calculated for $[M+Na]^+$ $C_{55}H_{40}N_5FNa^+$ 812.3160; found 812.3198.

5CzBN---2,3,4,5,6-penta(9*H*-carbazol-9-yl)benzonitrile (PC31)

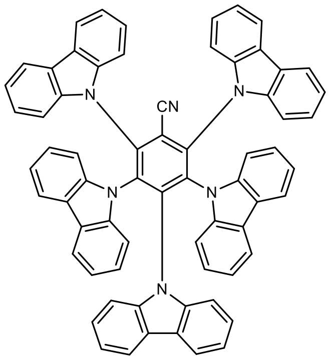

It was synthesized according to **General Procedure B-PC** in 0.5 mmol scale and purified by column chromatography (Hexanes/DCM). Yellow solid. Yield: 68% (0.32 g)

^{1}H NMR (500 MHz, CD_2Cl_2) δ 7.83 – 7.78 (m, 4H), 7.40 – 7.30 (m, 10H), 7.23 – 7.17 (m, 2H), 7.16 – 7.07 (m, 12H), 6.85 – 6.74 (m, 6H), 6.69 – 6.59 (m, 6H).

^{13}C NMR (126 MHz, CD_2Cl_2) δ 142.1, 140.8, 139.3, 138.2, 138.1, 137.2, 125.9, 124.9, 124.9, 124.3, 124.0, 123.9, 121.6, 121.1, 121.0, 120.6, 119.8, 117.2, 113.3, 111.2, 110.9, 110.8.

HRMS (ESI): calculated for $[M+Na]^+$ $C_{67}H_{40}N_6Na^+$ 951.3207; found 951.3191.

4CzTPN--- 2,3,5,6-tetra(9*H*-carbazol-9-yl)terephthalonitrile (PC32)

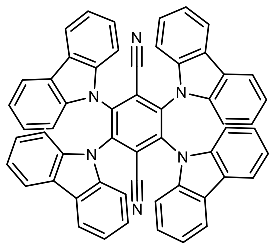

It was synthesized according to **General Procedure B-PC** in 0.5 mmol scale and purified by recrystallization from DCM/Acetone. Orange solid. Yield: 32% (0.17 g)

^{1}H NMR (500 MHz, CD_2Cl_2) δ 7.84 – 7.77 (m, 8H), 7.37 – 7.30 (m, 8H), 7.26 – 7.16 (m, 16H).

^{13}C NMR: too low solubility

HRMS (ESI): calculated for $[M+Na]^+$ $C_{56}H_{32}N_6Na^+$ 811.2581; found 811.2572.

4DPATPN--- 2,3,5,6-tetrakis(diphenylamino)terephthalonitrile (PC33)

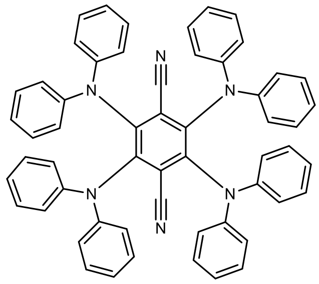

It was synthesized according to **General Procedure A-PC** in 0.5 mmol scale and purified by recrystallization from $CHCl_3$/Acetone. Red solid. Yield: 55% (0.43 g)

^{1}H NMR (500 MHz, $CDCl_3$) δ 7.12 (t, *J* = 7.7 Hz, 16H), 6.88 (t, *J* = 7.3 Hz, 8H), 6.78 (d, *J* = 7.9 Hz, 16H).

^{13}C NMR (126 MHz, $CDCl_3$) δ 146.1, 144.8, 128.9, 123.7, 122.6, 122.2, 113.4.

HRMS (ESI): calculated for $[M+Na]^+$ $C_{56}H_{40}N_6Na^+$ 819.3207; found 819.3187.

4(MeOPhNAr^{1})IPN---2,4,5,6-tetrakis((3,5-bis(trifluoromethyl)phenyl)(4-methoxyphenyl)amino)isophthalonitrile (PC34)

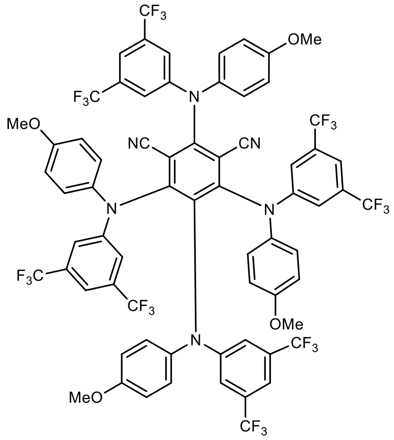

It was synthesized according to **General Procedure A-PC** in 0.5 mmol scale and purified by column chromatography (Hexanes/DCM). Orange solid. Yield: 46% (0.41 g)

^{1}H NMR (500 MHz, CD_2Cl_2) δ 7.54 (s, 1H), 7.37 (s, 2H), 7.32 (s, 2H), 7.20 (s, 1H), 7.15 – 7.10 (m, 2H), 7.06 – 6.57 (m, 20H), 3.83 (s, 3H), 3.77 (s, 6H), 3.66 (s, 3H).

^{13}C NMR (126 MHz, CD_2Cl_2) δ 159.3, 158.9, 158.0, 154.0, 151.9, 148.0, 147.6, 145.9, 141.1, 136.8, 135.6, 134.8, 133.9, 133.6, 133.4, 133.3, 133.0, 133.0, 132.7, 132.4, 132.4, 132.1, 131.9, 131.6, 127.2, 126.9, 126.6, 126.5, 124.4, 124.4, 124.0, 122.3, 122.2, 121.1, 120.1, 120.0, 118.5, 118.3, 117.5, 116.5, 116.1, 115.9, 115.8, 115.0, 113.5, 112.9, 55.9, 55.9, 55.6..

^{19}F NMR (471 MHz, CD_2Cl_2) δ -63.48, -63.66.

HRMS (ESI): calculated for $[M+Na]^+$ $C_{68}H_{40}F_{24}N_6O_4Na^+$ 1483.2620; found 1483.2612.

4(Ar^{1}Cz)IPN---2,4,5,6-tetrakis(3,6-bis(3,5-bis(trifluoromethyl)phenyl)-9*H*-carbazol-9-yl)isophthalonitrile (PC35)

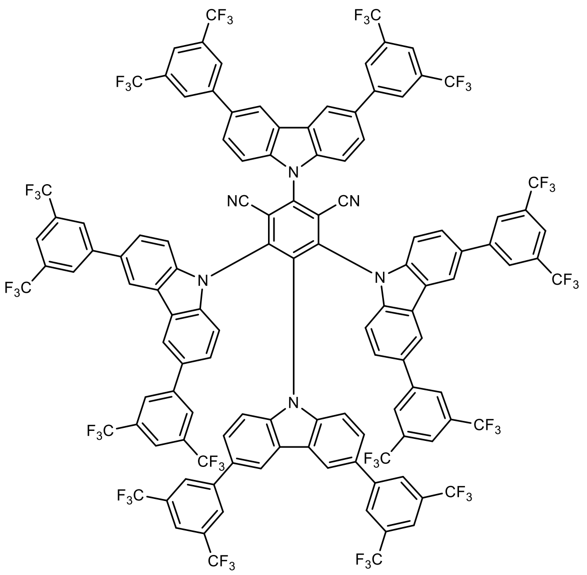

It was synthesized according to **General Procedure B-PC** in 0.5 mmol scale and purified by recrystallization from 1,2-dichloroethane/EtOH. Yellow solid. Yield: 38% (0.47 g)

^{1}H NMR (500 MHz, Acetone) δ 9.11 (s, 2H), 8.62 (d, *J* = 1.5 Hz, 4H), 8.53 (s, 4H), 8.35 – 8.31 (m, 4H), 8.22 (d, *J* = 10.4 Hz, 10H), 8.11 (s, 2H), 8.06 – 7.97 (m, 12H), 7.93 (s, 2H), 7.87 – 7.81 (m, 6H), 7.46 (dd, *J* = 8.6, 1.7 Hz, 2H).

^{13}C NMR (126 MHz, Acetone) δ 47.1, 146.4, 144.5, 144.1, 144.0, 141.9, 141.0, 140.3, 137.8, 133.6, 133.2, 133.1, 133.0, 132.9, 132.8, 132.8, 132.7, 132.5, 132.5, 132.4, 132.3, 132.2, 128.6, 128.6, 128.1, 128.1, 128.0, 127.9, 127.8, 127.8, 127.7, 127.6, 126.7, 126.4, 126.2, 126.0, 125.7, 125.7, 125.5, 125.5, 123.6, 123.4, 123.3, 121.9, 121.6, 121.5, 121.5, 121.4, 121.4, 121.3, 121.2, 121.2, 121.2, 121.1, 120.2, 119.0, 113.1, 113.0, 113.0, 112.5.

^{19}F NMR (471 MHz, Acetone) δ -63.2, -63.4, -63.5.

HRMS (ESI): calculated for $[M+H]^+$ $C_{120}H_{49}F_{48}N_6^+$ 2485.3247; found 2485.3318.

4(Ar^{6}DPA)TPN--- 2,3,5,6-tetrakis(bis(4'-methoxy-3',5'-dimethyl-[1,1'-biphenyl]-4-yl)amino)terephthalonitrile (PC36)

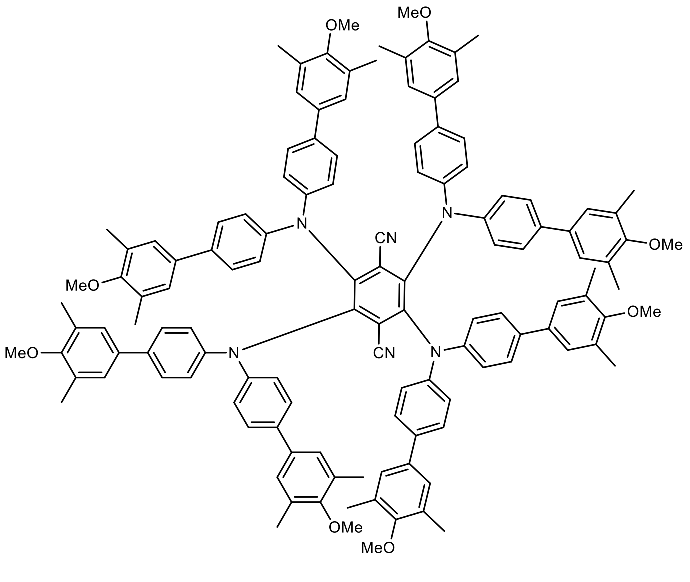

It was synthesized according to **General Procedure A-PC** in 0.5 mmol scale and purified by recrystallization from DCM/EtOH. Red solid. Yield: 85% (0.8 g)

^{1}H NMR (500 MHz, CD_2Cl_2) δ 7.40 – 7.35 (m, 16H), 7.09 (s, 16H), 6.99 – 6.93 (m, 16H), 3.70 (s, 24H), 2.23 (s, 48H).

^{13}C NMR (126 MHz, CD_2Cl_2) δ 156.9, 146.4, 143.9, 136.6, 136.1, 131.5, 127.6, 127.4, 123.9, 122.5, 113.9, 60.0, 16.3.

HRMS (ESI): calculated for $[M+Na]^+$ $C_{128}H_{120}N_6O_8Na^+$ 1891.9060; found 1891.9020.

4(2MeODPA)TPN---2,3,5,6-tetrakis(bis(3,5-dimethoxyphenyl)amino)terephthalonitrile (PC37)

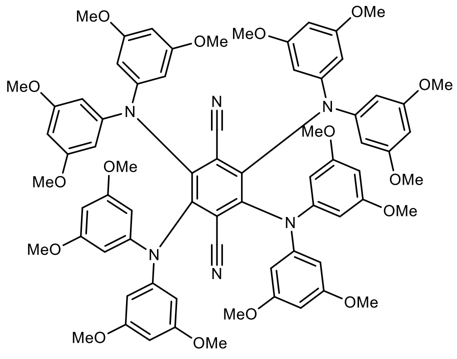

It was synthesized according to **General Procedure A-PC** in 0.5 mmol scale and purified by recrystallization from DCM/EtOH. Red solid. Yield: 31% (0.84 g)

^{1}H NMR (500 MHz, CD_2Cl_2) δ 6.09 – 6.04 (m, 8H), 6.04 – 5.98 (m, 16H), 3.66 (s, 48H).

^{13}C NMR (126 MHz, CD_2Cl_2) δ 161.4, 147.3, 146.7, 123.7, 113.1, 101.5, 95.8, 55.5.

HRMS (ESI): calculated for $[M+Na]^+$ $C_{72}H_{72}N_6O_{16}Na^+$ 1299.4897; found 1299.4835.

3CzBN-3,4,5---3,4,5-tri(9H-carbazol-9-yl)benzonitrile (PC38)

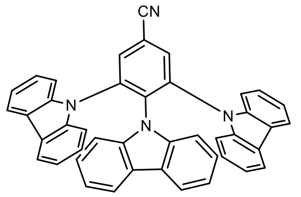

It was synthesized according to **General Procedure B-PC** in 0.5 mmol scale and purified by column chromatography (Hexanes/DCM). Pale yellow solid. Yield: 50% (0.15 g). The spectral data are consistent with those reported in the literature. [5]

^{1}H NMR (400 MHz, $CDCl_3$) δ 8.36 (s, 2H), 7.78 (d, *J* = 7.2 Hz, 4H), 7.37 (d, *J* = 7.6 Hz, 2H), 7.19 (d, *J* = 7.8 Hz, 4H), 7.09 – 6.98 (m, 8H), 6.92 (d, *J* = 8.2 Hz, 2H), 6.77 (t, *J* = 7.4 Hz, 2H), 6.64 (t, *J* = 7.7 Hz, 2H).

3CzBN-2,4,6---2,4,6-tri(9H-carbazol-9-yl)benzonitrile (PC39)

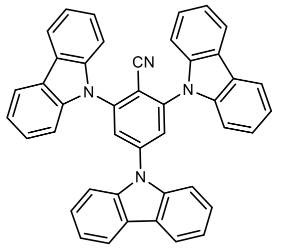

It was synthesized according to **General Procedure B-PC** in 0.5 mmol scale and purified by column chromatography (Hexanes/DCM). Pale yellow solid. Yield: 67% (0.2 g). The spectral data are consistent with those reported in the literature.[6]

^{1}H NMR (400 MHz, $CDCl_3$) δ 8.20 (d, *J* = 7.7 Hz, 4H), 8.13 (d, *J* = 7.7 Hz, 2H), 8.07 (s, 2H), 7.67 (d, *J* = 8.2 Hz, 2H), 7.57 (d, *J* = 6.3 Hz, 8H), 7.50 – 7.32 (m, 8H).

2CzIPN---4,6-di(9H-carbazol-9-yl)isophthalonitrile (PC40)

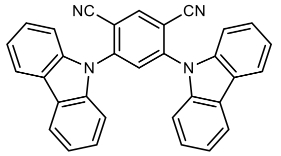

It was synthesized according to **General Procedure B-PC** in 0.5 mmol scale and purified by column chromatography (Hexanes/DCM). Yellow solid. Yield: 74% (0.17 g). The spectral data are consistent with those reported in the literature. [7]

^{1}H NMR (500 MHz, $CDCl_3$) δ 8.49 (s, 1H), 8.14 (d, *J* = 7.7 Hz, 4H), 7.96 (s, 1H), 7.49 (t, *J* = 7.7 Hz, 4H), 7.41 – 7.34 (m, 8H).

2CzPN---4,5-di(9H-carbazol-9-yl)phthalonitrile (PC41)

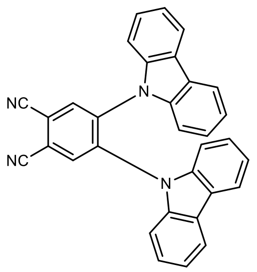

It was synthesized according to **General Procedure B-PC** in 0.5 mmol scale and purified by column chromatography (Hexanes/DCM). Yellow solid. Yield: 43% (0.10 g). The spectral data are consistent with those reported in the literature. [8]

^{1}H NMR (500 MHz, $CDCl_3$) δ 8.32 (s, 2H), 7.80 (d, *J* = 7.6 Hz, 4H), 7.14 – 7.04 (m, 12H).

2.2.3. Cyclic voltammograms of the donors and cores

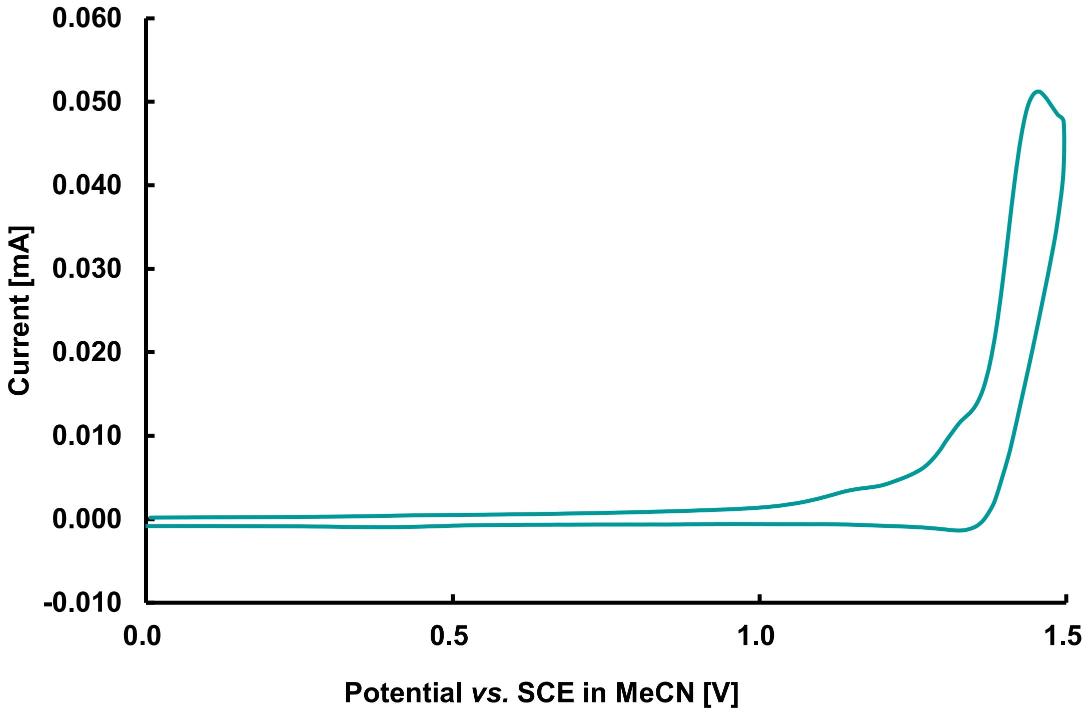

Figure S21. Cyclic voltammetry of **ClCz** in degassed MeCN (1 mM) using 0.1 M $^{n}Bu_4NPF_6$ as electrolyte.

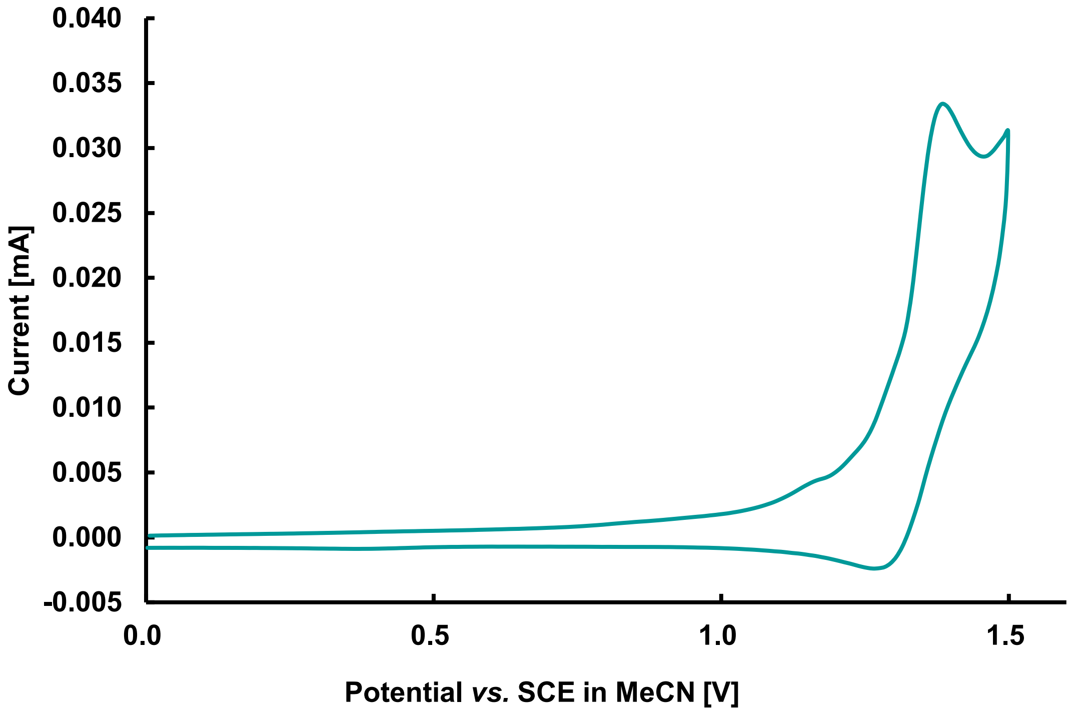

Figure S22. Cyclic voltammetry of **Ar^{1}Cz** in degassed MeCN (1 mM) using 0.1 M $^{n}Bu_4NPF_6$ as electrolyte.

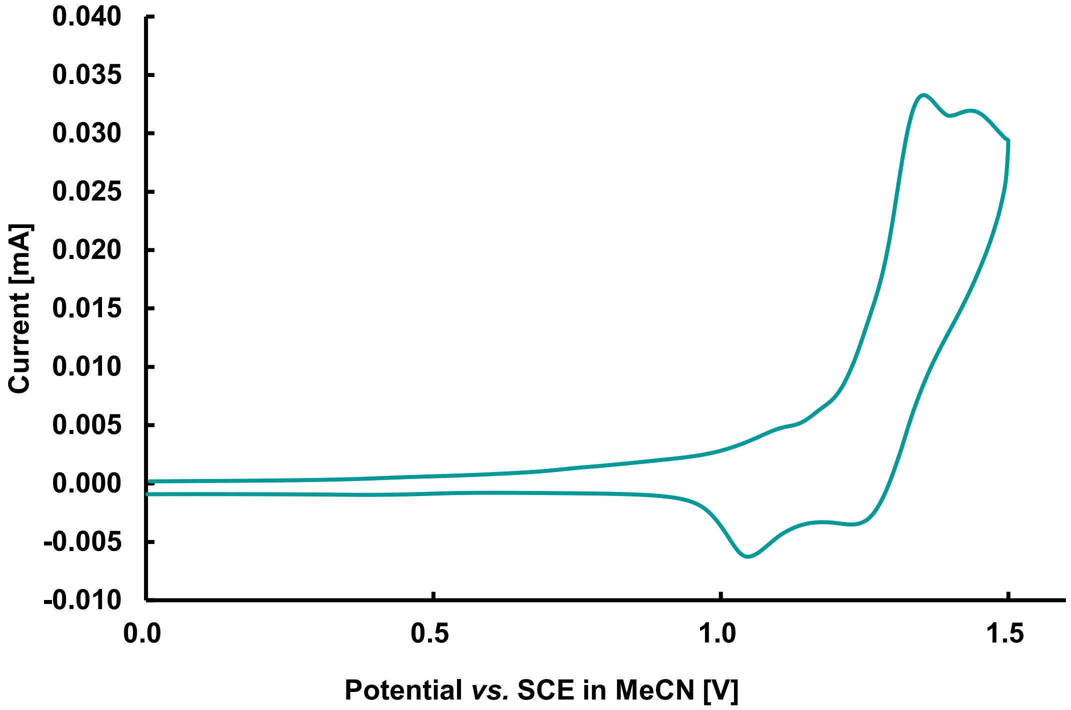

Figure S23. Cyclic voltammetry of **Ar²Cz** in degassed MeCN (1 mM) using 0.1 M $^{n}Bu_4NPF_6$ as electrolyte.

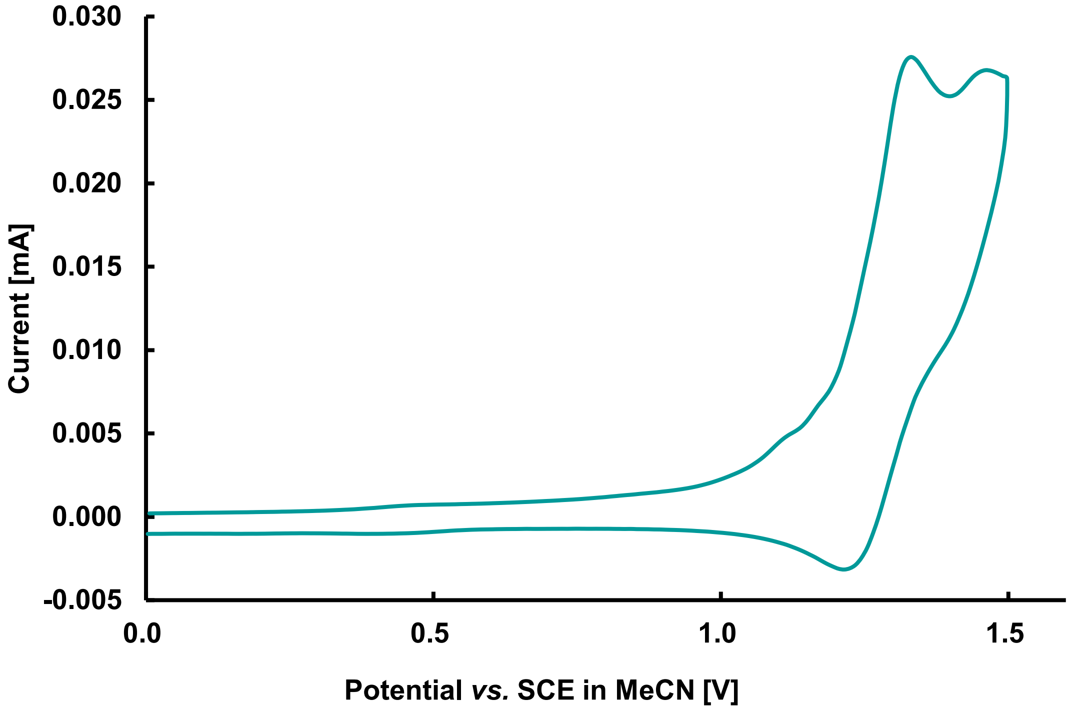

Figure S24. Cyclic voltammetry of **Ar³Cz** in degassed MeCN (1 mM) using 0.1 M $^{n}Bu_4NPF_6$ as electrolyte.

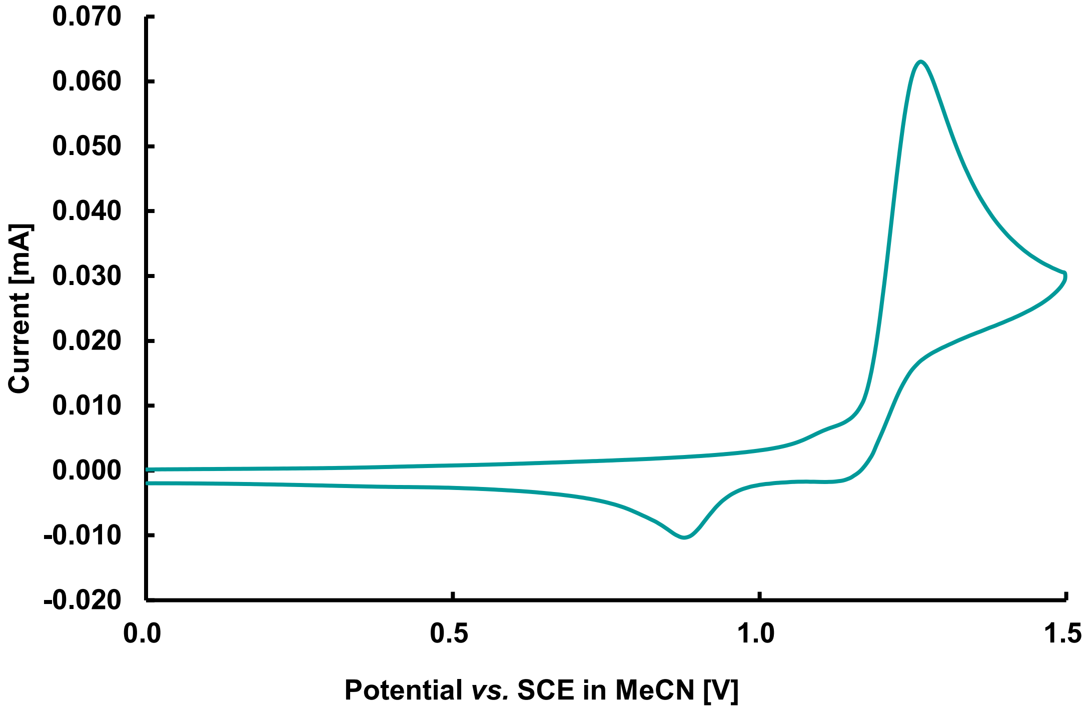

Figure S25. Cyclic voltammetry of **Cz** in degassed MeCN (1 mM) using 0.1 M $^{n}Bu_4NPF_6$ as electrolyte.

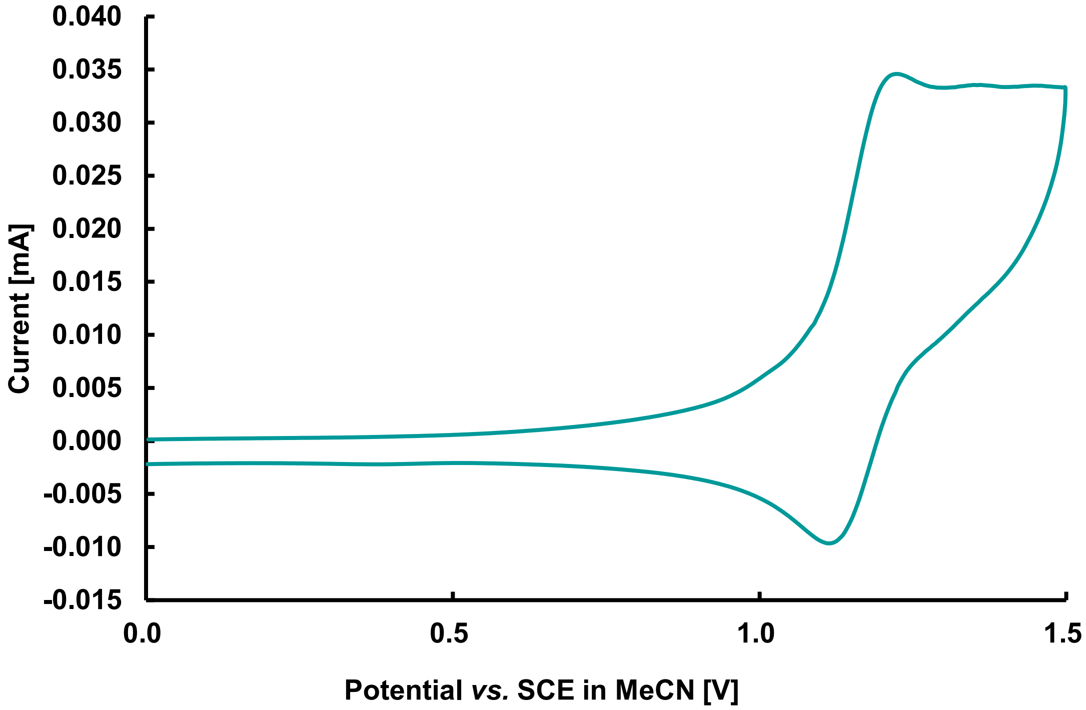

Figure S26. Cyclic voltammetry of **PhCz** in degassed MeCN (1 mM) using 0.1 M $^{n}Bu_4NPF_6$ as electrolyte.

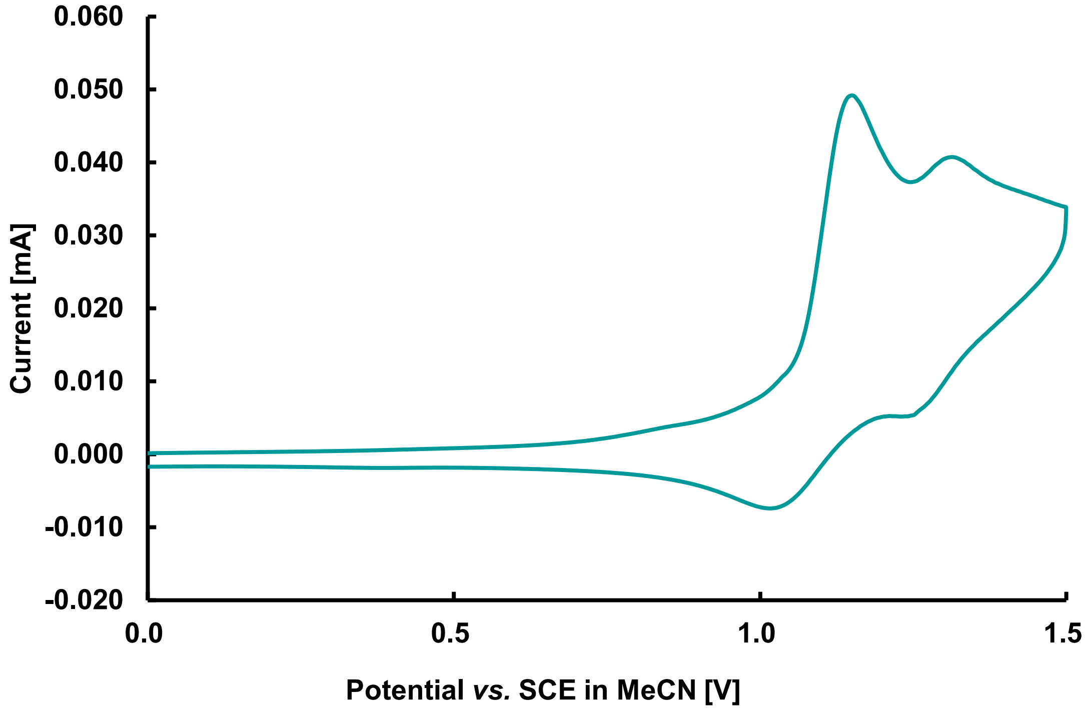

Figure S27. Cyclic voltammetry of **tBuCz** in degassed MeCN (1 mM) using 0.1 M $^{n}Bu_4NPF_6$ as electrolyte.

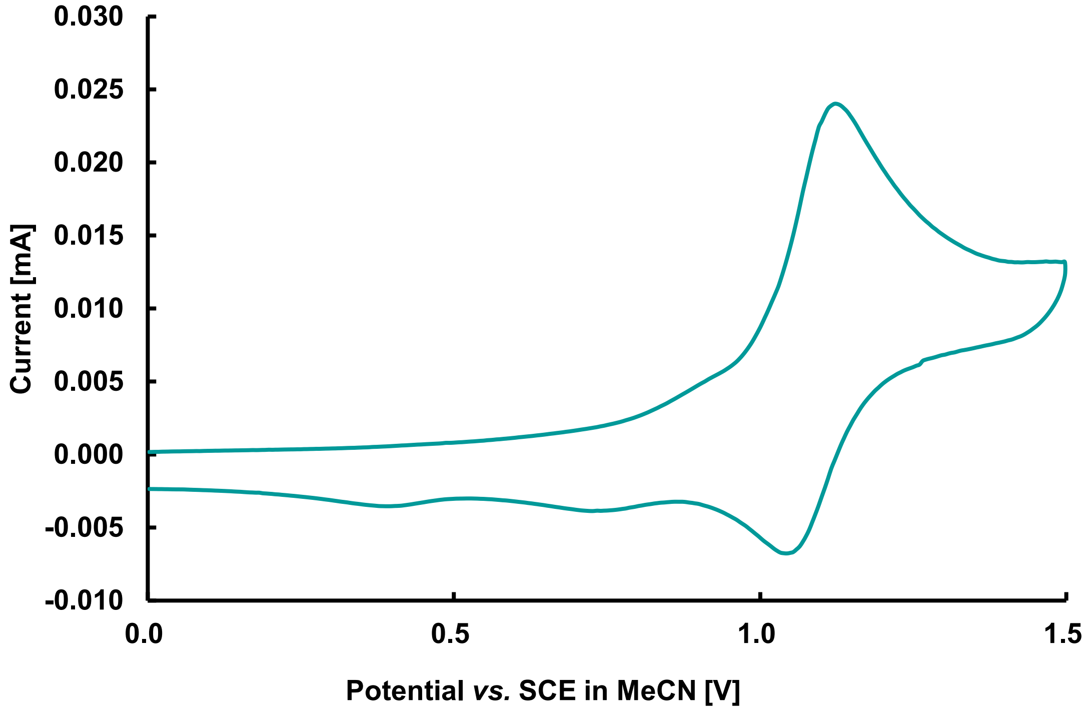

Figure S28. Cyclic voltammetry of **BrDPA** in degassed MeCN (1 mM) using 0.1 M $^{n}Bu_4NPF_6$ as electrolyte.

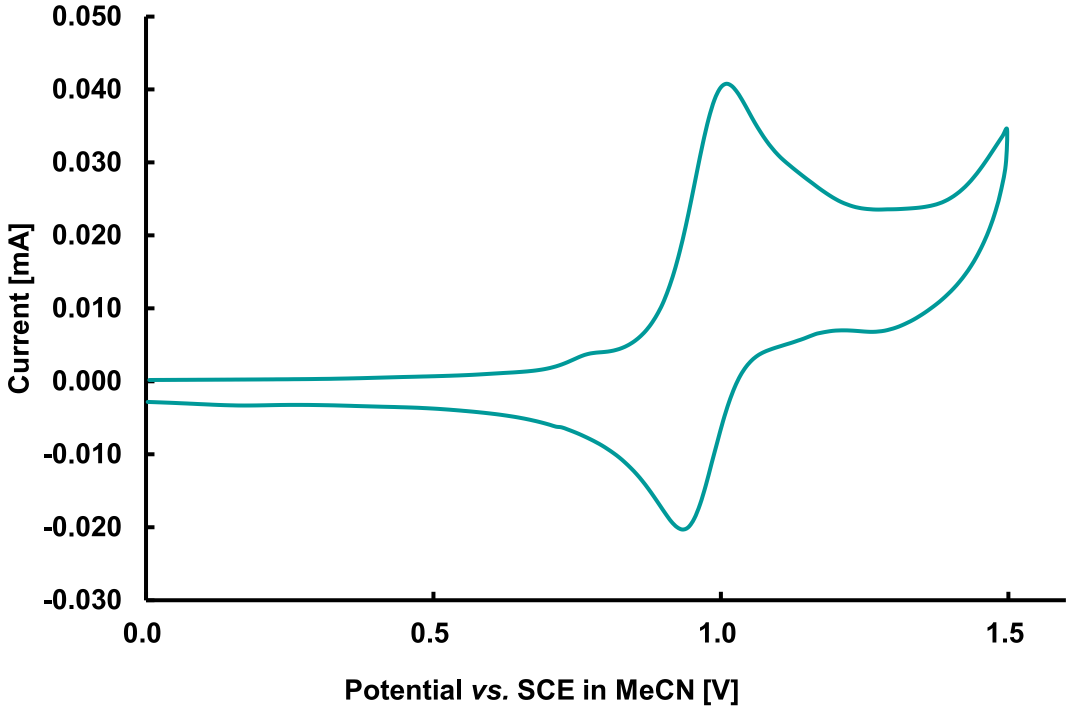

Figure S29. Cyclic voltammetry of **MeOPhNHAr^1** in degassed MeCN (1 mM) using 0.1 M ^{n}Bu$_4$NPF$_6$ as electrolyte.

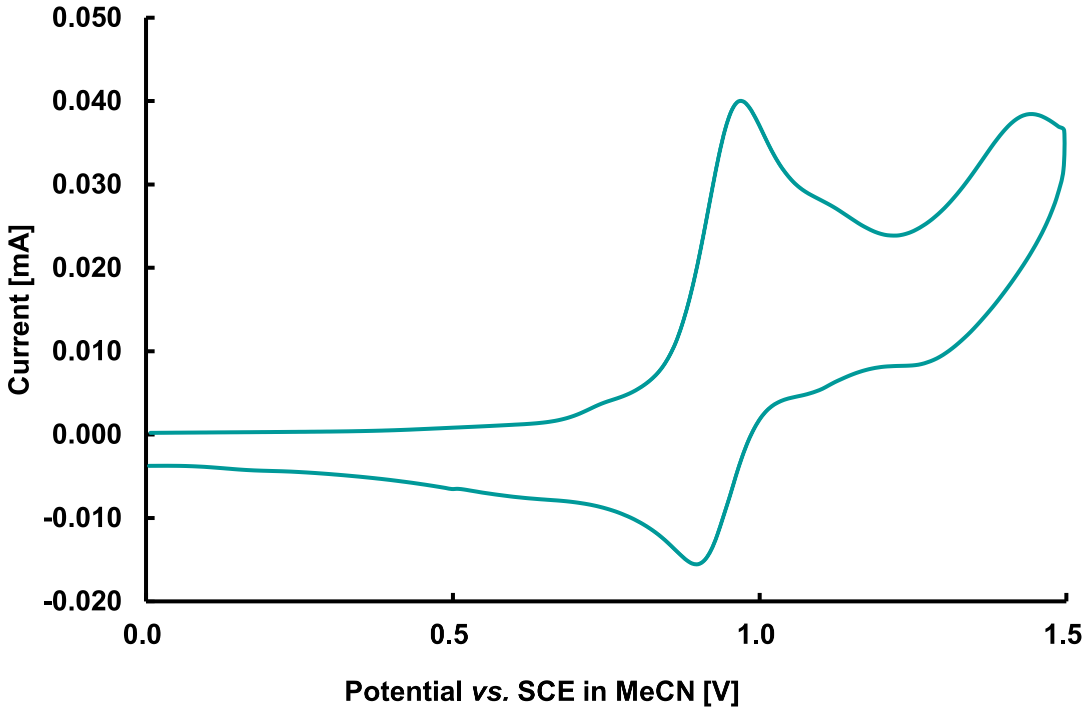

Figure S30. Cyclic voltammetry of **MeOPhNHAr^2** in degassed MeCN (1 mM) using 0.1 M ^{n}Bu$_4$NPF$_6$ as electrolyte.

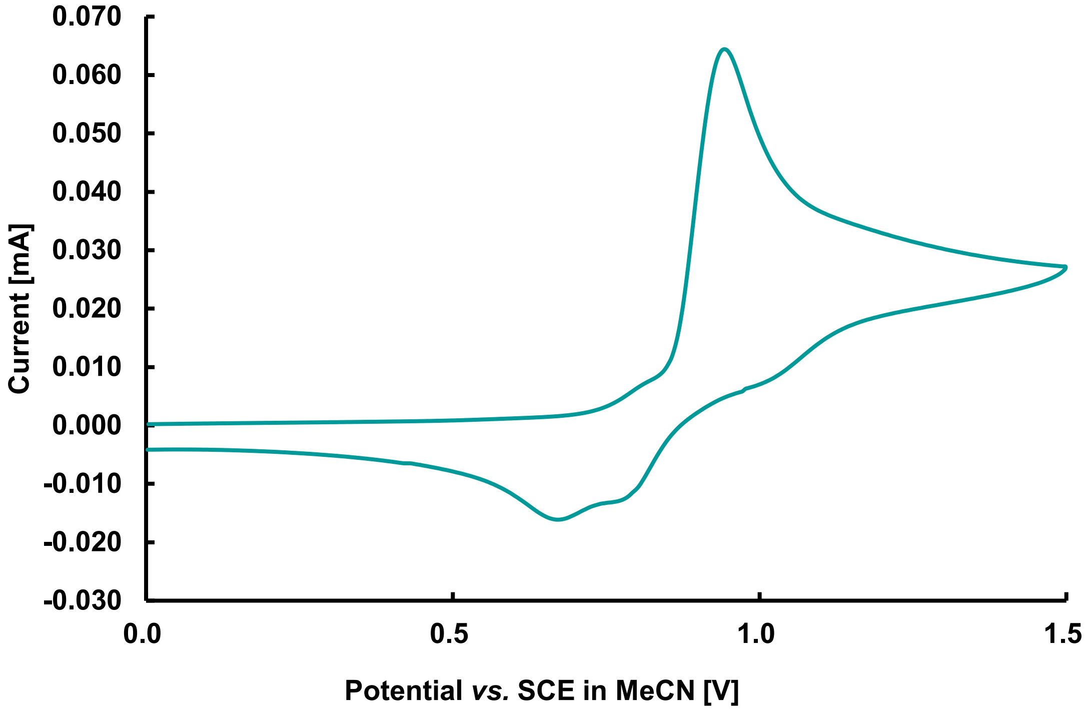

Figure S31. Cyclic voltammetry of **DPA** in degassed MeCN (1 mM) using 0.1 M $^{n}Bu_4NPF_6$ as electrolyte.

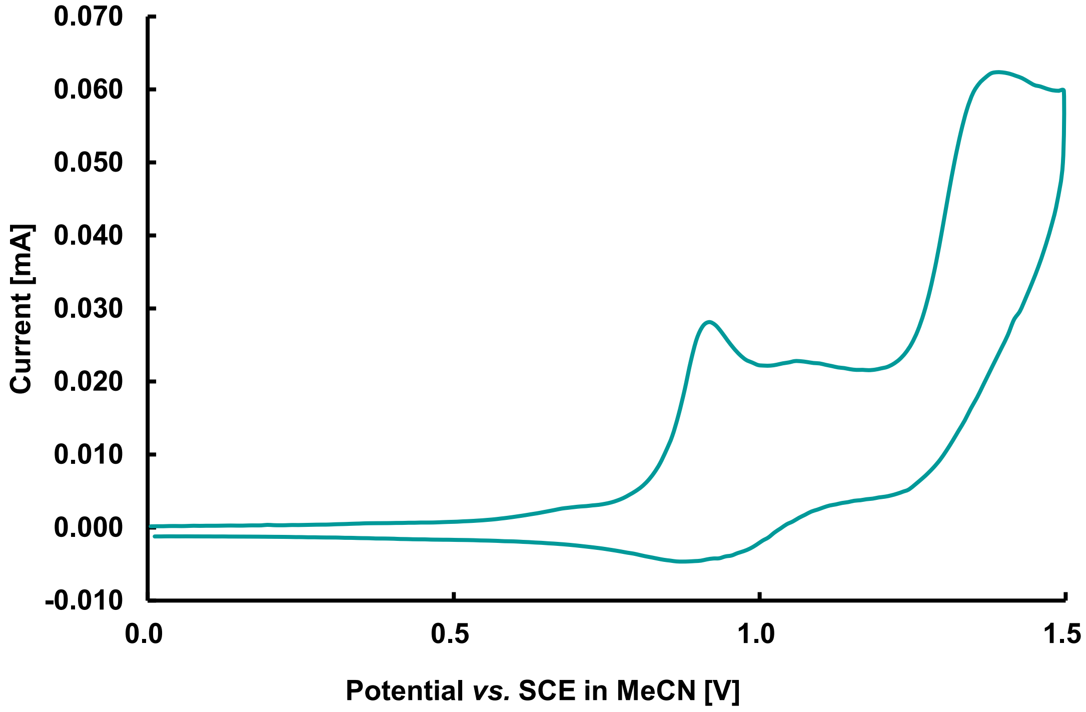

Figure S32. Cyclic voltammetry of **Ar^{4}DPA** in degassed MeCN (1 mM) using 0.1 M $^{n}Bu_4NPF_6$ as electrolyte.

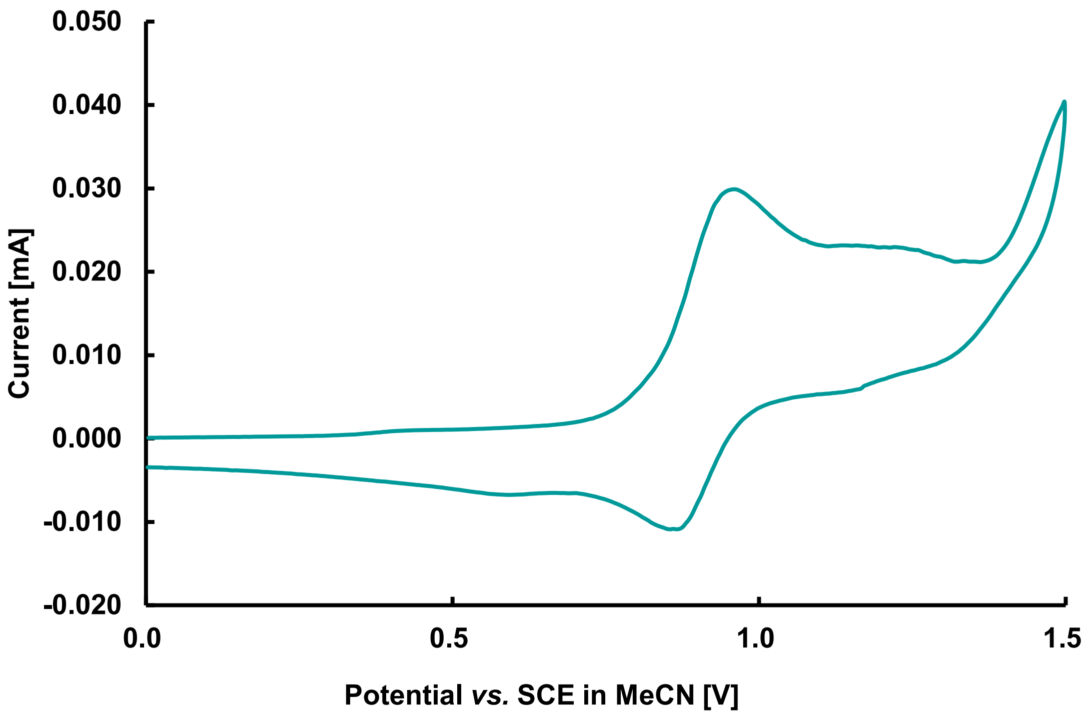

Figure S33. Cyclic voltammetry of **MeOCz** in degassed MeCN (1 mM) using 0.1 M $^{n}Bu_4NPF_6$ as electrolyte.

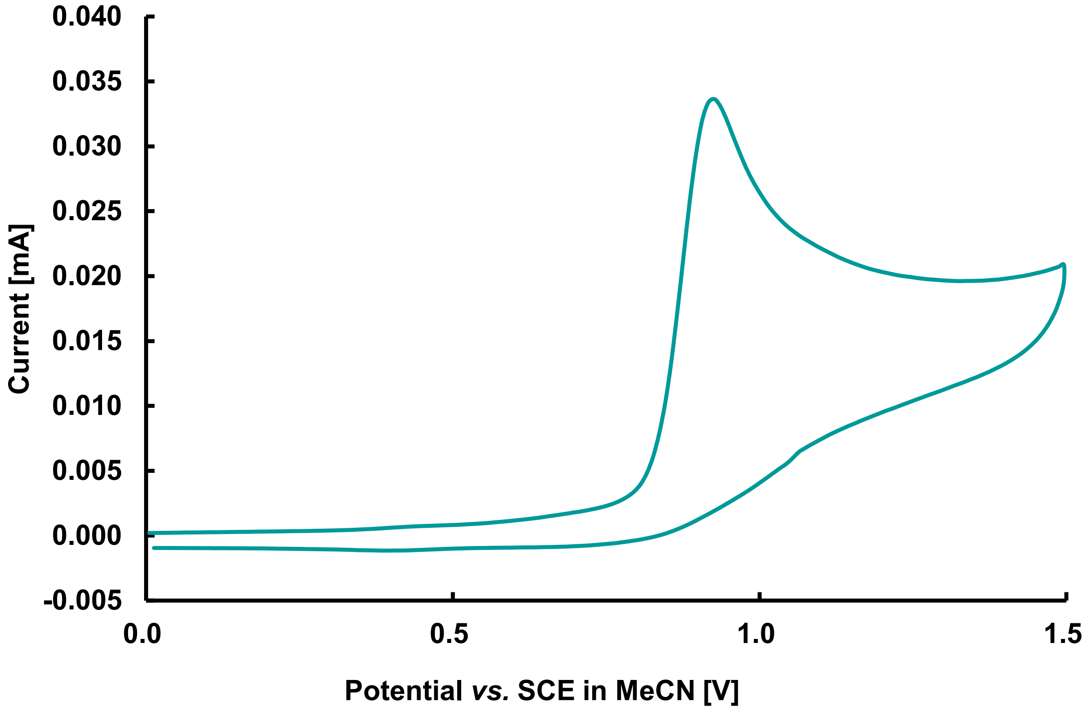

Figure S34. Cyclic voltammetry of **2MeODPA** in degassed MeCN (1 mM) using 0.1 M $^{n}Bu_4NPF_6$ as electrolyte.

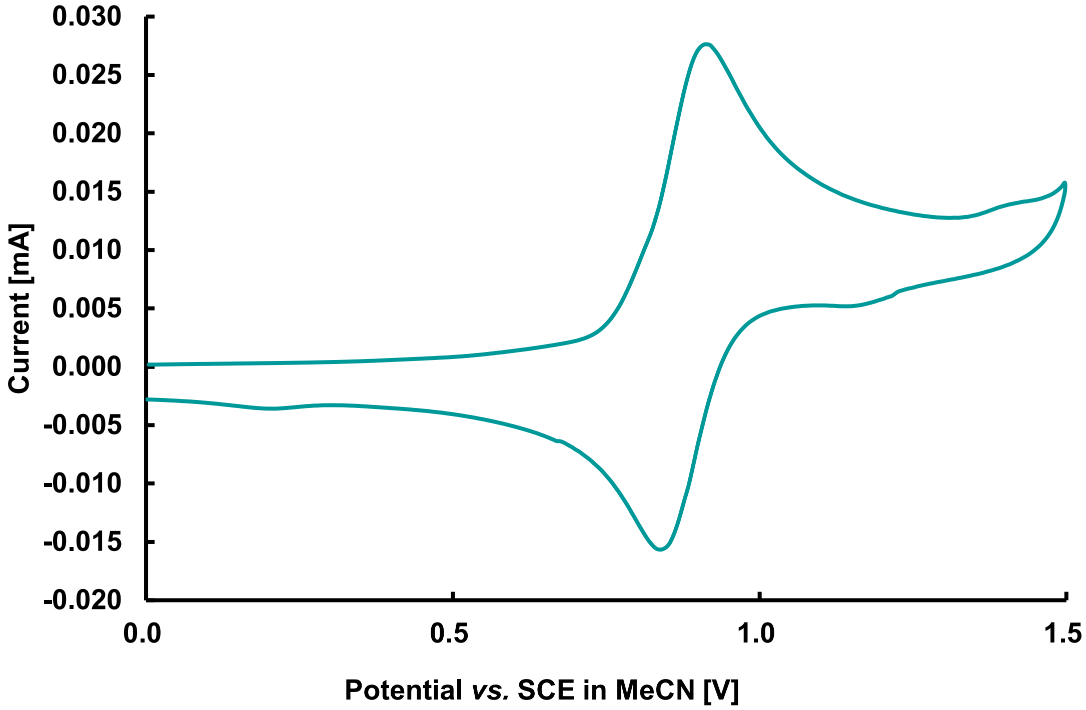

Figure S35. Cyclic voltammetry of **PhDPA** in degassed MeCN (1 mM) using 0.1 M $^{n}Bu_4NPF_6$ as electrolyte.

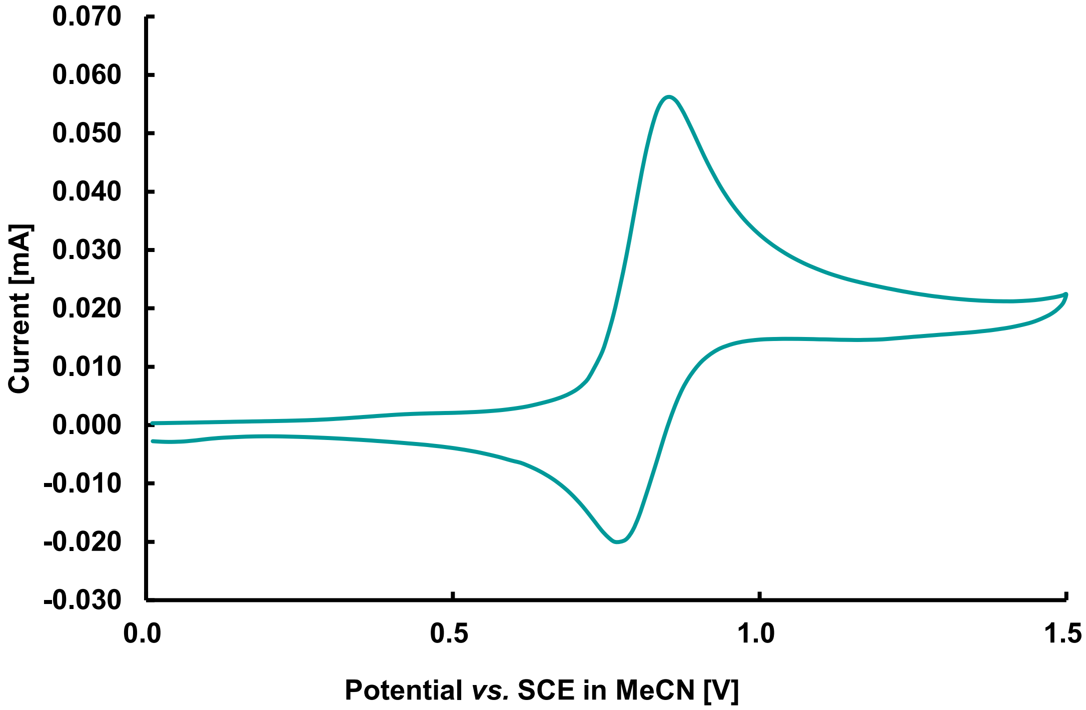

Figure S36. Cyclic voltammetry of **MeDPA** in degassed MeCN (1 mM) using 0.1 M $^{n}Bu_4NPF_6$ as electrolyte.

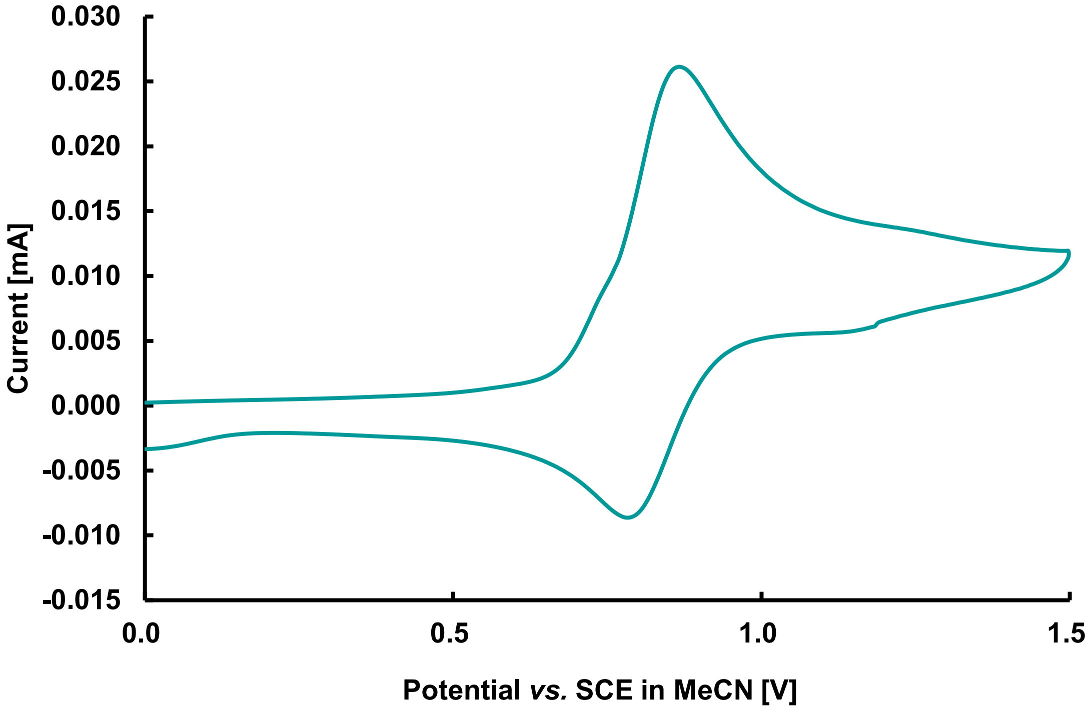

Figure S37. Cyclic voltammetry of **tBuDPA** in degassed MeCN (1 mM) using 0.1 M $^{n}Bu_4NPF_6$ as electrolyte.

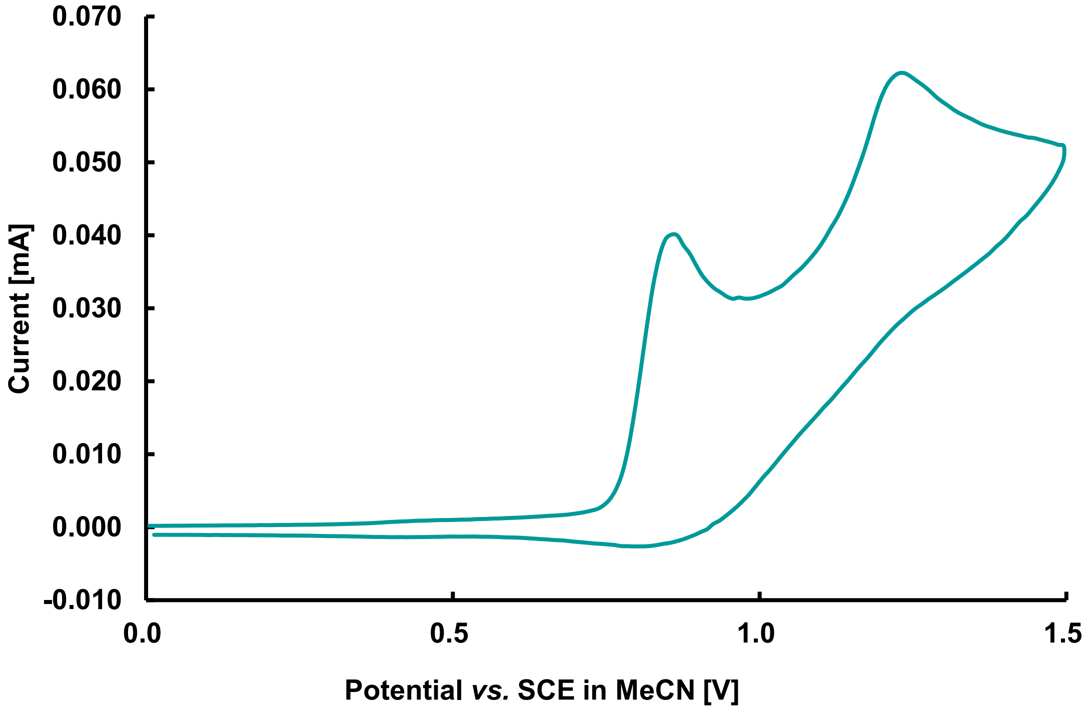

Figure S38. Cyclic voltammetry of **2NPA** in degassed MeCN (1 mM) using 0.1 M $^{n}Bu_4NPF_6$ as electrolyte.

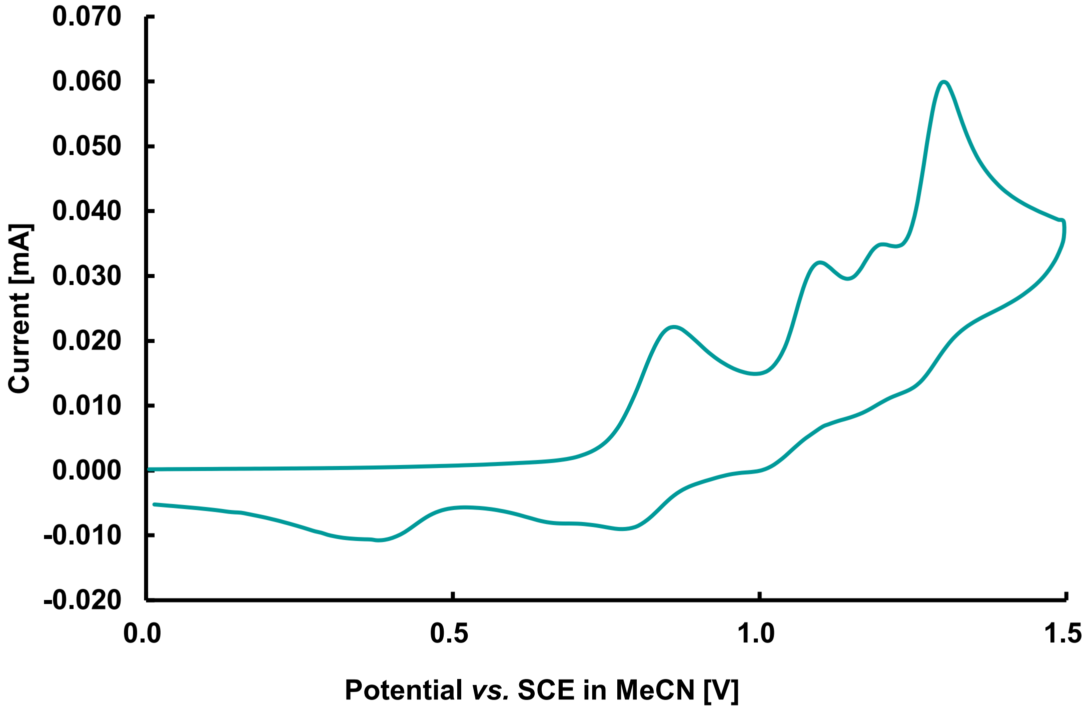

Figure S39. Cyclic voltammetry of **Ar[5]DPA** in degassed MeCN (1 mM) using 0.1 M $^{n}Bu_4NPF_6$ as electrolyte.

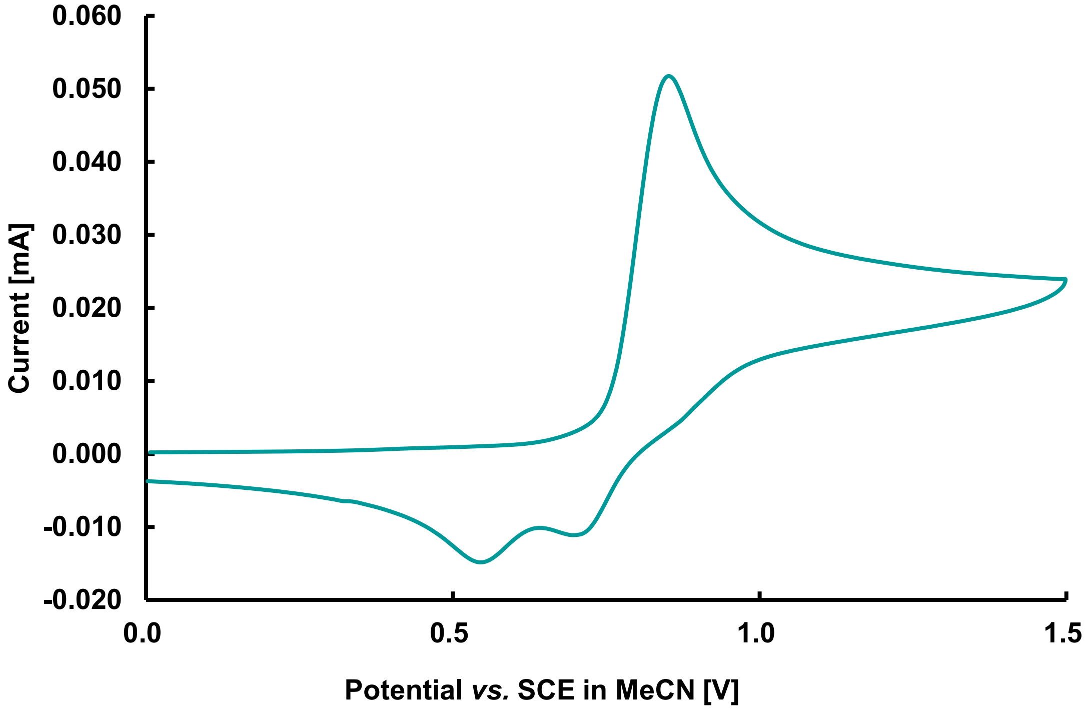

Figure S40. Cyclic voltammetry of **HBAz** in degassed MeCN (1 mM) using 0.1 M $^{n}Bu_4NPF_6$ as electrolyte.

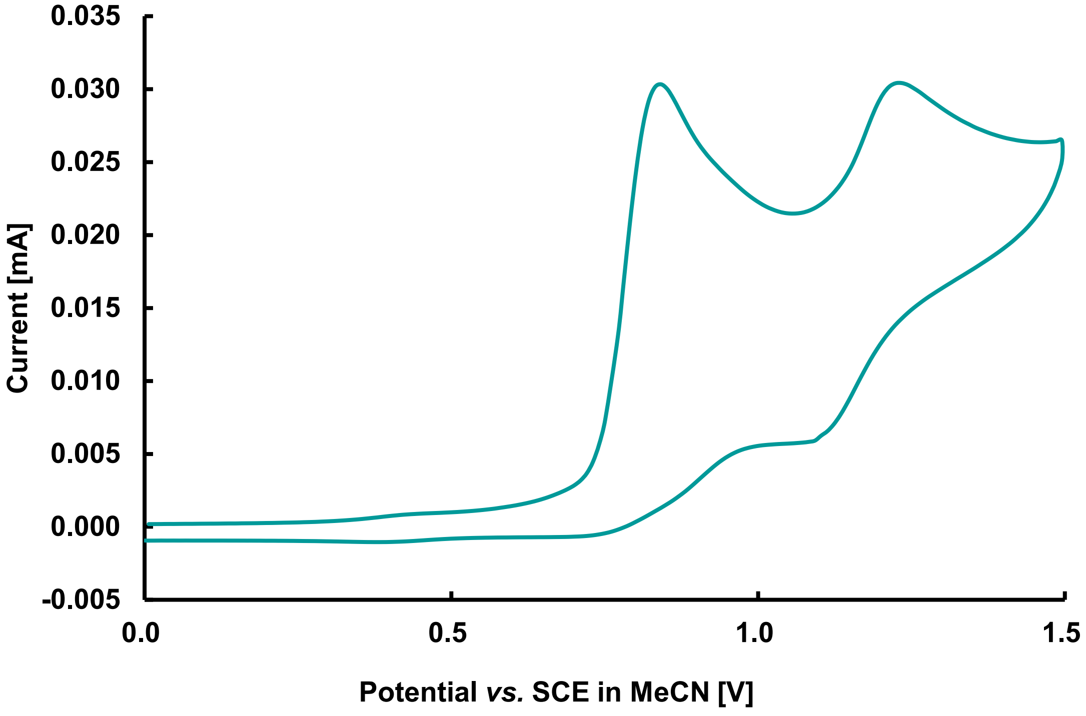

Figure S41. Cyclic voltammetry of **2DNA** in degassed MeCN (1 mM) using 0.1 M ^{n}Bu$_4$NPF$_6$ as electrolyte.

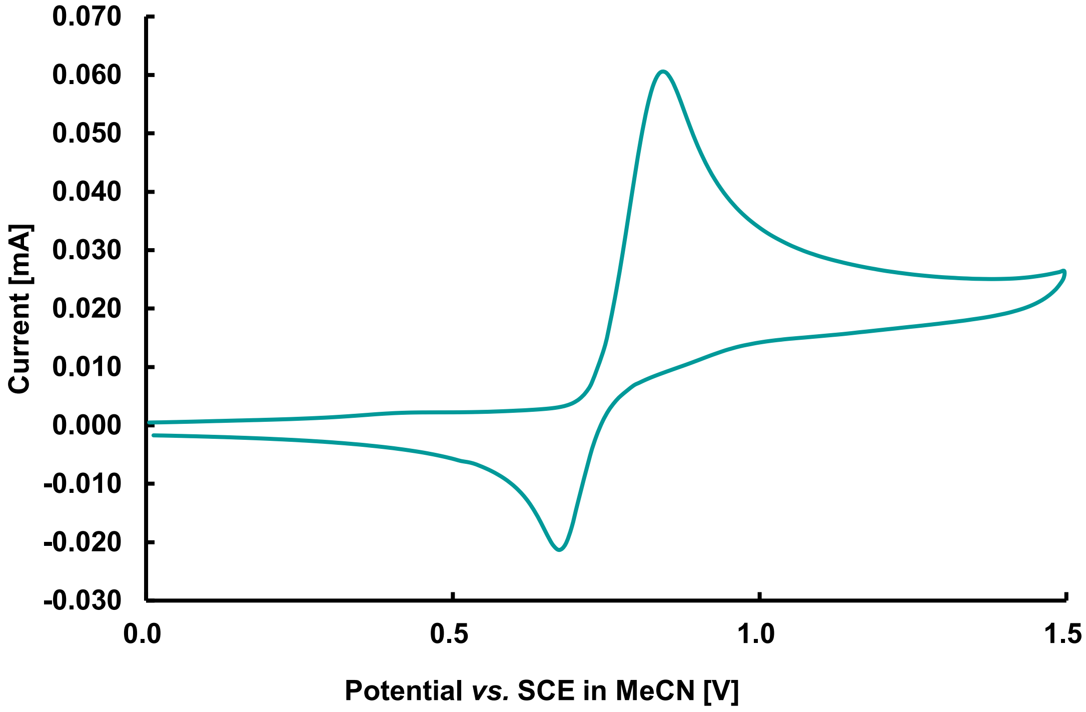

Figure S42. Cyclic voltammetry of **1NPA** in degassed MeCN (1 mM) using 0.1 M ^{n}Bu$_4$NPF$_6$ as electrolyte.

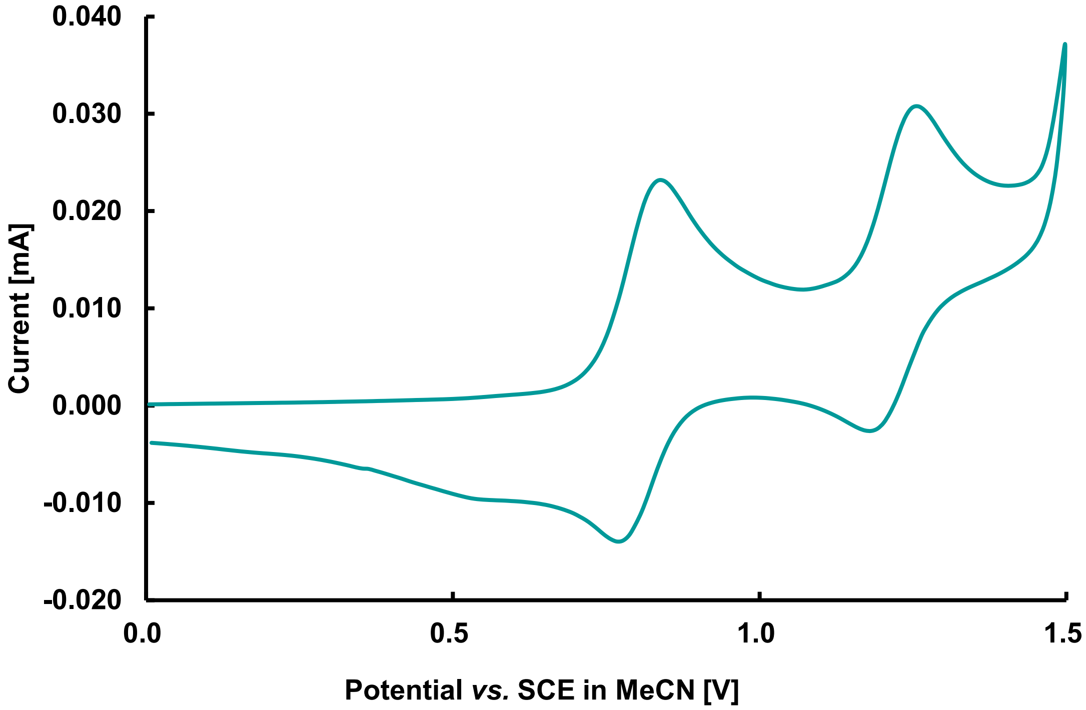

Figure S43. Cyclic voltammetry of **Ar[6]DPA** in degassed MeCN (1 mM) using 0.1 M $^{n}Bu_4NPF_6$ as electrolyte.

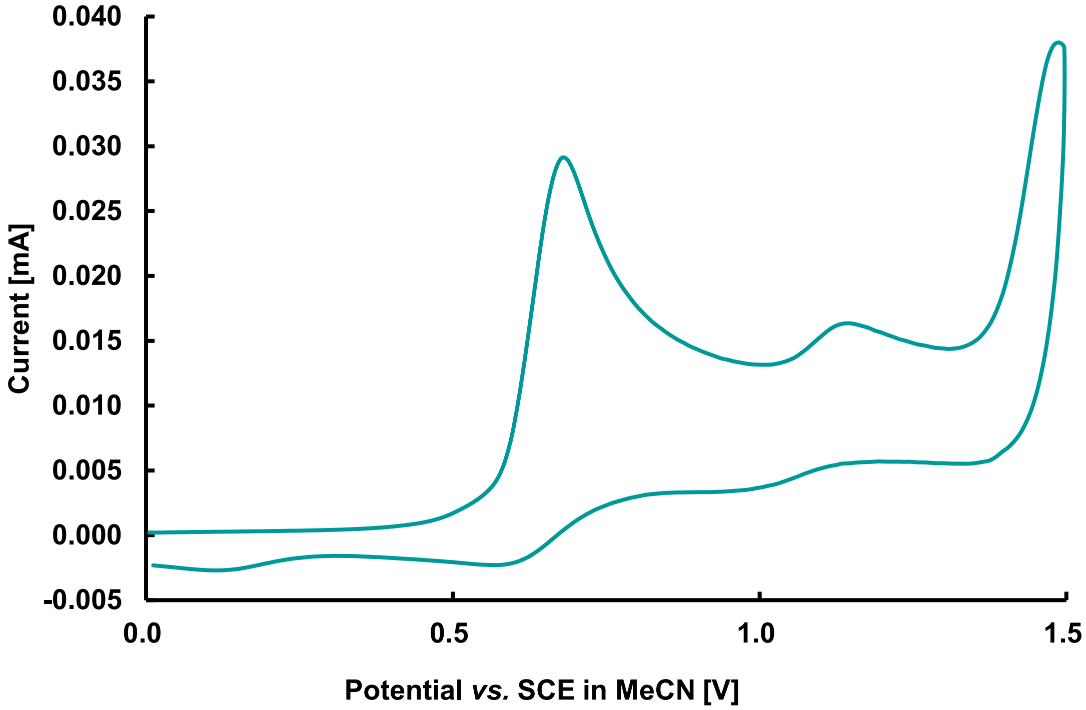

Figure S44. Cyclic voltammetry of **3MeODPA** in degassed MeCN (1 mM) using 0.1 M $^{n}Bu_4NPF_6$ as electrolyte.

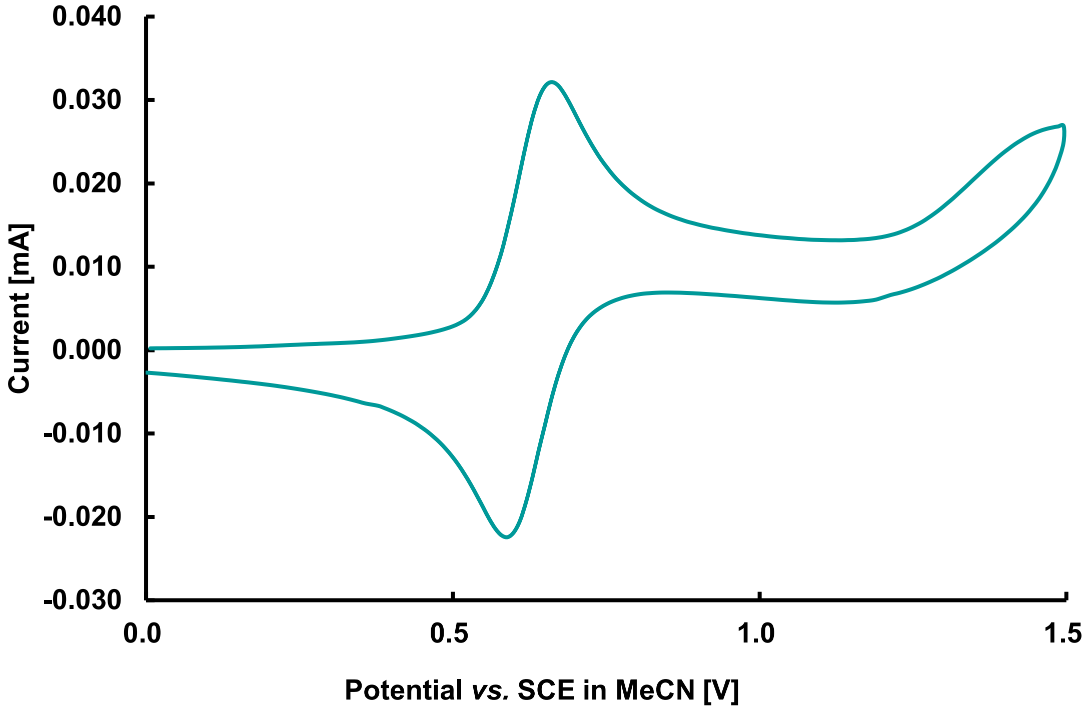

Figure S45. Cyclic voltammetry of **POAz** in degassed MeCN (1 mM) using 0.1 M $^{n}Bu_4NPF_6$ as electrolyte.

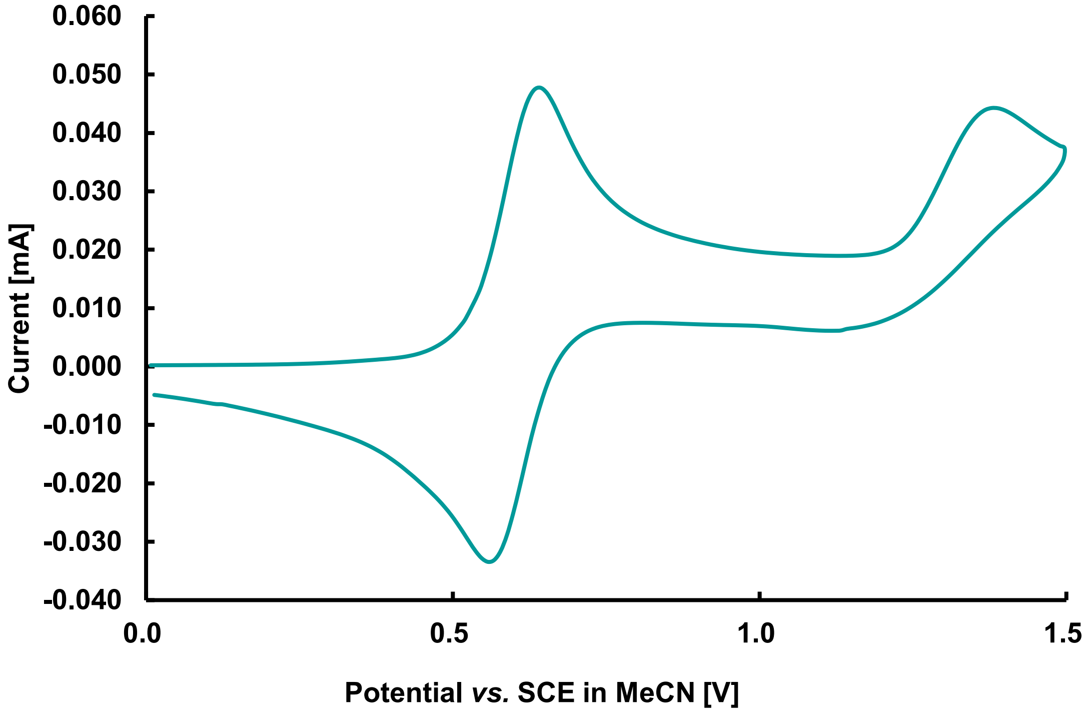

Figure S46. Cyclic voltammetry of **PTAz** in degassed MeCN (1 mM) using 0.1 M $^{n}Bu_4NPF_6$ as electrolyte.

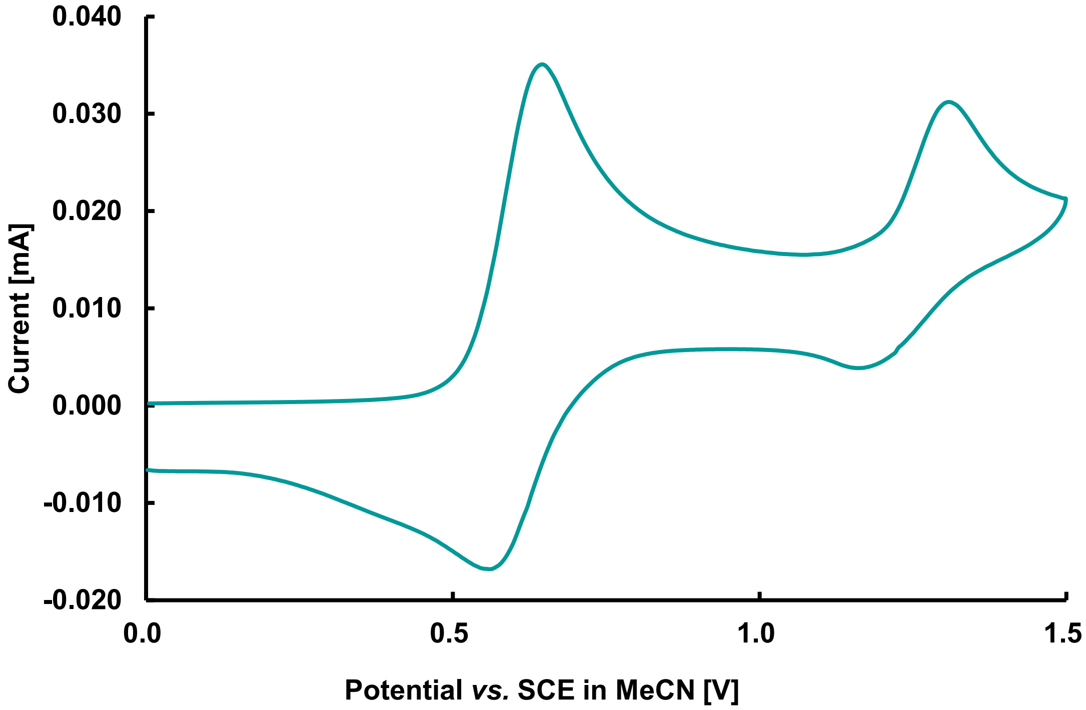

Figure S47. Cyclic voltammetry of **MeODPA** in degassed MeCN (1 mM) using 0.1 M $^{n}Bu_4NPF_6$ as electrolyte.

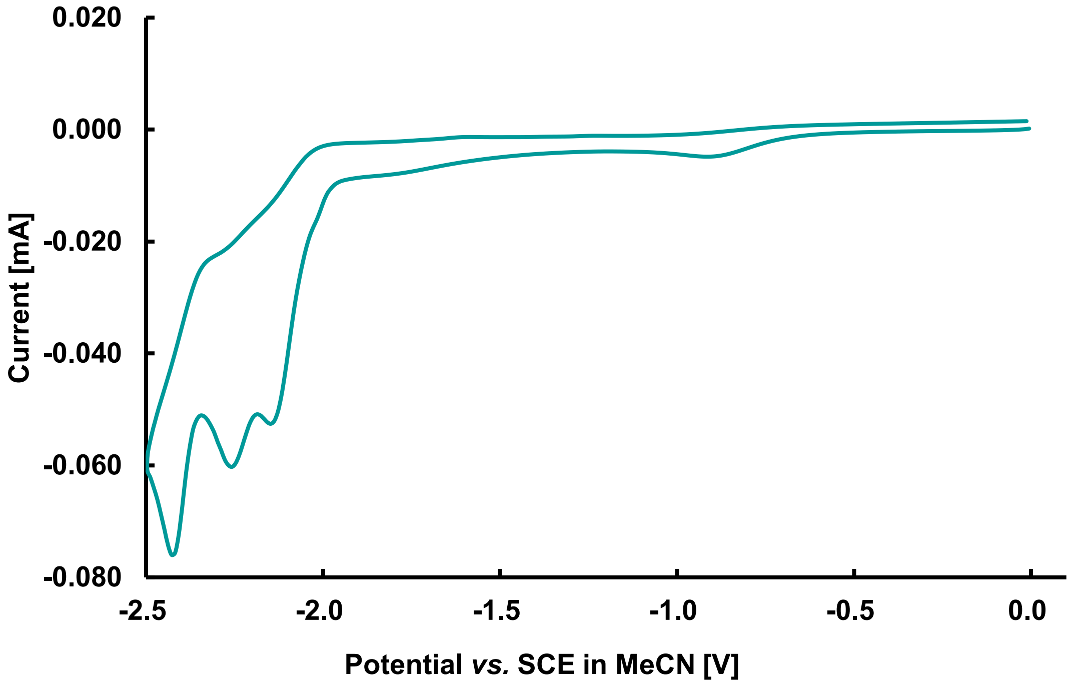

Figure S48. Cyclic voltammetry of **3FIPN-2,4,6** in degassed MeCN (1 mM) using 0.1 M $^{n}Bu_4NPF_6$ as electrolyte.

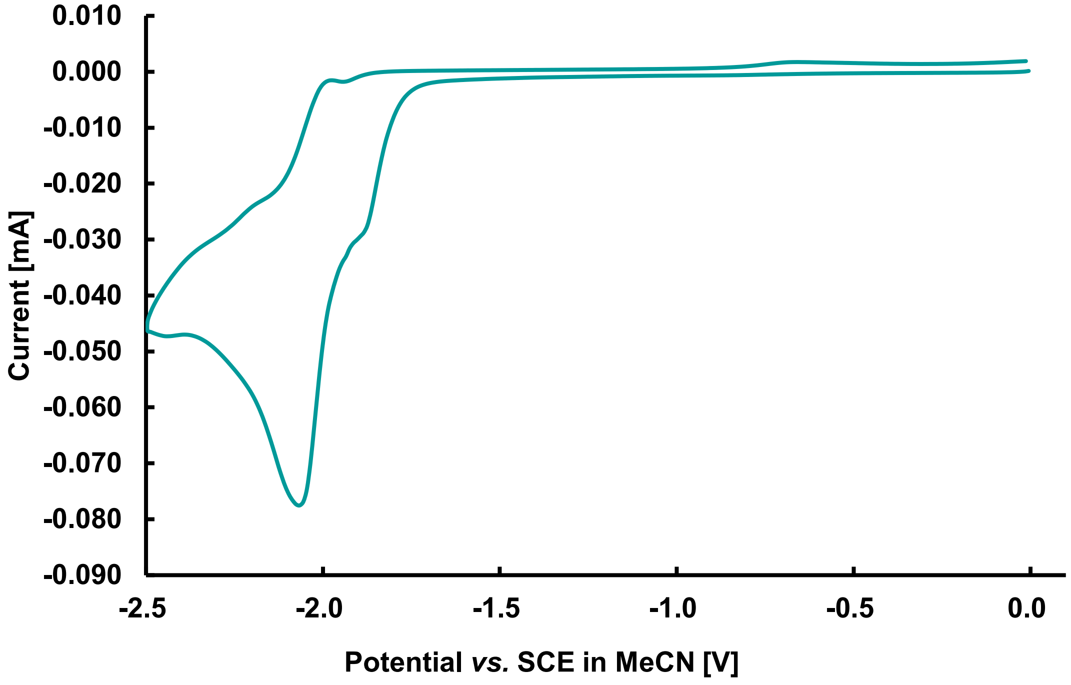

Figure S49. Cyclic voltammetry of **3FIPN-3,4,5** in degassed MeCN (1 mM) using 0.1 M $^{n}Bu_4NPF_6$ as electrolyte.

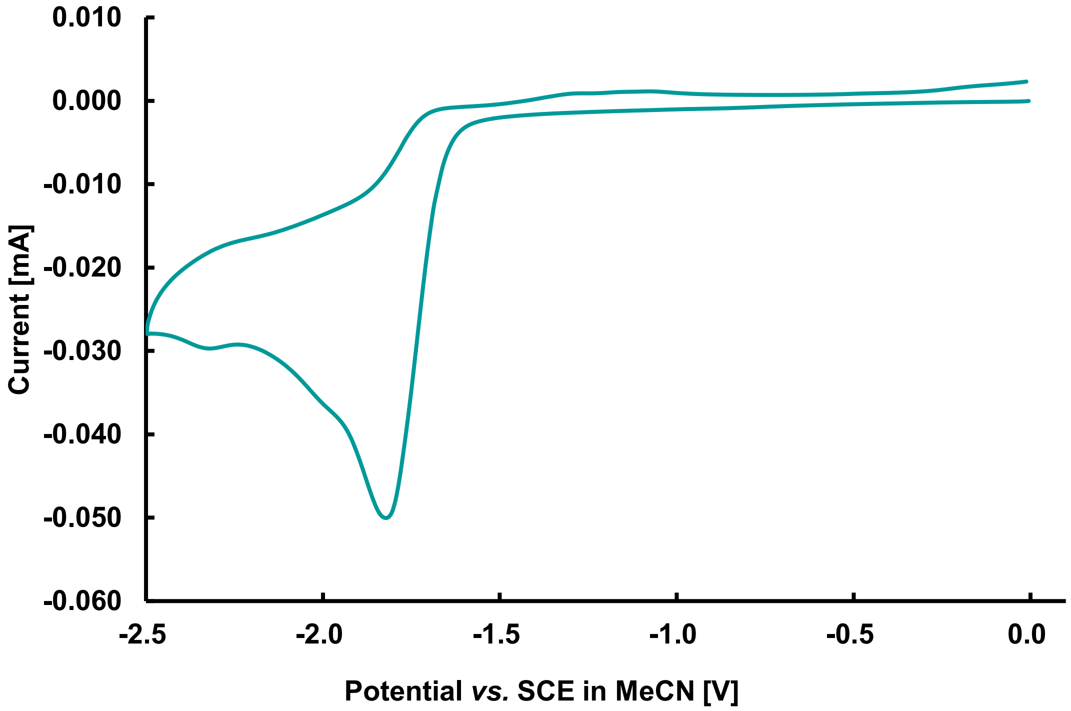

Figure S50. Cyclic voltammetry of **2FIPN** in degassed MeCN (1 mM) using 0.1 M $^{n}Bu_4NPF_6$ as electrolyte.

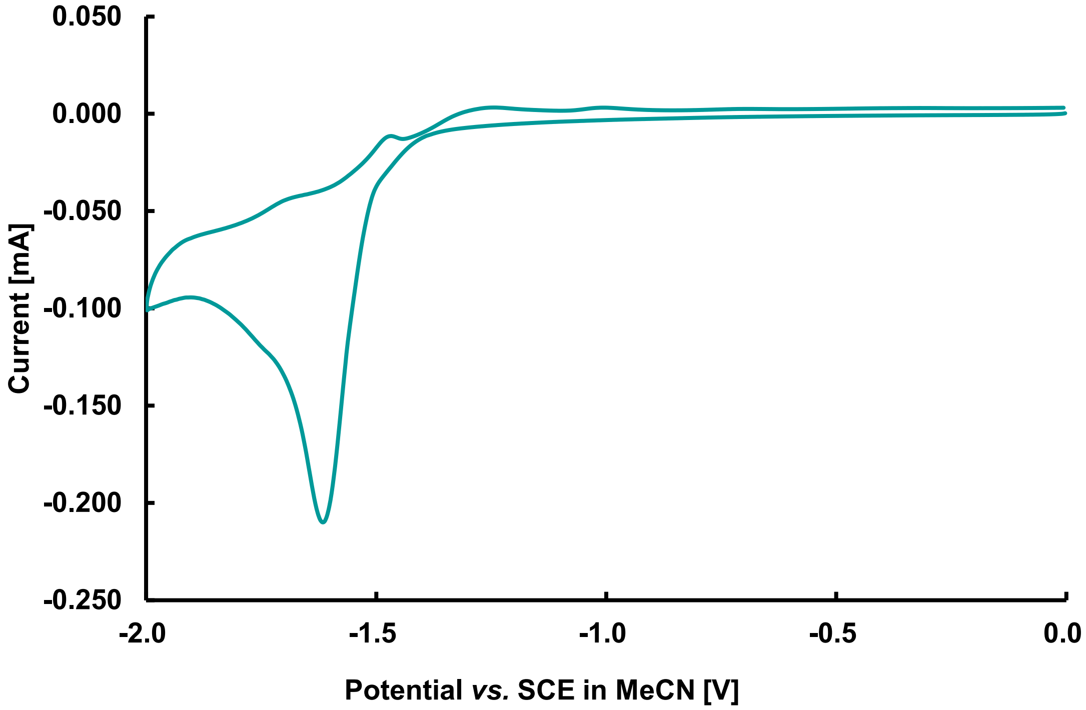

Figure S51. Cyclic voltammetry of **5FBN** in degassed MeCN (1 mM) using 0.1 M ^{n}Bu$_4$NPF$_6$ as electrolyte.

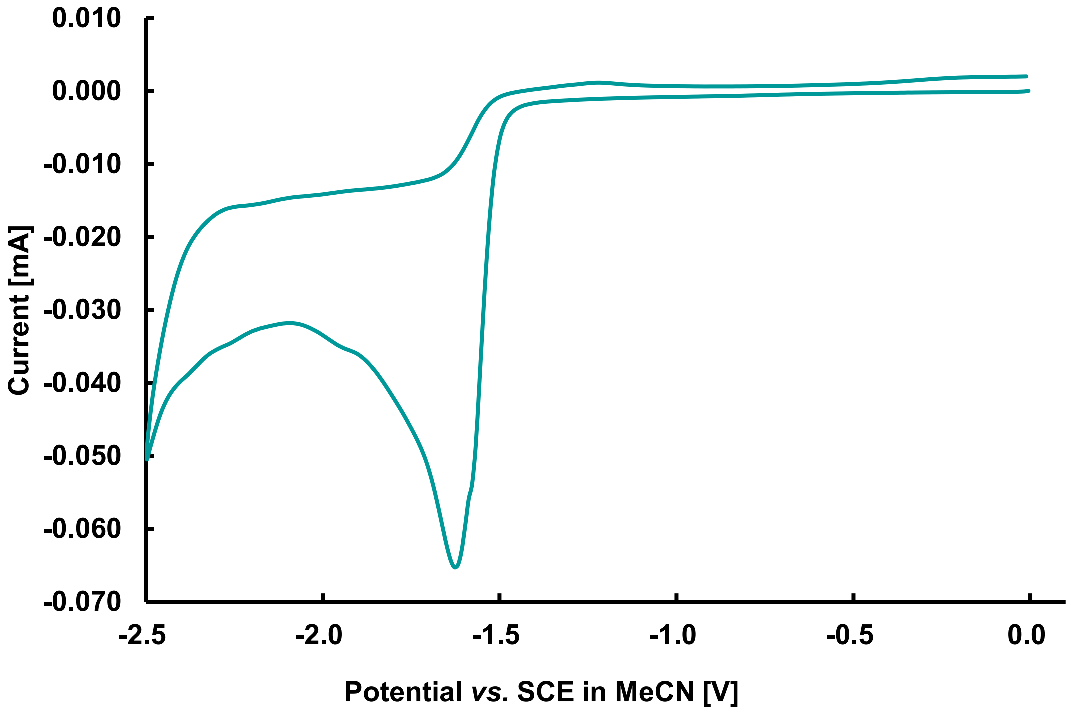

Figure S52. Cyclic voltammetry of **2FPN** in degassed MeCN (1 mM) using 0.1 M ^{n}Bu$_4$NPF$_6$ as electrolyte.

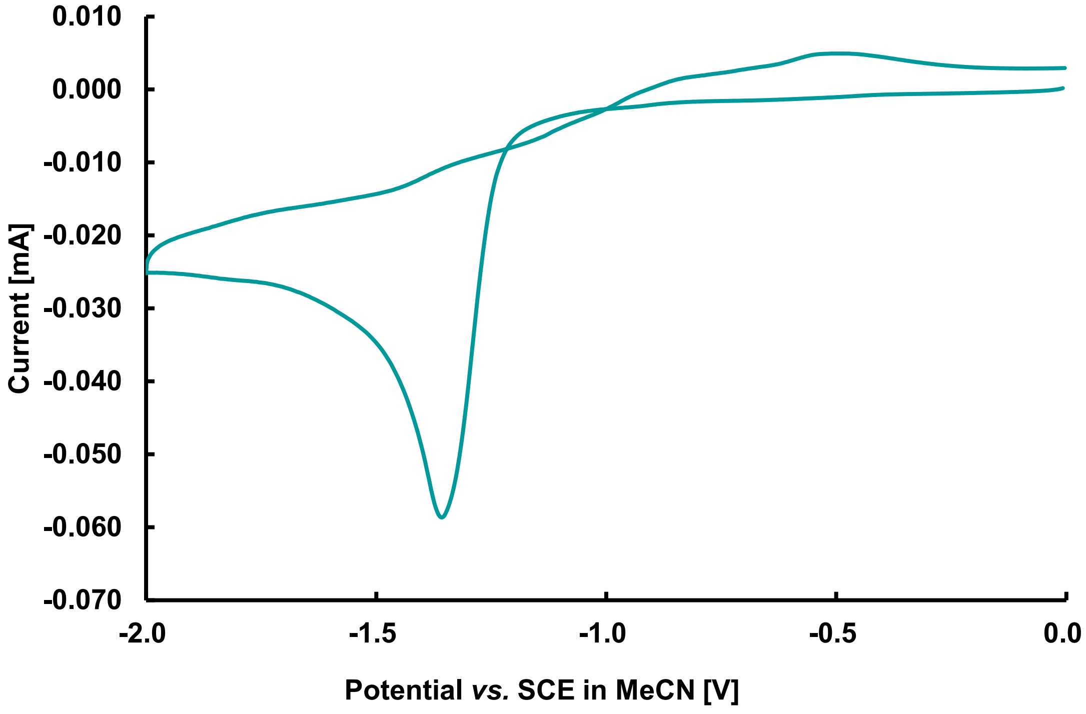

Figure S53. Cyclic voltammetry of **4FIPN** in degassed MeCN (1 mM) using 0.1 M $^{n}Bu_4NPF_6$ as electrolyte.

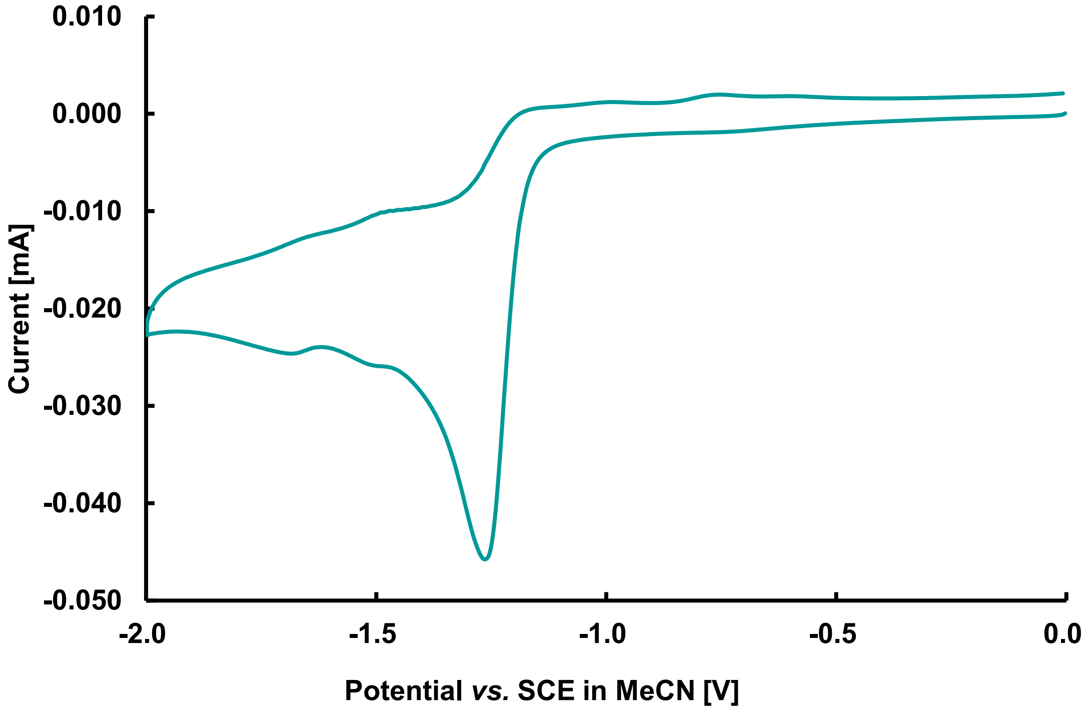

Figure S54. Cyclic voltammetry of **4FINN** in degassed MeCN (1 mM) using 0.1 M $^{n}Bu_4NPF_6$ as electrolyte.

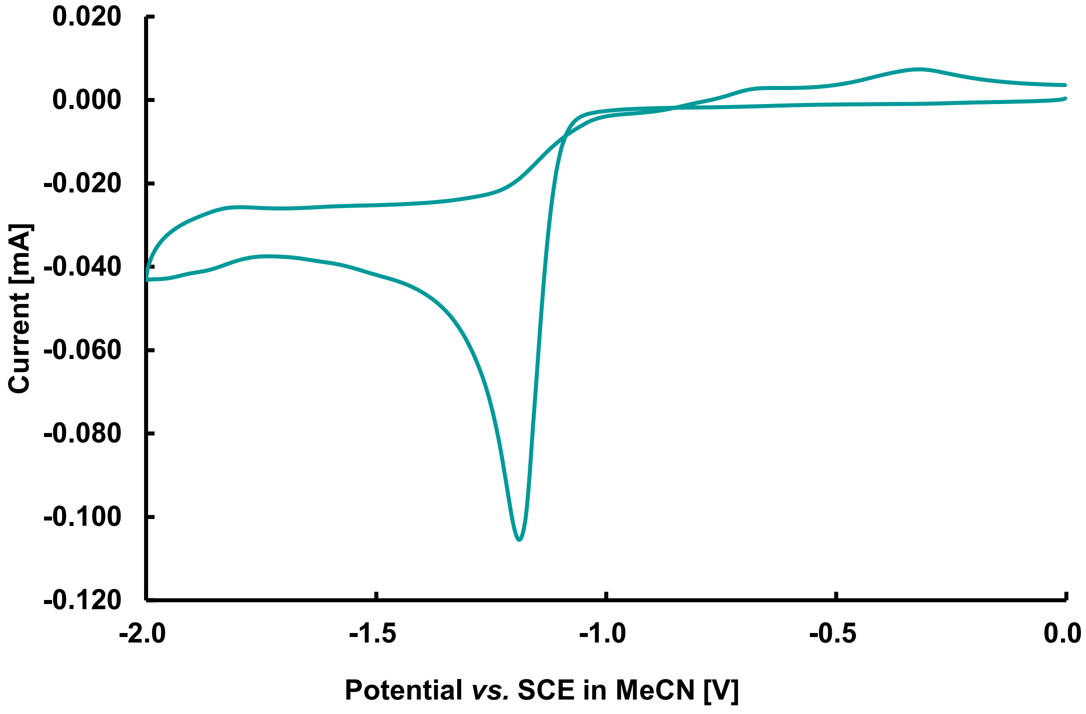

Figure S55. Cyclic voltammetry of **4FPN** in degassed MeCN (1 mM) using 0.1 M $^{n}Bu_4NPF_6$ as electrolyte.

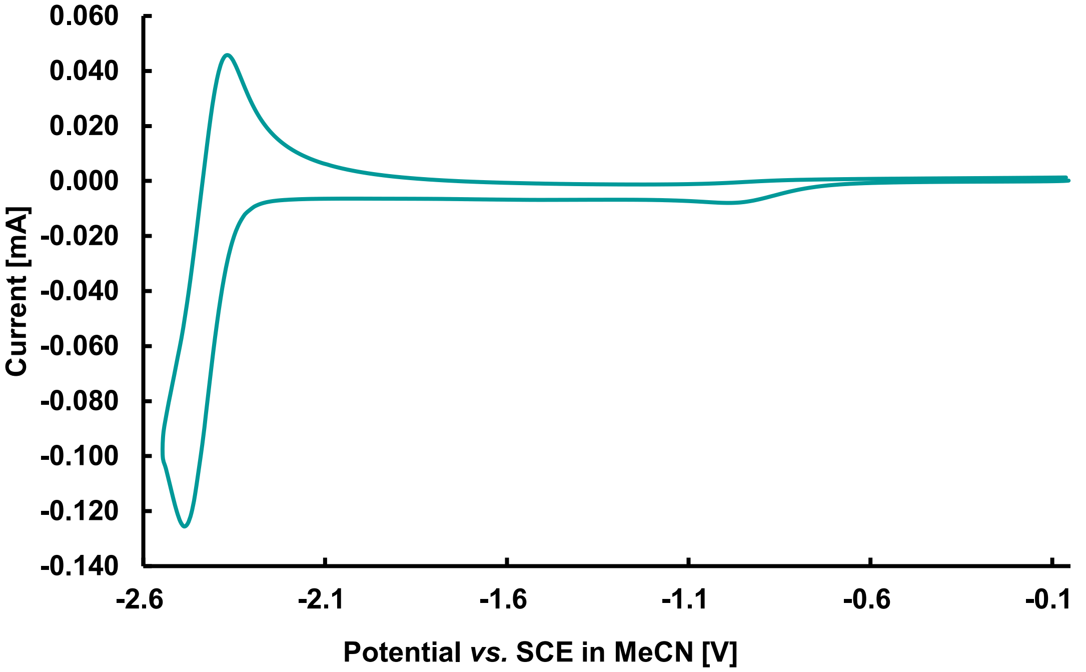

Figure S56. Cyclic voltammetry of **5HBN** in degassed MeCN (1 mM) using 0.1 M $^{n}Bu_4NPF_6$ as electrolyte.

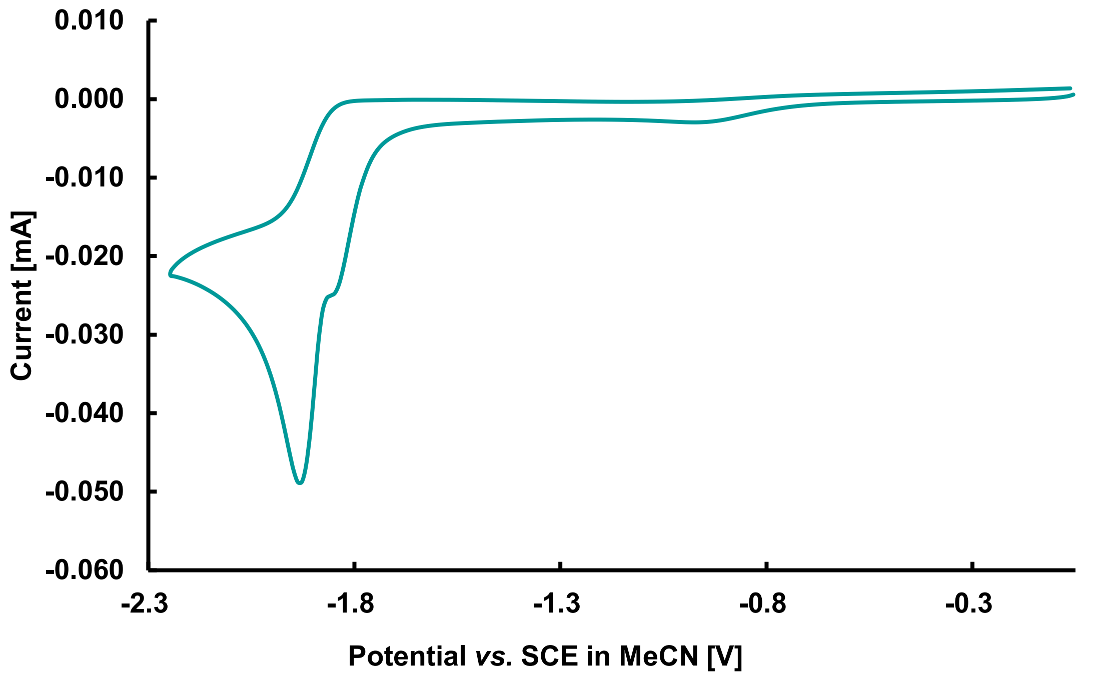

Figure S57. Cyclic voltammetry of **4HIPN** in degassed MeCN (1 mM) using 0.1 M $^{n}Bu_4NPF_6$ as electrolyte.

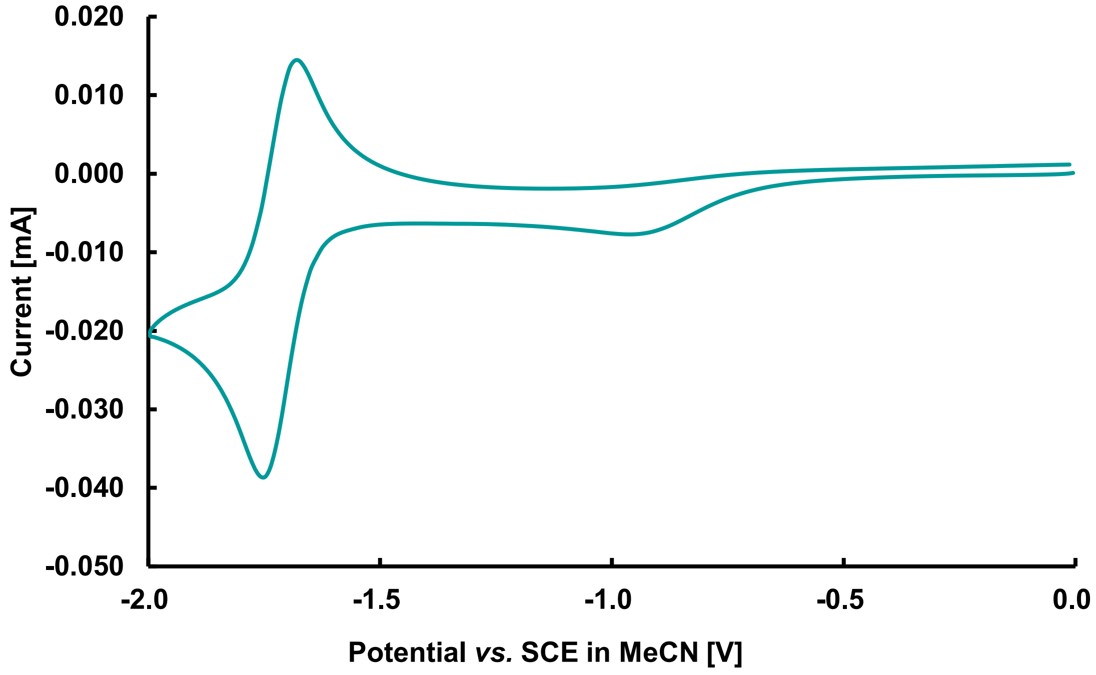

Figure S58. Cyclic voltammetry of **4HPN** in degassed MeCN (1 mM) using 0.1 M $^{n}Bu_4NPF_6$ as electrolyte.

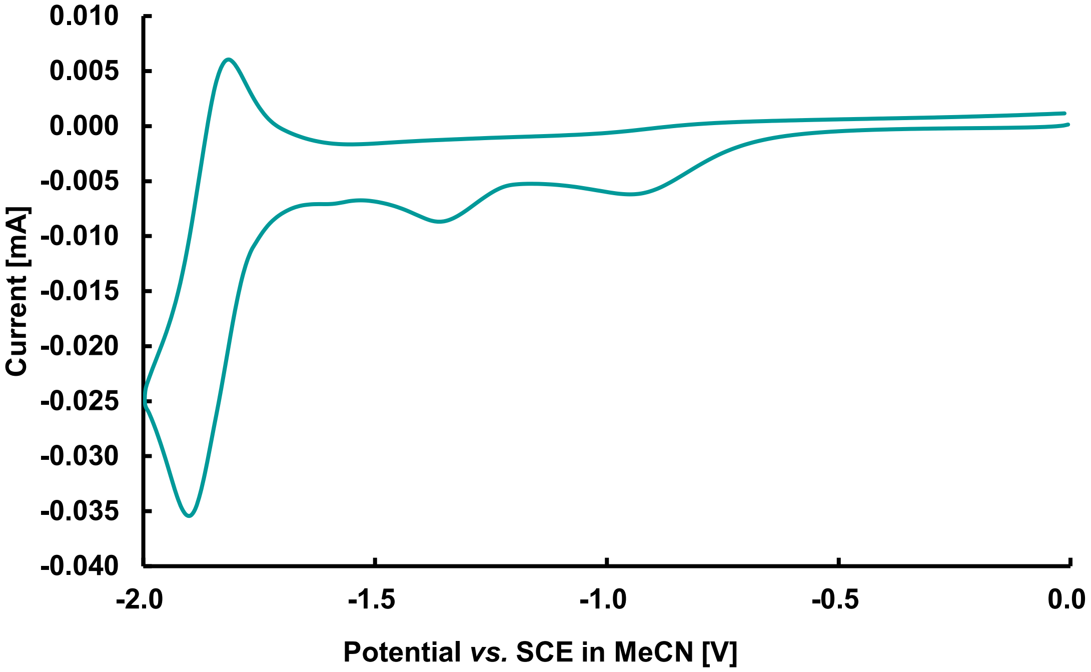

Figure S59. Cyclic voltammetry of **4HINN** in degassed MeCN (1 mM) using 0.1 M $^{n}Bu_4NPF_6$ as electrolyte.

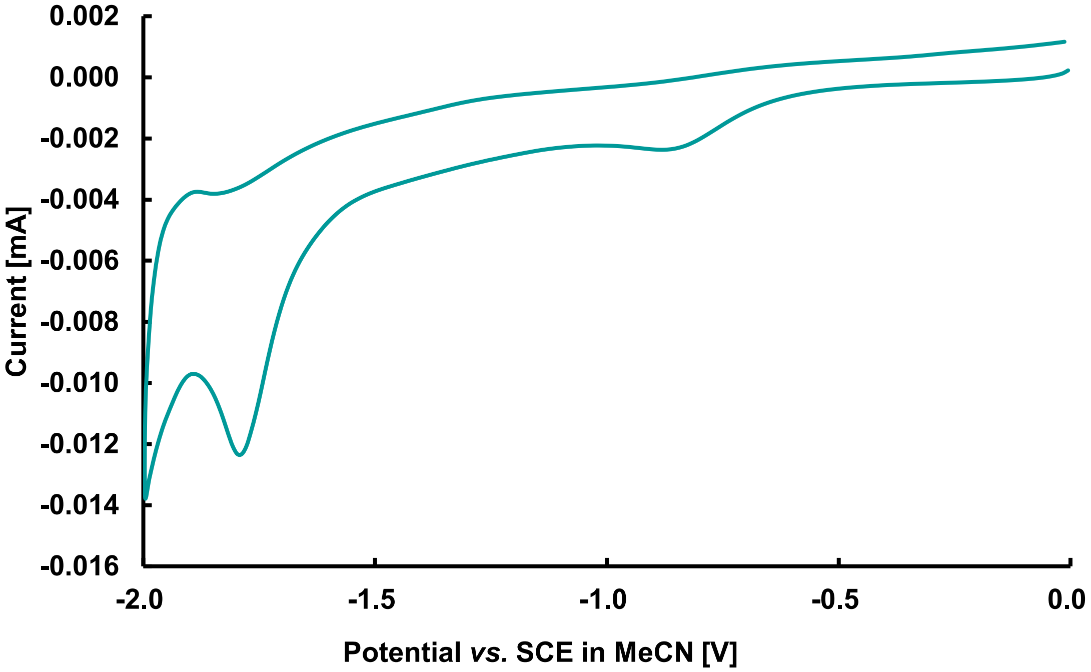

Figure S60. Cyclic voltammetry of **4HTPN** in degassed MeCN (1 mM) using 0.1 M $^{n}Bu_4NPF_6$ as electrolyte.

2.2.4. Photophysical data of the photocatalysts PC1-PC37

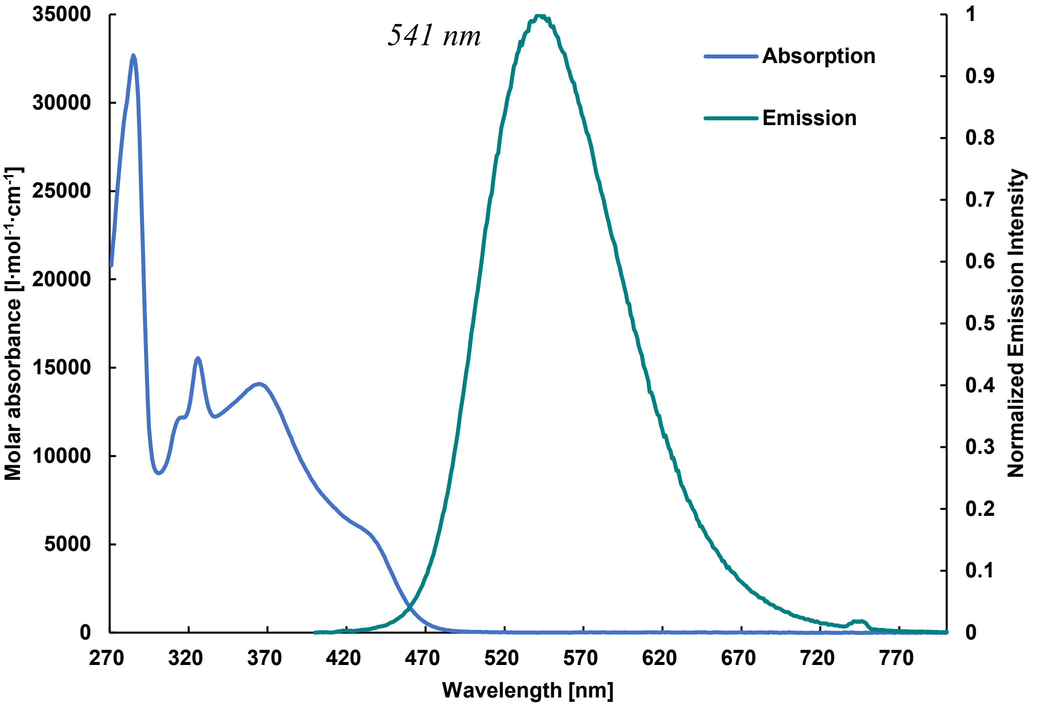

Figure S61. UV/Vis absorption and emission spectrum of **PC1** in degassed MeCN (20 μM). Excitation wavelength – 394 nm.

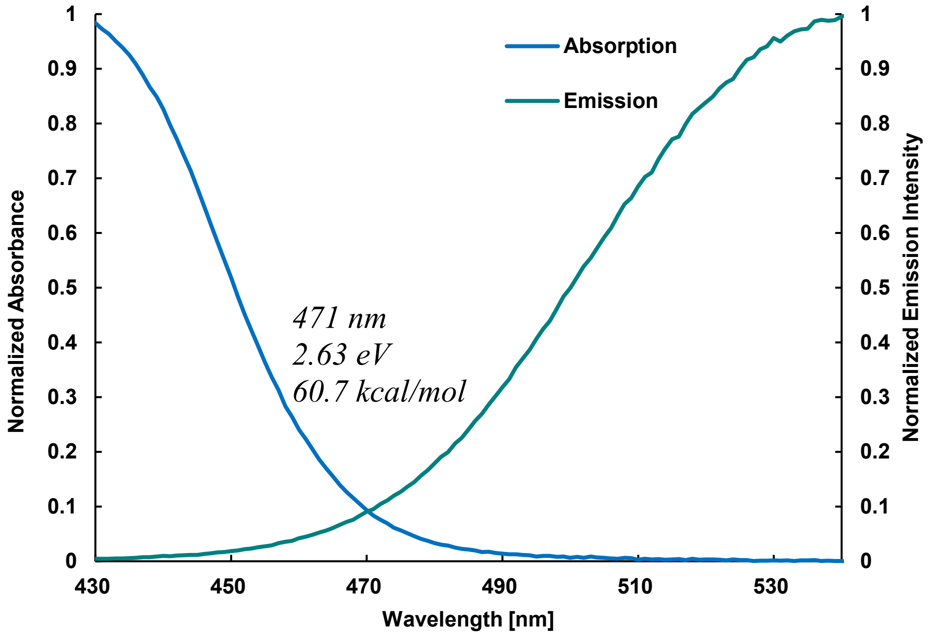

Figure S62. $E_{0\text{-}0}$ estimation at normalized emission and absorption overlap of **PC1**

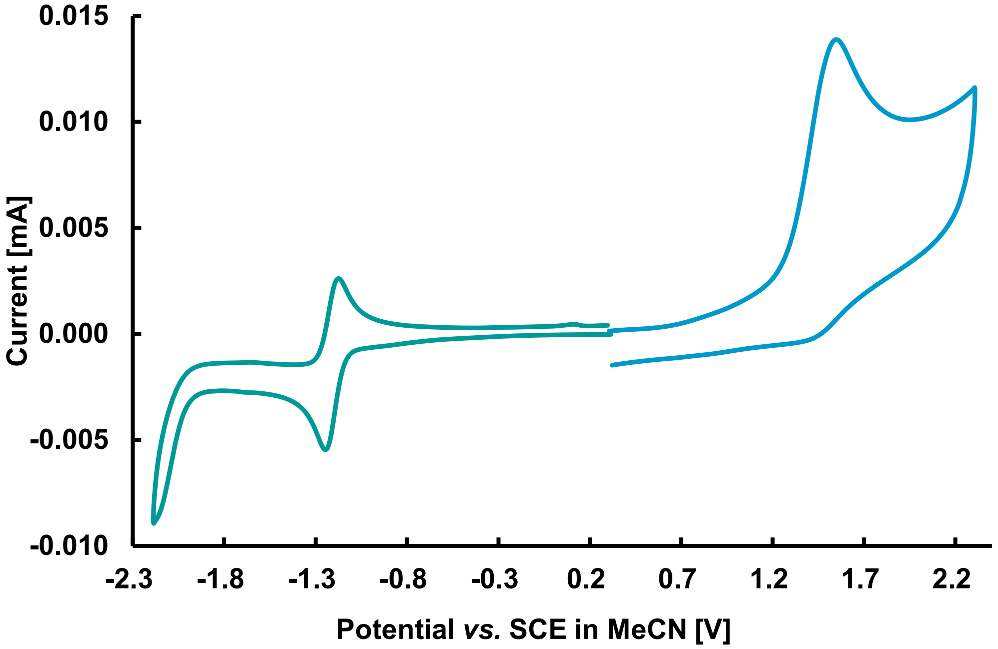

Figure S63. Cyclic voltammetry of **PC1** in degassed MeCN (0.4 mM) using 0.1 M nBu4NPF6 as electrolyte.

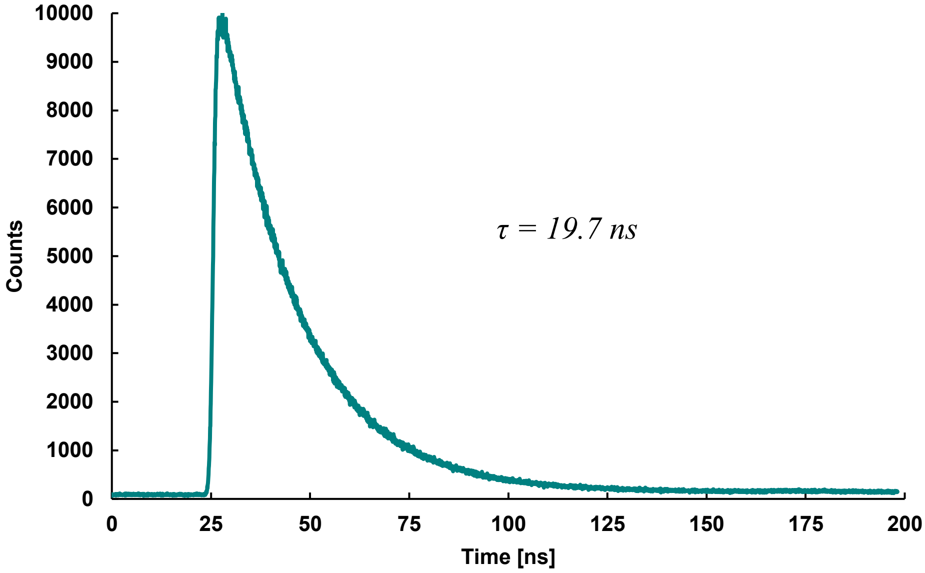

Figure S64. Fluorescence decay curve of **PC1** in degassed MeCN (20 µM). Excitation wavelength – 394 nm.

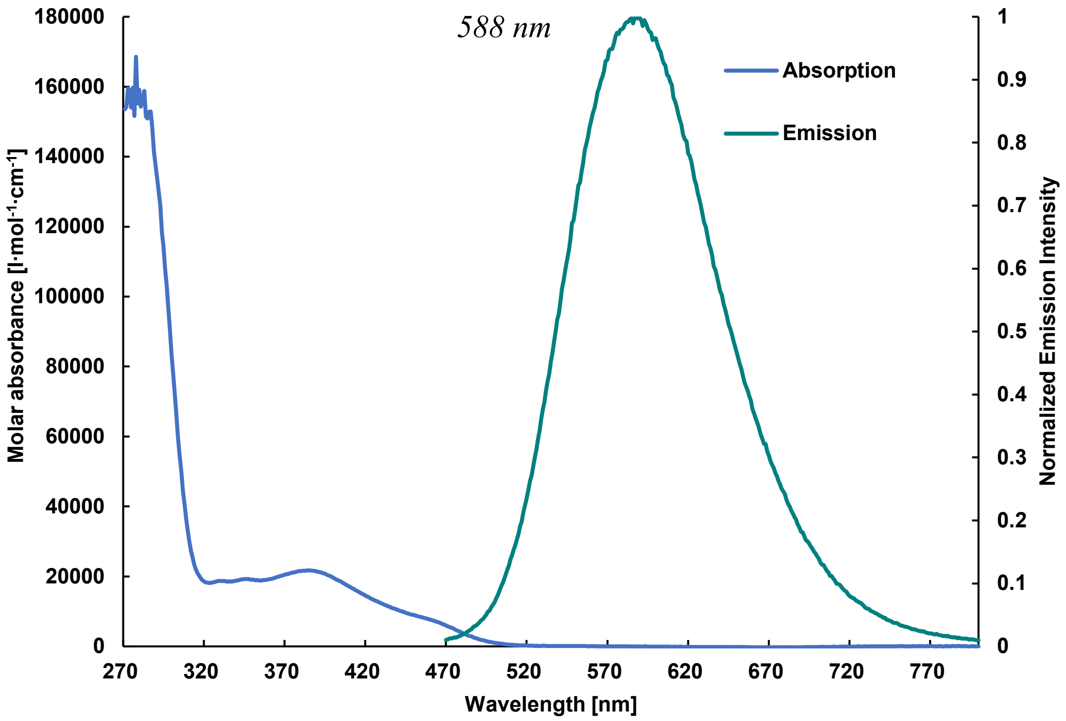

Figure S65. UV/Vis absorption and emission spectrum of **PC2** in degassed MeCN (20 μM). Excitation wavelength – 456 nm.

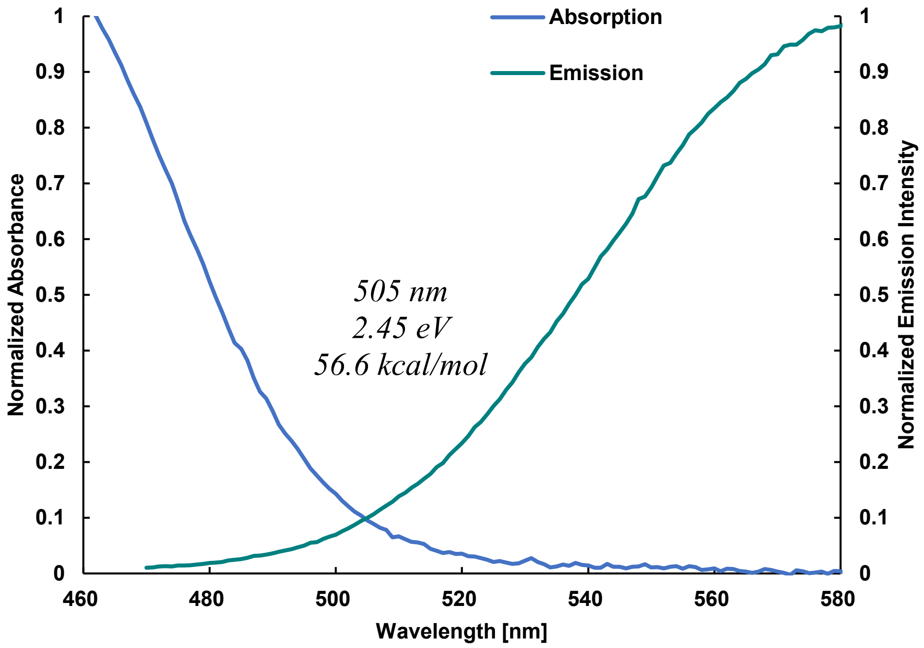

Figure S66. E_{0-0} estimation at normalized emission and absorption overlap of **PC2**.

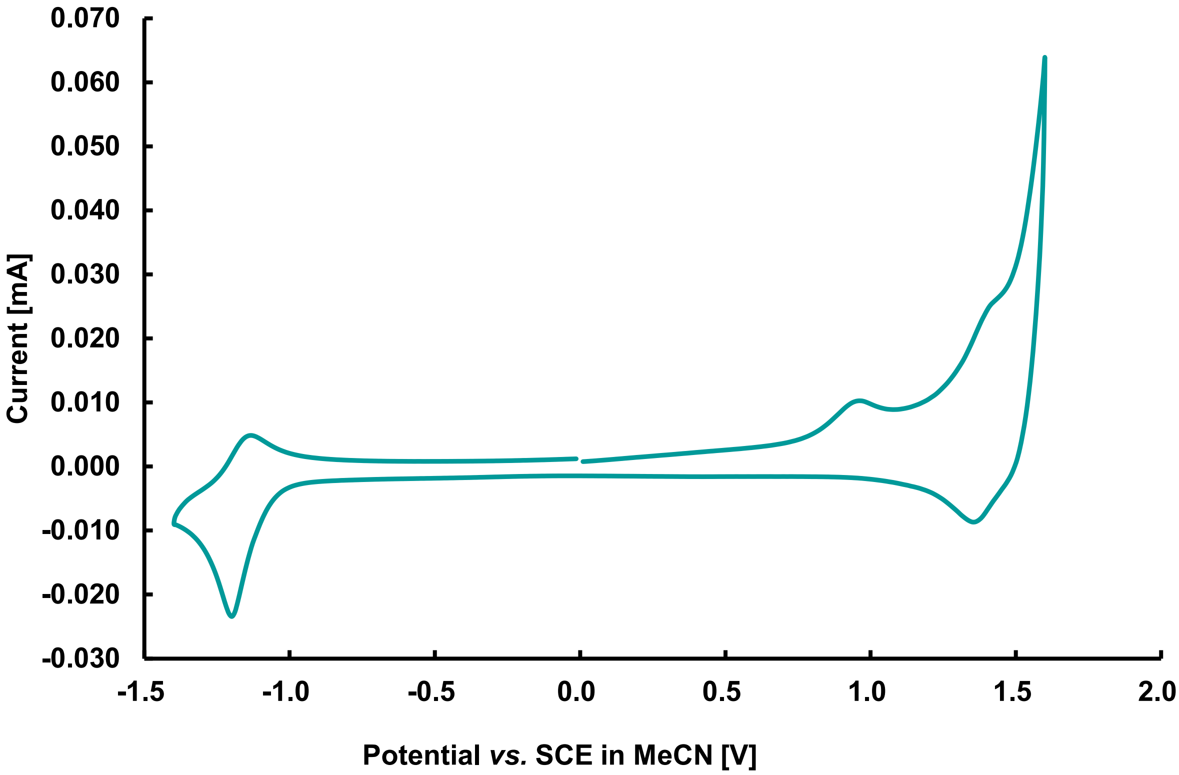

Figure S67. Cyclic voltammetry of **PC2** in degassed MeCN (0.4 mM) using 0.1 M $^{n}Bu_4NPF_6$ as electrolyte.

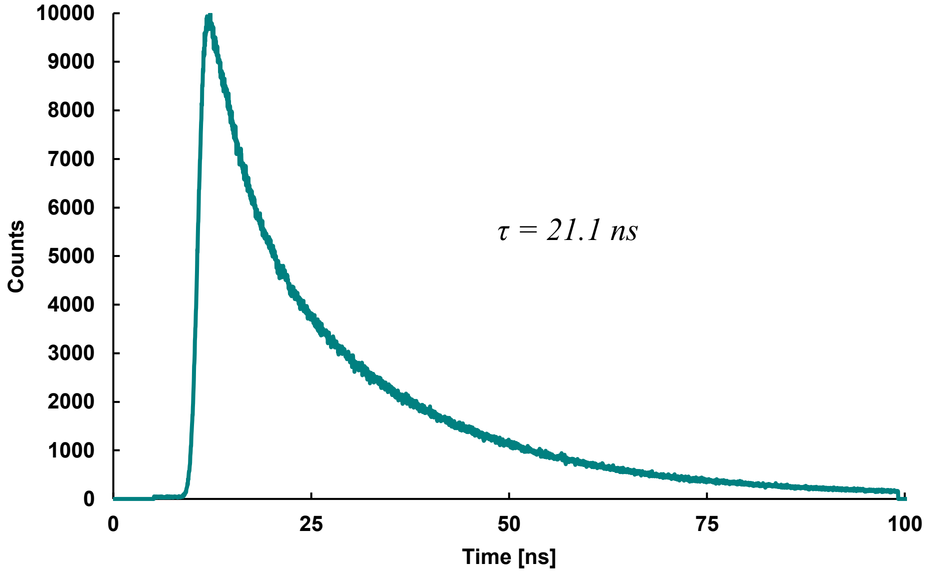

Figure S68. Fluorescence decay curve of **PC2** in degassed MeCN (20 μM). Excitation wavelength – 370 nm.

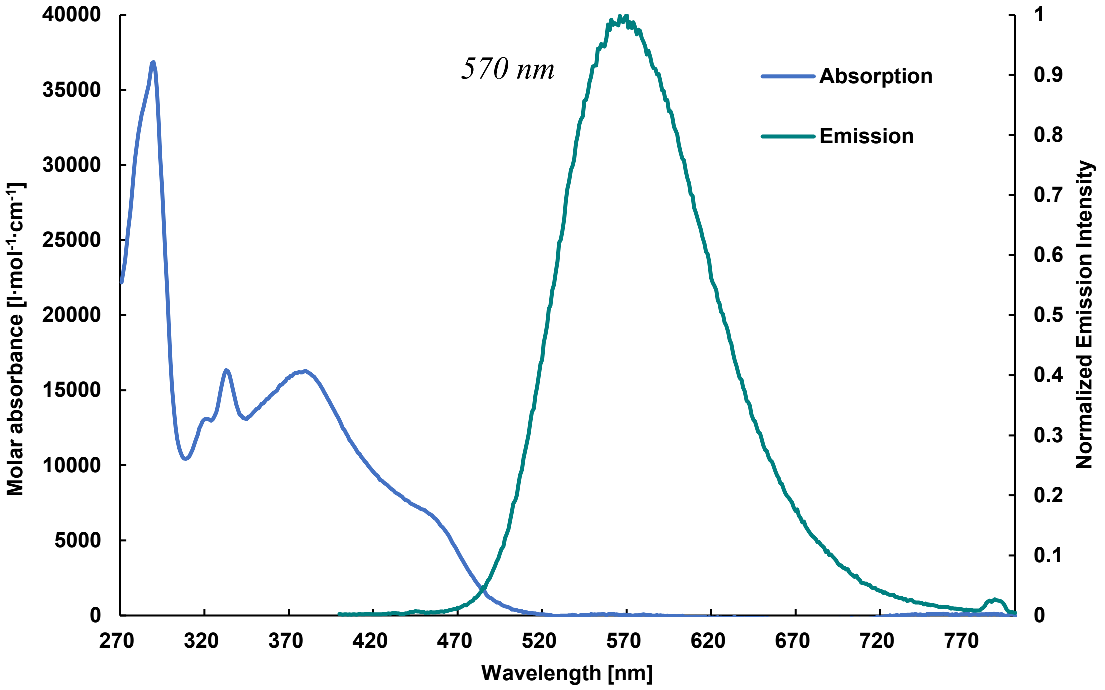

Figure S69. UV/Vis absorption and emission spectrum of **PC3** in degassed MeCN (20 µM). Excitation wavelength – 394 nm.

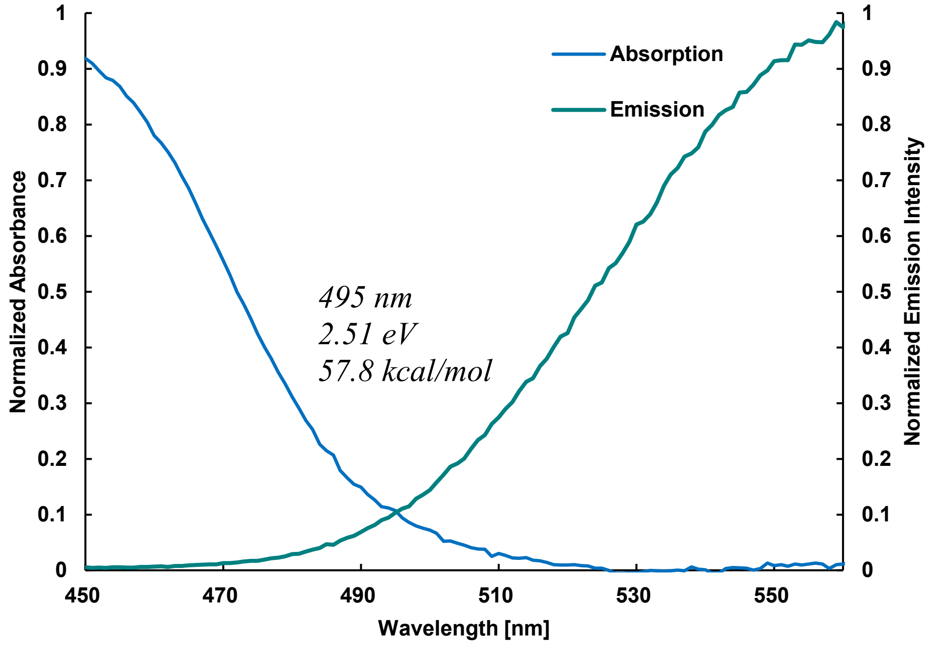

Figure S70. $\boldsymbol{E_{0\text{-}0}}$ estimation at normalized emission and absorption overlap of **PC3**.

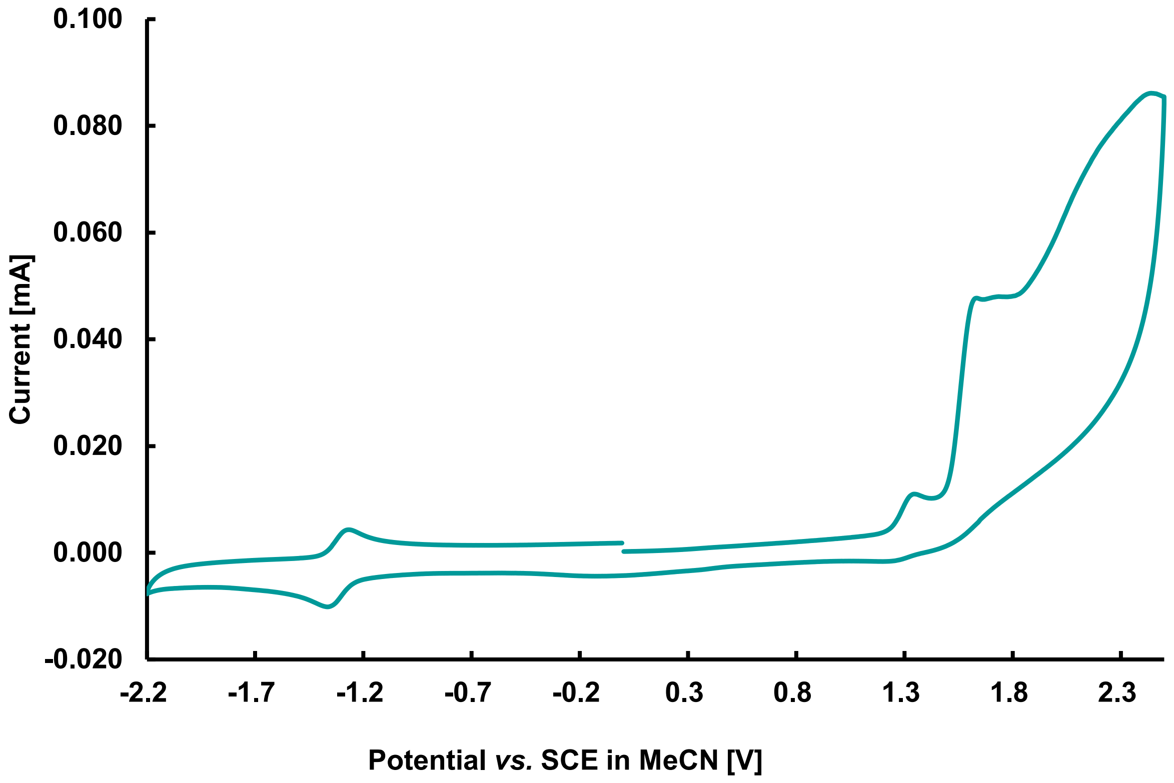

Figure S71. Cyclic voltammetry of **PC3** in degassed MeCN (0.4 mM) using 0.1 M $^{n}Bu_4NPF_6$ as electrolyte.

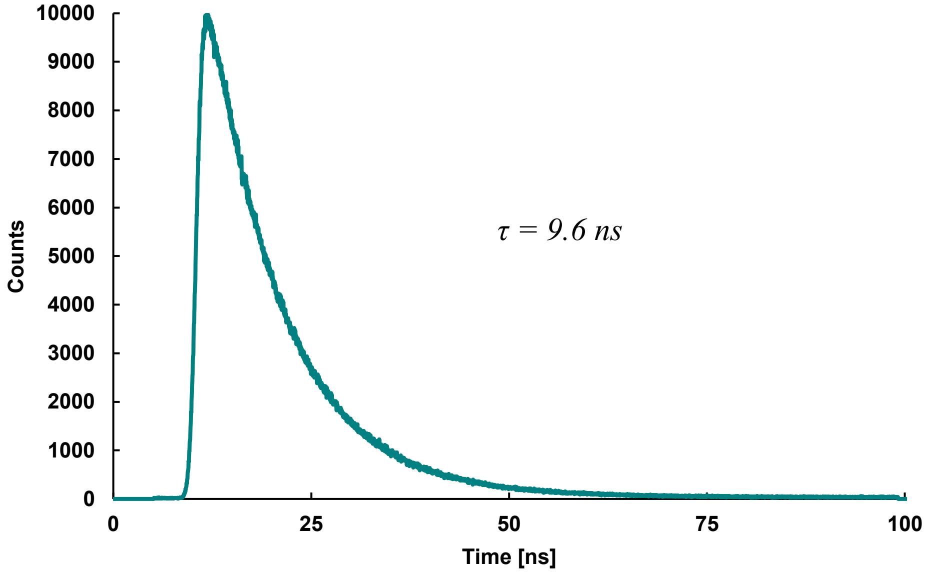

Figure S72. Fluorescence decay curve of **PC3** in degassed MeCN (20 µM). Excitation wavelenth – 394 nm.

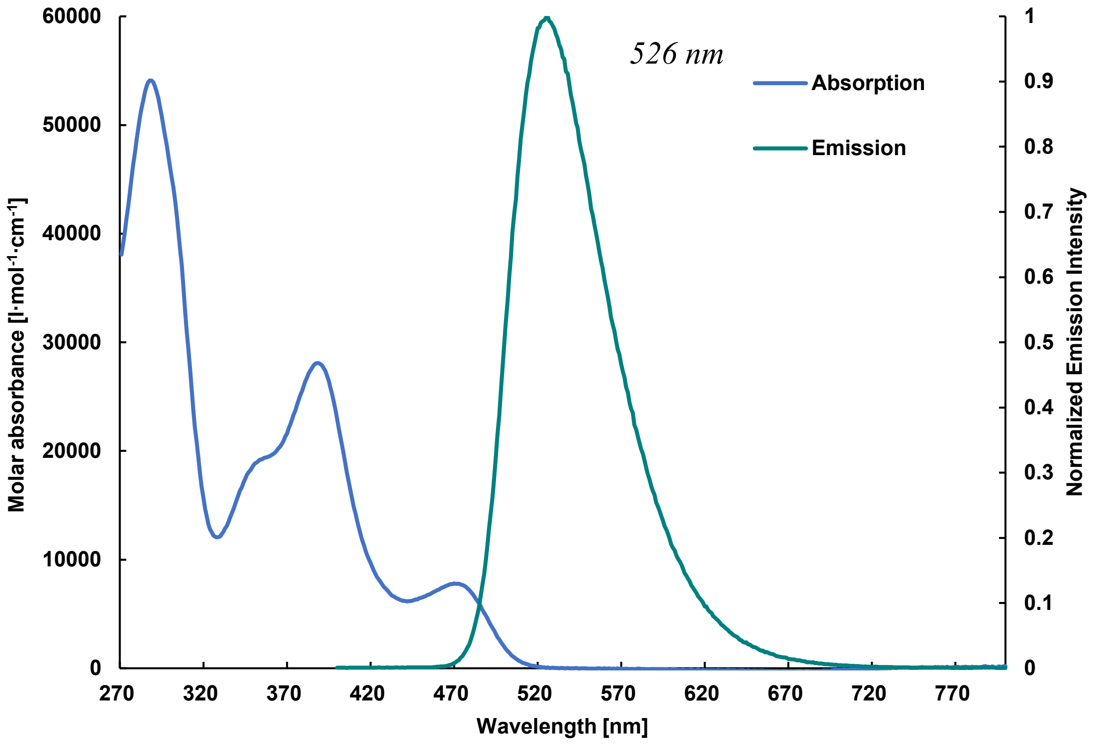

Figure S73. UV/Vis absorption and emission spectrum of **PC4** in degassed MeCN (20 µM). Excitation wavelength – 394 nm.

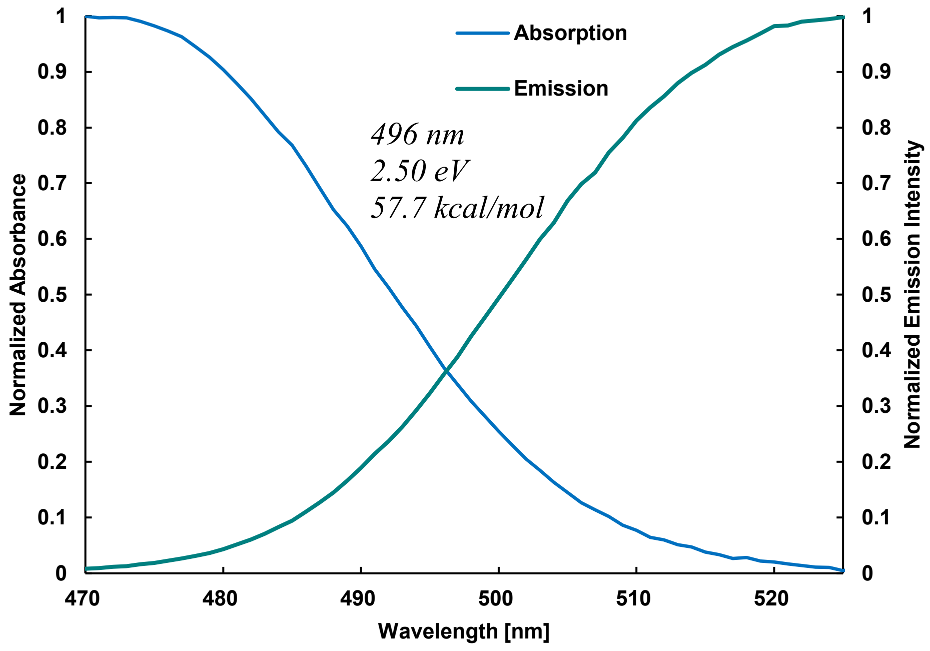

Figure S74. $E_{0\text{-}0}$ estimation at normalized emission and absorption overlap of **PC4**.74

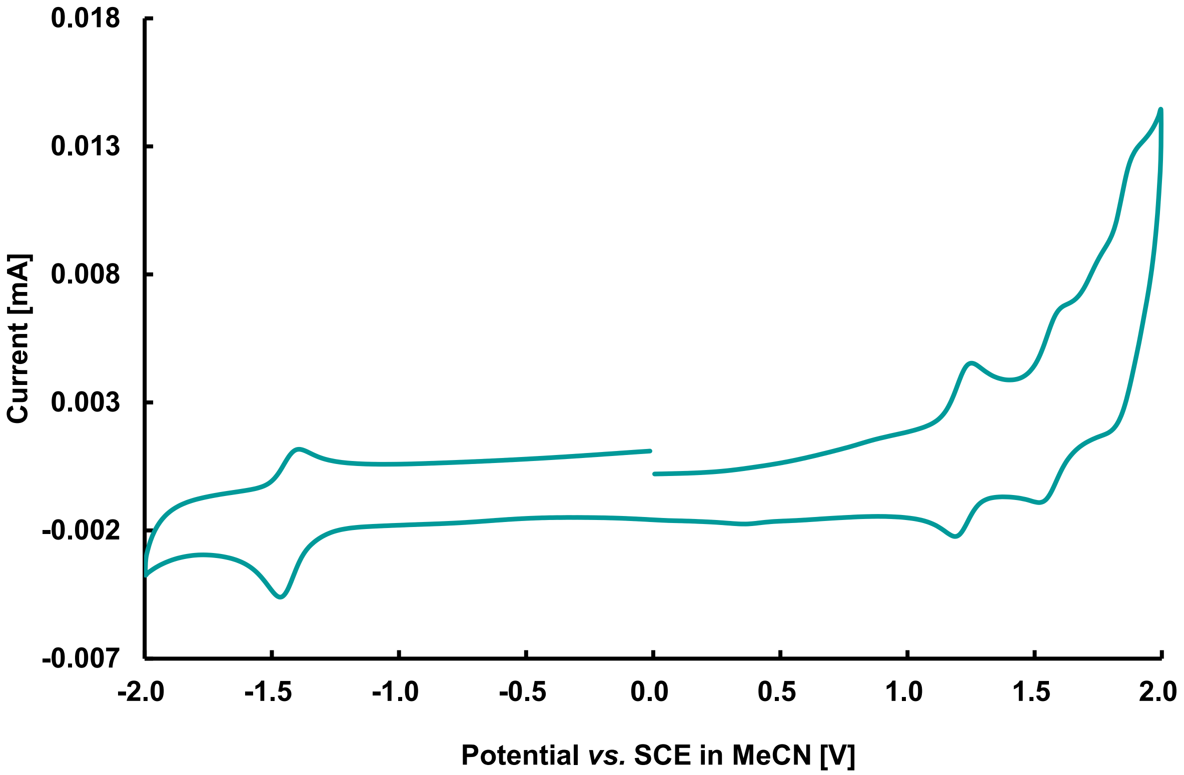

Figure S75. Cyclic voltammetry of **PC4** in degassed MeCN (0.4 mM) using 0.1 M $^{n}Bu_4NPF_6$ as electrolyte.

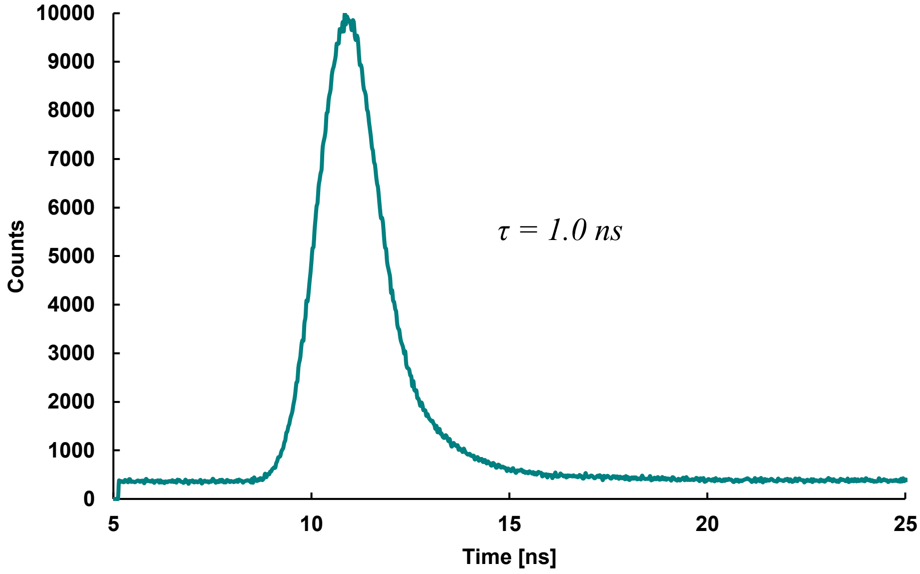

Figure S76. Fluorescence decay curve of **PC4** in degassed MeCN (20 μM). Excitation wavelenth – 394 nm.

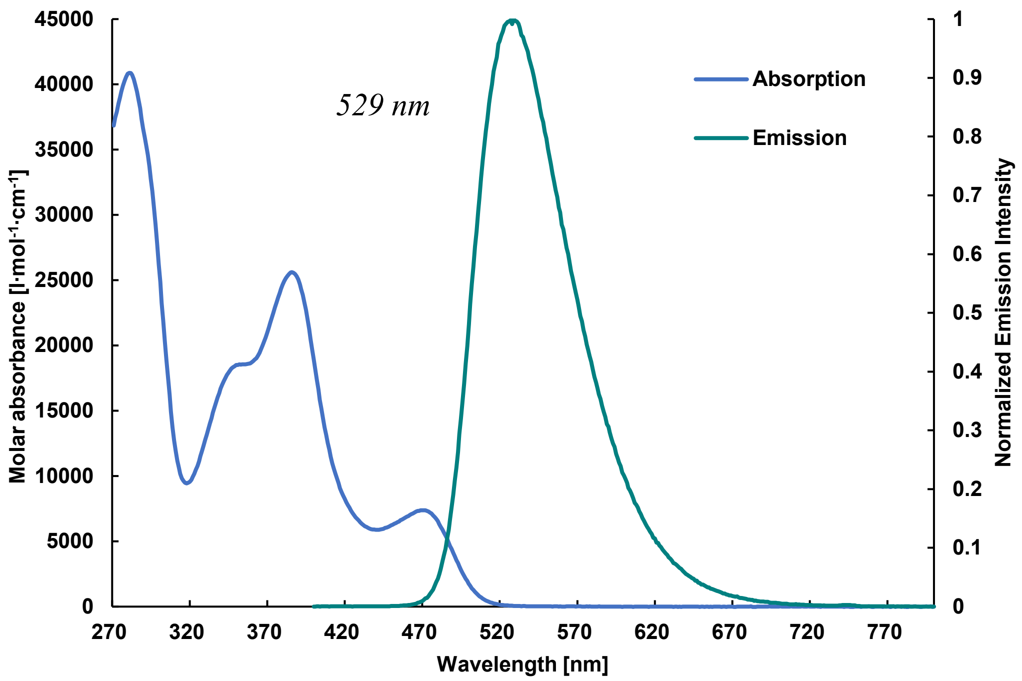

Figure S77. UV/Vis absorption and emission spectrum of **PC5** in degassed MeCN (20 μM). Excitation wavelength – 394 nm.

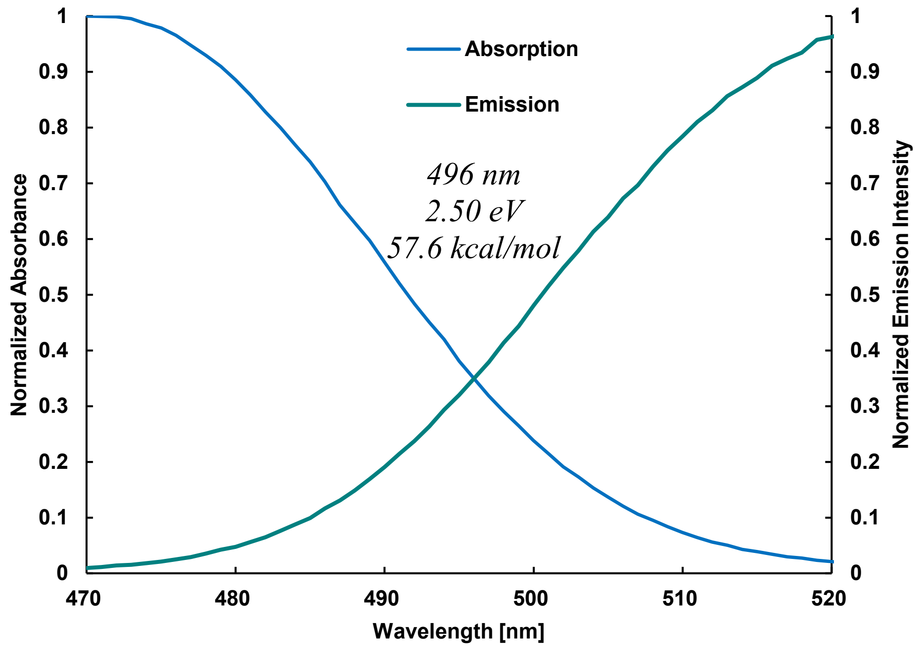

Figure S78. $E_{0\text{-}0}$ estimation at normalized emission and absorption overlap of **PC5**.

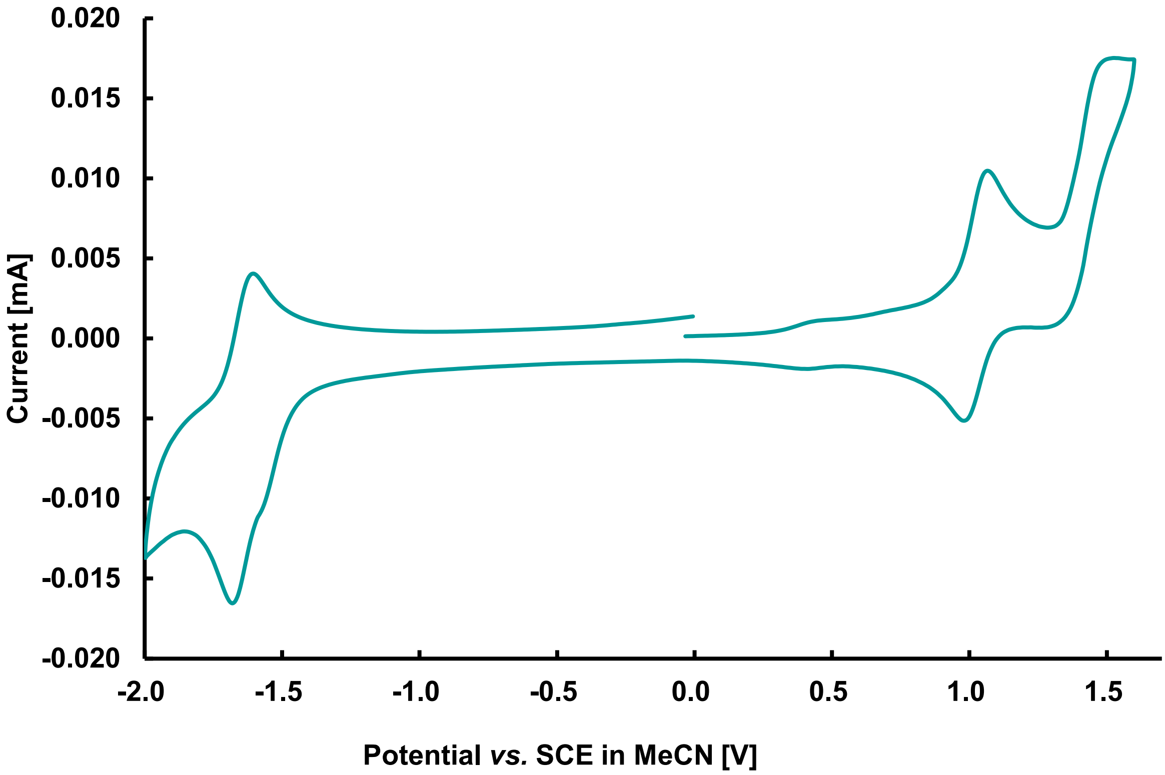

Figure S79. Cyclic voltammetry of **PC5** in degassed MeCN (0.4 mM) using 0.1 M $^{n}Bu_4NPF_6$ as electrolyte.

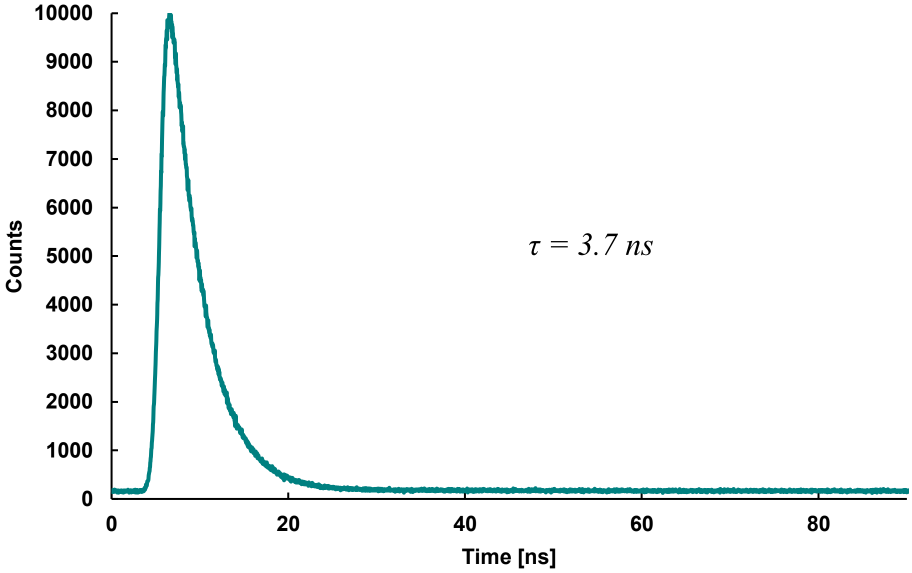

Figure S80. Fluorescence decay curve of **PC5** in degassed MeCN (20 μM). Excitation wavelength – 394 nm.

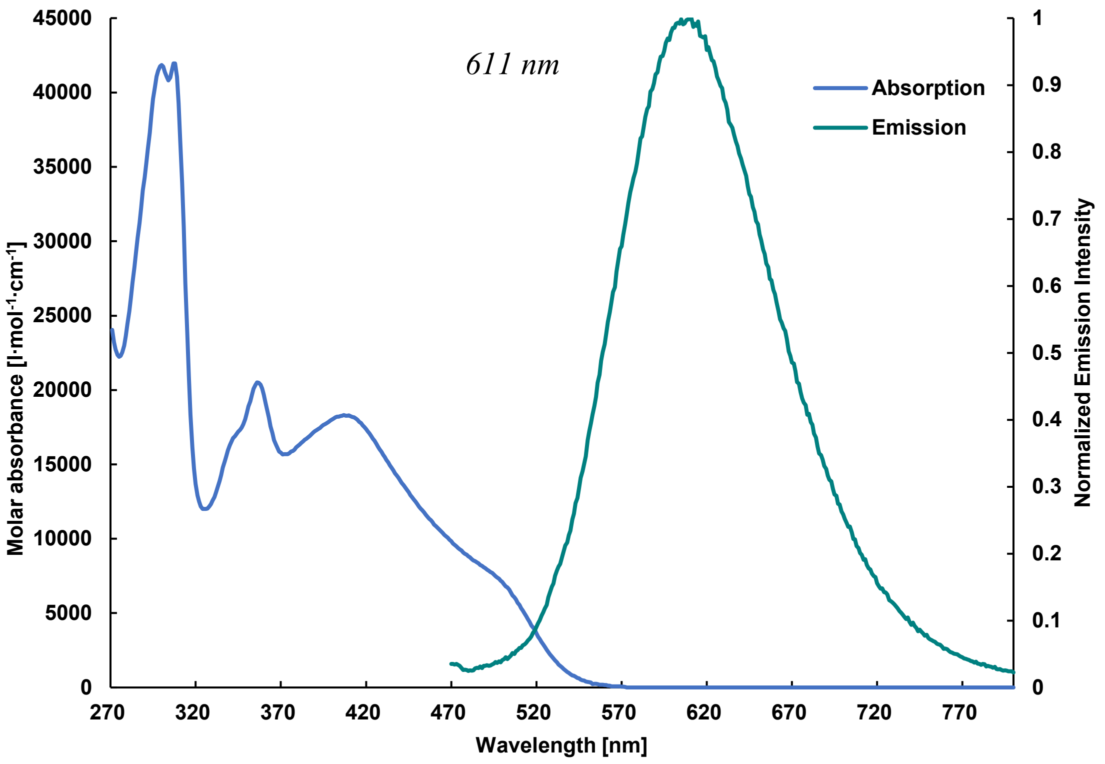

Figure S81. UV/Vis absorption and emission spectrum of **PC6** in degassed DCM (20 µM). Excitation wavelength – 456 nm.

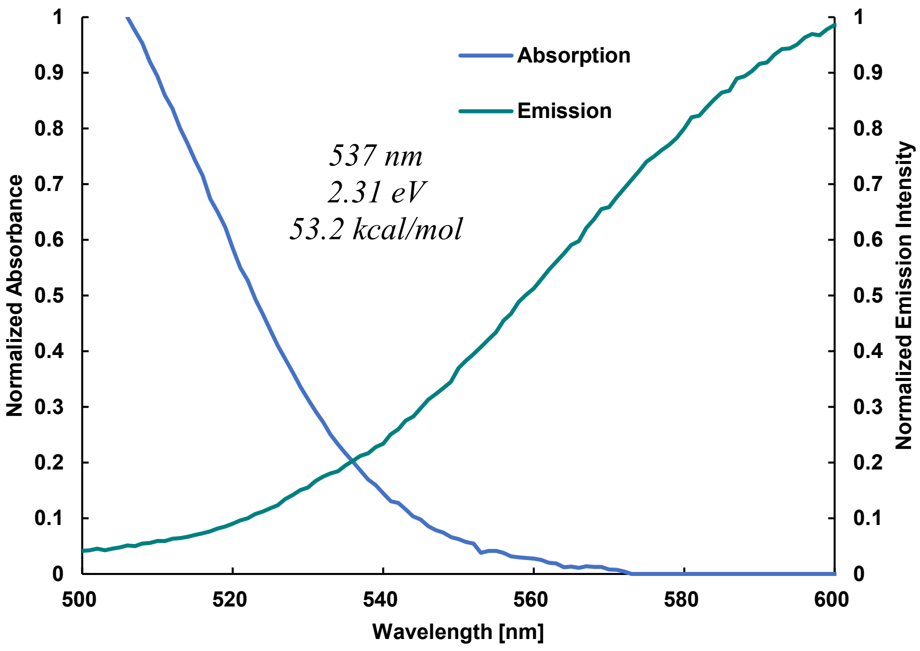

Figure S82. $E_{0\text{-}0}$ estimation at normalized emission and absorption overlap of **PC6**.

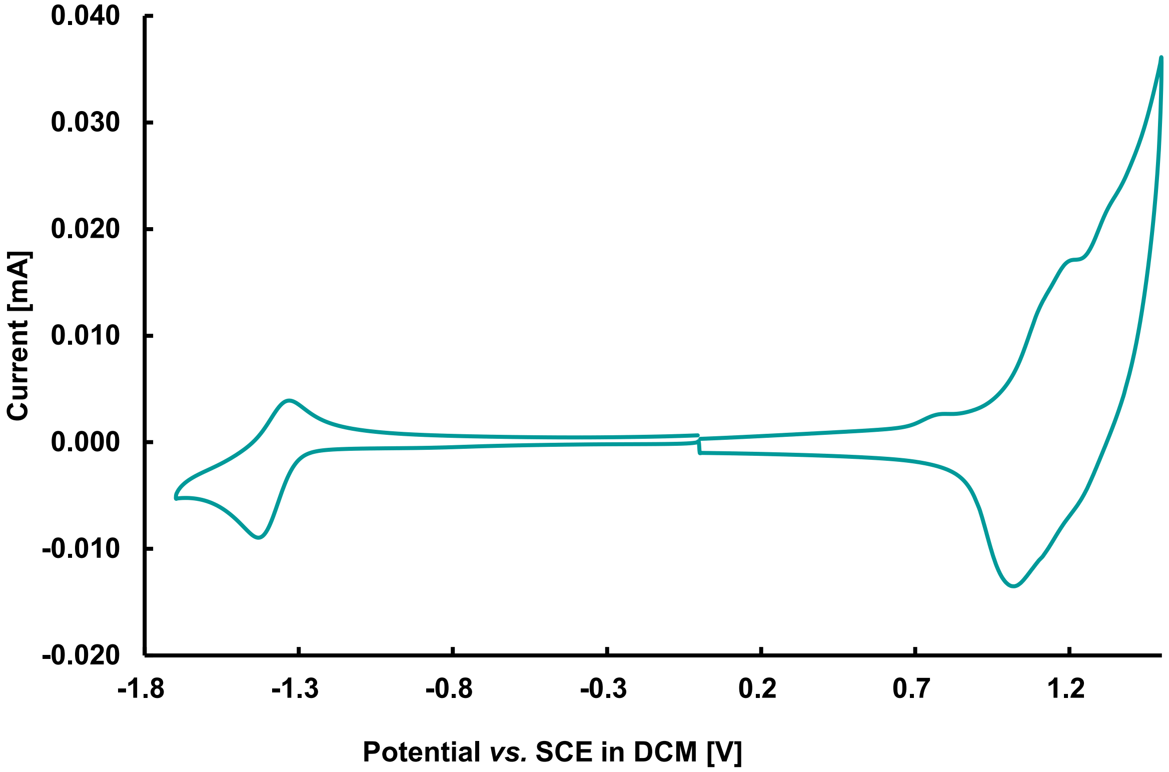

Figure S83. Cyclic voltammetry of **PC6** in degassed DCM (0.4 mM) using 0.1 M $^{n}Bu_4NPF_6$ as electrolyte.

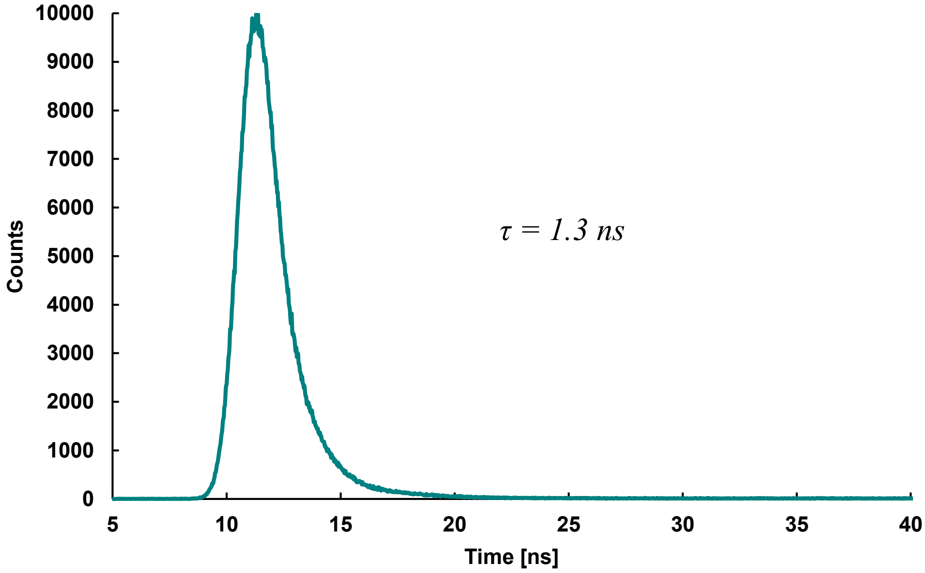

Figure S84. Fluorescence decay curve of **PC6** in degassed DCM (20 µM). Excitation wavelength – 457 nm.

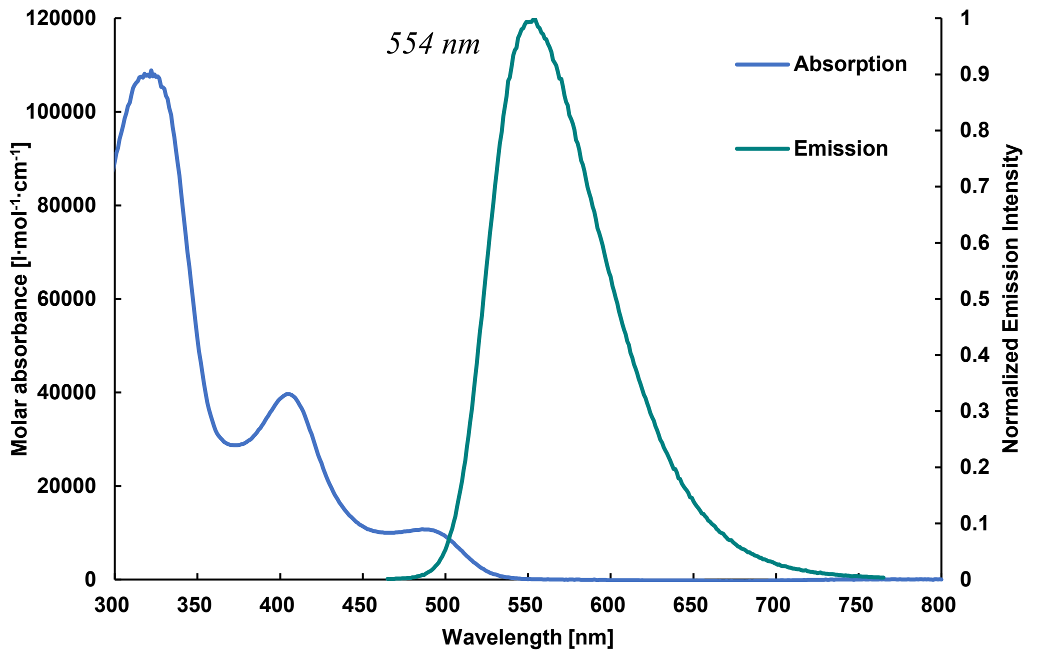

Figure S85. UV/Vis absorption and emission spectrum of **PC7** in degassed DCM (20 µM). Excitation wavelength – 394 nm.

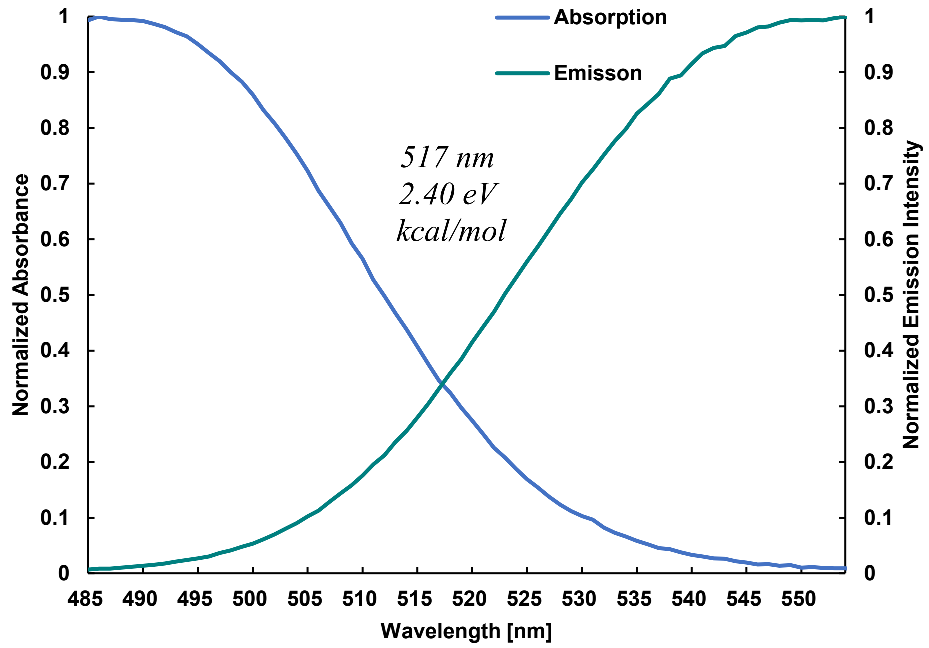

Figure S86. $E_{0\text{-}0}$ estimation at normalized emission and absorption overlap of **PC7**.

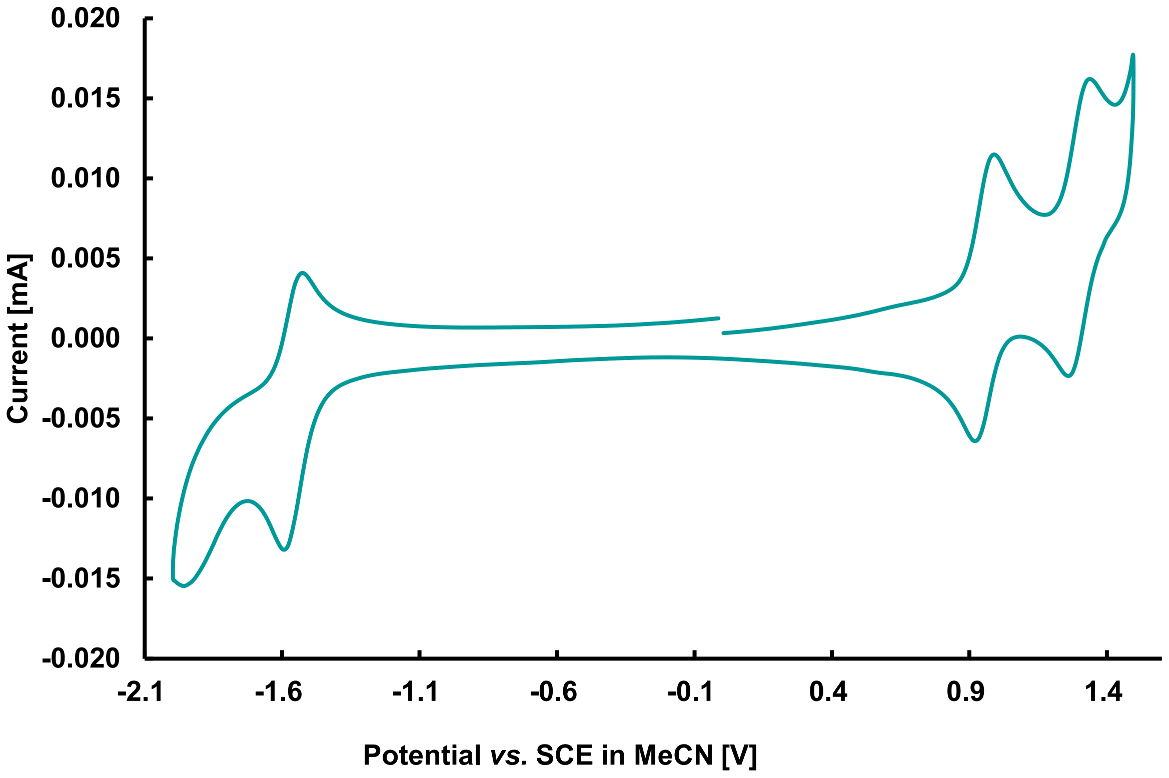

Figure S87. Cyclic voltammetry of **PC7** in degassed MeCN (0.4 mM) using 0.1 M $^{n}Bu_4NPF_6$ as electrolyte.

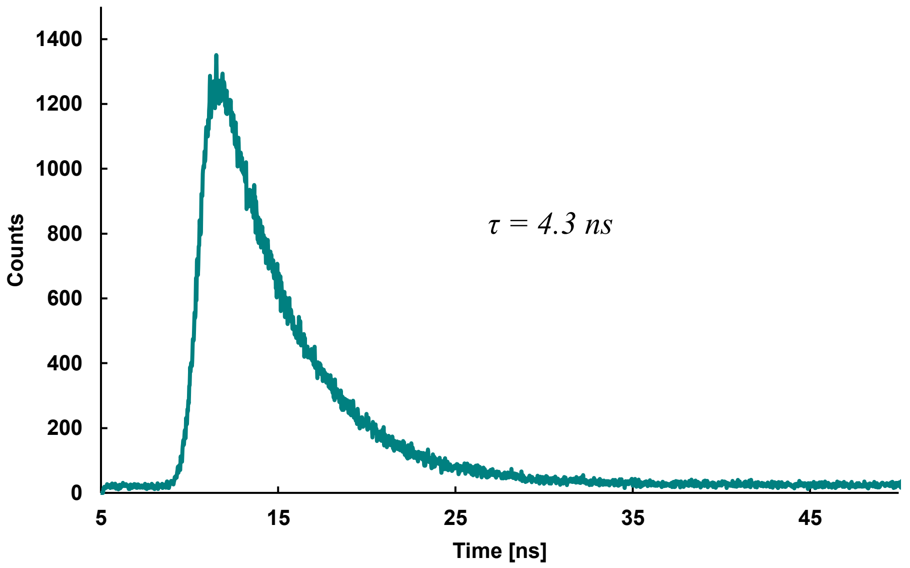

Figure S88. Fluorescence decay curve of **PC7** in degassed MeCN (20 µM). Excitation wavelength – 394 nm.

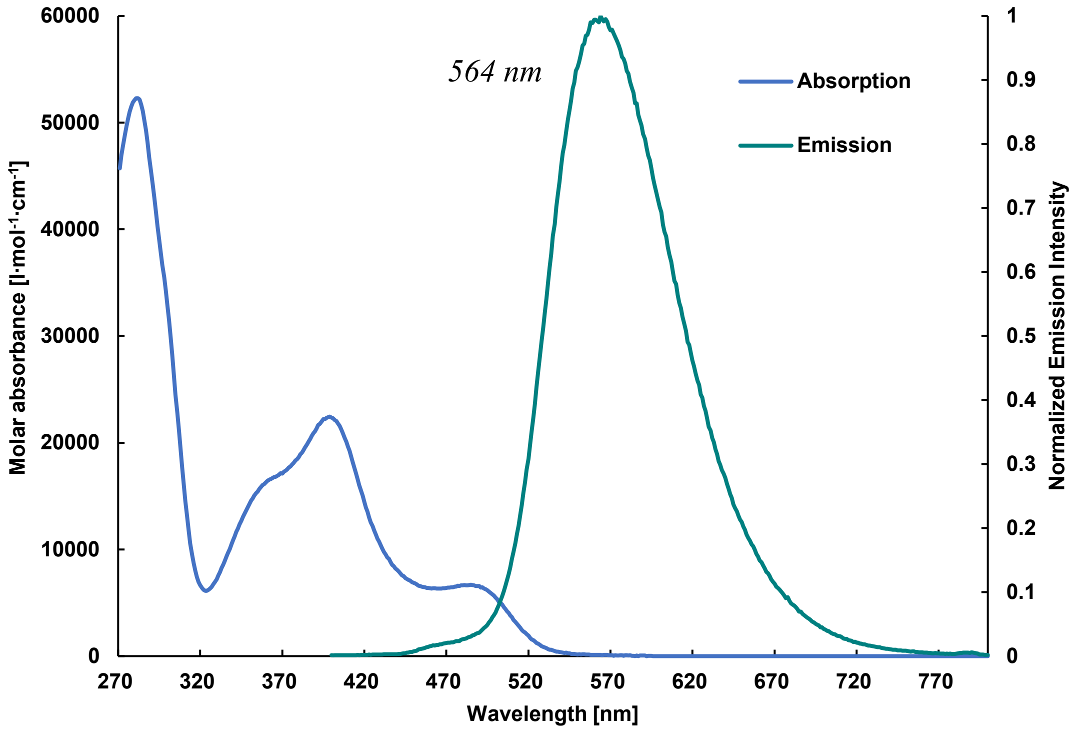

Figure S89. UV/Vis absorption and emission spectrum of **PC8** in degassed MeCN (20 μM). Excitation wavelength – 394 nm.

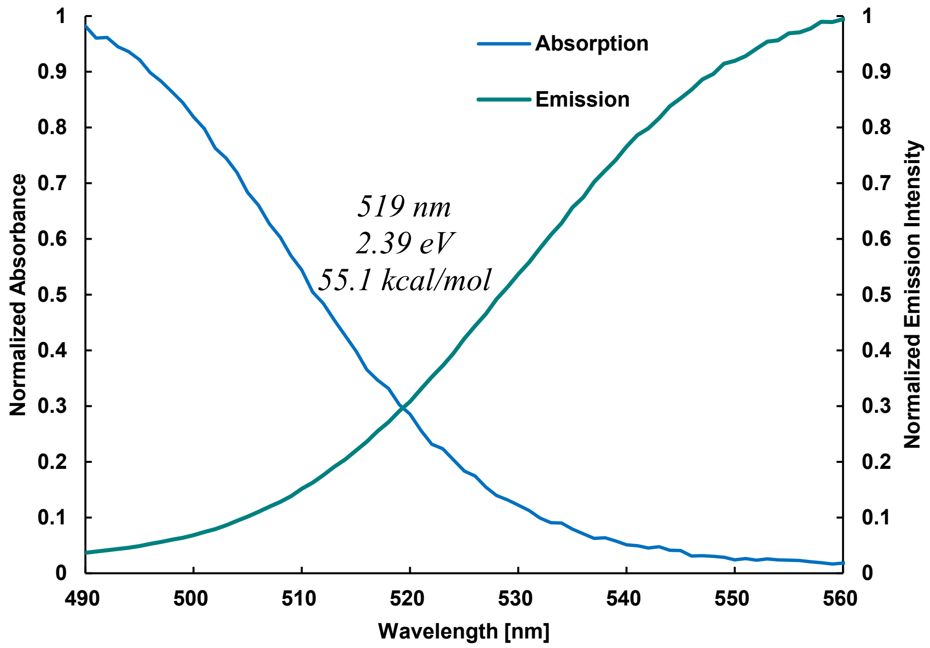

Figure S90. $E_{0\text{-}0}$ estimation at normalized emission and absorption overlap of **PC8.**

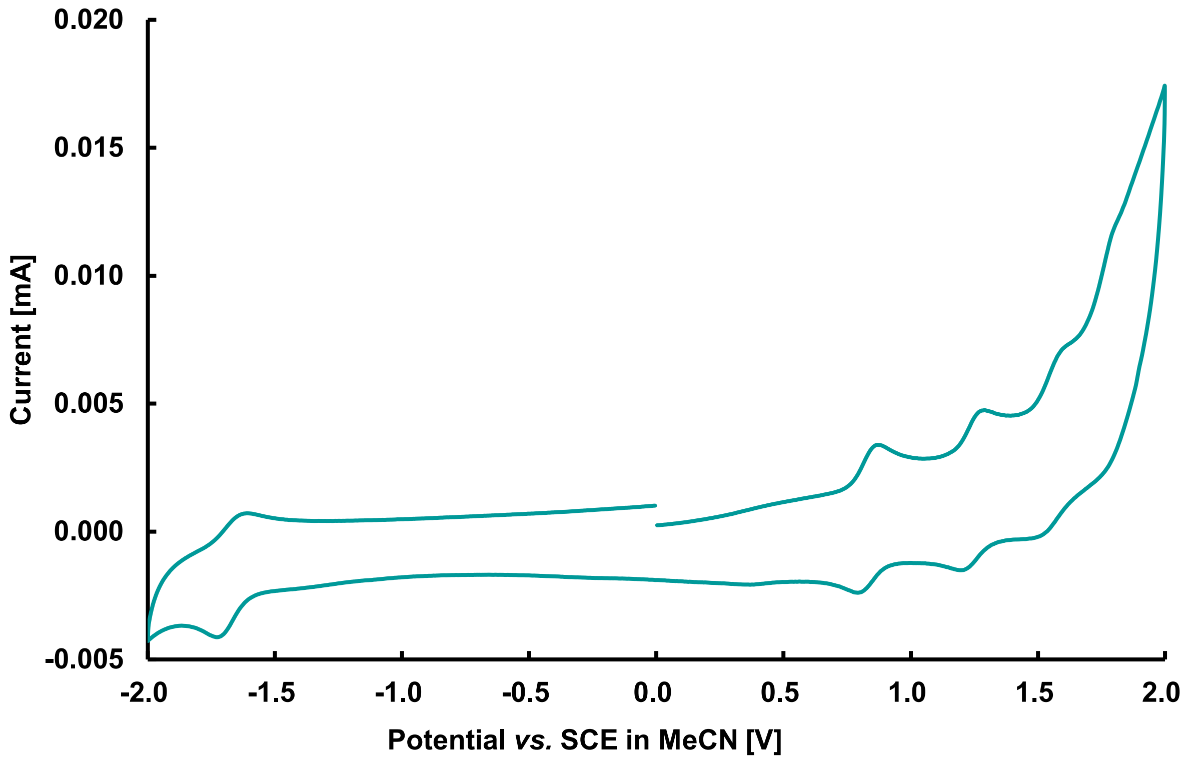

Figure S91. Cyclic voltammetry of **PC8** in degassed MeCN (0.4 mM) using 0.1 M $^{n}Bu_4NPF_6$ as electrolyte.

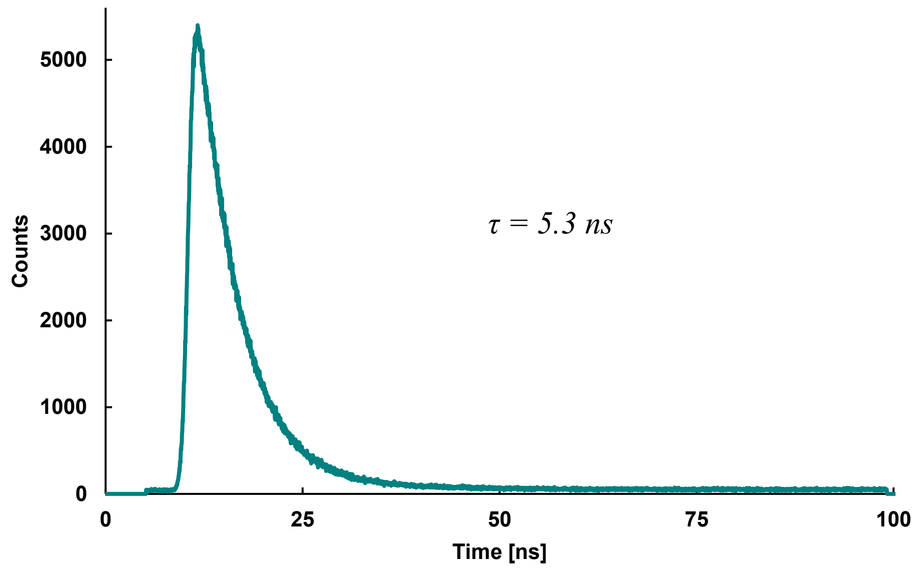

Figure S92. Fluorescence decay curve of **PC8** in degassed MeCN (20 µM). Excitation wavelength – 394 nm.

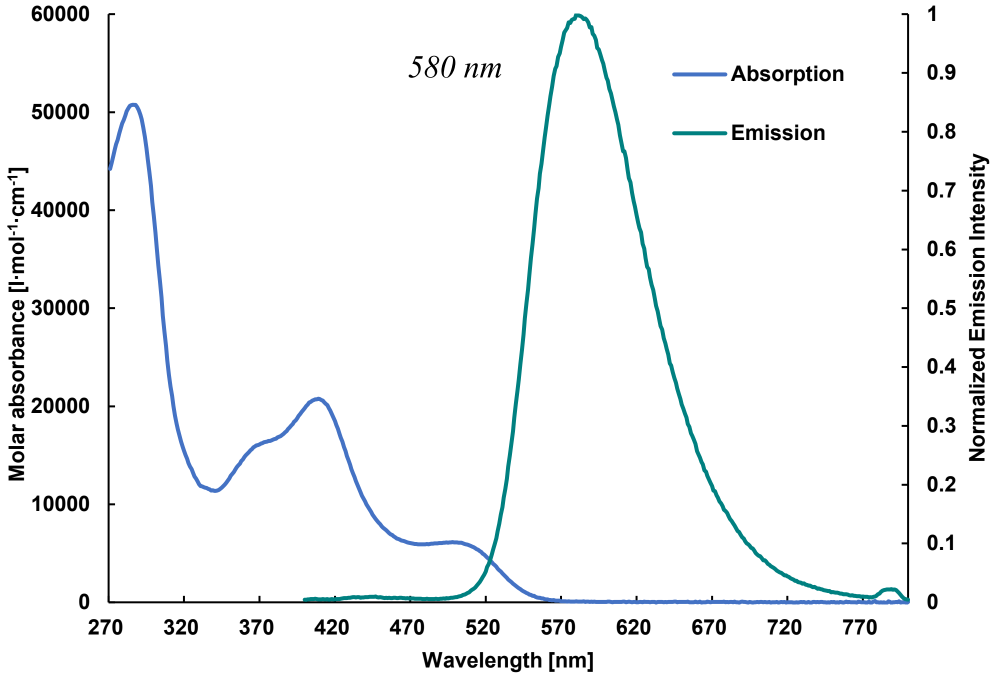

Figure S93. UV/Vis absorption and emission spectrum of **PC9** in degassed DCM (20 μM). Excitation wavelength – 394 nm.

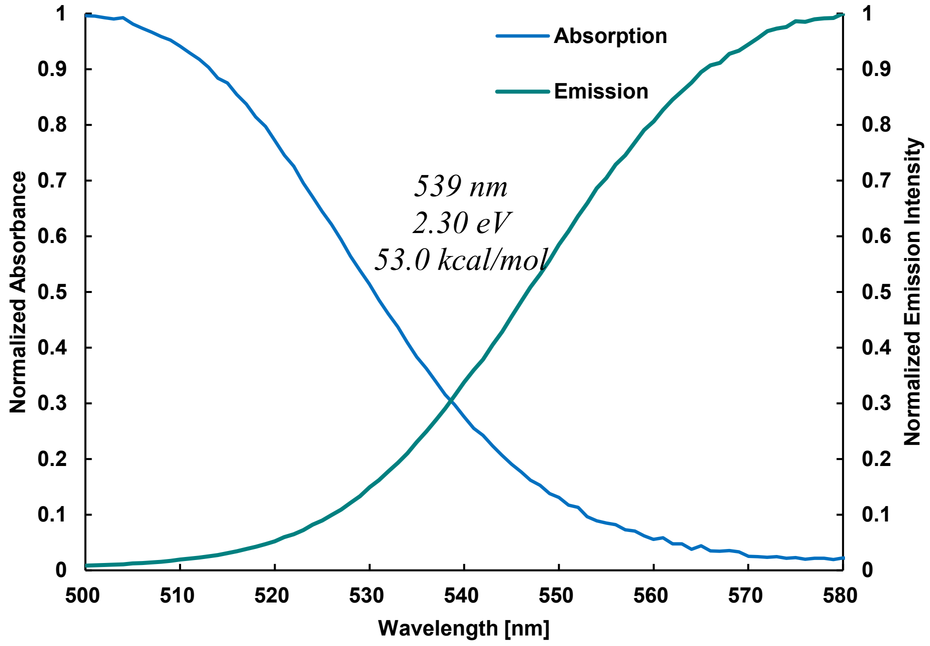

Figure S94. $E_{0\text{-}0}$ estimation at normalized emission and absorption overlap of **PC9**.

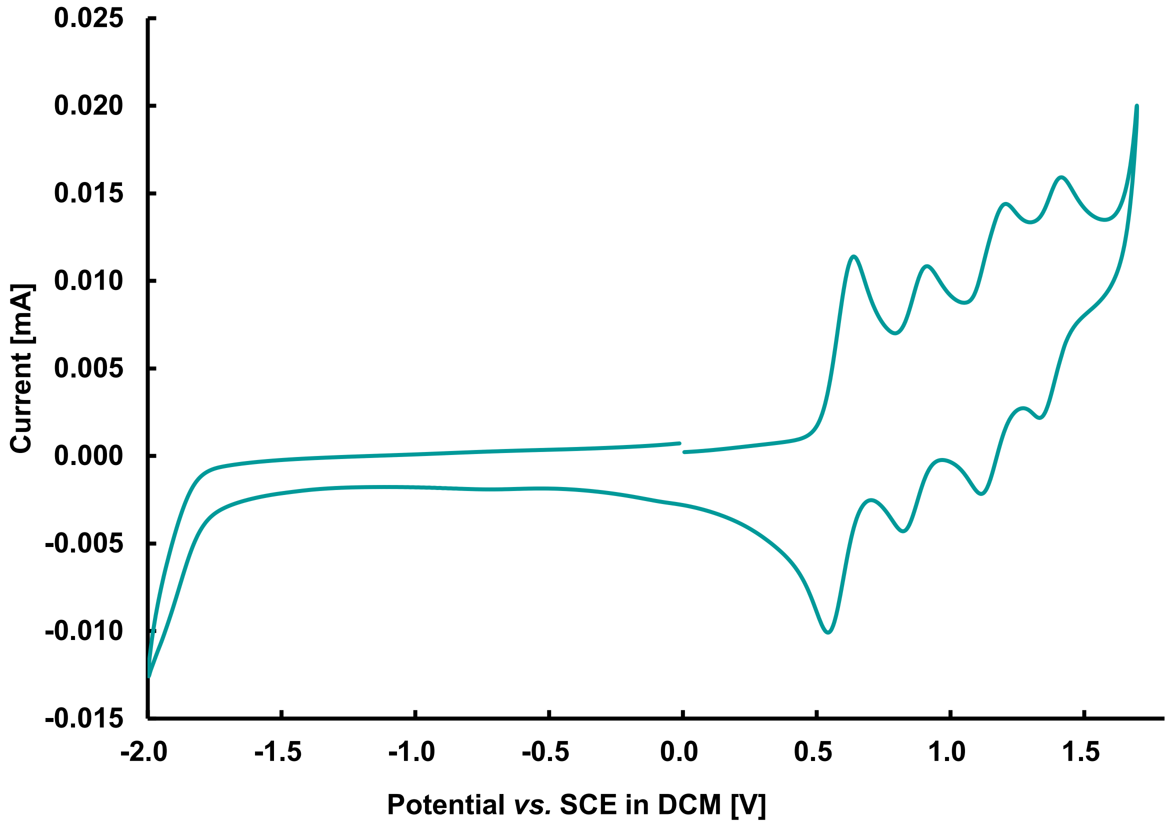

Figure S95. Cyclic voltammetry of **PC9** in degassed DCM (0.4 mM) using 0.1 M $^{n}Bu_4NPF_6$ as electrolyte.

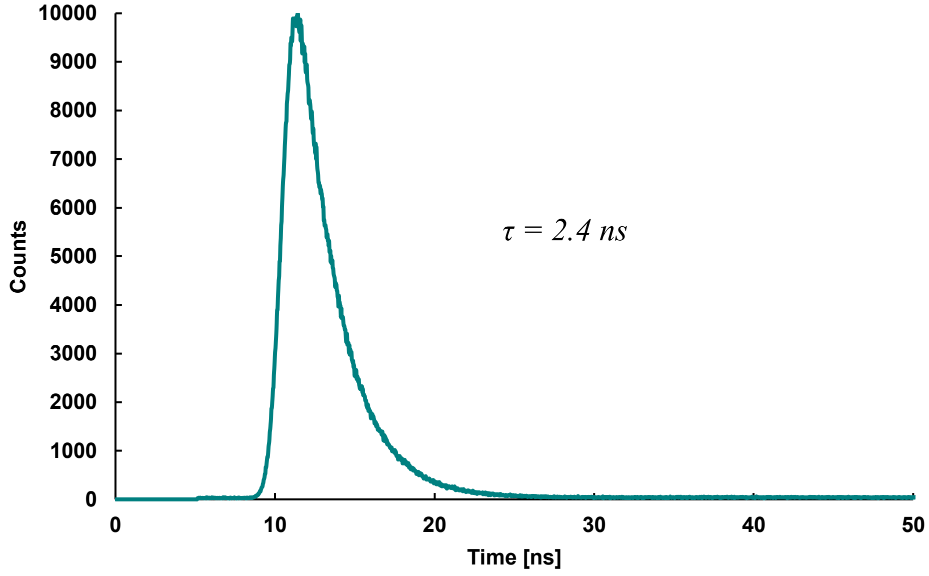

Figure S96. Fluorescence decay curve of **PC9** in degassed DCM (20 µM). Excitation wavelength – 394 nm.

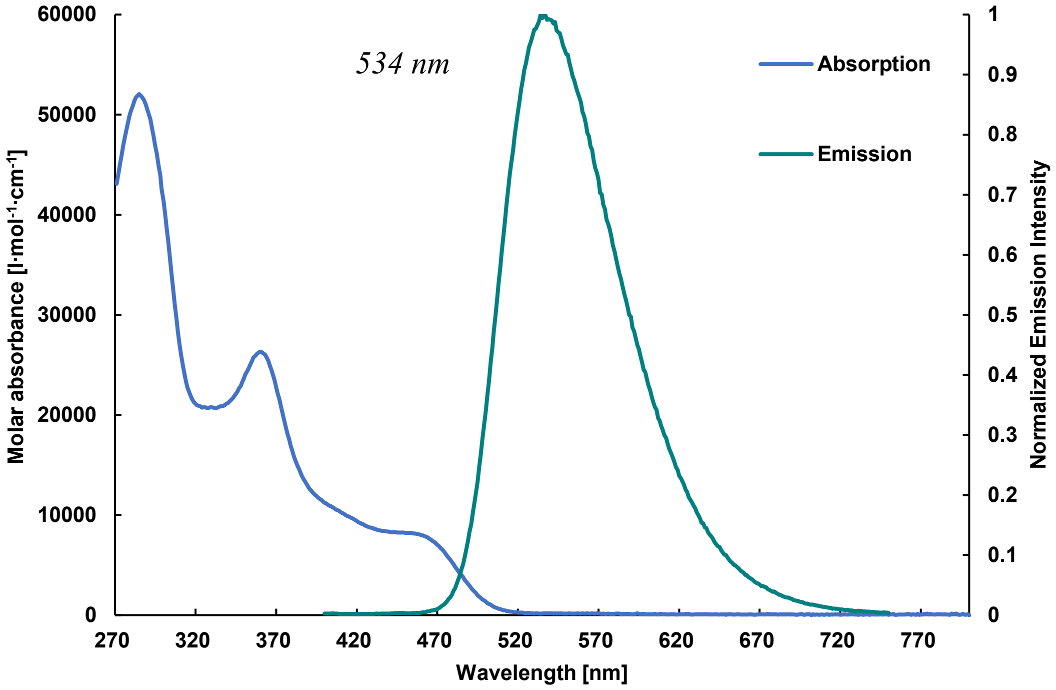

Figure S97. UV/Vis absorption and emission spectrum of **PC10** in degassed DCM (20 µM). Excitation wavelength – 394 nm.

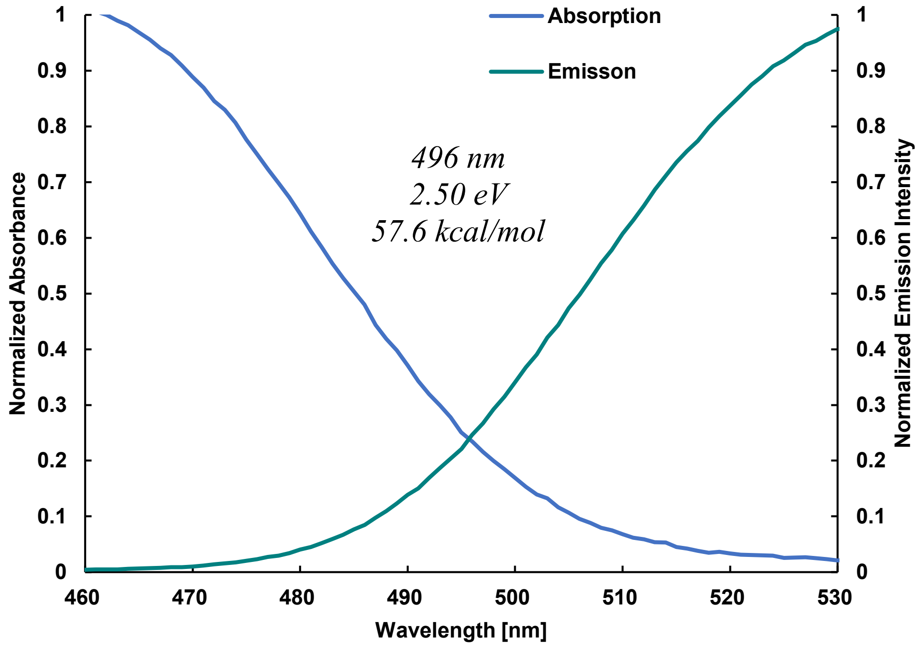

Figure S98. E_{0-0} estimation at normalized emission and absorption overlap of **PC10**.

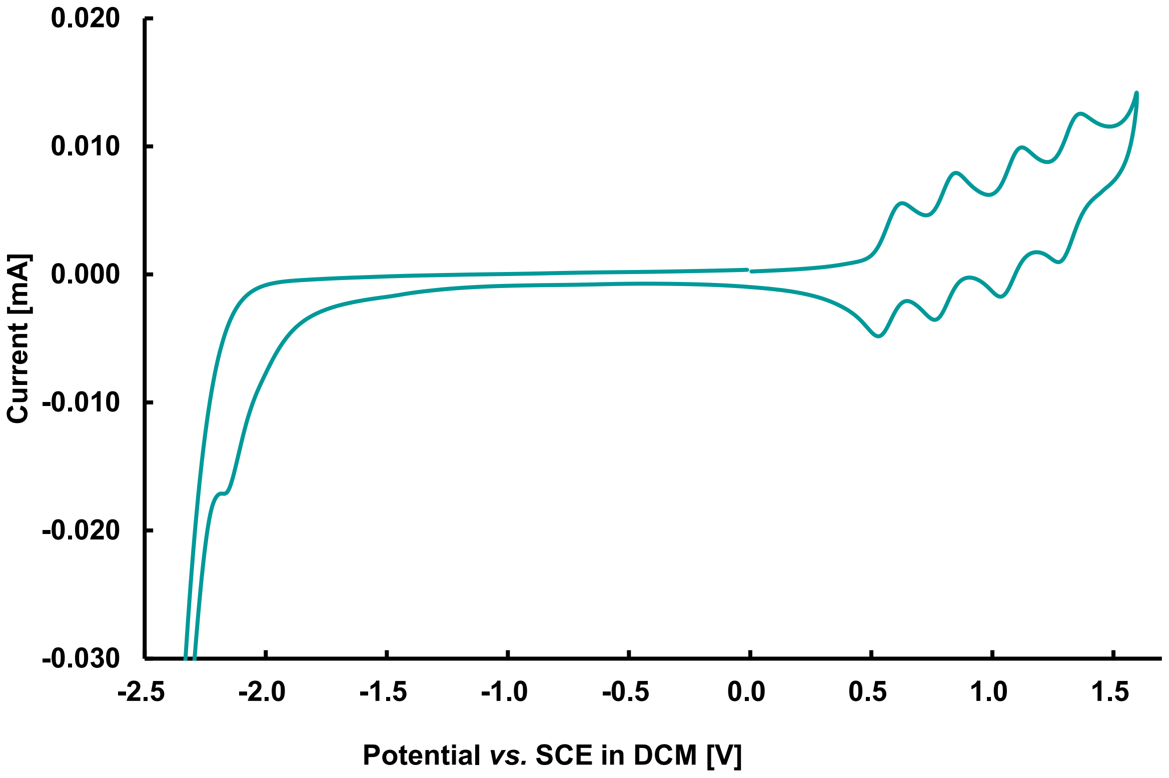

Figure S99. Cyclic voltammetry of **PC10** in degassed DCM (0.4 mM) using 0.1 M $^{n}Bu_4NPF_6$ as electrolyte. The scan rate is 50 mV/sec.

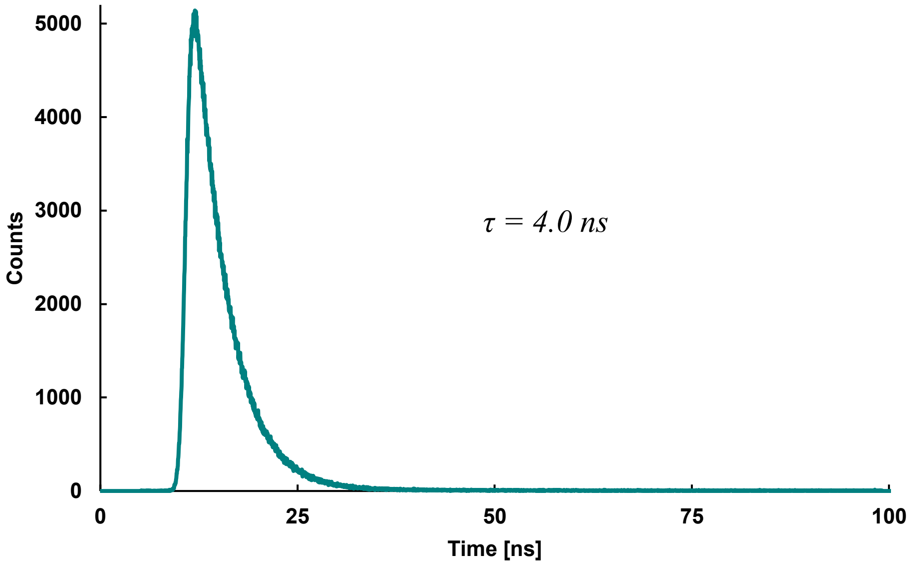

Figure S100. Fluorescence decay curve of **PC10** in degassed DCM (20 µM). Excitation wavelength – 457 nm.

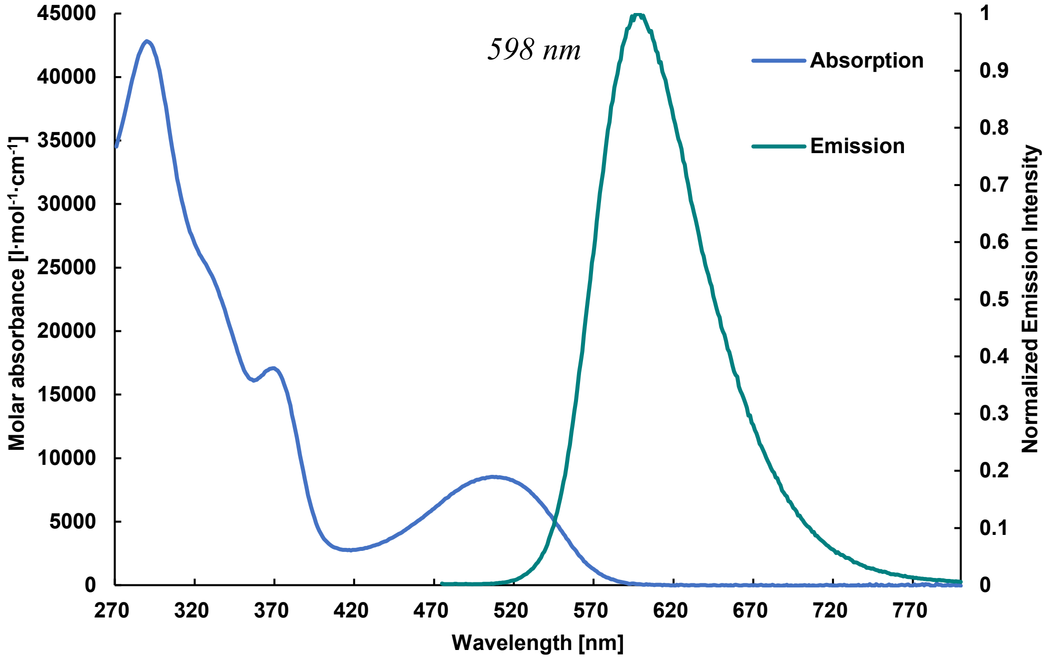

Figure S101. UV/Vis absorption and emission spectrum of **PC11** in degassed DCM (20 µM). Excitation wavelength – 456 nm.

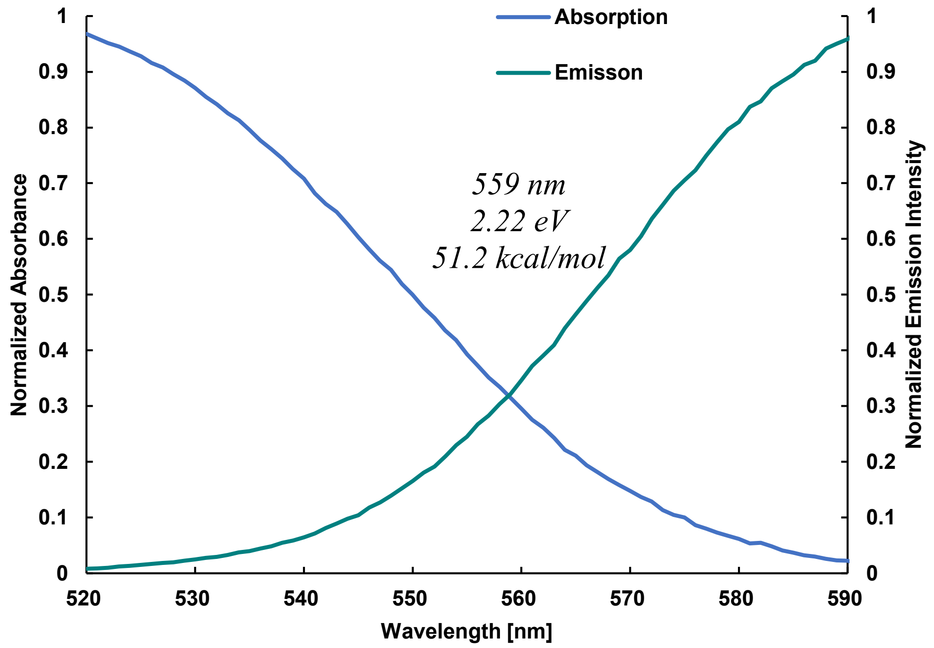

Figure S102. $E_{0\text{-}0}$ estimation at normalized emission and absorption overlap of **PC11**.

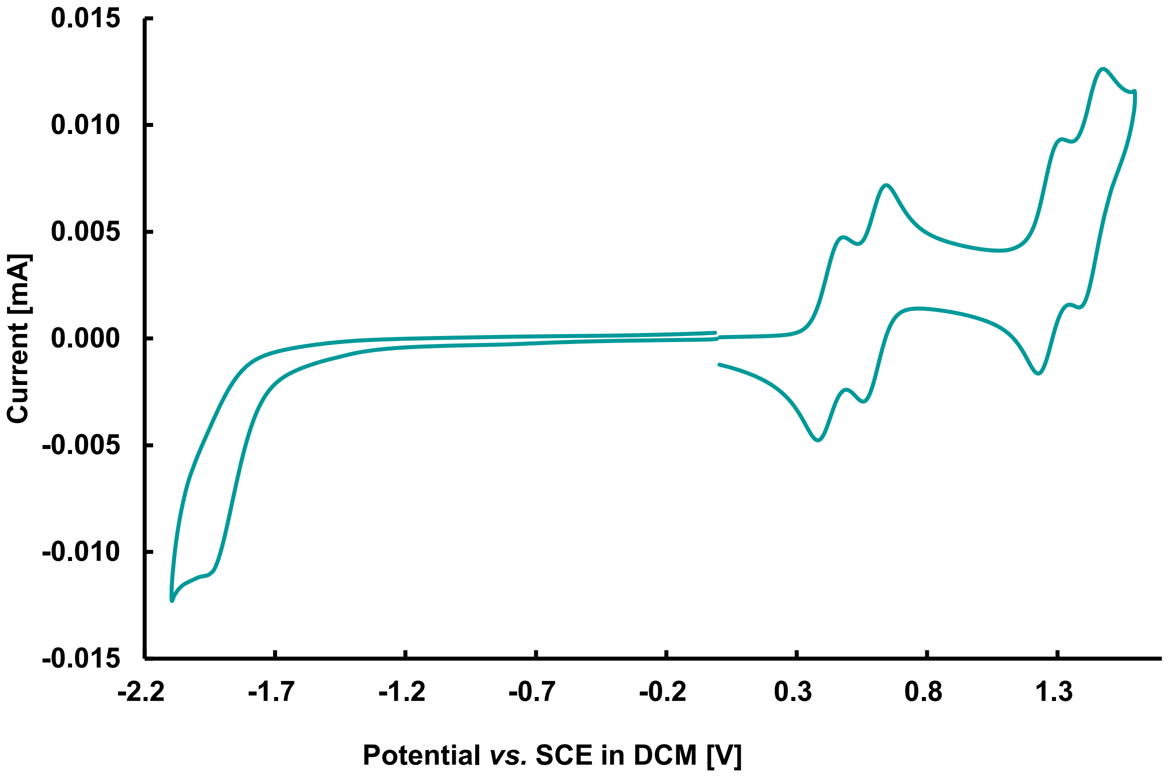

Figure S103. Cyclic voltammetry of **PC11** in degassed DCM (0.4 mM) using 0.1 M $^{n}Bu_4NPF_6$ as electrolyte. The scan rate is 50 mV/sec.

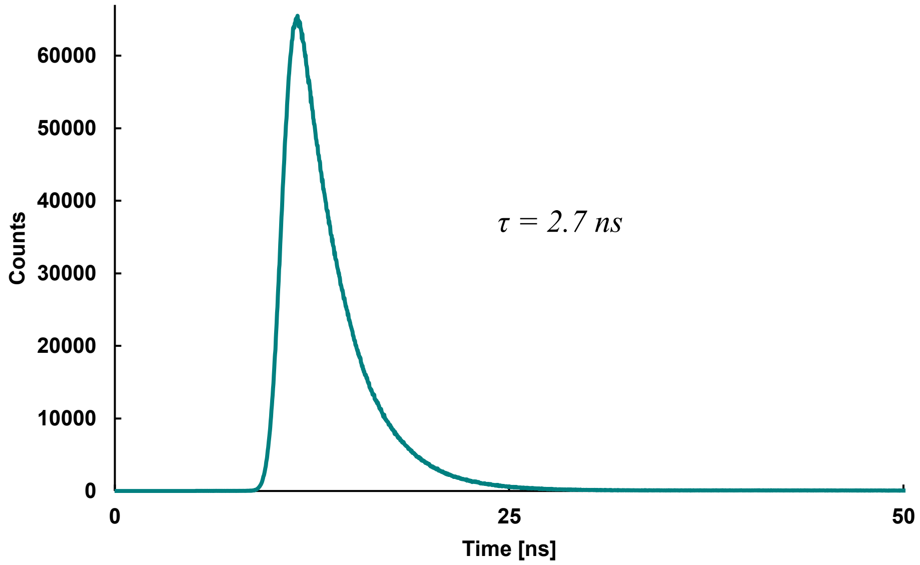

Figure S104. Fluorescence decay curve of **PC11** in degassed DCM (20 µM). Excitation wavelength – 457 nm.

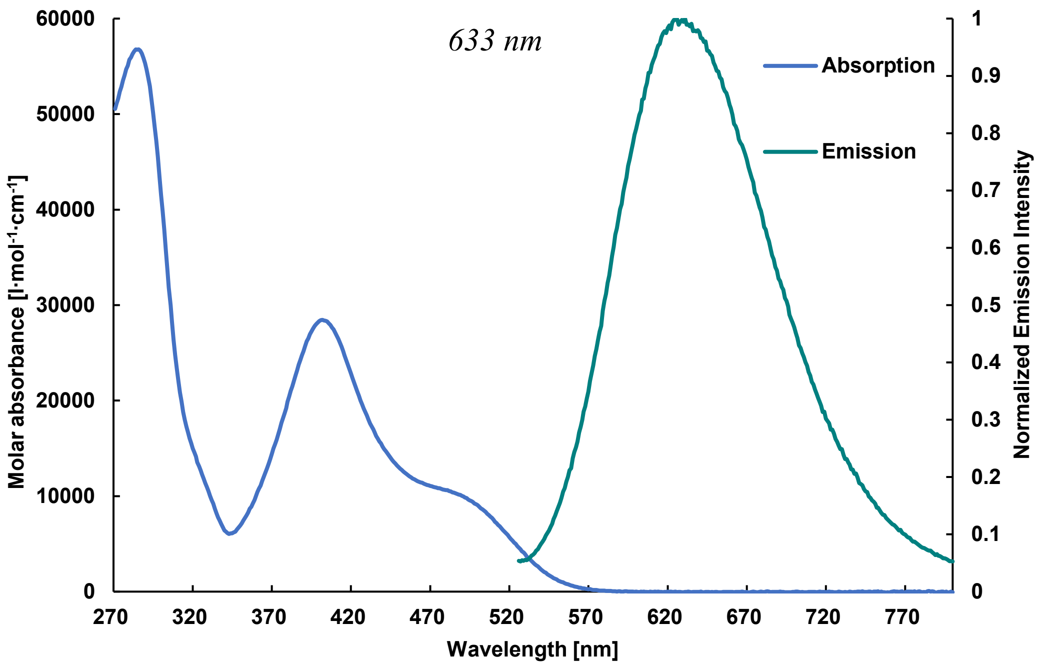

Figure S105. UV/Vis absorption and emission spectrum of **PC12** in degassed DCM (20 µM). Excitation wavelength – 500 nm.

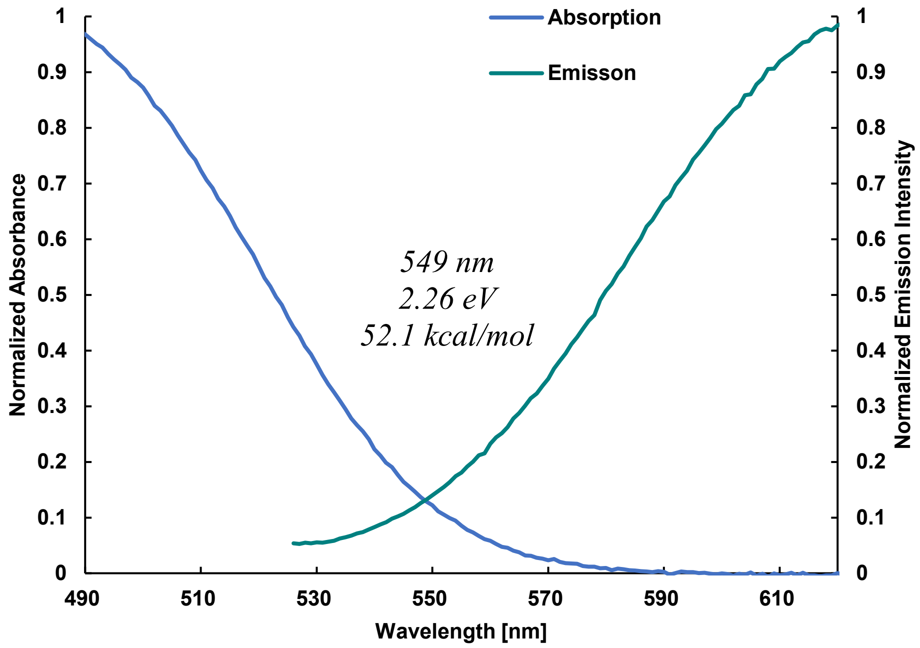

Figure S106. $E_{0\text{-}0}$ estimation at normalized emission and absorption overlap of **PC12**.

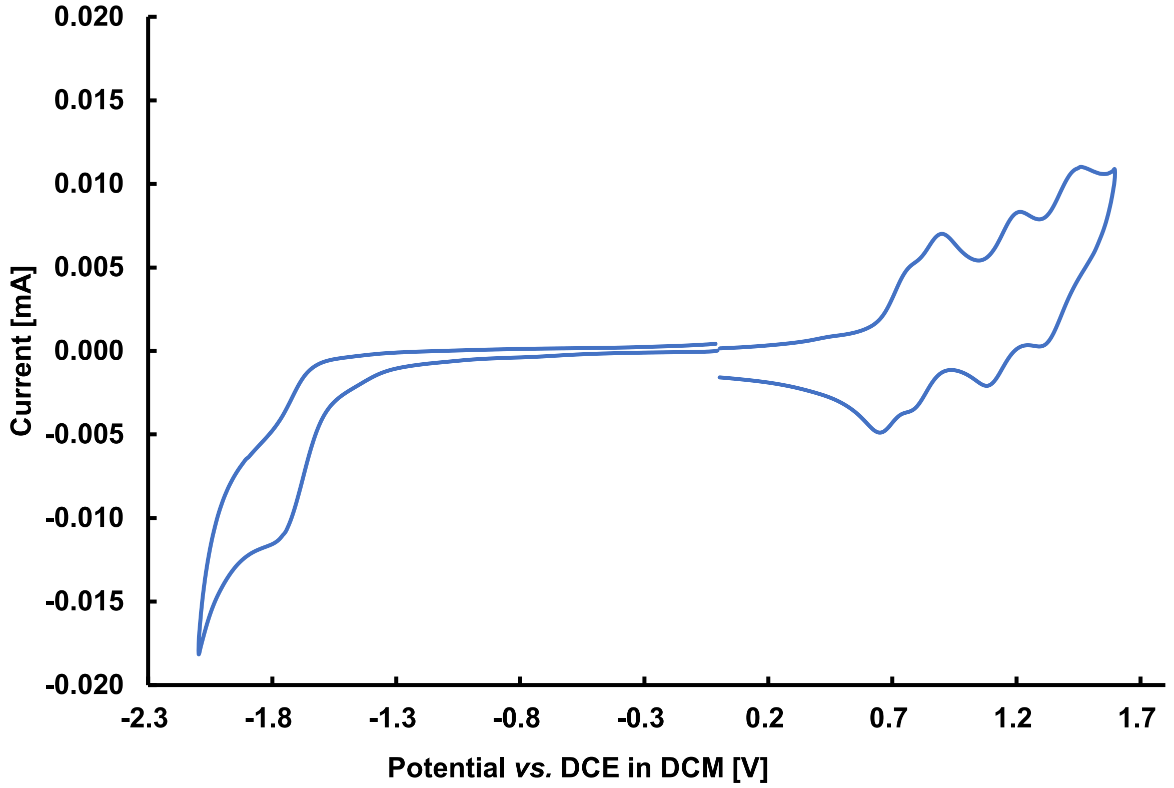

Figure S107. Cyclic voltammetry of **PC12** in degassed DCM (0.4 mM) using 0.1 M $^{n}Bu_4NPF_6$ as electrolyte. The scan rate is 50 mV/sec.

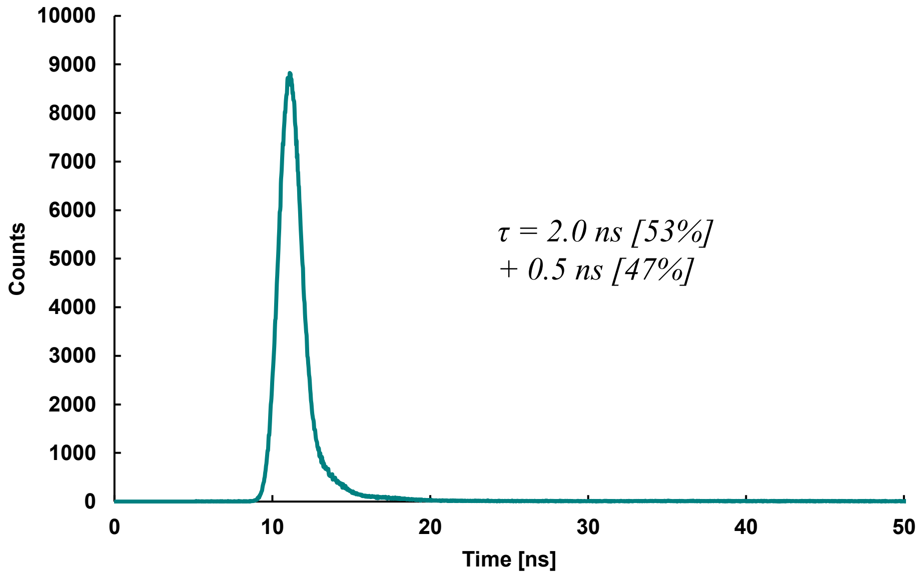

Figure S108. Fluorescence decay curve of **PC12** in degassed DCM (20 μM). Excitation wavelength – 457 nm.

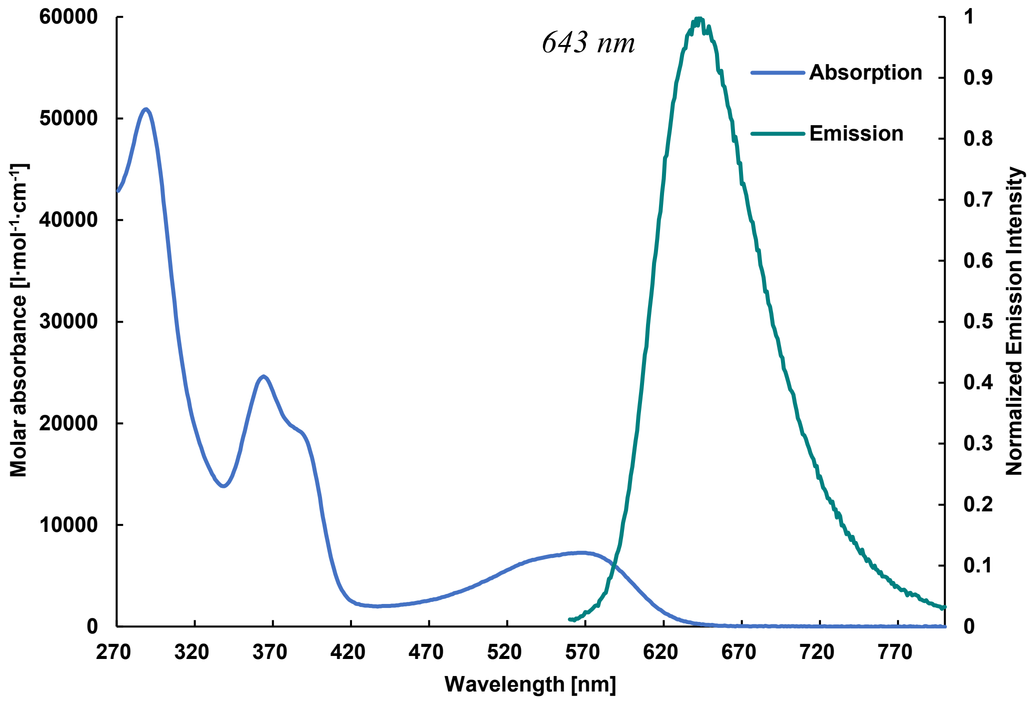

Figure S109 UV/Vis absorption and emission spectrum of **PC13** in degassed DCM (20 µM). Excitation wavelength – 550 nm.

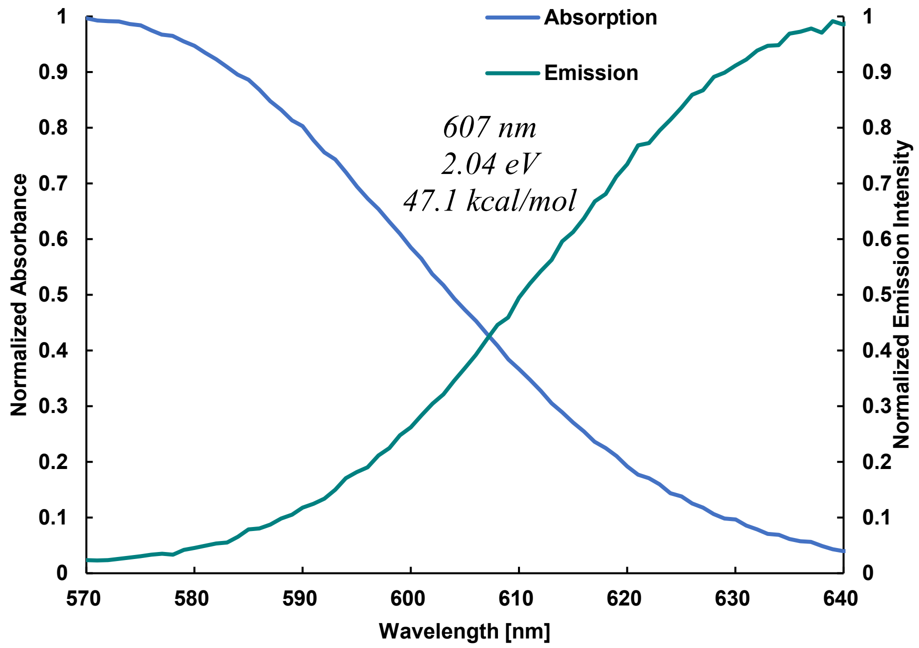

Figure S110. E_{0-0} estimation at normalized emission and absorption overlap of **PC13**.

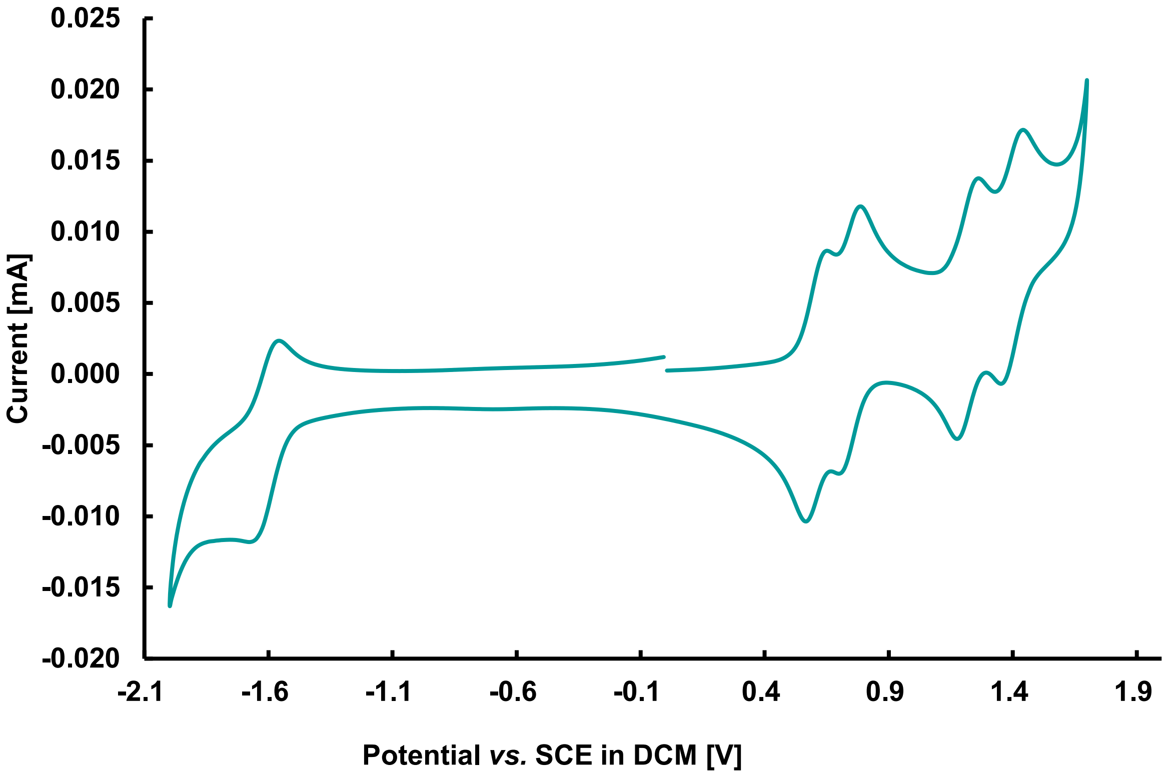

Figure S111. Cyclic voltammetry of **PC13** in degassed DCM (0.4 mM) using 0.1 M $^{n}Bu_4NPF_6$ as electrolyte.

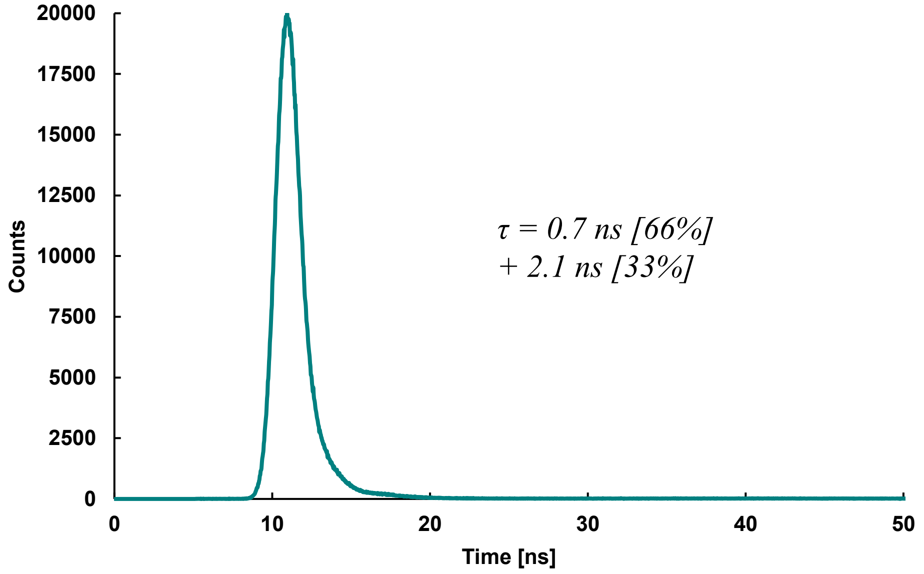

Figure S112. Fluorescence decay curve of **PC13** in degassed DCM (20 µM). Excitation wavelength – 394 nm.

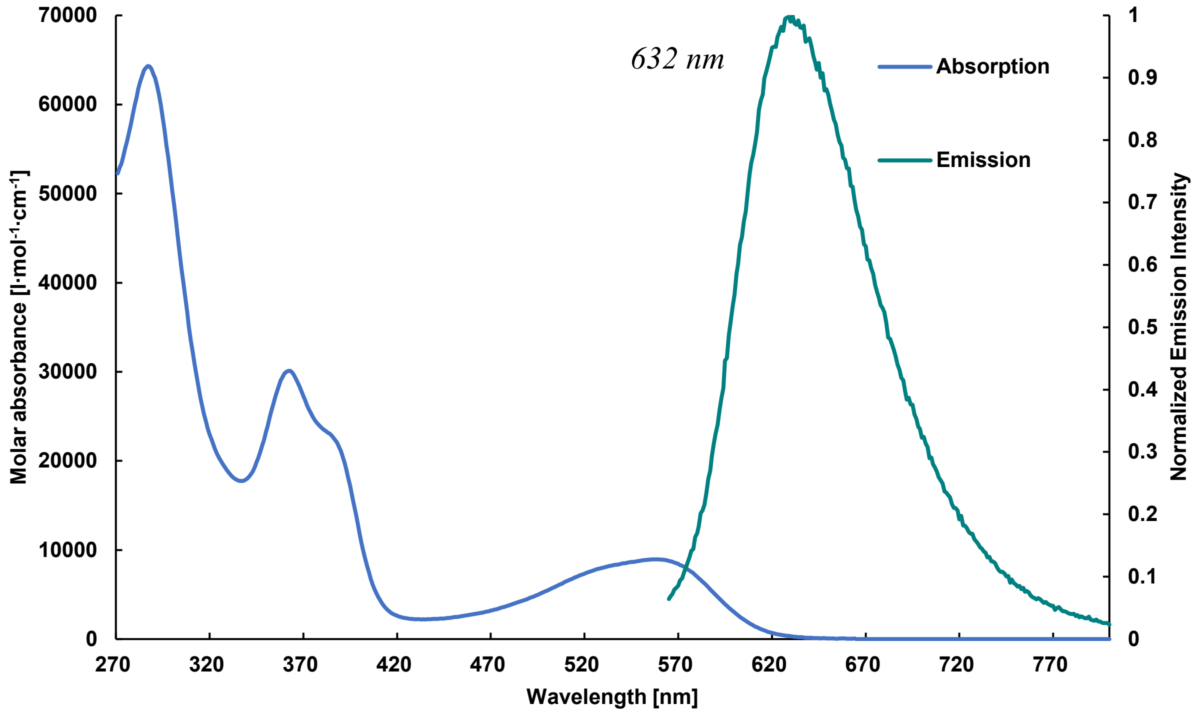

Figure S113. UV/Vis absorption and emission spectrum of **PC13’** in degassed DMA (20 μM). Excitation wavelength – 550 nm.

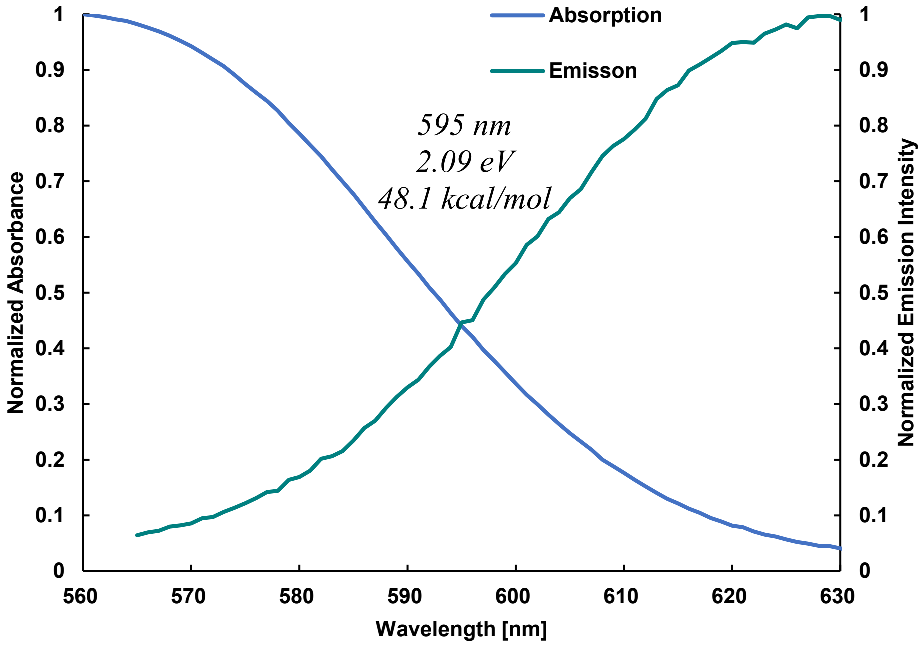

Figure S114. $E_{0\text{-}0}$ estimation at normalized emission and absorption overlap of **PC13’**.

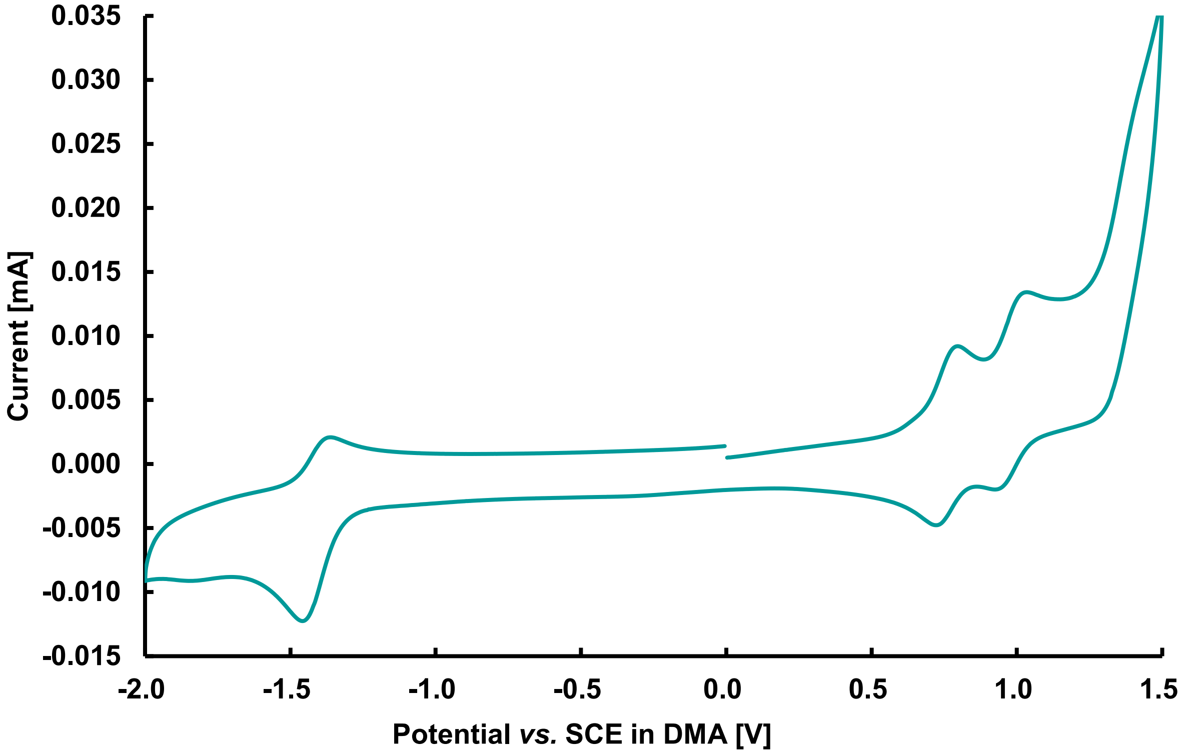

Figure S115. Cyclic voltammetry of **PC13'** in degassed DMA (0.4 mM) using 0.1 M $^{n}Bu_4NPF_6$ as electrolyte.

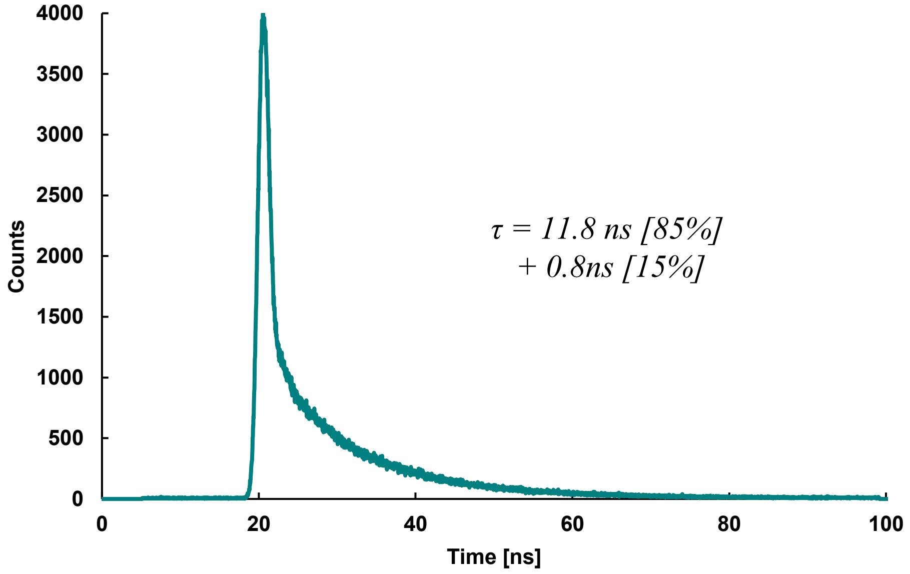

Figure S116. Fluorescence decay curve of **PC13'** in degassed DMA (20 µM). Excitation wavelength – 457 nm.

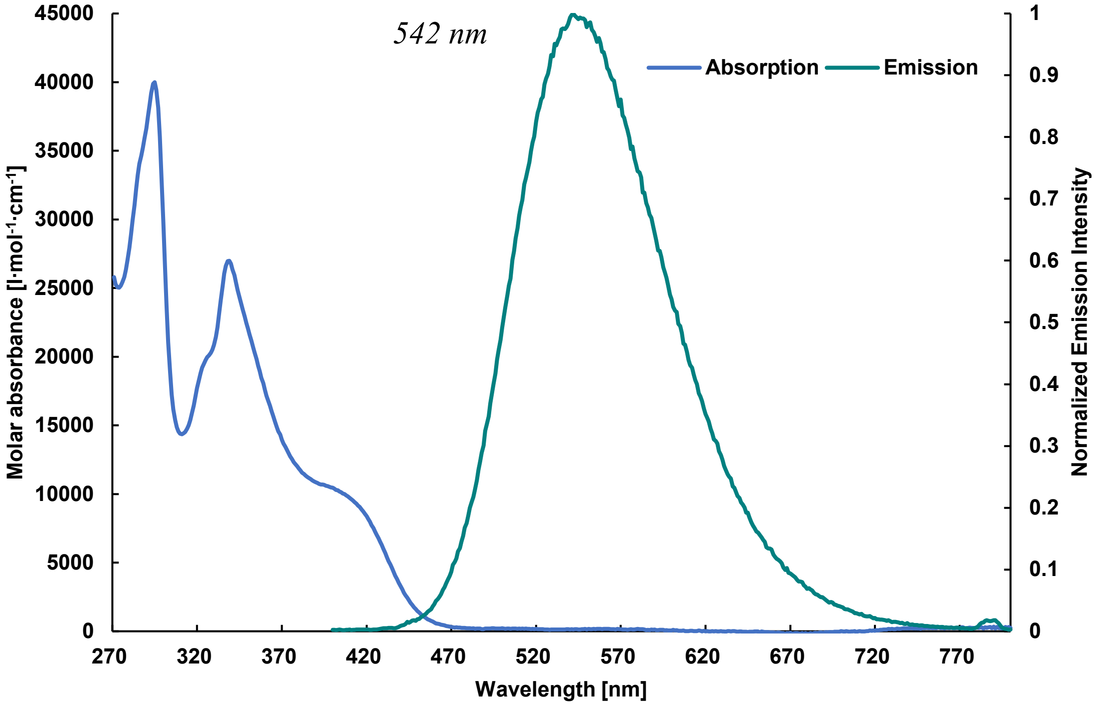

Figure S117. UV/Vis absorption and emission spectrum of **PC14** in degassed MeCN (20 µM). Excitation wavelength – 394 nm.

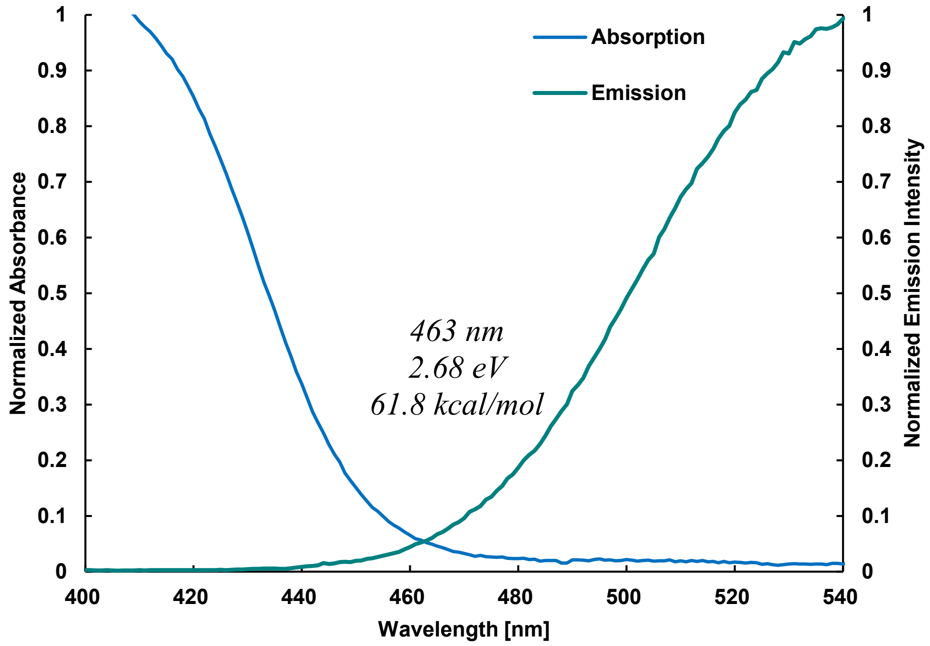

Figure S118. $E_{0\text{-}0}$ estimation at normalized emission and absorption overlap of **PC14**.

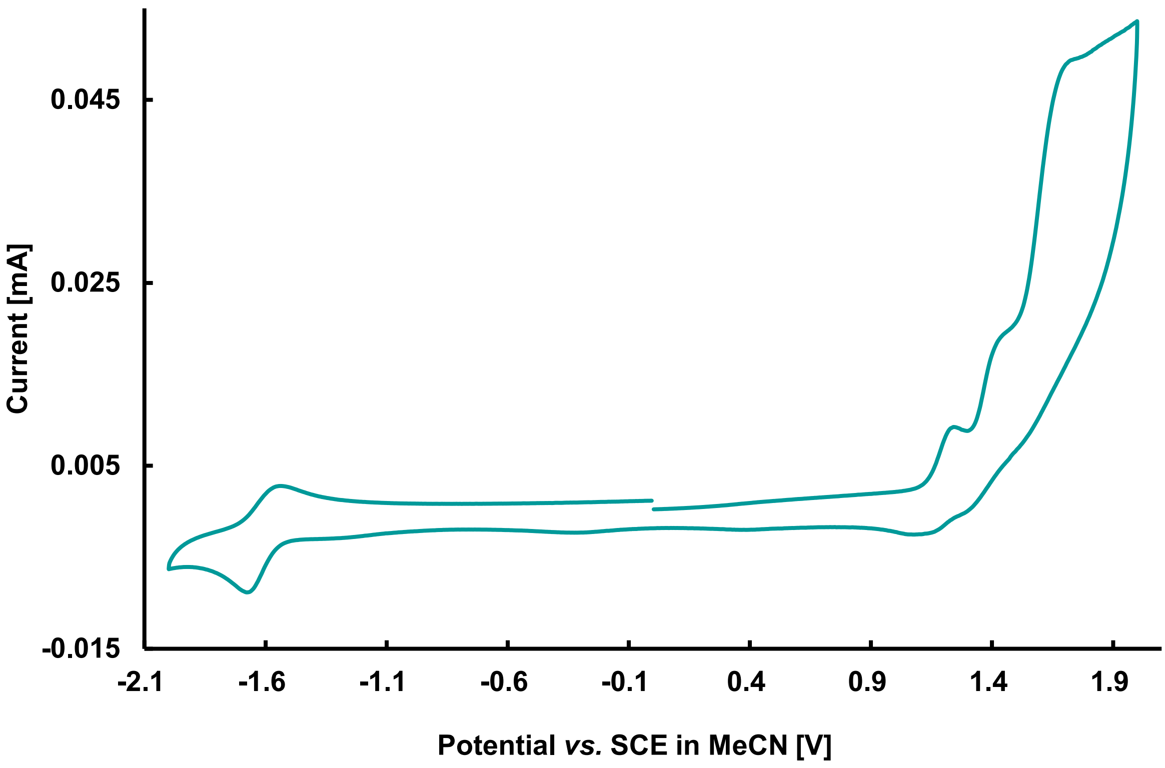

Figure S119. Cyclic voltammetry of **PC14** in degassed MeCN (0.4 mM) using 0.1 M $^{n}Bu_4NPF_6$ as electrolyte.

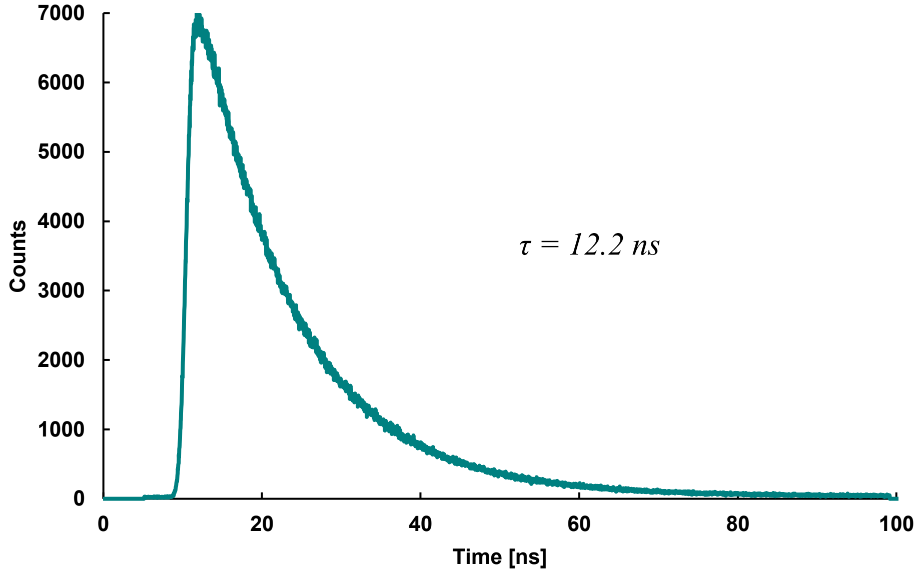

Figure S120. Fluorescence decay curve of **PC14** in degassed MeCN (20 μM). Excitation wavelength – 394 nm.

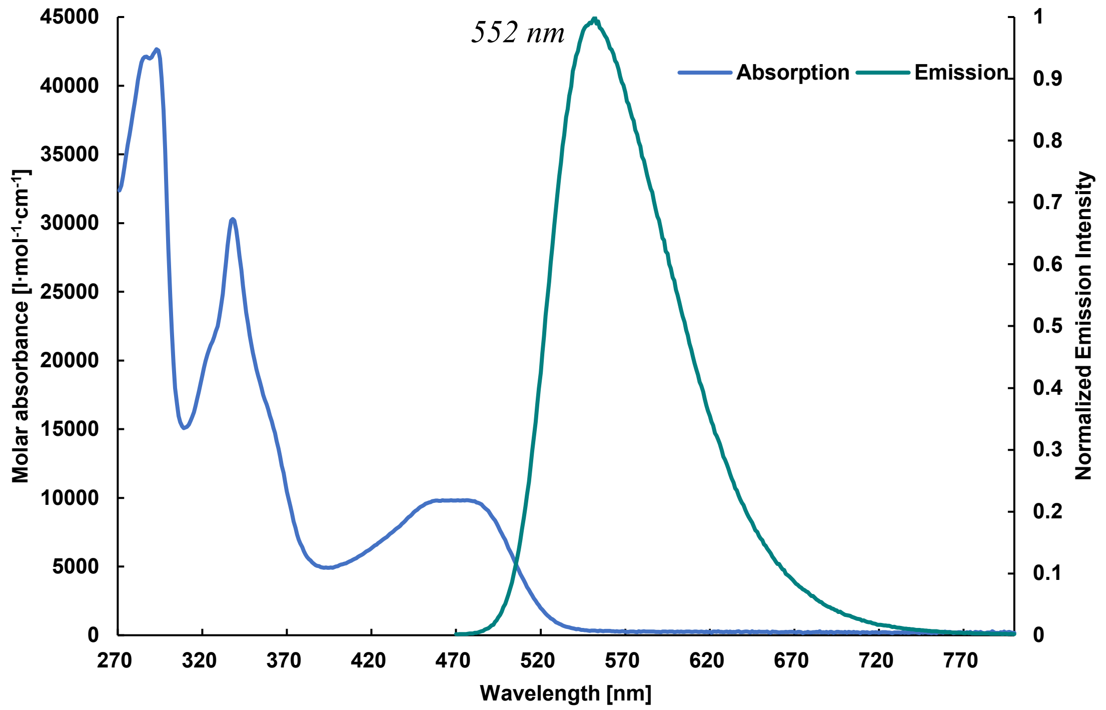

Figure S121. UV/Vis absorption and emission spectrum of **PC15** in degassed DCM (20 μM). Excitation wavelength – 456 nm.

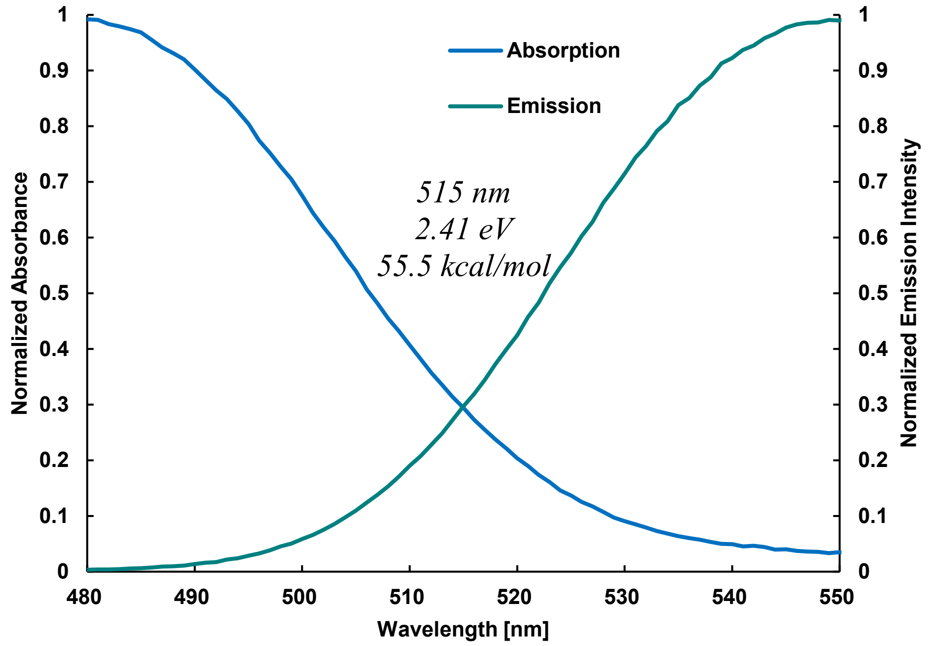

Figure S122. $E_{0\text{-}0}$ estimation at normalized emission and absorption overlap of **PC15**.

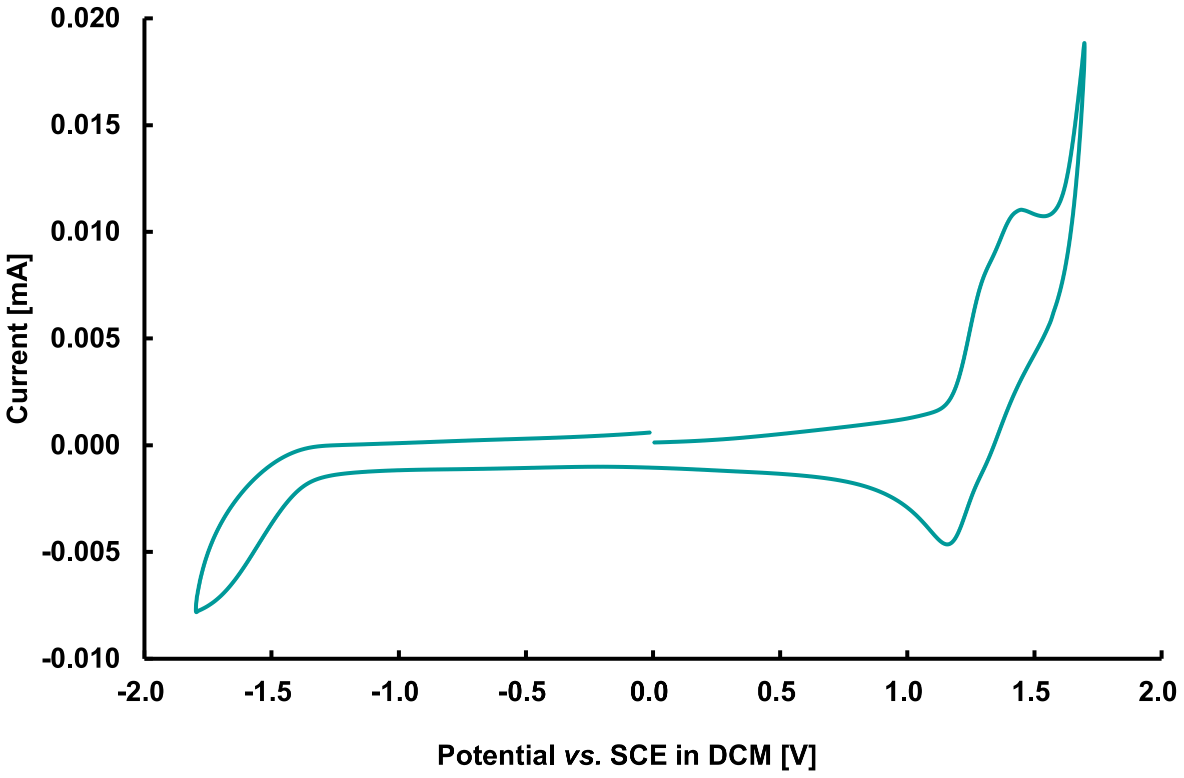

Figure S123. Cyclic voltammetry of **PC15** in degassed DCM (0.4 mM) using 0.1 M $^{n}Bu_4NPF_6$ as electrolyte.

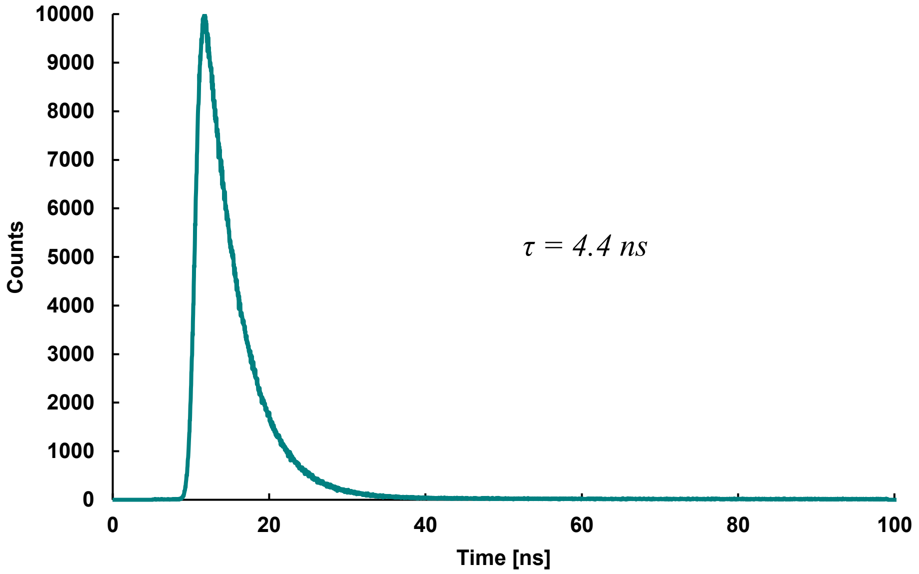

Figure S124. Fluorescence decay curve of **PC15** in degassed DCM (20 μM). Excitation wavelength – 394 nm.

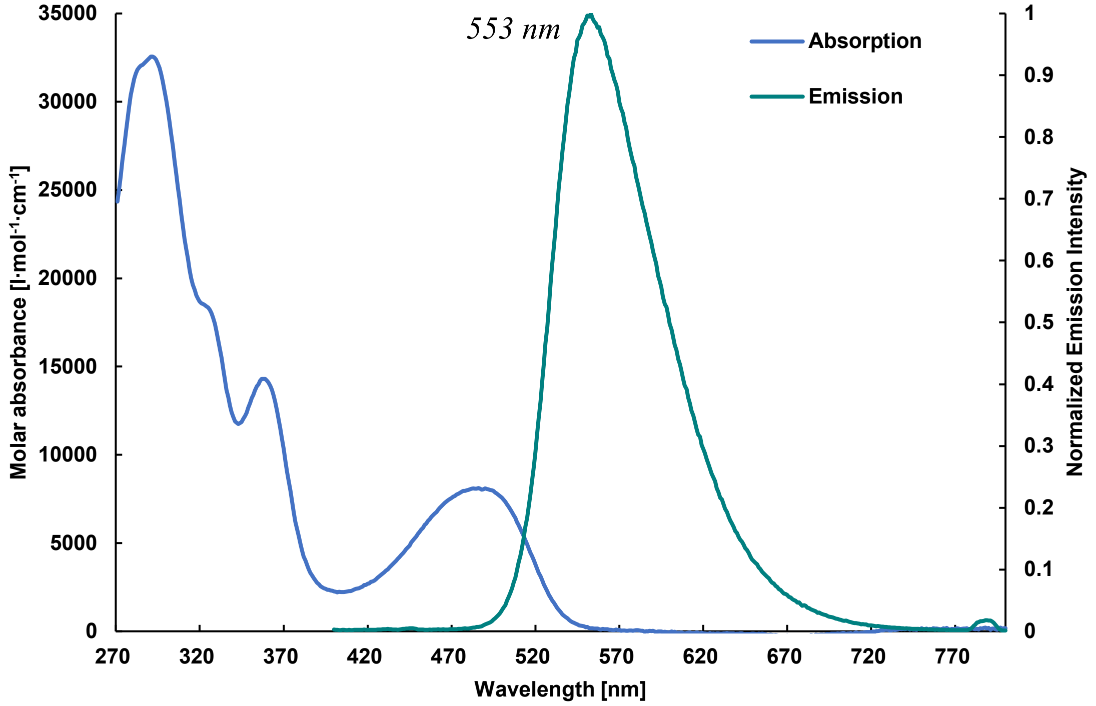

Figure S125. UV/Vis absorption and emission spectrum of **PC16** in degassed MeCN (20 µM). Excitation wavelength – 394 nm.

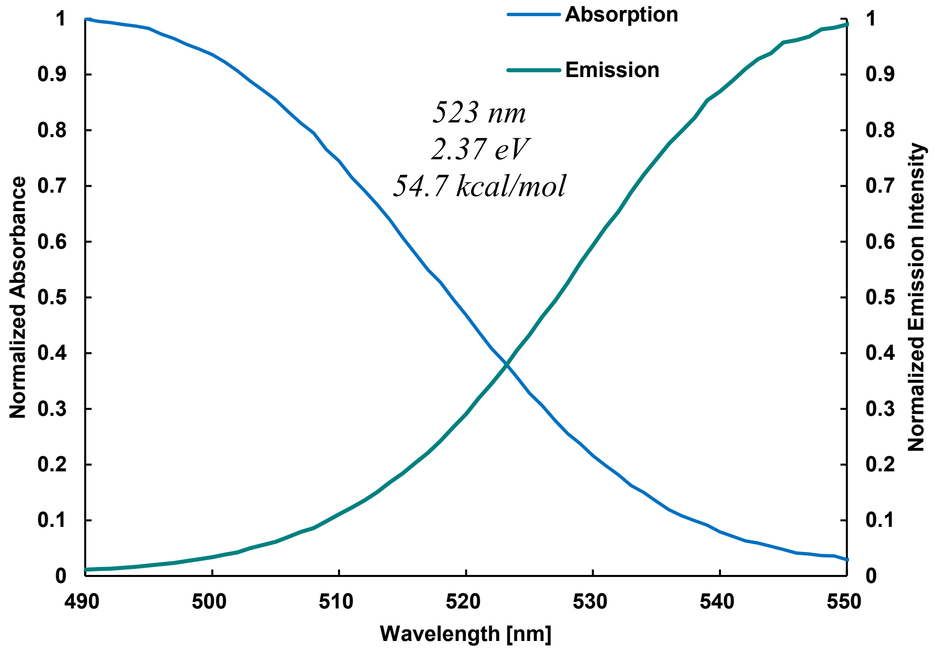

Figure S126. $E_{0\text{-}0}$ estimation at normalized emission and absorption overlap of **PC16**.

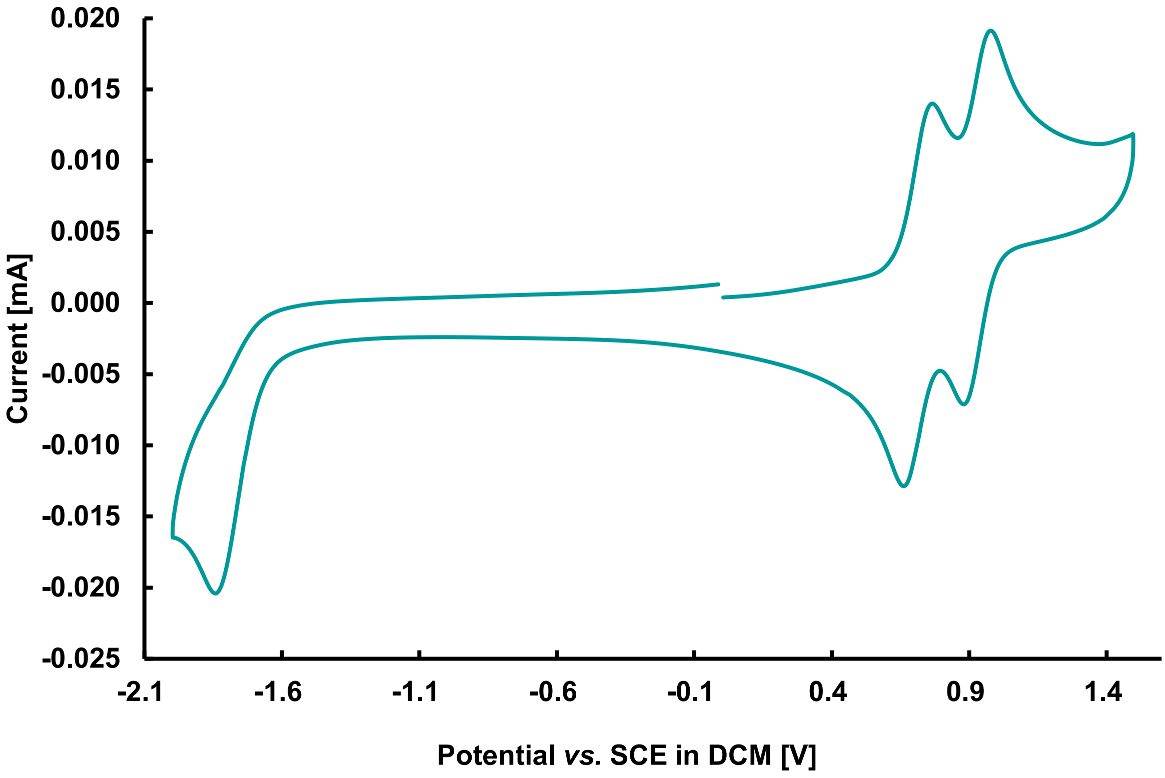

Figure S127. Cyclic voltammetry of **PC16** in degassed MeCN (0.4 mM) using 0.1 M $^{n}Bu_4NPF_6$ as electrolyte.

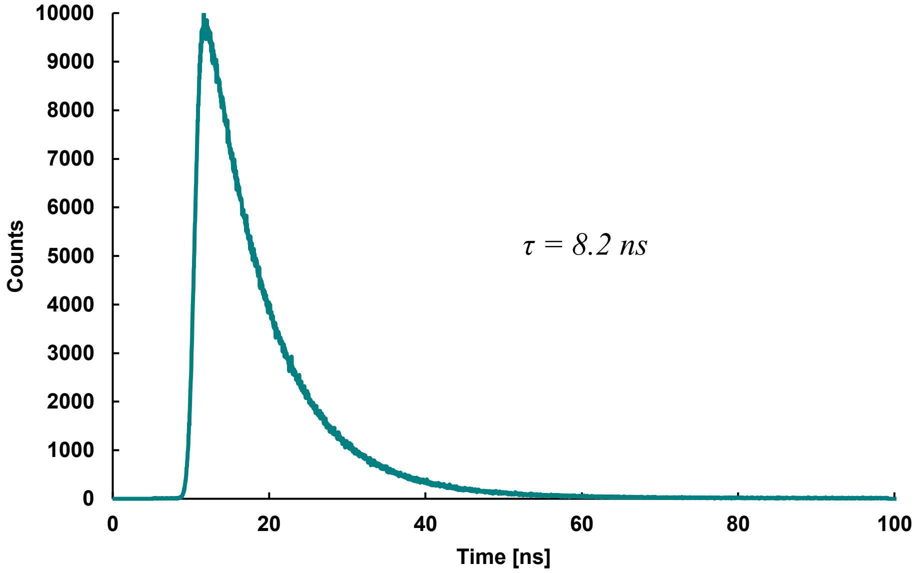

Figure S128. Fluorescence decay curve of **PC16** in degassed MeCN (20 µM). Excitation wavelength – 394 nm.

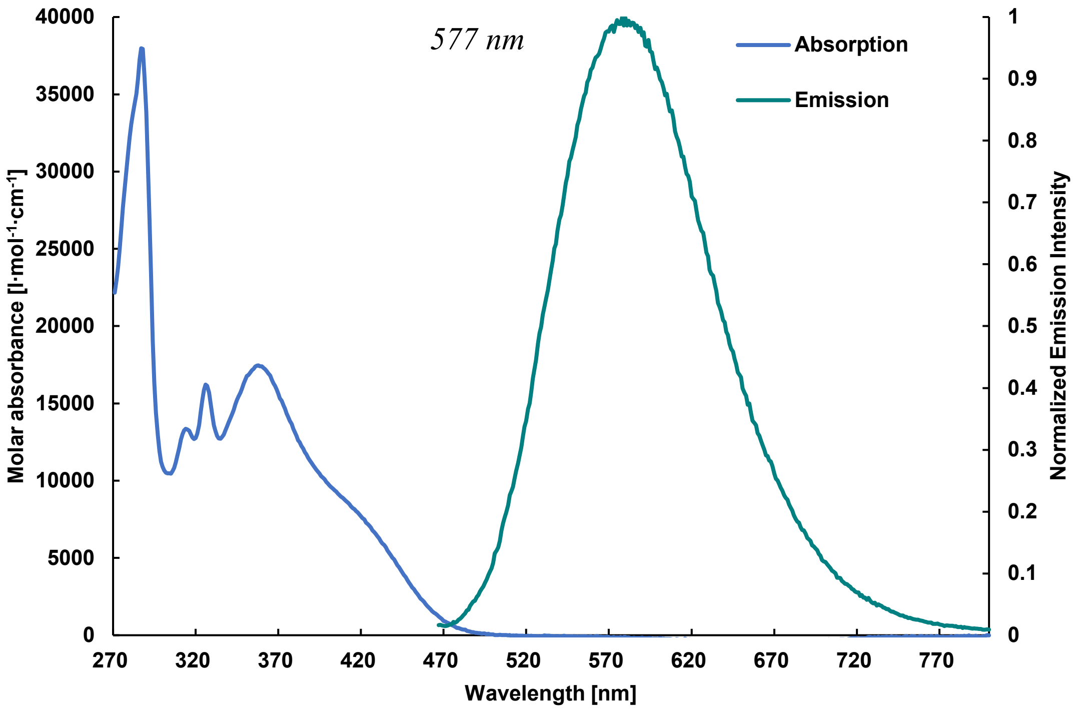

Figure S129. UV/Vis absorption and emission spectrum of **PC17** in degassed MeCN (20 μM). Excitation wavelength – 456 nm.

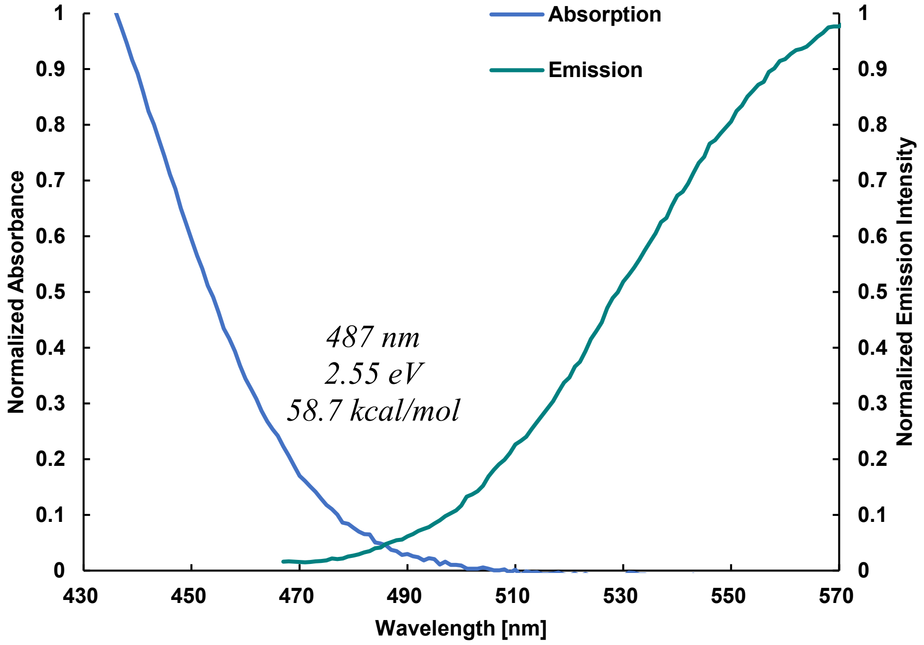

Figure S130. $E_{0\text{-}0}$ estimation at normalized emission and absorption overlap of **PC17**.

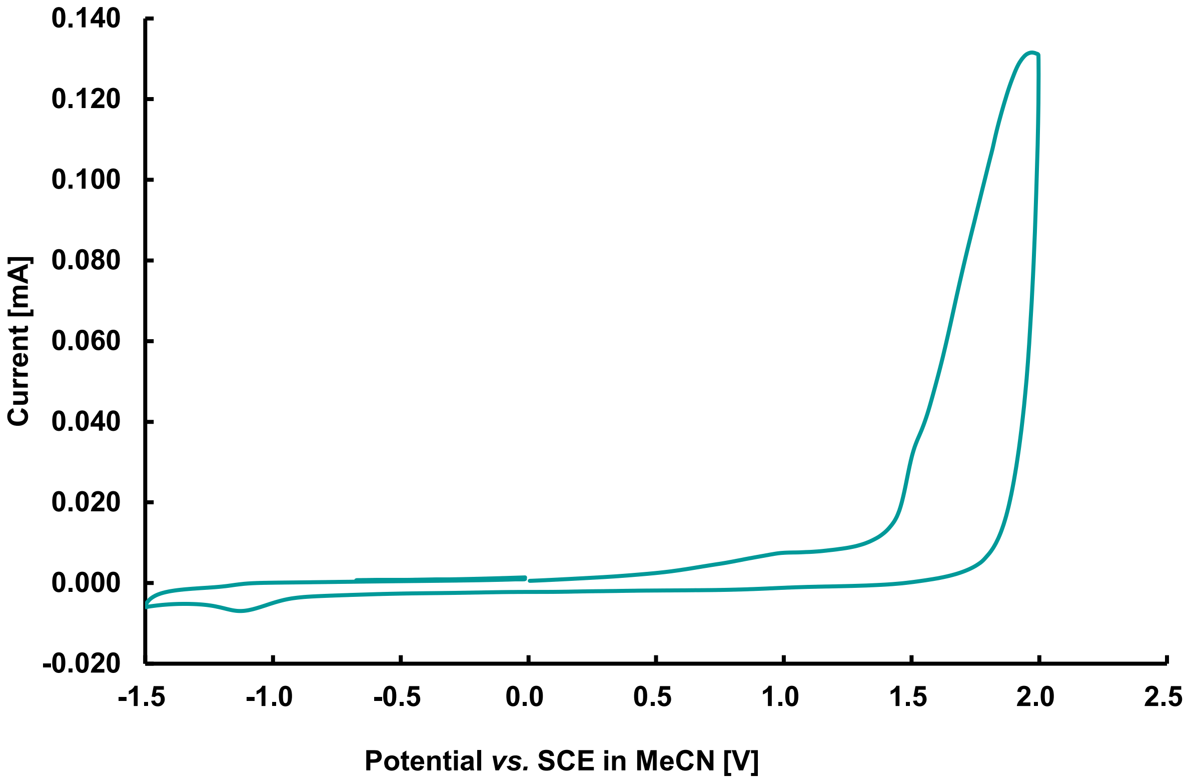

Figure S131. Cyclic voltammetry of **PC17** in degassed MeCN (0.4 mM) using 0.1 M $^{n}Bu_4NPF_6$ as electrolyte.

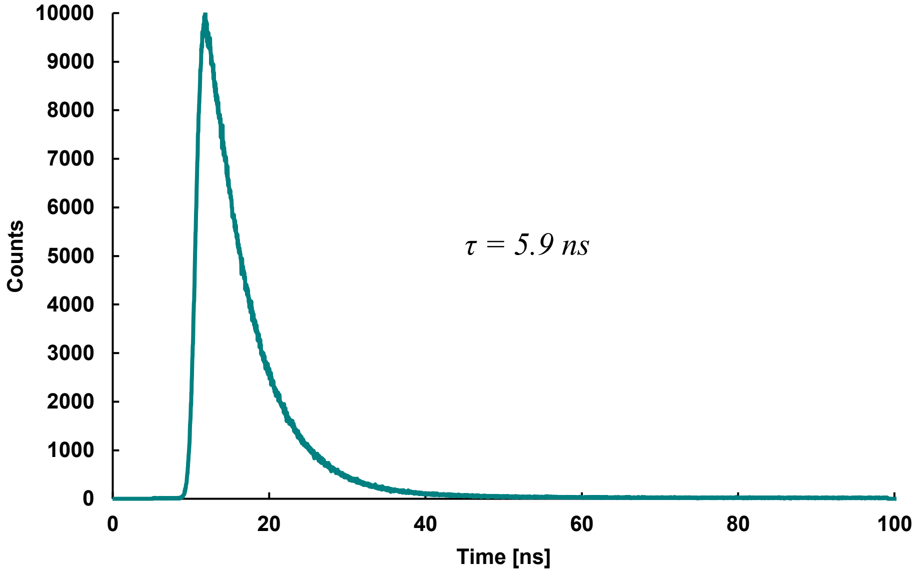

Figure S132. Fluorescence decay curve of **PC17** in degassed MeCN (20 µM). Excitation wavelength – 394 nm.

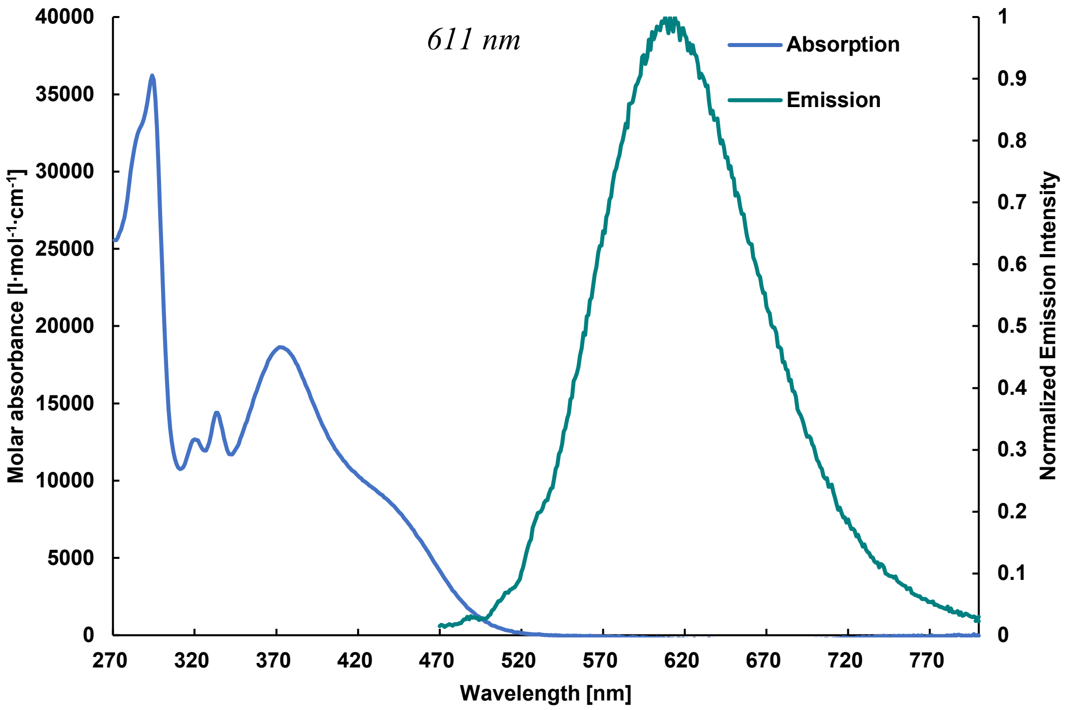

Figure S133. UV/Vis absorption and emission spectrum of **PC18** in degassed MeCN (20 μM). Excitation wavelength – 456 nm.

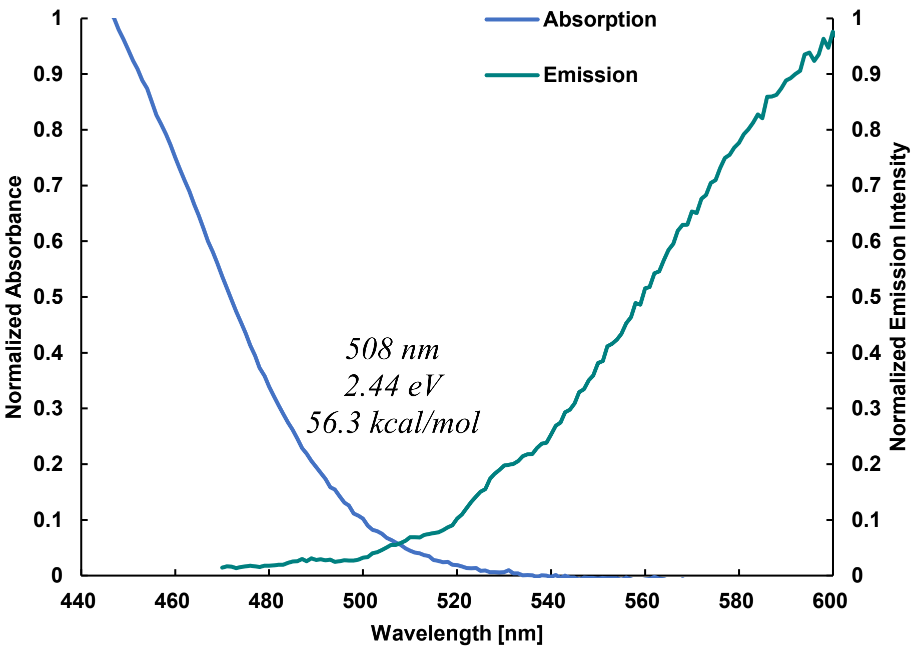

Figure S134. $E_{0\text{-}0}$ estimation at normalized emission and absorption overlap of **PC18**.

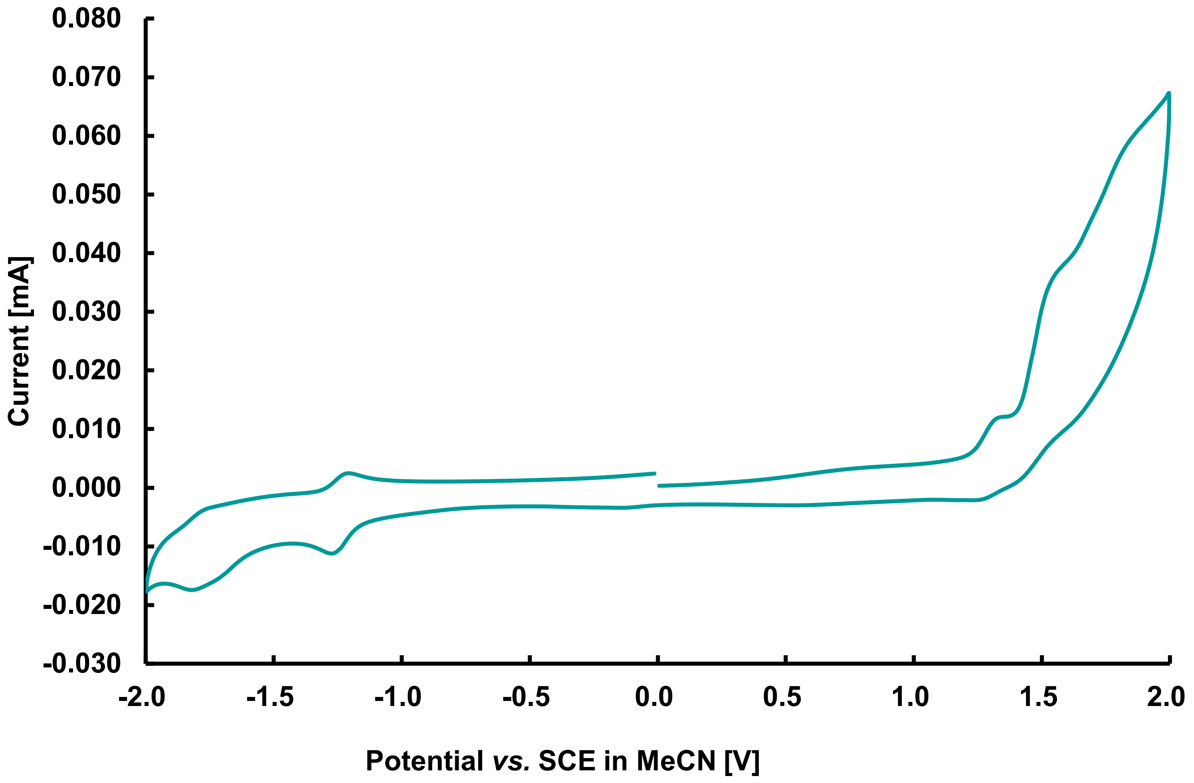

Figure S135. Cyclic voltammetry of **PC18** in degassed MeCN (0.4 mM) using 0.1 M $^{n}Bu_4NPF_6$ as electrolyte.

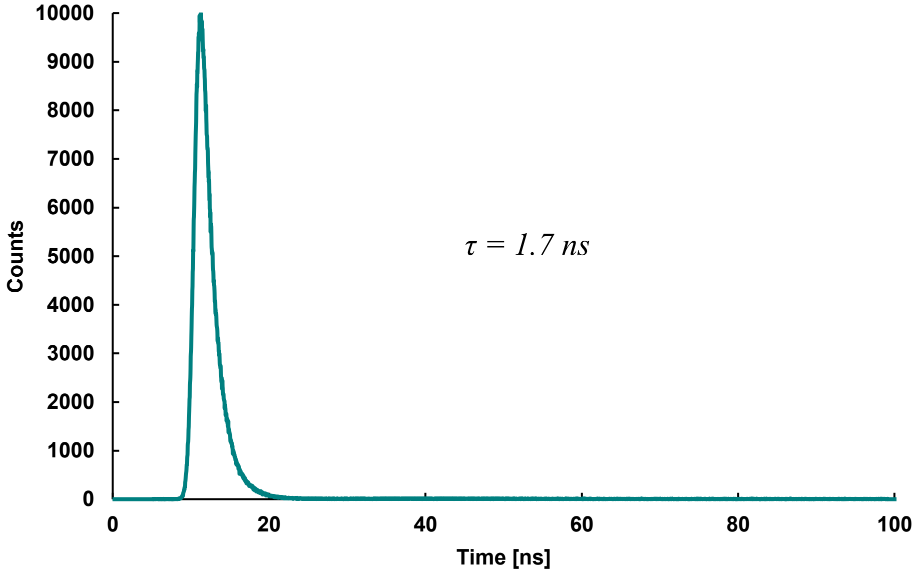

Figure S136. Fluorescence decay curve of **PC18** in degassed MeCN (20 µM). Excitation wavelength – 394 nm.

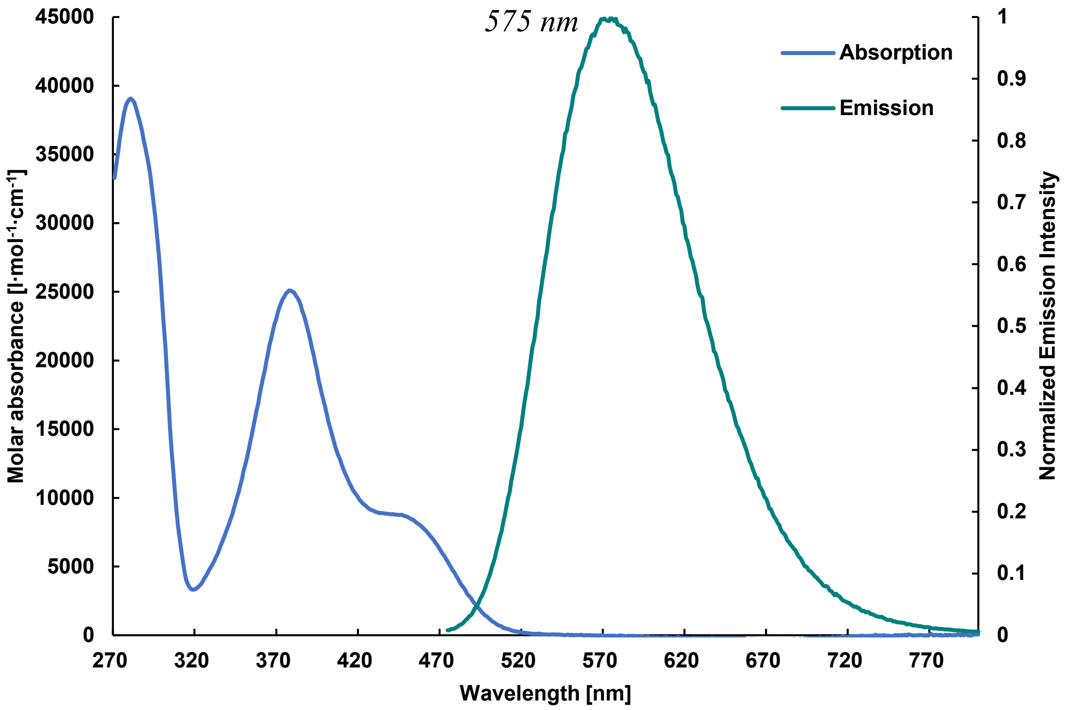

Figure S137. UV/Vis absorption and emission spectrum of **PC19** in degassed MeCN (20 µM). Excitation wavelength – 456 nm.

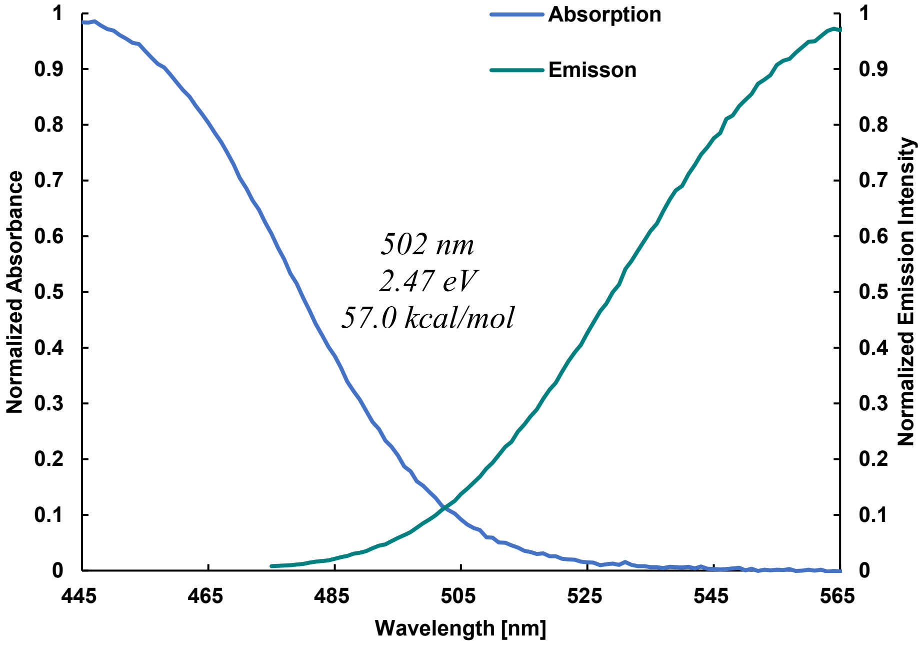

Figure S138. $E_{0\text{-}0}$ estimation at normalized emission and absorption overlap of **PC19**.

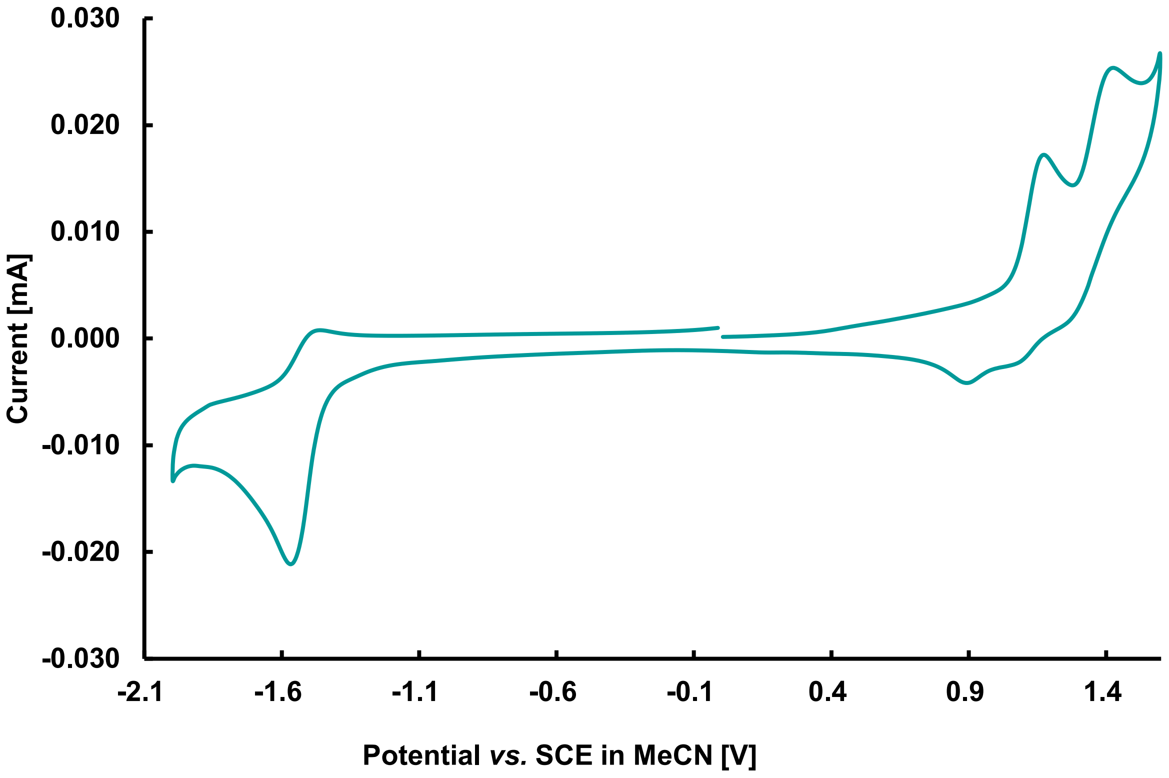

Figure S139. Cyclic voltammetry of **PC19** in degassed MeCN (0.4 mM) using 0.1 M $^{n}Bu_4NPF_6$ as electrolyte.

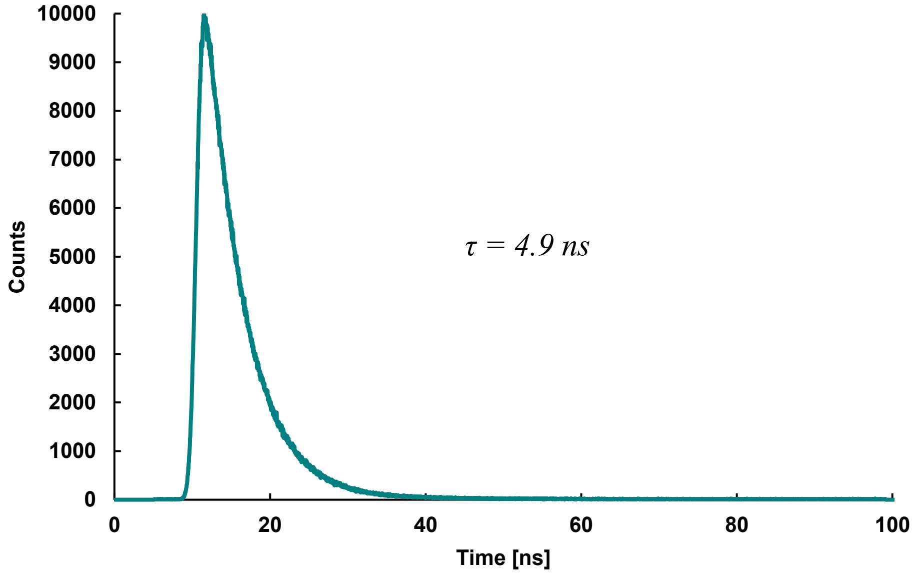

Figure S140. Fluorescence decay curve of **PC19** in degassed MeCN (20 μM). Excitation wavelength – 394 nm.

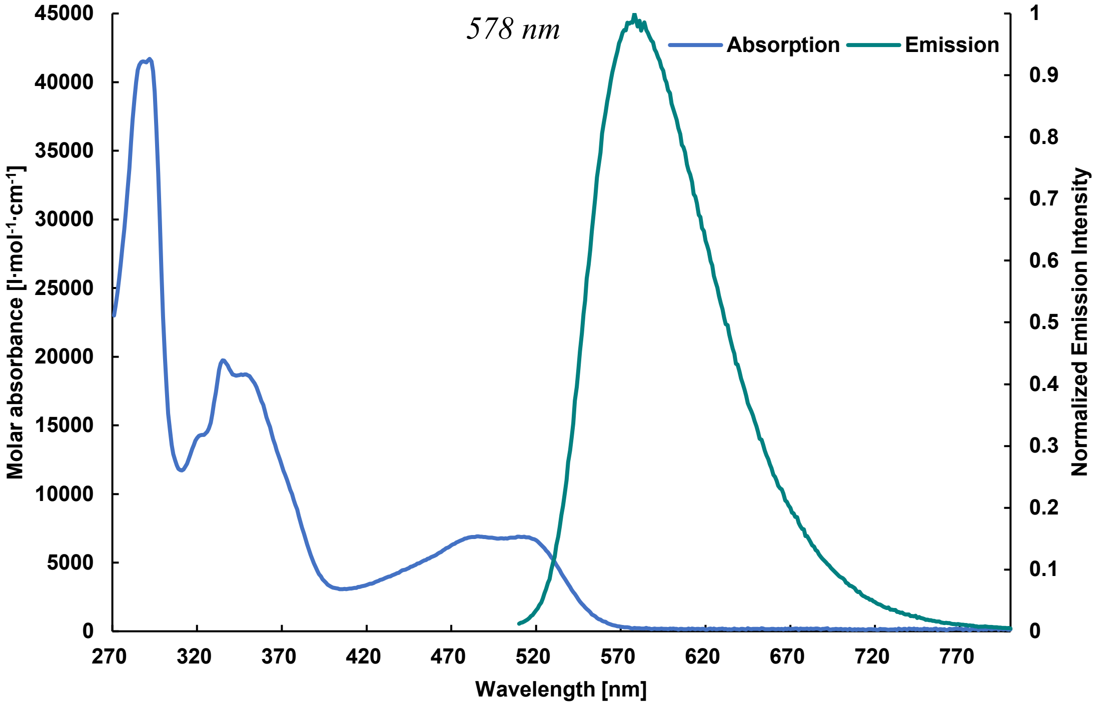

Figure S141. UV/Vis absorption and emission spectrum of **PC20** in degassed DCM (20 μM). Excitation wavelength – 500 nm.

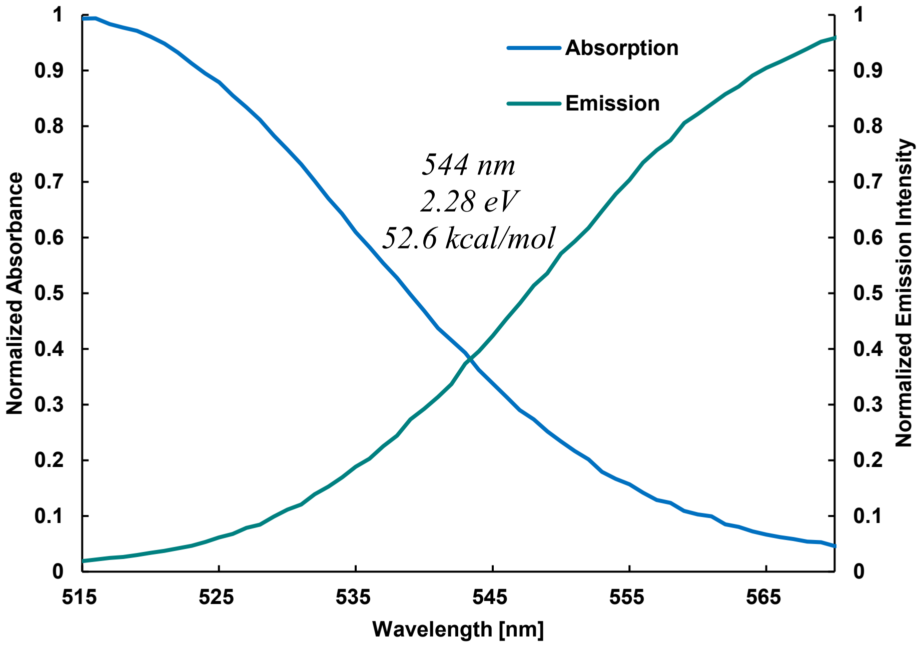

Figure S142. $E_{0\text{-}0}$ estimation at normalized emission and absorption overlap of **PC20**.

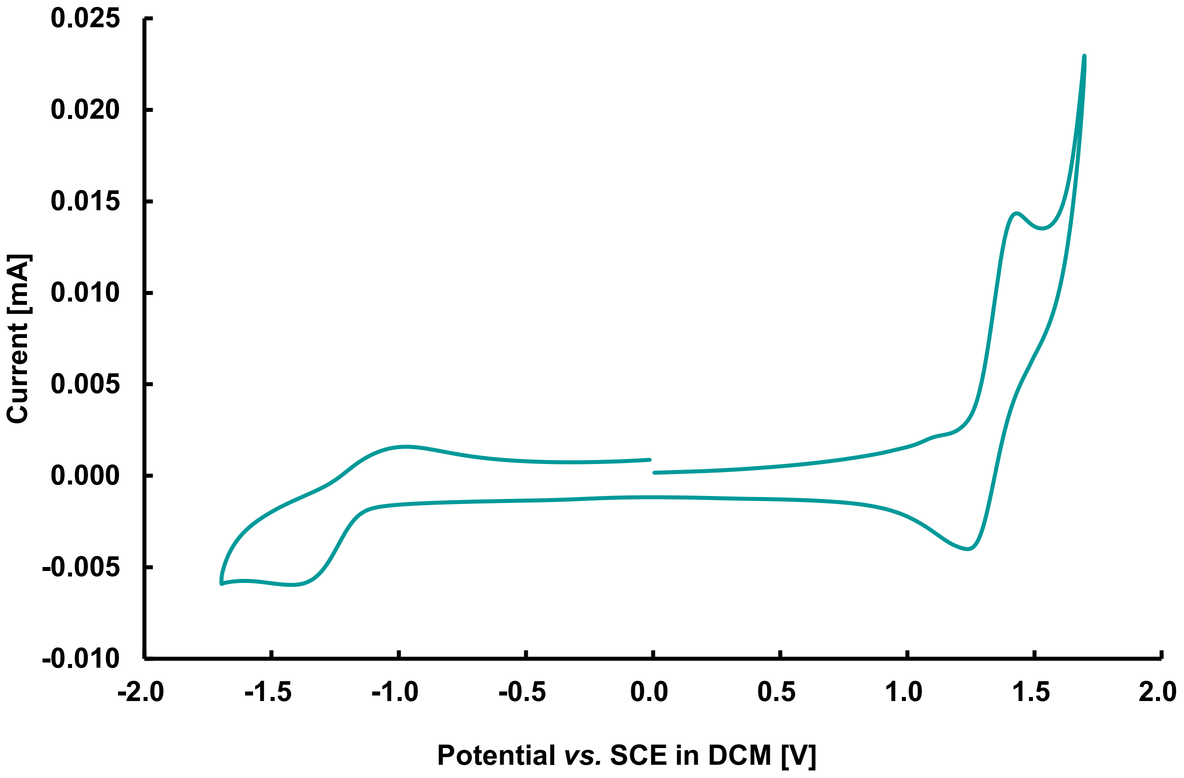

Figure S143. Cyclic voltammetry of **PC20** in degassed DCM (0.4 mM) using 0.1 M $^{n}Bu_4NPF_6$ as electrolyte.

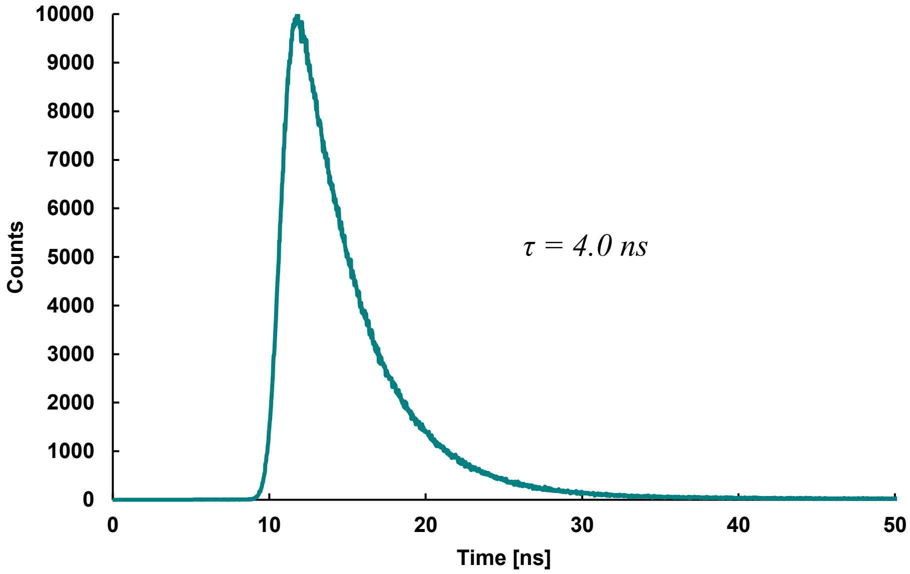

Figure S144. Fluorescence decay curve of **PC20** in degassed DCM (20 µM). Excitation wavelength – 456 nm.

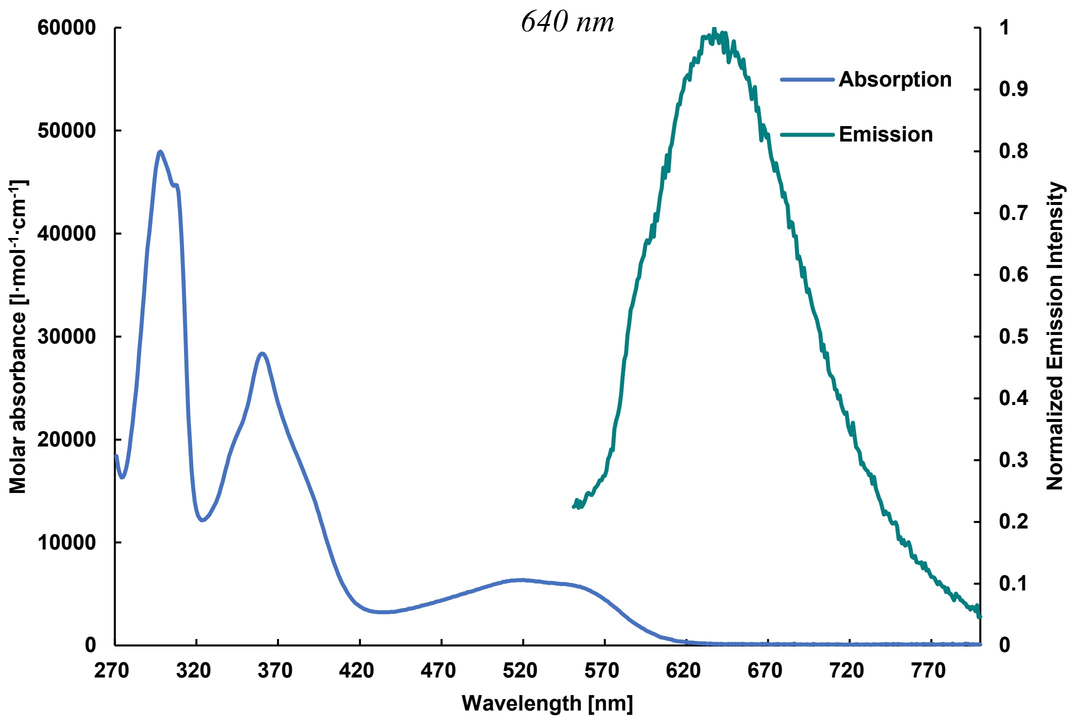

Figure S145. UV/Vis absorption and emission spectrum of **PC21** in degassed DCM (20 µM). Excitation wavelength – 500 nm.

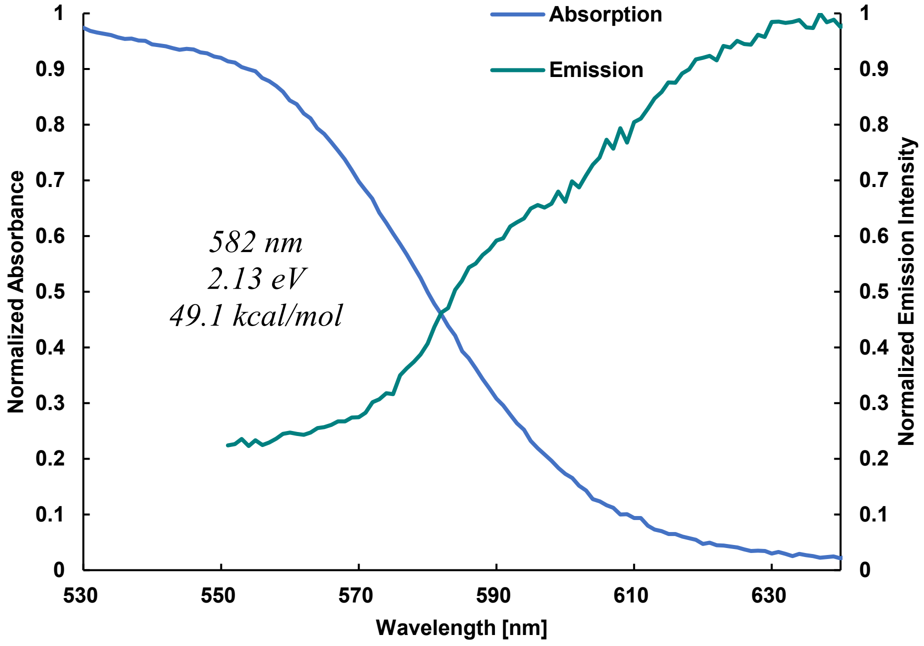

Figure S146. $E_{0\text{-}0}$ estimation at normalized emission and absorption overlap of **PC21**.

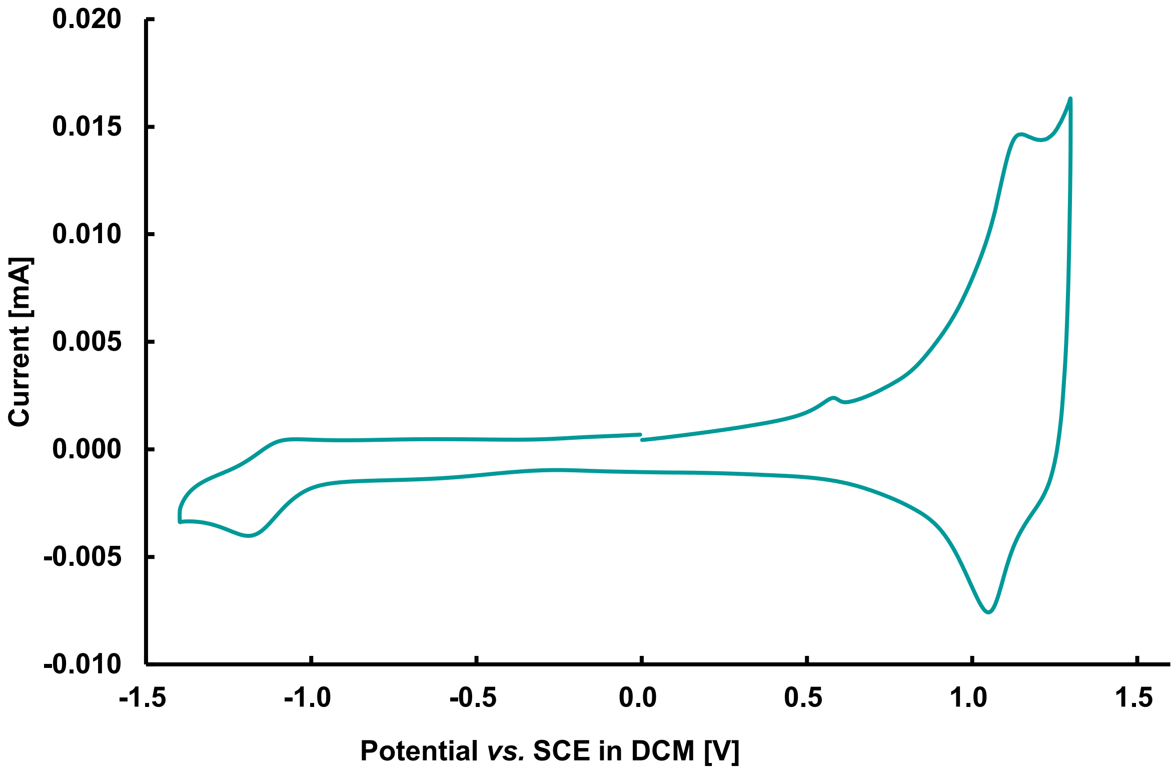

Figure S147. Cyclic voltammetry of **PC21** in degassed DCM (0.4 mM) using 0.1 M ${}^{n}Bu_4NPF_6$ as electrolyte.

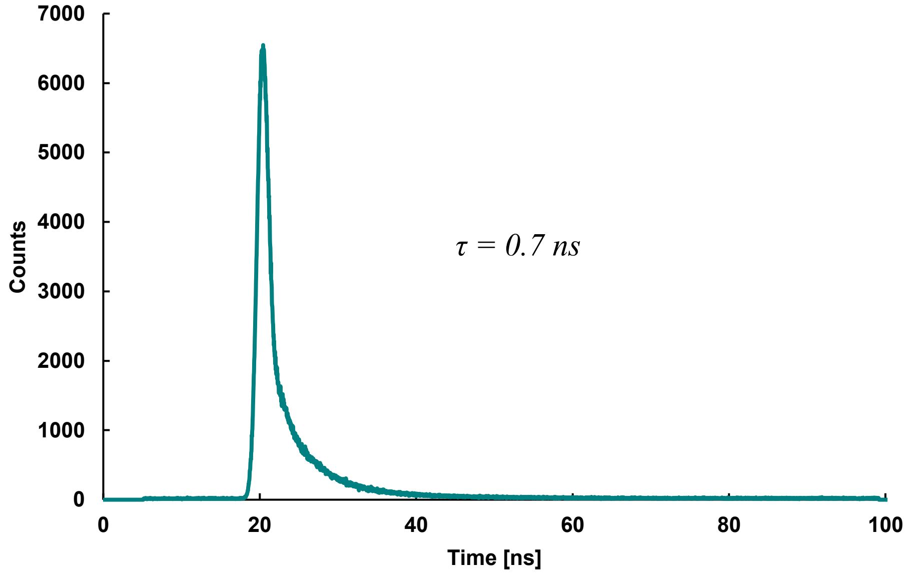

Figure S148. Fluorescence decay curve of **PC21** in degassed DCM (20 μM). Excitation wavelength – 456 nm.

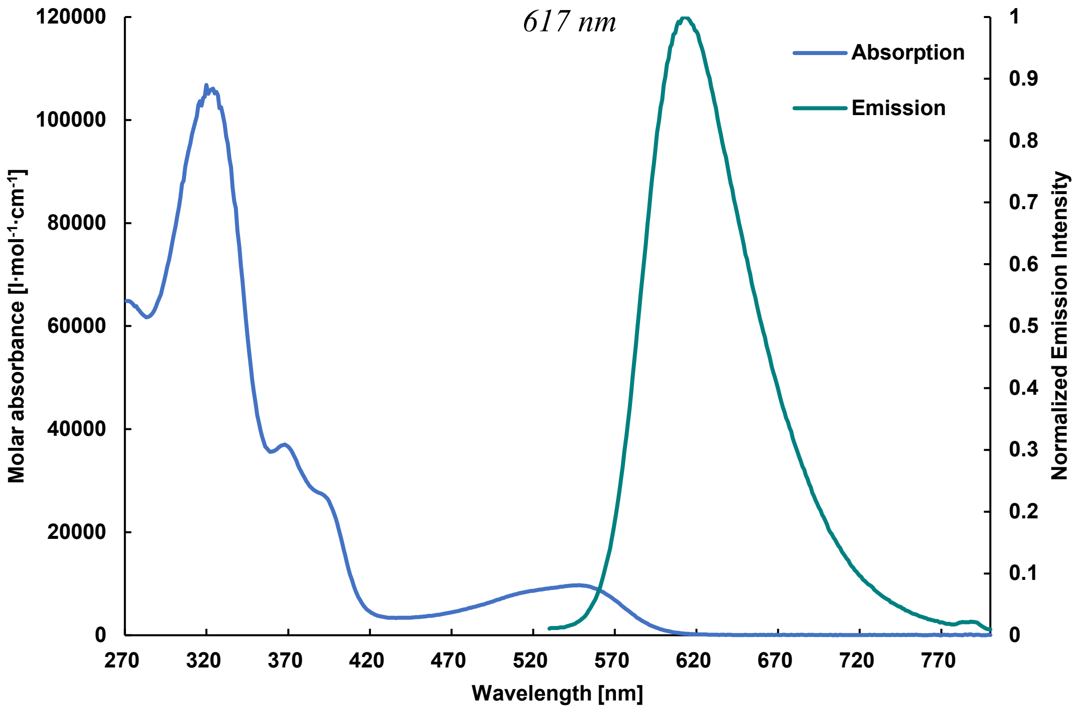

Figure S149. UV/Vis absorption and emission spectrum of **PC22** in degassed DCM (20 µM). Excitation wavelength – 500 nm.

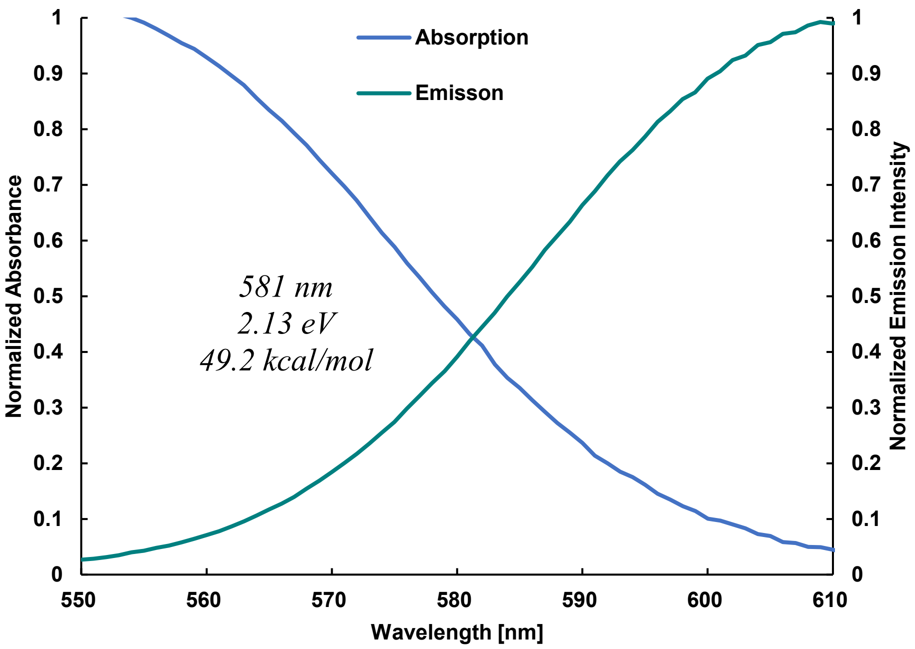

Figure S150. $E_{0\text{-}0}$ estimation at normalized emission and absorption overlap of **PC22**.

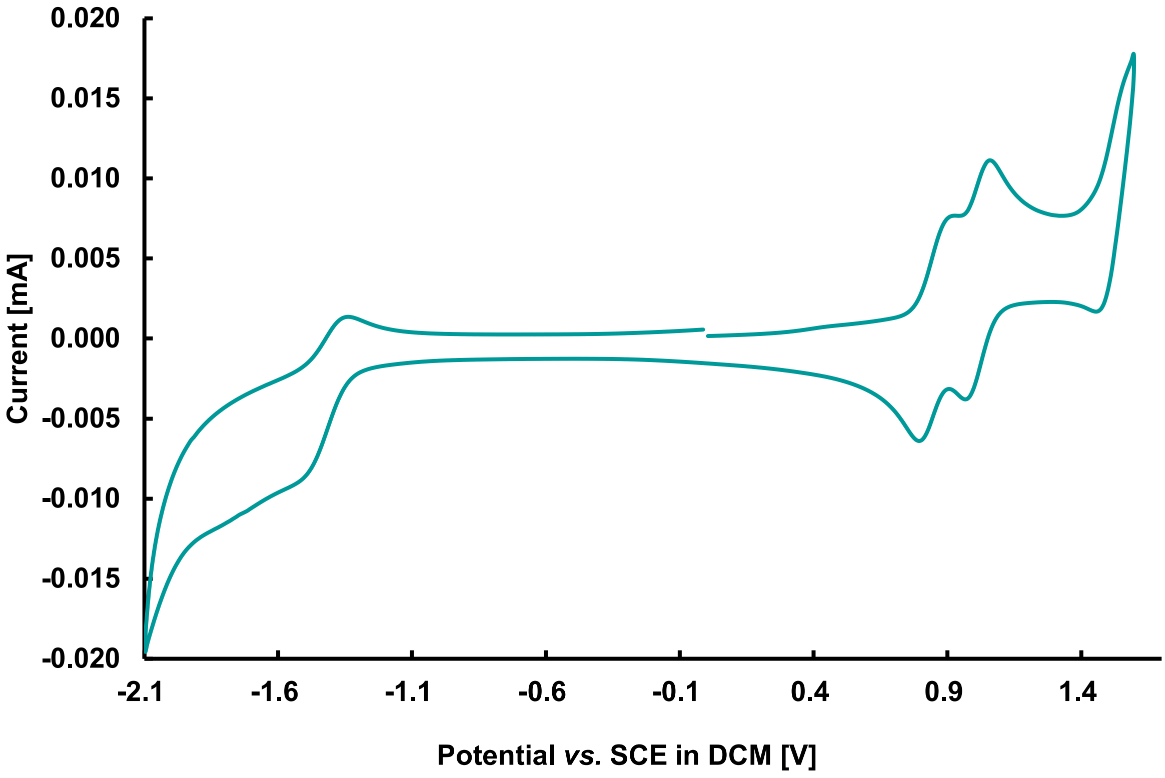

Figure S151. Cyclic voltammetry of **PC22** in degassed DCM (0.4 mM) using 0.1 M $^{n}Bu_4NPF_6$ as electrolyte.

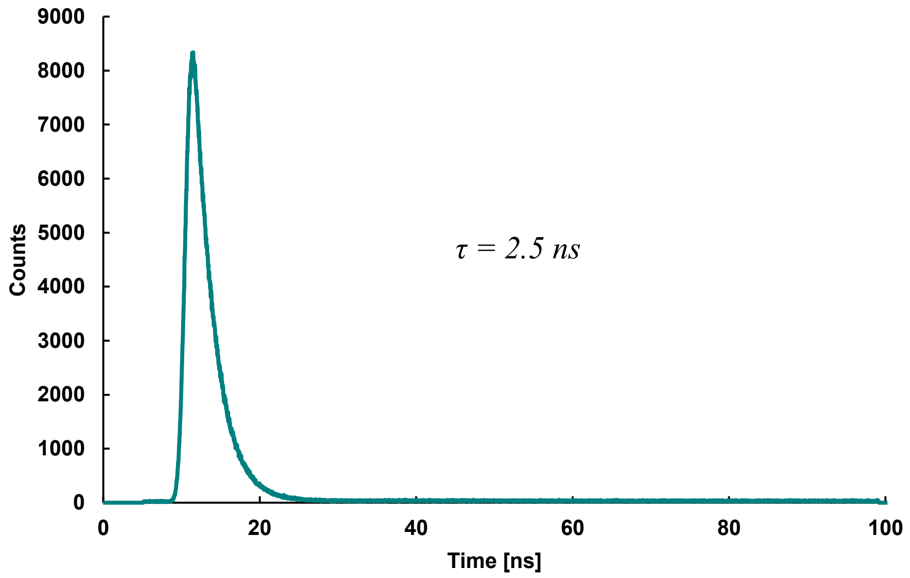

Figure S152. Fluorescence decay curve of **PC22** in degassed DCM (20 μM). Excitation wavelength – 394 nm.

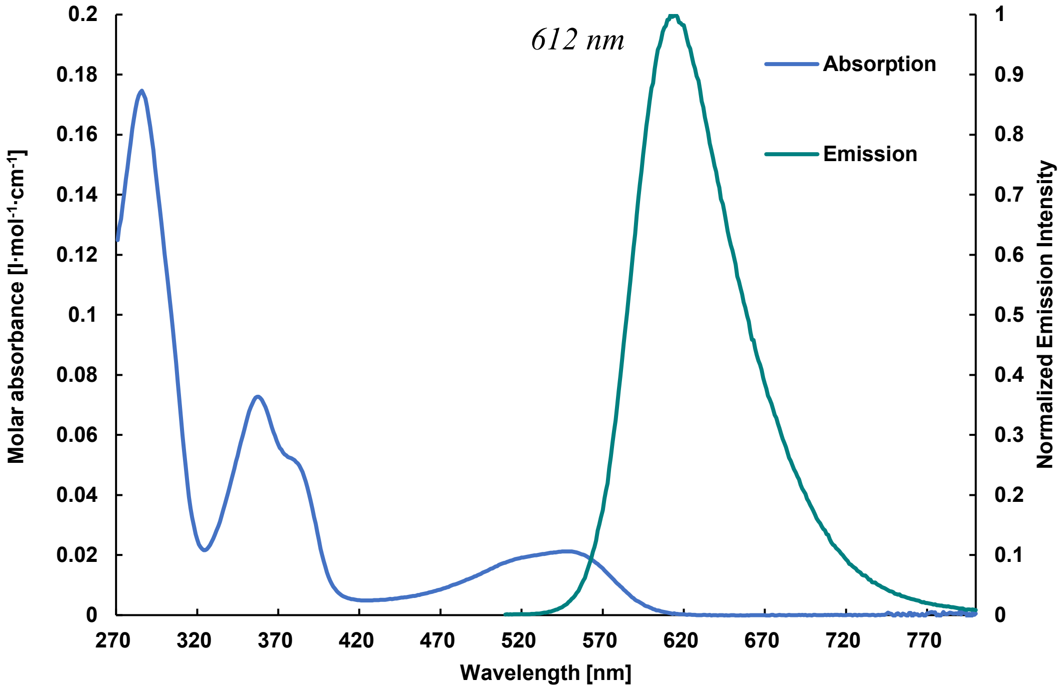

Figure S153. UV/Vis absorption and emission spectrum of **PC23** in degassed DCM (20 µM). Excitation wavelength – 456 nm.

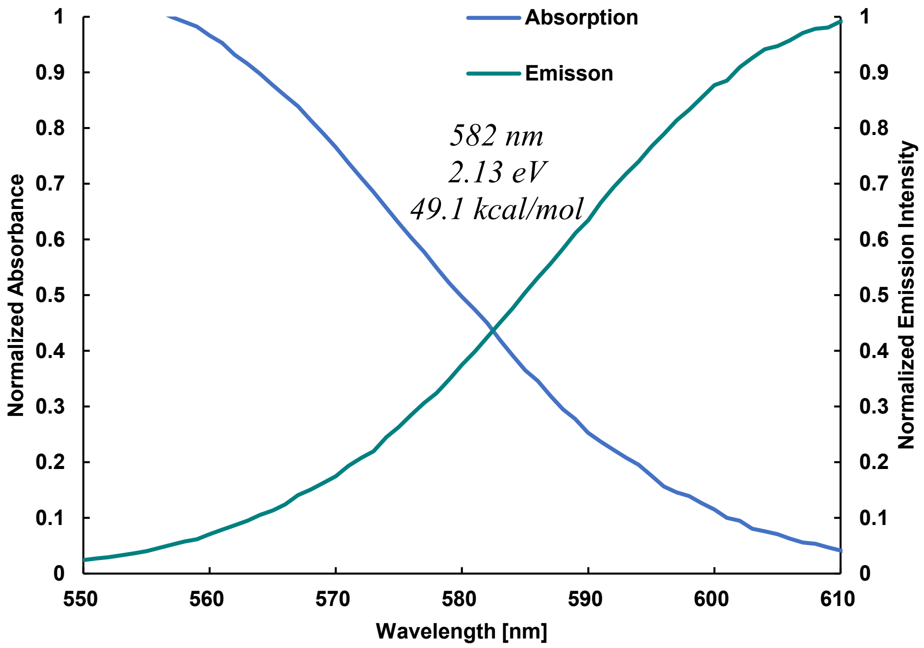

Figure S154. $E_{0\text{-}0}$ estimation at normalized emission and absorption overlap of **PC23**.

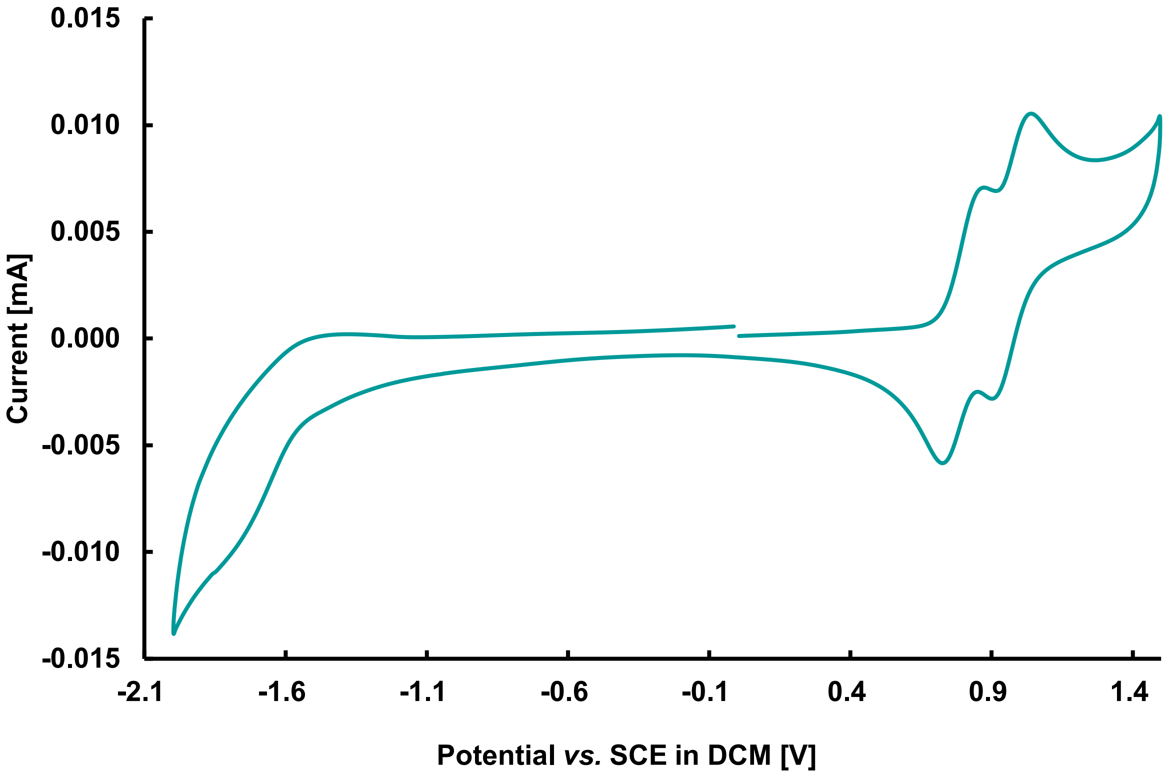

Figure S155. Cyclic voltammetry of **PC23** in degassed DCM (0.4 mM) using 0.1 M $^{n}Bu_4NPF_6$ as electrolyte.

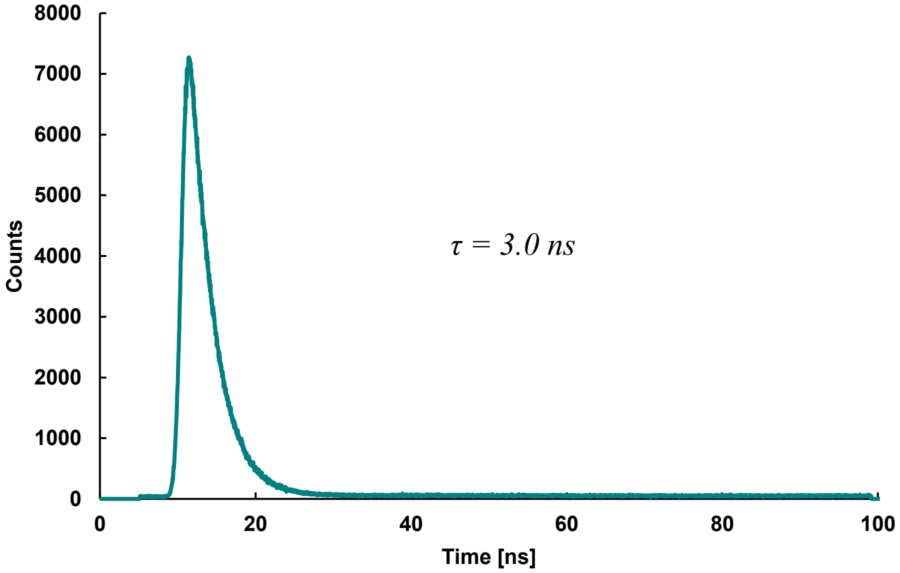

Figure S156. Fluorescence decay curve of **PC23** in degassed DCM (20 μM). Excitation wavelength – 394 nm.

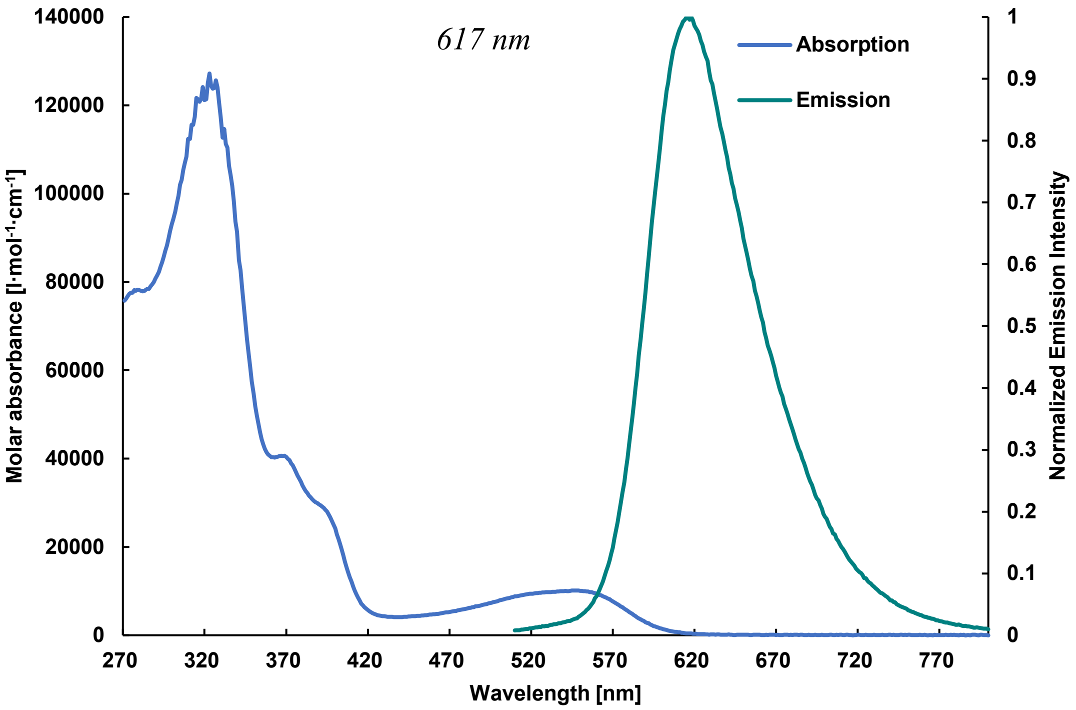

Figure S157. UV/Vis absorption and emission spectrum of **PC24** in degassed DCM (20 µM). Excitation wavelength – 457 nm.

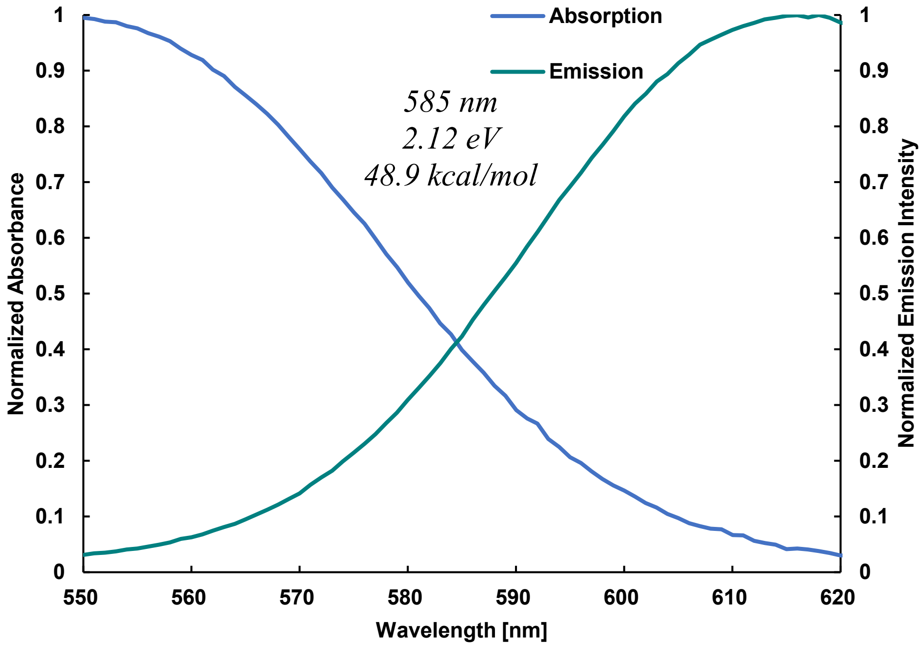

Figure S158. $E_{0\text{-}0}$ estimation at normalized emission and absorption overlap of **PC24**.

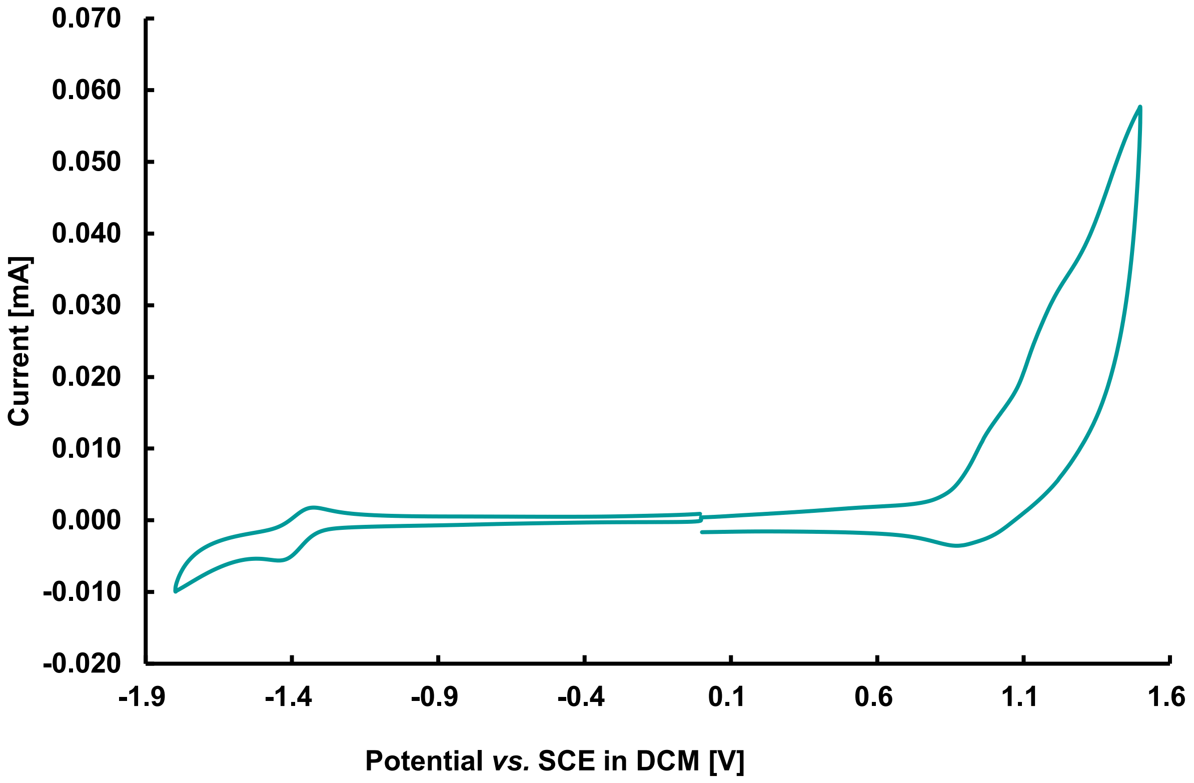

Figure S159. Cyclic voltammetry of **PC24** in degassed DCM (0.4 mM) using 0.1 M $^{n}Bu_4NPF_6$ as electrolyte.

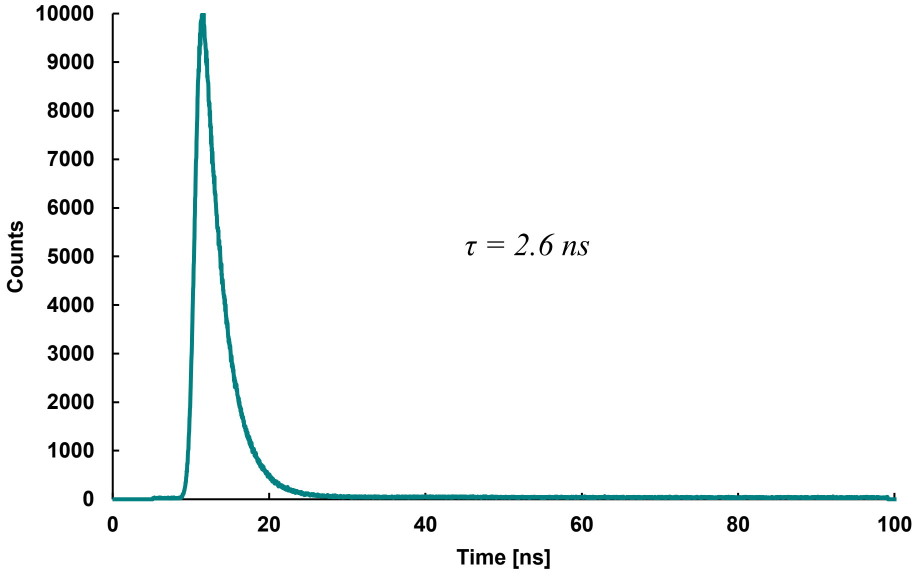

Figure 160. Fluorescence decay curve of **PC24** in degassed DCM (20 µM). Excitation wavelength – 394 nm

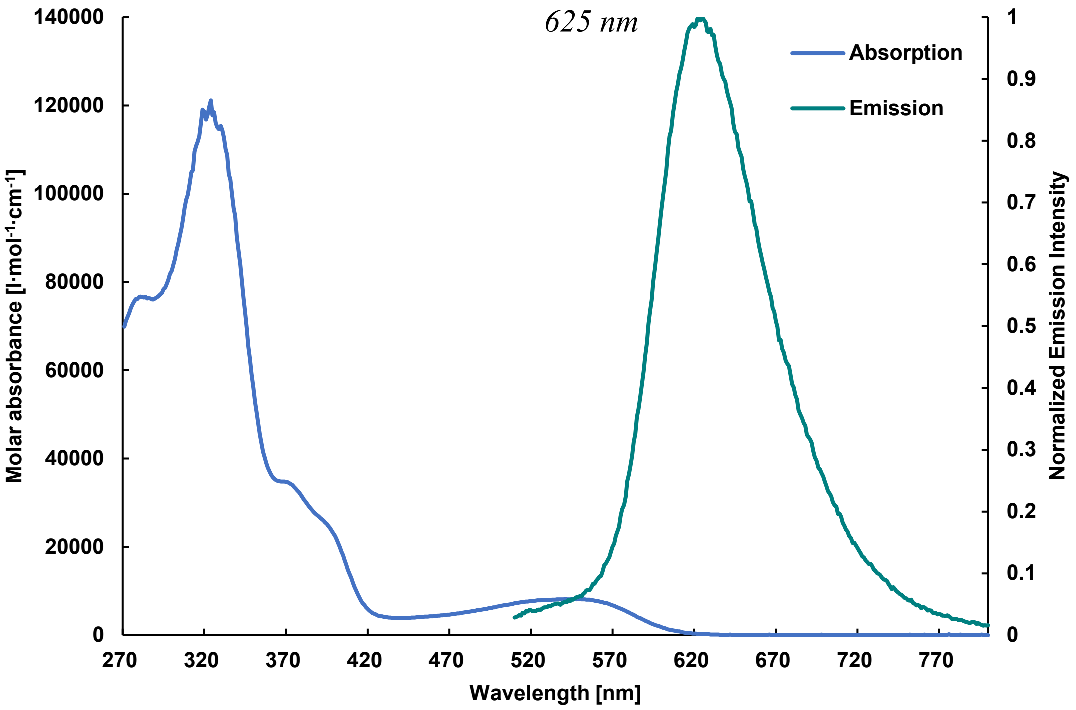

Figure S161. UV/Vis absorption and emission spectrum of **PC25** in degassed DCM (20 µM). Excitation wavelength – 457 nm.

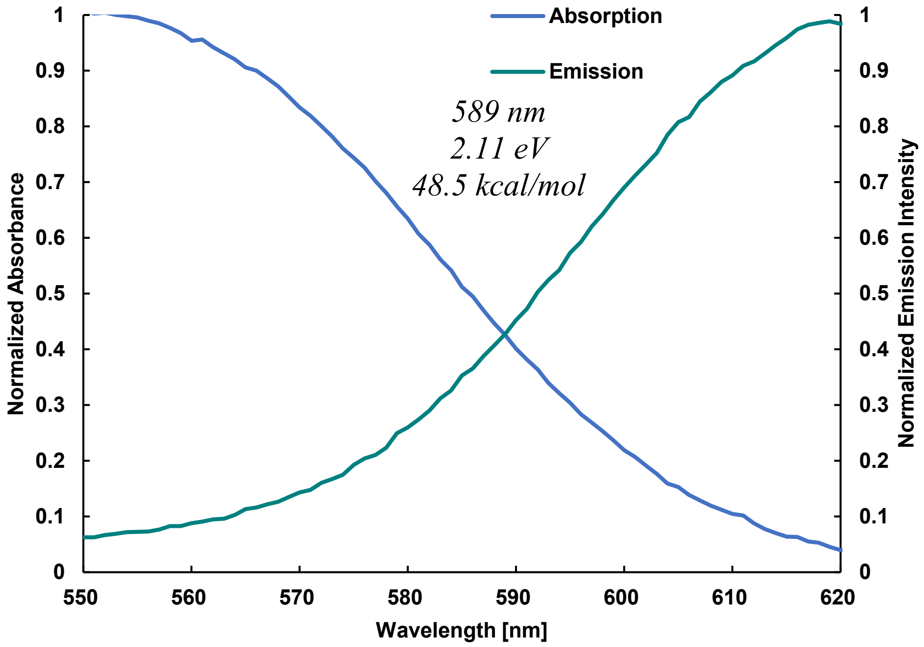

Figure S162. $E_{0\text{-}0}$ estimation at normalized emission and absorption overlap of **PC25**.

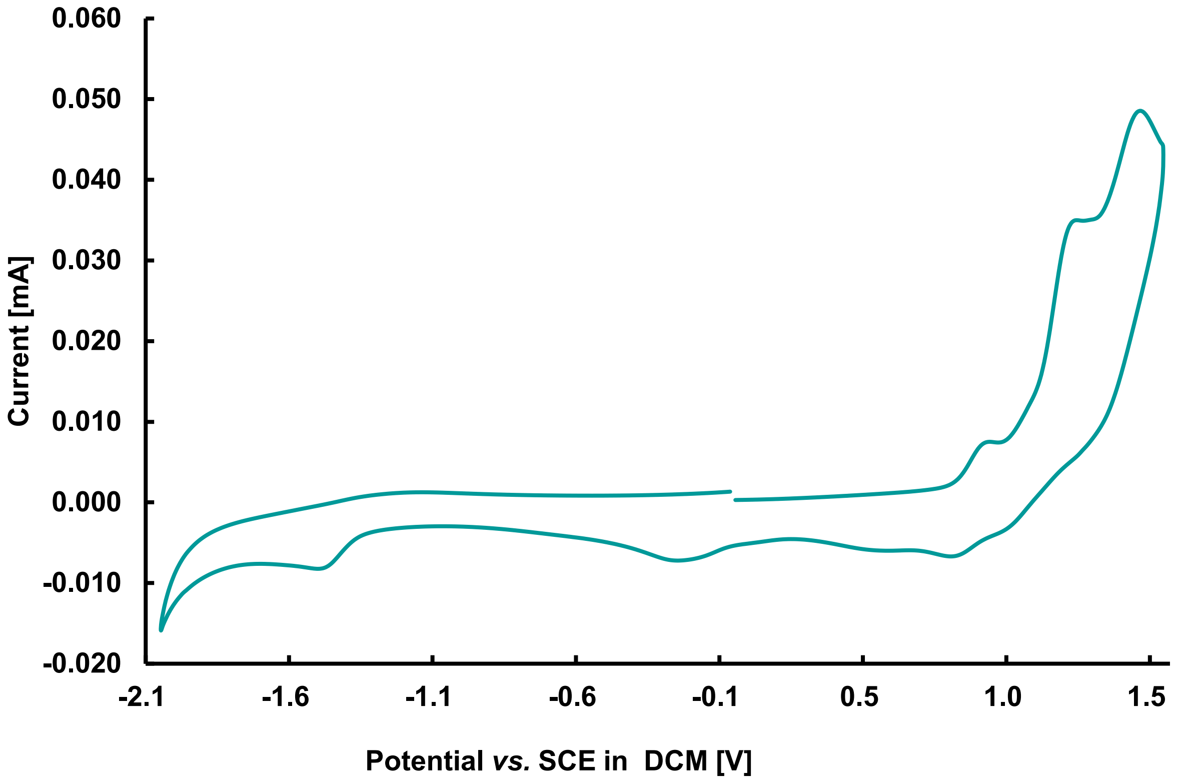

Figure S163. Cyclic voltammetry of **PC25** in degassed DCM (0.4 mM) using 0.1 M $^{n}Bu_4NPF_6$ as electrolyte.

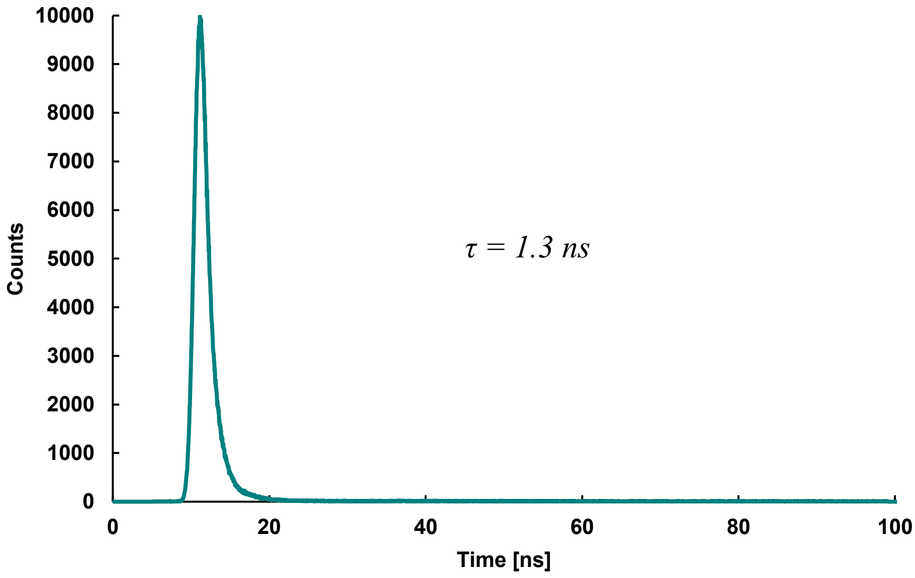

Figure S164. Fluorescence decay curve of **PC25** in degassed DCM (20 μM). Excitation wavelength – 394 nm.

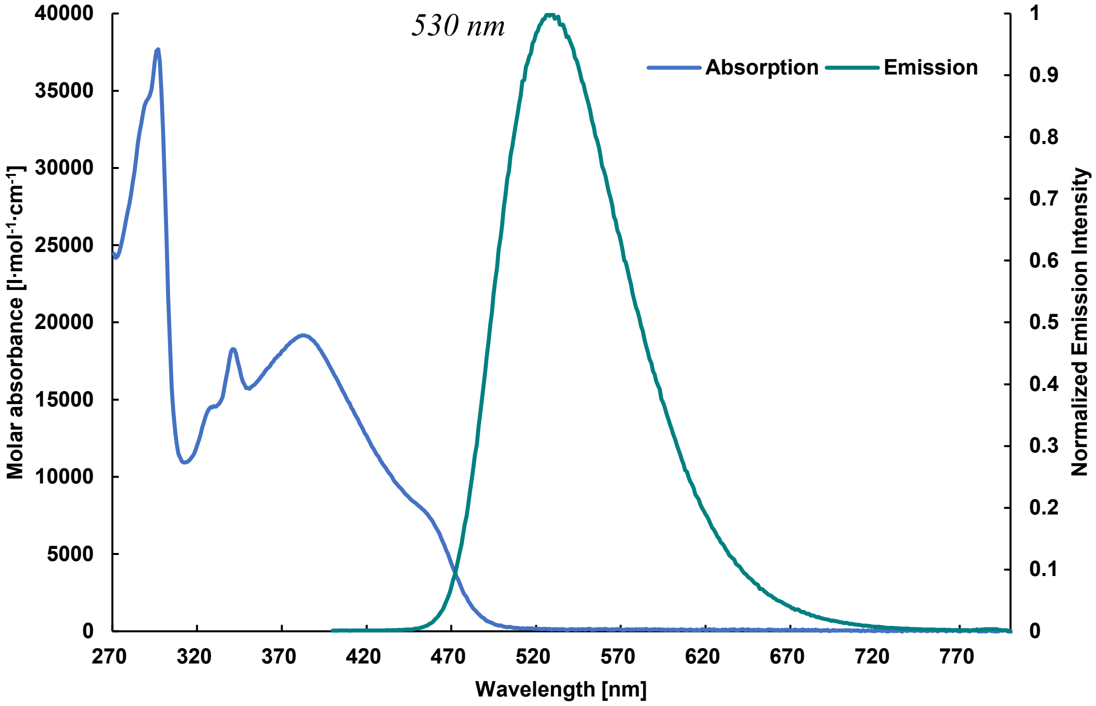

Figure S165. UV/Vis absorption and emission spectrum of **PC26** in degassed DCM (20 µM). Excitation wavelength – 394 nm.

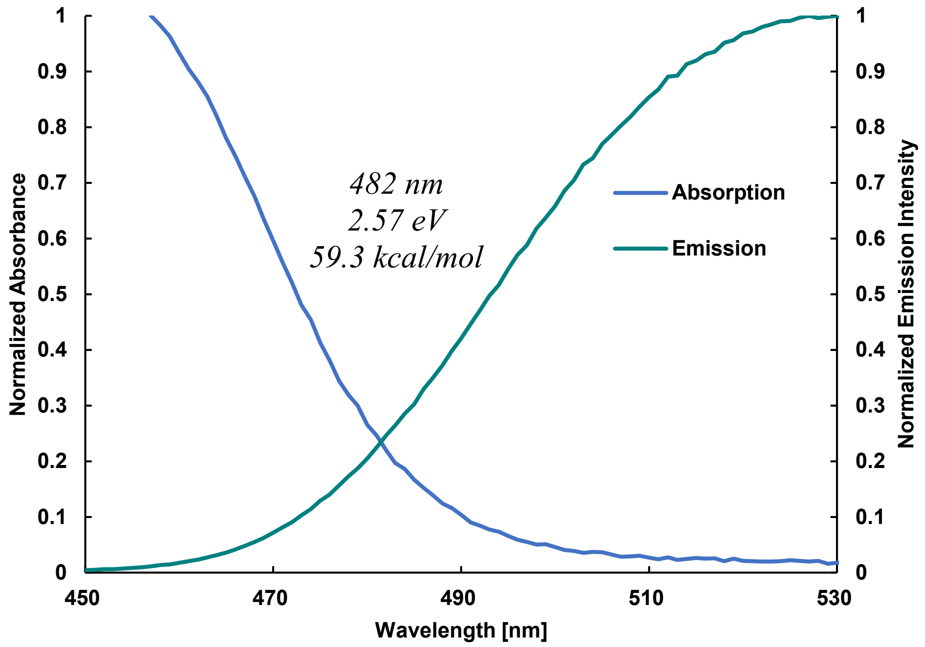

Figure S166. ***E***$E_{0\text{-}0}$ estimation at normalized emission and absorption overlap of **PC26**.

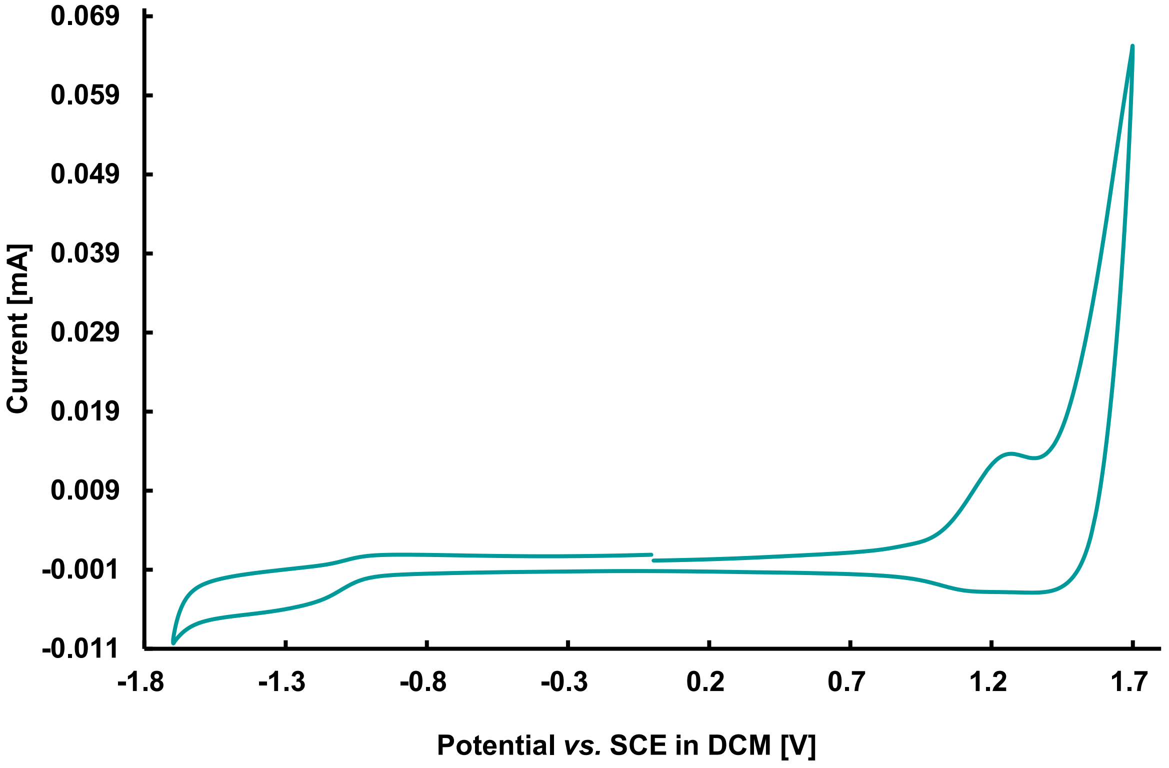

Figure S167. Cyclic voltammetry of **PC26** in degassed DCM (0.4 mM) using 0.1 M $^{n}Bu_4NPF_6$ as electrolyte.

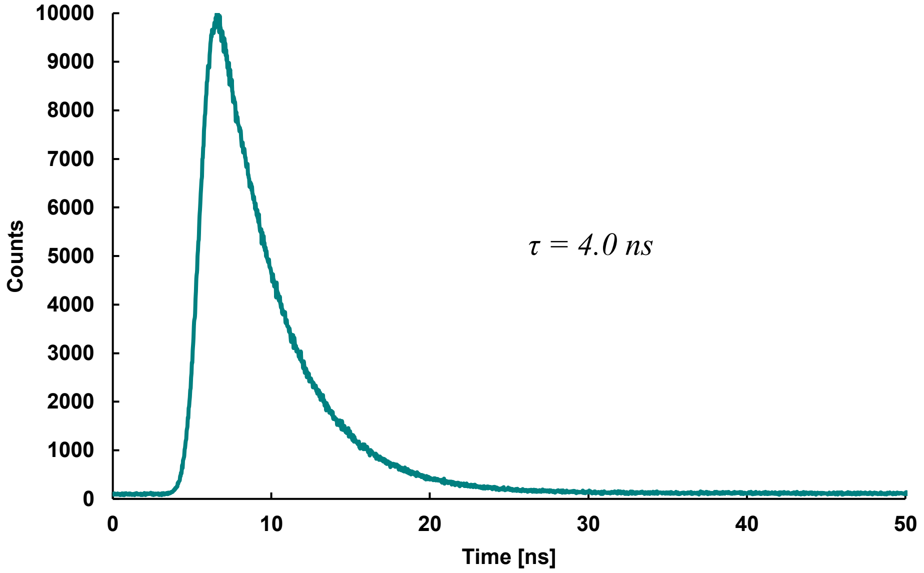

Figure S168. Fluorescence decay curve of **PC26** in degassed DCM (20 μM). Excitation wavelength – 394 nm.

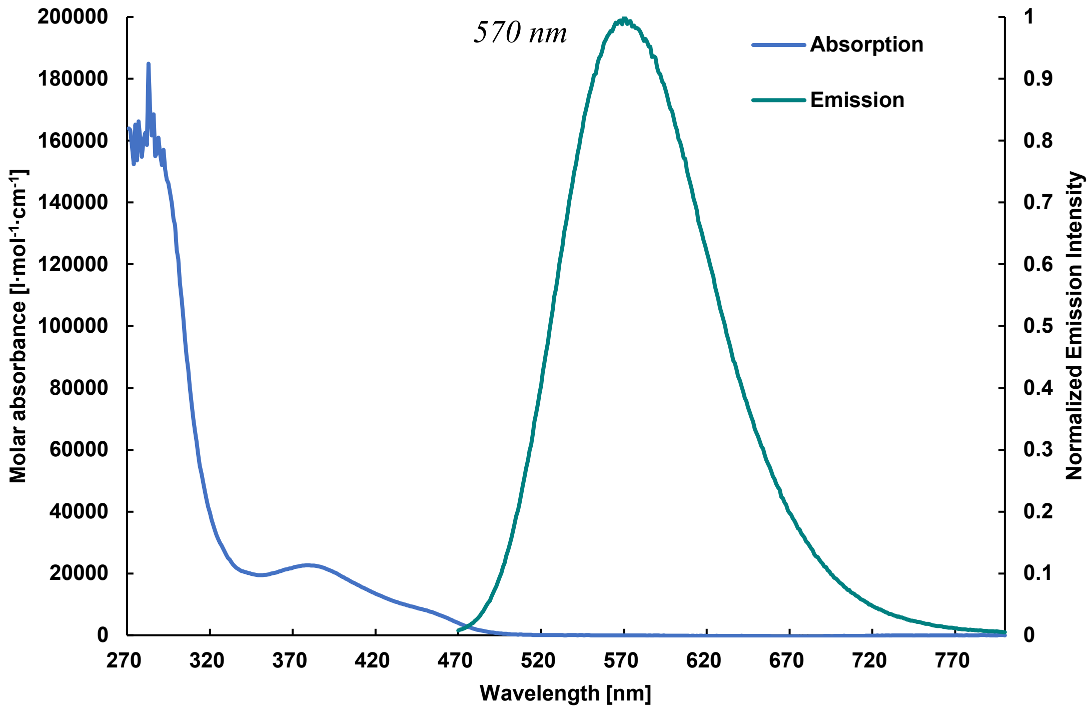

Figure S169. UV/Vis absorption and emission spectrum of **PC27** in degassed MeCN (20 µM). Excitation wavelength – 394 nm.

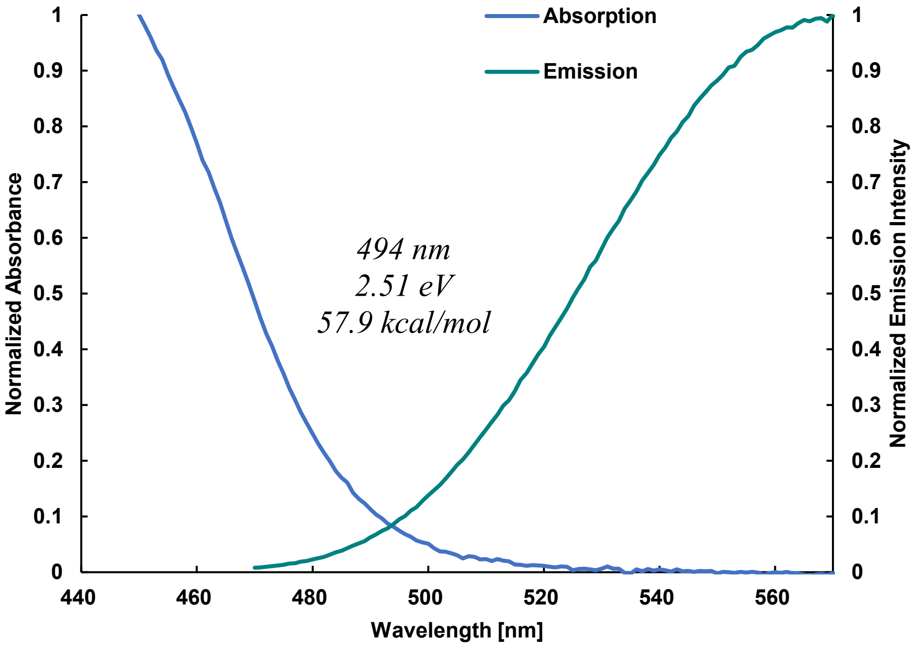

Figure S170. $E_{0\text{-}0}$ estimation at normalized emission and absorption overlap of **PC27**.

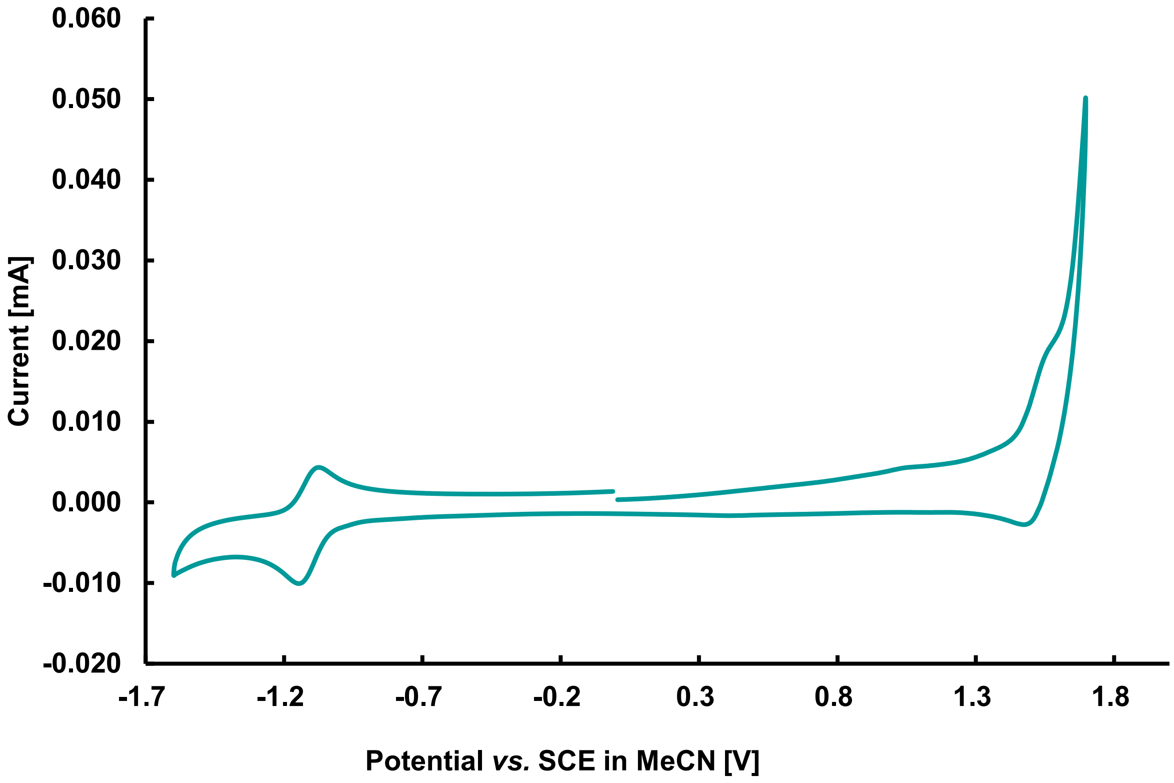

Figure S171. Cyclic voltammetry of **PC27** in degassed MeCN (0.4 mM) using 0.1 M $^{n}Bu_4NPF_6$ as electrolyte.

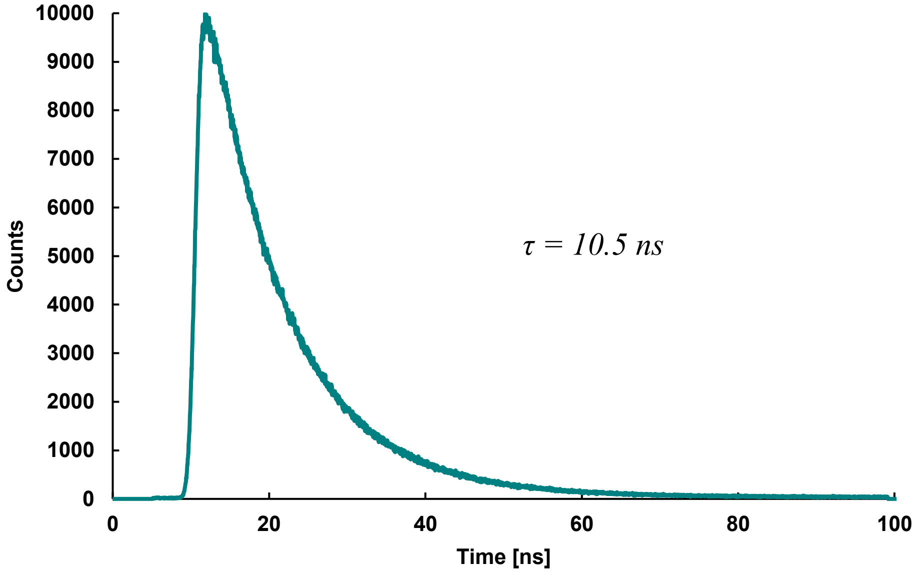

Figure 172. Fluorescence decay curve of **PC27** in degassed MeCN (20 µM). Excitation wavelength – 394 nm.

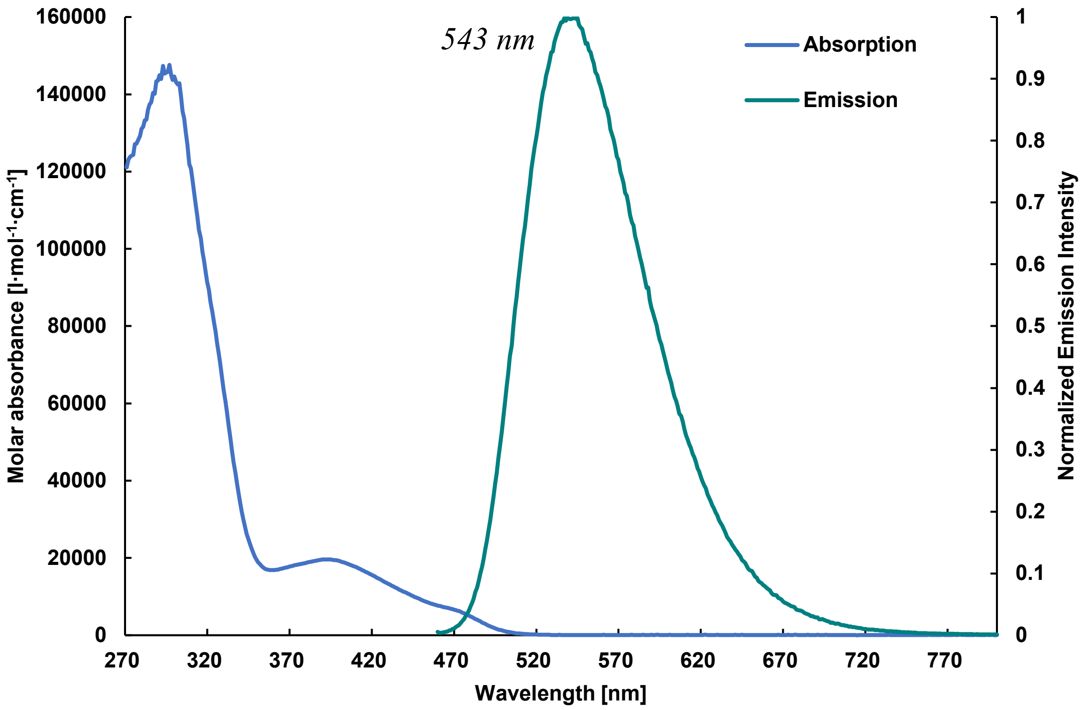

Figure S173. UV/Vis absorption and emission spectrum of **PC28** in degassed DCM (20 µM). Excitation wavelength – 456 nm.

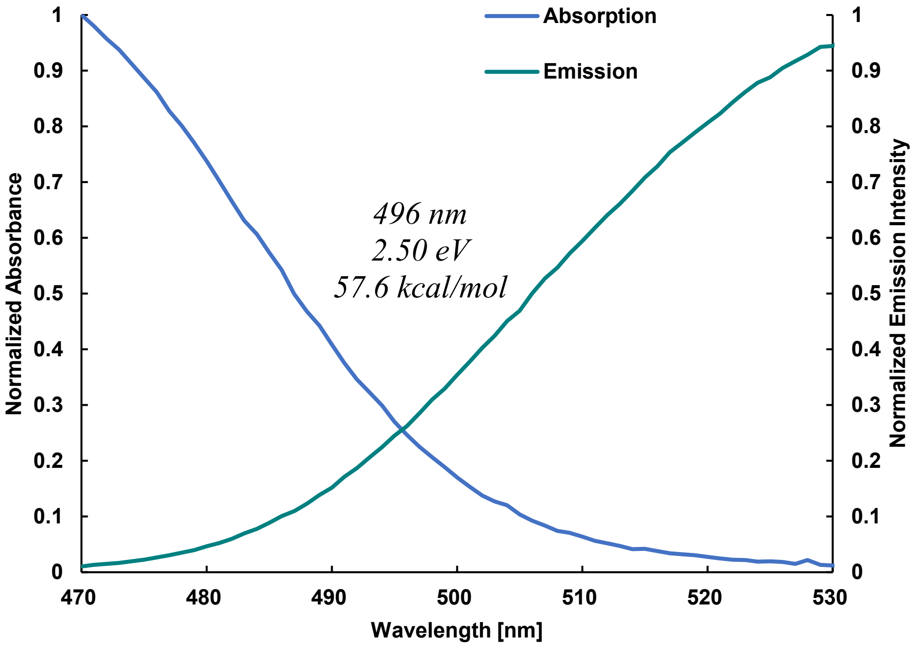

Figure S174. $E_{0\text{-}0}$ estimation at normalized emission and absorption overlap of **PC28**.

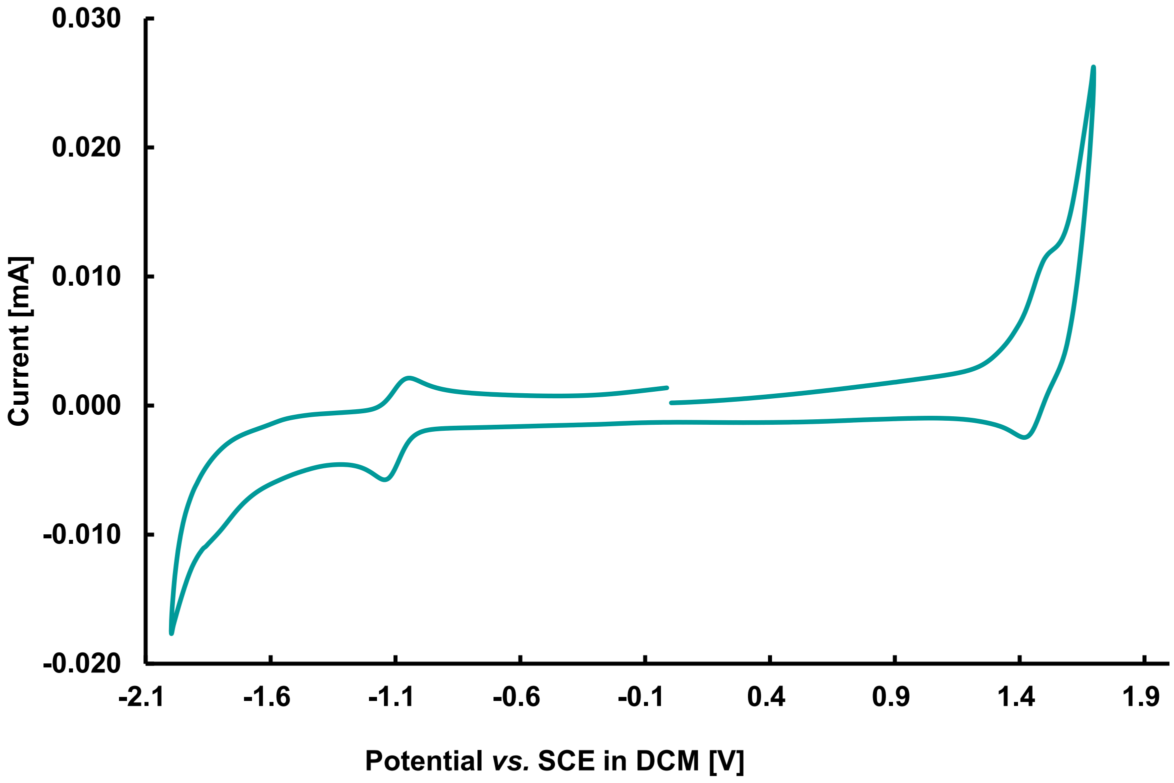

Figure S175. Cyclic voltammetry of **PC28** in degassed DCM (0.4 mM) using 0.1 M $^nBu_4NPF_6$ as electrolyte.

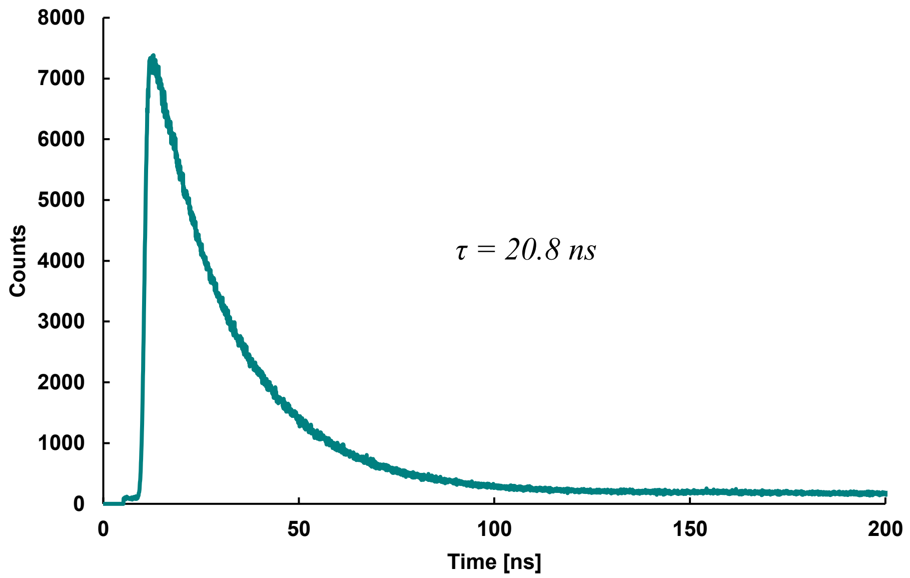

Figure S176 Fluorescence decay curve of **PC28** in degassed DCM (20 µM). Excitation wavelength – 394 nm.

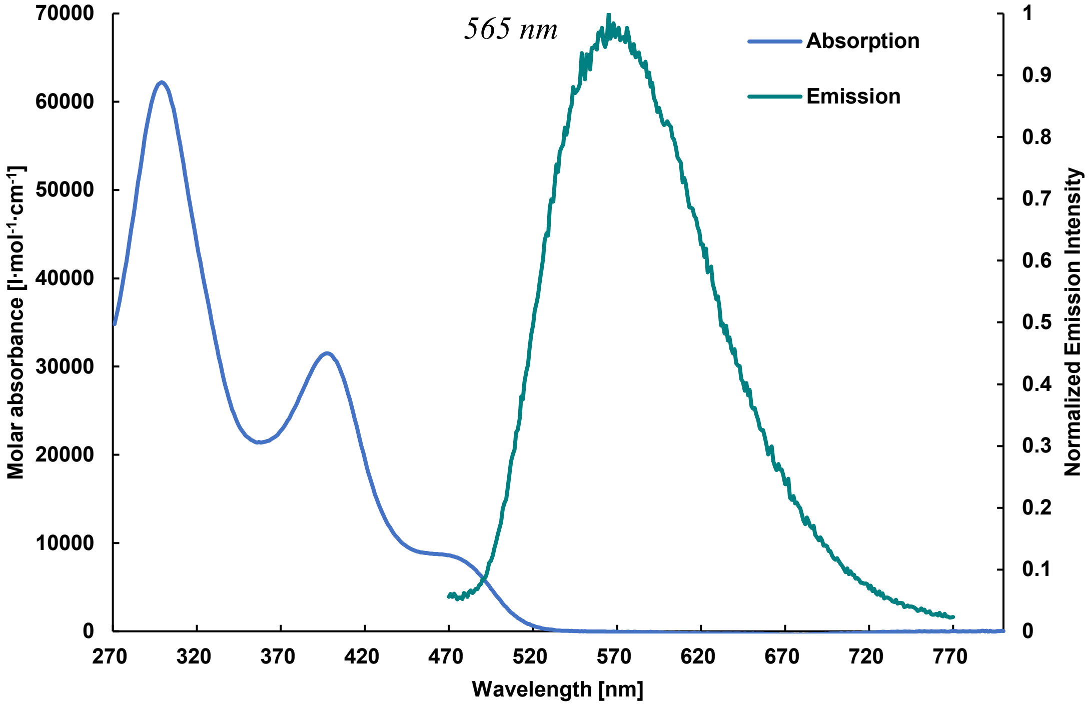

Figure S177. UV/Vis absorption and emission spectrum of **PC29** in degassed MeCN (20 µM). Excitation wavelength – 394 nm.

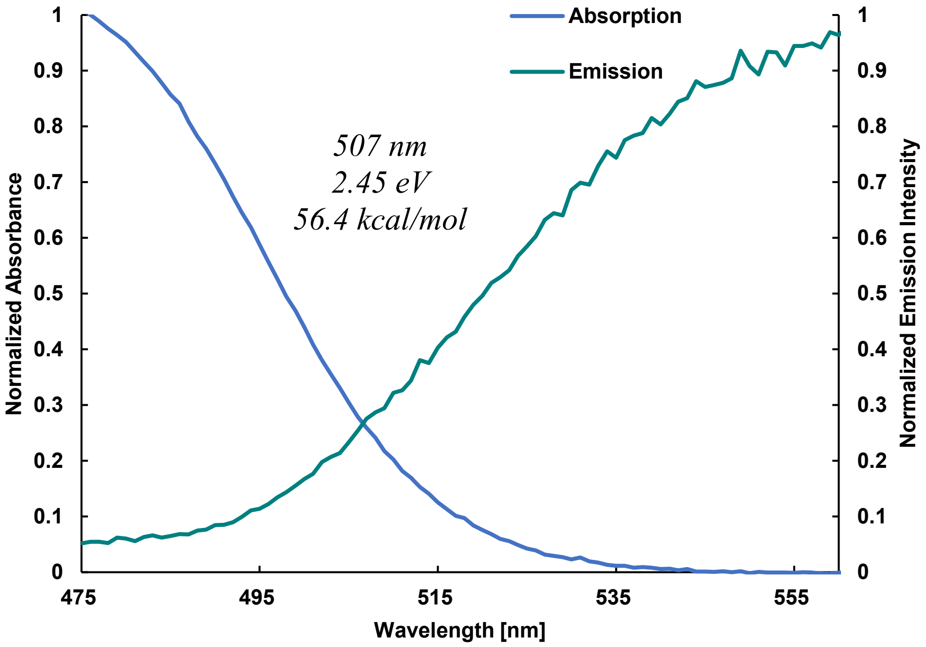

Figure S178. $E_{0\text{-}0}$ estimation at normalized emission and absorption overlap of **PC29**.

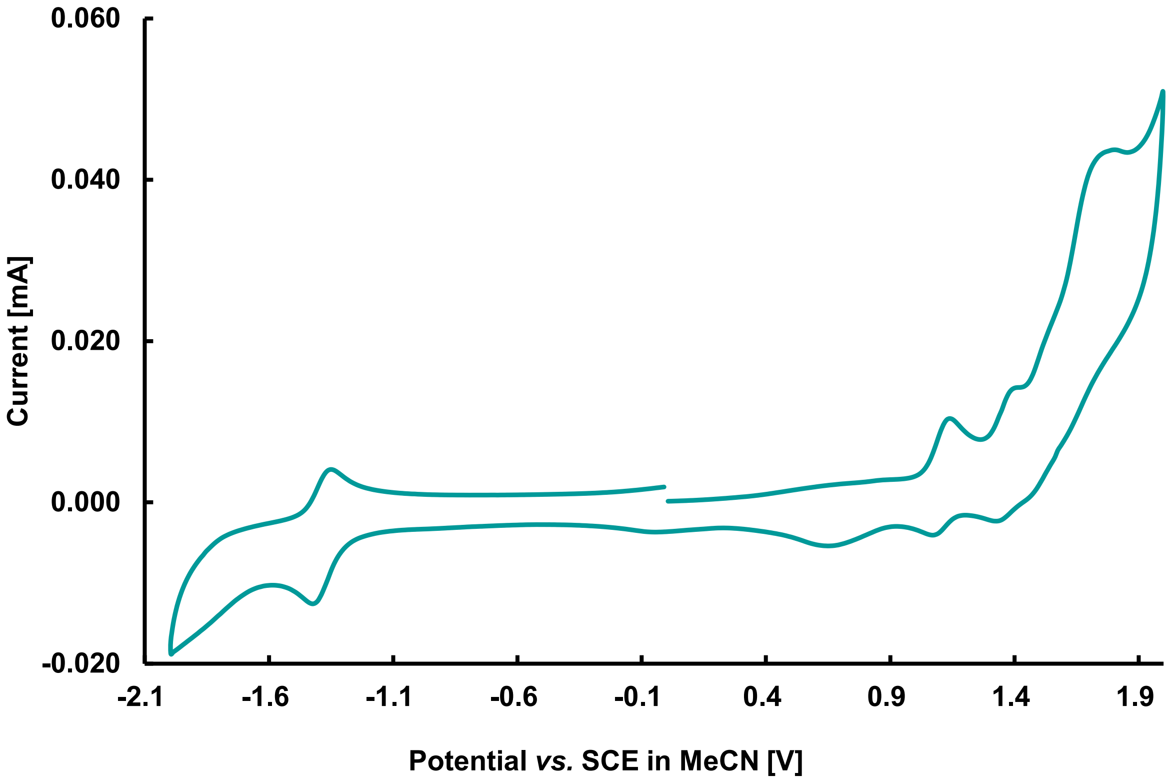

Figure S179. Cyclic voltammetry of **PC29** in degassed MeCN (0.4 mM) using 0.1 M $^{n}Bu_4NPF_6$ as electrolyte.

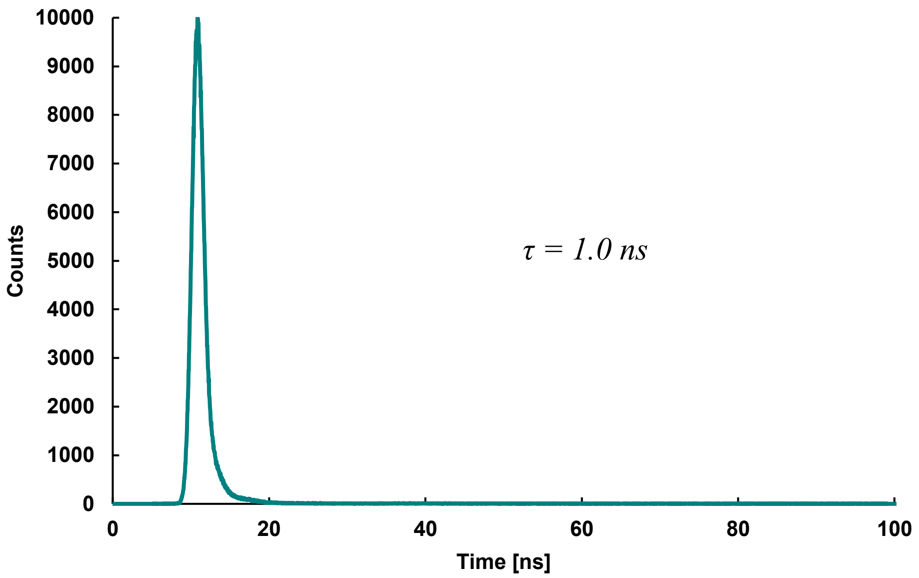

Figure S180. Fluorescence decay curve of **PC29** in degassed MeCN (20 μM). Excitation wavelength – 394 nm.

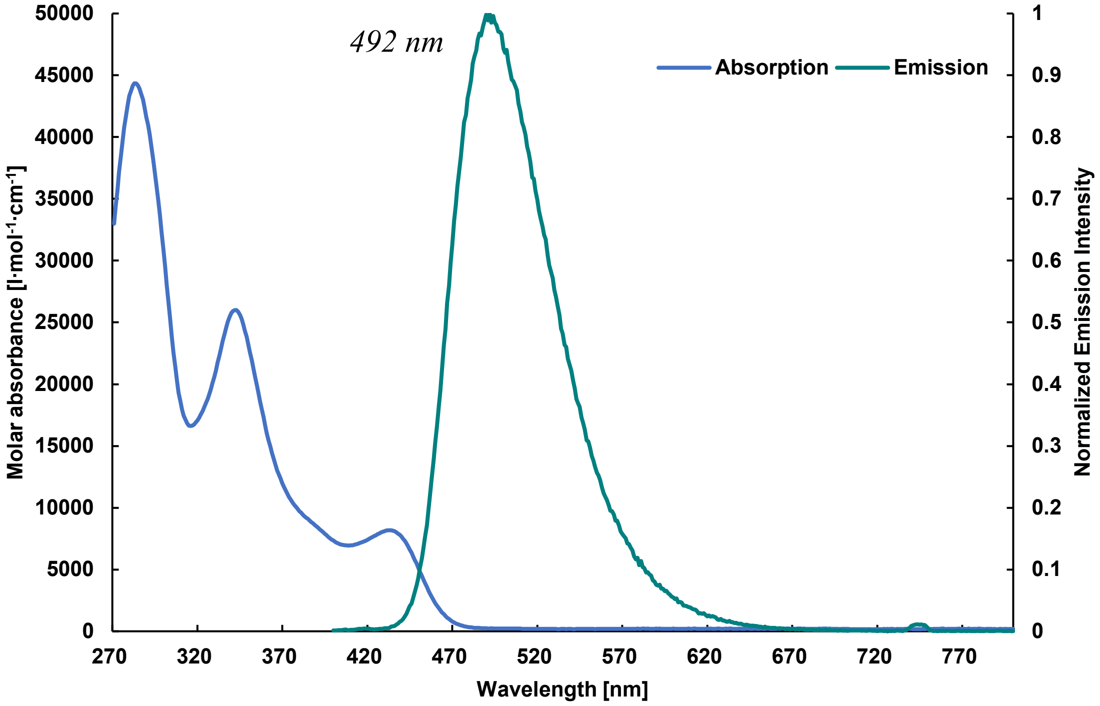

Figure S181. UV/Vis absorption and emission spectrum of **PC30** in degassed MeCN (20 µM). Excitation wavelength – 394 nm.

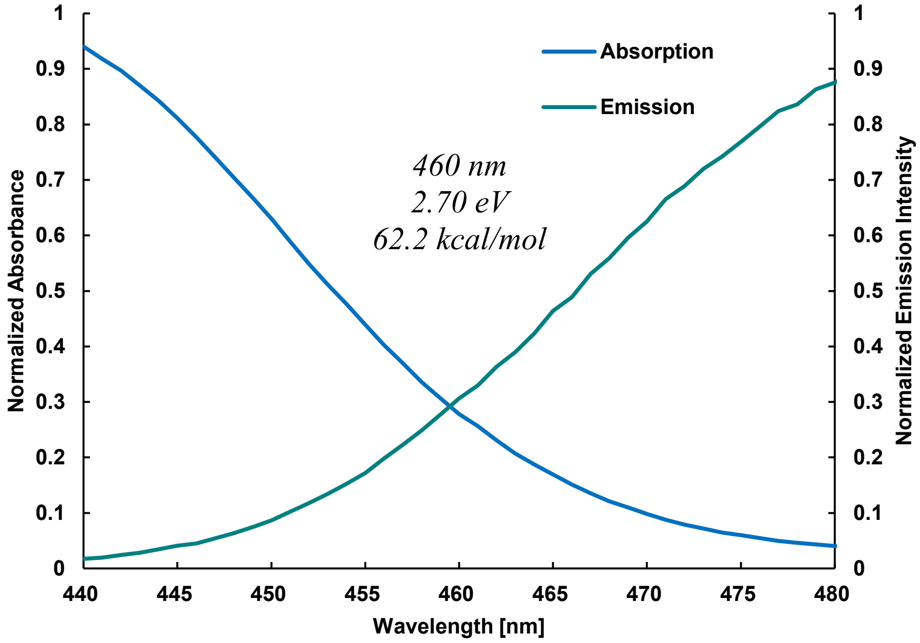

Figure S182. $E_{0\text{-}0}$ estimation at normalized emission and absorption overlap of **PC30**.

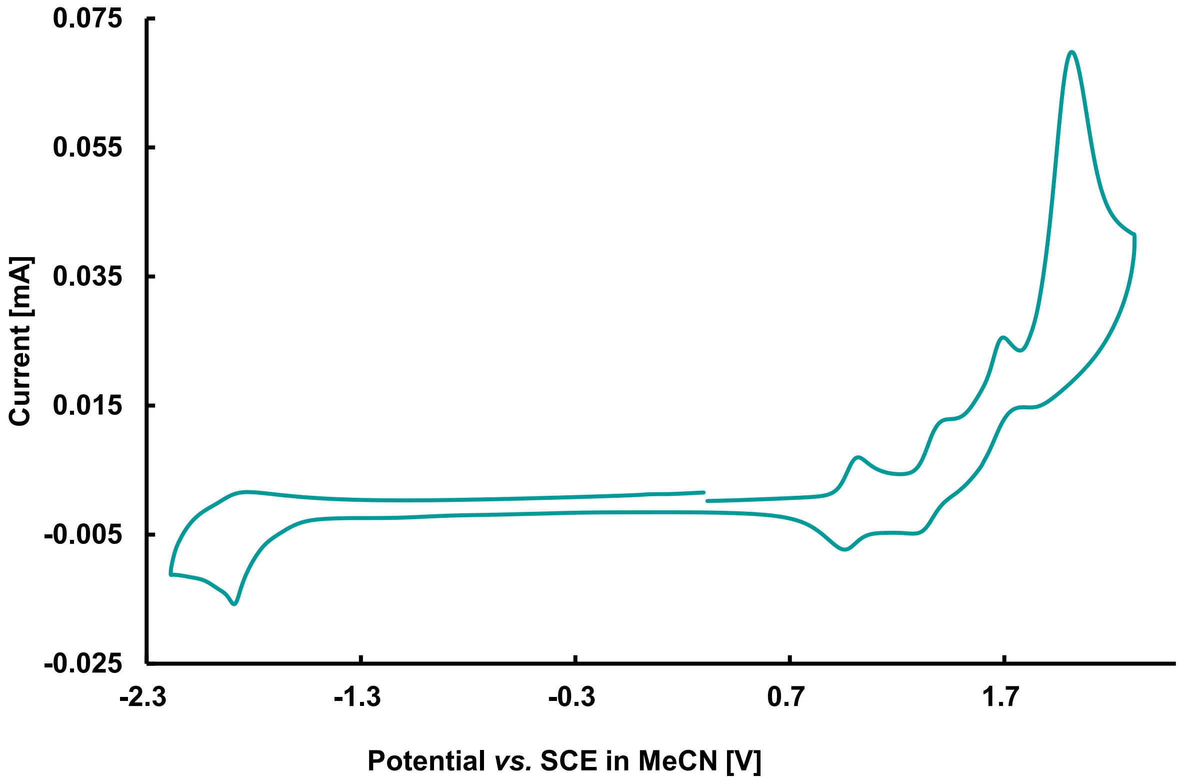

Figure S183. Cyclic voltammetry of **PC30** in degassed MeCN (0.4 mM) using 0.1 M $^{n}Bu_4NPF_6$ as electrolyte.

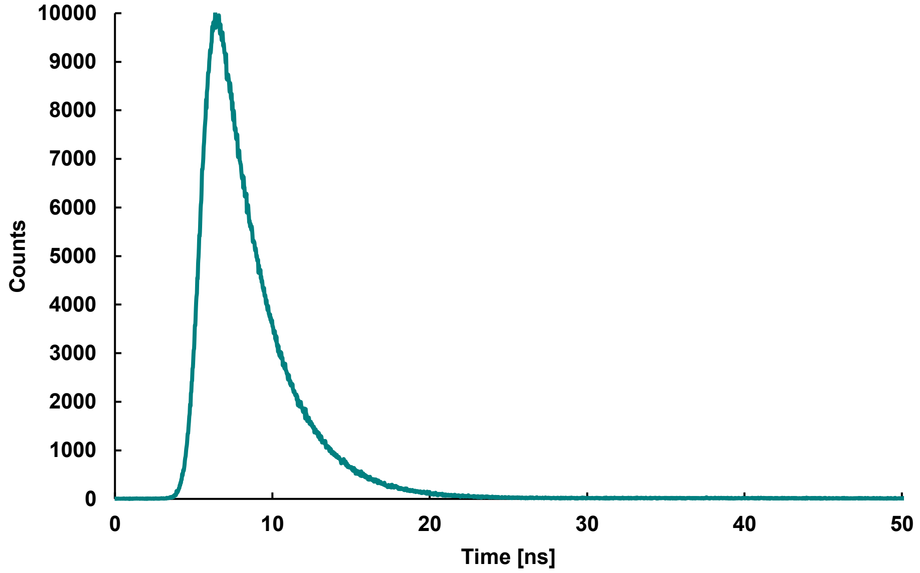

Figure S184. Fluorescence decay curve of **PC30** in degassed MeCN (20 μM). Excitation wavelength – 394 nm.

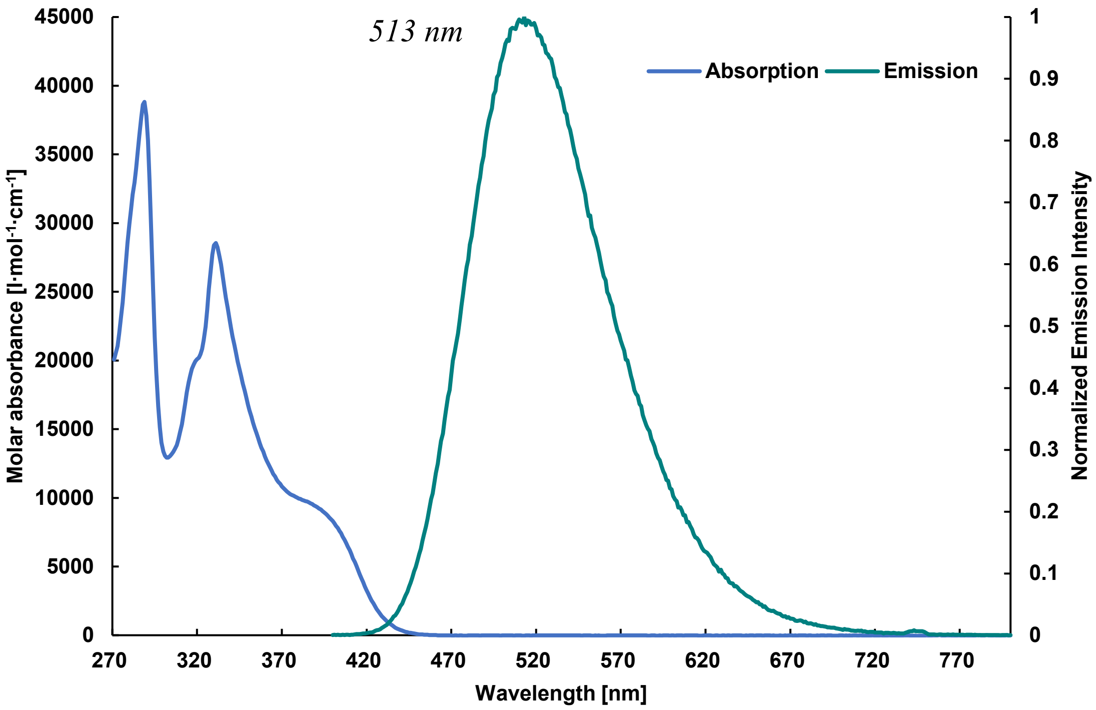

Figure S185. UV/Vis absorption and emission spectrum of **PC31** in degassed MeCN (20 μM). Excitation wavelength – 394 nm.

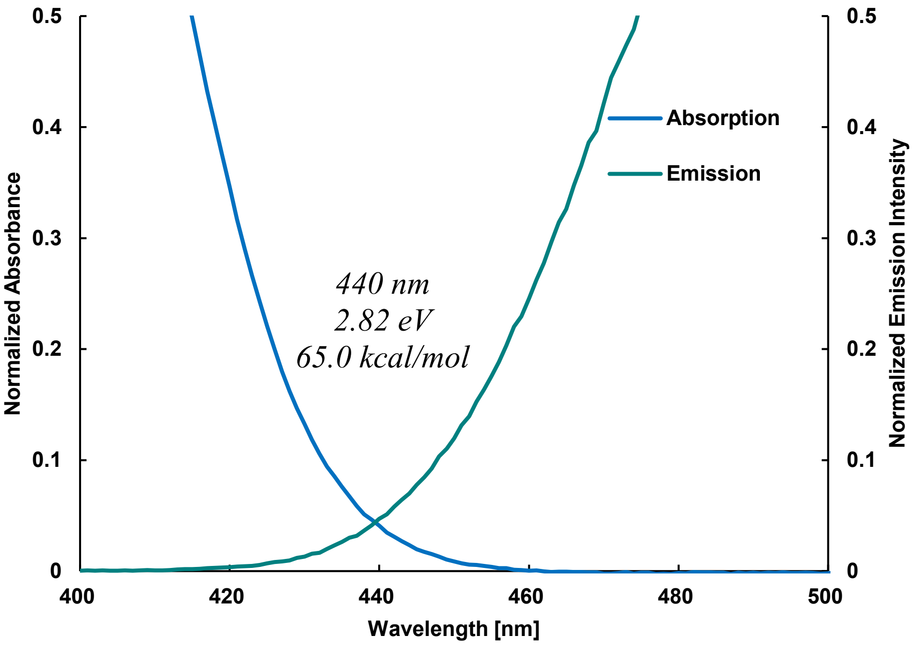

Figure S186. $E_{0\text{-}0}$ estimation at normalized emission and absorption overlap of **PC31**.

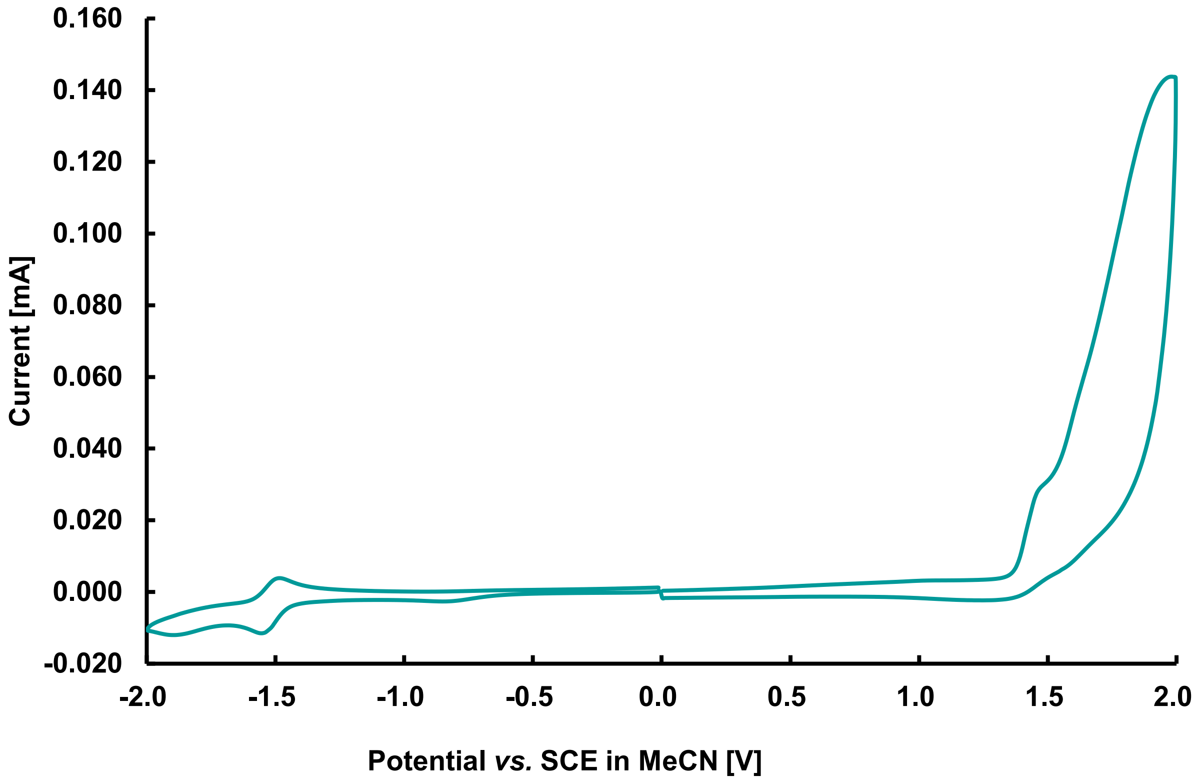

Figure S187. Cyclic voltammetry of **PC31** in degassed MeCN (0.4 mM) using 0.1 M $^{n}Bu_4NPF_6$ as electrolyte.

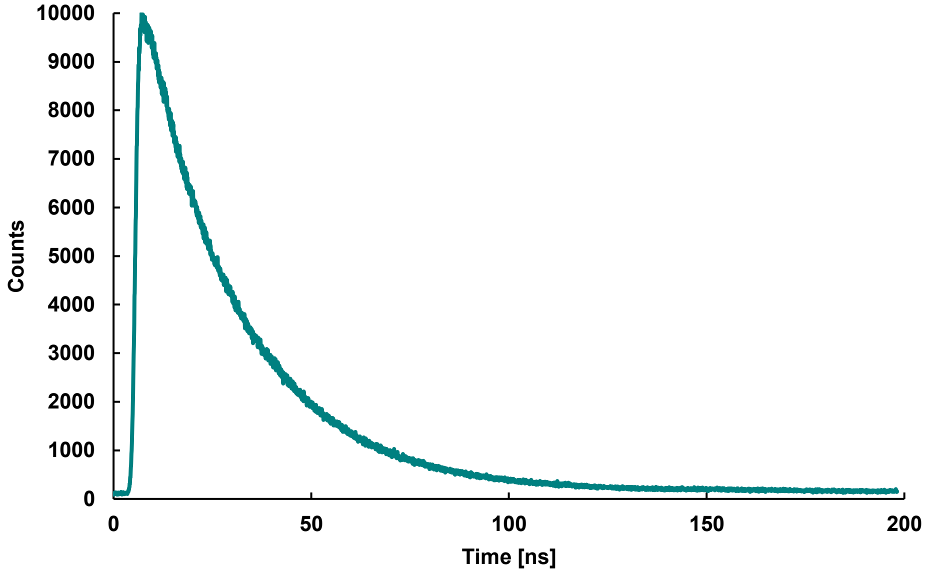

Figure S188. Fluorescence decay curve of **PC31** in degassed MeCN (20 µM). Excitation wavelength – 394 nm.

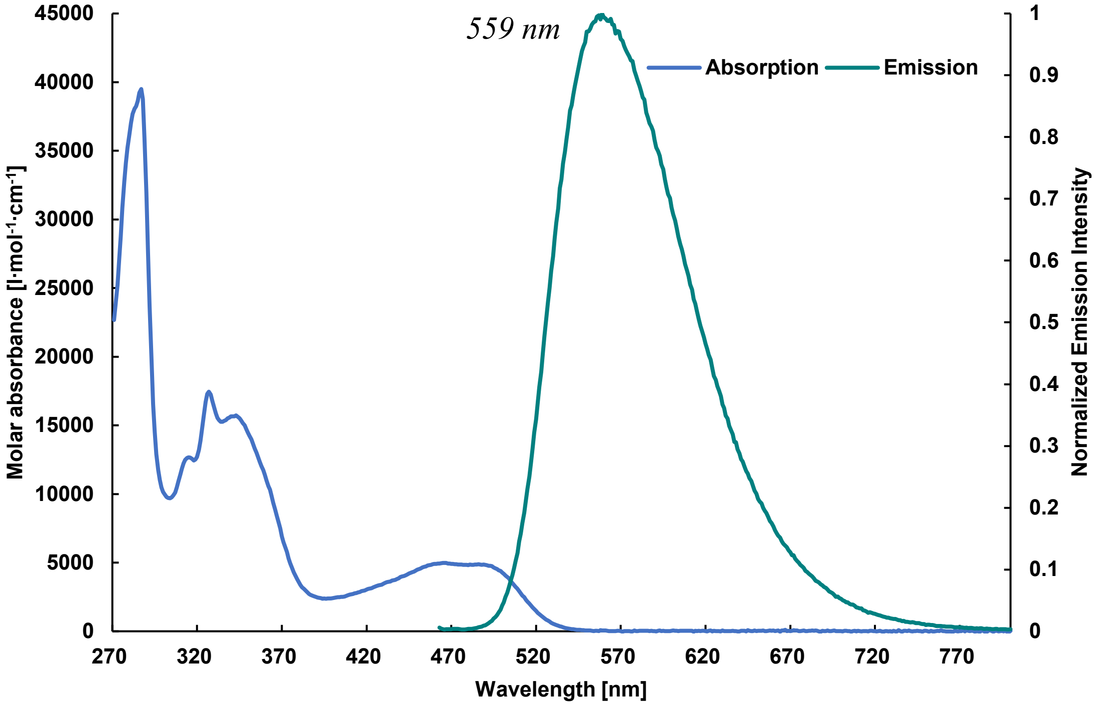

Figure S189. UV/Vis absorption and emission spectrum of **PC32** in degassed DCM (20 μM). Excitation wavelength – 456 nm.

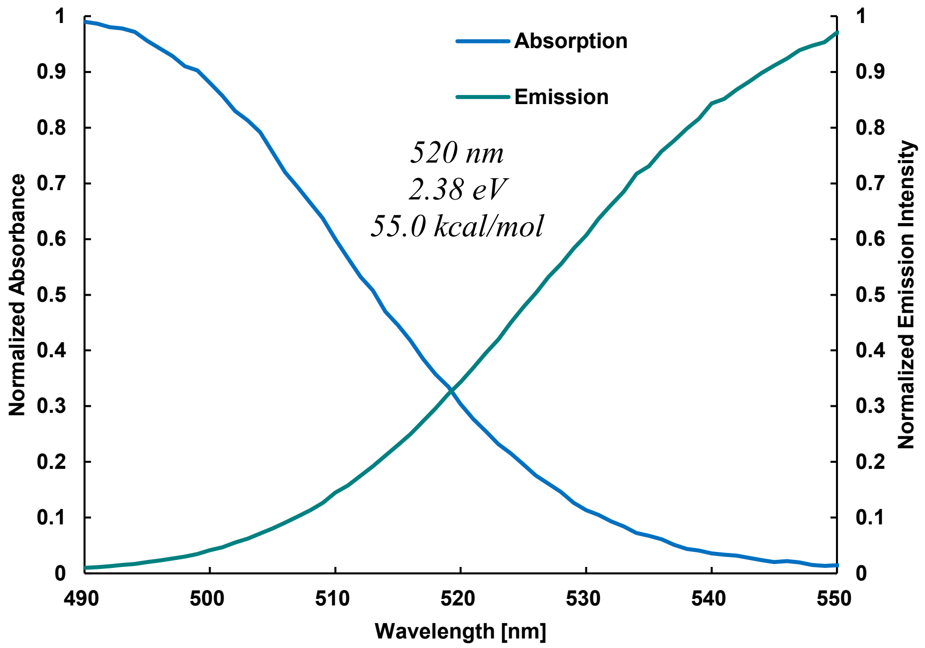

Figure S190. $E_{0\text{-}0}$ estimation at normalized emission and absorption overlap of **PC32**.

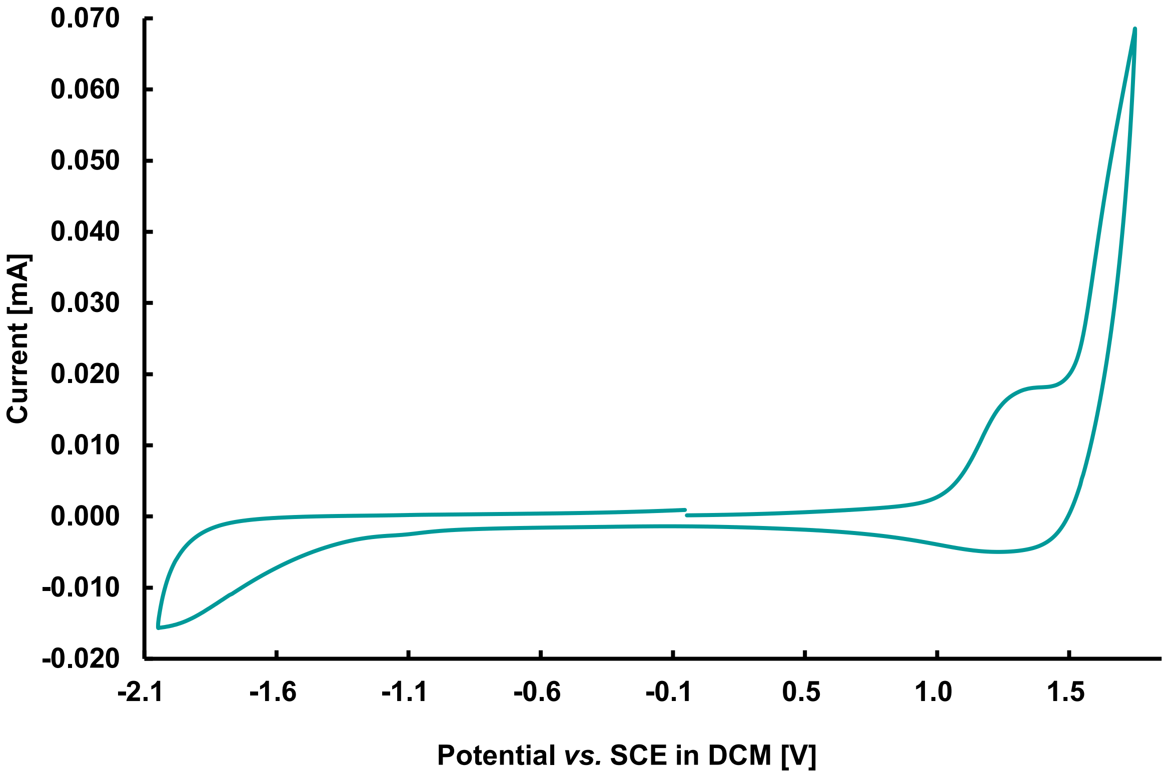

Figure S191. Cyclic voltammetry of **PC32** in degassed DCM (0.4 mM) using 0.1 M $^{n}Bu_4NPF_6$ as electrolyte.

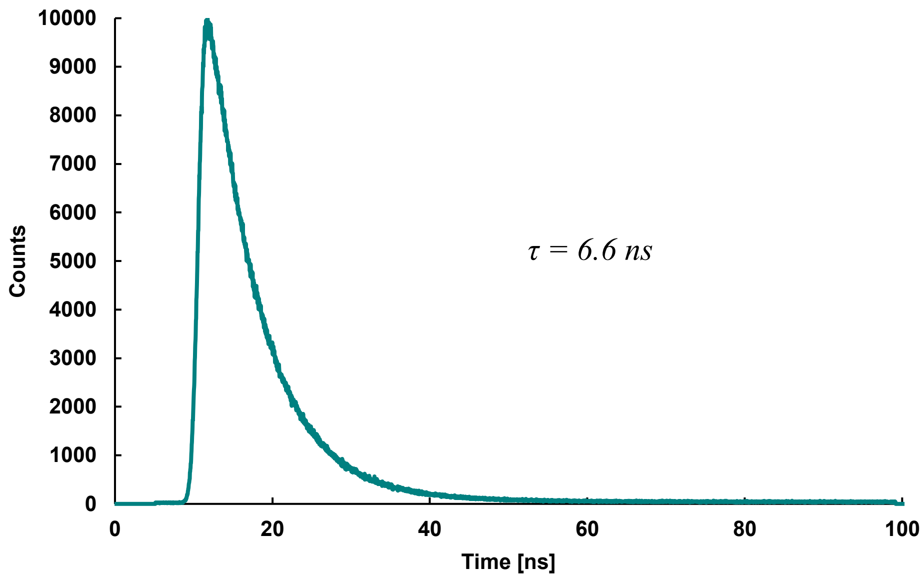

Figure S192. Fluorescence decay curve of **PC32** in degassed DCM (20 μM). Excitation wavelength – 394 nm.

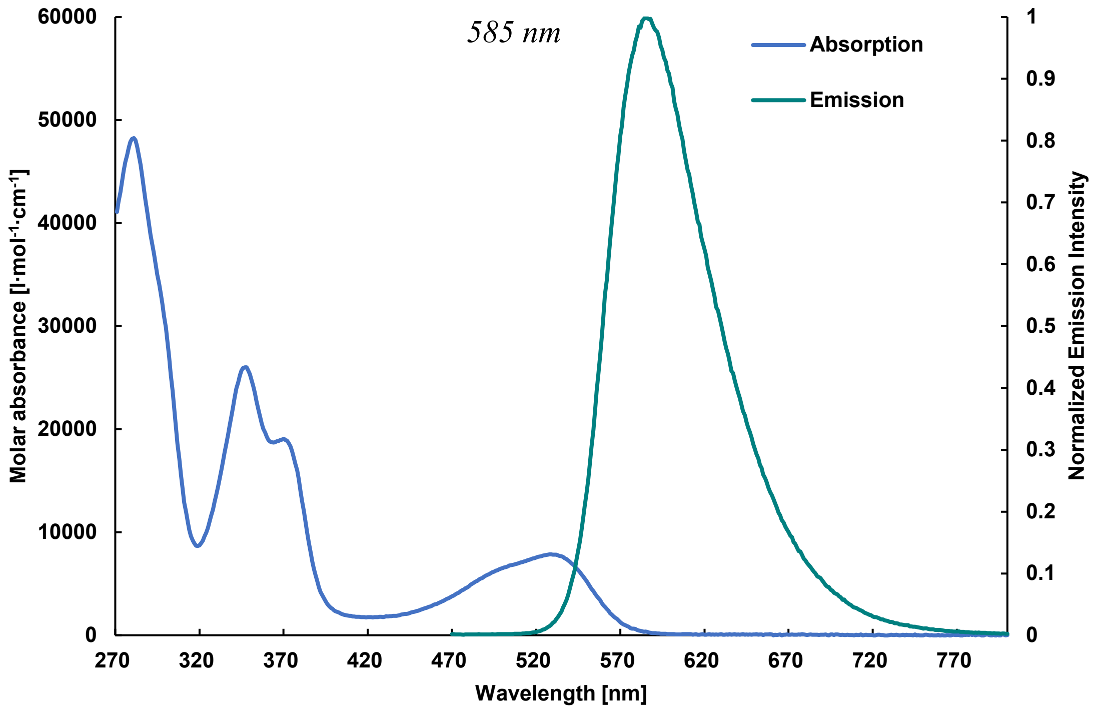

Figure S193. UV/Vis absorption and emission spectrum of **PC33** in degassed DCM (20 µM). Excitation wavelength – 456 nm.

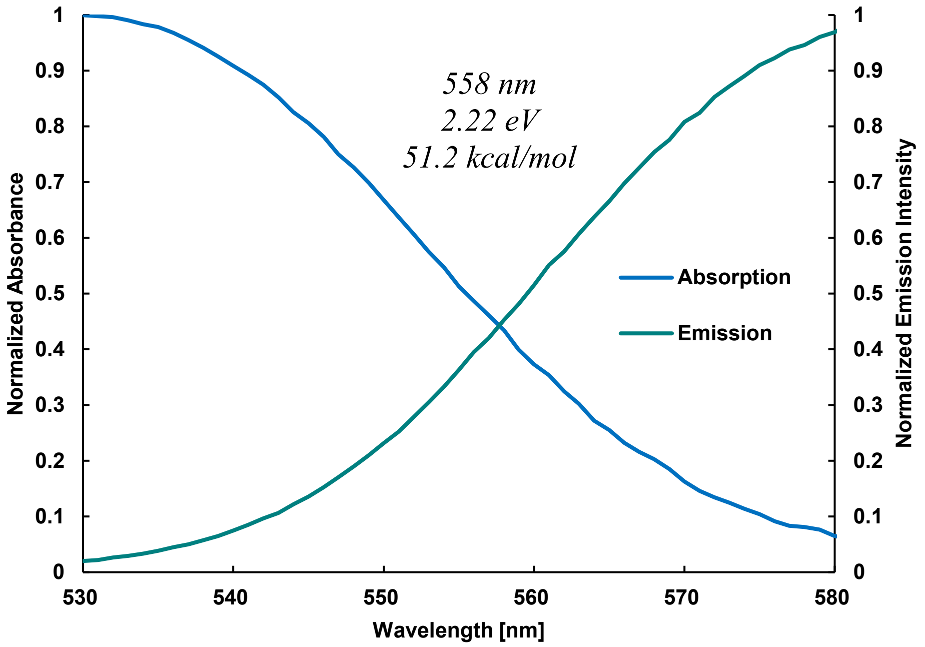

Figure S194. $\boldsymbol{E_{0\text{-}0}}$ estimation at normalized emission and absorption overlap of **PC33**.

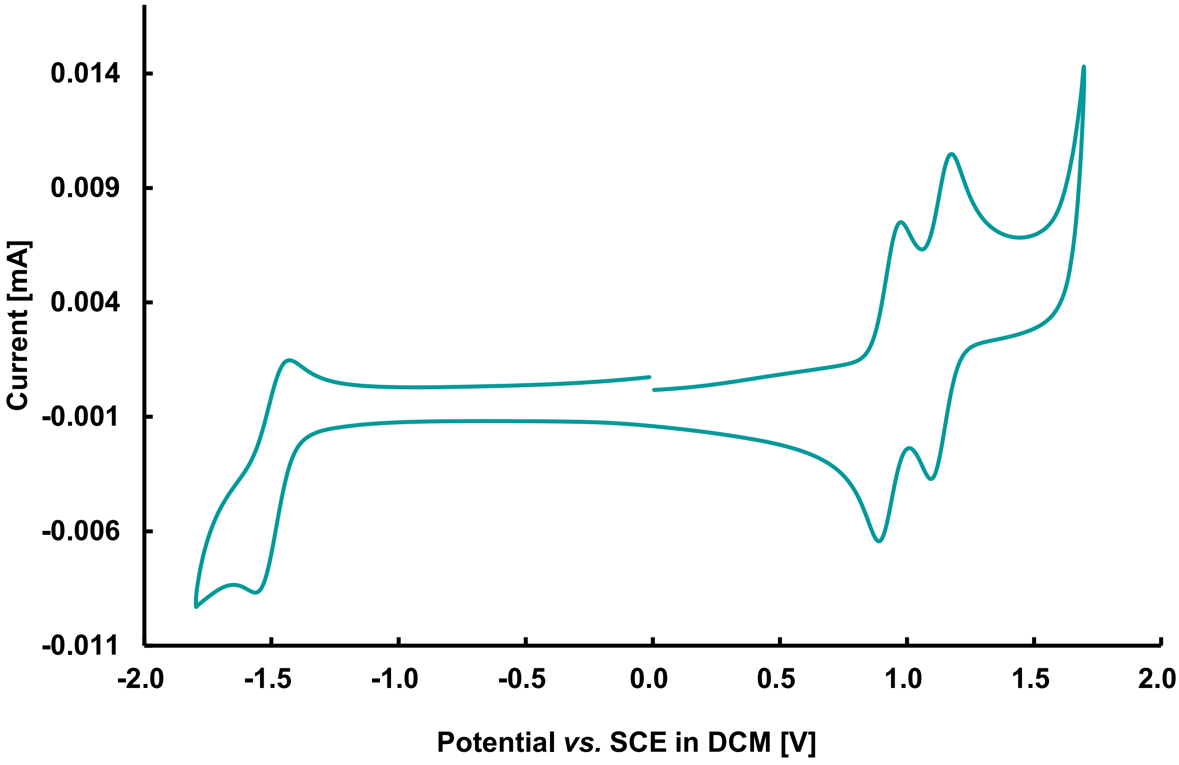

Figure S195. Cyclic voltammetry of **PC33** in degassed DCM (0.4 mM) using 0.1 M $^{n}Bu_4NPF_6$ as electrolyte.

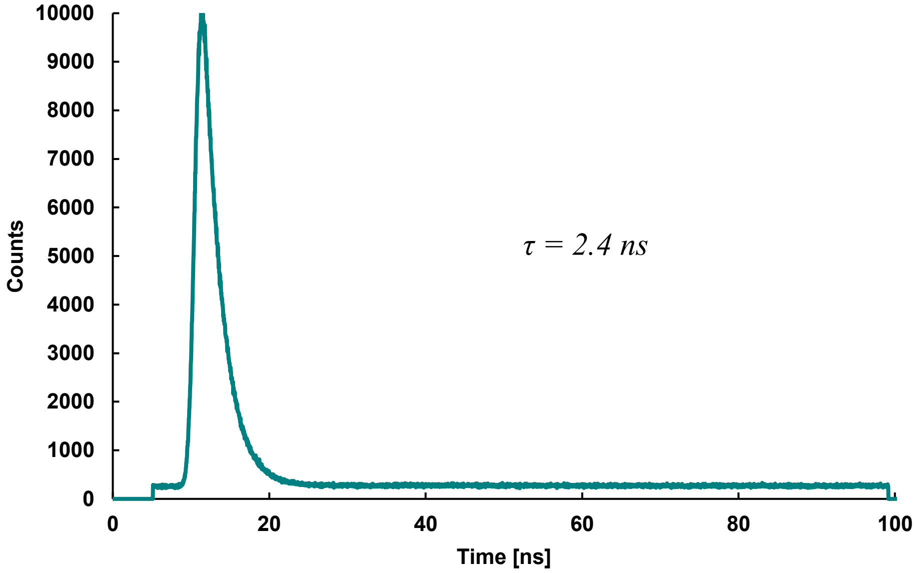

Figure S196. Fluorescence decay curve of **PC33** in degassed DCM (20 μM). Excitation wavelength – 394 nm.

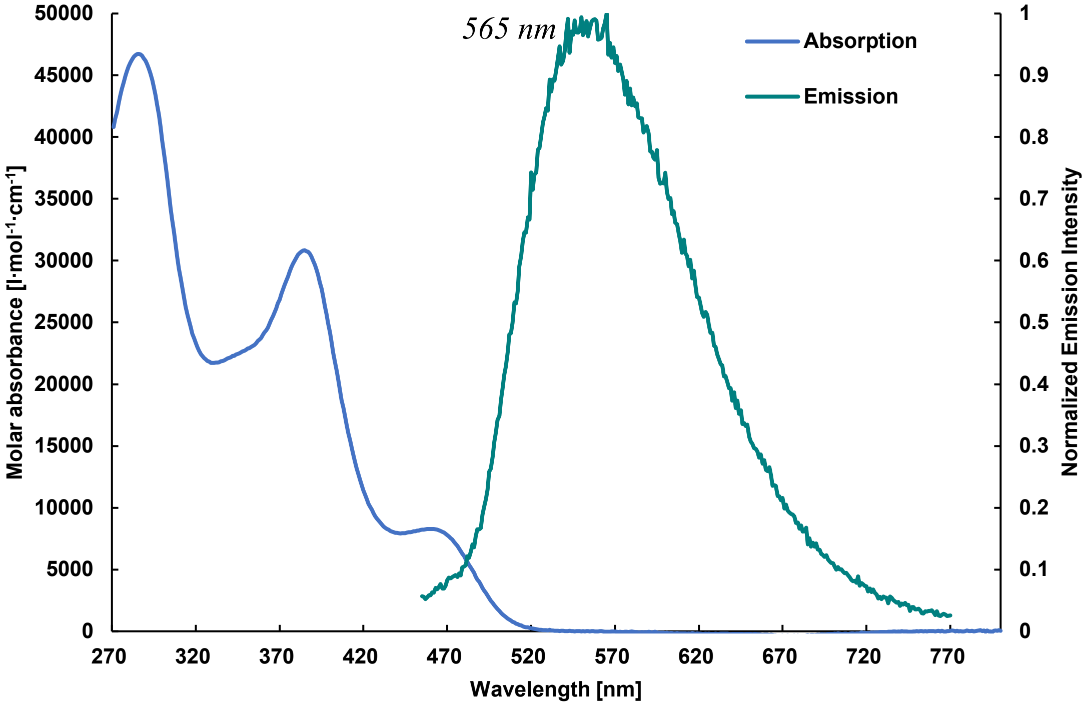

Figure S197. UV/Vis absorption and emission spectrum of **PC34** in degassed MeCN (20 μM). Excitation wavelength – 394 nm.

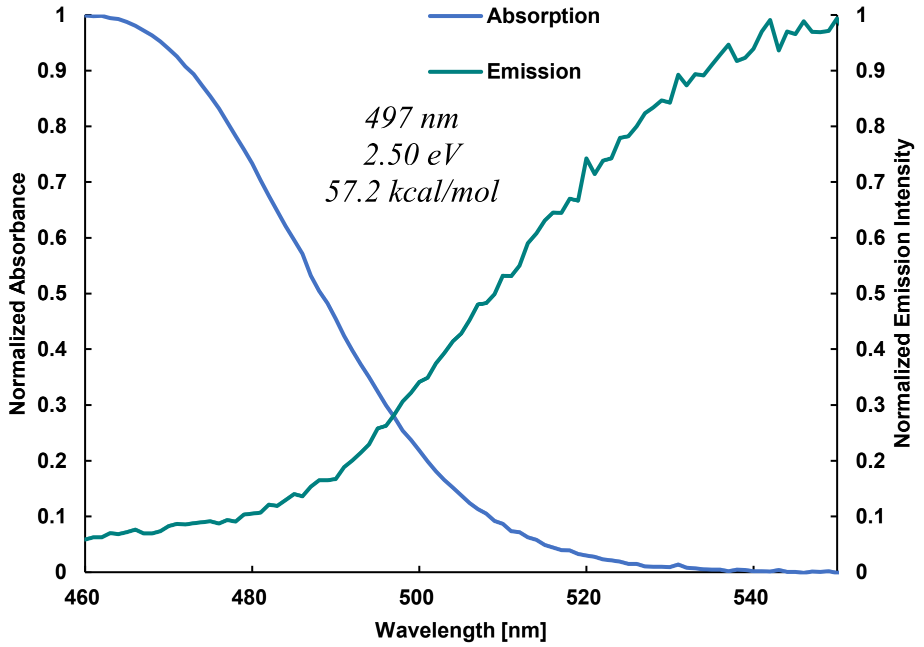

Figure S198. E_{0-0} estimation at normalized emission and absorption overlap of **PC34**.

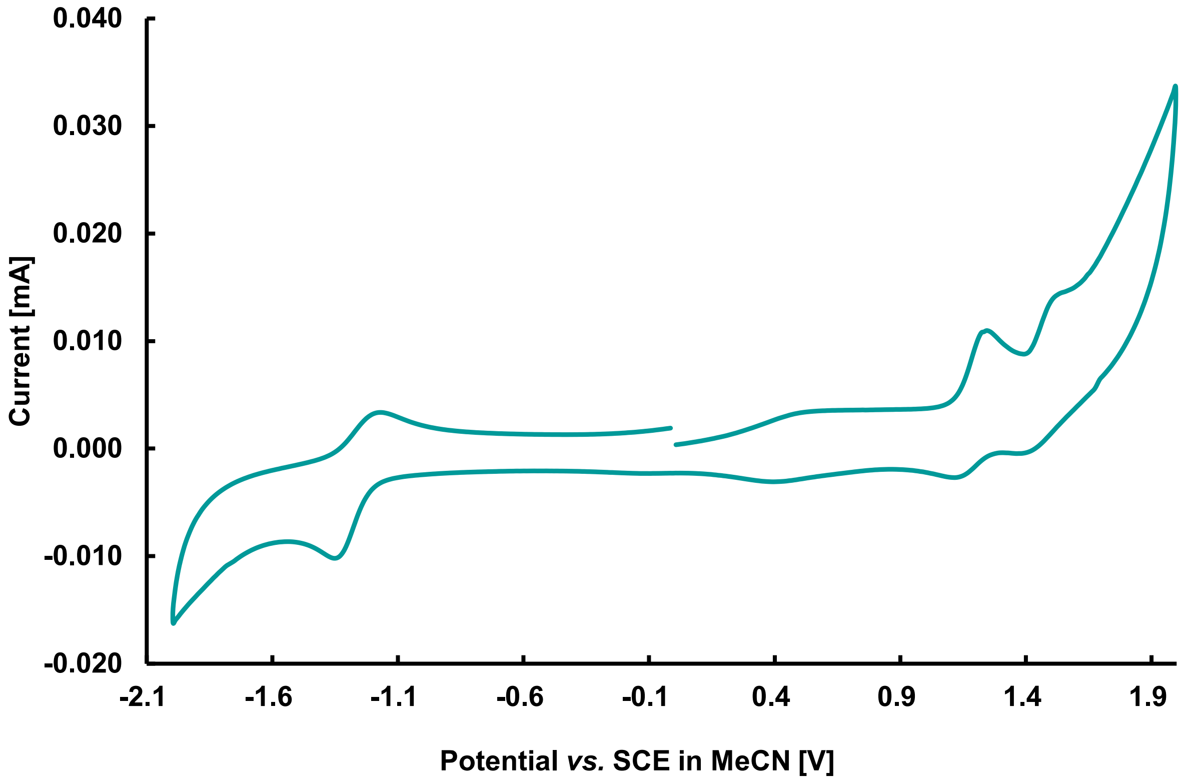

Figure S199. Cyclic voltammetry of **PC34** in degassed MeCN (0.4 mM) using 0.1 M $^{n}Bu_4NPF_6$ as electrolyte.

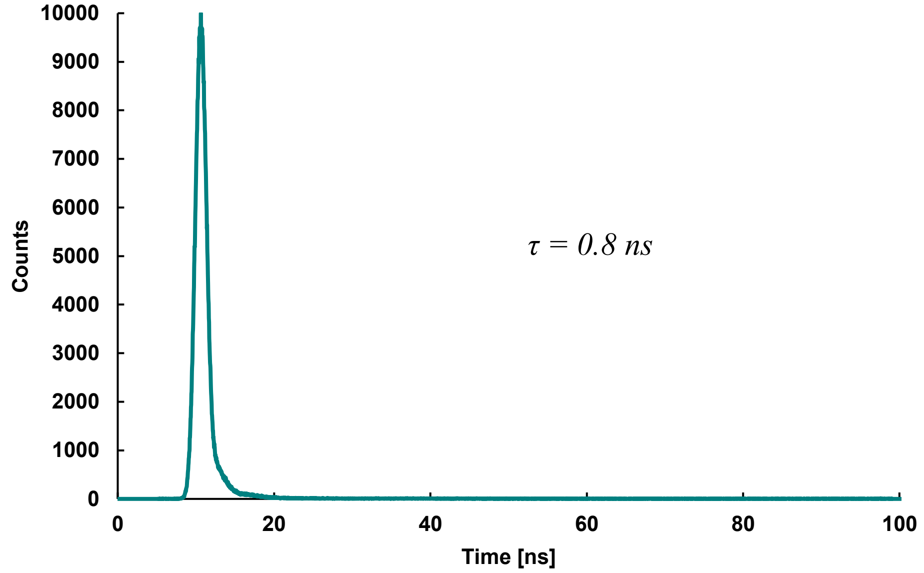

Figure S200. Fluorescence decay curve of **PC34** in degassed MeCN (20 µM). Excitation wavelength – 394 nm.

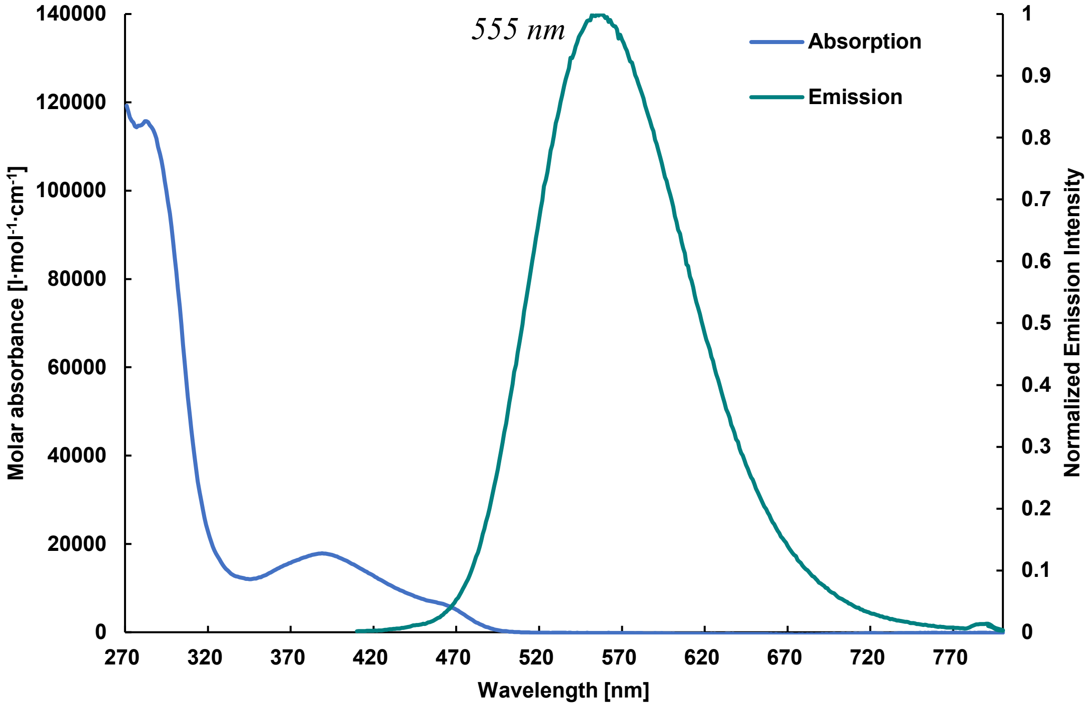

Figure S201. UV/Vis absorption and emission spectrum of **PC35** in degassed MeCN (20 µM). Excitation wavelength – 394 nm.

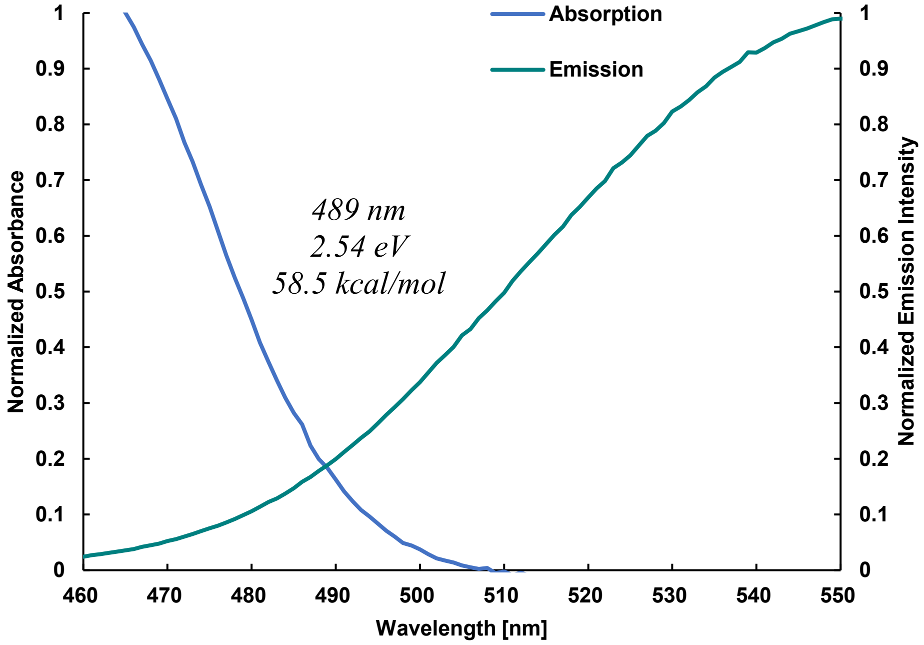

Figure S202. $E_{0\text{-}0}$ estimation at normalized emission and absorption overlap of **PC35**.

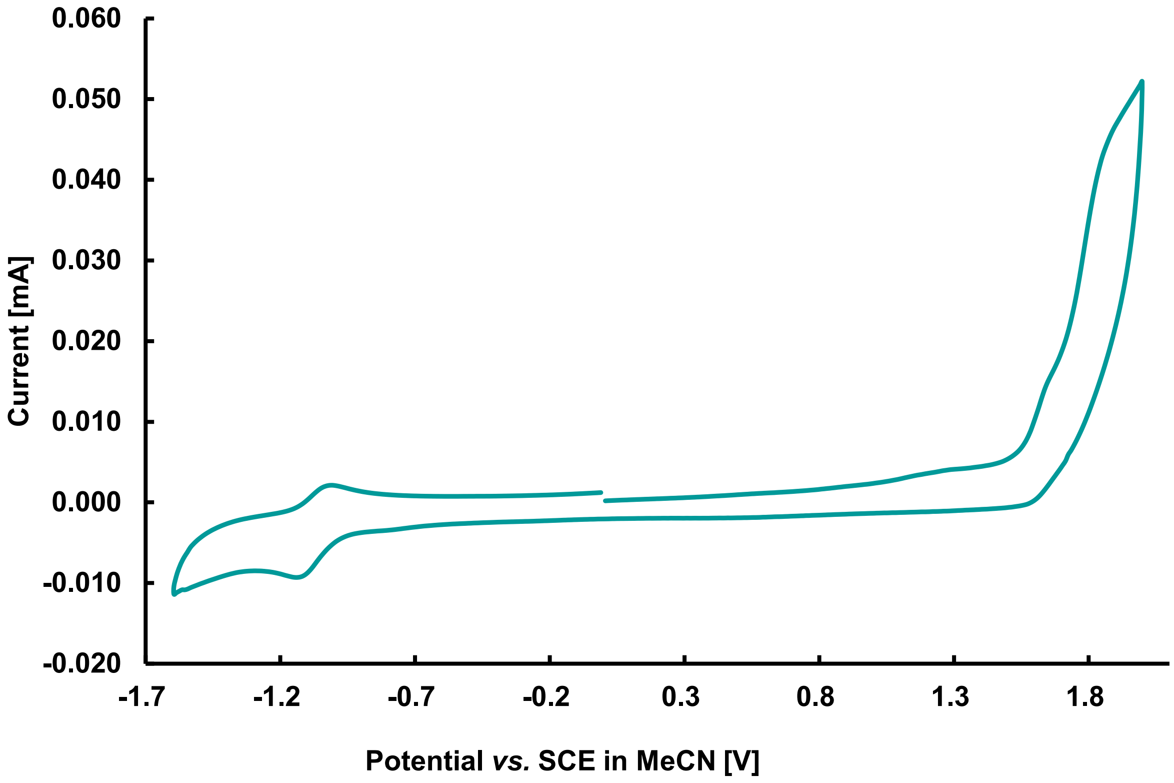

Figure S203. Cyclic voltammetry of **PC35** in degassed MeCN (0.4 mM) using 0.1 M $^{n}Bu_4NPF_6$ as electrolyte.

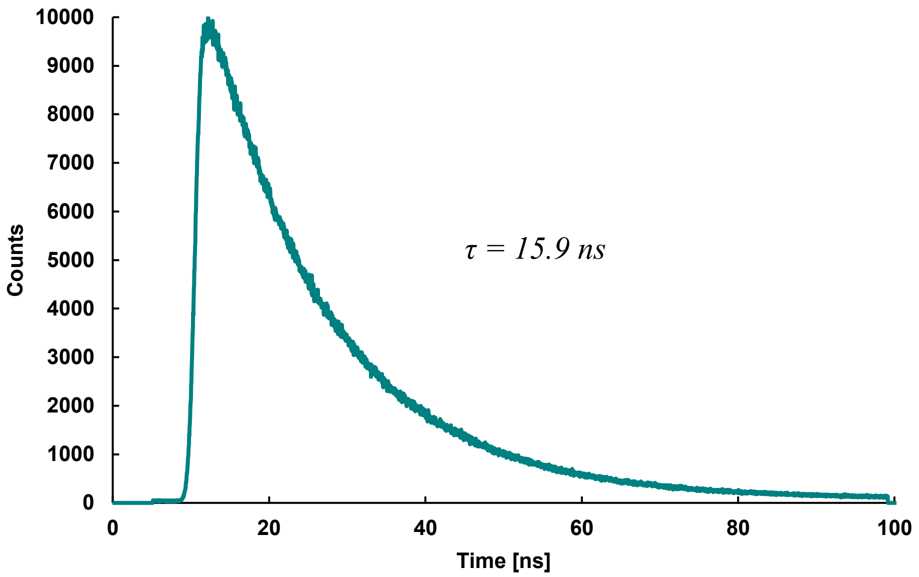

Figure S204. Fluorescence decay curve of **PC35** in degassed MeCN (20 µM). Excitation wavelength – 394 nm.

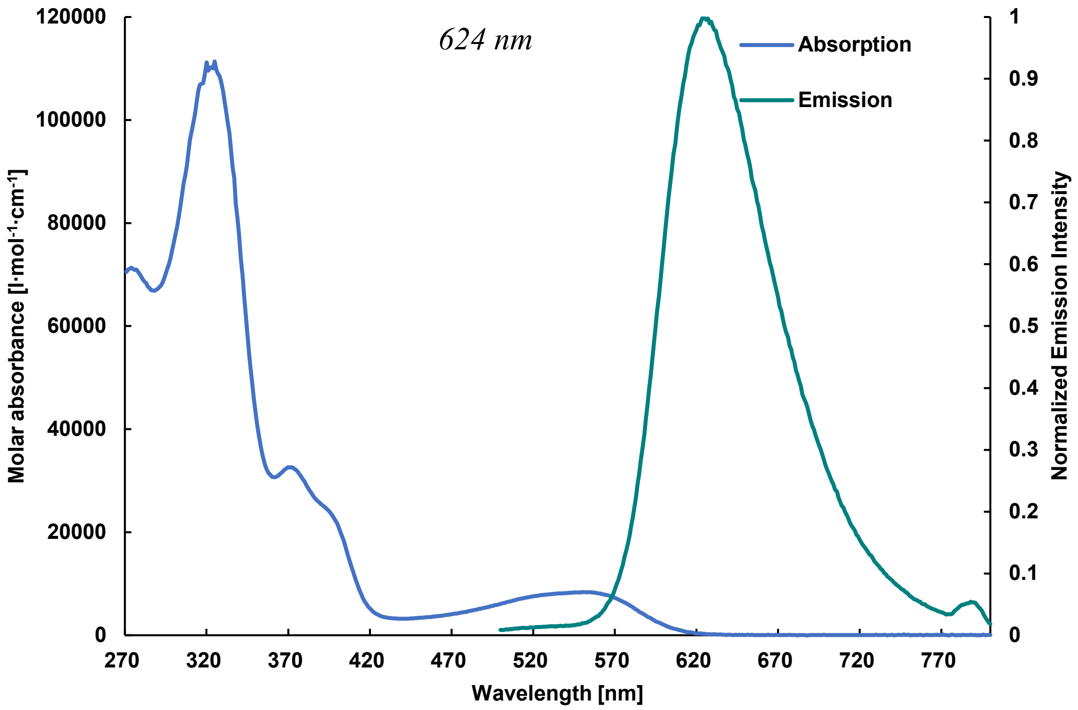

Figure S205. UV/Vis absorption and emission spectrum of **PC36** in degassed DCM (20 μM). Excitation wavelength – 394 nm.

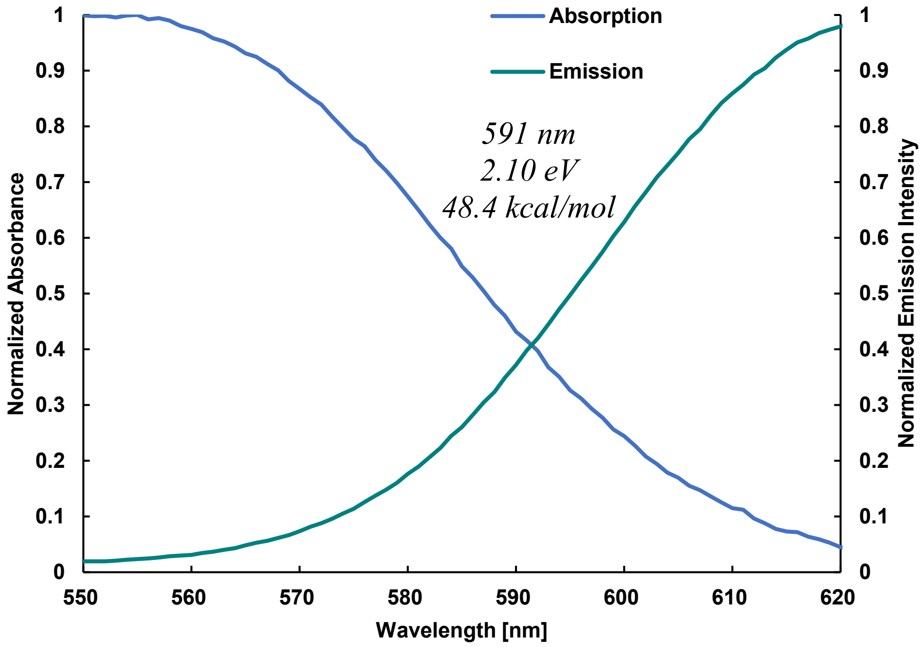

Figure S206. $E_{0\text{-}0}$ estimation at normalized emission and absorption overlap of **PC36**.

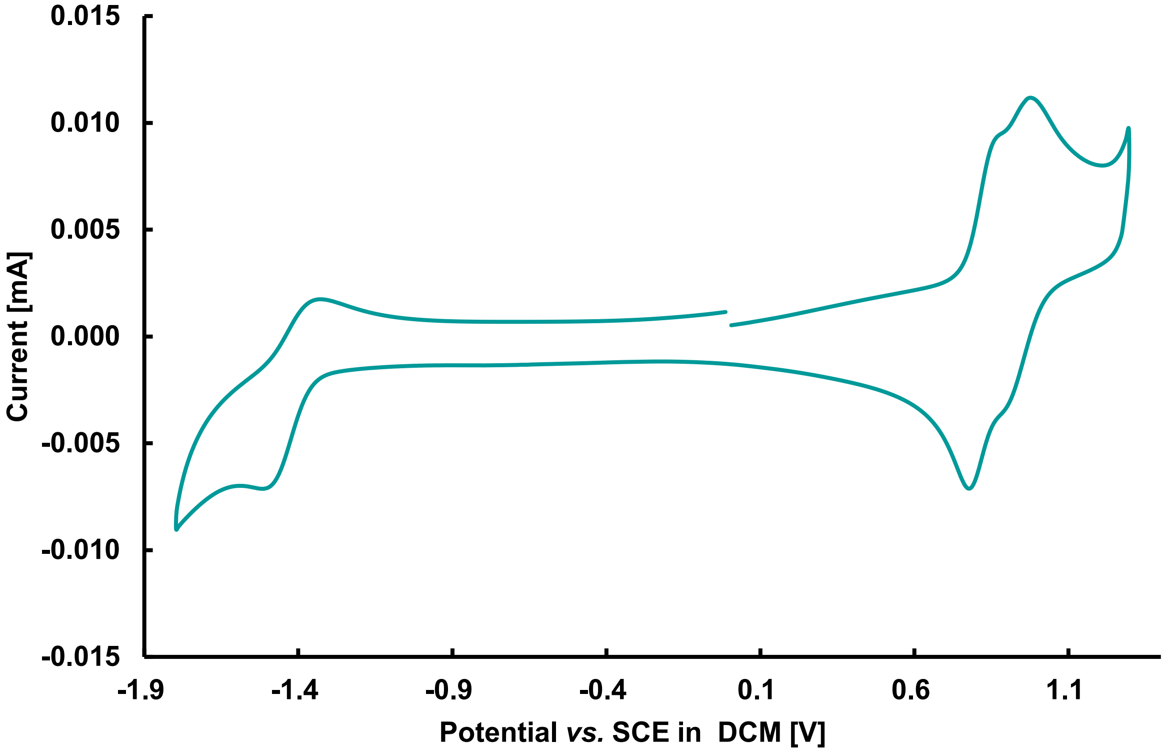

Figure S207. Cyclic voltammetry of **PC36** in degassed DCM (0.4 mM) using 0.1 M $^{n}Bu_4NPF_6$ as electrolyte.

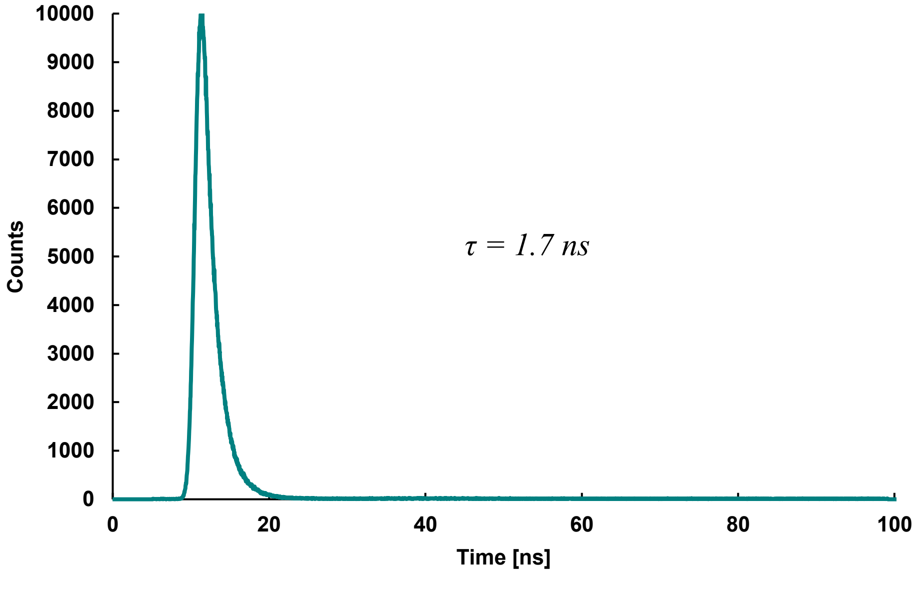

Figure S208. Fluorescence decay curve of **PC36** in degassed DCM (20 μM). Excitation wavelength – 394 nm.

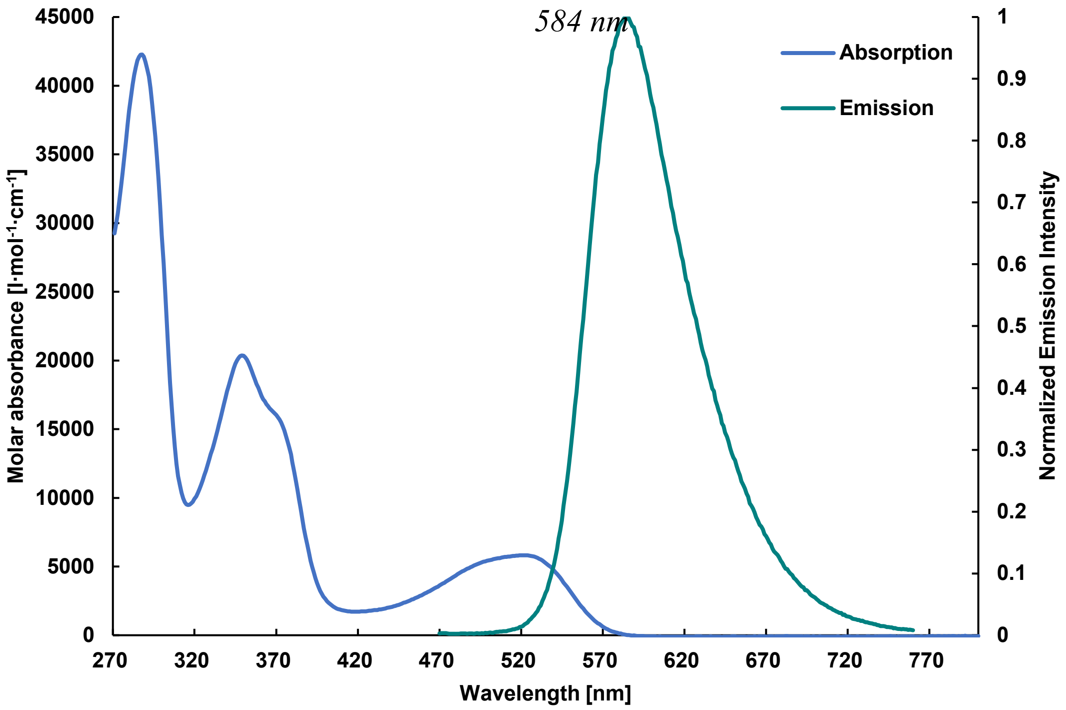

Figure S209. UV/Vis absorption and emission spectrum of **PC37** in degassed DCM (20 μM). Excitation wavelength – 457 nm.

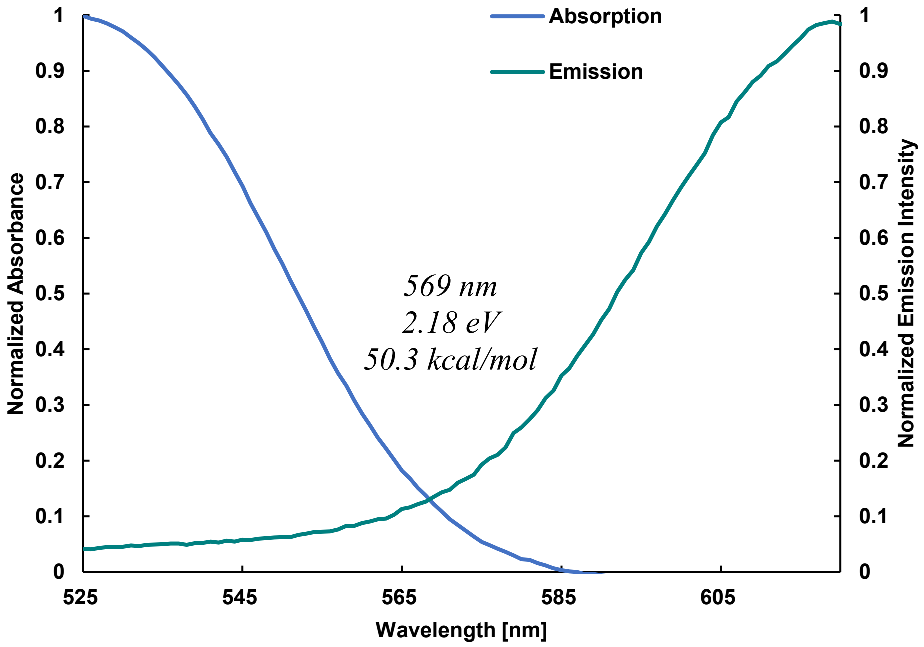

Figure S210. $E_{0\text{-}0}$ estimation at normalized emission and absorption overlap of **PC37**.

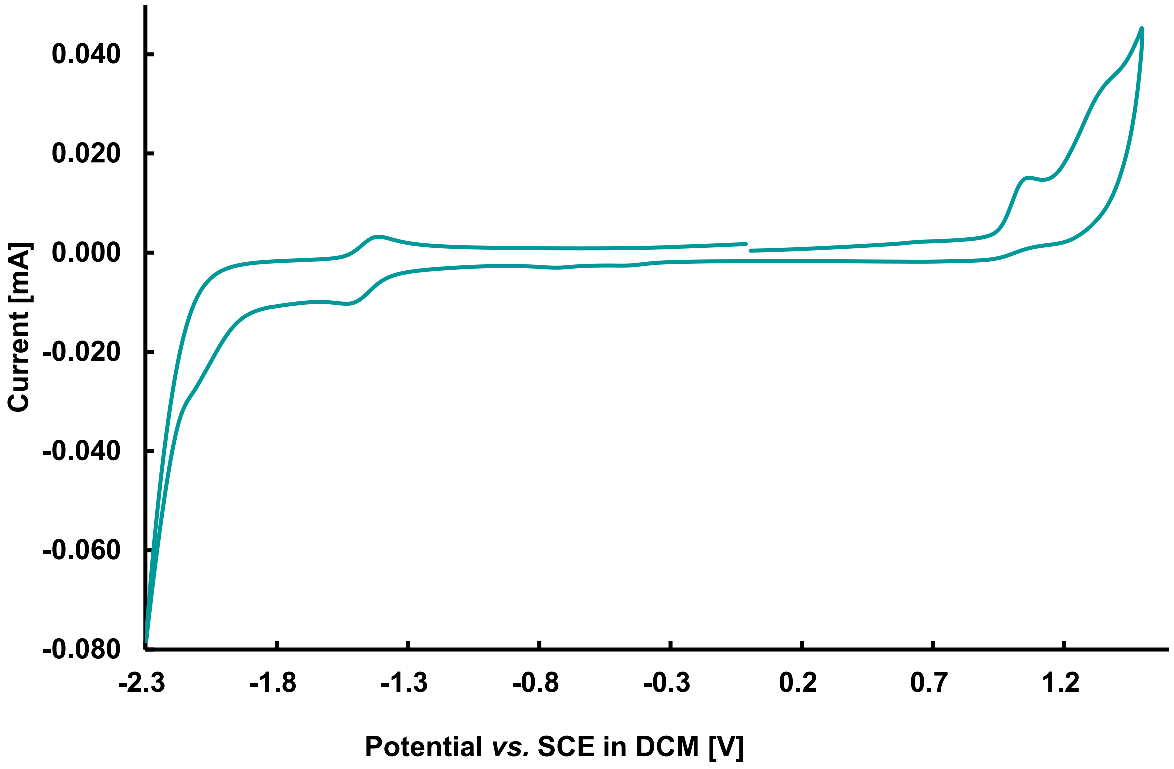

Figure S211. Cyclic voltammetry of **PC37** in degassed DCM (0.4 mM) using 0.1 M $^{n}Bu_4NPF_6$ as electrolyte.

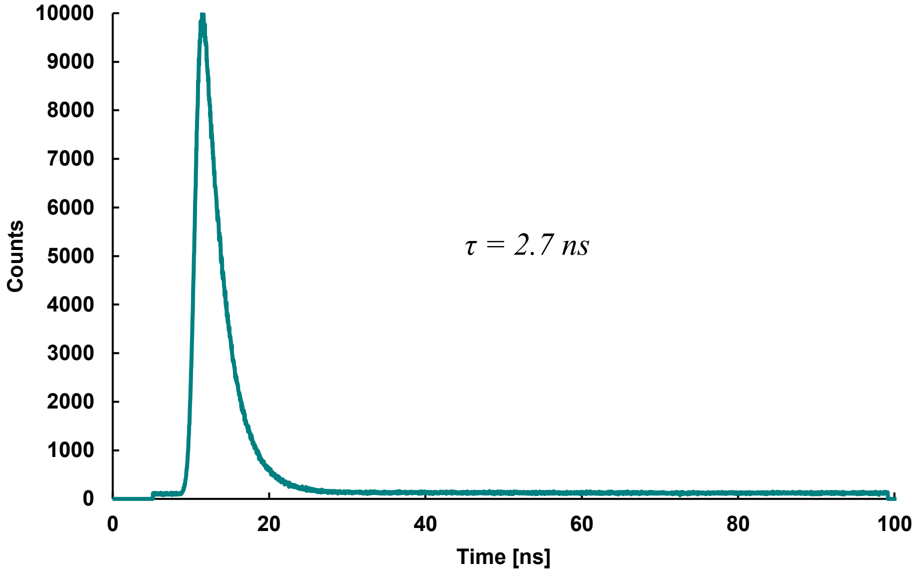

Figure S212. Fluorescence decay curve of **PC37** in degassed DCM (20 µM). Excitation wavelength – 394 nm.

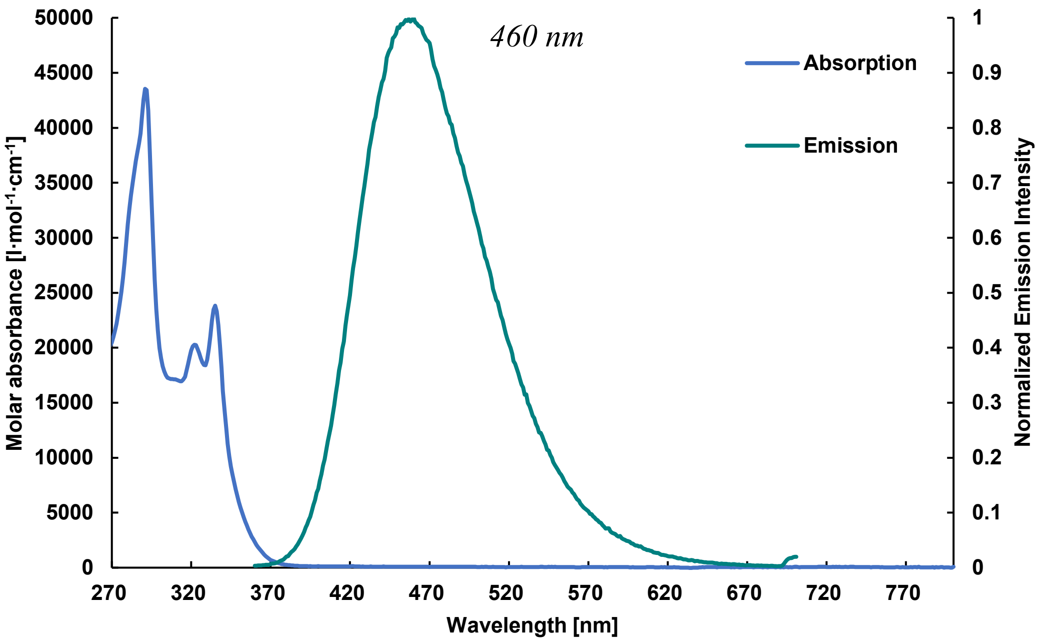

Figure S213. UV/Vis absorption and emission spectrum of **PC38** in degassed MeCN (20 µM). Excitation wavelength – 370 nm.

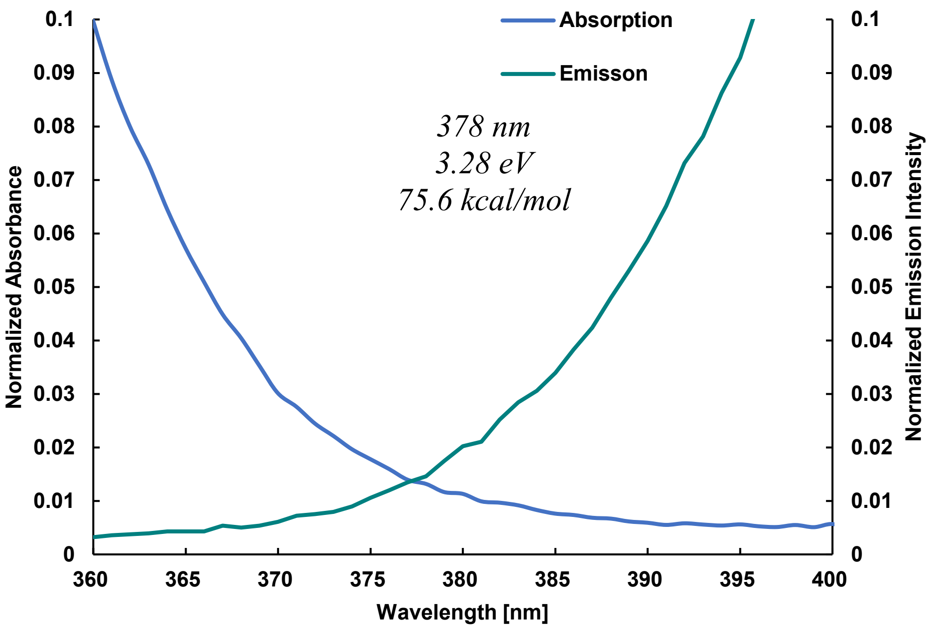

Figure S214. $E_{0\text{-}0}$ estimation at normalized emission and absorption overlap of **PC38**.

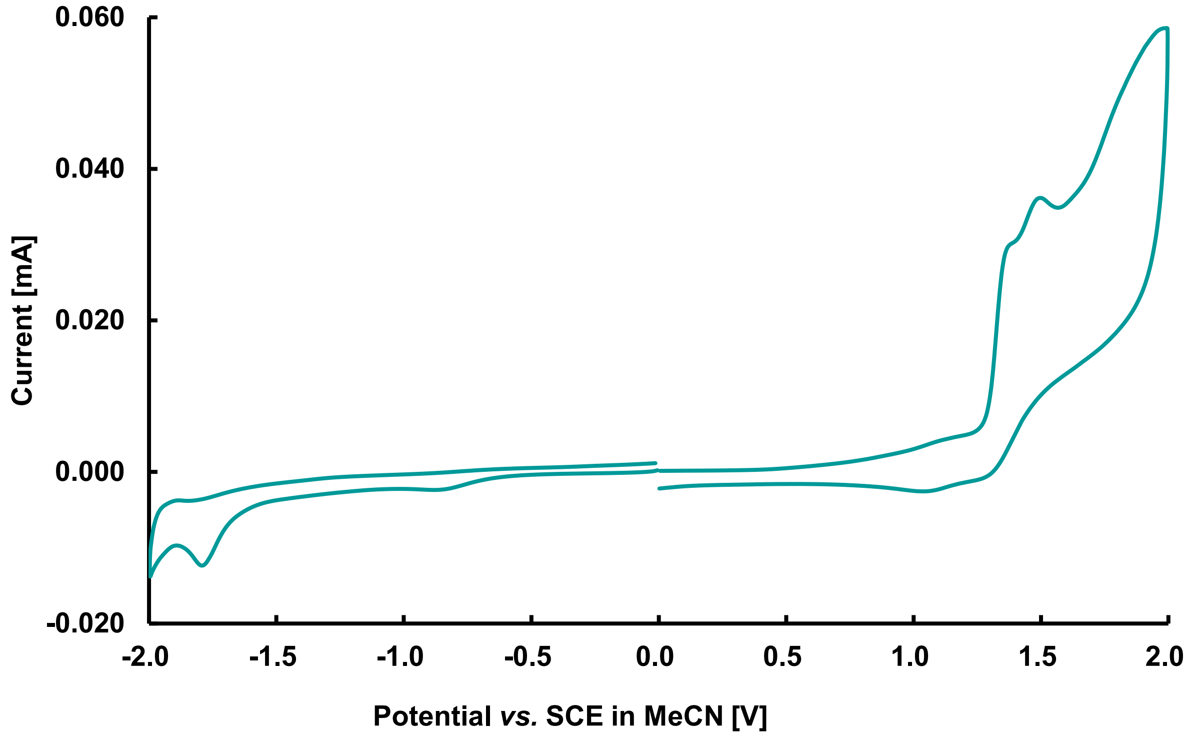

Figure S215. Cyclic voltammetry of **PC38** in degassed MeCN (0.4 mM) using 0.1 M $^{n}Bu_4NPF_6$ as electrolyte.

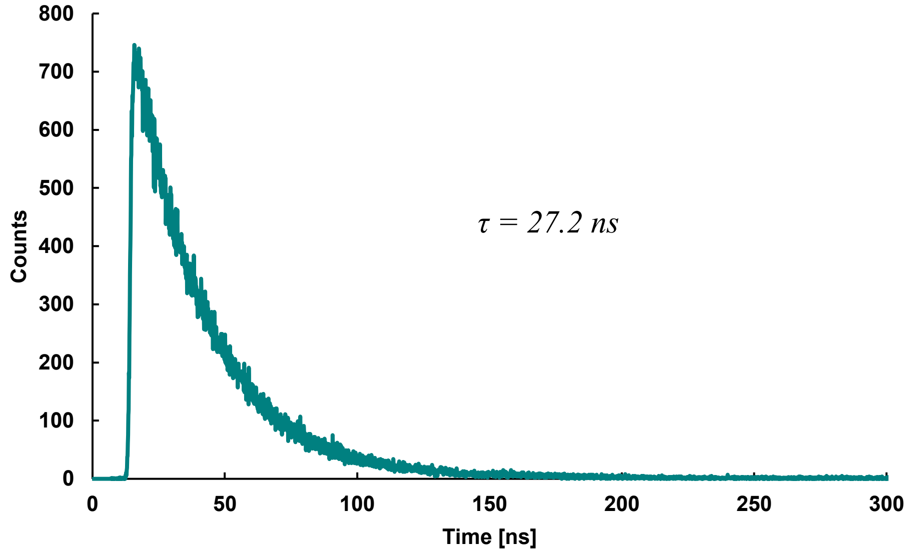

Figure S216. Fluorescence decay curve of **PC38** in degassed MeCN (20 µM). Excitation wavelength – 370 nm.

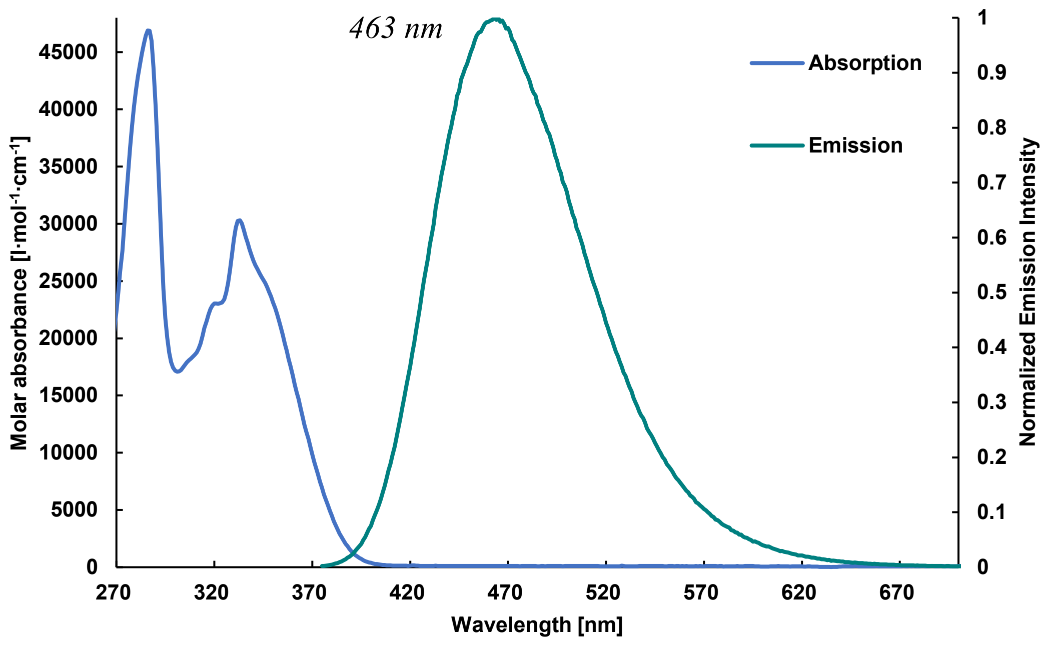

Figure S217. UV/Vis absorption and emission spectrum of **PC39** in degassed MeCN (20 µM). Excitation wavelength – 370 nm.

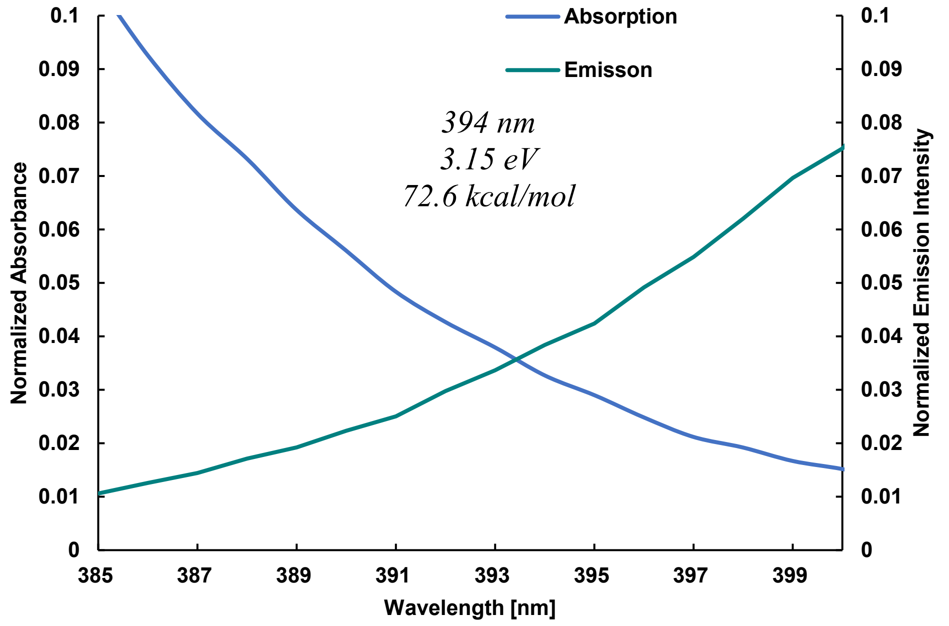

Figure S218. $E_{0\text{-}0}$ estimation at normalized emission and absorption overlap of **PC39**.

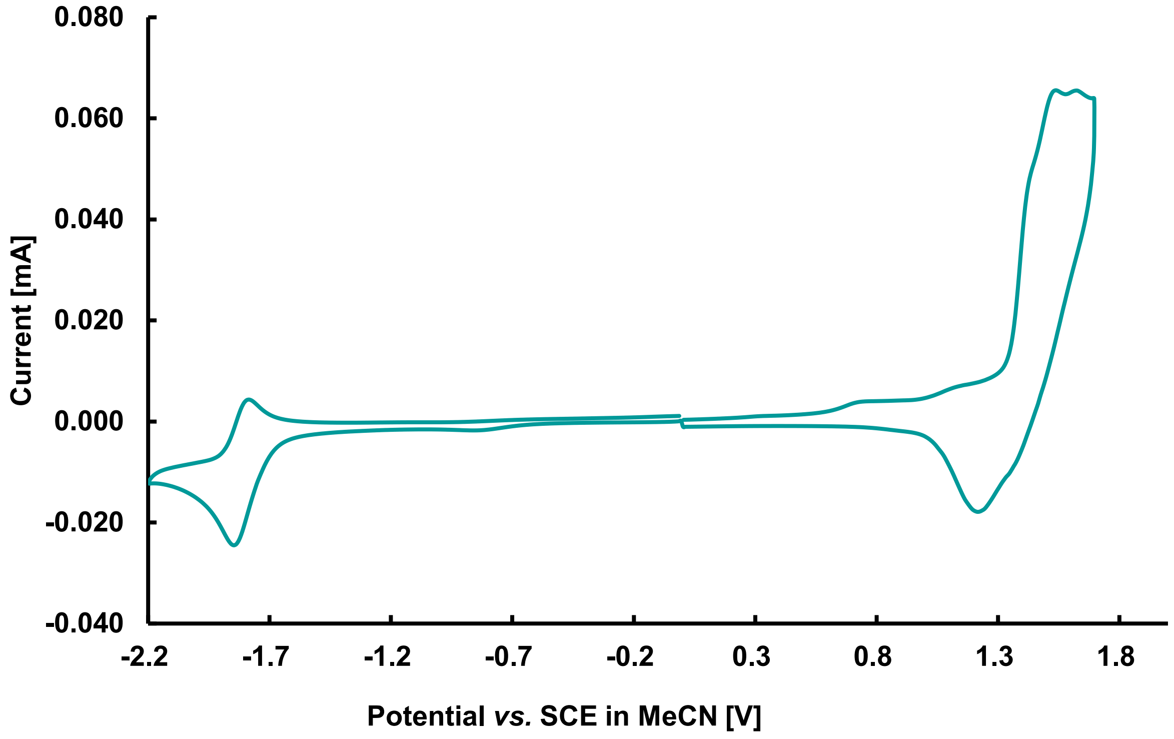

Figure S219. Cyclic voltammetry of **PC39** in degassed MeCN (0.4 mM) using 0.1 M $^{n}Bu_4NPF_6$ as electrolyte.

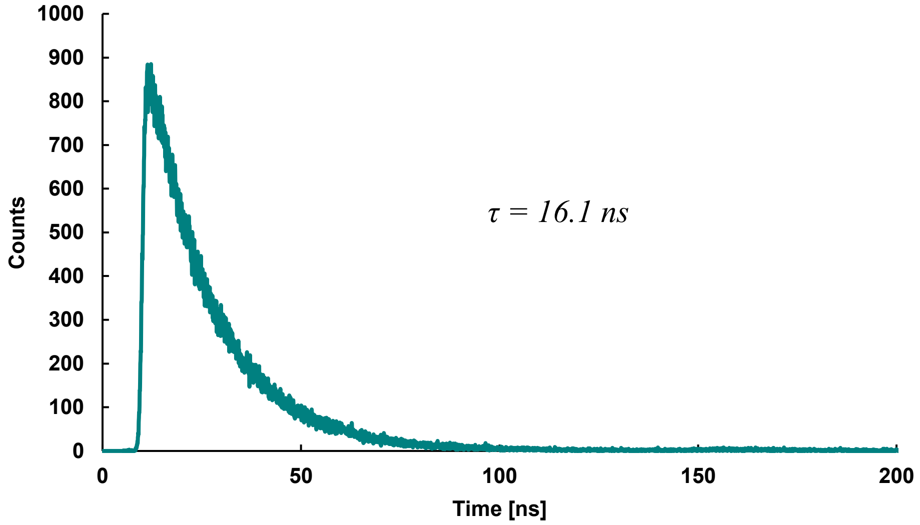

Figure S220. Fluorescence decay curve of **PC39** in degassed MeCN (20 μM). Excitation wavelength – 370 nm.

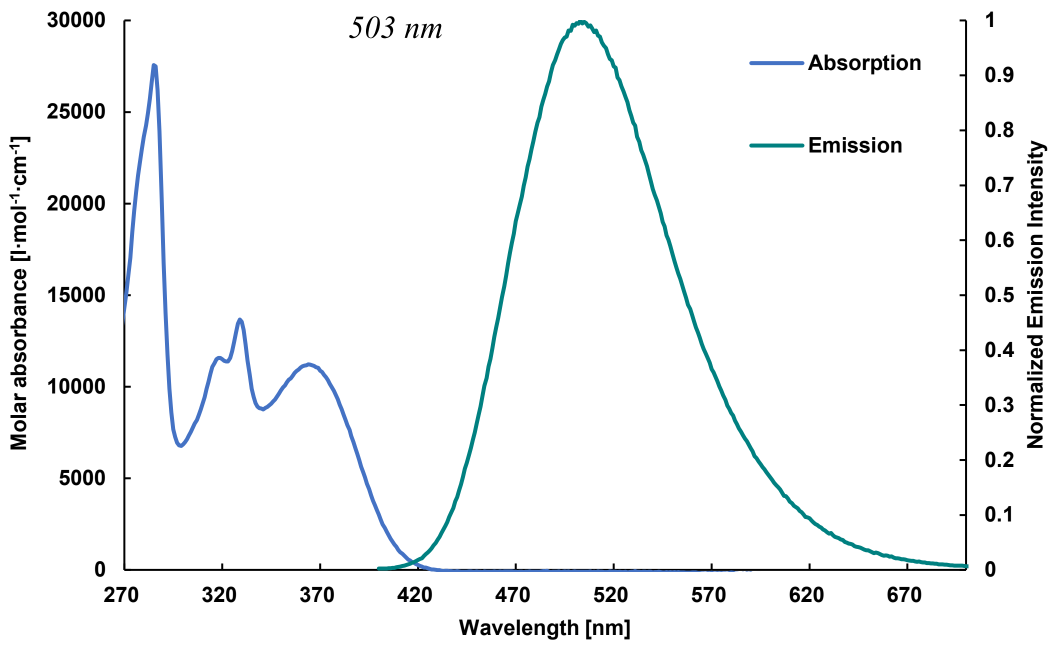

Figure S221. UV/Vis absorption and emission spectrum of **PC40** in degassed MeCN (20 µM). Excitation wavelength – 394 nm.

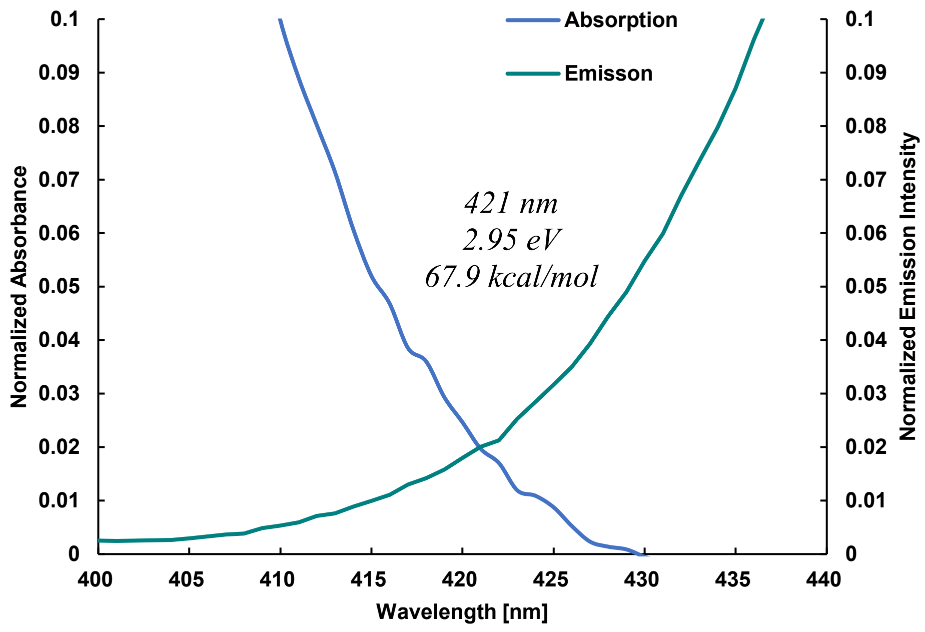

Figure S222. ***E*₀₋₀** estimation at normalized emission and absorption overlap of **PC40**.

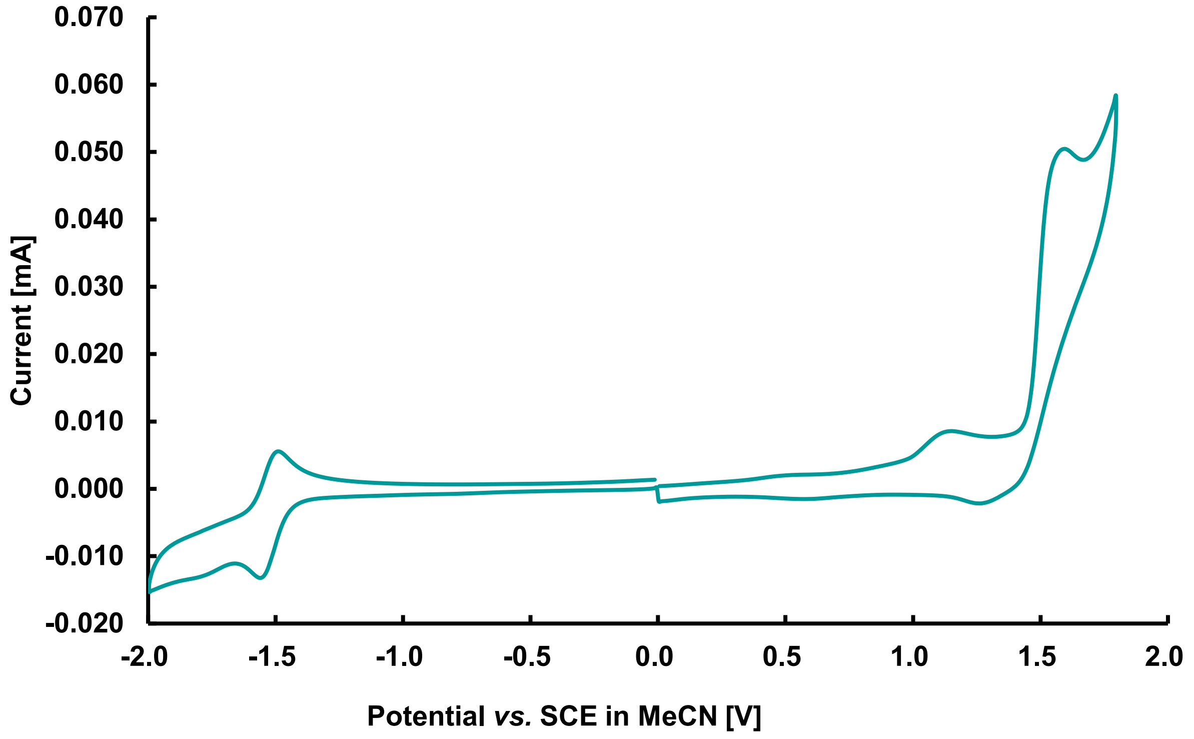

Figure S223. Cyclic voltammetry of **PC40** in degassed MeCN (0.4 mM) using 0.1 M $^{n}Bu_4NPF_6$ as electrolyte.

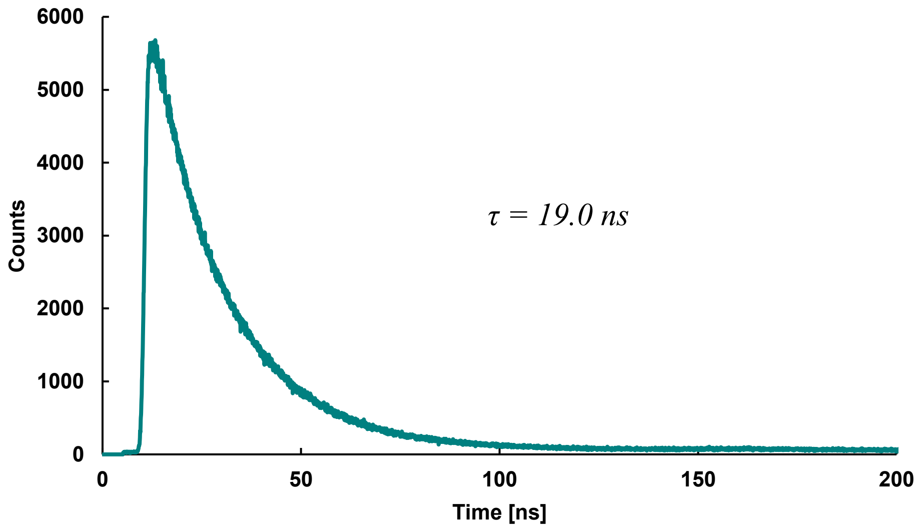

Figure S224. Fluorescence decay curve of **PC40** in degassed MeCN (20 µM). Excitation wavelength – 394 nm.

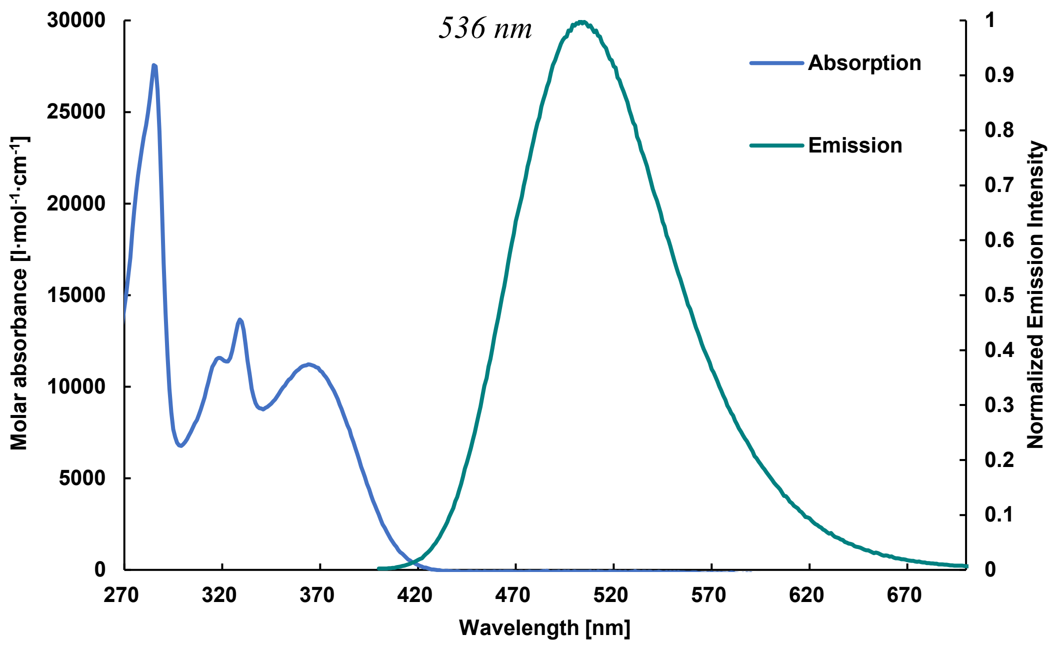

Figure S225. UV/Vis absorption and emission spectrum of **PC41** in degassed MeCN (20 µM). Excitation wavelength – 394 nm.

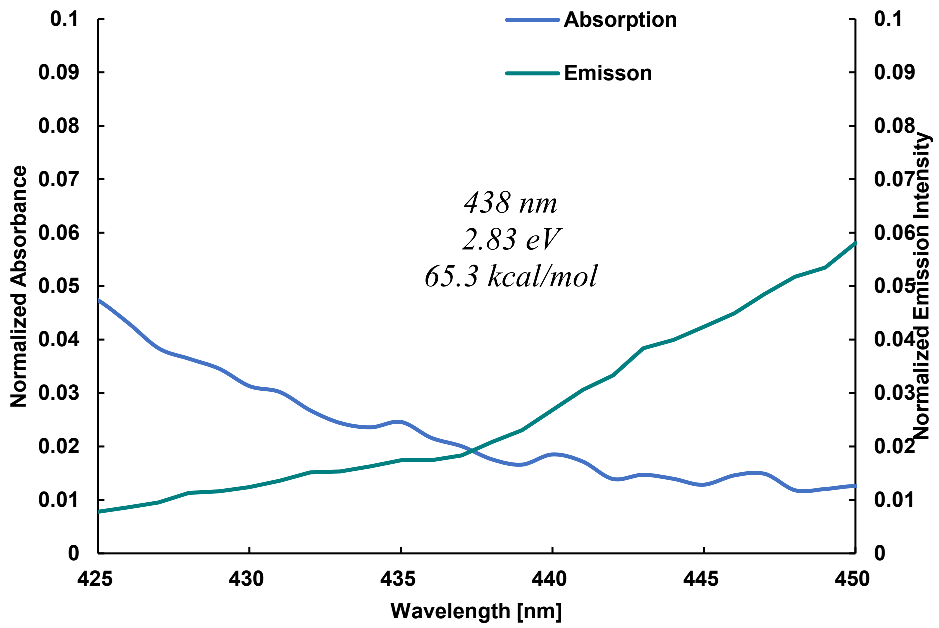

Figure S226. $E_{0\text{-}0}$ estimation at normalized emission and absorption overlap of **PC41**.

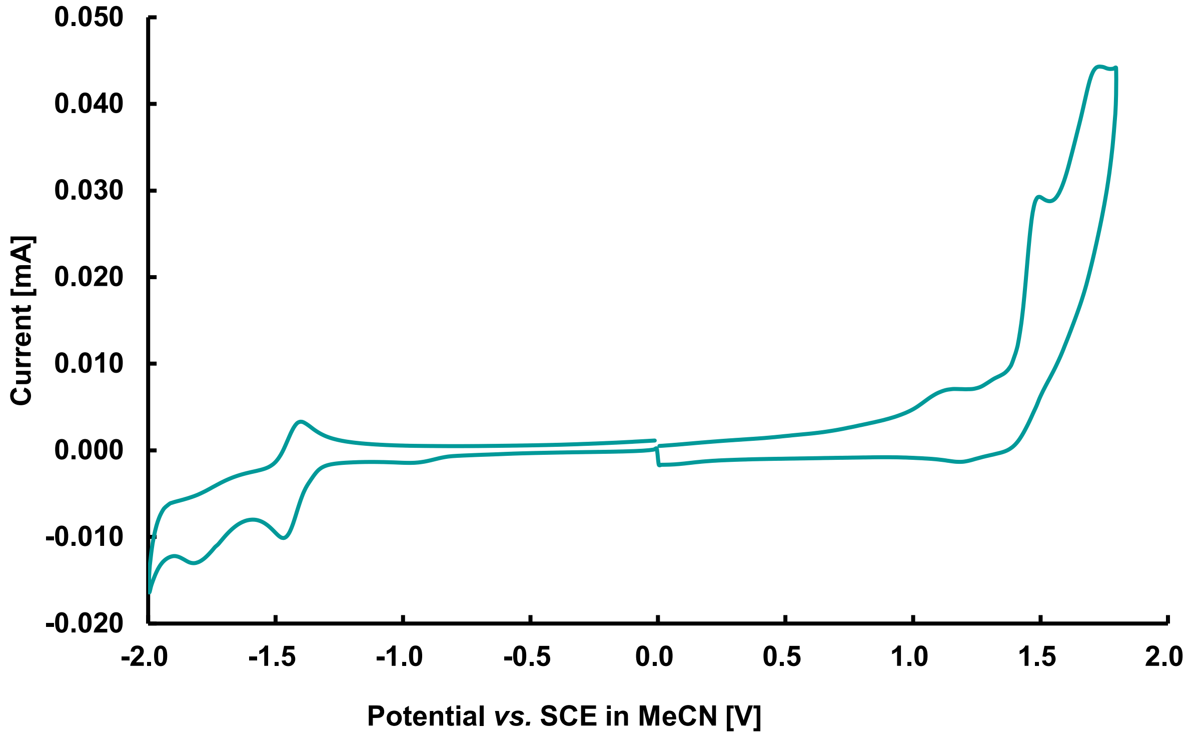

Figure S227. Cyclic voltammetry of **PC41** in degassed MeCN (0.4 mM) using 0.1 M $^{n}Bu_4NPF_6$ as electrolyte.

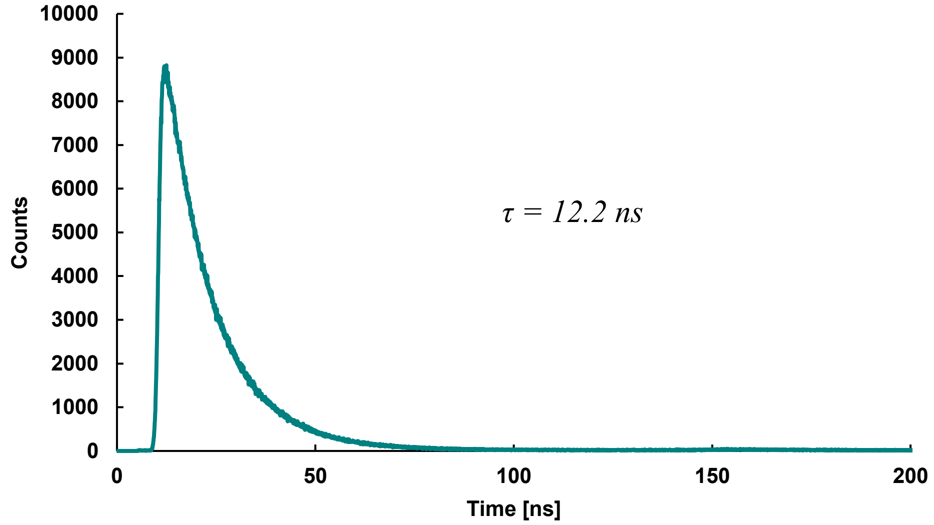

Figure S228. Fluorescence decay curve of **PC41** in degassed MeCN (20 μM). Excitation wavelength – 394 nm.

3. Optimization of the catalytic reaction conditions

Table S13. Optimization and Control Reactions for cross-coupling of 4-bromo-1,1'-biphenyl with piperidine.

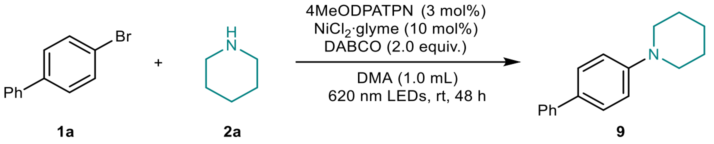

Entry	variation from the conditions above	Yield [%][a]
1	none	89 (87[b])
2[c]	DMSO as solvent	68
3[c]	DMF as solvent	70
4[c]	NMP as solvent	78
5[c]	DMA	80
6	1.5 equiv. of piperidine	54
7	1 mol% 4MeODPATPN	85
8	Air instead of Ar	14
9	W/O DABCO	84
10	W/O PC	ND
11	W/O Ni	ND
12	W/O light	ND

[a]Reactions were performed with 0.2 mmol of 4-bromo-1,1'-biphenyl, 0.6 mmol of piperidine, 3 mol% 4MeODPATPN photocatalyst, 10 mol% $NiCl_2$·glyme, 2.0 equiv. DABCO, 1mL DMA, 620 nm LEDs, rt, 48 h. Yields were determined by GC-FID using dodecane as the internal standard. [b]Isolated yield. [c]40 h. Ar, Argon. ND, not detected.

Table S14. Optimization for cross-coupling of aryl 1-bromo-4-(trifluoromethyl)benzene with 4-methoxyaniline.

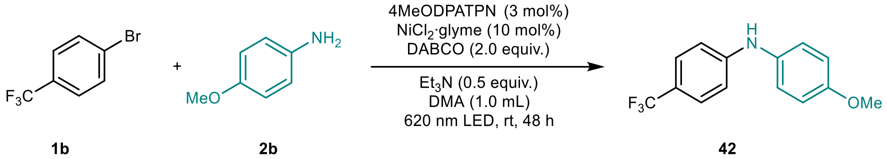

Entry	variation from the conditions above	Yield [%][a]
1	none	88 (86[b])
2	1.5 equiv. aniline	82
3[c]	0.1 equiv. pyrrolidine instead of 0.5 equiv. Et_3N	64
4[cd]	1.3 equiv. cyclohexylamine instead of 2 equiv. DABCO	82
5	W/O Et_3N	54
6	W/O DABCO	38
7	W/O PC	ND
8	W/O Ni	ND
9	W/O DABCO, W/O Et_3N	5
10	W/O light	ND

[a]Reactions were performed with 0.2 mmol of 1-bromo-4-(trifluoromethyl)benzene, 0.6 mmol of 4-methoxyaniline, 3 mol% 4MeODPATPN photocatalyst, 10 mol% $NiCl_2$·glyme, 2.0 equiv. DABCO, 0.5 equiv. Et_3N, 1mL DMA, 620 nm LEDs, rt, 48 h. Yields were determined by GC-FID using dodecane as the internal standard. [b]Isolated yield. [c]Without Et_3N. [d]2 equiv. 4-methoxyaniline. ND, not detected.

Table S15 and S16. Optimization for cross-coupling of 1-bromo-4-(trifluoromethyl)benzene with 4-methylbenzenesulfonamide.

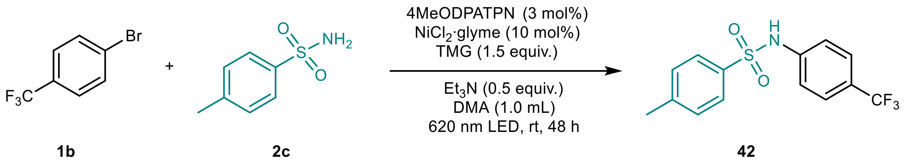

Entry	variation from the conditions above	Yield [%][a]
1	none	47
2	0.5 mL DMA	44
3	BTMG instead of TMG	50
4	cyclohexylamine instead of TMG	76
5	cyclohexylamine instead of TMG, W/O Et_3N	64
6[c]	cyclohexylamine instead of TMG, 0.5 mL DMA	66

[a]Reactions were performed with 0.2 mmol of 1-bromo-4-(trifluoromethyl)benzene, 0.4 mmol of 4-methylbenzenesulfonamide, 3 mol% 4MeODPATPN photocatalyst, 10 mol% $NiCl_2$·glyme, 1.5 equiv. TMG and 0.5 equiv. Et_3N, 1mL DMA, 620 nm LEDs, rt, 48 h. Yields were determined by GC-FID using dodecane as the internal standard. [b]Isolated yield. [c]Without Et_3N. ND, not detected.

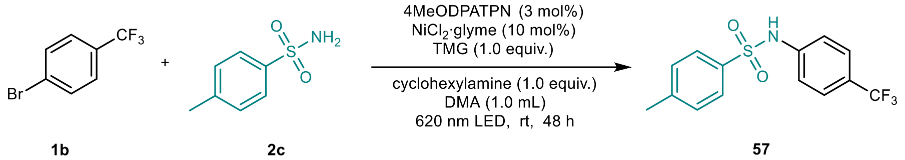

Entry	variation from the conditions above	Yield [%][a]
1	none	87 (85[b])
2	W/O PC	ND
3	W/O Ni	ND
4	W/O light	ND
5	W/O TMG, W/O cyclohexylamine	ND

[a]Reactions were performed with 0.2 mmol of 1-bromo-4-(trifluoromethyl)benzene, 0.6 mmol of 4-methylbenzenesulfonamide, 3 mol% 4MeODPATPN photocatalyst, 10 mol% $NiCl_2$·glyme, 1.0 equiv. TMG and 1.0 equiv. cyclohexylamine, 1 mL DMA, 620 nm LEDs, rt, 48 h. Yields were determined by GC-FID using dodecane as the internal standard. ND, not detected.

Table S17. Optimization for cross-coupling of 1-bromo-4-(trifluoromethyl)benzene with benzamides.

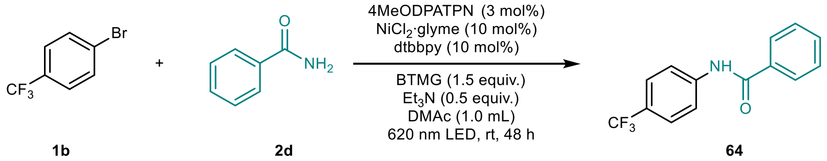

Entry	variation from the conditions above	Yield [%][a]
1	none	65 (61[b])
2[c]	TMG instead of BTMG	21
3[cd]	1.0 equiv. TMG and 1.0 equiv. cyclohexylamine	14
4[c]	TMG instead of BTMG, 65 h	26
5[c]	added 0.5 equiv. TMG	30
6	W/O dtbbpy	ND
7	W/O BTMG + Et_3N	ND
8	W/O Et_3N	46
9[e]	W/O BTMG	ND
10	W/O PC	ND
11	W/O Ni	ND
12	W/O light	ND

[a]Reactions were performed with 0.2 mmol of 1-bromo-4-(trifluoromethyl)benzene, 0.6 mmol of benzamide, 3 mol% 4MeODPATPN photocatalyst, 10 mol% $NiCl_2$·glyme, 10 mol% dtbbpy, 1.5 equiv. BTMG and 0.5 equiv. Et_3N, 1mL

DMA, 620 nm LEDs, rt, 48 h. Yields were determined by GC-FID using dodecane as the internal standard. [b]Isolated yield. [c]Without dtbbpy. [d]Without BTMG, without Et_3N. [e]Without Et_3N. ND, not detected.

Table S18. Optimization and Control Reactions for cross-coupling of 4-bromo-1,1'-biphenyl with benzenethiol.

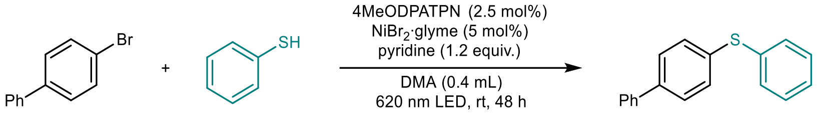

1a 2e 88

Entry	variation from the conditions above	Yield [%][a]
1	none	82 (78 [b])
2	DABCO instead of pyridine	3
3	TMG instead of pyridine	3
4	cyclohexylamine instead of pyridine	5
5	Cs_2CO_3 instead of pyridine	4
6	10 mol% of $NiBr_2$·glyme	80
7	10 mol% of $NiCl_2$·glyme	21
8[c]	10 mol% of $NiCl_2$·glyme	5
9[d]	2.0 equiv. benzenethiol	56
10	640 nm instead of 620 nm	69
11	W/O pyridine	6
12	W/O PC	2
13	W/O Ni	ND
14[d]	W/O light	ND

[a]Reactions were performed with 0.2 mmol of 4-bromo-1,1'-biphenyl, 0.3 mmol of benzenethiol, 2.5 mol% 4MeODPATPN photocatalyst, 5 mol% $NiBr_2$·glyme, 1.2 equiv. pyridine, 0.4 mL DMA, 620 nm LEDs, rt, 48 h. Yields were determined by GC-FID using dodecane as the internal standard. [b]Isolated yield. [c]2.0 equiv. benzenethiol. [d]10 mol% $NiBr_2$·glyme. [e]Dark, 60 ℃. ND, not detected.

Table S19. Optimization and Control Reactions for cross-coupling of 4-bromo-1,1'-biphenyl with sodium 4-methylbenzenesulfinate.

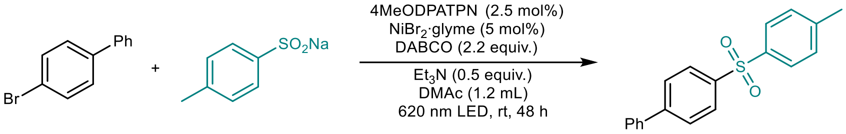

1a 2f 97

Entry	variation from the conditions above	Yield [%][a]
1	none	89 (83 [b])
2	W/O PC	ND
3	W/O Ni	ND
4[c]	W/O light	ND
5	W/O DABCO	ND
6	W/O Et_3N	60

[a]Reactions were performed with 0.2 mmol of 4-bromo-1,1'-biphenyl, 0.36 mmol of sodium 4-methylbenzenesulfinate, 2.5 mol% 4MeODPATPN photocatalyst, 5 mol% $NiBr_2$·glyme, 2.2 equiv. DABCO, 0.5 equiv. Et_3N, 1.2 mL DMA, 620 nm LEDs, rt, 48 h. Yields were determined by GC-FID using dodecane as the internal standard. [b]Isolated yield. [c]Dark, 35 ℃. ND, not detected.

Table S20. Various electron-rich elecrophiles for cross-coupling of 1-bromo-4-methoxybenzene with piperidine.

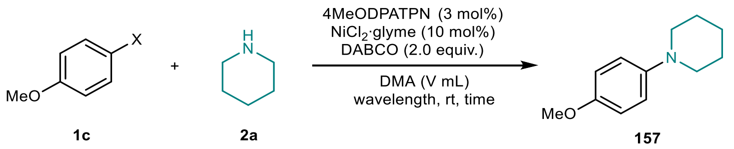

Entry	X =	V, mL	Wavelength, nm	Yield [%][a]
1	Br	1.0	620	32 [b]
2	I	1.0	620	90 [b]
3	TT[d], BF_4^-	2.0	620	80 [b]
4	TT[d], BF_4^-	2.0	456	71[c]

[a]Yields were determined by GC-FID using dodecane as the internal standard. [b]Reactions were performed with 0.2 mmol of **1c**, 0.6 mmol of piperidine, 3 mol% 4MeODPATPN photocatalyst, 10 mol% $NiCl_2$·glyme, 2.0 equiv. DABCO, 1-2 mL DMA, 620 nm LEDs, rt, 48 h under Ar atmosphere. [c]Reaction was performed with 0.2 mmol of **1c**, 0.6 mmol of piperidine, 10 mol% $NiCl_2$·glyme, 2 mL DMA, 2x456 nm Kessil lamps, rt, 24 h under Ar atmosphere. [d]TT = arylthianthrenium salt with BF_4^- as counter-anion.

The entry 4 was adapted from ref[9].

4. Experimental procedures for catalytic reactions

4.1. General procedures

General Procedure A

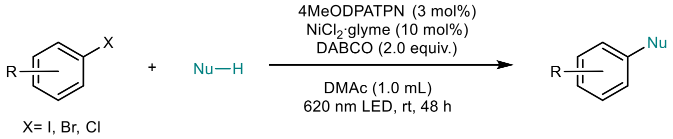

A dry 4-mL vial equipped with a Teflon-coated magnetic stir bar was charged with aryl bromide (0.2 mmol, 1 equiv., if solid), the respective coupling partner (0.6 mmol, 3 equiv., if solid), $NiCl_2$·glyme (4.4 mg, 0.02 mmol, 10 mol %), DABCO (44.9 mg, 0.4 mmol, 2.0 equiv.) and 4MeODPATPN (6.2 mg, 0.006 mmol, 0.03 equiv.). The vial was introduced to a glovebox filled with argon atmosphere. Then anhydrous and degassed DMA (1.0 mL), aryl bromide (0.2 mmol, 1 equiv., if liquid) and the respective coupling partner (0.6 mmol, 3 equiv.) were added. The vial was capped with Teflon septum and removed from the glovebox. Then, the reaction mixture was stirred at room temperature for 48 h under irradiation with 620 nm LED lamps (AL3 parallel photoreactor, set up 15W) with fan cooling. Upon completion, brine (20 mL) was added and the mixture was extracted with EtOAc (20 mL) three times. The combined organic layer was washed with H_2O (20 mL) and brine (20 mL). The organic layer was dried with anhydrous Na_2SO_4, then concentrated under vacuum. The product was purified by flash column chromatography on silica gel using hexane: EtOAc as eluent.

General Procedure B

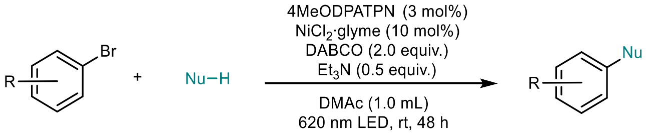

A dry 4-mL vial equipped with a Teflon-coated magnetic stir bar was charged with aryl bromide (0.2 mmol, 1 equiv., if solid), the respective coupling partner (0.6 mmol, 3 equiv., if solid), $NiCl_2$·glyme (4.4 mg, 0.02 mmol, 10 mol %), DABCO (44.9 mg, 0.4 mmol, 2.0 equiv.) and 4MeODPATPN (6.2 mg, 0.006 mmol, 0.03 equiv.). The vial was introduced to a glovebox filled with argon atmosphere. Then anhydrous and degassed DMA (1.0 mL), aryl bromide (0.2 mmol, 1 equiv., if liquid), the respective coupling partner (0.6 mmol, 3 equiv., if liquid) and triethylamine (13.9 µL, 0.1 mmol, 0.5 equiv.) were added. The vial was capped with Teflon septum and removed from the glovebox. Then the reaction mixture was stirred at room temperature for 48 h under irradiation with 620 nm LED lamps (AL3 parallel photoreactor, set up 15 W) with fan cooling. After the reaction was completed, brine (20 mL) was added and the mixture was extracted with EtOAc (20 mL) three times. The combined organic layer was washed with H_2O (20 mL) and brine (20 mL). The organic layer was dried with anhydrous Na_2SO_4, then concentrated under vacuum. The product was purified by flash column chromatography on silica gel using hexane: EtOAc as eluent.

General Procedure C

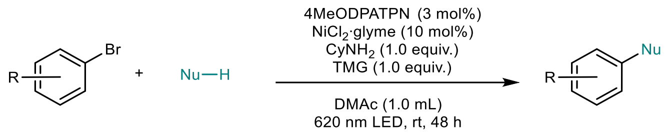

A dry 4-mL vial equipped with a Teflon-coated magnetic stir bar was charged with aryl bromide (0.2 mmol, 1 equiv., if solid), the respective coupling partner (0.6 mmol, 3 equiv., if solid), $NiCl_2$·glyme (4.4 mg, 0.02 mmol, 10 mol %) and 4MeODPATPN (6.2 mg, 0.006 mmol, 0.03 equiv.). The vial was introduced to a glovebox filled with argon atmosphere. Then anhydrous and degassed DMA (1.0 mL), aryl bromide (0.2 mmol, 1 equiv., if liquid), the respective coupling partner (0.6 mmol, 3 equiv., if liquid), 1,1,3,3-tetramethylguanidine (25.1 µL, 0.2 mmol, 1.0 equiv.) and cyclohexylamine (19.8 µL, 0.2 mmol, 1.0 equiv.) were added. The vial was capped with Teflon septum and removed from the glovebox. Then the reaction mixture was stirred at room temperature for 48 h under irradiation with 620 nm LED lamps (AL3 parallel photoreactor, set up 15W) with fan cooling. After the reaction was completed, brine (20 mL) was added and the mixture was extracted with EtOAc (20 mL) three times. The combined organic layer was washed with H_2O (20 mL) and brine (20 mL). The organic layer was dried with anhydrous Na_2SO_4, then concentrated under vacuum. The product was purified by flash column chromatography on silica gel using hexane: EtOAc as eluent.

General Procedure D

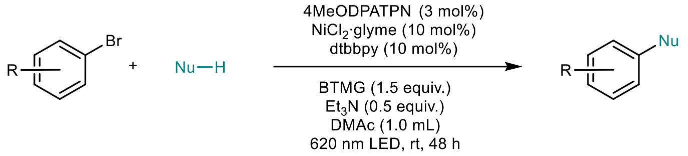

A dry 4-mL vial equipped with a Teflon-coated magnetic stir bar was charged with aryl bromide (0.2 mmol, 1 equiv., if solid), the respective coupling partner (0.6 mmol, 3 equiv., if solid), $NiCl_2$·glyme (4.4 mg, 0.02 mmol, 10 mol %), 4,4'-di-*tert*-butyl-2,2'-bipyridine (5.4 mg, 0.02 mmol, 10 mol %) and 4MeODPATPN (6.2 mg, 0.006 mmol, 0.03 equiv.). The vial was introduced to a glovebox filled with argon atmosphere. Then anhydrous and degassed DMA (1.0 mL), aryl bromide (0.2 mmol, 1 equiv., if liquid), the respective coupling partner (0.6 mmol, 3 equiv., if liquid), 2-*tert*-butyl-1,1,3,3-tetramethylguanidine (60.5 µL, 0.3 mmol, 1.5 equiv.) and triethylamine (13.9 µL, 0.1 mmol, 0.5 equiv.) were added. The vial was capped with Teflon septum and removed from the glovebox. Then the reaction mixture was stirred at room temperature for 48 h under irradiation with 620 nm LED lamps (AL3 parallel photoreactor, set up 15W) with fan cooling. After the reaction was completed, brine (20 mL) was added and the mixture was extracted with EtOAc (20 mL) three times. The combined organic layer was washed with H_2O (20 mL) and brine (20 mL). The organic layer was dried with anhydrous Na_2SO_4, then concentrated under vacuum. The product was purified by flash column chromatography on silica gel using hexane: EtOAc as eluent.

General Procedure E

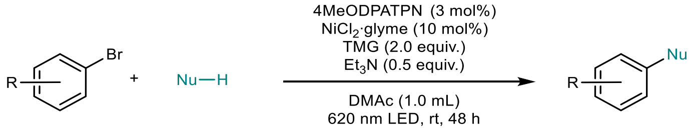

A dry 4-mL vial equipped with a Teflon-coated magnetic stir bar was charged with aryl bromide (0.2 mmol, 1 equiv., if solid), the respective coupling partner (0.6 mmol, 3 equiv., if solid), $NiCl_2$·glyme (4.4 mg, 0.02 mmol, 10 mol %), and 4MeODPATPN (6.2 mg, 0.006 mmol, 0.03 equiv.). The vial was introduced to a glovebox filled with argon atmosphere. Then anhydrous and degassed DMA (1.0 mL), aryl bromide (0.2 mmol, 1 equiv., if liquid), the respective coupling partner (0.6 mmol, 3 equiv., if liquid), 1,1,3,3-tetramethylguanidine (50.3 µL, 0.4 mmol, 2.0 equiv.) and triethylamine (13.9 µL, 0.1 mmol, 0.5 equiv.) were added. The vial was capped with Teflon septum and removed from the glovebox. Then the reaction mixture was stirred at room temperature for 48 h under irradiation with 620 nm LED lamps (AL3 parallel photoreactor, set up 15W) with fan cooling. After the reaction was completed, brine (20 mL) was added and the mixture was extracted with EtOAc (20 mL) three times. The combined organic layer was washed with H_2O (20 mL) and brine (20 mL). The organic layer was dried with anhydrous Na_2SO_4, then concentrated under vacuum. The product was purified by flash column chromatography on silica gel using hexane: EtOAc as eluent.

General Procedure F

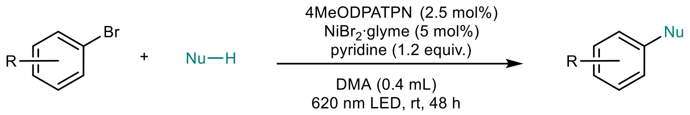

A dry 4-mL vial equipped with a Teflon-coated magnetic stir bar was charged with aryl bromide (0.2 mmol, 1 equiv., if solid), the respective coupling partner (0.3 mmol, 1.5 equiv., if solid), $NiBr_2$·glyme (3.2 mg, 0.01 mmol, 5 mol %), and 4MeODPATPN (5.2 mg, 0.005 mmol, 0.025 equiv.). The vial was introduced to a glovebox filled with argon atmosphere. Then anhydrous and degassed DMA (0.4 mL), aryl bromide (0.2 mmol, 1 equiv., if liquid) and the respective coupling partner (0.3 mmol, 1.5 equiv., if liquid), pyridine (19.5 µL, 0.24 mmol, 1.2 equiv.) were added successively. The vial was capped with Teflon septum and removed from the glovebox. Then the reaction mixture was stirred at room temperature for 48 h under irradiation with 620 nm LED lamps (AL3 parallel photoreactor, set up 15W) with fan cooling. After the reaction was completed, brine (20 mL) was added and the mixture was extracted with EtOAc (20 mL) three times. The combined organic layer was washed with H_2O (20 mL) and brine (20 mL). The organic layer was dried with anhydrous Na_2SO_4, then concentrated under vacuum. The product was purified by flash column chromatography on silica gel using hexane: EtOAc as eluent.

General Procedure G

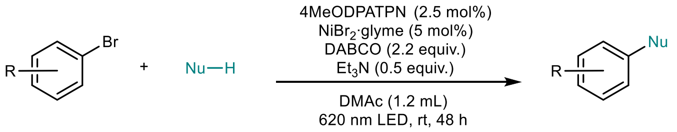

A dry 4-mL vial equipped with a Teflon-coated magnetic stir bar was charged with aryl bromide (0.2 mmol, 1 equiv., if solid), the respective coupling partner (0.36 mmol, 1.8 equiv., if solid), $NiBr_2$·glyme (3.2 mg, 0.01 mmol, 5 mol %), DABCO (49.2 mg, 0.44 mmol, 2.2 equiv.) and 4MeODPATPN (5.2 mg, 0.005 mmol, 0.025 equiv.). The vial was introduced to a glovebox filled with argon atmosphere. Then anhydrous and degassed DMA (1.2 mL), aryl bromide (0.2 mmol, 1 equiv., if liquid), the respective coupling partner (0.36 mmol, 1.8 equiv., if liquid) and triethylamine (13.9 µL, 0.1 mmol, 0.5 equiv.) were added sequentially. The vial was capped with Teflon septum and removed from the glovebox.

Then the reaction mixture was stirred at room temperature for 48 h under irradiation with 620 nm LED lamps (AL3 parallel photoreactor, set up 15W) with fan cooling. After the reaction was completed, brine (20 mL) was added and the mixture was extracted with EtOAc (20 mL) three times. The combined organic layer was washed with H_2O (20 mL) and brine (20 mL). The organic layer was dried with anhydrous Na_2SO_4, then concentrated under vacuum. The product was purified by flash column chromatography on silica gel using hexane: EtOAc as eluent.

General Procedure H

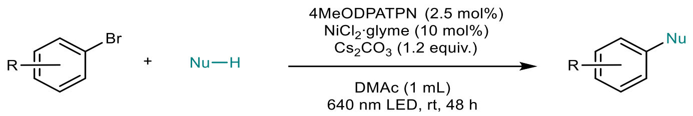

A dry 4-mL vial equipped with a Teflon-coated magnetic stir bar was charged with aryl bromide (0.2 mmol, 1 equiv., if solid), the respective coupling partner (0.3 mmol, 1.5 equiv., if solid), $NiCl_2$·glyme (4.4 mg, 0.02 mmol, 10 mol %), Cs_2CO_3 (78.2 mg, 0.24 mmol, 1.2 equiv.) and 4MeODPATPN (5.2 mg, 0.005 mmol, 0.025 equiv.). The vial was introduced to a glovebox filled with argon atmosphere. Then anhydrous and degassed DMA (2.0 mL), aryl bromide (0.2 mmol, 1 equiv., if liquid), and the respective coupling partner (0.3 mmol, 1.5 equiv., if liquid) were added sequentially. The vial was capped with Teflon septum and removed from the glovebox. Then the reaction mixture was stirred at room temperature for 48 h under irradiation with 640 nm LED lamps (AL3 parallel photoreactor, set up 15W) with fan cooling. After the reaction was completed, brine (20 mL) was added and the mixture was extracted with EtOAc (20 mL) three times. The combined organic layer was washed with H_2O (20 mL) and brine (20 mL). The organic layer was dried with anhydrous Na_2SO_4, then concentrated under vacuum. The product was purified by flash column chromatography on silica gel using hexane: EtOAc as eluent.

4.2. Scale-up reactions

General procedure for a 10 mmol scale with C-N cross-coupling reaction

A dry 50-mL vial equipped with a Teflon-coated magnetic stir bar was charged with 4-bromobenzonitrile (1.82 g, 10 mmol, 1 equiv.), $NiCl_2$·glyme (110 mg, 0.5 mmol, 5 mol %), DABCO (2.243 g, 20 mmol, 2.0 equiv.) and 4MeODPATPN (52 mg, 0.5 mmol, 0.005 equiv.). The vial was introduced to a glovebox filled with argon atmosphere. Then anhydrous and degassed DMA (6.5 mL) and piperidine (2.96 mL, 30 mmol, 3 equiv.) were added. The vial was capped with Teflon septum and removed from the glovebox. Then the reaction mixture was stirred at room temperature for 48 h under irradiation with 620 nm LED lamps (AL3 parallel photoreactor, set up 15W) with fan cooling. After the reaction was completed, brine (40 mL) was added and the mixture was extracted with EtOAc (40 mL) three times. The combined organic layer was washed with H_2O (40 mL) and brine (40 mL). The organic layer was dried with anhydrous Na_2SO_4, then concentrated under vacuum. The product (97%, 1.806 g) was purified by flash column chromatography on silica gel using hexane: EtOAc as eluent.

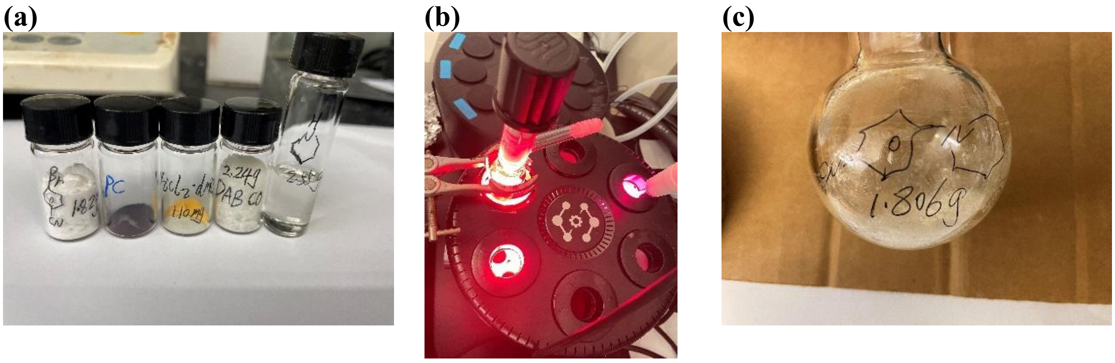

Figure S229. Pictures of the 10.0 mmol scale reaction for C-N cross-coupling: (a) Starting materials, photocatalyst, $NiCl_2$•glyme, DABCO, piperidine. **(b)** The reaction mixture photo-irradiation ongoing. **(c)** The desired product after purification with column chromatography.

General procedure for a 10 mmol scale with C-S cross-coupling reaction

A dry 50-mL vial equipped with a Teflon-coated magnetic stir bar was charged with 4-bromobenzonitrile (1.82 g, 10 mmol, 1 equiv.), $NiBr_2$·glyme (32 mg, 0.1 mmol, 1 mol %) and 4MeODPATPN (130 mg, 1.25 mmol, 0.0125 equiv.). The vial was introduced to a glovebox filled with argon atmosphere. Then anhydrous and degassed DMA (5.0 mL), 1-decanethiol (3.174 mL, 15 mmol, 1.5 equiv.) and pyridine (0.95 mL, 12 mmol, 1.2 equiv.) were added sequentially. The vial was capped with Teflon septum and removed from the glovebox. Then the reaction mixture was stirred at room temperature for 48 h under irradiation with 620 nm LED lamps (AL3 parallel photoreactor, set up 15W) with fan cooling. After the reaction was completed, brine (40 mL) was added and the mixture was extracted with EtOAc (40 mL) three times. The combined organic layer was washed with H_2O (40 mL) and brine (40 mL). The organic layer was dried with anhydrous Na_2SO_4, then concentrated under vacuum. The product (95%, 2.612 g) was purified by flash column chromatography on silica gel using hexane: EtOAc as eluent. .

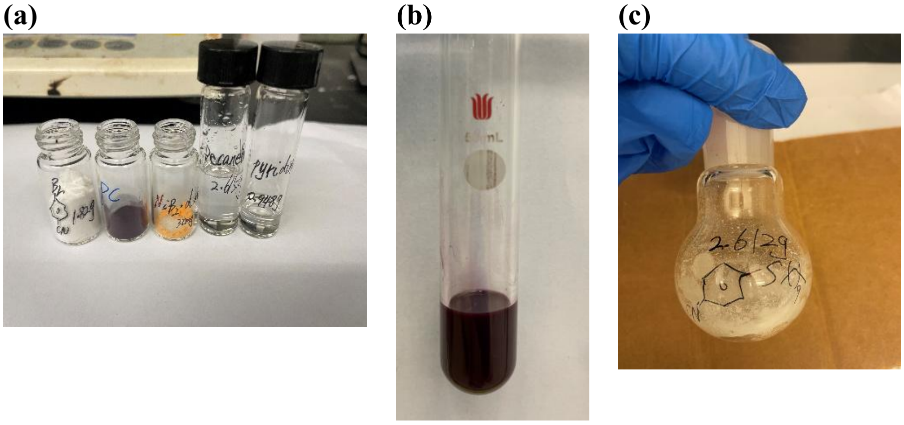

Figure S230. Pictures of the 10.0 mmol scale reaction for C-S cross-coupling: (a) Aryl bromide, photocatalyst, $NiBr_2$•glyme,1-decanethiol, pyridine. **(b)** The reaction mixture after completion. **(c)** The desired product after purification with column chromatography.

General procedure for a 20 mmol scale with C-S cross-coupling reaction

A dried 100-mL round-bottom flask was charged with a Teflon-coated magnetic stir bar was charged with 4-bromobenzonitrile (3.64 g, 20 mmol, 1 equiv.), $NiBr_2$·glyme (64 mg, 0.2 mmol, 0.1 mol %) and 4MeODPATPN (20.8 mg, 0.02 mmol, 0.001 equiv.). The vial was introduced to a glovebox filled with argon atmosphere. Then anhydrous and degassed DMA (10.0 mL), 1-decanethiol (6.348 mL, 30 mmol, 30 equiv.) and pyridine (1.9 mL, 24 mmol, 2.4 equiv.) were added sequentially. The vial was capped with Teflon septum and removed from the glovebox. Then the reaction mixture was stirred at room temperature for 48 h under irradiation with 640 nm LED lamps with fan cooling. After the reaction was completed, brine (40 mL) was added and the mixture was extracted with EtOAc (40 mL) three times. The combined organic layer was washed with H_2O (40 mL) and brine (40 mL). The organic layer was dried with anhydrous Na_2SO_4, then concentrated under vacuum. The product (99%, 5.459 g) was purified by flash column chromatography on silica gel using hexane: EtOAc as eluent. The NMR spectra is as below.

(a) **(b)** **(c)**

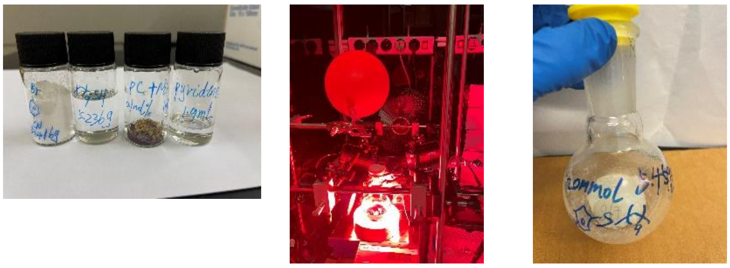

Figure S231. Pictures of the 20.0 mmol scale reaction for C-S cross-coupling: (a) Aryl bromide, photocatalyst, $NiBr_2$•glyme,1-decanethiol, pyridine. **(b)** The reaction mixture after completion. **(c)** The desired product after purification with column chromatography.

^{1}H NMR

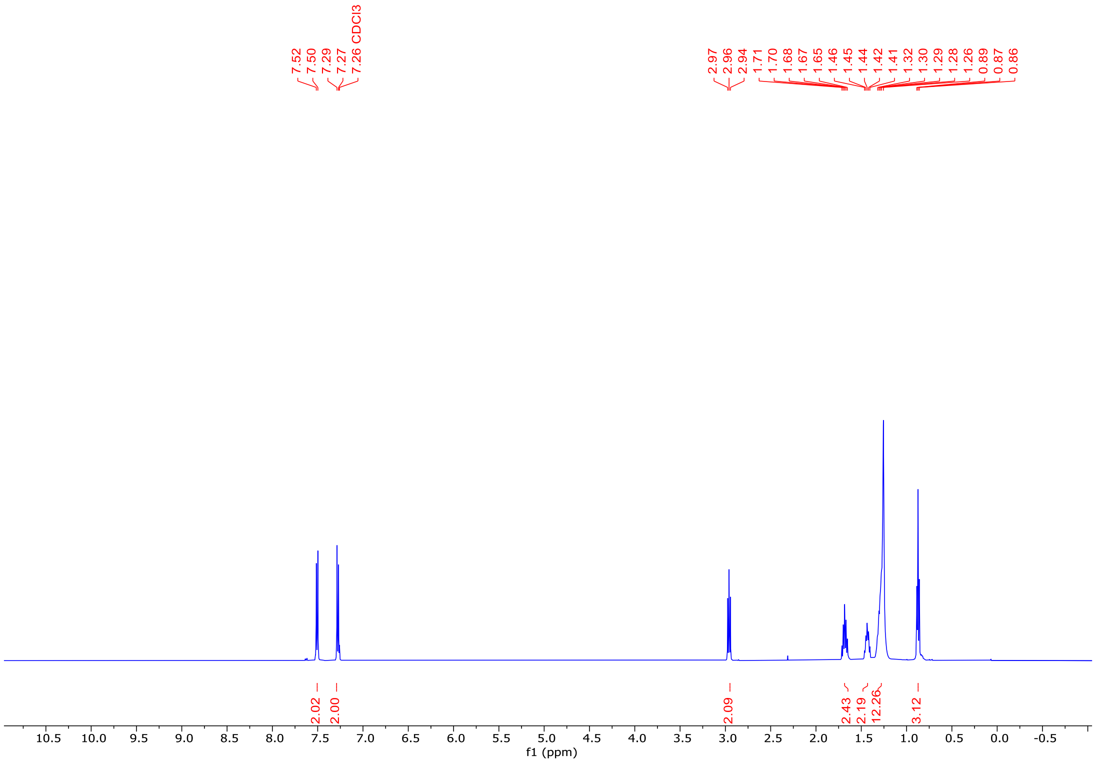

^{13}C NMR

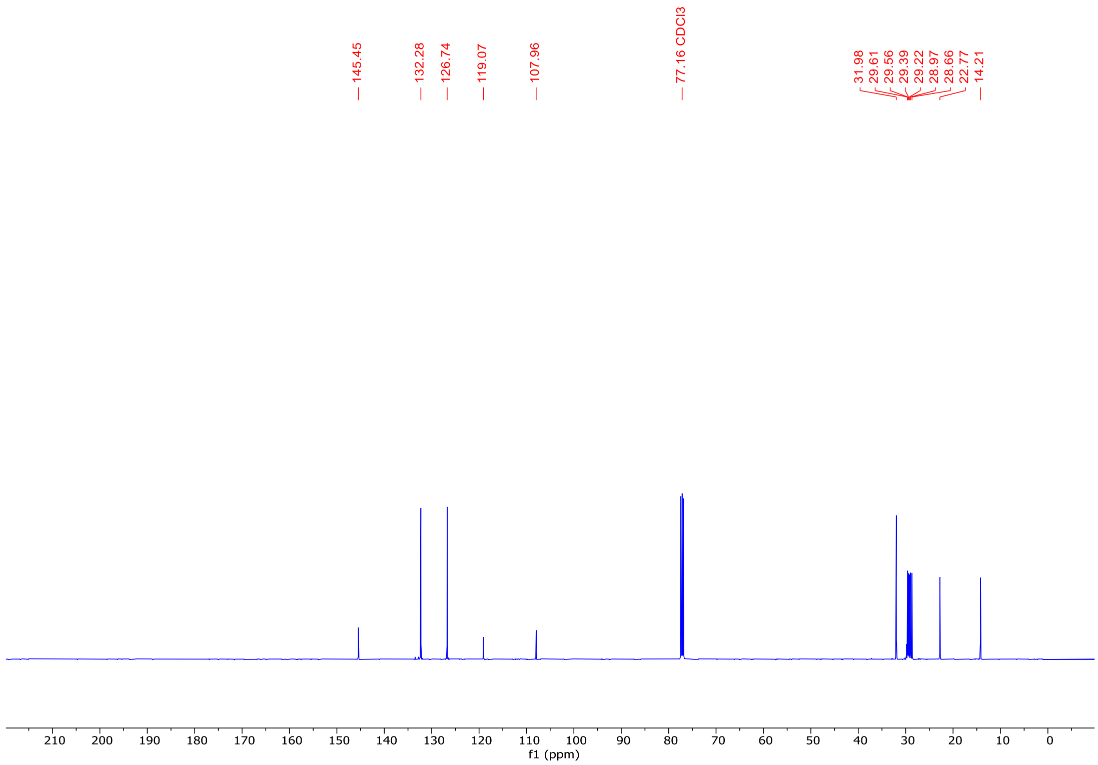

A dried 100-mL round-bottom flask was charged with a Teflon-coated magnetic stir bar was charged with 4-bromobenzonitrile (3.64 g, 20 mmol, 1 equiv.), $NiBr_2$·glyme (32 mg, 0.1 mmol, 0.05 mol %) and 4MeODPATPN (20.8 mg, 0.02 mmol, 0.001 equiv.). The vial was introduced to a glovebox filled with argon atmosphere. Then anhydrous and degassed DMA (10.0 mL), *N*-(*tert*-butoxycarbonyl)-L-cysteine methyl ester (6.2 mL, 30 mmol, 30 equiv.) and pyridine (1.9 mL, 24 mmol, 2.4 equiv.) were added sequentially. The vial was capped with Teflon septum and removed from the glovebox. Then the reaction mixture was stirred at room temperature for 48 h under irradiation with 640 nm LED lamps with fan cooling. After the reaction was completed, brine (40 mL) was added and the mixture was extracted with EtOAc (40 mL) three times. The combined organic layer was washed with H_2O (40 mL) and brine (40 mL). The organic layer was dried with anhydrous Na_2SO_4, then concentrated under vacuum. The crude product was purified by flash column chromatography on silica gel using hexane: EtOAc as eluent. The product (70%, 5.316 g). was purified by recrystallization from hexane. The NMR spectra is as below.

(a)

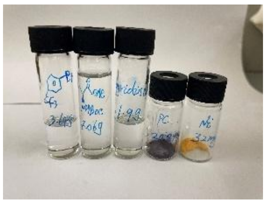

(b)

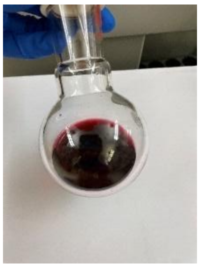

(c)

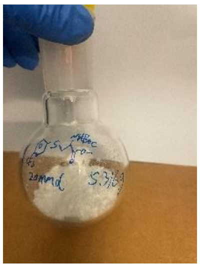

Figure S232. Pictures of the 20.0 mmol scale reaction for C-S cross-coupling: (a) Aryl bromide, photocatalyst, $NiBr_2$•glyme, *N*-(*tert*-butoxycarbonyl)-L-cysteine methyl ester, pyridine. **(b)** The reaction mixture before photo-irradiation. **(c)** The desired product after purification with column chromatography and recrystallization.

^{1}H NMR

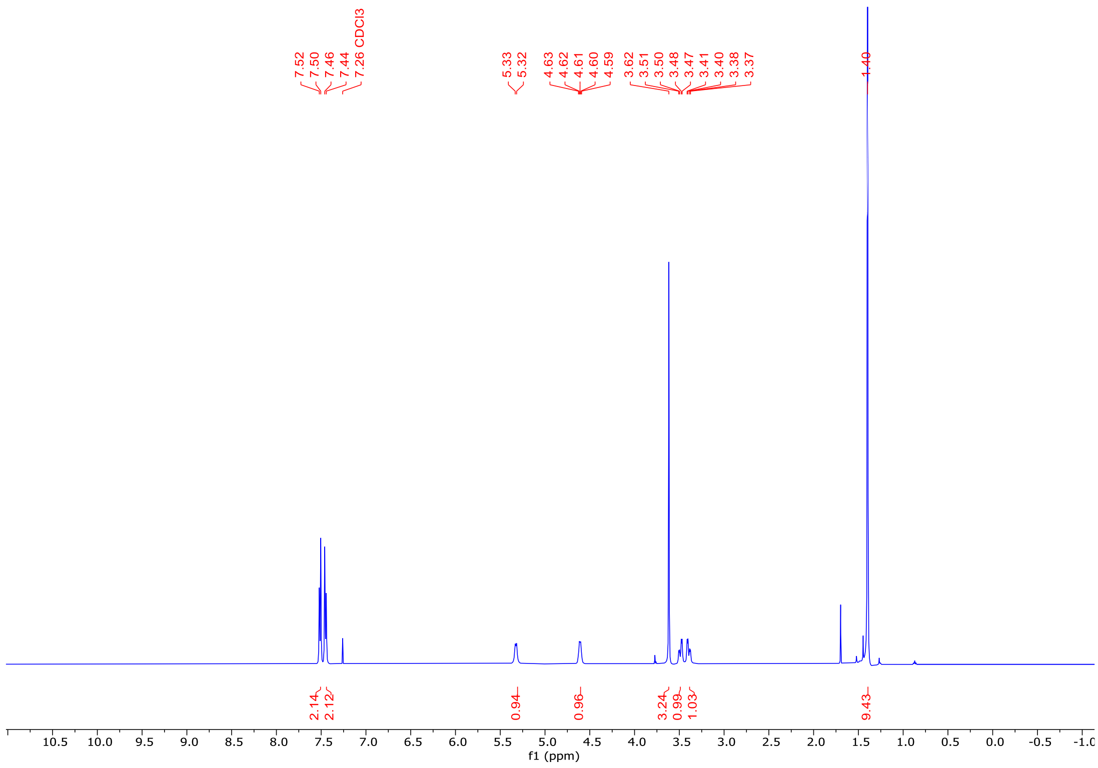

^{13}C NMR

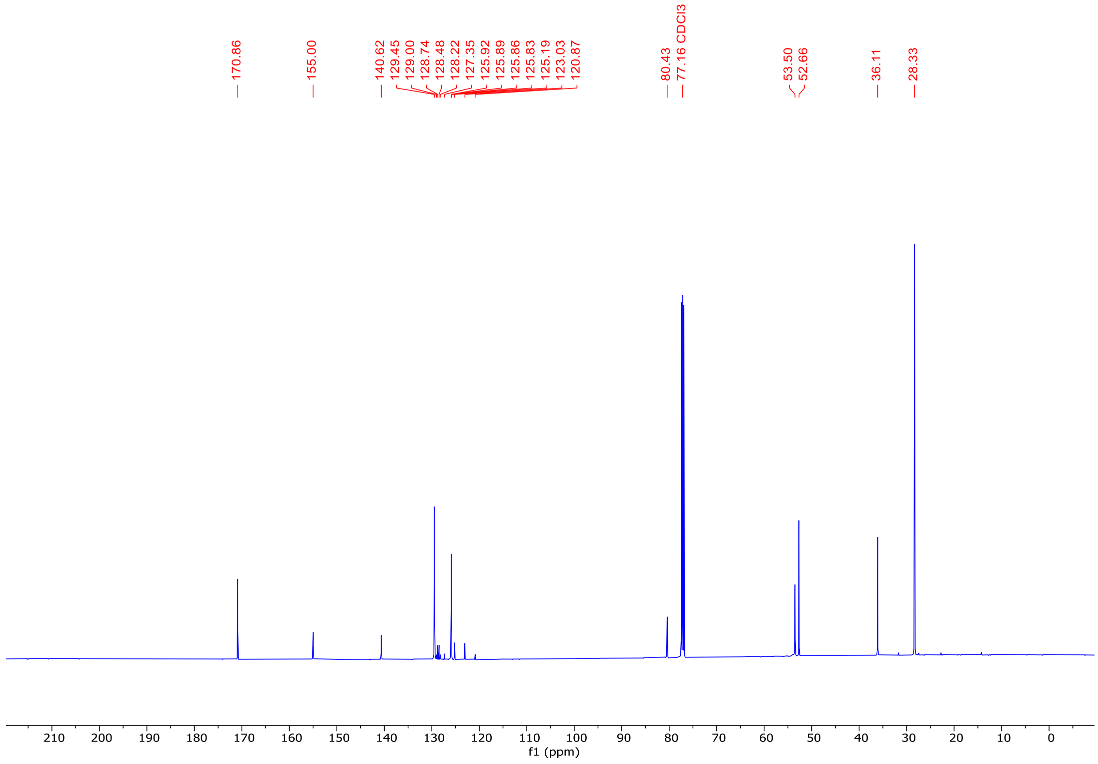

^{19}F NMR

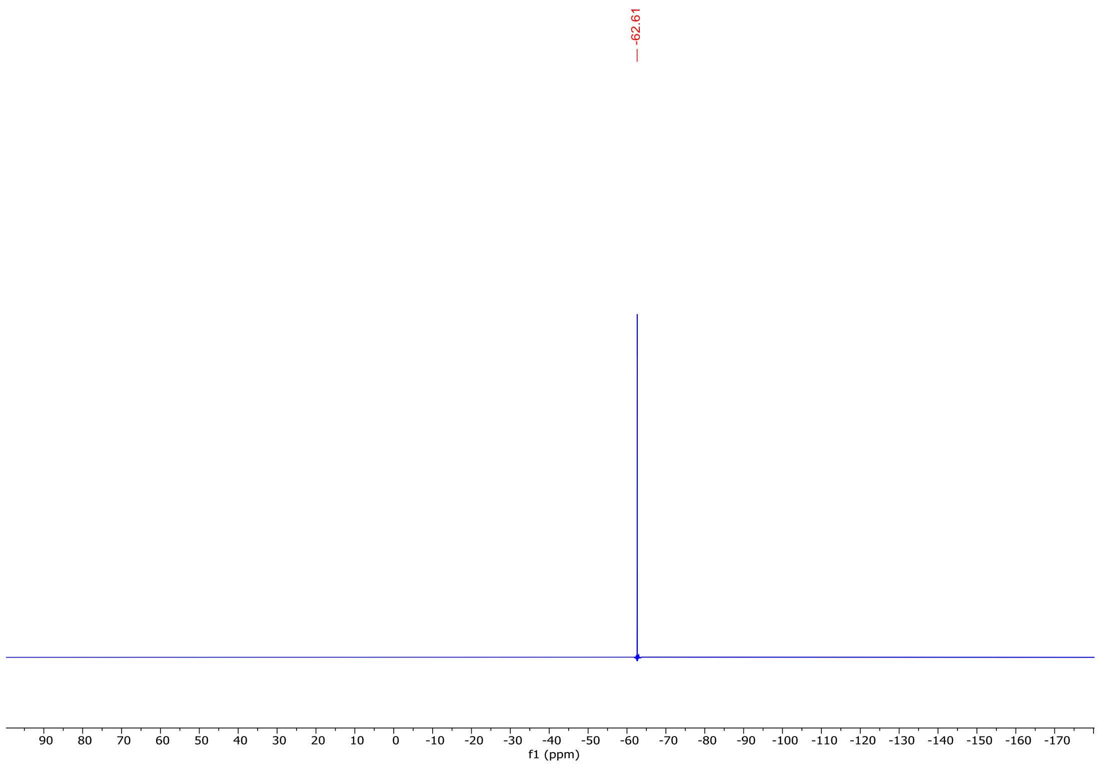

General procedure for a 100 mmol scale with C-N cross-coupling reaction

A dried 250-mL round-bottom flask equipped with a Teflon-coated magnetic stir bar was charged with 4-bromobenzonitrile (18.2 g, 100 mmol, 1 equiv.), $NiCl_2$·glyme (110 mg, 0.05 mmol, 0.5 mol %), DABCO (22.434 g, 200 mmol, 2.0 equiv.) and 4MeODPATPN (52 mg, 0.05 mmol, 0.0005 equiv.). The vial was introduced to a glovebox filled with Argon atmosphere. Then anhydrous and degassed DMA (65 mL) and piperidine (19.70 mL, 200 mmol, 2 equiv.) were added. The vial was capped with Teflon septum and removed from the glovebox. Then the reaction mixture was stirred at room temperature for 48 h under irradiation with 640 nm LED lamps with fan cooling. After the reaction was completed, brine (40 mL) was added and the mixture was extracted with EtOAc (40 mL) three times. The combined organic layer was washed with H_2O (40 mL) and brine (40 mL). The organic layer was dried with anhydrous Na_2SO_4, then concentrated under vacuum. The product (80%, 14.901 g) was purified by flash column chromatography on silica gel using hexane: EtOAc as eluent.

(a) **(b)** **(c)**

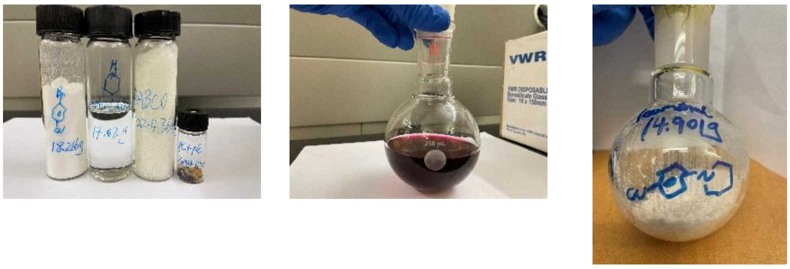

Figure S233. Pictures of the 100.0 mmol scale reaction for C-N cross-coupling: (a) Starting materials, photocatalyst, $NiCl_2$•glyme, DABCO, piperidine. **(b)** The reaction mixture before photo-irradiation. **(c)** The desired product after purification with column chromatography.

^{1}H NMR

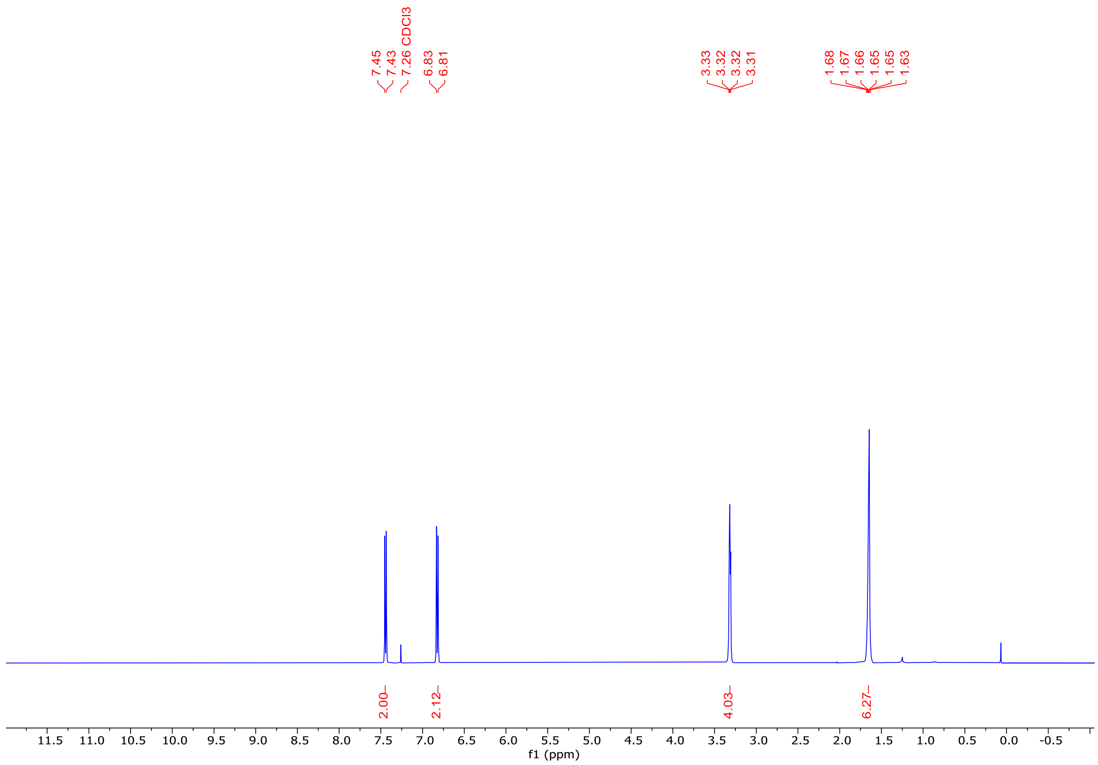

^{13}C NMR

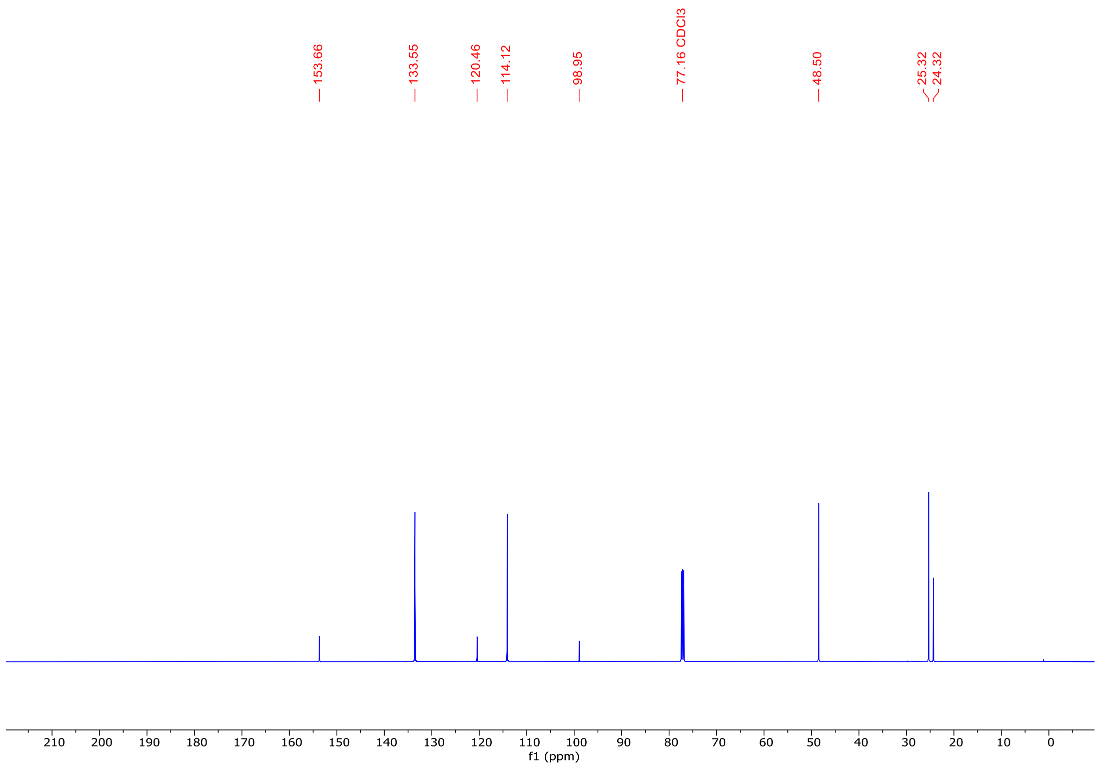

5. Mechanistic studies

5.1. Radical-trapping experiments

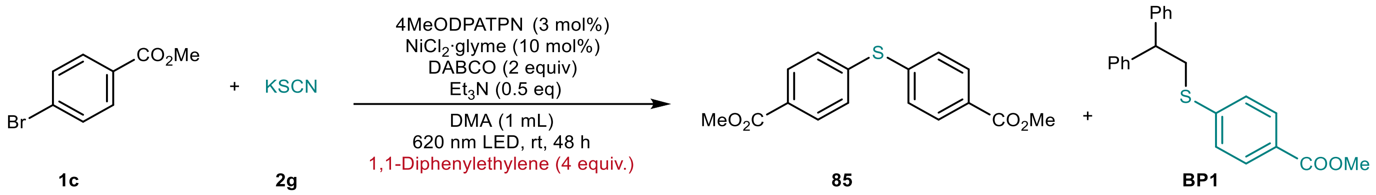

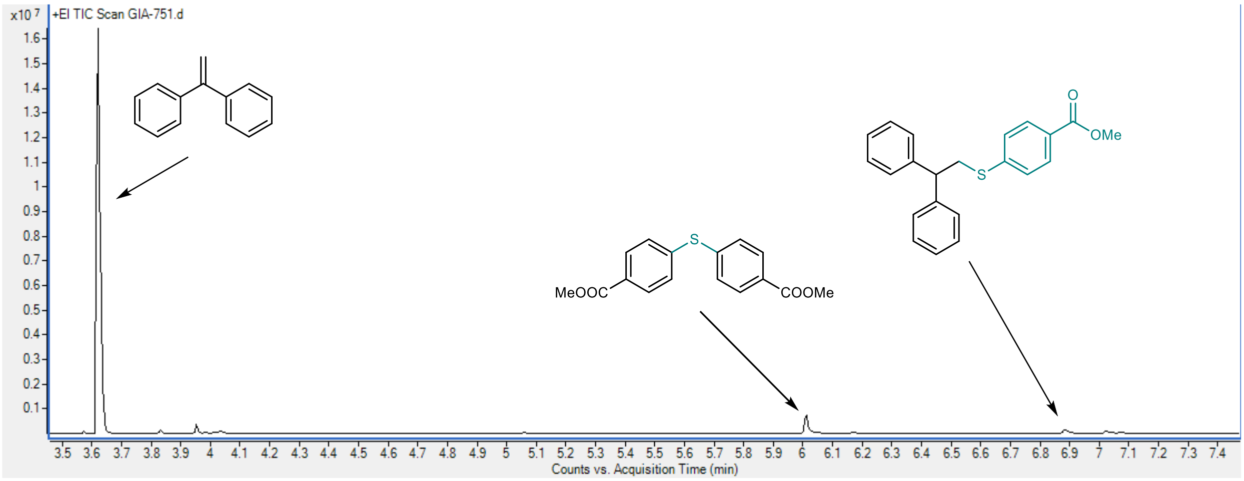

Figure S234. Radical trapping reaction.

A dry 4-mL vial equipped with a Teflon-coated magnetic stir bar was charged with methyl 4-bromobenzoate (43 mg, 0.2 mmol, 1 equiv.), potassium thiocyanate (31 mg, 0.3 mmol, 1.5 equiv.), $NiCl_2$·glyme (4.4 mg, 0.02 mmol, 10 mol %), DABCO (44.9 mg, 0.4 mmol, 2.0 equiv.) and 4MeODPATPN (6.2 mg, 0.006 mmol, 0.03 equiv.). The vial was introduced to a glovebox filled with argon atmosphere. Then anhydrous and degassed DMA (1.0 mL), triethylamine (13.9 µL, 0.1 mmol, 0.5 equiv.), 1,1-diphenylethylene (DPE) (144 µL, 0.2 mmol, 4 equiv.) were added. The vial was capped with Teflon septum and removed from the glovebox. Then the reaction mixture was stirred at room temperature for 48 h under irradiation with 620 nm LED lamps (AL3 parallel photoreactor, set up 15W) with fan cooling. After that, the reaction mixture was passed through a pad of silica gel and washed with ethyl acetate. Then internal standard (dodecane, 0.2 mmol) was added. The solution was analyzed by GC- FID and GC-MS. The trace amount of radical-adduct product **BP1** was detected. (**Fig. S234**) The results indicated that a sulfur radical was involved in the transformation.

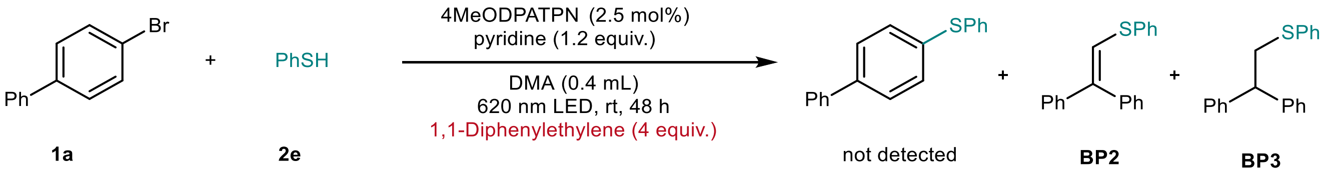

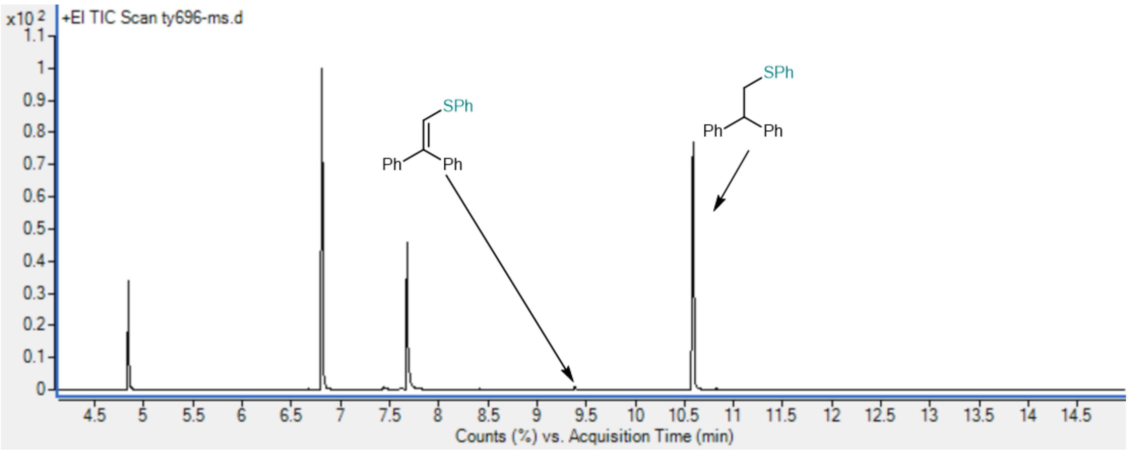

Figure S235. Radical trapping reaction.

A dry 4-mL vial equipped with a Teflon-coated magnetic stir bar was charged with 4-bromobenzonitrile (36.4 g, 0.2 mmol, 1 equiv.), $NiBr_2$·glyme (3.2 mg, 0.01 mmol, 5 mol %), and 4MeODPATPN (5.2 mg, 0.005 mmol, 0.025 equiv.). The vial was introduced to a glovebox filled with argon atmosphere. Then anhydrous and degassed DMA (0.4 mL), thiophenol (31 µL, 0.3 mmol, 1.5 equiv.), pyridine (19.5 µL, 0.36 mmol, 1.2 equiv.) and 1,1-Diphenylethylene (DPE) (140 µL, 0.8 mmol, 4 equiv.) were added sequentially. The vial was capped with Teflon septum and removed from the glovebox. Then the reaction mixture was stirred at room temperature for 48 h under irradiation with 620 nm LED lamps (AL3 parallel photoreactor, set up 15W) with fan cooling. After that, the reaction mixture was passed through a pad of silica gel and washed with ethyl acetate. Then the internal standard (dodecane, 0.2 mmol) was added. The solution was analyzed by GC-FID and GC-MS. The **BP2** and **BP3** were detected. The results indicated that a sulfur radical was involved in the transformation. (**Fig. S235**)

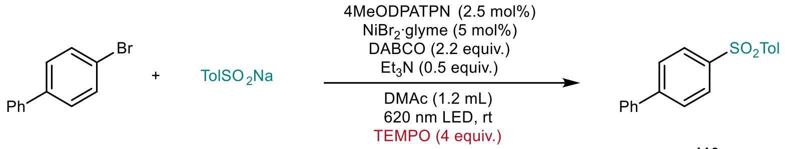

Figure S236. Radical trapping reaction.

A dry 4-mL vial equipped with a Teflon-coated magnetic stir bar was charged with 4-bromobiphenyl (46.6 mg, 0.2 mmol, 1.0 equiv.), sodium 4-methylbenzenesulfinate (64.2 mg, 0.36 mmol, 1.8 equiv.), $NiBr_2$·glyme (3.2 mg, 0.01 mmol, 5 mol %), DABCO (49.2 mg, 0.44 mmol, 2.2 equiv.) and 4MeODPATPN (5.2 mg, 0.005 mmol, 0.025 equiv.). The vial was introduced to a glovebox filled with argon atmosphere. Then anhydrous and degassed DMA (1.2 mL) and triethylamine (13.9 µL, 0.1 mmol, 0.5 equiv.) were added sequentially. Then the reaction mixture was stirred at room temperature for 48 h under irradiation with 620 nm LED lamps (AL3 parallel photoreactor, set up 15W) with fan cooling. After that, the reaction mixture was passed through a pad of silica gel and washed with ethyl acetate. Then internal standard (dodecane, 0.2 mmol) was added. The solution was analyzed by GC- FID and GC-MS. The

reaction was completely inhibited and no desired product was detected. This result suggested that a sulfonyl radical was involved in the transformation.[10] (**Fig. S236**)

5.2. Reactivity comparison of the red and blue light protocols by using carboxylic acids as a cross-coupling partner

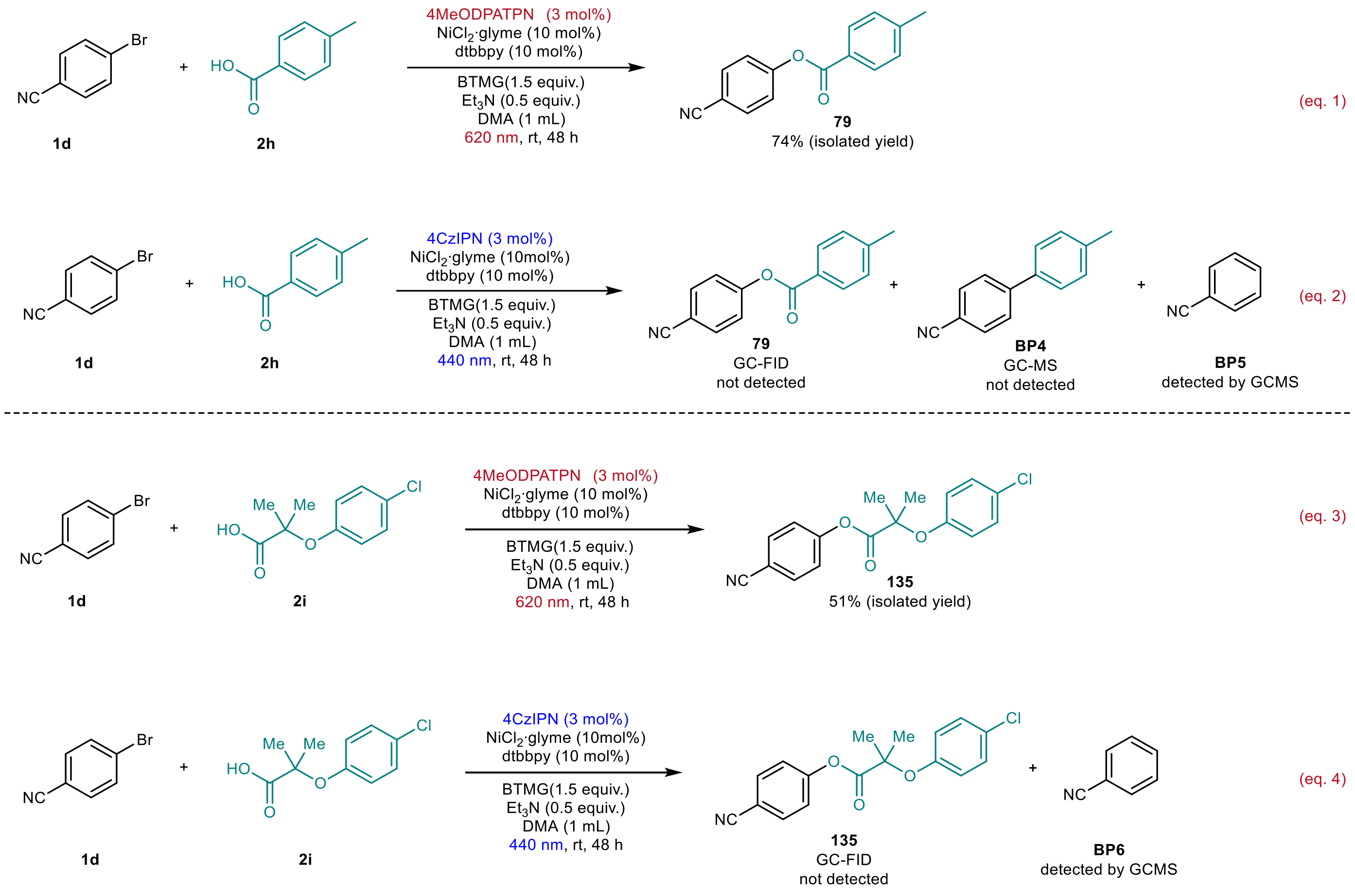

Figure S237.

We compared the product formation from the carboxylic acids (**2h**, **2i**, **2j**) under 440 and 620 nm lamps. Under 620 nm irradiation, the carboxylic acid coupled successfully, affording the corresponding C-O cross-coupling product (**79**, **135**, **136**) in good yield (eq. 1, eq. 3, eq. 5). Under this protocol, only trace amounts of hydrodehalogenation products (**161**) from corresponding aryl bromide (**1d**) were detected. As a comparison, under 440 nm irradiation, no C-O cross-coupling products (**79**, **135**, **136**) or decarboxylative arylation products (**BP4**) were found (eq. 2, eq. 4, eq. 6). In contrast, a significant amount of hydrodehalogenation product (**BP5**) was formed from aryl bromide (eq. 2, eq. 4, eq. 6). Notably, (*R*)-2-(6-methoxynaphthalen-2-yl)propanoic acid (**2j**) led to decarboxylation (**BP6**) and decarboxylative homo-coupling products (**BP7**, **BP8**) (eq. 6). These results demonstrate the effectiveness and wide applicability of **PC13** in our red light protocol. (**Fig. S237, S238**)

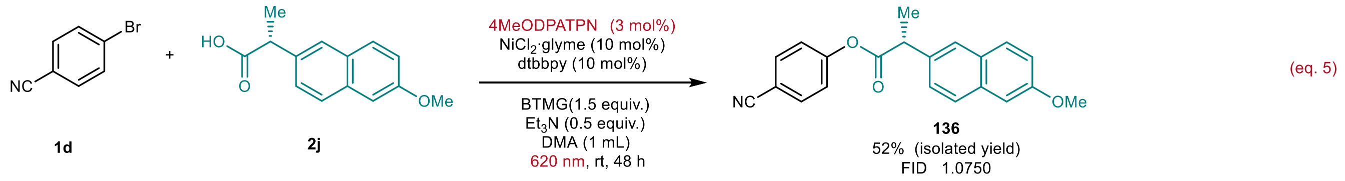

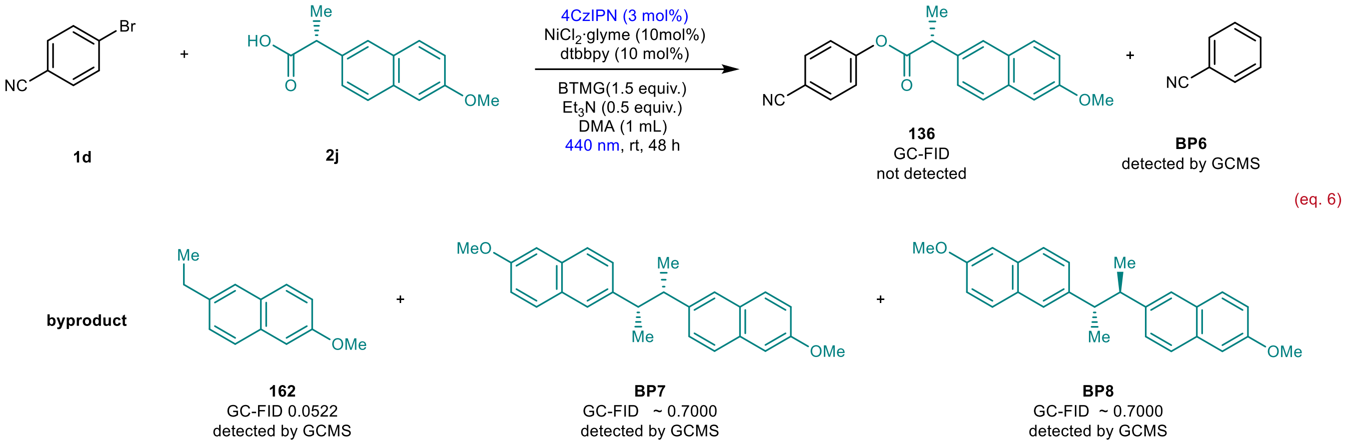

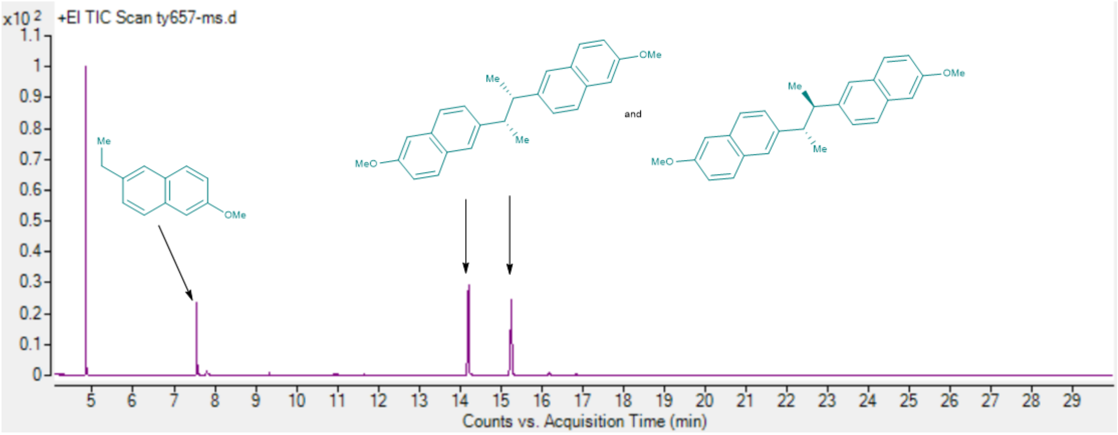

Figure S238.

General Procedure (**red light**)

A dry 4-mL vial equipped with a Teflon-coated magnetic stir bar was charged with 4-bromobenzonitrile (36.4 mg, 0.2 mmol,1 equiv.), the respective carboxylic acid (0.6 mmol, 3 equiv., if solid), $NiCl_2$·glyme (4.4 mg, 0.02 mmol, 10 mol %), 4,4'-di-*tert*-butyl-2,2'-bipyridine (5.4 mg, 0.02 mmol, 10 mol %) and 4MeODPATPN (6.2 mg, 0.006 mmol, 0.03 equiv.). The vial was introduced to a glovebox filled with argon atmosphere. Then anhydrous and degassed DMA (1.0 mL), the respective carboxylic acid (0.6 mmol, 3 equiv., if liquid), 2-*tert*-butyl-1,1,3,3-tetramethylguanidine (60.5 µL, 0.3 mmol, 1.5 equiv.) and triethylamine (13.9 µL, 0.1 mmol, 0.5 equiv.) were added. The vial was capped with Teflon septum and removed from the glovebox. Then the reaction mixture was stirred at room temperature for 48 h under irradiation with 620 nm LED lamps (AL3 parallel photoreactor, set up 15W) with fan cooling. After that, the reaction mixture was passed through a pad of silica gel and washed with ethyl acetate. Then internal standard (dodecane, 0.2 mmol) was added. The solution was analyzed by GC-FID and/or GC-MS.

General Procedure (**blue light)**

A dry 4-mL vial equipped with a Teflon-coated magnetic stir bar was charged with 4-bromobenzonitrile (36.4 mg, 0.2 mmol,1 equiv.), the respective carboxylic acid (0.6 mmol, 3 equiv., if solid), $NiCl_2$·glyme (4.4 mg, 0.02 mmol, 10 mol %), 4,4'-di-*tert*-butyl-2,2'-bipyridine (5.4 mg, 0.02 mmol, 10 mol %) a 4 CzIPN (4.6 mg, 0.006 mmol, 0.03 equiv.). The vial was introduced to a glovebox filled with Argon atmosphere. Then anhydrous and degassed DMA (1.0 mL), the respective carboxylic acid (0.6 mmol, 3 equiv., if liquid), 2-*tert*-butyl-1,1,3,3-tetramethylguanidine (60.5 µL, 0.3 mmol, 1.5 equiv.) and triethylamine (13.9 µL, 0.1 mmol, 0.5 equiv.) were added. The vial was capped with Teflon septum and removed from the glovebox. Then, the reaction mixture was stirred at room temperature for 48 h under irradiation with 440 nm LED lamps (AL3 parallel photoreactor, set up 15W) with fan cooling. After that, the reaction mixture was passed through a pad of silica gel and washed with ethyl acetate. Then, internal standard (dodecane, 0.2 mmol) was added. The solution was analyzed by GC-FID and/or GC-MS.

5.3. Reaction progress monitoring

Serval model reactions, (4-bromo-1,1'-biphenyl and piperidine as starting materials) following **General Procedure A**, were set up parallelly at the same time under the irradiation of 620 nm LED lamps. One vial was worked up when the reaction was run for 1h, 2h, 6h, 10h, 16h, 24h, 48. Each reaction was passed through a pad of silica gel and washed with ethyl acetate. The yields were determined via GC-FID analysis.

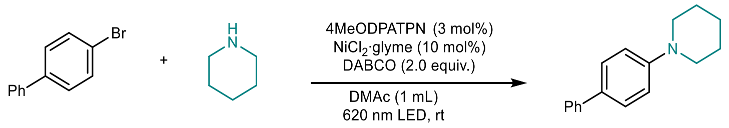

Reaction time	Yield
1h	9%
2h	23%
3h	36%
6h	49%
10h	65%
16h	80%
24h	90%
48h	93%

Serval model reactions, (4-bromobenzoate and potassium thiocyanate as starting materials) following **General Procedure B**, were set up parallelly at the same time under the irradiation of 620 nm LED lamps. One vial was worked up when the reaction was run for 3h, 6h, 9h, 16h, 24h, 32h, 48h. Each reaction passed through a pad of silica gel and washed with ethyl acetate. The yields were determined via GC-FID analysis.

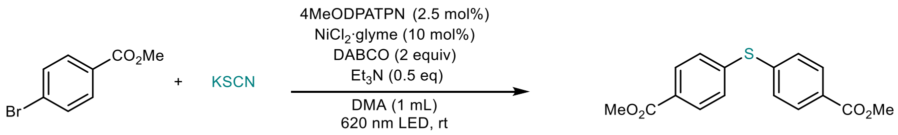

Reaction time	Yield
3h	10%
6h	30%
9h	50%
16h	79%
24h	89%
32h	89%
48h	90%

5.4. Light on-off studies

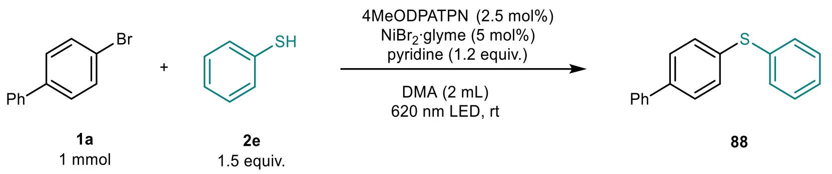

A dry 4-mL vial equipped with a Teflon-coated magnetic stir bar was charged with 4-bromo-1,1'-biphenyl (223 mg, 1 mmol, 1 equiv.), $NiBr_2$·glyme (16 mg, 0.05 mmol, 5 mol %), and 4MeODPATPN (26 mg, 0.01 mmol, 0.025 equiv.). The vial was introduced to a glovebox which with argon atmosphere. Then, anhydrous and degassed DMA (2 mL), thiophenol (160 µL, 1.5 mmol, 1.5 equiv.) and pyridine (96 µL, 1.2 mmol, 1.2 equiv.) were added sequentially. The vial was capped with Teflon septum and removed from the glovebox. Then, the reaction mixture was stirred at room temperature under irradiation with 620 nm LED lamps (AL3 parallel photoreactor, set up 15W) with fan cooling. Aliquots of the reaction mixture were then taken at the indicated times. Then, the mixture passed through a pad of silica gel, washed with ethyl acetate, and analyzed by GC-FID. When the light was turned off, the reaction almost ceased to take place; when the light was turned back on, the reaction resumed, indicating that the reaction can only take place under continuous irradiation.

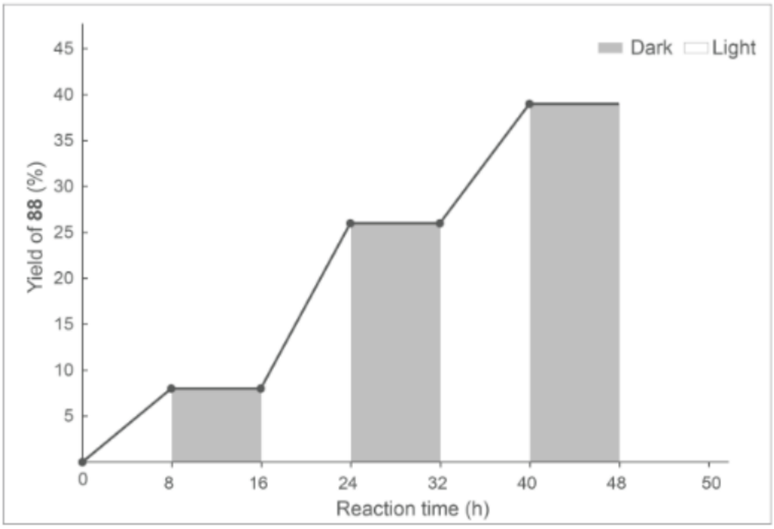

Figure S239. On/off experiments.

5.5. Decomposition pathways of the photocatalysts, isolation procedures and characterization.

It is essential to evaluate the stability of **PC**s to understand potential degradation mechanisms that may impact their catalytic activity over time.[11-18] Initial **PC13** was present in crude mixtures of compounds **1-154**, excluding **77**. Only the crude mixture of compound **77** exhibited complete decomposition of the photocatalyst. Consequently, further photostability studies of **PC13** were conducted by examining all bases utilized in standard reaction conditions in DMA solution.

All reactions were conducted with 5 µmol photocatalyst in 1 mL degassed solvent unless otherwise mentioned, for reactions with **PC13** 620 nm LED was used, for **4CzIPN** – 445 nm. The stability of photocatalysts was analyzed via TLC (1:1 hexane/DCM). If no additional spots appeared on the TLC plate and the photocatalyst remained, the reaction was called "stable". If the photocatalyst persisted and there were new spots—termed "moderate conversion”. Complete consumption of the initial photocatalyst resulted in "full conversion".

Initially, **PC13** demonstrated stability under irradiation for up to 3 days (**Table S21**, entries 1-4). Then, the duration time and/or the loading of bases were increased (**Table S21**, entries 5-12 and **Table S22**, entries 8-16). Therefore, entries with high concentration of DABCO, Et_3N, BTMG and $CyNH_2$ showed the additional yellow spot on the TLC with the same R_f value, and the solution with saturated DABCO (**Table S22**, entry 8) had the best conversion. The samples containing Py and TMG demonstrated stability. As a control experiment, **PC1** (4CzIPN) was tested with a significant excess of DABCO to assess stability, resulting in decomposition (**Table S22**, entry 6).

Table S21. Conditions showed stability of the photocatalyst.

Entry	Additive	Duration	Observation
1	None	3/6 days	Stable
2	Under air	3/6 days	Stable
3	10 equiv. DABCO	3 days	Stable
4	10 equiv. BTMG	3/6 days	Stable
5	10 equiv. $CyNH_2$	6 days	Stable
6	10 equiv. TMG	6 days	Stable
7	10 equiv. Pyridine	6 days	Stable
8	5 equiv.TMG + 5 equiv. $CyNH_2$	6 days	Stable
9	10 equiv. TMG + 2.5 equiv. Et_3N	6 days	Stable
10	10 equiv. BTMG + 2.5 equiv. Et_3N	6 days	Stable
11	150 equiv. TMG	6 days	Stable
12	150 equiv. Pyridine	6 days	Stable

The reaction of **PC13** with *p*-methoxy-*N*,*N*-dimethylaniline (*p*-$MeOPhNMe_2$) was investigated in DMA. For comparison, the reaction was also conducted with **PC1**. The red solution of **PC13** changed to an orange one. TLC analysis revealed no spots corresponding to **PC13** or **PC1**, only the major spot of the newly formed catalyst (**Table S22**, entries 1-5). Furthermore, an attempt was made to generate a stable radical-anion of **PC13** using an excess of KC_8 in the presence of [2.2.2]-cryptand.[19] However, the catalyst decomposed, resulting in a green solution, which turned yellow upon exposure to air.

Table S22. Conditions showed instability of the photocatalyst.

Entry	PC	Additive	Conversion time	Observation
1	**PC13**	10 equiv. *p*-$MeOPhNMe_2$	24-30 h	Full conversion
2	**PC13**	10 equiv. *p*-$MeOPhNMe_2$ + 10 equiv. DABCO	8-16 h	Full conversion
3	**PC13**	10 equiv. *p*-$MeOPhNMe_2$ + 10 equiv. Cs_2CO_3	8-16 h	Full conversion
4	**4CzIPN**	10 equiv. *p*-$MeOPhNMe_2$	< 2 h	Full conversion
5	**4CzIPN**	10 equiv. *p*-$MeOPhNMe_2$ + 10 equiv. DABCO	< 2 h	Full conversion
6	**4CzIPN**	Saturated DABCO solution	< 18 h	Full conversion
7	**PC13**	10 equiv. KC_8 + 2 equiv. [2.2.2]cryptand, no light	18-24 h	Full conversion
8	**PC13**	Saturated DABCO solution	> 4 days	Moderate conversion
9	**PC13**	10 equiv. DABCO	> 6 days	Moderate conversion
10	**PC13**	10 equiv. Et_3N	> 6 days	Moderate conversion
11	**PC13**	10 equiv.DABCO + 2.5 equiv. Et_3N	> 6 days	Moderate conversion
12	**PC13**	100 equiv. TMG + 100 equiv. $CyNH_2$	> 6 days	Moderate conversion
13	**PC13**	150 equiv. $CyNH_2$	> 6 days	Moderate conversion
14	**PC13**	150 equiv. Et_3N	> 6 days	Moderate conversion
15	**PC13**	150 equiv. BTMG	> 6 days	Moderate conversion
16	**PC13**	100 equiv. BTMG + 50 equiv. Et_3N	> 6 days	Moderate conversion

After the reactions' scale-up and further isolation of formed products (**Table S22,** entries 2, 6, 7, 8) **PC1-1**, **PC13-1**, **PC13-2**, and **PC13-3**, it turned out that *p*-MeOPhNMe$_2$ substitutes one of the nitrile groups of **PC13** forming **PC13-1** (**Fig. S240. A**). No attempt was made to isolate the product resulting from the reaction of **PC1** with *p*-MeOPhNMe$_2$. The reaction with DABCO yielded the photocatalysts **PC1-1** and **PC13-2** with substituted nitrile group by DMA molecule (**Fig. S240. B-C**). The reaction occurs similarly to a previously reported proccess probably *via* radical recombination of α-amino radical and radical-anion of photocatalyst with subsequent cyanide elimination.[15] The reaction with KC$_8$ produced the decyanation product **PC13-3** (**Fig. S240 D**).

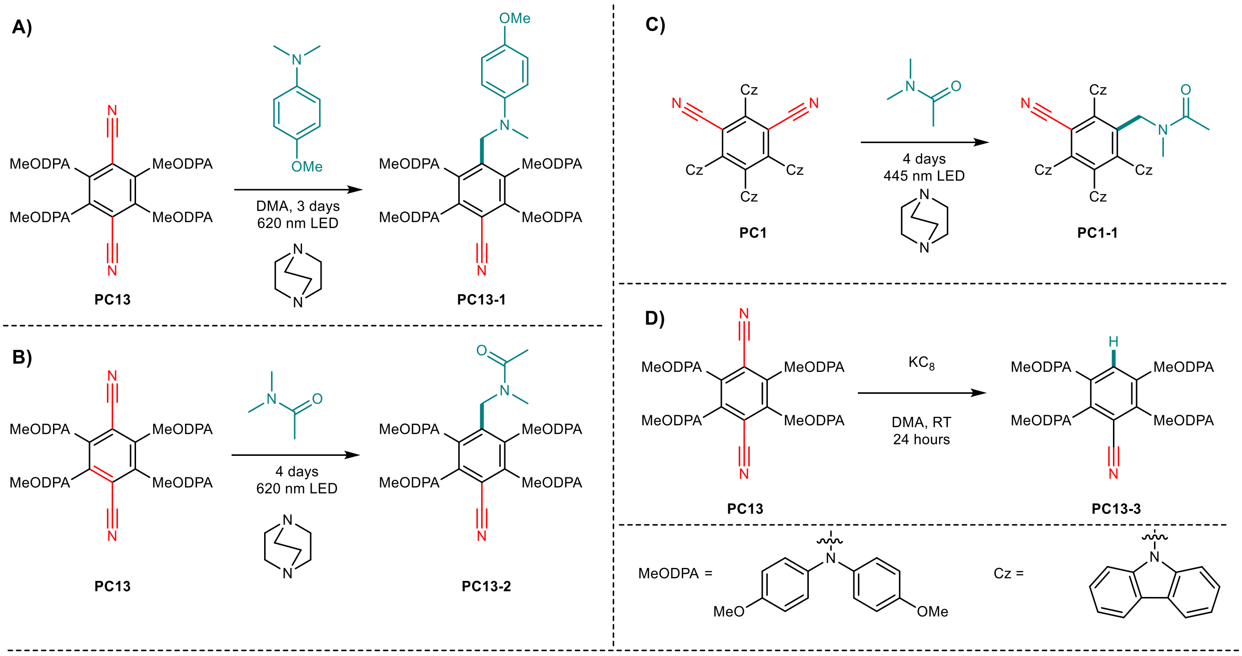

Figure S240. Photocatalysts decomposition under different conditions.

Hence, the substitution of a nitrile group in **PC1** and **PC13** induces a blue shift in absorption and emission spectra (**Table S1**) and enhances the reduction potential of the resulting photocatalyst. From a mechanistic standpoint, these stability experiments, combined with the obtained CV data (refer to **Table S22** and **Table S23**), provide insights that excited **PC13** can undergo SET transfer with DABCO, *p*-MeOPhNMe$_2$, CyNH$_2$, and Et$_3$N, yielding a **PC13** radical-anion which can initiate Ni$^{I/III}$ dark cycle. Moreover, they confirm that the newly formed photocatalysts **PC13-1** and **PC13-2** do not absorb 620 nm light, therefore, do not affect the main reaction. However, it should be noted that such photosubstitution products of dicyanobenzene-based photocatalysts are generally catalytically active and often the main photocatalyst. The blue-shifted absorption makes them inactive only under red-light irradiation.

Table S23. CV measured the redox potentials of bases and substrates.

Base/substrate	E (D^{+}/D) (V. vs SCE)
**DABCO**	0.68
**Et$_3$N**	0.93
**TMG**	0.91
**CyNH$_2$**	0.92
**BTMG**	0.93
***p*-MeOPhNMe$_2$**	0.66
**PhSH**	0.98
**Pyridine**	>1.5
**PhSH + Pyridine**	0.91
**TolSO$_2$Na**	0.37

5.5.1. Synthesis and spectroscopic data of PC1 and PC13 decompositon products

N-methyl-N-(2,3,4,6-tetra(9H-carbazol-9-yl)-5-cyanobenzyl)acetamide (PC1-1)

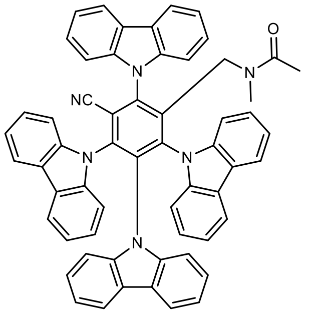

Due to slow conversion, the synthesis was conducted as 4 small scale reactions which were combined for workup. 4 vials were charged with **PC13** (30 µmol, 23.67 mg, 1 equiv.) and 505 mg DABCO (150 equiv., slightly above the solubility limit). The vials were transferred into a glovebox, and 2 mL DMA was added. The samples were irradiated at 445 nm for 4 days. The conversion turned out to be moderate. For isolation, the reaction mixture was diluted with EtOAc, washed with water and brine, dried over Na_2SO_4, and the solvent evaporated under reduced pressure. Recrystallization of the crude mixture from DCM/EtOAc was unsuccessful due to a large amount of unreacted 4CzIPN. Therefore, it was purified by column chromatography using hexane/DCM 1:1 until 4CzIPN eluted, then switched to 100% DCM to elute the product. Hexane/EtOAc mixtures are not recommended due to very low solubility in this solvent mixture. Separation of product from 4CzIPN with hexane/DCM mixtures is feasible. The resulting product had moderate purity after the column. Then, it was recrystallized from DCM/EtOAc by dissolving in a minimal amount of DCM, adding approximately 2 mL EtOAc and concentrating under reduced pressure to a volume of approximately 0.5 mL. Yield: 21% (21 mg).

^{1}H NMR (500 MHz, CD_2Cl_2) δ 8.27 (d, J = 7.7 Hz, 2H), 7.76 (d, J = 7.4 Hz, 6H), 7.66 (d, J = 8.2 Hz, 2H), 7.49 (t, J = 7.5 Hz, 2H), 7.34 (dd, J = 18.2, 7.9 Hz, 4H), 7.22 (d, J = 8.1 Hz, 2H), 7.18 – 7.05 (m, 8H), 7.02 (d, J = 8.2 Hz, 2H), 6.81 (t, J = 7.5 Hz, 2H), 6.70 (t, J = 7.7 Hz, 2H), 4.16 (s, 2H), 1.55 (s, 3H), 1.02 (s, 3H).

^{13}C NMR (126 MHz, CD_2Cl_2) δ 170.8, 142.9, 141.9, 140.7 (2C), 140.5, 139.4, 139.3, 138.5, 136.4, 127.4, 126.1, 126.0, 124.8, 124.5, 124.2, 124.0, 123.7, 121.8, 121.5, 121.4, 121.1, 120.6, 120.5 (2C), 119.6, 117.8, 113.1, 110.8, 110.6, 110.5, 110.2, 109.9, 48.3, 37.0, 21.3..

HRMS (ESI): calculated for $[M+Na]^+$ $C_{59}H_{40}N_6ONa^+$ 871.3156; found 871.3196.

N-methyl-N-(2,3,5,6-tetrakis(bis(4-methoxyphenyl)amino)-4-cyanobenzyl)acetamide (PC13-1)

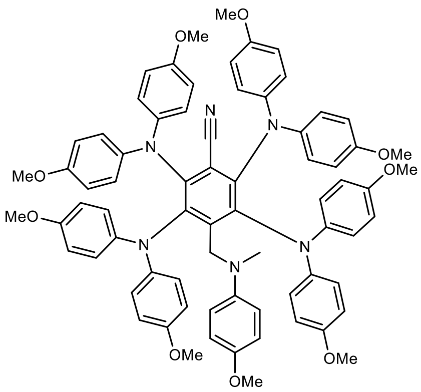

Due to slow conversion, the synthesis was conducted as 4 small scale reactions which were combined for workup. 4 vials were chaged with **PC13** (15 µmol, 15.56 mg, 1 equiv.), *p*-methoxy-*N*,*N*-dimethylaniline (150 µmol, 22.68 mg, 10 equiv.), and DABCO (150 µmol, 16.83 mg, 10 equiv.). The vials were transferred into a glovebox, and 3 mL DMA was added. The reactions were then irradiated at 620 nm for 3 days. After the reaction was completed (evident from a color change to yellow/orange), the content of the vials was diluted with EtOAc (approximately 50 mL), washed with H_2O and brine, dried over Na_2SO_4 and the solvent evaporated under reduced pressure. The residue was washed with hexane to remove most of the excess aniline. The crude product was then purified via column chromatography (PE/EtOAc). Recrystallization from DCM/MeCN is possible and yields the product as a crystalline, orange solid. Yield: 75% (52 mg).

Note: the compound is light-sensitive in solution and turns brown under ambient conditions within hours.

^{1}H NMR (500 MHz, CD_2Cl_2) δ 6.74 – 6.63 (m, 16H), 6.62 – 6.56 (m, 8H), 6.54 – 6.48 (m, 10H), 6.16 – 6.10 (m, 2H), 3.71 (s, 12H), 3.67 (s, 12H), 3.64 (s, 3H), 3.29 (s, 2H), 2.50 (s, 3H).

^{13}C NMR (126 MHz, CD_2Cl_2) δ 171.2, 155.4 (2C), 154.9, 154.8, 153.8, 147.6, 147.2, 147.0, 145.6, 143.2, 142.4, 139.8 (2C), 123.3, 123.2, 122.3, 121.6, 119.8, 118.2, 115.5, 114.3, 114.2, 114.0, 113.8, 60.6, 55.7 (2C), 50.8, 44.4..

HRMS (ESI): calculated for $[M]^+$ $C_{72}H_{68}N_6O_9^+$ 1160.5043; found 1160.5088.

***N*-methyl-*N*-(2,3,5,6-tetrakis(bis(4-methoxyphenyl)amino)- 4-cyanobenzyl)acetamide (PC13-2)**

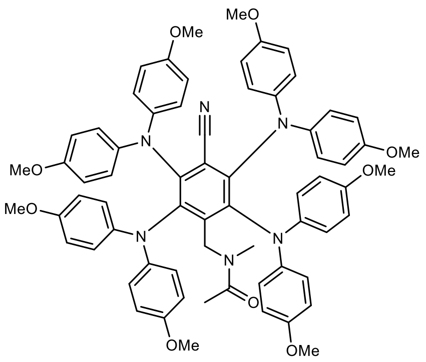

Due to slow conversion, the synthesis was conducted as 6 small scale reactions which were combined for workup. 6 vials were charged with **PC13** (10 µmol, 10.38 mg, 1 equiv.), DABCO (336.5 mg, 300 equiv., almost saturated solution). The vials were transferred into a glovebox, and 2 mL DMA was added. The samples were irradiated for 4 days at 620 nm. It showed moderate conversion, according to TLC. For isolation, the reaction mixture was diluted with EtOAc, washed with water and brine, dried over Na_2SO_4, and the solvent evaporated under reduced pressure. The residue was purified by flash chromatography on silica gel using 100% DCM to elute the remaining **PC13**, then 20→50% EtOAc in DCM to elute the product. Later, the concentrated product was crystallized by dissolving in a minimal amount of DCM, adding approximately 2 mL EtOAc, and concentrating under reduced pressure to a volume of approximately 1 mL. Yellow/orange, crystalline solid. Yield: 58% (38 mg). X-ray quality crystal **PC13-2** was grown from DCM/EtOAc solution by slow evaporation. CCDC deposit number is 2370570.

^{1}H NMR (500 MHz, CD_2Cl_2) δ 6.78 (d, J = 8.9 Hz, 8H), 6.72 – 6.56 (m, 24H), 3.80 (s, 2H), 3.70 (s, 12H), 3.68 (s, 12H), 2.09 (s, 3H), 1.68 (s, 3H).

^{13}C NMR (126 MHz, CD_2Cl_2) δ 171.8, 155.0, 154.4, 147.3, 145.7, 142.1, 139.3, 139.0, 122.8, 121.7, 118.4, 115.0, 113.9, 113.7, 113.5, 55.4, 55.3, 48.9, 38.1, 22.7.

HRMS (ESI): calculated for $[M+Na]^+$ $C_{67}H_{64}N_6O_9Na^+$ 1119.4627; found 1119.4647.

2,3,5,6-tetrakis(bis(4-methoxyphenyl)amino)benzonitrile (PC13-3)

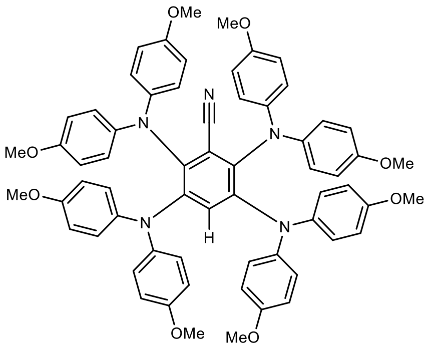

20 ml vial was charged with **PC13 (**30 µmol, 31.1 mg, 1 equiv), [2.2.2]cryptand (60 µmol, 22.6 mg, 2 equiv). The vial was transferred into a glovebox, KC_8 (15 µmol, 20.3 mg, 5 equiv) and 10 mL DMA were added. The reaction mixture was stirred for 24 hours, resulting in the green solution turning yellow after contact with oxygen. The solution was diluted with EtOAc, washed with water and brine, dried over Na_2SO_4, and the solvent evaporated under reduced pressure. It was recrystallized by dissolving in a minimum amount of DCM, adding about 2 mL EtOAc, and concentrating under reduced pressure until approximately 1 mL solvent was left. The product crystallized from the solution as yellow/orange crystalline solid. Yield: 86% (26 mg). X-ray quality crystal **PC13-3** was grown from DCM/EtOAc solution by slow evaporation. CCDC deposit number is 2370594.

^{1}H NMR (500 MHz, CD_2Cl_2) δ 6.79 (s, 1H), 6.75 – 6.65 (m, 16H), 6.65 – 6.54 (m, 16H), 3.72 (s, 12H), 3.68 (s, 12H).

^{13}C NMR (126 MHz, CD_2Cl_2) δ 155.8, 155.3, 143.7, 141.8, 140.9, 139.8, 134.3, 124.5, 122.9, 121.9, 115.5, 114.3, 114.1, 55.7 (2C).

HRMS (ESI): calculated for $[M]^+$ $C_{63}H_{57}N_5O_8^+$ 1011.4202; found 1011.4215.

5.5.2. Crystal structure determination for photocatalysts

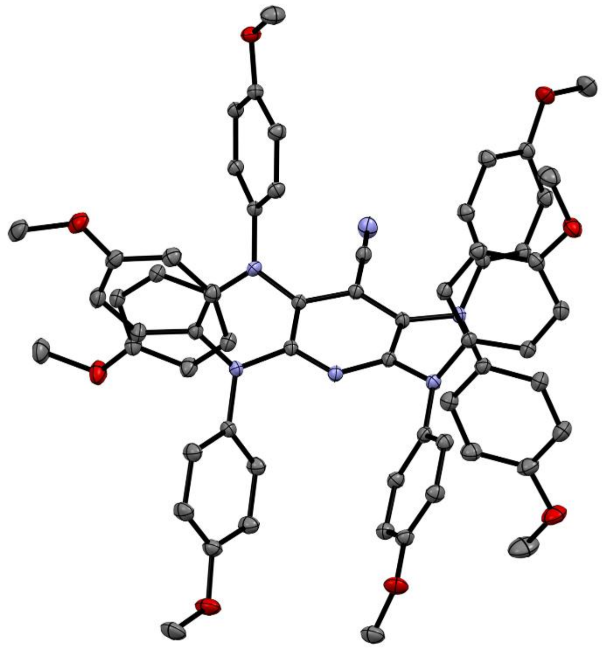

Figure S241. Thermal ellipsoid representation (50% probability ellipsoids) of the molecular structure of **PC11.** All hydrogen atoms and CH_2Cl_2 molecules are omitted for clarity.

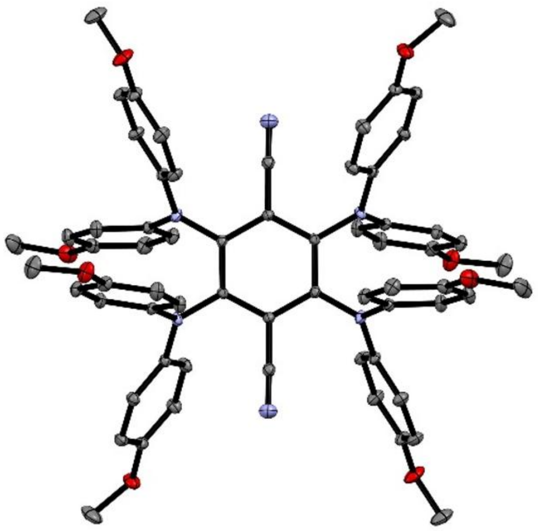

Figure S242. Thermal ellipsoid representation (50% probability ellipsoids) of the molecular structure of **PC13.** All hydrogen atoms and CH_2Cl_2 molecule are omitted for clarity.

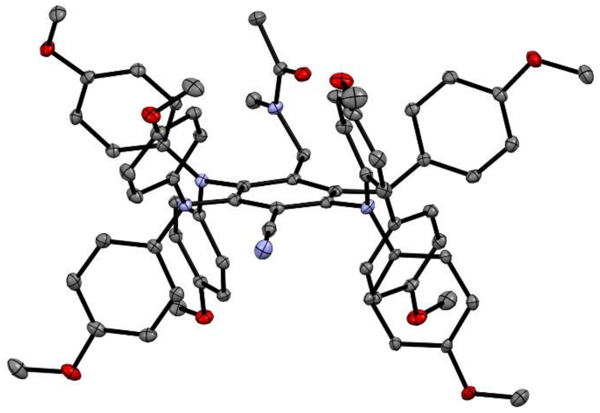

Figure S243. Thermal ellipsoid representation (30% probability ellipsoids) of the molecular structure of **PC13-2.** All hydrogen atoms are omitted for clarity.

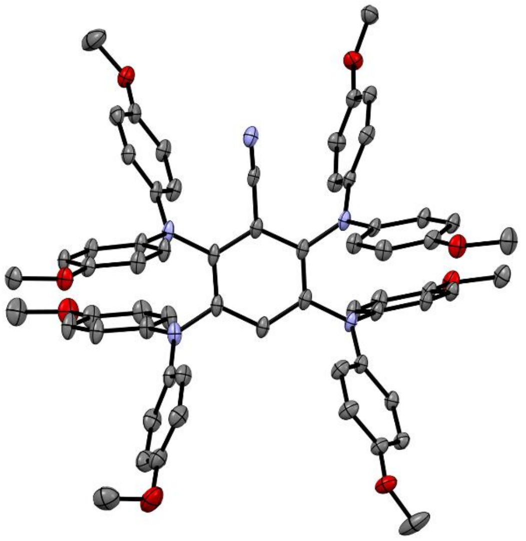

Figure S244. Thermal ellipsoid representation (50% probability ellipsoids) of the molecular structure of **PC13-3.** All hydrogen atoms are omitted for clarity.

X-ray quality crystal was immersed in cryo-oil, mounted in a Nylon loop, and measured at 100 K. Intensity data were collected using a Bruker D8 Venture and Bruker D8 Metaljet diffractometer with monochromated Mo−Kα (λ = 0.71073 Å) and Ga−Kα (λ = 1.34138 Å) radiation, respectively. Cell parameters were retrieved and refined using Bruker SAINT[20] on all the observed reflections. Data were corrected for absorption effects using the multi-scan method (SADABS)[20]. Structures were solved by direct methods using SHELXT[21] and refined with SHELXL[22] and Olex-2[23] software. All hydrogen atoms in the compound were inserted in geometrically calculated positions with fixed thermal parameters. All the hydrogen atoms were refined isotropically, while all the nonhydrogen atoms were refined anisotropically. In **PC13-3** the nitrile group of the core aromatic ring is disordered. It was treated with SADI command on C-CN and C≡N bonds, and with DELU on the atoms of the nitrile group. In **PC13-2,** one of the *p*-methoxyphenyl rings is disordered. It was treated with SADI on the distances between all aromatic ring carbons and distances of C-O-

Me bonds. SIMU command was applied to the all aromatic ring atoms and methoxy group atoms. A solvent mask was calculated for **PC13-3** with Olex-2[23-24], recovering a density of 308 electrons per unit cell, corresponding to 6 ethylacetate molecules per unit cell. The final least-squares refinements (R_1) for **PC13/PC13-2/PC11/PC13-3** based on I >2σ(I) converged to 0.0506/0.0568/0.0625/0.1140, respectively.

Table S24. Crystal data and structure refinement for PC11 and PC13.

**Identification code**	**PC11**	**PC13**
Empirical formula	$C_{63}H_{58}Cl_2N_6O_8$	$C_{66}H_{60}Cl_4N_6O_8$
Formula weight	1098.05	1207.00
Temperature/K	100.00	100.00
Crystal system	monoclinic	triclinic
Space group	$P2_1/c$	P-1
a/Å	13.2915(16)	11.9019(7)
b/Å	12.2807(15)	12.2312(8)
c/Å	34.425(4)	12.8100(8)
α/°	90	69.489(2)
β/°	100.043(3)	67.054(2)
γ/°	90	63.313(2)
Volume/Å^3	5533.0(12)	1499.09(17)
Z	4	1
ρ_{calc}g/cm^3	1.318	1.337
μ/mm^{-1}	1.024	0.259
F(000)	2304.0	630.0
Crystal size/mm^3	0.1 × 0.1 × 0.1	0.271 × 0.194 × 0.181
Radiation	GaKα (λ = 1.34138)	MoKα (λ = 0.71073)
2Θ range for data collection/°	4.536 to 147.532	3.534 to 64.92
Index ranges	-18 ≤ h ≤ 18, -16 ≤ k ≤ 17, -48 ≤ l ≤ 47	-17 ⩽ h ⩽ 17, -18 ⩽ k ⩽ 18, -19 ⩽ l ⩽ 19
Reflections collected	154041	80728
Independent reflections	15438 [R_{int} = 0.0755, R_{sigma} = 0.0614]	10785 [R_{int} = 0.0918, R_{sigma} = 0.0515]
Data/restraints/parameters	15438/0/720	10785/0/383
Goodness-of-fit on F^2	1.124	1.019
Final R indexes [I>=2σ (I)]	R_1 = 0.0625, wR_2 = 0.1631	R_1 = 0.0506, wR_2 = 0.1287
Final R indexes [all data]	R_1 = 0.0683, wR_2 = 0.1675	R_1 = 0.0706, wR_2 = 0.1445
Largest diff. peak/hole / e Å^{-3}	0.89/-1.11	0.48/-0.9

Table S25. Crystal data and structure refinement for PC13-2 and PC13-3.

**Identification code**	**PC13-2**	**PC13-3**
Empirical formula	$C_{67}H_{64}N_6O_9$	$C_{69}H_{69}N_5O_{11}$ ($C_{63}H_{57}N_5O_8 \cdot 1.5C_4H_8O_2$)
Formula weight	1097.24	1144.29
Temperature/K	100.00	100.00
Crystal system	triclinic	monoclinic
Space group	P-1	$P2_1/c$
a/Å	12.9384(10)	11.9545(10)
b/Å	13.5693(10)	21.2188(19)
c/Å	17.7293(14)	24.139(2)
α/°	72.955(4)	90
β/°	73.052(3)	93.819(4)
γ/°	72.394(3)	90
Volume/Å^3	2765.4(4)	6109.5(9)
Z	2	4
ρ_{calc}g/cm^3	1.318	1.244
μ/mm^{-1}	0.453	0.440
F(000)	1160.0	2424.0
Crystal size/mm^3	0.266 × 0.233 × 0.131	0.3 × 0.2 × 0.2
Radiation	GaKα (λ = 1.34138)	GaKα (λ = 1.34139)
2Θ range for data collection/°	4.652 to 113.998	4.828 to 144.702
Index ranges	$-16 \le h \le 15$, $-16 \le k \le 16$, $-22 \le l \le 22$	$-16 \leqslant h \leqslant 16$, $-27 \leqslant k \leqslant 30$, $-33 \leqslant l \leqslant 34$
Reflections collected	126211	243456
Independent reflections	11275 [R_{int} = 0.1389, R_{sigma} = 0.0841]	18104 [R_{int} = 0.0848, R_{sigma} = 0.0518]
Data/restraints/parameters	11275/58/823	18104/5/712
Goodness-of-fit on F^2	1.112	1.034
Final R indexes [I>=2σ (I)]	R_1 = 0.0568, wR_2 = 0.1489	R_1 = 0.1138, wR_2 = 0.3171
Final R indexes [all data]	R_1 = 0.0901, wR_2 = 0.1557	R_1 = 0.1274, wR_2 = 0.3289
Largest diff. peak/hole / e Å^{-3}	0.88/-0.39	1.48/-0.70

5.5.3. Photophysical data of the photocatalysts decomposition products

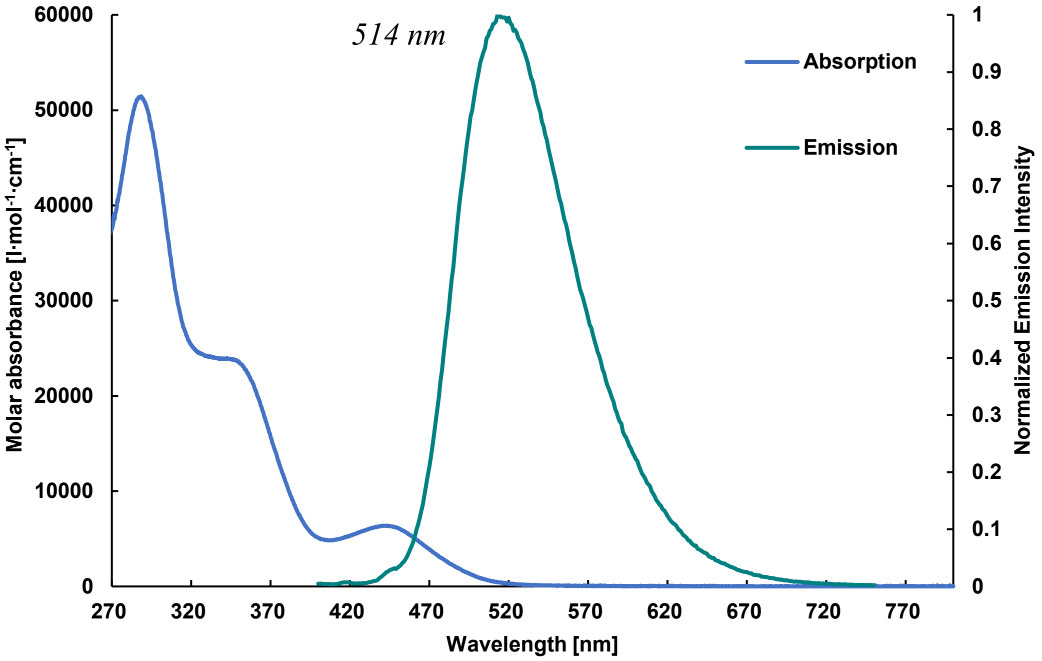

Figure S245. UV/Vis absorption and emission spectrum of **PC13-1** in degassed DMA (20 µM). Excitation wavelength – 394 nm.

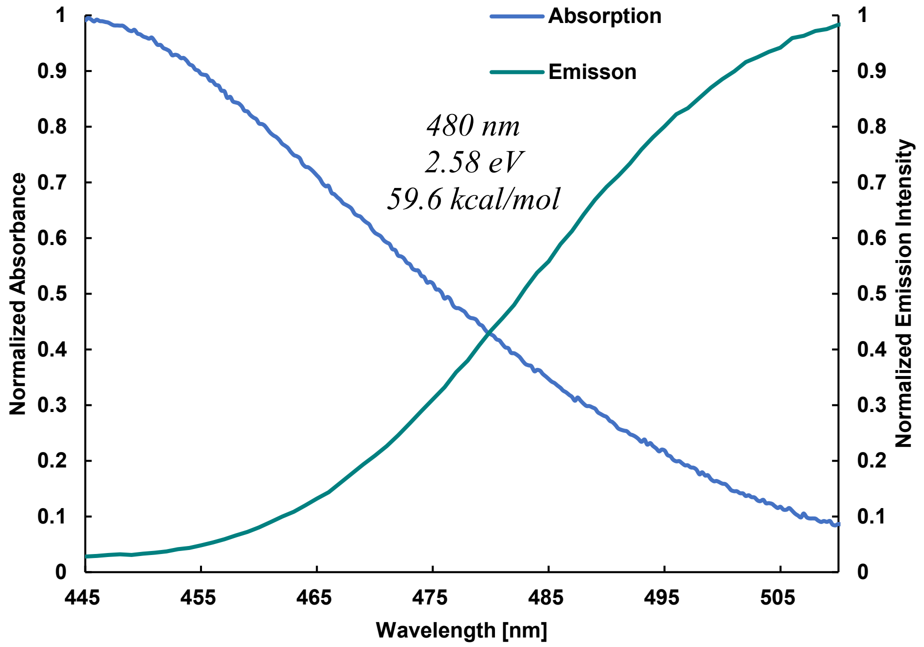

Figure S246. $E_{0\text{-}0}$ estimation at normalized emission and absorption overlap of **PC13-1**.

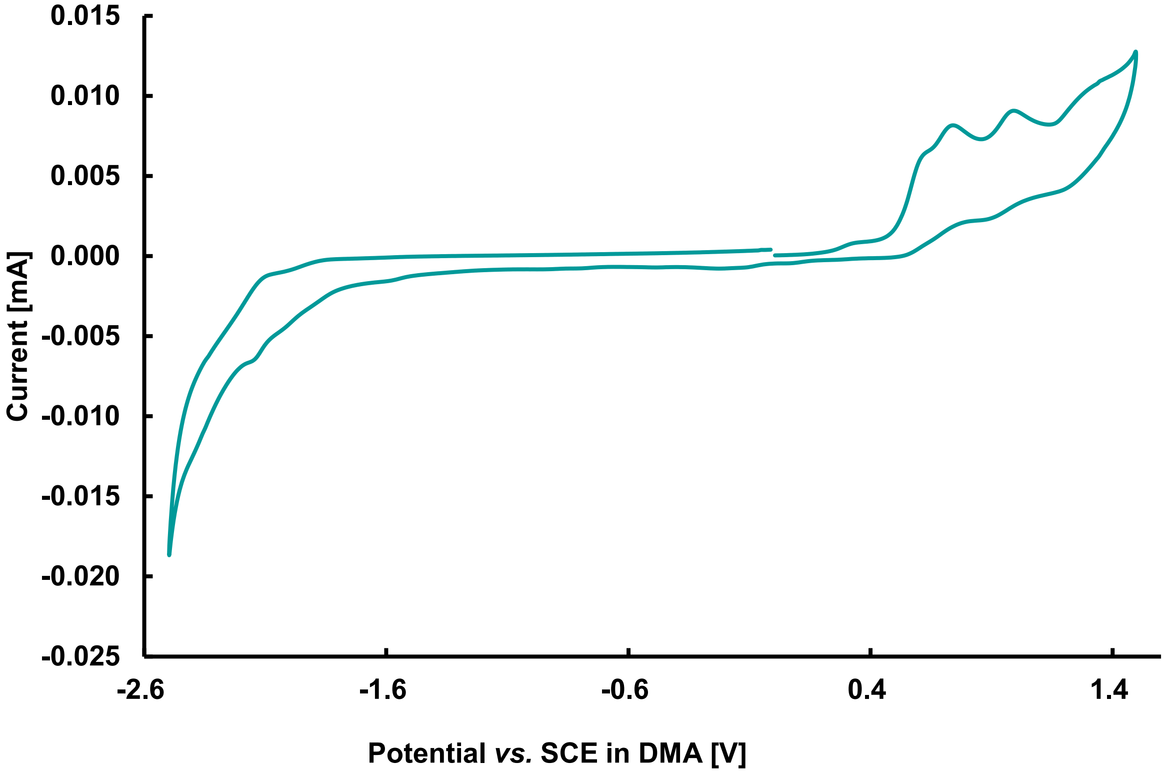

Figure S247. Cyclic voltammetry of **PC13-1** in degassed DMA (0.4 mM) using 0.1 M $^{n}Bu_4NPF_6$ as electrolyte. The scan rate is 50 mV/sec.

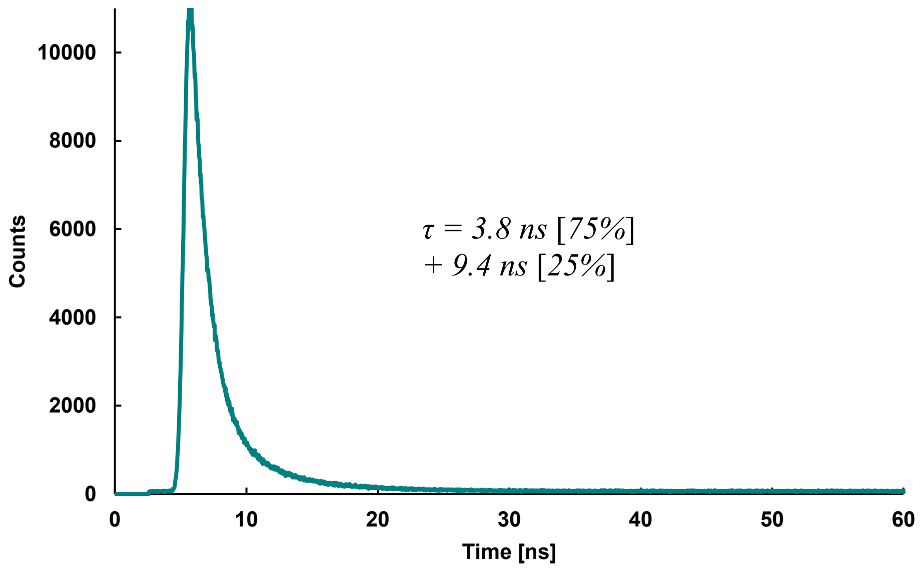

Figure S248. Fluorescence decay curve of **PC13-1** in degassed DMA (20 μM). Excitation wavelength – 394 nm.

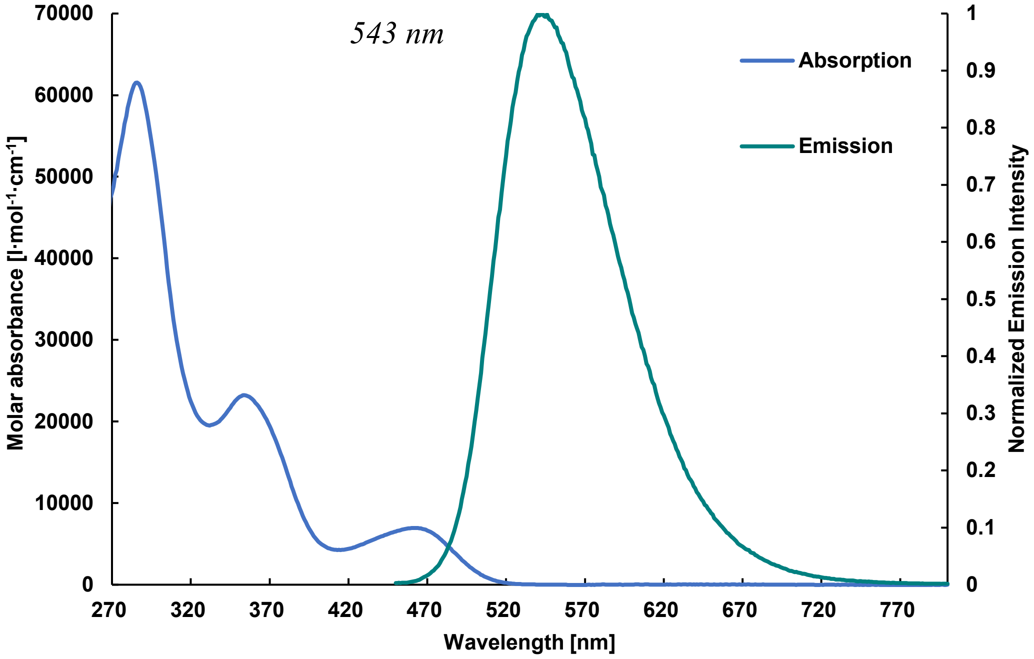

Fig S249. UV/Vis absorption and emission spectrum of **PC13-2** in degassed DMA (20 µM). Excitation wavelength – 440 nm.

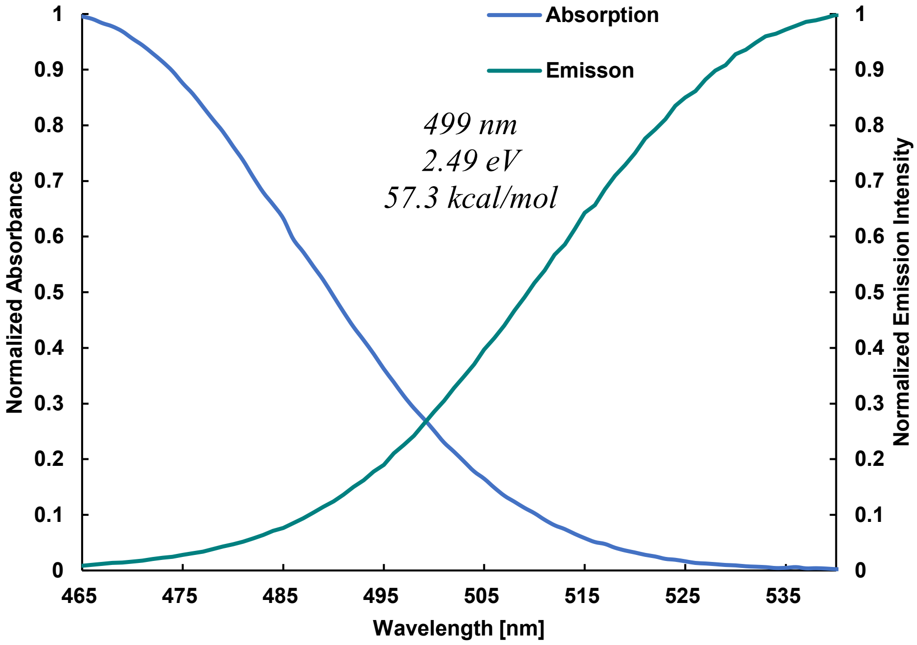

Fig S250. $E_{0\text{-}0}$ estimation at normalized emission and absorption overlap of **PC13-2**.

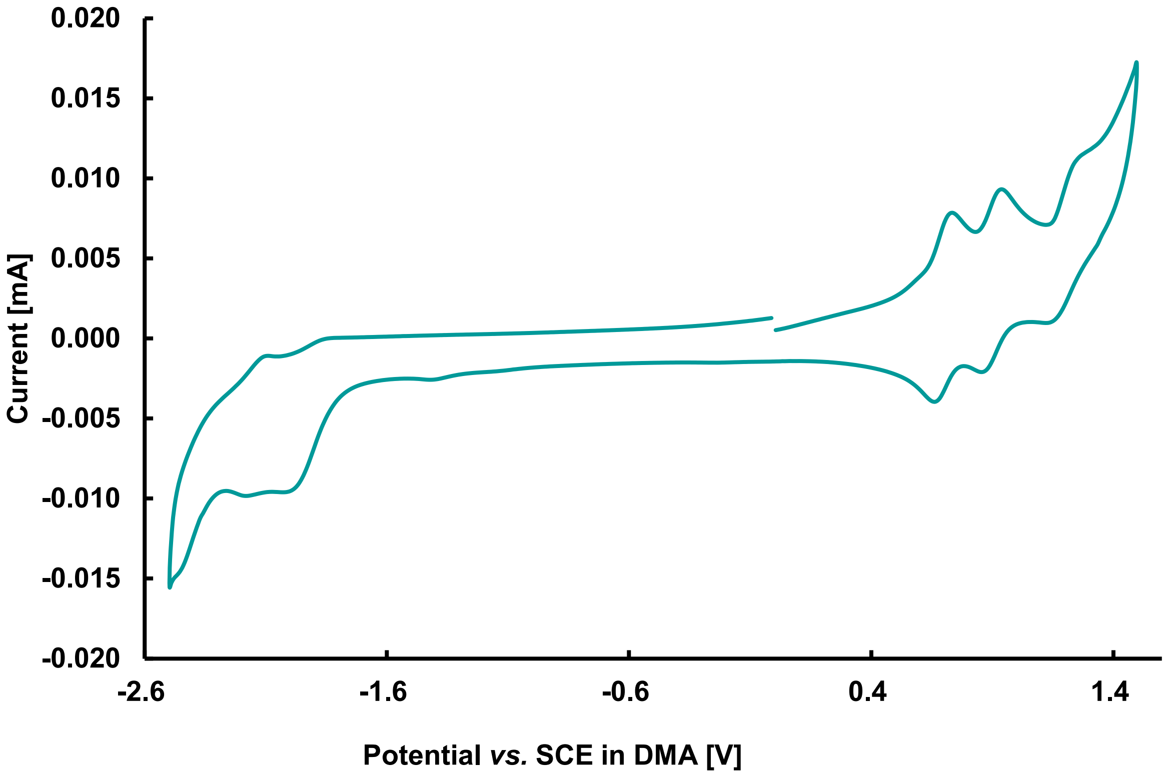

Fig S251. Cyclic voltammetry of **PC13-2** in degassed DMA (0.4 mM) using 0.1 M $^{n}Bu_4NPF_6$ as electrolyte. The scan rate is 50 mV/sec.

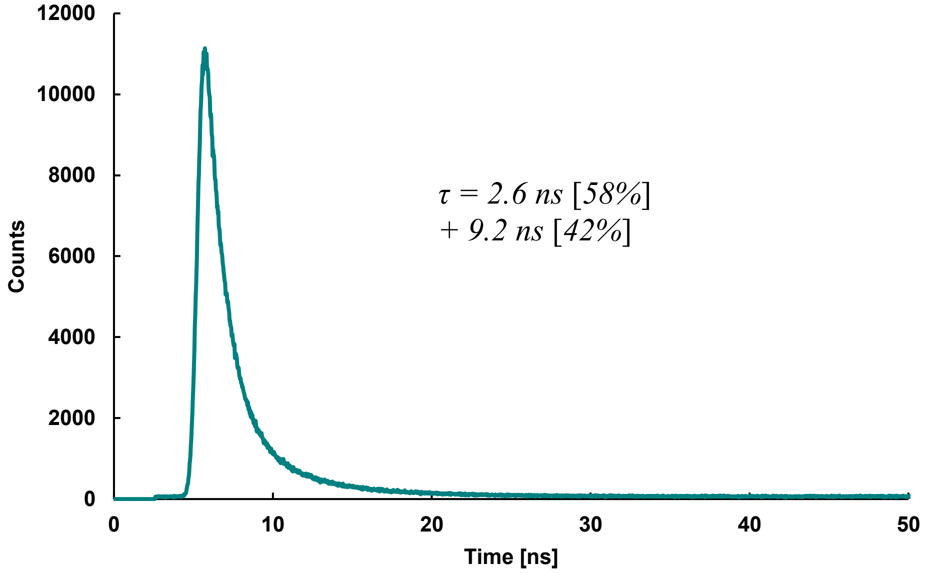

Fig S252. Fluorescence decay curve of **PC13-2** in degassed DMA (20 µM). Excitation wavelength – 394 nm.

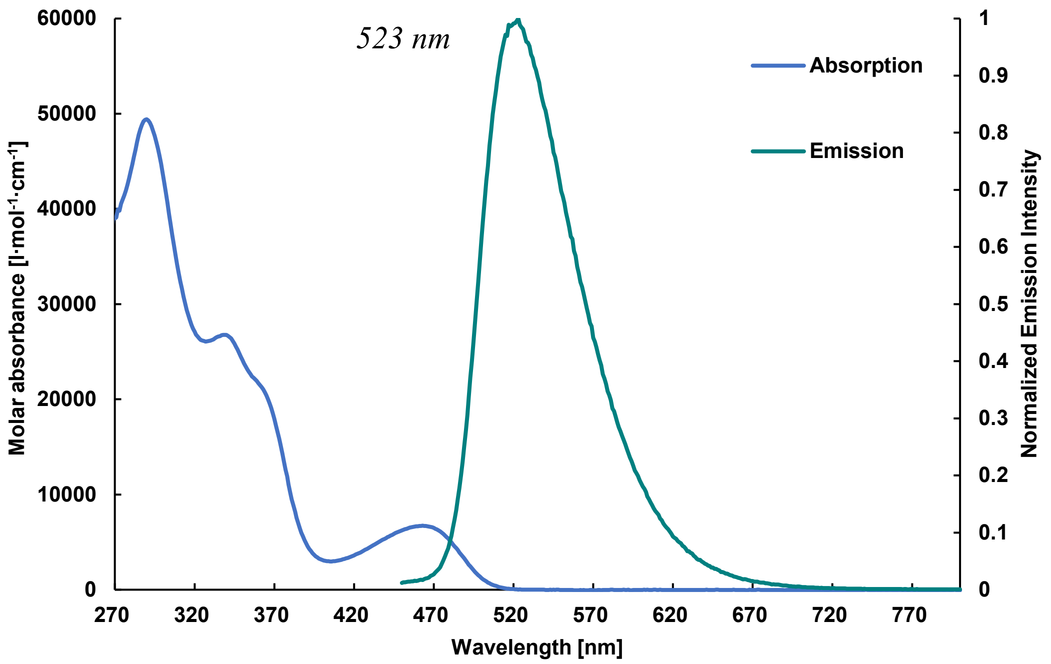

Fig S235. UV/Vis absorption and emission spectrum of **PC13-3** in degassed DMA (20 µM). Excitation wavelength – 440 nm.

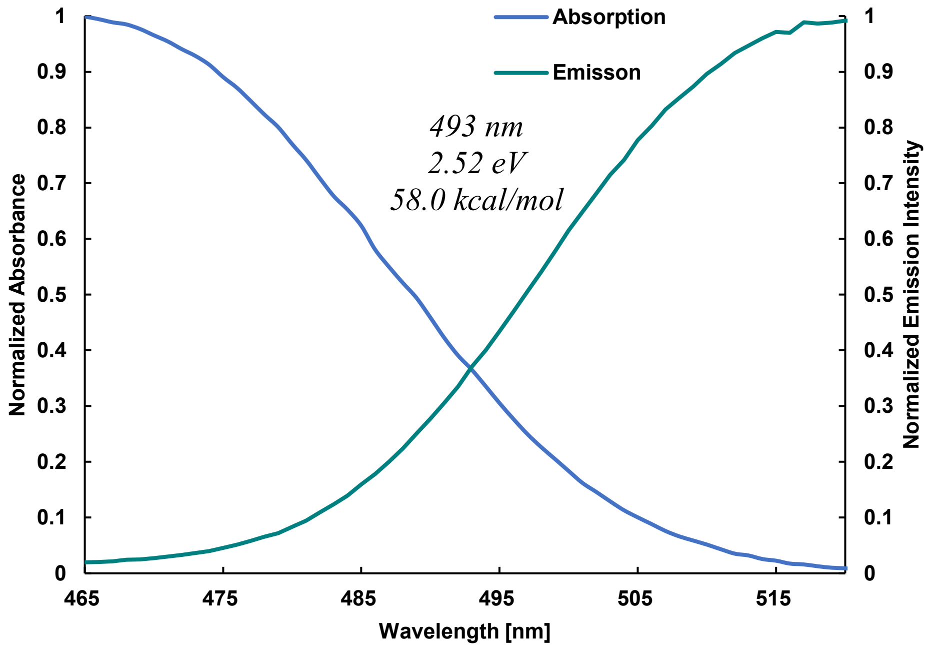

Fig S236. $E_{0\text{-}0}$ estimation at normalized emission and absorption overlap of **PC13-3**.

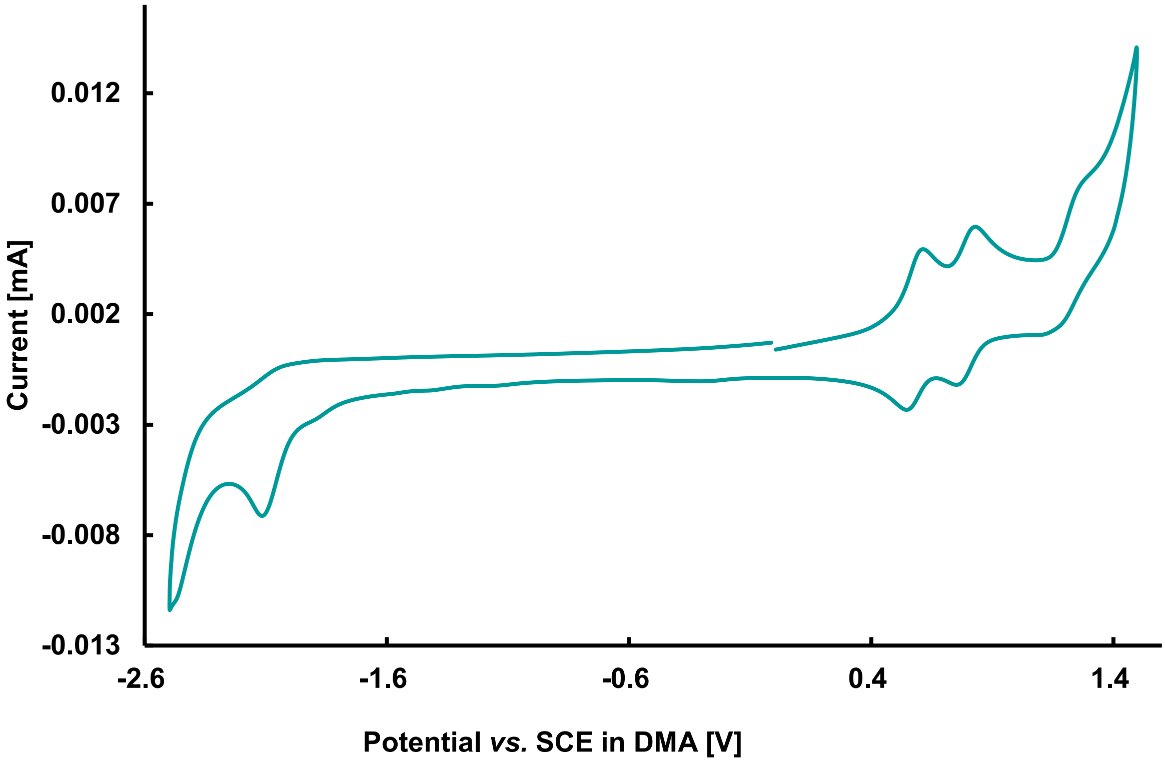

Fig S237. Cyclic voltammetry of **PC13-3** in degassed DMA (0.4 mM) using 0.1 M $^{n}Bu_4NPF_6$ as electrolyte. The scan rate is 50 mV/sec.

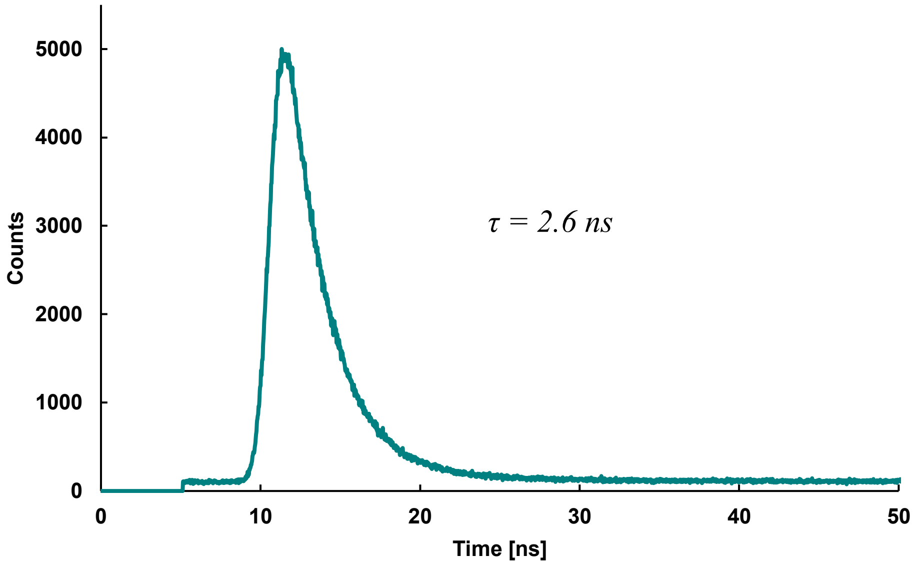

Fig S238. Fluorescence decay curve of **PC13-3** in degassed DMA (20 µM). Excitation wavelength – 394 nm.

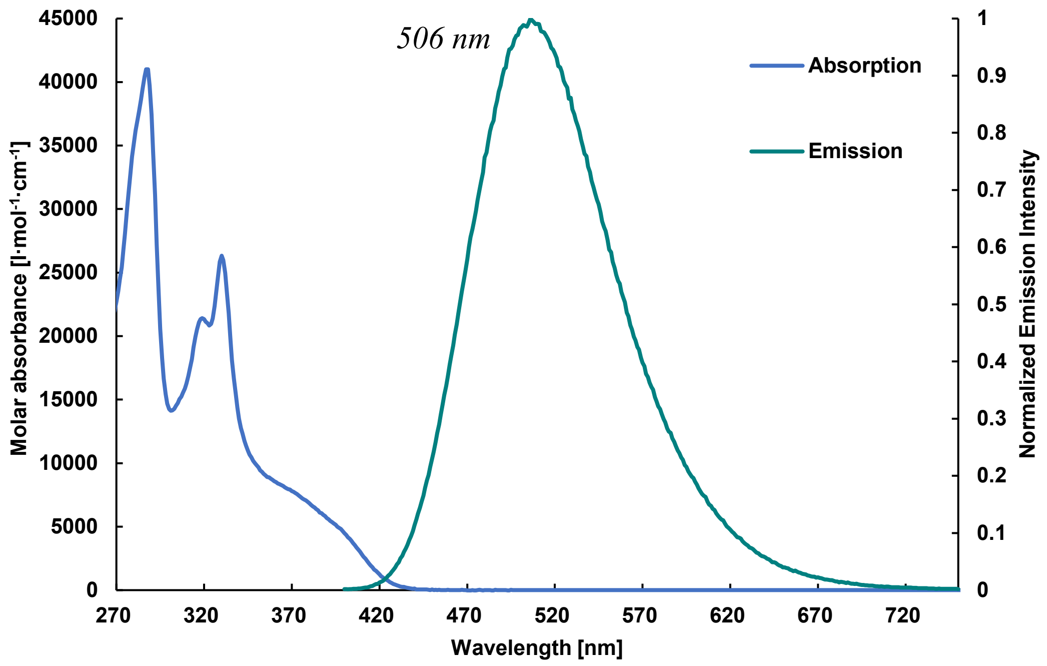

Fig S239. UV/Vis absorption and emission spectrum of **PC1-1** in degassed MeCN (20 μM). Excitation wavelength – 394 nm.

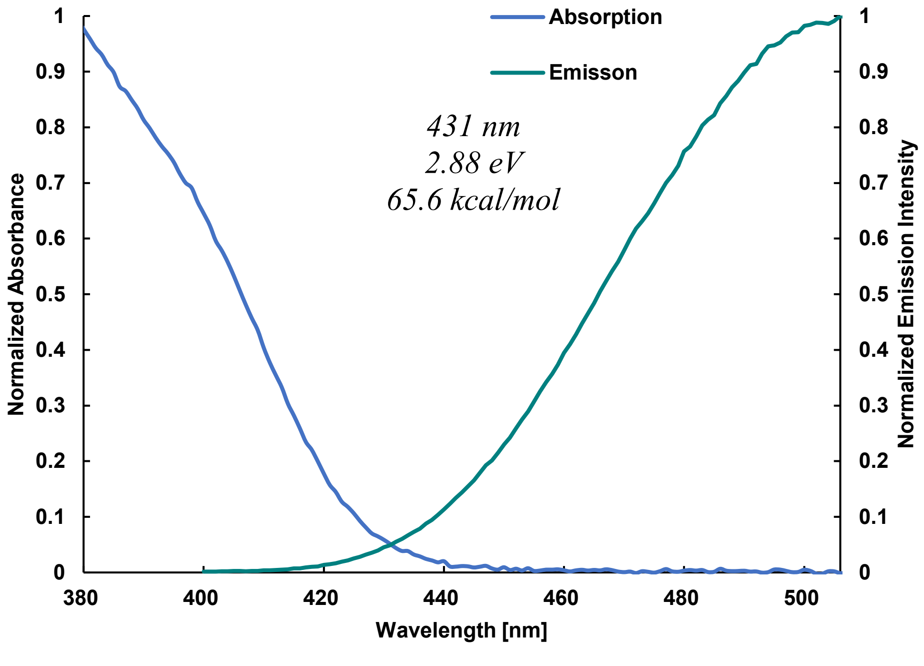

Fig S240. $E_{0\text{-}0}$ estimation at normalized emission and absorption overlap of **PC1-1**.

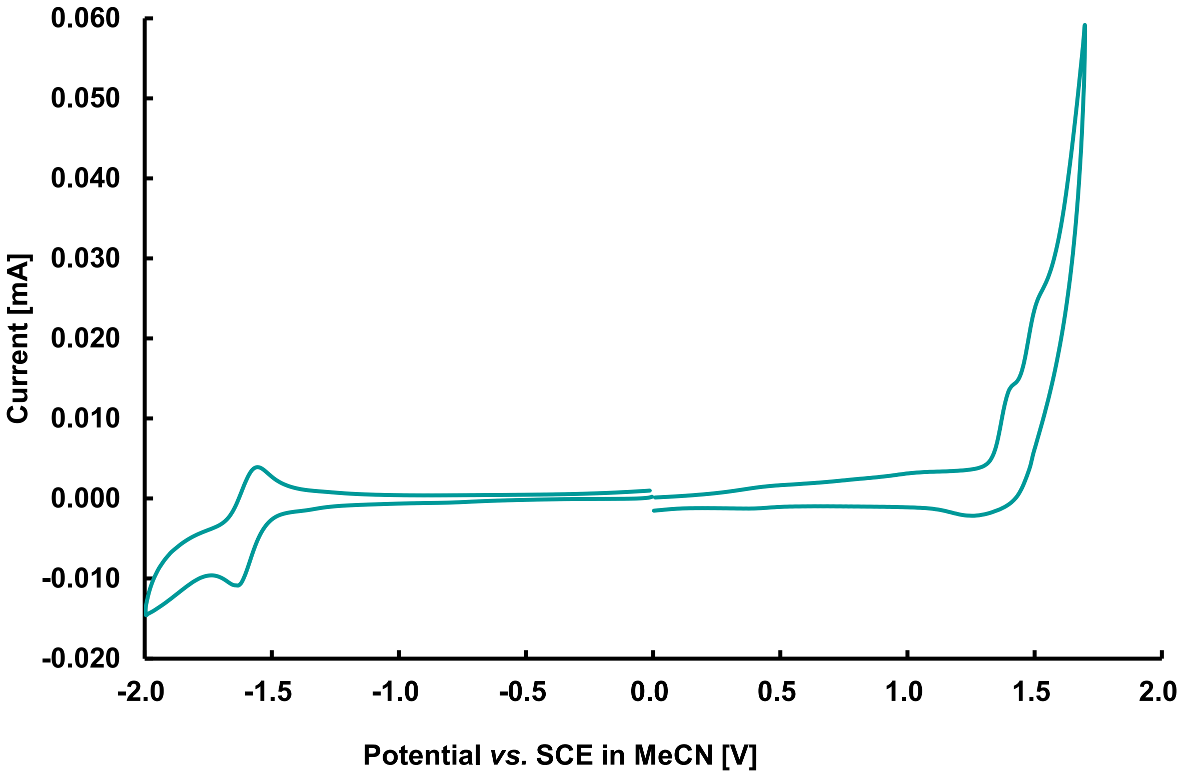

Fig S241. Cyclic voltammetry of **PC1-1** in degassed MeCN (0.4 mM) using 0.1 M $^{n}Bu_4NPF_6$ as electrolyte.

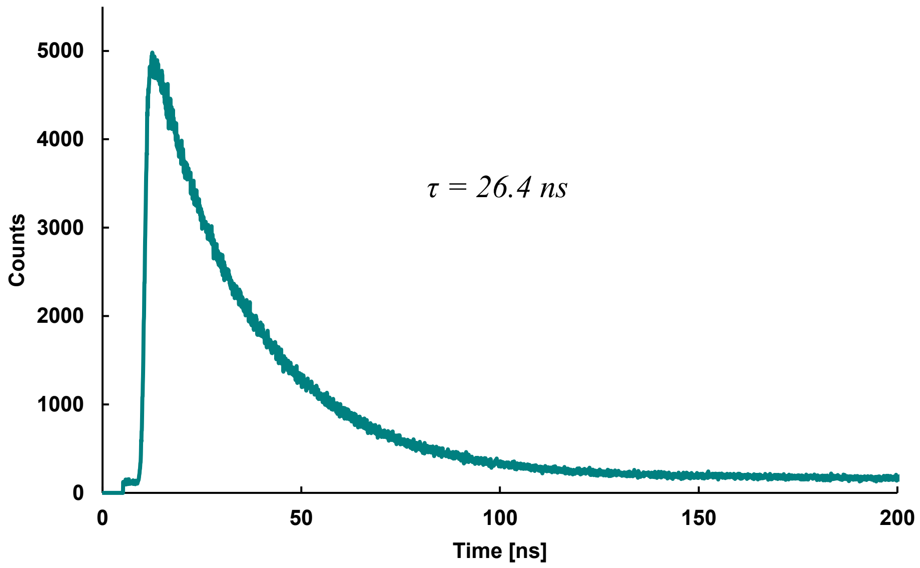

Fig S242. Fluorescence decay curve of **PC1-1** in degassed MeCN (20 µM). Excitation wavelength – 394 nm.

5.5.3. Cyclic voltammetry of organic base and substrates

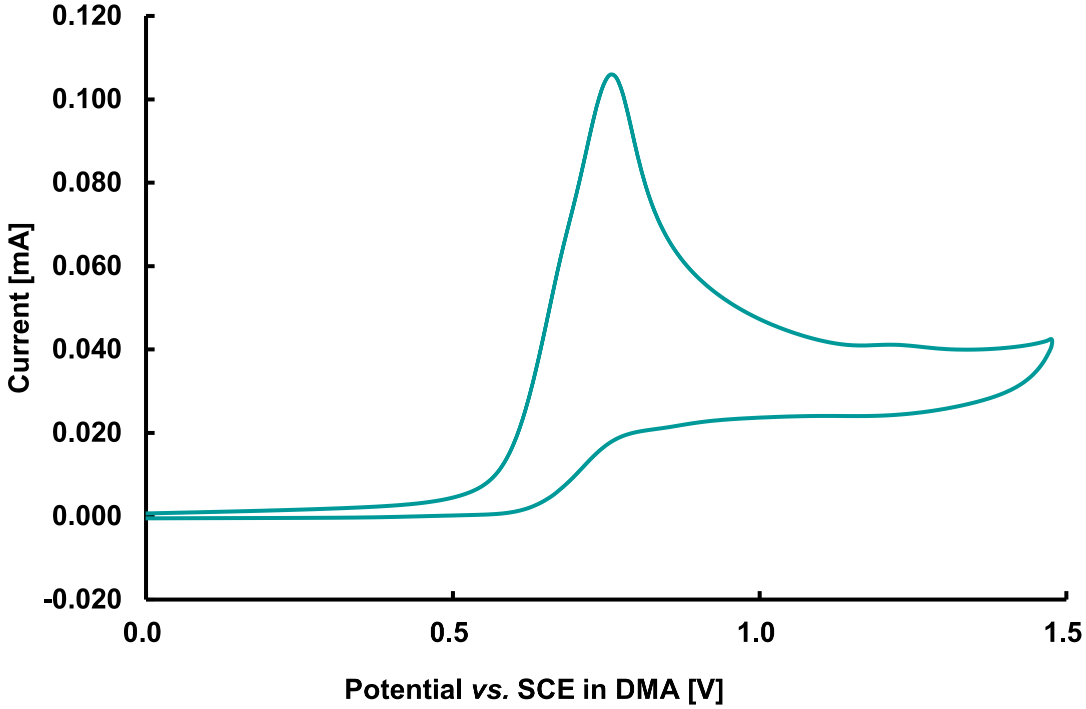

Figure S249. Cyclic voltammetry of **DABCO** in degassed DMA (10 mM) using 0.1 M $^{n}Bu_4NPF_6$ as electrolyte. The scan rate is 50 mV/sec.

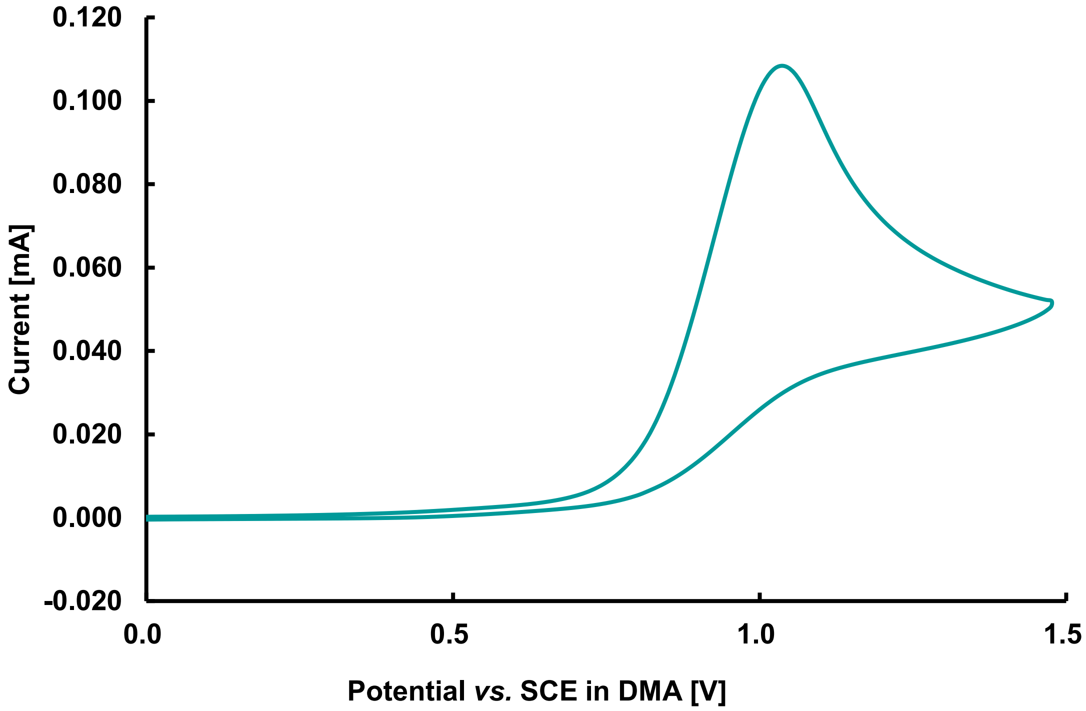

Figure S250. Cyclic voltammetry of **Et_3N** in degassed DMA (10 mM) using 0.1 M $^{n}Bu_4NPF_6$ as electrolyte. The scan rate is 50 mV/sec.

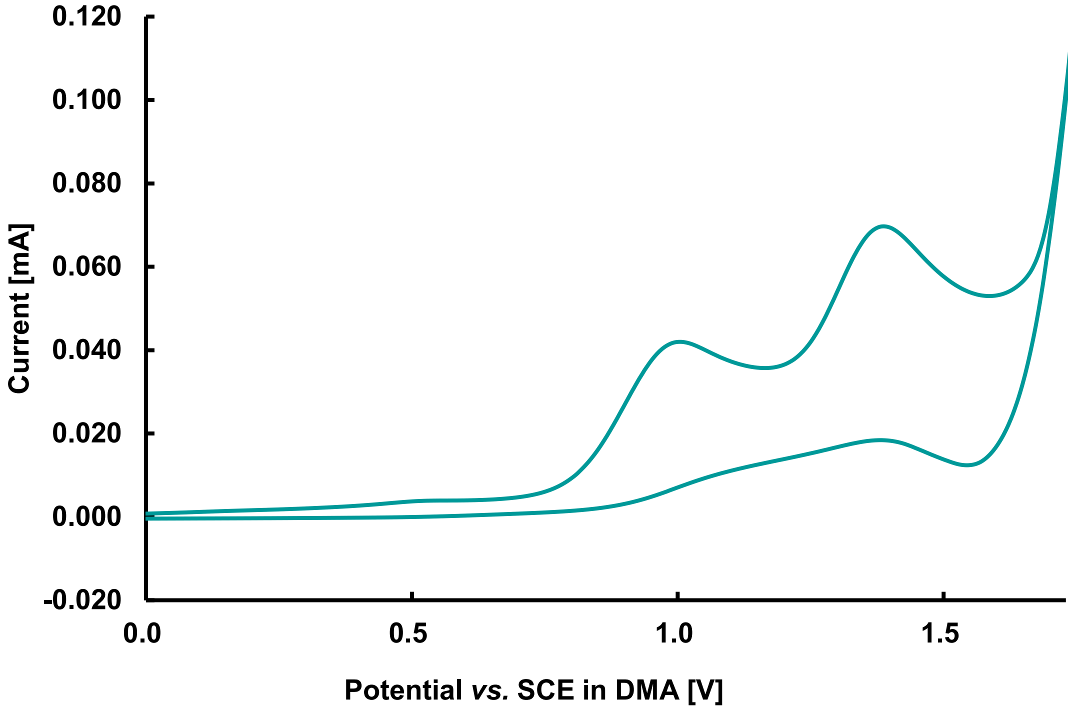

Figure S251. Cyclic voltammetry of **TMG** in degassed DMA (10 mM) using 0.1 M $^{n}Bu_4NPF_6$ as electrolyte. The scan rate is 50 mV/sec.

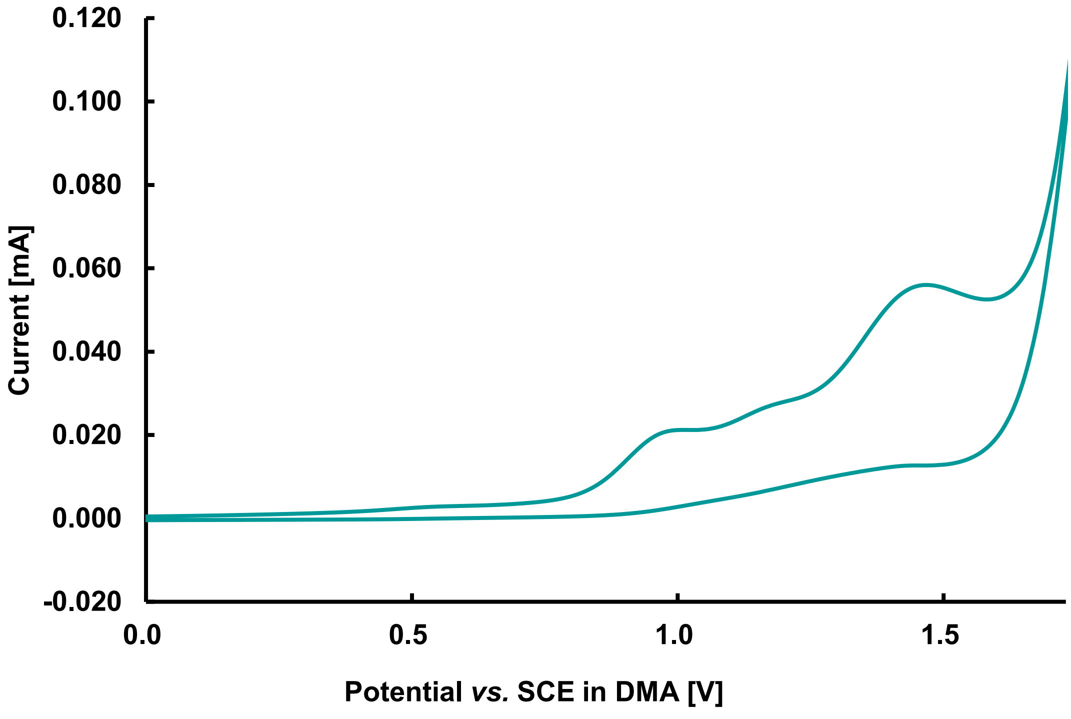

Figure S252. Cyclic voltammetry of **CyNH₂** in degassed DMA (10 mM) using 0.1 M $^{n}Bu_4NPF_6$ as electrolyte. The scan rate is 50 mV/sec.

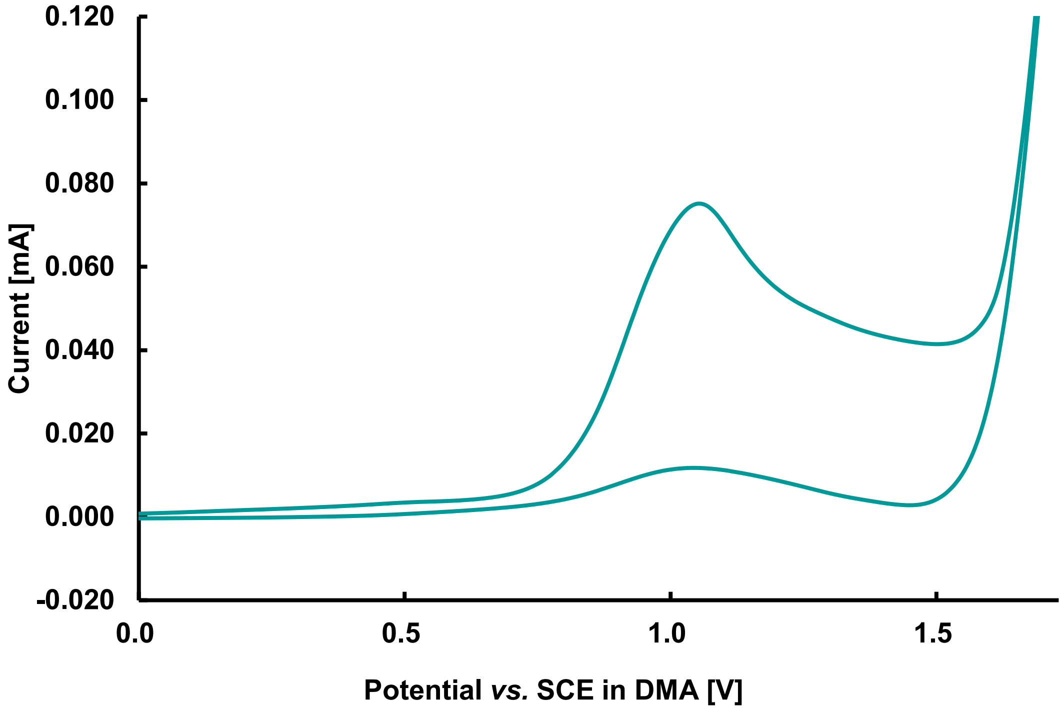

Figure S253. Cyclic voltammetry of **BTMG** in degassed DMA (10 mM) using 0.1 M $^{n}Bu_4NPF_6$ as electrolyte. The scan rate is 50 mV/sec.

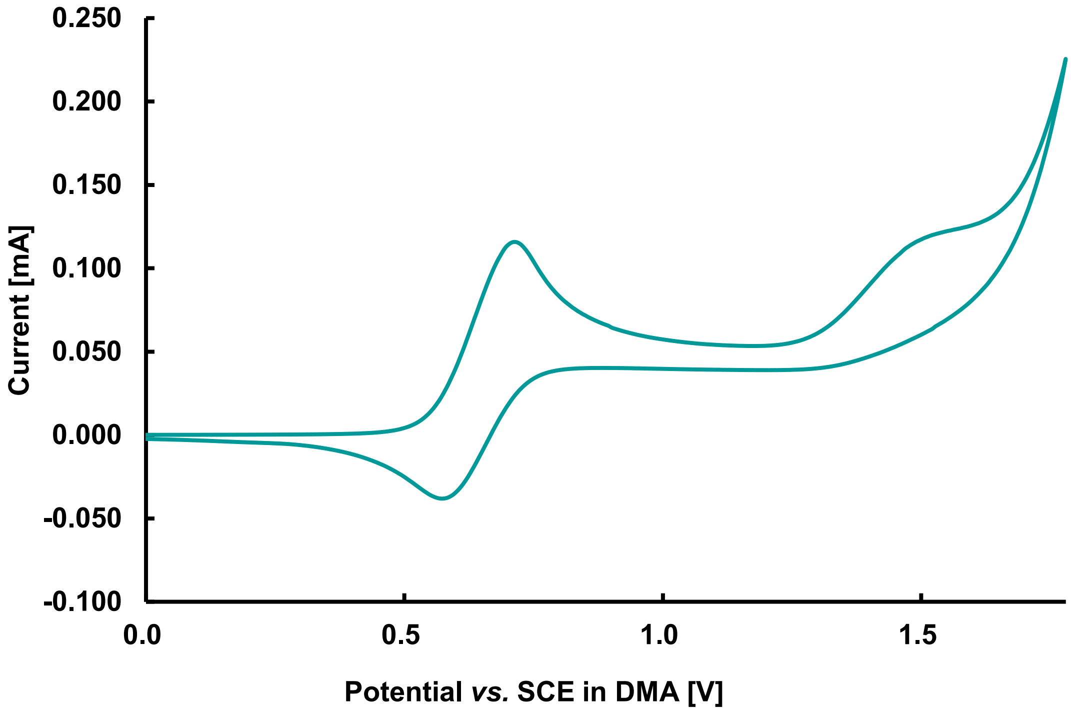

Figure S254. Cyclic voltammetry of ***p*-MeOPhNMe$_2$** in degassed DMA (10 mM) using 0.1 M $^{n}Bu_4NPF_6$ as electrolyte. The scan rate is 50 mV/sec.

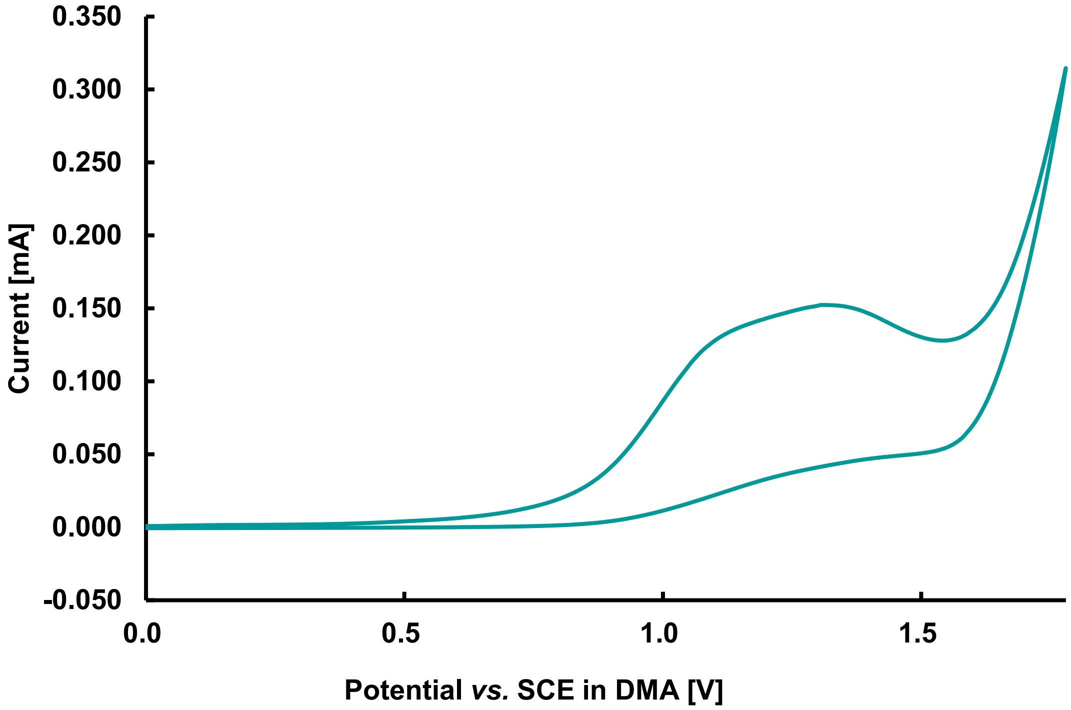

Figure S255. Cyclic voltammetry of **PhSH** in degassed DMA (10 mM) using 0.1 M $^{n}Bu_4NPF_6$ as electrolyte. The scan rate is 50 mV/sec.

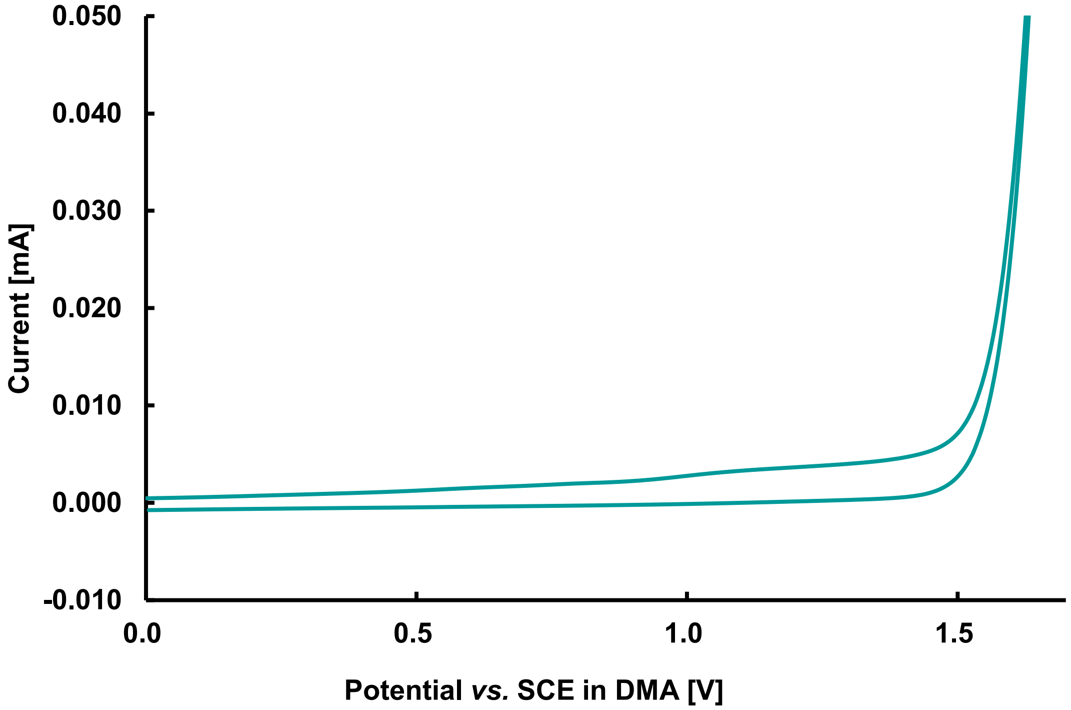

Figure S256. Cyclic voltammetry of **Py** in degassed DMA (10 mM) using 0.1 M $^{n}Bu_4NPF_6$ as electrolyte. The scan rate is 50 mV/sec.

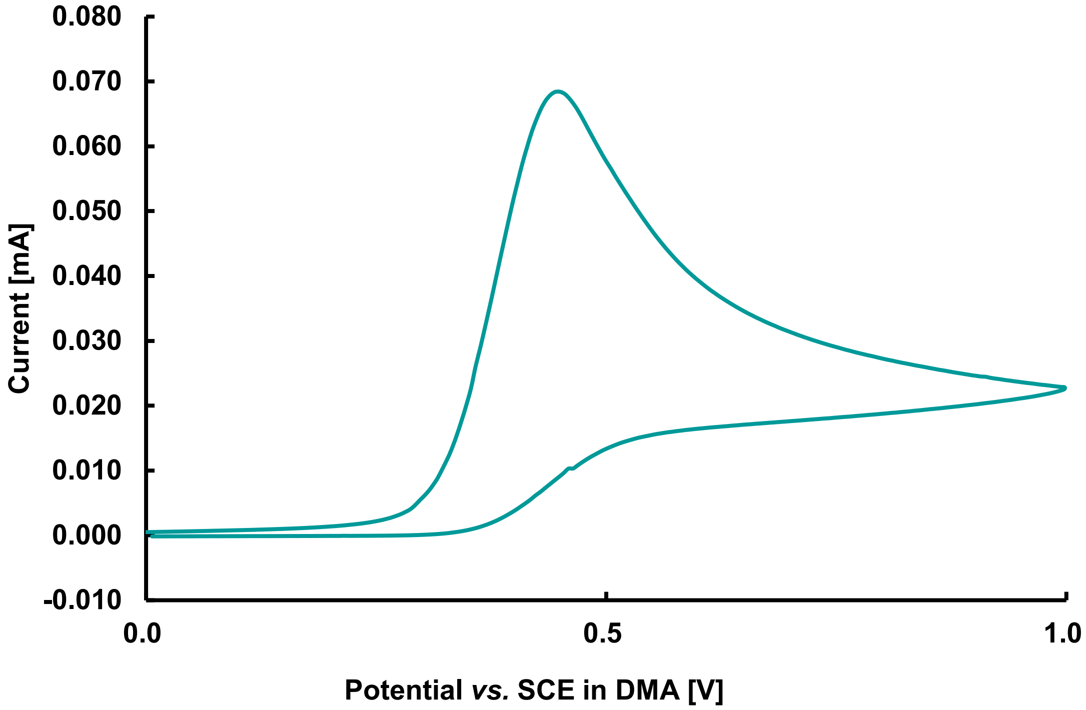

Figure S257. Cyclic voltammetry of **TolSO$_2$Na** in degassed DMA (10 mM) using 0.1 M ^{n}Bu$_4$NPF$_6$ as electrolyte. The scan rate is 50 mV/sec.

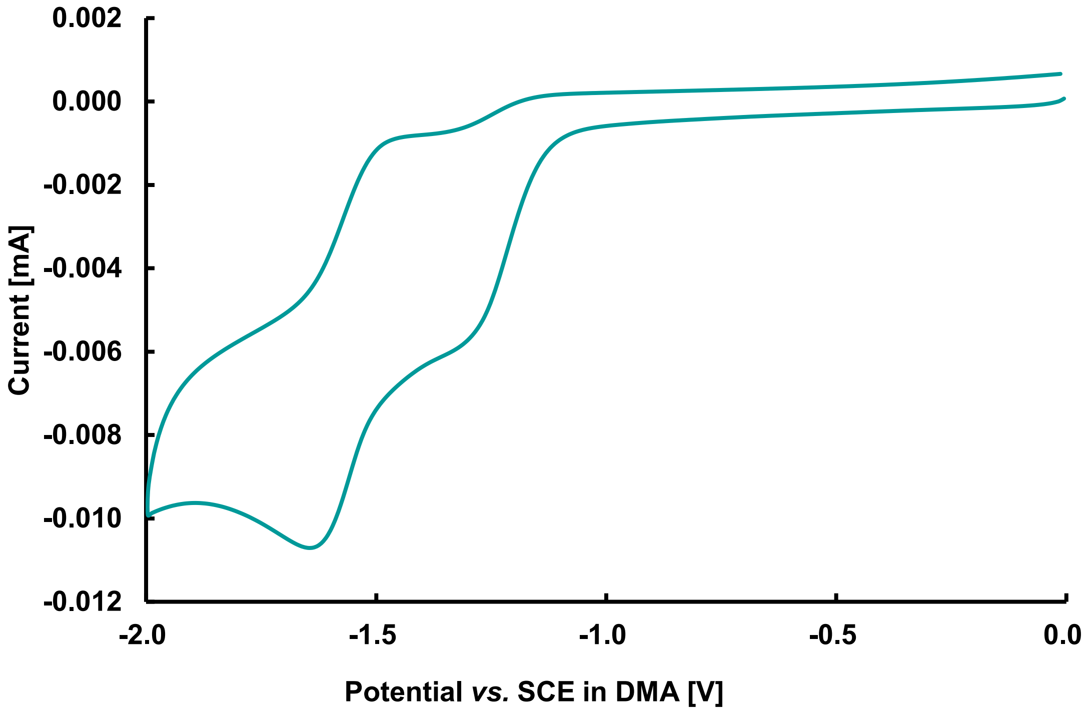

Figure S258. Cyclic voltammetry of **1,3-dioxoisoindolin-2-yl 2-(2-fluoro-[1,1'-biphenyl]-4-yl)propanoate** in degassed DMA (1 mM) using 0.1 M ^{n}Bu$_4$NPF$_6$ as electrolyte. The scan rate is 100 mV/sec.

6. Low $E_{0\text{-}0}$ photocatalysts performance comparison

PC11, **PC13**, **PC21–PC25**, **PC36**, and **PC37** were benchmarked in a simple C–N cross-coupling to give **147**. The absorption profiles (**Fig. S259-S260**) confirm that **PC13** has the most red-shifted spectrum, while the other PCs still retain measurable absorption in the 595–620 nm range and show tails toward longer wavelengths. We therefore evaluated activity under several LEDs (595–660 nm). The reaction were performed following **General Procedure A.**

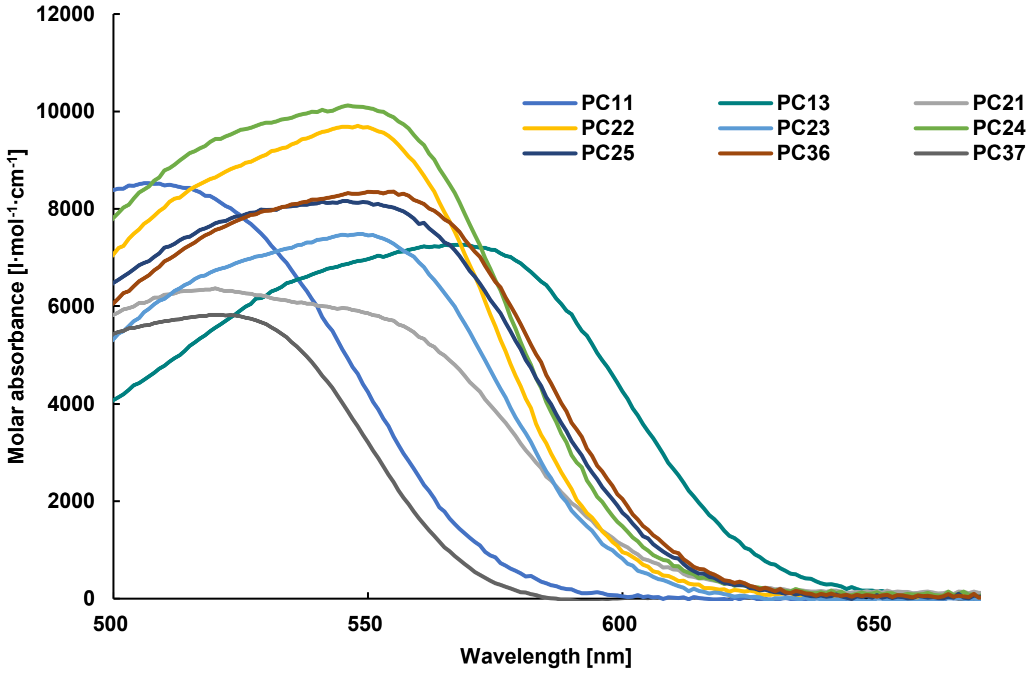

Figure S259. Absorption profile of PCs with low $E_{0\text{-}0}$ energy.

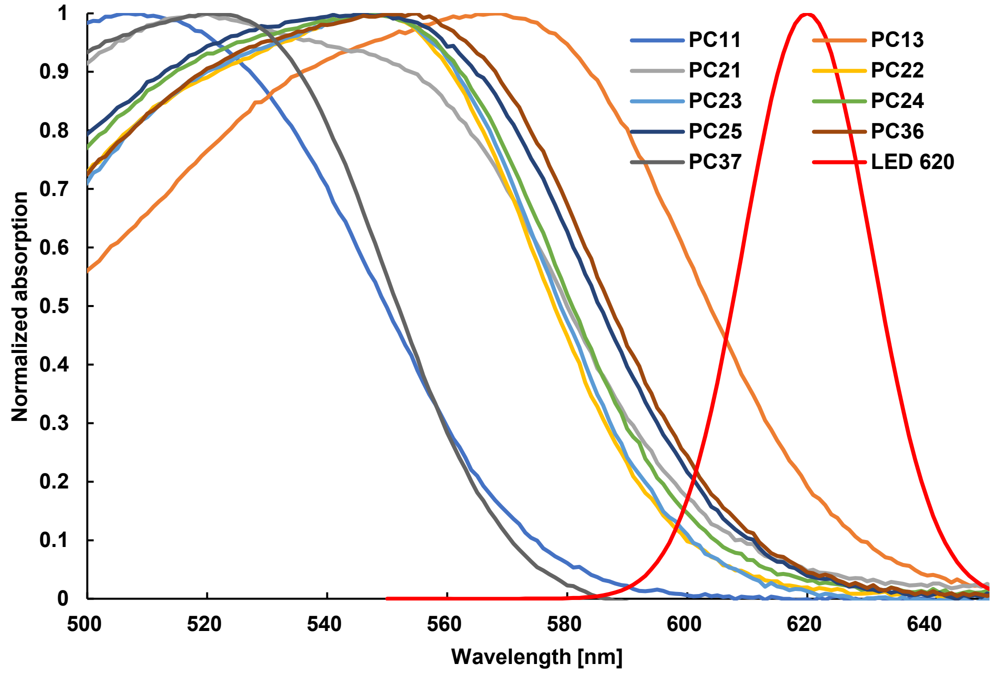

Figure S260. Normalized absorption profile of PCs with low $E_{0\text{-}0}$ energy. 620 nm LED emission is simulated as Gaussian curve with FWHM = 25 nm.

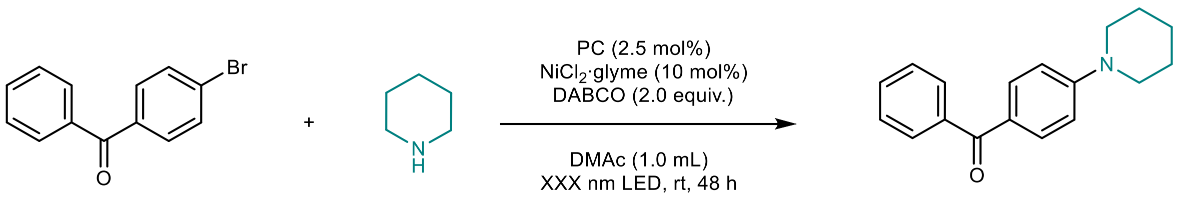

1e **2a** **147**

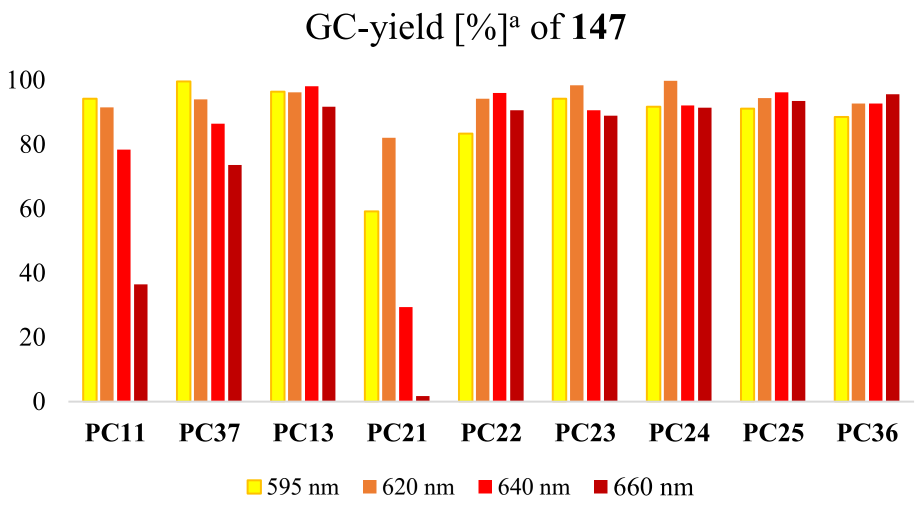

Figure S261. GC-yield of **147** under different wavelength. [a]Yields were determined by GC-FID using dodecane as the internal standard. Reactions were performed with 0.2 mmol of **1c**, 0.6 mmol of piperidine, 2.5 mol% photocatalyst, 10 mol% $NiCl_2$·glyme, 2.0 equiv. DABCO, 1 mL DMA, 595/620/640/660 nm LEDs, rt, 48 h under Ar atmosphere.

Although **PC13** consistently delivered high yield, most catalysts were active from 595 to 660 nm (**Fig. S261**). Apparent activity at 660 nm, despite weak absorbance in that region, is explained by the finite emission bandwidth of the LEDs, which provides usable photon flux within each PC's absorption tail.

Trends across the set align with the expected determinants. **PC11 and PC37** (more blue-shifted) showed declining yields as wavelength increased, consistent with reduced spectral overlap. **PC22–PC25 and PC36** gave similar GC yields in the 595-660 nm window, where their absorption is comparable. **PC21** was an outlier with low activity, which we attribute to poor solubility in DMA and a less reducing ground-state potential, $\boldsymbol{E}$ (Pc/Pc$^{\bullet-}$) = -1.08 V, which is less favorable for the Ni(II/I) reduction step than the other catalysts that are ≤−1.2 V (Table S26).

Table S26. Redox potential of the tested PCs.

Name	$\boldsymbol{E}$ (Pc$^{\bullet+}$/Pc) (V. vs. SCE)	$\boldsymbol{E}$ (Pc/Pc$^{\bullet-}$) (V. vs. SCE)	$\boldsymbol{E}$ (Pc$^{\bullet+}$/Pc*) (V. vs. SCE)	$\boldsymbol{E}$ (Pc*/Pc$^{\bullet-}$) (V. vs. SCE)	$\boldsymbol{E}_{0\text{-}0}$, eV
**PC11**	0.43	-1.83	-1.79	0.39	2.22
**PC13**	0.61	-1.62	-1.43	0.42	2.04
**PC21**	1.03	-1.08	-1.1	1.05	2.13
**PC22**	0.85	-1.41	-1.28	0.72	2.13
**PC23**	0.8	-1.67	-1.33	0.46	2.13
**PC24**	0.91	-1.36	-1.21	0.76	2.12

**PC25**	0.81	-1.36	-1.3	0.75	2.11
**PC36**	0.81	-1.43	-1.29	0.67	2.1
**PC37**	1	-1.46	-1.18	0.72	2.18

Despite these differences, nearly all PCs had excited-state oxidation potentials ***E*** ($Pc^{*}/Pc^{\bullet-}$) > 0.4 V. vs. SCE supporting oxidation of DABCO/piperidine. Taken together, these results show that catalytic performance is governed by four linked factors: (1) spectral overlap, selected by $\boldsymbol{E}_{0\text{-}0}$ (2) ground-/excited-state redox matching for the dual catalytic cycle, (3) solubility in the reaction medium, and (4) redox neutrality of the reaction.

The selected photocatalysts were additionally benchmarked in copper-catalyzed cyanation to give **158**, a redox non-neutral reaction (**Fig**. **S262**) that provides additional insight. Reactions were evaluated under 595 and 620 nm LEDs. Only **PC11**, **PC13**, **PC23**, and **PC37** gave acceptable yields. **PC21** was inactive, while **PC22**, **PC24**, **PC25**, and **PC36** gave low yields. Unexpectedly, most catalysts performed worse at 595 nm than at 620 nm. This effect is likely due to poor solubility in DMA; undissolved particles may scatter or block (self-shading by suspended particles which reduces photon penetration depth) 595 nm light more effectively than 620 nm.

For the cyanation, 1f must be reduced by the excited photocatalyst, and the oxidized $\mathbf{PC}^{\bullet+}$ should subsequently oxidize Cu(I) to Cu(II). Thus, ***E*** ($Pc^{\bullet+}/Pc^{*}$) should be ⩽ ***E*** (**1f/1f**$^{\bullet-}$) =-1.21 V *vs*. SCE (Fig SXXX). This requirement explains the superior performance of **PC11**, **PC13**, and **PC23**, which combine sufficiently strong ***E***($Pc^{\bullet+}/Pc^{*}$) values with good solubility in DMA.

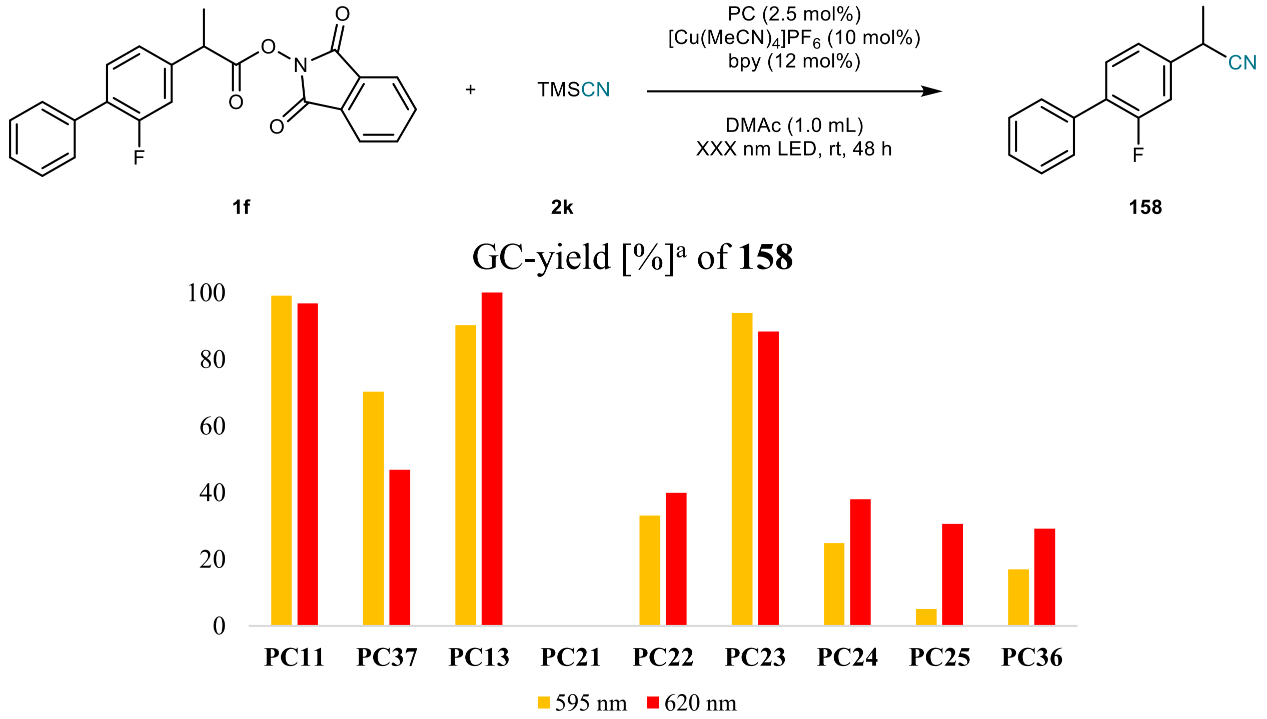

Figure S262. GC-yield of **158** under different wavelength. [a]Yields were determined by GC-FID using dodecane as the internal standard. Reactions were performed with 0.1 mmol of **1c**, 0.2 mmol of TMSCN, 2.5 mol% photocatalyst, 10 mol% $[Cu(MeCN)_4]PF_6$, 12 mol% bpy, 1 mL DMA, 595 or 620 nm LEDs, rt, 48 h under Ar atmosphere.

Therefore, most of the tested photocatalysts with low $\boldsymbol{E_{0\text{-}0}}$ energy have the potential in red/orange light mediated reactions. Moreover, across both benchmarks, **PC13** is optimal, combining the most red-shifted absorption, suitable redox potentials, and good solubility, alongside straightforward synthesis from commercial materials.

General Procedure (**cyanation)**

A dry 4-mL vial equipped with a Teflon-coated magnetic stir bar was charged with 1,3-dioxoisoindolin-2-yl 2-(2-fluoro-[1,1'-biphenyl]-4-yl)propanoate (38.9 mg, 0.1 mmol, 1 equiv.), 2,2'-bipyridyl (1.87 mg, 0.012 mmol, 12 mol %) and photocatalyst (0.0025 mmol, 0.025 equiv.). The vial was introduced to a glovebox filled with Argon atmosphere. Then $[Cu(MeCN)_4]PF_6$ (3.73 mg, 0.01 mmol, 10 mol %), anhydrous and degassed DMA (1.0 mL), and trimethylsilyl cyanide (0.2 mmol, 2 equiv., 26.7 μl) were added. The vial was capped with Teflon septum and removed from the glovebox. Then, the reaction mixture was stirred at room temperature for 48 h under irradiation with 595 or 620 nm LED lamps (AL3 parallel photoreactor, set up 15W) with fan cooling. After that, the reaction mixture was passed through a pad of silica gel and washed with ethyl acetate. Then, internal standard (dodecane, 0.1 mmol) was added. The solution was analyzed by GC-FID and/or GC-MS.

7. Spectroscopic data of the products

(4-(cyclohexylamino)phenyl)(phenyl)methanone (1)

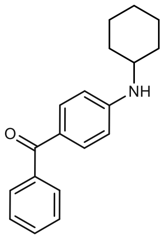

The compound was prepared according to **General Procedure A** with 4-bromobenzophenone (52.2 mg, 0.2 mmol, 1.0 equiv.) and cyclohexylamine (68.6 μL, 0.6 mmol, 3.0 equiv.). The product was purified as a pale yellow solid by flash column chromatography on silica gel using hexane/EtOAc as eluent. Yield: 74% (41.5 mg).

^{1}H NMR (400 MHz, CDCl$_3$) δ 7.77 – 7.66 (m, 4H), 7.55 – 7.48 (m, 1H), 7.48 – 7.40 (m, 2H), 6.61 – 6.52 (m, 2H), 4.26 (br s, 1H), 3.36 (tt, *J* = 10.2, 3.8 Hz, 1H), 2.07 (dt, *J* = 12.9, 3.7 Hz, 2H), 1.78 (dt, *J* = 13.3, 3.9 Hz, 2H), 1.67 (dt, *J* = 12.6, 3.9 Hz, 1H), 1.45 – 1.31 (m, 2H), 1.31 – 1.12 (m, 3H).

^{13}C NMR (101 MHz, CDCl$_3$) δ 195.2, 151.2, 139.4, 133.2, 131.2, 129.6, 128.1, 125.8, 111.8, 51.6, 33.2, 25.8, 25.0.

HRMS (ESI): calculated for $[M+Na]^+$ $C_{19}H_{21}NONa^+$ 302.1515; found 302.1516.

(4-(hexylamino)phenyl)(phenyl)methanone (2)

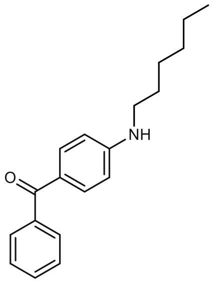

The compound was prepared according to **General Procedure A** with 4-bromobenzophenone (52.2 mg, 0.2 mmol, 1.0 equiv.) and n-hexylamine (79.3 μL, 0.6 mmol, 3.0 equiv.). The product was purified as a pale yellow solid by flash column chromatography on silica gel using hexane/EtOAc as eluent. Yield: 90% (50.8 mg).

^{1}H NMR (400 MHz, CDCl$_3$) δ 7.78 – 7.67 (m, 4H), 7.56 – 7.49 (m, 1H), 7.45 (dd, *J* = 8.2, 6.6 Hz, 2H), 6.59 (d, *J* = 8.6 Hz, 2H), 4.50 (br s, 1H), 3.18 (t, *J* = 7.2 Hz, 2H), 1.70 – 1.55 (m, 2H), 1.46 – 1.25 (m, 6H), 0.96 – 0.84 (m, 3H).

^{13}C NMR (101 MHz, CDCl$_3$) δ 195.3, 152.2, 139.3, 133.2, 131.3, 129.6, 128.1, 126.1, 111.5, 43.6, 31.7, 29.3, 26.9, 22.7, 14.2. The spectral data are consistent with those reported in the literature.[25]

(4-(allylamino)phenyl)(phenyl)methanone (3)

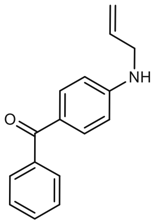

The compound was prepared according to **General Procedure A** with 4-bromobenzophenone (52.2 mg, 0.2 mmol, 1.0 equiv.) and allylamine (44.9 μL, 0.6 mmol, 3.0 equiv.). The product was purified as a pale yellow solid by flash column chromatography on silica gel using hexane/EtOAc as eluent. Yield: 86% (41.0 mg).

^{1}H NMR (400 MHz, CDCl$_3$) δ 7.78 – 7.69 (m, 4H), 7.56 – 7.49 (m, 1H), 7.45 (dd, *J* = 8.2, 6.6 Hz, 2H), 6.64 – 6.58 (m, 2H), 5.93 (ddt, *J* = 17.2, 10.4, 5.3 Hz, 1H), 5.29 (dq, *J* = 17.2, 1.7 Hz, 1H), 5.21 (dq, *J* = 10.3, 1.5 Hz, 1H), 4.54 (br s, 1H), 3.86 (dt, *J* = 5.3, 1.7 Hz, 2H).

^{13}C NMR (101 MHz, CDCl$_3$) δ 195.3, 151.9, 139.2, 134.2, 133.1, 131.4, 129.6, 128.2, 126.5, 117.1, 111.7, 46.0.

HRMS (ESI): calculated for $[M+Na]^+$ $C_{16}H_{15}NONa^+$ 260.1046; found 260.1051.

(4-((4-methoxybenzyl)amino)phenyl)(phenyl)methanone (4)

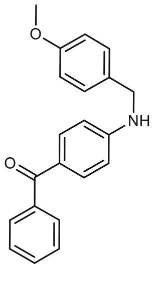

The compound was prepared according to **General Procedure A** with 4-bromobenzophenone (52.2 mg, 0.2 mmol, 1.0 equiv.) and p-anisylamine (77.9 µL, 0.6 mmol, 3.0 equiv.). The product was purified as a pale yellow solid by flash column chromatography on silica gel using hexane/EtOAc as eluentYield: 80% (50.6 mg).

^{1}H NMR (500 MHz, CDCl$_3$) δ 7.77 – 7.69 (m, 4H), 7.55 – 7.49 (m, 1H), 7.44 (t, *J* = 7.3 Hz, 2H), 7.28 (d, *J* = 8.3 Hz, 2H), 6.89 (d, *J* = 8.4 Hz, 2H), 6.64 (d, *J* = 8.4 Hz, 2H), 4.34 (s, 2H), 3.81 (s, 3H).

^{13}C NMR (126 MHz, CDCl$_3$) δ 195.3, 159.3, 151.8, 139.2, 133.1, 131.4, 130.1, 129.6, 129.0, 128.2, 126.7, 114.3, 111.9, 55.5, 47.4.

HRMS (ESI): calculated for $[M+Na]^+$ $C_{21}H_{19}NO_2Na^+$ 340.1308; found 340.1309.

(4-(cyclopropylamino)phenyl)(phenyl)methanone (5)

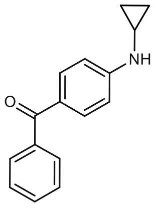

The compound was prepared according to **General Procedure A** with 4-bromobenzophenone (52.2 mg, 0.2 mmol, 1.0 equiv.) and cyclopropylamine (41.6 µL, 0.6 mmol, 3.0 equiv.). The product was purified as a pale yellow solid by flash column chromatography on silica gel using hexane/EtOAc as eluent. Yield: 51% (24.3 mg).

^{1}H NMR (400 MHz, CDCl$_3$) δ 7.79 – 7.69 (m, 4H), 7.57 – 7.50 (m, 1H), 7.50 – 7.42 (m, 2H), 6.86 – 6.79 (m, 2H), 2.53 (tt, *J* = 6.8, 3.6 Hz, 1H), 0.81 (td, *J* = 6.8, 4.8 Hz, 2H), 0.65 – 0.55 (m, 2H).

^{13}C NMR (101 MHz, CDCl$_3$) δ 195.5, 152.1, 139.1, 132.9, 131.5, 129.7, 128.2, 112.6, 25.4, 7.6.

HRMS (ESI): calculated for $[M+Na]^+$ $C_{16}H_{15}NONa^+$ 260.1046; found 260.1055.

(4-((4-hydroxybutyl)amino)phenyl)(phenyl)methanone (6)

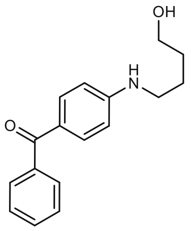

The compound was prepared according to **General Procedure A** with 4-bromobenzophenone (55.3 µL, 0.2 mmol, 1.0 equiv.) and 4-aminobutan-1-ol (55.3 µL, 0.6 mmol, 3.0 equiv.). The product was purified as a pale yellow solid by flash column chromatography on silica gel using DCM/MeOH as eluent. Yield: 40% (21.3 mg).

^{1}H NMR (500 MHz, CDCl$_3$) δ 7.75 – 7.68 (m, 4H), 7.55 – 7.48 (m, 1H), 7.48 – 7.40 (m, 2H), 6.64 – 6.58 (m, 2H), 3.69 (t, *J* = 6.2 Hz, 2H), 3.22 (t, *J* = 6.8 Hz, 2H), 1.80 – 1.62 (m, 4H).

^{13}C NMR (126 MHz, CDCl$_3$) δ 195.4, 151.8, 139.1, 133.1, 131.4, 129.6, 128.2, 126.4, 111.9, 62.4, 43.7, 30.1, 25.7.

HRMS (ESI): calculated for $[M+Na]^+$ $C_{17}H_{19}NO_2Na^+$ 292.1308; found 292.1308.

(4-(morpholinoamino)phenyl)(phenyl)methanone (7)

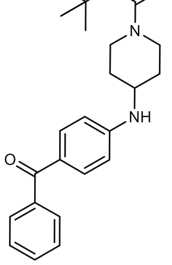

The compound was prepared according to **General Procedure A** with 4-bromobenzophenone (52.2 mg, 0.2 mmol, 1.0 equiv.) and tert-butyl 4-amino-1-piperidinecarboxylate (120 mg, 0.6 mmol, 3.0 equiv.). The product was purified as a pale yellow solid by flash column chromatography on silica gel using hexane/EtOAc as eluent. Yield: 77% (58.8 mg).

^{1}H NMR (500 MHz, CDCl$_3$) δ 7.76 – 7.69 (m, 4H), 7.55 – 7.49 (m, 1H), 7.46 – 7.41 (m, 2H), 6.64 (d, *J* = 8.3 Hz, 2H), 4.19 – 3.94 (m, 2H), 3.52 (td, *J* = 10.2, 5.0 Hz, 1H), 2.92 (t, *J* = 12.5 Hz, 2H), 2.05 (dt, *J* = 14.0, 3.5 Hz, 2H), 1.46 (s, 10H), 1.40 (dq, *J* = 11.7, 8.3, 6.3 Hz, 2H).

^{13}C NMR (126 MHz, CDCl$_3$) δ 195.2, 154.8, 139.0, 133.1, 131.5, 129.6, 128.2, 112.4, 79.9, 50.3, 42.6, 32.0, 28.5.

HRMS (ESI): calculated for $[M+H]^+$ $C_{23}H_{29}N_2O_3^+$ 381.2173; found 381.2155.

(4-(morpholinoamino)phenyl)(phenyl)methanone (8)

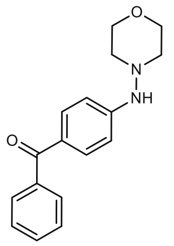

The compound was prepared according to **General Procedure A** with 4-bromobenzophenone (52.2 mg, 0.2 mmol, 1.0 equiv.) and morpholin-4-amine (57.9 µL, 0.6 mmol, 3.0 equiv.). The product was purified as a pale yellow solid by flash column chromatography on silica gel using hexane/EtOAc as eluent. Yield: 61% (34.3 mg).

^{1}H NMR (500 MHz, CDCl$_3$) δ 7.78 – 7.70 (m, 4H), 7.57 – 7.51 (m, 1H), 7.45 (t, J = 7.6 Hz, 2H), 6.96 (d, J = 8.7 Hz, 2H), 5.38 (s, 1H), 3.85 (t, J = 4.6 Hz, 4H), 2.91 – 2.77 (m, 4H).

^{13}C NMR (126 MHz, CDCl$_3$) δ 195.5, 150.7, 138.9, 132.9, 131.7, 129.7, 128.8, 128.2, 112.4, 66.8, 56.3.

HRMS (ESI): calculated for $[M+Na]^+$ $C_{17}H_{18}N_2O_2Na^+$ 305.1260; found 305.1251.

1-([1,1'-biphenyl]-4-yl)piperidine (9)

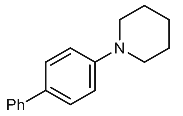

The compound was prepared according to **General Procedure A** with 4-bromobiphenyl (46.6 mg, 0.2 mmol, 1.0 equiv.) and piperidine (55 µL, 0.6 mmol, 3.0 equiv.). The product was purified as a white solid by flash column chromatography on silica gel using hexane/EtOAc as eluent. Yield: 87% (41.5 mg).

^{1}H NMR (400 MHz, CDCl$_3$) δ 7.64 – 7.57 (m, 2H), 7.57 – 7.52 (m, 2H), 7.48 – 7.38 (m, 2H), 7.35 – 7.28 (m, 1H), 7.11 – 7.00 (m, 2H), 3.28 – 3.21 (m, 4H), 1.82 – 1.72 (m, 4H), 1.68 – 1.59 (m, 2H).

^{13}C NMR (101 MHz, CDCl$_3$) δ 151.4, 141.1, 131.9, 128.8, 127.8, 126.6, 126.4, 116.6, 50.6, 25.9, 24.4. The spectral data are consistent with those reported in the literature.[26]

1-phenylpiperidine (10)

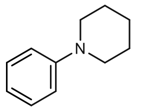

The compound was prepared according to **General Procedure A** with 4-bromobenzene (21.1 µL, 0.2 mmol, 1.0 equiv.) and piperidine (55 µL, 0.6 mmol, 3.0 equiv.). The product was purified as a colorless oil by flash column chromatography on silica gel using hexane/EtOAc as eluent. Yield: 59% (19.1 mg).

^{1}H NMR (400 MHz, CDCl$_3$) δ 7.31 – 7.22 (m, 2H), 6.97 (d, J = 8.1 Hz, 2H), 6.88 – 6.81 (m, 1H), 3.21 – 3.12 (m, 4H), 1.79 – 1.69 (m, 4H), 1.63 – 1.55 (m, 2H).

^{13}C NMR (101 MHz, CDCl$_3$) δ 152.3, 119.5, 116.7, 50.9, 26.0, 24.4. The spectral data are consistent with those reported in the literature.[27]

1-(4-(*tert*-butyl)phenyl)piperidine (11)

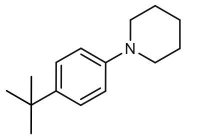

The compound was prepared according to **General Procedure A** with 1-bromo-4-*tert*-butylbenzene (34.1 µL, 0.2 mmol, 1.0 equiv.) and piperidine (55 µL, 0.6 mmol, 3.0 equiv.). The product was purified as a colorless oil by flash column chromatography on silica gel using hexane/EtOAc as eluent. Yield: 62% (26.8 mg).

^{1}H NMR (400 MHz, CDCl$_3$) δ 7.32 – 7.27 (m, 2H), 6.95 – 6.89 (m, 2H), 3.19 – 3.08 (m, 4H), 1.78 – 1.68 (m, 4H), 1.63 – 1.54 (m, 2H), 1.31 (s, 9H).

^{13}C NMR (101 MHz, CDCl$_3$) δ 150.0, 142.1, 125.9, 116.4, 51.1, 34.0, 31.6, 26.1, 24.4. The spectral data are consistent with those reported in the literature.[27]

1-(4-(trimethylsilyl)phenyl)piperidine (12)

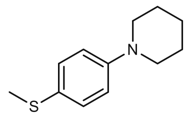

The compound was prepared according to **General Procedure A** with 4-bromothioanisole (40.6 mg, 0.2 mmol, 1.0 equiv.) and piperidine (55 µL, 0.6 mmol, 3.0 equiv.). The product was purified as a colorless oil by flash column chromatography on silica gel using hexane/EtOAc as eluent. Yield: 75% (31.1 mg).

^{1}H NMR (400 MHz, $CDCl_3$) δ 7.31 – 7.23 (m, 2H), 6.94 – 6.88 (m, 2H), 3.20 – 3.11 (m, 4H), 2.46 (s, 3H), 1.77 – 1.69 (m, 4H), 1.64 – 1.56 (m, 2H).

^{13}C NMR (101 MHz, $CDCl_3$) δ 150.7, 130.2, 126.8, 117.1, 50.7, 26.0, 24.3, 18.3. The spectral data are consistent with those reported in the literature.[28]

1-(4-(trimethylsilyl)phenyl)piperidine (13)

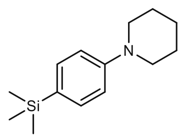

The compound was prepared according to **General Procedure A** with (4-bromophenyl)trimethylsilane (39.1 µL, 0.2 mmol, 1.0 equiv.) and piperidine (55 µL, 0.6 mmol, 3.0 equiv.). The product as a colorless oil was purified by flash column chromatography on silica gel using hexane/EtOAc as eluent. Yield: 83% (38.8 mg).

^{1}H NMR (400 MHz, $CDCl_3$) δ 7.45 – 7.40 (m, 2H), 6.99 – 6.93 (m, 2H), 3.26 – 3.17 (m, 4H), 1.78 – 1.67 (m, 4H), 1.64 – 1.56 (m, 2H), 0.26 (s, 9H).

^{13}C NMR (101 MHz, $CDCl_3$) δ 152.5, 134.4, 128.9, 115.6, 50.2, 25.9, 24.5, -0.8. The spectral data are consistent with those reported in the literature.[29]

1-(4-chlorophenyl)piperidine (14)

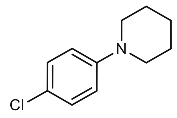

The compound was prepared according to **General Procedure A** with 4-bromochlorobenzene (38.3 mg, 0.2 mmol, 1.0 equiv.) and piperidine (55 µL, 0.6 mmol, 3.0 equiv.). The product was purified as a white solid by flash column chromatography on silica gel using hexane/EtOAc as eluent. Yield: 84% (32.9 mg).

^{1}H NMR (400 MHz, $CDCl_3$) δ 7.22 – 7.14 (m, 2H), 6.92 – 6.81 (m, 2H), 3.16 – 3.07 (m, 4H), 1.77 – 1.66 (m, 4H), 1.61 – 1.53 (m, 2H).

^{13}C NMR (101 MHz, $CDCl_3$) δ 150.7, 129.0, 124.3, 117.9, 50.9, 25.8, 24.2. The spectral data are consistent with those reported in the literature.[27]

methyl 4-(piperidin-1-yl)benzoate (15)

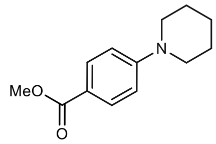

The compound was prepared according to **General Procedure A** with methyl 4-bromobenzoate (43 mg, 0.2 mmol, 1.0 equiv.) and piperidine (55 µL, 0.6 mmol, 3.0 equiv.). The product was purified as a white solid by flash column chromatography on silica gel using hexane/EtOAc as eluent. Yield: 92% (40.2 mg).

^{1}H NMR (400 MHz, $CDCl_3$) δ 7.92 – 7.86 (m, 2H), 6.85 (d, J = 8.6 Hz, 2H), 3.85 (s, 3H), 3.32 (t, J = 5.1 Hz, 4H), 1.71 – 1.60 (m, 6H).

^{13}C NMR (101 MHz, $CDCl_3$) δ 167.4, 154.6, 131.4, 118.8, 113.7, 51.7, 48.9, 25.5, 24.5. The spectral data are consistent with those reported in the literature.[26]

1-(4-(piperidin-1-yl)phenyl)ethan-1-one (16)

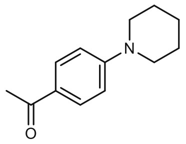

The compound was prepared according to **General Procedure A** with 4-bromoacetophenone (39.8 mg, 0.2 mmol, 1.0 equiv.) and piperidine (55 μL, 0.6 mmol, 3.0 equiv.). The product was purified as a white solid by flash column chromatography on silica gel using hexane/EtOAc as eluent. Yield: 92% (37.5 mg).

^{1}H NMR (400 MHz, CDCl$_3$) δ 7.88 – 7.82 (m, 2H), 6.93 – 6.78 (m, 2H), 3.35 (t, *J* = 5.1 Hz, 4H), 2.51 (s, 3H), 1.73 – 1.59 (m, 6H).

^{13}C NMR (101 MHz, CDCl$_3$) δ 196.5, 154.4, 130.6, 126.8, 113.4, 48.8, 26.2, 25.4, 24.4. The spectral data are consistent with those reported in the literature.[30]

4-(piperidin-1-yl)benzaldehyde (17)

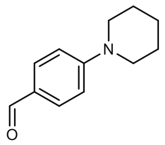

The compound was prepared according to **General Procedure A** with 4-bromobenzaldehyde (37 mg, 0.2 mmol, 1.0 equiv.) and piperidine (55 μL, 0.6 mmol, 3.0 equiv.). The product was purified as a white solid by flash column chromatography on silica gel using hexane/EtOAc as eluent. Yield: 67% (25.4 mg).

^{1}H NMR (400 MHz, CDCl$_3$) δ 9.75 (s, 1H), 7.78 – 7.68 (m, 2H), 7.02 – 6.82 (m, 2H), 3.41 (t, *J* = 5.3 Hz, 4H), 1.74 – 1.62 (m, 6H).

^{13}C NMR (101 MHz, CDCl$_3$) δ 190.4, 155.1, 132.1, 126.5, 113.6, 48.8, 25.4, 24.4. The spectral data are consistent with those reported in the literature.[31]

1-(4-(trifluoromethyl)phenyl)piperidine (18)

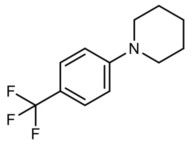

The compound was prepared according to **General Procedure A** with 4-bromobenzotrifluoride (28 μL, 0.2 mmol, 1.0 equiv.) and piperidine (55 μL, 0.6 mmol, 3.0 equiv.). The product was purified as a colorless oil by flash column chromatography on silica gel using hexane/EtOAc as eluent. Yield: 88% (40.4 mg).

^{1}H NMR (400 MHz, CDCl$_3$) δ 7.50 – 7.42 (m, 1H), 6.96 – 6.89 (m, 1H), 3.31 – 3.24 (m, 2H), 1.76 – 1.57 (m, 4H).

^{13}C NMR (101 MHz, CDCl$_3$) δ 153.9, 126.5 (q, *J* = 3.8 Hz), 125.0 (q, *J* = 270.4 Hz), 119.7 (d, *J* = 32.7 Hz), 114.7, 49.5, 25.5, 24.4.

^{19}F NMR (377 MHz, CDCl$_3$) δ -61.18. The spectral data are consistent with those reported in the literature.[31]

4-(piperidin-1-yl)benzenesulfonamide (19)

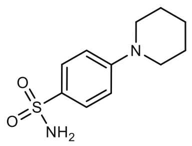

The compound was prepared according to **General Procedure A** with 4-bromobenzenesulfonamide (47.2 mg, 0.2 mmol, 1.0 equiv.) and piperidine (55 μL, 0.6 mmol, 3.0 equiv.). The product was purified as a white solid by flash column chromatography on silica gel using dichloromethane as eluent. Yield: 73% (35.1 mg).

^{1}H NMR (400 MHz, CDCl$_3$) δ 7.78 – 7.69 (m, 2H), 6.97 – 6.84 (m, 2H), 4.89 (s, 2H), 3.39 – 3.26 (m, 4H), 1.78 – 1.57 (m, 6H).

^{13}C NMR (101 MHz, CDCl$_3$) δ 154.0, 128.4, 114.2, 49.1, 25.3, 24.3..

HRMS (ESI): calculated for $[M+Na]^+$ $C_{11}H_{16}N_2O_2SNa^+$ 263.0825; found 263.0830.

1-(4-(methylsulfonyl)phenyl)piperidine (20)

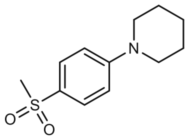

The compound was prepared according to **General Procedure A** with 1-bromo-4-(methylsulfonyl)benzene (47 mg, 0.2 mmol, 1.0 equiv.) and piperidine (55 µL, 0.6 mmol, 3.0 equiv.). The product was purified as a white solid by flash column chromatography on silica gel using hexane/EtOAc as eluent. Yield: 92% (43.9 mg).

^{1}H NMR (400 MHz, CDCl$_3$) δ 7.75 – 7.69 (m, 2H), 6.94 – 6.88 (m, 2H), 3.35 (t, J = 4.4 Hz, 4H), 3.00 (s, 3H), 1.72 – 1.60 (m, 6H).

^{13}C NMR (101 MHz, CDCl$_3$) δ 154.5, 129.2, 127.6, 113.9, 48.8, 45.1, 25.3, 24.3. The spectral data are consistent with those reported in the literature. [31]

1-(4-(4,4,5,5-tetramethyl-1,3,2-dioxaborolan-2-yl)phenyl)piperidine (21)

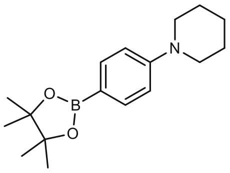

The compound was prepared according to **General Procedure A** with 2-(4-bromomethyl-phenyl)-4,4,5,5-tetramethyl-[1,3,2]dioxaborolane (56.6 mg, 0.2 mmol, 1.0 equiv.) and piperidine (55 µL, 0.6 mmol, 3.0 equiv.). The product was purified as a white solid by flash column chromatography on silica gel using hexane/EtOAc as eluent. Yield: 77% (43.9 mg).

^{1}H NMR (400 MHz, CDCl$_3$) δ 7.72 – 7.65 (m, 2H), 6.90 (d, J = 8.1 Hz, 2H), 3.29 – 3.19 (m, 4H), 1.74 – 1.55 (m, 6H), 1.33 (s, 12H).

^{13}C NMR (101 MHz, CDCl$_3$) δ 154.0, 136.2, 114.7, 83.4, 49.6, 25.6, 25.0, 24.5.

^{11}B NMR (128 MHz, CDCl$_3$) δ 30.88. The spectral data are consistent with those reported in the literature.[32]

5-(piperidin-1-yl)picolinonitrile (22)

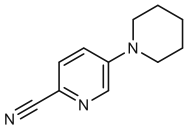

The compound was prepared according to **General Procedure A** with 5-bromopicolinonitrile (36.6 mg, 0.2 mmol, 1.0 equiv.) and piperidine (55 µL, 0.6 mmol, 3.0 equiv.). The product was purified as a colorless oil by flash column chromatography on silica gel using hexane/EtOAc as eluent. Yield: 79% (29.4 mg).

^{1}H NMR (400 MHz, CDCl$_3$) δ 8.25 (d, J = 2.9 Hz, 1H), 7.44 (d, J = 8.8 Hz, 1H), 7.04 (dd, J = 8.8, 3.0 Hz, 1H), 3.35 (t, J = 4.7 Hz, 4H), 1.72 – 1.63 (m, 6H).

^{13}C NMR (101 MHz, CDCl$_3$) δ 148.2, 138.0, 129.1, 120.1, 119.0, 118.7, 47.9, 25.1, 24.0. The spectral data are consistent with those reported in the literature.

HRMS (ESI): calculated for $[M+Na]^+$ $C_{11}H_{13}N_3Na^+$ 210.1002; found 210.0993

4-(piperidin-1-yl)benzonitrile (23,24, 25)

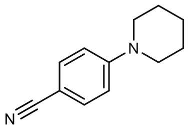

The compound was prepared according to **General Procedure A** with 4-iodobenzonitrile/bromobenzonitrile/chlorobenzonitrile (45.8/36.4/27.5 mg, 0.2 mmol, 1.0 equiv.) and piperidine (55 µL, 0.6 mmol, 3.0 equiv.). The product was purified as a white solid by flash column chromatography on silica gel using hexane/EtOAc as eluent. Yield: 92/91/91% (34.3 mg, 33.9 mg, 33.9 mg).

^{1}H NMR (400 MHz, CDCl$_3$) δ 7.50 – 7.43 (m, 2H), 6.89 – 6.80 (m, 2H), 3.32 (t, J = 4.3 Hz, 4H), 1.70 – 1.60 (m, 6H).

^{13}C NMR (101 MHz, CDCl$_3$) δ 153.6, 133.6, 120.5, 114.3, 99.2, 48.6, 25.3, 24.3.The spectral data are consistent with those reported in the literature.[31]

1-(3,5-dimethoxyphenyl)piperidine (26)

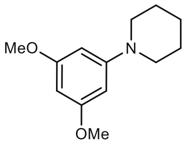

The compound was prepared according to **General Procedure A** with 1-bromo-3,5-dimethoxybenzene (43.4 mg, 0.2 mmol, 1.0 equiv.) and piperidine (55 μL, 0.6 mmol, 3.0 equiv.). The product was purified as a colorless oil by flash column chromatography on silica gel using hexane/EtOAc as eluent. Yield: 71% (34.2 mg).

^{1}H NMR (400 MHz, $CDCl_3$) δ 6.15 (d, J = 2.2 Hz, 2H), 6.01 (t, J = 2.1 Hz, 1H), 3.77 (s, 6H), 3.19 – 3.12 (m, 4H), 1.76 – 1.65 (m, 4H), 1.62 – 1.53 (m, 2H).

^{13}C NMR (101 MHz, $CDCl_3$) δ 161.5, 153.7, 95.6, 91.7, 55.3, 51.0, 25.7, 24.4. The spectral data are consistent with those reported in the literature.[33]

1-(naphthalen-2-yl)piperidine (27)

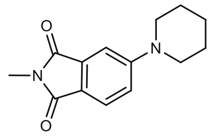

The compound was prepared according to **General Procedure A** with 4-bromo-N-methylphthalimide (48.0 mg, 0.2 mmol, 1.0 equiv.) and piperidine (55 μL, 0.6 mmol, 3.0 equiv.). The product was purified as a yellow solid by flash column chromatography on silica gel using hexane/EtOAc as eluent. Yield: 91% (44.3 mg).

^{1}H NMR (400 MHz, $CDCl_3$) δ 7.61 (d, J = 8.4 Hz, 1H), 7.24 (d, J = 2.4 Hz, 1H), 6.98 (dd, J = 8.5, 2.4 Hz, 1H), 3.43 – 3.36 (m, 4H), 3.11 (s, 3H), 1.73 – 1.61 (m, 6H).

^{13}C NMR (101 MHz, $CDCl_3$) δ 169.4, 168.8, 155.4, 134.9, 124.8, 119.4, 117.4, 108.5, 49.1, 25.3, 24.3, 23.9. The spectral data are consistent with those reported in the literature.[34]

5-(piperidin-1-yl)-2,3-dihydro-1*H*-inden-1-one (28)

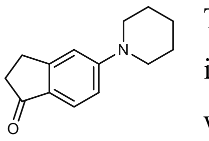

The compound was prepared according to **General Procedure A** with 5-bromo-2,3-dihydro-1*H*-inden-1-one (42.2 mg, 0.2 mmol, 1.0 equiv.) and piperidine (55 μL, 0.6 mmol, 3.0 equiv.). The product was purified as a pale yellow solid by flash column chromatography on silica gel using hexane/EtOAc as eluent. Yield: 65% (27.9 mg).

^{1}H NMR (400 MHz, $CDCl_3$) δ 7.60 (d, J = 8.7 Hz, 1H), 6.92 – 6.71 (m, 2H), 3.39 (t, J = 4.9 Hz, 4H), 3.06 – 2.93 (m, 2H), 2.70 – 2.55 (m, 2H), 1.75 – 1.58 (m, 6H).

^{13}C NMR (101 MHz, $CDCl_3$) δ 205.0, 158.2, 156.1, 127.1, 125.3, 114.4, 109.7, 49.1, 36.6, 26.0, 25.4, 24.5. The spectral data are consistent with those reported in the literature.[35]

4-(piperidin-1-yl)-2,3-dihydro-1*H*-inden-1-one (29)

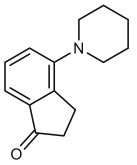

The compound was prepared according to **General Procedure A** with 4-bromo-2,3-dihydro-1*H*-inden-1-one (42.2 mg, 0.2 mmol, 1.0 equiv.) and piperidine (55 μL, 0.6 mmol, 3.0 equiv.). The product was purified as a yellow oil by flash column chromatography on silica gel using hexane/EtOAc as eluent. Yield: 77% (33.3 mg).

^{1}H NMR (400 MHz, $CDCl_3$) δ 7.38 (d, J = 7.5 Hz, 1H), 7.30 (t, J = 7.6 Hz, 1H), 7.12 (d, J = 7.8 Hz, 1H), 3.18 – 3.03 (m, 2H), 2.99 (t, J = 5.3 Hz, 4H), 2.70 – 2.60 (m, 2H), 1.84 – 1.49 (m, 6H).

^{13}C NMR (101 MHz, $CDCl_3$) δ 207.7, 151.9, 149.0, 138.4, 128.5, 123.1, 116.9, 52.3, 36.4, 26.5, 24.8, 24.3.

HRMS (ESI): calculated for $[M+Na]^+$ $C_{14}H_{17}NONa^+$ 238.1202; found 238.1213.

1-(thiophen-3-yl)piperidine (30)

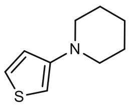

The compound was prepared according to **General Procedure A** with 3-bromothiophene (18.7 µL, 0.2 mmol, 1.0 equiv.) and piperidine (55 µL, 0.6 mmol, 3.0 equiv.). The product was purified as a colorless oil by flash column chromatography on silica gel using hexane/EtOAc as eluent. Yield: 42% (14.1mg).

^{1}H NMR (400 MHz, CDCl$_3$) δ 7.22 (dd, *J* = 5.3, 3.1 Hz, 1H), 6.89 (dd, *J* = 5.3, 1.6 Hz, 1H), 6.19 (dd, *J* = 3.2, 1.6 Hz, 1H), 3.10 – 3.03 (m, 4H), 1.77 – 1.67 (m, 4H), 1.60 – 1.51 (m, 2H).

^{13}C NMR (101 MHz, CDCl$_3$) δ 153.3, 125.2, 120.7, 100.4, 52.0, 25.8, 24.2. The spectral data are consistent with those reported in the literature.[36]

4-(piperidin-1-yl)picolinonitrile (31)

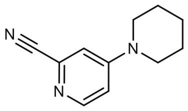

The compound was prepared according to **General Procedure A** with 4-bromo-2-cyanopyridine (36.6 mg, 0.2 mmol, 1.0 equiv.) and piperidine (55 µL, 0.6 mmol, 3.0 equiv.). The product was purified as a white solid by flash column chromatography on silica gel using hexane/EtOAc as eluent. Yield: 70% (26.1 mg).

^{1}H NMR (400 MHz, CDCl$_3$) δ 8.22 (d, *J* = 6.1 Hz, 1H), 7.00 (d, *J* = 2.8 Hz, 1H), 6.73 (dd, *J* = 6.2, 2.8 Hz, 1H), 3.37 (t, *J* = 5.1 Hz, 4H), 1.75 – 1.59 (m, 6H).

^{13}C NMR (101 MHz, CDCl$_3$) δ 154.4, 150.9, 134.3, 118.1, 113.0, 110.0, 47.1, 25.0, 24.1. The spectral data are consistent with those reported in the literature.[37]

1-(naphthalen-2-yl)piperidine (32)

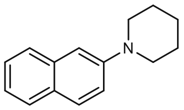

The compound was prepared according to **General Procedure A** with 2-bromonapthalene (41.4 mg, 0.2 mmol, 1.0 equiv.) and piperidine (55 µL, 0.6 mmol, 3.0 equiv.). The product was purified as a white solid by flash column chromatography on silica gel using hexane/EtOAc as eluent. Yield: 84% (35.6 mg).

^{1}H NMR (400 MHz, CDCl$_3$) δ 7.77 – 7.67 (m, 3H), 7.44 – 7.37 (m, 1H), 7.35 – 7.27 (m, 2H), 7.22 – 7.15 (m, 1H), 3.36 – 3.20 (m, 4H), 1.89 – 1.73 (m, 4H), 1.69 – 1.57 (m, 2H).

^{13}C NMR (101 MHz, CDCl$_3$) δ 149.9, 134.8, 128.7, 128.6, 127.5, 126.9, 126.3, 123.4, 120.3, 110.8, 51.4, 25.9, 24.4. The spectral data are consistent with those reported in the literature.[27]

3-(piperidin-1-yl)quinoline (33)

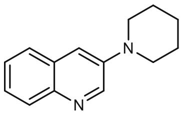

The compound was prepared according to **General Procedure A** with 3-bromoquinoline (27.2 µL, 0.2 mmol, 1.0 equiv.) and piperidine (55 µL, 0.6 mmol, 3.0 equiv.). The product was purified as a white solid by flash column chromatography on silica gel using hexane/EtOAc as eluent. Yield: 95% (40.5 mg).

^{1}H NMR (400 MHz, CDCl$_3$) δ 8.80 (d, *J* = 2.8 Hz, 1H), 7.98 (dd, *J* = 8.0, 1.6 Hz, 1H), 7.65 (dd, *J* = 7.7, 1.9 Hz, 1H), 7.52 – 7.40 (m, 2H), 7.33 (d, *J* = 2.8 Hz, 1H), 3.30 – 3.23 (m, 4H), 1.81 – 1.73 (m, 4H), 1.68 – 1.58 (m, 2H).

^{13}C NMR (101 MHz, CDCl$_3$) δ 145.8, 145.7, 142.5, 129.1, 128.8, 126.9, 126.6, 126.3, 116.9, 50.7, 25.8, 24.2. The spectral data are consistent with those reported in the literature.[38]

phenyl(4-(pyrrolidin-1-yl)phenyl)methanone (34)

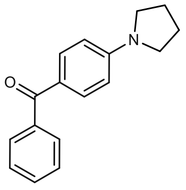

The compound was prepared according to **General Procedure A** with 4-bromobenzophenone (52.2 mg, 0.2 mmol, 1.0 equiv.) and pyrrolidine (49.3 µL, 0.6 mmol, 3.0 equiv.). The product was purified as a pale yellow solid by flash column chromatography on silica gel using hexane/EtOAc as eluent. Yield: 89% (44.6 mg).

^{1}H NMR (400 MHz, $CDCl_3$) δ 7.83 – 7.76 (m, 2H), 7.74 – 7.68 (m, 2H), 7.56 – 7.40 (m, 3H), 6.56 – 6.51 (m, 2H), 3.43 – 3.29 (m, 4H), 2.12 – 1.97 (m, 4H).

^{13}C NMR (101 MHz, $CDCl_3$) δ 195.3, 151.0, 139.6, 133.1, 131.1, 129.5, 128.1, 124.3, 110.7, 47.7, 25.6. The spectral data are consistent with those reported in the literature.[39]

(4-morpholinophenyl)(phenyl)methanone (35)

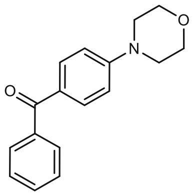

The compound was prepared according to **General Procedure A** with 4-bromobenzophenone (52.2 mg, 0.2 mmol, 1.0 equiv.) and morpholine (52.3 µL, 0.6 mmol, 3.0 equiv.). The product was purified as a pale yellow solid by flash column chromatography on silica gel using hexane/EtOAc as eluent. Yield: 88% (47.2 mg).

^{1}H NMR (400 MHz, $CDCl_3$) δ 7.84 – 7.77 (m, 2H), 7.77 – 7.70 (m, 2H), 7.58 – 7.50 (m, 1H), 7.50 – 7.42 (m, 2H), 6.95 – 6.87 (m, 2H), 3.92 – 3.82 (m, 4H), 3.37 – 3.28 (m, 4H).

^{13}C NMR (101 MHz, $CDCl_3$) δ 195.4, 154.0, 138.8, 132.6, 131.7, 129.7, 128.3, 128.1, 113.4, 66.7, 47.8. The spectral data are consistent with those reported in the literature.[40]

Tert-butyl 4-(4-benzoylphenyl)piperazine-1-carboxylate (36)

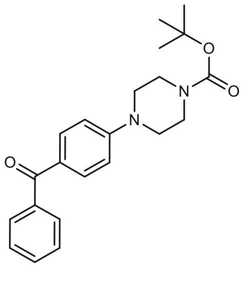

The compound was prepared according to **General Procedure A** with 4-bromobenzophenone (52.2 mg, 0.2 mmol, 1.0 equiv.) and *tert*-Butyl piperazine-1-carboxylate (112 mg, 0.6 mmol, 3.0 equiv.). The product was purified as a pale yellow solid by flash column chromatography on silica gel using hexane/EtOAc as eluent. Yield: 85% (62.1 mg).

^{1}H NMR (400 MHz, $CDCl_3$) δ 7.84 – 7.77 (m, 2H), 7.77 – 7.70 (m, 2H), 7.58 – 7.50 (m, 1H), 7.50 – 7.42 (m, 2H), 6.95 – 6.87 (m, 2H), 3.92 – 3.82 (m, 4H), 3.37 – 3.28 (m, 4H).

^{13}C NMR (101 MHz, $CDCl_3$) δ 195.4, 154.0, 138.8, 132.6, 131.7, 129.7, 128.3, 128.1, 113.4, 66.7, 47.8.

HRMS (ESI): calculated for $[M+Na]^+$ $C_{22}H_{26}N_2O_3Na^+$ 389.1836; found 389.1841.

4-methoxy-N-phenylaniline (37)

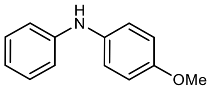

The compound was prepared according to **General Procedure B** with bromobenzene (21.1 µL, 0.2 mmol, 1.0 equiv.) and p-anisidine (73.9 mg, 0.6 mmol, 3.0 equiv.). The product was purified as a white solid by flash column chromatography on silica gel using hexane/EtOAc as eluent. Yield: 97% (38.6 mg).

^{1}H NMR (500 MHz, $CDCl_3$) δ 7.25 – 7.19 (m, 2H), 7.14 – 7.02 (m, 2H), 6.96 – 6.90 (m, 2H), 6.90 – 6.79 (m, 3H), 3.81 (s, 3H).

^{13}C NMR (126 MHz, $CDCl_3$) δ 155.4, 145.3, 135.8, 129.4, 122.4, 119.8, 115.8, 114.8, 55.7. The spectral data are consistent with those reported in the literature.[41]

4-(*tert*-butyl)-*N*-(4-methoxyphenyl)aniline (38)

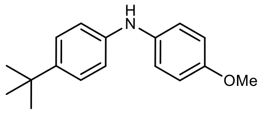

The compound was prepared according to **General Procedure B** with 1-bromo-4-*tert*-butylbenzene (34.1 µL, 0.2 mmol, 1.0 equiv.) and *p*-anisidine (73.9 mg, 0.6 mmol, 3.0 equiv.). The product was purified as a colorless oil by flash column chromatography on silica gel using hexane/EtOAc as eluent. Yield: 64% (35.5 mg).

^{1}H NMR (400 MHz, $CDCl_3$) δ 7.31 – 7.21 (m, 2H), 7.16 – 6.78 (m, 6H), 3.81 (s, 3H), 1.32 (s, 9H).

^{13}C NMR (101 MHz, $CDCl_3$) δ 155.1, 143.1, 142.6, 136.2, 126.2, 121.6, 116.2, 114.8, 55.7, 34.2, 31.6. The spectral data are consistent with those reported in the literature.[42]

***N*-(4-methoxyphenyl)-[1,1'-biphenyl]-4-amine (39)**

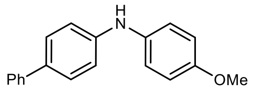

The compound was prepared according to **General Procedure B** with 4-bromobiphenyl (46.6 mg, 0.2 mmol, 1.0 equiv.) and p-anisidine (73.9 mg, 0.6 mmol, 3.0 equiv.). The product was purified as a white solid by flash column chromatography on silica gel using hexane/EtOAc as eluent. Yield: 89% (48.9 mg).

^{1}H NMR (500 MHz, $CDCl_3$) δ 7.58 (d, *J* = 7.6 Hz, 2H), 7.50 (d, *J* = 8.1 Hz, 2H), 7.43 (t, *J* = 7.6 Hz, 2H), 7.31 (t, *J* = 7.4 Hz, 1H), 7.24 – 6.93 (m, 4H), 6.93 – 6.88 (m, 2H), 3.83 (s, 3H).

^{13}C NMR (126 MHz, $CDCl_3$) δ 155.6, 144.7, 141.1, 135.5, 132.5, 128.8, 128.1, 126.5 (2C), 122.6, 115.9, 114.8, 55.7. The spectral data are consistent with those reported in the literature.[43]

methyl 4-((4-methoxyphenyl)amino)benzoate (40)

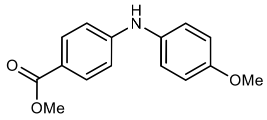

The compound was prepared according to **General Procedure B** with methyl 4-bromobenzoate (43 mg, 0.2 mmol, 1.0 equiv.) and p-anisidine (73.9 mg, 0.6 mmol, 3.0 equiv.). The product was purified as a white solid by flash column chromatography on silica gel using hexane/EtOAc as eluent. Yield: 98% (42.0 mg).

^{1}H NMR (500 MHz, $CDCl_3$) δ 7.87 (d, *J* = 8.4 Hz, 2H), 7.13 (d, *J* = 8.0 Hz, 2H), 6.90 (d, *J* = 8.7 Hz, 2H), 6.81 (d, *J* = 8.3 Hz, 2H), 3.86 (s, 3H), 3.82 (s, 3H).

^{13}C NMR (126 MHz, $CDCl_3$) δ 167.2, 156.7, 149.9, 133.5, 131.7, 124.6, 120.2, 114.9, 113.4, 55.7, 51.8. The spectral data are consistent with those reported in the literature.[44]

(4-((4-methoxyphenyl)amino)phenyl)(phenyl)methanone (41)

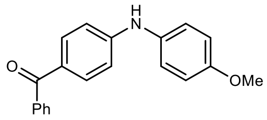

The compound was prepared according to **General Procedure B** with 4-bromobenzophenone (52.2 mg, 0.2 mmol, 1.0 equiv.) and *p*-anisidine (73.9 mg, 0.6 mmol, 3.0 equiv.). The product was purified as a pale yellow solid by flash column chromatography on silica gel using hexane/EtOAc as eluent. Yield: 81% (49.0 mg).

^{1}H NMR (500 MHz, $CDCl_3$) δ 7.79 – 7.69 (m, 4H), 7.54 (t, *J* = 7.4 Hz, 1H), 7.45 (t, *J* = 7.5 Hz, 2H), 7.16 (d, *J* = 8.3 Hz, 2H), 6.91 (d, *J* = 8.8 Hz, 2H), 6.85 (d, *J* = 8.3 Hz, 2H), 3.82 (s, 3H).

^{13}C NMR (126 MHz, $CDCl_3$) δ 195.3, 156.9, 150.0, 139.0, 133.2, 133.0, 131.6, 129.7, 128.2, 128.0, 124.8, 115.0, 113.3, 55.7. The spectral data are consistent with those reported in the literature.[45]

4-methoxy-N-(4-(trifluoromethyl)phenyl)aniline (42)

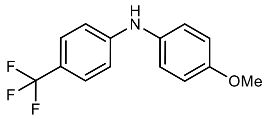

The compound was prepared according to **General Procedure B** with 4-bromobenzotrifluoride (28 µL, 0.2 mmol, 1.0 equiv.) and p-anisidine (73.9 mg, 0.6 mmol, 3.0 equiv.). The product was purified as a colorless oil by flash column chromatography on silica gel using hexane/EtOAc as eluent. Yield: 86% (46.0 mg).

^{1}H NMR (500 MHz, $CDCl_3$) δ 7.42 (d, J = 8.4 Hz, 2H), 7.12 (d, J = 8.4 Hz, 2H), 6.94 – 6.89 (m, 2H), 6.86 (d, J = 8.4 Hz, 2H), 3.82 (s, 3H).

^{13}C NMR (126 MHz, $CDCl_3$) δ 156.6, 148.7, 133.8, 126.8 (q, J = 3.9 Hz), 124.9 (q, J = 270.3 Hz), 124.4, 120.6 (q, J = 32.7 Hz), 115.0, 113.9, 55.7.

^{19}F NMR (471 MHz, $CDCl_3$) δ -61.24. The spectral data are consistent with those reported in the literature.[31]

N-(4-methoxyphenyl)naphthalen-2-amine (43)

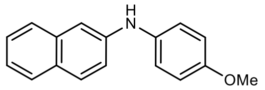

The compound was prepared according to **General Procedure B** with 2-bromonaphthalene (41.4 mg, 0.2 mmol, 1.0 equiv.) and p-anisidine (73.9 mg, 0.6 mmol, 3.0 equiv.). The product was purified as a white solid by flash column chromatography on silica gel using hexane/EtOAc as eluent. Yield: 62% (30.8 mg).

^{1}H NMR (400 MHz, $CDCl_3$) δ 7.74 – 7.69 (m, 2H), 7.61 (d, J = 8.2 Hz, 1H), 7.42 – 7.36 (m, 1H), 7.31 – 7.27 (m, 2H), 7.23 – 7.18 (m, 2H), 7.15 (dd, J = 8.8, 2.3 Hz, 1H), 6.94 – 6.88 (m, 2H), 3.82 (s, 3H).

^{13}C NMR (101 MHz, $CDCl_3$) δ 155.9, 142.4, 135.2, 134.8, 129.4, 129.0, 127.8, 126.6, 126.5, 123.3, 122.9, 119.1, 114.9, 109.8, 55.7. The spectral data are consistent with those reported in the literature.[46]

N-phenyl-4-(trifluoromethyl)aniline (44)

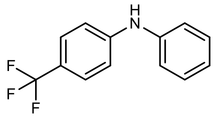

The compound was prepared according to **General Procedure B** with 4-bromobenzotrifluoride (28 µL, 0.2 mmol, 1.0 equiv.) and aniline (55.9 mg, 0.6 mmol, 3.0 equiv.). The product was purified as a white solid by flash column chromatography on silica gel using hexane/EtOAc as eluent. Yield: 93% (44.3 mg).

^{1}H NMR (400 MHz, $CDCl_3$) δ 7.52 – 7.46 (m, 2H), 7.38 – 7.32 (m, 2H), 7.19 – 7.14 (m, 2H), 7.11 – 7.03 (m, 3H), 5.88 (s, 1H).

^{13}C NMR (101 MHz, $CDCl_3$) δ 146.8, 141.2, 129.7, 126.8 (q, J = 3.8 Hz), 126.1, 124.7 (q, J = 270.9 Hz), 123.4, 123.1, 121.8 (q, J = 32.7 Hz), 120.2, 115.5.

^{19}F NMR (377 MHz, $CDCl_3$) δ -61.4. The spectral data are consistent with those reported in the literature.[31]

4-chloro-N-(4-(trifluoromethyl)phenyl)aniline (45)

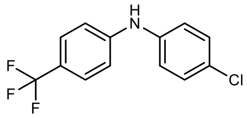

The compound was prepared according to **General Procedure B** with 4-bromobenzotrifluoride (28 µL, 0.2 mmol, 1.0 equiv.) and *p*-chloroaniline (76.5 mg, 0.6 mmol, 3.0 equiv.). The product was purified as a colorless oil by flash column chromatography on silica gel using hexane/EtOAc as eluent. Yield: 86% (46.9 mg).

^{1}H NMR (400 MHz, $CDCl_3$) δ 7.49 (d, J = 8.5 Hz, 2H), 7.32 – 7.25 (m, 2H), 7.12 – 7.04 (m, 2H), 7.02 (d, J = 8.4 Hz, 2H).

^{13}C NMR (101 MHz, $CDCl_3$) δ 146.4, 139.9, 129.7, 127.8, 126.9 (q, J = 3.8 Hz), 124.6 (q, J = 270.8 Hz), 123.3, 122.3 (q, J = 32.8 Hz), 121.2, 115.7, 29.9.

^{19}F NMR (377 MHz, $CDCl_3$) δ -61.5. The spectral data are consistent with those reported in the literature.[47]

bis(4-(trifluoromethyl)phenyl)amine (46)

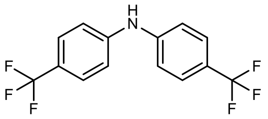

The compound was prepared according to **General Procedure B** with 4-bromobenzotrifluoride (28 µL, 0.2 mmol, 1.0 equiv.) and *p*-aminobenzotrifluoride (75.4 µL, 0.6 mmol, 3.0 equiv.). The product was purified as a colorless oil by flash column chromatography on silica gel using hexane/EtOAc as eluent. Yield: 63% (38.7 mg).

^{1}H NMR (500 MHz, $CDCl_3$) δ 7.55 (d, *J* = 8.3 Hz, 4H), 7.16 (d, *J* = 8.3 Hz, 4H), 6.07 (s, 1H).

^{13}C NMR (126 MHz, $CDCl_3$) δ 144.9, 127.0 (q, *J* = 3.8 Hz), 124.5 (q, *J* = 271.1 Hz), 123.7 (q, *J* = 33.0 Hz), 117.5.

^{19}F NMR (471 MHz, $CDCl_3$) δ -61.75. The spectral data are consistent with those reported in the literature.[48]

1-(3-((4-(trifluoromethyl)phenyl)amino)phenyl)ethan-1-one (47)

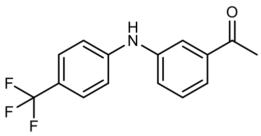

The compound was prepared according to **General Procedure B** with 4-bromobenzotrifluoride (28 µL, 0.2 mmol, 1.0 equiv.) and 3-aminoacetophenone (81.1 mg, 0.6 mmol, 3.0 equiv.). The product was purified as a white solid by flash column chromatography on silica gel using hexane/EtOAc as eluent. Yield: 83% (46.3 mg).

^{1}H NMR (500 MHz, $CDCl_3$) δ 7.72 (t, *J* = 2.0 Hz, 1H), 7.60 (dt, *J* = 7.7, 1.3 Hz, 1H), 7.50 (d, *J* = 8.3 Hz, 2H), 7.41 (t, *J* = 7.8 Hz, 1H), 7.35 (ddd, *J* = 8.0, 2.4, 1.1 Hz, 1H), 7.08 (d, *J* = 8.4 Hz, 2H), 2.60 (s, 3H).

^{13}C NMR (126 MHz, $CDCl_3$) δ 198.0, 146.1, 142.0, 138.7, 129.9, 127.0 (q, *J* = 3.8 Hz), 124.6 (q, *J* = 270.9 Hz), 123.9, 122.8, 122.7 (q, *J* = 32.8 Hz), 118.7, 116.1, 26.9.

^{19}F NMR (471 MHz, $CDCl_3$) δ -61.6.

HRMS (ESI): calculated for $[M+Na]^+$ $C_{10}H_{12}N_3F_3O_3Na^+$ 302.0723; found 302.0731.

2-methoxy-*N*-(4-(trifluoromethyl)phenyl)aniline (48)

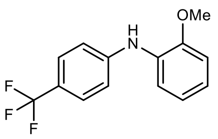

The compound was prepared according to **General Procedure B** with 4-bromobenzotrifluoride (28 µL, 0.2 mmol, 1.0 equiv.) and m-anisidine (73.9 mg, 0.6 mmol, 3.0 equiv.). The product was purified as a white solid by flash column chromatography on silica gel using hexane/EtOAc as eluent. Yield: 95% (51.0 mg).

^{1}H NMR (500 MHz, $CDCl_3$) δ 7.51 (d, *J* = 8.4 Hz, 2H), 7.39 (dd, *J* = 7.2, 2.3 Hz, 1H), 7.15 (d, *J* = 8.4 Hz, 2H), 7.04 – 6.98 (m, 1H), 6.98 – 6.92 (m, 2H), 3.90 (s, 3H).

^{13}C NMR (126 MHz, $CDCl_3$) δ 149.5, 146.4, 130.9, 126.7 (q, *J* = 3.8 Hz), 124.8 (q, *J* = 270.8 Hz), 122.2, 121.9 (q, *J* = 32.8 Hz), 120.9, 117.4, 116.1, 111.0, 55.7.

^{19}F NMR (471 MHz, $CDCl_3$) δ -61.4. The spectral data are consistent with those reported in the literature.[42]

***N*-(4-(trifluoromethyl)phenyl)pyridin-3-amine (49)**

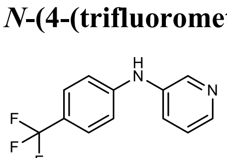

The compound was prepared according to **General Procedure B** with 4-bromobenzotrifluoride (28 µL, 0.2 mmol, 1.0 equiv.) and 3-aminopyridine (56.5 mg, 0.6 mmol, 3.0 equiv.). The product

was purified as a white solid by flash column chromatography on silica gel using hexane/EtOAc as eluent. Yield: 68% (32.2 mg).

^{1}H NMR (400 MHz, $CDCl_3$) δ 8.55 (d, *J* = 2.7 Hz, 1H), 8.24 (dd, *J* = 4.8, 1.4 Hz, 1H), 7.60 (ddd, *J* = 8.3, 2.8, 1.4 Hz, 1H), 7.54 – 7.47 (m, 2H), 7.30 (dd, *J* = 8.3, 4.8 Hz, 1H), 7.15 – 7.08 (m, 2H), 6.69 (s, 1H).

^{13}C NMR (101 MHz, $CDCl_3$) δ 145.4, 141.8, 140.6, 139.1, 127.0 (q, *J* = 3.8 Hz), 126.2, 124.5 (q, *J* = 270.7 Hz), 124.5, 123.3 (q, *J* = 32.5 Hz), 116.5.

^{19}F NMR (377 MHz, $CDCl_3$) δ -61.7. The spectral data are consistent with those reported in the literature.[49]

methyl 4-(3-(*tert*-butyl)ureido)benzoate (50)

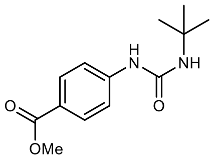

The compound was prepared according to **General Procedure B** with methyl 4-bromobenzoate (43.0 mg, 0.2 mmol, 1.0 equiv.), sodium, cyanate (19.5 mg, 0.3 mmol, 1.5 equiv.) and *tert*-butylamine (31.5 µL, 0.3 mmol, 1.5 equiv.). The product was purified as a white solid by flash column chromatography on silica gel using hexane/EtOAc as eluent. Yield: 95% (47.4 mg).

^{1}H NMR (400 MHz, $CDCl_3$) δ 8.10 (s, 1H), 7.87 – 7.81 (m, 2H), 7.38 – 7.32 (m, 2H), 3.84 (s, 3H), 1.31 (s, 9H).

^{13}C NMR (101 MHz, $CDCl_3$) δ 167.4, 155.2, 144.4, 130.9, 123.2, 117.7, 52.1, 50.8, 29.3.

HRMS (ESI): calculated for $[M+Na]^+$ $C_{13}H_{18}N_2O_3Na^+$ 273.1210; found 273.1215.

methyl 4-(((benzyloxy)carbonyl)amino)benzoate (51)

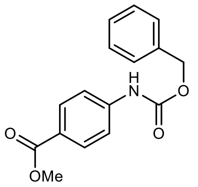

The compound was prepared according to **General Procedure B** with methyl 4-bromobenzoate (43.0 mg, 0.2 mmol, 1.0 equiv.), sodium, cyanate (19.5 mg, 0.3 mmol, 1.5 equiv.) and benzyl alcohol (61.8 µL, 0.6 mmol, 3 equiv.). The product was purified as a white solid by flash column chromatography on silica gel using hexane/EtOAc as eluent. Yield: 80% (45.5 mg).

^{1}H NMR (500 MHz, $CDCl_3$) δ 8.02 – 7.95 (m, 2H), 7.50 – 7.43 (m, 2H), 7.41 – 7.33 (m, 5H), 6.98 (s, 1H), 5.21 (s, 2H), 3.89 (s, 3H).

^{13}C NMR (126 MHz, $CDCl_3$) δ 166.8, 153.0, 142.2, 135.8, 131.1, 128.8, 128.7, 128.5, 125.0, 117.7, 67.5, 52.1. The spectral data are consistent with those reported in the literature.[50]

methyl 4-((oxodiphenyl-l6-sulfaneylidene)amino)benzoate (52)

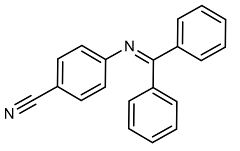

The compound was prepared according to **General Procedure B** with 4-bromobenzonitrile (36.4 mg, 0.2 mmol, 1.0 equiv.), benzophenone imine (100 µL, 0.6 mmol, 3.0 equiv.). The product was purified as a pale yellow solid by flash column chromatography on silica gel using hexane/EtOAc with addition of Et_3N as eluent. Yield: 77% (44.0 mg

^{1}H NMR (400 MHz, $CDCl_3$) δ 7.79 – 7.70 (m, 2H), 7.56 – 7.48 (m, 1H), 7.48 – 7.37 (m, 4H), 7.36 – 7.23 (m, 3H), 7.15 – 7.04 (m, 2H), 6.82 – 6.73 (m, 2H).

^{13}C NMR (101 MHz, $CDCl_3$) δ 169.7, 155.5, 138.7, 135.3, 132.9, 131.6, 129.7, 129.4, 128.5, 128.3, 121.5, 119.4, 106.3. The spectral data are consistent with those reported in the literature.[51]

(53)

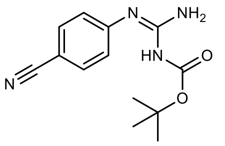

The compound was prepared according to **General Procedure B** with 4-bromobenzonitrile (36.4 mg, 0.2 mmol, 1.0 equiv.), *tert*-butoxycarbonylguanidine (95.5 mg, 0.6 mmol, 3.0 equiv.). The product was purified as a white solid by flash column chromatography on silica gel using DCM/EtOAc as eluent. Yield: 93% (48.5 mg).

^{1}H NMR (400 MHz, DMSO) δ 9.23 (s, 1H), 7.67 – 7.59 (m, 2H), 7.56 – 7.48 (m, 2H), 7.47 – 7.18 (m, 1H), 1.33 (s, 9H).

^{13}C NMR (101 MHz, DMSO) δ 133.1, 120.5, 119.4, 103.7, 77.9, 28.1. The spectral data are consistent with those reported in the literature.[52]

methyl 4-((oxodiphenyl-l6-sulfaneylidene)amino)benzoate (54)

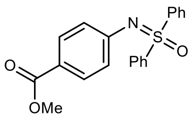

The compound was prepared according to modified **General Procedure B** with methyl 4-bromobenzoate (43.0 mg, 0.2 mmol, 1.0 equiv.), S,S-diphenylsulfoximine (130 mg, 0.6 mmol, 3.0 equiv.) within 72 hours. The product was purified as a white solid by flash column chromatography on silica gel using DCM/MeOH as eluent. Yield: 66% (46.7 mg).

^{1}H NMR (500 MHz, $CDCl_3$) δ 8.07 – 8.02 (m, 4H), 7.85 – 7.80 (m, 2H), 7.55 – 7.45 (m, 6H), 7.18 – 7.14 (m, 2H), 3.82 (s, 3H).

^{13}C NMR (126 MHz, $CDCl_3$) δ 167.2, 149.9, 140.4, 133.1, 130.9, 129.6, 128.5, 123.2, 123.2, 51.8. The spectral data are consistent with those reported in the literature.[53]

methyl 4-((dimethyl(oxo)-l6-sulfaneylidene)amino)benzoate (55)

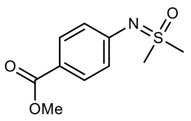

The compound was prepared according to **General Procedure B** with methyl 4-bromobenzoate (43.0 mg, 0.2 mmol, 1.0 equiv.) and S,S-dimethylsulfoximine (55.9 mg, 0.6 mmol, 3.0 equiv.). The product was purified as a white solid by flash column chromatography on silica gel using DCM/MeOH as eluent. Yield: 90% (40.8 mg).

^{1}H NMR (500 MHz, $CDCl_3$) δ 7.92 – 7.85 (m, 2H), 7.10 – 7.02 (m, 2H), 3.85 (s, 3H), 3.17 (s, 6H).

^{13}C NMR (126 MHz, $CDCl_3$) δ 167.1, 150.4, 131.1, 123.4, 122.3, 51.9, 42.4. The spectral data are consistent with those reported in the literature.[53]

((4-benzoylphenyl)imino)dimethyl-l6-sulfanone (56)

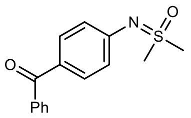

The compound was prepared according to **General Procedure B** with 4-bromobenzophenone (52.2 mg, 0.2 mmol, 1.0 equiv.) and dimethylsulfoximine (55.9 mg, 0.6 mmol, 3.0 equiv.). The product was purified as a beige solid by flash column chromatography on silica gel using DCM/MeOH as eluent. Yield: 94% (51.3 mg).

^{1}H NMR (400 MHz, $CDCl_3$) δ 7.77 – 7.68 (m, 4H), 7.56 – 7.50 (m, 1H), 7.47 – 7.40 (m, 2H), 7.14 – 7.08 (m, 2H), 3.20 (s, 6H).

^{13}C NMR (101 MHz, $CDCl_3$) δ 195.8, 150.4, 138.3, 132.1, 131.9, 130.8, 129.7, 128.2, 122.2, 42.4.

HRMS (ESI): calculated for $[M+Na]^+$ $C_{15}H_{15}NSO_2Na^+$ 296.0716; found 296.0714.

4-methyl-N-(4-(trifluoromethyl)phenyl)benzenesulfonamide (57)

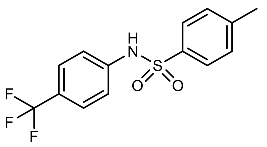

The compound was prepared according to **General Procedure C** with 4-bromobenzotrifluoride (28 μL, 0.2 mmol, 1.0 equiv.), *p*-toluenesulfonamide (103 mg, 0.6 mmol, 3.0 equiv.). The product was purified as a white solid by flash column chromatography on silica gel using hexane/EtOAc as eluent. Yield: 85% (53.8 mg).

^{1}H NMR (500 MHz, CDCl$_3$) δ 8.00 (s, 1H), 7.83 – 7.78 (m, 2H), 7.51 – 7.46 (m, 2H), 7.31 – 7.22 (m, 4H), 2.40 (s, 3H).

^{13}C NMR (126 MHz, CDCl$_3$) δ 144.6, 140.0, 135.6, 130.0, 127.3, 126.6 (q, *J* = 3.8 Hz), 126.5 (q, *J* = 33.0 Hz), 123.9 (q, *J* = 271.8 Hz), 119.6, 21.5.

^{19}F NMR (471 MHz, CDCl$_3$) δ -62.2. The spectral data are consistent with those reported in the literature.[54]

N-(4-benzoylphenyl)-4-methylbenzenesulfonamide (58)

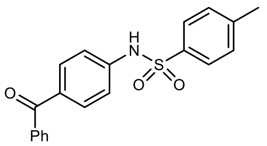

The compound was prepared according to **General Procedure C** with 4-bromobenzophenone (52.2 mg, 0.2 mmol, 1.0 equiv.), *p*-toluenesulfonamide (103 mg, 0.6 mmol, 3.0 equiv.). The product was purified as a white solid by flash column chromatography on silica gel using hexane/EtOAc as eluent. Yield: 84% (58.8 mg).

^{1}H NMR (400 MHz, CDCl$_3$) δ 7.80 – 7.74 (m, 2H), 7.74 – 7.69 (m, 4H), 7.60 – 7.54 (m, 2H), 7.49 – 7.43 (m, 2H), 7.29 – 7.24 (m, 2H), 7.21 – 7.16 (m, 2H), 2.39 (s, 3H).

^{13}C NMR (101 MHz, CDCl$_3$) δ 195.7, 144.6, 140.9, 137.7, 135.9, 133.5, 132.5, 132.0, 130.1, 130.0, 128.4, 127.4, 118.8, 21.7. The spectral data are consistent with those reported in the literature.[55]

N-(4-(trifluoromethyl)phenyl)methanesulfonamide (59)

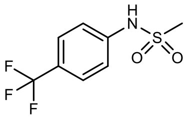

The compound was prepared according to **General Procedure C** with 4-bromobenzotrifluoride (28 μL, 0.2 mmol, 1.0 equiv.), methanesulfonamide (57.1 mg, 0.6 mmol, 3.0 equiv.). The product was purified as a white solid by flash column chromatography on silica gel using hexane/EtOAc as eluent. Yield: 90% (43.1 mg).

^{1}H NMR (400 MHz, CDCl$_3$) δ 7.63 – 7.58 (m, 2H), 7.43 (s, 1H), 7.35 – 7.30 (m, 2H), 3.10 (s, 3H).

^{13}C NMR (101 MHz, CDCl$_3$) δ 140.2, 127.2 (q, *J* = 3.7 Hz), 127.0 (q, *J* = 33.0 Hz), 124.0 (q, *J* = 271.8 Hz), 119.2, 40.0.

^{19}F NMR (377 MHz, CDCl$_3$) δ -62.3. The spectral data are consistent with those reported in the literature.[56]

benzyl 2-(4-(methoxycarbonyl)phenyl)hydrazine-1-carboxylate (60)

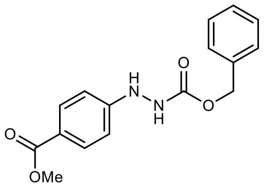

The compound was prepared according to **General Procedure C** with methyl 4-bromobenzoate (43.0 mg, 0.2 mmol, 1.0 equiv.), benzyl carbazate (99.7 mg, 0.6 mmol, 3.0 equiv.). The product was purified as a white solid by flash column chromatography on neutral alumina using DCM/MeOH as eluent. Yield: 77% (46.1 mg).

^{1}H NMR (400 MHz, CDCl$_3$) δ 7.90 – 7.85 (m, 2H), 7.42 – 7.27 (m, 5H), 6.91 (s, 1H), 6.76 – 6.70 (m, 2H), 5.15 (s, 2H), 3.85 (s, 3H).

^{13}C NMR (101 MHz, CDCl$_3$) δ 167.1, 156.9, 152.0, 135.6, 131.4, 128.7, 122.2, 111.9, 67.9, 51.9.

HRMS (ESI): calculated for $[M+Na]^+$ $C_{16}H_{16}N_2O_4Na^+$ 323.1002; found 323.1005.

***tert*-butyl 2-(4-(methoxycarbonyl)phenyl)hydrazine-1-carboxylate (61)**

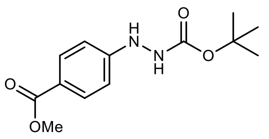

The compound was prepared according to **General Procedure C** with methyl methyl 4-bromobenzoate (43.0 mg, 0.2 mmol, 1.0 equiv.), *tert*-butyl carbazate (79.3 mg, 0.6 mmol, 3.0 equiv.). The product was purified as a white solid by flash column chromatography on neutral alumina using DCM/MeOH as eluent. Yield: 79% (42.1 mg).

^{1}H NMR (500 MHz, DMSO) δ 8.94 (s, 1H), 8.32 (s, 1H), 7.79 – 7.73 (m, 2H), 6.70 – 6.64 (m, 2H), 3.76 (s, 3H), 1.42 (s, 9H).

^{13}C NMR (126 MHz, DMSO) δ 166.2, 155.8, 153.8, 130.8, 118.5, 110.5, 79.2, 51.4, 28.1. The spectral data are consistent with those reported in the literature.[57]

***N*'-(4-cyanophenyl)benzohydrazide (62)**

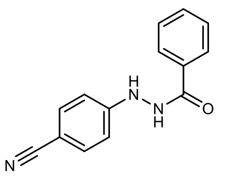

The compound was prepared according to **General Procedure C** with 4-bromobenzonitrile (36.4 mg, 0.2 mmol, 1.0 equiv.), phenylhydrazide (81.7 mg, 0.6 mmol, 3.0 equiv.). The product was purified as a white solid by flash column chromatography on neutral alumina using DCM/MeOH as eluent. Yield: 65% (31.0 mg).

^{1}H NMR (500 MHz, DMSO) δ 10.59 – 10.45 (m, 1H), 8.81 (s, 1H), 7.96 – 7.89 (m, 2H), 7.63 – 7.48 (m, 5H), 6.86 – 6.79 (m, 2H).

^{13}C NMR (126 MHz, DMSO) δ 166.4, 153.1, 133.5, 132.5, 132.0, 128.6, 127.4, 120.1, 111.8, 99.0. The spectral data are consistent with those of the literature.[58]

1-(diphenylmethylene)-2-(4-(trifluoromethyl)phenyl)hydrazine (63)

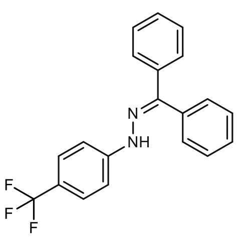

The compound was prepared according to **General Procedure A** with 4-bromobenzotrifluoride (28 µL, 0.2 mmol, 1.0 equiv.) and benzophenone hydrazone (118 mg, 0.6 mmol, 3.0 equiv.). The product was purified as a yellow solid by flash column chromatography on neutral alumina using hexane/EtOAc as eluent. Yield: 91% (62.1 mg).

^{1}H NMR (500 MHz, $CDCl_3$) δ 7.69 – 7.65 (m, 1H), 7.65 – 7.59 (m, 4H), 7.59 – 7.54 (m, 1H), 7.53 – 7.47 (m, 2H), 7.39 – 7.31 (m, 5H), 7.16 – 7.10 (m, 2H).

^{13}C NMR (126 MHz, $CDCl_3$) δ 147.2, 146.4, 138.0, 132.5, 129.9, 129.7, 129.1, 128.7, 128.4, 126.9, 126.7 (q, J = 3.9 Hz), 124.9 (q, J = 270.7 Hz), 121.7 (q, J = 32.6 Hz), 112.6. The spectral data are consistent with those reported in the literature.[59]

***N*-(4-(trifluoromethyl)phenyl)benzamide (64)**

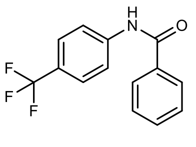

The compound was prepared according to **General Procedure D** with 4-bromobenzotrifluoride (28 µL, 0.2 mmol, 1.0 equiv.), benzamide (72.7 mg, 0.6 mmol, 3.0 equiv.). The product was purified as a white solid by flash column chromatography on silica gel using hexane/EtOAc as eluent. Yield: 61% (32.6 mg).

^{1}H NMR (500 MHz, $CDCl_3$) δ 7.94 (s, 1H), 7.91 – 7.86 (m, 2H), 7.81 – 7.76 (m, 2H), 7.66 – 7.62 (m, 2H), 7.61 – 7.56 (m, 1H), 7.55 – 7.49 (m, 2H). **^{13}C NMR (126 MHz, $CDCl_3$)** δ 165.9, 141.1, 134.6, 132.5, 129.1, 127.2, 126.6 (q, J = 4.0 Hz), 126.5 (q, J = 31.8 Hz), 124.2 (q, J = 271.7 Hz), 119.9.

^{19}F NMR (471 MHz, $CDCl_3$) δ -62.1. The spectral data are consistent with those reported in the literature.[60]

1-(4-(trifluoromethyl)phenyl)pyrrolidin-2-one (65)

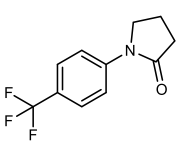

The compound was prepared according to **General Procedure D** with 4-bromobenzotrifluoride (28 μL, 0.2 mmol, 1.0 equiv.), 2-pyrrolidinone (51.1 mg, 0.6 mmol, 3.0 equiv.). The product was purified as a white solid by flash column chromatography on silica gel using hexane/EtOAc as eluent. Yield: 79% (36.1 mg).

^{1}H NMR (500 MHz, $CDCl_3$) δ 7.77 (d, *J* = 8.5 Hz, 2H), 7.61 (d, *J* = 8.6 Hz, 2H), 3.89 (t, *J* = 7.0 Hz, 2H), 2.64 (t, *J* = 8.1 Hz, 2H), 2.24 – 2.15 (m, 2H).

^{13}C NMR (126 MHz, $CDCl_3$) δ 174.7, 142.3, 126.0 (q, *J* = 3.8 Hz), 126.0 (q, *J* = 32.8 Hz), 124.1 (q, *J* = 271.5 Hz), 119.2, 48.5, 32.8, 17.9.

^{19}F NMR (471 MHz, $CDCl_3$) δ -62.2. The spectral data are consistent with those reported in the literature.[61]

methyl 4-(2,2,2-trifluoroacetamido)benzoate (66)

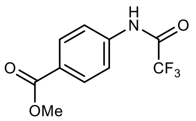

The compound was prepared according to **General Procedure D** with methyl 4-bromobenzoate (43.0 mg, 0.2 mmol, 1.0 equiv.), trifluoroacetamide (67.8 mg, 0.6 mmol, 3.0 equiv.). The product was purified as a white solid by flash column chromatography on silica gel using hexane/EtOAc as eluent. Yield: 79% (39.2 mg).

^{1}H NMR (500 MHz, $CDCl_3$) δ 8.57 (s, 1H), 8.07 – 8.01 (m, 2H), 7.72 – 7.67 (m, 2H), 3.91 (s, 3H).

^{13}C NMR (126 MHz, $CDCl_3$) δ 166.5, 155.2 (q, *J* = 37.8 Hz), 139.5, 131.1, 127.8, 120.1, 115.7 (q, *J* = 288.6 Hz), 52.4.

^{19}F NMR (471 MHz, $CDCl_3$) δ -75.7. The spectral data are consistent with those reported in the literature.[62]

N-(4-(trifluoromethyl)phenyl)butyramide (67)

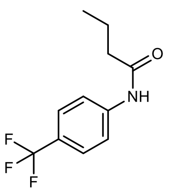

The compound was prepared according to **General Procedure D** with 4-bromobenzotrifluoride (28 μL, 0.2 mmol, 1.0 equiv.), butyramide (52.3 mg, 0.6 mmol, 3.0 equiv.). The product was purified as a white solid by flash column chromatography on silica gel using hexane/EtOAc as eluent. Yield: 69% (32.1 mg).

^{1}H NMR (500 MHz, $CDCl_3$) δ 7.65 (d, *J* = 8.4 Hz, 2H), 7.56 (d, *J* = 8.5 Hz, 2H), 7.39 (s, 1H), 2.37 (t, *J* = 7.4 Hz, 2H), 1.81 – 1.72 (m, 2H), 1.01 (t, *J* = 7.4 Hz, 3H).

^{13}C NMR (126 MHz, $CDCl_3$) δ 171.7, 141.1, 126.4 (q, J = 3.8 Hz), 126.1 (q, J = 32.7 Hz), 124.2 (q, J = 271.3 Hz), 119.4, 39.8, 19.1, 13.8. **^{19}F NMR (471 MHz, $CDCl_3$)** δ -62.1.

HRMS (ESI): calculated for $[M+Na]^+$ $C_{11}H_{12}NOF_3Na^+$ 254.0763; found 254.0770.

benzyl (4-(trifluoromethyl)phenyl)carbamate (68)

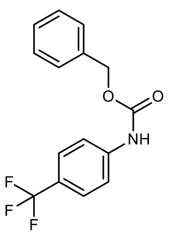

The compound was prepared according to **General Procedure D** with 4-bromobenzotrifluoride (28 μL, 0.2 mmol, 1.0 equiv.), benzylcarbamate (90.7 mg, 0.6 mmol, 3.0 equiv.). The product was purified as a white solid by flash column chromatography on silica gel using hexane/EtOAc as eluent. Yield: 58% (34.5mg).

^{1}H NMR (400 MHz, DMSO) δ 10.18 (s, 1H), 7.67 – 7.58 (m, 4H), 7.43 – 7.29 (m, 5H), 5.14 (s, 2H).

^{13}C NMR (101 MHz, DMSO) δ 153.3, 142.9, 136.3, 128.5, 128.3, 128.2, 126.2 (q, J = 3.9 Hz), 124.5 (q, J = 271.1 Hz), 122.6 (q, J = 32.1 Hz), 117.9, 66.2.

^{19}F NMR (377 MHz, DMSO) δ -60.2. The spectral data are consistent with those reported in the literature.[62]

methyl 4-((methoxycarbonyl)amino)benzoate (69)

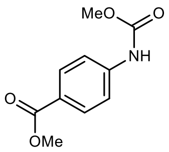

The compound was prepared according to **General Procedure D** with methyl 4-bromobenzoate (43.0 mg, 0.2 mmol, 1.0 equiv.), methyl carbamate (90.7 mg, 0.6 mmol, 3.0 equiv.). The product was purified as a white solid by flash column chromatography on silica gel using hexane/EtOAc as eluent. Yield: 66% (27.8 mg).

^{1}H NMR (500 MHz, CDCl$_3$) δ 8.01 – 7.97 (m, 2H), 7.48 – 7.43 (m, 2H), 6.91 – 6.85 (m, 1H), 3.89 (s, 3H), 3.79 (s, 3H).

^{13}C NMR (126 MHz, CDCl$_3$) δ 166.8, 153.7, 142.3, 131.1, 125.0, 117.7, 52.7, 52.1. The spectral data are consistent with those reported in the literature.[63]

methyl 4-(2-oxooxazolidin-3-yl)benzoate (70)

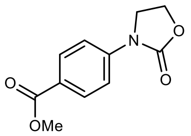

The compound was prepared according to **General Procedure D** with methyl 4-bromobenzoate (43.0 mg, 0.2 mmol, 1.0 equiv.), 2-oxazolidinone (52.2 mg, 0.6 mmol, 3.0 equiv.). The product was purified as a white solid by flash column chromatography on silica gel using DCM as eluent. Yield: 67% (29.7 mg

^{1}H NMR (400 MHz, CDCl$_3$) δ 8.08 – 8.02 (m, 2H), 7.66 – 7.60 (m, 2H), 4.56 – 4.47 (m, 2H), 4.15 – 4.06 (m, 2H), 3.91 (s, 3H).

^{13}C NMR (101 MHz, CDCl$_3$) δ 166.7, 155.0, 142.3, 130.9, 125.5, 117.3, 61.5, 52.2, 45.1. The spectral data are consistent with those reported in the literature.[64]

P,P-diphenyl-N-(4-(trifluoromethyl)phenyl)phosphinic amide (71)

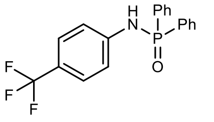

The compound was prepared according to **General Procedure D** with 4-bromobenzotrifluoride (28 µL, 0.2 mmol, 1.0 equiv.), diphenylphosphinamide (130 mg, 0.6 mmol, 3.0 equiv.). The product was purified as a white solid by flash column chromatography on neutral alumina using DCM/MeOH as eluent. Yield: 58% (41.8 mg).

^{1}H NMR (500 MHz, CDCl$_3$) δ 7.89 – 7.80 (m, 4H), 7.55 – 7.48 (m, 2H), 7.48 – 7.39 (m, 4H), 7.33 (d, *J* = 8.4 Hz, 2H), 7.05 (d, *J* = 8.4 Hz, 2H), 6.37 (s, 1H).

^{13}C NMR (126 MHz, CDCl$_3$) δ 143.8, 132.8 (d, *J* = 2.9 Hz), 132.0 (d, *J* = 10.3 Hz), 130.7 (d, *J* = 129.5 Hz), 129.1 (d, *J* = 13.2 Hz), 126.6 (q, *J* = 4.0 Hz), 124.4 (q, *J* = 270.9 Hz), 123.8 (q, *J* = 32.7 Hz), 118.3 (d, *J* = 6.7 Hz).

^{19}F NMR (471 MHz, CDCl$_3$) δ -61.9.

^{31}P NMR (202 MHz, CDCl$_3$) δ 20.17. The spectral data are consistent with those reported in the literature.[65]

methyl 4-((diphenylphosphoryl)amino)benzoate (72)

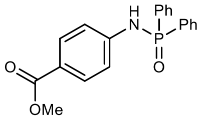

The compound was prepared according to **General Procedure D** with methyl 4-bromobenzoate (43.0 mg, 0.2 mmol, 1.0 equiv.), diphenylphosphinamide (130 mg, 0.6 mmol, 3.0 equiv.). The product was purified as a white solid by flash column chromatography on neutral alumina using DCM/MeOH as eluent. Yield: 53% (37.4 mg).

^{1}H NMR (400 MHz, DMSO) δ 8.79 (s, 1H), 7.82 – 7.75 (m, 3H), 7.75 – 7.70 (m, 2H), 7.63 – 7.56 (m, 2H), 7.56 – 7.50 (m, 2H), 3.75 (s, 3H).

^{13}C NMR (101 MHz, DMSO) δ 165.9, 147.2, 132.9, 132.2 (d, *J* = 3.0 Hz), 131.6 (d, *J* = 10.4 Hz), 130.5, 128.9 (d, *J* = 12.6 Hz), 121.5, 117.6 (d, *J* = 7.3 Hz), 51.7.

^{31}P NMR (162 MHz, DMSO) δ 17.1.

HRMS (ESI): calculated for $[M+Na]^+$ $C_{20}H_{18}NO_3PNa^+$ 374.0917; found 374.0906

1-(4-(trifluoromethyl)phenyl)indoline (73)

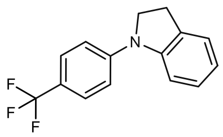

The compound was prepared according to **General Procedure A** with 4-bromobenzotrifluoride (28.0 μL, 0.2 mmol, 1.0 equiv.) and indoline (67.3 μL, 0.6 mmol, 3.0 equiv.). The product was purified as a white solid by flash column chromatography on silica gel using hexane/EtOAc as eluent. Yield: 80% (42.2 mg).

^{1}H NMR (500 MHz, CDCl$_3$) δ 7.57 (d, *J* = 8.6 Hz, 2H), 7.30 – 7.18 (m, 4H), 7.13 (t, *J* = 7.7 Hz, 1H), 6.84 (t, *J* = 7.4 Hz, 1H), 3.99 (t, *J* = 8.4 Hz, 2H), 3.16 (t, *J* = 8.4 Hz, 2H).

^{13}C NMR (126 MHz, CDCl$_3$) δ 146.8, 145.7, 131.9, 127.3, 126.5 (q, *J* = 3.9 Hz), 125.5, 124.8 (q, *J* = 270.6 Hz), 121.7 (q, *J* = 32.8 Hz), 120.3, 116.1, 109.2, 52.0, 28.2.

^{19}F NMR (471 MHz, CDCl$_3$) δ -61.44. The spectral data are consistent with those reported in the literature.[66]

4-(9*H*-carbazol-9-yl)benzonitrile (74)

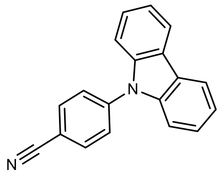

The compound was prepared according to **General Procedure D** with 4-bromobenzonitrile (36.4 mg, 0.2 mmol, 1.0 equiv.), carbazole (100 mg, 0.6 mmol, 3.0 equiv.). The product was purified as a white solid by flash column chromatography on silica gel using hexane/EtOAc as eluent. Yield: 84% (45.0 mg).

^{1}H NMR (500 MHz, CDCl$_3$) δ 8.18 – 8.12 (m, 2H), 7.93 – 7.88 (m, 2H), 7.77 – 7.71 (m, 2H), 7.49 – 7.41 (m, 4H), 7.39 – 7.31 (m, 2H).

^{13}C NMR (126 MHz, CDCl$_3$) δ 142.2, 140.0, 134.1, 127.2, 126.5, 124.1, 121.1, 120.7, 118.5, 110.6, 109.7. The spectral data are consistent with those reported in the literature.[67]

4-(5-methoxy-1*H*-indol-1-yl)benzonitrile (75)

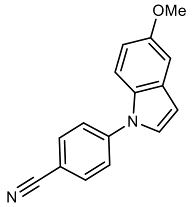

The compound was prepared according to **General Procedure D** with 4-bromobenzonitrile (36.4 mg, 0.2 mmol, 1.0 equiv.), 5-methoxy-1*H*-indole (88.3 mg, 0.6 mmol, 3.0 equiv.). The product was purified as a white solid by flash column chromatography on silica gel using hexane/EtOAc as eluent. Yield: 74% (36.9 mg).

^{1}H NMR (400 MHz, CDCl$_3$) δ 7.82 – 7.77 (m, 2H), 7.64 – 7.59 (m, 2H), 7.51 (d, *J* = 9.0 Hz, 1H), 7.32 (d, *J* = 3.4 Hz, 1H), 7.14 (d, *J* = 2.5 Hz, 1H), 6.92 (dd, *J* = 9.0, 2.5 Hz, 1H), 6.67 (dd, *J* = 3.3, 0.8 Hz, 1H), 3.88 (s, 3H).

^{13}C NMR (101 MHz, CDCl$_3$) δ 155.3, 143.8, 133.9, 130.8, 130.4, 127.6, 123.5, 118.6, 113.2, 111.4, 109.1, 105.6, 103.3, 55.9. The spectral data are consistent with those reported in the literature.[68]

4-(1*H*-benzo[*d*]imidazol-1-yl)benzonitrile (76)

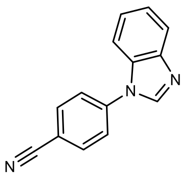

The compound was prepared according to modified **General Procedure D** with 4-bromobenzonitrile (36.4 mg, 0.2 mmol, 1.0 equiv.), 5-methoxy-1*H*-indole (88.3 mg, 0.6 mmol, 3.0 equiv.) within 72 hours. The product was purified as a white solid by flash column chromatography on neutral alumina using DCM/MeOH as eluent. Yield: 63% (27.6 mg).

^{1}H NMR (400 MHz, CDCl$_3$) δ 8.27 (s, 1H), 7.95 – 7.88 (m, 3H), 7.74 – 7.67 (m, 2H), 7.61 – 7.55 (m, 1H), 7.40 (dd, *J* = 6.1, 3.2 Hz, 2H).

^{13}C NMR (101 MHz, CDCl$_3$) δ 143.8, 141.6, 140.1, 134.4, 132.8, 124.8, 124.2, 124.0, 121.0, 117.9, 111.9, 110.4. The spectral data are consistent with those reported in the literature.[69]

4-(((4-methoxyphenyl)(methyl)amino)methyl)benzonitrile (77)

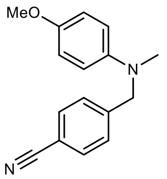

The compound was prepared according to modified **General Procedure D** with 4-bromobenzonitrile (36.4 mg, 0.2 mmol, 1.0 equiv.), 4-methoxy-*N*,*N*-dimethylaniline (190 µL, 0.6 mmol, 3.0 equiv.) and without addition of triethylamine. The product was purified as a white solid by flash column chromatography on silica gel using hexane/EtOAc as eluent. Yield: 70% (35.2 mg).

^{1}H NMR (400 MHz, CDCl$_3$) δ 7.63 – 7.56 (m, 2H), 7.39 – 7.33 (m, 2H), 6.85 – 6.80 (m, 2H), 6.73 – 6.66 (m, 2H), 4.47 (s, 2H), 3.75 (s, 3H), 2.95 (s, 3H).

^{13}C NMR (101 MHz, CDCl$_3$) δ 152.3, 145.2, 144.1, 132.4, 127.8, 119.0, 114.9, 114.7, 110.8, 58.0, 55.8, 39.6.

HRMS (ESI): calculated for $[M+Na]^+$ $C_{16}H_{16}N_2ONa^+$ 275.1155; found 275.1141.

methyl [1,1'-biphenyl]-4-carboxylate (78)

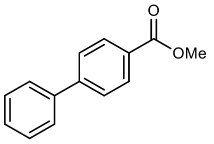

The compound was prepared according to modified **General Procedure E** with methyl 4-bromobenzoate (86.0 mg, 0.4 mmol, 2.0 equiv.), phenylhydrazine (19.8 µL, 0.2 mmol, 1.0 equiv.), TMG (90.5 µL, 0.72 mmol, 3.6 equiv) and without addition of triethylamine. The product was purified as a pale yellow solid by flash column chromatography on silica gel using hexane/EtOAc as eluent. Yield: 46% (19.6 mg) (with respect to phenylhydrazine).

^{1}H NMR (400 MHz, CDCl$_3$) δ 8.13 – 8.08 (m, 2H), 7.69 – 7.60 (m, 4H), 7.50 – 7.44 (m, 2H), 7.43 – 7.37 (m, 1H), 3.94 (s, 3H).

^{13}C NMR (101 MHz, CDCl$_3$) δ 167.2, 145.8, 140.1, 130.2, 129.1, 129.0, 128.3, 127.4, 127.2, 52.3. The spectral data are consistent with those reported in the literature.[70]

4-cyanophenyl 4-methylbenzoate (79)

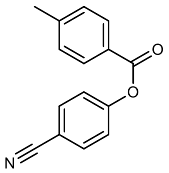

The compound was prepared according to **General Procedure D** with 4-bromobenzonitrile (36.4 mg, 0.2 mmol, 1.0 equiv.), *p*-toluic acid (81.7 mg, 0.6 mmol, 3.0 equiv.). The product was purified as a white solid by flash column chromatography on silica gel using hexane/EtOAc as eluent. Yield: 74% (35.2 mg).

^{1}H NMR (500 MHz, CDCl$_3$) δ 8.10 – 8.05 (m, 2H), 7.75 – 7.70 (m, 2H), 7.39 – 7.30 (m, 4H), 2.46 (s, 3H).

^{13}C NMR (126 MHz, CDCl$_3$) δ 164.5, 154.5, 145.3, 133.8, 130.4, 129.6, 126.0, 123.1, 118.4, 109.8, 21.9. The spectral data are consistent with those reported in the literature.[71]

4-cyanophenyl 4-methylbenzoate (80)

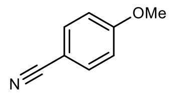

The compound was prepared according to modified **General Procedure E** with 4-bromobenzonitrile (36.4 mg, 0.2 mmol, 1.0 equiv.), methanol (50 μL, 1.2 mmol, 6.0 equiv.) and TMG (37.7 μL, 0.3 mmol, 1.5 equiv.). The product was purified as a white solid by flash column chromatography on silica gel using hexane/EtOAc as eluent. Yield: 90% (24.0 mg).

^{1}H NMR (400 MHz, CDCl$_3$) δ 7.60 – 7.55 (m, 2H), 6.96 – 6.91 (m, 2H), 3.85 (s, 3H).

^{13}C NMR (101 MHz, CDCl$_3$) δ 162.9, 134.1, 119.3, 114.8, 104.0, 55.6. The spectral data are consistent with those reported in the literature.[72]

4-(hexyloxy)benzonitrile (81)

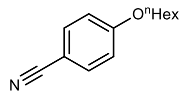

The compound was prepared according to **General Procedure E** with 4-bromobenzonitrile (36.4 mg, 0.2 mmol, 1.0 equiv.), *n*-hexanol (74.8 μL, 0.6 mmol, 3.0 equiv.). The product was purified as a white solid by flash column chromatography on silica gel using hexane/EtOAc as eluent. Yield: 69% (28.2 mg).

^{1}H NMR (400 MHz, CDCl$_3$) δ 7.60 – 7.53 (m, 2H), 6.96 – 6.89 (m, 2H), 3.99 (t, J = 6.5 Hz, 2H), 1.84 – 1.74 (m, 2H), 1.49 – 1.41 (m, 2H), 1.37 – 1.30 (m, 4H), 0.93 – 0.86 (m, 3H).

^{13}C NMR (101 MHz, CDCl$_3$) δ 162.6, 134.1, 119.5, 115.3, 103.7, 68.5, 31.6, 29.1, 25.7, 22.7, 14.1. The spectral data are consistent with those reported in the literature.[73]

methyl 4-(4-methoxyphenoxy)benzoate (82)

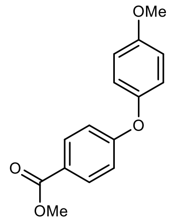

The compound was prepared according to **General Procedure D** with methyl 4-bromobenzoate (43.0 mg, 0.2 mmol, 1.0 equiv.), 4-methoxyphenol (74.5 mg, 0.6 mmol, 3.0 equiv.). The product was purified as a white solid by flash column chromatography on silica gel using hexane/EtOAc as eluent. Yield: 50% (25.9 mg).

^{1}H NMR (500 MHz, CDCl$_3$) δ 8.00 – 7.95 (m, 2H), 7.04 – 6.99 (m, 2H), 6.95 – 6.89 (m, 4H), 3.89 (s, 3H), 3.82 (s, 3H).

^{13}C NMR (126 MHz, CDCl$_3$) δ 166.8, 162.9, 156.8, 148.8, 131.8, 124.0, 121.8, 116.5, 115.2, 55.8, 52.1. The spectral data are consistent with those reported in the literature.[74]

4-hydroxybenzonitrile (83)

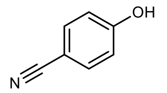

The compound was prepared according to **General Procedure B** with 4-bromobenzonitrile (36.4 mg, 0.2 mmol, 1.0 equiv.), water (50 μL, 2.8 mmol, 13.9 equiv.). The product was purified as a white solid by flash column chromatography on silica gel using hexane/EtOAc as eluent. Yield: 98% (23.4 mg).

^{1}H NMR (400 MHz, CDCl$_3$) δ 7.59 – 7.51 (m, 2H), 6.97 – 6.91 (m, 2H).

^{13}C NMR (101 MHz, CDCl$_3$) δ 160.4, 134.5, 119.4, 116.6, 103.1. The spectral data are consistent with those reported in the literature.[75]

4-chlorobenzonitrile (84)

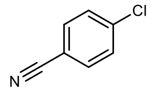

The compound was prepared according to **General Procedure B** with 4-bromobenzonitrile (36.4 mg, 0.2 mmol, 1.0 equiv.), lithium chloride (25.4 mg, 0.6 mmol, 3.0 equiv.). The product was purified as a white solid by flash column chromatography on silica gel using hexane/EtOAc as eluent. Yield: 73% (20.0 mg).

^{1}H NMR (400 MHz, CDCl$_3$) δ 7.63 – 7.56 (m, 2H), 7.50 – 7.43 (m, 2H).
^{13}C NMR (101 MHz, CDCl$_3$) δ 139.7, 133.5, 129.8, 118.1, 110.9. The spectral data are consistent with those reported in the literature.[72]

dimethyl 4,4'-thiodibenzoate (85)

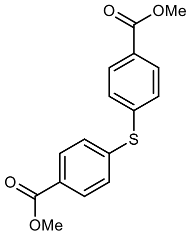

The compound was prepared according to **General Procedure B** with methyl 4-bromobenzoate (43.0 mg, 0.2 mmol, 2.0 equiv.), potassium thiocyanate (29.2 mg, 0.3 mmol, 3 equiv.). The product was purified as a white solid by flash column chromatography on silica gel using hexane/EtOAc as eluent. Yield: 82% (24.7 mg).
^{1}H NMR (500 MHz, CDCl$_3$) δ 8.00 – 7.94 (m, 4H), 7.40 – 7.35 (m, 4H), 3.91 (s, 6H).
^{13}C NMR (126 MHz, CDCl$_3$) δ 166.6, 140.9, 130.6 (2C), 129.2, 52.4. The spectral data are consistent with those reported in the literature.[76]

4-(decylthio)benzonitrile (86)

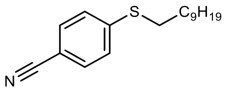

The compound was prepared according to **General Procedure F** with 4-bromobenzonitrile (36.4 mg, 0.2 mmol, 1.0 equiv.), decane-1-thiol (63.5 μL, 0.3 mmol, 1.5 equiv.). The product was purified as a pale-yellow solid by flash column chromatography on silica gel using hexane/EtOAc as eluent. Yield: 98% (54.0 mg).
^{1}H NMR (500 MHz, CDCl$_3$) δ 7.51 (d, J = 8.6 Hz, 2H), 7.28 (d, J = 8.6 Hz, 2H), 2.96 (t, J = 7.4 Hz, 2H), 1.68 (p, J = 7.5 Hz, 2H), 1.43 (p, J = 7.2 Hz, 2H), 1.31 – 1.24 (m, 12H), 0.87 (t, J = 7.0 Hz, 3H).
^{13}C NMR (126 MHz, CDCl$_3$) δ 145.5, 132.3, 126.7, 119.1, 108.0, 32.0, 29.6(2C), 29.4, 29.2, 29.0, 28.7, 22.8, 14.2. The spectral data are consistent with those reported in the literature.[77]

4-(cyclohexylthio)benzonitrile (87)

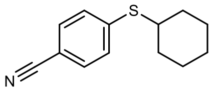

The compound was prepared according to **General Procedure F** with 4-bromobenzonitrile (36.4 mg, 0.2 mmol, 1.0 equiv.), cyclohexanethiol (36.7 μL, 0.3 mmol, 1.5 equiv.). The product was purified as a yellow oil by flash column chromatography on silica gel using hexane/EtOAc as eluent. Yield: 65% (28.3 mg).
^{1}H NMR (500 MHz, CDCl$_3$) δ 7.54 (d, J = 8.6 Hz, 2H), 7.36 (d, J = 8.6 Hz, 2H), 3.31 (tt, J = 10.2, 3.6 Hz, 1H), 2.09 – 2.01 (m, 2H), 1.85 – 1.79 (m, 2H), 1.70 – 1.65 (m, 1H), 1.50 – 1.29 (m, 5H).
^{13}C NMR (126 MHz, CDCl$_3$) δ 144.1, 132.3, 128.7, 119.0, 108.5, 45.0, 33.1, 26.0, 25.7. Data in accordance with the literature. (Org.Lett.2016, 18, 876–879---3f). The spectral data are consistent with those reported in the literature.[78]

methyl(4-(phenylthio)phenyl)sulfane (88)

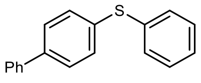

The compound was prepared according to **General Procedure F** with 4-bromobiphenyl (46.6 mg, 0.2 mmol, 1.0 equiv.), benzenethiol (31 μL, 0.3mmol, 1.5 equiv.). The product was purified as a white crystal by flash column chromatography on silica gel using hexane/EtOAc as eluent. Yield: 78% (41.1 mg).
^{1}H NMR (500 MHz, CDCl$_3$) δ 7.56 (dq, J = 8.4, 1.6 Hz, 2H), 7.54 – 7.49 (m, 2H), 7.45 – 7.36 (m, 6H), 7.36 – 7.27 (m, 3H), 7.27 – 7.22 (m, 1H).

^{13}C NMR (126 MHz, $CDCl_3$) δ 140.4, 140.1, 135.8, 135.0, 131.4, 131.3, 129.4, 129.0, 128.0, 127.6, 127.3, 127.1. The spectral data are consistent with those reported in the literature.[79]

[1,1'-biphenyl]-4-yl(phenyl)sulfane (89)

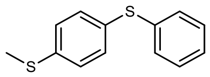

The compound was prepared according to **General Procedure F** with 4-bromothioanisole (40.6 mg, 0.2 mmol, 1.0 equiv.), benzenethiol (31 µL, 0.3 mmol, 1.5 equiv.). The product was purified as a yellow oil by flash column chromatography on silica gel using hexane/EtOAc as eluent. Yield: 77% (35.8 mg).

^{1}H NMR (500 MHz, $CDCl_3$) δ 7.35 (d, J = 1.8 Hz, 1H), 7.34 – 7.29 (m, 5H), 7.27 – 7.22 (m, 3H), 2.52 (s, 3H).

^{13}C NMR (126 MHz, $CDCl_3$) δ 138.3, 136.6, 132.5, 131.5, 130.3, 129.3, 127.3, 126.9, 15.9. The spectral data are consistent with those reported in the literature.[79]

1-(4-(phenylthio)phenyl)ethan-1-one (90)

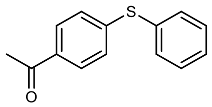

The compound was prepared according to **General Procedure F** with 1-(4-bromophenyl)ethan-1-one (39.8 mg, 0.2 mmol, 1.0 equiv.), benzenethiol (31 µL, 0.3 mmol, 1.5 equiv.). The product was purified as a pale-yellow oil by flash column chromatography on silica gel using hexane/EtOAc as eluent. Yield: 55% (25.1 mg).

^{1}H NMR (500 MHz, $CDCl_3$) δ 7.82 (d, J = 8.3 Hz, 2H), 7.53 – 7.45 (m, 2H), 7.44 – 7.31 (m, 3H), 7.21 (d, J = 8.2 Hz, 2H), 2.55 (s, 3H).

^{13}C NMR (126 MHz, $CDCl_3$) δ 197.3, 145.1, 134.6, 134.0, 132.2, 129.8, 129.0, 128.9, 127.6, 26.6. The spectral data are consistent with those reported in the literature.[79]

4-(phenylthio)benzonitrile (91)

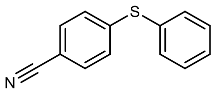

The compound was prepared according to **General Procedure F** with 4-bromobenzonitrile (36.4 mg, 0.2 mmol, 1.0 equiv.), benzenethiol (31 µL, 0.3 mmol, 1.5 equiv.). The product was purified as a colorless oil by flash column chromatography on silica gel using hexane/EtOAc as eluent. Yield: 73% (30.9 mg).

^{1}H NMR (500 MHz, $CDCl_3$) δ 7.56 – 7.50 (m, 2H), 7.47 (d, J = 8.4 Hz, 2H), 7.43 (h, J = 2.4, 3H), 7.16 (d, J = 8.4 Hz, 2H).

^{13}C NMR (126 MHz, $CDCl_3$) δ 145.8, 134.6, 132.5, 130.9, 130.0, 129.5, 127.4, 118.9, 108.8. The spectral data are consistent with those reported in the literature.[79]

4,4,5,5-tetramethyl-2-(4-(phenylthio)phenyl)-1,3,2-dioxaborolane (92)

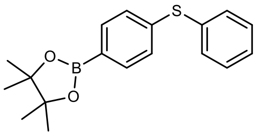

The compound was prepared according to **General Procedure F** with 2-(4-bromophenyl)-4,4,5,5-tetramethyl-1,3,2-dioxaborolane (56.6 mg, 0.2 mmol, 1.0 equiv), benzenethiol (31 µL, 0.3 mmol, 1.5 equiv.). The product was purified as a white solid by flash column chromatography on silica gel using hexane/EtOAc as eluent. Yield: 89% (55.5 mg).

^{1}H NMR (500 MHz, $CDCl_3$) δ 7.69 (d, J = 8.2 Hz, 2H), 7.42 – 7.35 (m, 2H), 7.30 (tt, J = 6.3, 1.2 Hz, 2H), 7.28 – 7.23 (m, 3H), 1.31 (s, 12H).

^{13}C NMR (126 MHz, $CDCl_3$) δ 140.5, 135.5, 134.7, 132.2, 129.4, 129.1, 127.7, 84.0, 25.0. The spectral data are consistent with those reported in the literature.[80]

naphthalen-2-yl(phenyl)sulfane (93)

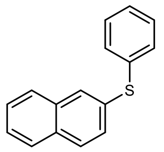

The compound was prepared according to **General Procedure F** with 2-bromonaphthalene (41.5 mg, 0.2 mmol, 1.0 equiv.), benzenethiol (31 µL, 0.3 mmol, 1.5 equiv.). The product was purified as a yellow solid by flash column chromatography on silica gel using hexane/EtOAc as eluent. Yield: 80% (37.8 mg).

^{1}H NMR (500 MHz, CDCl$_3$) δ 7.89 (d, J = 1.9 Hz, 1H), 7.87 – 7.83 (m, 1H), 7.81 (d, J = 8.6 Hz, 1H), 7.80 – 7.75 (m, 1H), 7.55 – 7.49 (m, 2H), 7.49 – 7.40 (m, 3H), 7.36 (dd, J = 14.8, 1.4 Hz, 2H), 7.33 – 7.28 (m, 1H).

^{13}C NMR (126 MHz, CDCl$_3$) δ 136.0, 133.9, 133.1, 132.4, 131.1, 130.0, 129.4, 129.0, 128.9, 127.9, 127.6, 127.2, 126.7, 126.3. The spectral data are consistent with those reported in the literature.[81]

3-(phenylthio)quinoline (94)

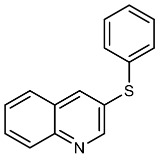

The compound was prepared according to **General Procedure F** with 3-bromoquinoline (41.6 mg, 0.2 mmol, 1.0 equiv.), benzenethiol (31 µL, 0.3 mmol, 1.5 equiv.). The product was purified as a yellow oil by flash column chromatography on silica gel using hexane/EtOAc as eluent. Yield: 77% (36.5 mg).

^{1}H NMR (500 MHz, CDCl$_3$) δ 8.81 (d, J = 2.2 Hz, 1H), 8.13 – 8.06 (m, 2H), 7.70 (td, J = 8.4, 2.1 Hz, 2H), 7.55 (t, J = 7.5 Hz, 1H), 7.45 – 7.38 (m, 2H), 7.37 – 7.28 (m, 3H).

^{13}C NMR (126 MHz, CDCl$_3$) δ 152.0, 146.5, 137.4, 134.3, 131.6, 130.3, 129.8, 129.7, 129.3, 128.4, 128.0, 127.5, 127.4. The spectral data are consistent with those reported in the literature.[82]

4-((3-methoxyphenyl)thio)benzonitrile (95)

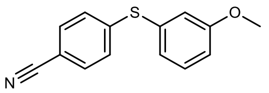

The compound was prepared according to **General Procedure F** 4-bromobenzonitrile (36.4 mg, 0.2 mmol, 1.0 equiv.), 3-methoxybenzenethiol (37.2 µL, 0.3 mmol, 1.5 equiv.). The product was purified as a pale-yellow solid by flash column chromatography on silica gel using hexane/EtOAc as eluent. Yield: 89% (43.0 mg).

^{1}H NMR (400 MHz, CDCl$_3$) δ 7.53 – 7.48 (m, 2H), 7.36 (t, J = 8.0 Hz, 1H), 7.24 – 7.19 (m, 2H), 7.11 (ddd, J = 7.6, 1.7, 1.0 Hz, 1H), 7.06 (dd, J = 2.6, 1.7, 1H), 6.98 (ddd, J = 8.4, 2.6, 0.9 Hz, 1H), 3.83 (s, 3H).

^{13}C NMR (101 MHz, CDCl$_3$) δ 160.5, 145.6, 132.5, 132.0, 130.8, 127.6, 126.6, 119.5, 118.9, 115.4, 108.9, 55.5. The spectral data are consistent with those reported in the literature.[81]

4-phenyl-2-(phenylthio)thiazole (96)

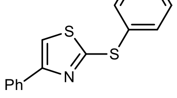

The compound was prepared according to **General Procedure F** with 2-bromo-4-phenylthiazole (48.1 mg, 0.2 mmol, 1.0 equiv.), benzenethiol (31 µL, 0.3 mmol, 1.5 equiv.). The product was purified as a brown solid by flash column chromatography on silica gel using hexane/EtOAc as eluent. Yield: 87% (47.0 mg).

^{1}H NMR (500 MHz, CDCl$_3$) δ 7.88 (dd, J = 8.4, 1.3 Hz, 2H), 7.74 – 7.60 (m, 2H), 7.47 – 7.38 (m, 5H), 7.34 (d, J = 5.7 Hz, 2H).

^{13}C NMR (126 MHz, CDCl$_3$) δ 166.3, 156.3, 134.1, 131.8, 129.9, 129.8, 128.8, 128.4, 126.4, 114.0. The spectral data are consistent with those reported in the literature.[83]

4-tosyl-1,1'-biphenyl (97)

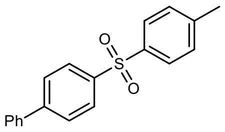

The compound was prepared according to **General Procedure G** with 4-bromobiphenyl (46.6 mg, 0.2 mmol, 1.0 equiv.), sodium 4-methylbenzenesulfinate (64.2 mg, 0.36 mmol, 1.8 equiv.). The product was purified as a white solid by flash column chromatography on silica gel using hexane/EtOAc as eluent. Yield: 83% (51.2 mg).

^{1}H NMR (400 MHz, CDCl$_3$) δ 7.99 (d, *J* = 8.4 Hz, 2H), 7.87 (d, *J* = 8.3 Hz, 2H), 7.69 (d, *J* = 8.5 Hz, 2H), 7.59 – 7.53 (m, 2H), 7.48 – 7.43 (m, 2H), 7.43 – 7.36 (m, 1H), 7.31 (d, *J* = 8.0 Hz, 2H), 2.40 (s, 3H).

^{13}C NMR (101 MHz, CDCl$_3$) δ 146.1, 144.3, 140.6, 139.3, 138.9, 130.1, 129.2, 128.7, 128.2, 128.0, 127.8, 127.5, 21.7. The spectral data are consistent with those reported in the literature.[84]

1-methoxy-4-tosylbenzene (98)

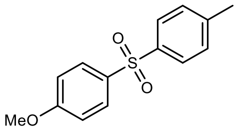

The compound was prepared according to **General Procedure G** with 1-bromo-4-methoxybenzene (25 µL, 0.2 mmol, 1.0 equiv.), sodium 4-methylbenzenesulfinate (64.2 mg, 0.36 mmol, 1.8 equiv.). The product was purified as a white solid by flash column chromatography on silica gel using hexane/EtOAc as eluent. Yield: 51% (26.7 mg).

^{1}H NMR (500 MHz, CDCl$_3$) δ 7.85 (d, *J* = 9.0 Hz, 2H), 7.79 (d, *J* = 8.4 Hz, 2H), 7.27 (d, *J* = 7.8 Hz, 2H), 6.94 (d, *J* = 9.0 Hz, 2H), 3.83 (s, 3H), 2.38 (s, 3H)

^{13}C NMR (126 MHz, CDCl$_3$) δ 163.3, 143.9, 139.5, 133.7, 130.0, 129.8, 127.5, 114.6, 55.7, 21.7. The spectral data are consistent with those reported in the literature.[84]

methyl(4-tosylphenyl)sulfane (99)

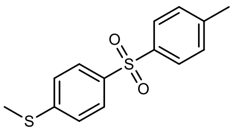

The compound was prepared according to **General Procedure G** with (4-bromophenyl)(methyl)sulfan (40.6 mg, 0.2 mmol, 1.0 equiv.), sodium 4-methylbenzenesulfinate (64.2 mg, 0.36 mmol, 1.8 equiv.). The product was purified as a white solid by flash column chromatography on silica gel using hexane/EtOAc as eluent. Yield: 82% (46.0 mg).

^{1}H NMR (400 MHz, CDCl$_3$) δ 7.83 – 7.76 (m, 4H), 7.27 (dd, *J* = 8.5, 7.0 Hz, 4H), 2.48 (s, 3H), 2.38 (s, 3H).

^{13}C NMR (101 MHz, CDCl$_3$) δ 146.5, 144.1, 139.0, 137.7, 130.0, 127.9, 127.6, 125.6, 21.7, 14.9. The spectral data are consistent with those reported in the literature.[85]

trimethyl(4-tosylphenyl)silane (100)

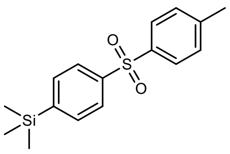

The compound was prepared according to **General Procedure G** with (4-bromophenyl)trimethylsilane (45.8 mg, 0.2 mmol, 1.0 equiv.), sodium 4-methylbenzenesulfinate (64.2 mg, 0.36 mmol, 1.8 equiv.). The product was purified as a pale-yellow solid by flash column chromatography on silica gel using hexane/EtOAc as eluent. Yield: 87% (53.1 mg).

^{1}H NMR (400 MHz, CDCl$_3$) δ 7.87 (d, *J* = 8.4 Hz, 2H), 7.83 (d, *J* = 8.4 Hz, 2H), 7.62 (d, *J* = 8.4 Hz, 2H), 7.29 (d, *J* = 7.9 Hz, 2H), 2.38 (s, 3H), 0.26 (s, 9H).

^{13}C NMR (101 MHz, CDCl$_3$) δ 147.6, 144.2, 142.2, 138.8, 134.2, 130.0, 127.8, 126.4, 21.7, 1.3. The spectral data are consistent with those reported in the literature.[85]

4-tosylbenzonitrile (101)

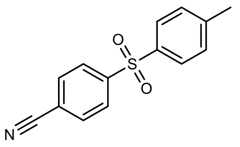

The compound was prepared according to **General Procedure G** with 4-bromobenzonitrile (36.4 mg, 0.2 mmol, 1.0 equiv.), sodium 4-methylbenzenesulfinate (64.2 mg, 0.36 mmol, 1.8 equiv.). The product was purified as a white solid by flash column chromatography on silica gel using hexane/EtOAc as eluent. Yield: 96% (49.4 mg).

^{1}H NMR (400 MHz, CDCl$_3$) δ 8.05 (d, *J* = 8.5 Hz, 2H), 7.84 (d, *J* = 8.4 Hz, 2H), 7.80 (d, *J* = 8.7 Hz, 2H), 7.35 (d, *J* = 8.2 Hz, 2H), 2.43 (s, 3H).

^{13}C NMR (101 MHz, CDCl$_3$) δ 146.3, 145.4, 137.2, 133.1, 130.4, 128.2, 128.1, 117.3, 116.8, 21.7. The spectral data are consistent with those reported in the literature.[10]

1-methyl-4-((4-(trifluoromethyl)phenyl)sulfonyl)benzene (102)

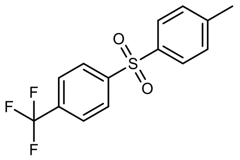

The compound was prepared according to **General Procedure G** with 1-bromo-4-(trifluoromethyl)benzene (45 mg, 0.2 mmol, 1.0 equiv.), sodium 4-methylbenzenesulfinate (64.2 mg, 0.36 mmol, 1.8 equiv.). The product was purified as a white solid by flash column chromatography on silica gel using hexane/EtOAc as eluent. Yield: 71% (42.7 mg).

^{1}H NMR (400 MHz, CDCl$_3$) δ 8.05 (d, *J* = 8.2 Hz, 2H), 7.88 – 7.80 (m, 2H), 7.75 (d, *J* = 8.3 Hz, 2H), 7.32 (d, *J* = 8.1 Hz, 2H), 2.40 (s, 3H).

^{13}C NMR (101 MHz, CDCl$_3$) δ 145.7, 145.1, 137.7, 134.9, 134.6 (q, *J* = 31.0 Hz), 130.3, 128.2, 128.1, 126.6 (q, *J* = 4.0 Hz), 123.2 (q, *J* = 273.7 Hz), 21.7.

^{19}F NMR (471 MHz, CDCl$_3$) δ -63.18. The spectral data are consistent with those reported in the literature.[10]

1-methyl-4-((4-(methylsulfonyl)phenyl)sulfonyl)benzene (103)

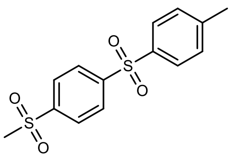

The compound was prepared according to **General Procedure G** with 1-bromo-4-(methylsulfonyl)benzene (47.0 mg, 0.2 mmol, 1.0 equiv.), sodium 4-methylbenzenesulfinate (64.2 mg, 0.36 mmol, 1.8 equiv.). The product was purified as a white solid by flash column chromatography on silica gel using hexane/EtOAc as eluent. Yield: 78% (48.5 mg).

^{1}H NMR (400 MHz, CDCl$_3$) δ 8.11 (d, *J* = 8.8 Hz, 2H), 8.05 (d, *J* =8.7 Hz, 2H), 7.83 (d, *J* = 8.4 Hz, 2H), 7.33 (d, *J* = 7.9 Hz, 2H), 3.05 (s, 3H), 2.40 (s, 3H).

^{13}C NMR (101 MHz, CDCl$_3$) δ 147.2, 145.4, 144.6, 137.2, 130.4, 128.6, 128.6, 128.2, 44.3, 21.8.

HRMS (ESI): calculated for $[M+Na]^+$ $C_{14}H_{14}O_4S_2Na^+$ 333.0226; found 333.0216.

1-(4-tosylphenyl)ethan-1-one (104)

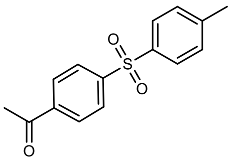

The compound was prepared according to **General Procedure G** with 1-(4-bromophenyl)ethan-1-one (39.8 mg, 0.2 mmol, 1.0 equiv.), sodium 4-methylbenzenesulfinate (64.2 mg, 0.36 mmol, 1.8 equiv.). The product was purified as a white solid by flash column chromatography on silica gel using hexane/EtOAc as eluent. Yield:60% (32.9 mg).

^{1}H NMR (400 MHz, CDCl$_3$) δ 8.06 – 7.98 (m, 4H), 7.85 – 7.75 (m, 2H), 7.31 (d, *J* = 8.1 Hz, 2H), 2.60 (s, 3H), 2.39 (s, 3H).

^{13}C NMR (101 MHz, CDCl$_3$) δ 196.9, 145.9, 144.9, 140.3, 137.9, 130.2, 129.1, 128.0, 127.9, 77.5, 77.2, 76.8, 27.0, 21.7. The spectral data are consistent with those reported in the literature.[10]

5-tosylisobenzofuran-1(3*H*)-one (105)

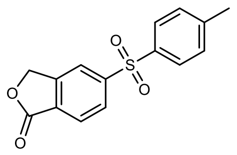

The compound was prepared according to **General Procedure G** with 5-bromoisobenzofuran-1(3*H*)-one (42.6 mg, 0.2 mmol, 1.0 equiv.), sodium 4-methylbenzenesulfinate (64.2 mg, 0.36 mmol, 1.8 equiv.). The product was purified as a pale-yellow solid by flash column chromatography on silica gel using hexane/EtOAc as eluent. Yield: 65% (37.5 mg).

^{1}H NMR (400 MHz, CDCl$_3$) δ 8.10 (s, 1H), 8.06 (dd, *J* = 8.0, 1.4 Hz, 1H), 8.00 (d, *J* = 8.0 Hz, 1H), 7.87 – 7.81 (m, 2H), 7.33 (d, *J* = 8.1 Hz, 2H), 5.37 (s, 2H), 2.40 (s, 3H).

^{13}C NMR (101 MHz, CDCl$_3$) δ 169.3, 147.8, 147.2, 145.4, 137.3, 130.4, 129.7, 128.5, 128.1, 126.9, 121.8, 69.7, 21.8. Data in accordance with the literature.

HRMS (ESI): calculated for $[M+Na]^+$ $C_{15}H_{12}O_4SNa^+$ 311.0349; found 311.0346.

2-methyl-5-tosylisoindoline-1,3-dione (106)

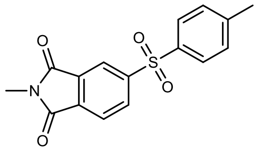

The compound was prepared according to **General Procedure G** with 5-bromo-2-methylisoindoline-1,3-dione (48.1 mg, 0.2 mmol, 1.0 equiv.), sodium 4-methylbenzenesulfinate (64.2 mg, 0.36 mmol, 1.8 equiv.). The product was purified as a pale-yellow solid by flash column chromatography on silica gel using hexane/EtOAc as eluent. Yield: 58% (36.6 mg).

^{1}H NMR (400 MHz, CDCl$_3$) δ 8.38 – 8.25 (m, 2H), 7.97 (d, *J* = 7.8 Hz, 1H), 7.90 – 7.82 (m, 2H), 7.36 (d, *J* = 8.1 Hz, 2H), 3.20 (s, 3H), 2.43 (s, 3H).

^{13}C NMR (101 MHz, CDCl$_3$) δ 166.8, 166.7, 148.1, 145.5, 137.1, 135.6, 133.2, 130.5, 128.2, 124.2, 122.5, 77.5, 77.2, 76.8, 24.5, 21.8.

HRMS (ESI): calculated for $[M+Na]^+$ $C_{16}H_{13}NO_4SNa^+$ 338.0457; found 338.0449.

5-tosylpyrimidine (107)

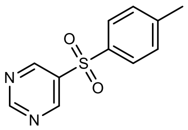

The compound was prepared according to **General Procedure G** with 5-bromopyrimidine (31.8 mg, 0.2 mmol, 1.0 equiv.), sodium 4-methylbenzenesulfinate (64.2 mg, 0.36 mmol, 1.8 equiv.). The product was purified as a white needle solid by flash column chromatography on silica gel using hexane/EtOAc as eluent. Yield: 53% (24.9 mg).

^{1}H NMR (500 MHz, CDCl$_3$) δ 9.34 (s, 1H), 9.18 (s, 2H), 7.87 (d, *J* = 8.4 Hz, 2H), 7.37 (d, *J* = 7.9 Hz, 2H), 2.43 (s, 3H).

^{13}C NMR (126 MHz, CDCl$_3$) δ 161.4, 155.9, 146.0, 137.4, 137.1, 130.7, 128.1, 77.4, 77.2, 76.9, 21.8. The spectral data are consistent with those reported in the literature.[85]

3-tosylquinoline (108)

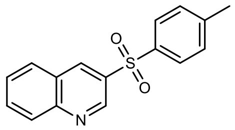

The compound was prepared according to **General Procedure G** with 3-bromoquinoline (41.6 mg, 0.2 mmol, 1.0 equiv.), sodium 4-methylbenzenesulfinate (64.2 mg, 0.36 mmol, 1.8 equiv.). The product was purified as a white solid by flash column chromatography on silica gel using hexane/EtOAc as eluent. Yield: 92% (52.2 mg).

^{1}H NMR (400 MHz, CDCl$_3$) δ 9.25 (d, *J* = 2.4 Hz, 1H), 8.80 (d, *J* = 2.3 Hz, 1H), 8.15 (d, *J* = 8.5 Hz, 1H), 7.99 – 7.80 (m, 4H), 7.66 (t, *J* = 7.5 Hz, 1H), 7.32 (d, *J* = 8.0 Hz, 2H), 2.39 (s, 3H).

^{13}C NMR (101 MHz, CDCl$_3$) δ 149.2, 147.1, 145.0, 138.1, 136.9, 135.2, 132.8, 130.3, 129.6, 129.3, 128.5, 128.0, 126.5, 77.5, 77.2, 76.8, 21.7. The spectral data are consistent with those reported in the literature.[10]

3-tosylthiophene (109)

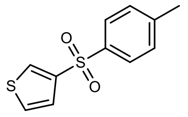

The compound was prepared according to **General Procedure G** with 2-bromothiophene (19.5 µL, 0.2 mmol, 1.0 equiv.), sodium 4-methylbenzenesulfinate (64.2 mg, 0.36 mmol, 1.8 equiv.). The product was purified as an orange solid by flash column chromatography on silica gel using hexane/EtOAc as eluent. Yield: 90% (42.9 mg).

^{1}H NMR (400 MHz, CDCl$_3$) δ 8.02 (dd, *J* = 3.1, 1.3 Hz, 1H), 7.82 – 7.76 (m, 2H), 7.32 (dd, *J* = 5.2, 3.1 Hz, 1H), 7.26 (dd, *J* = 7.0, 1.8 Hz, 3H), 2.36 (s, 3H).

^{13}C NMR (101 MHz, CDCl$_3$) δ 144.4, 142.5, 138.8, 131.3, 130.1, 128.4, 127.6, 125.9, 77.5, 77.2, 76.8, 21.7. The spectral data are consistent with those reported in the literature.[10]

4-((4-methoxyphenyl)sulfonyl)-1,1'-biphenyl (110)

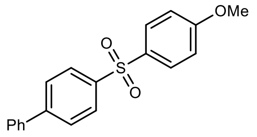

The compound was prepared according to **General Procedure G** with 4-bromobiphenyl (46.6 mg, 0.2 mmol, 1.0 equiv.), sodium 4-methoxybenzenesulfinate (69.9 mg, 0.36 mmol, 1.8 equiv.). The product was purified as a white solid by flash column chromatography on silica gel using hexane/EtOAc as eluent. Yield: 85% (55.2 mg).

^{1}H NMR (400 MHz, CDCl$_3$) δ 8.03 – 7.97 (m, 4H), 7.75 – 7.68 (m, 2H), 7.59 – 7.54 (m, 2H), 7.50 – 7.44 (m, 2H), 7.43 – 7.39 (m, 1H), 7.22 – 7.16 (m, 2H).

^{13}C NMR (101 MHz, CDCl$_3$) δ 166.8, 164.3, 146.4, 140.0, 139.2, 137.9, 137.9, 130.6, 130.5, 129.2, 128.8, 128.2, 128.1, 127.5, 116.9, 116.6, 77.5, 77.2, 76.8.

HRMS (ESI): calculated for $[M+Na]^+$ $C_{19}H_{16}O_3SNa^+$ 347.0712; found 347.0706.

4-((4-fluorophenyl)sulfonyl)-1,1'-biphenyl (111)

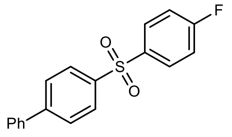

The compound was prepared according to **General Procedure G** with 4-bromobiphenyl (46.6 mg, 0.2 mmol, 1.0 equiv.), sodium 4-fluorobenzenesulfinate (65.6 mg, 0.36 mmol, 1.8 equiv.). The product was purified as a white solid by flash column chromatography on silica gel using hexane/EtOAc as eluent. Yield: 76% (47.5 mg).

^{1}H NMR (500 MHz, CDCl$_3$) δ 7.97 (d, *J* = 8.2 Hz, 2H), 7.92 (d, *J* = 8.9 Hz, 2H), 7.68 (d, *J* = 8.5 Hz, 2H), 7.55 (d, *J* = 7.3 Hz, 2H), 7.46 (t, *J* = 7.9 Hz, 2H), 7.40 (t, *J* = 6.6 Hz, 1H), 6.98 (d, *J* = 9.0 Hz, 2H), 3.85 (s, 3H).

^{13}C NMR (126 MHz, CDCl$_3$) δ 165.5 (d, *J* = 257.6 Hz), 146.3, 141.0, 139.4, 137.9 (d, *J* = 3.0 Hz), 130.6 (d, *J* = 9.0 Hz), 129.2, 128.8, 128.2, 128.1, 127.5, 116 (d, *J* = 22.2 Hz).

^{19}F NMR (471 MHz, CDCl$_3$) δ -104.2. The spectral data are consistent with those reported in the literature.[86]

4-((4-(trifluoromethyl)phenyl)sulfonyl)-1,1'-bipheny (112)

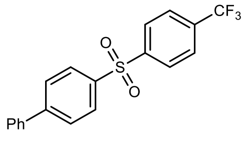

The compound was prepared according to **General Procedure G** with 4-bromobiphenyl (46.6 mg, 0.2 mmol, 1.0 equiv.), sodium 4-(trifluoromethyl)benzenesulfinate (83.6 mg, 0.36 mmol, 1.8 equiv.). The product was purified as a white solid by flash column chromatography on silica gel using hexane/EtOAc as eluent. Yield: 61% (44.2 mg).

^{1}H NMR (500 MHz, CDCl$_3$) δ 8.14 (d, *J* = 8.2 Hz, 2H), 8.05 (d, *J* = 8.5 Hz, 2H), 7.81 (d, *J* = 8.3 Hz, 2H), 7.76 (d, *J* = 8.5 Hz, 2H), 7.62 – 7.57 (m, 2H), 7.52 – 7.47 (m, 2H), 7.47 – 7.41 (m, 1H).

^{13}C NMR (126 MHz, CDCl$_3$) δ 147.0, 145.5, 139.1, 139.0, 135.0 (q, *J* = 26.3 Hz), 129.2, 128.9, 128.6, 128.3 (2C), 127.5, 126.6 (q, *J* = 3.0 Hz), 123.3 (q, *J* = 219.2 Hz).

^{19}F NMR (471 MHz, CDCl$_3$) δ -63.2.

HRMS (ESI): calculated for $[M+Na]^+$ $C_{19}H_{13}F_3O_2SNa^+$ 385.0481; found 385.0469.

N-(4-([1,1'-biphenyl]-4-ylsulfonyl)phenyl)acetamide (113)

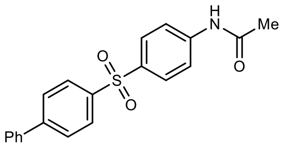

The compound was prepared according to **General Procedure G** with 4-bromobiphenyl (46.6 mg, 0.2 mmol, 1.0 equiv.), sodium 4-acetamidobenzenesulfinate (79.7 mg, 0.36 mmol, 1.8 equiv.). The product was purified as a pale brown solid by flash column chromatography on silica gel using hexane/EtOAc as eluent. Yield: 74% (52.0 mg).

^{1}H NMR (500 MHz, DMSO) δ 10.38 (s, 1H), 7.97 (d, *J* = 8.3 Hz, 2H), 7.92 (d, *J* = 8.7 Hz, 2H), 7.87 (d, *J* = 8.4 Hz, 2H), 7.79 (d, *J* = 8.9 Hz, 2H), 7.69 (dd, *J* = 7.6 Hz, 1.7, 2H), 7.48 (t, *J* = 7.5 Hz, 2H), 7.45 – 7.39 (m, 1H), 2.06 (s, 3H).

^{13}C NMR (126 MHz, DMSO) δ 169.6, 145.4, 144.4, 140.9, 138.8, 134.9, 129.6, 129.2, 129.2, 128.4, 128.2, 127.6, 119.5, 24.6.

HRMS (ESI): calculated for $[M+Na]^+$ $C_{20}H_{17}NO_3SNa^+$ 374.0821; found 374.0812.

3-([1,1'-biphenyl]-4-ylsulfonyl)pyridine (114)

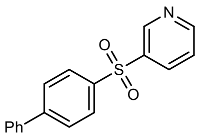

The compound was prepared according to **General Procedure G** with 4-bromobiphenyl (46.6 mg, 0.2 mmol, 1.0 equiv.), sodium pyridine-3-sulfinate (59.5 mg, 0.36 mmol, 1.8 equiv.). The product was purified as a pink crystal by flash column chromatography on silica gel using hexane/EtOAc as eluent. Yield: 72% (43.6 mg).

^{1}H NMR (400 MHz, CDCl$_3$) δ 9.19 (s, 1H), 8.80 (dd, *J* = 5.0 Hz, 1.6, 1H), 8.27 (dt, *J* = 8.1, 2.0 Hz, 1H), 8.05 – 8.00 (m, 2H), 7.76 – 7.71 (m, 2H), 7.59 – 7.54 (m, 2H), 7.50 – 7.39 (m, 4H).

^{13}C NMR (101 MHz, CDCl$_3$) δ 153.6, 148.6, 147.0, 139.2, 139.0, 138.6, 135.5, 129.2, 128.9, 128.5, 128.3, 127.5, 124.1. Data in accordance with the literature.

HRMS (ESI): calculated for $[M+Na]^+$ $C_{17}H_{13}NO_2SK^+$ 318.0559; found 318.0559.

4-(cyclopropylsulfonyl)-1,1'-biphenyl (115)

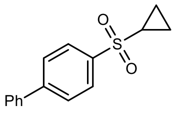

The compound was prepared according to **General Procedure G** with 4-bromobiphenyl (46.6 mg, 0.2 mmol, 1.0 equiv.), sodium cyclopropanesulfinate (46.2 mg, 0.36 mmol, 1.8 equiv.). The product was purified as a pale brown solid by flash column chromatography on silica gel using hexane/EtOAc as eluent. Yield: 90% (46.6 mg).

^{1}H NMR (500 MHz, CDCl$_3$) δ 7.99 – 7.94 (m, 2H), 7.76 (d, *J* = 8.1 Hz, 2H), 7.64 – 7.59 (m, 2H), 7.49 (t, *J* = 7.6 Hz, 2H), 7.45 – 7.40 (m, 1H), 2.51 (tt, *J* = 8.0, 4.9 Hz, 1H), 1.38 (dt, *J* = 6.6, 3.4 Hz, 2H), 1.06 (tt, *J* = 6.9, 2.9 Hz, 2H).

^{13}C NMR (126 MHz, CDCl$_3$) δ 146.4, 139.4, 129.2, 128.7, 128.2, 128.0, 127.5, 33.2, 6.1.

HRMS (ESI): calculated for $[M+H]^+$ $C_{15}H_{15}O_2S^+$ 259.0787; found 259.0799.

1-(4-(methylsulfonyl)phenyl)ethan-1-one (116)

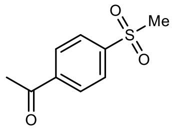

The compound was prepared according to the modified **General Procedure G** with 1-(4-bromophenyl)ethan-1-one (39.8 mg, 0.2 mmol, 1.0 equiv.), sodium methanesulfinate (36.8 mg, 0.36 mmol, 1.8 equiv.), 4MeODPATPN (10.4 mg, 0.01 mmol, 0.05 equiv.). The product was purified as a pale pink solid by flash column chromatography on silica gel using hexane/EtOAc as eluent. Yield: 81% (32.1 mg).

^{1}H NMR (500 MHz, CDCl$_3$) δ 8.13 – 8.09 (m, 2H), 8.06 – 8.00 (m, 2H), 3.07 (s, 3H), 2.65 (s, 3H).

^{13}C NMR (126 MHz, CDCl$_3$) δ 196.8, 144.3, 141.0, 129.2, 127.9, 44.4, 27.0. The spectral data are consistent with those reported in the literature.[87]

[1,1'-biphenyl]-4-yldiphenylphosphine oxide (117)

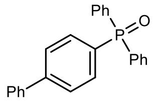

The compound was prepared according to **General Procedure H** with 4-bromobiphenyl (46.6 mg, 0.2 mmol, 1.0 equiv.), diphenylphosphine oxide (60.6 mg, 0.3 mmol, 1.5 equiv.). The product was purified as a white solid by flash column chromatography on silica gel using hexane/EtOAc/MeOH as eluent. Yield: 63% (44.7 mg).

^{1}H NMR (500 MHz, CDCl$_3$) δ 7.76 – 7.66 (m, 8H), 7.59 (dd, J = 8.3 Hz, 1.3, 2H), 7.57 – 7.52 (m, 2H), 7.50 – 7.42 (m, 6H), 7.40 – 7.35 (m, 1H).

^{13}C NMR (126 MHz, CDCl$_3$) δ 144.9 (d, J_{C-P} = 2.5 Hz), 140.0, 132.7 (d, J_{C-P} = 104.6 Hz), 132.7 (d, J_{C-P} = 11.3 Hz), 132.2 (d, J_{C-P} = 10.1 Hz), 132.1(d, J_{C-P} = 2.5 Hz), 131.0 (d, J_{C-P} = 105.8 Hz), 129.1, 128.7 (d, J_{C-P} = 12.6 Hz), 128.3, 127.4, 127.2.

^{31}P NMR (202 MHz, CDCl$_3$) δ29.5. The spectral data are consistent with those reported in the literature.[88]

diphenyl(4-(trifluoromethyl)phenyl)phosphine oxide (118)

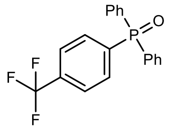

The compound was prepared according to **General Procedure H** with 1-bromo-4-(trifluoromethyl)benzene (45.0 mg, 0.2 mmol, 1.0 equiv.), diphenylphosphine oxide (60.6 mg, 0.3 mmol, 1.5 equiv.). The product was purified as a white solid by flash column chromatography on silica gel using hexane/EtOAc/MeOH as eluent. Yield: 77% (53.3 mg).

^{1}H NMR (500 MHz, CDCl$_3$) δ 7.81 (dd, J = 11.5, 8.0 Hz, 2H), 7.74 – 7.69 (m, 2H), 7.69 – 7.61 (m, 4H), 7.57 (td, J = 7.3, 1.5 Hz, 2H), 7.51 – 7.44 (m, 4H).

^{13}C NMR (126 MHz, CDCl$_3$) δ 137.2 (d, J_{C-P} = 100.8 Hz), 133.8 (qd, J_{C-F} = 32.8 Hz, J_{C-P} = 3.8 Hz), 132.7 (d, J_{C-P} = 10.1 Hz), 132.5 (d, J_{C-P} = 10.1 Hz), 132.2 (d, J_{C-P} = 2.5 Hz), 131.3 (d, J_{C-P} = 104.6 Hz), 128.9 (d, J_{C-P} = 12.6 Hz), 125.5 (dd, J_{C-P} = 11.3 Hz, J_{C-F} = 3.8 Hz), 123.7 (d, J_{C-F} = 273.4 Hz).

^{31}P NMR (202 MHz, CDCl$_3$) δ28.1.

^{19}F NMR (471 MHz, CDCl$_3$) δ-63.2. The spectral data are consistent with those reported in the literature.[88]

methyl 4-(diphenylphosphoryl)benzoate (119)

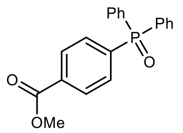

The compound was prepared according to **General Procedure H** with methyl 4-bromobenzoate (43.0 mg, 0.2 mmol, 1.0 equiv.), diphenylphosphine oxide (60.6 mg, 0.3 mmol, 1.5 equiv.). The product was purified as a white solid by flash column chromatography on silica gel using hexane/EtOAc/MeOH as eluent. Yield: 76% (51.2 mg).

^{1}H NMR (400 MHz, CDCl$_3$) δ 8.12 – 8.07 (m, 2H), 7.78 – 7.71 (m, 2H), 7.68 – 7.61 (m, 4H), 7.59 – 7.52 (m, 2H), 7.46 (m, 4H), 3.92 (s, 3H).

^{13}C NMR (101 MHz, CDCl$_3$) δ 166.3, 137.7 (d, J_{C-P} = 100.8 Hz), 133.3 (d, J_{C-P} = 2.5 Hz), 132.4 (d, J_{C-P} = 2.5 Hz), 132.3(d, J_{C-P} = 10.1 Hz), 132.1(d, J_{C-P} = 10.1 Hz), 131.4, 129.6 (d, J_{C-P} = 12.6 Hz), 128.8(d, J_{C-P} = 12.6 Hz), 52.6.

^{31}P NMR (202 MHz, CDCl$_3$) δ 28.4. The spectral data are consistent with those reported in the literature.[88]

isopropyl 2-methyl-2-(4-(4-(piperidin-1-yl)benzoyl)phenoxy)propanoate (120)

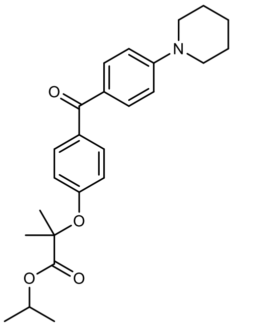

The compound was prepared according to **General Procedure A** with fenofibrate (72.2 mg, 0.2 mmol, 1.0 equiv.) and piperidine (55 μL, 0.6 mmol, 3.0 equiv.). The product was purified as a yellow solid by flash column chromatography on silica gel using hexane/EtOAc as eluent. Yield: 94% (77.1 mg).

^{1}H NMR (500 MHz, CDCl$_3$) δ 7.69 (dd, J = 17.9, 8.4 Hz, 4H), 6.92 – 6.80 (m, 4H), 5.06 (hept, J = 6.8, 6.3 Hz, 1H), 3.33 (t, J = 5.1 Hz, 4H), 1.73 – 1.56 (m, 12H), 1.18 (d, J = 6.4 Hz, 6H).

^{13}C NMR (126 MHz, CDCl$_3$) δ 194.0, 173.3, 158.7, 154.0, 132.3, 132.1, 131.5, 126.8, 117.2, 113.4, 79.3, 69.2, 48.8, 25.4, 25.3, 24.3, 21.6.

HRMS (ESI): calculated for $[M+Na]^+$ $C_{25}H_{31}NO_4Na^+$ 432.2145; found 432.2145.

4-(piperidin-1-yl)phenyl adamantane-1-carboxylate (121)

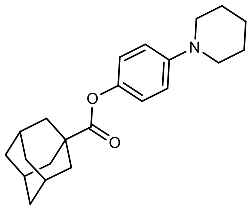

The compound was prepared according to **General Procedure A** with 4-iodophenyl-adamantane-1-carboxylate (76.4 mg, 0.2 mmol, 1.0 equiv.) and piperidine (55 μL, 0.6 mmol, 3.0 equiv.). The product was purified as a white solid by flash column chromatography on silica gel using hexane/EtOAc as eluent. Yield: 54% (36.7 mg).

^{1}H NMR (500 MHz, CDCl$_3$) δ 7.08 – 6.87 (m, 4H), 3.18 – 3.07 (m, 4H), 2.11 – 1.98 (m, 10H), 1.76 (s, 11H).

^{13}C NMR (126 MHz, CDCl$_3$) δ 176.6, 122.0, 118.1, 52.0, 41.1, 38.9, 36.6, 25.7, 24.1.

HRMS (ESI): calculated for $[M+Na]^+$ $C_{22}H_{29}NO_2Na^+$ 362.2090; found 362.2084.

((5R,5aS,8aS,8bR)-2,2,7,7-tetramethyltetrahydro-5*H*-bis([1,3]dioxolo)[4,5-b:4',5'-d]pyran-5-yl)methyl 4-(piperidin-1-yl)benzoate (122)

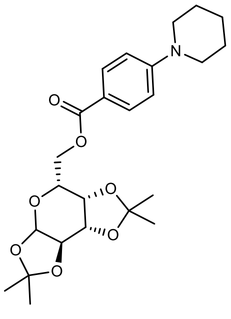

The compound was prepared according to **General Procedure A** with ((5*R*,5a*S*,8a*S*,8b*R*)-2,2,7,7-tetramethyltetrahydro-5*H*-bis([1,3]dioxolo)[4,5-b:*4*',5'-*d*]pyran-5-yl)methyl 4-bromobenzoate (88.7 mg, 0.2 mmol, 1.0 equiv.) and piperidine (55 μL, 0.6 mmol, 3.0 equiv.). The product was purified as a white solid by flash column chromatography on silica gel using hexane/EtOAc as eluent. Yield: 98% (88.0 mg).

^{1}H NMR (500 MHz, CDCl$_3$) δ 7.89 (d, J = 8.9 Hz, 2H), 6.84 (d, J = 8.6 Hz, 2H), 5.53 (d, J = 5.0 Hz, 1H), 4.62 (dd, J = 7.9, 2.4 Hz, 1H), 4.46 (dd, J = 11.4, 5.1 Hz, 1H), 4.38 – 4.28 (m, 3H), 4.14 (ddd, J = 7.1, 5.1, 1.8 Hz, 1H), 3.30 (t, J = 5.3 Hz, 4H), 1.63 (dt, J = 19.9, 5.2 Hz, 6H), 1.49 (s, 3H), 1.45 (s, 3H), 1.32 (d, J = 12.1 Hz, 6H). **^{13}C NMR (126 MHz, CDCl$_3$)** δ 166.5, 154.4, 131.5, 118.7, 113.7, 109.6, 108.8, 96.4, 71.2, 70.8, 70.6, 66.3, 63.2, 49.0, 26.1, 26.0, 25.3, 25.1, 24.5, 24.3.

HRMS (ESI): calculated for $[M+Na]^+$ $C_{24}H_{33}NO_7Na^+$ 470.2149; found 470.2140.

((5*R*,5a*S*,8a*S*,8b*R*)-2,2,7,7-tetramethyltetrahydro-5*H*-bis([1,3]dioxolo)[4,5-*b*:4',5'-*d*]pyran-5-yl)methyl 4-(piperidin-1-yl)benzoate (123)

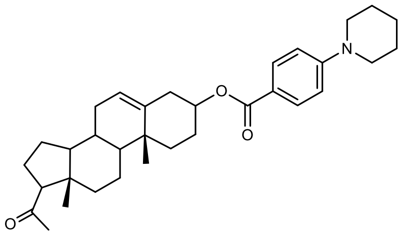

The compound was prepared according to **General Procedure A** with (10*R*,13*S*)-17-acetyl-10,13-dimethyl-2,3,4,7,8,9,10,11,12,13,14,15,16,17-tetradecahydro-1*H*-cyclopenta[*a*]phenanthren-3-yl 4-bromobenzoate (75.6 mg, 0.15 mmol, 1.0 equiv.), piperidine (41.2 µL, 0.45 mmol, 3.0 equiv.) and 3 ml of DMA. The product was purified as a white solid by flash column chromatography on silica gel using hexane/EtOAc as eluent. Yield: 88% (66.6 mg).

^{1}H NMR (400 MHz, CDCl$_3$) δ 7.95 – 7.84 (m, 2H), 6.94 – 6.76 (m, 2H), 5.40 (dt, *J* = 3.6, 1.7 Hz, 1H), 4.81 (dddd, *J* = 14.6, 10.9, 6.7, 4.4 Hz, 1H), 3.32 (t, *J* = 5.2 Hz, 4H), 2.54 (t, *J* = 8.9 Hz, 1H), 2.47 – 2.40 (m, 2H), 2.13 (s, 4H), 2.08 – 1.87 (m, 4H), 1.78 – 1.53 (m, 11H), 1.53 – 1.41 (m, 3H), 1.29 – 1.14 (m, 3H), 1.06 (s, 4H), 0.63 (s, 3H).

^{13}C NMR (101 MHz, CDCl$_3$) δ 209.8, 166.2, 154.5, 140.0, 131.3, 122.4, 119.6, 113.8, 73.8, 63.8, 57.0, 50.0, 49.1, 44.1, 38.9, 38.4, 37.2, 36.8, 32.0, 31.9, 31.7, 28.1, 25.4, 24.6, 24.4, 22.9, 21.2, 19.5, 13.4.

HRMS (ESI): calculated for $[M+Na]^+$ $C_{33}H_{45}NO_3Na^+$ 526.3292; found 526.3277.

***N*,*N*-diethyl-5-(piperidin-1-yl)nicotinamide (124)**

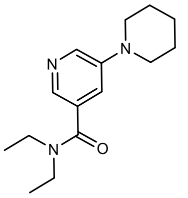

The compound was prepared according to **General Procedure A** with 5-bromo-*N*,*N*-diethylnicotinamide (51.4 mg, 0.2 mmol, 1.0 equiv.) and piperidine (41.2 µL, 0.45 mmol, 3.0 equiv.). The product was purified as a colorless oil by flash column chromatography on silica gel using DCM/MeOH as eluent. Yield: 98% (51.1 mg).

^{1}H NMR (400 MHz, CDCl$_3$) δ 8.27 – 8.22 (m, 1H), 7.98 – 7.91 (m, 1H), 7.14 – 7.09 (m, 1H), 3.54 – 3.42 (m, 2H), 3.26 – 3.19 (m, 2H), 3.19 – 3.13 (m, 4H), 1.68 – 1.60 (m, 4H), 1.59 – 1.50 (m, 2H), 1.24 – 1.14 (m, 3H), 1.12 – 1.01 (m, 3H).

^{13}C NMR (101 MHz, CDCl$_3$) δ 169.1, 147.3, 138.8, 136.0, 132.9, 120.1, 49.3, 43.4, 39.4, 25.4, 24.0, 14.4, 12.9.

HRMS (ESI): calculated for $[M+H]^+$ $C_{15}H_{24}N_3O^+$ 262.1914; found 262.1922.

(4-((2-(benzo[*d*][1,3]dioxol-5-yl)ethyl)amino)phenyl)(phenyl)methanone (125)

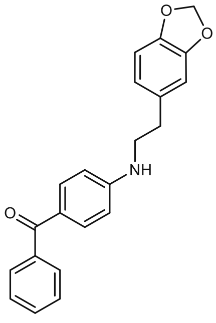

The compound was prepared according to **General Procedure A** with 4-bromobenzophenone (55.3 mg, 0.2 mmol, 1.0 equiv.) and homopiperonylamine (80.9 µL, 0.6 mmol, 3.0 equiv.). The product was purified as a pale yellow solid by flash column chromatography on silica gel using hexane/EtOAc as eluent. Yield: 93% (64.2 mg).

^{1}H NMR (500 MHz, CDCl$_3$) δ 7.78 – 7.69 (m, 4H), 7.55 – 7.50 (m, 1H), 7.47 – 7.42 (m, 2H), 6.77 – 6.73 (m, 1H), 6.71 – 6.68 (m, 1H), 6.67 – 6.63 (m, 1H), 6.62 – 6.57 (m, 2H), 5.92 (s, 2H), 3.41 (t, *J* = 7.0 Hz, 2H), 2.84 (t, *J* = 7.0 Hz, 2H).

^{13}C NMR (126 MHz, CDCl$_3$) δ 195.2, 151.7, 147.9, 146.3, 139.1, 133.1, 132.4, 131.3, 129.5, 128.1, 126.3, 121.7, 111.7, 109.1, 108.5, 101.0, 44.7, 34.9.

HRMS (ESI): calculated for $[M+Na]^+$ $C_{22}H_{19}NO_3Na^+$ 368.1257; found 368.1251.

(4-(4-(8-chloro-5,6-dihydro-11*H*-benzo[5,6]cyclohepta[1,2-*b*]pyridin-11-ylidene)piperidin-1-yl)phenyl)(phenyl)methanone (126)

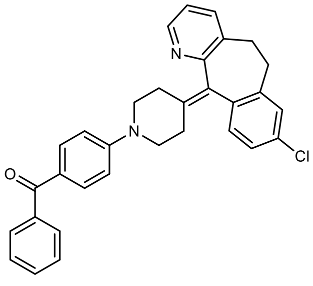

The compound was prepared according to **General Procedure A** with 4-bromobenzophenone (52.2 mg, 0.2 mmol, 1.0 equiv.) and desloratadine (186.0 mg, 0.6 mmol, 3.0 equiv.). The product was purified as a pale yellow solid by flash column chromatography on silica gel using hexane/EtOAc as eluent. Yield: 82% (80.2 mg).

^{1}H NMR (400 MHz, CDCl$_3$) δ 8.43 (dd, *J* = 4.9, 1.7 Hz, 1H), 7.81 – 7.67 (m, 4H), 7.55 – 7.39 (m, 4H), 7.20 – 7.09 (m, 4H), 6.87 – 6.79 (m, 2H), 3.79 – 3.66 (m, 2H), 3.45 – 3.31 (m, 2H), 3.25 – 3.15 (m, 2H), 2.92 – 2.70 (m, 3H), 2.62 – 2.53 (m, 1H), 2.51 – 2.40 (m, 2H).

^{13}C NMR (101 MHz, CDCl$_3$) δ 195.2, 156.6, 153.4, 146.3, 139.6, 139.0, 138.2, 137.9, 137.6, 133.8, 133.5, 133.1, 132.7, 131.4, 130.7, 129.6, 129.1, 128.1, 126.5, 126.3, 122.5, 113.0, 48.4, 48.2, 31.7, 31.6, 30.3, 30.0.

HRMS (ESI): calculated for $[M+K]^+$ $C_{32}H_{27}N_2ClOK^+$ 529.1443; found 529.1420.

(4-(4-(2-chlorodibenzo[*b*,*f*][1,4]oxazepin-11-yl)piperazin-1-yl)phenyl)(phenyl)methanone (127)

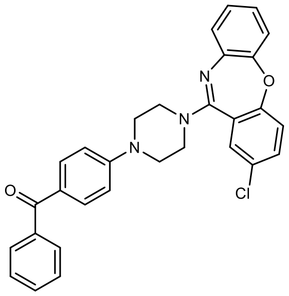

The compound was prepared according to **General Procedure A** with 4-bromobenzophenone (52.2 mg, 0.2 mmol, 1.0 equiv.) and amoxapine (188 mg, 0.6 mmol, 3.0 equiv.). The product was purified as a pale yellow solid by flash column chromatography on silica gel using hexane/EtOAc as eluent. Yield: 76% (75.1 mg).

^{1}H NMR (400 MHz, CDCl$_3$) δ 7.87 – 7.81 (m, 2H), 7.80 – 7.74 (m, 2H), 7.60 – 7.54 (m, 1H), 7.52 – 7.43 (m, 3H), 7.40 (d, *J* = 2.6 Hz, 1H), 7.31 – 7.22 (m, 2H), 7.15 (td, *J* = 5.9, 5.4, 2.8 Hz, 2H), 7.10 – 7.03 (m, 1H), 6.99 – 6.93 (m, 2H), 3.85 – 3.68 (m, 4H), 3.61 – 3.42 (m, 4H).

^{13}C NMR (101 MHz, CDCl$_3$) δ 195.4, 159.6, 159.0, 153.8, 152.1, 138.8, 133.2, 132.6, 131.7, 130.6, 129.7, 129.2, 128.2, 128.0, 127.2, 126.1, 125.4, 124.6, 123.1, 120.3, 113.8, 47.4.

HRMS (ESI): calculated for $[M+Na]^+$ $C_{30}H_{24}N_3ClO_2Na^+$ 516.1449; found 516.1466.

((5*R*,5a*S*,8a*S*,8b*R*)-2,2,7,7-tetramethyltetrahydro-5*H*-bis([1,3]dioxolo)[4,5-*b*:4',5'-*d*]pyran-5-yl)methyl 4-((4-methoxyphenyl)amino)benzoate (128)

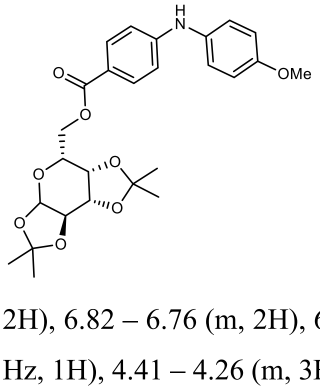

The compound was prepared according to **General Procedure B** with ((5*R*,5a*S*,8a*S*,8b*R*)-2,2,7,7-tetramethyltetrahydro-5*H*-bis([1,3]dioxolo)[4,5-*b*:4',5'-*d*]pyran-5-yl)methyl 4-bromobenzoate (88.7 mg, 0.2 mmol, 1.0 equiv.) and p-anisidine (73.9 mg, 0.6 mmol, 3.0 equiv.). The product was purified as a white solid by flash column chromatography on silica gel using hexane/EtOAc as eluent. Yield: 72% (70.1 mg).

^{1}H NMR (400 MHz, CDCl$_3$) δ 7.90 – 7.82 (m, 2H), 7.15 – 7.07 (m, 2H), 6.91 – 6.84 (m, 2H), 6.82 – 6.76 (m, 2H), 6.05 (s, 1H), 5.55 (d, *J* = 4.9 Hz, 1H), 4.63 (dd, *J* = 7.9, 2.5 Hz, 1H), 4.48 (dd, *J* = 11.4, 5.0 Hz, 1H), 4.41 – 4.26 (m, 3H), 4.19 – 4.12 (m, 1H), 3.79 (s, 3H), 1.50 (s, 3H), 1.46 (s, 3H), 1.34 (s, 3H), 1.32 (s, 3H).

^{13}C NMR (101 MHz, CDCl$_3$) δ166.5, 156.5, 150.0, 133.5, 131.7, 124.4, 119.7, 114.8, 113.2, 109.7, 108.9, 96.4, 71.2, 70.8, 70.6, 66.3, 63.4, 55.6, 26.1 (2C), 25.1, 24.5.

HRMS (ESI): calculated for $[M+Na]^+$ $C_{26}H_{31}NO_8Na^+$ 508.1942; found 508.1944.

2-(diethylamino)ethyl 4-((4-(trifluoromethyl)phenyl)amino)benzoate (129)

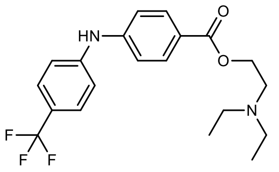

The compound was prepared according to **General Procedure B** with 4-bromobenzotrifluoride (28 μL, 0.2 mmol, 1.0 equiv.) and procaine (142 mg, 0.6 mmol, 3.0 equiv.). The product was purified as a white solid by flash column chromatography on basic alumina using hexane/DCM as eluent. Yield: 58% (44.2 mg).

^{1}H NMR (500 MHz, CD_3CN) δ 7.93 – 7.88 (m, 2H), 7.57 (d, *J* = 8.5 Hz, 2H), 7.51 (s, 1H), 7.27 (d, *J* = 8.4 Hz, 2H), 7.19 – 7.15 (m, 2H), 4.28 (t, *J* = 6.0 Hz, 2H), 2.76 (t, *J* = 6.0 Hz, 2H), 2.56 (q, *J* = 7.1 Hz, 4H), 1.00 (t, *J* = 7.1 Hz, 6H).

^{13}C NMR (126 MHz, CD_3CN) δ166.8, 147.6, 146.5, 132.1, 127.6 (q, *J* = 3.8 Hz), 125.8 (d, *J* = 270.1 Hz), 123.6, 123.0 (q, *J* = 32.2 Hz), 117.3, 64.0, 52.2, 48.4, 12.6.

^{19}F NMR (471 MHz, CD_3CN) δ -62.0.

HRMS (ESI): calculated for $[M+Na]^+$ $C_{20}H_{23}N_2F_3O_2Na^+$ 403.1604; found 403.1603.

methyl 4-(2-(4-isobutylphenyl)propanamido)benzoate (130)

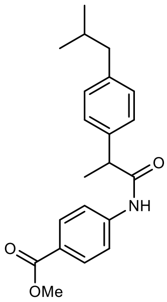

The compound was prepared according to **General Procedure D** with methyl 4-bromobenzoate (43.0 mg, 0.2 mmol, 1.0 equiv.), 2-(4-isobutylphenyl)propanamide (123 mg, 0.6 mmol, 3.0 equiv.). The product was purified as a white solid by flash column chromatography on silica gel using hexane/EtOAc as eluent. Yield: 79% (53.3 mg).

^{1}H NMR (500 MHz, $CDCl_3$) δ 7.97 – 7.92 (m, 2H), 7.52 – 7.47 (m, 2H), 7.29 – 7.23 (m, 2H), 7.18 – 7.14 (m, 2H), 3.87 (s, 3H), 3.71 (q, *J* = 7.1 Hz, 1H), 2.47 (d, *J* = 7.2 Hz, 2H), 1.86 (dh, *J* = 13.5, 6.8 Hz, 1H), 1.59 (d, *J* = 7.1 Hz, 3H), 0.91 (d, *J* = 6.6 Hz, 6H).

^{13}C NMR (126 MHz, $CDCl_3$) δ 173.0, 166.7, 142.2, 141.5, 137.8, 130.9, 130.1, 127.6, 125.6, 118.8, 52.1, 48.1, 45.1, 30.3, 22.5, 18.6. The spectral data are consistent with those reported in the literature.[89]

methyl 4-(4-(N,N-dipropylsulfamoyl)benzamido)benzoate (131)

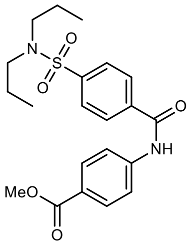

The compound was prepared according to **General Procedure D** with methyl 4-bromobenzoate (43.0 mg, 0.2 mmol, 1.0 equiv.), 4-(*N*,*N*-dipropylsulfamoyl)benzamide (171 mg, 0.6 mmol, 3.0 equiv.). The product was purified as a white solid by flash column chromatography on neutral alumina using hexane/DCM as eluent. Yield: 50% (41.5 mg).

^{1}H NMR (500 MHz, $CDCl_3$) δ 8.85 (s, 1H), 8.03 (d, *J* = 8.5 Hz, 2H), 7.90 (d, *J* = 8.1 Hz, 2H), 7.83 (d, *J* = 8.4 Hz, 2H), 7.67 (d, *J* = 8.5 Hz, 2H), 3.90 (s, 3H), 3.08 – 3.04 (m, 3H), 1.57 – 1.47 (m, 4H), 0.85 (t, *J* = 7.4 Hz, 6H).

^{13}C NMR (126 MHz, $CDCl_3$) δ 166.8, 165.2, 142.8, 142.4, 138.8, 130.9, 128.3, 127.3, 126.1, 119.6, 52.2, 50.1, 22.0, 11.3.

HRMS (ESI): calculated for $[M+Na]^+$ $C_{21}H_{26}N_2O_5SNa^+$ 441.1455; found 441.1454.

methyl 4-((4-(5-(p-tolyl)-3-(trifluoromethyl)-1*H*-pyrazol-1-yl)phenyl)sulfonamido)benzoate (132)

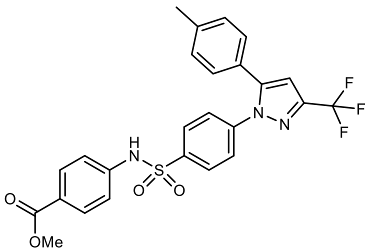

The compound was prepared according to **General Procedure C** with methyl 4-bromobenzoate (43.0 mg, 0.2 mmol, 1.0 equiv.), celecoxib (229 mg, 0.6 mmol, 3.0 equiv.). The product was purified as a white solid by flash column chromatography on silica gel using hexane/EtOAc as eluent. Yield: 83% (85.4 mg).

^{1}H NMR (500 MHz, CDCl$_3$) δ 7.94 – 7.89 (m, 2H), 7.82 – 7.77 (m, 2H), 7.55 (s, 1H), 7.42 – 7.37 (m, 2H), 7.17 – 7.09 (m, 4H), 7.04 – 7.00 (m, 2H), 6.71 (s, 1H), 3.89 (s, 3H), 2.36 (s, 3H).

^{13}C NMR (126 MHz, CDCl$_3$) δ 166.5, 145.4, 144.3 (q, *J* = 38.5 Hz), 143.1, 140.6, 140.0, 138.0, 131.3, 129.8, 128.8, 128.4, 126.9, 125.7, 121.1 (q, *J* = 269.1 Hz), 119.8, 106.5, 52.3, 21.4.

^{19}F NMR (471 MHz, CDCl$_3$) δ -62.5.

HRMS (ESI): calculated for $[M+Na]^+$ $C_{25}H_{20}F_3N_3O_4S_1Na^+$ 538.1019; found 538.1013.

((5*S*,5a*R*,8a*R*,8b*S*)-2,2,7,7-tetramethyltetrahydro-5*H*-bis([1,3]dioxolo)[4,5-*b*:4',5'-*d*]pyran-5-yl)methyl 4-((4-methylphenyl)sulfonamido)benzoate (133)

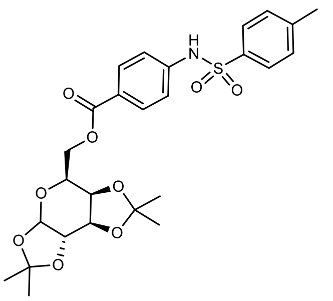

The compound was prepared according to **General Procedure C** with methyl ((5*R*,5a*S*,8a*S*,8b*R*)-2,2,7,7-tetramethyltetrahydro-5*H*-bis([1,3]dioxolo)[4,5-*b*:4',5'-*d*]pyran-5-yl)methyl 4-bromobenzoate (88.7 mg, 0.2 mmol, 1.0 equiv.), p-toluenesulfonamide (103 mg, 0.6 mmol, 3.0 equiv.). The product was purified as a white solid by flash column chromatography on silica gel using DCM/MeOH as eluent. Yield: 78% (83.3 mg).

^{1}H NMR (400 MHz, CDCl$_3$) δ 7.92 – 7.86 (m, 2H), 7.75 – 7.70 (m, 2H), 7.53 (s, 1H), 7.27 – 7.21 (m, 2H), 7.15 – 7.09 (m, 2H), 5.55 (d, *J* = 5.0 Hz, 1H), 4.64 (dd, *J* = 7.9, 2.5 Hz, 1H), 4.49 – 4.42 (m, 1H), 4.41 – 4.32 (m, 2H), 4.29 (dd, *J* = 7.9, 1.9 Hz, 1H), 4.17 – 4.10 (m, 1H), 2.37 (s, 3H), 1.49 (s, 3H), 1.45 (s, 3H), 1.34 (s, 3H), 1.33 (s, 3H).

^{13}C NMR (101 MHz, CDCl$_3$) δ 165.9, 144.5, 141.3, 135.9, 131.3, 130.0, 127.4, 126.1, 119.0, 109.8, 109.0, 96.4, 71.2, 70.8, 70.6, 66.3, 64.1, 26.1, 26.1, 25.1, 24.6, 21.7. The spectral data are consistent with those reported in the literature.[51]

4-(((5*R*,5a*S*,8a*S*,8b*R*)-2,2,7,7-tetramethyltetrahydro-5*H*-bis([1,3]dioxolo)[4,5-*b*:4',5'-d]pyran-5-yl)methoxy)benzonitrile (134)

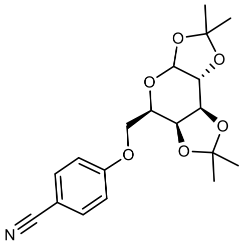

The compound was prepared according to **General Procedure E** with 4-bromobenzonitrile (36.4 mg, 0.2 mmol, 1.0 equiv.), ((5*R*,5a*S*,8a*S*,8b*R*)-2,2,7,7-tetramethyltetrahydro-5*H*-bis([1,3]dioxolo)[4,5-*b*:4',5'-*d*]pyran-5-yl)methanol (190 µL, 0.6 mmol, 3.0 equiv.). The product was purified as a white solid by flash column chromatography on silica gel using hexane/EtOAc as eluent. Yield: 48% (34.6 mg).

^{1}H NMR (400 MHz, CDCl$_3$) δ 7.59 – 7.53 (m, 2H), 7.01 – 6.96 (m, 2H), 5.55 (d, *J* = 5.0 Hz, 1H), 4.65 (dd, *J* = 7.9, 2.5 Hz, 1H), 4.37 – 4.30 (m, 2H), 4.21 – 4.11 (m, 3H), 3.46 (q, *J* = 7.0 Hz, 1H), 1.51 (s, 3H), 1.45 (s, 3H), 1.34 (s, 3H), 1.33 (s, 3H).

^{13}C NMR (101 MHz, CDCl$_3$) δ 162.0, 134.0, 119.3, 115.6, 109.7, 108.9, 104.3, 96.4, 71.0, 70.7, 70.6, 67.2, 66.3, 66.0, 26.1, 26.1, 25.0, 24.5,15.4. The spectral data are consistent with those reported in the literature.[90]

4-cyanophenyl (*S*)-2-(6-methoxynaphthalen-2-yl)propanoate (135)

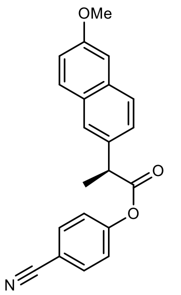

The compound was prepared according to **General Procedure D** with 4-bromobenzonitrile (36.4 mg, 0.2 mmol, 1.0 equiv.), (*S*)-naproxen (138 mg, 0.6 mmol, 3.0 equiv.). The product was purified as a white solid by flash column chromatography on silica gel using hexane/EtOAc as eluent. Yield: 52% (34.1 mg).

^{1}H NMR (500 MHz, $CDCl_3$) δ 7.80 – 7.71 (m, 3H), 7.67 – 7.56 (m, 2H), 7.50 – 7.44 (m, 1H), 7.23 – 7.16 (m, 1H), 7.16 – 7.05 (m, 3H), 4.11 (q, *J* = 7.1 Hz, 1H), 3.93 (s, 3H), 1.70 (d, *J* = 7.1 Hz, 3H).

^{13}C NMR (126 MHz, $CDCl_3$) δ 172.5, 158.0, 154.2, 134.5, 134.1, 133.7, 129.4, 129.1, 127.7, 126.3, 126.0, 122.7, 119.4, 118.3, 109.8, 105.7, 55.5, 45.7,18.4.

HRMS (ESI): calculated for $[M+Na]^+$ $C_{21}H_{17}NO_3Na^+$ 354.1101; found 354.1085

4-cyanophenyl 2-(4-chlorophenoxy)-2-methylpropanoate (136)

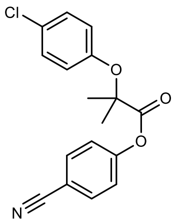

The compound was prepared according to **General Procedure D** with 4-bromobenzonitrile (36.4 mg, 0.2 mmol, 1.0 equiv.), clofibric acid (129 mg, 0.6 mmol, 3.0 equiv.). The product was purified as a colorless oil by flash column chromatography on silica gel using hexane/EtOAc as eluent. Yield: 51% (32.3 mg).

^{1}H NMR (500 MHz, $CDCl_3$) δ 7.75 – 7.67 (m, 2H), 7.30 – 7.25 (m, 2H), 7.20 – 7.15 (m, 2H), 6.94 – 6.86 (m, 2H), 1.76 (s, 6H).

^{13}C NMR (126 MHz, $CDCl_3$) δ172.1, 153.9, 153.8, 133.9, 129.5, 128.0, 122.6, 120.6, 118.2, 110.4, 79.7, 25.4.

HRMS (ESI): calculated for $[M+Na]^+$ $C_{17}H_{14}NO_3ClNa^+$ 338.0554; found 338.0540

1,3,7-trimethyl-8-(phenylthio)-3,7-dihydro-1*H*-purine-2,6-dione (137)

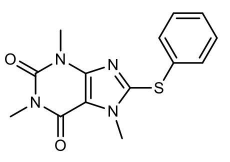

The compound was prepared according to **General Procedure F** with 8-bromo-1,3,7-trimethyl-3,7-dihydro-1*H*-purine-2,6-dione (54.6 mg, 0.2 mmol, 1.0 equiv.), benzenethiol (31 µL, 0.3 mmol, 1.5 equiv.). The product was purified as a pink solid by flash column chromatography on silica gel using hexane/EtOAc as eluent. Yield: 64% (38.8 mg).

^{1}H NMR (500 MHz, $CDCl_3$) δ7.36 – 7.27 (m, 5H), 3.91 (d, *J* = 1.4 Hz, 3H), 3.54 (d, *J* = 1.6 Hz, 3H), 3.38 (d, *J* = 1.5 Hz, 3H).

^{13}C NMR (126 MHz, $CDCl_3$) δ 155.0, 151.5, 148.1, 146.5, 131.0, 130.6, 129.7, 128.4, 109.7, 33.2, 30.0, 28.1. The spectral data are consistent with those reported in the literature.[91]

((5*R*,5a*S*,8a*S*,8b*R*)-2,2,7,7-tetramethyltetrahydro-5*H*-bis([1,3]dioxolo)[4,5-*b*:4',5'-*d*]pyran-5-yl)methyl 4-(phenylthio)benzoate (138)

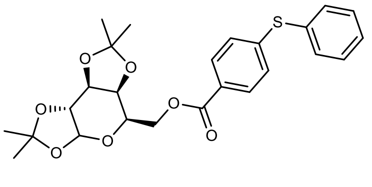

The compound was prepared according to **General Procedure F** with ((5*R*,5a*S*,8a*S*,8b*R*)-2,2,7,7-tetramethyltetrahydro-5*H*-bis([1,3]dioxolo)[4,5-*b*:4',5'-*d*]pyran-5-yl)methyl 4-bromobenzoate (88.7 mg, 0.2 mmol, 1.0 equiv.), benzenethiol (31 µL, 0.3mmol, 1.5 equiv.). The product was purified as a pink oil by flash column chromatography on silica gel using hexane/EtOAc as eluent. Yield: 78% (73.7 mg).

^{1}H NMR (500 MHz, $CDCl_3$) δ7.90 (d, *J* = 8.7 Hz, 2H), 7.48 (dd, *J* = 7.7, 1.8 Hz, 2H), 7.42 – 7.33 (m, 3H), 7.19 (d, *J* = 8.7 Hz, 2H), 5.55 (d, *J* = 5.0 Hz, 1H), 4.64 (dd, *J* = 7.9 Hz, 2.5, 1H), 4.50 (dd, *J* = 11.5, 4.8 Hz, 1H), 4.40 (dd, *J* =

11.5, 7.6 Hz, 1H), 4.34 (dd, J = 5.0, 2.5 Hz, 1H), 4.30 (dd, J = 7.9, 1.9 Hz, 1H), 4.15 (ddd, J = 7.2, 4.8, 1.9 Hz, 1H), 1.50 (s, 3H), 1.47 (s, 3H), 1.34 (s, 3H), 1.32 (s, 3H).

^{13}C NMR (126 MHz, CDCl$_3$) δ 166.1, 144.6, 133.8, 132.5, 130.3, 129.7, 128.8, 127.7, 127.5, 109.8, 108.9, 96.4, 71.2, 70.8, 70.6, 66.2, 64.0, 26.1 (2C), 25.1, 24.6.

HRMS (ESI): calculated for [M+Na]$^+$ $C_{25}H_{28}O_7SK^+$ 511.1187; found 511.1186.

4-((3-methoxyphenyl)thio)phenyl adamantane-1-carboxylate (139)

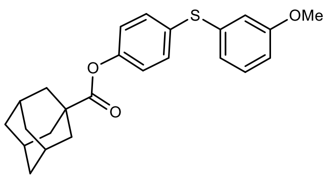

The compound was prepared according to **General Procedure F** 4-bromophenyl adamantane-1-carboxylate (76.6 mg, 0.2 mmol, 1.0 equiv.), 3-methoxybenzenethiol (37 µL, 0.3 mmol, 1.5 equiv.). The product was purified as a white solid by flash column chromatography on silica gel using hexane/EtOAc as eluent. Yield: 77% (60.8 mg).

^{1}H NMR (400 MHz, CDCl$_3$) δ7.42 – 7.36 (m, 2H), 7.19 (t, J = 8.0 Hz, 1H), 7.04 – 6.99 (m, 2H), 6.86 (m, 1H), 6.83 (t, J = 2.1 Hz, 1H), 6.76 (m, 1H), 3.75 (s, 3H), 2.08 (q, J = 3.0 Hz, 3H), 2.04 (d, J = 2.9 Hz, 6H), 1.76 (t, J = 3.3 Hz, 6H).

^{13}C NMR (101 MHz, CDCl3) δ 176.1, 160.2, 150.7, 137.7, 133.2, 131.8, 130.1, 122.6, 122.5, 115.5, 112.8, 55.4, 41.2, 38.8, 36.5, 28.0.

HRMS (ESI): calculated for [M+Na]$^+$ $C_{24}H_{26}O_3SNa^+$ 417.1495; found 417.1487.

methyl *N*-(*tert*-butoxycarbonyl)-*S*-(4-cyanophenyl)-*L*-cysteinate (140)

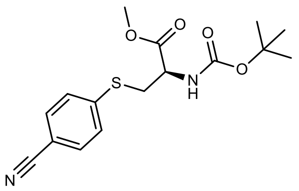

The compound was prepared according to **General Procedure F** 4-bromobenzonitrile (36.4 mg, 0.2 mmol, 1.0 equiv.), *N*-(*tert*-butoxycarbonyl)-*L*-cysteine methyl ester (62 µL, 0.3 mmol, 1.5 equiv.). The product was purified as a white solid by flash column chromatography on silica gel using hexane/EtOAc as eluent. Yield: 58% (39.1 mg).

^{1}H NMR (400 MHz, CDCl$_3$) δ7.53 (d, J = 8.5 Hz, 2H), 7.39 (d, J = 8.4 Hz, 2H), 5.33 (d, J = 7.7 Hz, 1H), 4.62 (dt, J = 8.3, 5.0 Hz, 1H), 3.66 (s, 3H), 3.51 (dd, J = 14.0, 4.9 Hz, 1H), 3.40 (dd, J = 14.0, 5.0 Hz, 1H), 1.41 (s, 9H).

^{13}C NMR (101 MHz, CDCl$_3$) δ 170.7, 155.0, 143.1, 132.5, 128.5, 118.7, 109.4, 80.6, 53.3, 52.8, 35.3, 28.4.

HRMS (ESI): calculated for [M+Na]$^+$ $C_{16}H_{20}N_2O_4SNa^+$ 359.1036; found 359.1024

methyl *N*-(*tert*-butoxycarbonyl)-*S*-(4-(trifluoromethyl)phenyl)-*L*-cysteinate (141)

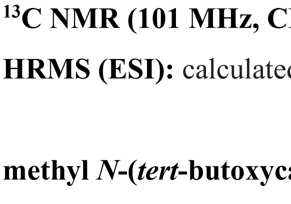

The compound was prepared according to **General Procedure F** 4-bromobenzonitrile (28 µL, 0.2 mmol, 1.0 equiv.), *N*-(*tert*-butoxycarbonyl)-L-cysteine methyl ester (62 µL, 0.3 mmol, 1.5 equiv.). The product was purified as a white solid by flash column chromatography on silica gel using hexane/EtOAc as eluent. Yield: 87% (65.9 mg).

^{1}H NMR (400 MHz, CDCl$_3$) δ7.52 (d, J = 8.4 Hz, 2H), 7.45 (d, J = 8.3 Hz, 2H), 5.33 (d, J = 7.8 Hz, 1H), 4.66 – 4.57 (m, 1H), 3.62 (s, 3H), 3.54 – 3.35 (m, 2H), 1.40 (s, 9H).

^{13}C NMR (101 MHz, CDCl$_3$) δ 170.9, 155.0, 140.6, 129.5, 128.6 (q, J = 28.2 Hz), 125.9 (q, J = 4.0 Hz), 124.1(q, J = 272.7 Hz), 80.4, 53.5, 52.7, 36.1, 28.3.

^{19}F NMR (377 MHz, CDCl$_3$) δ -62.6.

HRMS (ESI): calculated for [M+Na]$^+$ $C_{16}H_{20}F_3NO_4SNa^+$ 402.0968; found 402.0957.

methyl (*R*)-4-((2-((*tert*-butoxycarbonyl)amino)-3-methoxy-3-oxopropyl)thio)benzoate (142)

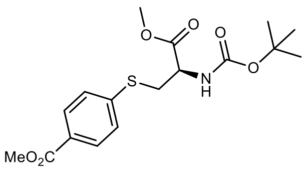

The compound was prepared according to **General Procedure F** 4-bromobenzoate (43.0 mg, 0.2 mmol, 1.0 equiv.), *N*-(*tert*-Butoxycarbonyl)-*L*-cysteine methyl ester (62 µL, 0.3 mmol, 1.5 equiv.). The product was purified as a white solid by flash column chromatography on silica gel using hexane/EtOAc as eluent. Yield: 70% (51.8 mg).

^{1}H NMR (400 MHz, CDCl$_3$) δ7.53 (d, *J* = 8.5 Hz, 2H), 7.39 (d, *J* = 8.4 Hz, 2H), 5.33 (d, *J* = 7.7 Hz, 1H), 4.62 (dt, *J* = 8.3, 5.0 Hz, 1H), 3.66 (s, 3H), 3.51 (dd, *J* = 14.0, 4.9 Hz, 1H), 3.40 (dd, *J* = 14.0, 5.0 Hz, 1H), 1.41 (s, 9H).

^{13}C NMR (101 MHz, CDCl$_3$) δ 170.7, 155.0, 143.1, 132.5, 128.5, 118.7, 109.4, 80.6, 53.3, 52.8, 35.3, 28.4.

HRMS (ESI): calculated for $[M+Na]^+$ $C_{17}H_{23}NO_6SNa^+$ 392.1138; found 392.1148.

((5*R*,5a*S*,8a*S*,8b*R*)-2,2,7,7-tetramethyltetrahydro-5*H*-bis([1,3]dioxolo)[4,5-*b*:4',5'-*d*]pyran-5-yl)methyl 4-bromobenzoate white solid (143)

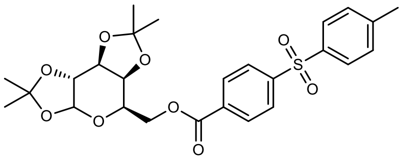

The compound was prepared according to **General Procedure G** ((5*R*,5a*S*,8a*S*,8b*R*)-2,2,7,7-tetramethyltetrahydro-5*H*-bis([1,3]dioxolo)[4,5-*b*:4',5'-*d*]pyran-5-yl)methyl 4-bromobenzoate (88.7 mg, 0.2 mmol, 1.0 equiv.), sodium 4-methylbenzenesulfinate (64.2 mg, 0.36 mmol, 1.8 equiv.). The product was purified as a white solid by flash column chromatography on silica gel using hexane/EtOAc as eluent. Yield: 89% (92.3 mg).

^{1}H NMR (500 MHz, CDCl$_3$) δ8.13 (d, *J* = 8.6 Hz, 2H), 7.97 (d, *J* = 8.6 Hz, 2H), 7.82 (d, *J* = 8.4 Hz, 2H), 7.30 (d, *J* = 8.1 Hz, 2H), 5.53 (d, *J* = 4.9 Hz, 1H), 4.64 (dd, *J* = 7.8, 2.5 Hz, 1H), 4.51 (dd, *J* = 11.6, 4.5 Hz, 1H), 4.44 (dd, *J* = 11.6, 7.8 Hz, 1H), 4.33 (dd, *J* = 5.0, 2.5 Hz, 1H), 4.28 (dd, *J* = 7.9, 1.9 Hz, 1H), 4.15 (ddd, *J* = 7.8, 4.5, 1.9 Hz, 1H), 2.39 (s, 3H), 1.47 (d, *J* = 12.4 Hz, 6H), 1.32 (d, *J* = 10.2 Hz, 6H).

^{13}C NMR (126 MHz, CDCl$_3$) δ 165.0, 146.0, 144.8, 138.0, 134.1, 130.6, 130.2, 128.0, 127.7, 109.9, 108.9, 96.4, 71.2, 70.8, 70.6, 66.1, 64.7, 26.1, 26.1, 25.0, 24.6, 21.7.

HRMS (ESI): calculated for $[M+Na]^+$ $C_{26}H_{30}O_9SNa^+$ 541.1503; found 541.1497.

isopropyl 2-(4-(4-bromobenzoyl)phenoxy)-2-methylpropanoate (144)

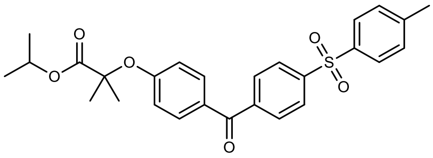

The compound was prepared according to **General Procedure G** isopropyl 2-(4-(4-bromobenzoyl)phenoxy)-2-methylpropanoate (81.1 mg, 0.2 mmol, 1.0 equiv.), sodium 4-methylbenzenesulfinate (64.2 mg, 0.36 mmol, 1.8 equiv.). The product was purified as a white solid by flash column chromatography on silica gel using hexane/EtOAc as eluent. Yield: 86 % (82.7 mg).

^{1}H NMR (500 MHz, CDCl$_3$) δ8.04 (d, *J* = 8.4 Hz, 2H), 7.87 (d, *J* = 8.3 Hz, 2H), 7.82 (d, *J* = 8.4 Hz, 2H), 7.73 (d, *J* = 8.8 Hz, 2H), 7.35 (d, *J* = 8.0 Hz, 2H), 6.87 (d, *J* = 8.8 Hz, 2H), 5.09 (hept, *J* = 6.3 Hz, 1H), 2.43 (s, 3H), 1.67 (s, 6H), 1.21 (d, *J* = 6.3 Hz, 6H).

^{13}C NMR (126 MHz, CDCl$_3$) δ 194.0, 173.1, 160.4, 144.8, 144.8, 142.4, 138.1, 132.3, 130.2, 129.5, 128.0, 127.6, 117.4, 79.6, 69.5, 25.5, 21.7, 21.6.

HRMS (ESI): calculated for $[M+Na]^+$ $C_{27}H_{28}O_6SNa^+$ 503.1499; found 503.1509.

(10*R*,13*S*)-17-acetyl-10,13-dimethyl-2,3,4,7,8,9,10,11,12,13,14,15,16,17-tetradecahydro-1*H*-cyclopenta[*a*]phenanthren-3-yl 4-bromobenzoate (145)

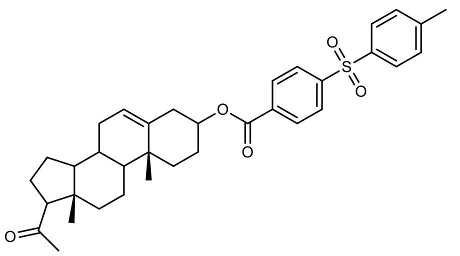

The compound was prepared according to **General Procedure G** (10*R*,13*S*)-17-acetyl-10,13-dimethyl-2,3,4,7,8,9,10,11,12,13,14,15,16,17-tetradecahydro-1*H*-cyclopenta[*a*]phenanthren-3-yl 4-bromobenzoate (99.9 mg, 0.2 mmol, 1.0 equiv.), sodium 4-methylbenzenesulfinate (64.2 mg, 0.36 mmol, 1.8 equiv.). The product was purified as a white solid by flash column chromatography on silica gel using hexane/EtOAc as eluent. Yield: 61% (70.2 mg).

^{1}H NMR (400 MHz, CDCl$_3$) δ8.15 (d, *J* = 8.5 Hz, 2H), 8.00 (d, *J* = 8.5 Hz, 2H), 7.85 (d, *J* = 8.3 Hz, 2H), 7.33 (d, *J* = 8.1 Hz, 2H), 5.48 – 5.40 (m, 1H), 4.94 – 4.83 (m, 1H), 2.57 (t, *J* = 8.9 Hz, 1H), 2.46 (d, *J* = 8.1 Hz, 2H), 2.42 (s, 3H), 2.25 – 2.17 (m, 1H), 2.15 (s, 3H), 2.09 – 1.92 (m, 4H), 1.80 – 1.66 (m, 5H), 1.60 – 1.44 (m, 4H), 1.20 (ddd, *J* = 18.2, 9.3, 4.8 Hz, 3H), 1.08 (s, 4H), 0.66 (s, 3H).

^{13}C NMR (101 MHz, CDCl$_3$) δ 209.7, 164.6, 145.8, 144.8, 139.4, 138.0, 134.9, 130.5, 130.2, 128.0, 127.6, 122.9, 63.8, 56.9, 50.0, 44.1, 38.9, 38.2, 37.1, 36.8, 31.9, 31.7, 27.9, 24.6, 23.0, 21.8, 21.2, 19.5, 13.4.

HRMS (ESI): calculated for [M+Na]$^+$ $C_{35}H_{42}O_5SNa^+$ 597.2645; found 597.2642.

2-(piperidin-1-yl)-9*H*-fluoren-9-one (146)

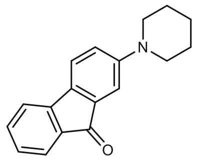

The compound was prepared according to **General Procedure A** with 2-bromo-9-fluorenone (51.8 mg, 0.2 mmol, 1.0 equiv.) and piperidine (55 μL, 0.6 mmol, 3.0 equiv.). The product was purified as a red solid by flash column chromatography on silica gel using hexane/DCM as eluent. Yield: 72% (38.1 mg).

^{1}H NMR (400 MHz, CDCl$_3$) δ 7.55 (d, *J* = 7.3 Hz, 1H), 7.37 (td, *J* = 7.4, 1.2 Hz, 1H), 7.34 – 7.28 (m, 2H), 7.22 (d, *J* = 2.5 Hz, 1H), 7.12 (td, *J* = 7.3, 1.2 Hz, 1H), 6.94 (d, *J* = 8.1 Hz, 1H), 3.25 – 3.18 (m, 4H), 1.75 – 1.65 (m, 4H), 1.64 – 1.53 (m, 2H).

^{13}C NMR (101 MHz, CDCl$_3$) δ 194.65, 152.69, 145.44, 135.54, 134.78, 134.32, 127.34, 124.16, 121.14, 120.81, 119.31, 112.09, 50.32, 25.49, 24.15 24.2. The spectral data are consistent with those reported in the literature.[26]

phenyl(4-(piperidin-1-yl)phenyl)methanone (147)

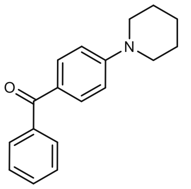

The compound was prepared according to **General Procedure A** with 4-bromobenzophenone (52.2 mg, 0.2 mmol, 1.0 equiv.) and piperidine (55 μL, 0.6 mmol, 3.0 equiv.). The product was purified as a pale yellow solid by flash column chromatography on silica gel using hexane/EtOAc as eluent. Yield: 92% (48.9 mg).

^{1}H NMR (400 MHz, CDCl$_3$) δ 7.82 – 7.70 (m, 4H), 7.56 – 7.49 (m, 1H), 7.48 – 7.42 (m, 2H), 6.88 (d, *J* = 8.6 Hz, 2H), 3.37 (t, *J* = 5.1 Hz, 4H), 1.73 – 1.66 (m, 6H).

^{13}C NMR (101 MHz, CDCl$_3$) δ 195.2, 154.3, 139.1, 132.7, 131.4, 129.6, 128.1, 126.2, 113.2, 48.7, 25.4, 24.4. The spectral data are consistent with those reported in the literature.[26]

1-(4-nitrophenyl)piperidine (148)

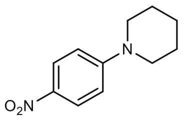

The compound was prepared according to **General Procedure A** with 1-bromo-4-nitrobenzene (40.4 mg, 0.2 mmol, 1.0 equiv.) and piperidine (55 μL, 0.6 mmol, 3.0 equiv.). The product was purified as a yellow solid by flash column chromatography on silica gel using hexane/EtOAc as eluent. Yield: 58% (24.0 mg).

^{1}H NMR (400 MHz, CDCl$_3$) δ 8.14 – 8.05 (m, 1H), 6.84 – 6.76 (m, 1H), 3.44 (t, *J* = 4.6 Hz, 4H), 1.71 – 1.66 (m, 6H).

^{13}C NMR (101 MHz, CDCl$_3$) δ 154.9, 137.7, 126.3, 112.6, 48.6, 25.4, 24. 3. The spectral data are consistent with those reported in the literature.[26]

1-(phenanthren-3-yl)piperidine (149)

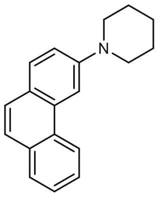

The compound was prepared according to **General Procedure A** with 3-bromophenanthrene (51.4 mg, 0.2 mmol, 1.0 equiv.) and piperidine (55 μL, 0.6 mmol, 3.0 equiv.). The product was purified as a white solid by flash column chromatography on silica gel using hexane/EtOAc as eluent. Yield: 90% (47.2 mg).

^{1}H NMR (500 MHz, CDCl$_3$) δ 8.65 (d, *J* = 8.2 Hz, 1H), 8.13 – 8.09 (m, 1H), 7.87 (dd, *J* = 7.8, 1.5 Hz, 1H), 7.79 (d, *J* = 8.8 Hz, 1H), 7.69 – 7.55 (m, 4H), 7.36 (dd, *J* = 8.8, 2.4 Hz, 1H), 3.42 – 3.36 (m, 4H), 1.88 – 1.78 (m, 4H), 1.72 – 1.65 (m, 2H).

^{13}C NMR (126 MHz, CDCl$_3$) δ 150.9, 132.7, 131.4, 130.0, 129.4, 128.7, 126.7, 126.4, 126.0, 126.0, 124.1, 122.7, 118.9, 107.6, 51.3, 26.0, 24. 5.

HRMS (ESI): calculated for $[M+H]^+$ $C_{19}H_{20}N^+$ 262.1590; found 262.1595.

1-(pyren-1-yl)piperidine (150)

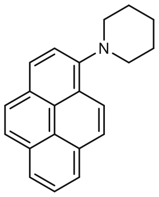

The compound was prepared according to **General Procedure A** with 1-bromopyrene (56.2 mg, 0.2 mmol, 1.0 equiv.) and piperidine (55 μL, 0.6 mmol, 3.0 equiv.). The product was purified as a white solid by flash column chromatography on silica gel using hexane/EtOAc as eluent. Yield: 86% (49.1 mg).

^{1}H NMR (500 MHz, CDCl$_3$) δ 8.49 (d, *J* = 9.2 Hz, 1H), 8.19 – 8.08 (m, 4H), 8.04 – 7.94 (m, 3H), 7.74 (d, *J* = 8.2 Hz, 1H), 3.24 (s, 4H), 2.02 – 1.94 (m, 4H), 1.75 (s, 2H).

^{13}C NMR (126 MHz, CDCl$_3$) δ 149.2, 131.7, 131.5, 127.5, 127.2, 126.5, 126.1, 126.0, 125.7, 125.5, 125.4, 124.9, 124.5, 124.4, 123.4, 117.1, 55.1, 26.9, 24.7.

HRMS (ESI): calculated for $[M+H]^+$ $C_{21}H_{20}N^+$ 286.1590; found 286.1594.

5-(piperidin-1-yl)quinoline-8-carbonitrile (151)

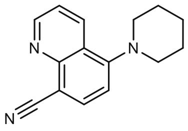

The compound was prepared according to **General Procedure A** with 5-bromoquinoline-8-carbonitrile (46.6 mg, 0.2 mmol, 1.0 equiv.) and piperidine (55 μL, 0.6 mmol, 3.0 equiv.). The product was purified as a yellow solid by flash column chromatography on silica gel using hexane/EtOAc as eluent. Yield: 86% (41.0 mg).

^{1}H NMR (400 MHz, CDCl$_3$) δ 9.01 (dd, *J* = 4.2, 1.7 Hz, 1H), 8.43 (dd, *J* = 8.5, 1.7 Hz, 1H), 7.97 (d, *J* = 8.0 Hz, 1H), 7.46 (dd, *J* = 8.6, 4.2 Hz, 1H), 7.02 (d, *J* = 8.0 Hz, 1H), 3.12 (t, *J* = 5.3 Hz, 4H), 1.92 – 1.79 (m, 4H), 1.74 – 1.62 (m, 2H).

^{13}C NMR (101 MHz, CDCl$_3$) δ 155.6, 151.9, 149.1, 136.3, 133.3, 123.5, 121.2, 118.2, 113.8, 106.0, 54.5, 26.2, 24.3. The spectral data are consistent with those reported in the literature.[26]

4-(piperidin-1-yl)benzo[*c*][1,2,5]thiadiazole (152)

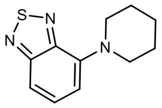

The compound was prepared according to **General Procedure A** with 4-bromobenzo[*c*][1,2,5]thiadiazole (43.0 mg, 0.2 mmol, 1.0 equiv.) and piperidine (55 µL, 0.6 mmol, 3.0 equiv.). The product was purified as a yellowish green oil by flash column chromatography on silica gel using hexane/EtOAc as eluent. Yield: 88% (38.7 mg).

^{1}H NMR (400 MHz, CDCl$_3$) δ 7.51 (dd, J = 8.7, 1.1 Hz, 1H), 7.45 (dd, J = 8.7, 7.2 Hz, 1H), 6.73 (dd, J = 7.2, 1.1 Hz, 1H), 3.51 – 3.44 (m, 4H), 1.88 – 1.82 (m, 4H), 1.71 – 1.65 (m, 2H).

^{13}C NMR (101 MHz, CDCl$_3$) δ 156.8, 150.1, 145.5, 130.7, 113.1, 111.6, 51.8, 26.124.67.

HRMS (ESI): calculated for [M+Na]$^+$ $C_{11}H_{13}N_3SNa^+$ 242.0722; found 242.0713

5-(piperidin-1-yl)benzo[*c*][1,2,5]thiadiazole (153)

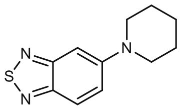

The compound was prepared according to **General Procedure A** with 5-bromobenzo[*c*][1,2,5]thiadiazole (43.0 mg, 0.2 mmol, 1.0 equiv.) and piperidine (55 µL, 0.6 mmol, 3.0 equiv.). The product was purified as a yellowish green oil by flash column chromatography on silica gel using hexane/EtOAc as eluent. Yield: 92% (40.2 mg).

^{1}H NMR (400 MHz, CDCl$_3$) δ 7.74 (d, J = 9.6 Hz, 1H), 7.43 (dd, J = 9.6, 2.4 Hz, 1H), 7.07 (d, J = 2.5 Hz, 1H), 3.31 – 3.23 (m, 4H), 1.77 – 1.67 (m, 4H), 1.67 – 1.58 (m, 2H).

^{13}C NMR (101 MHz, CDCl$_3$) δ 156.8, 152.8, 150.7, 125.5, 120.9, 101.2, 50.5, 25.6, 24.2.

HRMS (ESI): calculated for [M+Na]$^+$ $C_{11}H_{13}N_3SNa^+$ 242.0722; found 242.0725.

2-(piperidin-1-yl)quinoxaline (154)

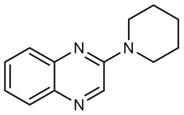

The compound was prepared according to **General Procedure A** with 2-bromoquinoxaline (41.8 mg, 0.2 mmol, 1.0 equiv.) and piperidine (55 µL, 0.6 mmol, 3.0 equiv.). The product was purified as a beige solid by flash column chromatography on silica gel using hexane/EtOAc as eluent. Yield: 43% (18.3 mg).

^{1}H NMR (400 MHz, CDCl$_3$) δ 8.58 (s, 1H), 7.88 – 7.82 (m, 1H), 7.71 – 7.65 (m, 1H), 7.59 – 7.51 (m, 1H), 7.40 – 7.32 (m, 1H), 3.81 – 3.74 (m, 4H), 1.75 – 1.66 (m, 6H).

^{13}C NMR (101 MHz, CDCl$_3$) δ 152.4, 141.8, 136.5, 136.2, 130.1, 128.7, 126.3, 124.5, 46.1, 25.8, 24.7. The spectral data are consistent with those reported in the literature.[26]

2-(piperidin-1-yl)pyrazine (155)

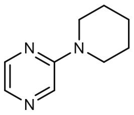

The compound was prepared according to **General Procedure A** with 2-bromoquinoxaline (18.1 µL, 0.2 mmol, 1.0 equiv.) and piperidine (55 µL, 0.6 mmol, 3.0 equiv.). The product was purified as a colorless oil by flash column chromatography on silica gel using hexane/EtOAc as eluent. Yield: 52% (16.8 mg).

^{1}H NMR (400 MHz, CDCl$_3$) δ 8.11 (s, 1H), 8.03 (t, J = 2.1 Hz, 1H), 7.75 (d, J = 2.7 Hz, 1H), 3.60 – 3.53 (m, 4H), 1.72 – 1.58 (m, 6H).

^{13}C NMR (101 MHz, CDCl$_3$) δ 24.6. The spectral data are consistent with those reported in the literature.[92]

3,6-di(piperidin-1-yl)-9*H*-fluoren-9-one (156)

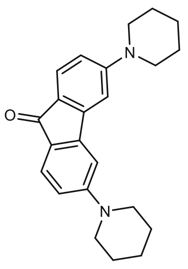

The compound was prepared according to modified **General Procedure A** with 3,6-dibromo-9*H*-fluoren-9-one (33.8 mg, 0.1 mmol, 1.0 equiv.), piperidine (55 µL, 0.6 mmol, 6.0 equiv.), DABCO (0.4 mmol, 4.0 equiv.), $NiCl_2$·dme (0.02 mmol, 0.2 equiv.), 4MeODPATPN (0.006 mmol, 0.06 equiv.). The product was purified as a red solid by flash column chromatography on silica gel using hexane/EtOAc as eluent. Yield: 68% (23.4 mg).

^{1}H NMR (400 MHz, $CDCl_3$) δ 7.52 – 7.45 (m, 2H), 7.00 (s, 2H), 6.70 – 6.63 (m, 2H), 3.40 (t, *J* = 5.2 Hz, 8H), 1.75 – 1.60 (m, 12H).

^{13}C NMR (101 MHz, $CDCl_3$) δ 191.4, 155.8, 146.0, 125.4, 113.3, 106.3, 49.5, 25.6, 24.4.

HRMS (ESI): calculated for $[M+Na]^+$ $C_{23}H_{26}N_2ONa^+$ 369.1937; found 369.1936.

1-(4-methoxyphenyl)piperidine (157)

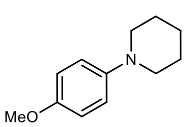

The compound was prepared according to modified **General Procedure A** with 1-iodo-4-methoxybenzene (46.8 mg, 0.2 mmol, 1.0 equiv.), piperidine (55 µL, 0.6 mmol, 6.0 equiv.), DABCO (0.4 mmol, 2.0 equiv.), $NiCl_2$·dme (0.02 mmol, 0.1 equiv.), 4MeODPATPN (0.006 mmol, 0.03 equiv.). The product was purified as a colorless oil by flash column chromatography on silica gel using hexane/EtOAc as eluent. Yield: 90% (34.4 mg).

^{1}H NMR (400 MHz, $CDCl_3$) δ 6.98 – 6.91 (m, 2H), 6.87 – 6.80 (m, 2H), 3.77 (s, 3H), 3.10 – 2.95 (m, 4H), 1.82 – 1.68 (m, 4H), 1.61 – 1.50 (m, 2H).

^{13}C NMR (101 MHz, $CDCl_3$) δ 153.9, 146.7, 119.0, 114.5, 77.4, 55.7, 52.6, 26.2, 24.2. The spectral data are consistent with those reported in the literature.[93]

2-(2-fluoro-[1,1'-biphenyl]-4-yl)propanenitrile (158)

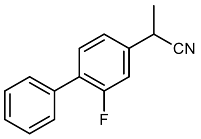

A dry 4-mL vial equipped with a Teflon-coated magnetic stir bar was charged with 1,3-dioxoisoindolin-2-yl 2-(2-fluoro-[1,1'-biphenyl]-4-yl)propanoate (38.9 mg, 0.1 mmol, 1 equiv.), 2,2'-bipyridyl (1.87 mg, 0.012 mmol, 12 mol %) and photocatalyst (0.0025 mmol, 0.025 equiv.). The vial was introduced to a glovebox filled with Argon atmosphere. Then $[Cu(MeCN)_4]PF_6$ (3.73 mg, 0.01 mmol, 10 mol %), anhydrous and degassed DMA (1.0 mL), and trimethylsilyl cyanide (0.2 mmol, 2 equiv., 26.7 µl) were added. The vial was capped with Teflon septum and removed from the glovebox. Then, the reaction mixture was stirred at room temperature for 48 h under irradiation with 620 nm LED lamps (AL3 parallel photoreactor, set up 15W) with fan cooling. Upon completion, brine (20 mL) was added and the mixture was extracted with EtOAc (20 mL) three times. The combined organic layer was washed with H_2O (20 mL) and brine (20 mL). The organic layer was dried with anhydrous Na_2SO_4, then concentrated under vacuum. The product was purified by flash column chromatography on silica gel using hexane: EtOAc as eluent. Yield: 91% (20.5 mg).

^{1}H NMR (500 MHz, $CDCl_3$) δ 7.54 (d, *J* = 7.6 Hz, 2H), 7.50 – 7.43 (m, 3H), 7.43 – 7.36 (m, 1H), 7.23 (dd, *J* = 8.0, 1.3 Hz, 1H), 7.18 (dd, *J* = 10.8, 1.6 Hz, 1H), 3.94 (q, *J* = 7.3 Hz, 1H), 1.69 (d, *J* = 7.3 Hz, 3H).[93]

^{13}C NMR (126 MHz, $CDCl_3$) δ 159.9 (d, *J* = 249.7 Hz), 138.3 (d, *J* = 7.7 Hz), 135.1, 131.6 (d, *J* = 4.0 Hz), 129.1 (d, *J* = 13.2 Hz), 129.1 (d, *J* = 3.3 Hz), 128.7, 128.1, 122.8 (d, *J* = 3.4 Hz), 121.1, 114.8 (d, *J* = 24.6 Hz), 30.9, 21.3.

^{19}F NMR (471 MHz, $CDCl_3$) δ -116.27. The spectral data are consistent with those reported in the literature.[94]

8. Copies of NMR spectra

2MeODPA---bis(3,5-dimethoxyphenyl)amine

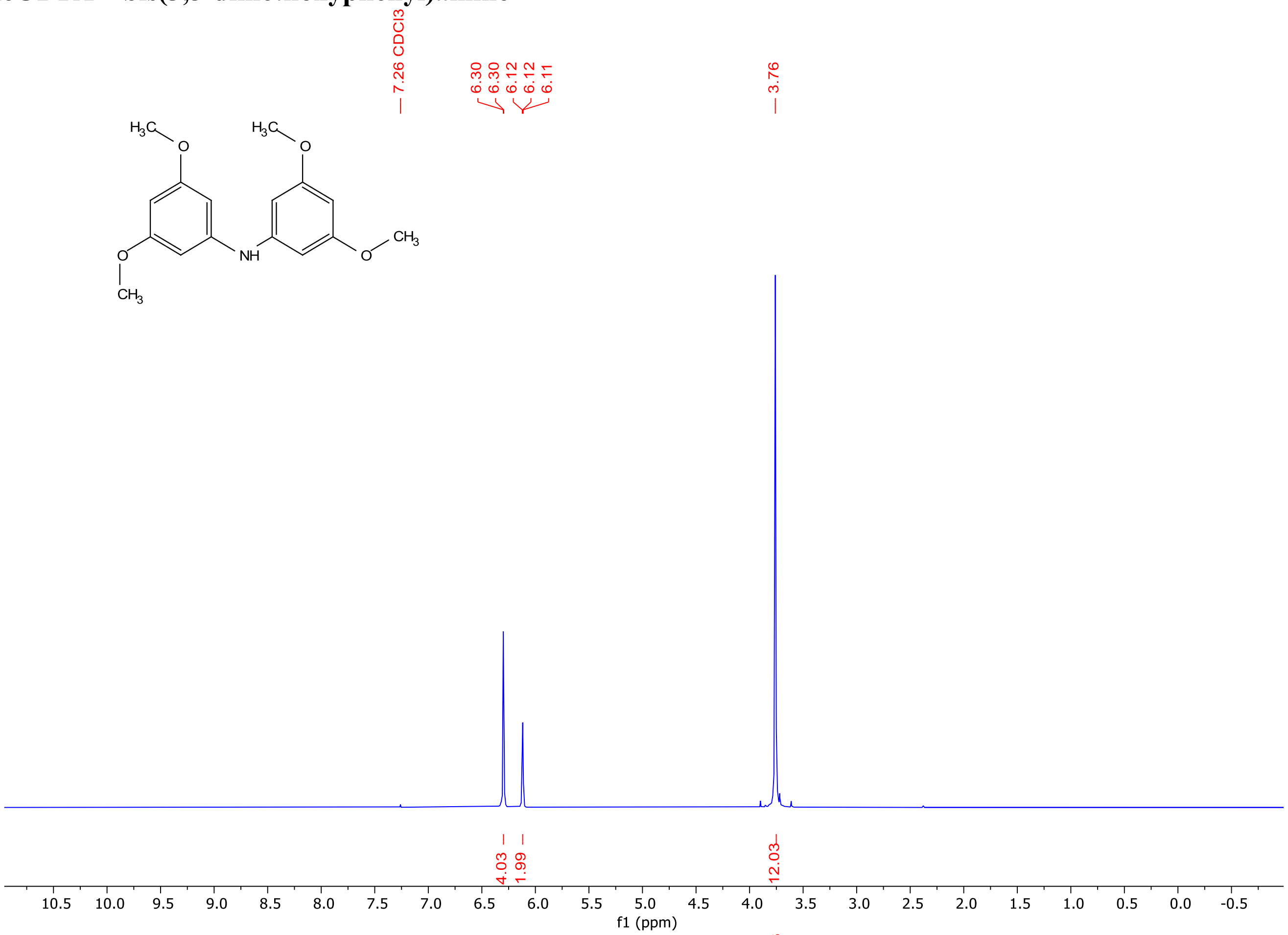

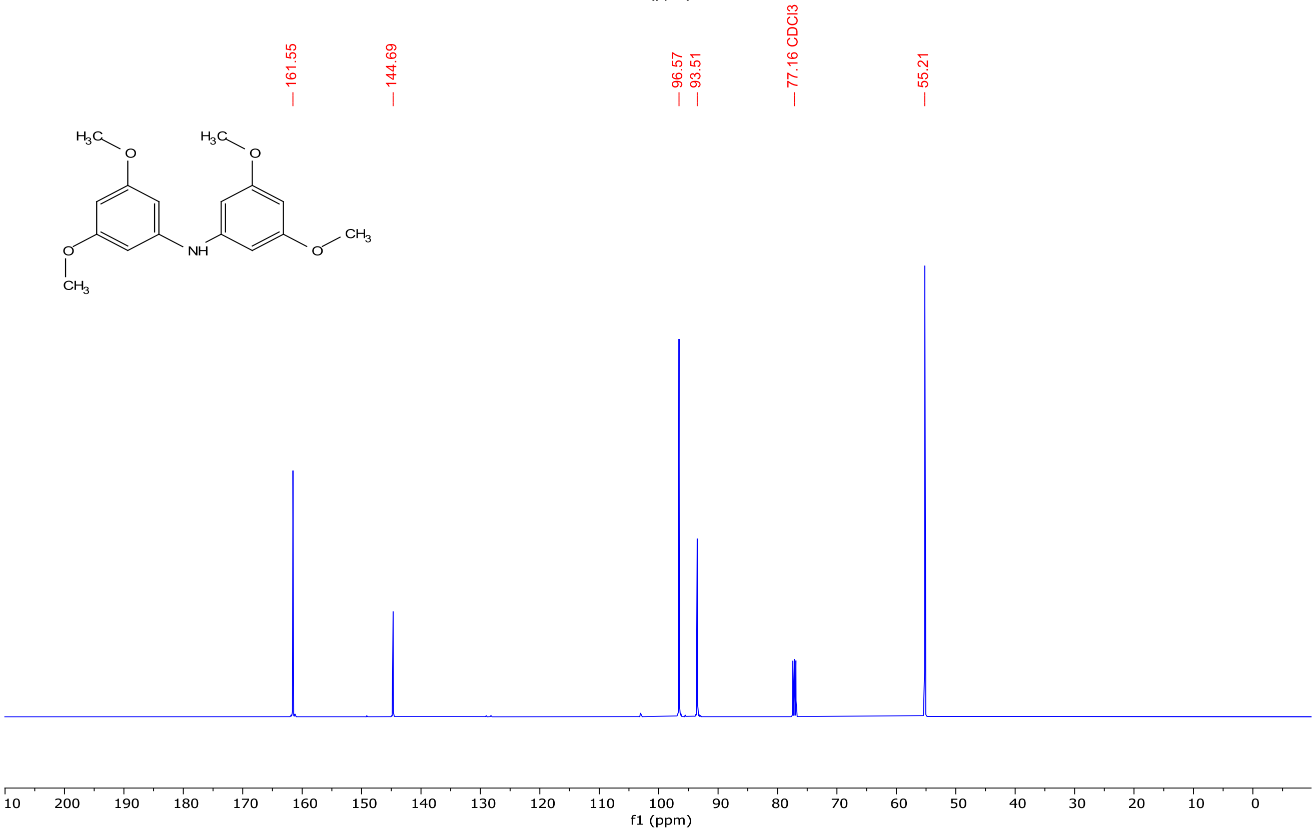

3MeODPA---bis(3,4,5-trimethoxyphenyl)amine

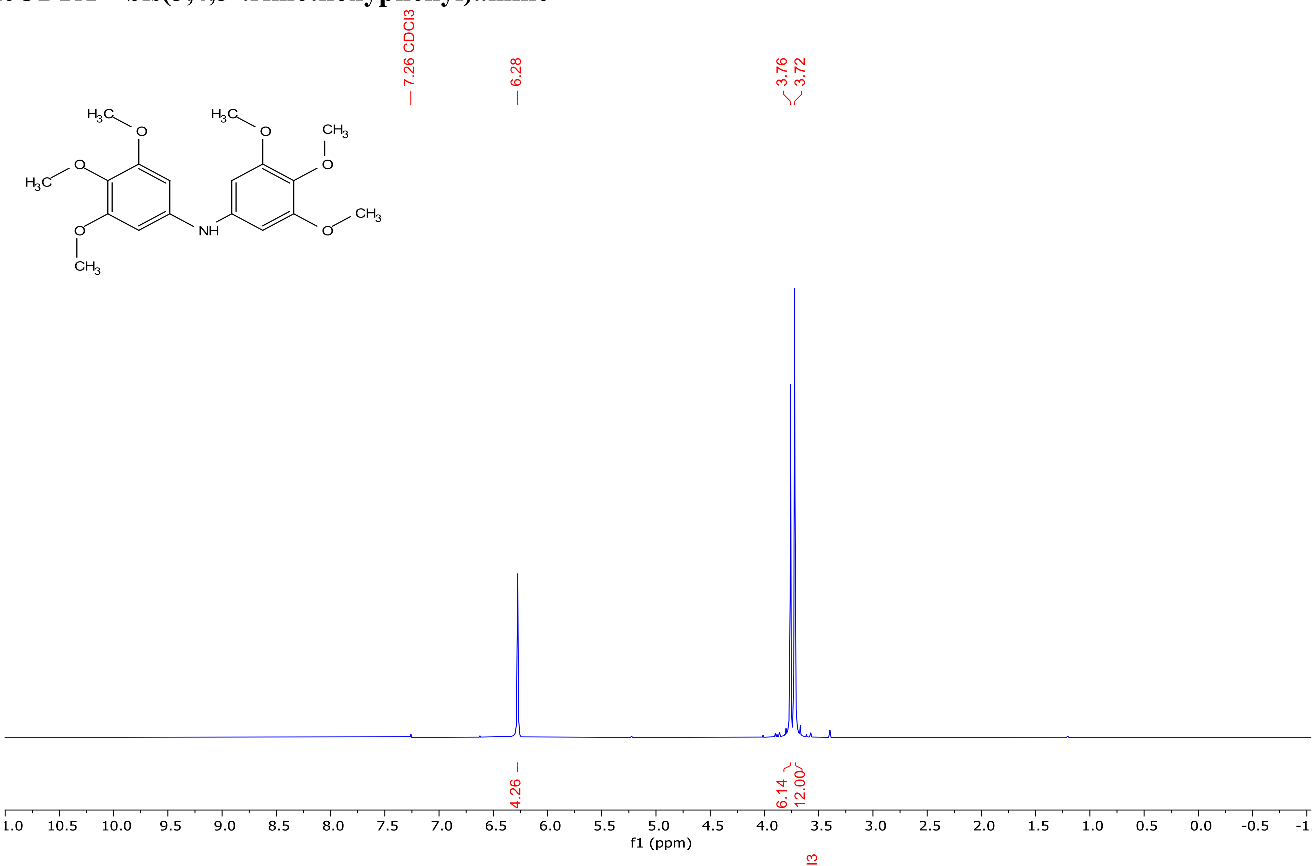

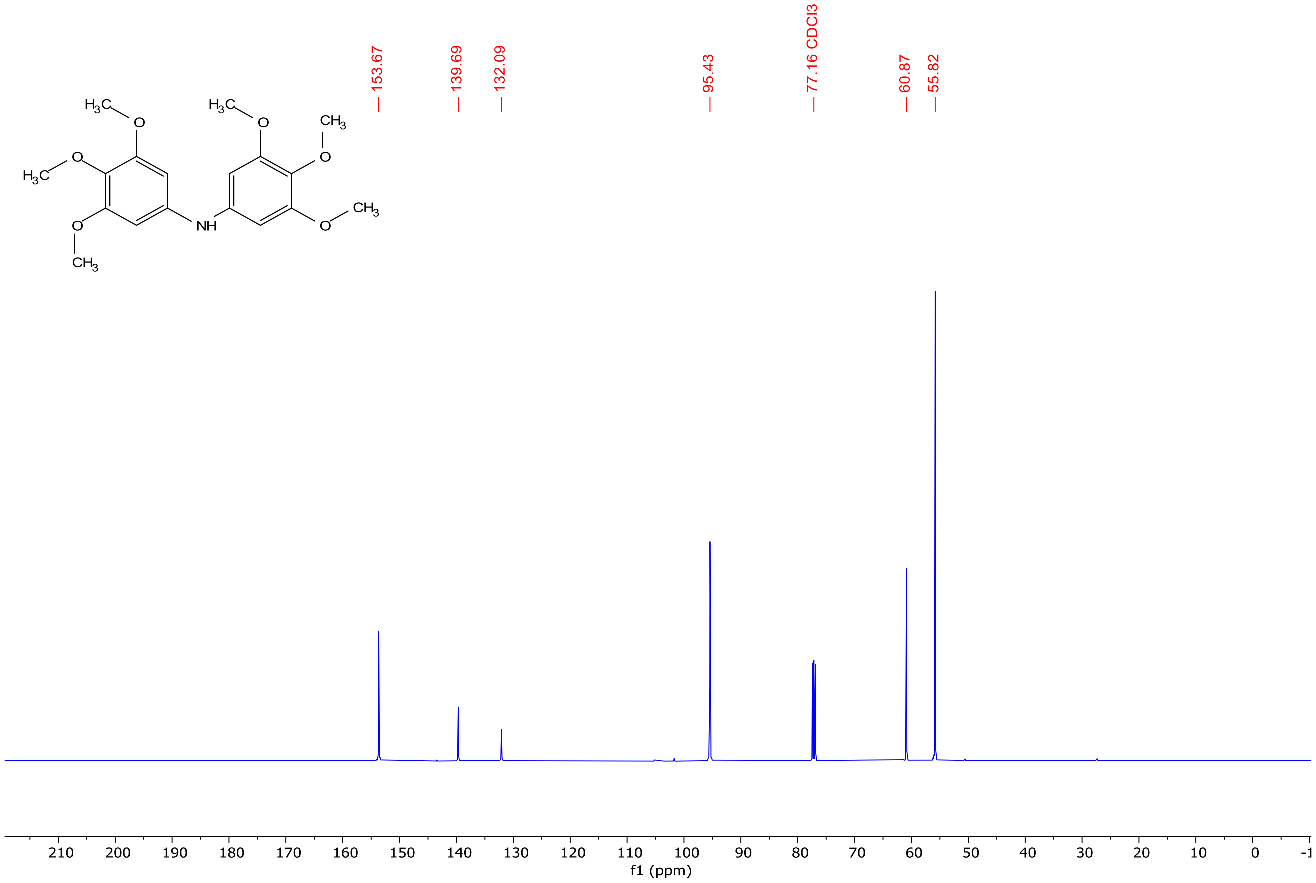

MeOPhNHAr[1]--- *N*-(4-methoxyphenyl)-3,5-bis(trifluoromethyl)aniline

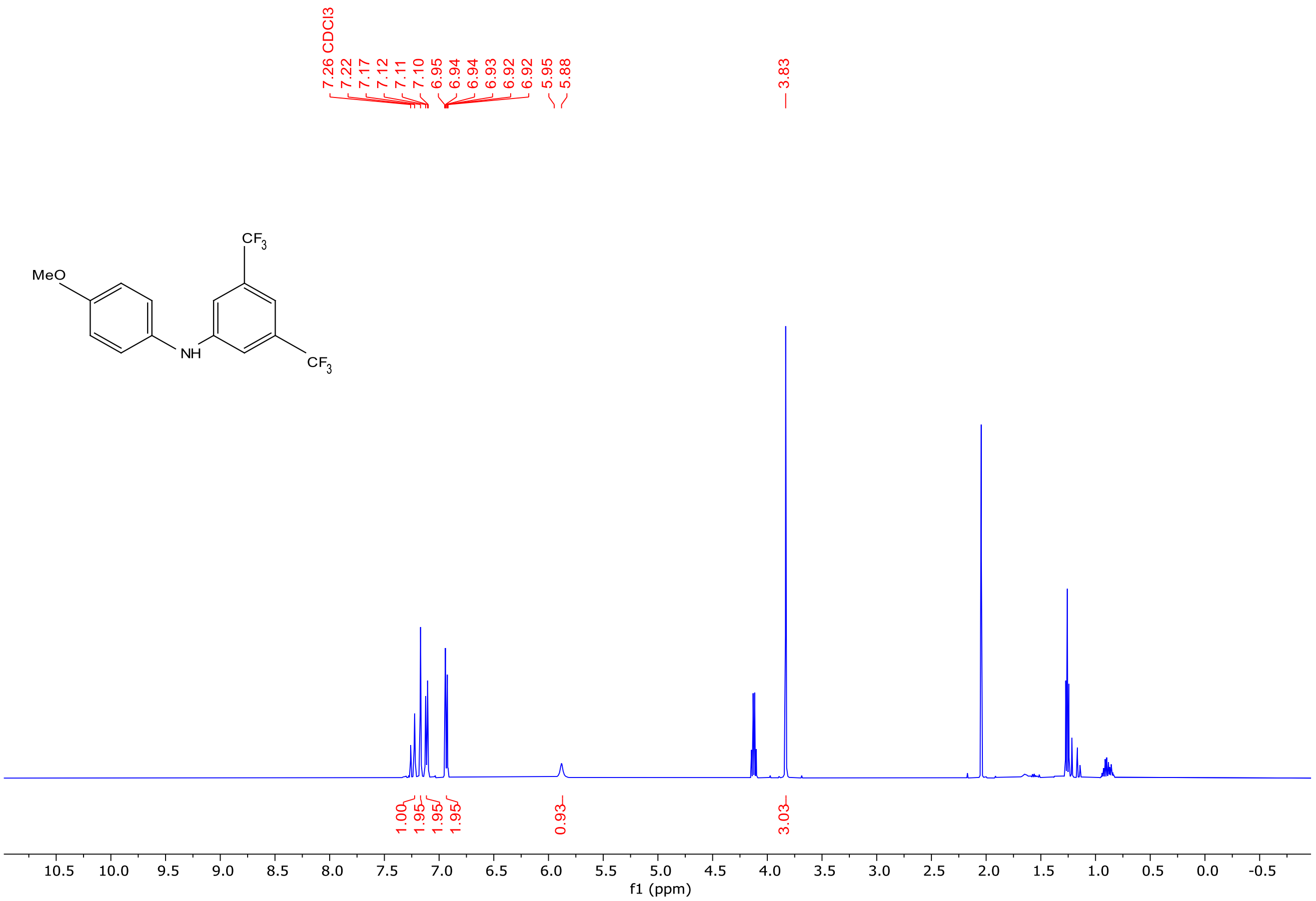

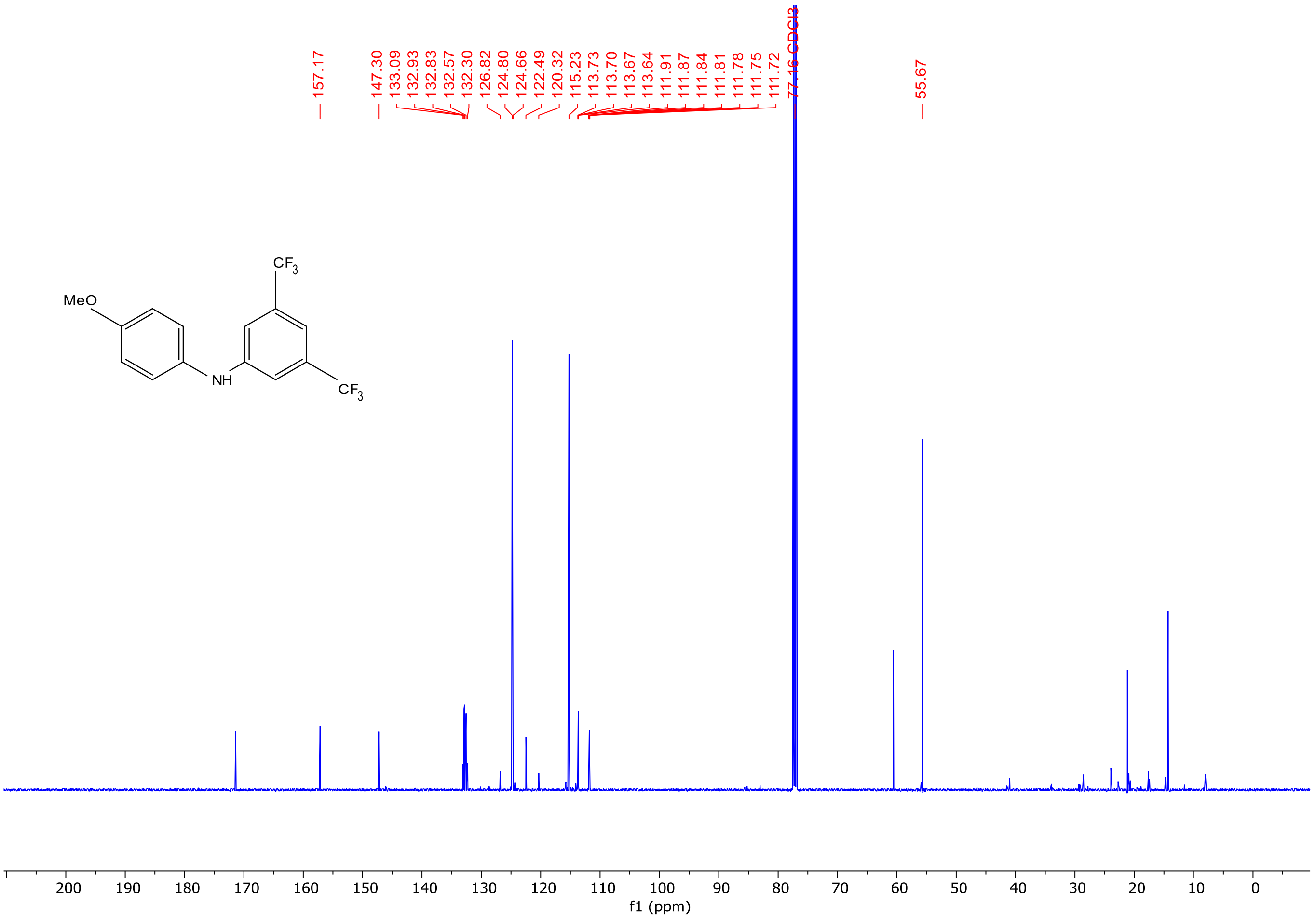

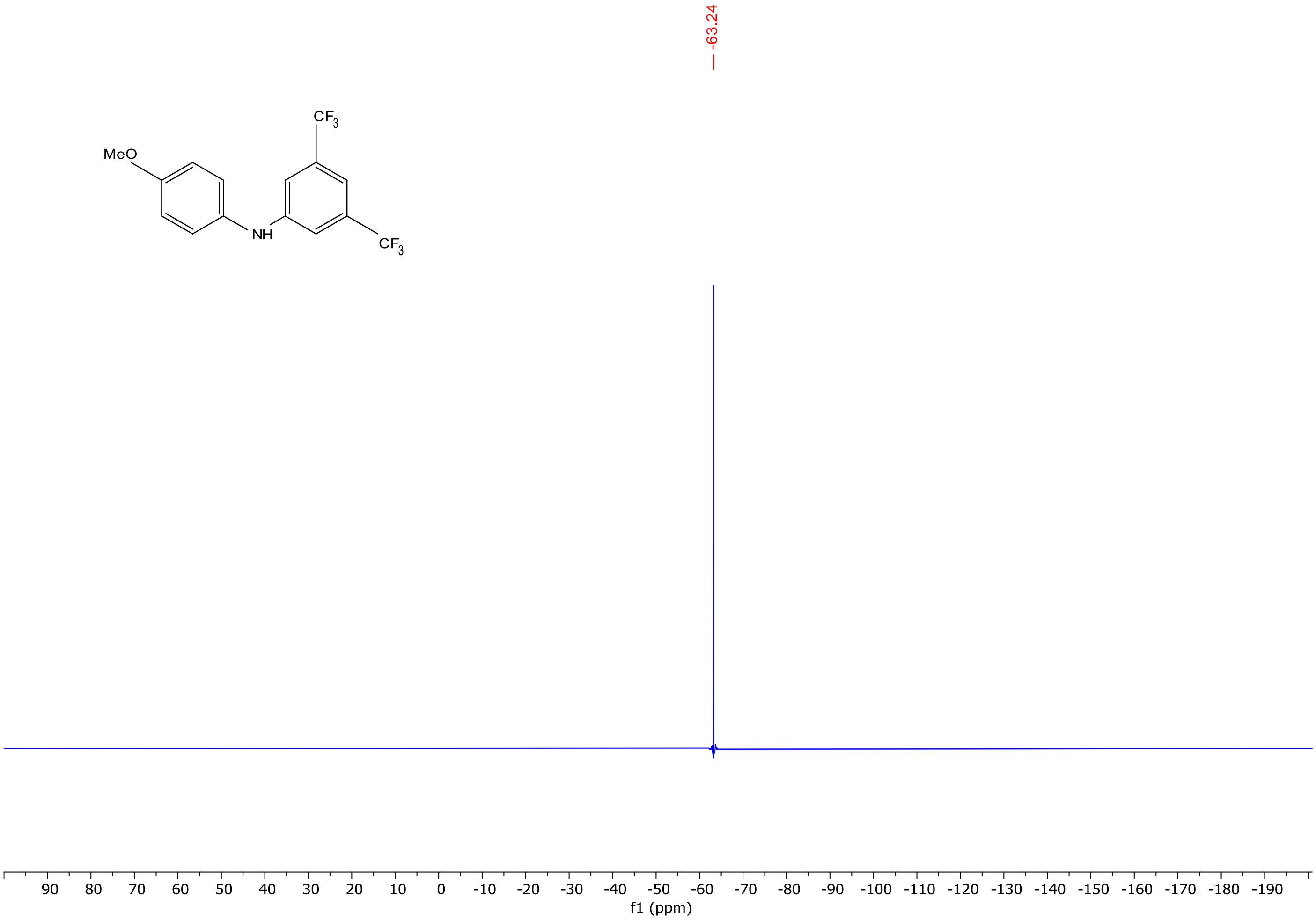
-63.24
MeO
NH
CF3
CF3
90 80 70 60 50 40 30 20 10 0 -10 -20 -30 -40 -50 -60 -70 -80 -90 -100 -110 -120 -130 -140 -150 -160 -170 -180 -190
f1 (ppm)

MeOPhNHAr^2--- 4-((4-methoxyphenyl)amino)benzonitrile

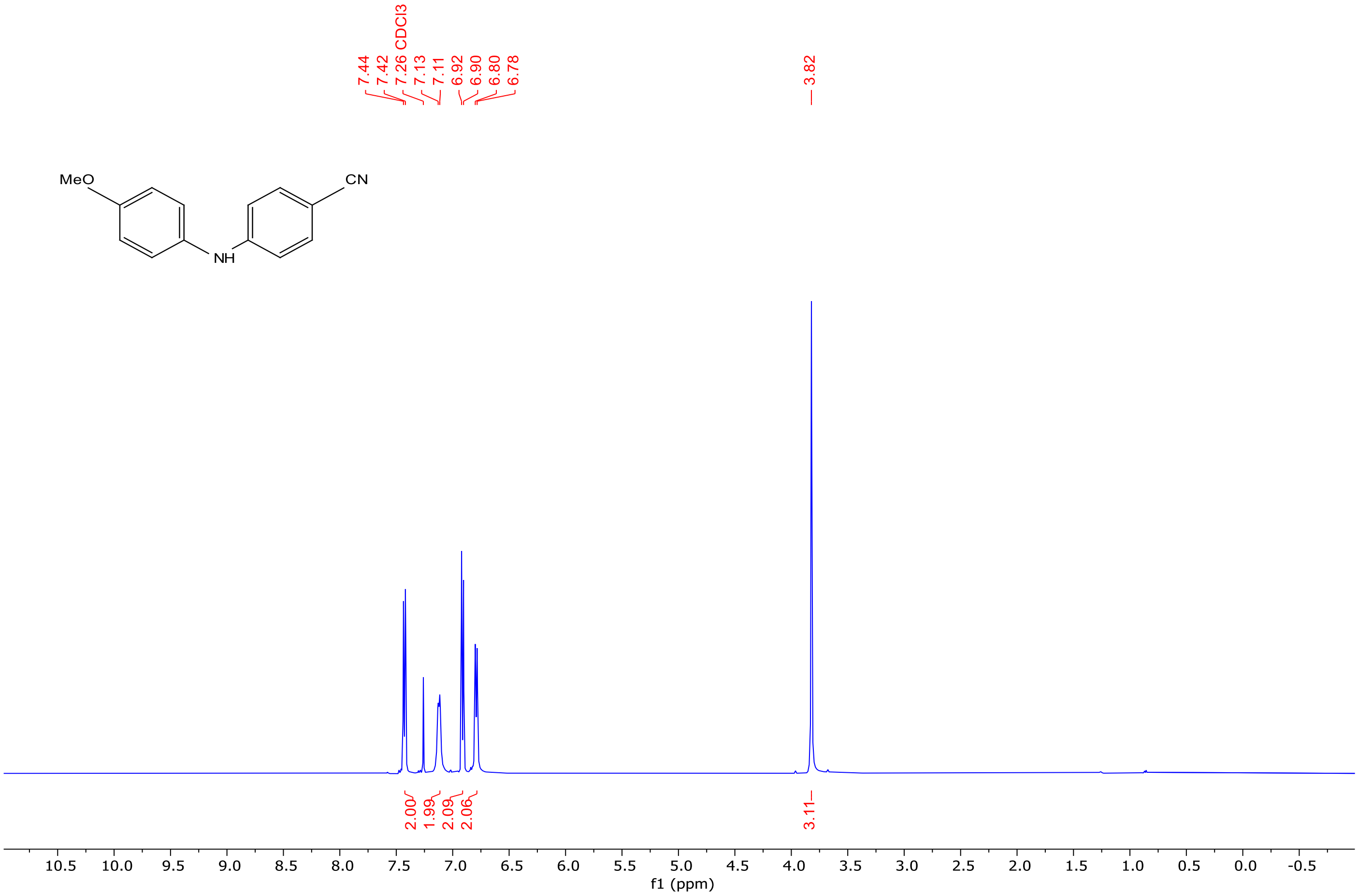

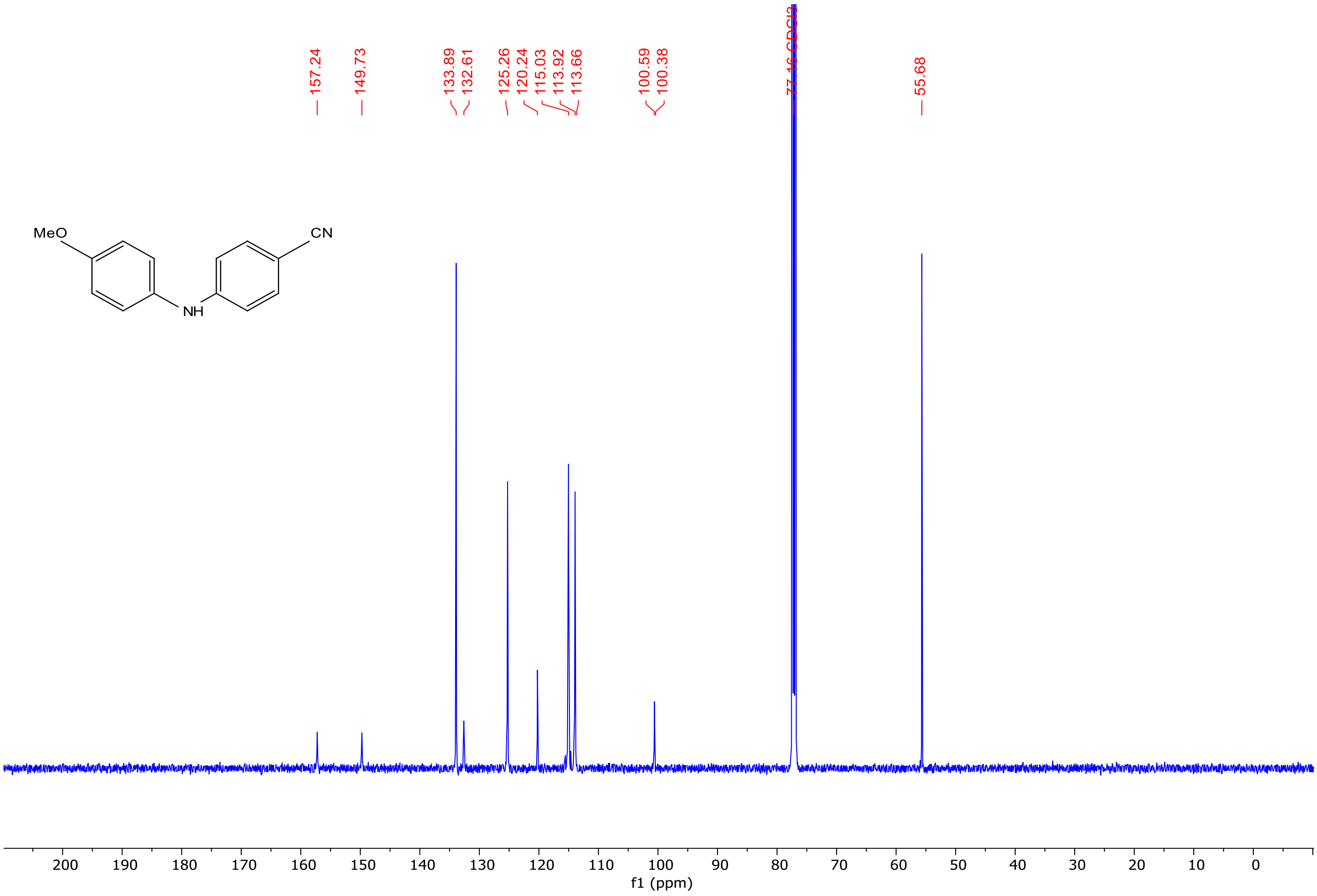

Ar^{1}Cz--- 3,6-bis(3,5-bis(trifluoromethyl)phenyl)-9*H*-carbazole

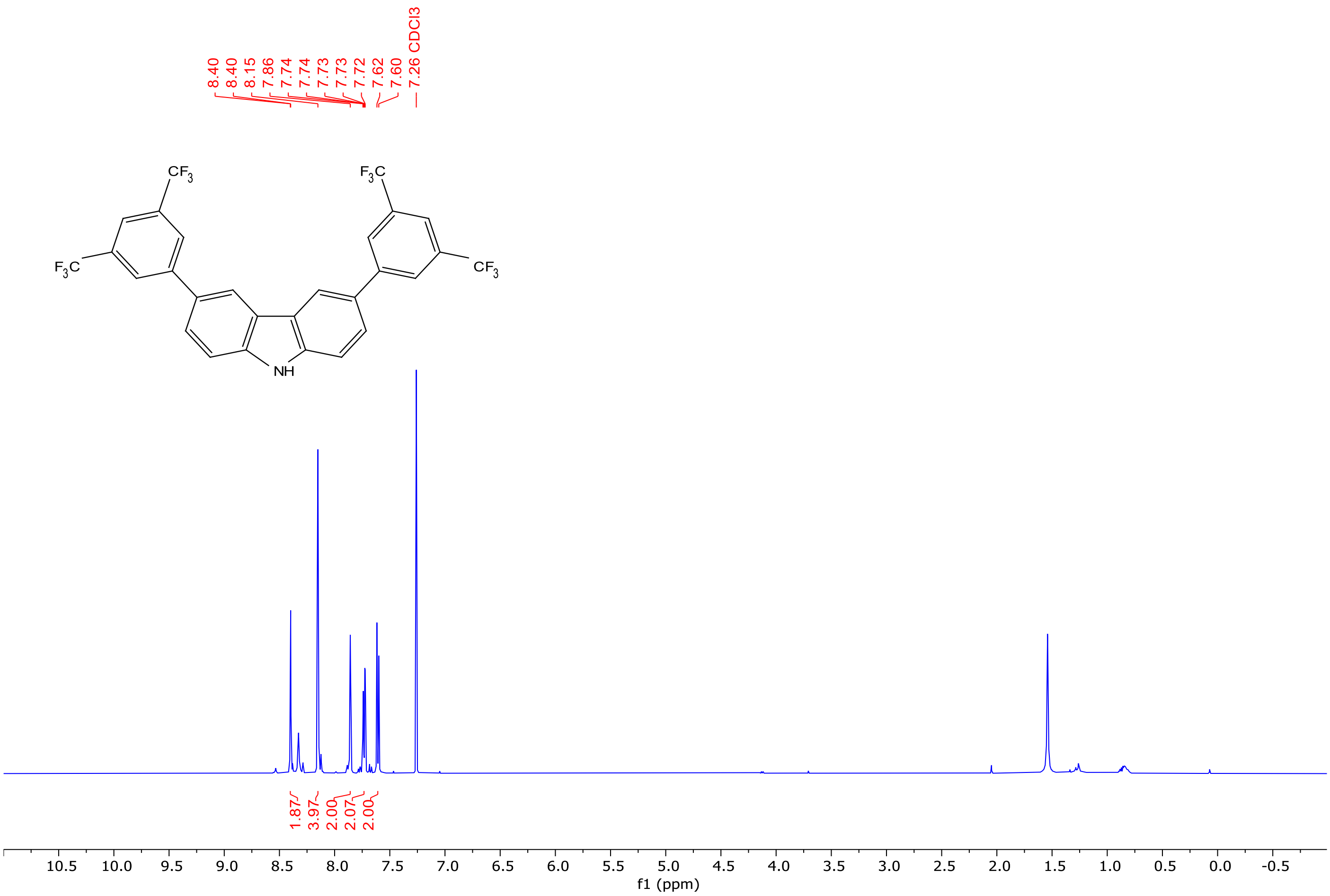

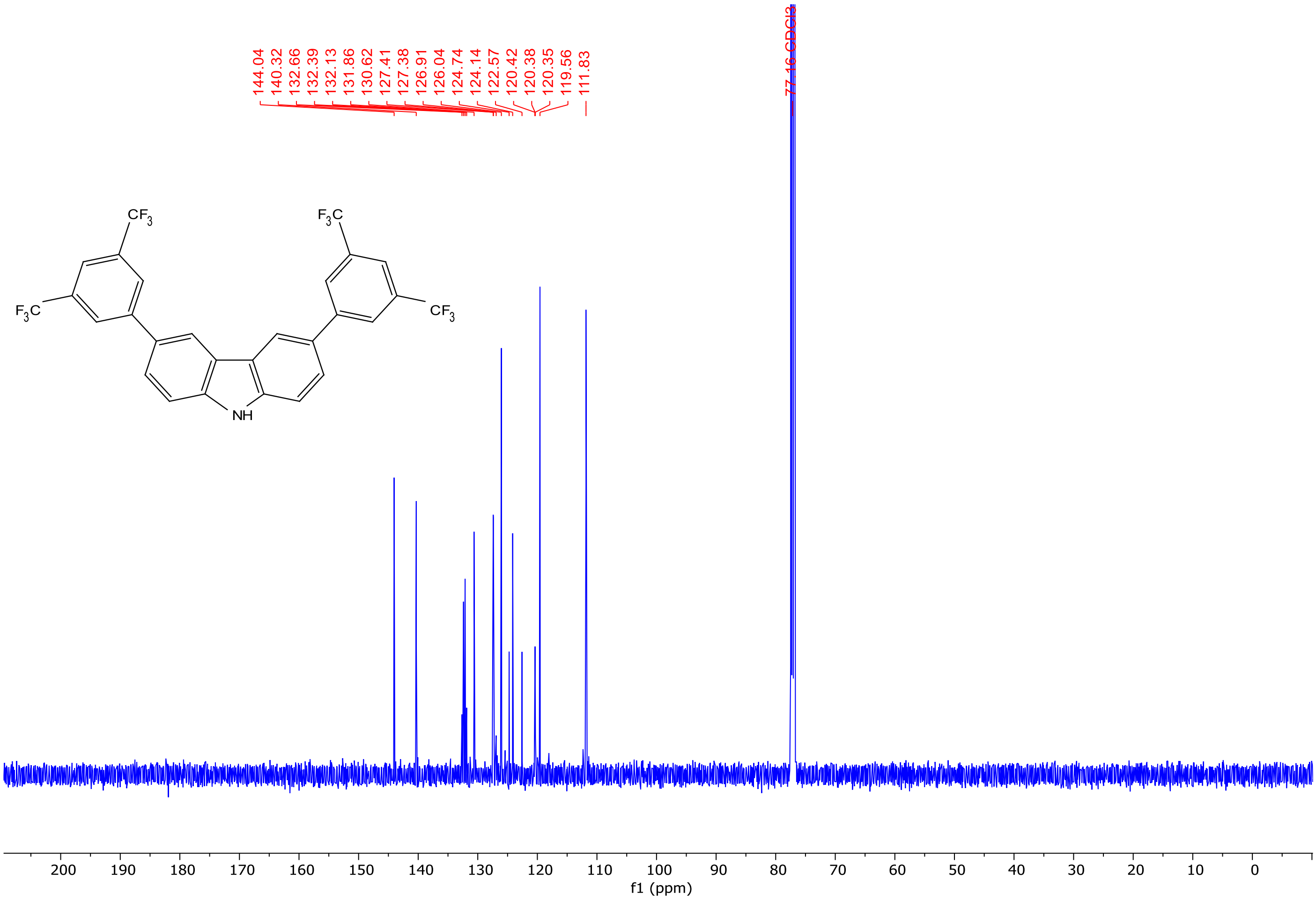

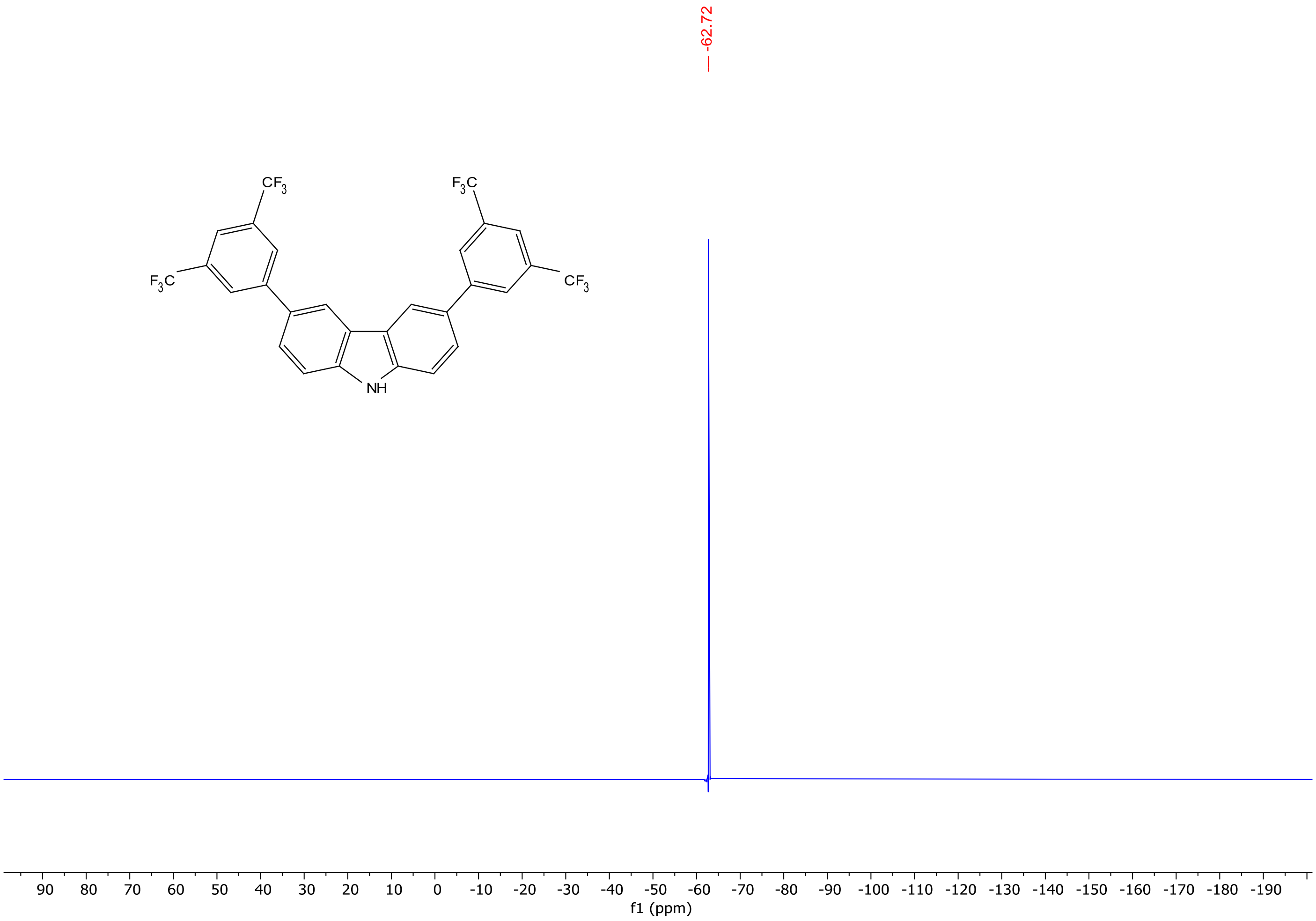
-62.72
CF3
F3C
F3C
CF3
NH
90 80 70 60 50 40 30 20 10 0 -10 -20 -30 -40 -50 -60 -70 -80 -90 -100 -110 -120 -130 -140 -150 -160 -170 -180 -190
f1 (ppm)

Ar^{2}Cz---4,4'-(9*H*-carbazole-3,6-diyl)dibenzonitrile

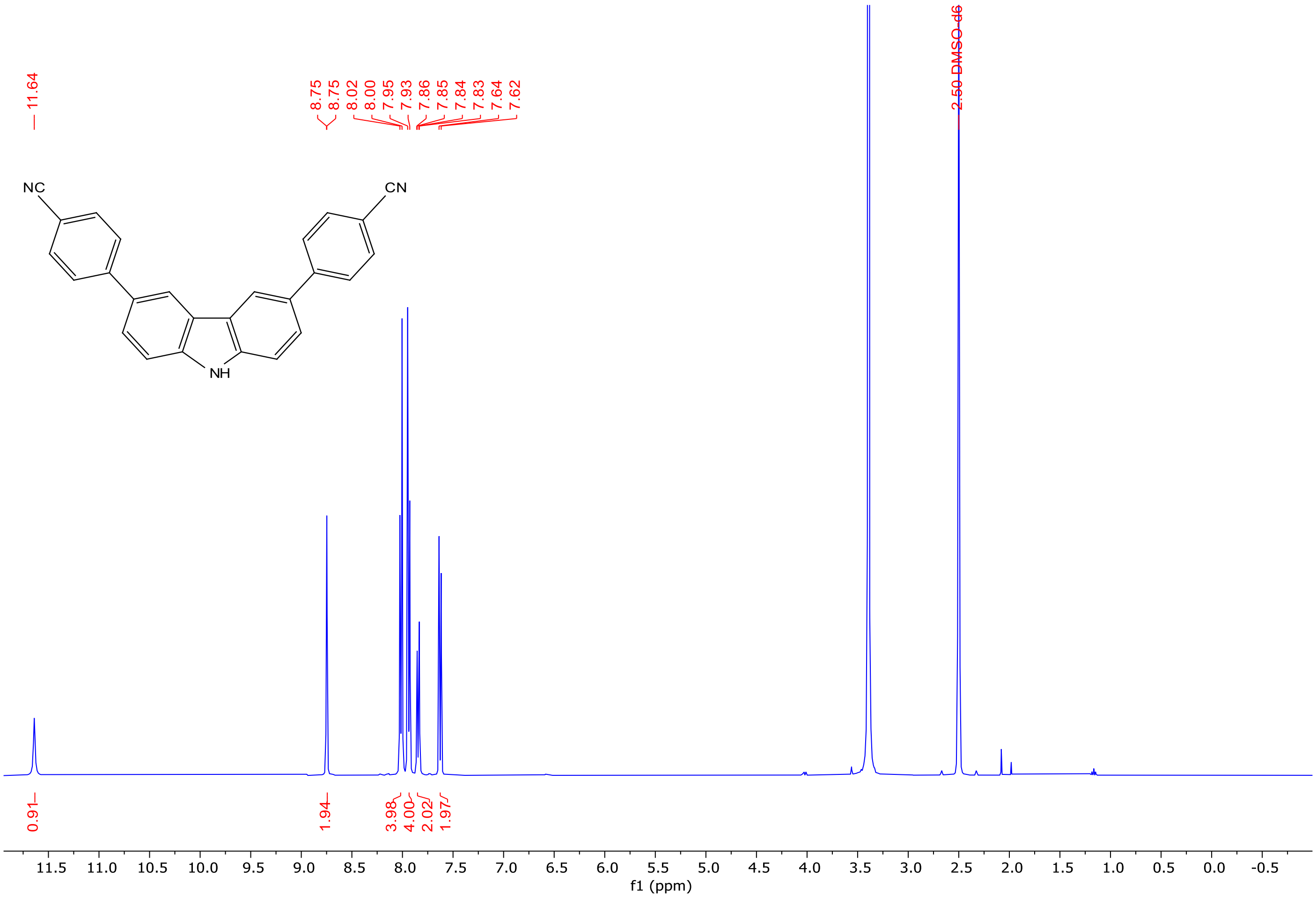

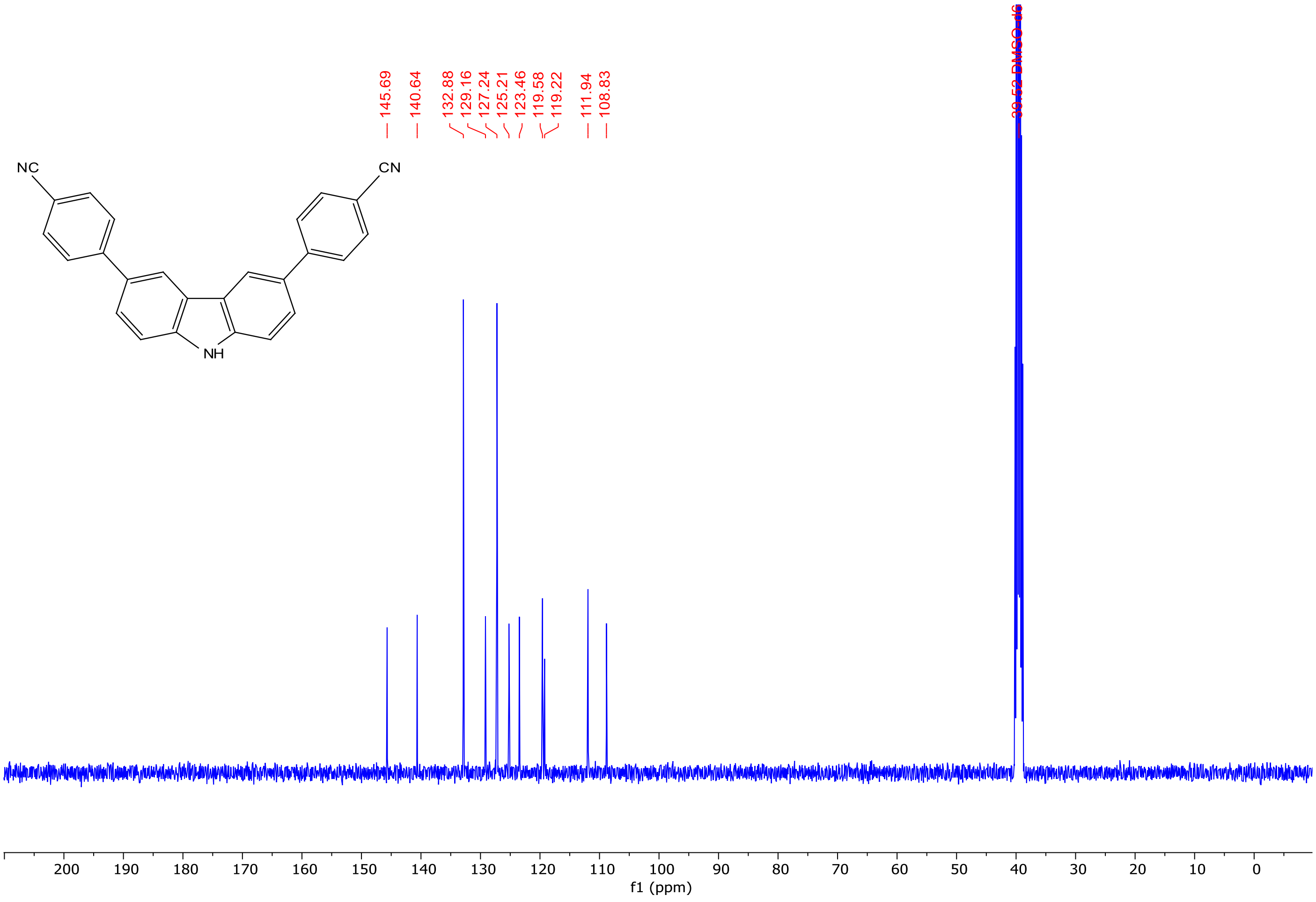

Ar³Cz--- 3,6-bis(4-(trifluoromethyl)phenyl)-9*H*-carbazole

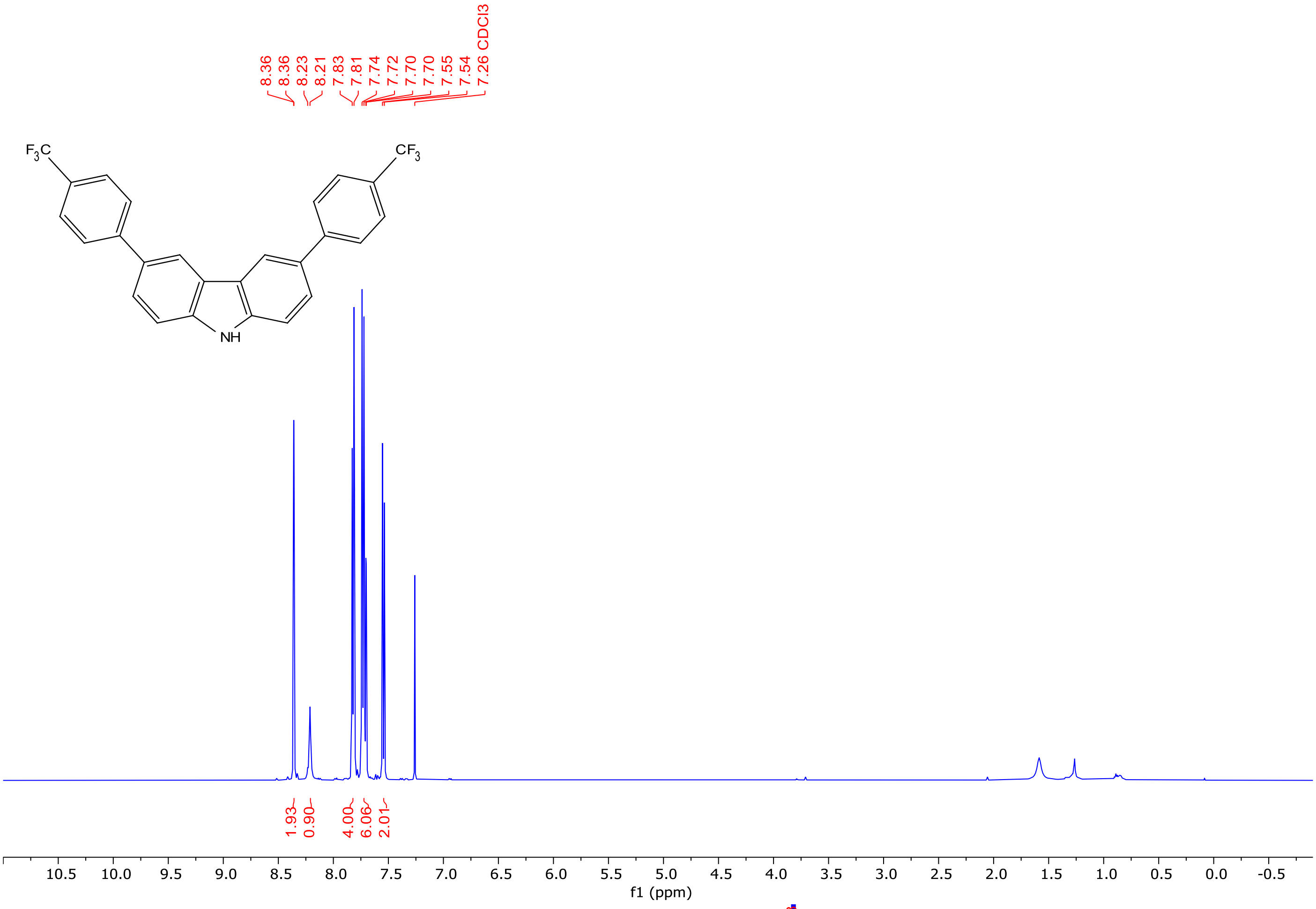

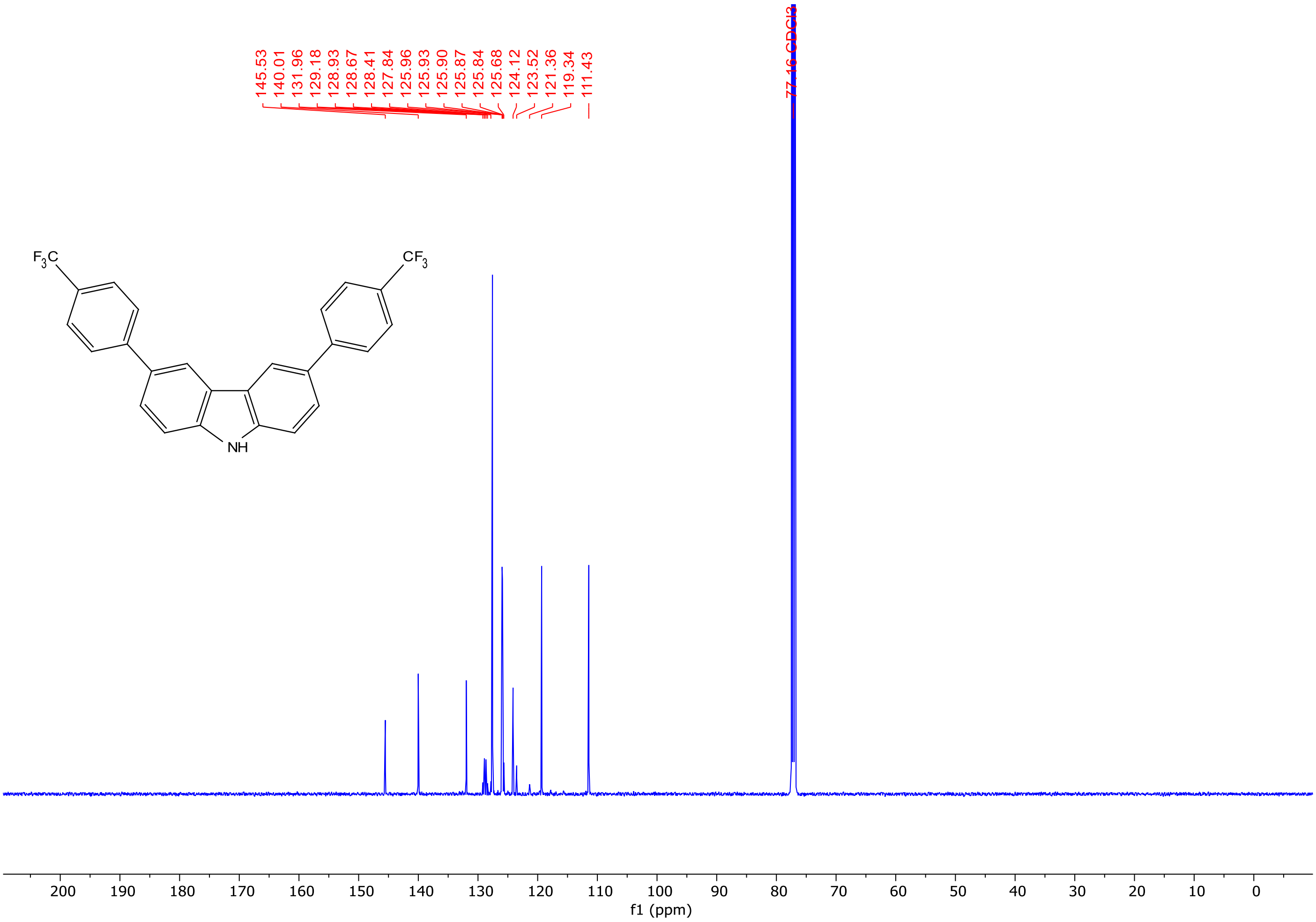

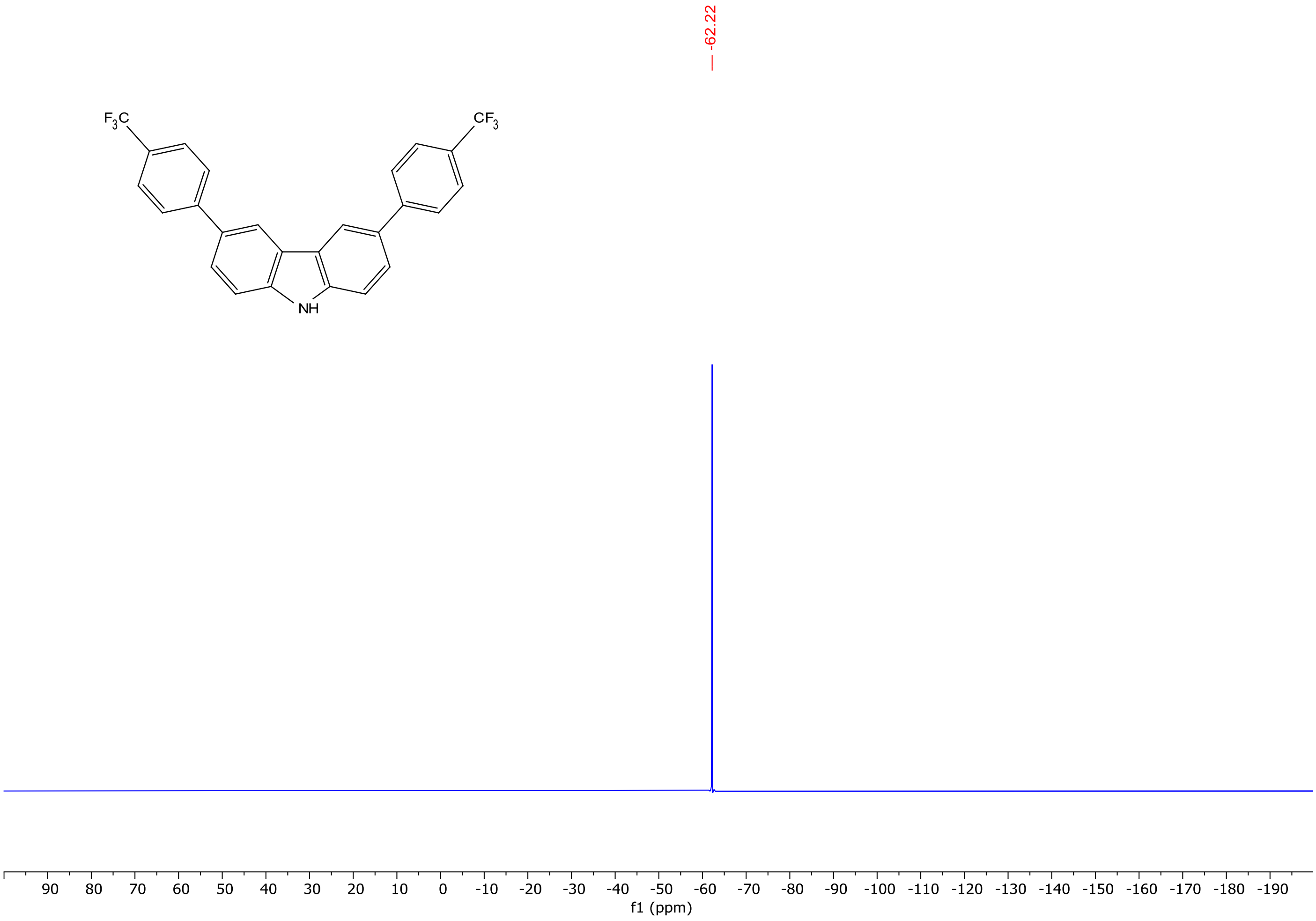
-62.22
F_3C
CF_3
NH
90 80 70 60 50 40 30 20 10 0 -10 -20 -30 -40 -50 -60 -70 -80 -90 -100 -110 -120 -130 -140 -150 -160 -170 -180 -190
f1 (ppm)

Ar[4]PhDPA----bis(3',5'-dimethoxy-[1,1'-biphenyl]-4-yl)amine

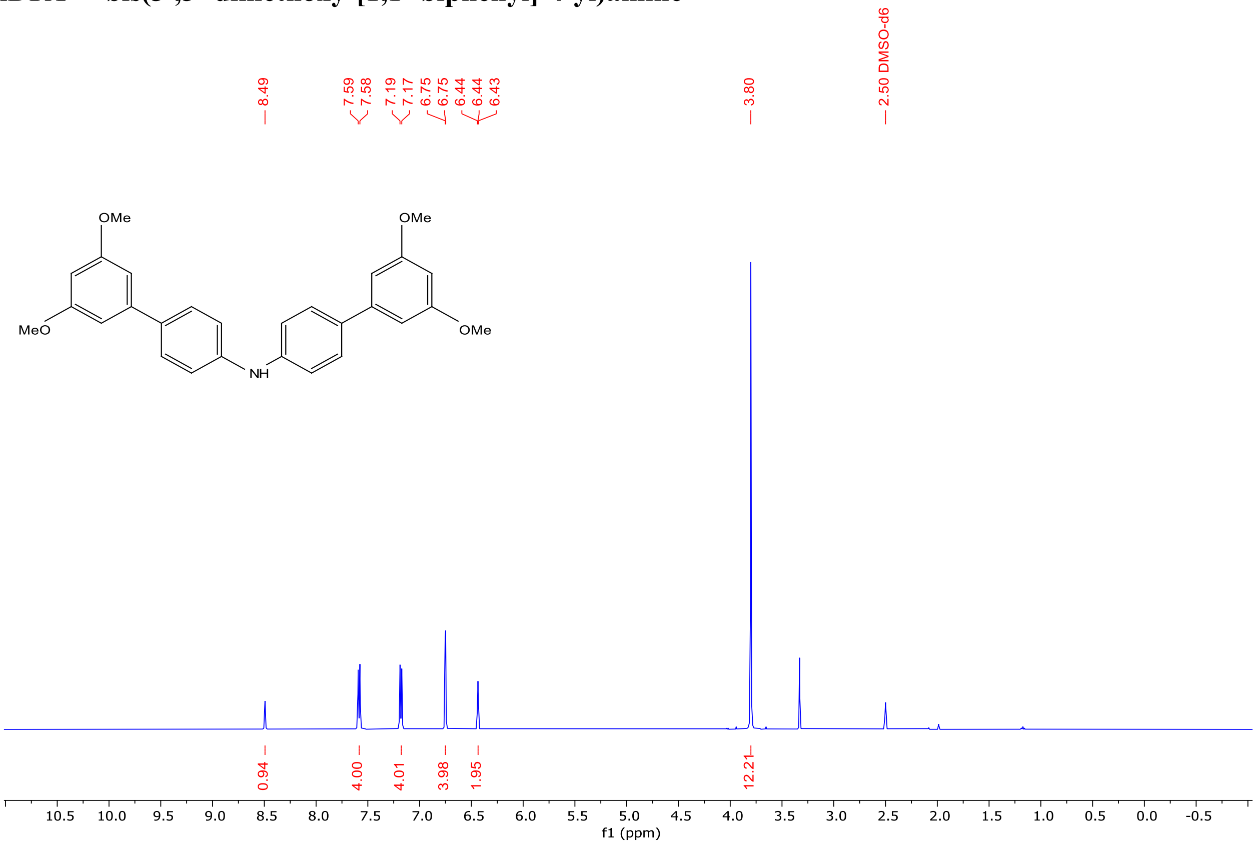

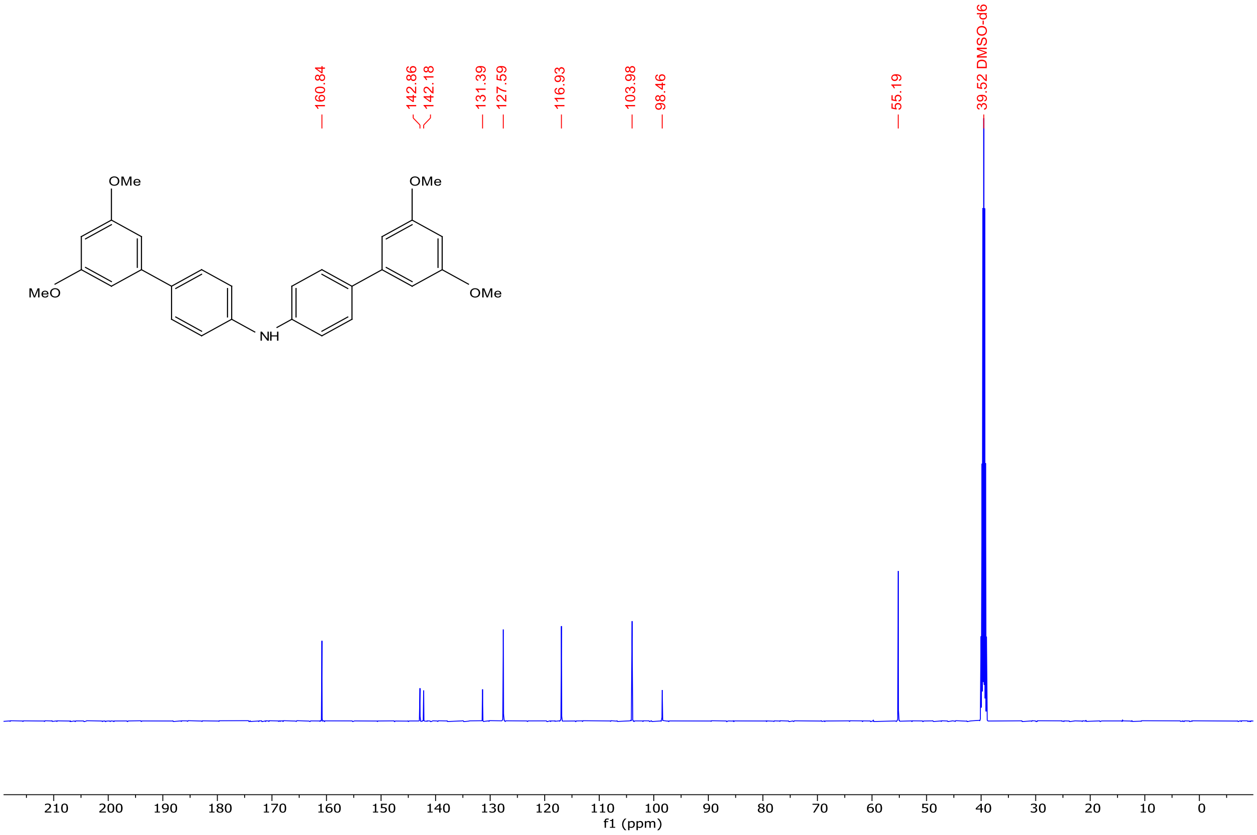

Ar⁵PhDPA---bis(3',4',5'-trimethoxy-[1,1'-biphenyl]-4-yl)amine

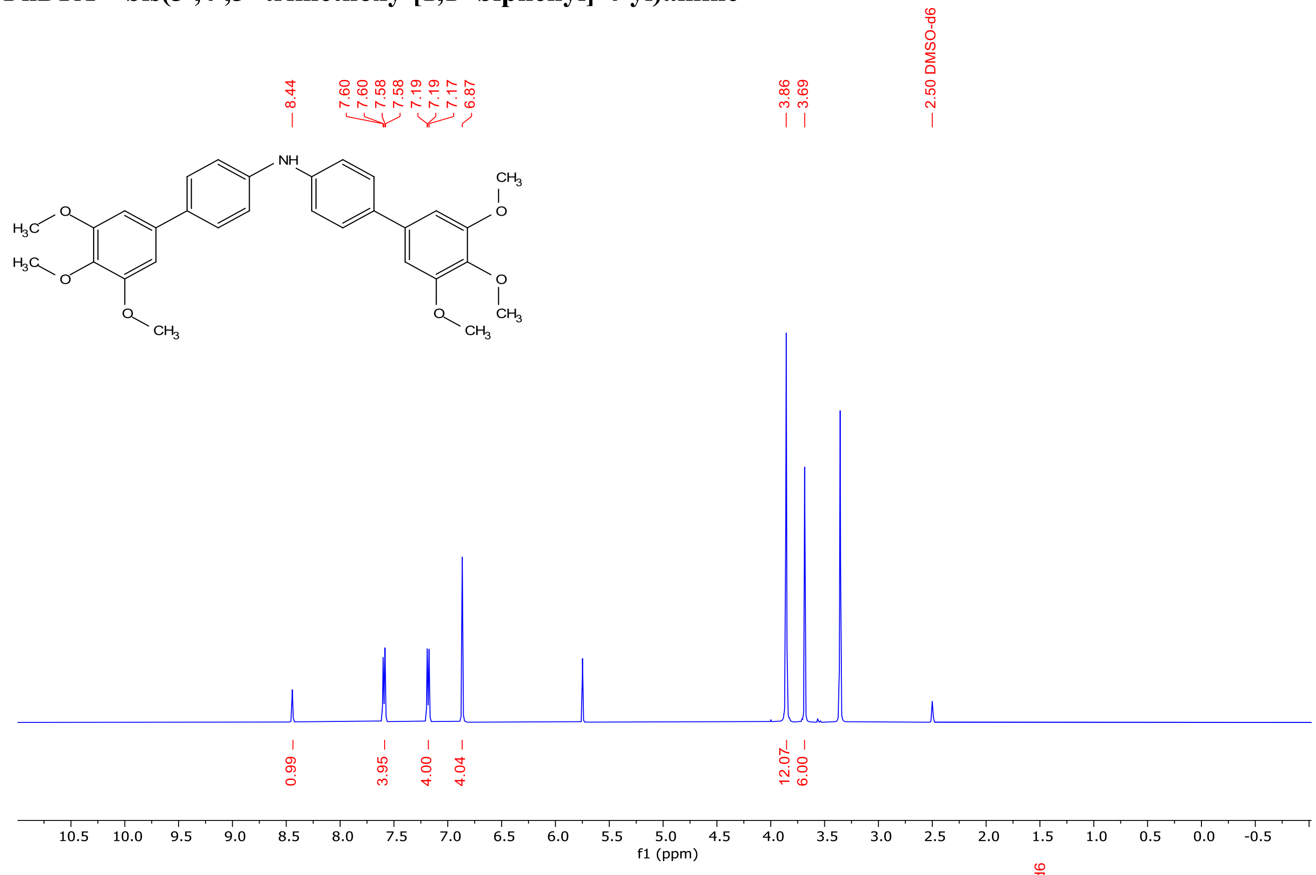

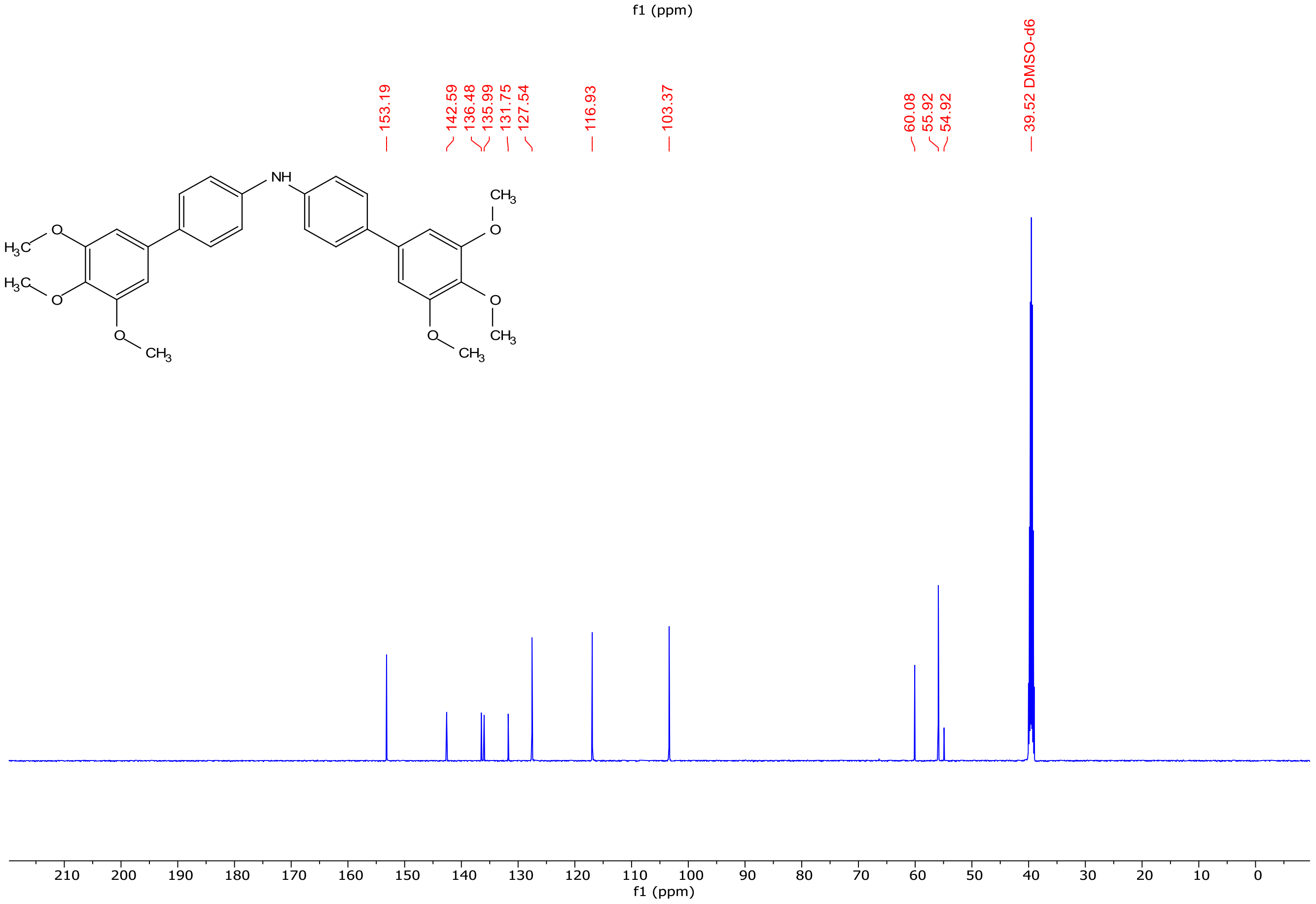

Ar^{6}PhDPA---bis(4'-methoxy-3',5'-dimethyl-[1,1'-biphenyl]-4-yl)amine

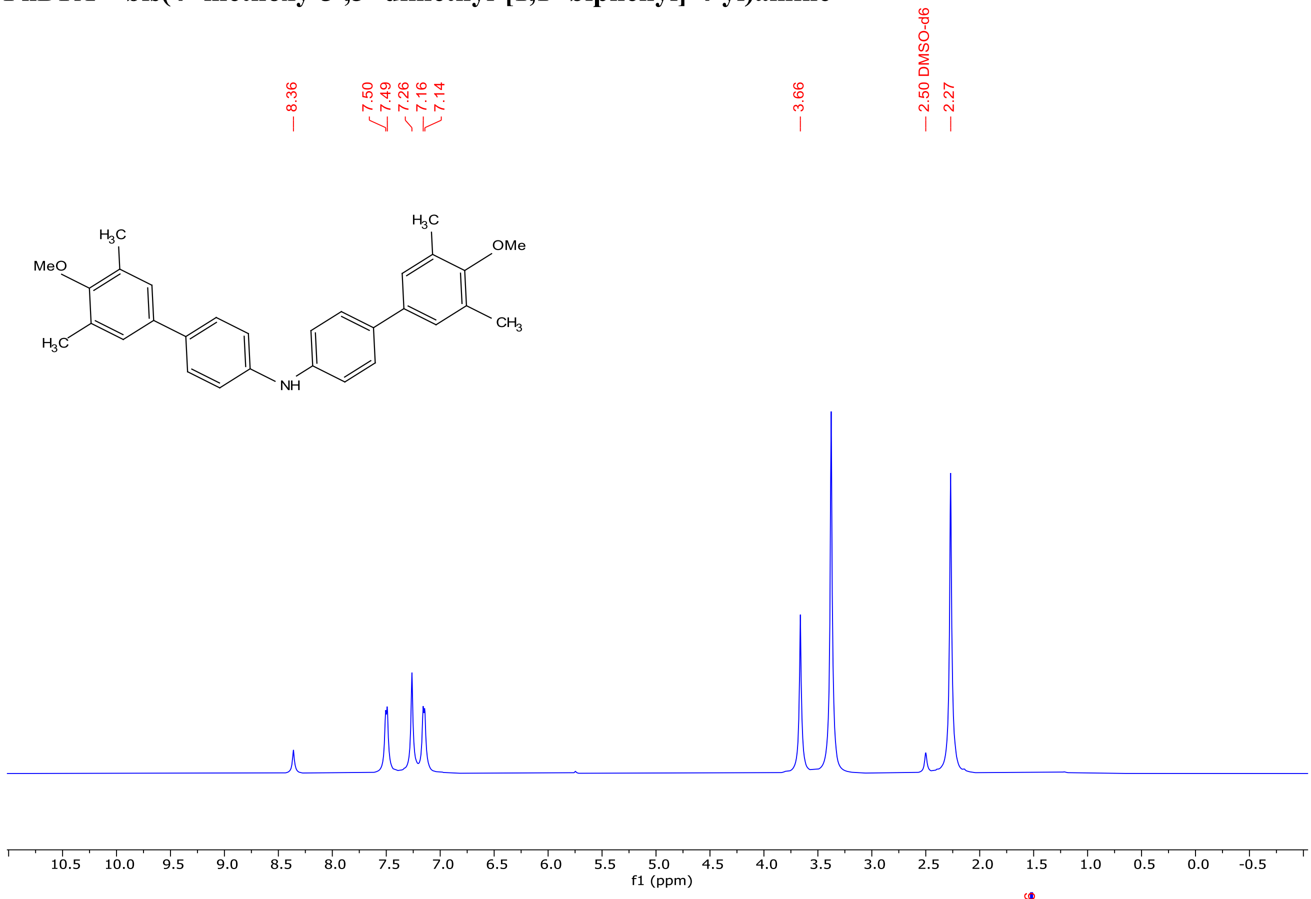

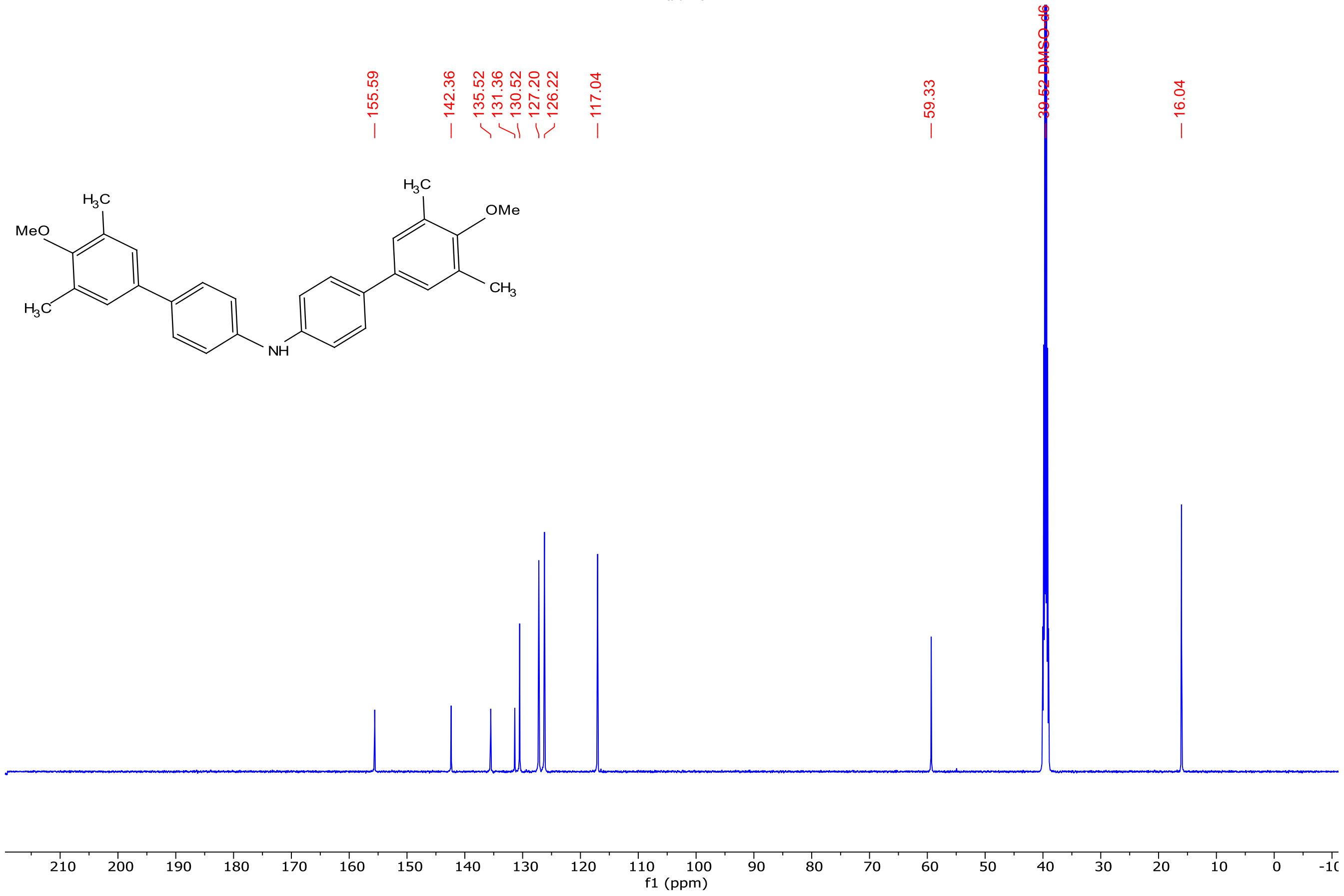

4CzIPN---2,4,5,6-tetra(9*H*-carbazol-9-yl)isophthalonitrile) (PC1)

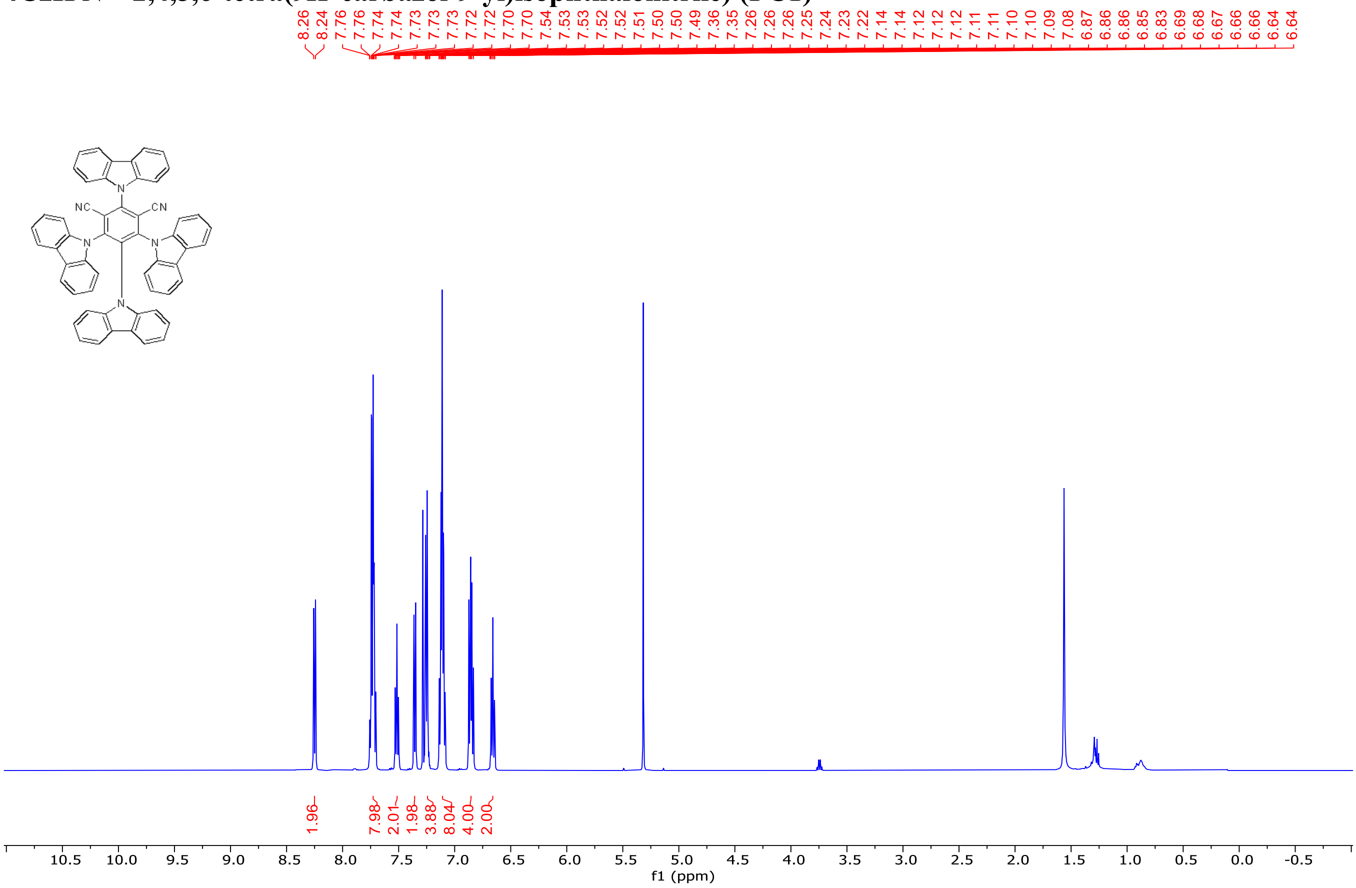

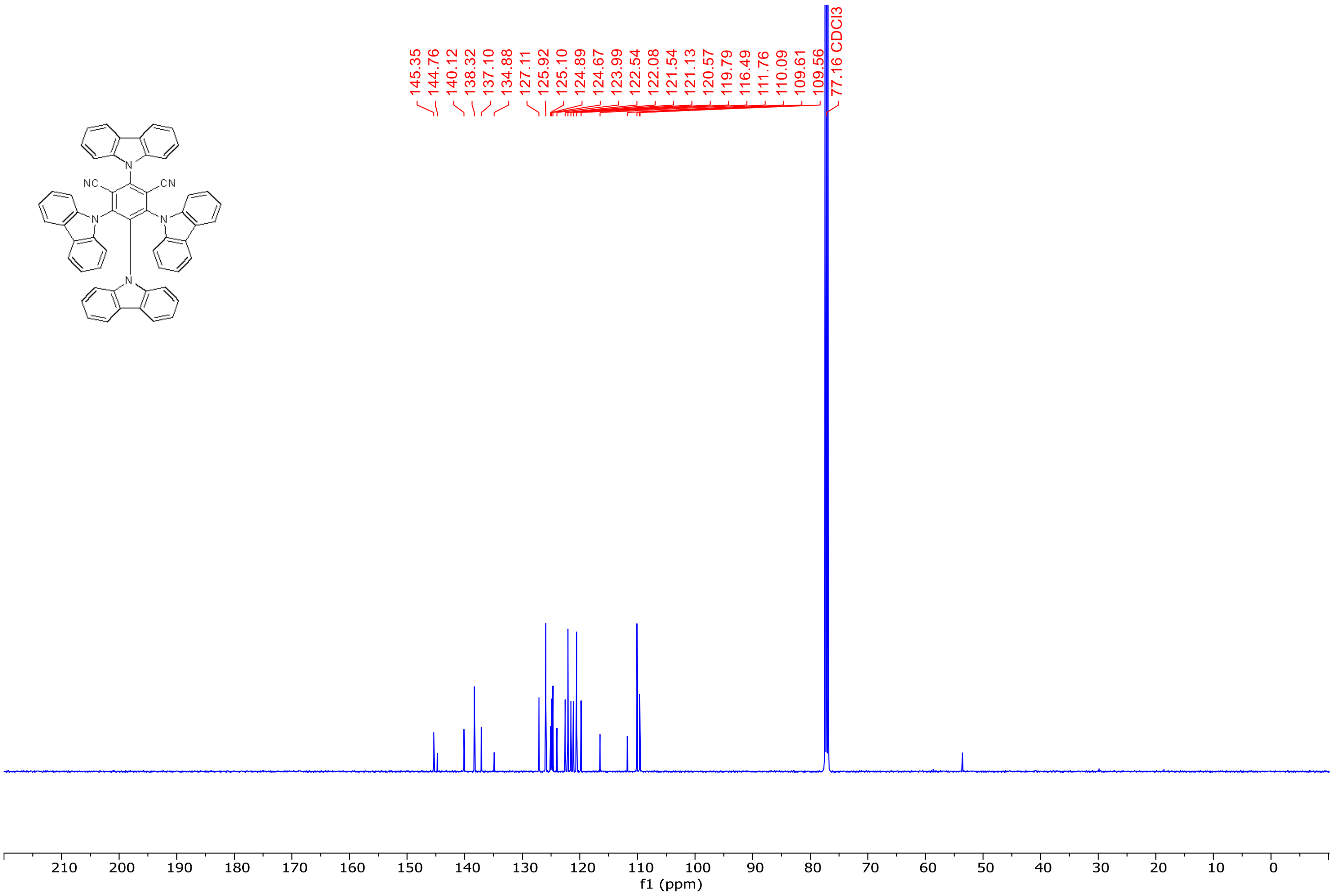

4PhCzIPN---2,4,5,6-tetrakis(3,6-diphenyl-9*H*-carbazol-9-yl)isophthalonitrile (PC2)

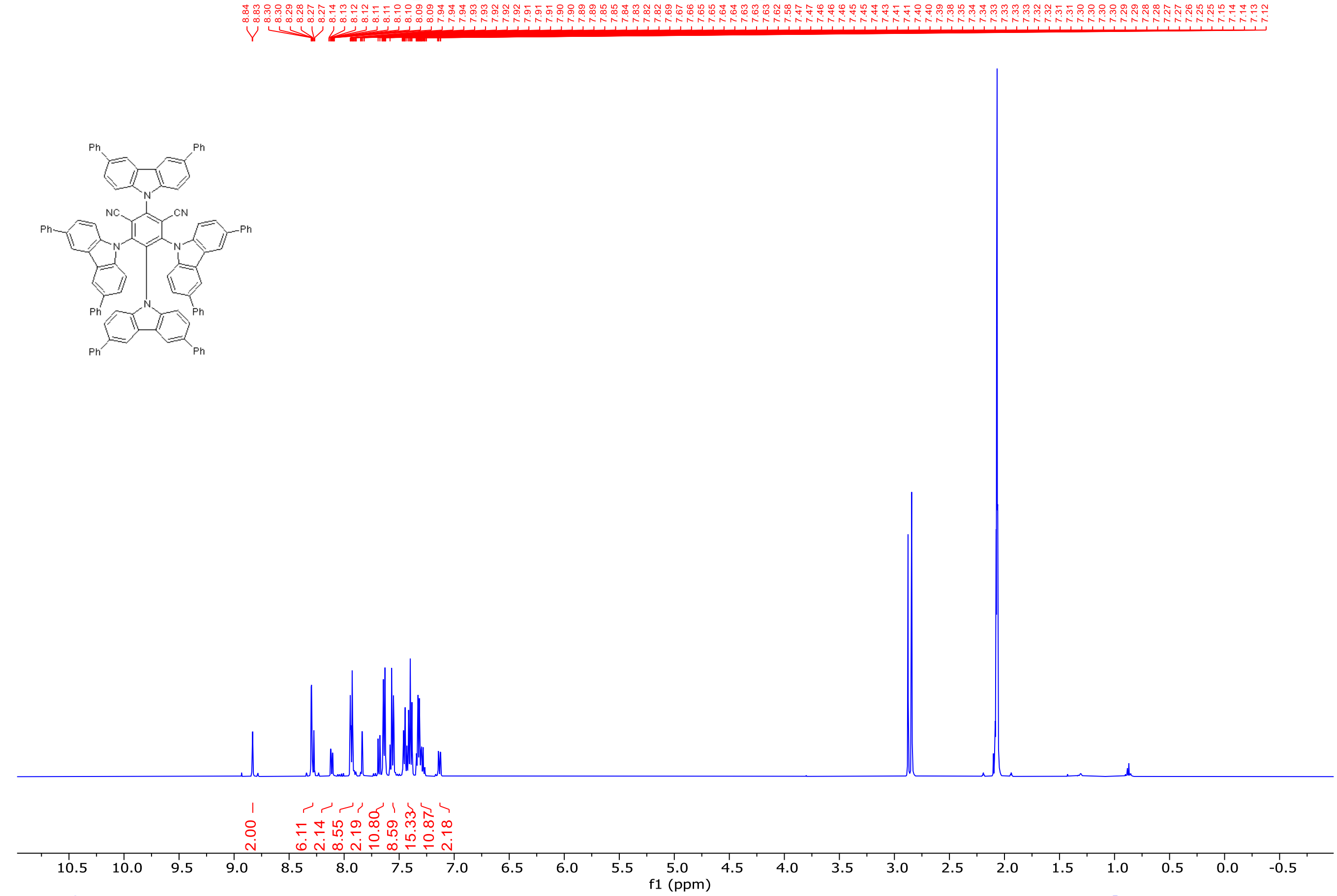

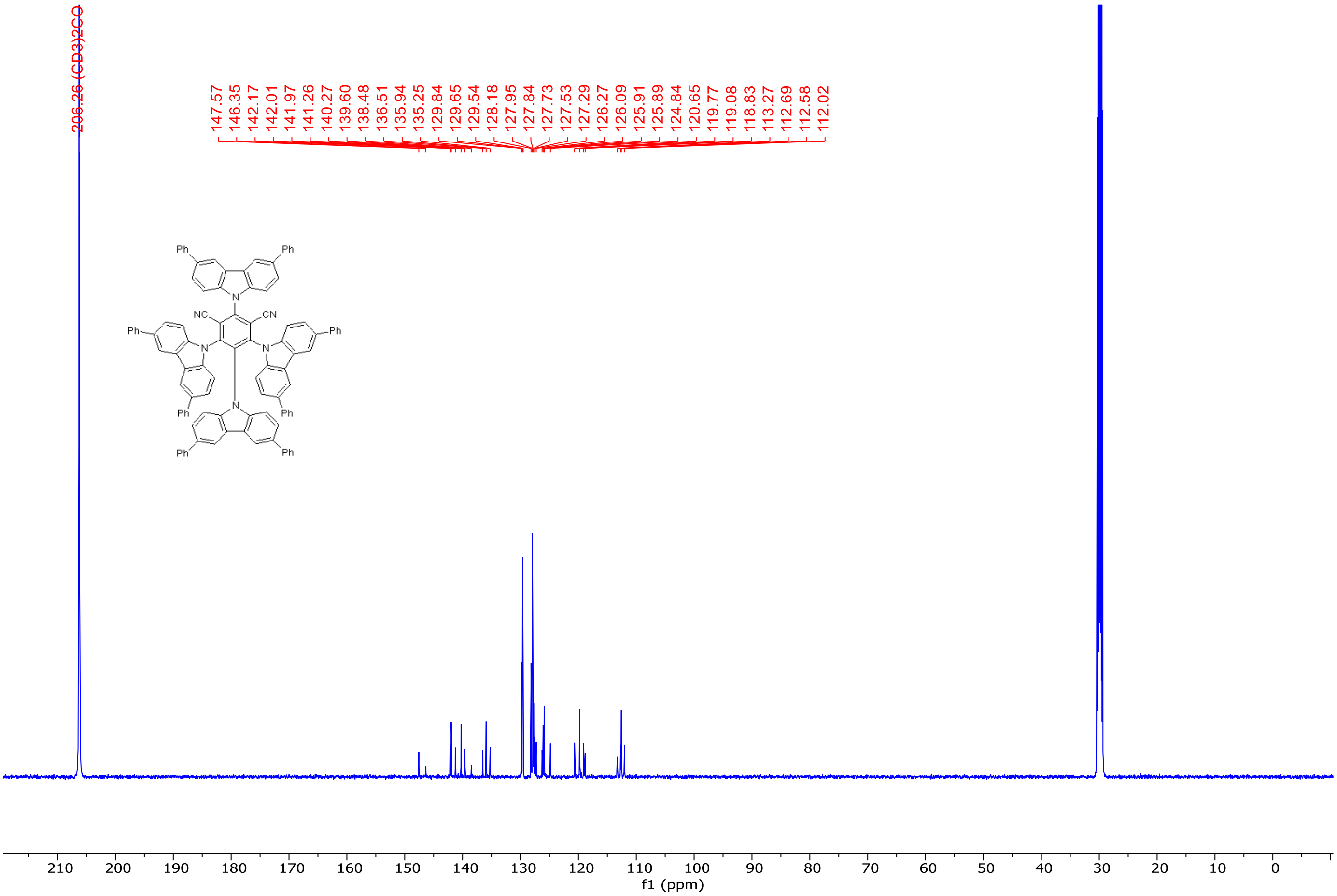

4^{t}BuCzIPN---2,4,5,6-tetrakis(3,6-di-*tert*-butyl-9*H*-carbazol-9-yl)isophthalonitrile (PC3)

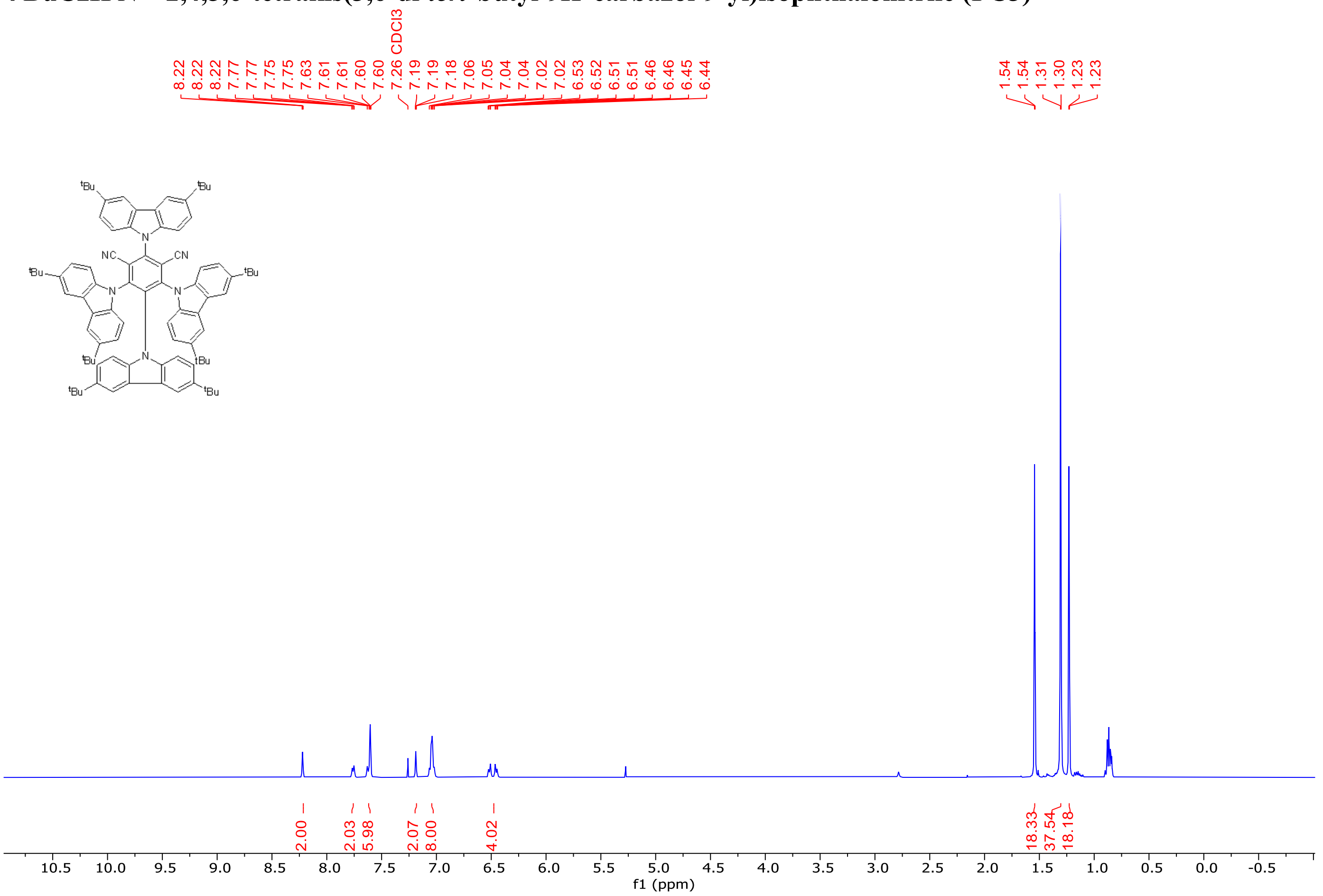

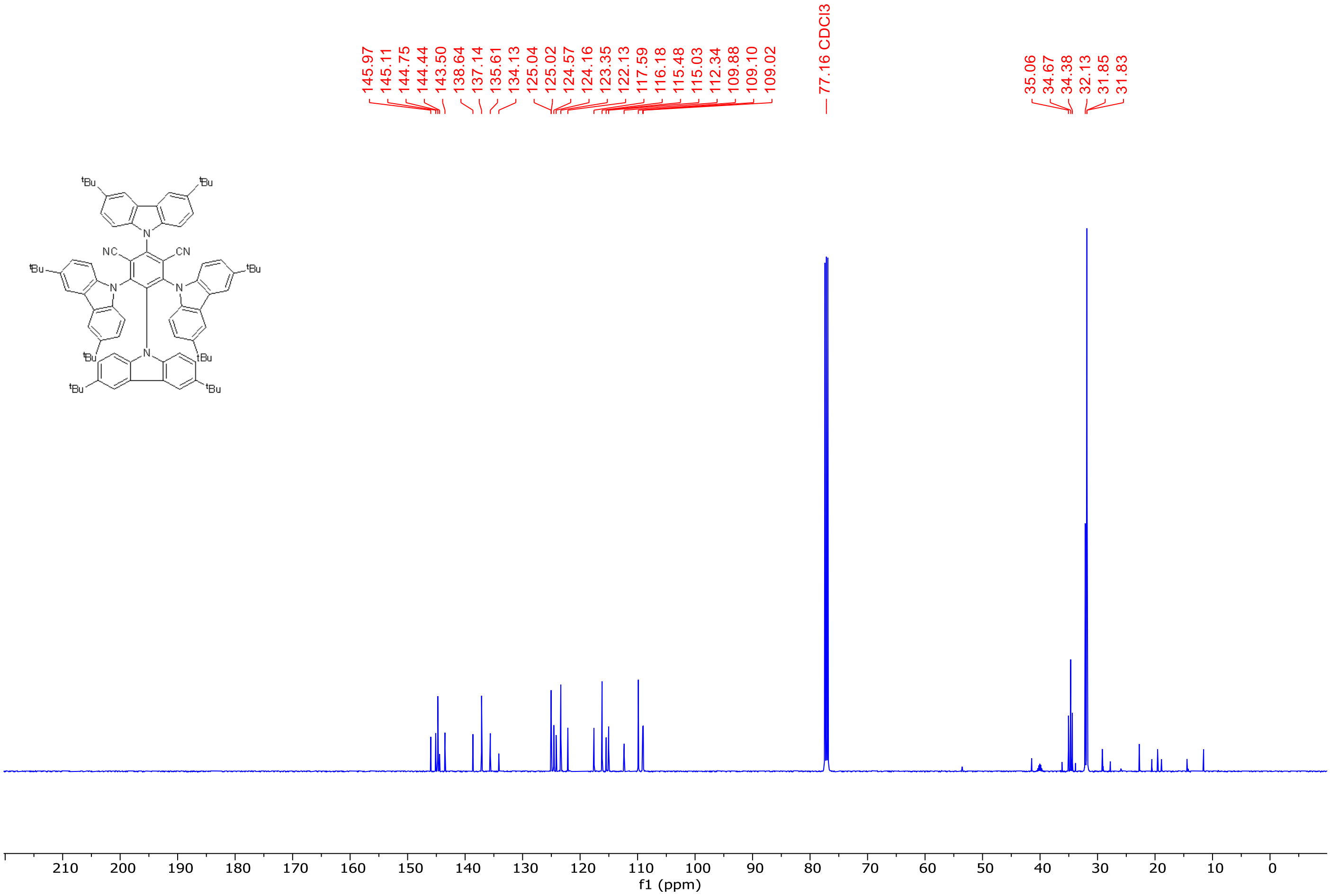

4BrDPAIPN---2,4,5,6-tetrakis(bis(4-bromophenyl)amino)isophthalonitrile (PC4)

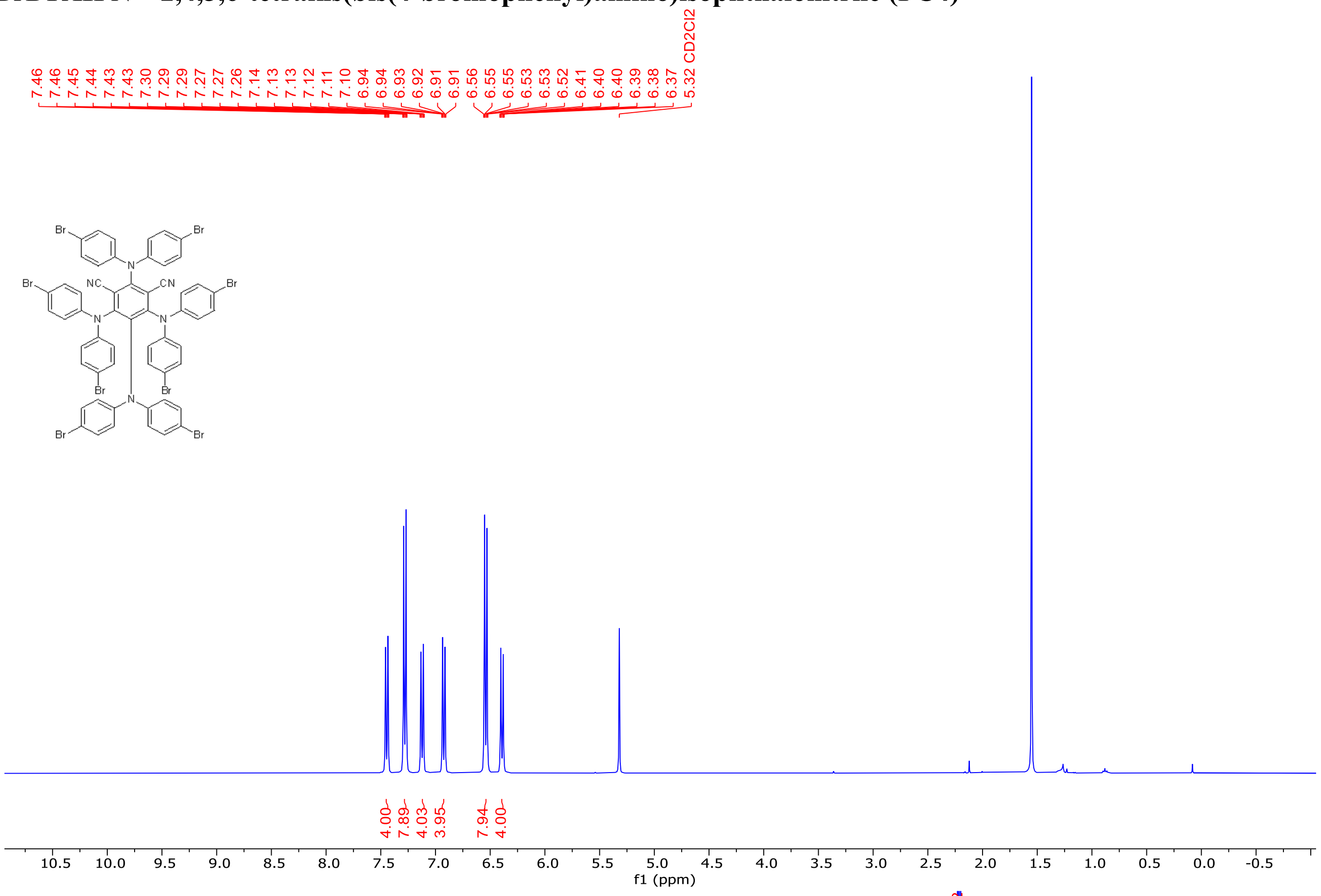

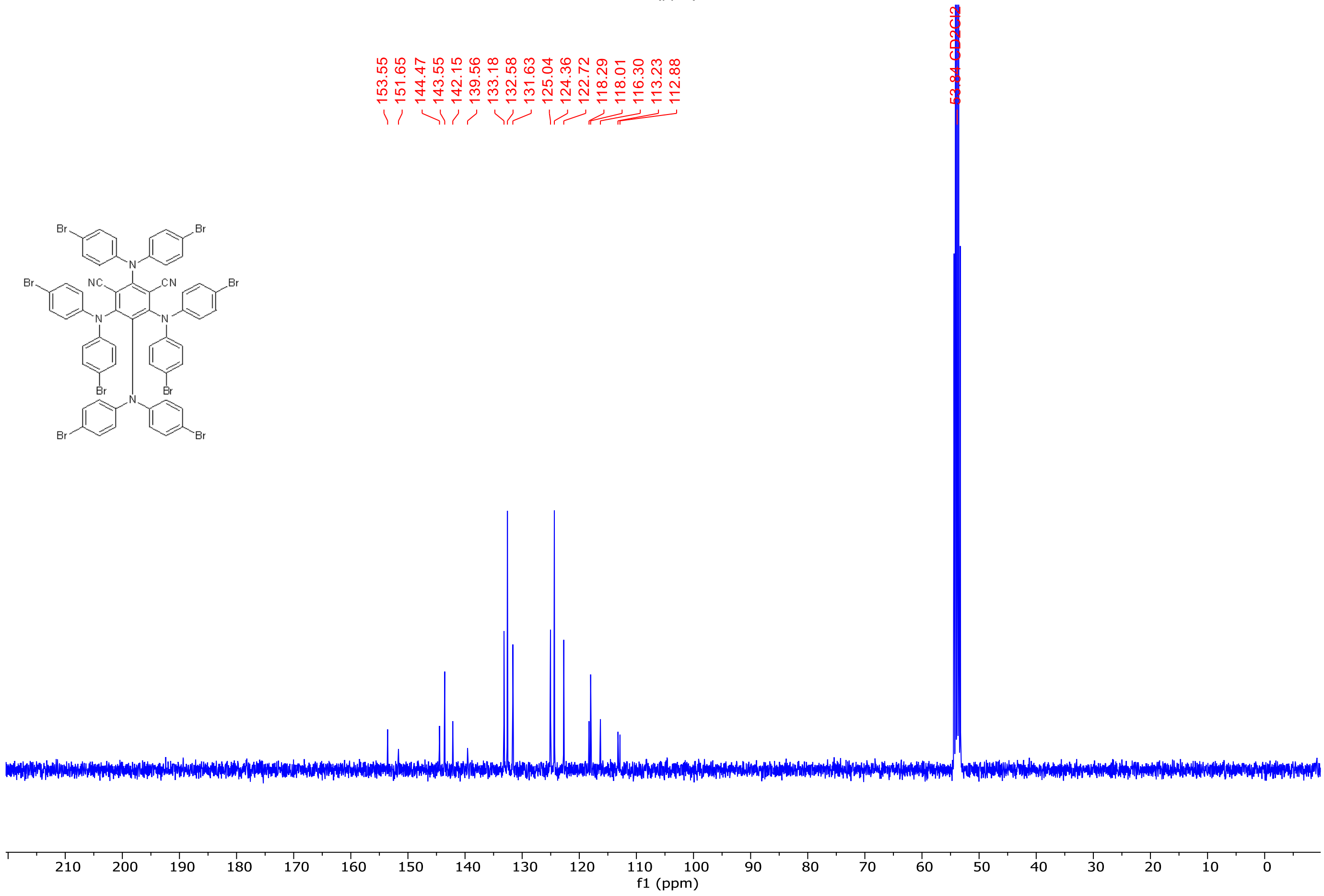

4DPAIPN---2,4,5,6-tetrakis(diphenylamino)isophthalonitrile (PC5)

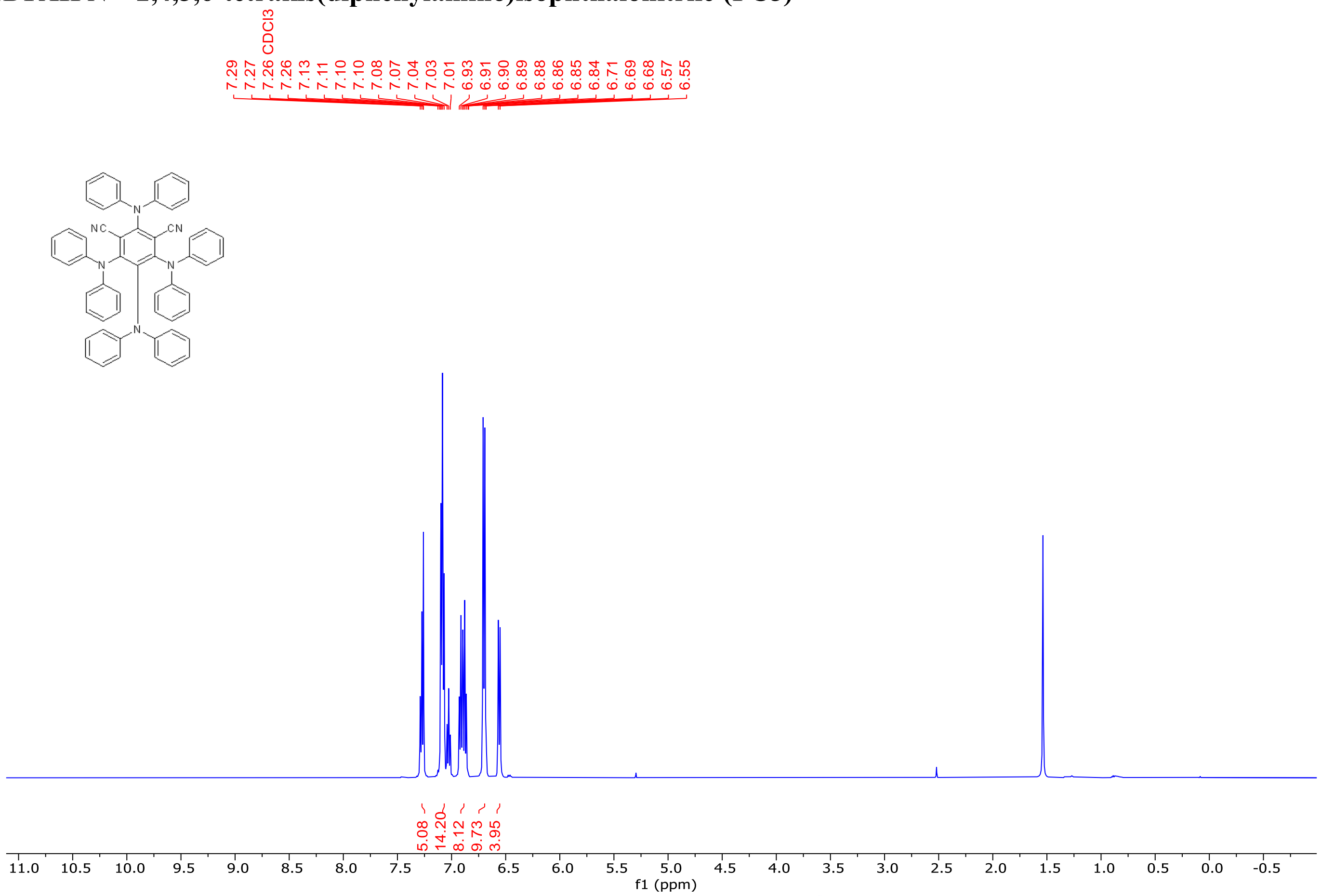

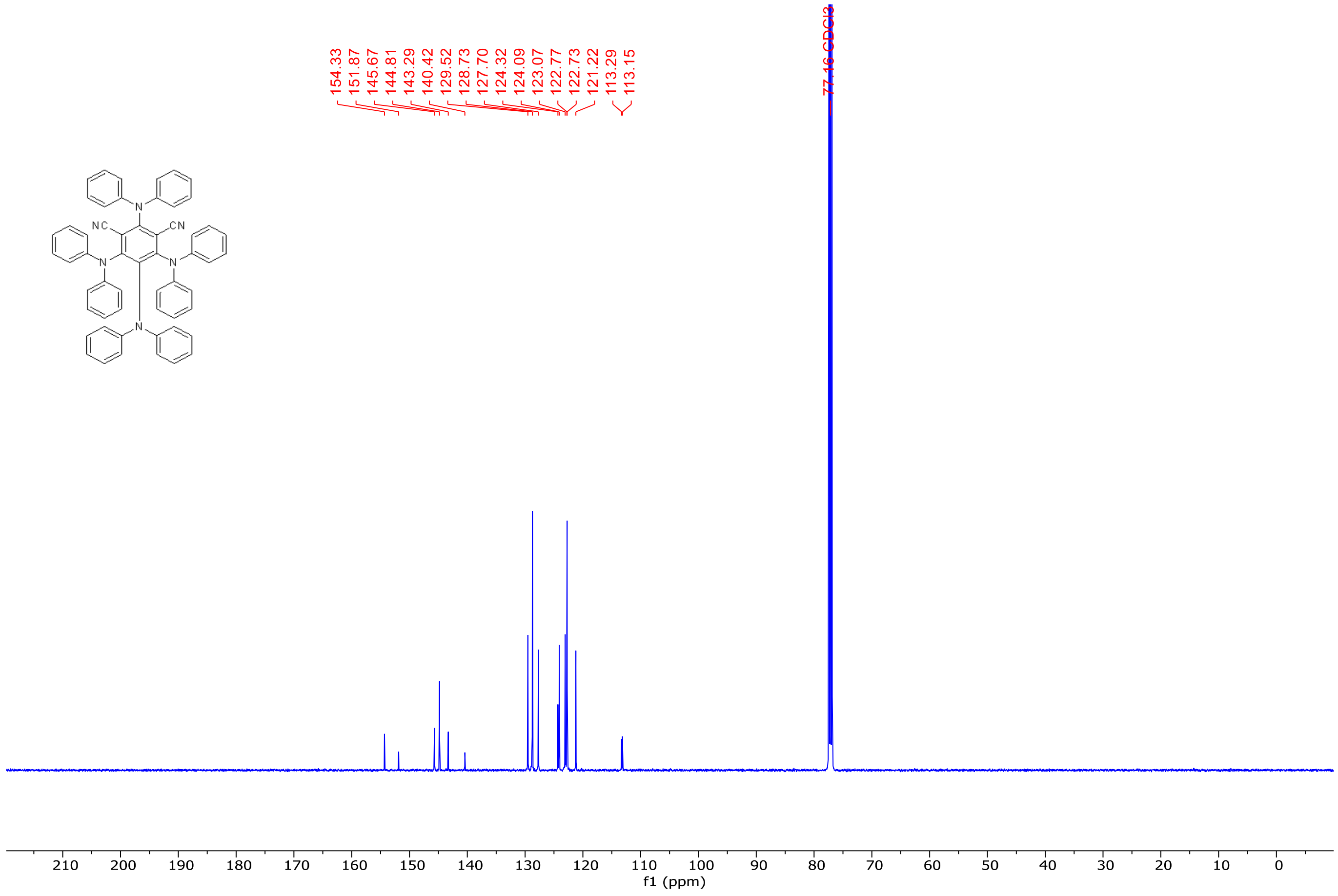

4MeOCzIPN---2,4,5,6-tetrakis(3,6-dimethoxy-9*H*-carbazol-9-yl)isophthalonitrile (PC6)

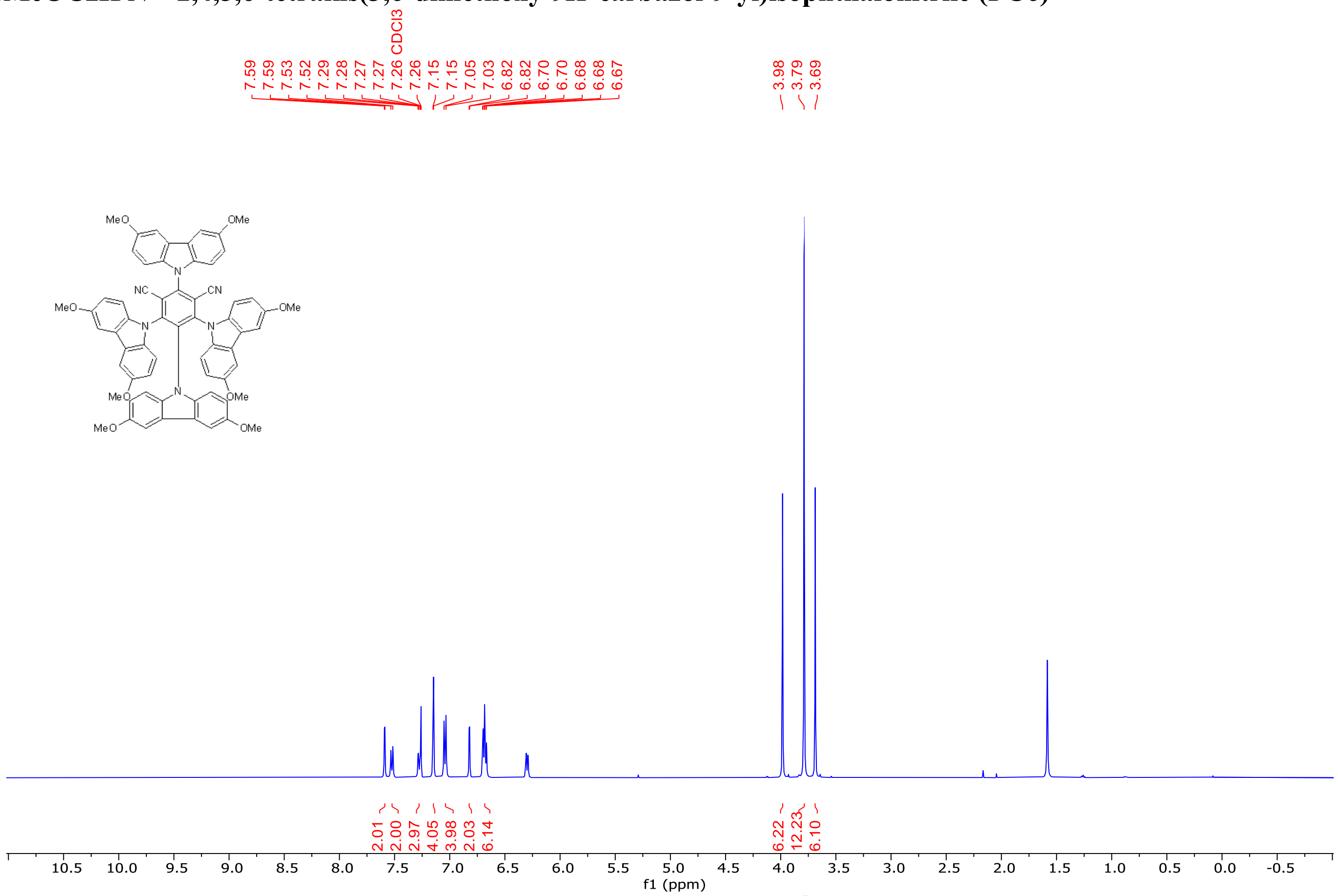

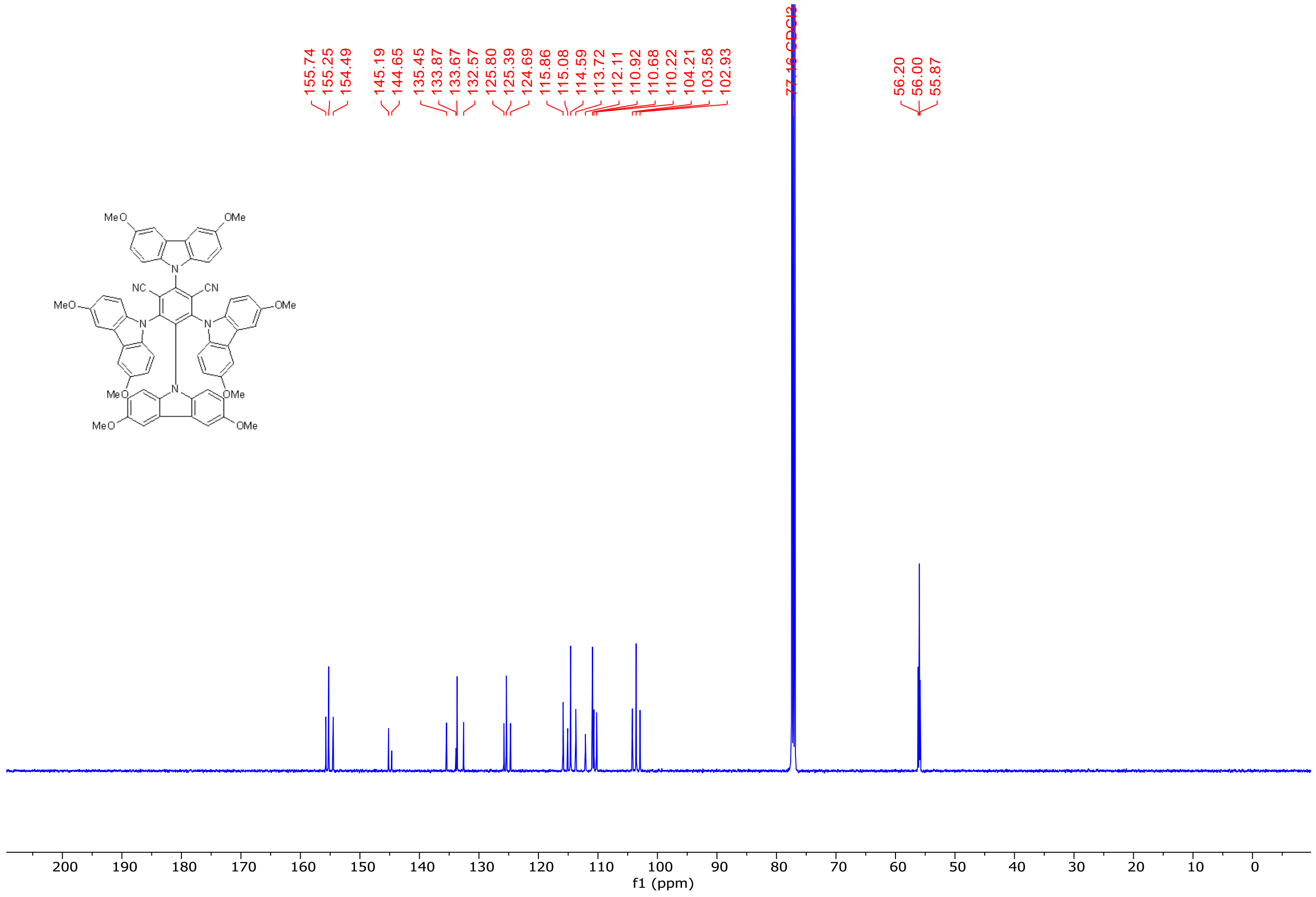

4PhDPAIPN---2,4,5,6-tetrakis(di([1,1'-biphenyl]-4-yl)amino) isophthalonitrile (PC7)

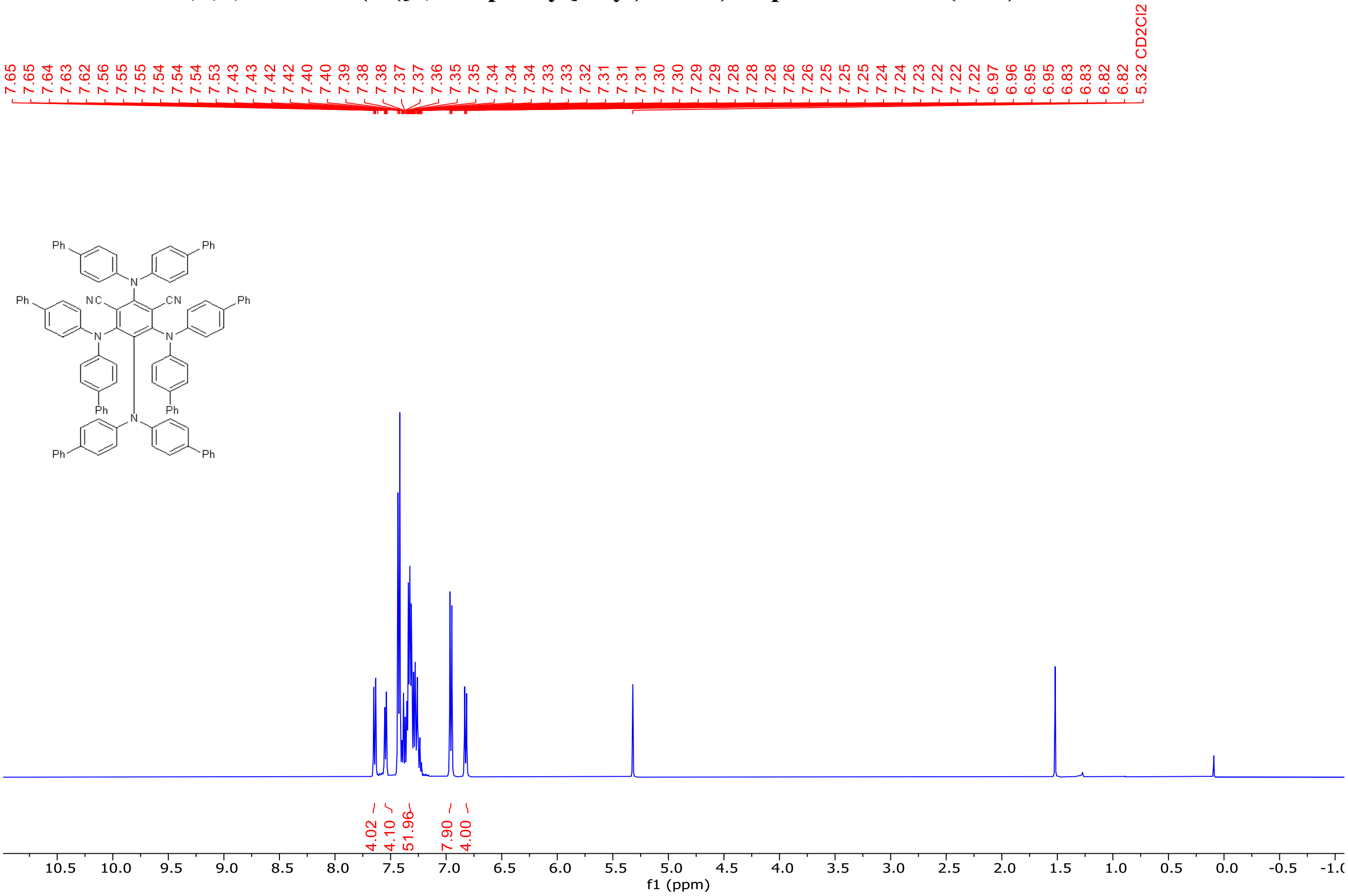

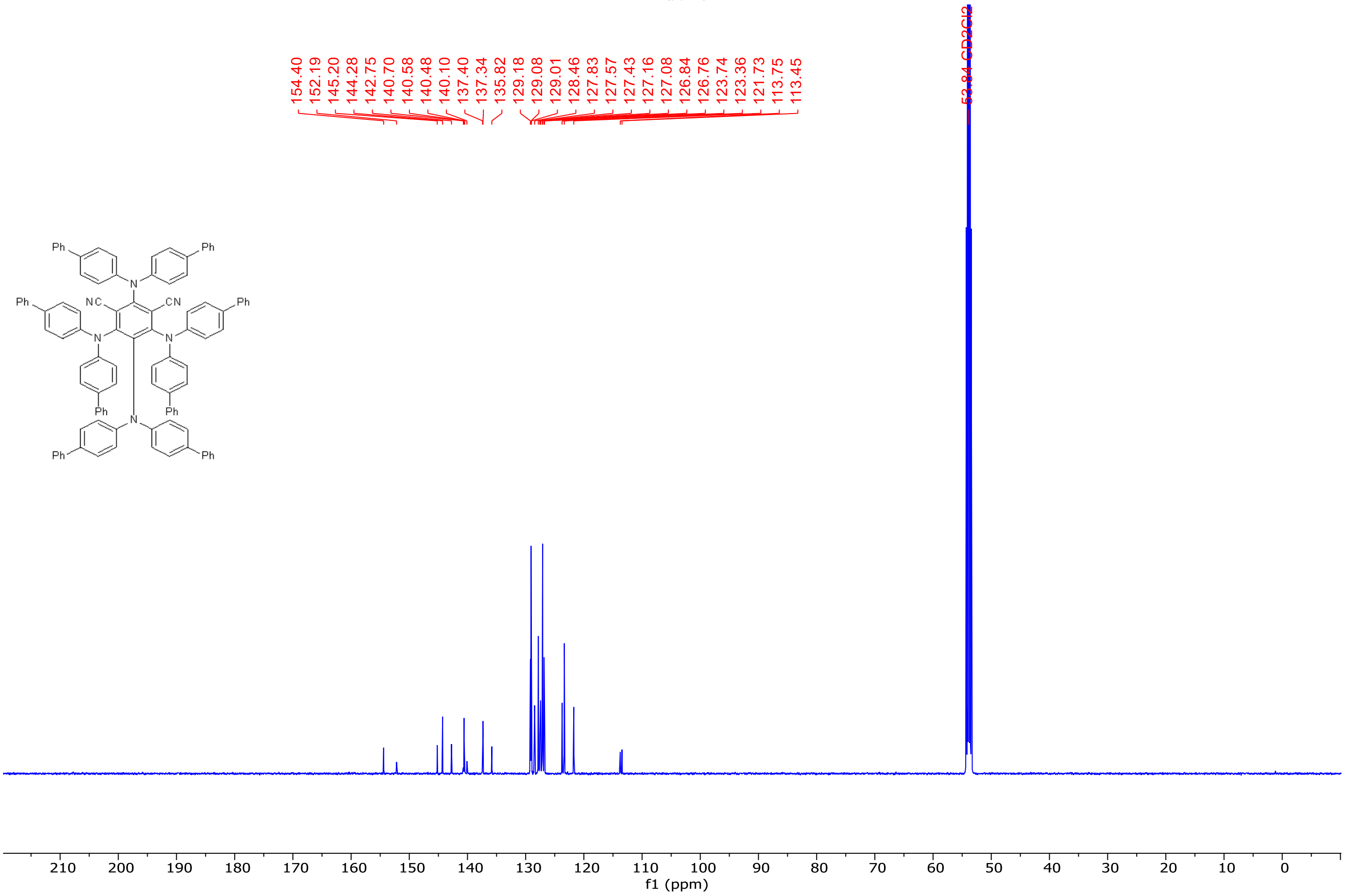

4^tBuDPAIPN---2,4,5,6-tetrakis(bis(4-(*tert*-butyl)phenyl)amino)isophthalonitrile (PC8)

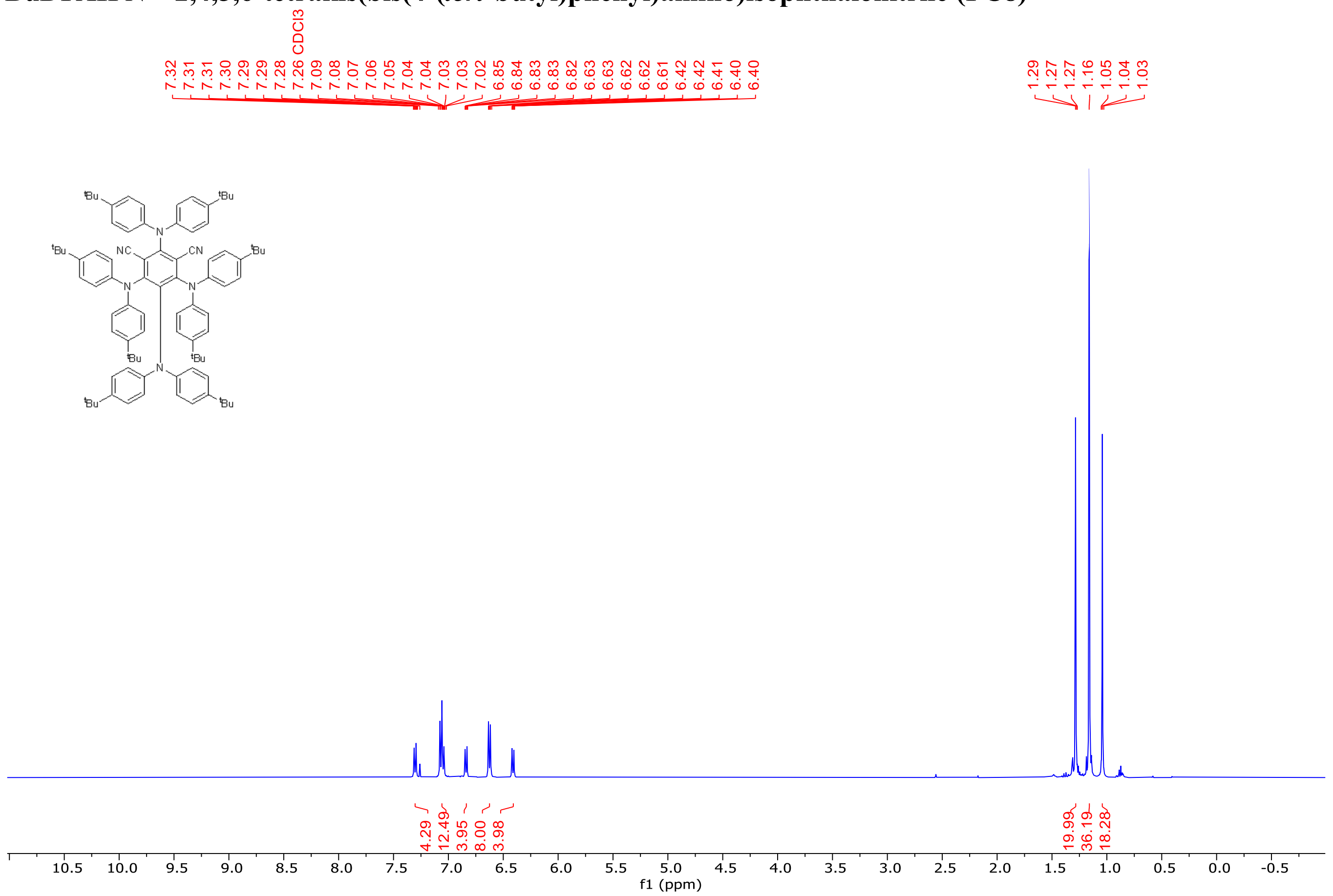

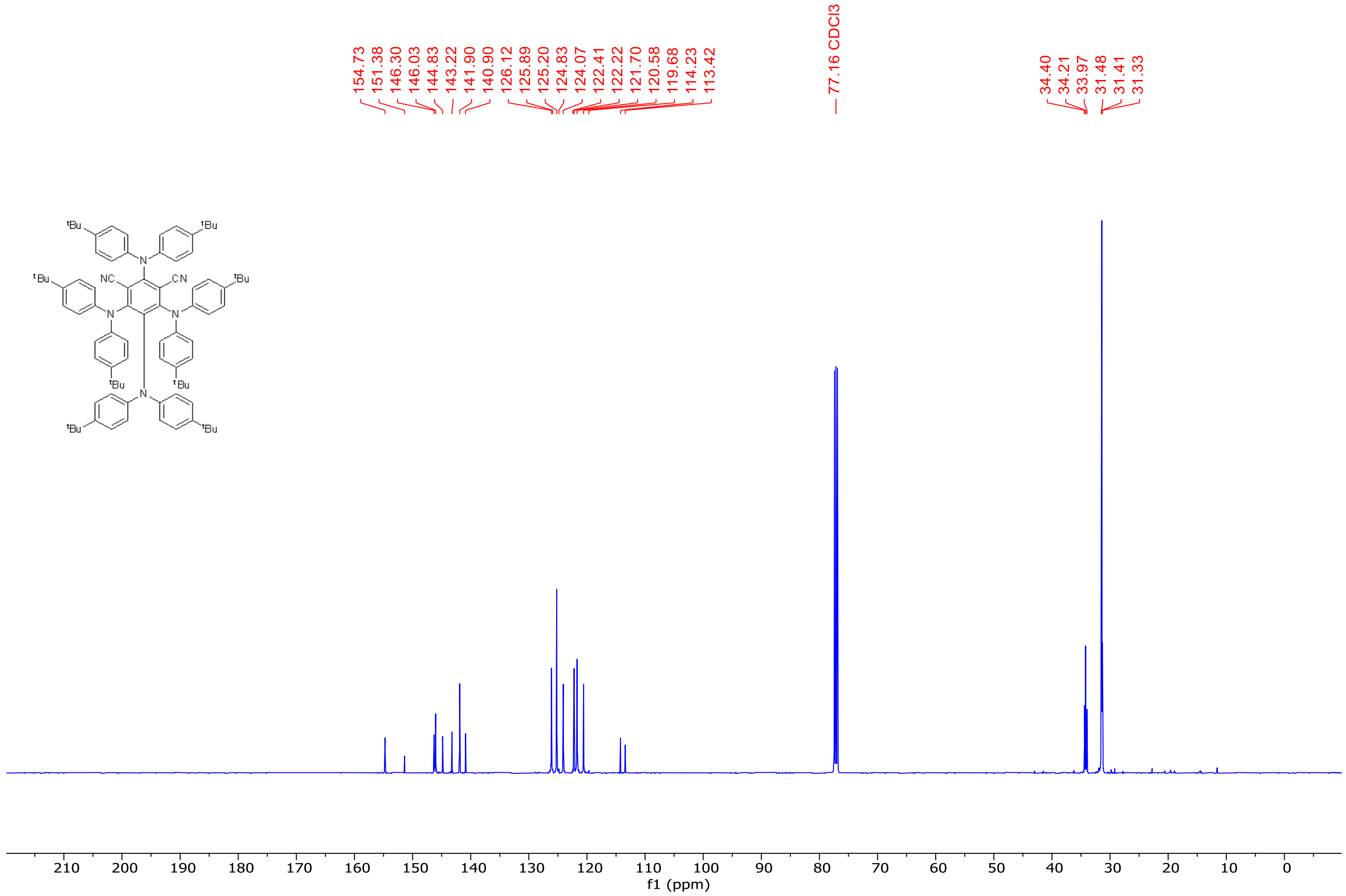

4MeODPAIPN---2,4,5,6-tetrakis(bis(4-methoxyphenyl)amino)isophthalonitrile (PC9)

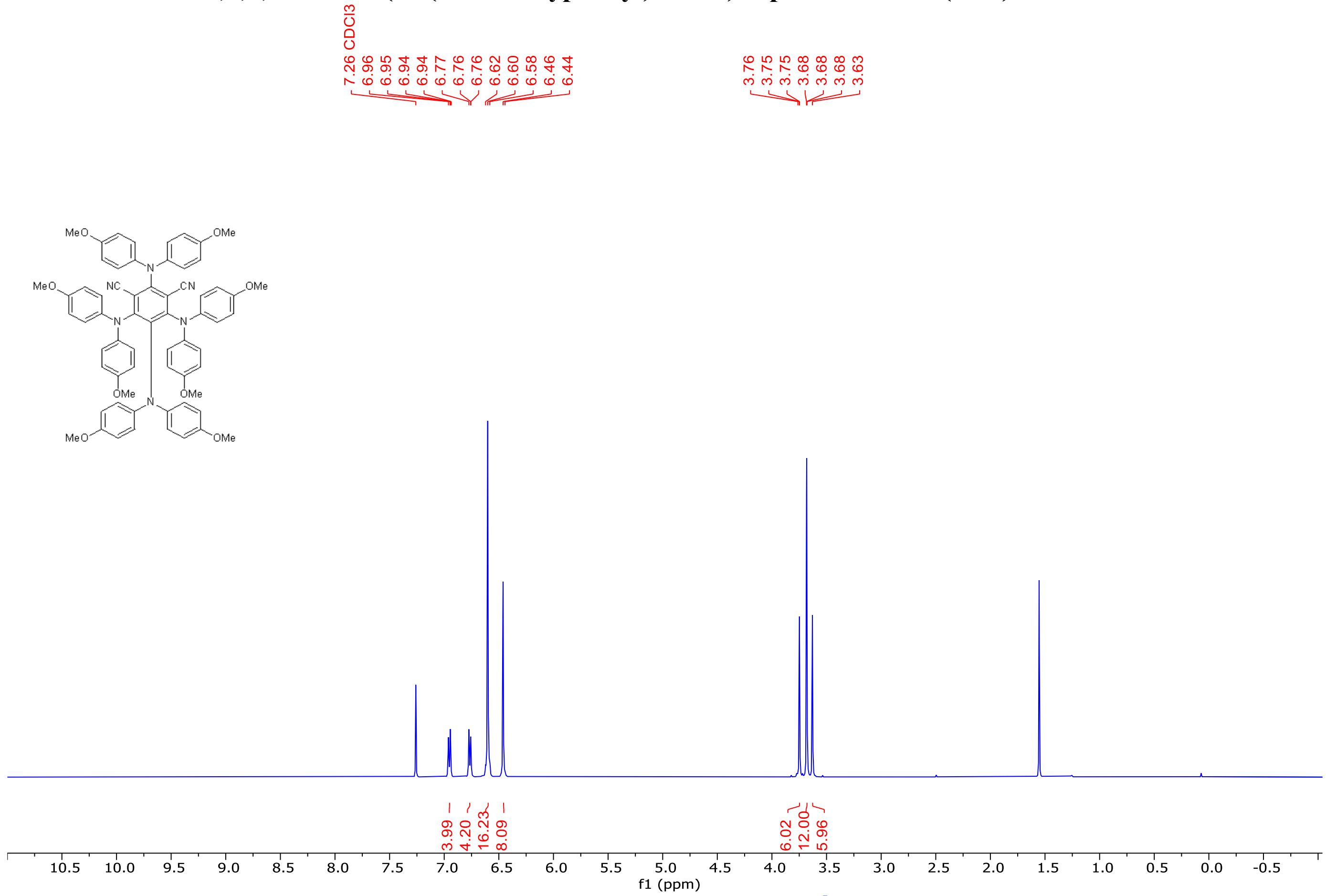

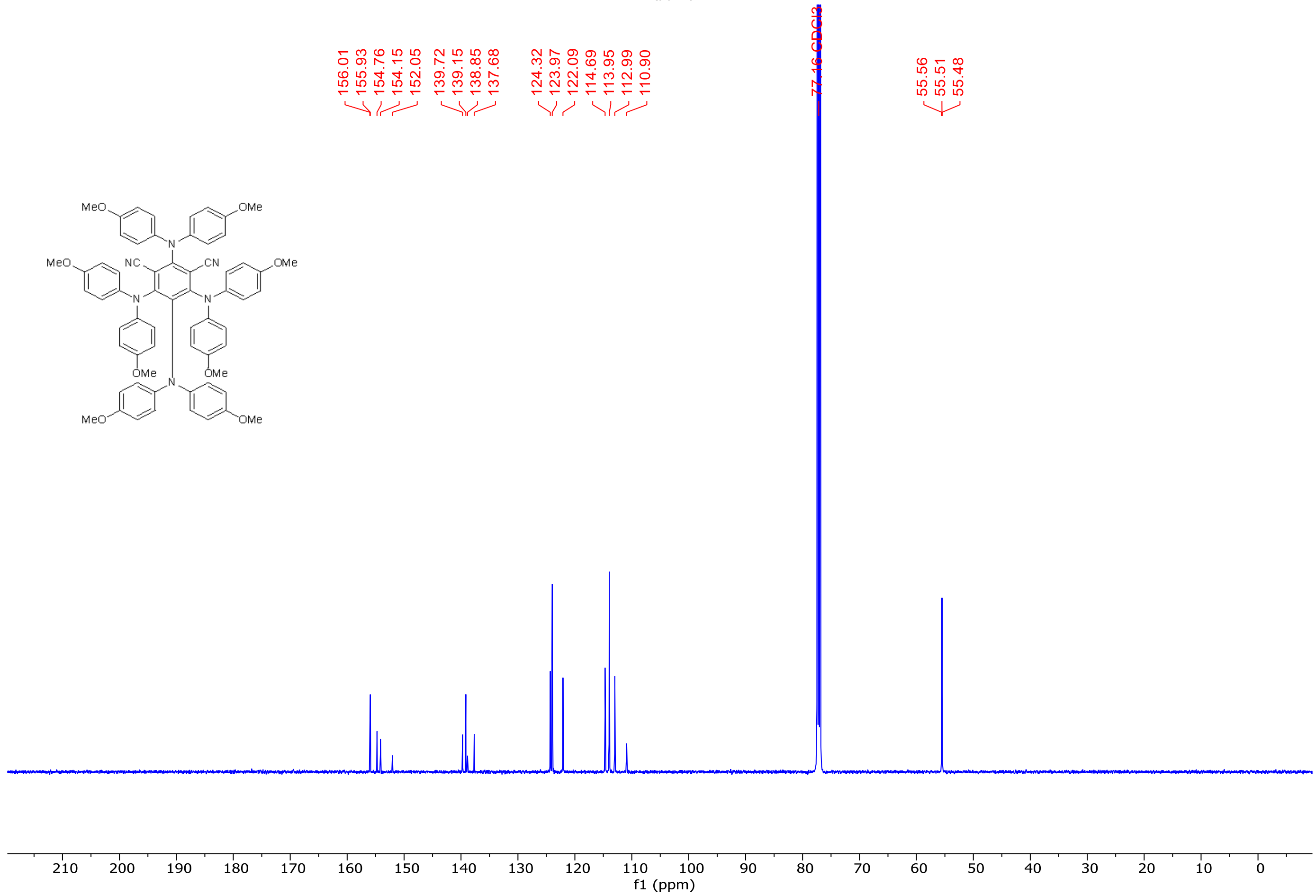

4MeODPAFBN---2,3,4,6-tetrakis(bis(4-methoxyphenyl)amino)-5-fluorobenzonitrile (PC10)

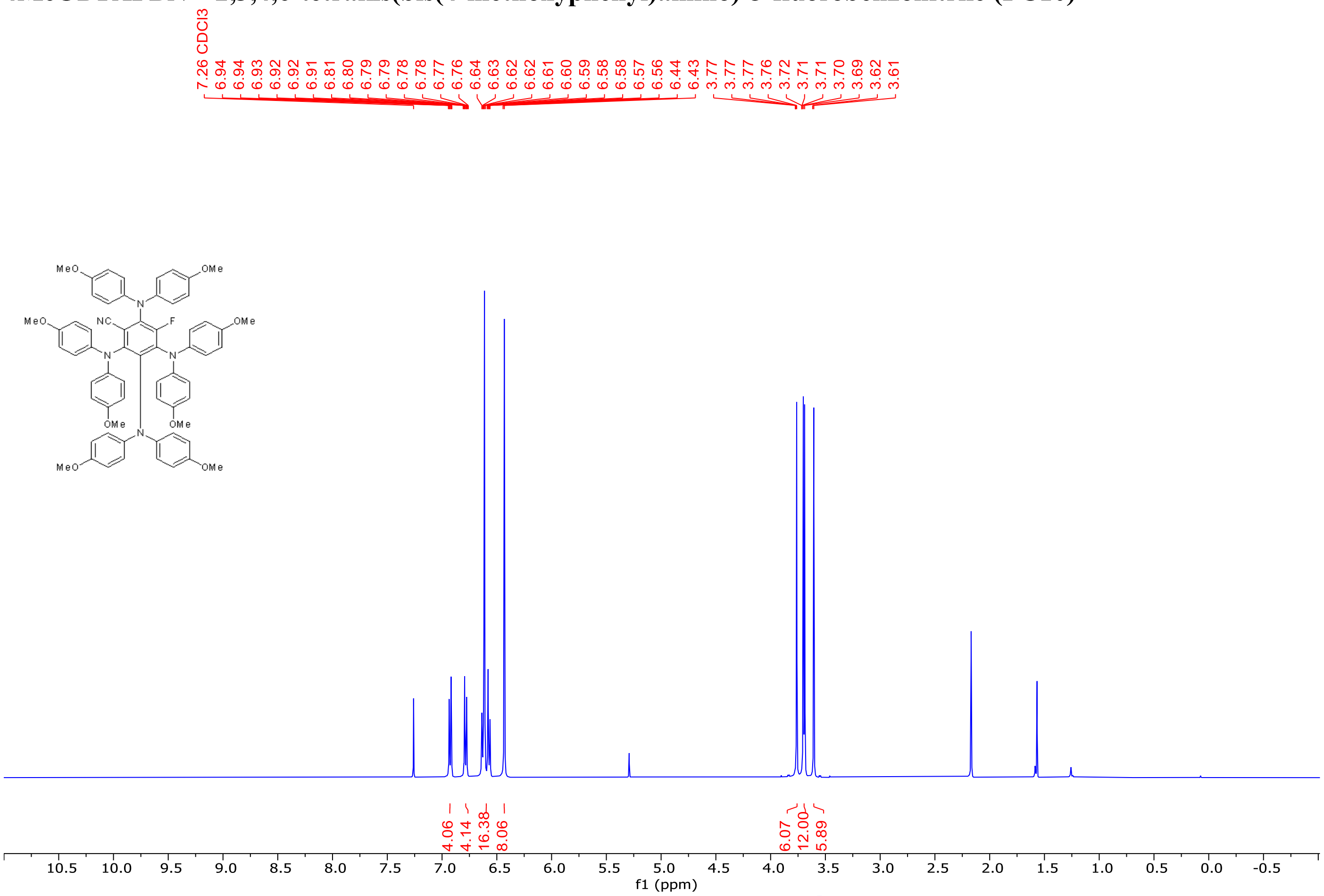

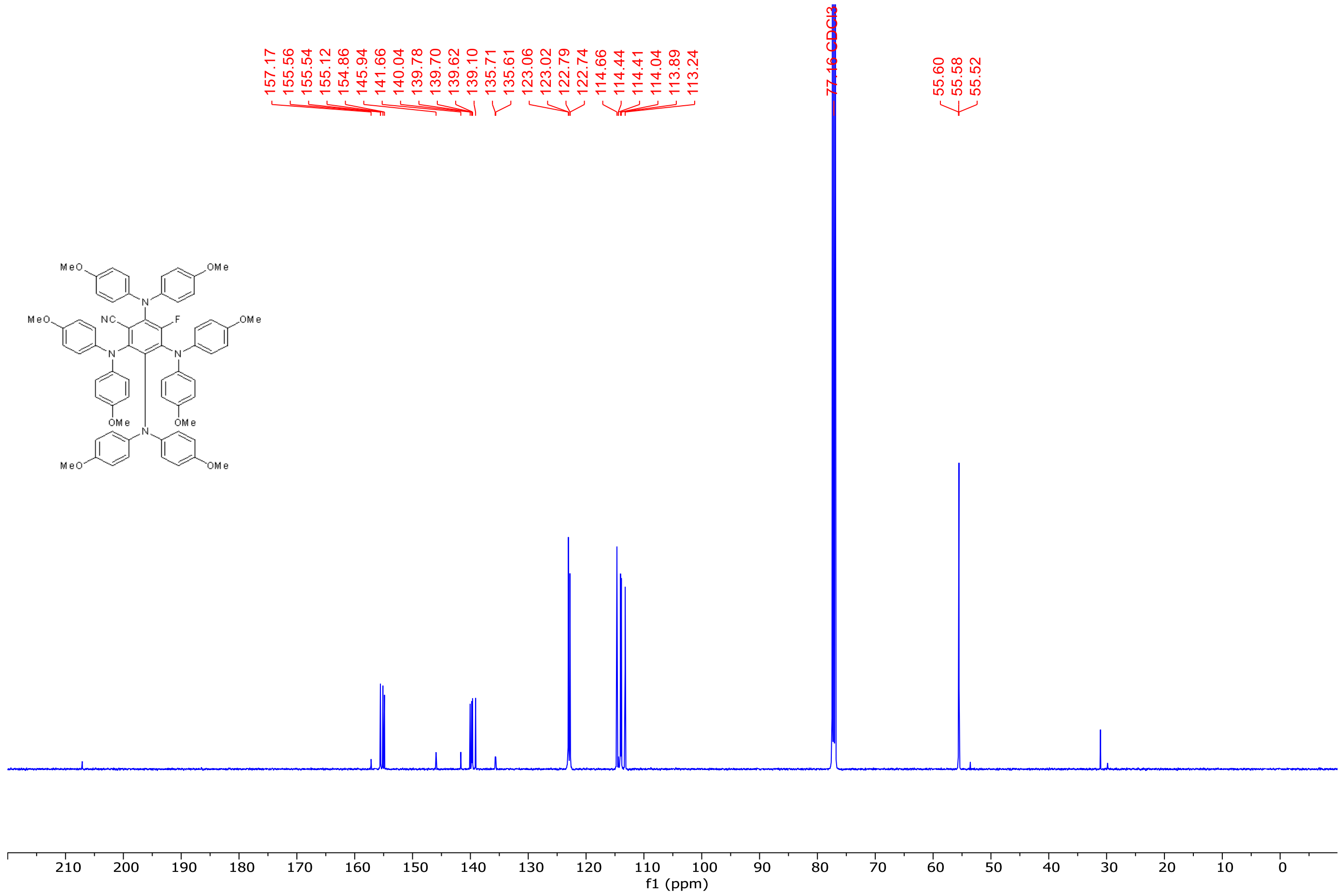

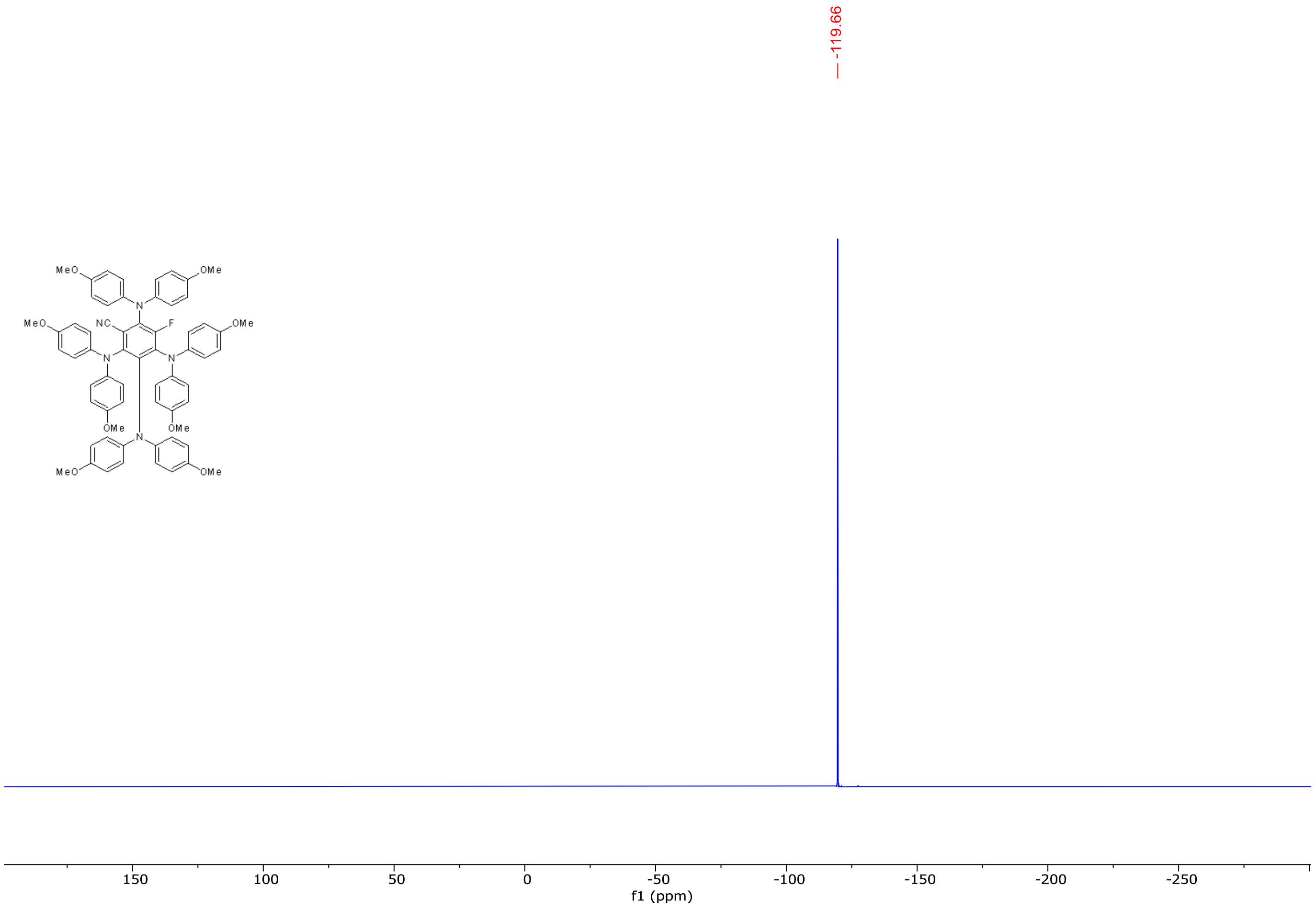

-119.66
MeO
OMe
NC
F
N
OMe
150
100
50
0
-50
-100
-150
-200
-250
f1 (ppm)

4MeODPAINN---2,3,5,6-tetrakis(bis(4-methoxyphenyl)amino)isonicotinonitrile (PC11)

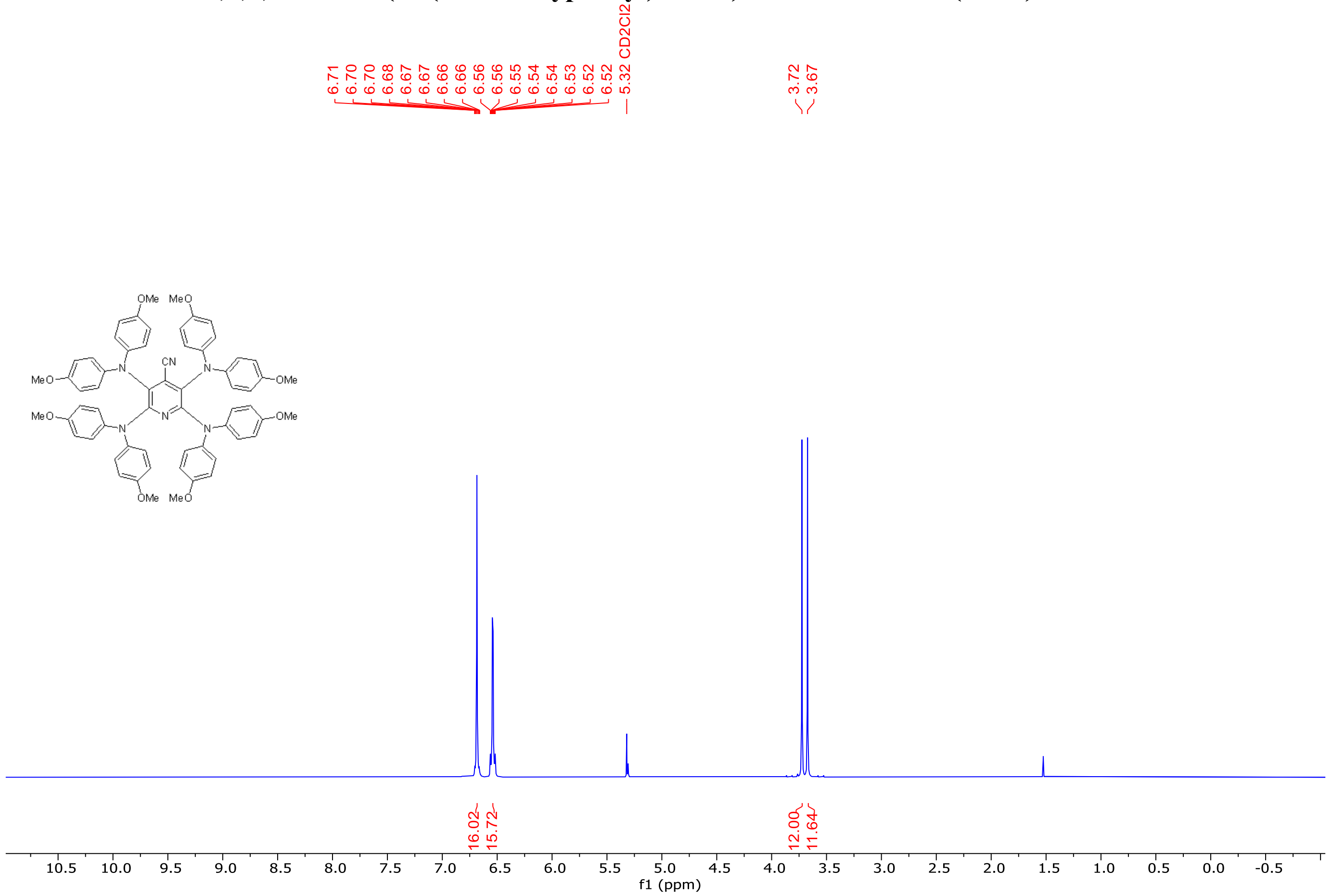

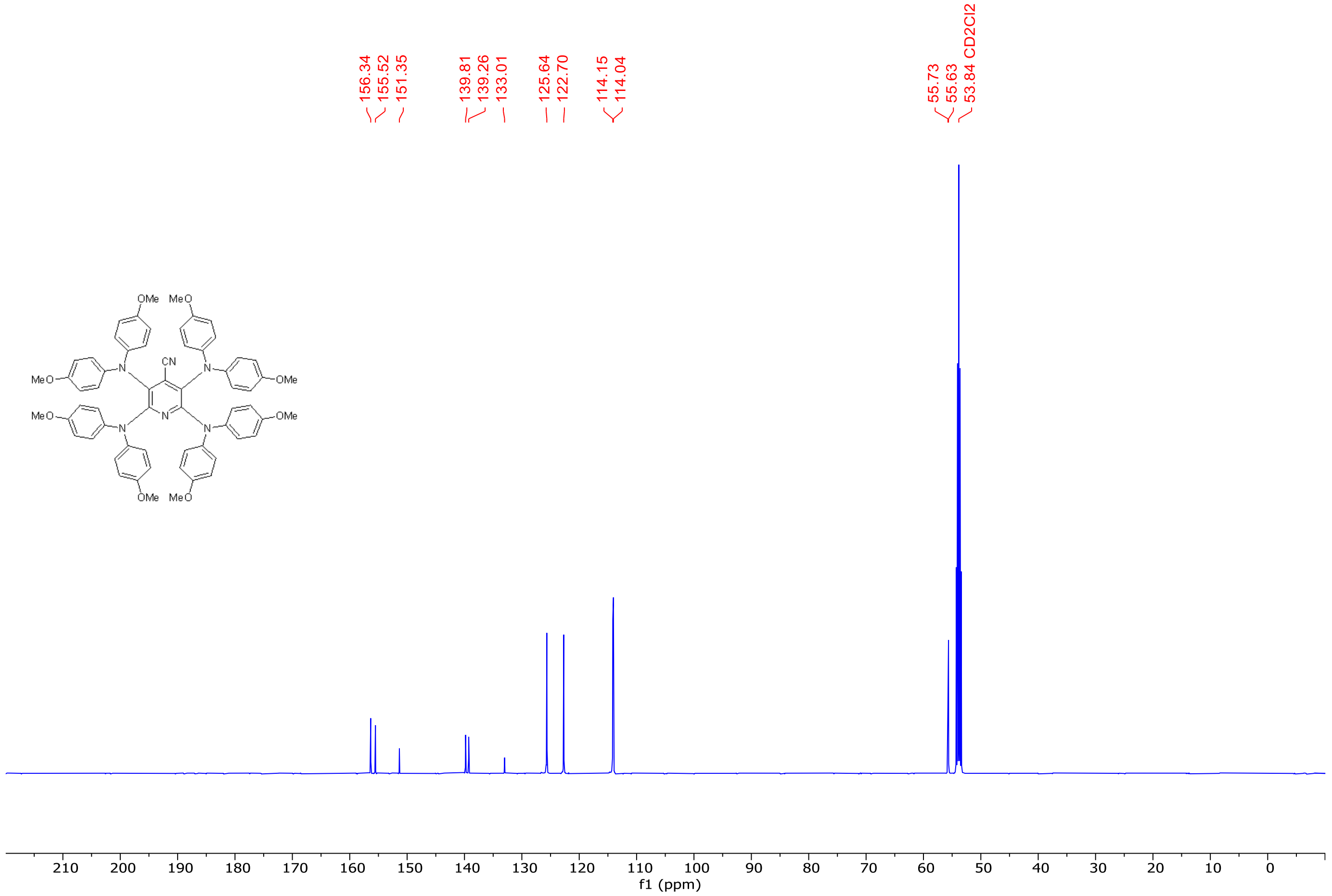

4MeODPAPN---3,4,5,6-tetrakis(bis(4-methoxyphenyl)amino)phthalonitrile (PC12)

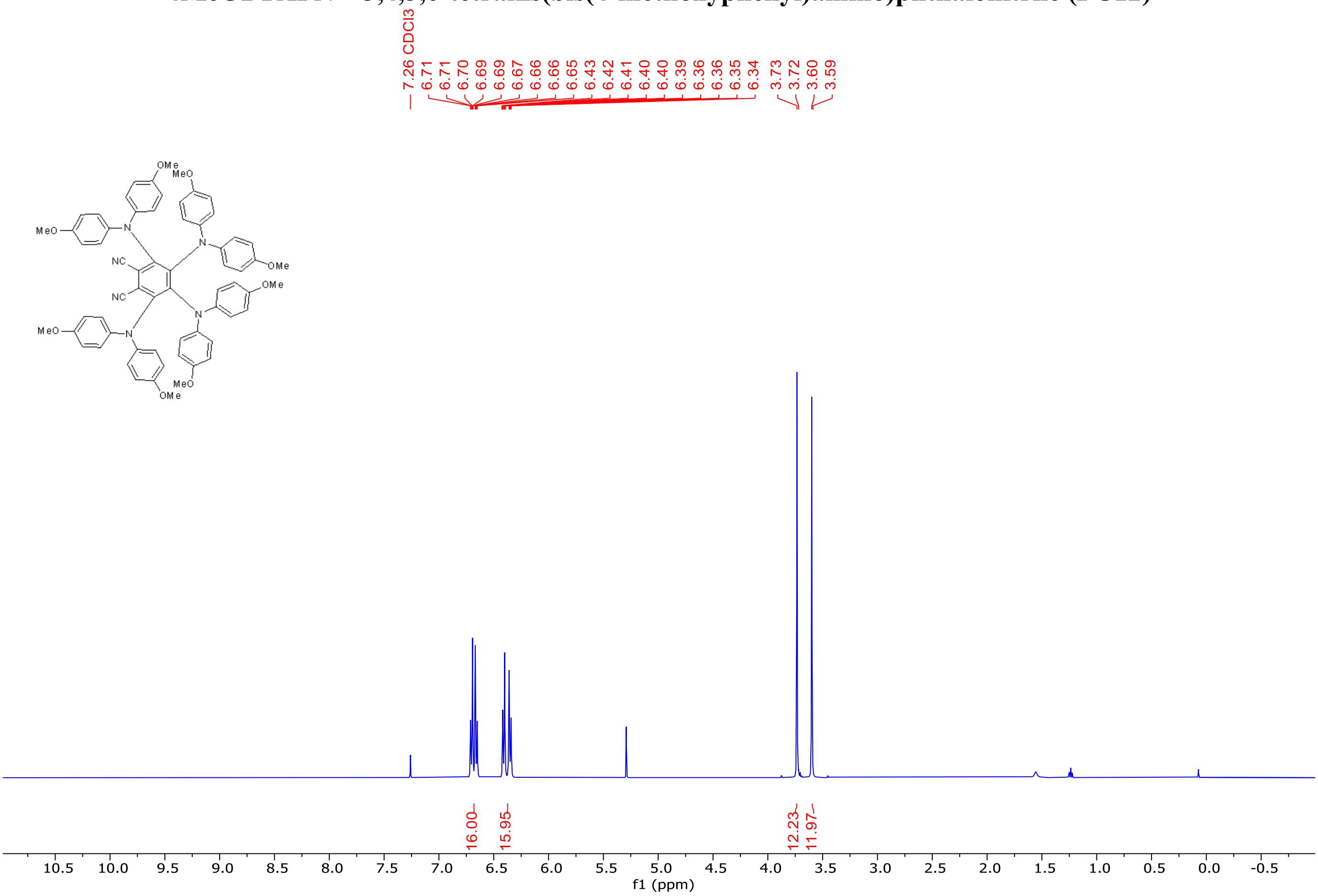

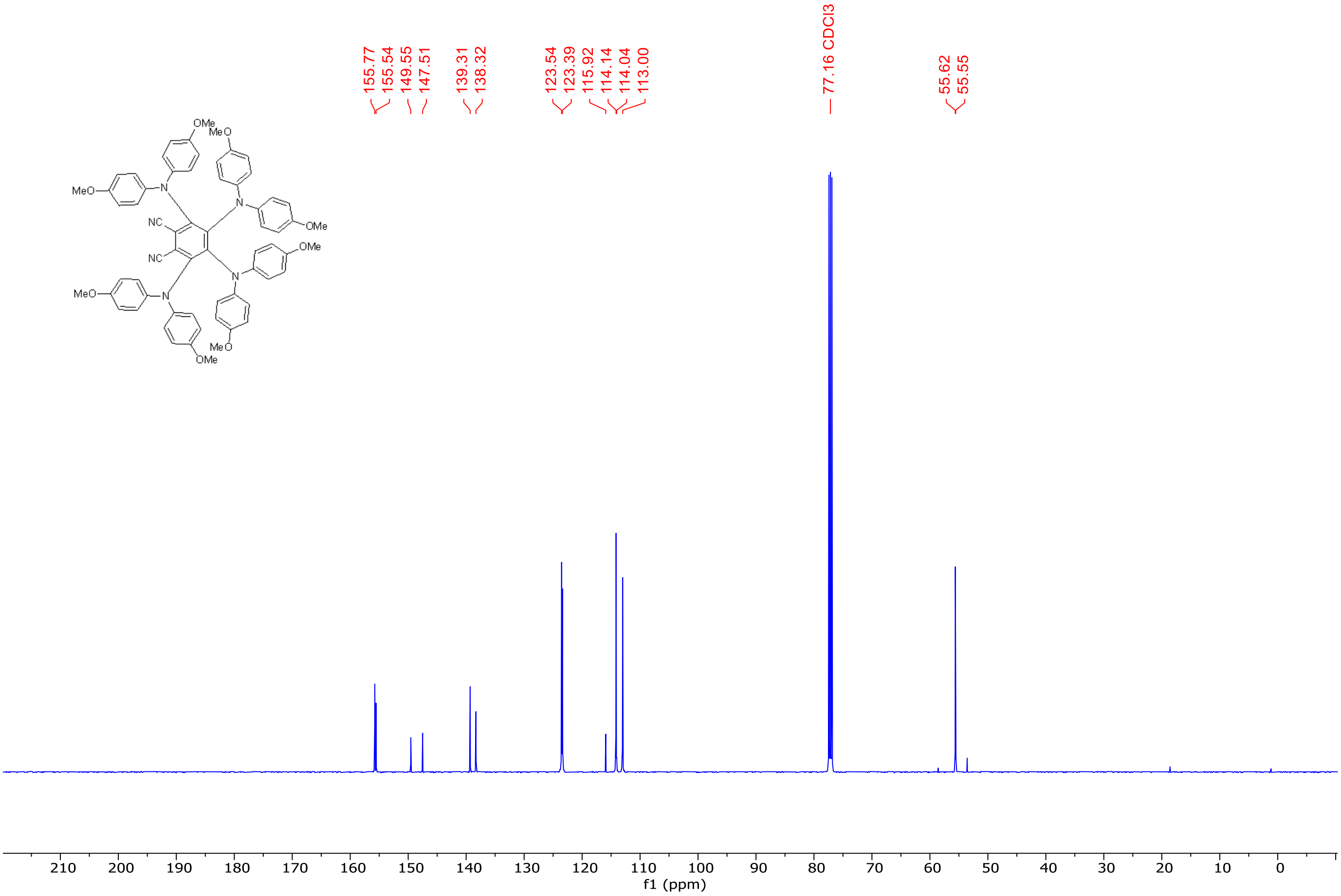

4MeODPATPN---2,3,5,6-tetrakis(bis(4-methoxyphenyl)amino)terephthalonitrile (PC13)

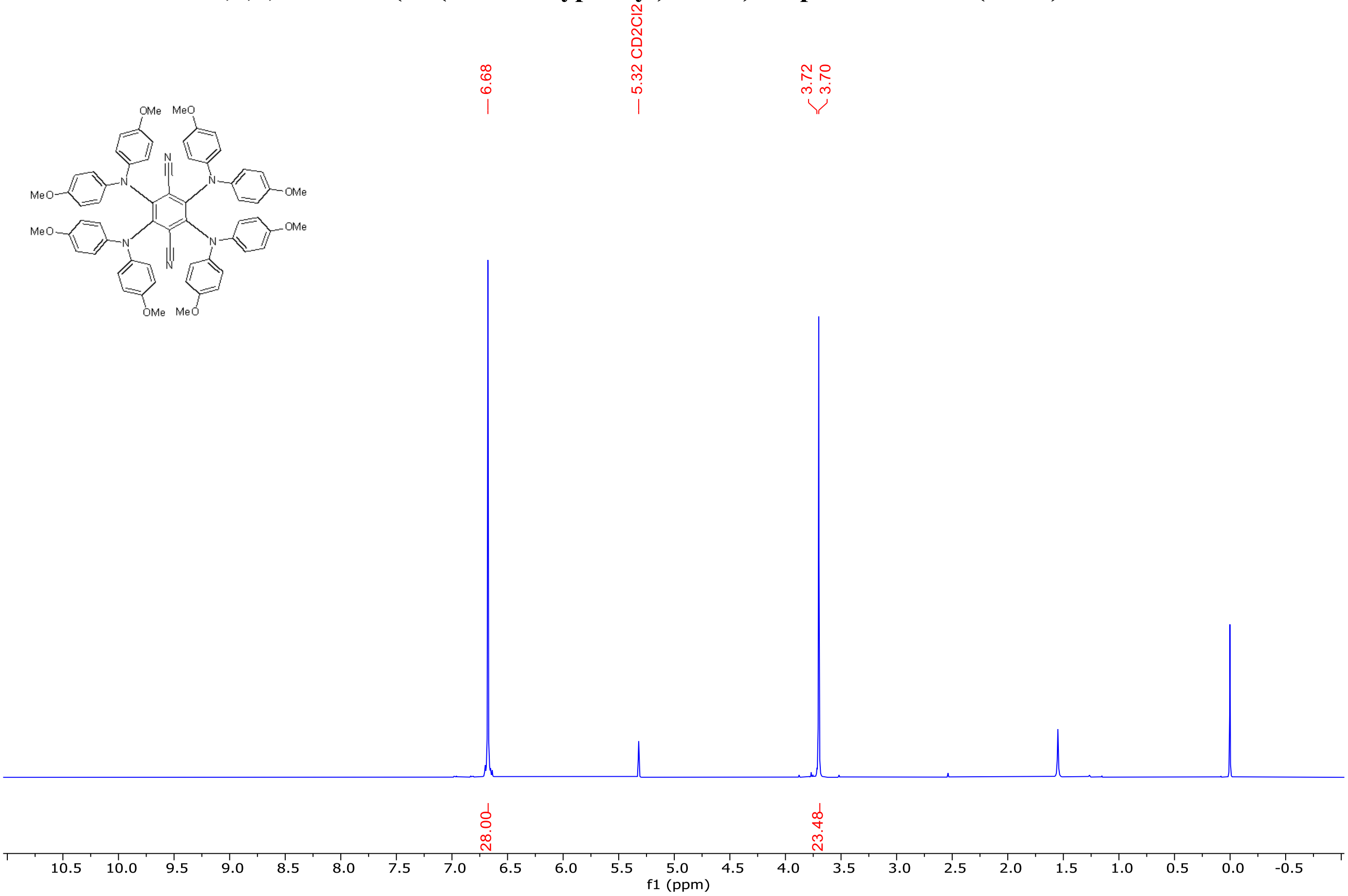

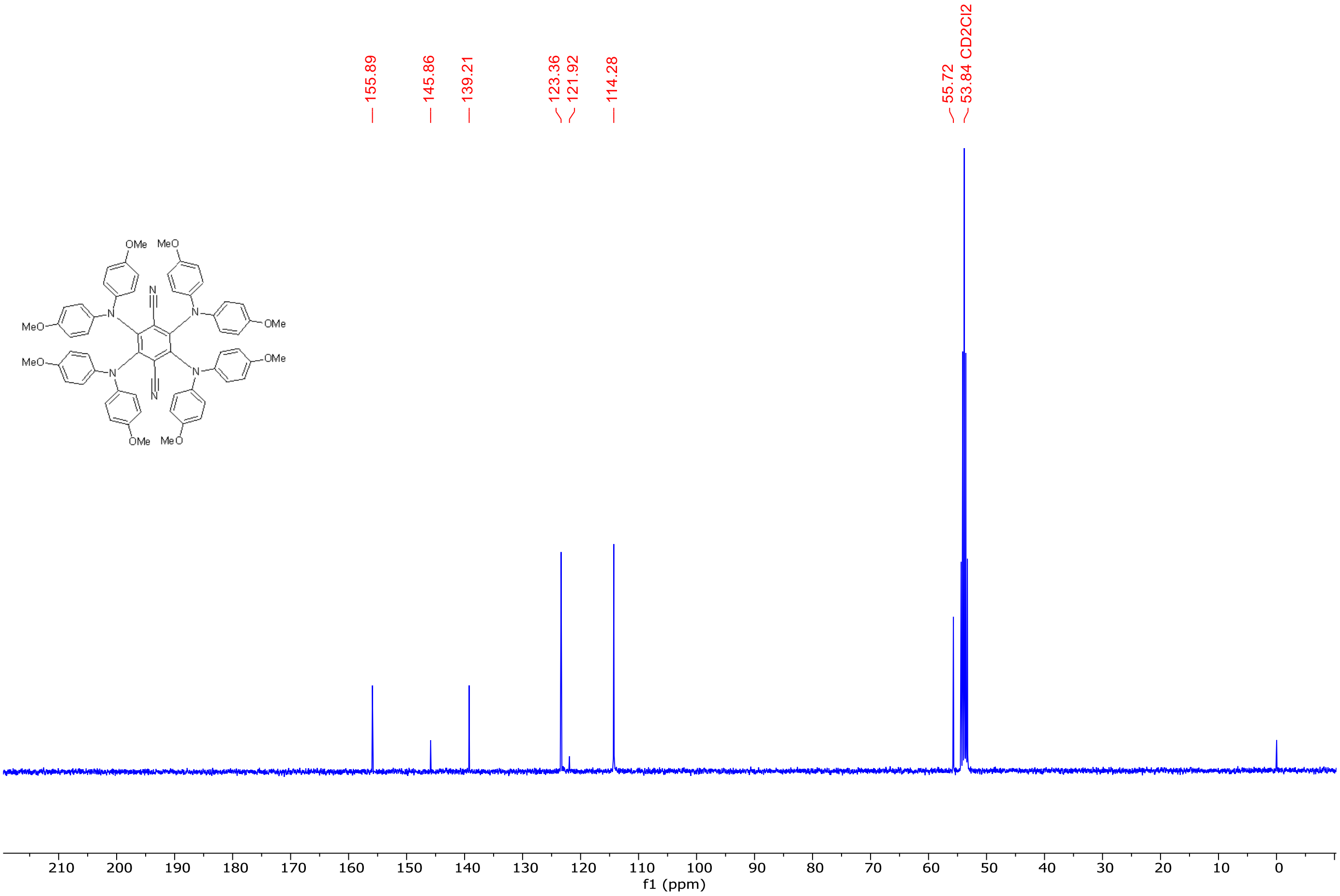

N-methyl-*N*-(2,3,4,6-tetra(9*H*-carbazol-9-yl)-5-cyanobenzyl)acetamide (PC1-1)

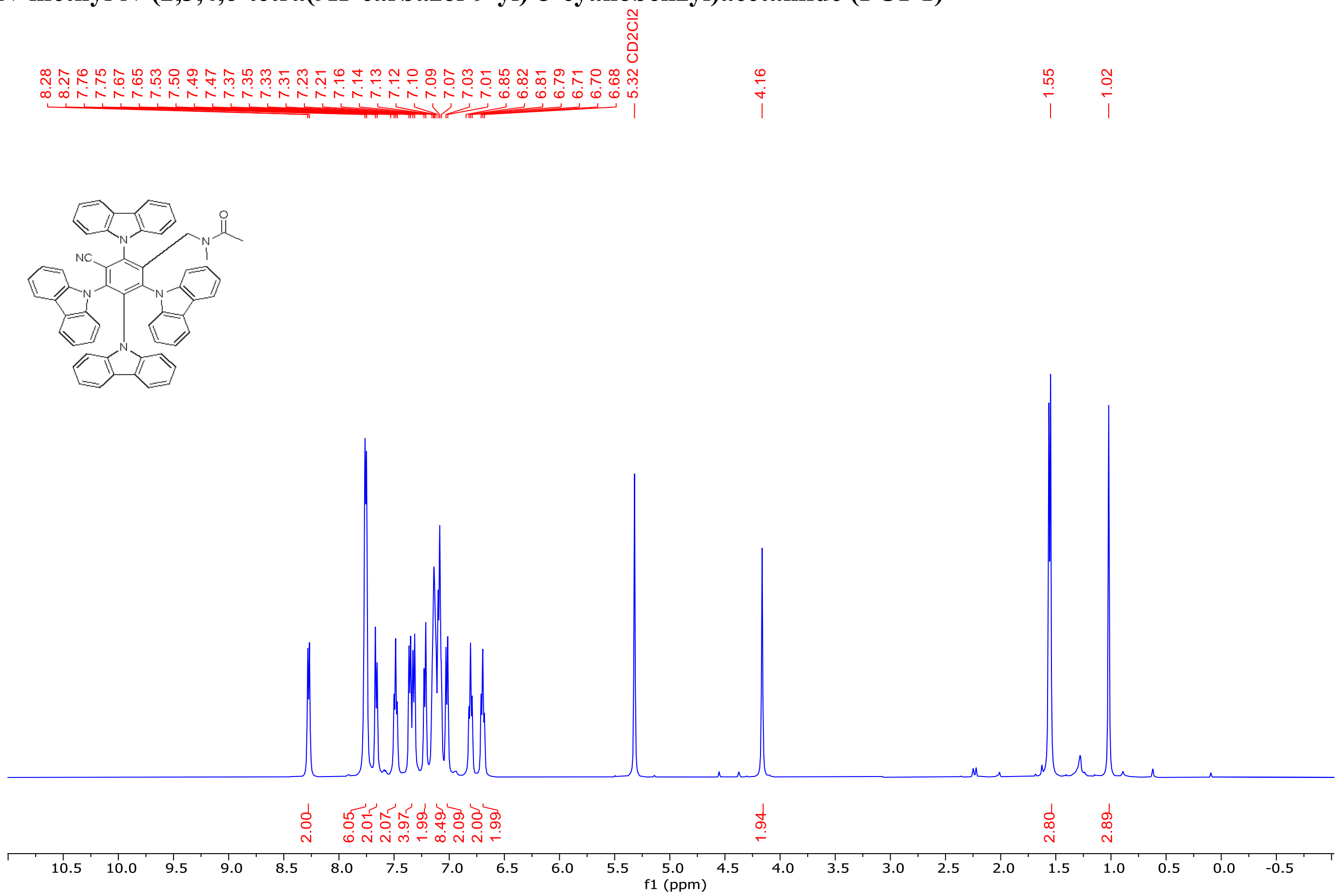

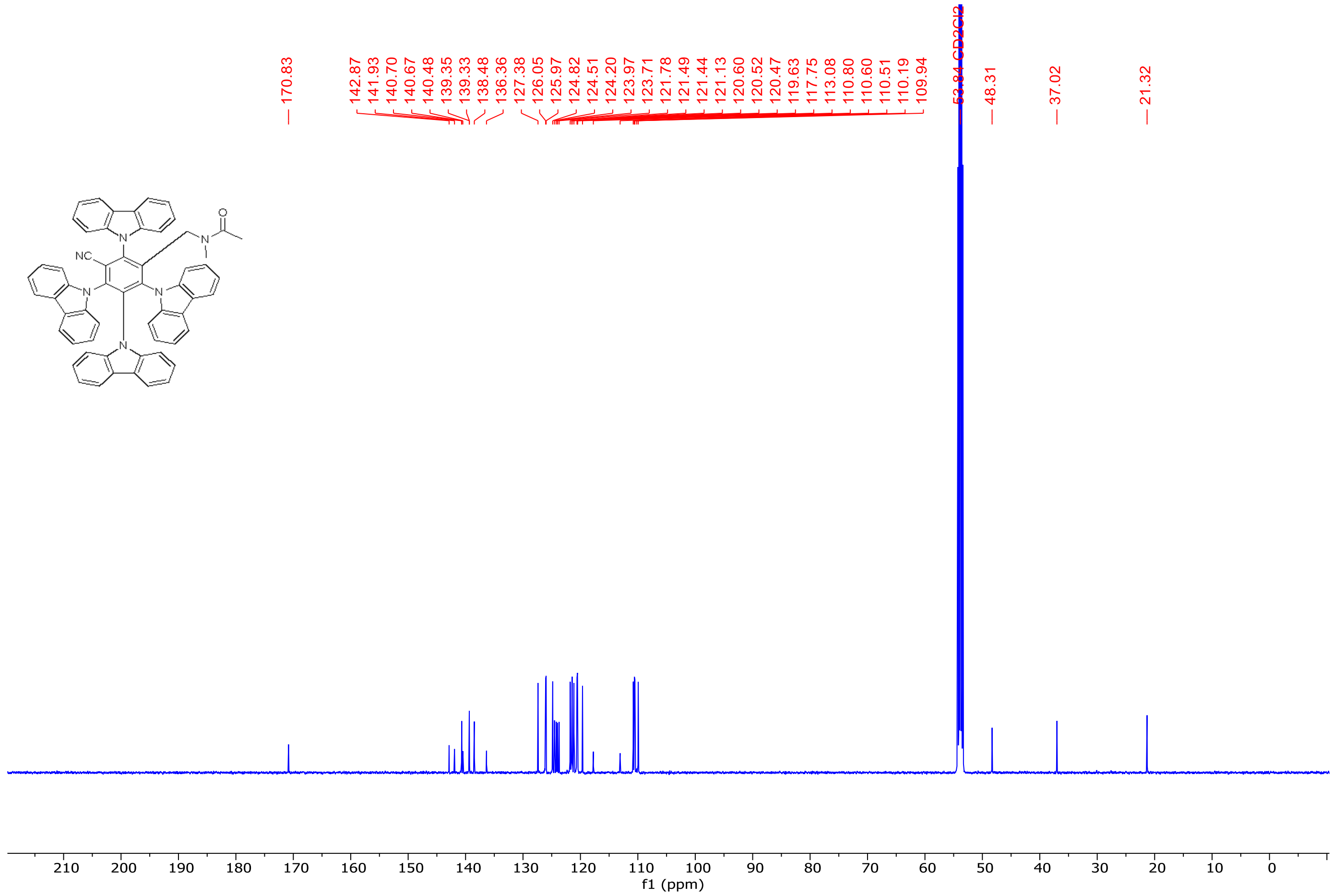

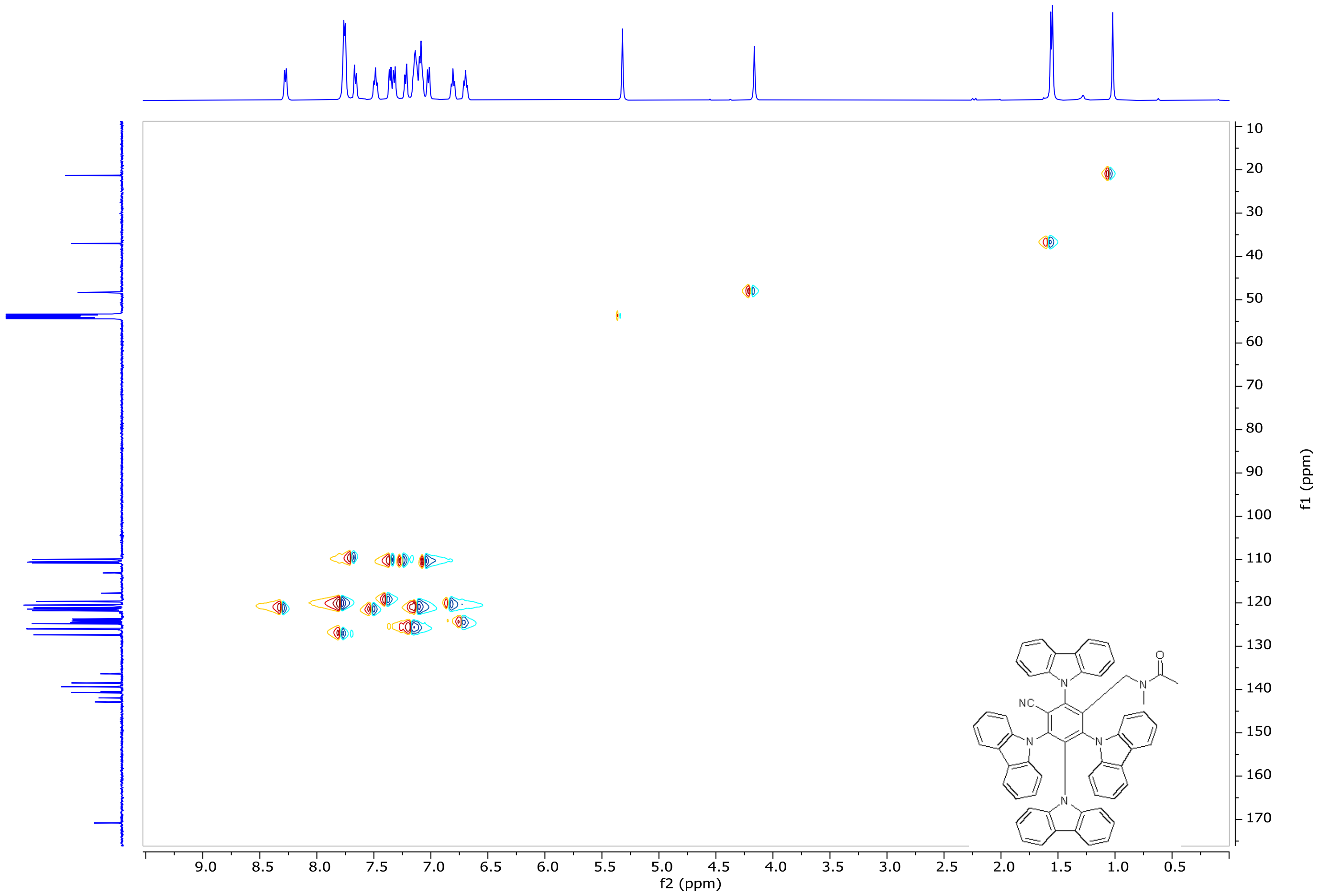
f2 (ppm)
f1 (ppm)
9.0
8.5
8.0
7.5
7.0
6.5
6.0
5.5
5.0
4.5
4.0
3.5
3.0
2.5
2.0
1.5
1.0
0.5
10
20
30
40
50
60
70
80
90
100
110
120
130
140
150
160
170
NC
N
O

***N*-methyl-*N*-(2,3,5,6-tetrakis(bis(4-methoxyphenyl)amino)-4-cyanobenzyl)acetamide (PC13-1)**

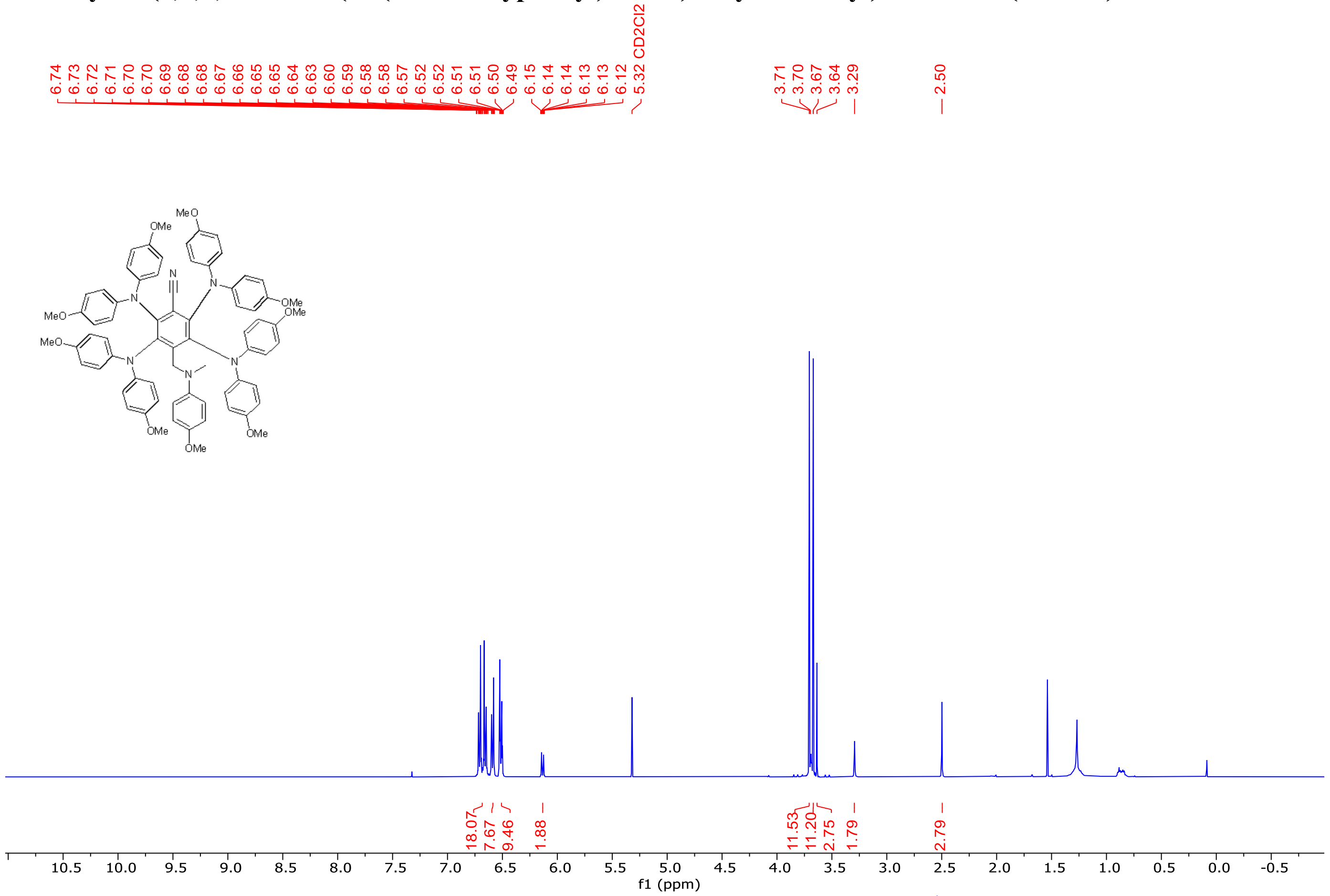

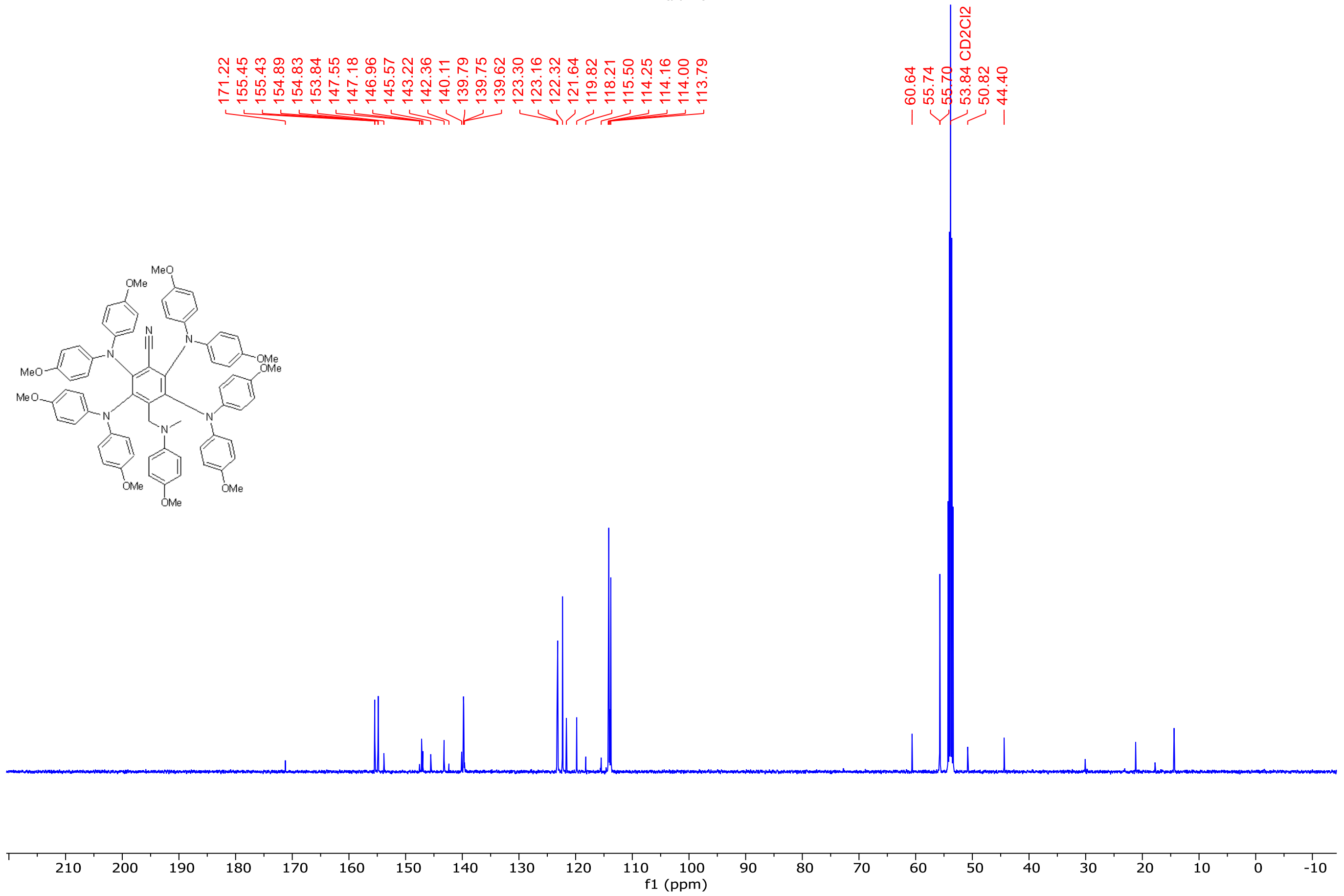

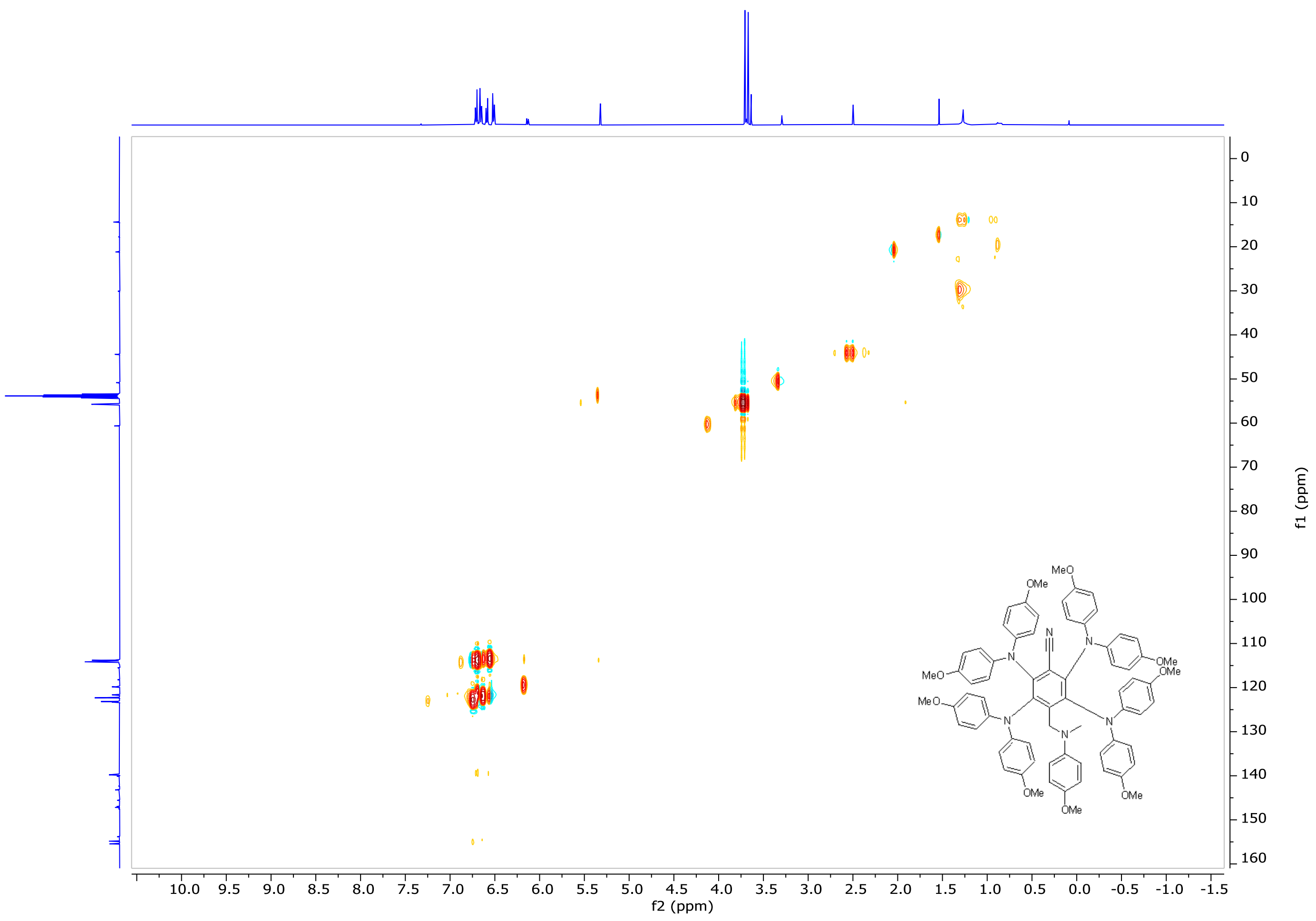
f1 (ppm)
f2 (ppm)
MeO
OMe
N
MeO
N
N
OMe
OMe
MeO
N
N
N
OMe
OMe
OMe

N-methyl-N-(2,3,5,6-tetrakis(bis(4-methoxyphenyl)amino)- 4-cyanobenzyl)acetamide (PC13-2)

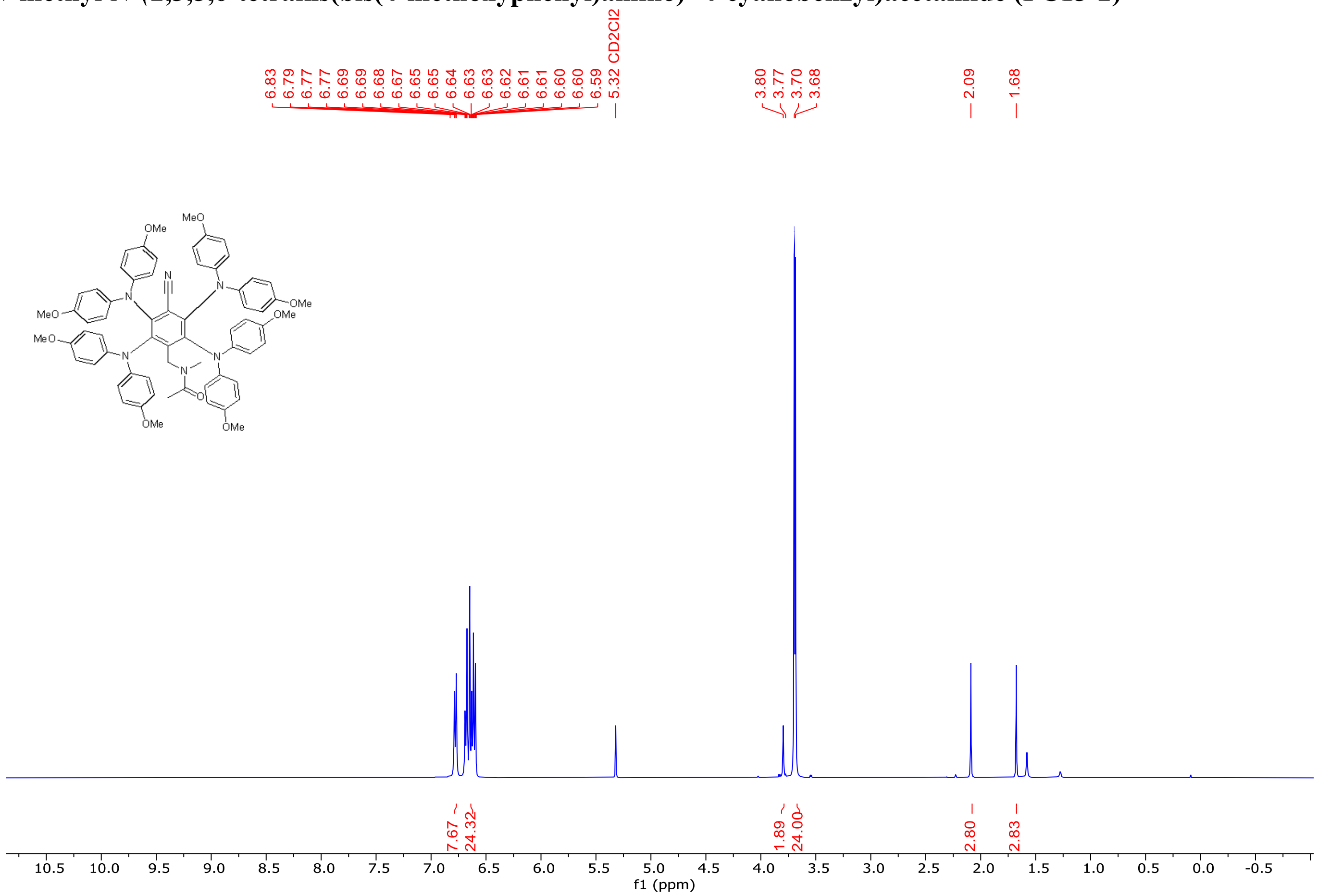

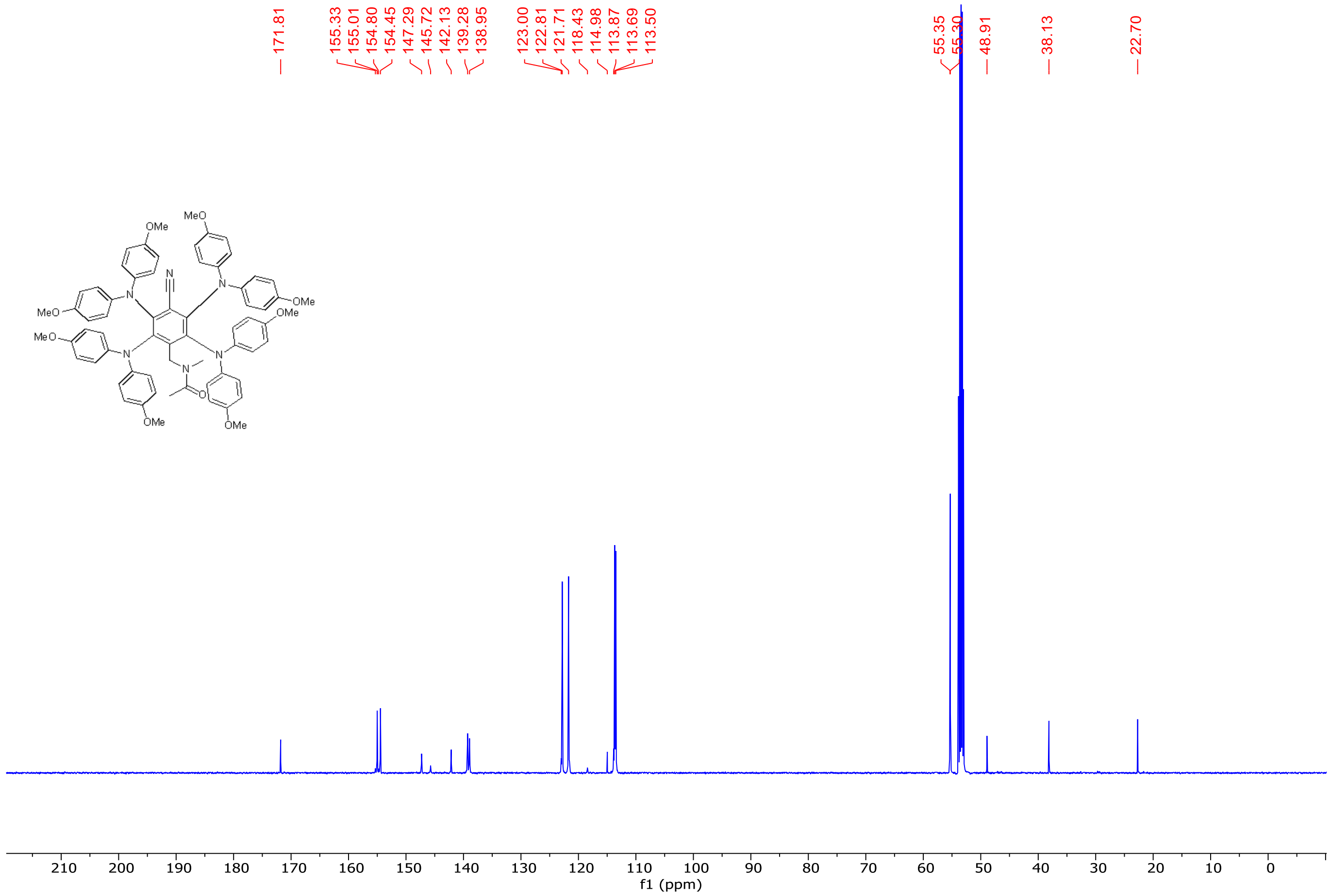

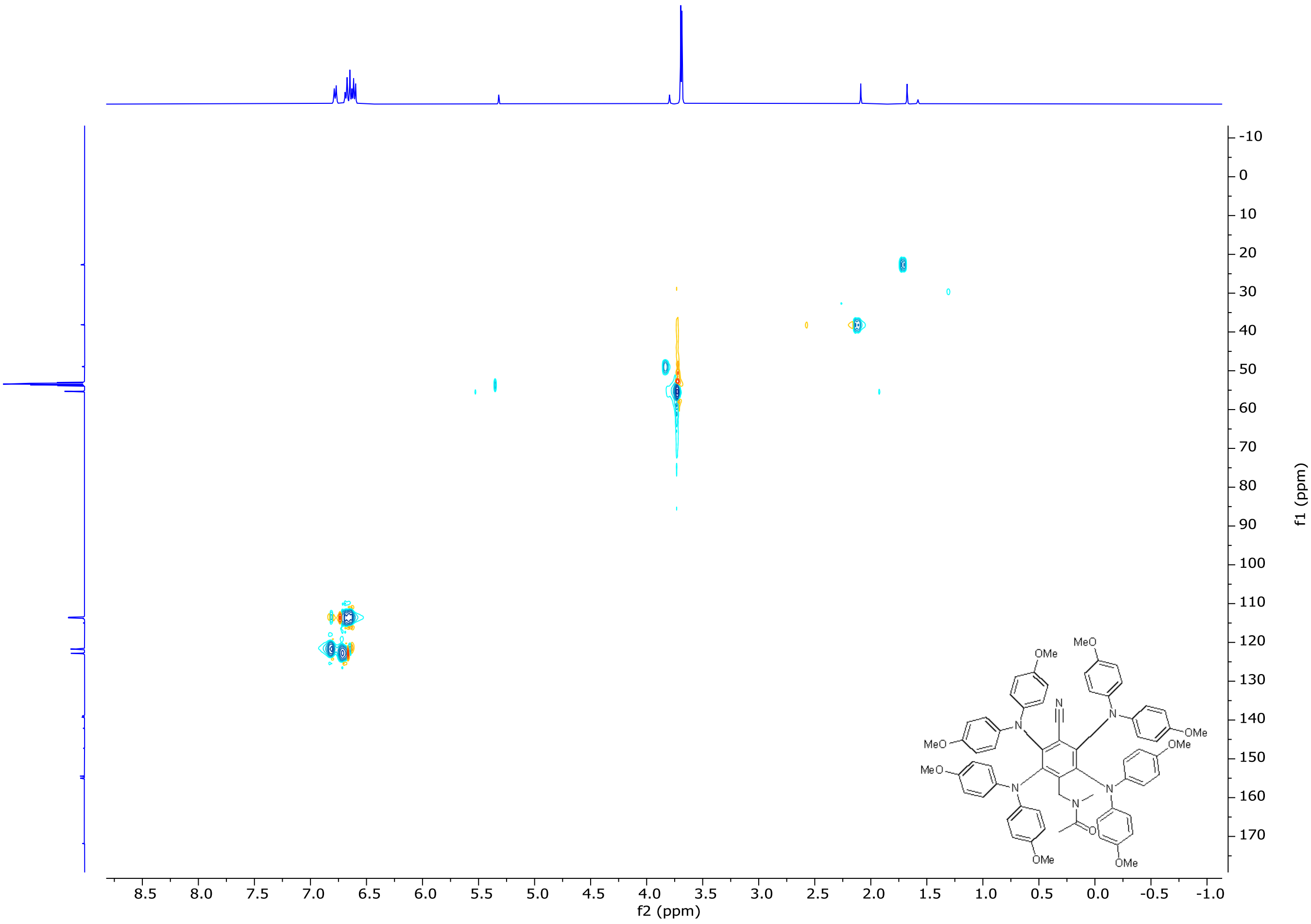

f2 (ppm)
f1 (ppm)
OMe
MeO
N
MeO
OMe
OMe
MeO
N
N
N
N
O
OMe
OMe

2,3,5,6-tetrakis(bis(4-methoxyphenyl)amino)benzonitrile (PC13-3)

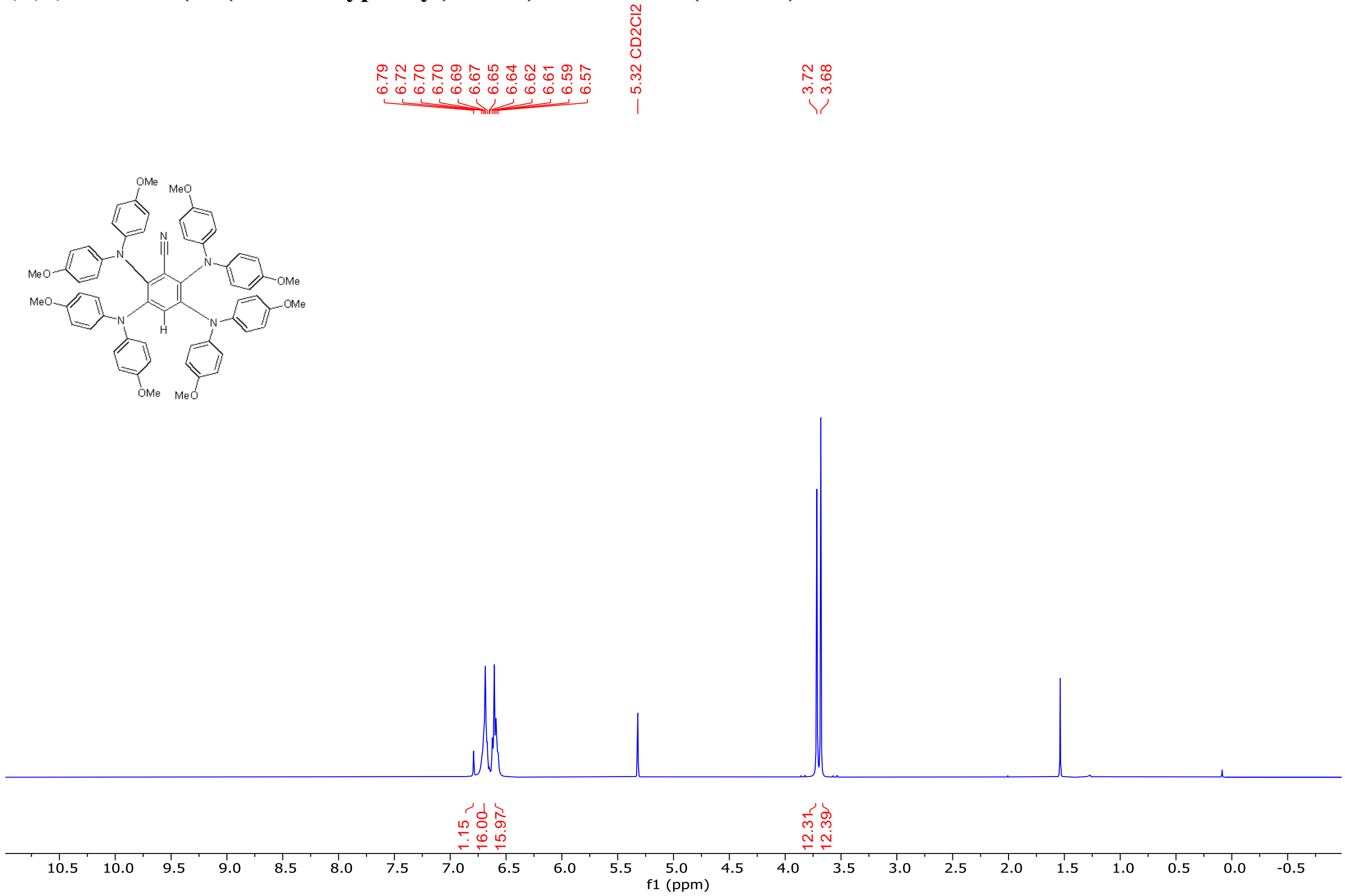

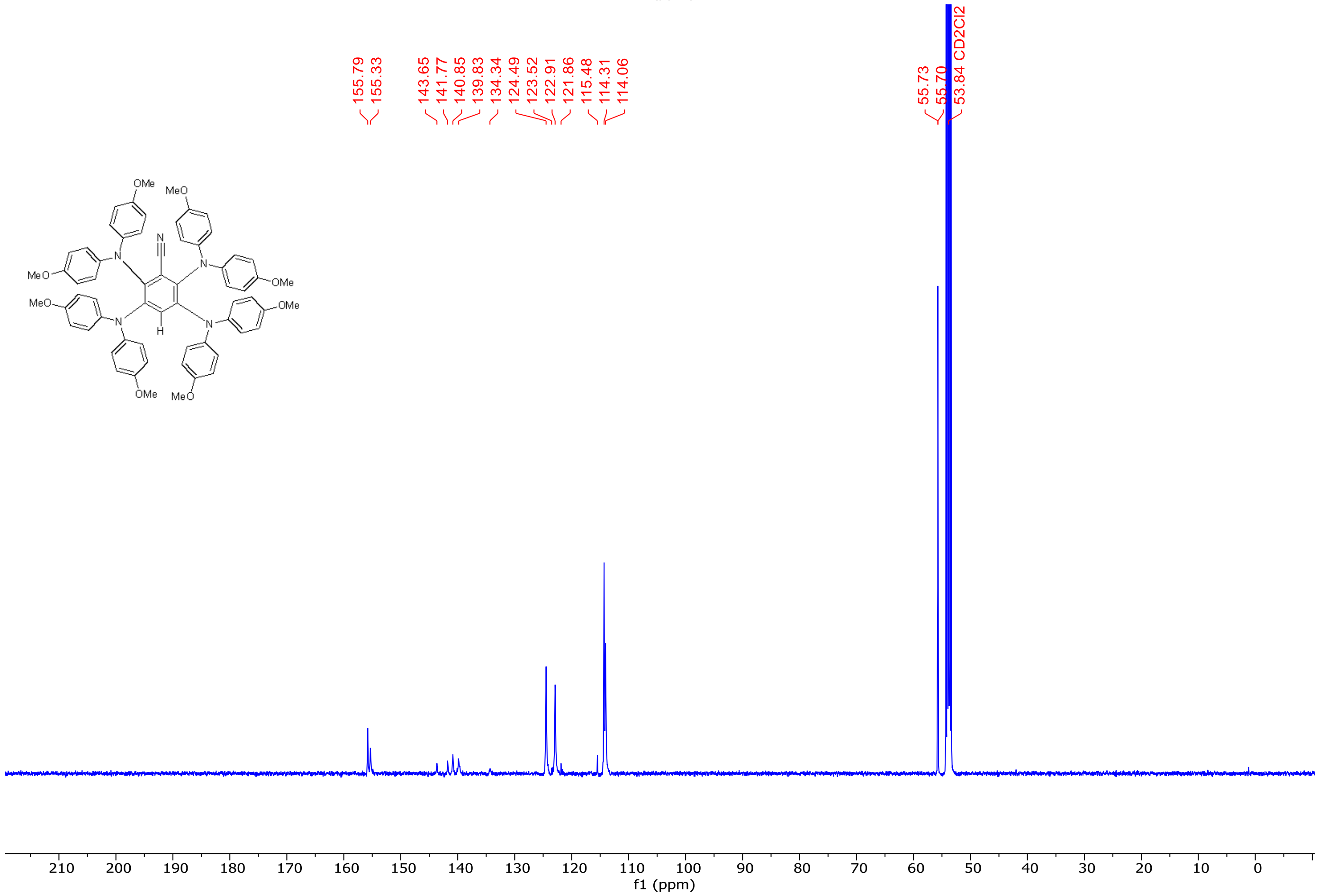

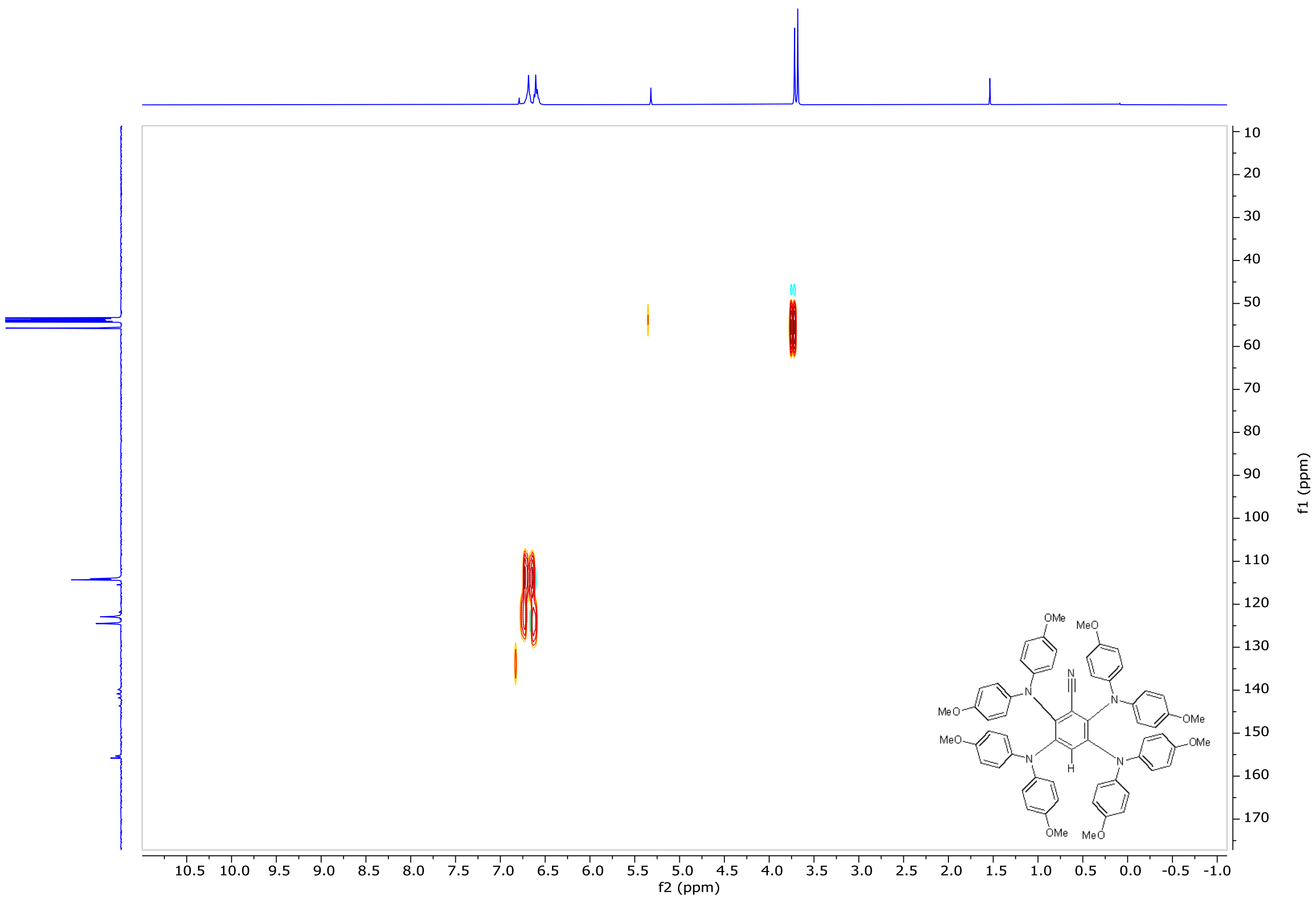
f2 (ppm)
f1 (ppm)
10.5 10.0 9.5 9.0 8.5 8.0 7.5 7.0 6.5 6.0 5.5 5.0 4.5 4.0 3.5 3.0 2.5 2.0 1.5 1.0 0.5 0.0 -0.5 -1.0
10 20 30 40 50 60 70 80 90 100 110 120 130 140 150 160 170
OMe
MeO
N
H

5^{t}BuBN--- 2,3,4,5,6-pentakis(3,6-di-*tert*-butyl-9H-carbazol-9-yl)benzonitrile (PC14)

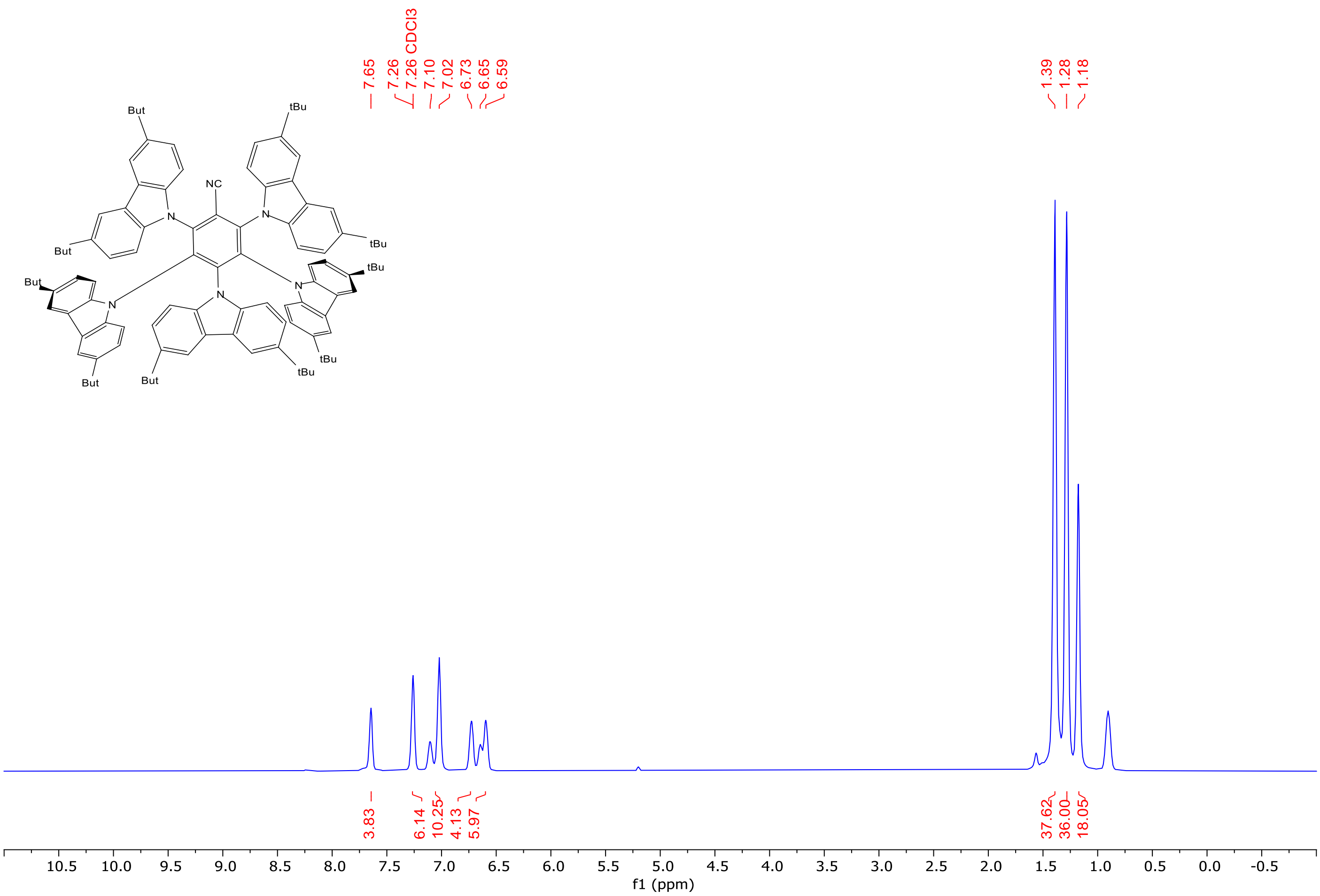

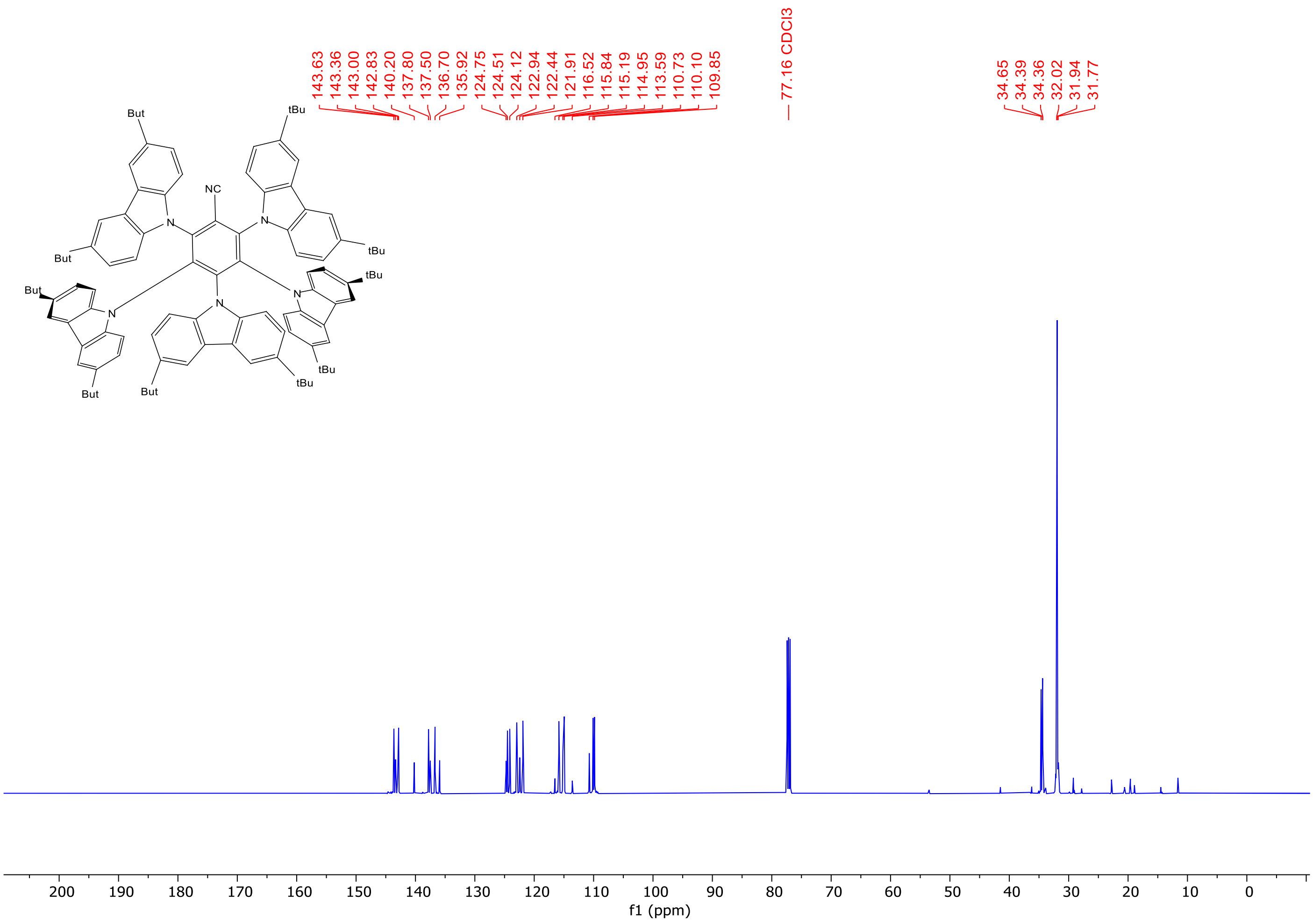

4tBuCzINN--- 2,3,5,6-tetrakis(3,6-di-*tert*-butyl-9*H*-carbazol-9-yl)isonicotinonitrile (PC15)

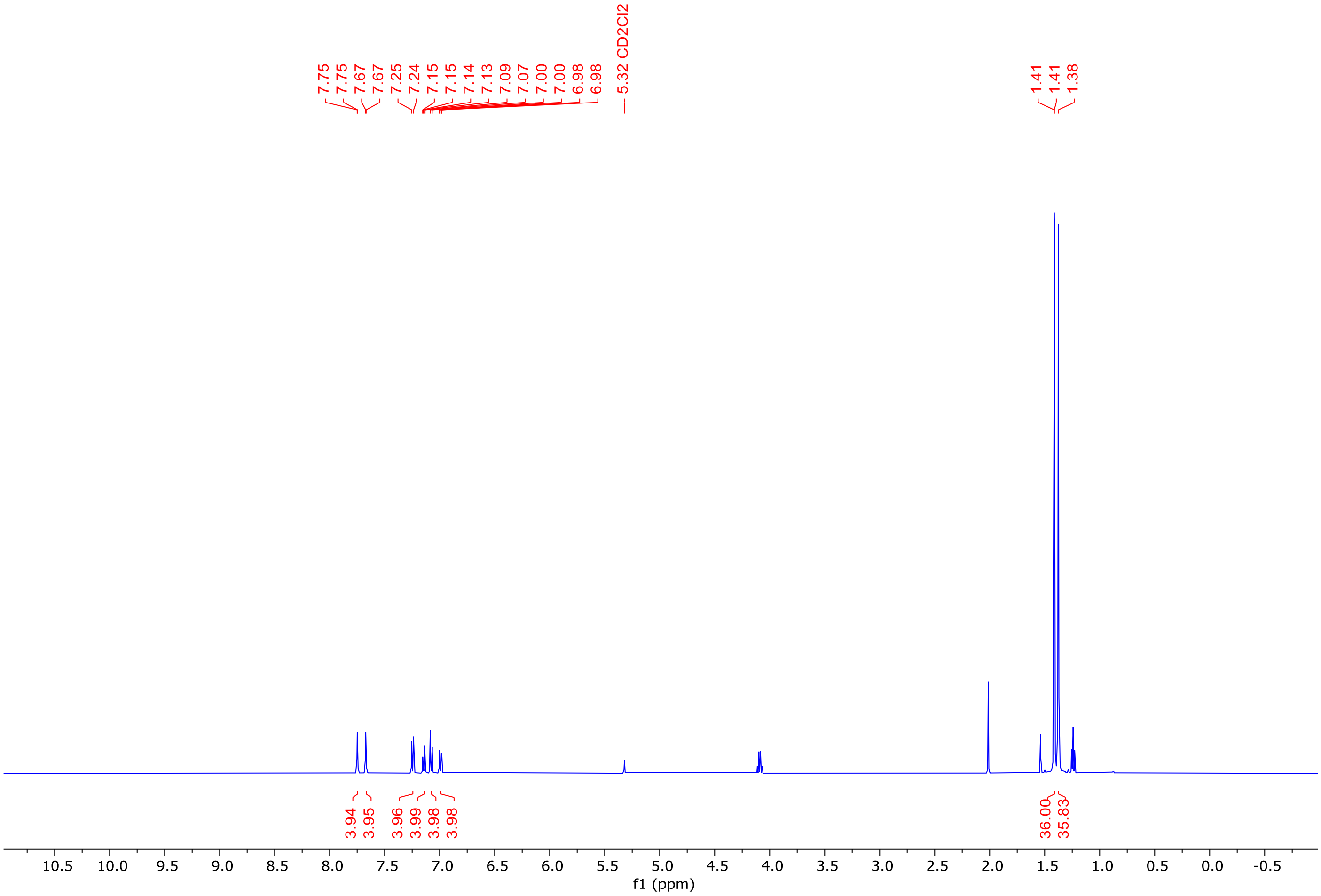

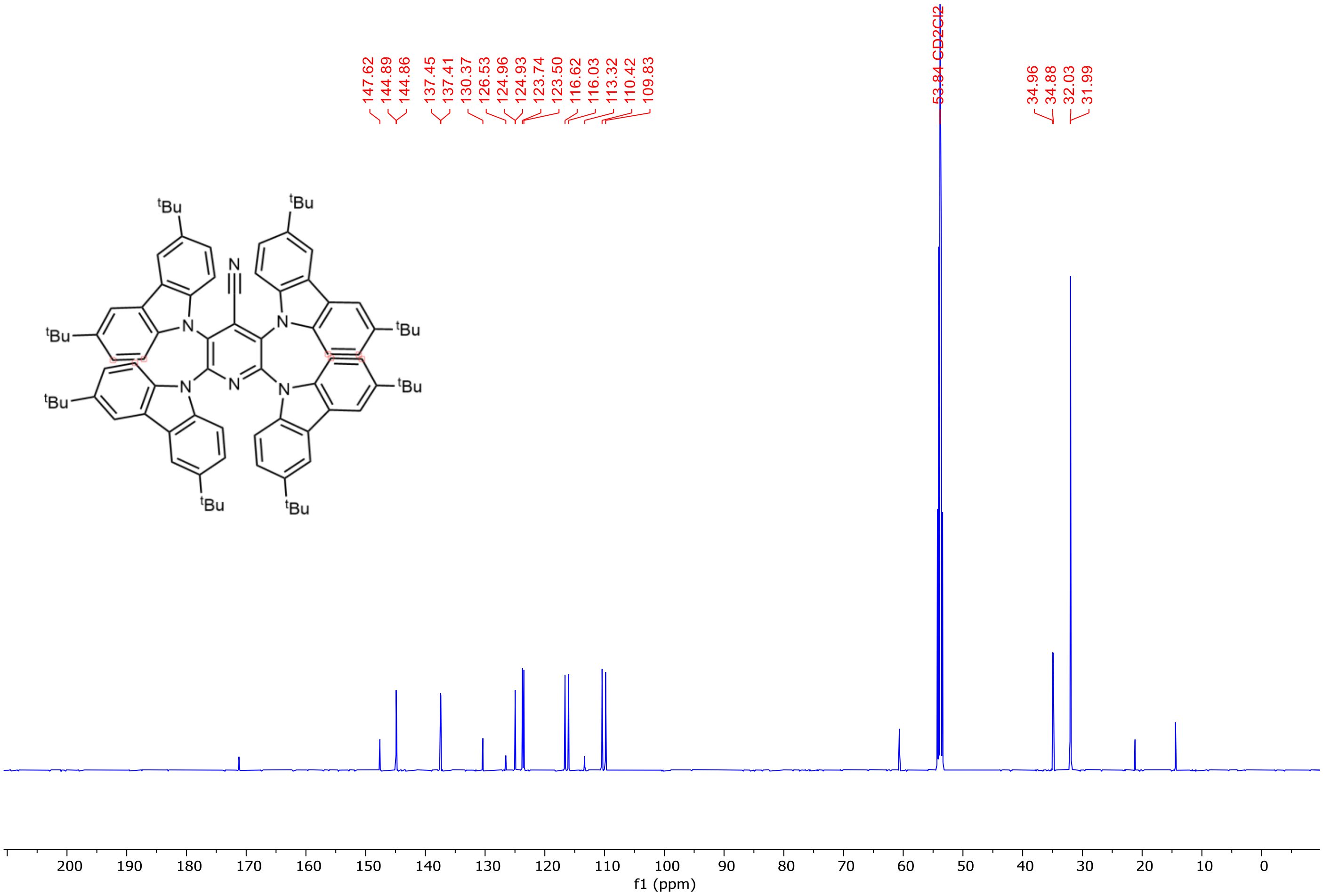

4DPAINN--- 2,3,5,6-tetrakis(diphenylamino)isonicotinonitrile (PC16)

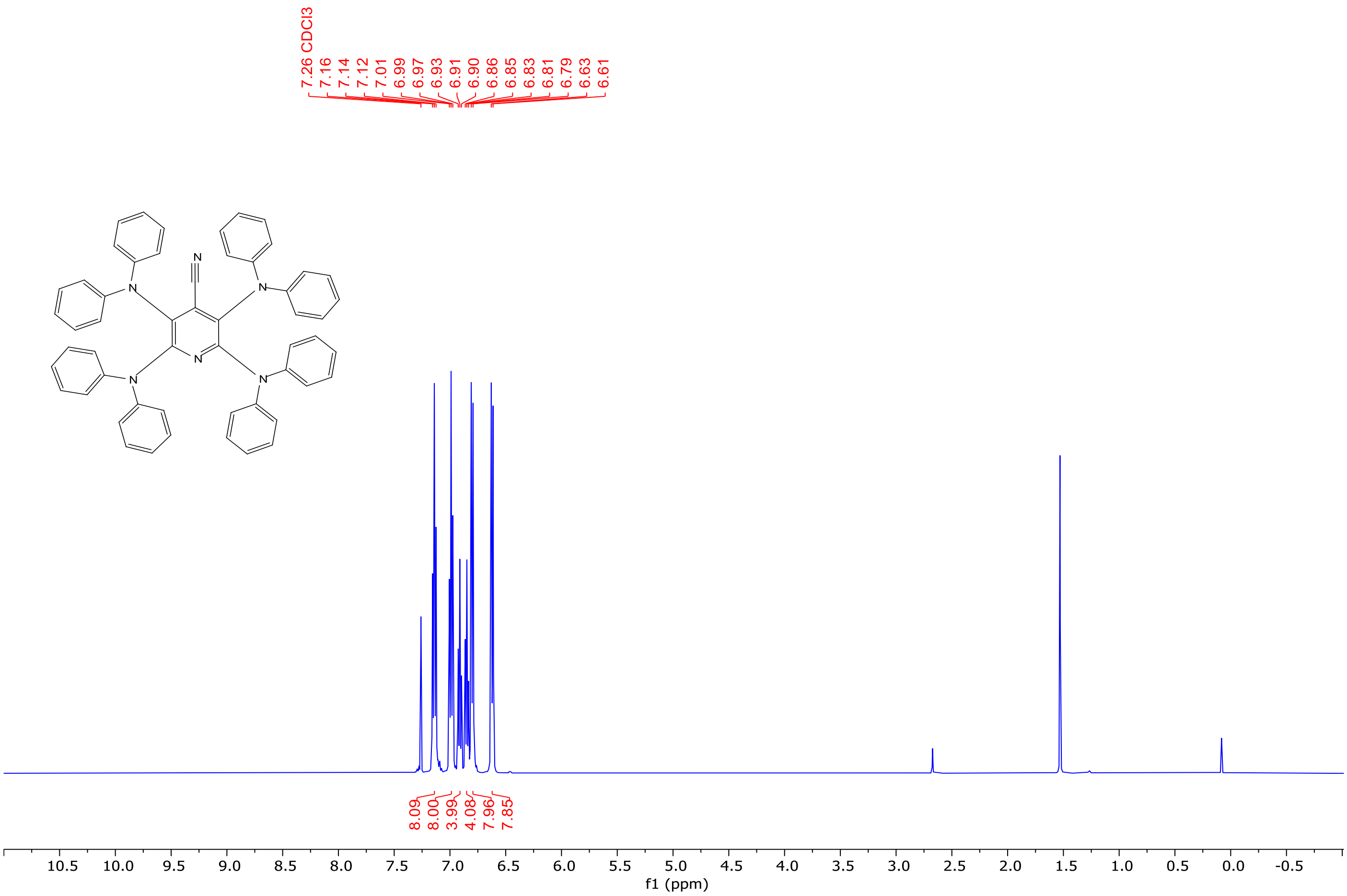

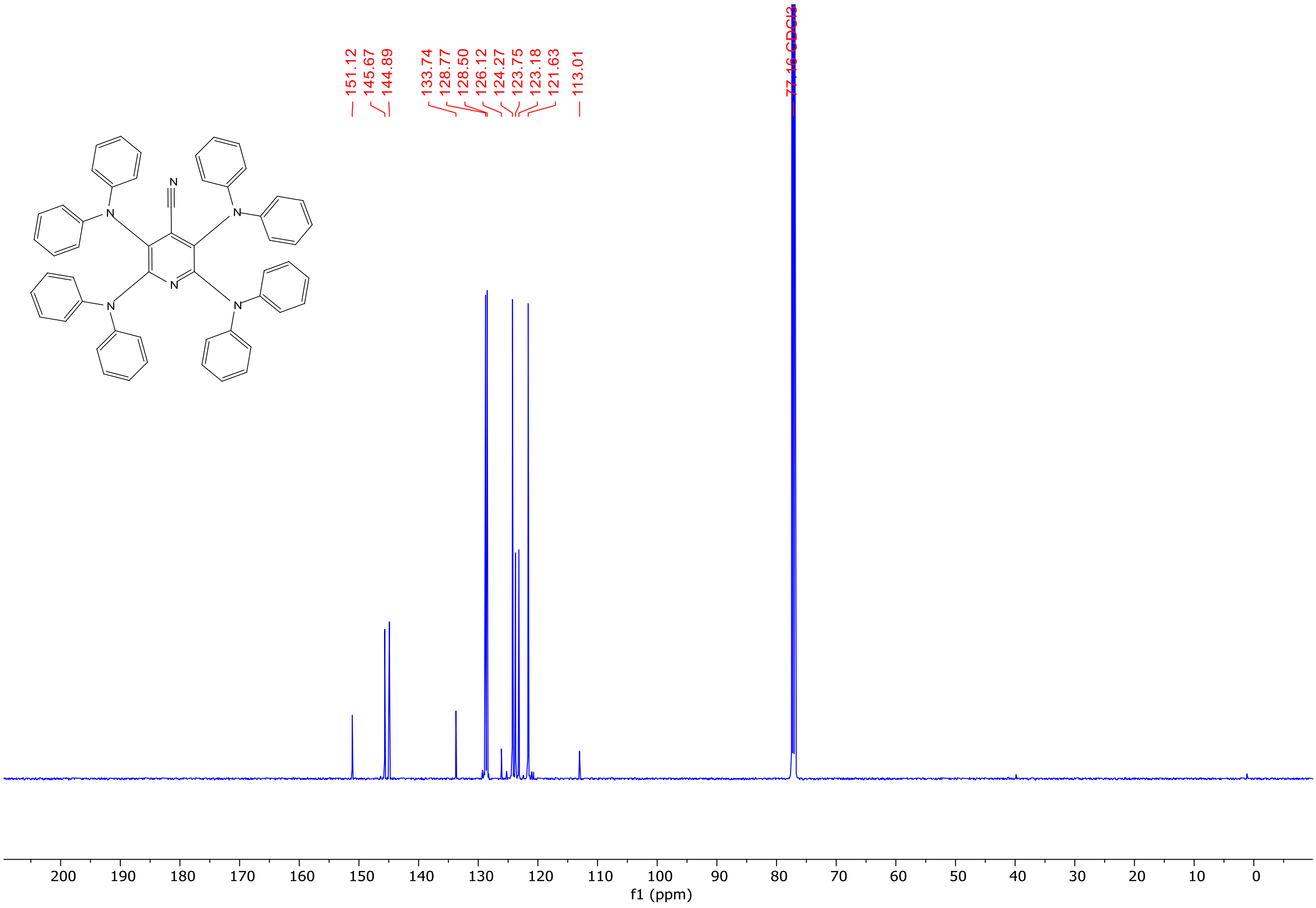

4CzPN---3,4,5,6-tetra(9*H*-carbazol-9-yl)phthalonitrile (PC17)

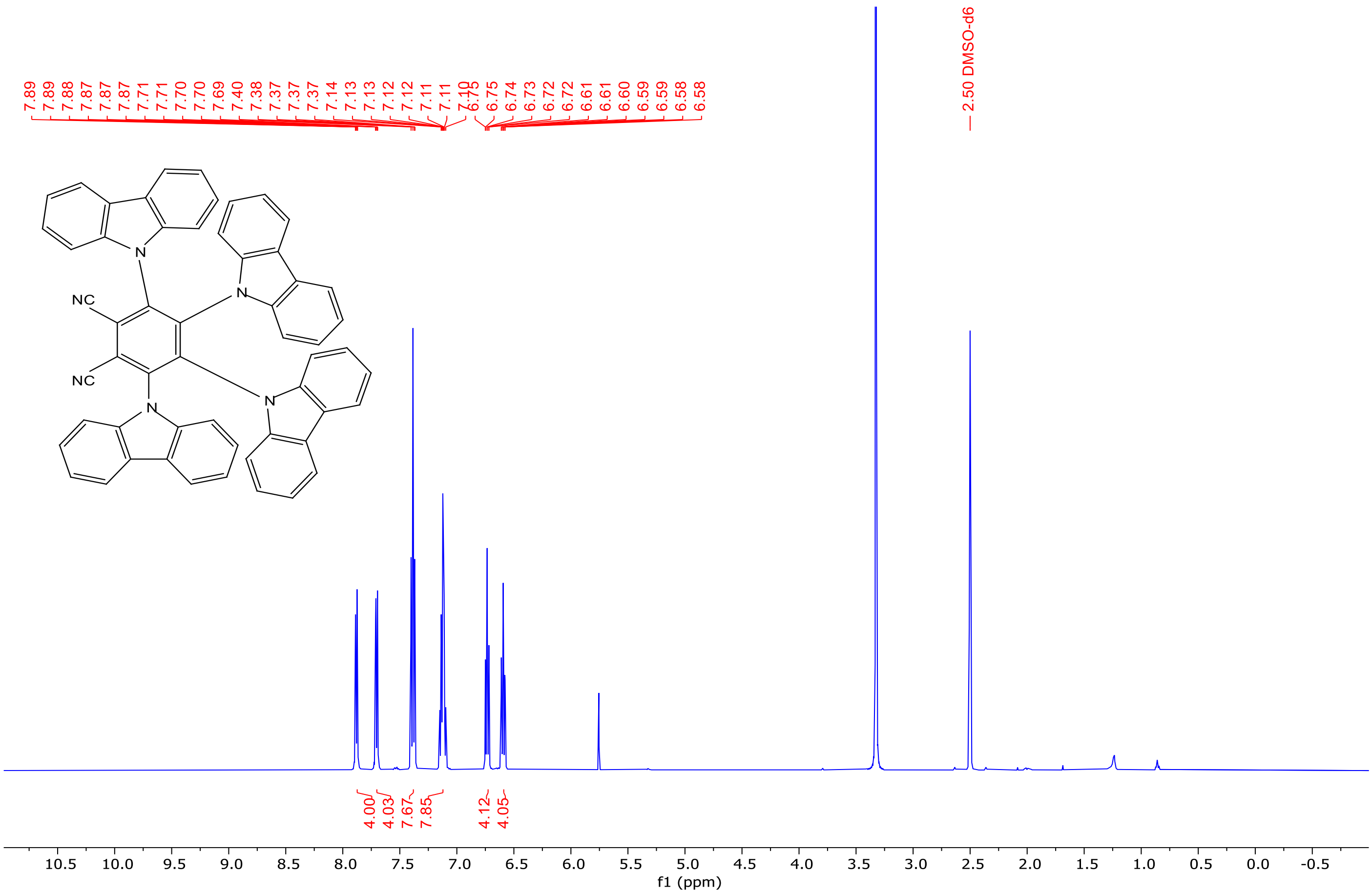

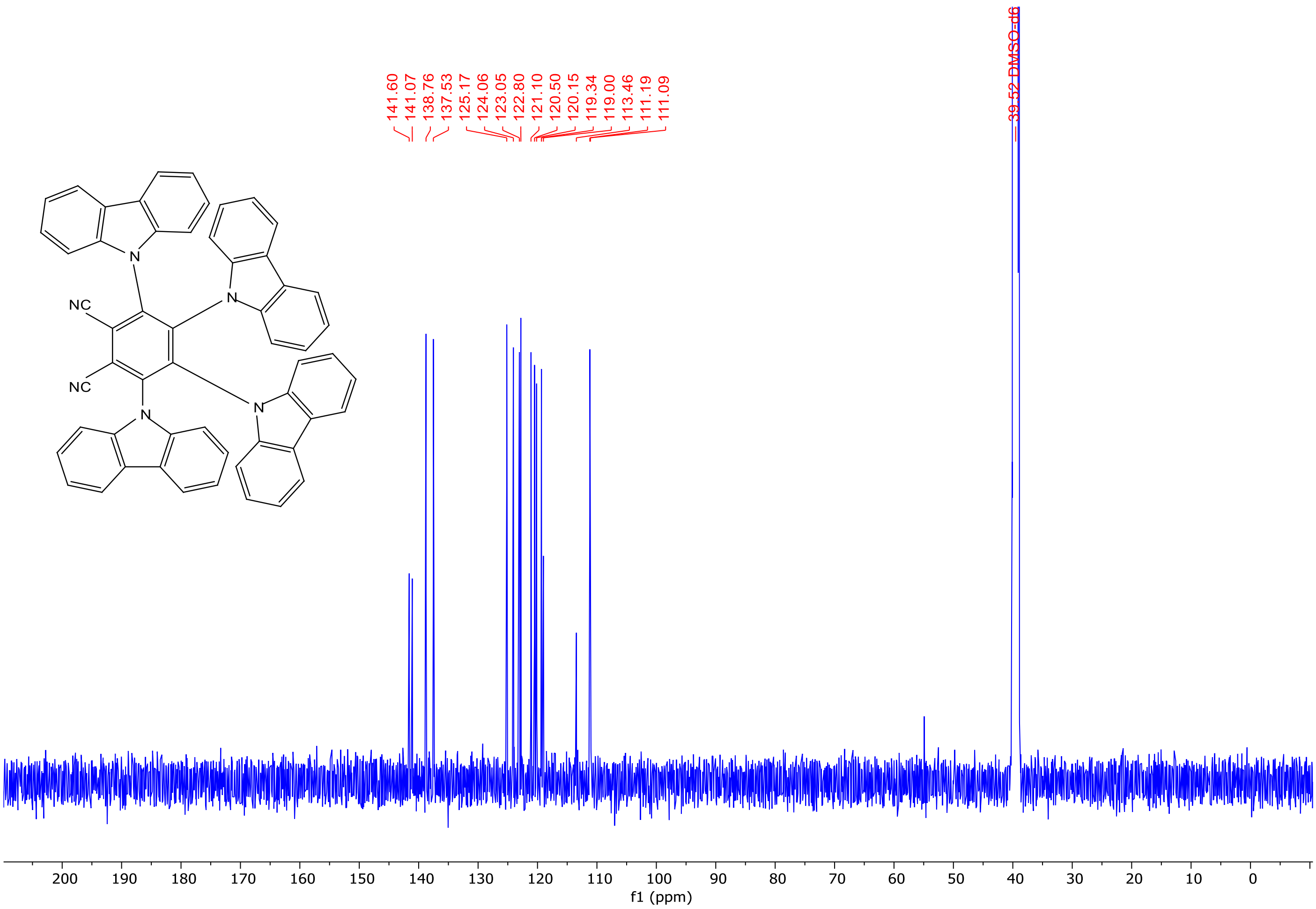

4^{t}BuCzPN---3,4,5,6-tetrakis(3,6-di-*tert*-butyl-9*H*-carbazol-9-yl)phthalonitrile (PC18)

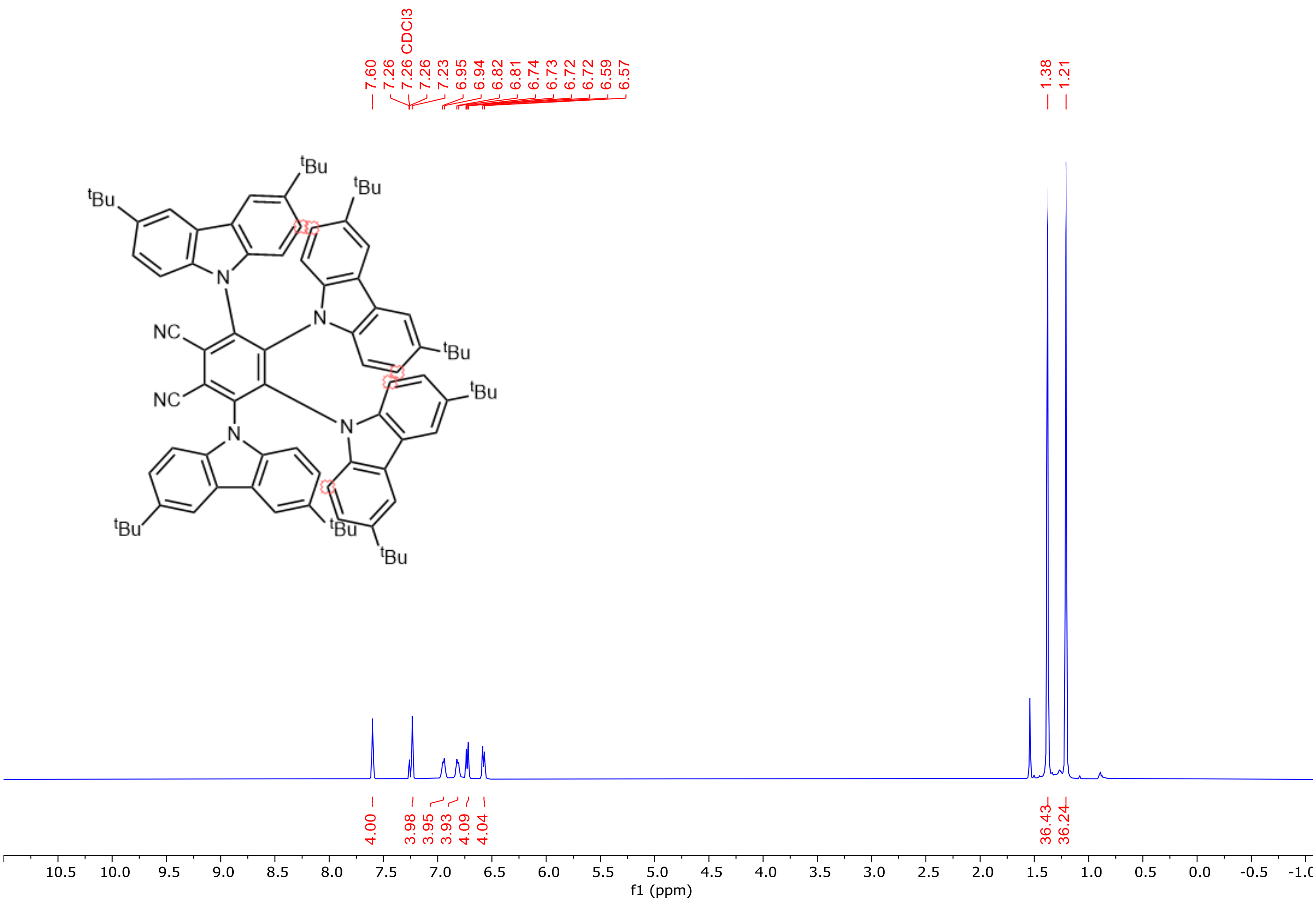

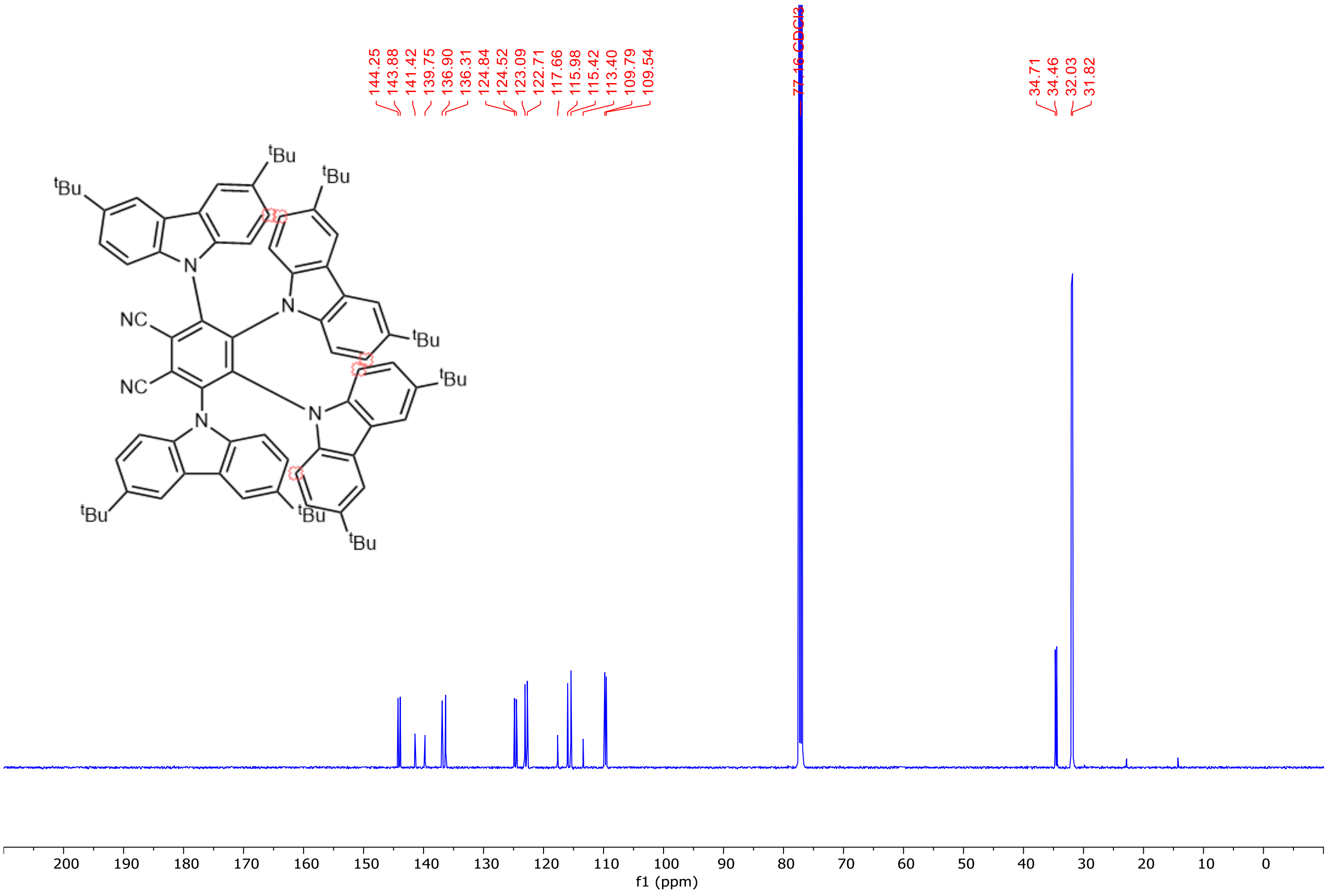

4DPAPN---3,4,5,6-tetrakis(diphenylamino)phthalonitrile (PC19)

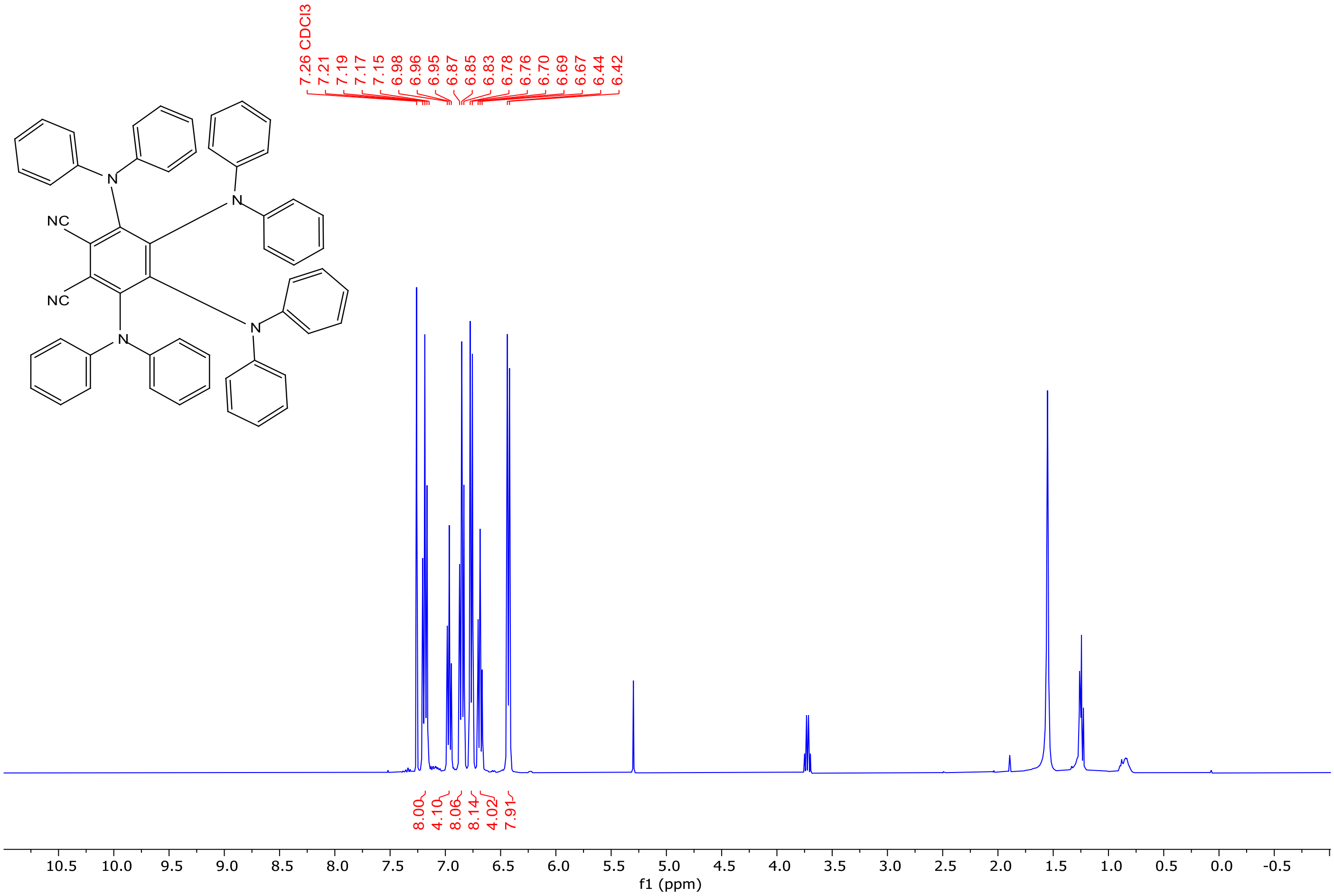

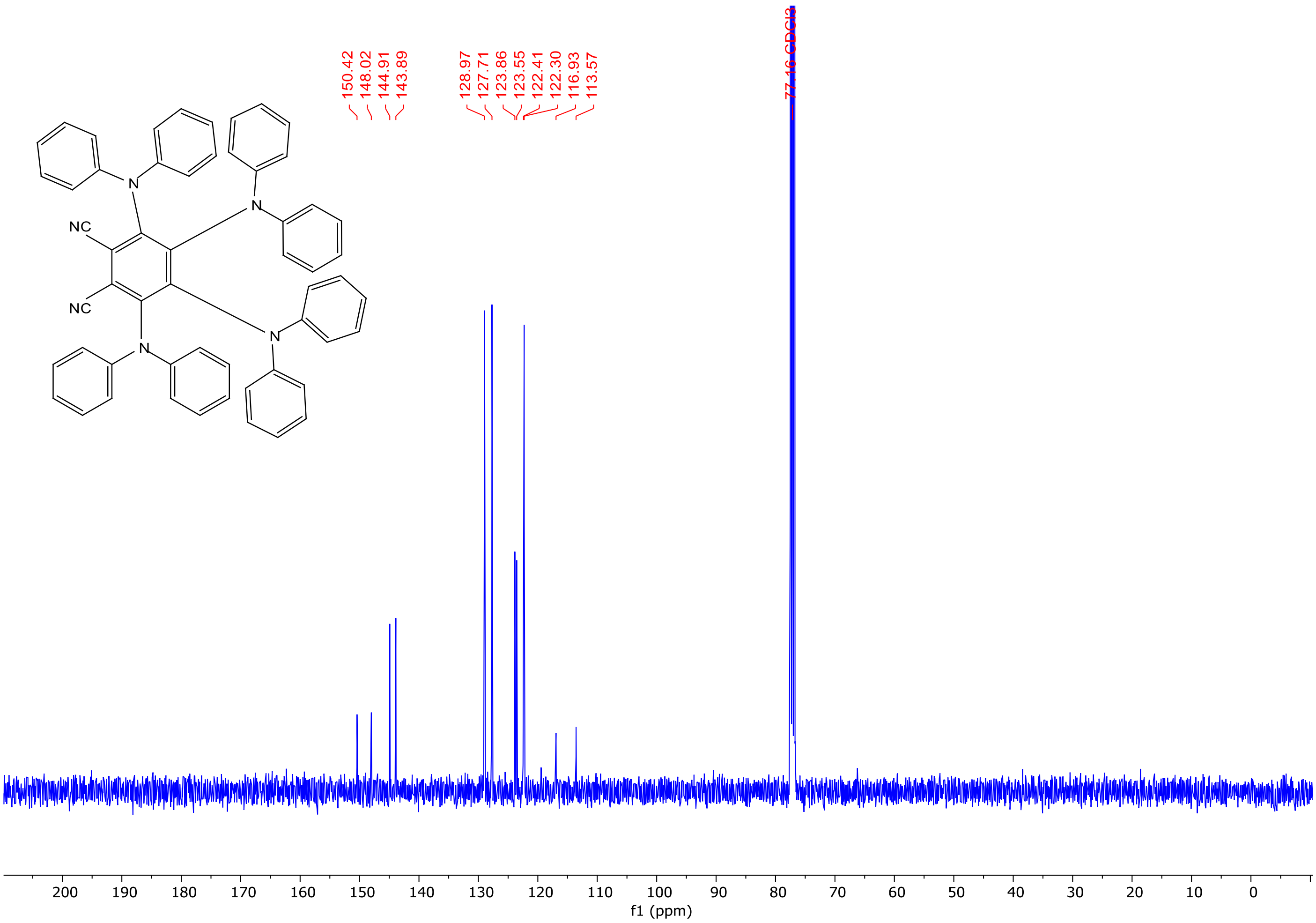

4^{t}BuCzTPN--- 2,3,5,6-tetrakis(3,6-di-*tert*-butyl-9*H*-carbazol-9-yl)terephthalonitrile (PC20)

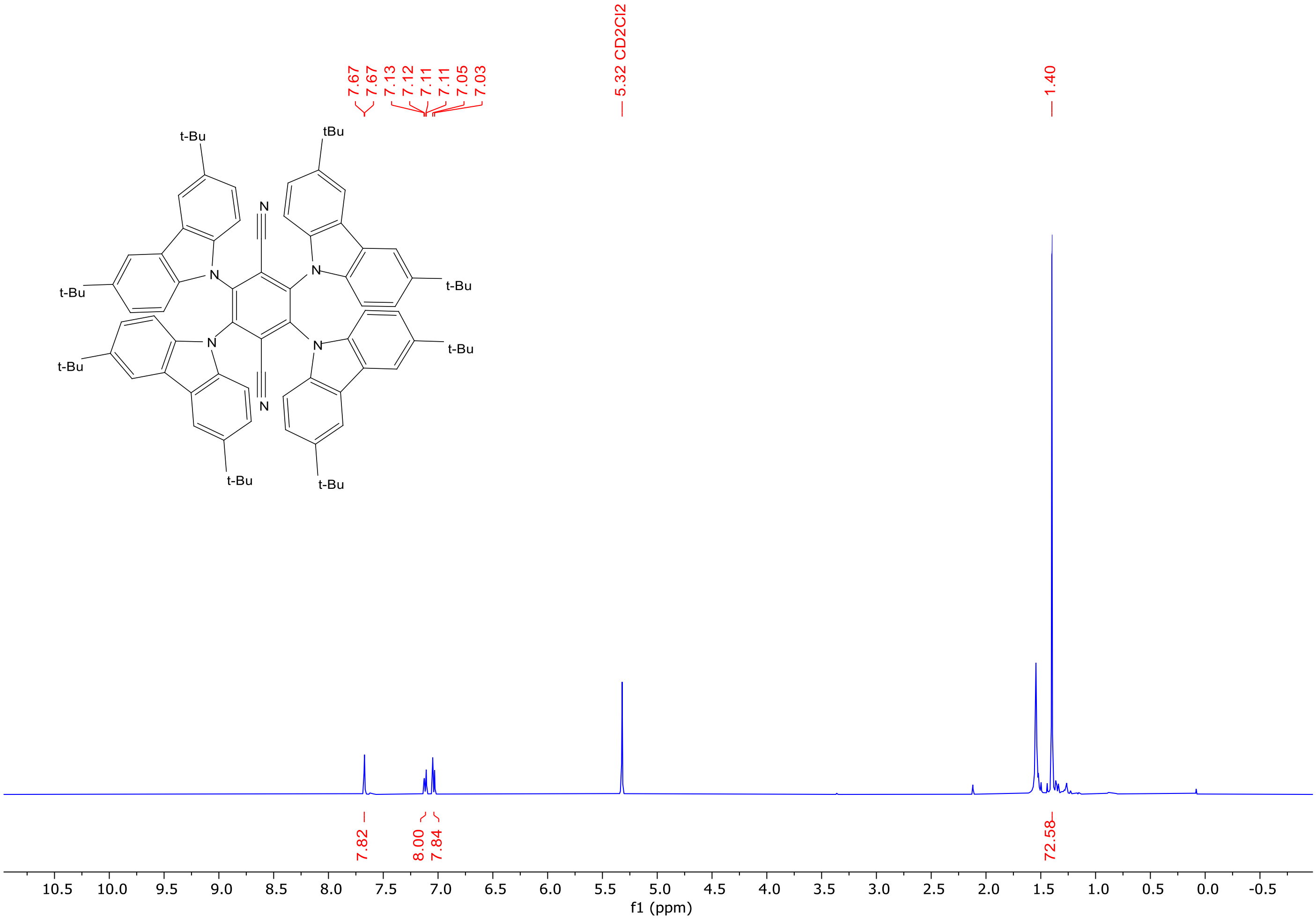

4MeOCzTPN---2,3,5,6-tetrakis(3,6-dimethoxy-9*H*-carbazol-9-yl)terephthalonitrile (PC21)

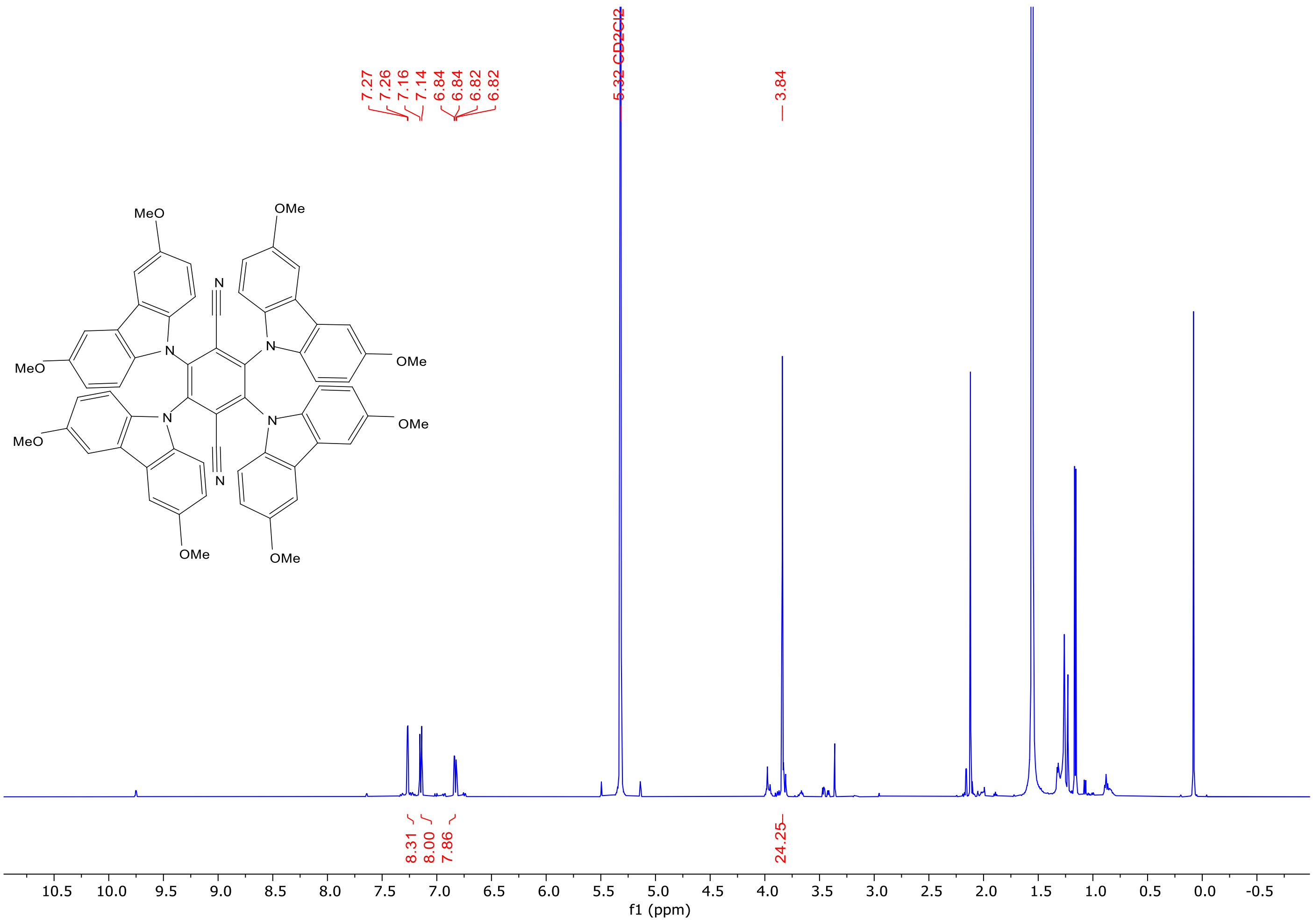

4PhDPATPN---2,3,5,6-tetrakis(di([1,1'-biphenyl]-4-yl)amino)terephthalonitrile (PC22)

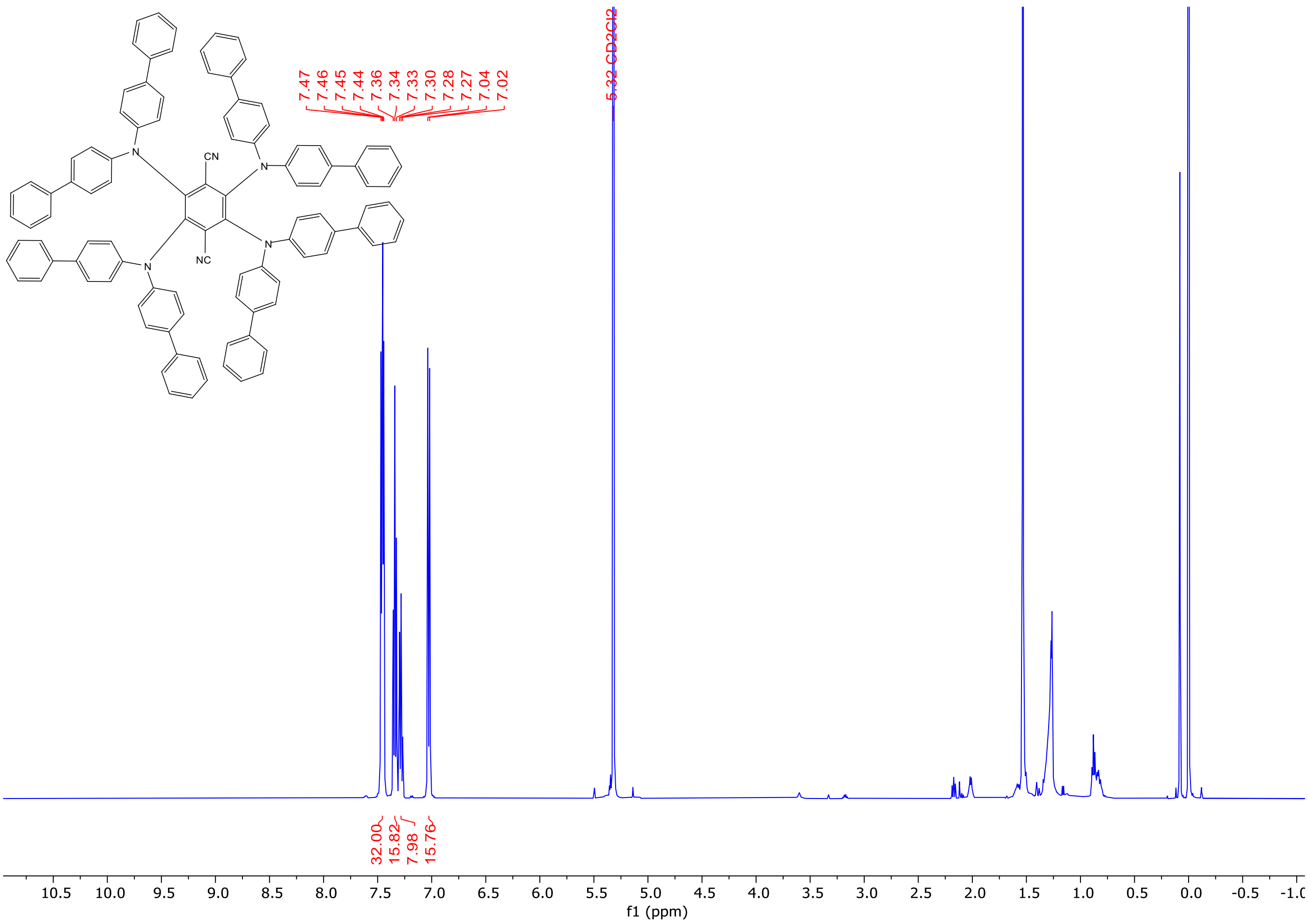

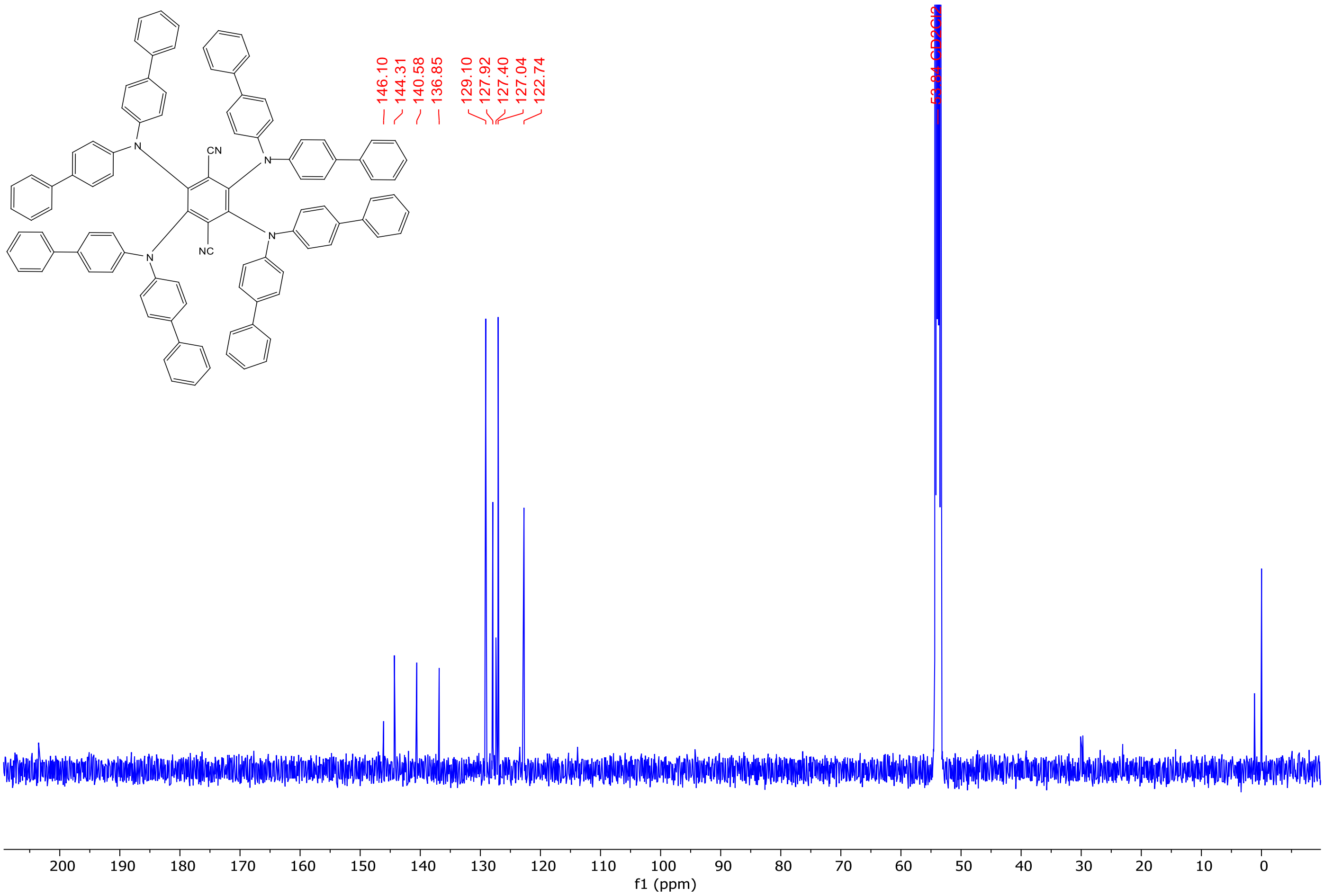

4tBuDPATPN---2,3,5,6-tetrakis(bis(4-(*tert*-butyl)phenyl)amino)terephthalonitrile (PC23)

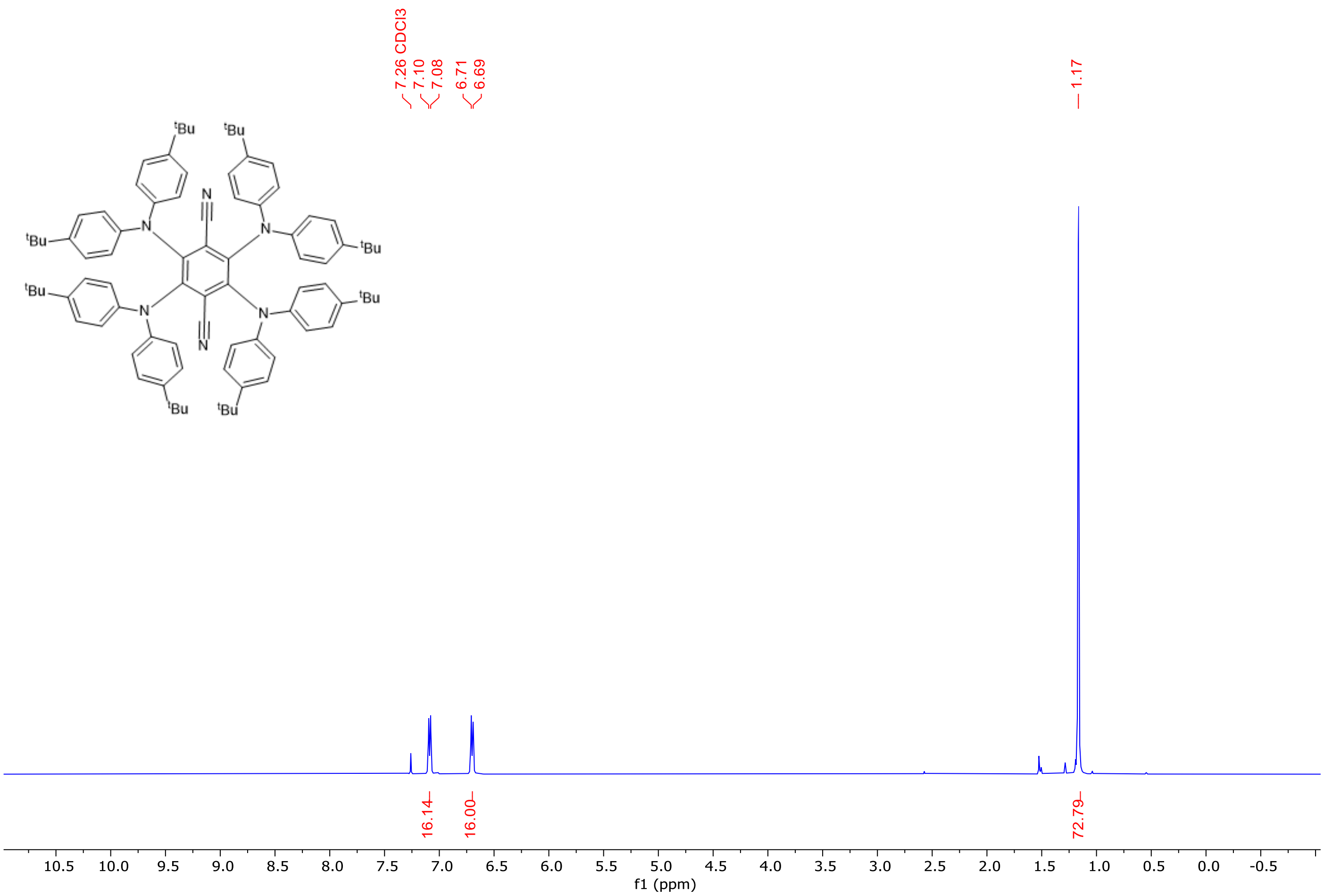

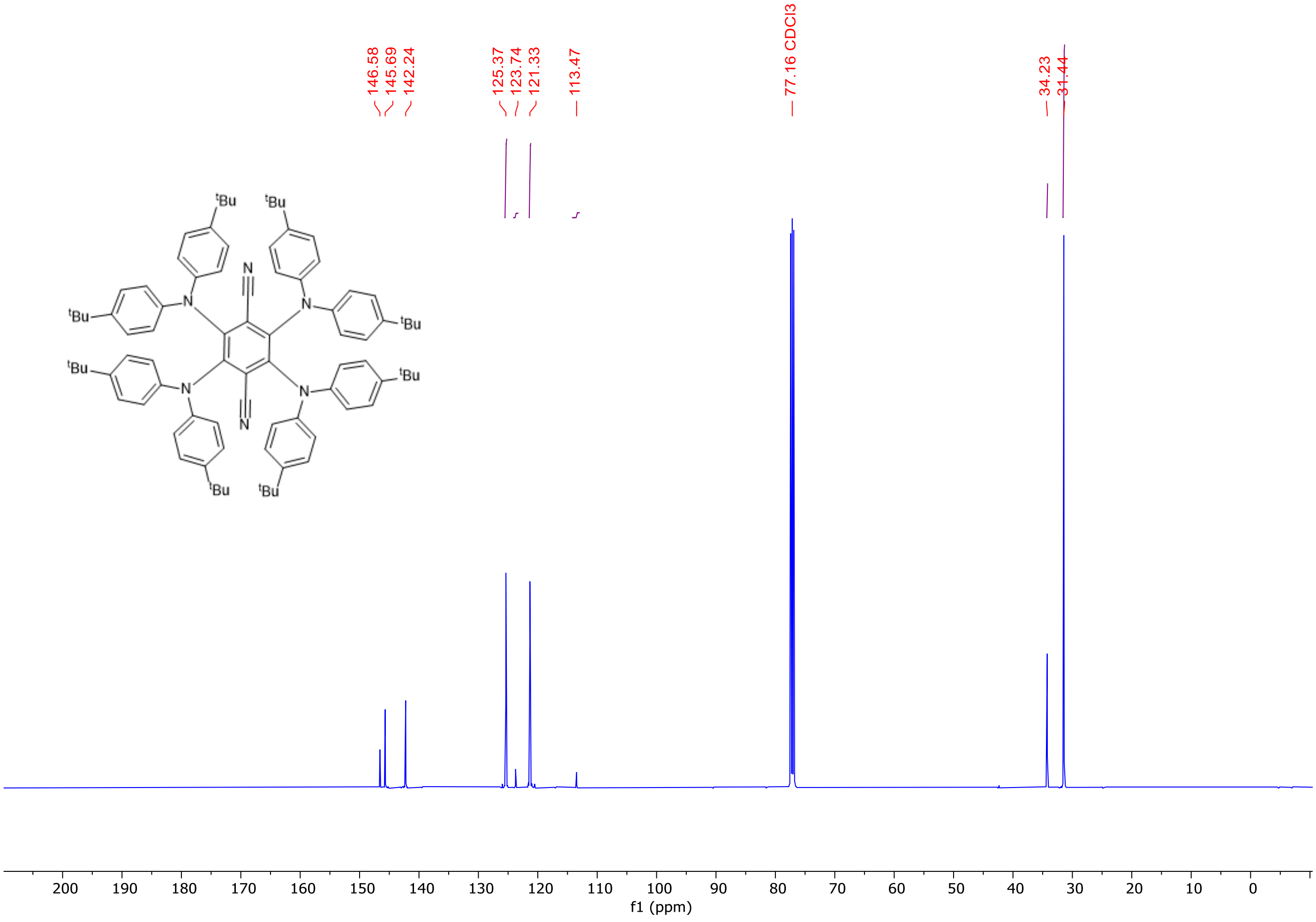

4(Ar^{4}DPA)TPN---2,3,5,6-tetrakis(bis(3',5'-dimethoxy-[1,1'-biphenyl]-4-yl)amino)terephthalonitrile (PC24)

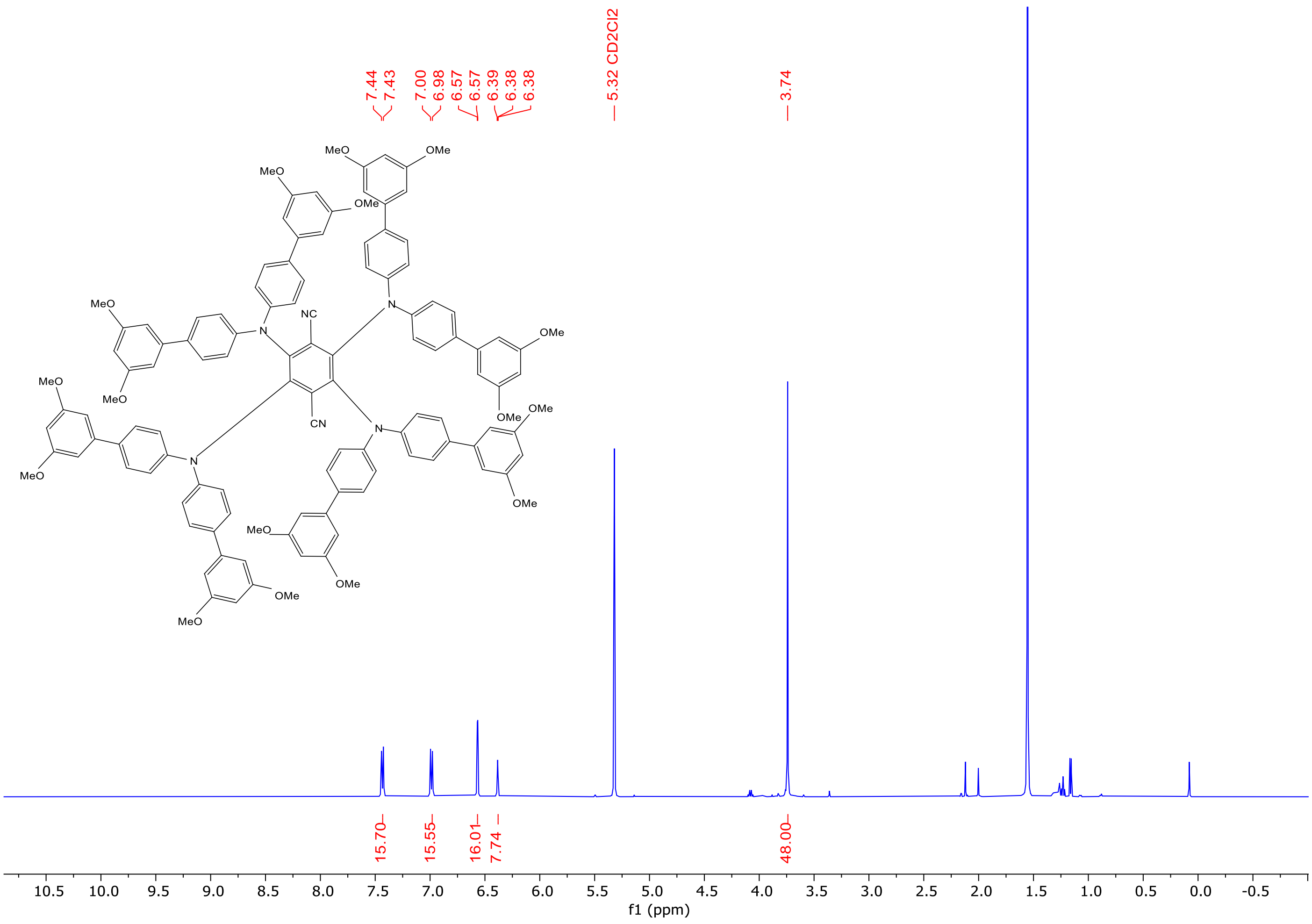

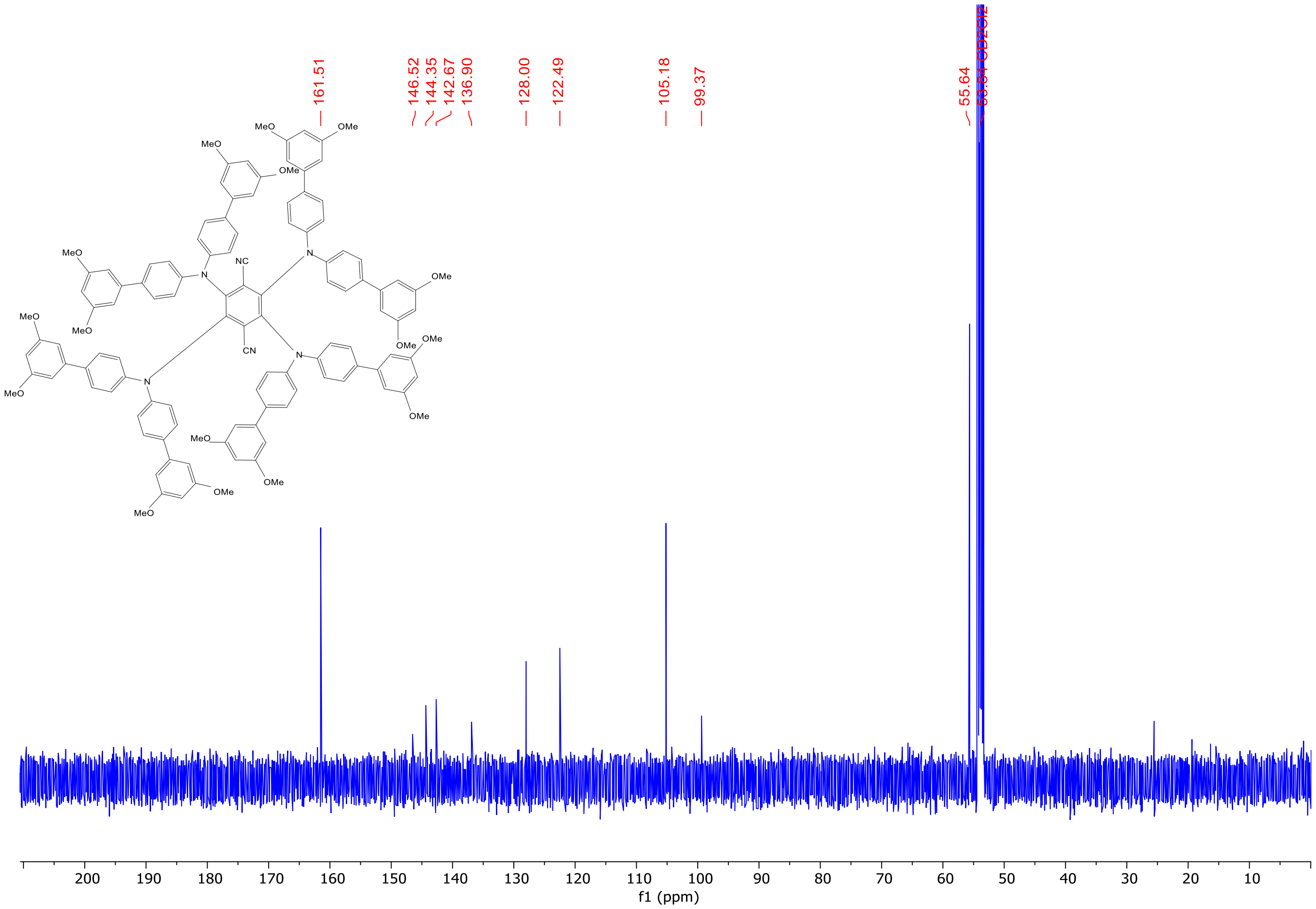

161.51
146.52
144.35
142.67
136.90
128.00
122.49
105.18
99.37
55.64
f1 (ppm)

4(Ar⁵PhDPA)TPN---2,3,5,6-tetrakis(bis(3',4',5'-trimethoxy-[1,1'-biphenyl]-4-yl)amino)terephthalonitrile (PC25)

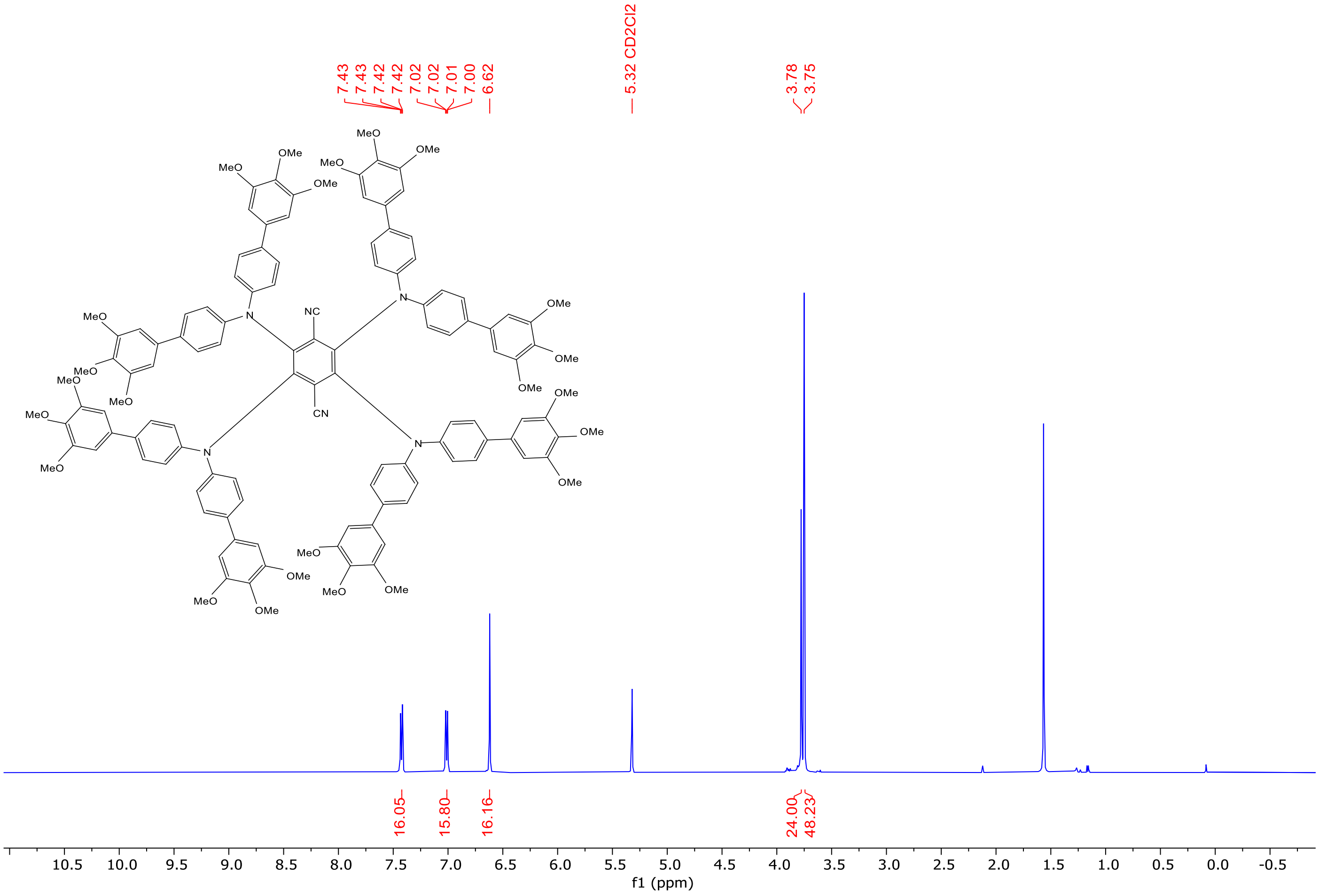

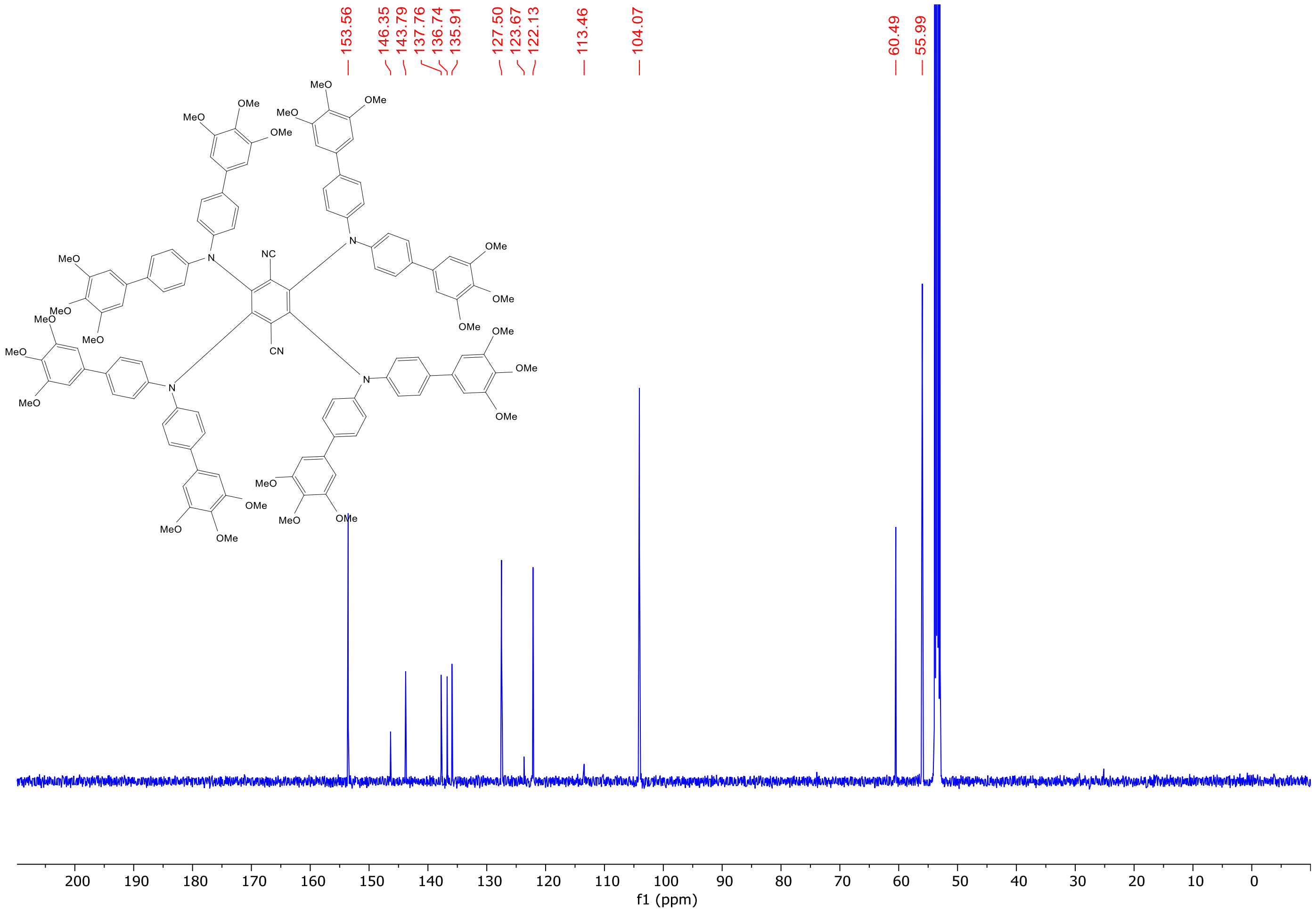

4ClCzIPN---2,4,5,6-tetrakis(3,6-dichloro-9*H*-carbazol-9-yl)isophthalonitrile (PC26)

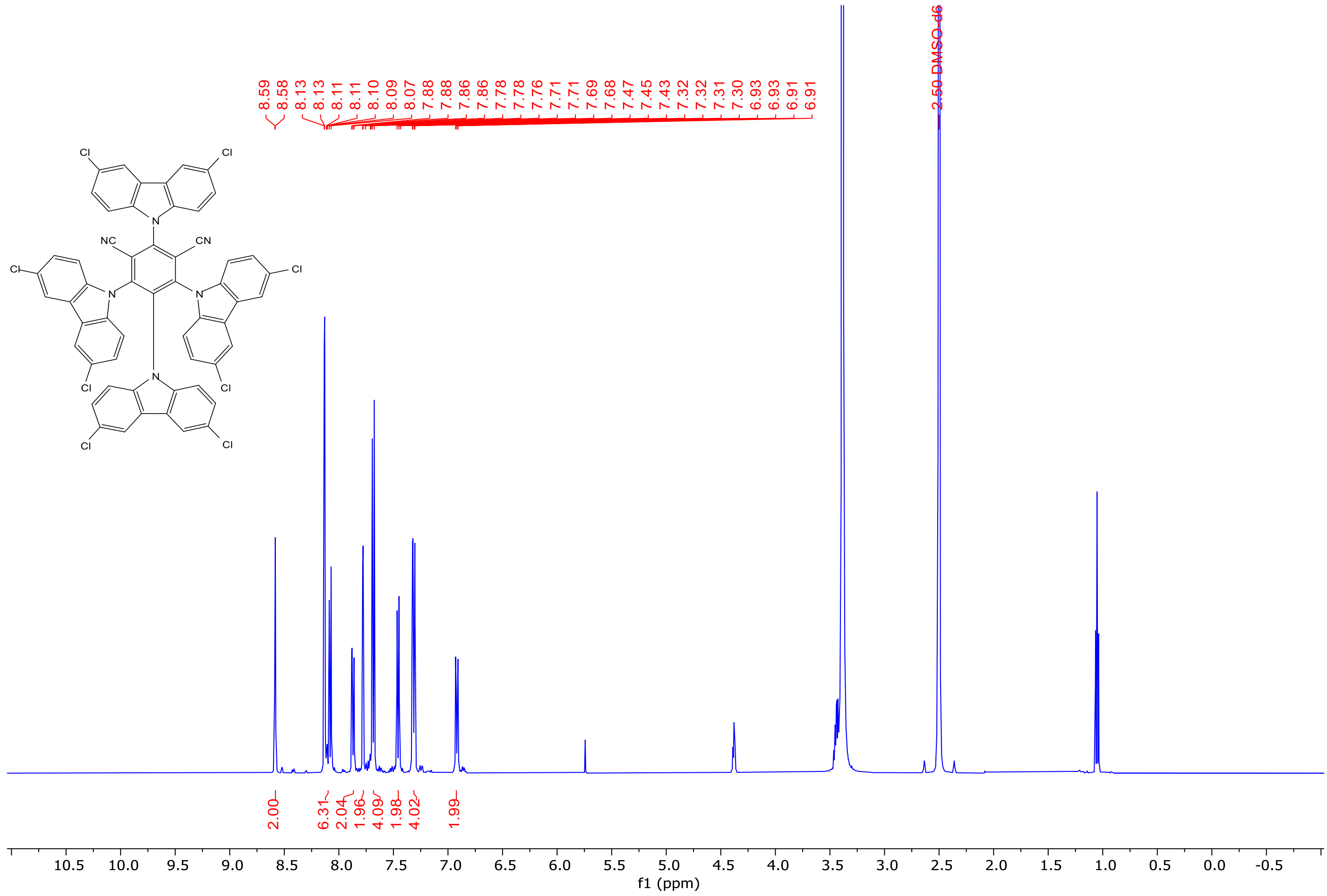

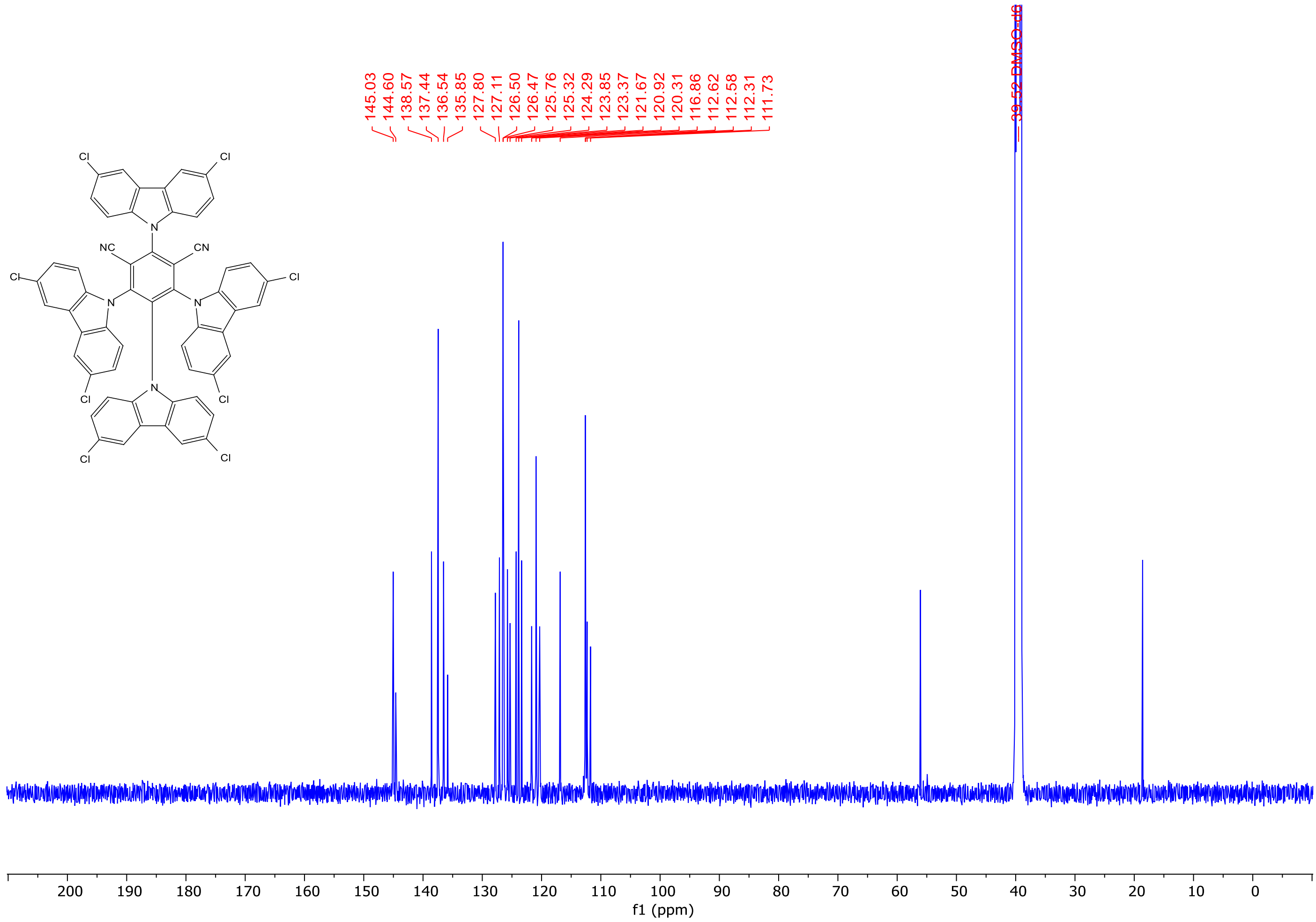

4(Ar[3]DPA)IPN---2,4,5,6-tetrakis(3,6-bis(4-(trifluoromethyl)phenyl)-9*H*-carbazol-9-yl)isophthalonitrile (PC27)

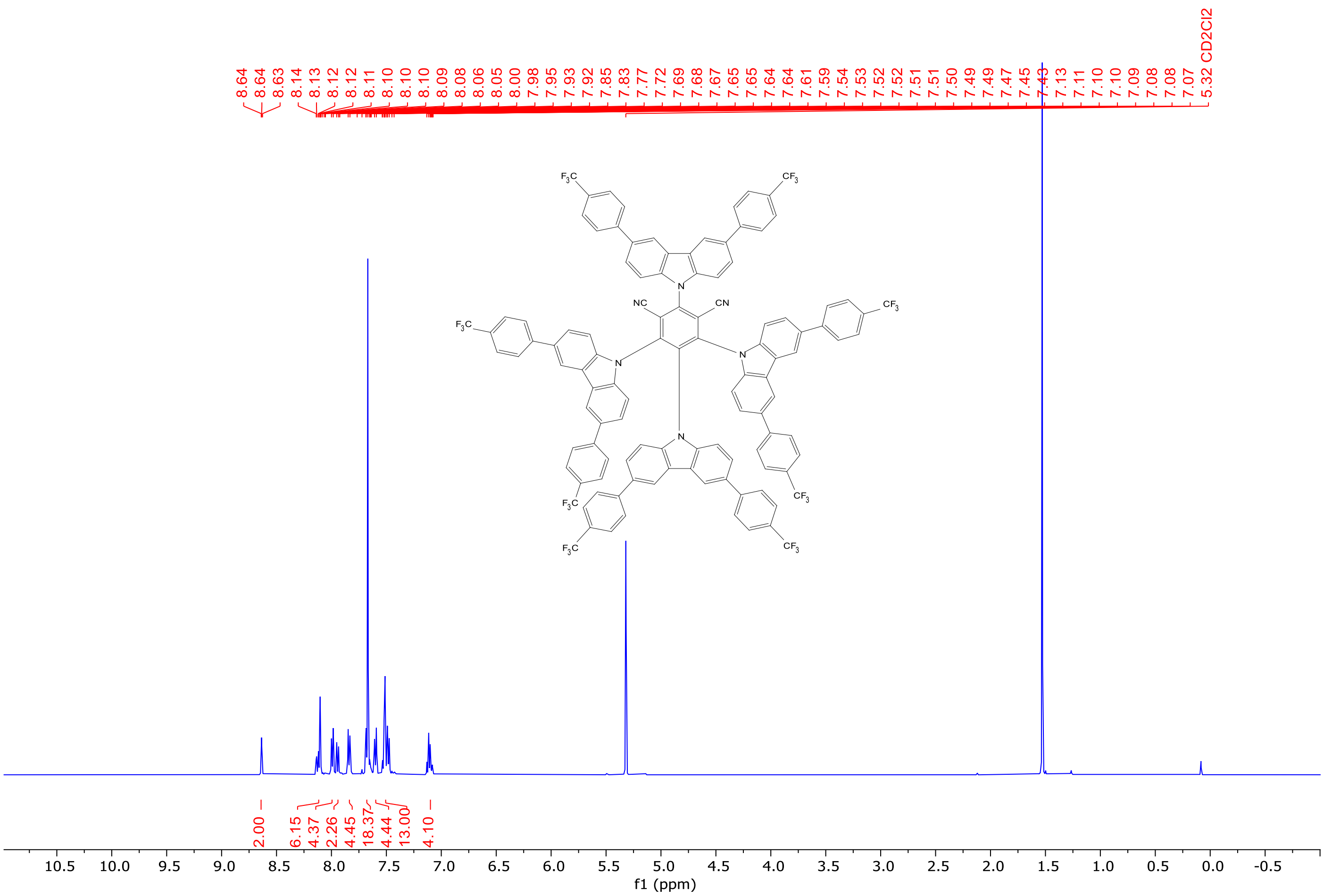

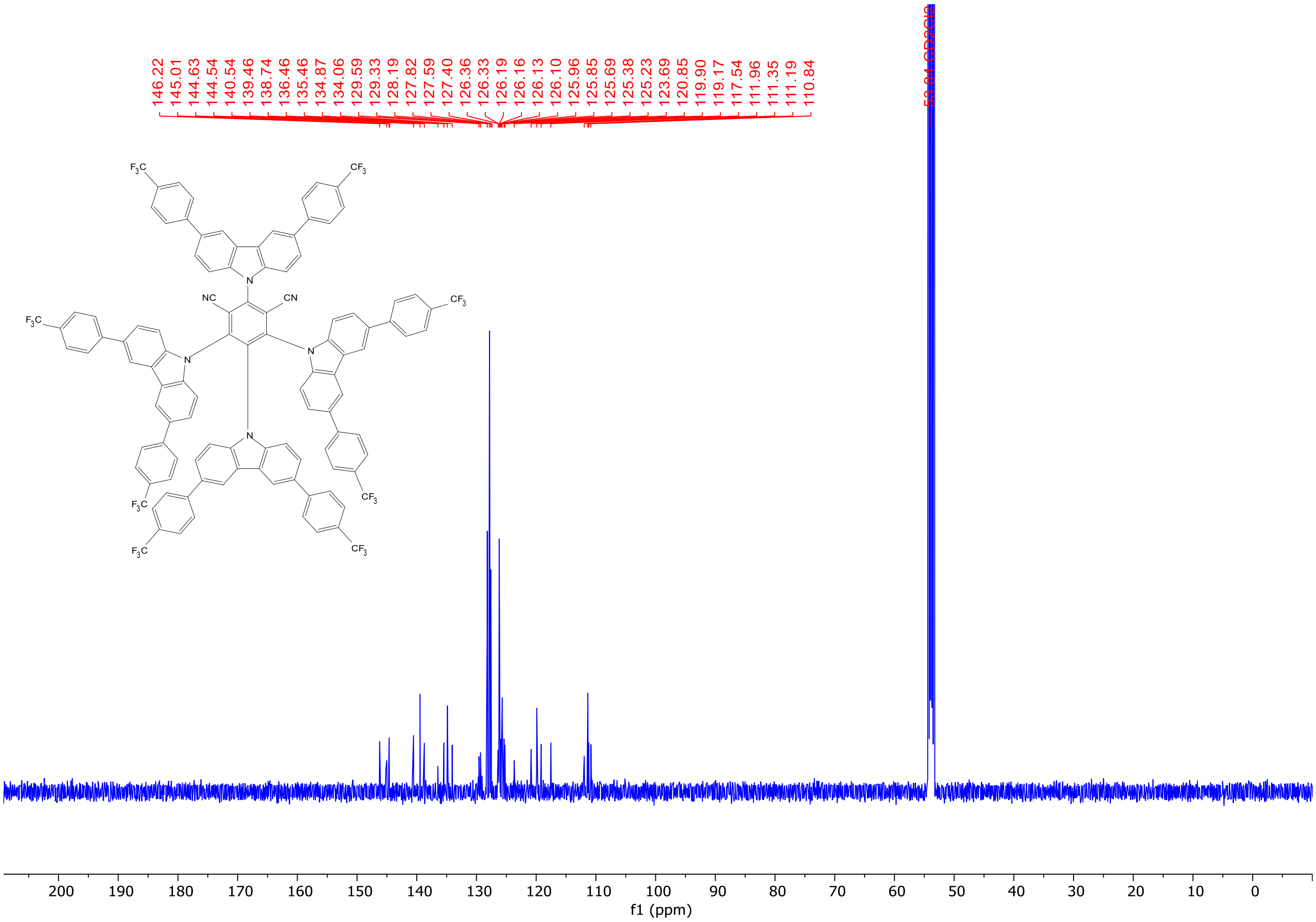

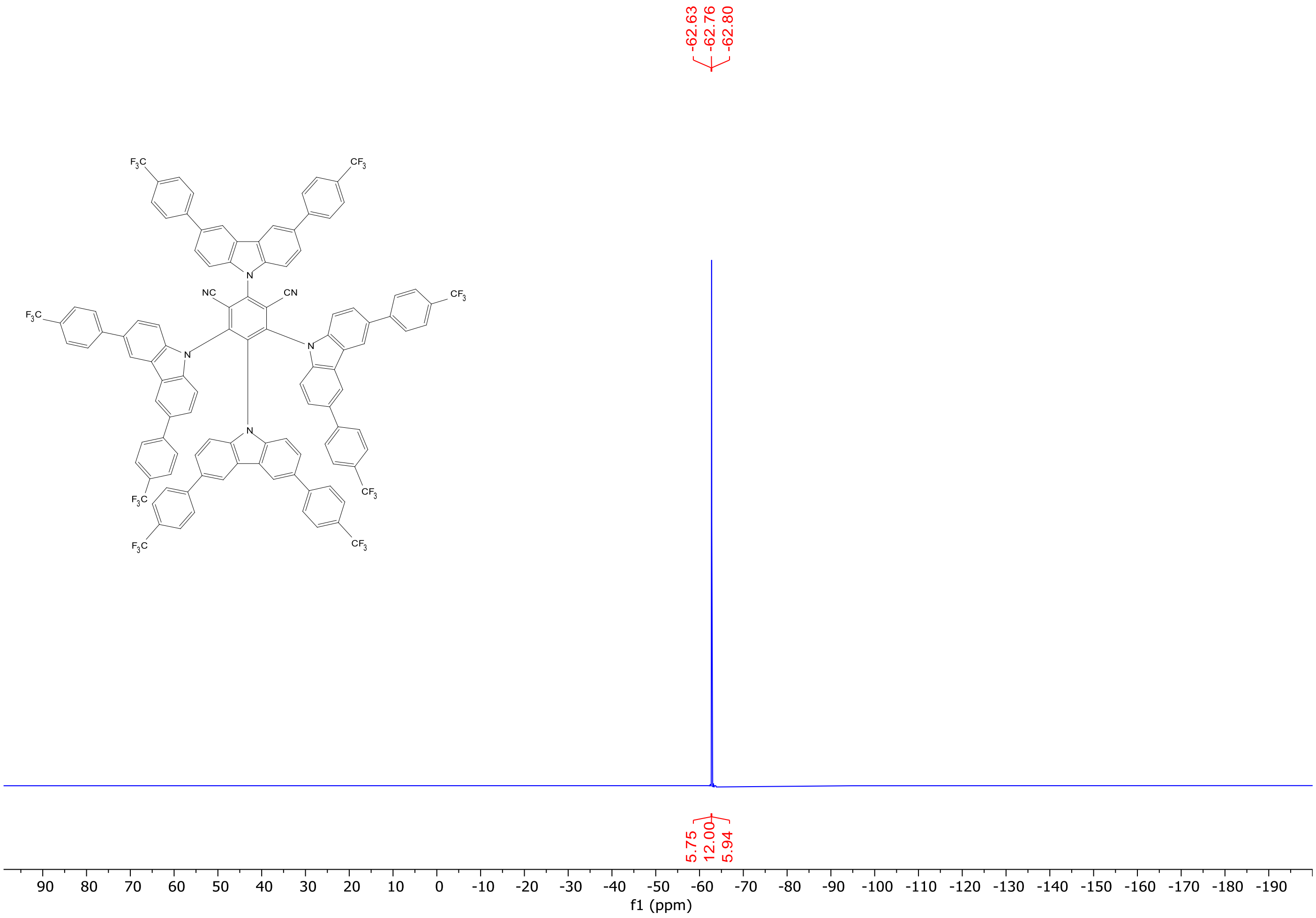

-62.63
-62.76
-62.80
F_3C
CF_3
NC
CN
N
5.75
12.00
5.94
90 80 70 60 50 40 30 20 10 0 -10 -20 -30 -40 -50 -60 -70 -80 -90 -100 -110 -120 -130 -140 -150 -160 -170 -180 -190
f1 (ppm)

4(Ar^{2}DPA)IPN---2,4,5,6-tetrakis(3,6-bis(4-cyanophenyl)-9*H*-carbazol-9-yl)isophthalonitrile (PC28)

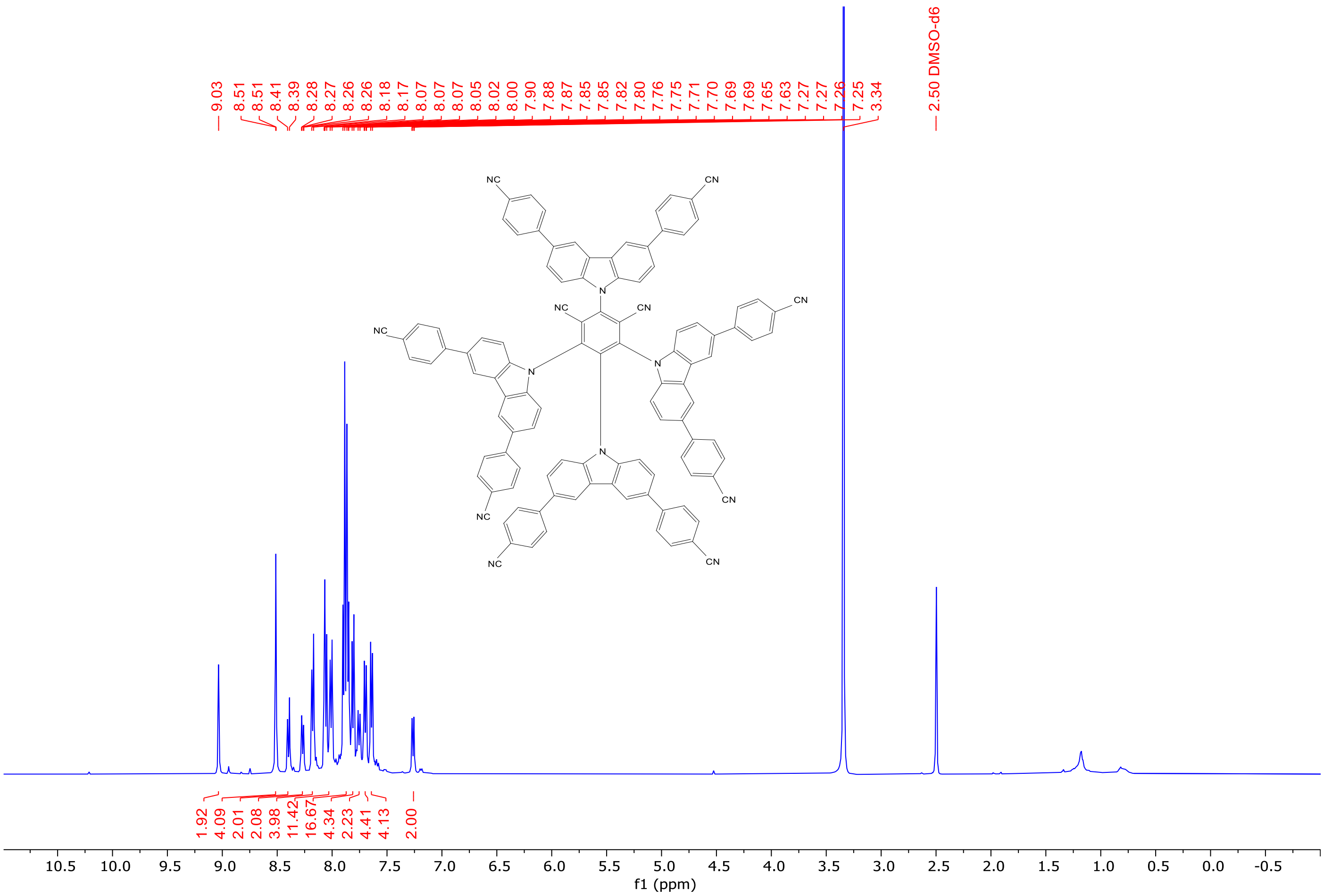

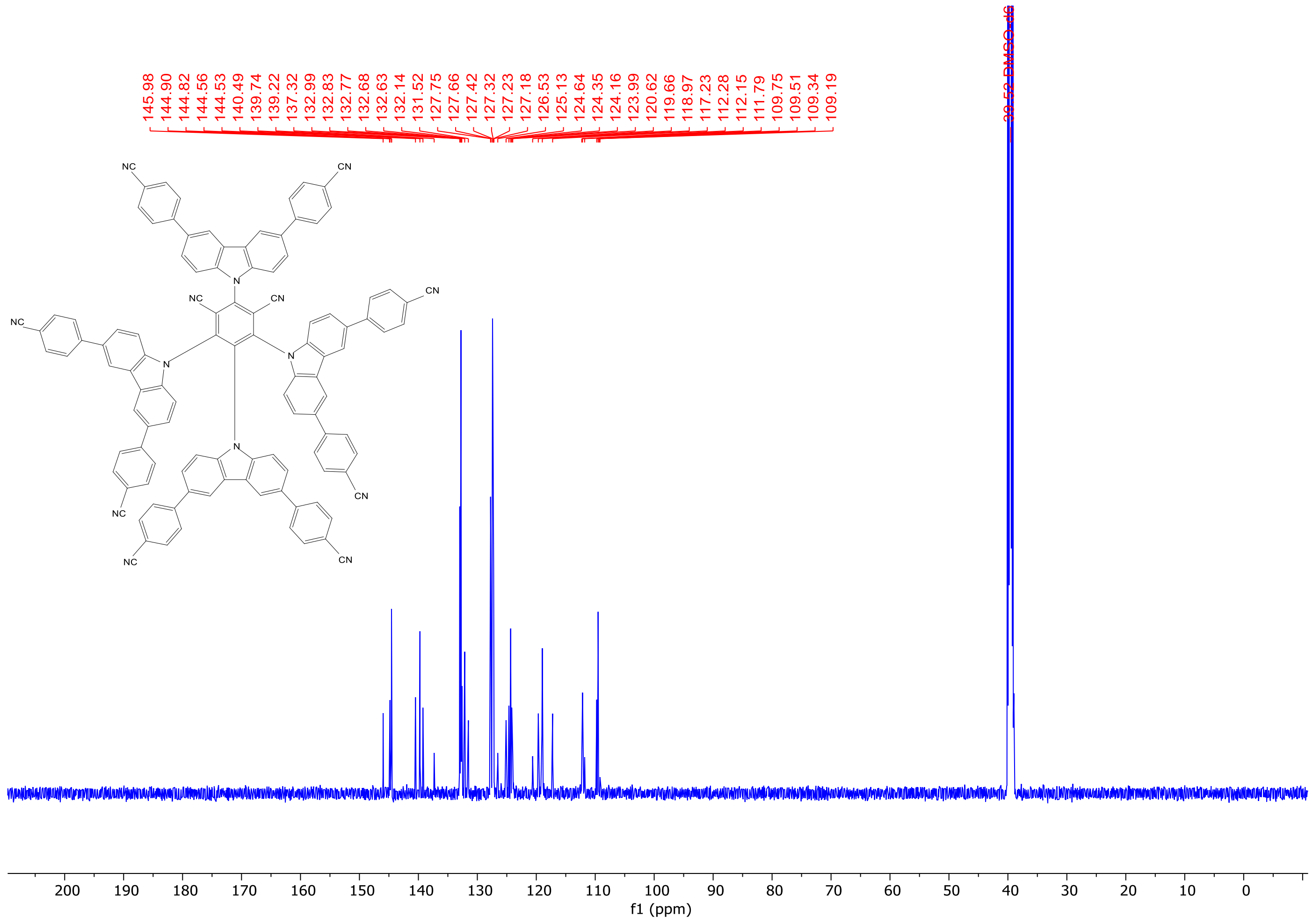

4(MeOPhNHAr^2)IPN---2,4,5,6-tetrakis((4-cyanophenyl)(4-methoxyphenyl)amino)isophthalonitrile (PC29)

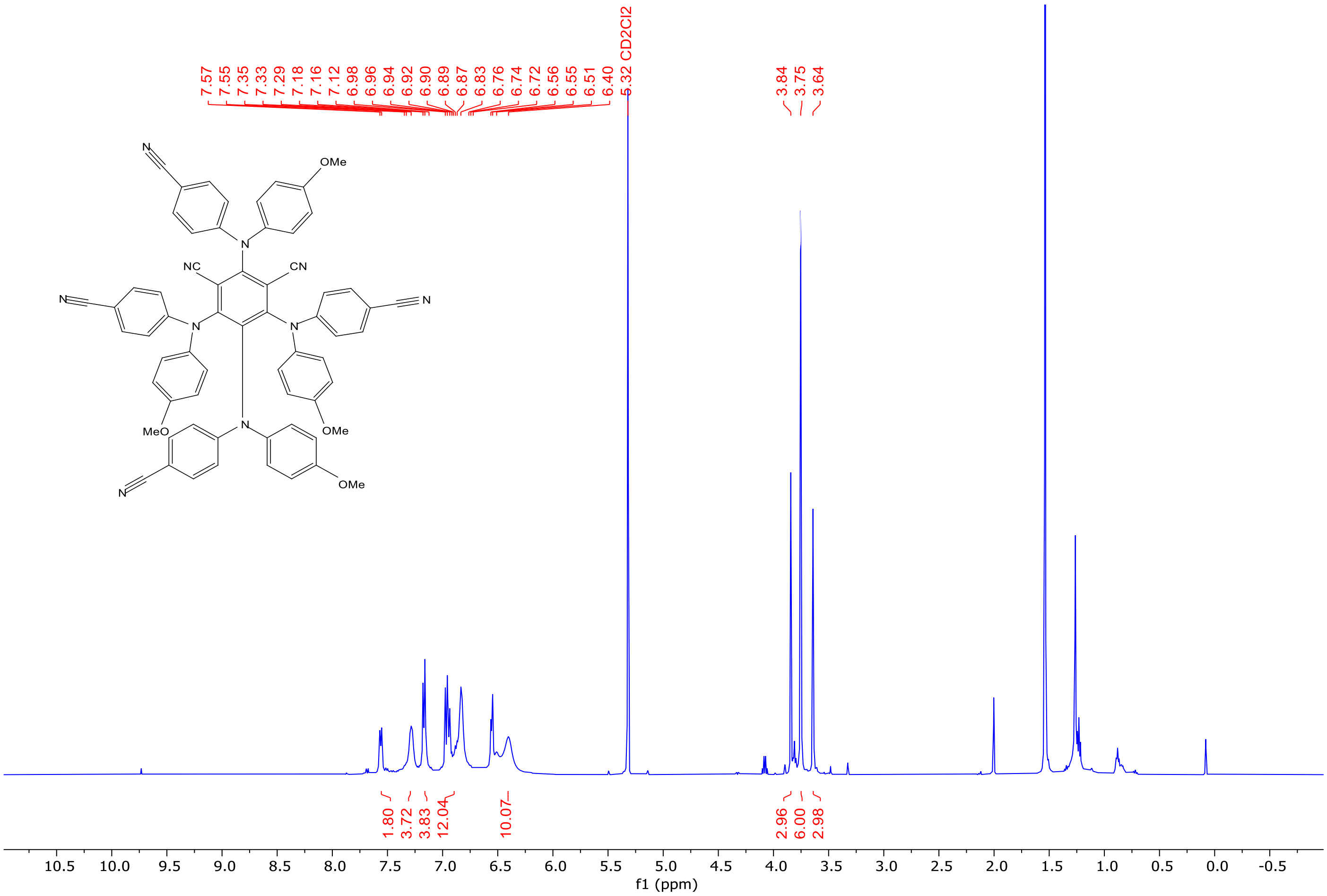

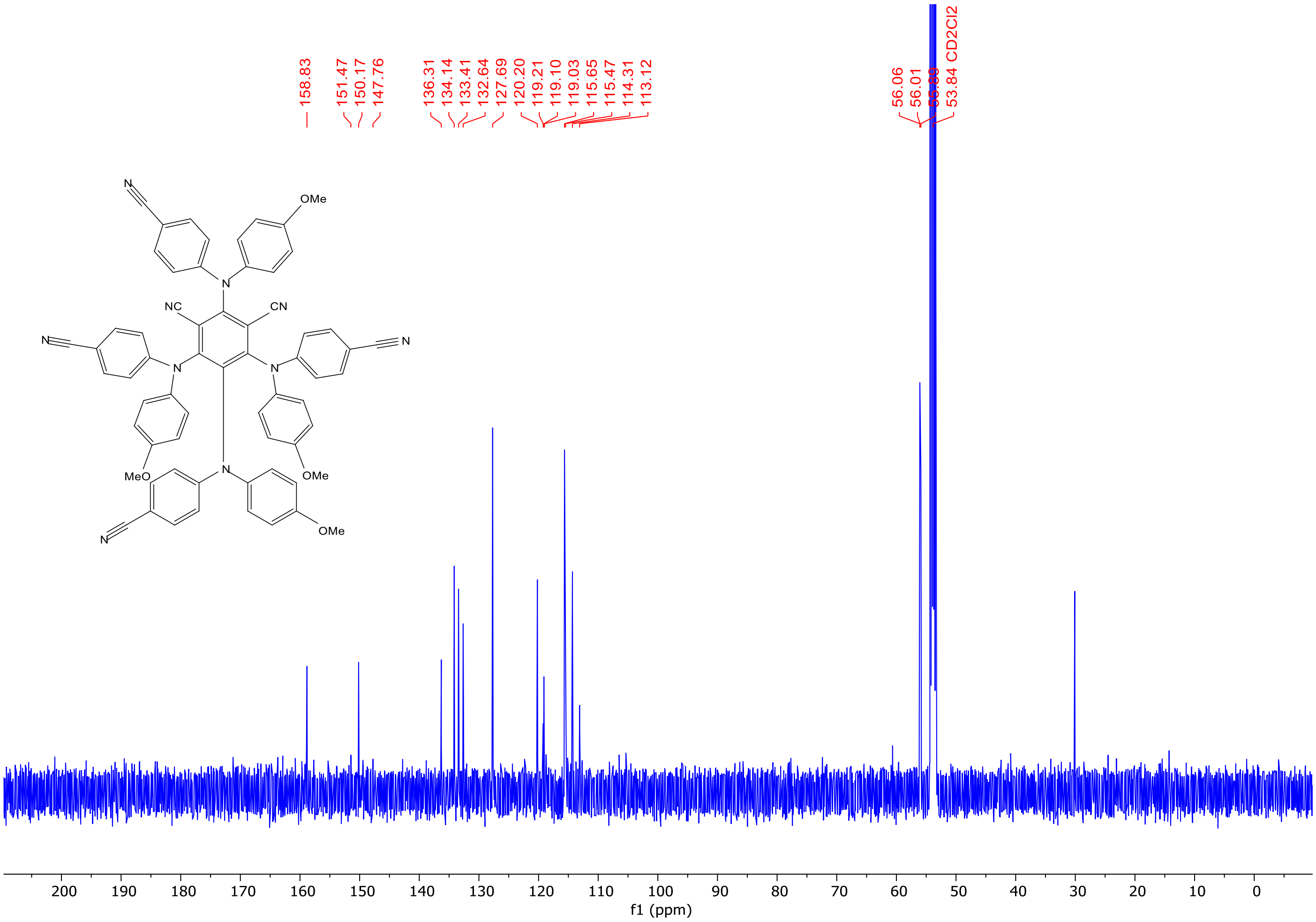

4DPAFBN---2,3,4,6-tetrakis(diphenylamino)-5-fluorobenzonitrile (PC30)

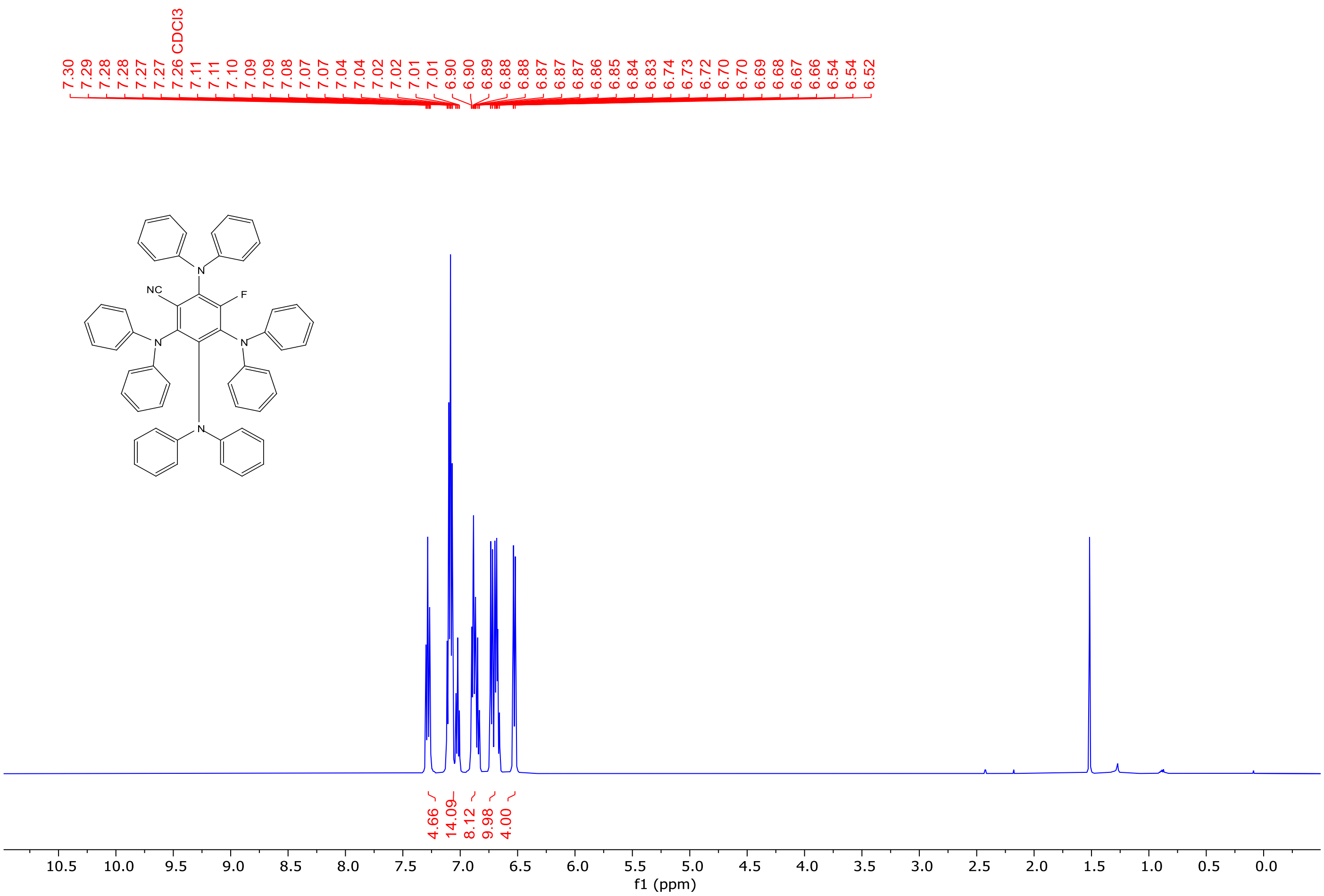

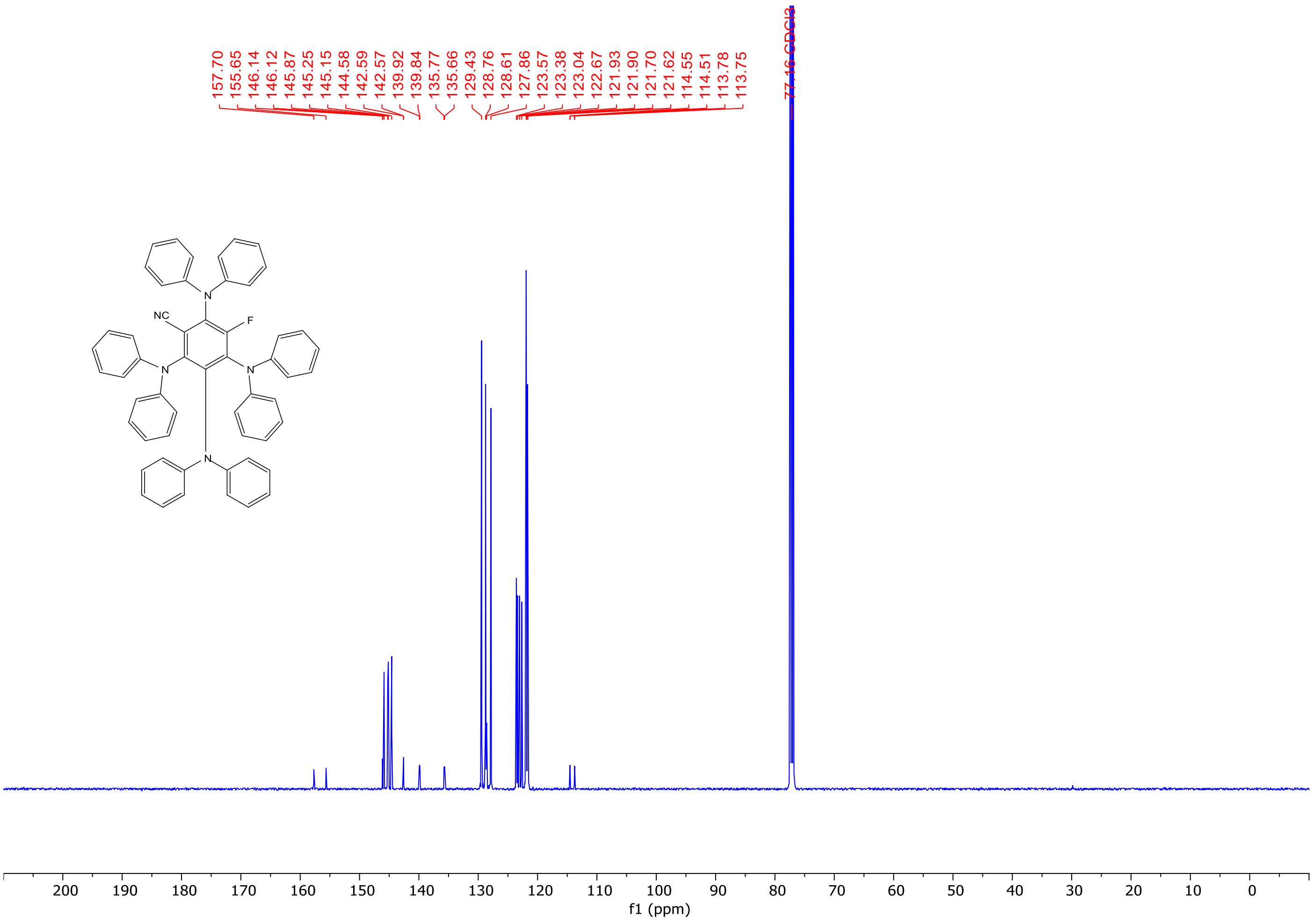

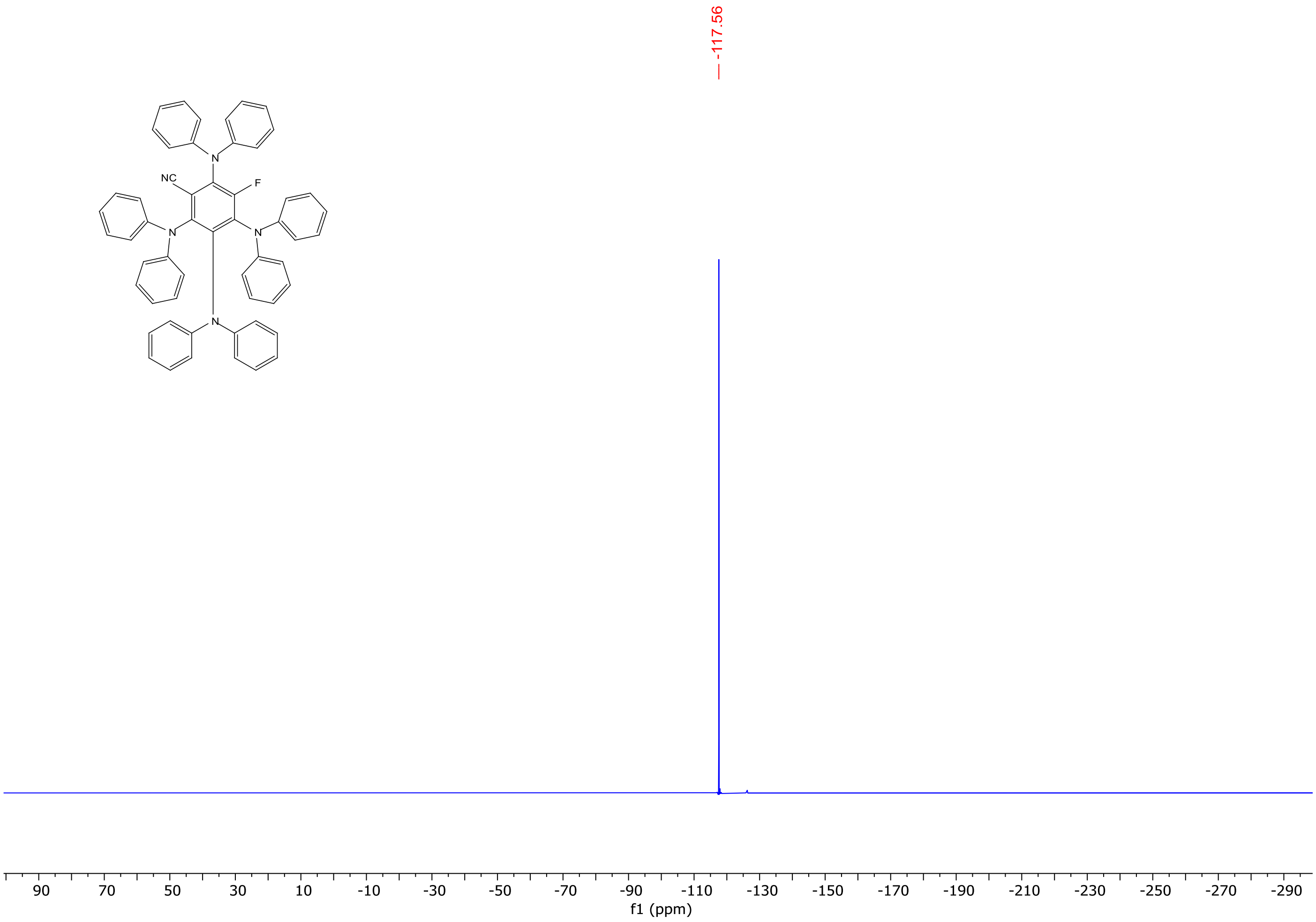
-117.56
NC
F
N
f1 (ppm)
90
70
50
30
10
-10
-30
-50
-70
-90
-110
-130
-150
-170
-190
-210
-230
-250
-270
-290

5CzBN---2,3,4,5,6-penta(9*H*-carbazol-9-yl)benzonitrile (PC31)

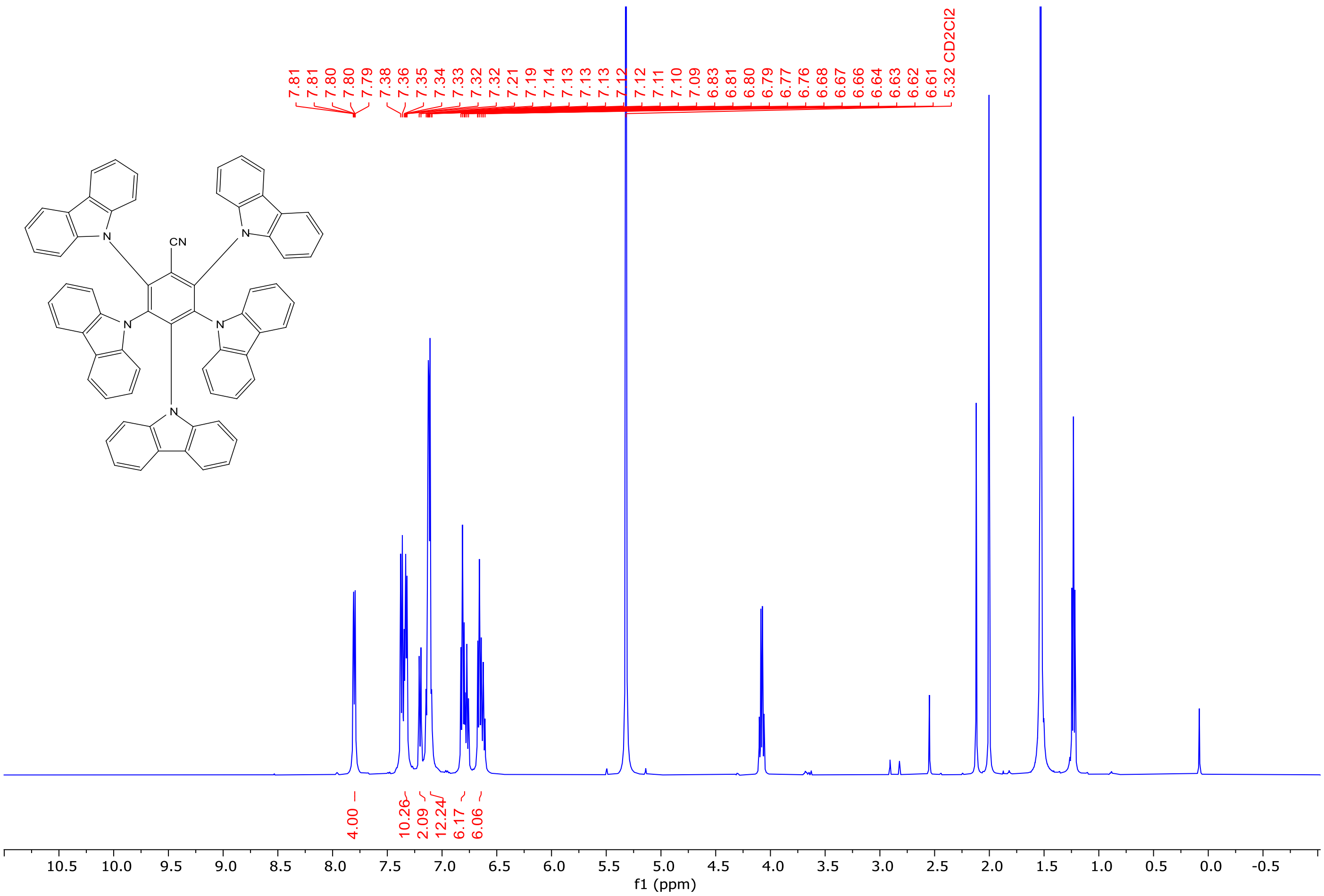

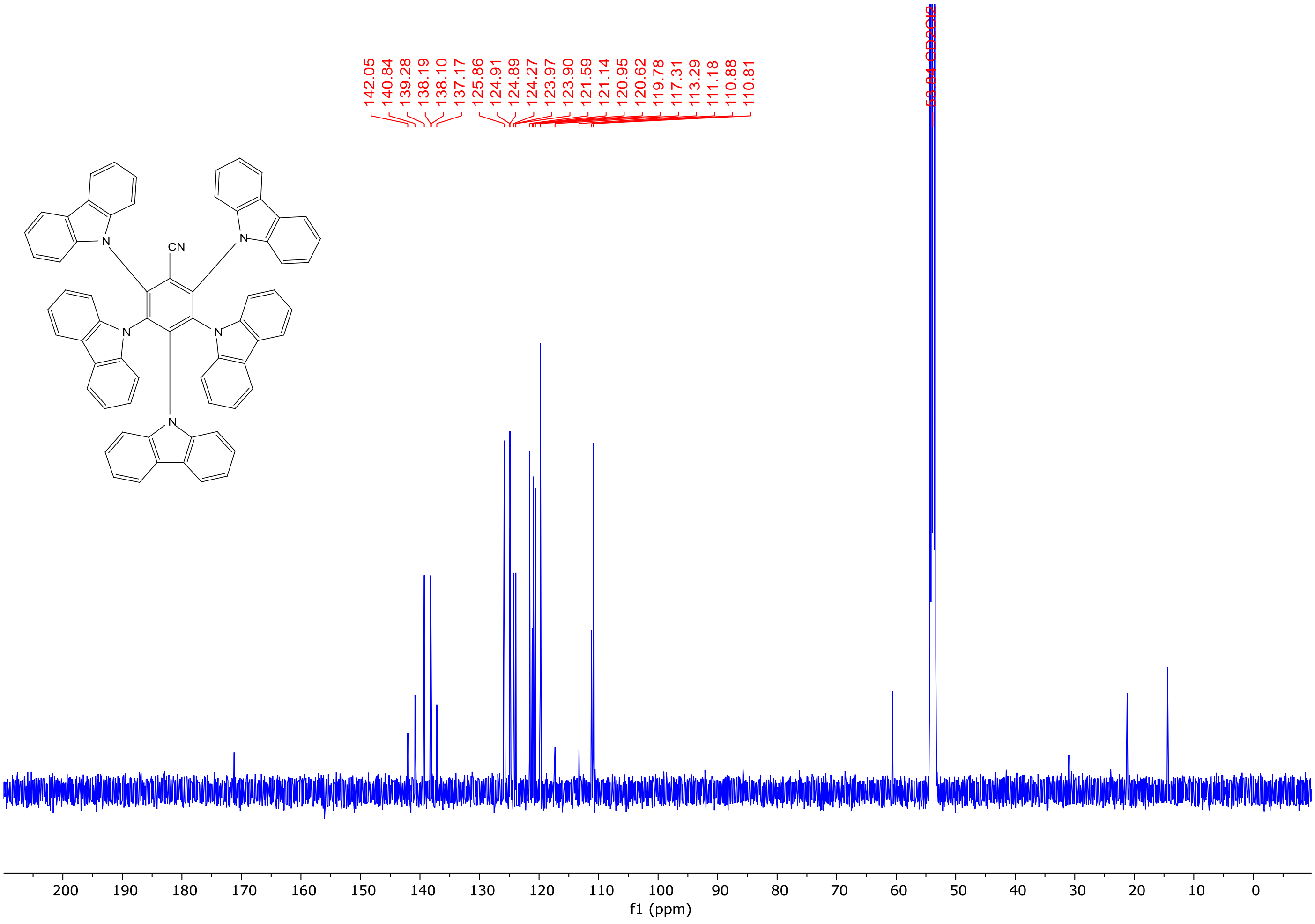

4CzTPN--- 2,3,5,6-tetra(9*H*-carbazol-9-yl)terephthalonitrile (PC32)

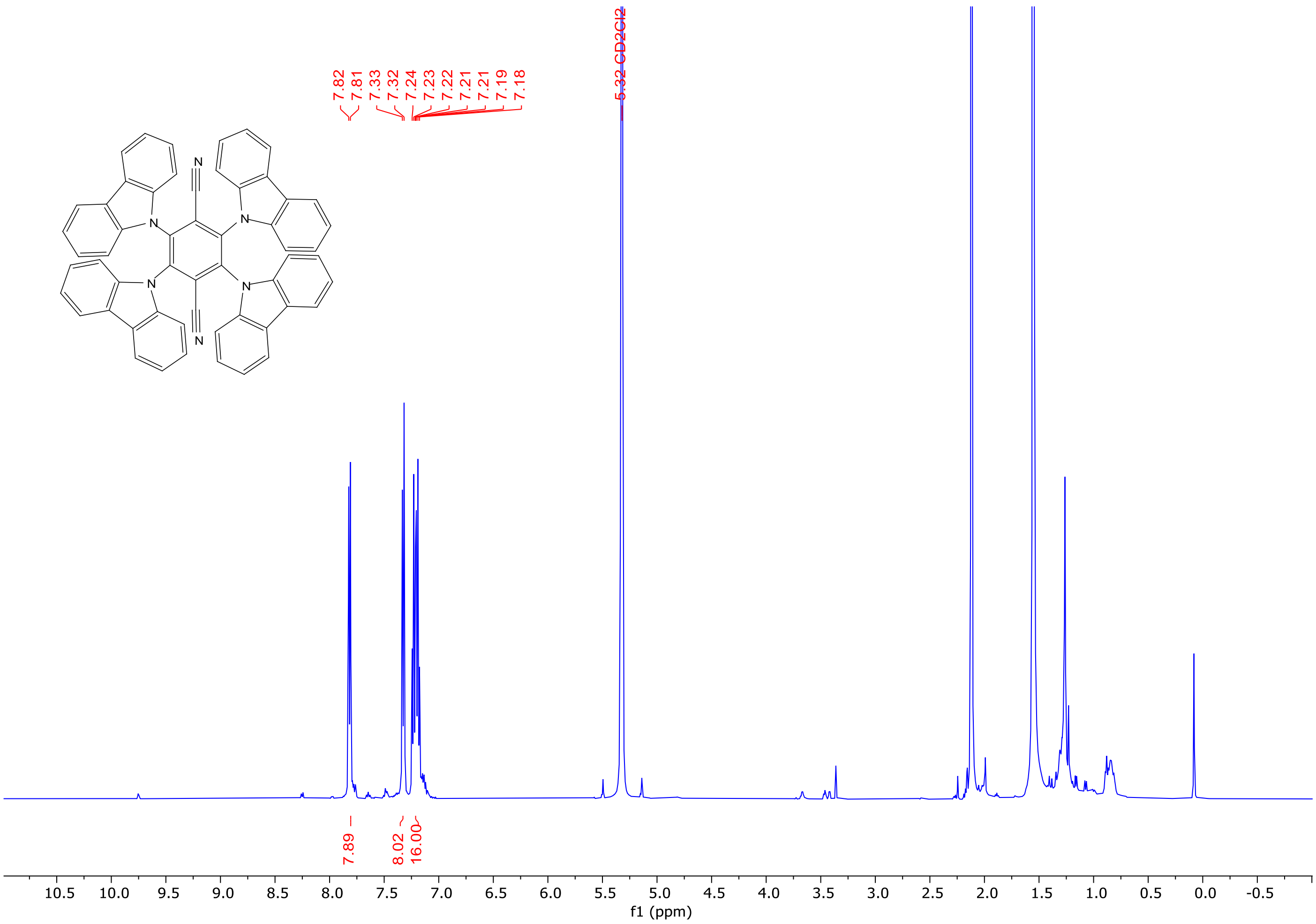

4DPATPN--- 2,3,5,6-tetrakis(diphenylamino)terephthalonitrile (PC33)

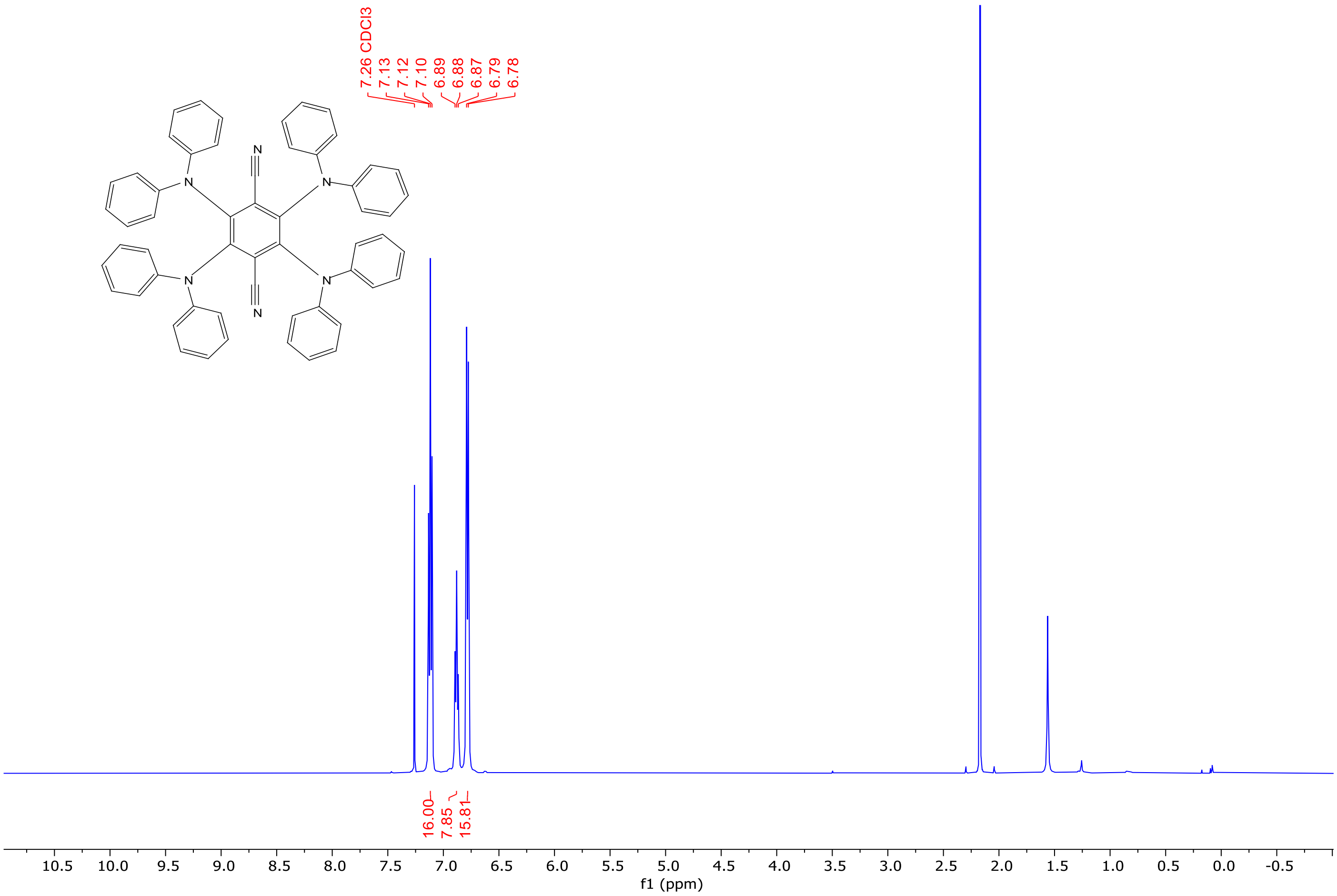

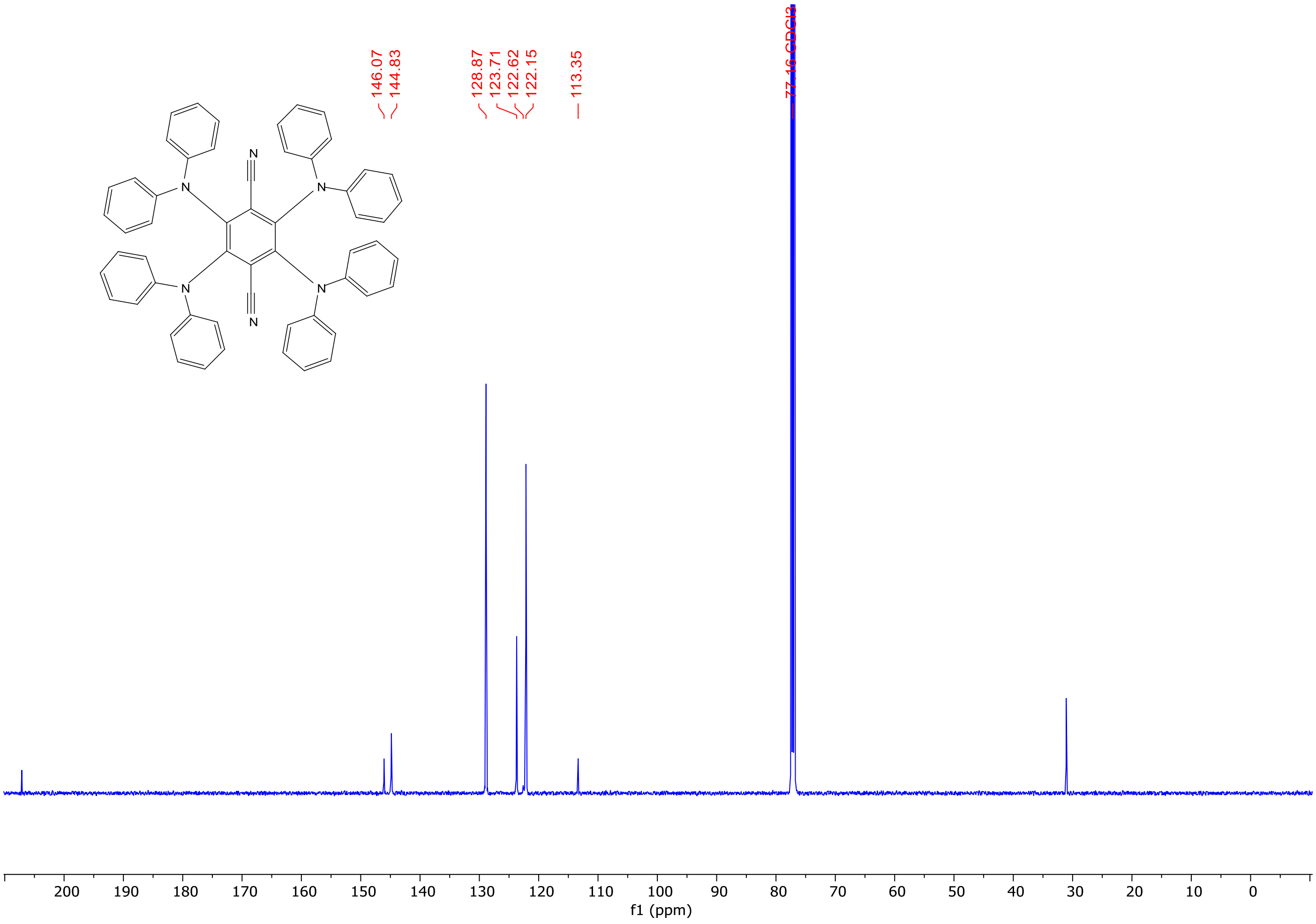

4(MeOPhNAr[1])IPN---2,4,5,6-tetrakis((3,5-bis(trifluoromethyl)phenyl)(4-methoxyphenyl)amino)isophthalonitrile (PC34)

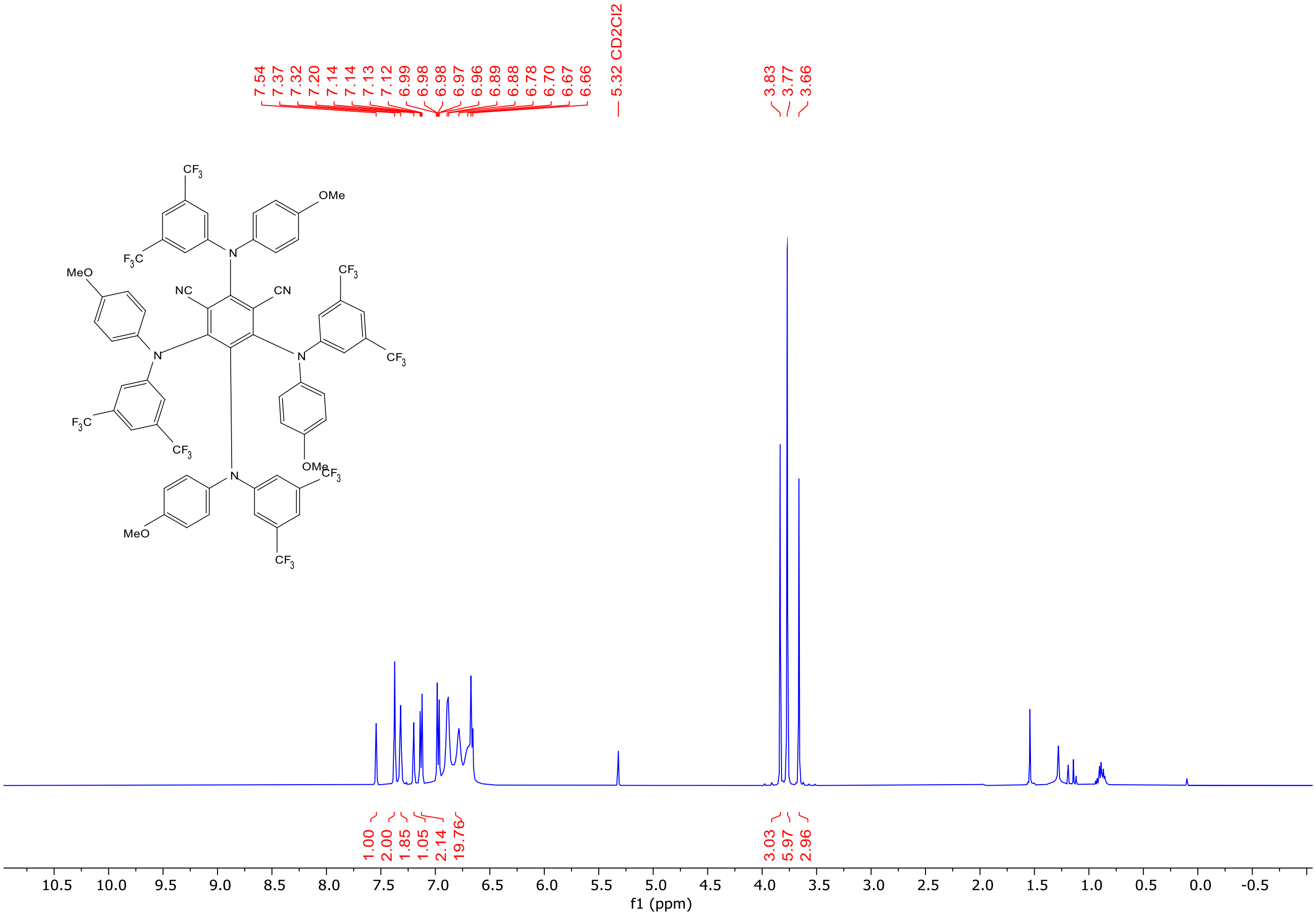

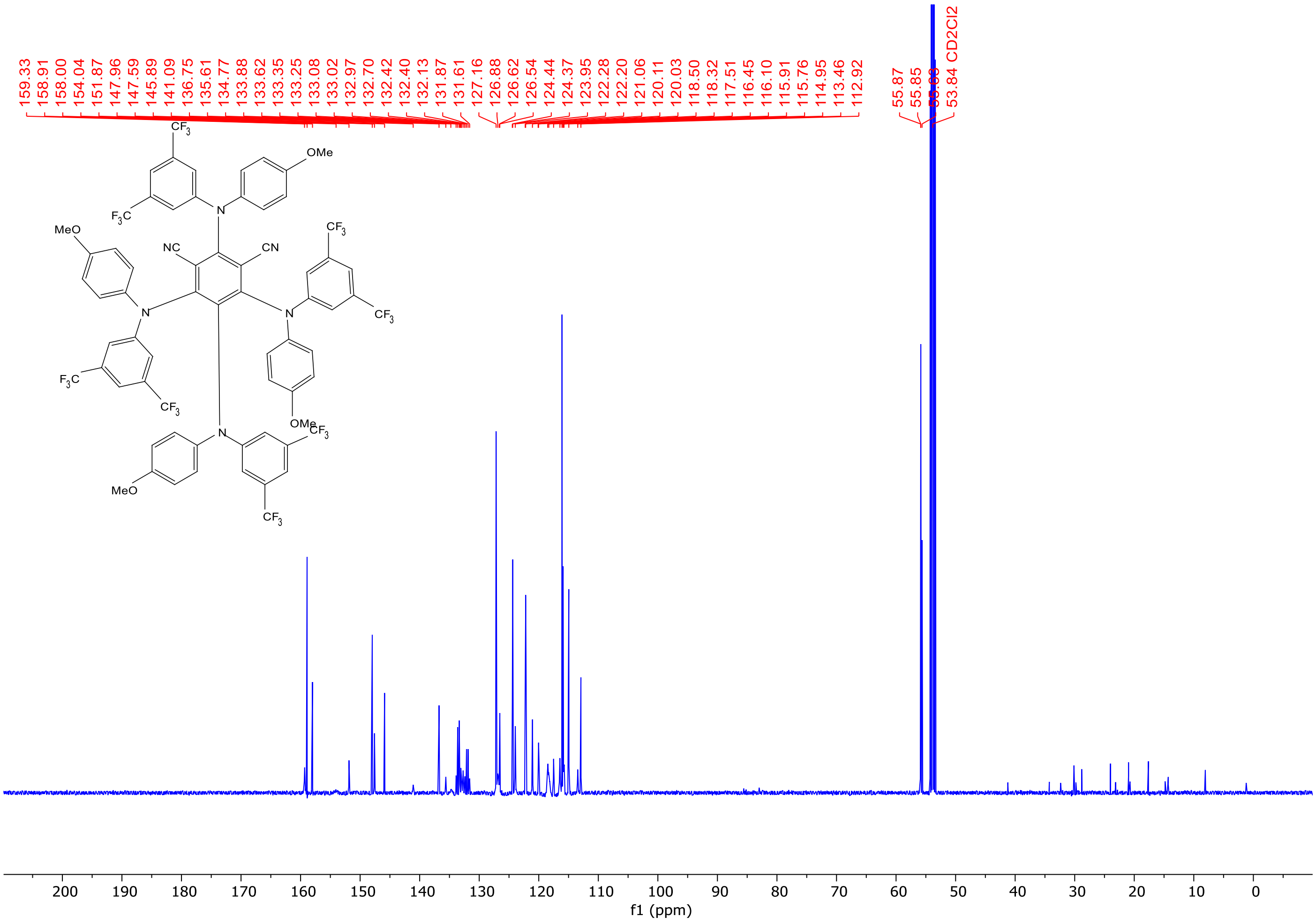
159.33 158.91 158.00 154.04 151.87 147.96 147.59 145.89 141.09 136.75 135.61 134.77 133.88 133.62 133.35 133.25 133.08 133.02 132.97 132.70 132.42 132.40 132.13 131.87 131.61 127.16 126.88 126.62 126.54 124.44 124.37 123.95 122.28 122.20 121.06 120.11 120.03 118.50 118.32 117.51 116.45 116.10 115.91 115.76 114.95 113.46 112.92
55.87 55.85
53.84 CD2Cl2
CF3
OMe
F3C
MeO
NC
CN
N
200 190 180 170 160 150 140 130 120 110 100 90 80 70 60 50 40 30 20 10 0
f1 (ppm)

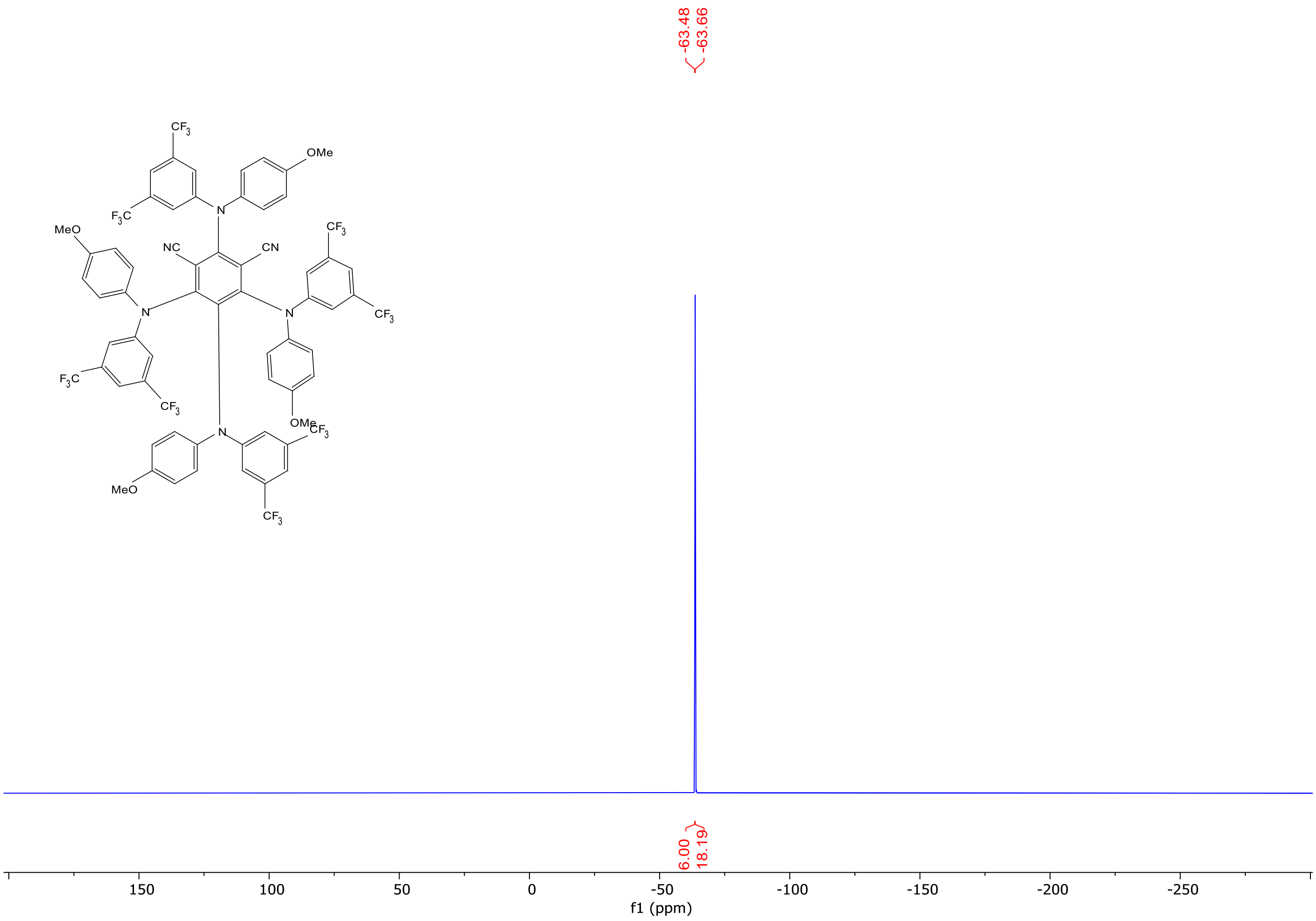
-63.48
-63.66
CF3
OMe
F3C
MeO
NC
CN
N
6.00
18.19
150 100 50 0 -50 -100 -150 -200 -250
f1 (ppm)

4(Ar[1]DPA)IPN---2,4,5,6-tetrakis(3,6-bis(3,5-bis(trifluoromethyl)phenyl)-9*H*-carbazol-9-yl)isophthalonitrile (PC35)

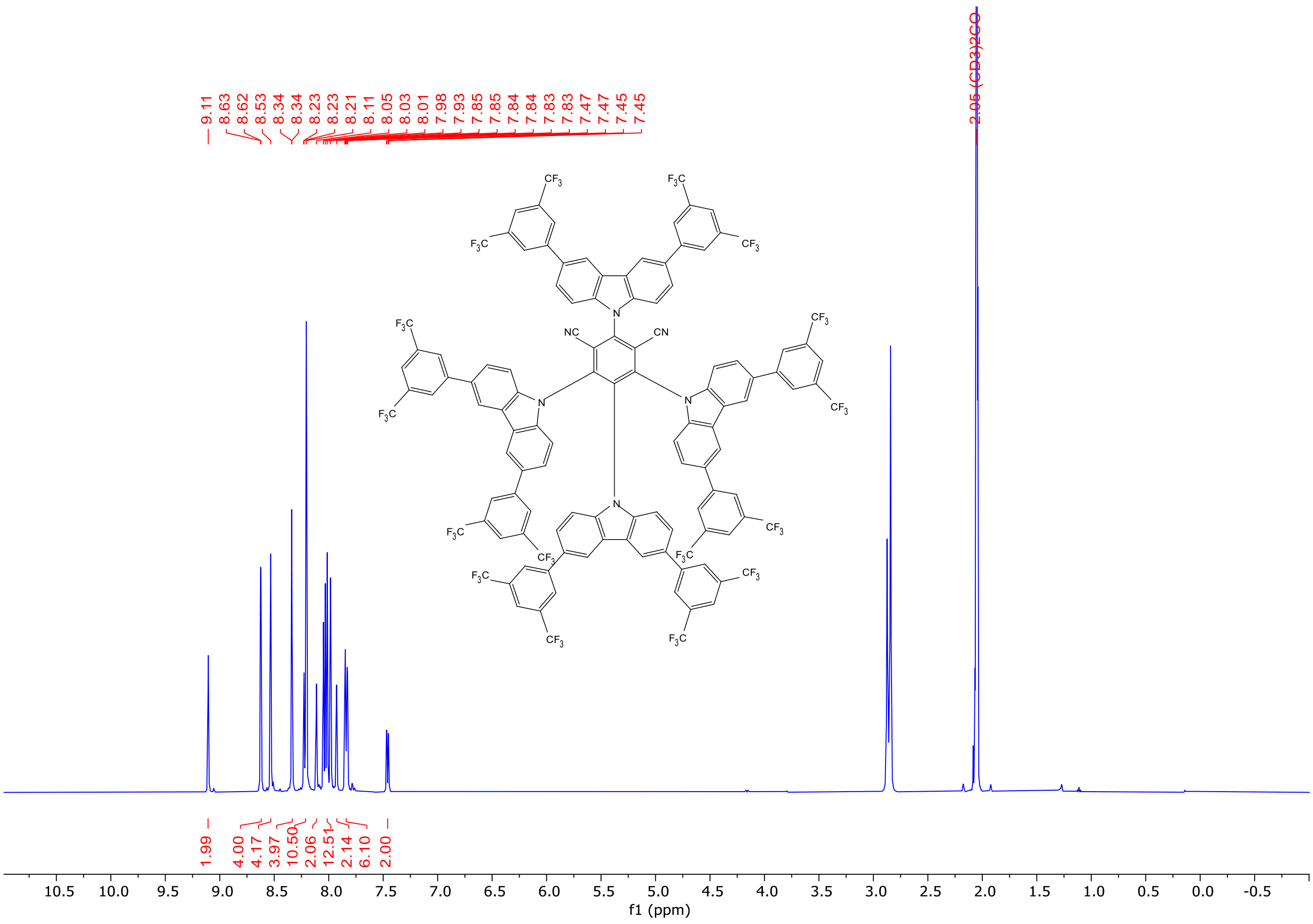

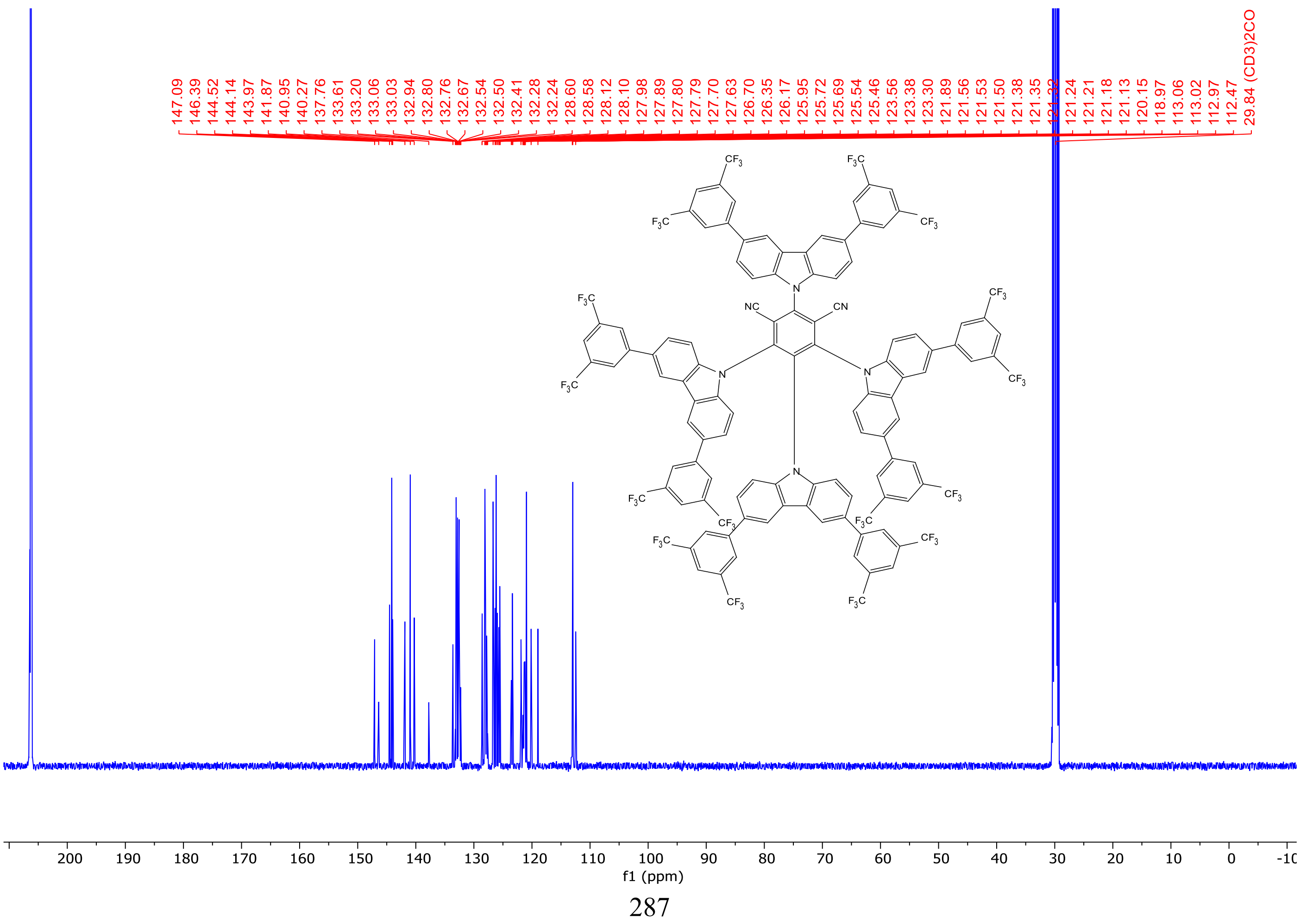

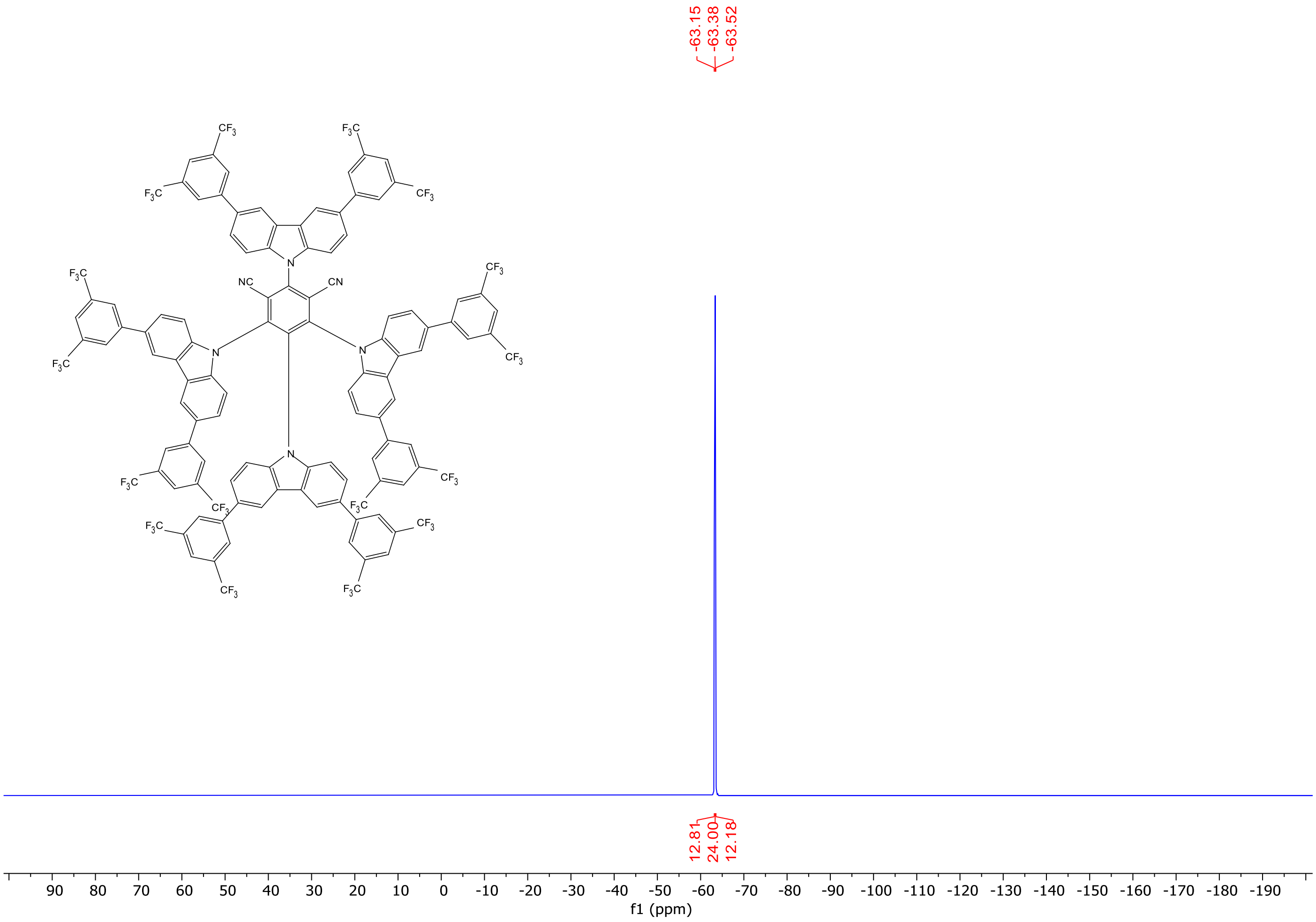
-63.15
-63.38
-63.52
12.81
24.00
12.18
90 80 70 60 50 40 30 20 10 0 -10 -20 -30 -40 -50 -60 -70 -80 -90 -100 -110 -120 -130 -140 -150 -160 -170 -180 -190
f1 (ppm)

4(Ar^{6}PhDPA)TPN--- 2,3,5,6-tetrakis(bis(4'-methoxy-3',5'-dimethyl-[1,1'-biphenyl]-4-yl)amino)terephthalonitrile (PC36)

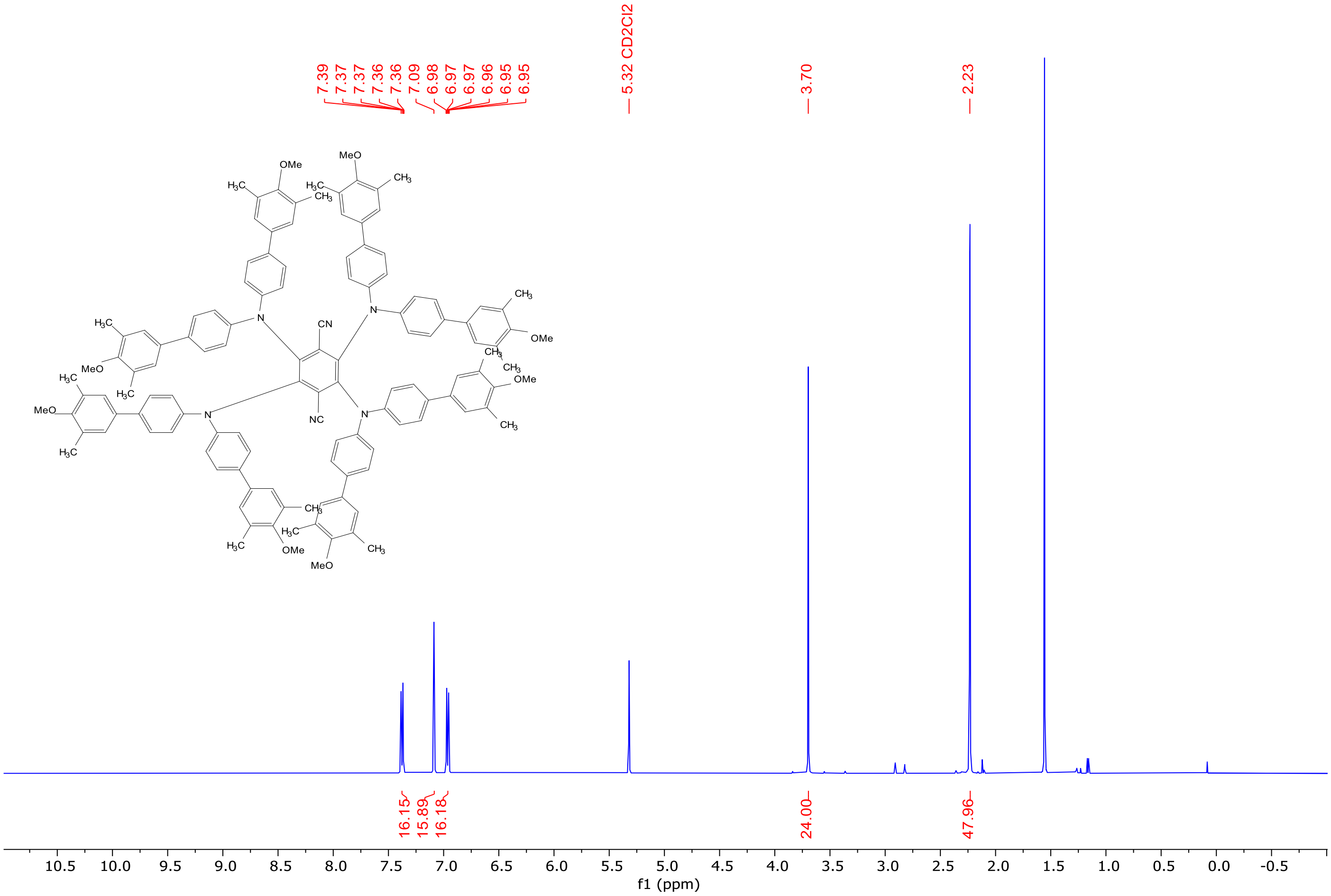

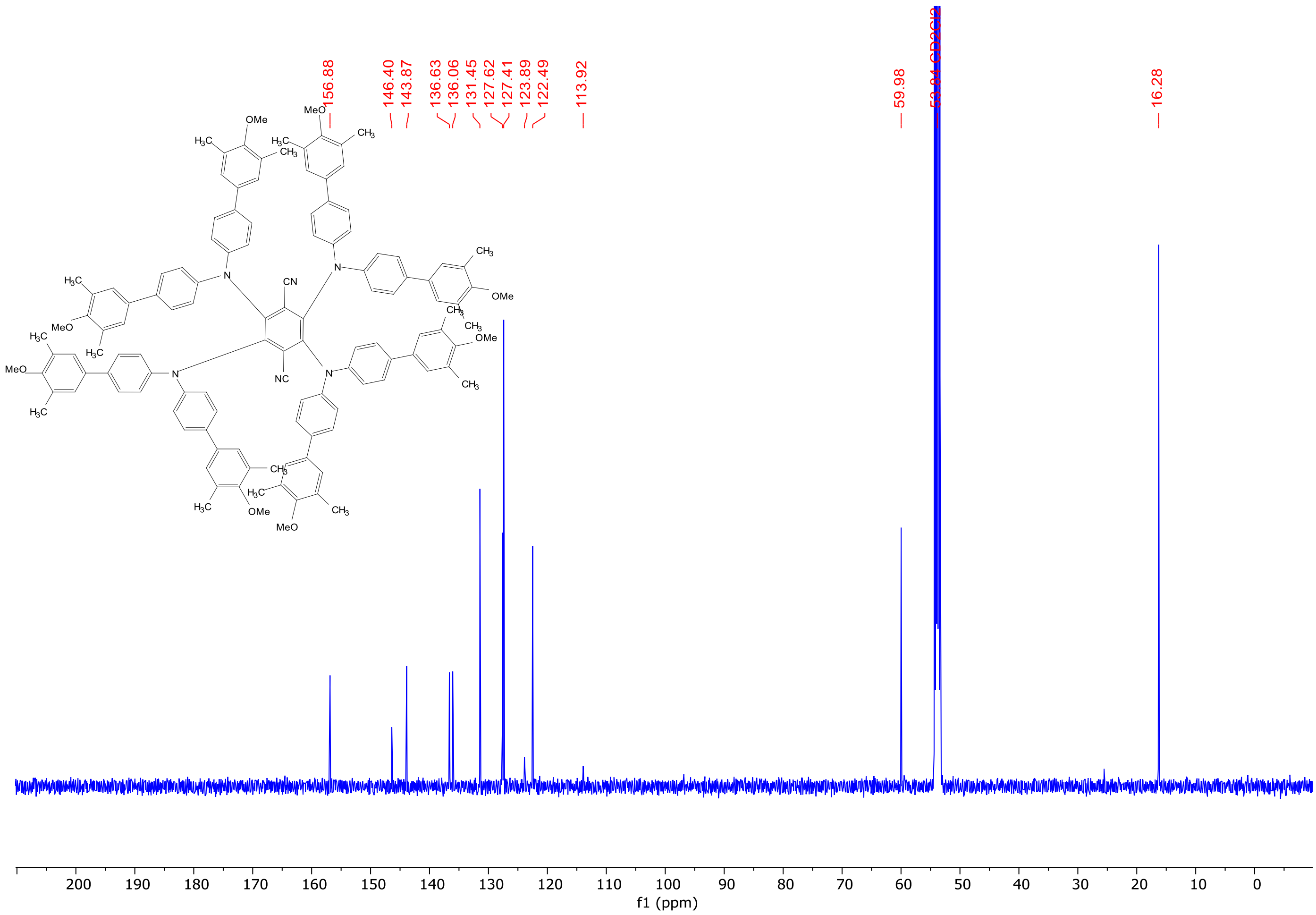

4(2MeODPA)TPN---2,3,5,6-tetrakis(bis(3,5-dimethoxyphenyl)amino)terephthalonitrile (PC37)

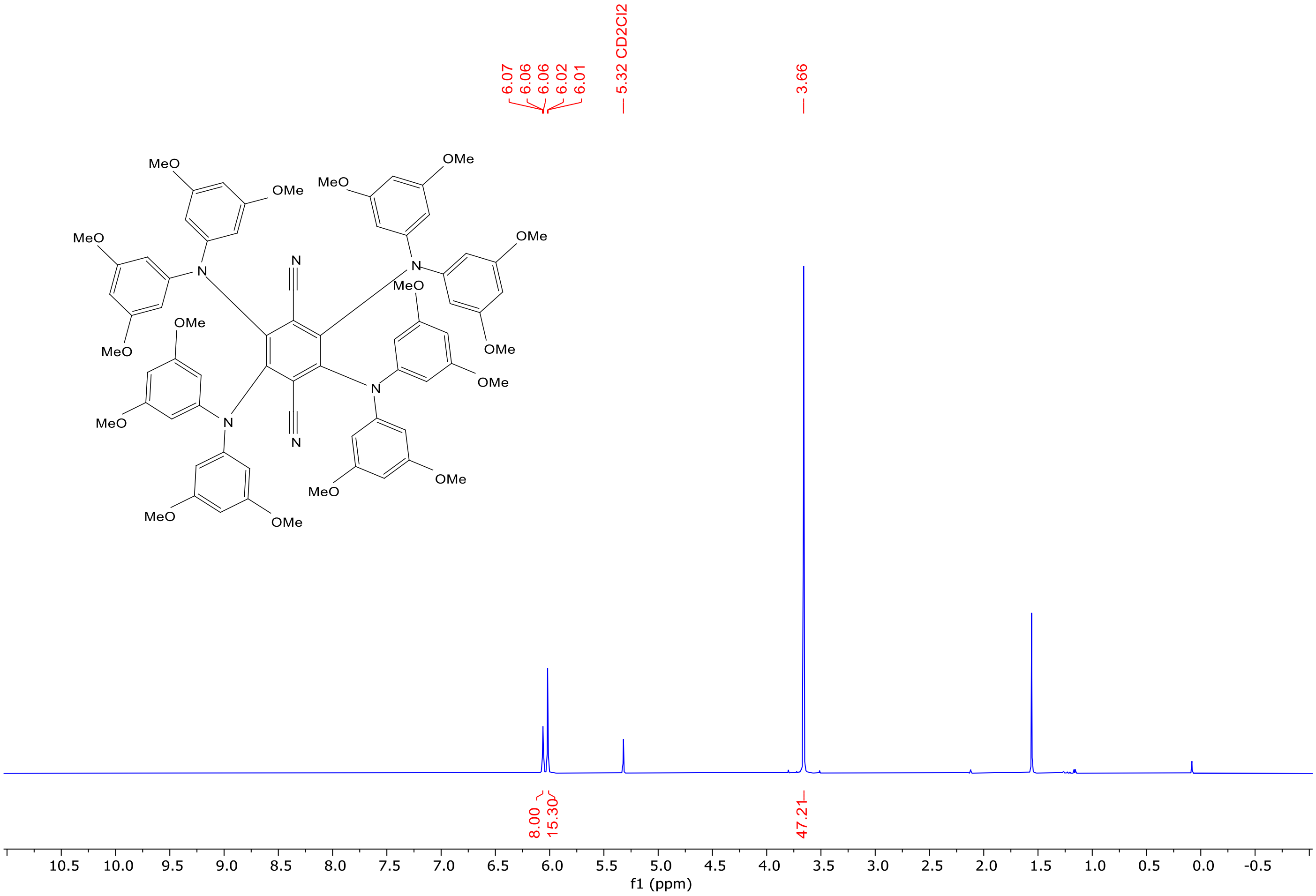

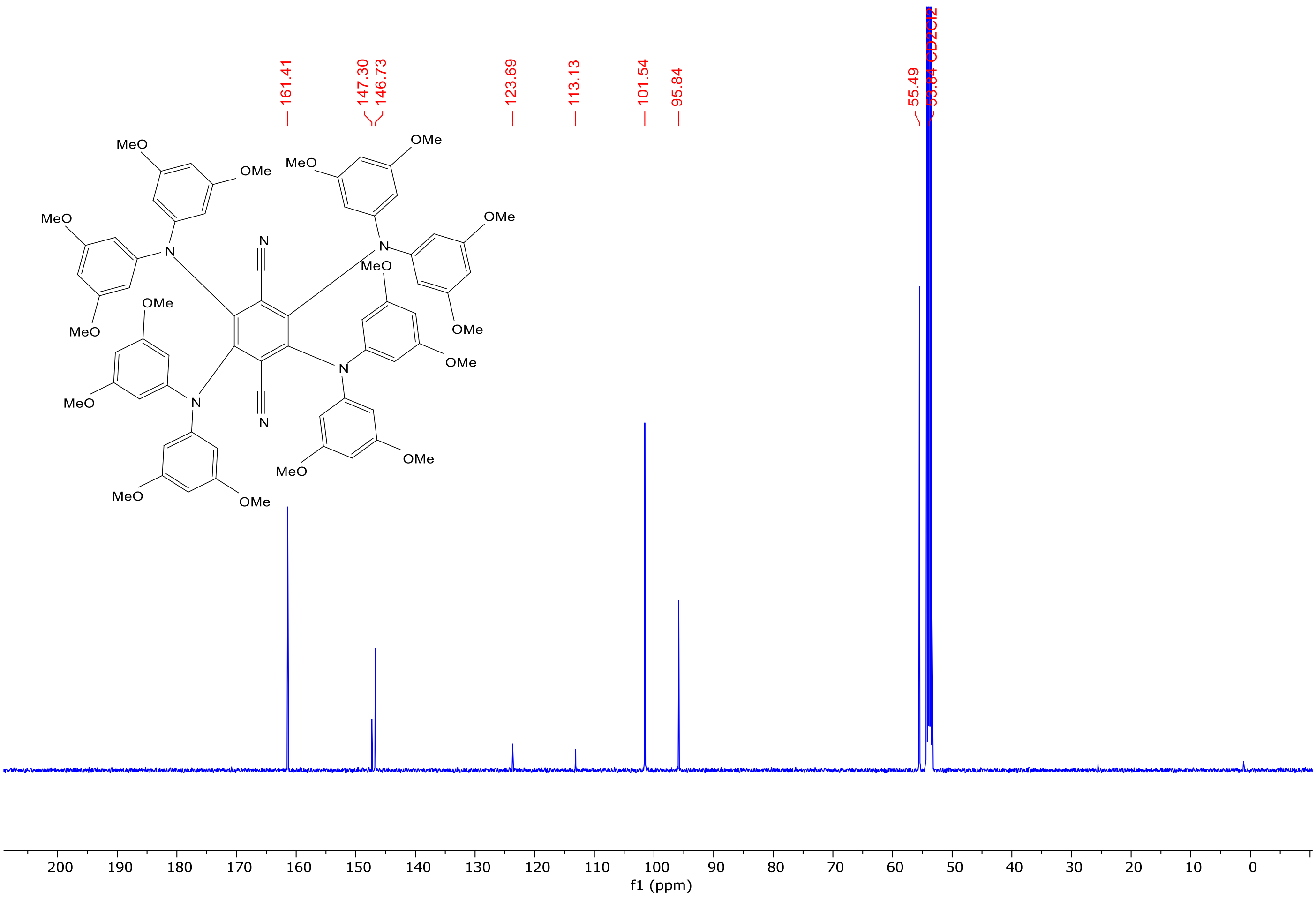

3CzBN-3,4,5---3,4,5-tri(9H-carbazol-9-yl)benzonitrile (PC38)

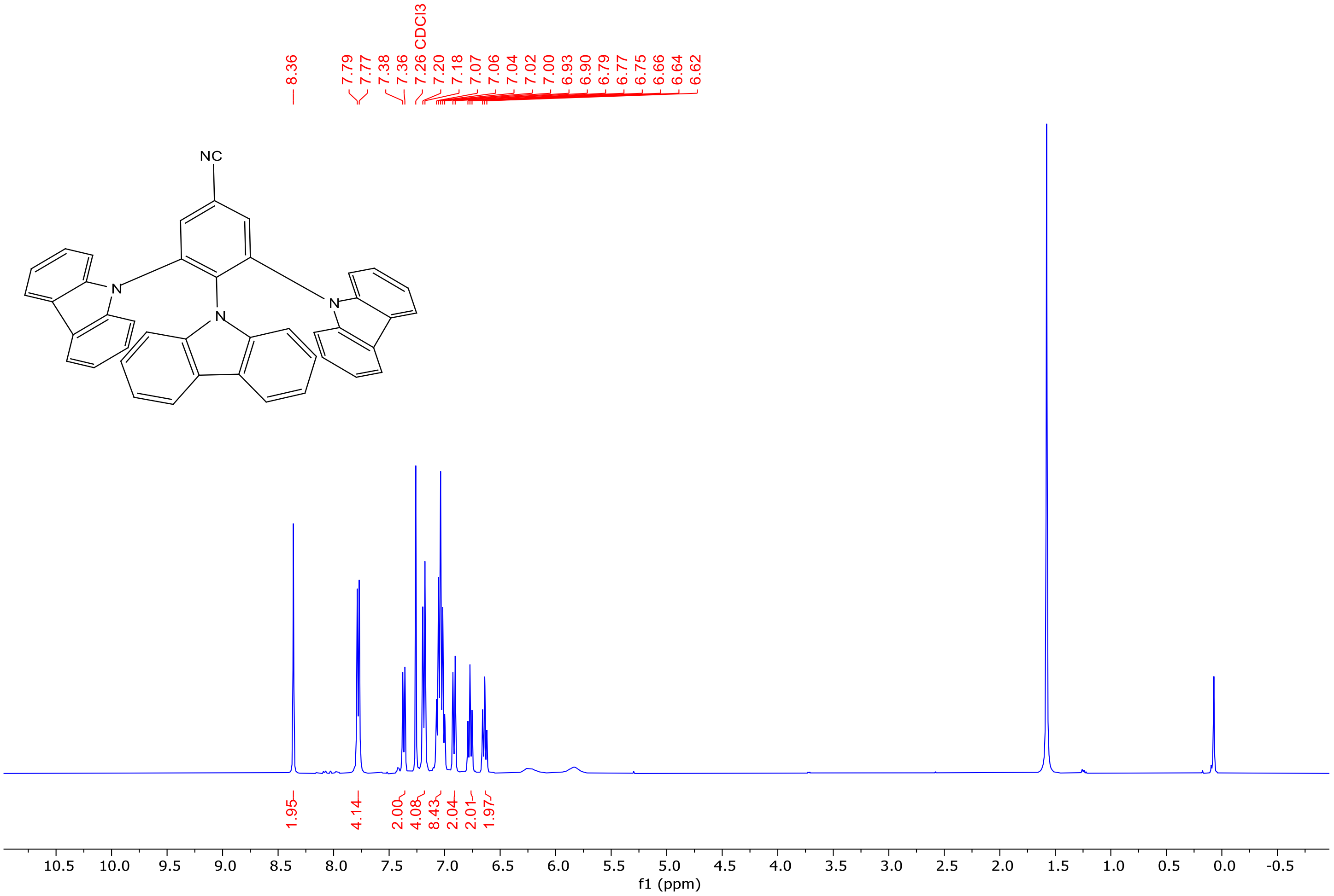

3CzBN-2,4,6---2,4,6-tri(9H-carbazol-9-yl)benzonitrile (PC39)

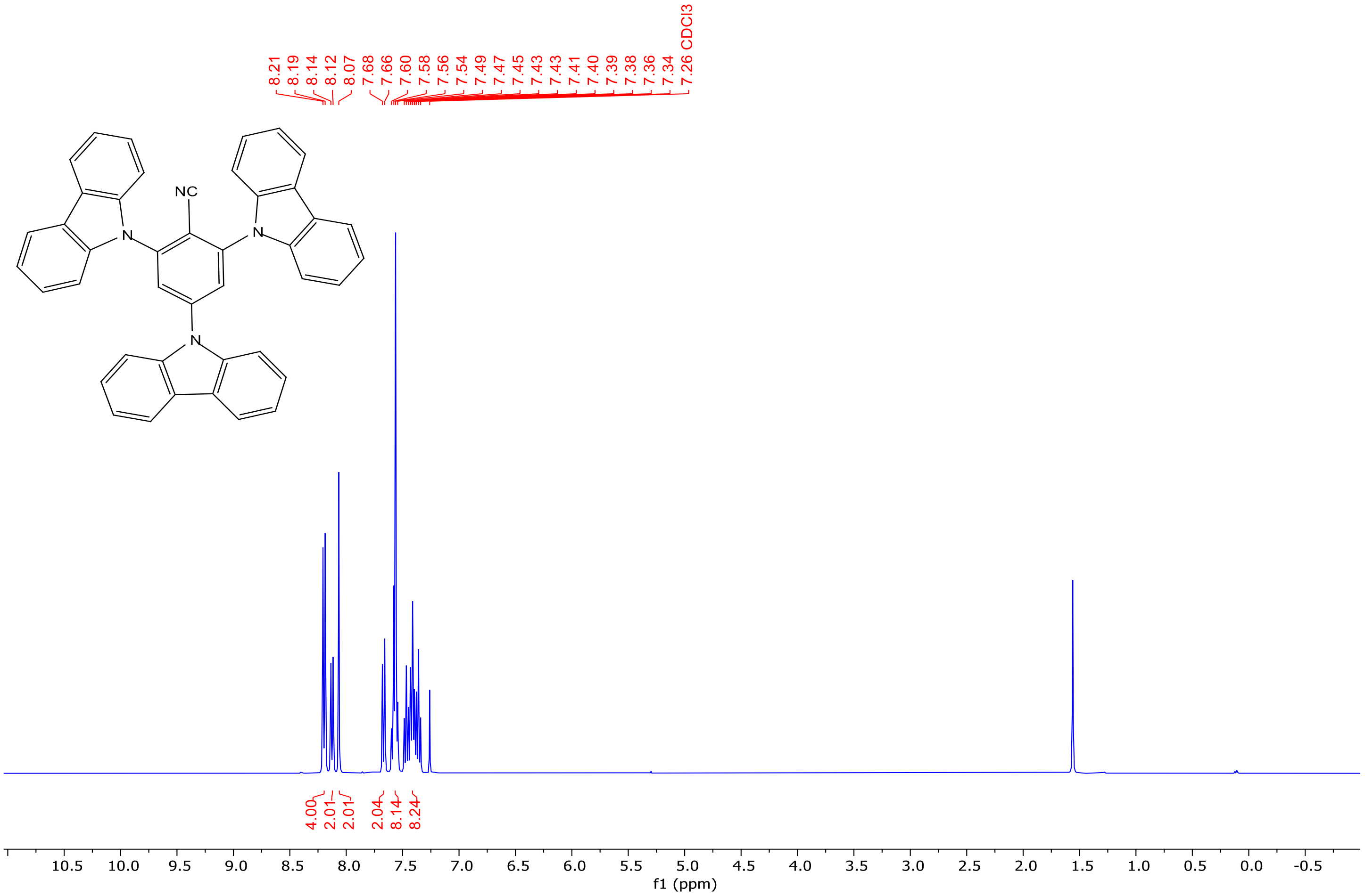

2CzIPN---4,6-di(9H-carbazol-9-yl)isophthalonitrile (PC40)

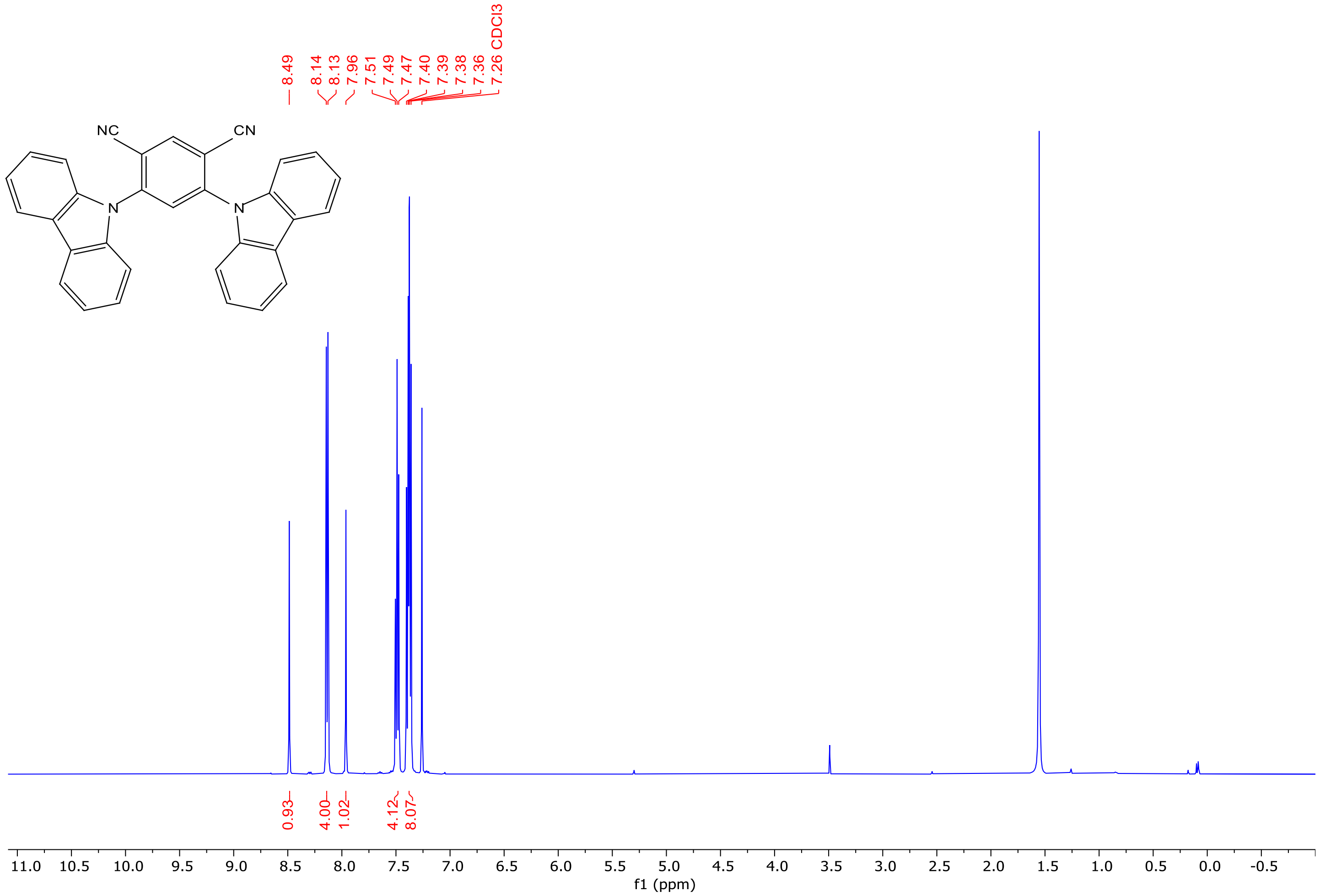

2CzPN---4,5-di(9H-carbazol-9-yl)phthalonitrile (PC41)

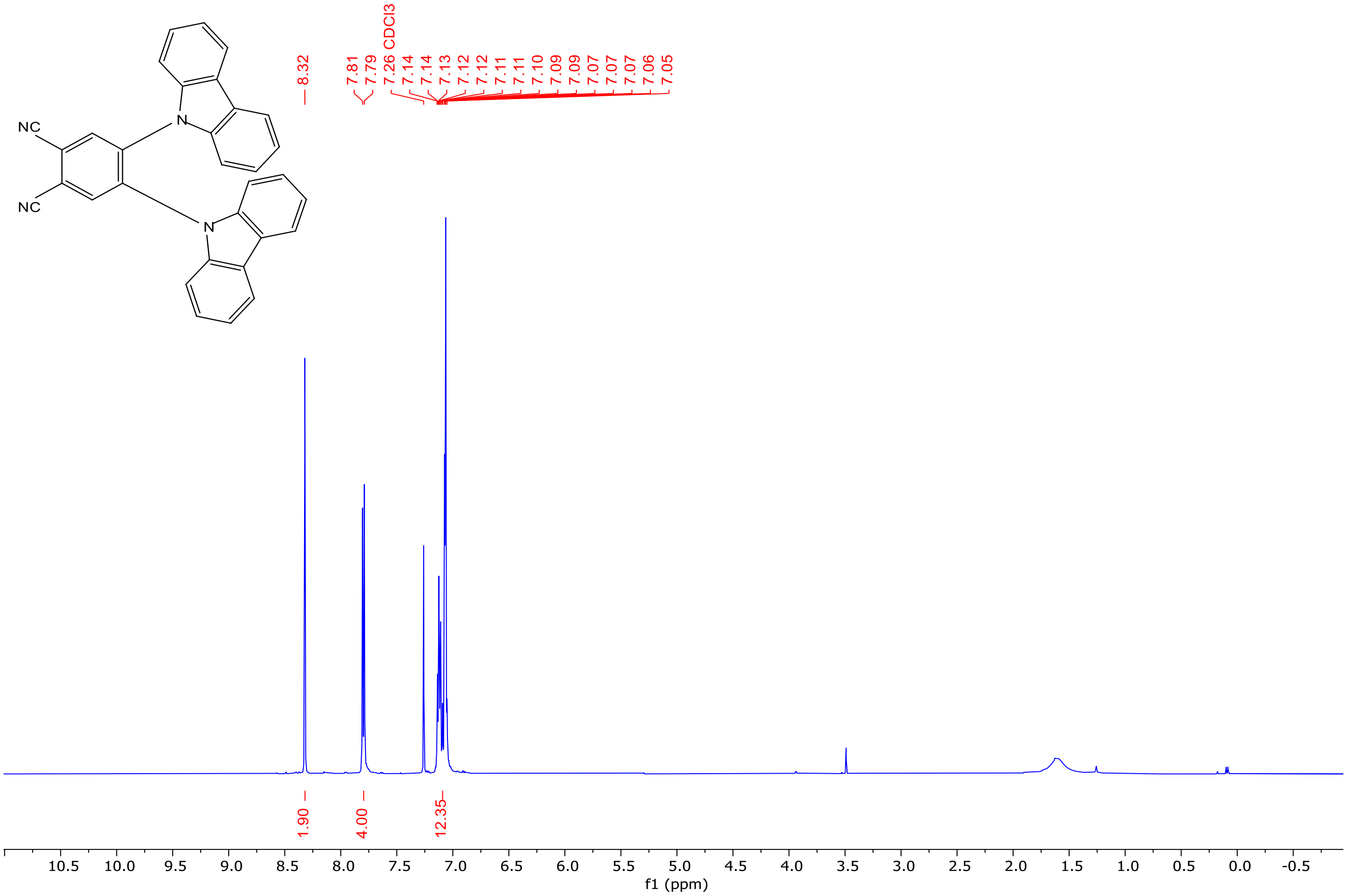

(4-(cyclohexylamino)phenyl)(phenyl)methanone (1)

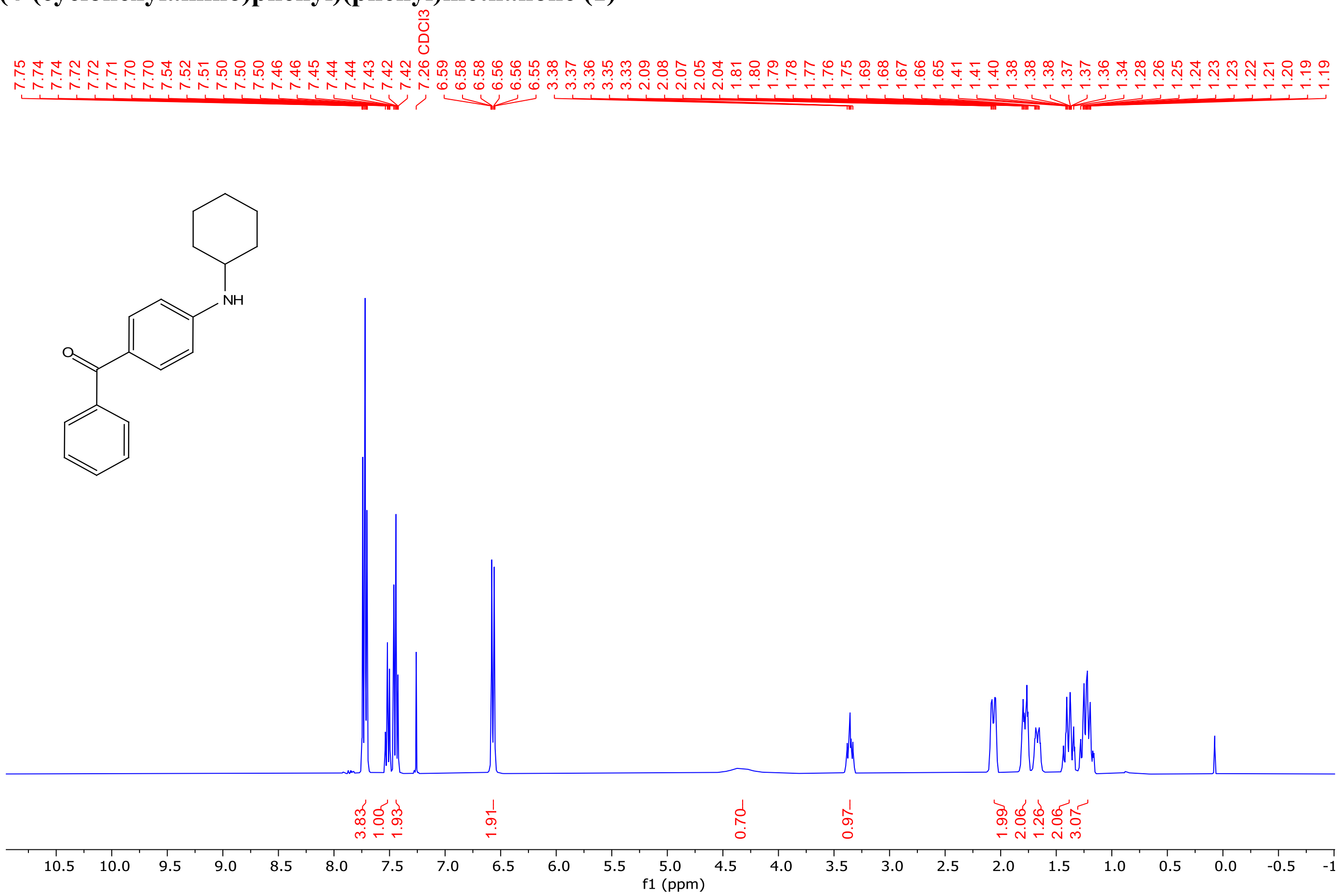

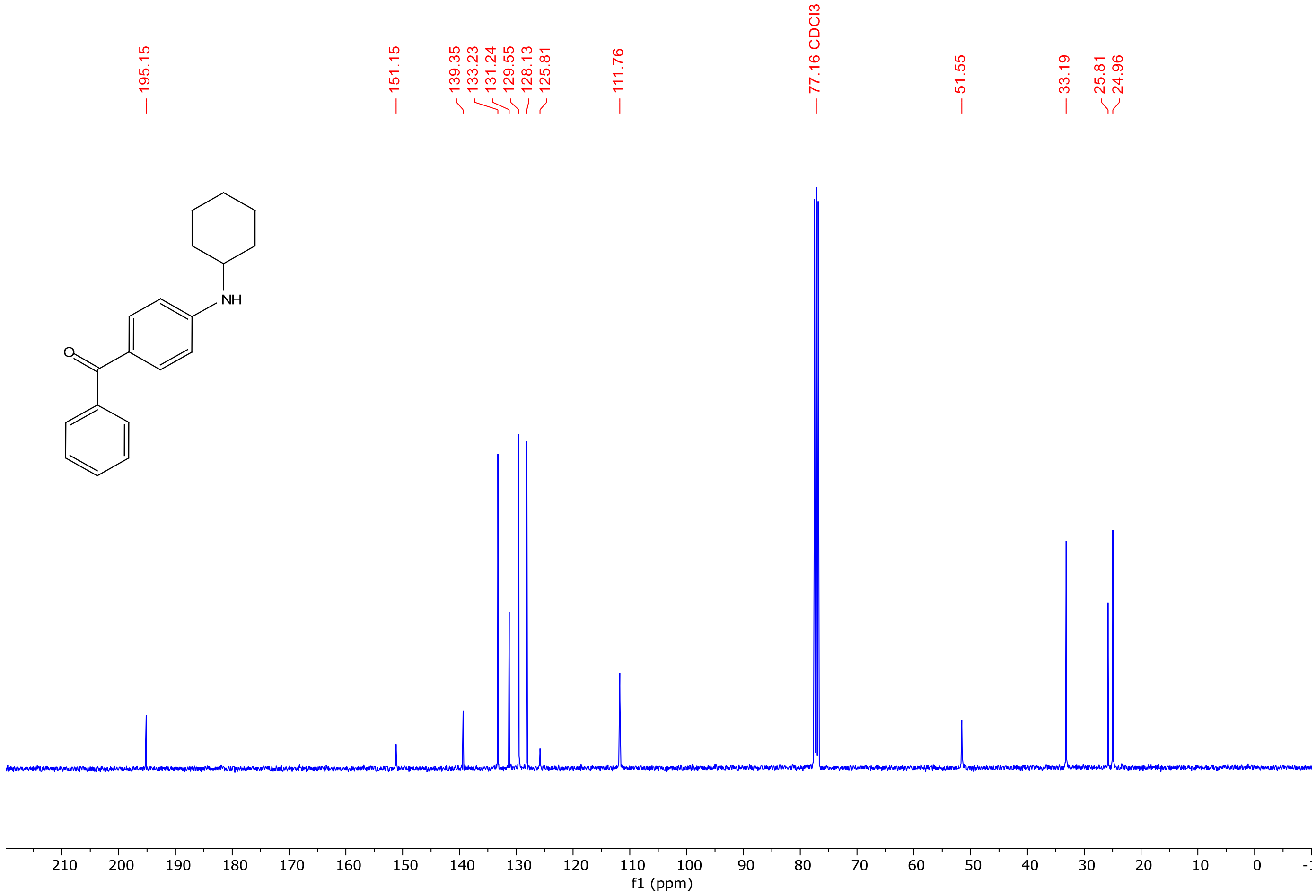

(4-(hexylamino)phenyl)(phenyl)methanone (2)

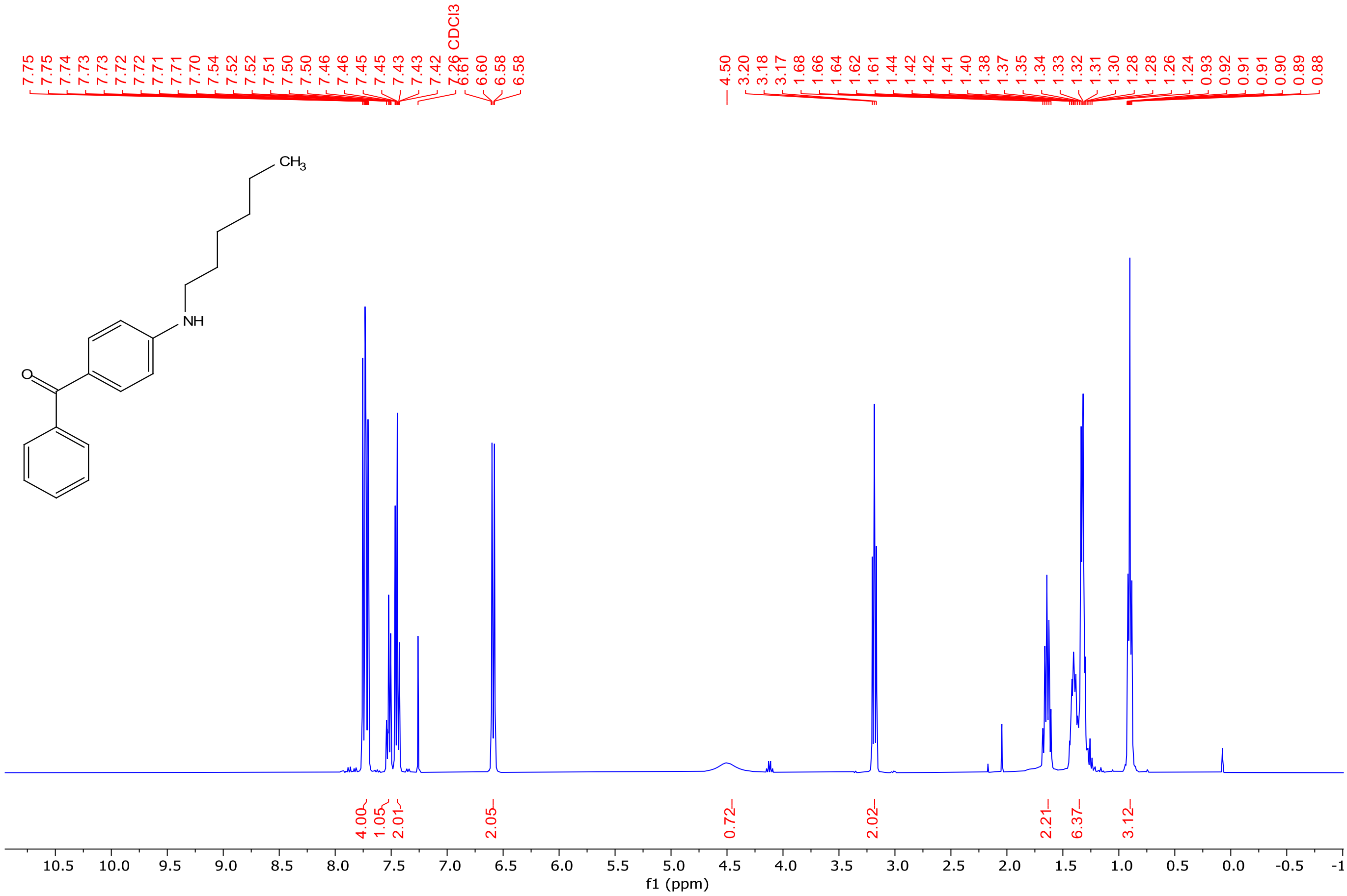

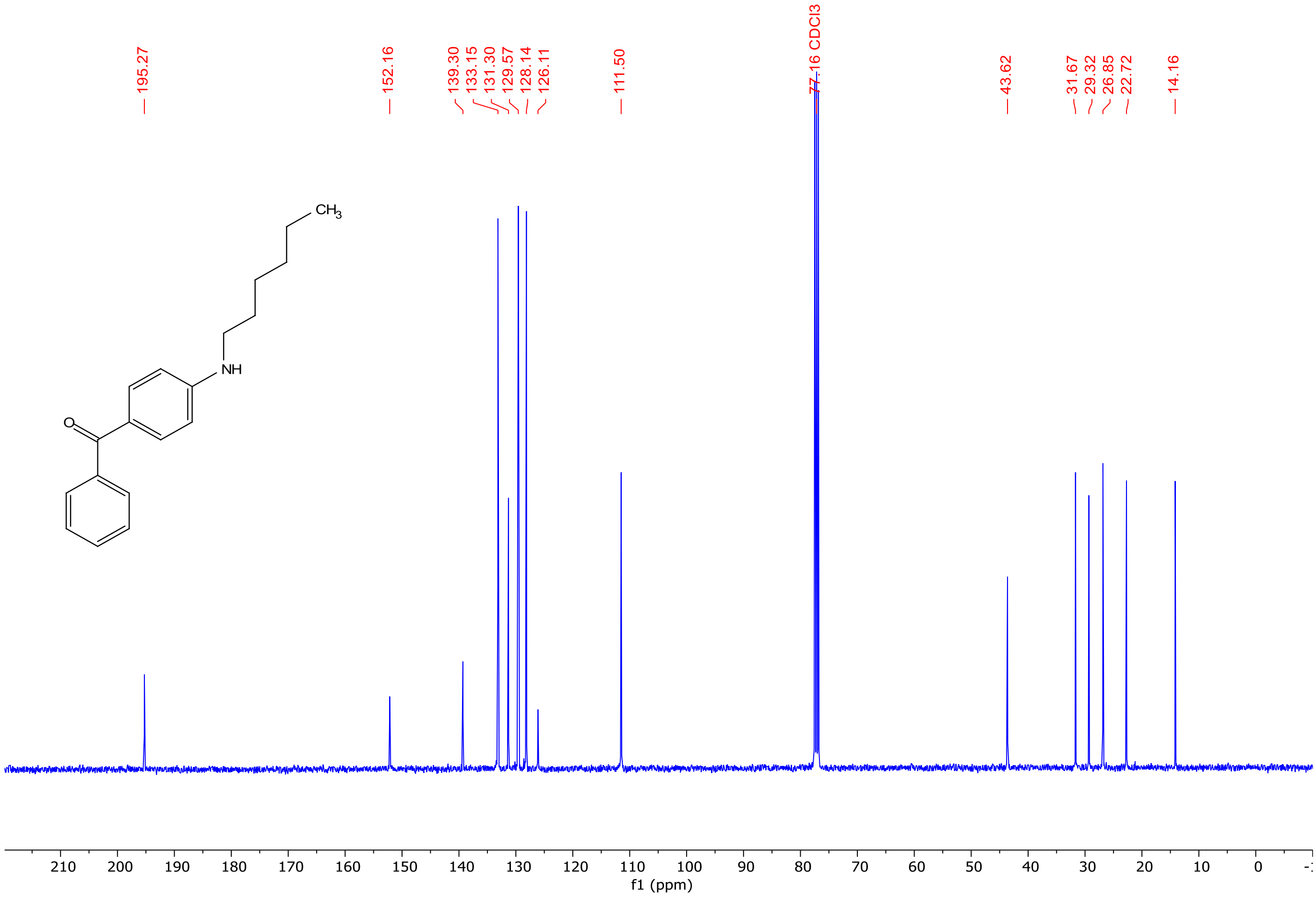

(4-(allylamino)phenyl)(phenyl)methanone (3)

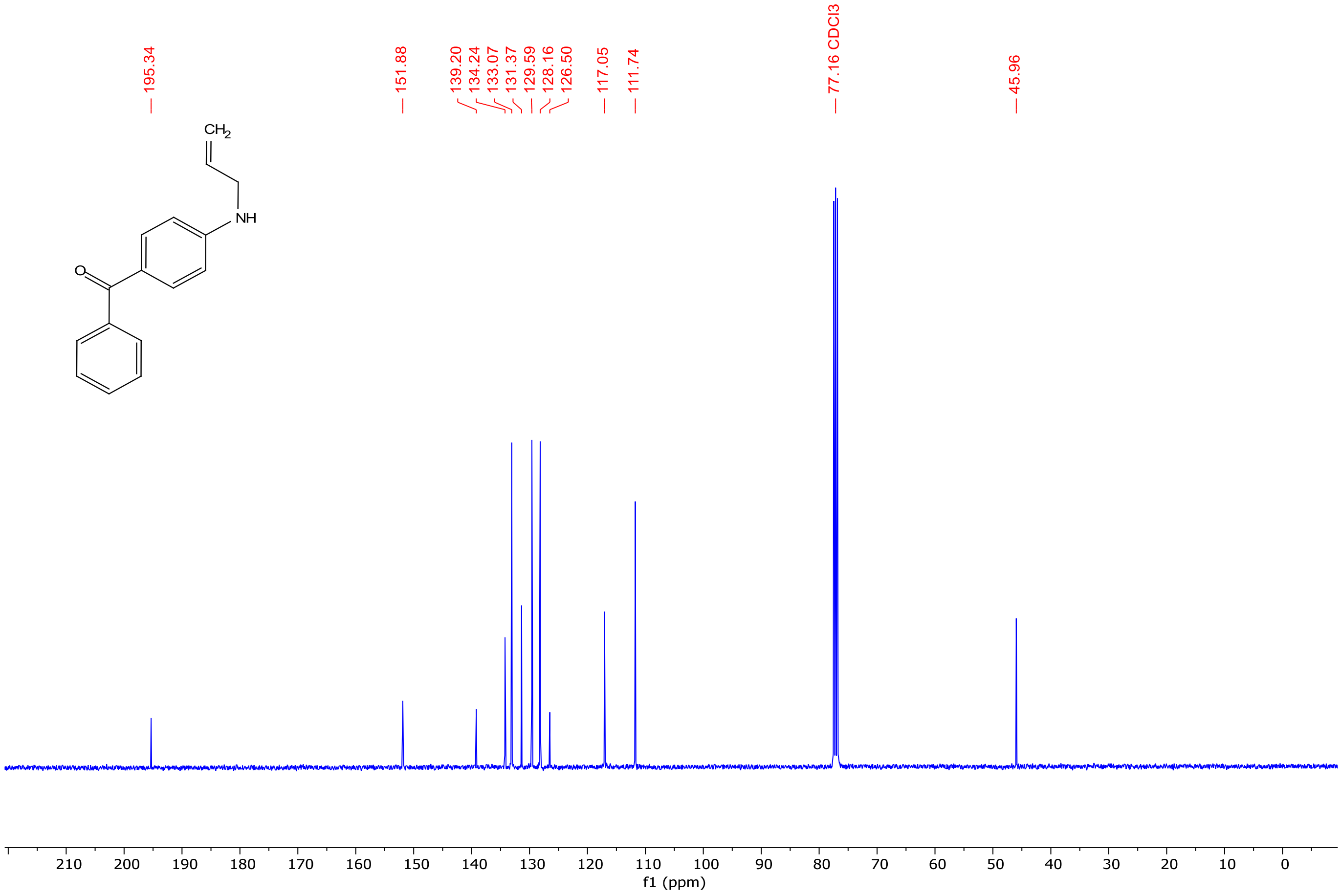

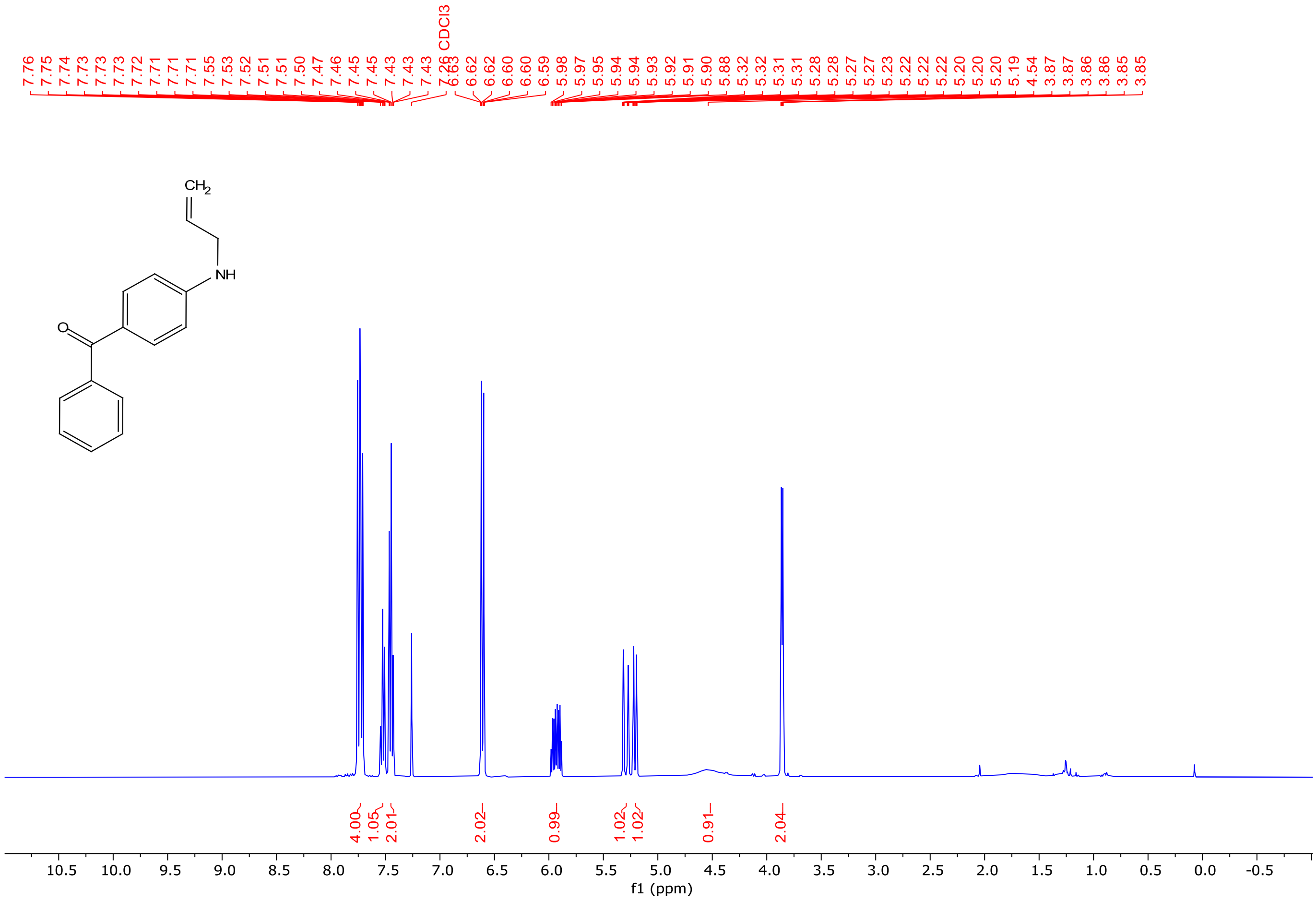

(4-((4-methoxybenzyl)amino)phenyl)(phenyl)methanone (4)

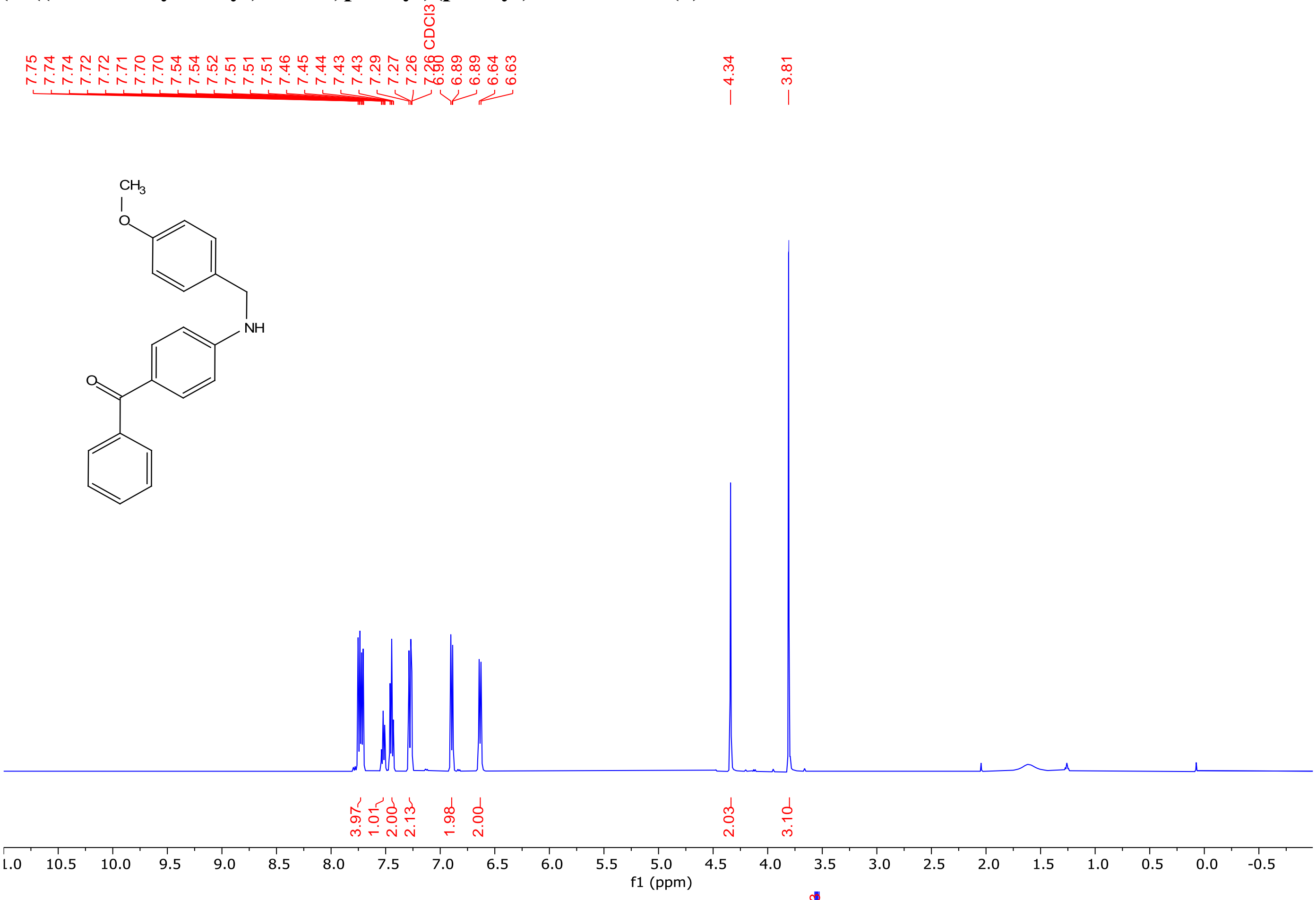

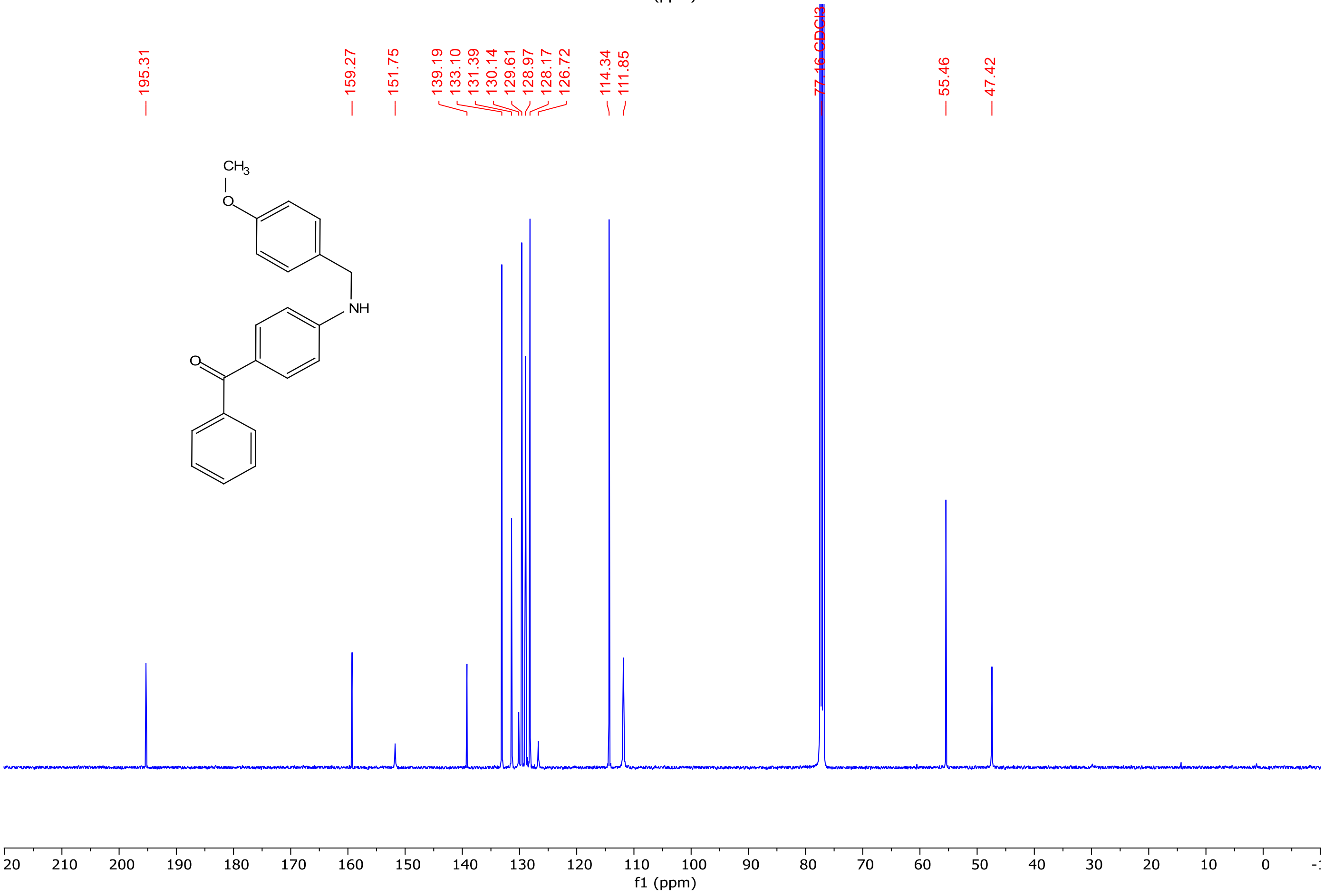

(4-(cyclopropylamino)phenyl)(phenyl)methanone (5)

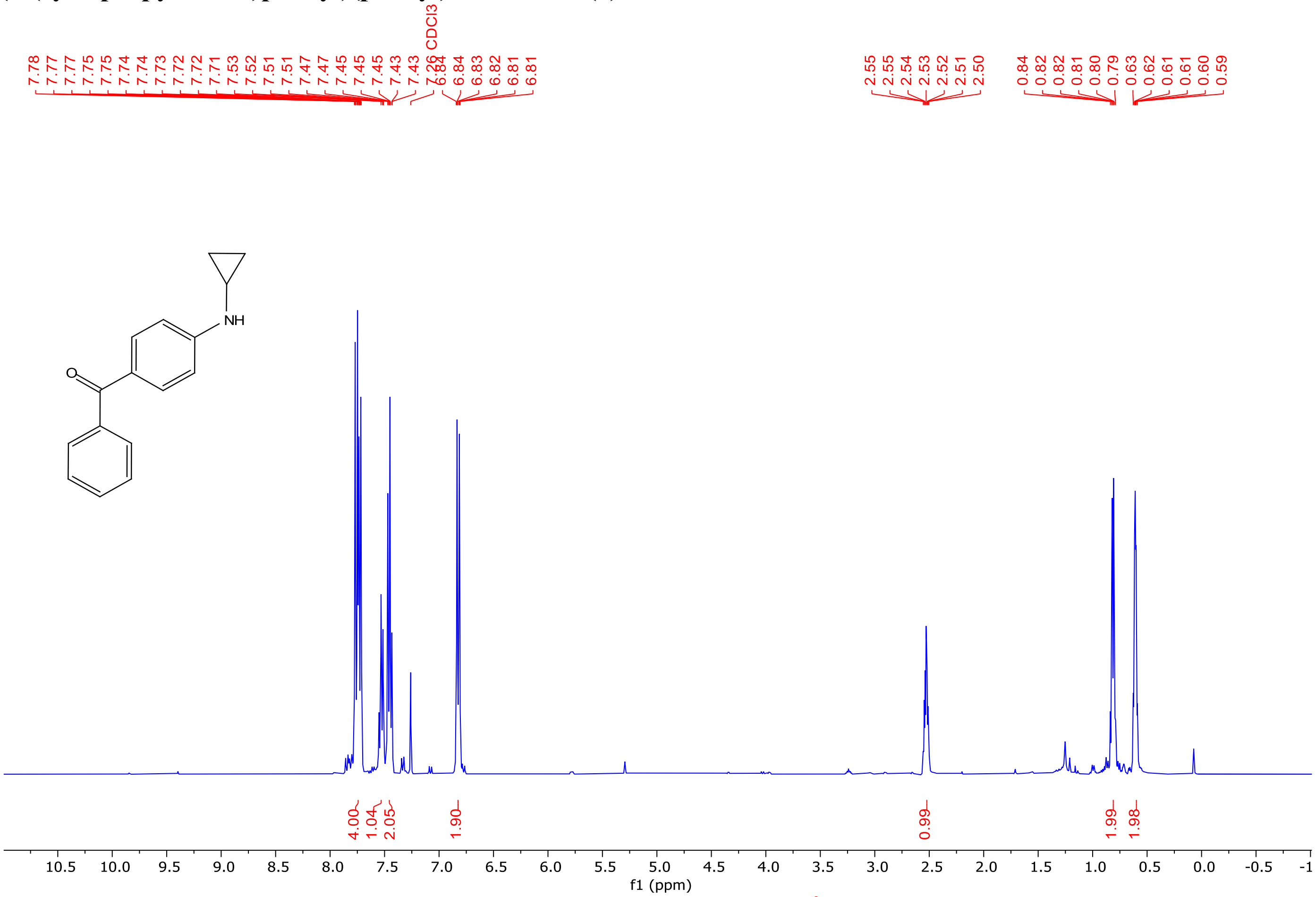

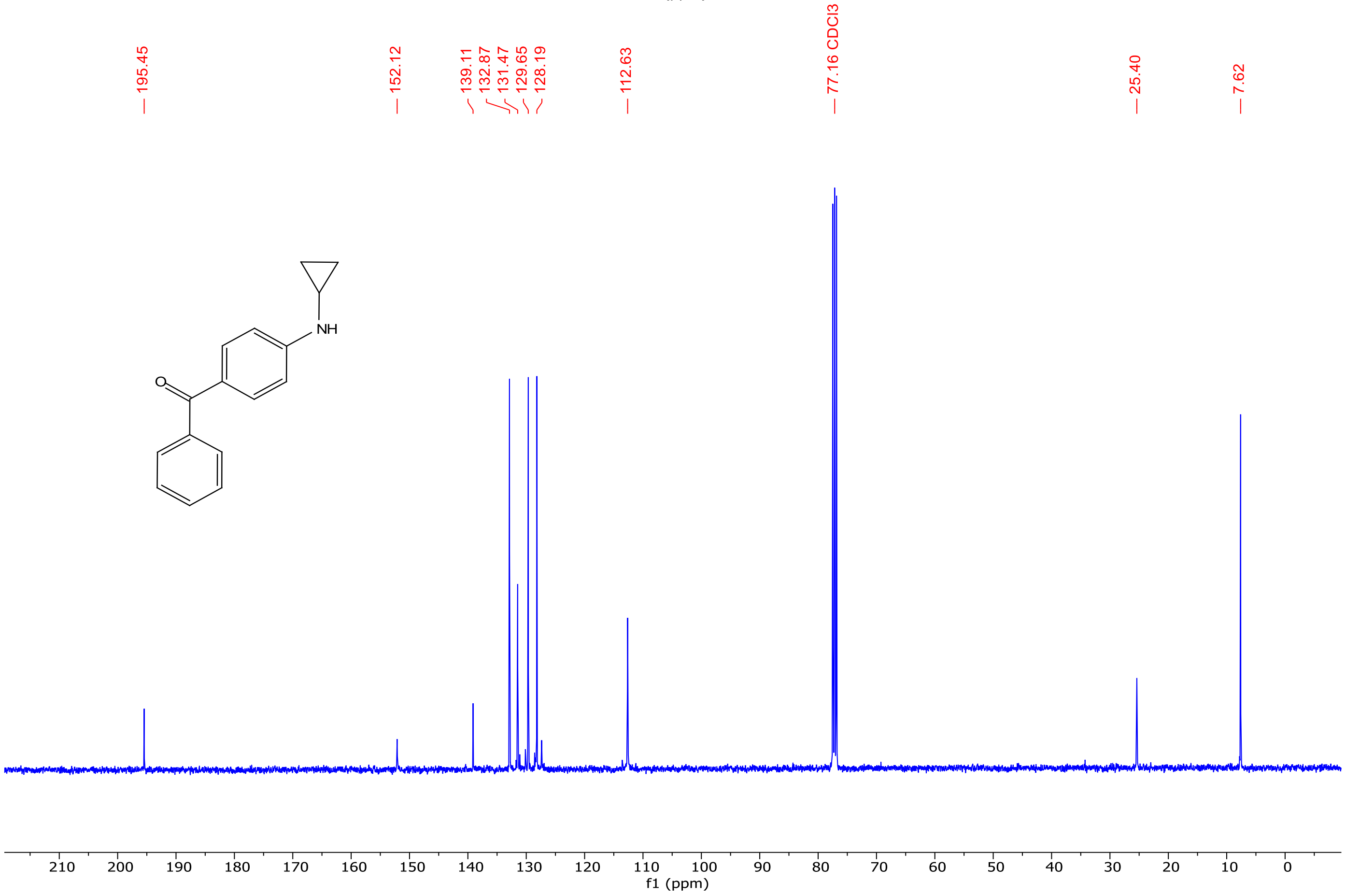

(4-((4-hydroxybutyl)amino)phenyl)(phenyl)methanone (6)

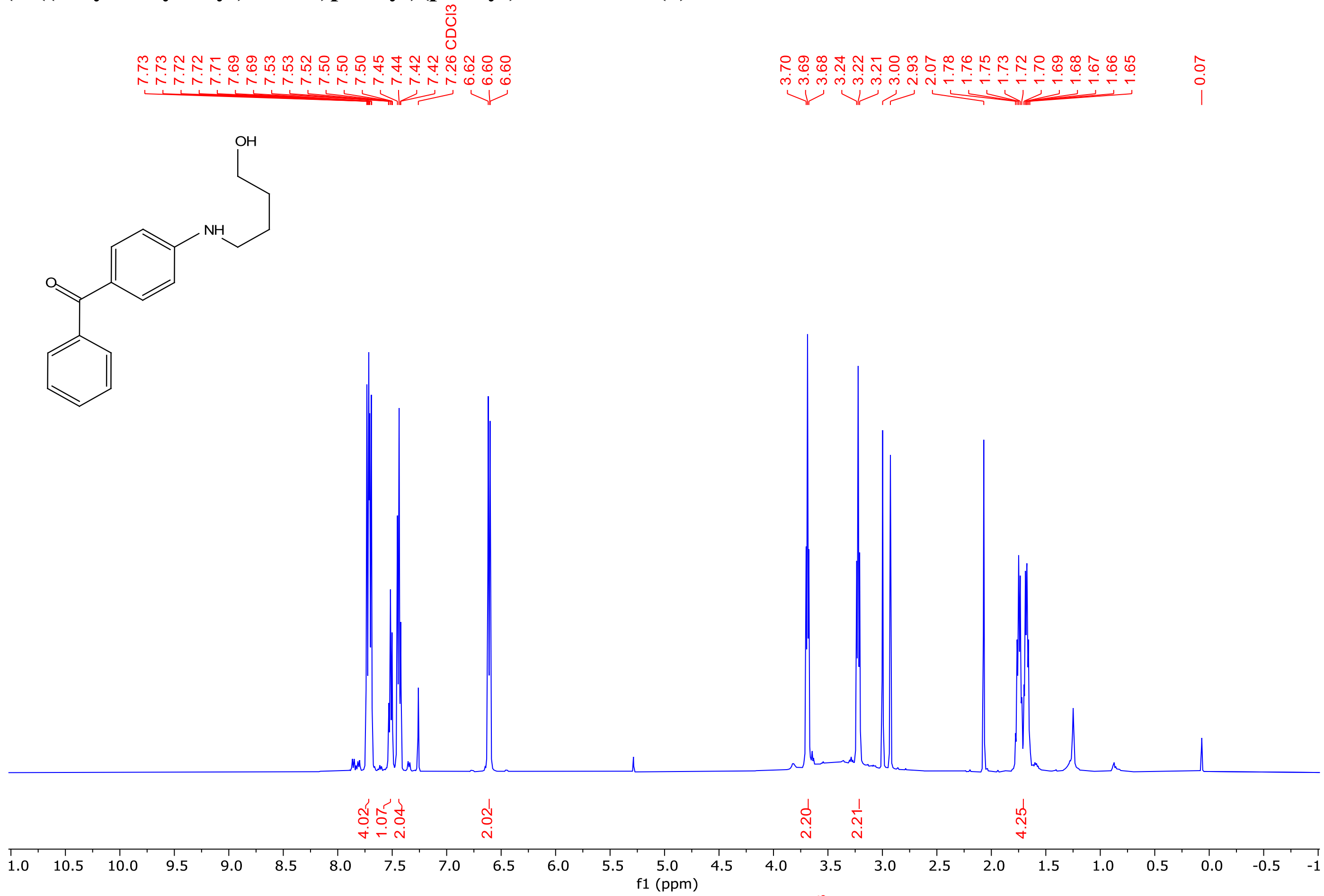

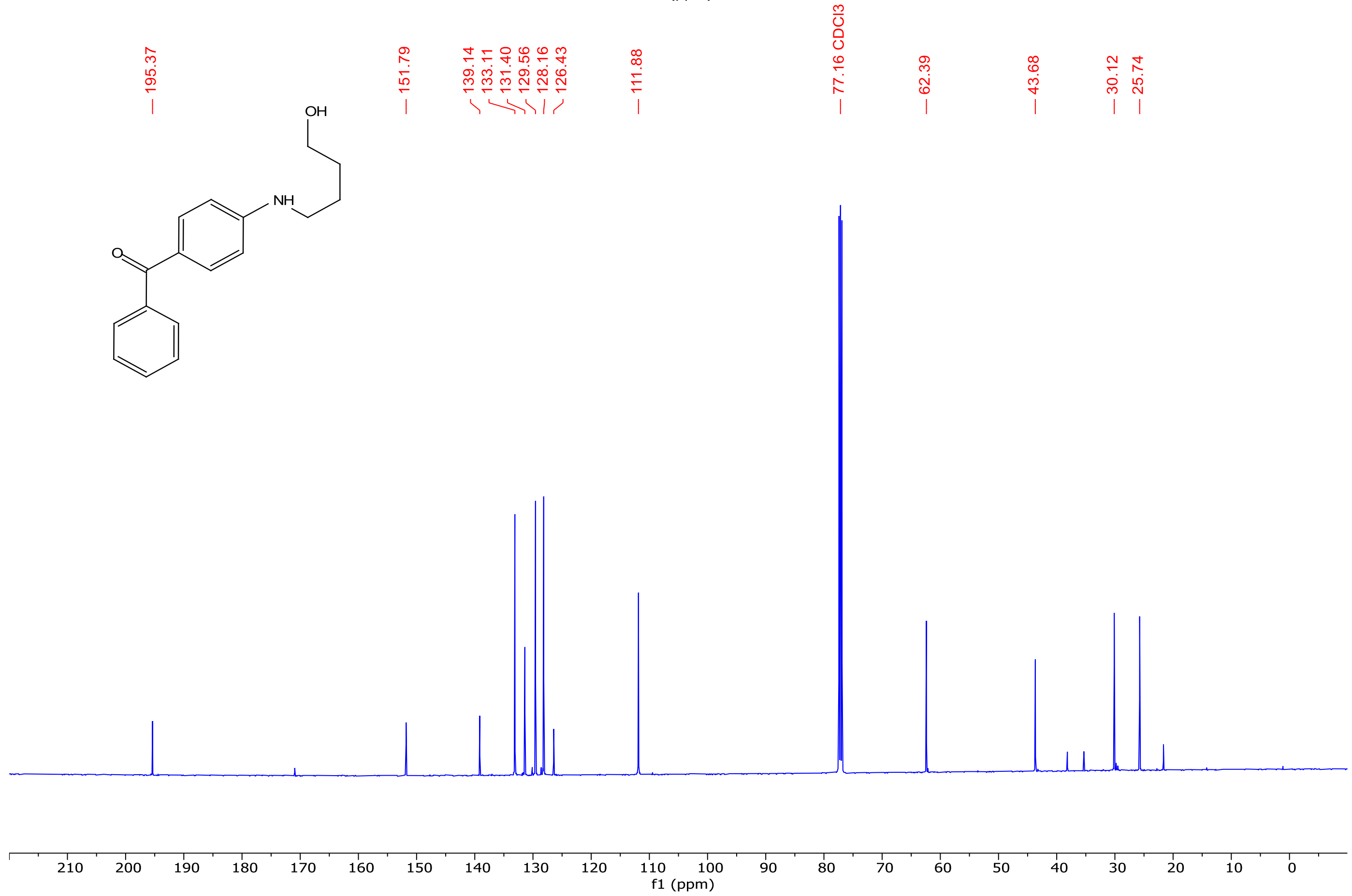

(4-(morpholinoamino)phenyl)(phenyl)methanone (7)

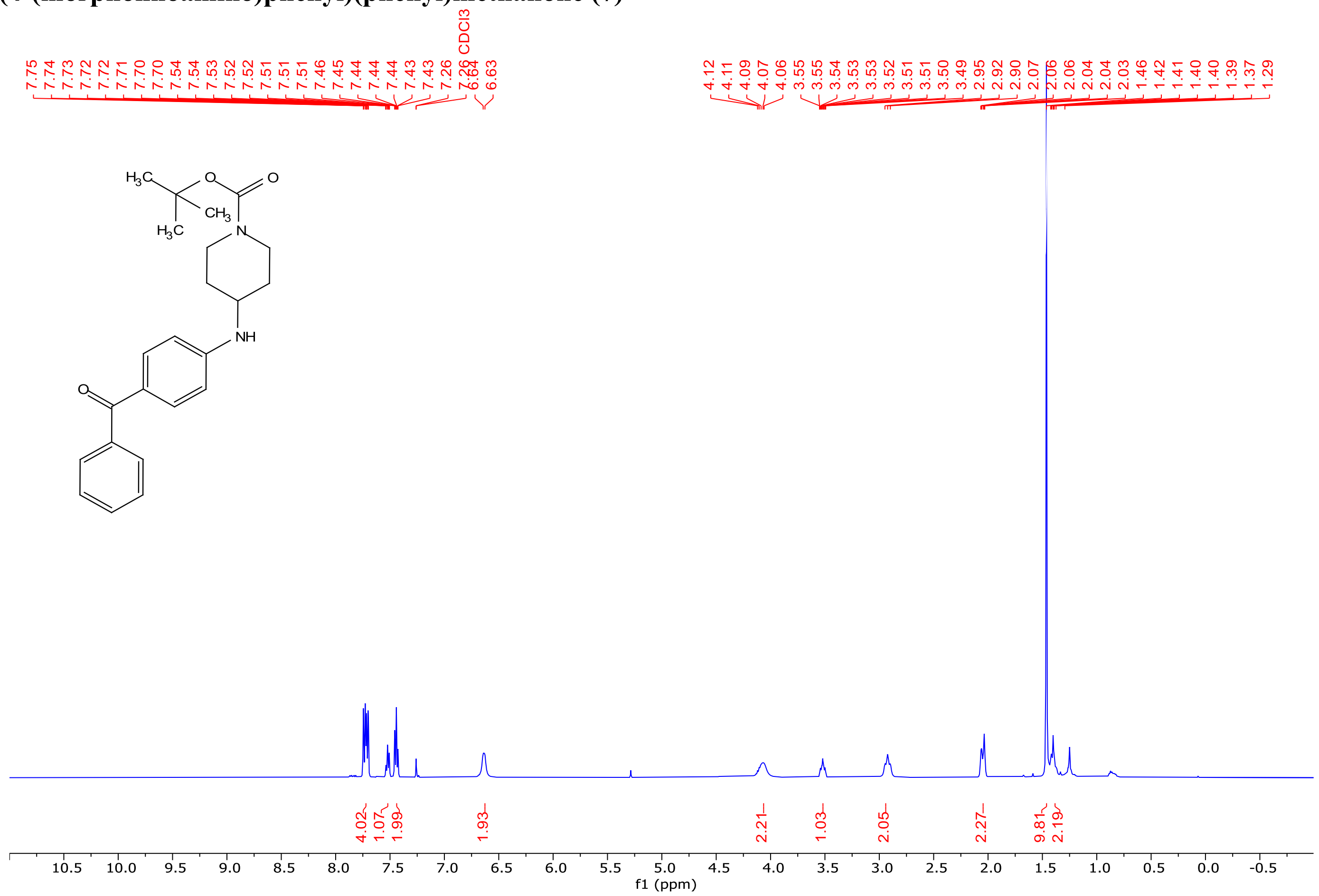

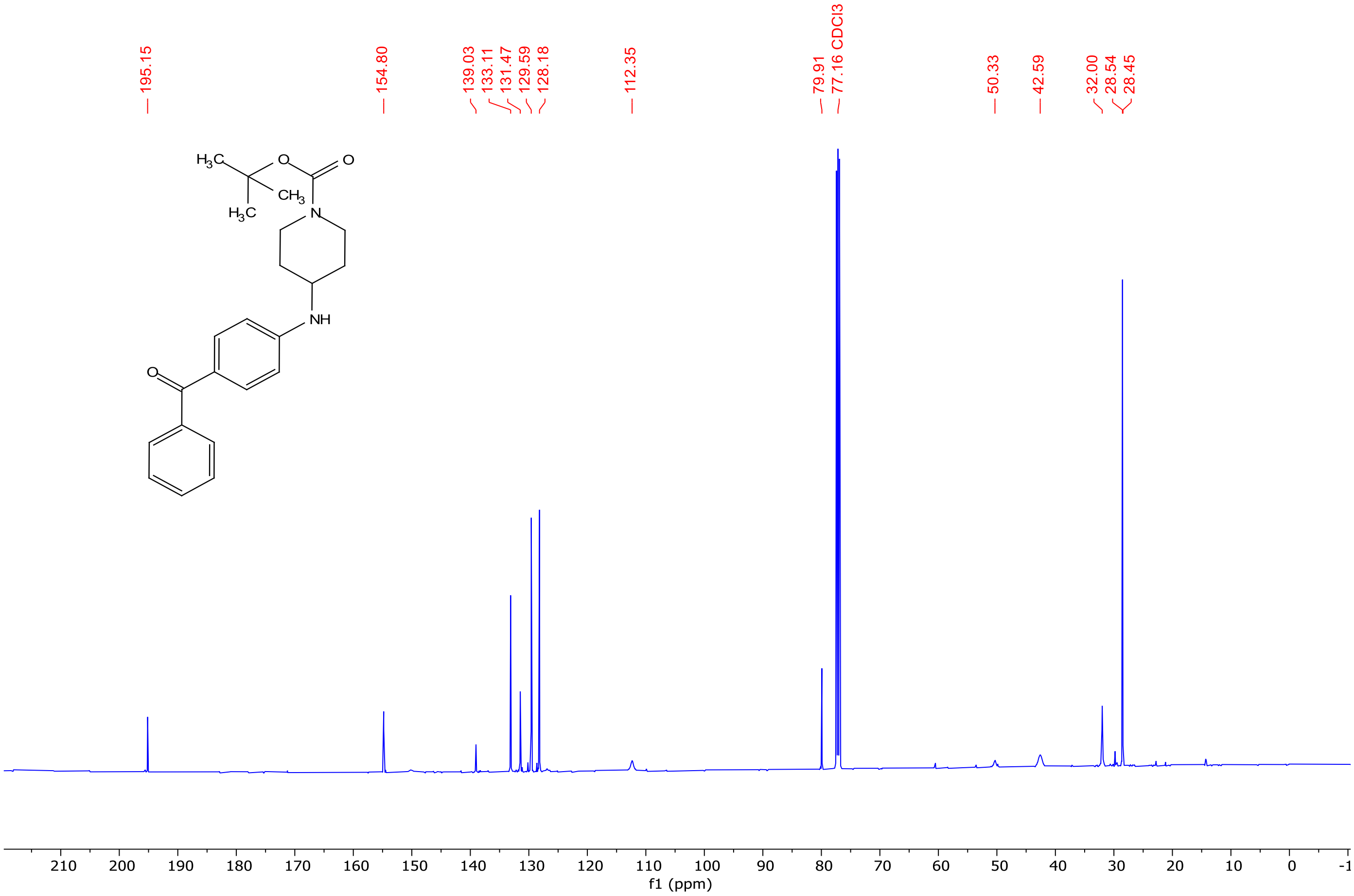

(4-(morpholinoamino)phenyl)(phenyl)methanone (8)

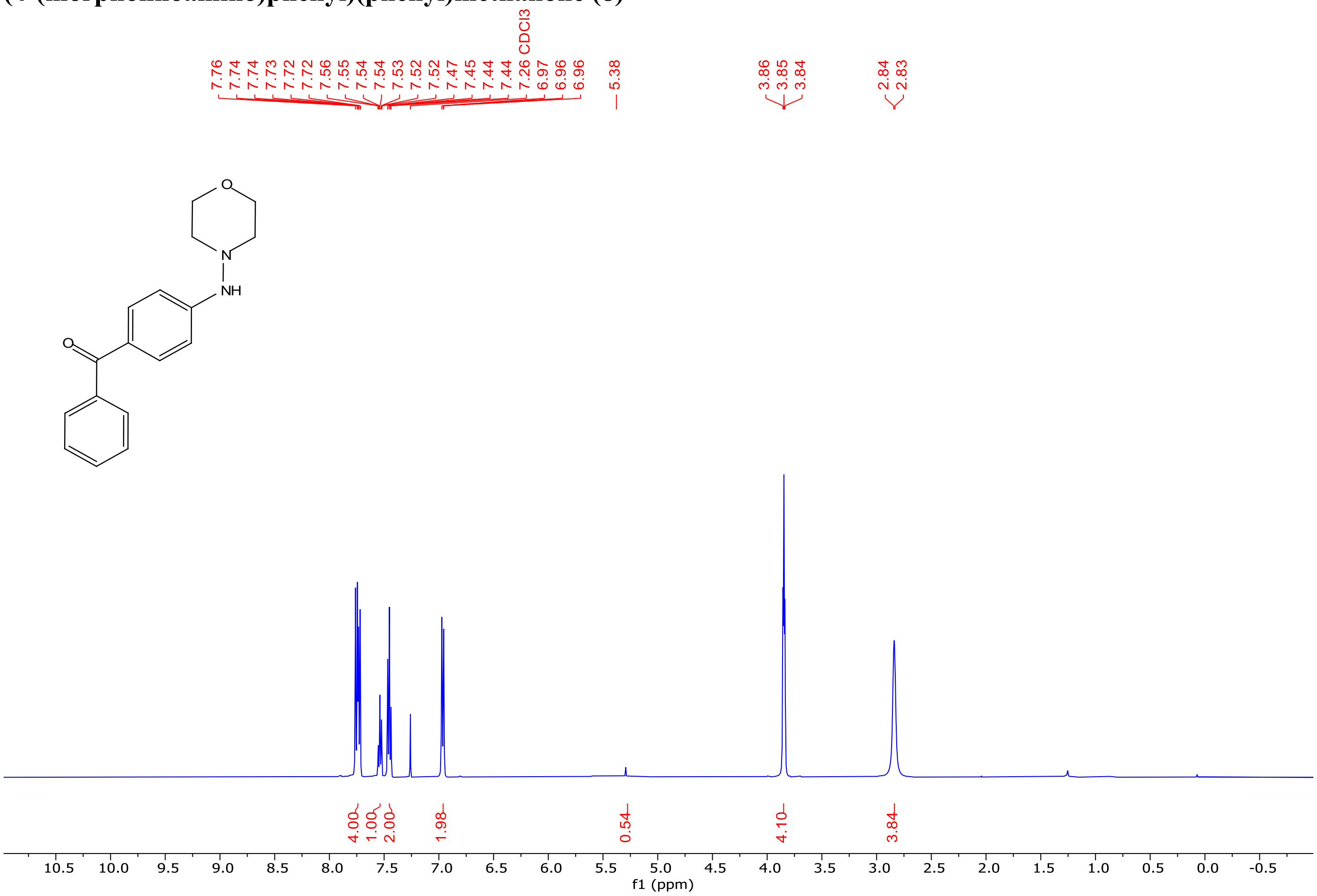

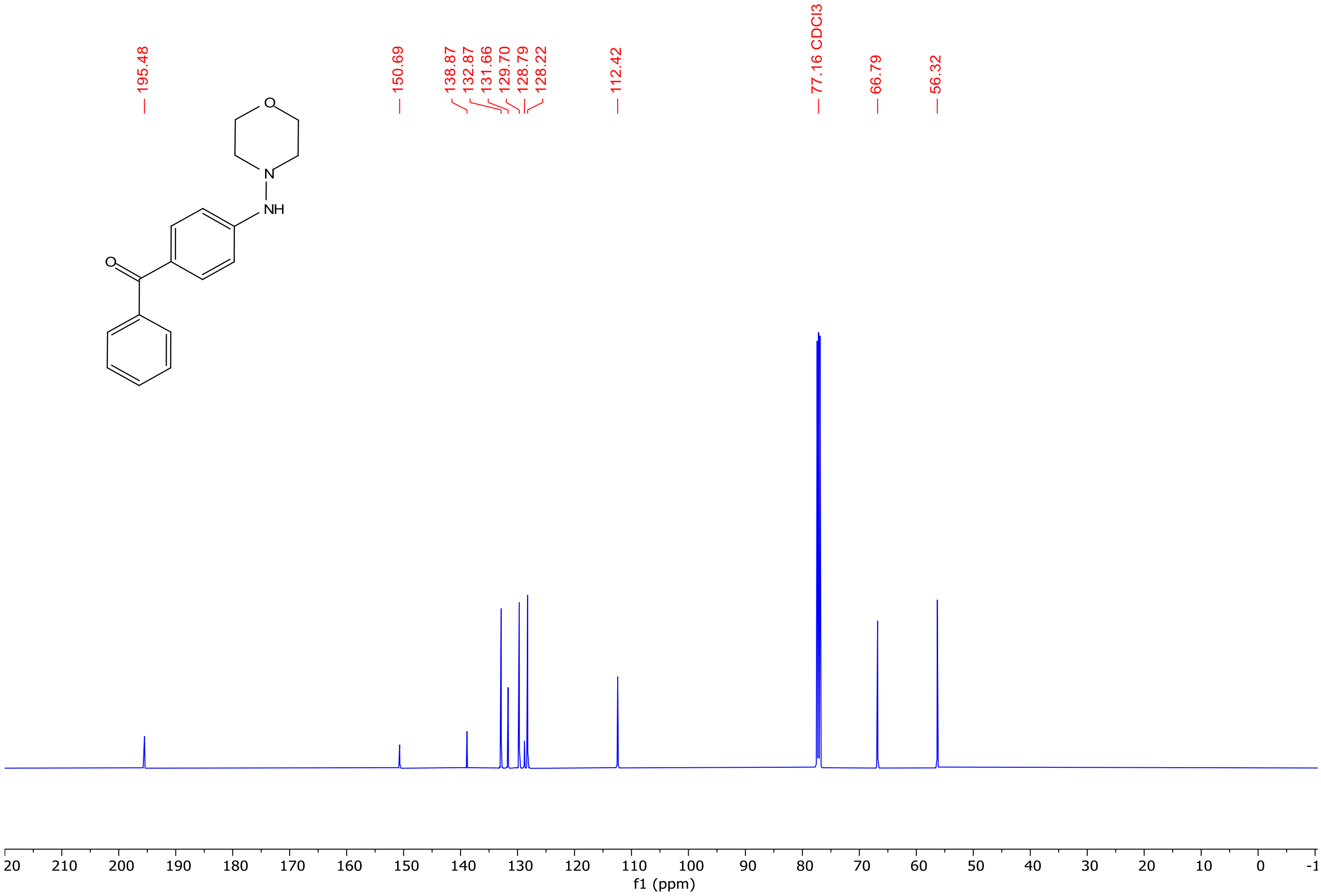

1-([1,1'-biphenyl]-4-yl)piperidine (9)

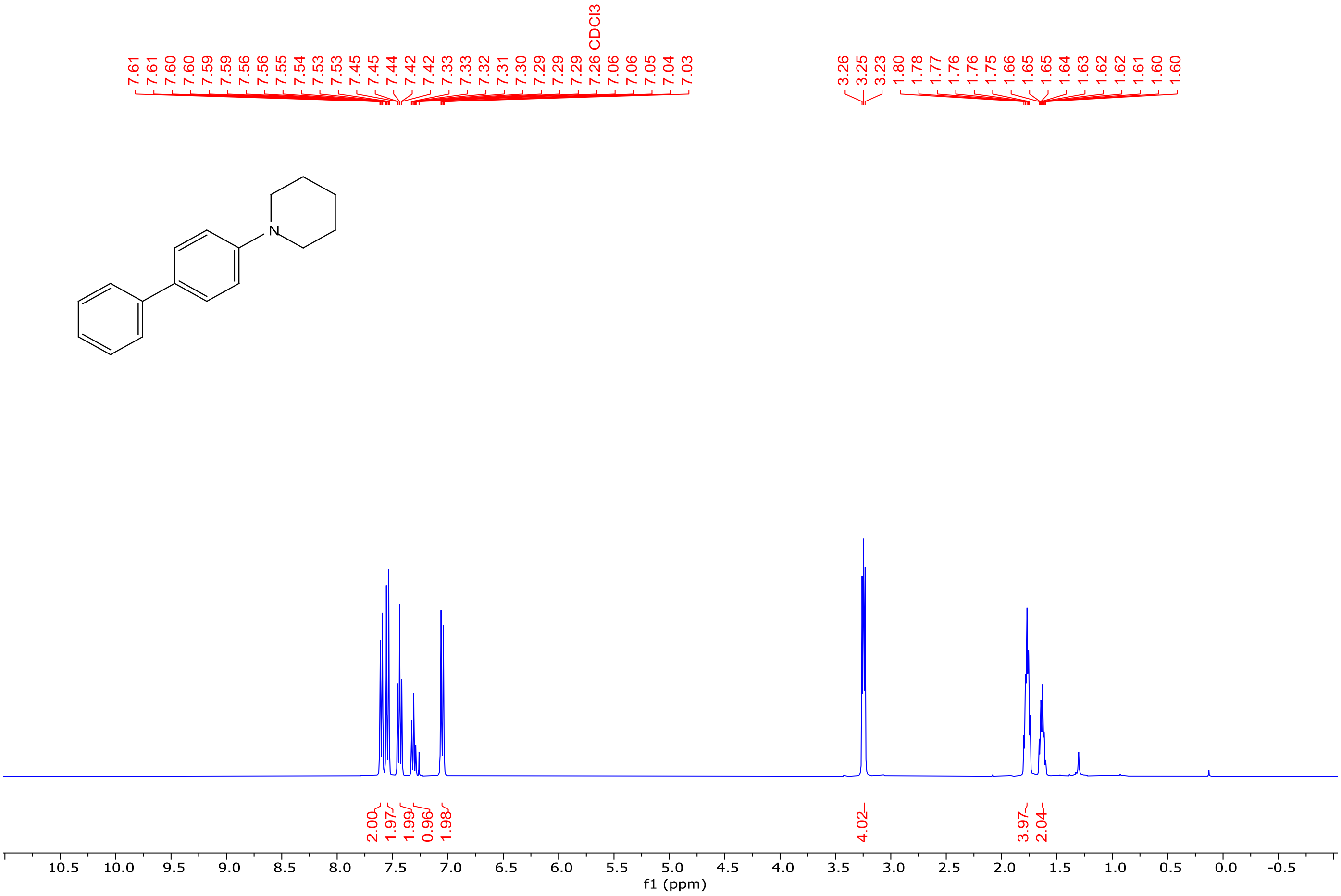

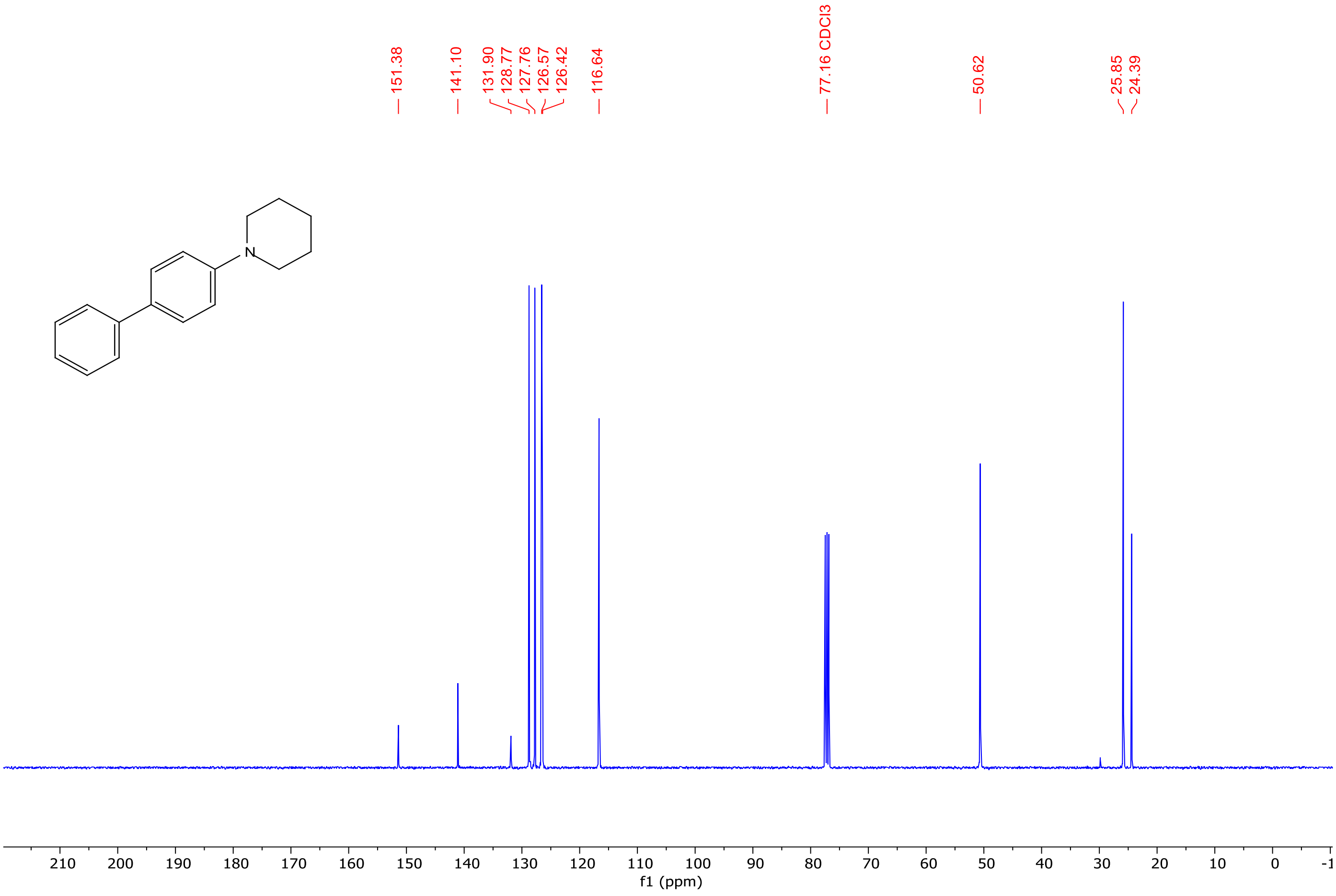

1-phenylpiperidine (10)

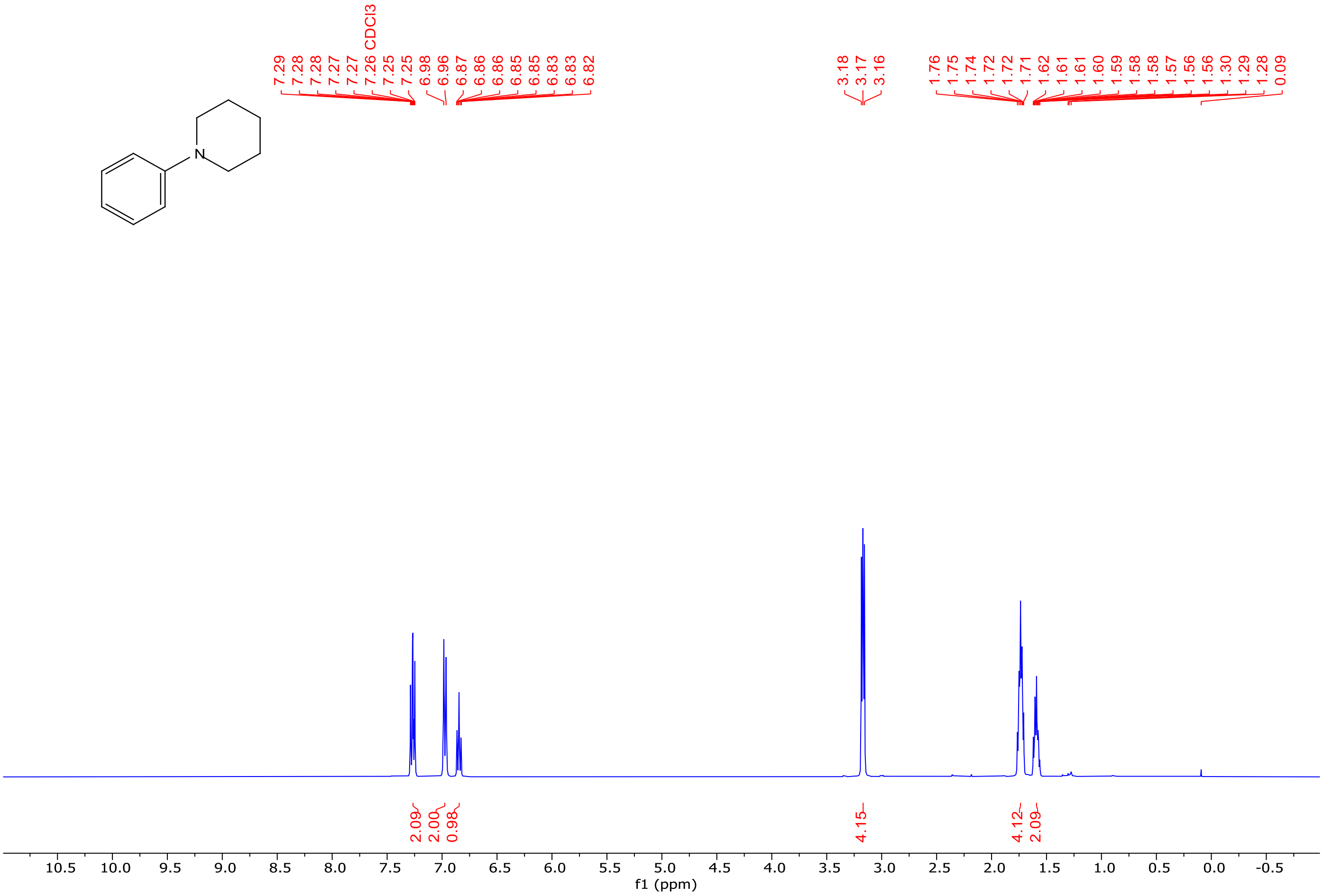

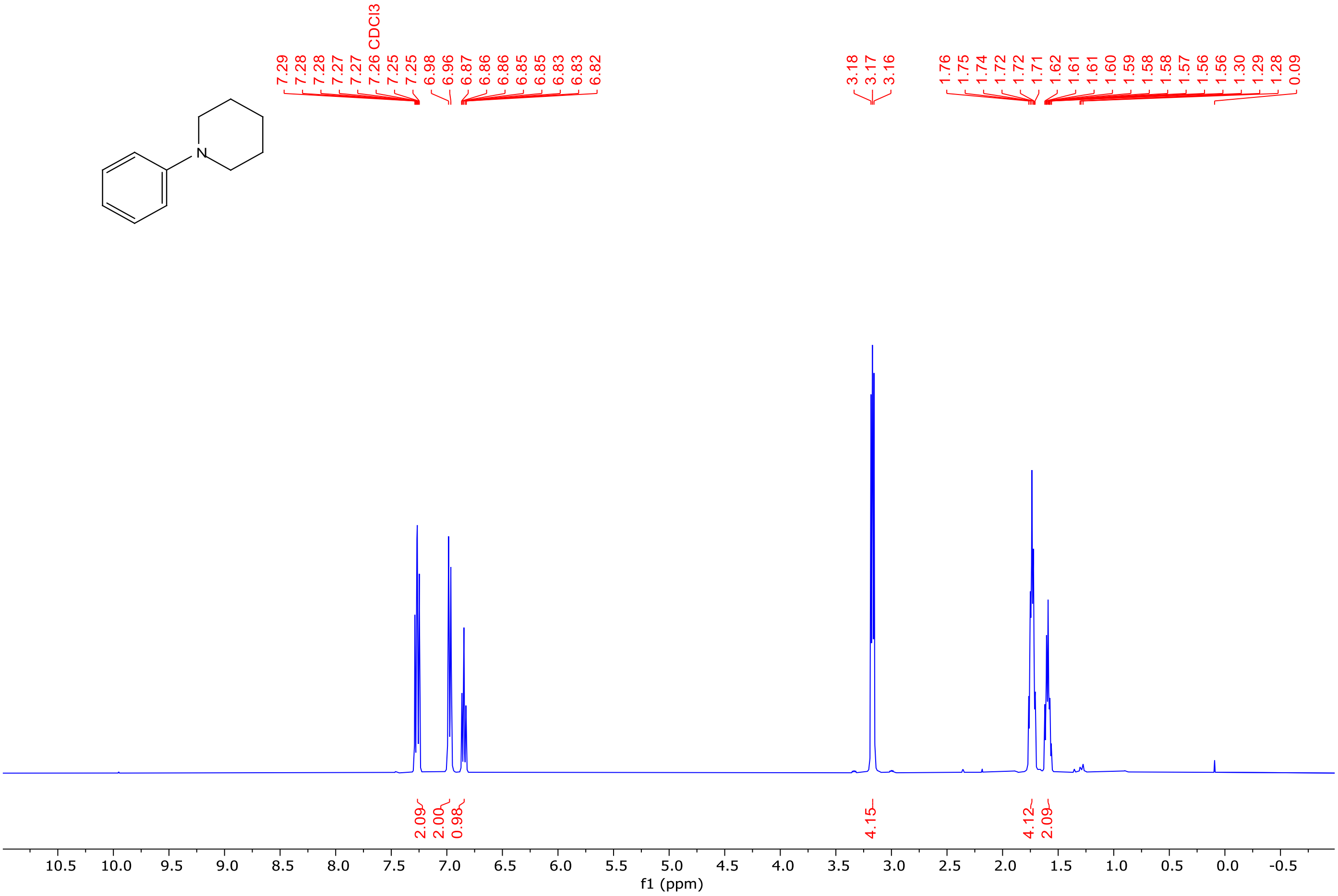

1-(4-(*tert*-butyl)phenyl)piperidine (11)

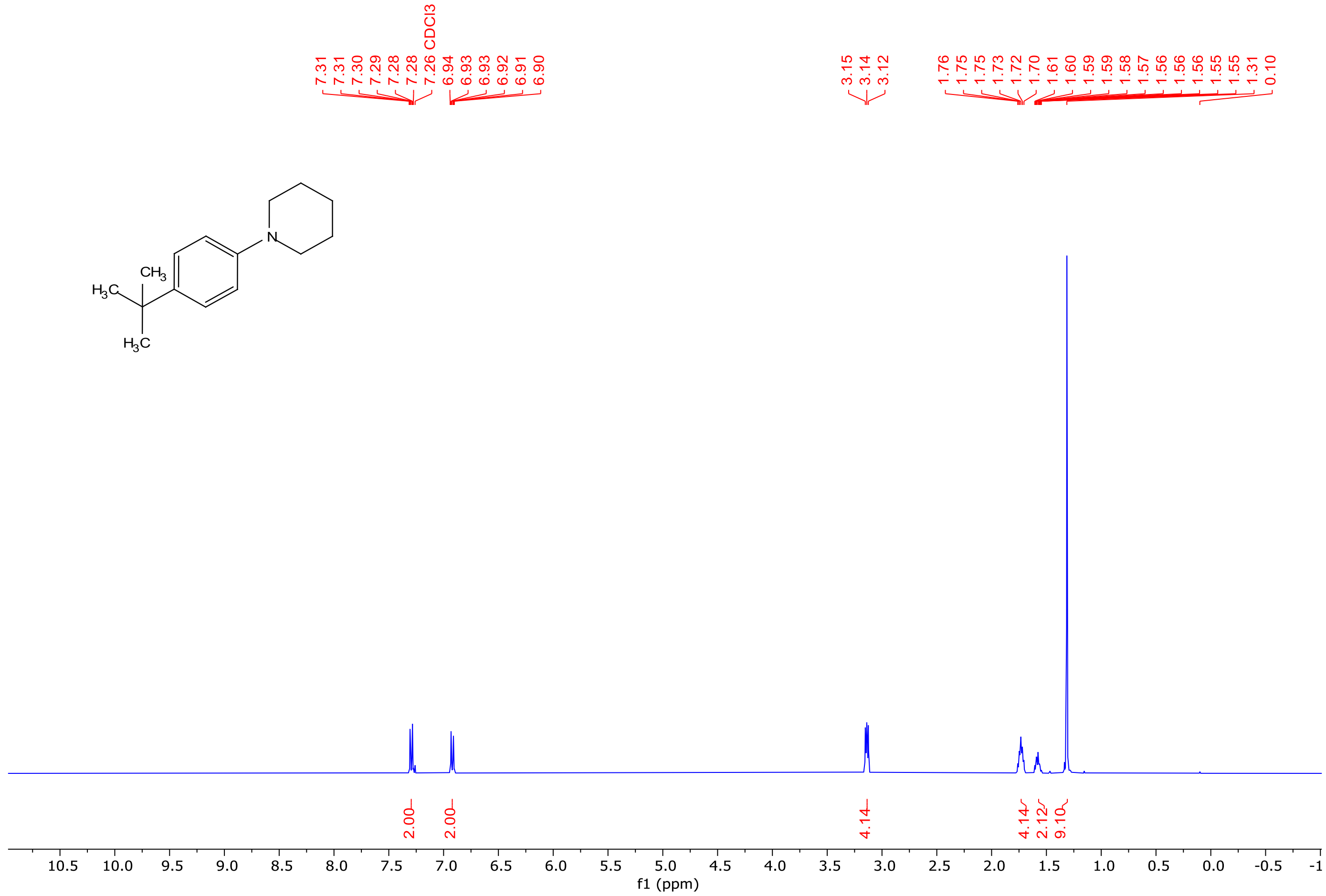

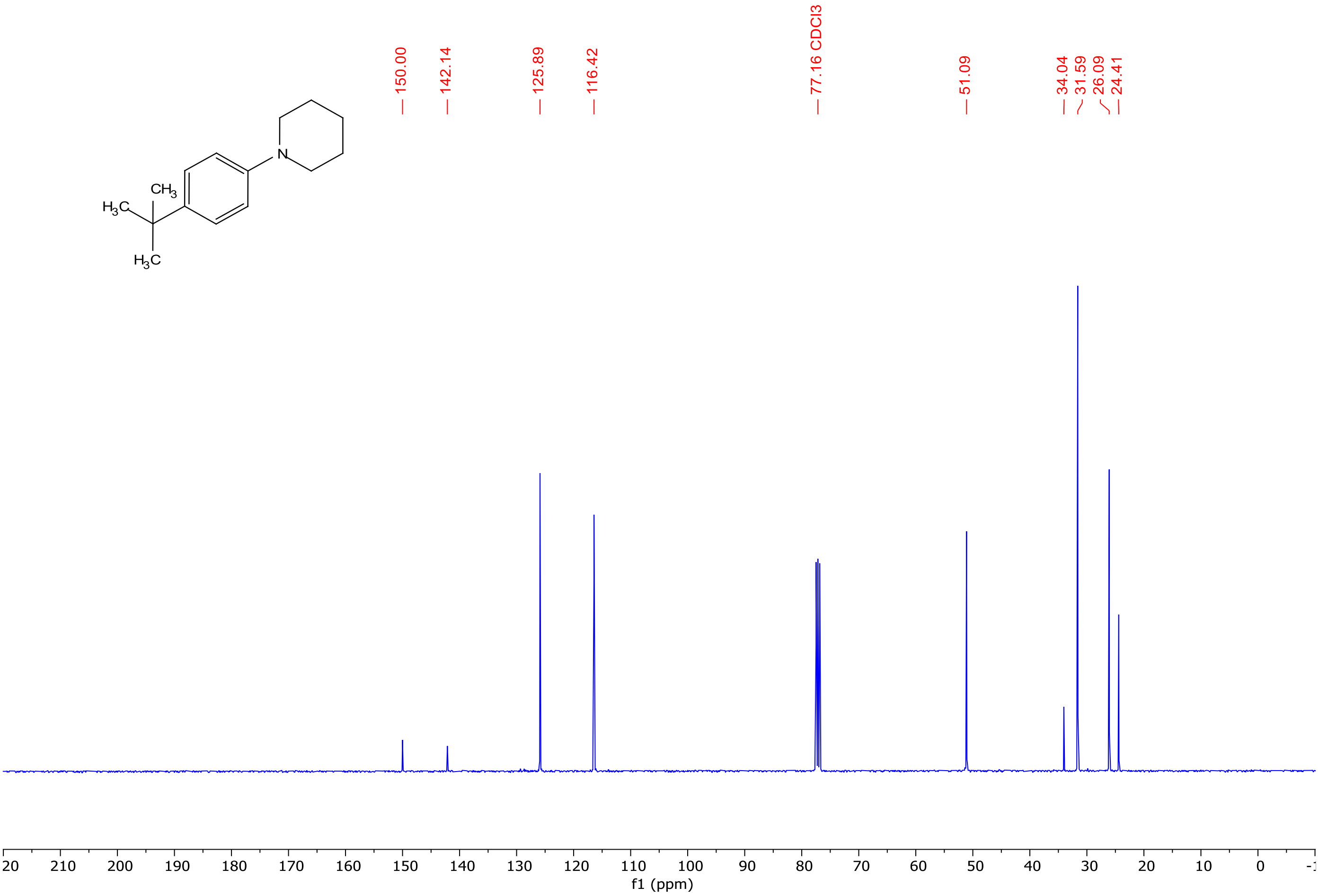

1-(4-(trimethylsilyl)phenyl)piperidine (12)

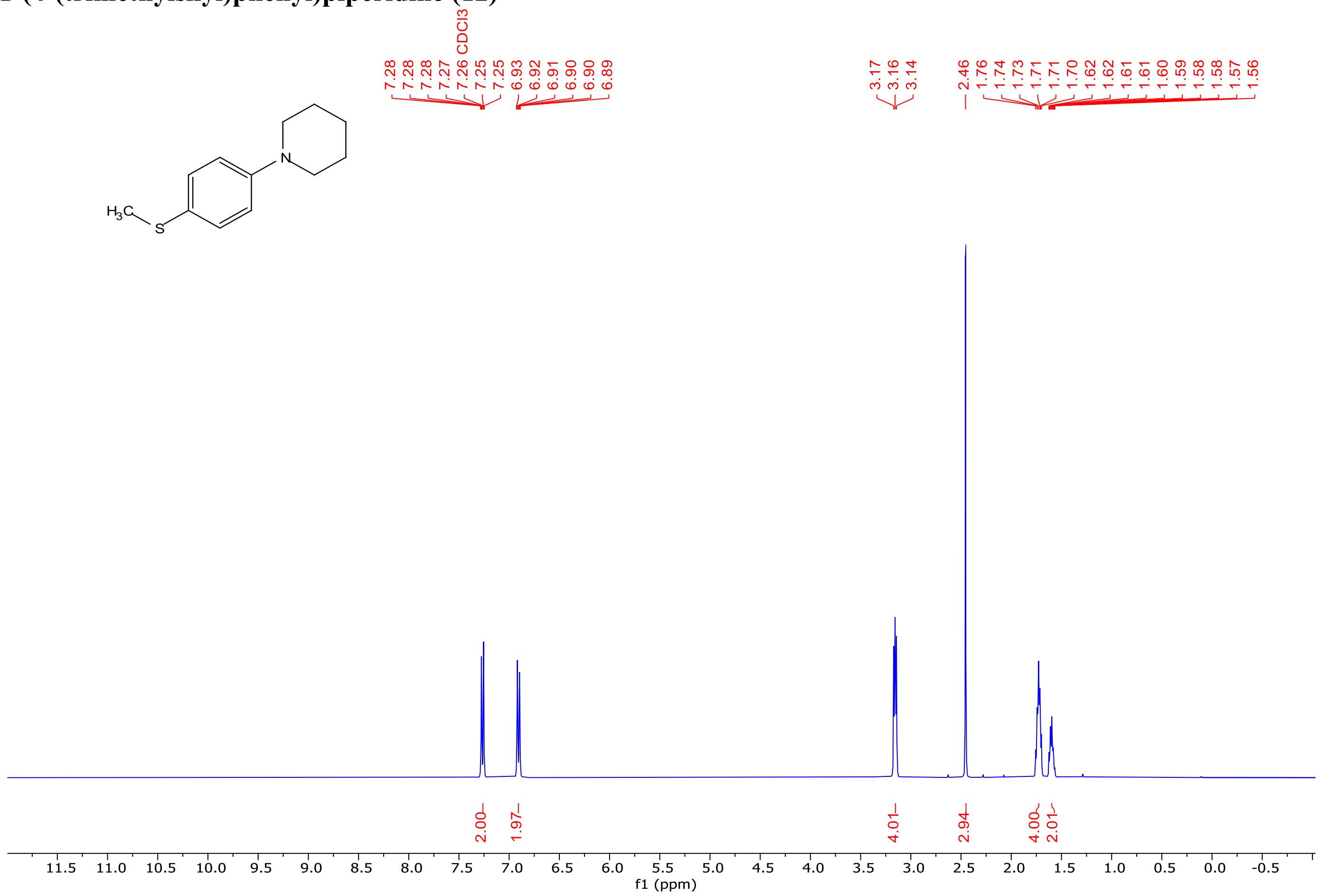

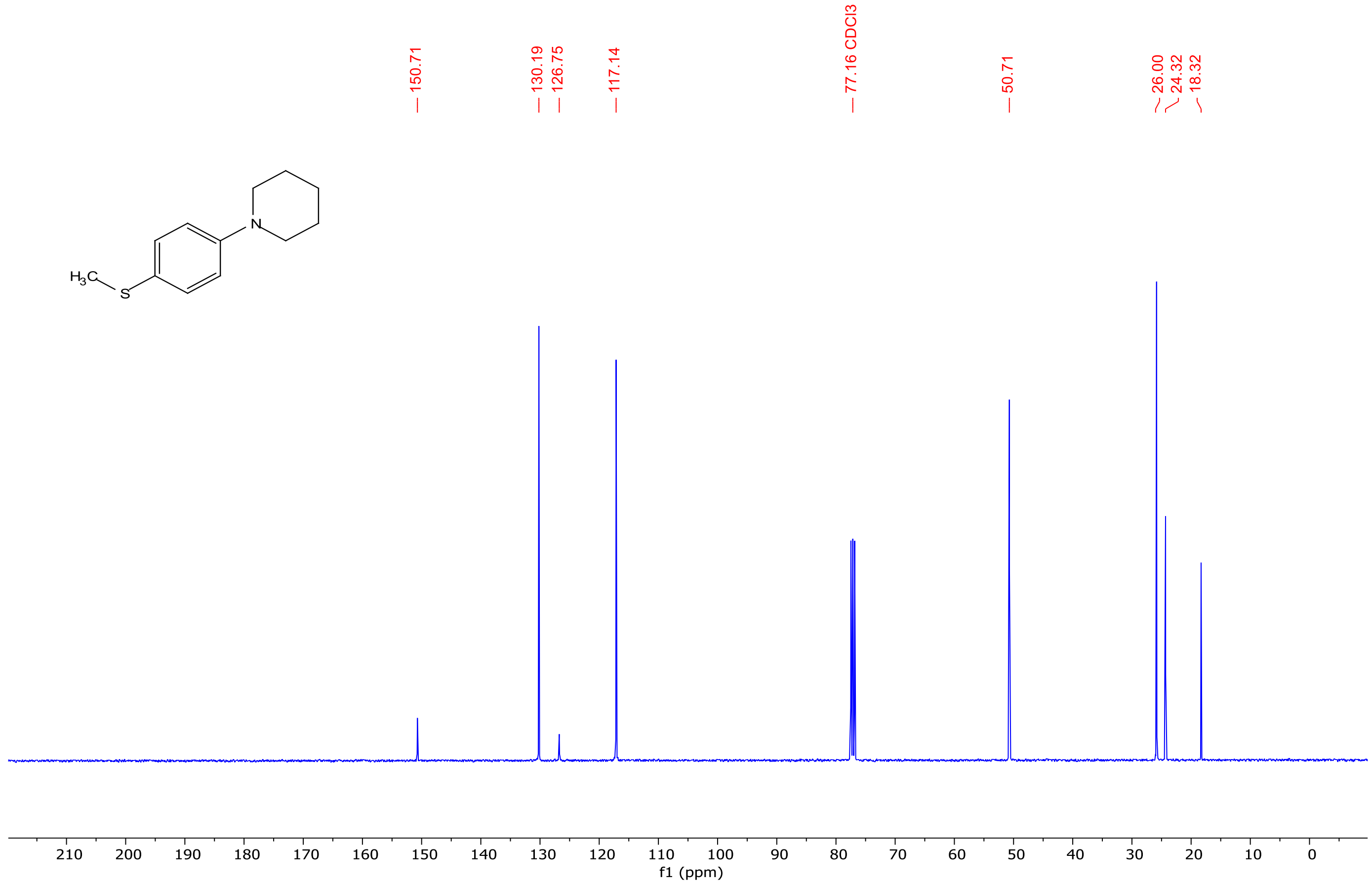

1-(4-(trimethylsilyl)phenyl)piperidine (13)

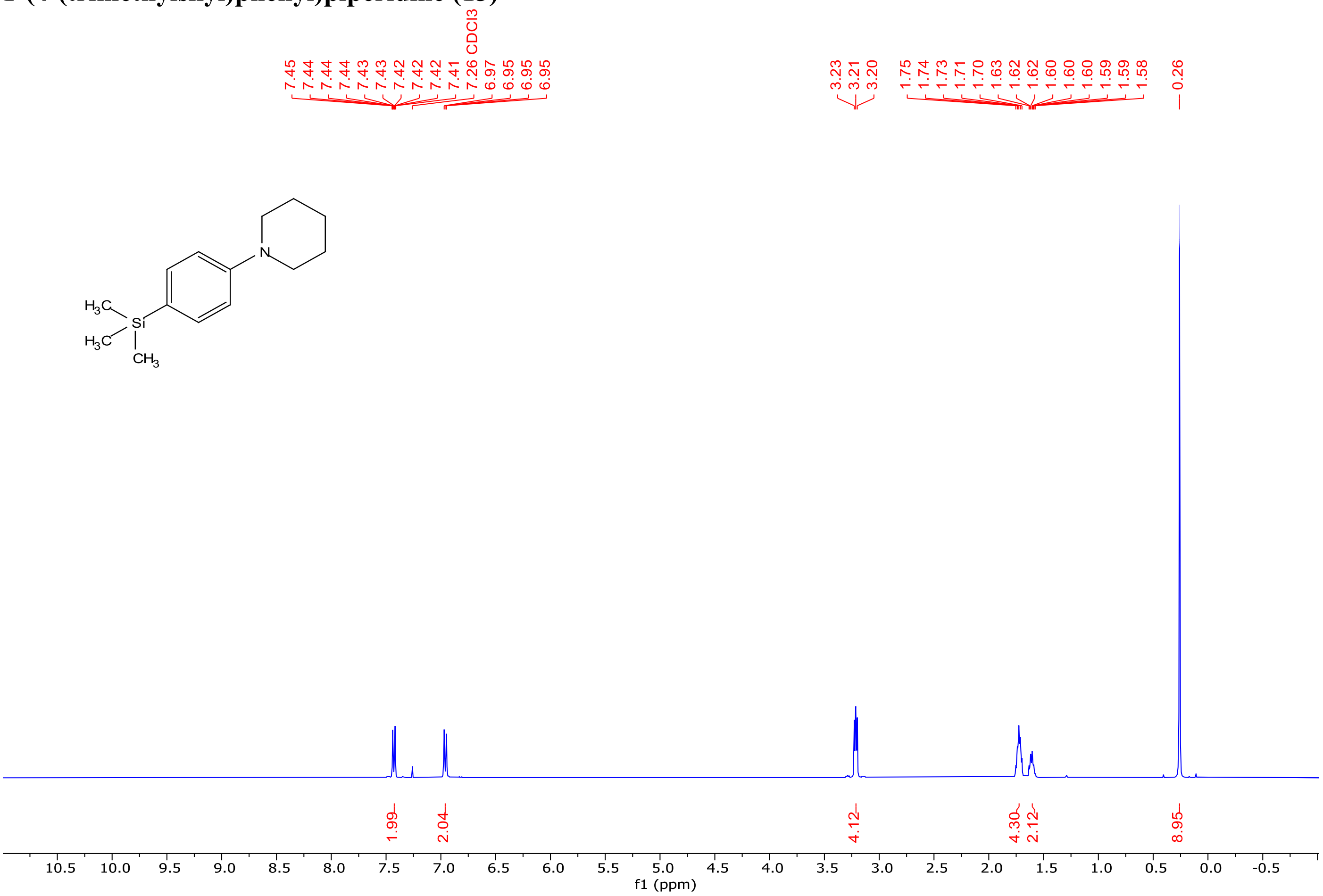

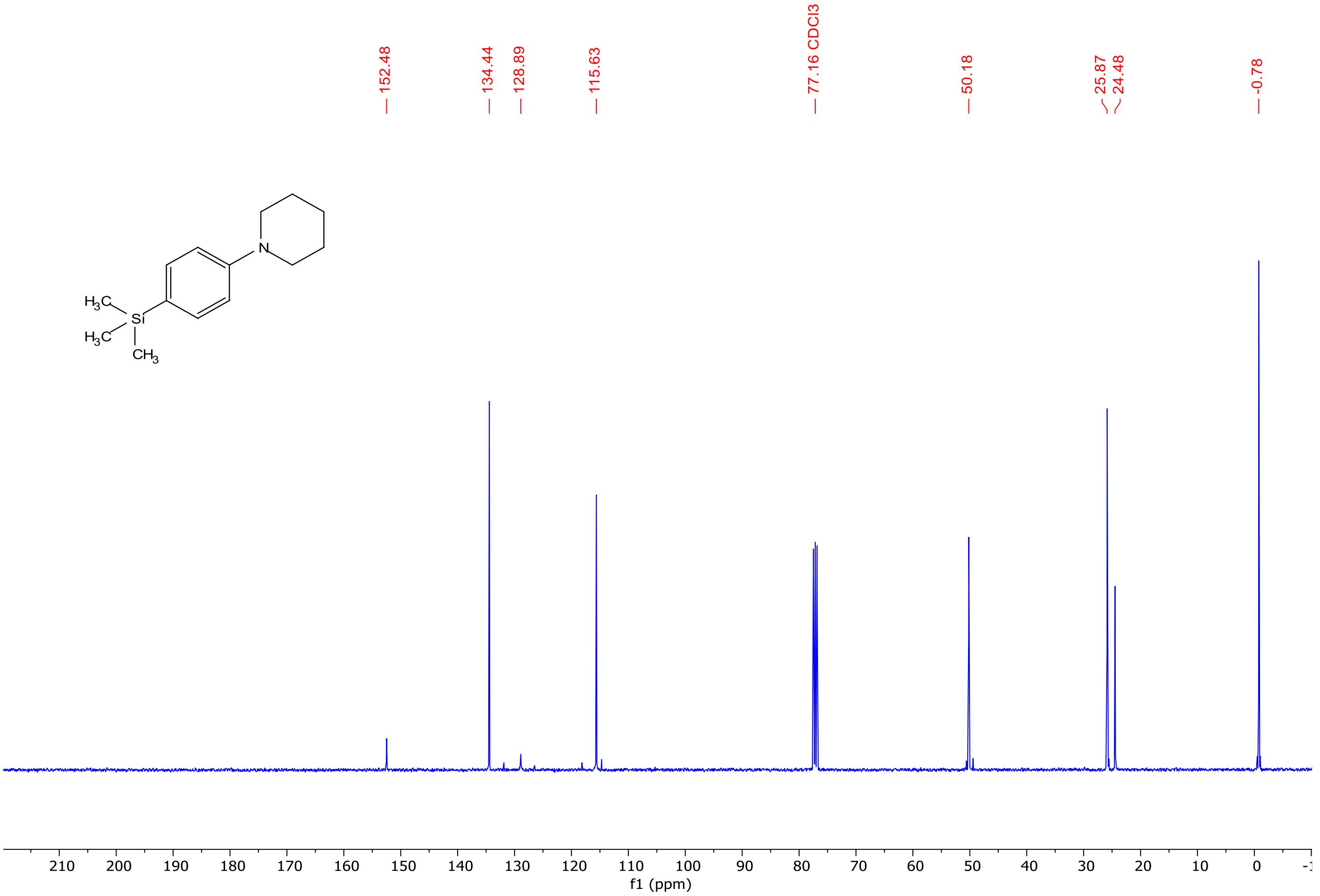

1-(4-chlorophenyl)piperidine (14)

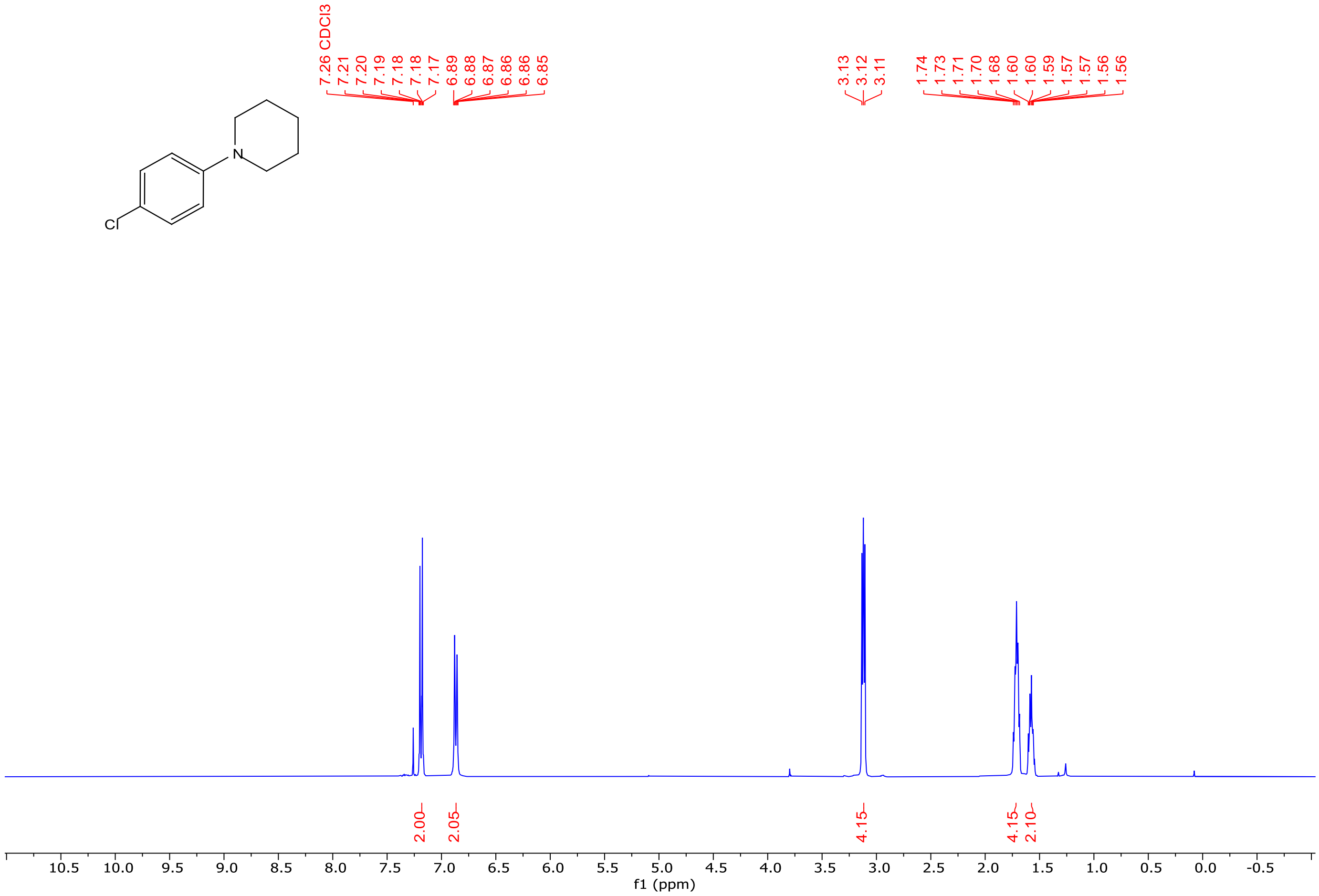

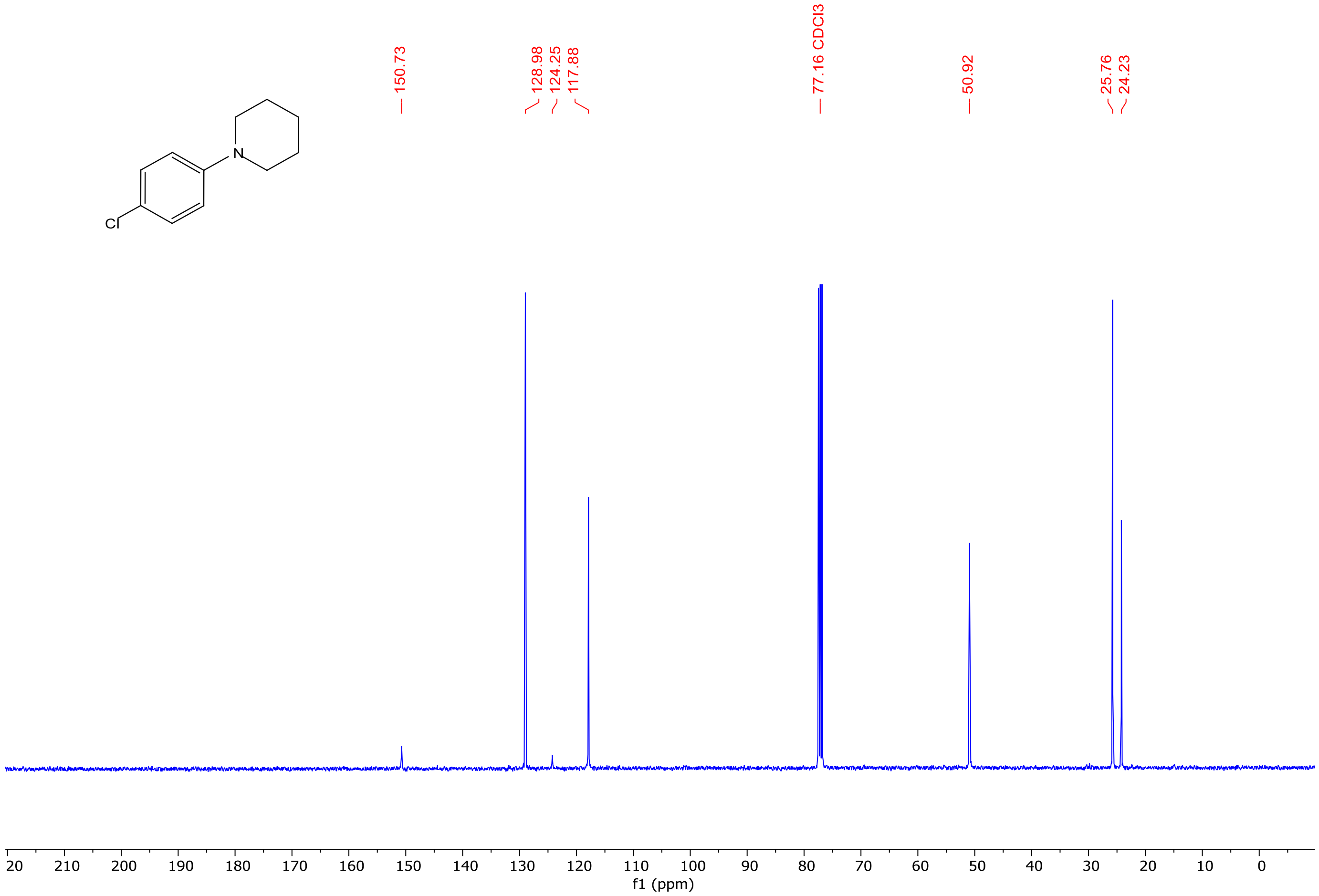

Methyl 4-(piperidin-1-yl)benzoate (15)

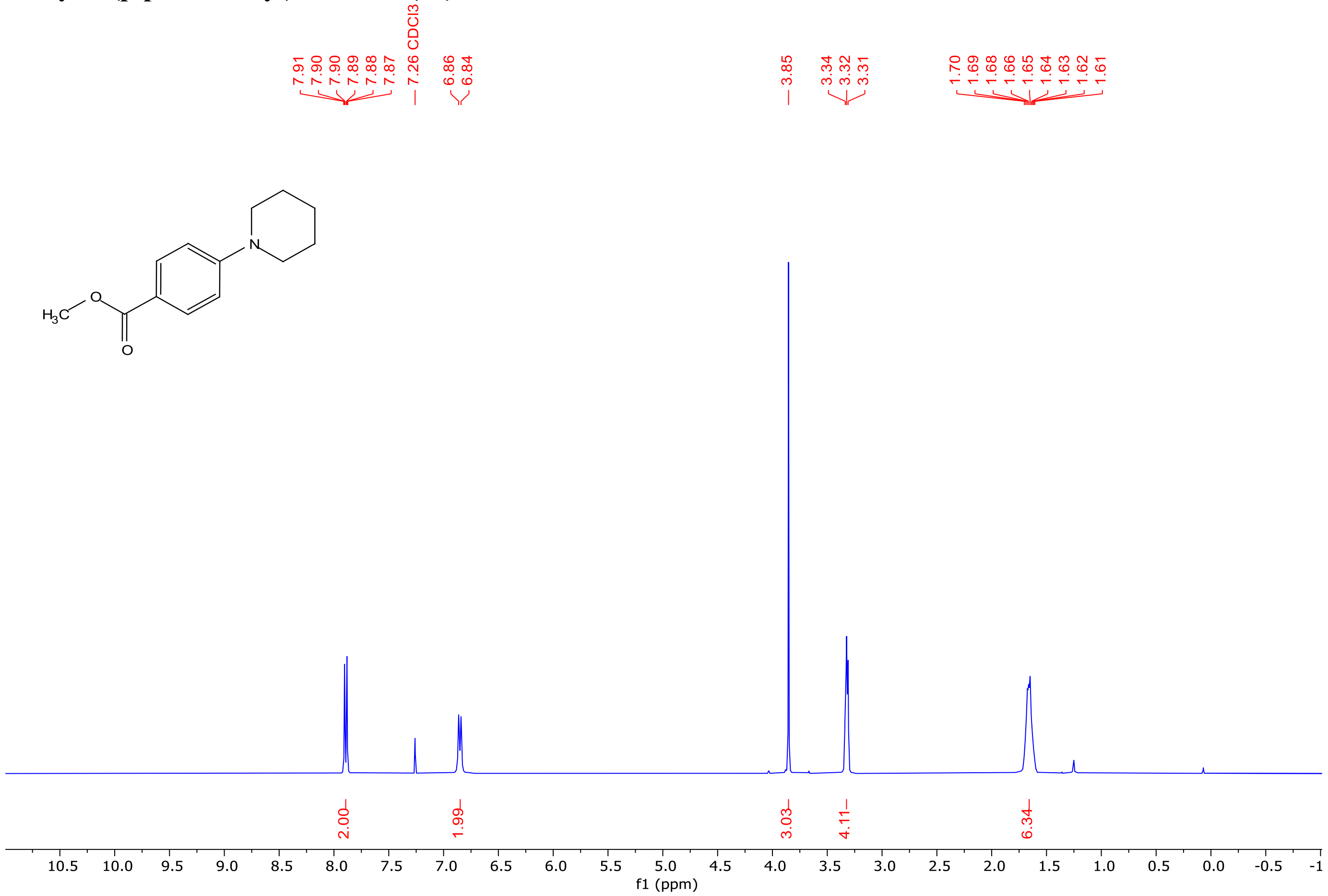

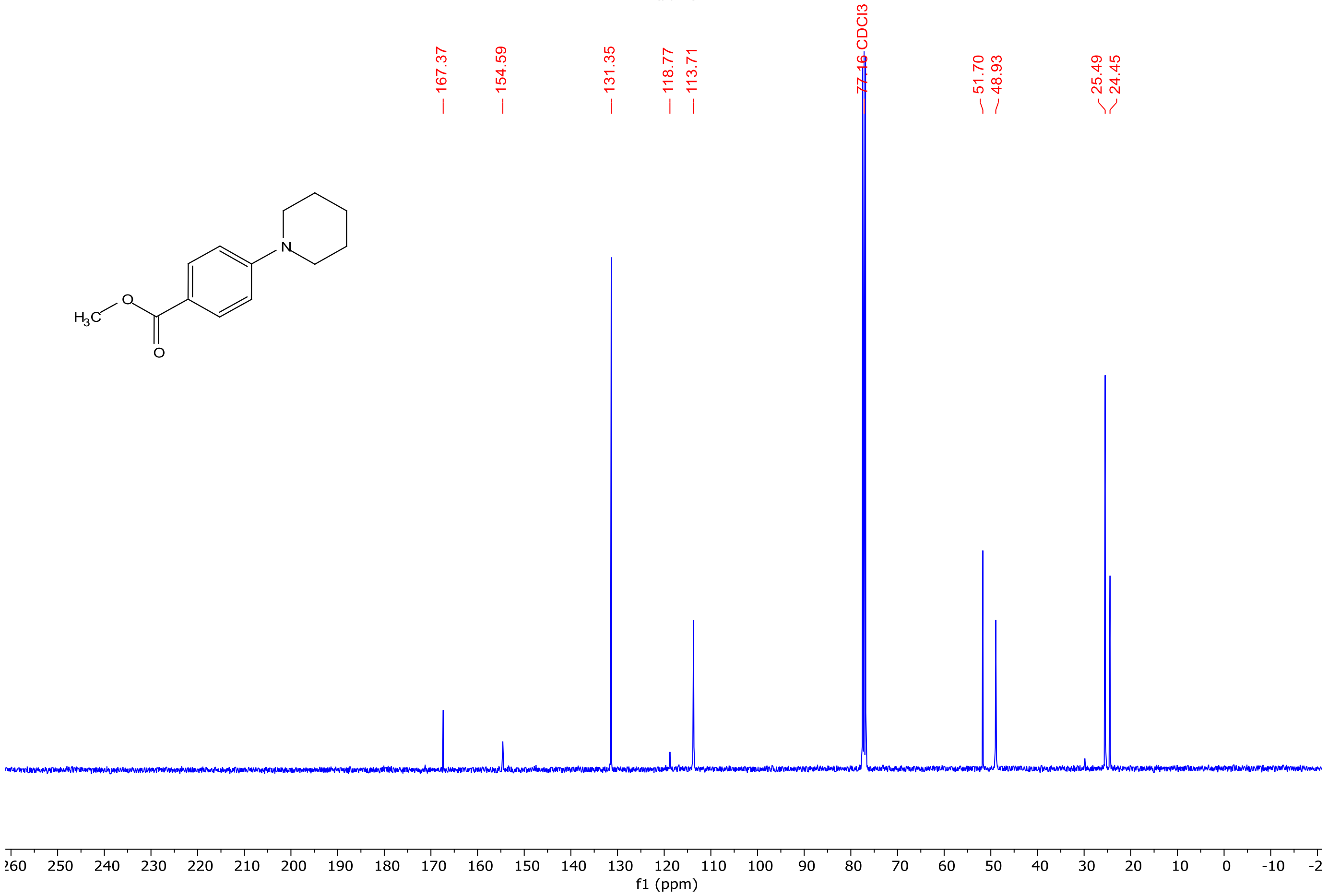

1-(4-(piperidin-1-yl)phenyl)ethan-1-one (16)

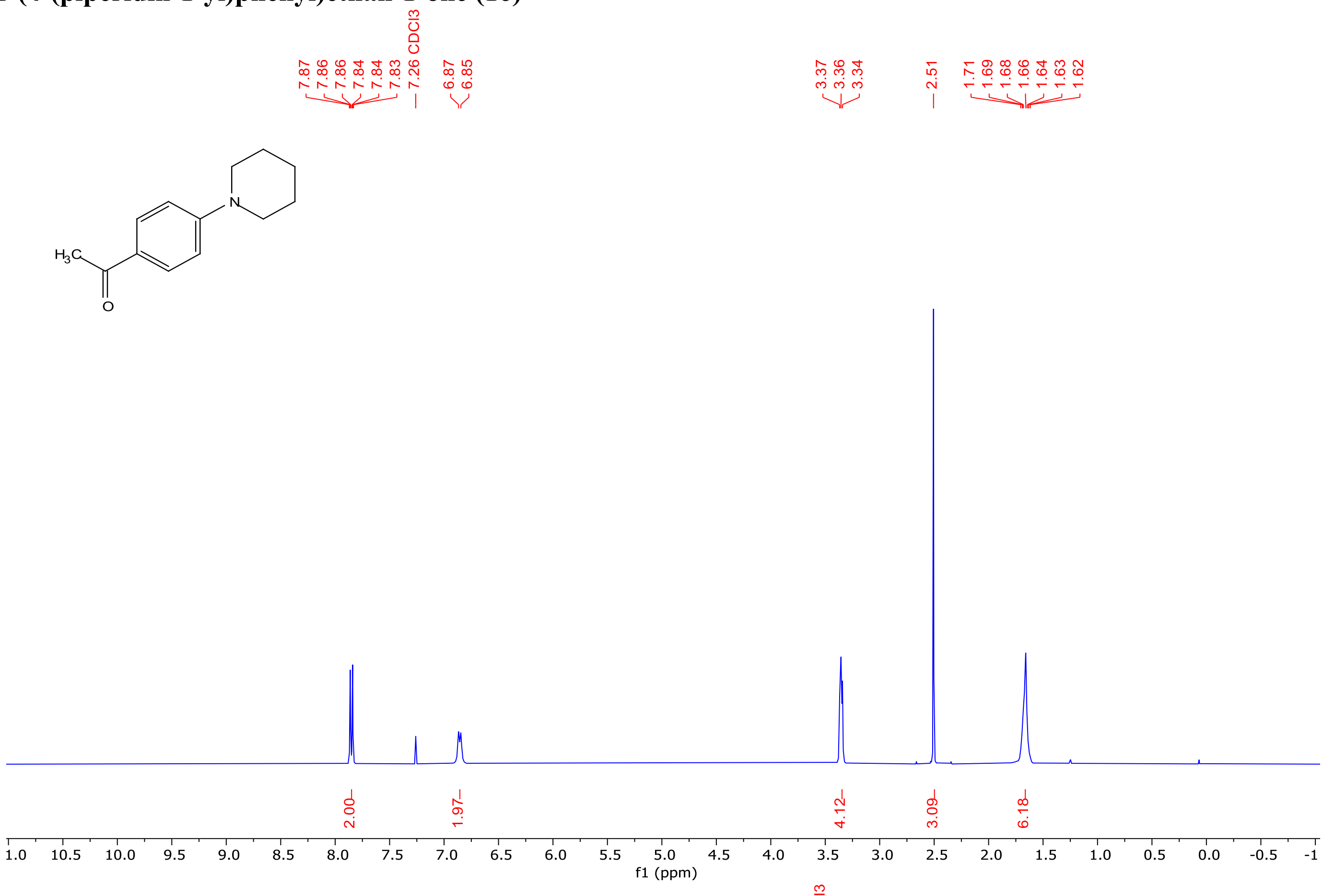

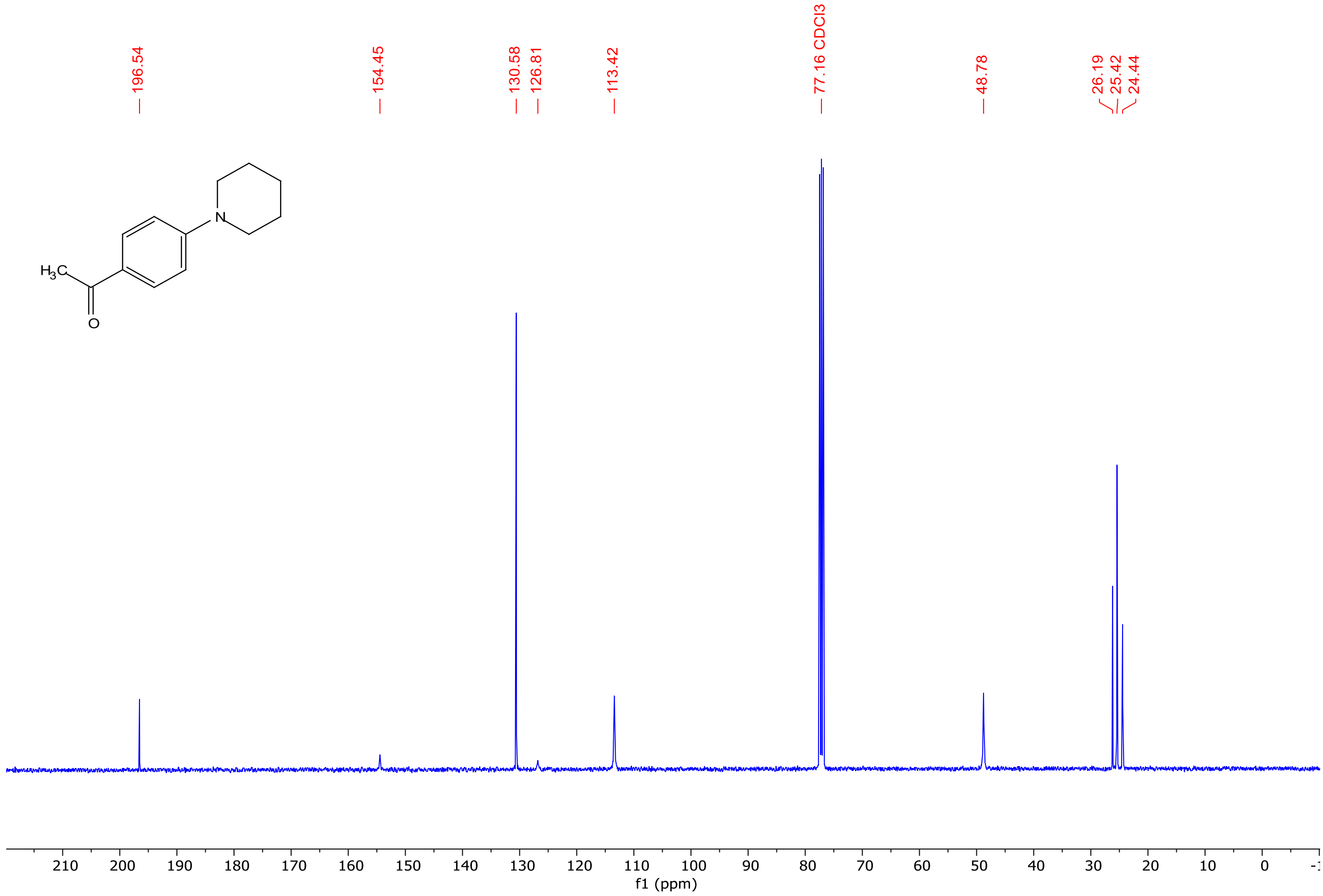

4-(piperidin-1-yl)benzaldehyde (17)

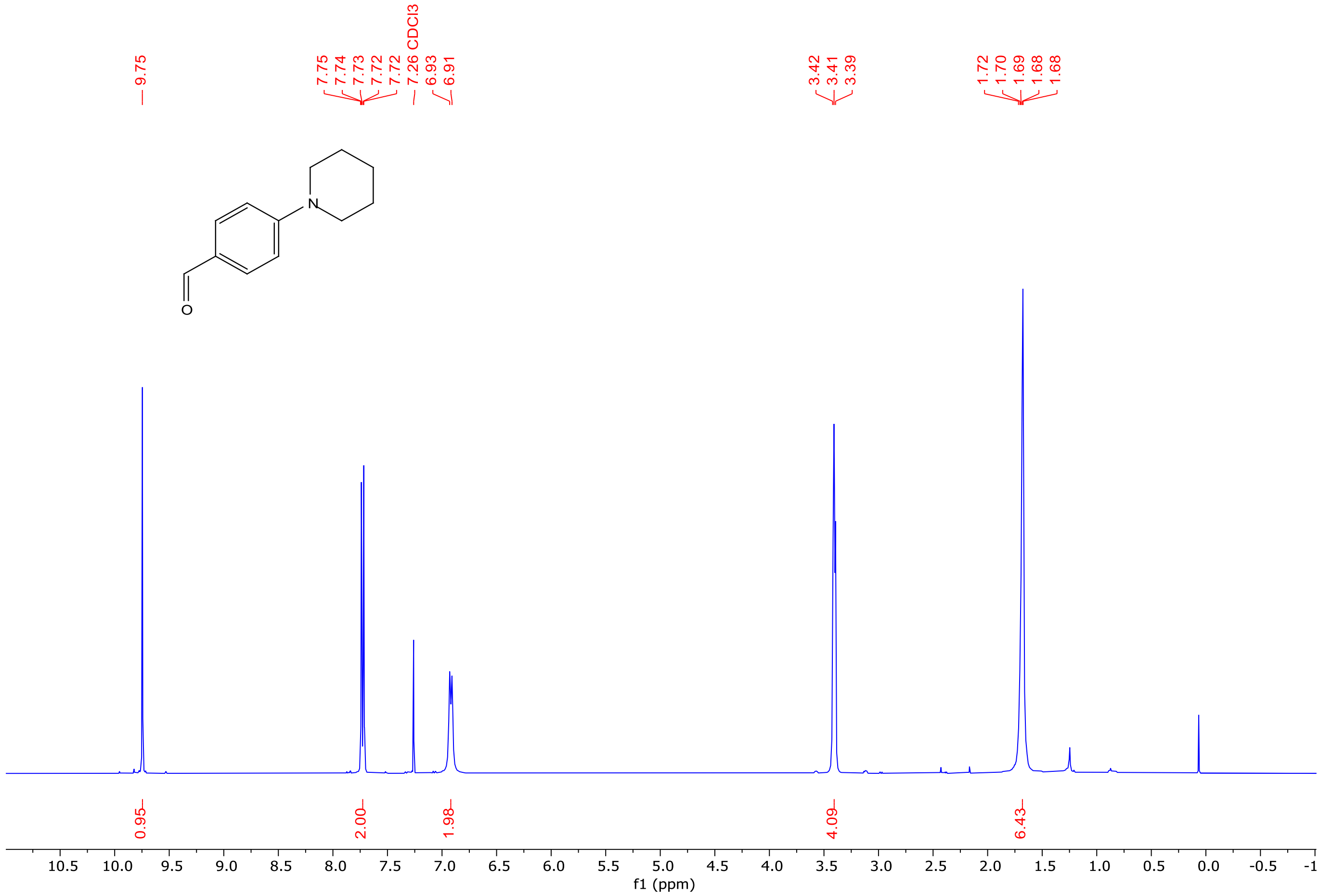

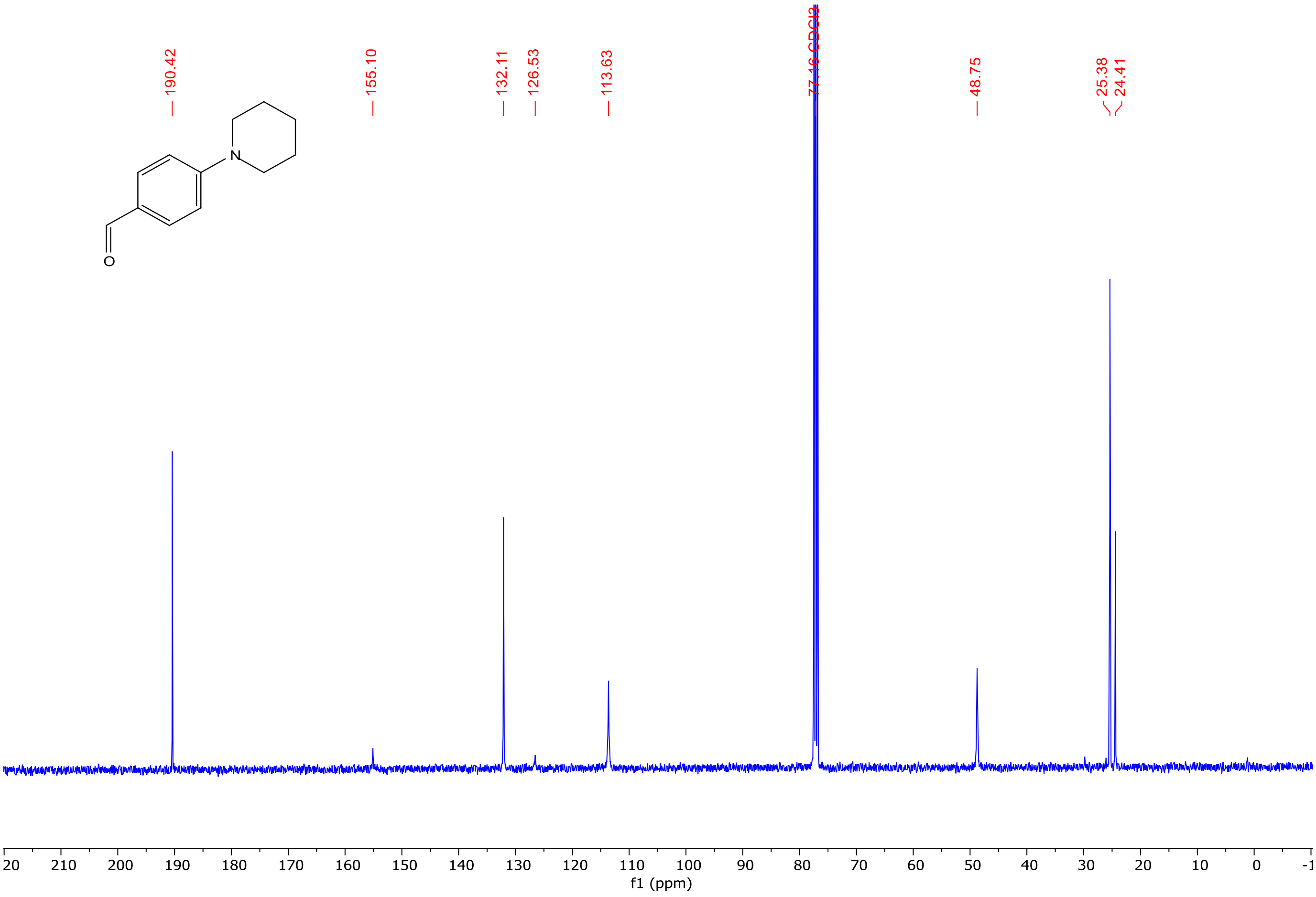

1-(4-(trifluoromethyl)phenyl)piperidine (18)

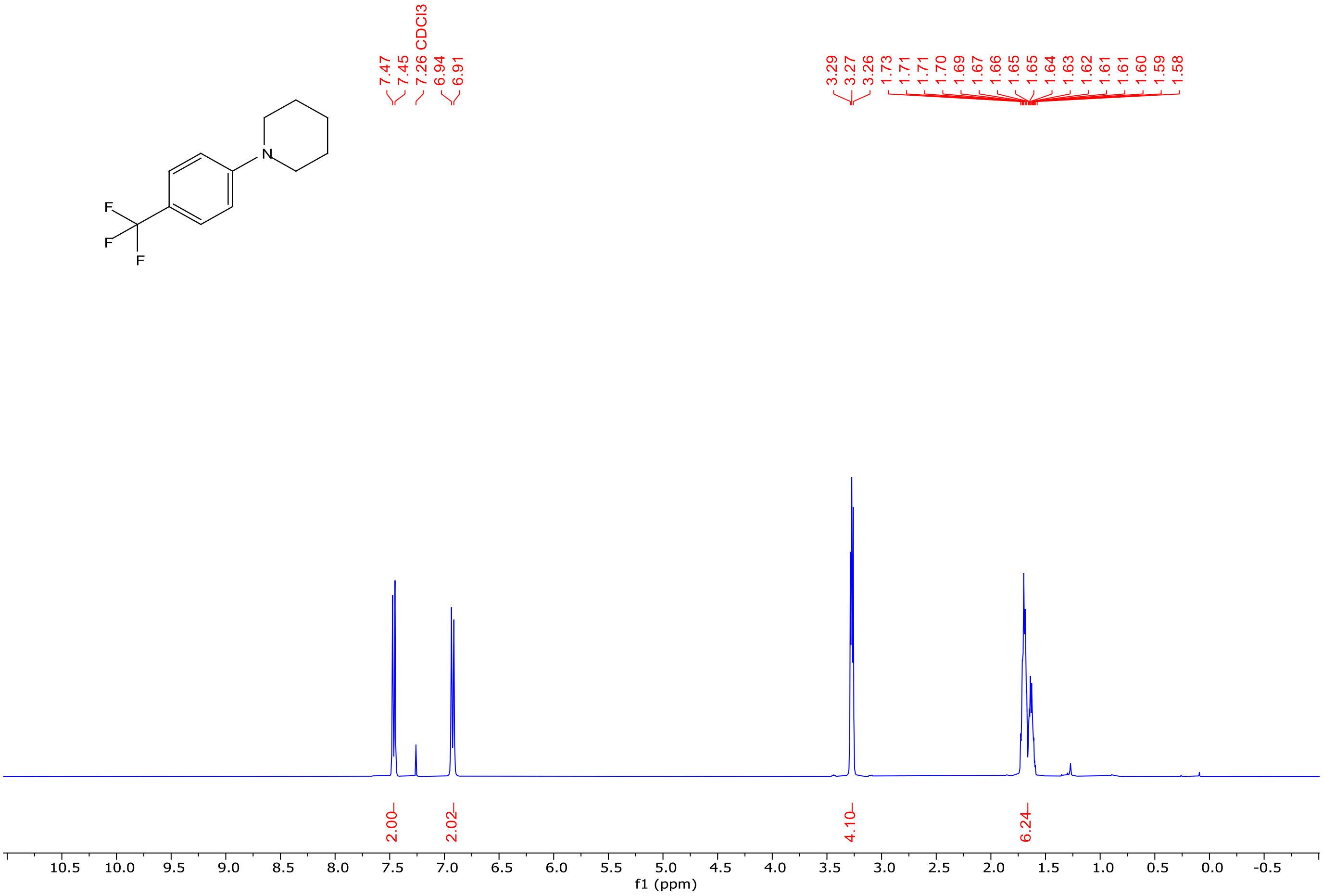

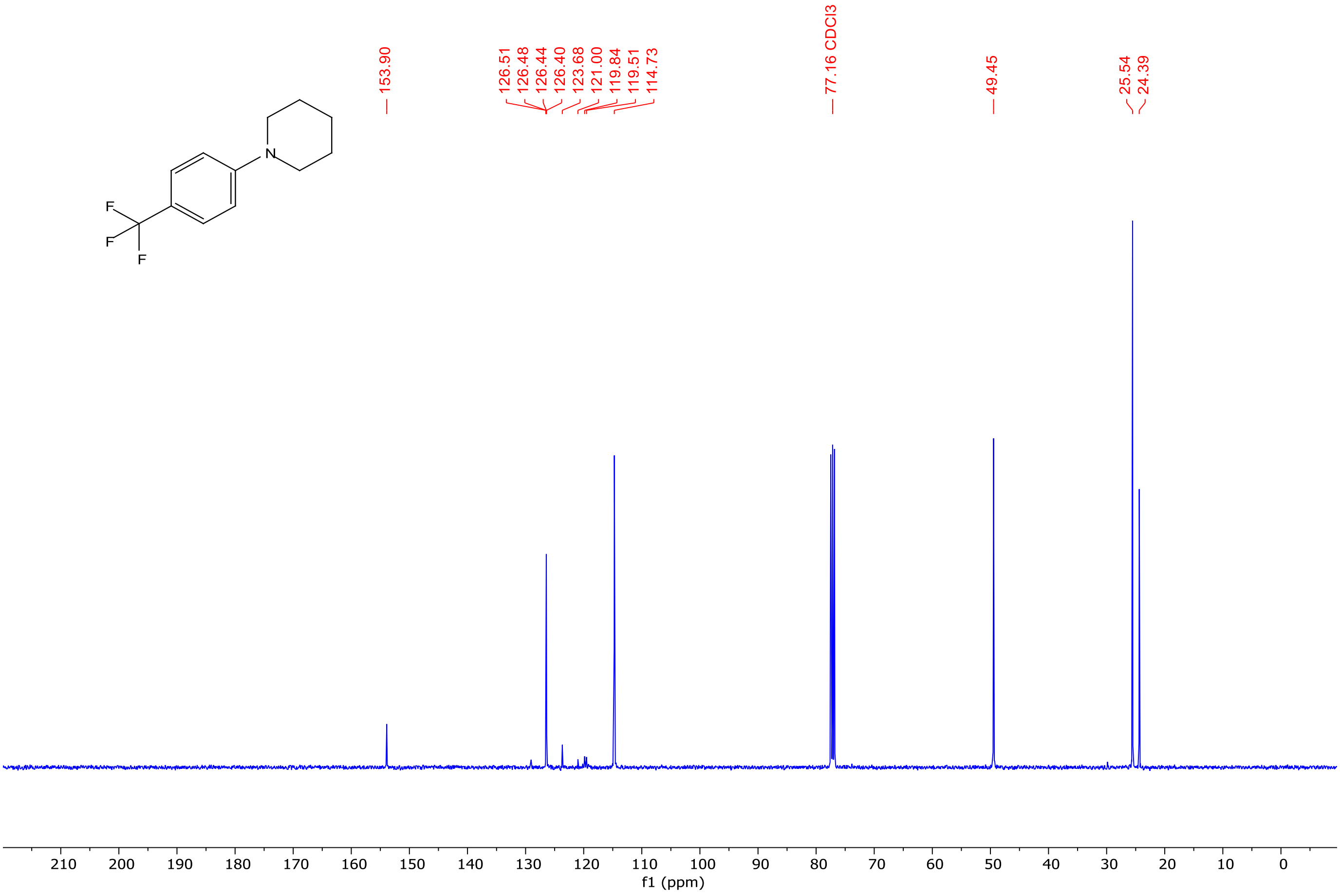

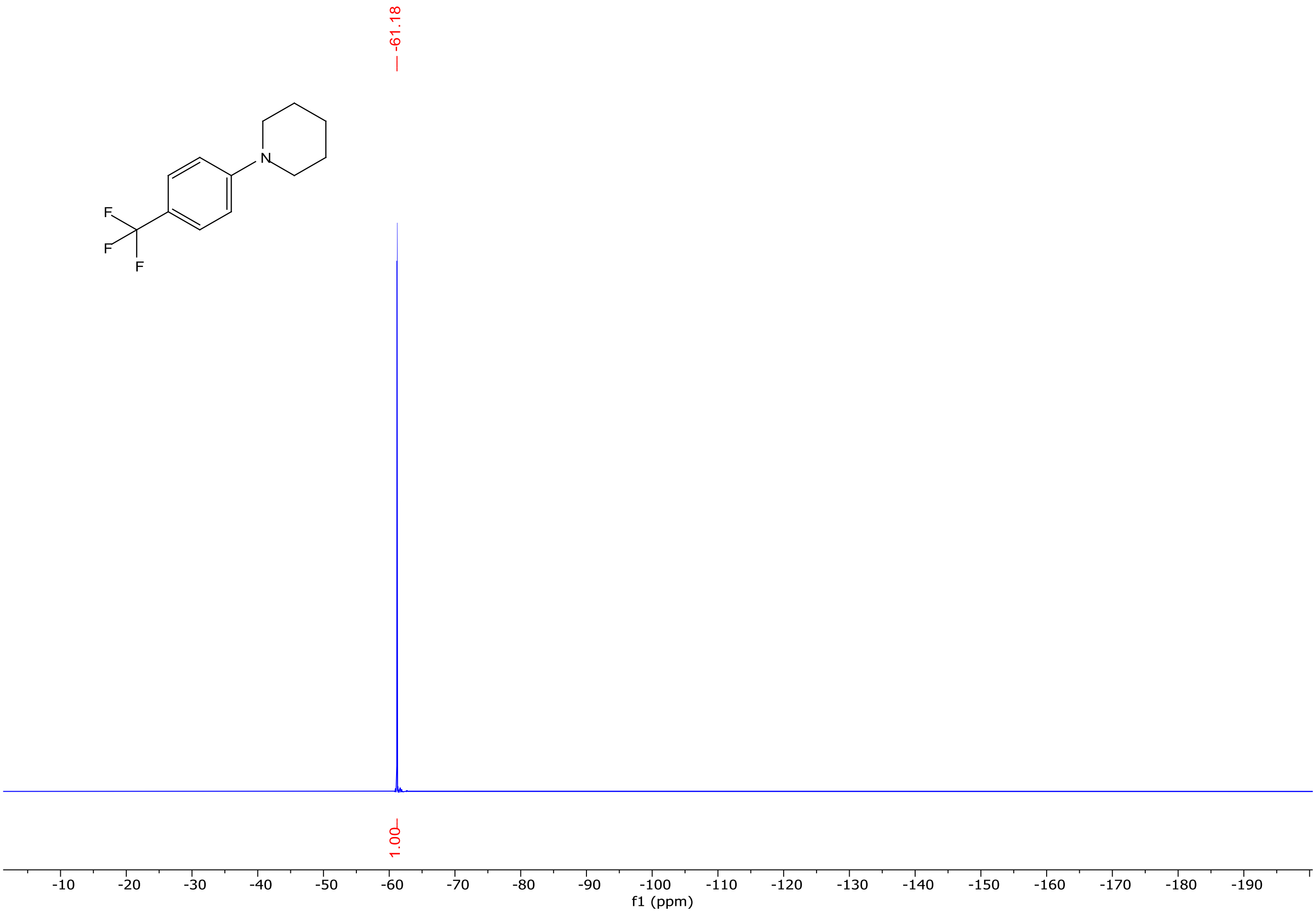
-61.18
1.00
-10
-20
-30
-40
-50
-60
-70
-80
-90
-100
-110
-120
-130
-140
-150
-160
-170
-180
-190
f1 (ppm)
F
F
F
N

4-(piperidin-1-yl)benzenesulfonamide (19)

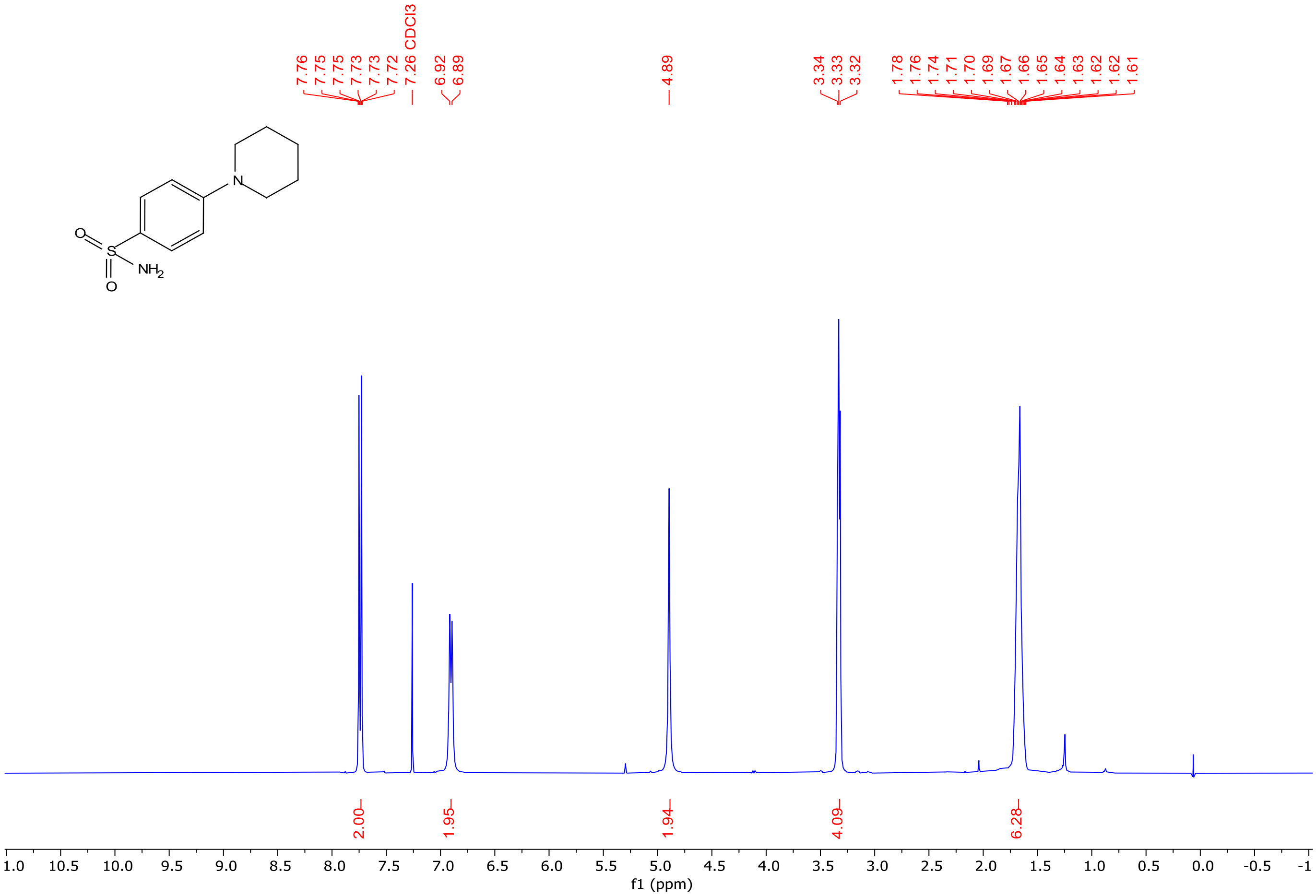

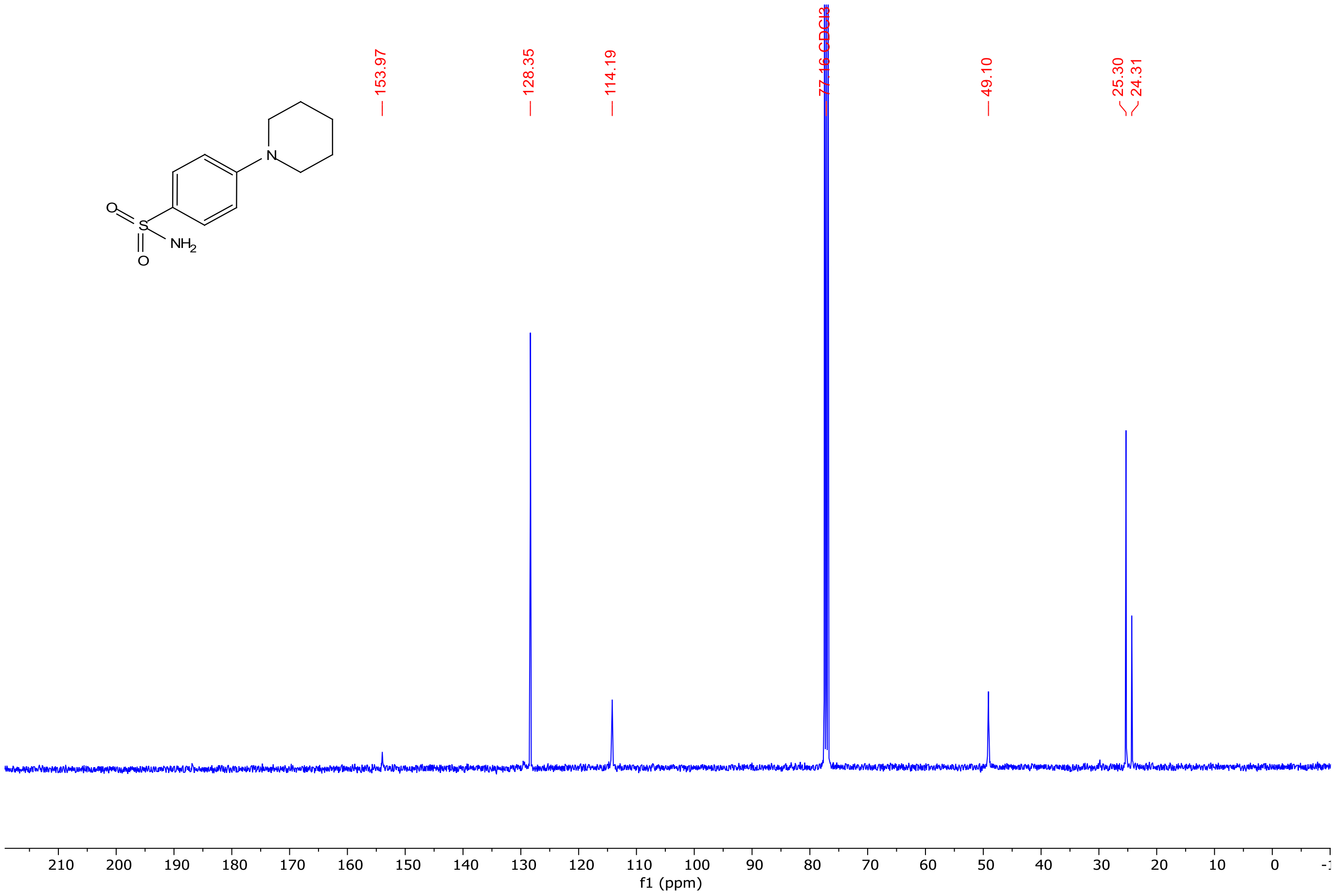

1-(4-(methylsulfonyl)phenyl)piperidine (20)

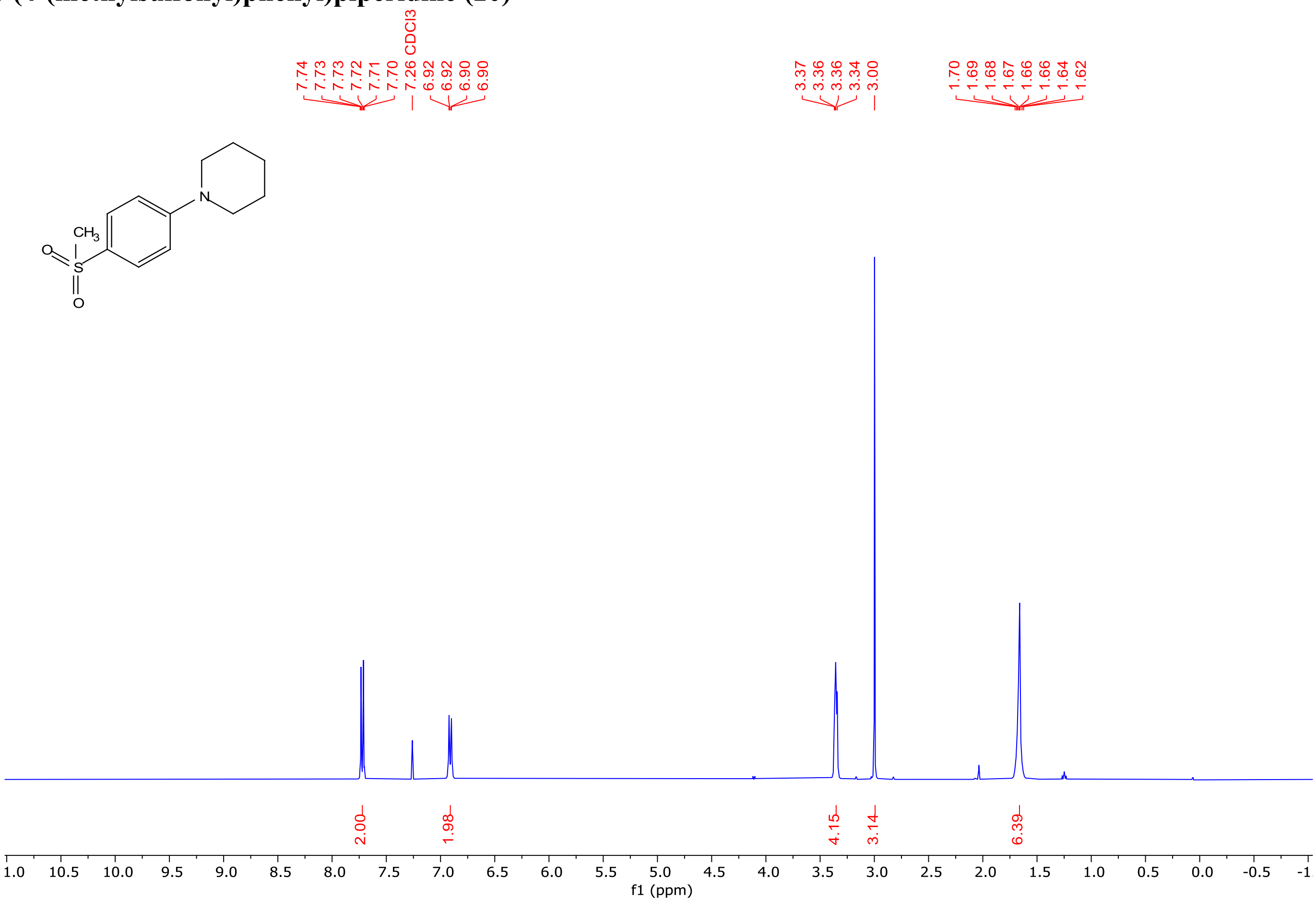

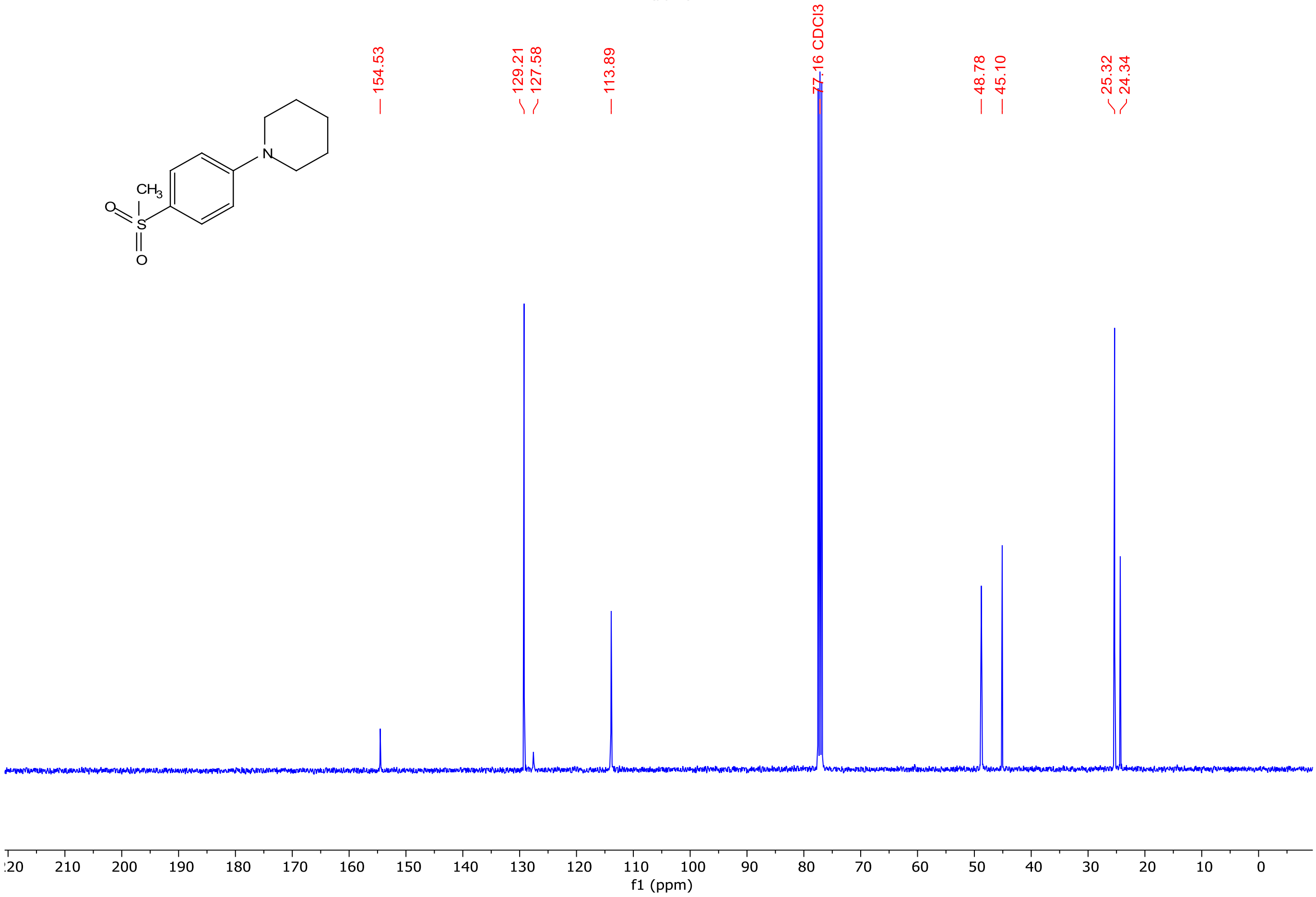

1-(4-(4,4,5,5-tetramethyl-1,3,2-dioxaborolan-2-yl)phenyl)piperidine (21)

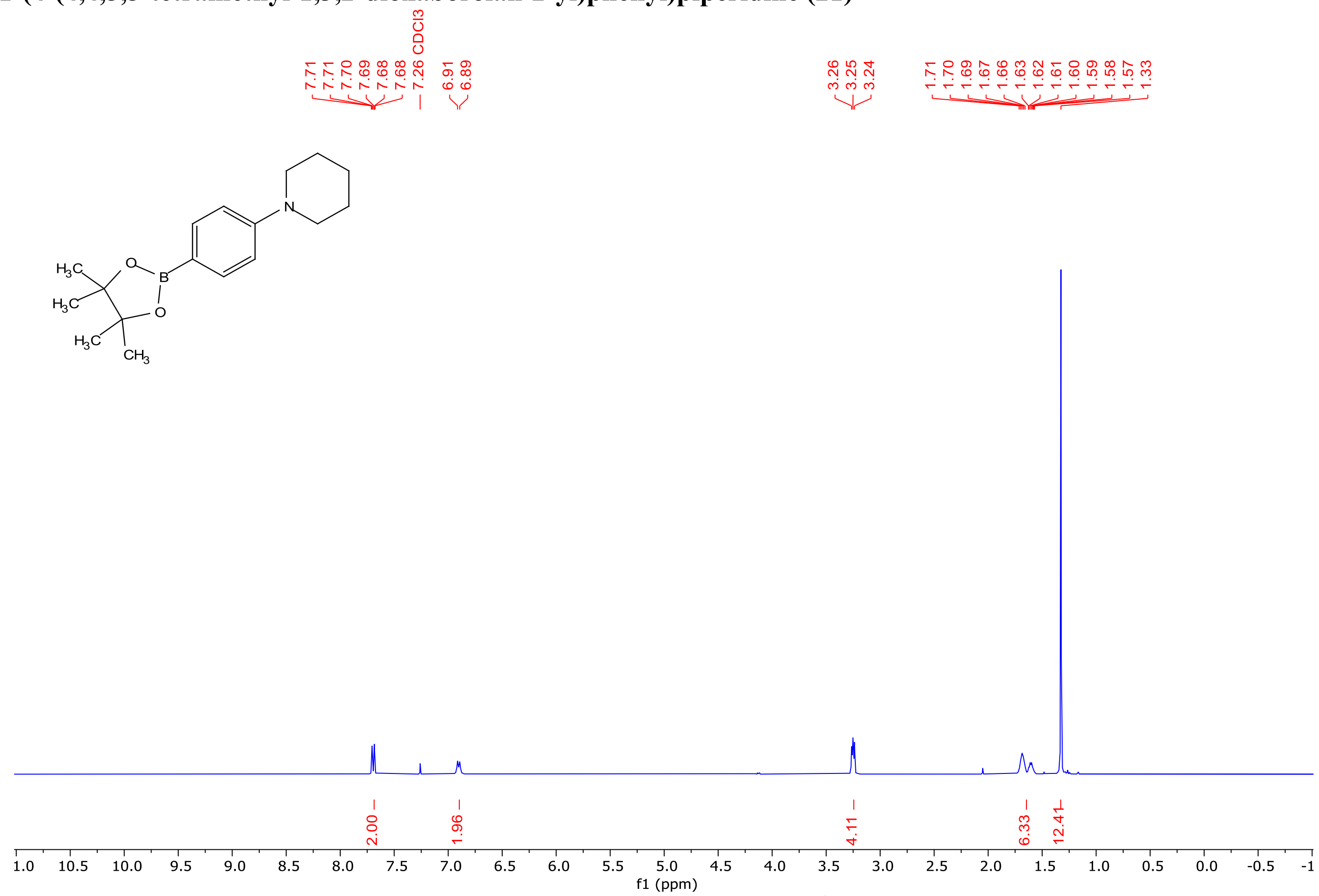

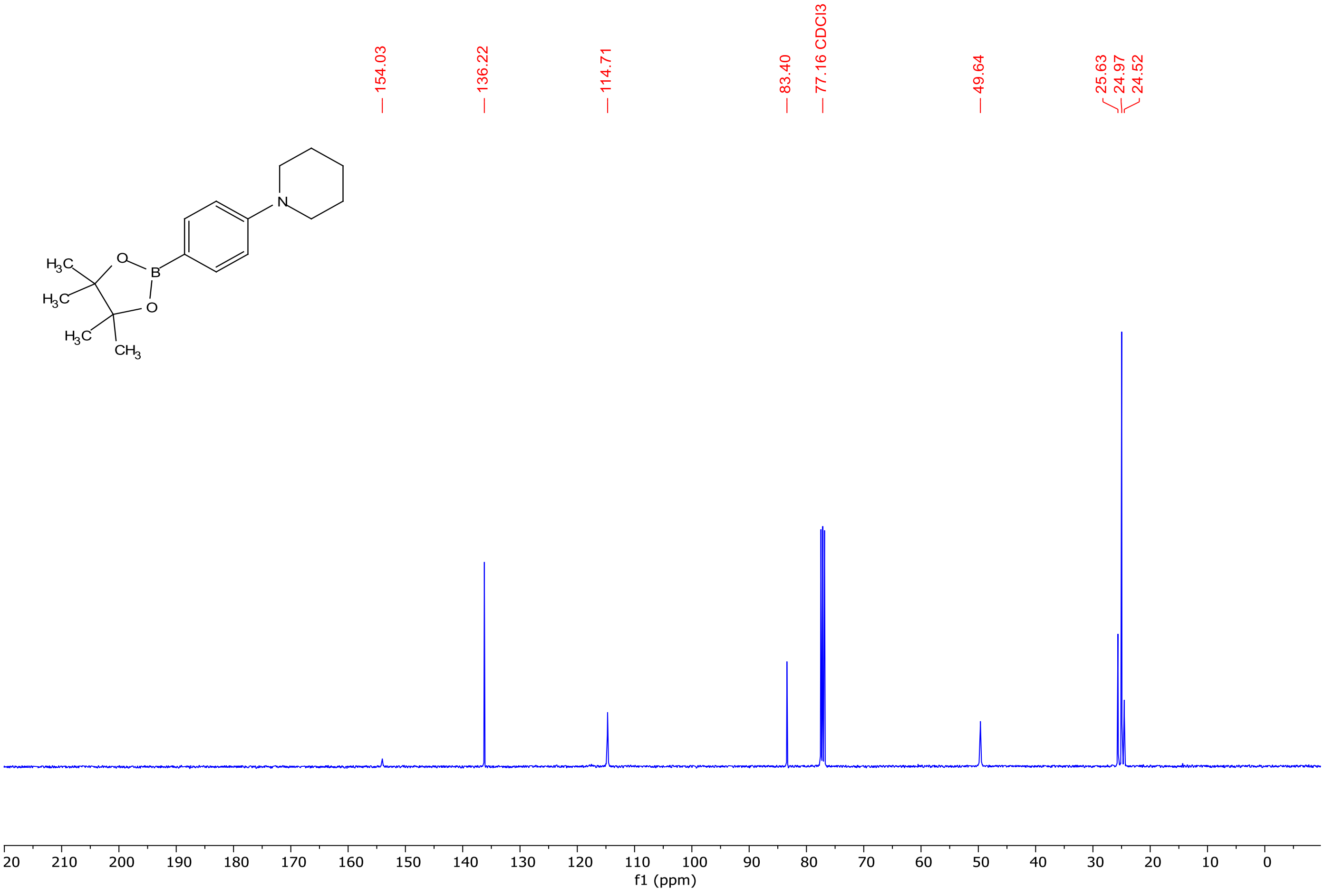

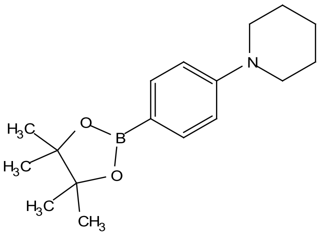
H3C
H3C
H3C
CH3
O
B
O
N

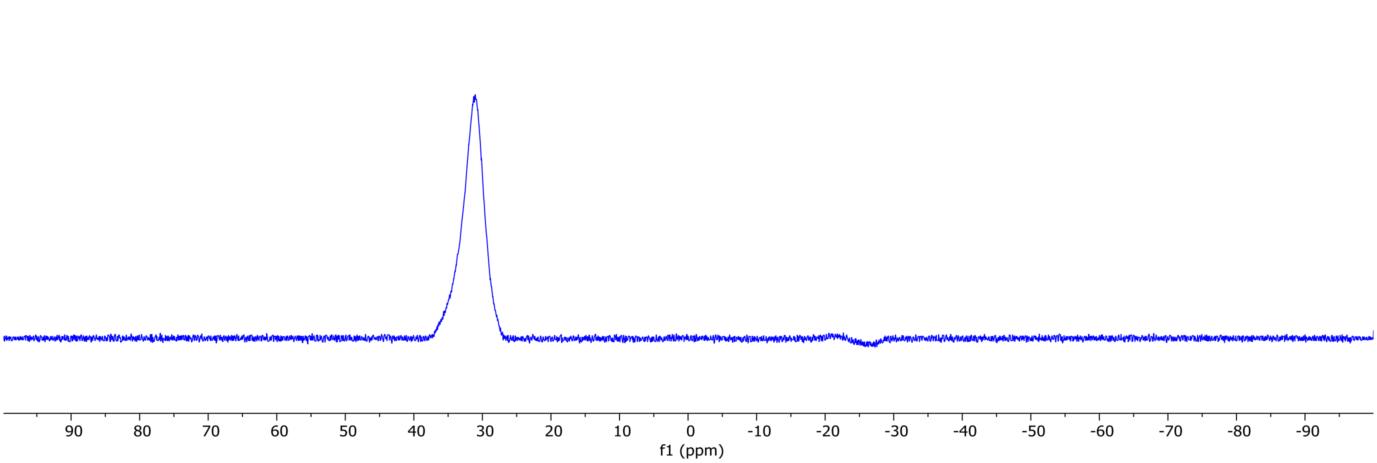
30.88
90
80
70
60
50
40
30
20
10
0
-10
-20
-30
-40
-50
-60
-70
-80
-90
f1 (ppm)

5-(piperidin-1-yl)picolinonitrile (22)

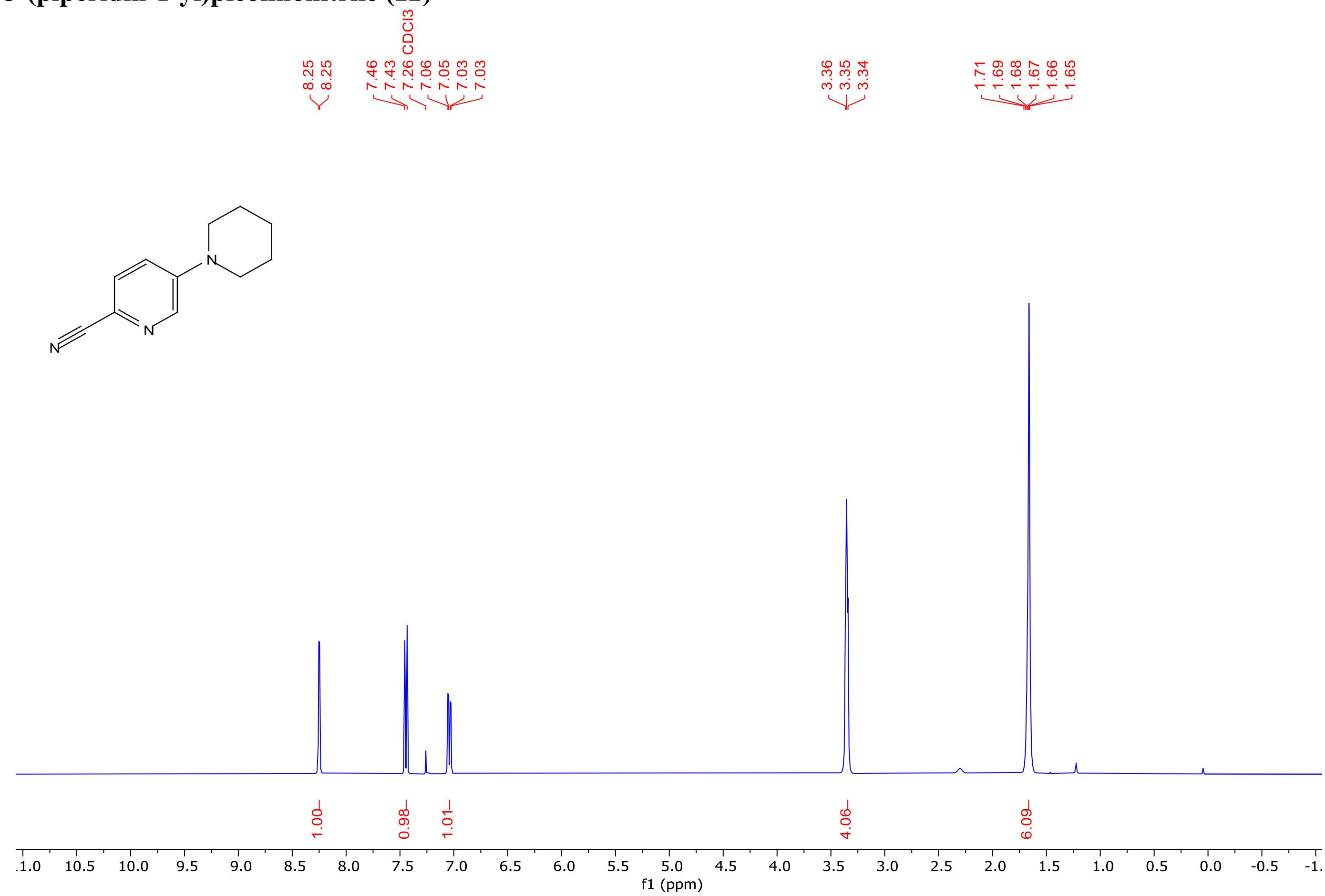

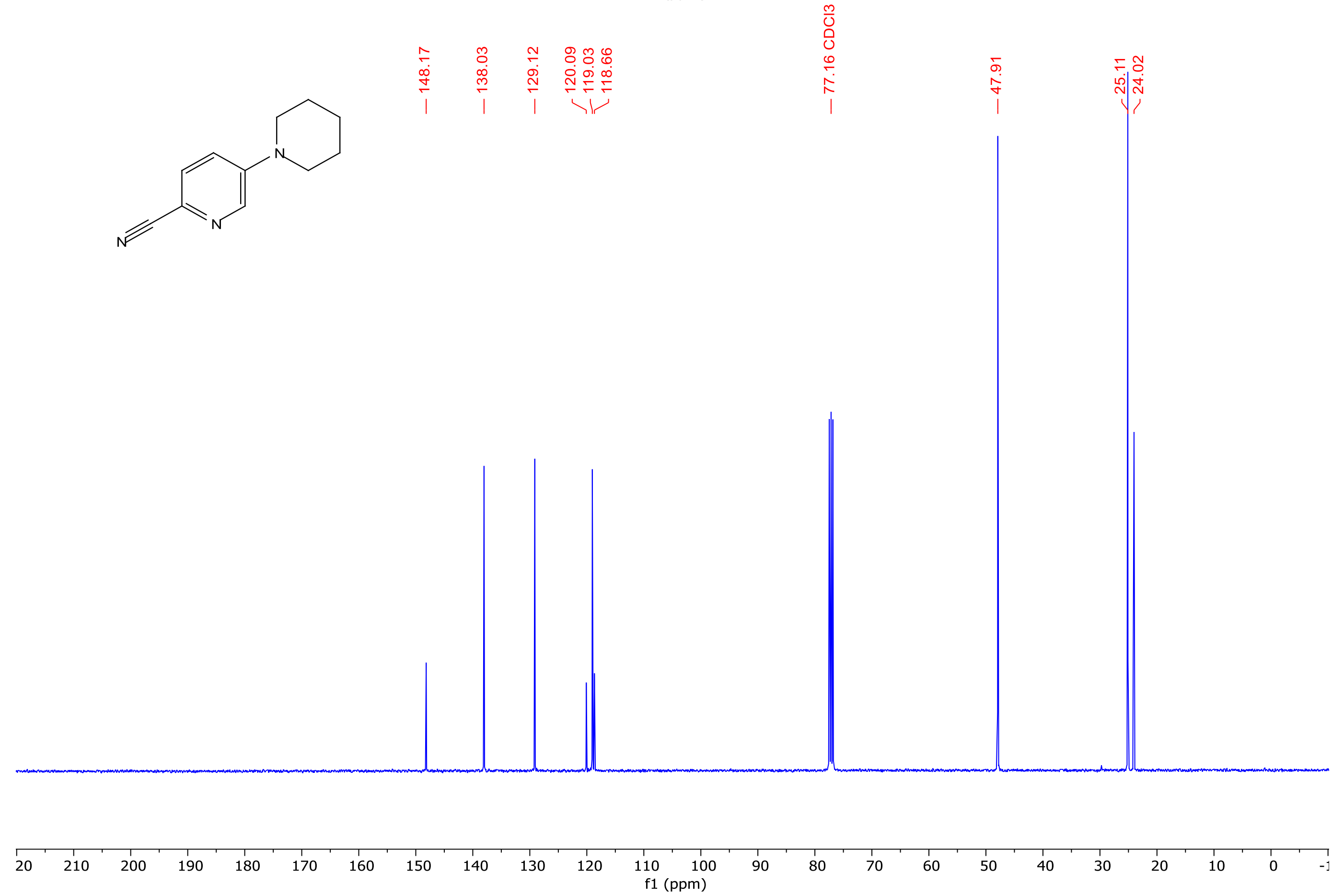

4-(piperidin-1-yl)benzonitrile (23/24/25)

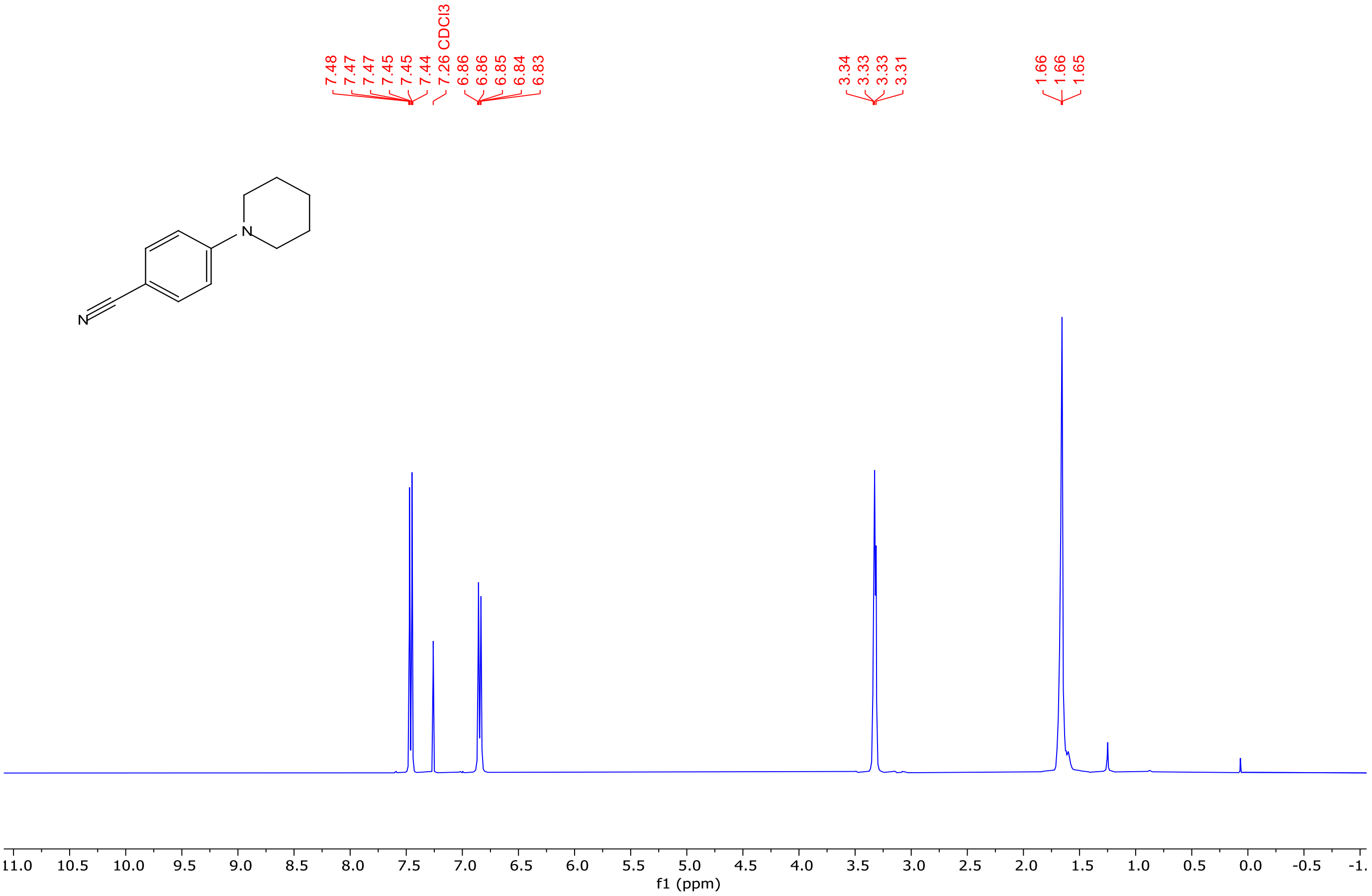

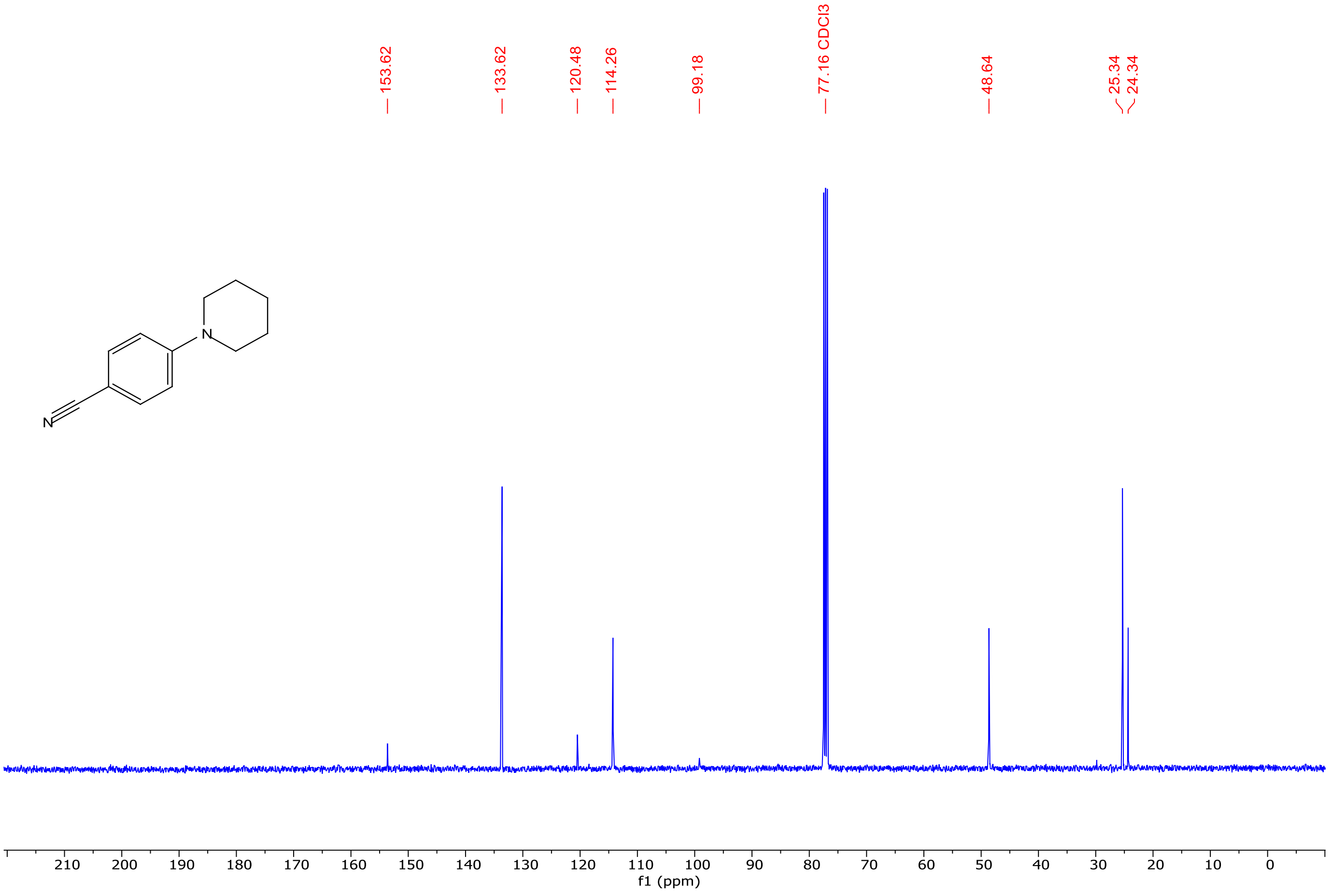

1-(3,5-dimethoxyphenyl)piperidine (26)

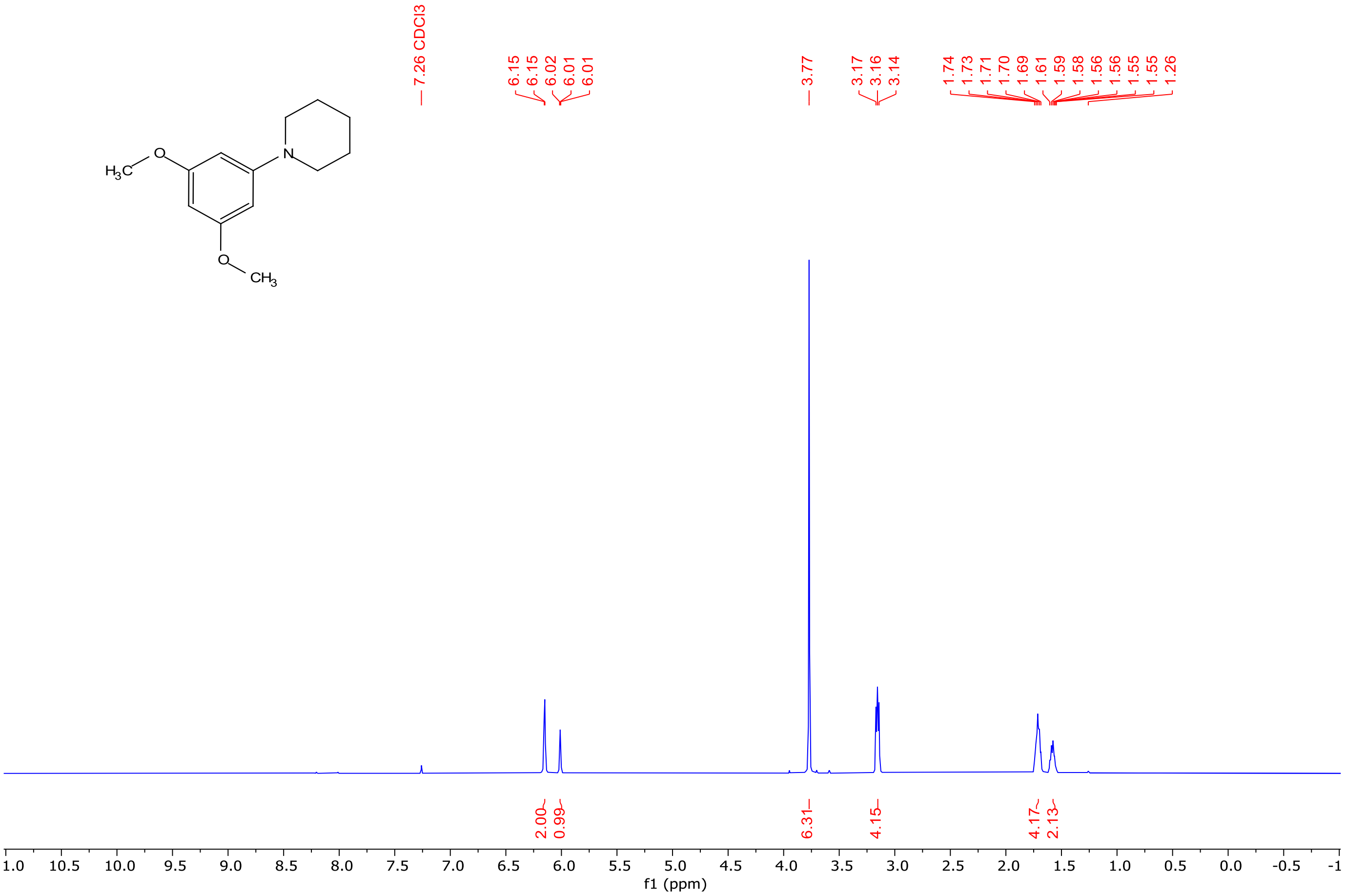

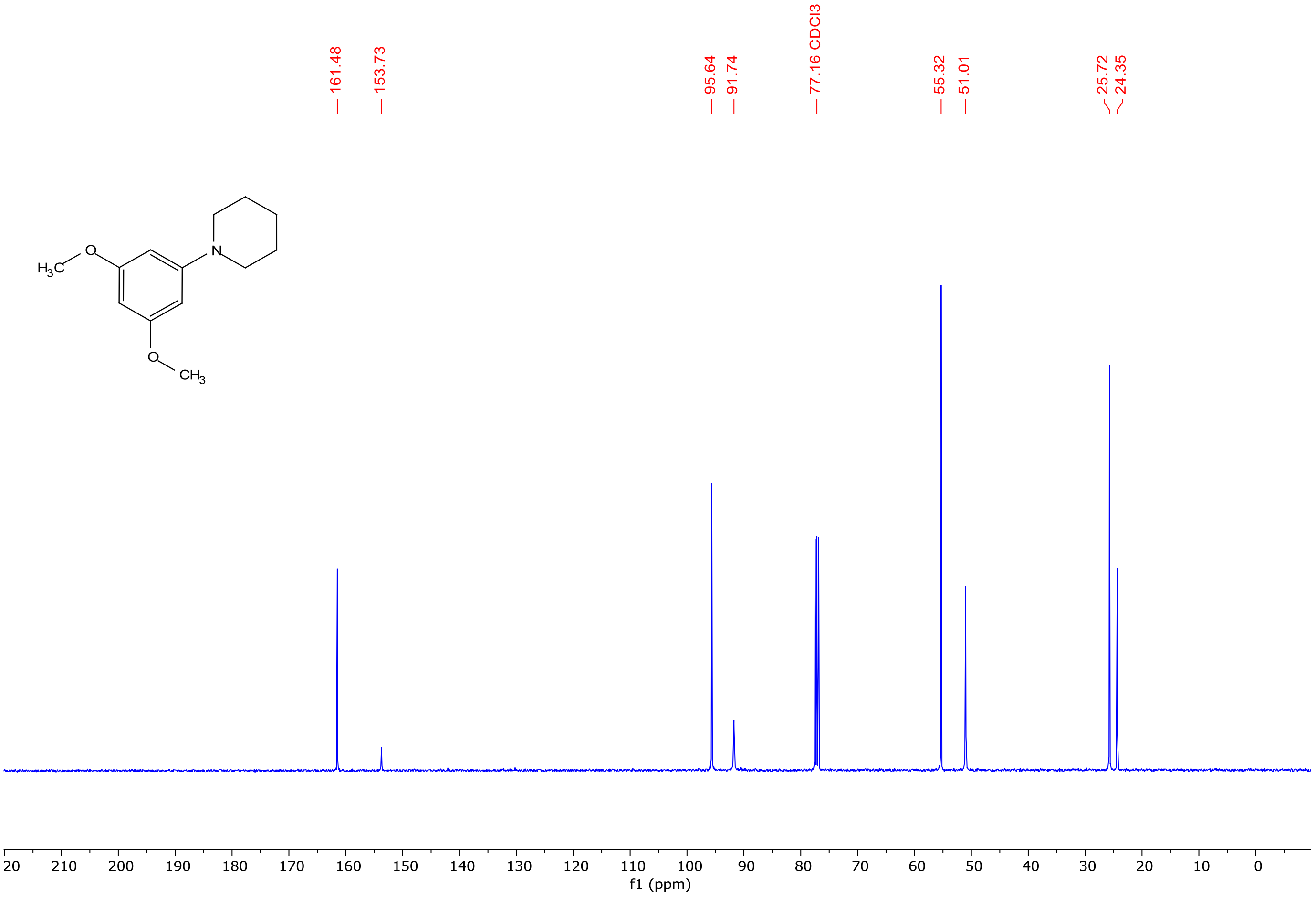

1-(naphthalen-2-yl)piperidine (27)

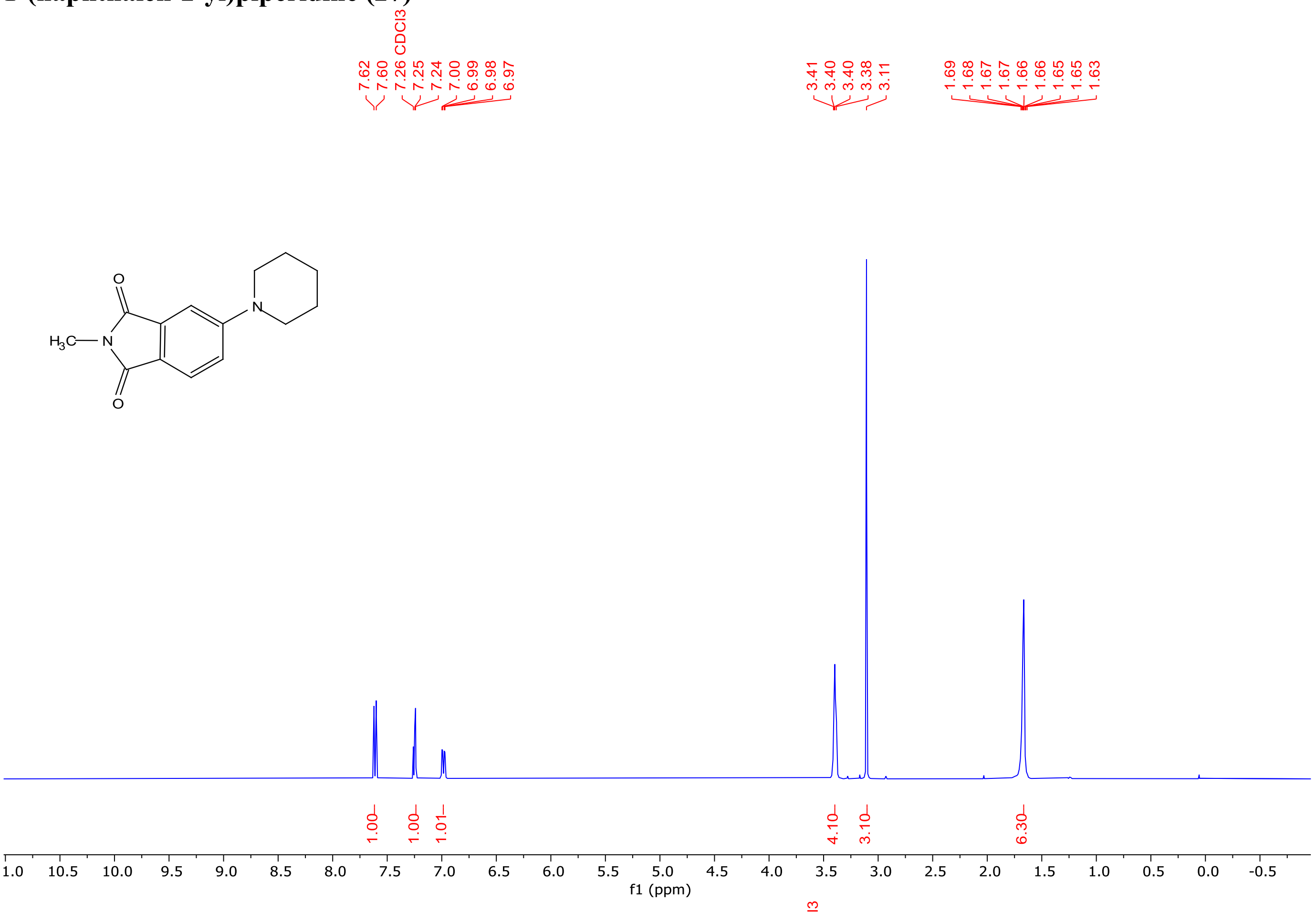

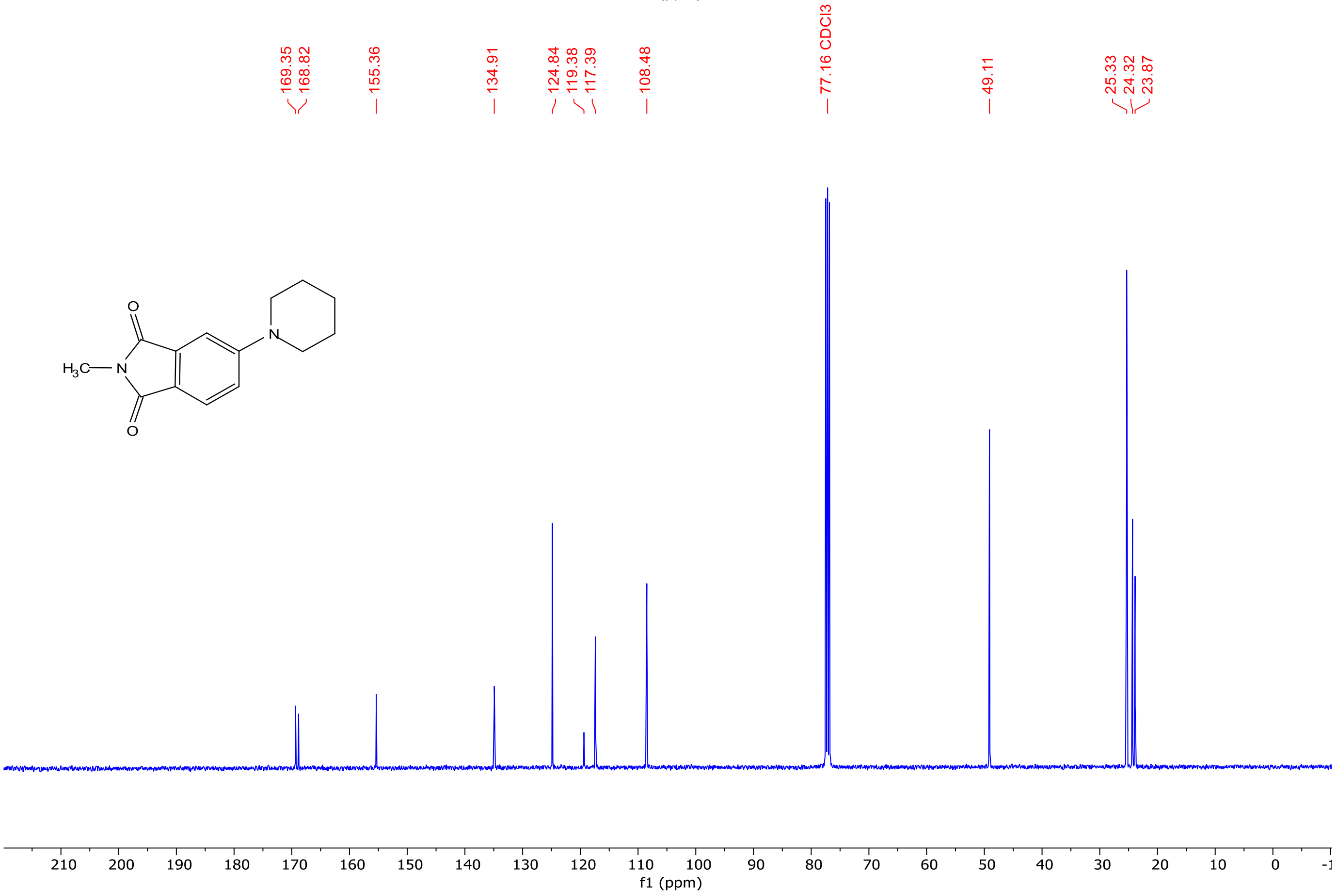

5-(piperidin-1-yl)-2,3-dihydro-1*H*-inden-1-one (28)

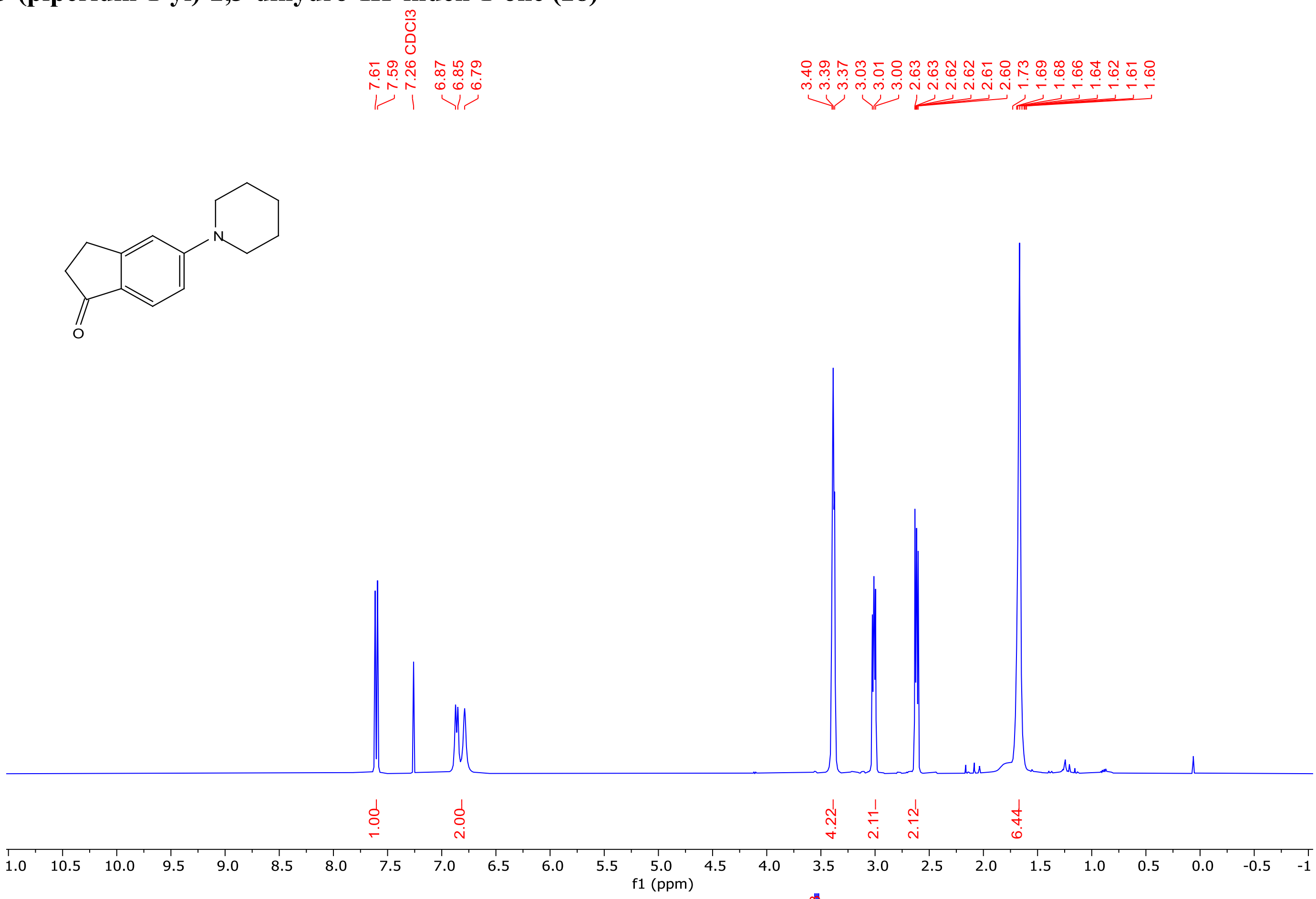

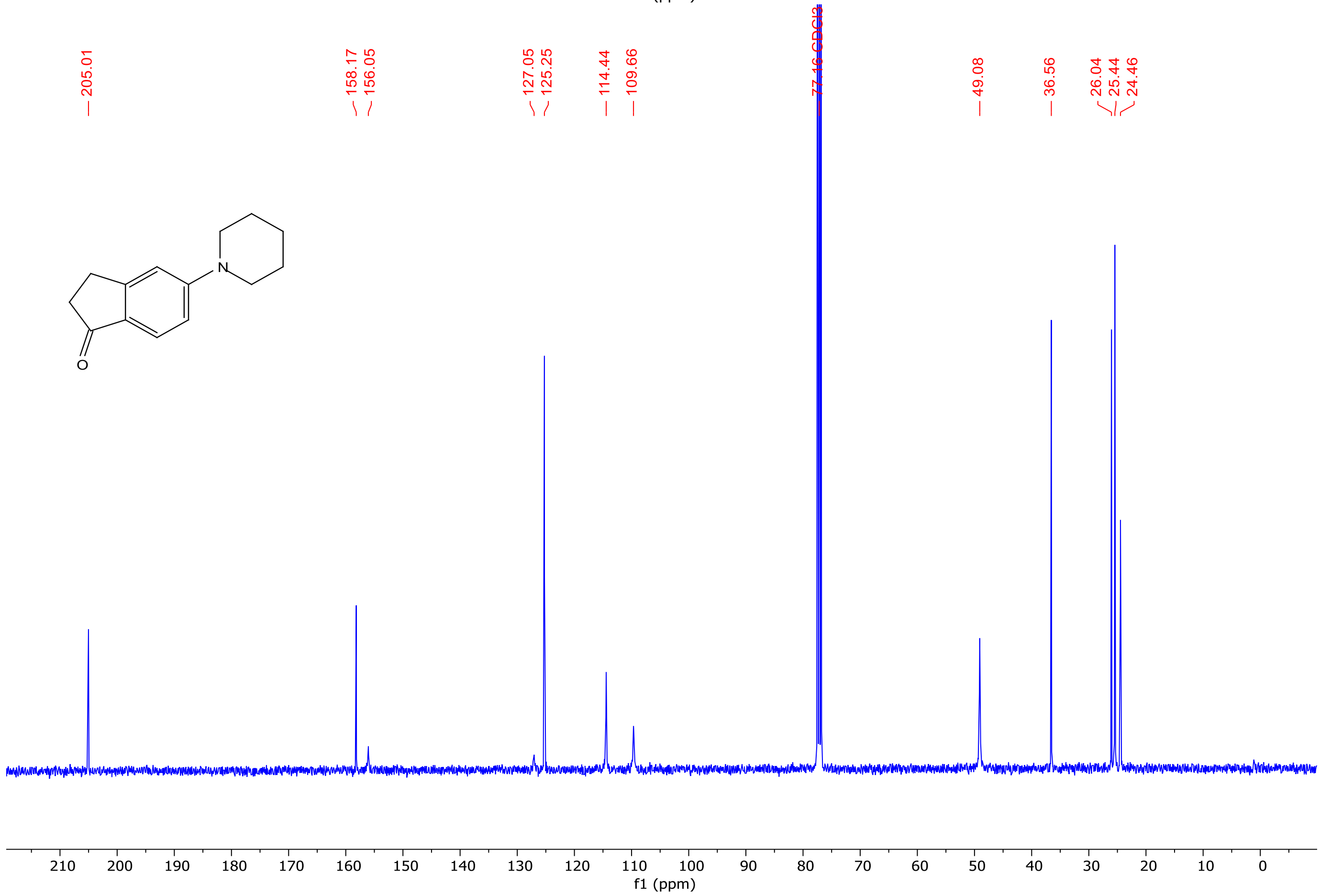

4-(piperidin-1-yl)-2,3-dihydro-1*H*-inden-1-one (29)

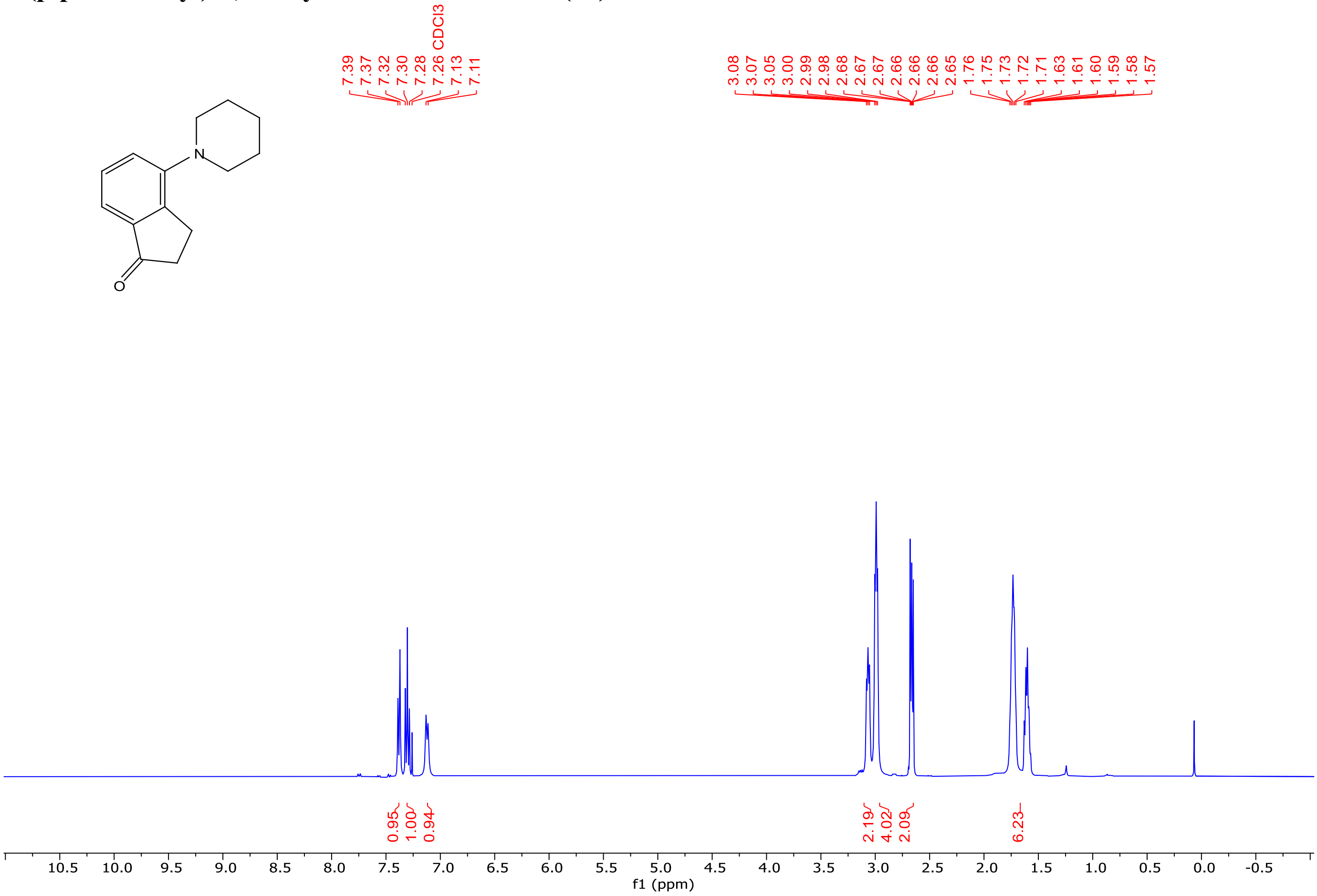

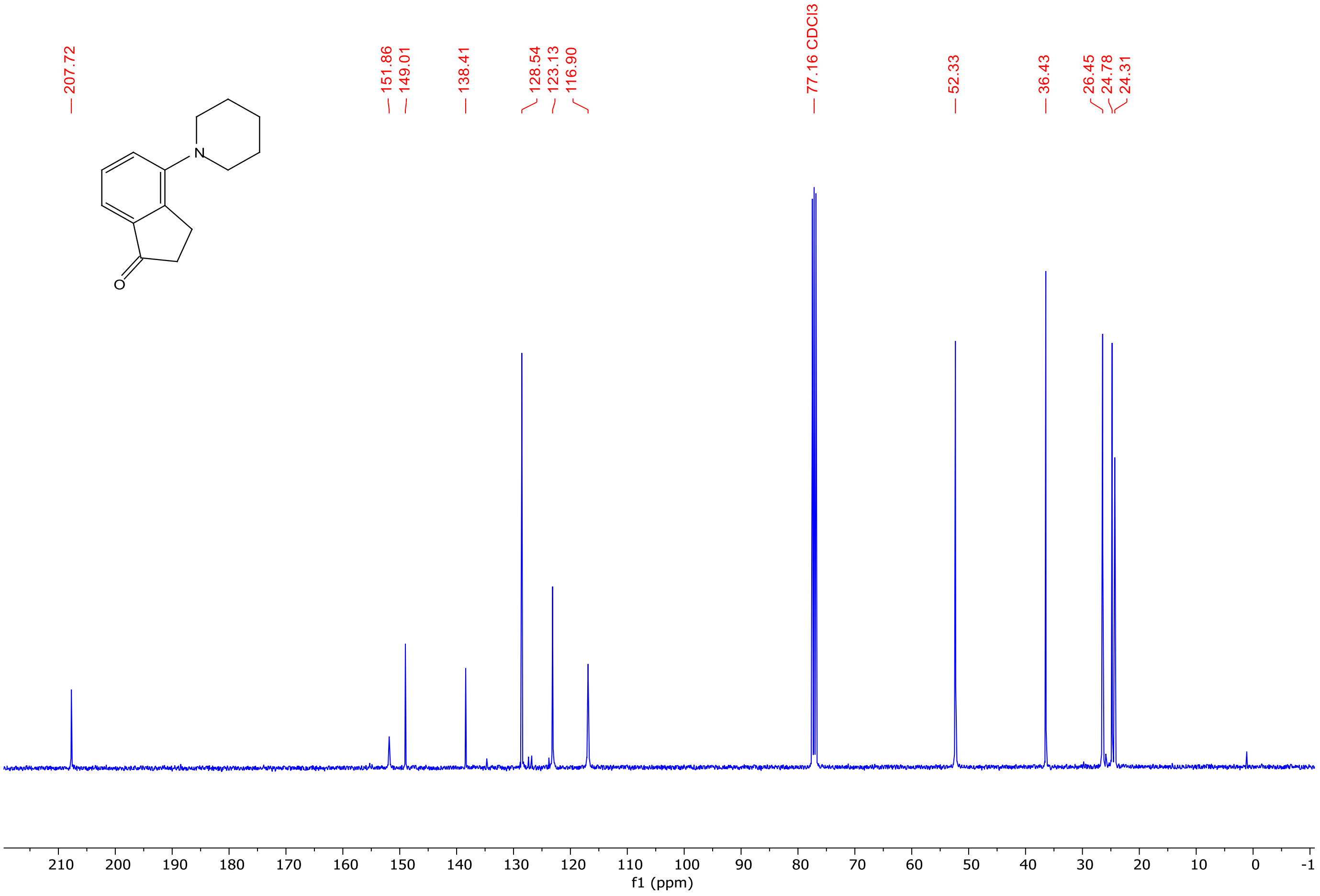

1-(thiophen-3-yl)piperidine (30)

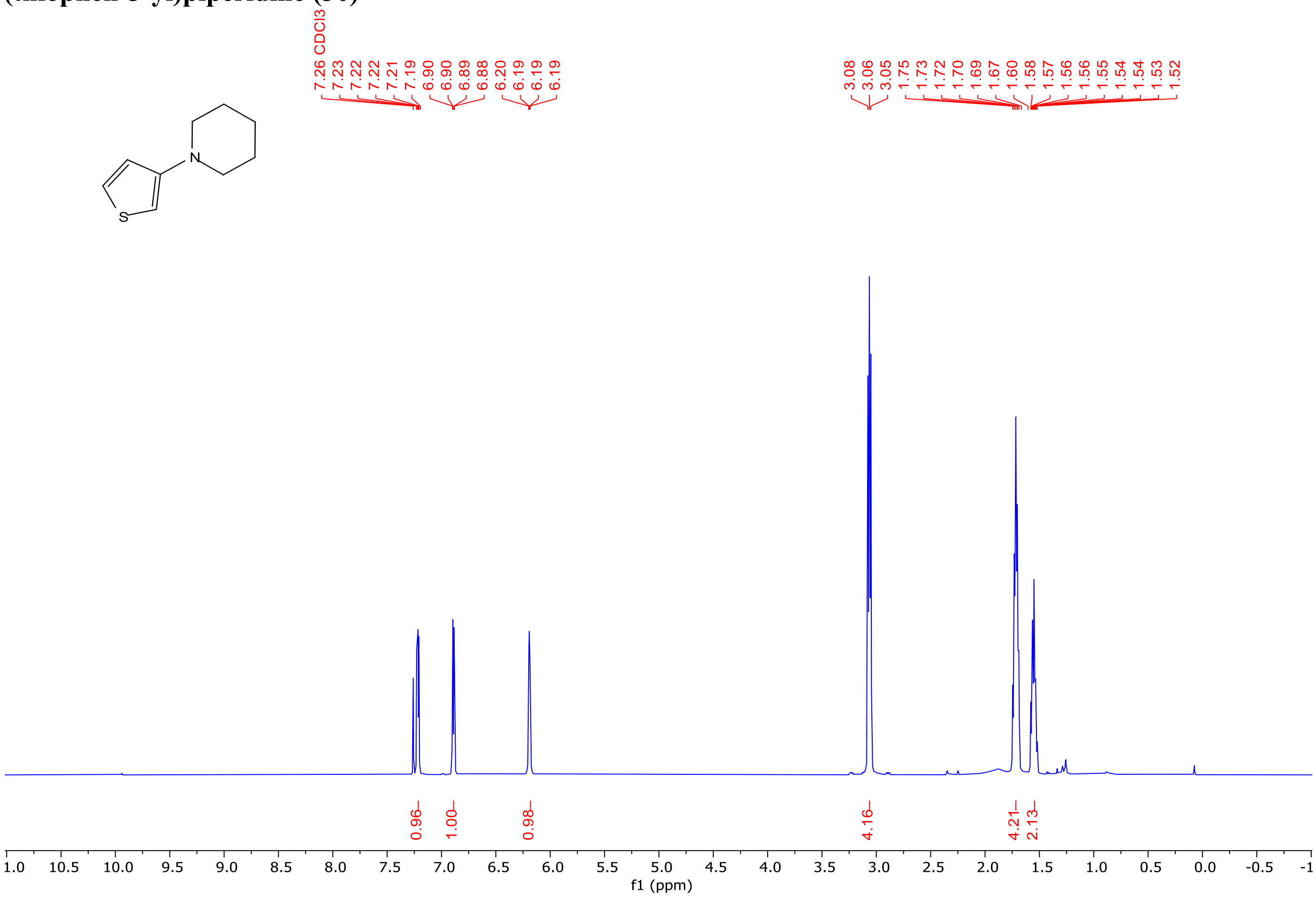

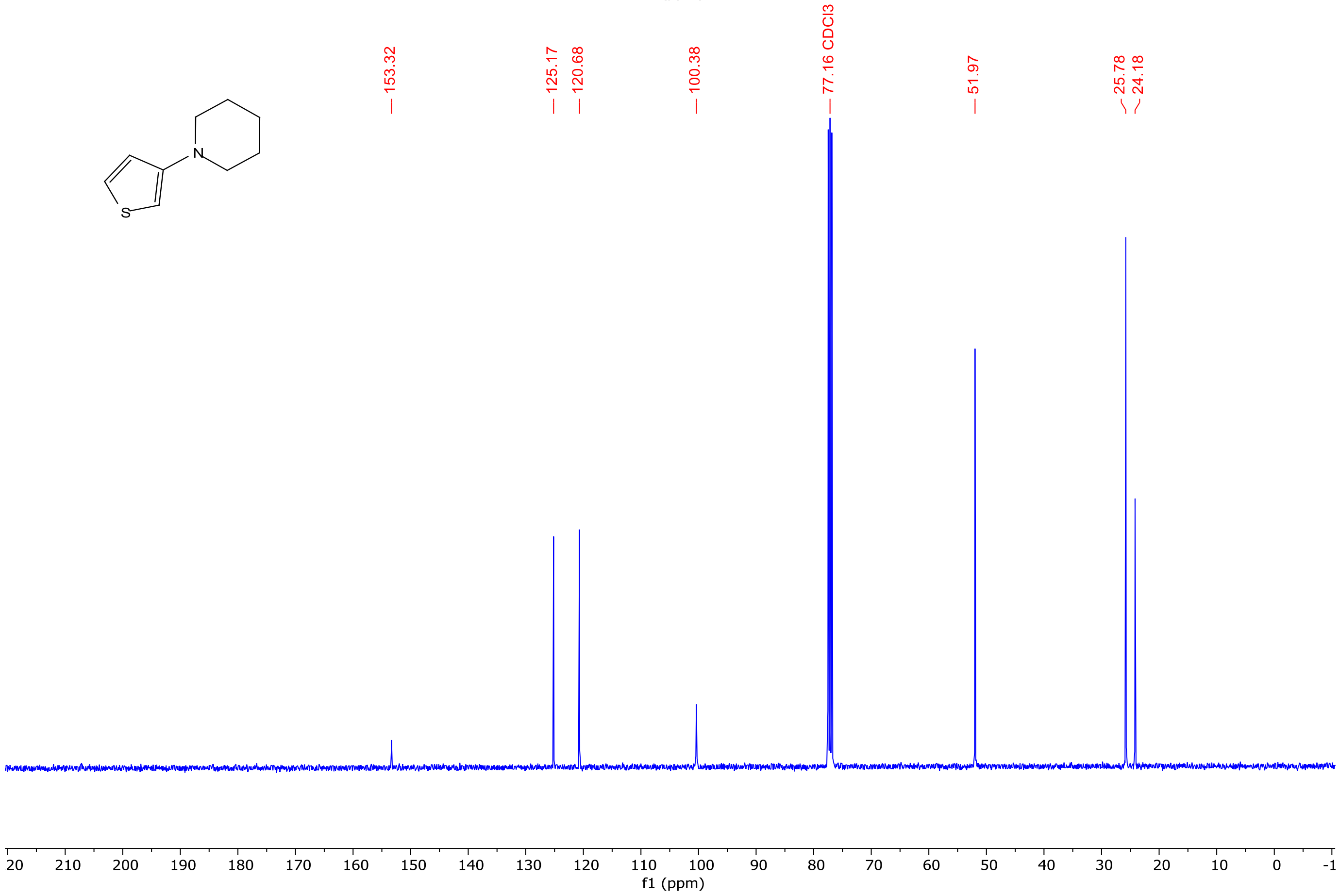

4-(piperidin-1-yl)picolinonitrile (31)

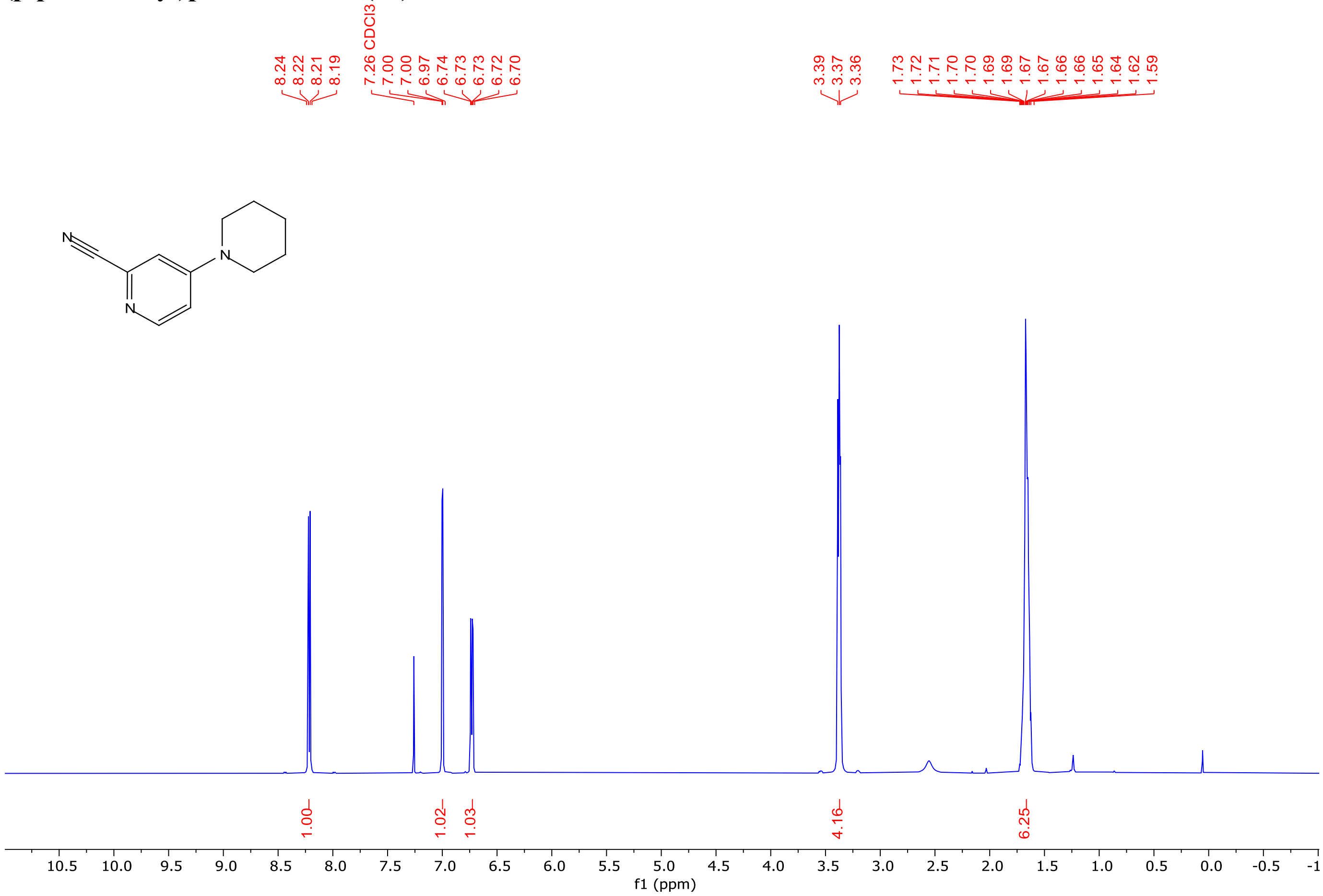

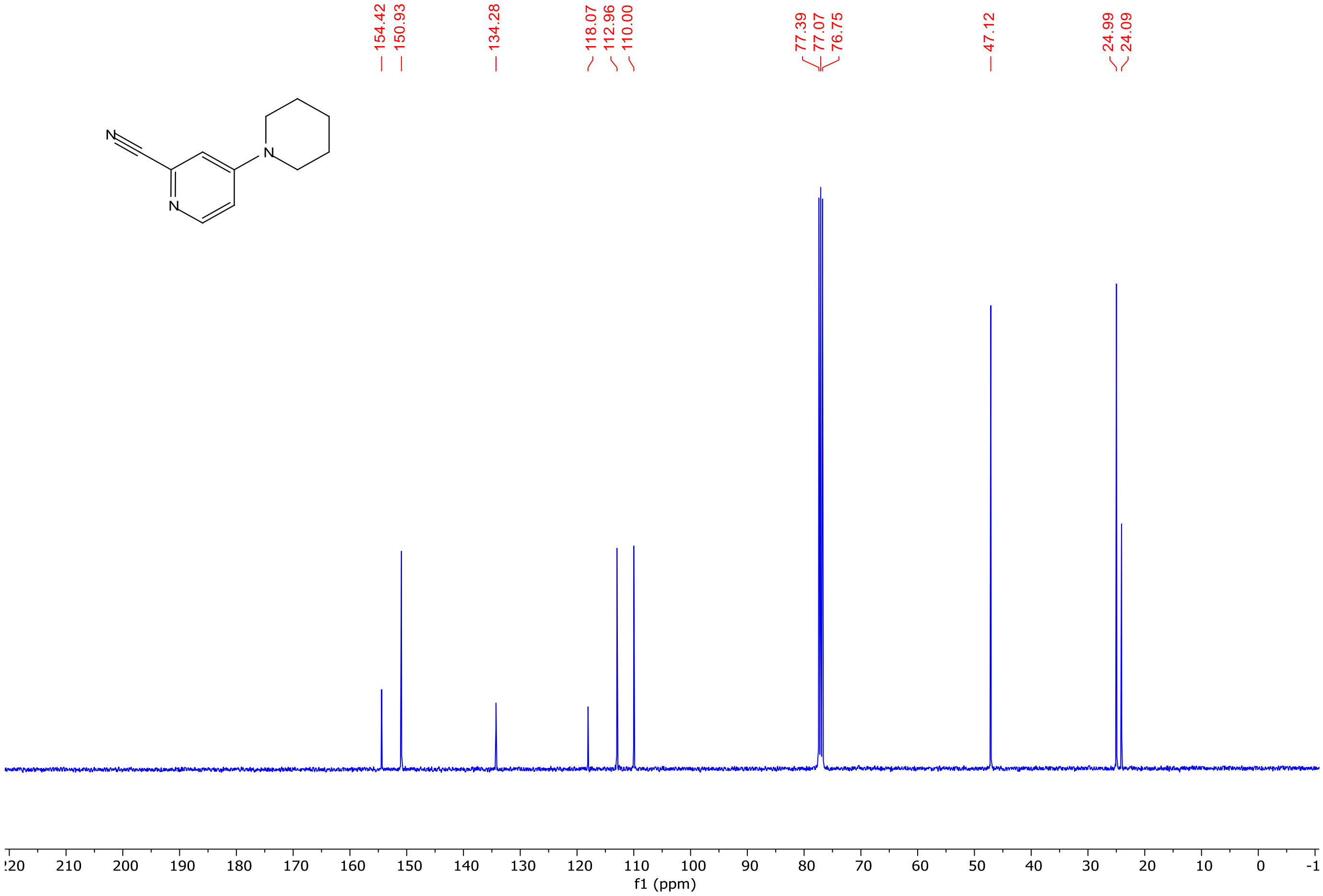

1-(naphthalen-2-yl)piperidine (32)

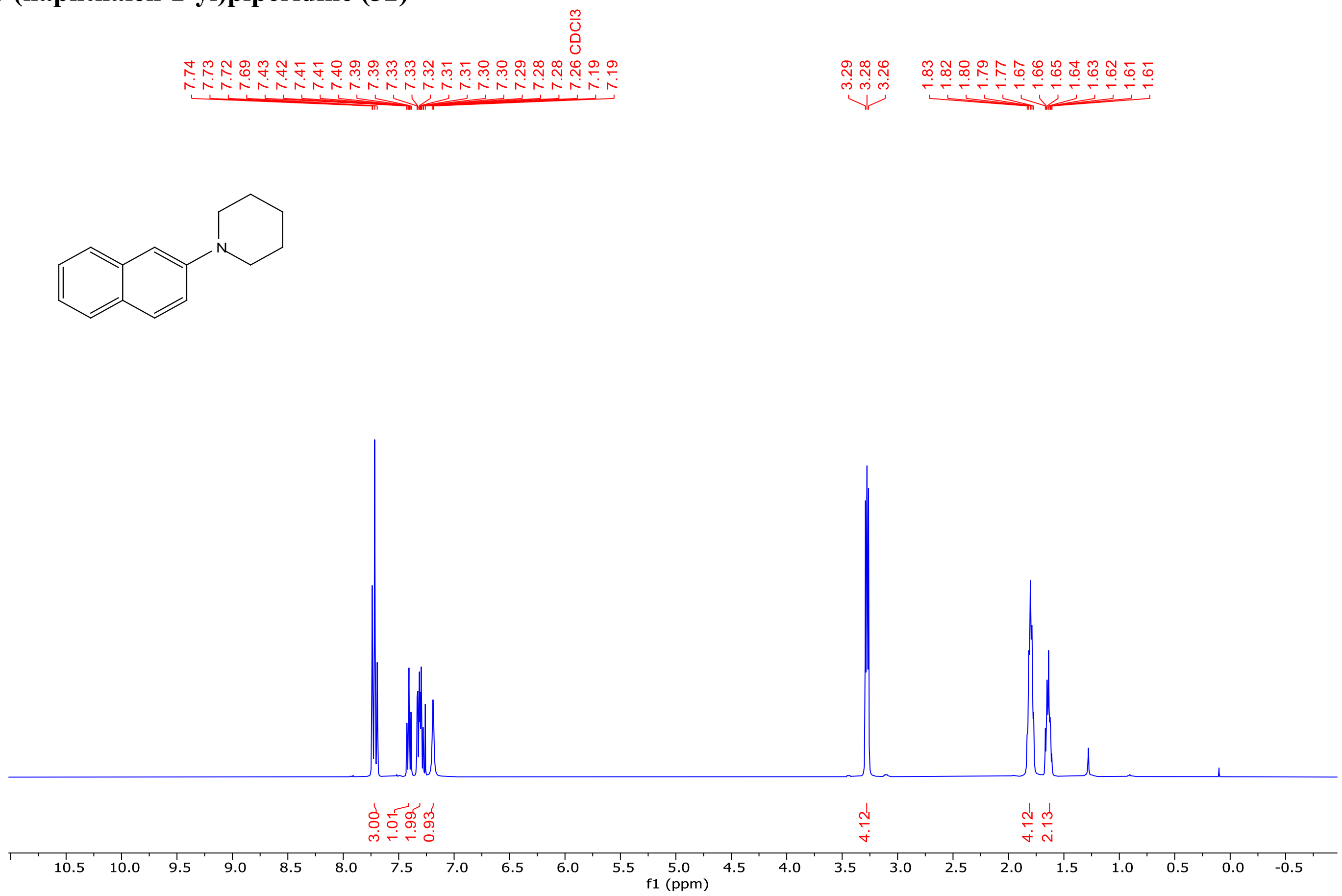

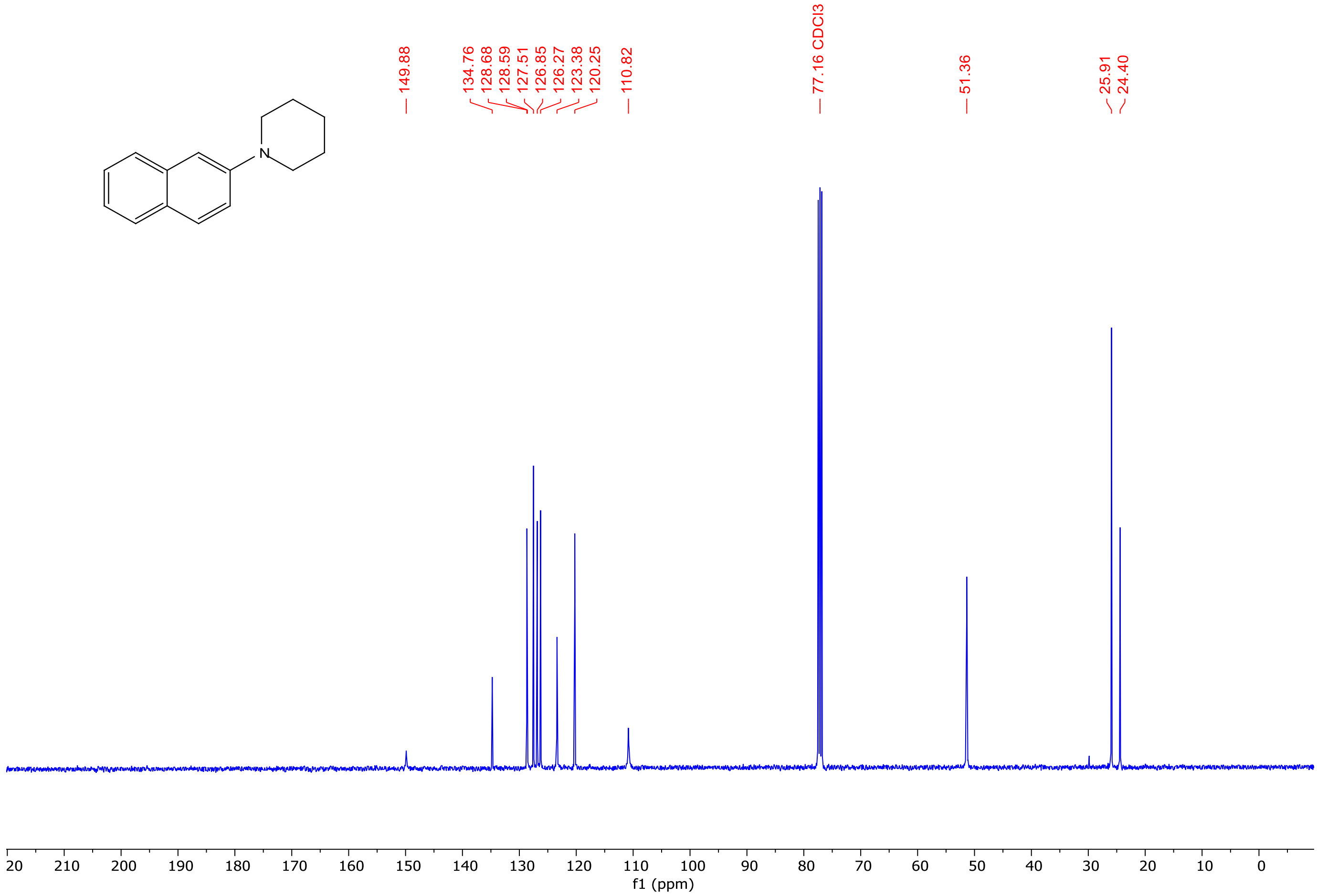

phenyl 3-(piperidin-1-yl)quinoline (33)

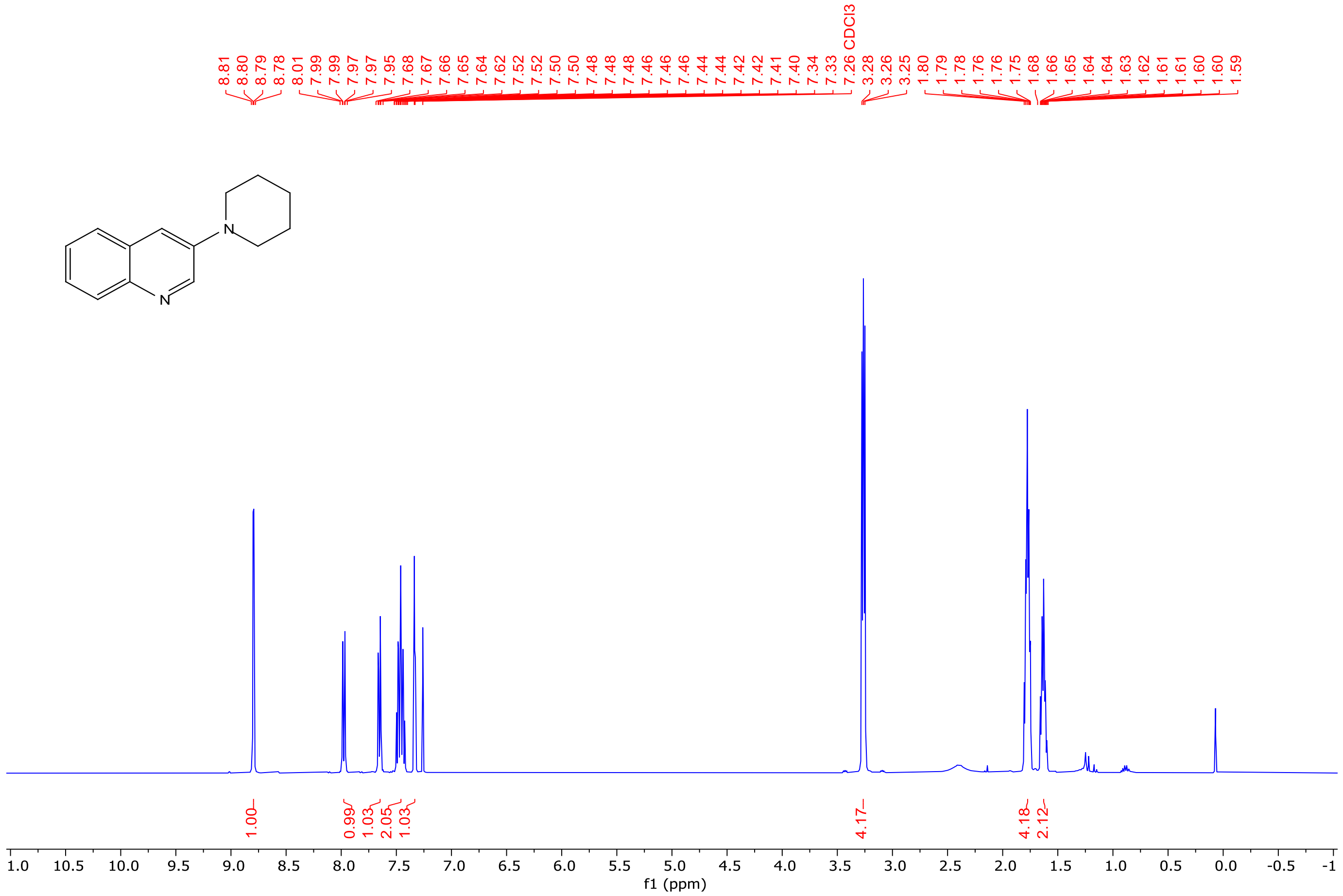

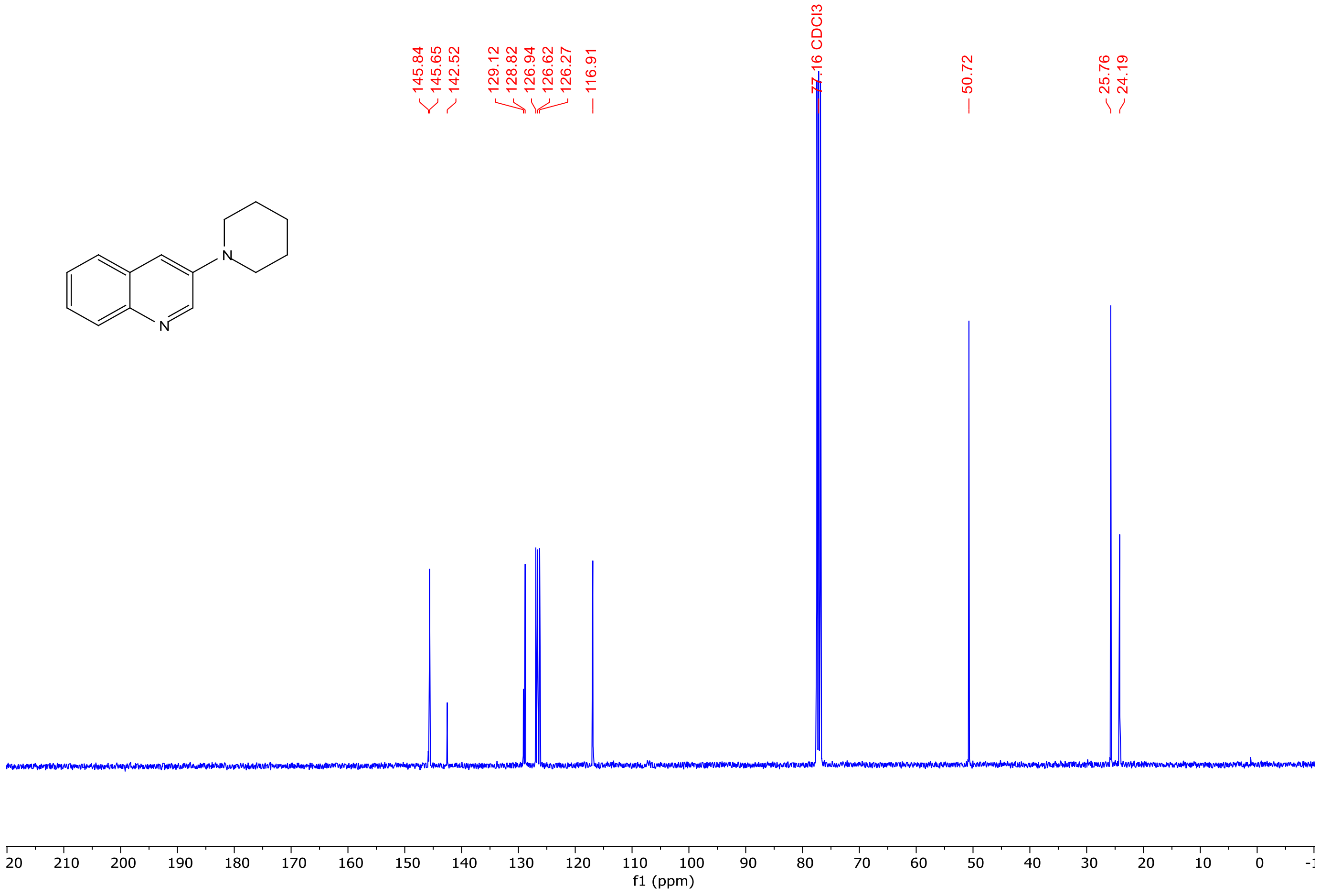

(4-(pyrrolidin-1-yl)phenyl)methanone (34)

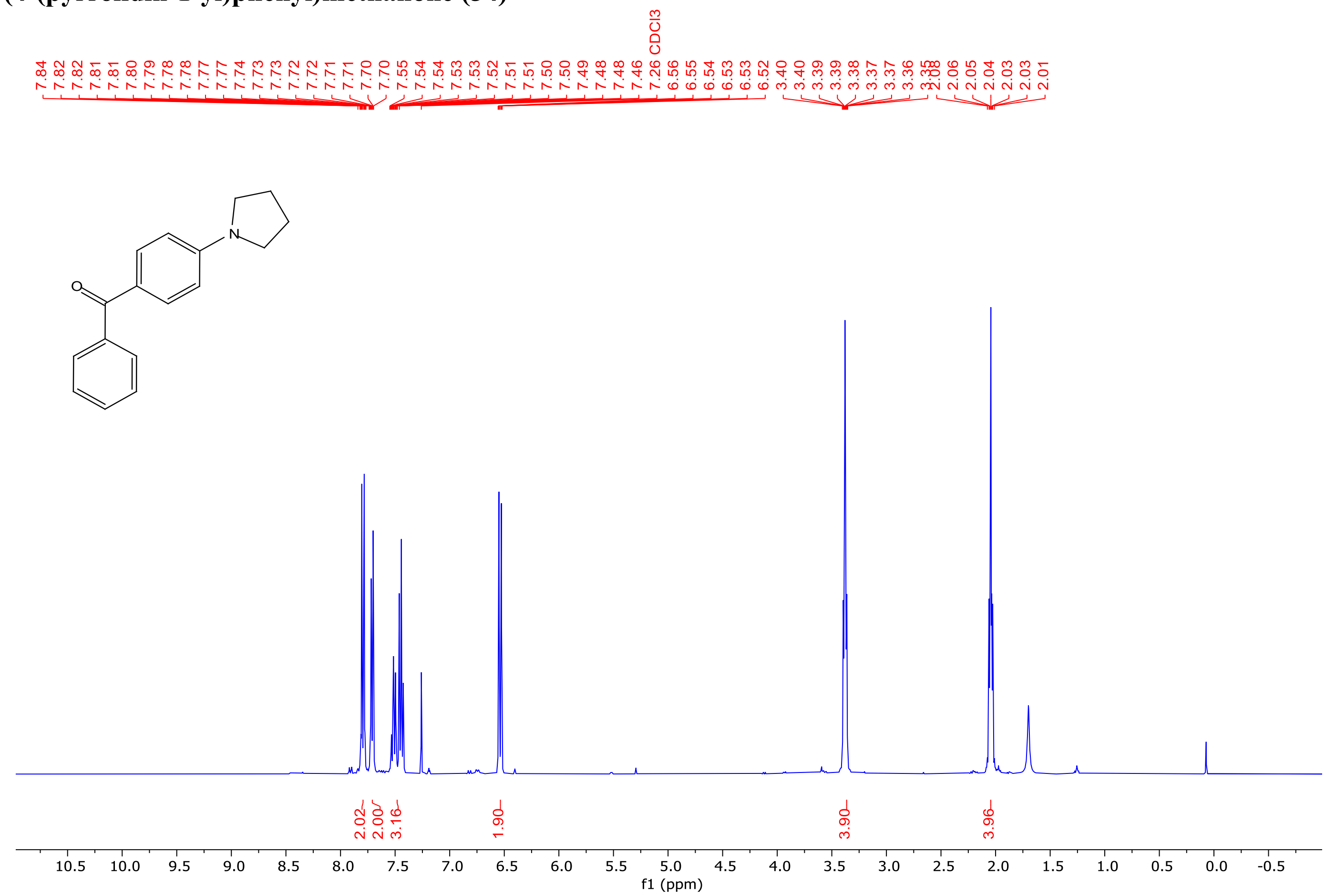

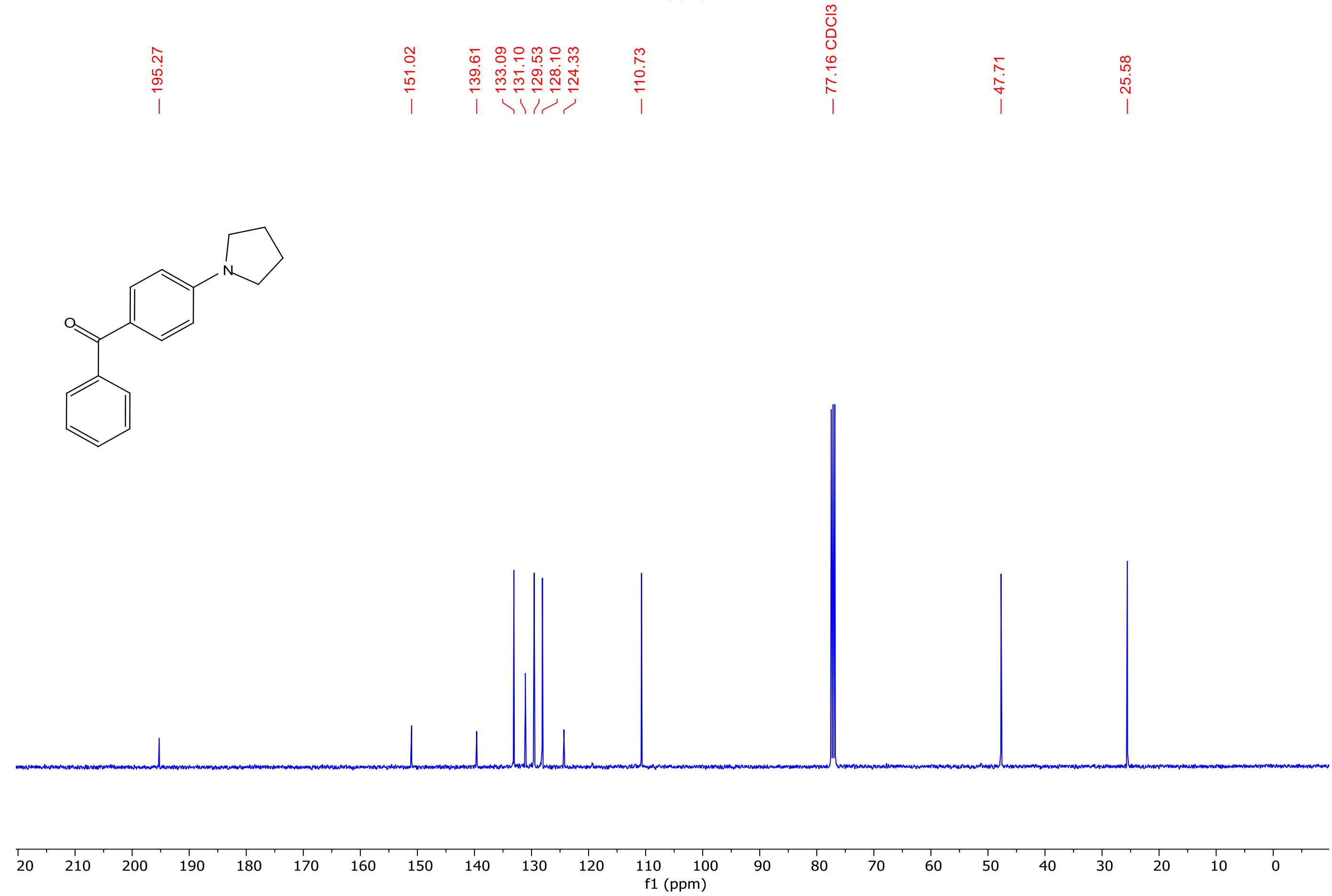

(4-morpholinophenyl)(phenyl)methanone (35)

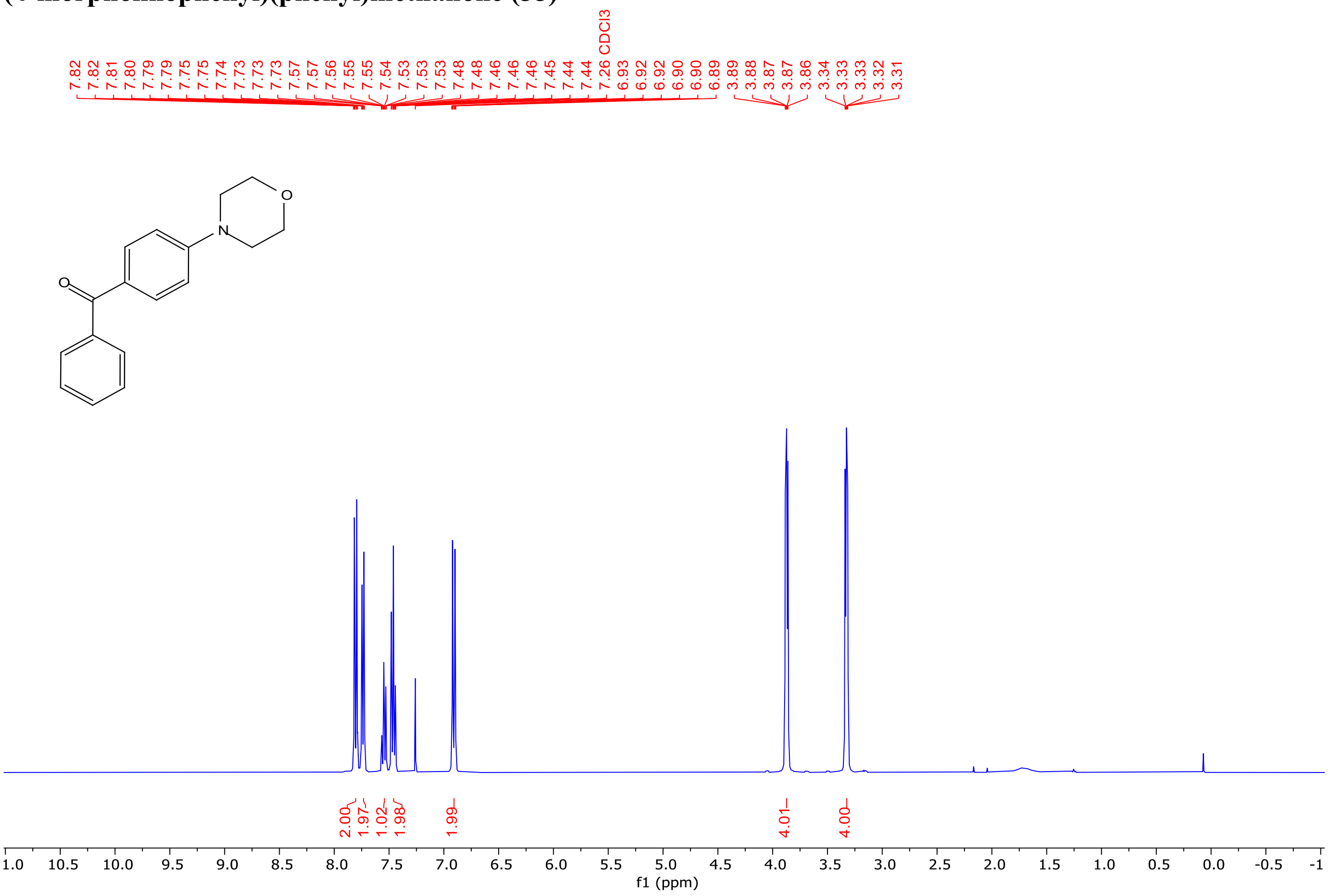

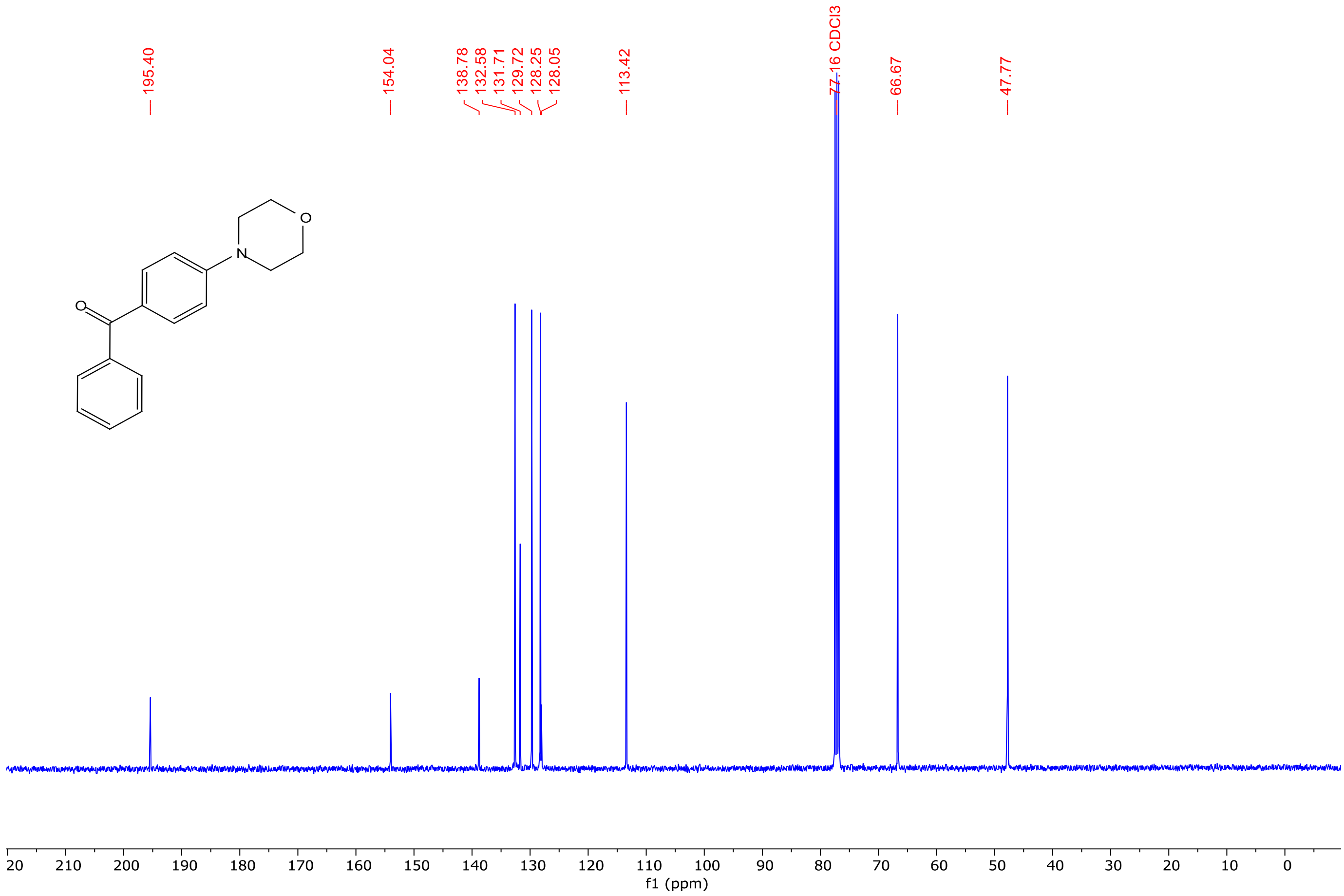

***tert*-butyl 4-(4-benzoylphenyl)piperazine-1-carboxylate (36)**

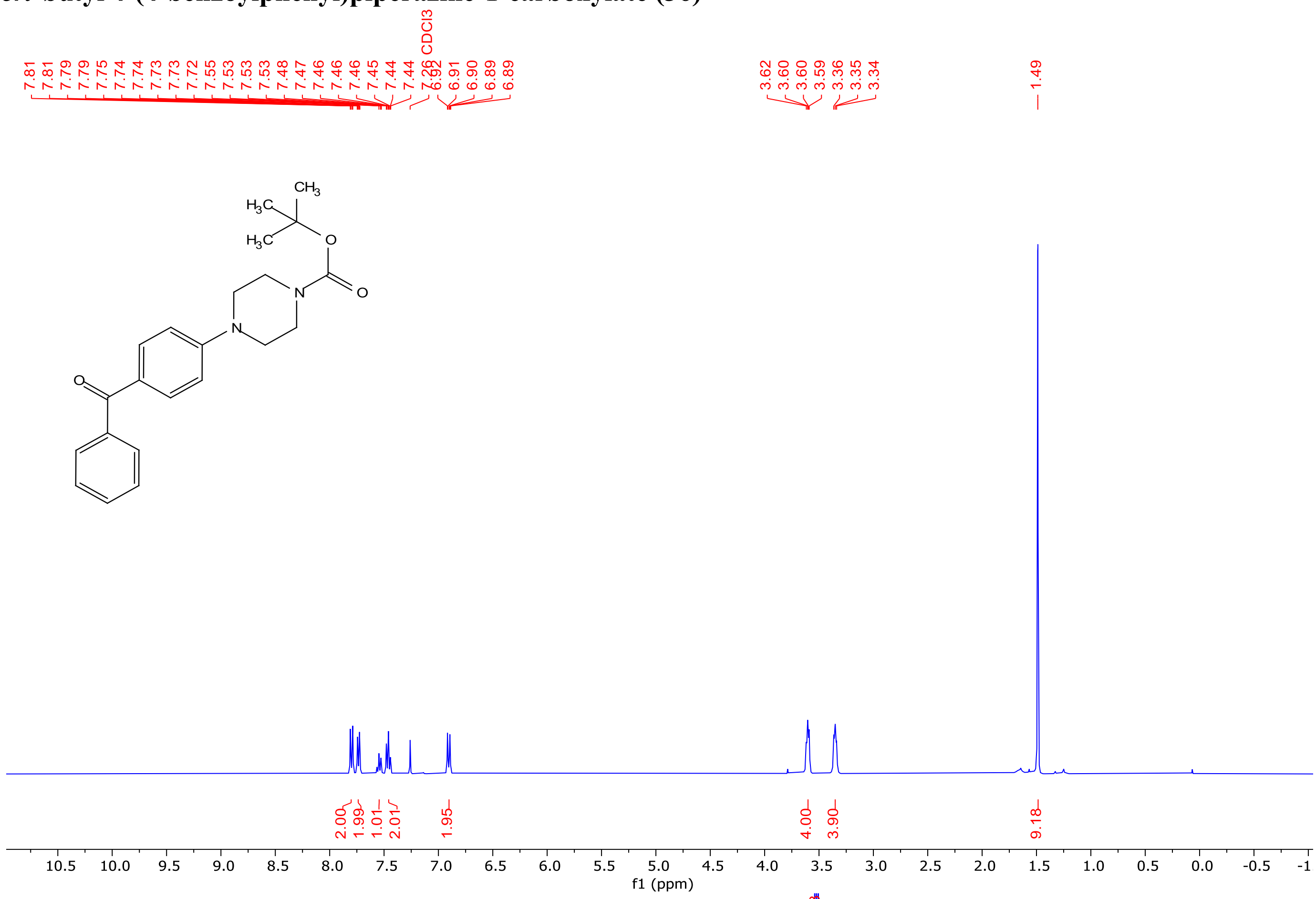

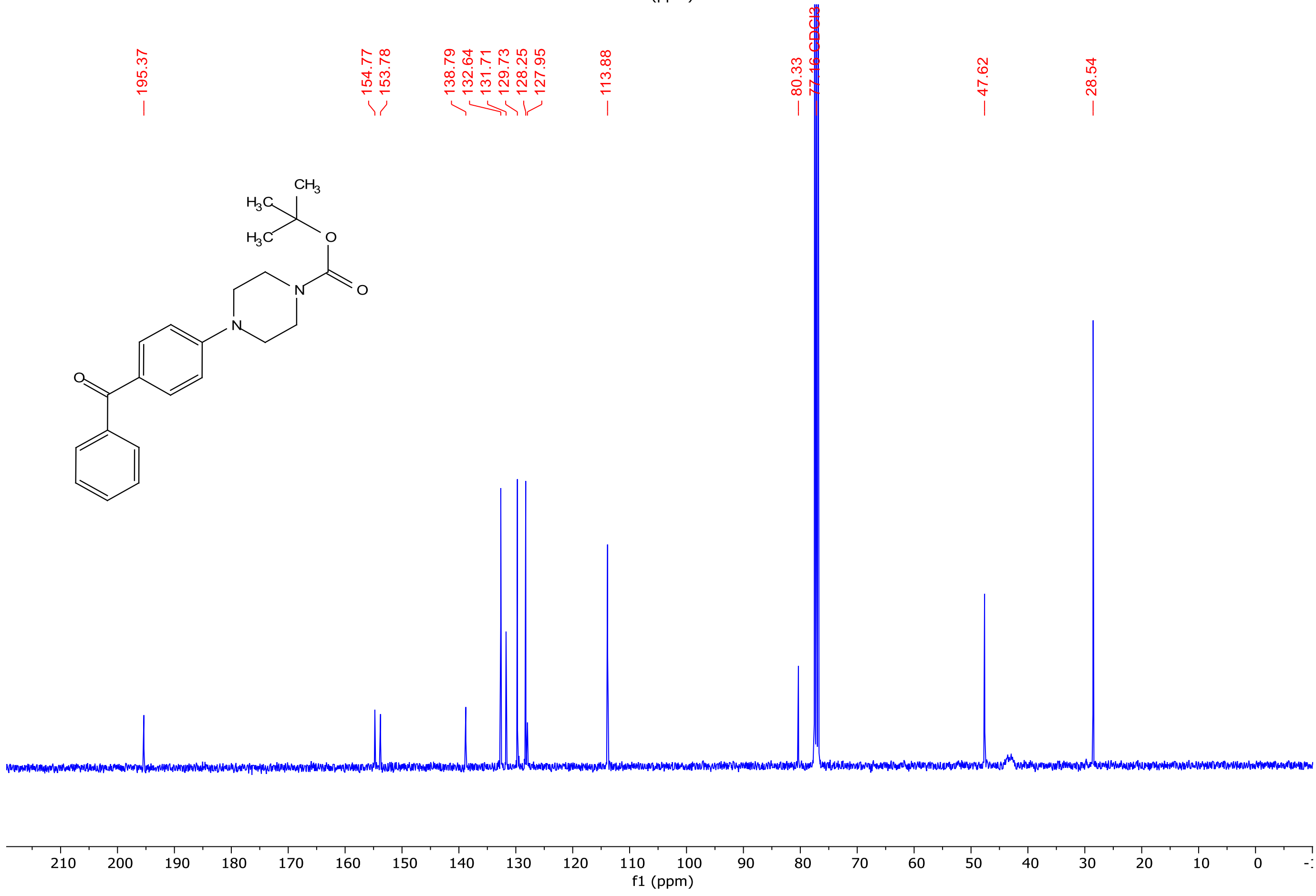

4-methoxy-*N*-phenylaniline (37)

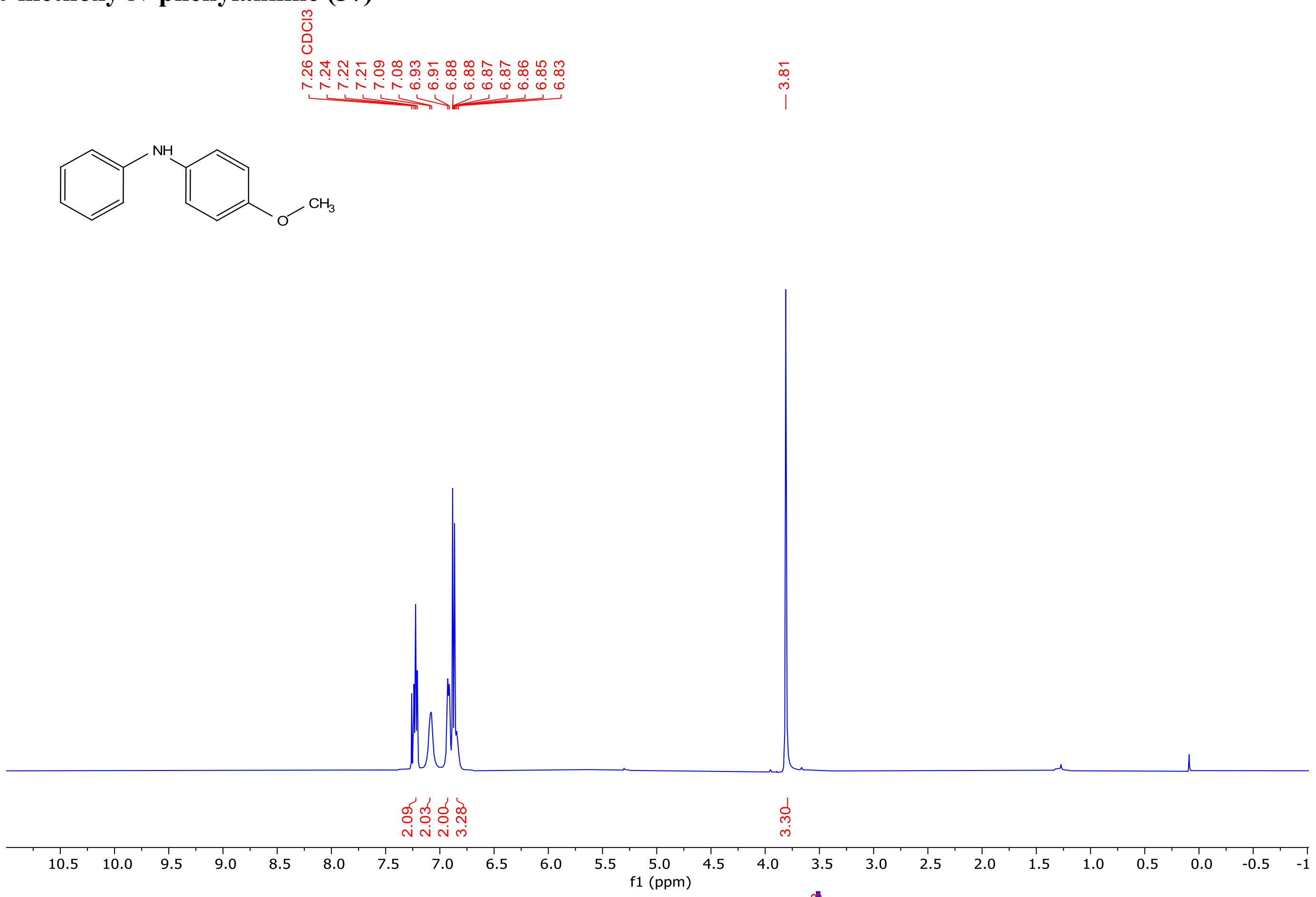

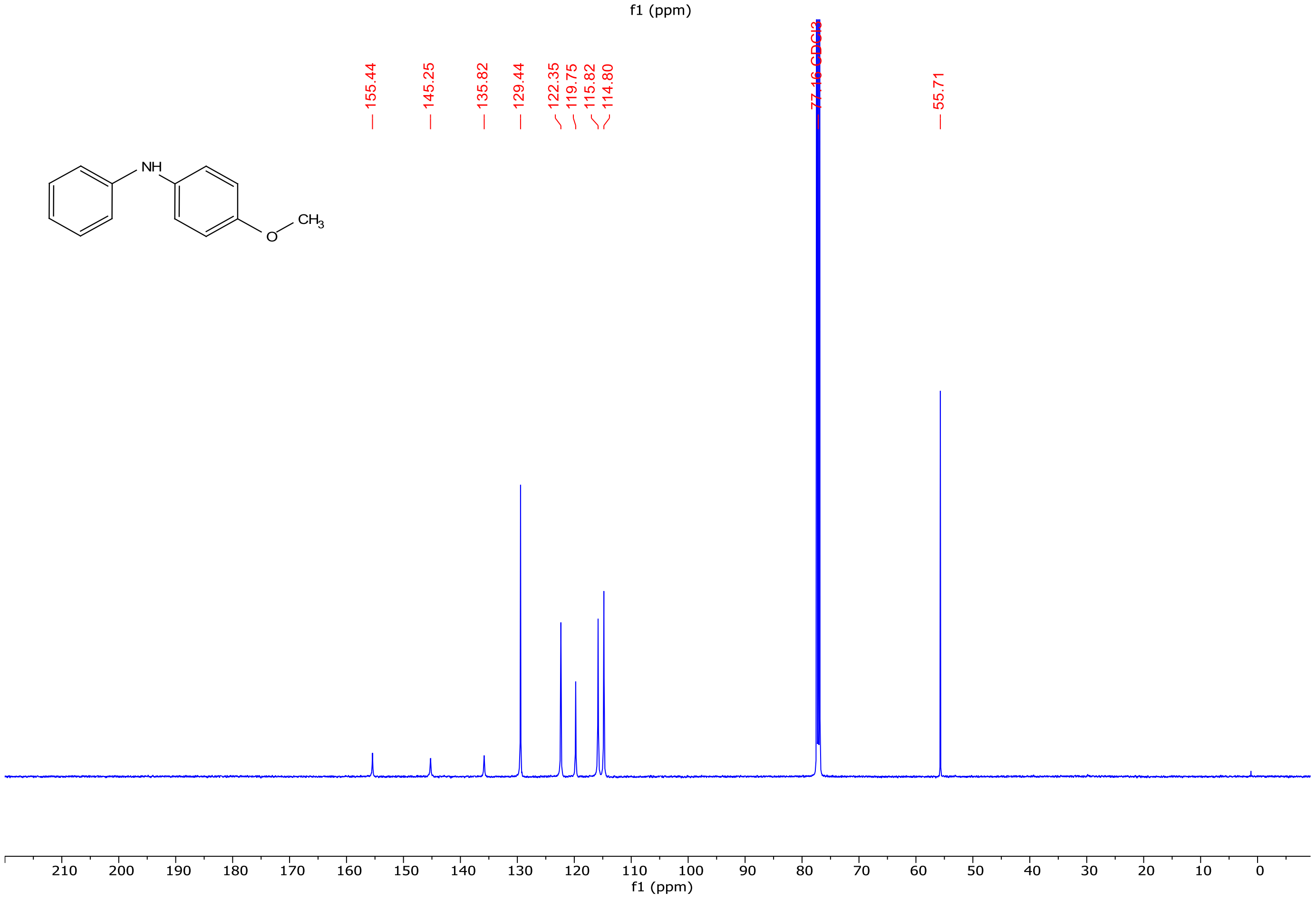

4-(*tert*-butyl)-*N*-(4-methoxyphenyl)aniline (38)

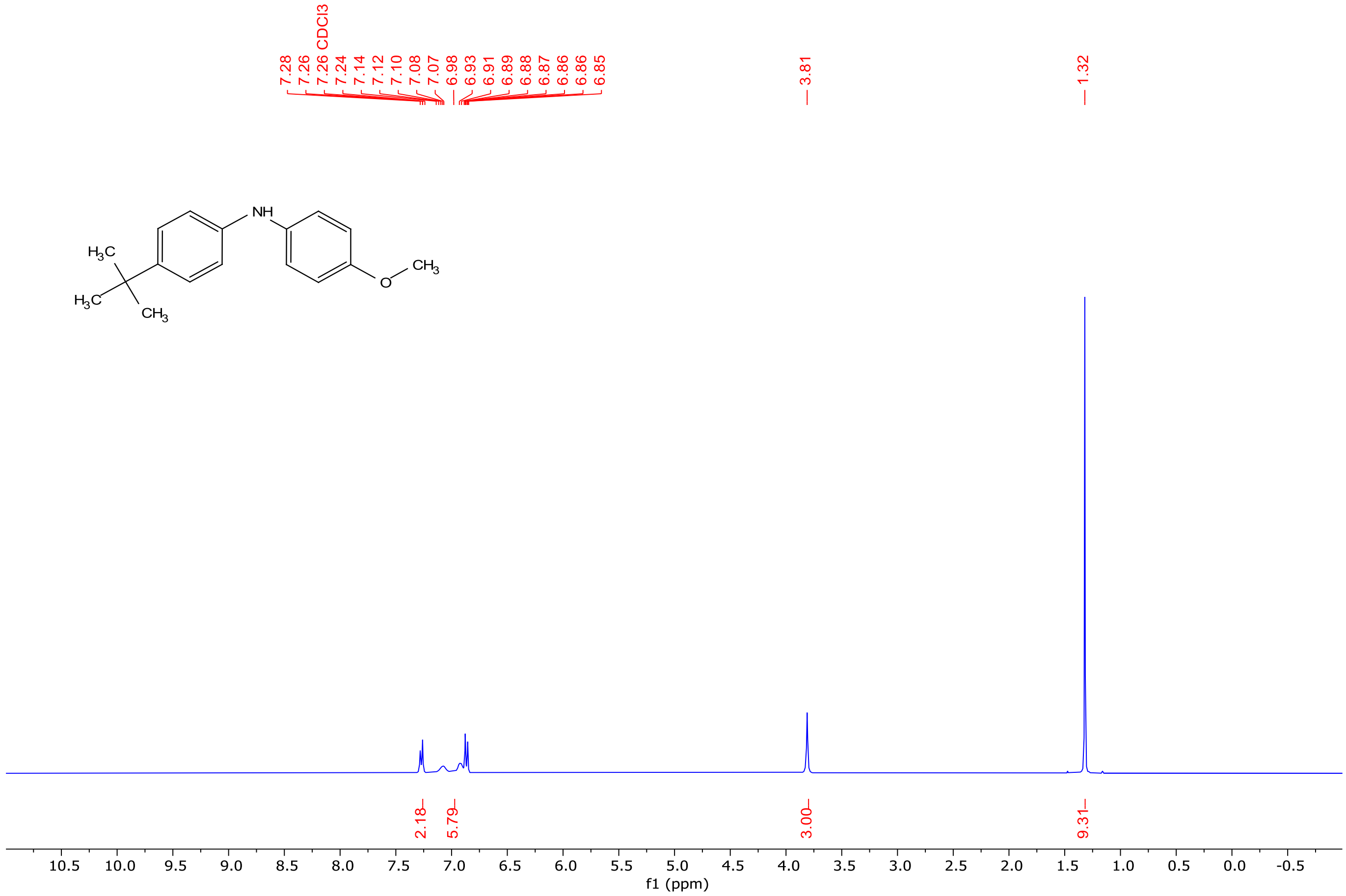

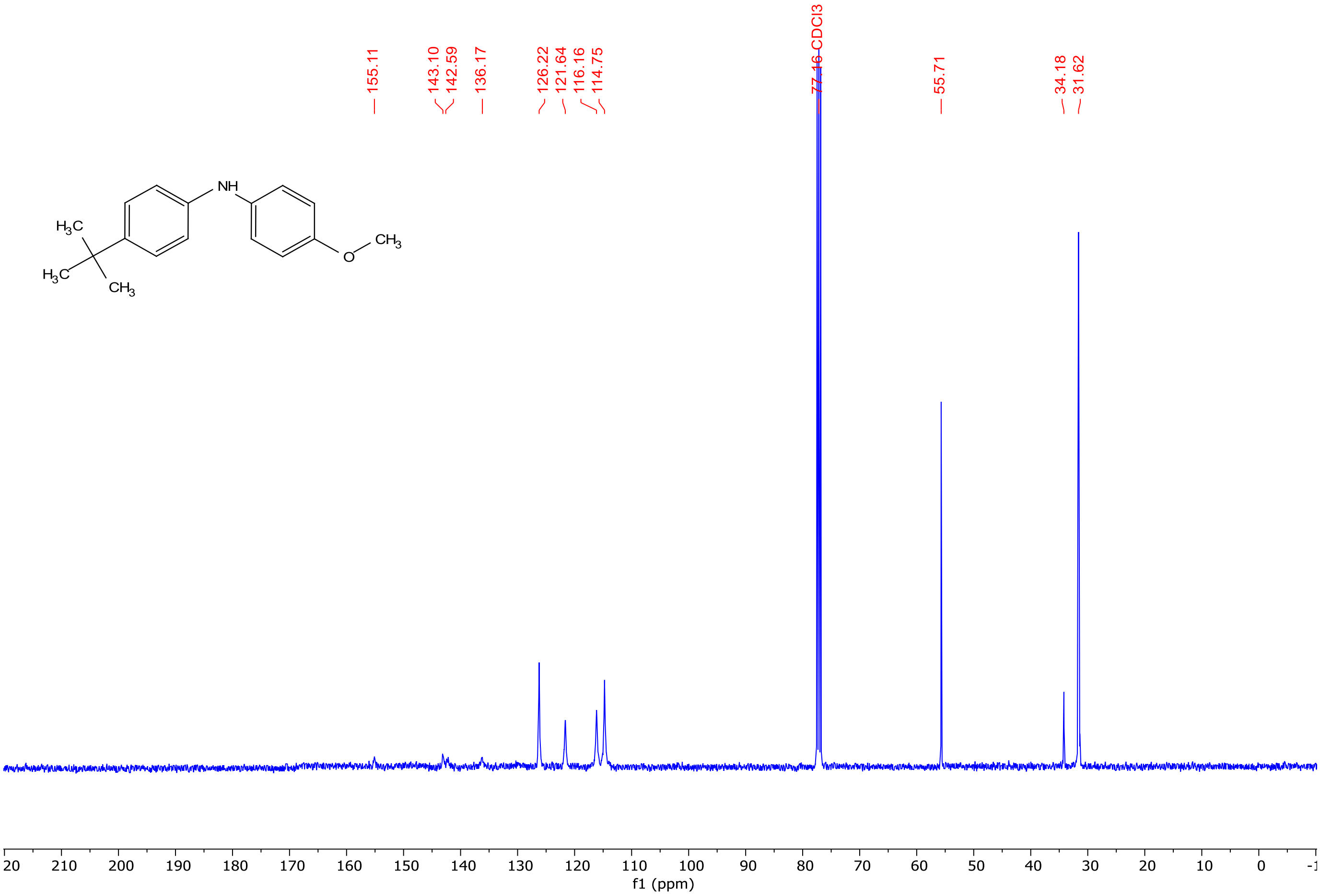

N-(4-methoxyphenyl)-[1,1'-biphenyl]-4-amine (39)

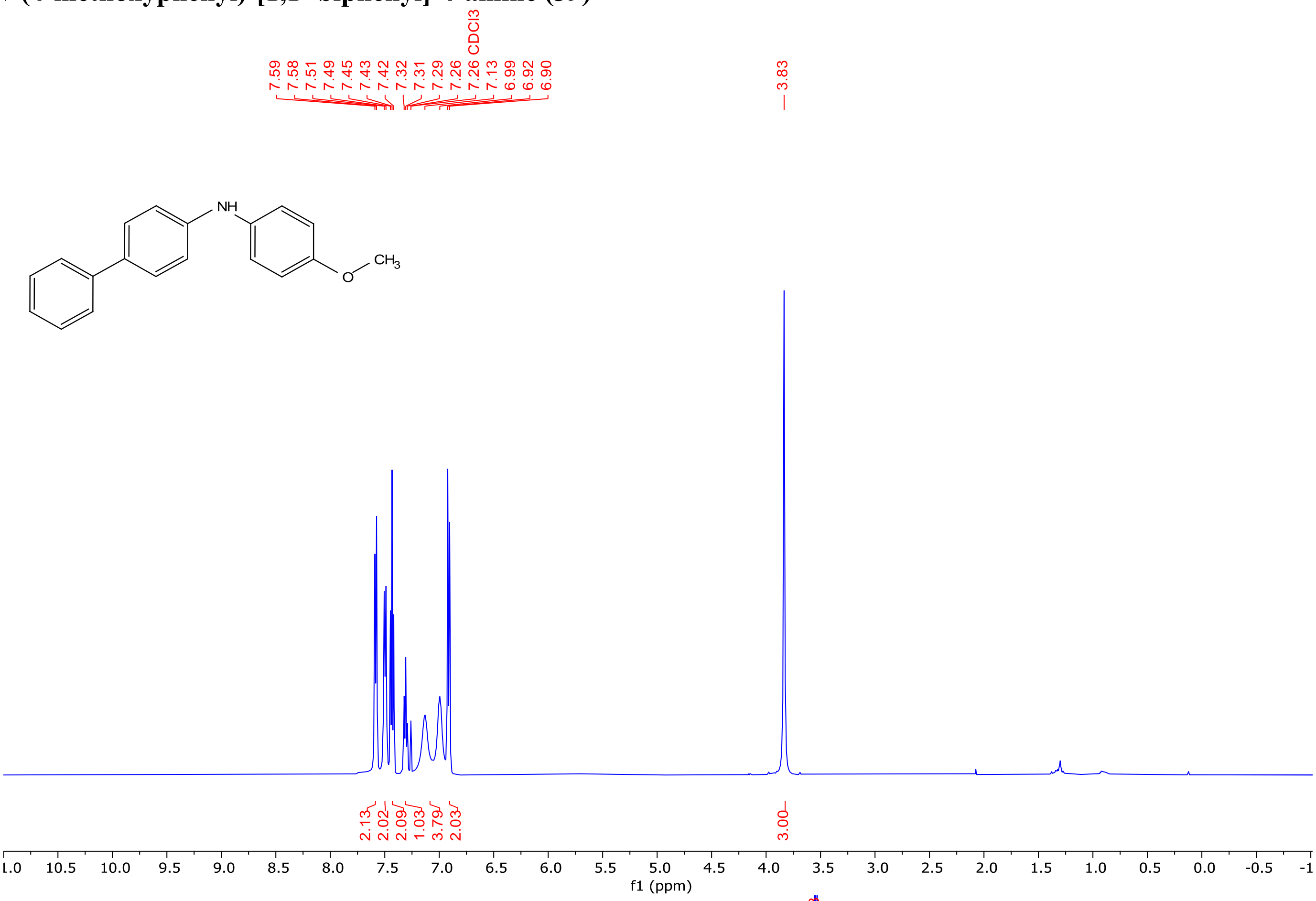

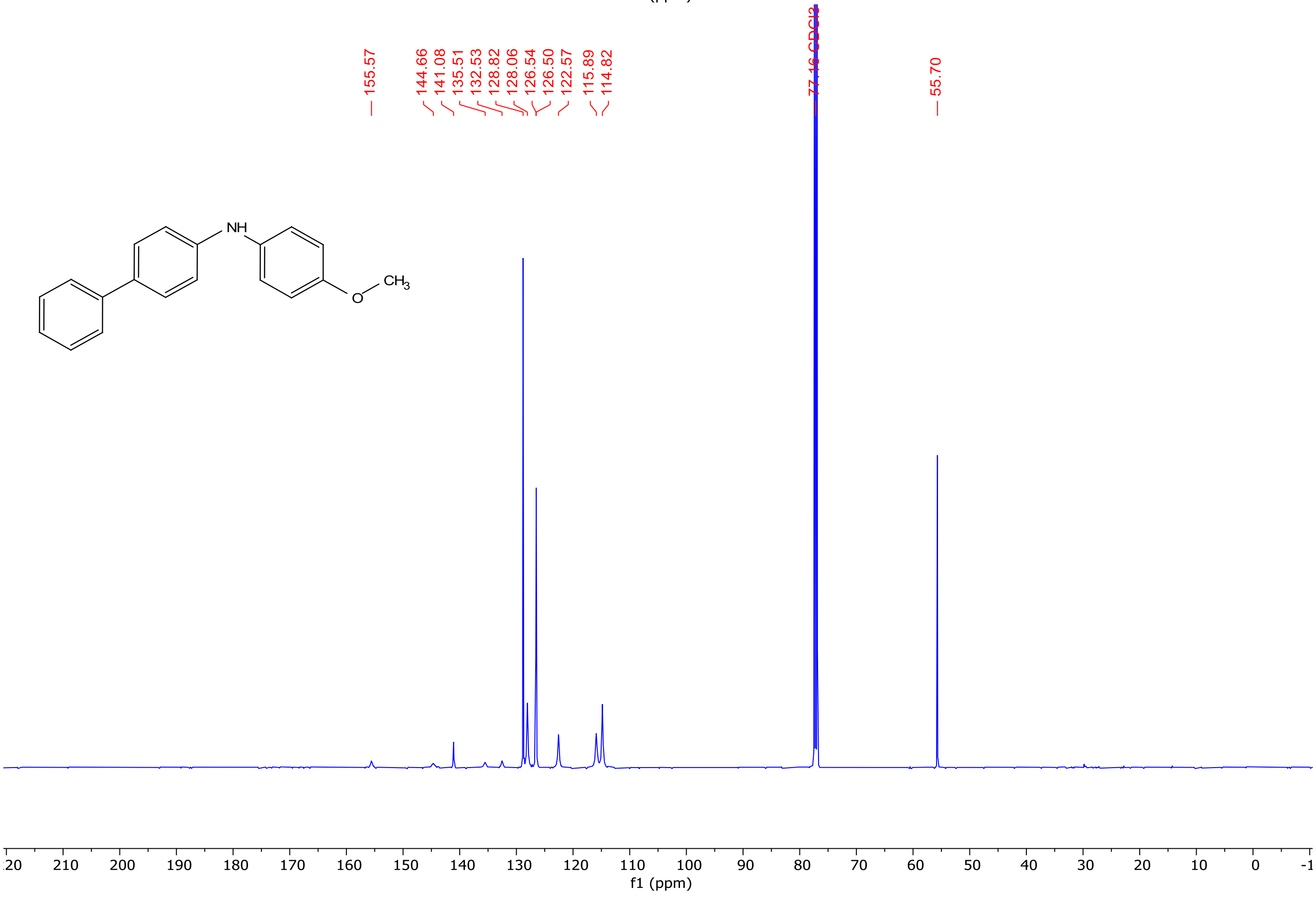

methyl 4-((4-methoxyphenyl)amino)benzoate (40)

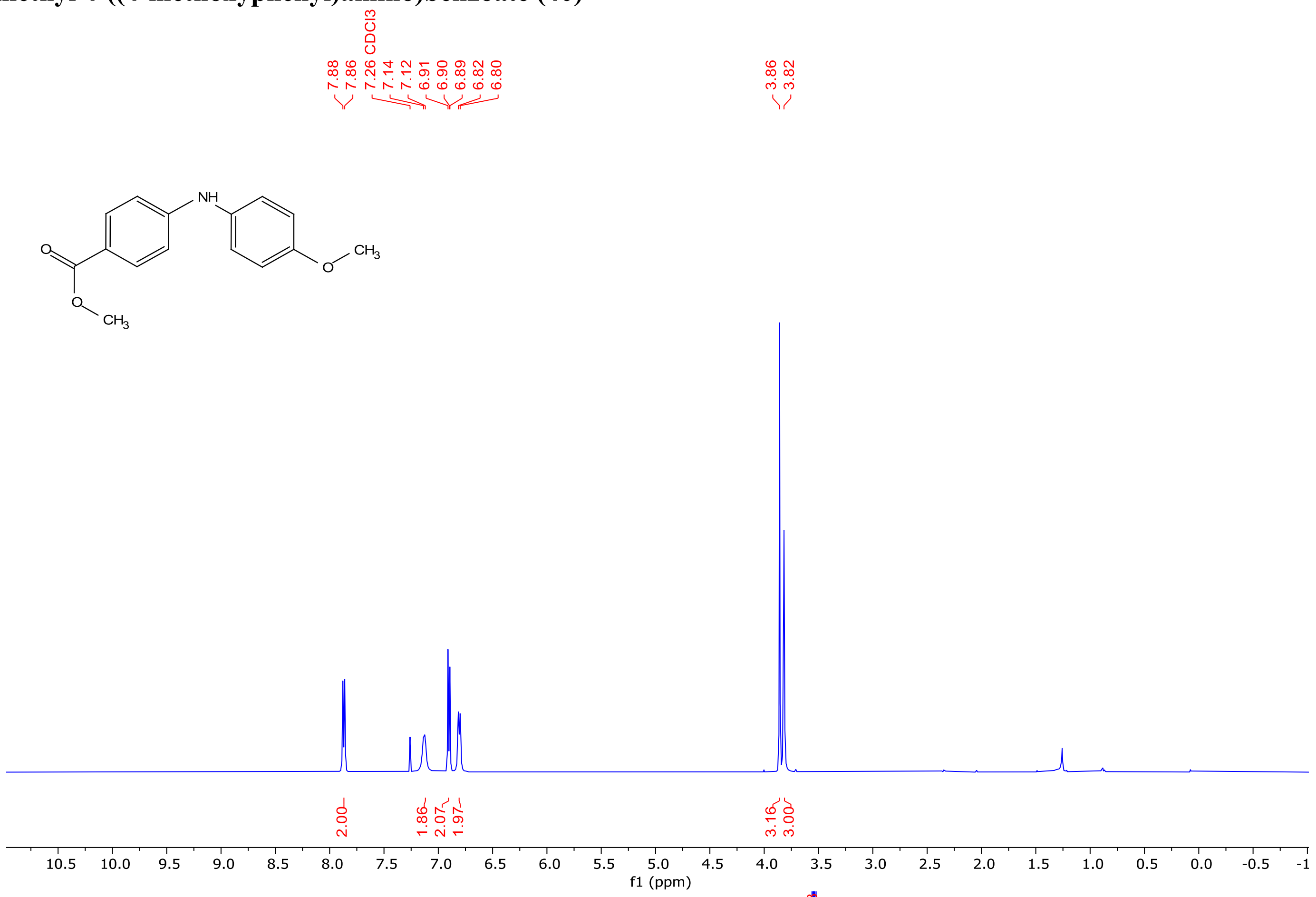

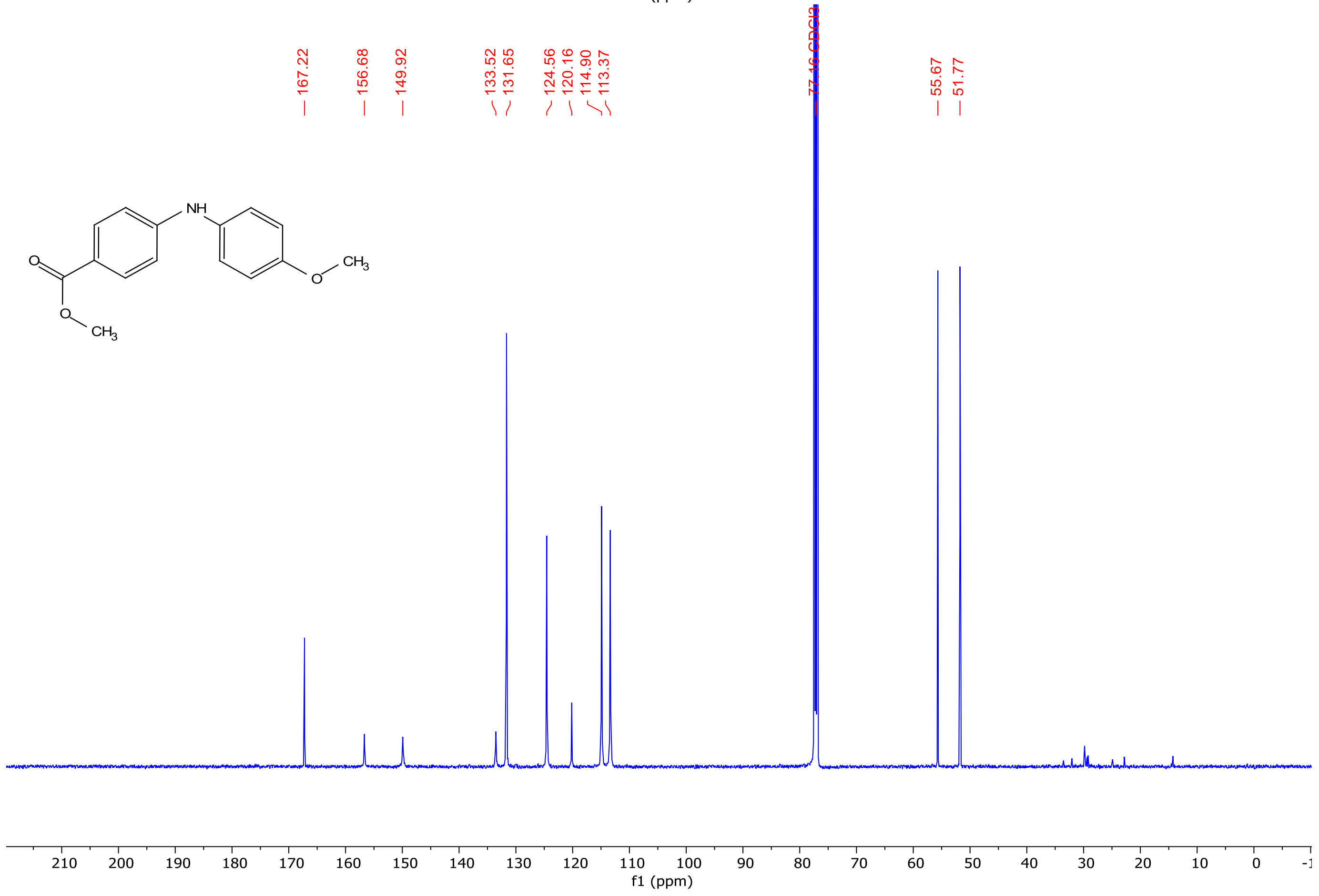

(4-((4-methoxyphenyl)amino)phenyl)(phenyl)methanone (41)

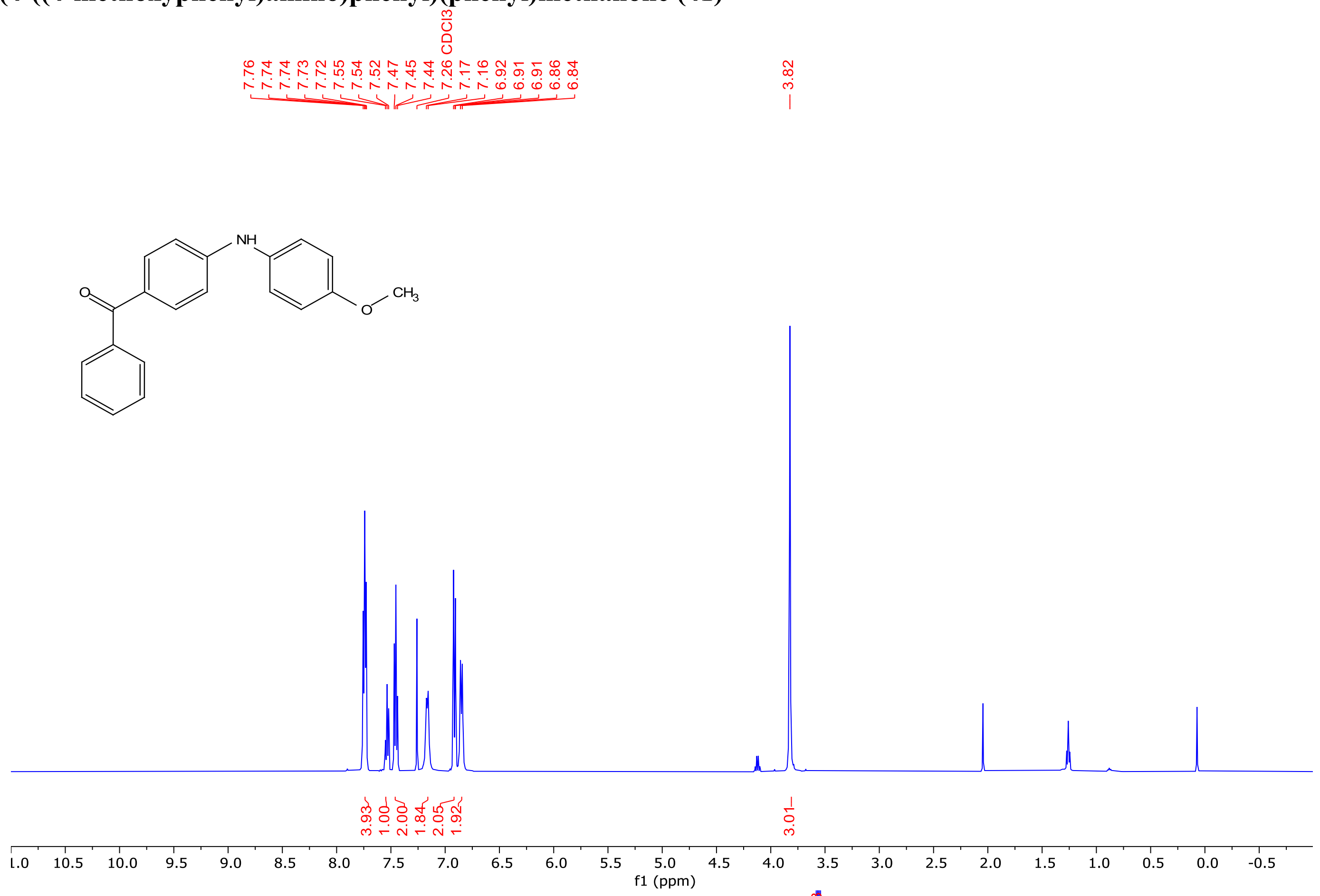

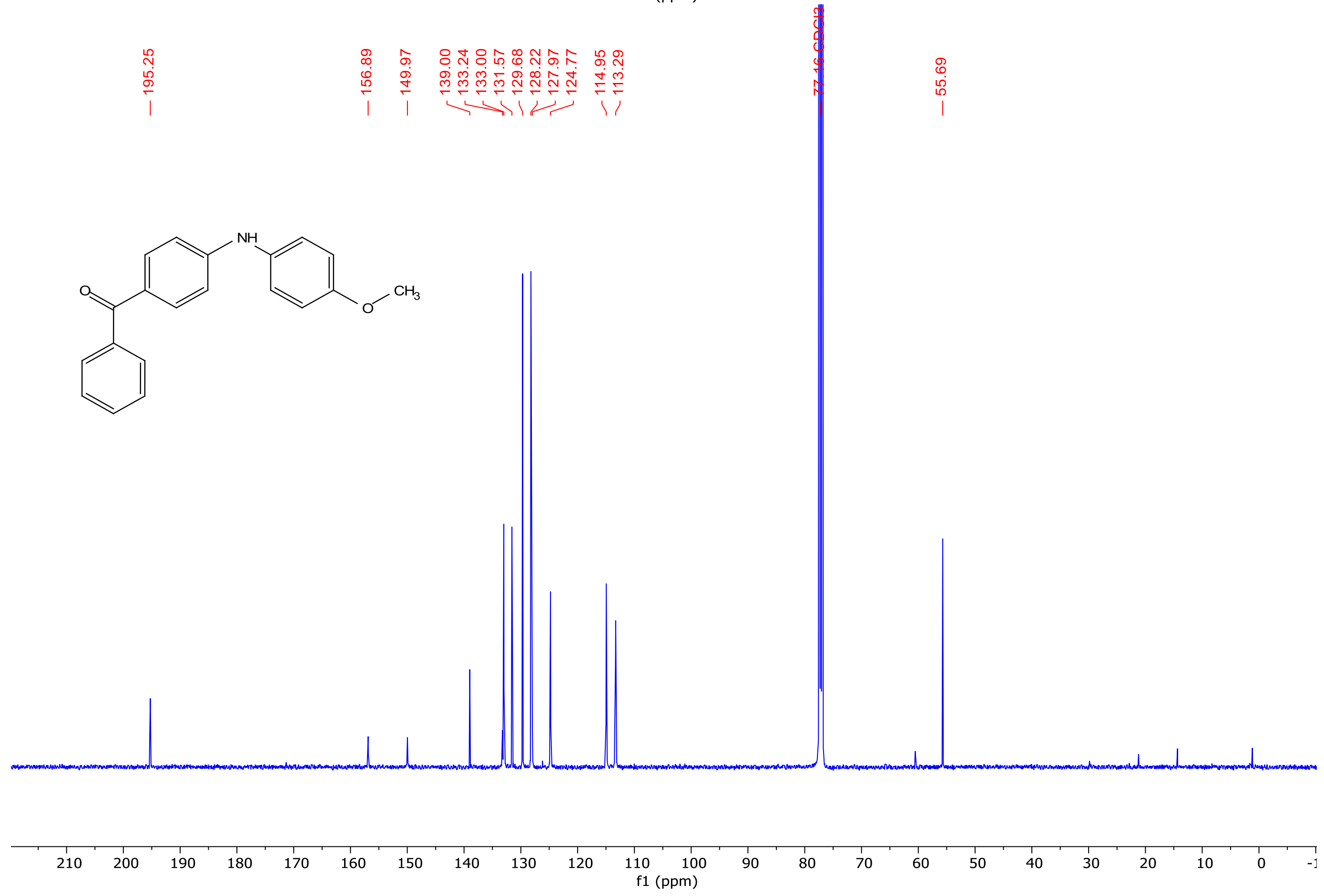

4-methoxy-*N*-(4-(trifluoromethyl)phenyl)aniline (42)

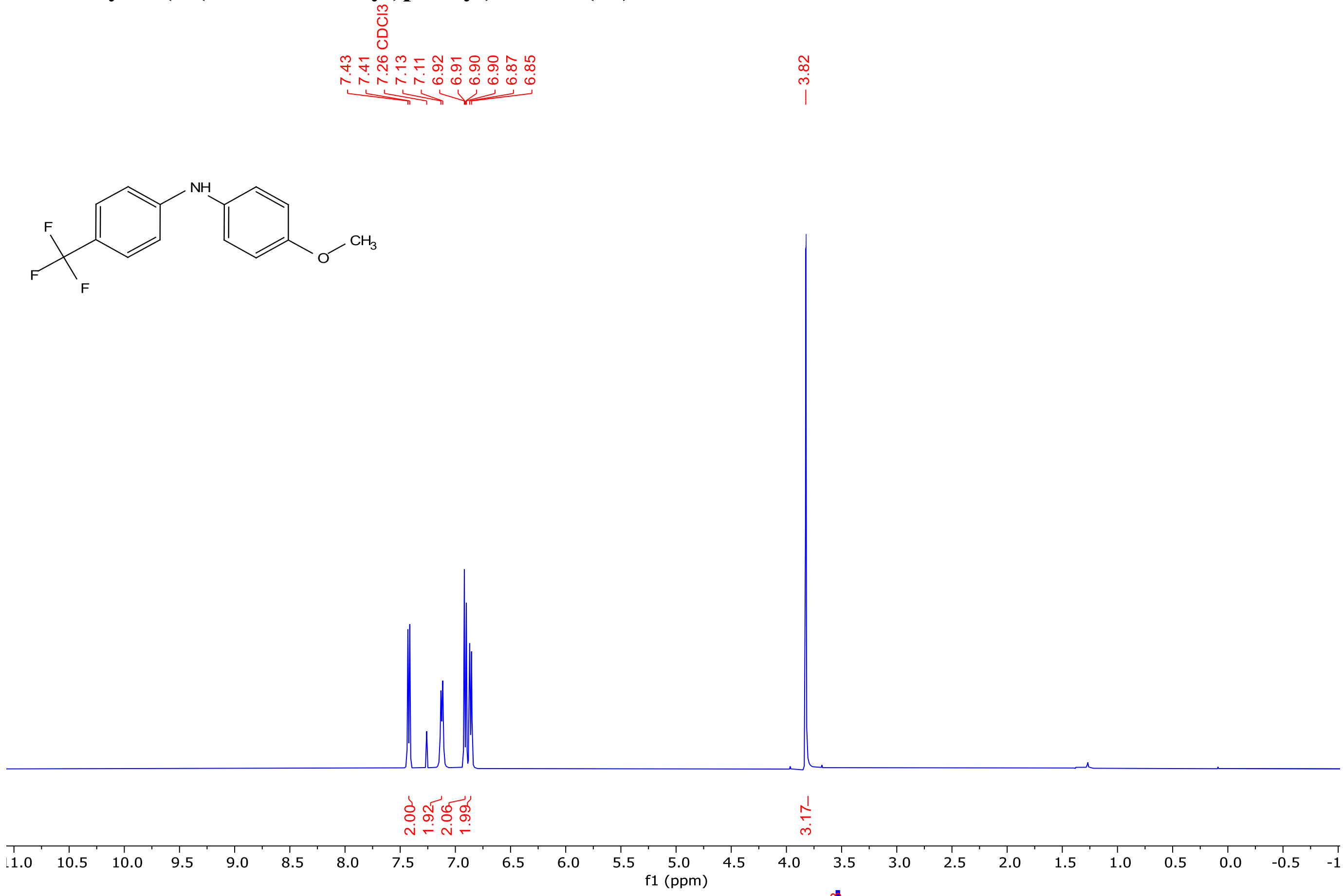

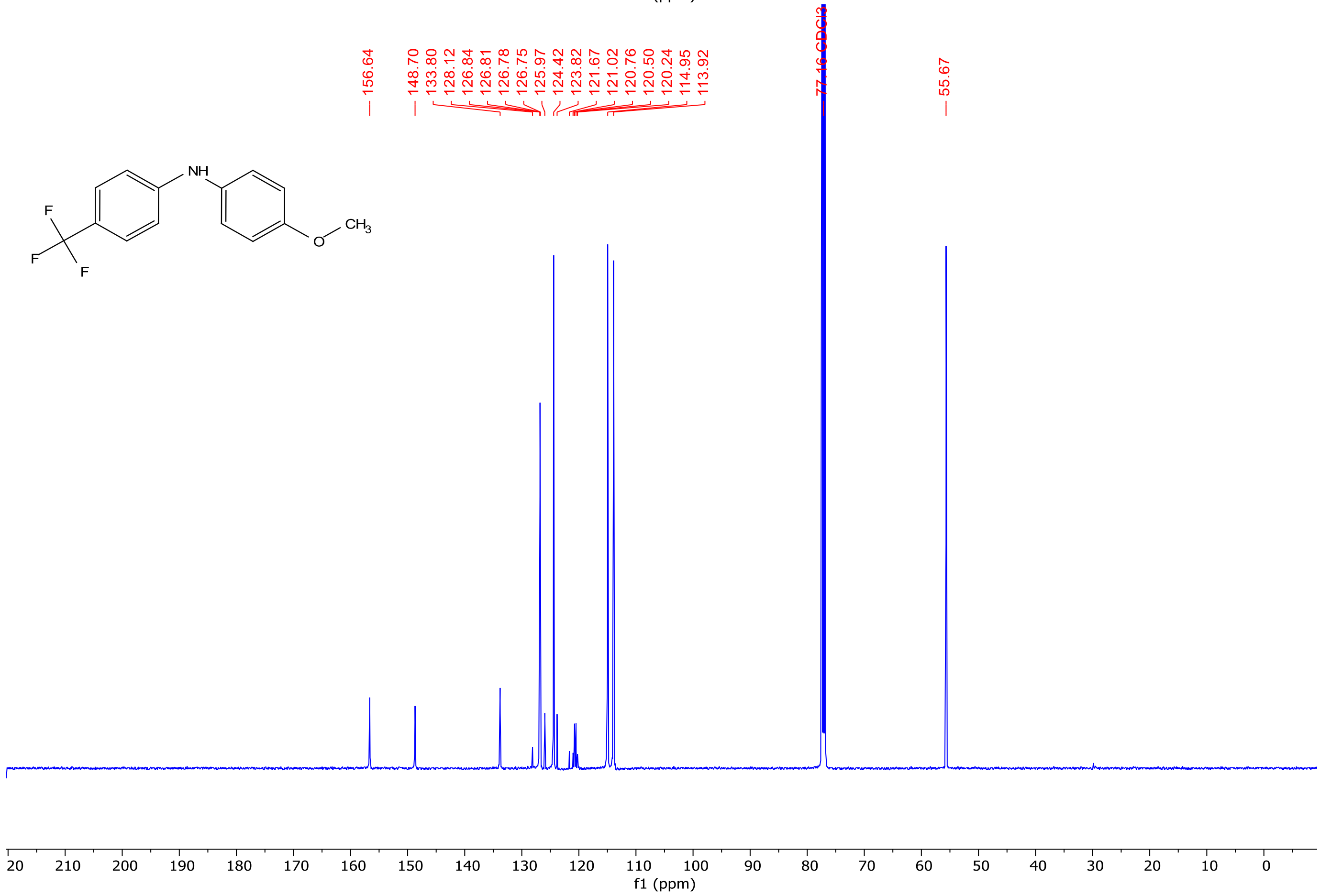

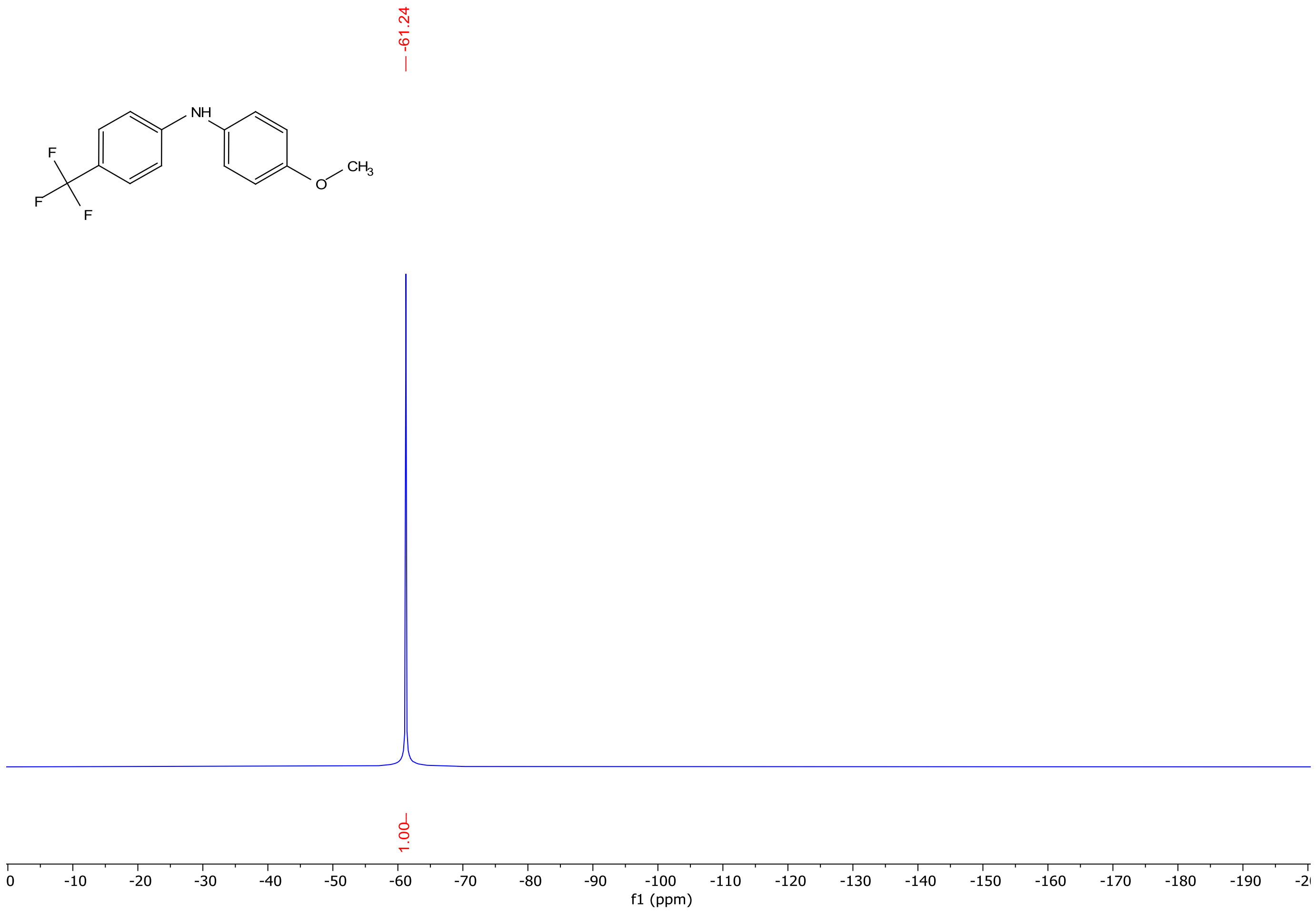
-61.24
NH
F
F
F
O
CH3
1.00
0
-10
-20
-30
-40
-50
-60
-70
-80
-90
-100
-110
-120
-130
-140
-150
-160
-170
-180
-190
f1 (ppm)

N-(4-methoxyphenyl)naphthalen-2-amine (43)

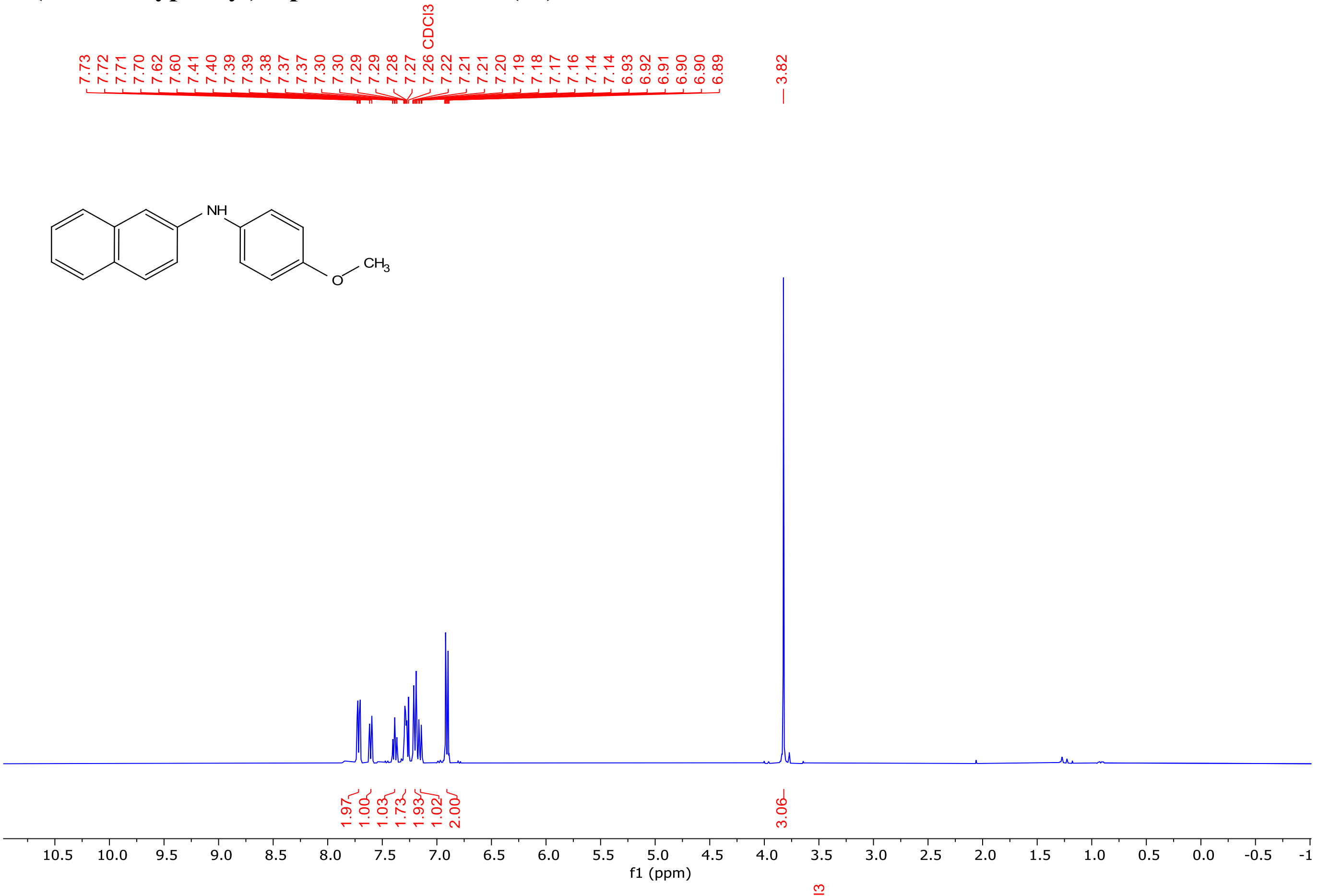

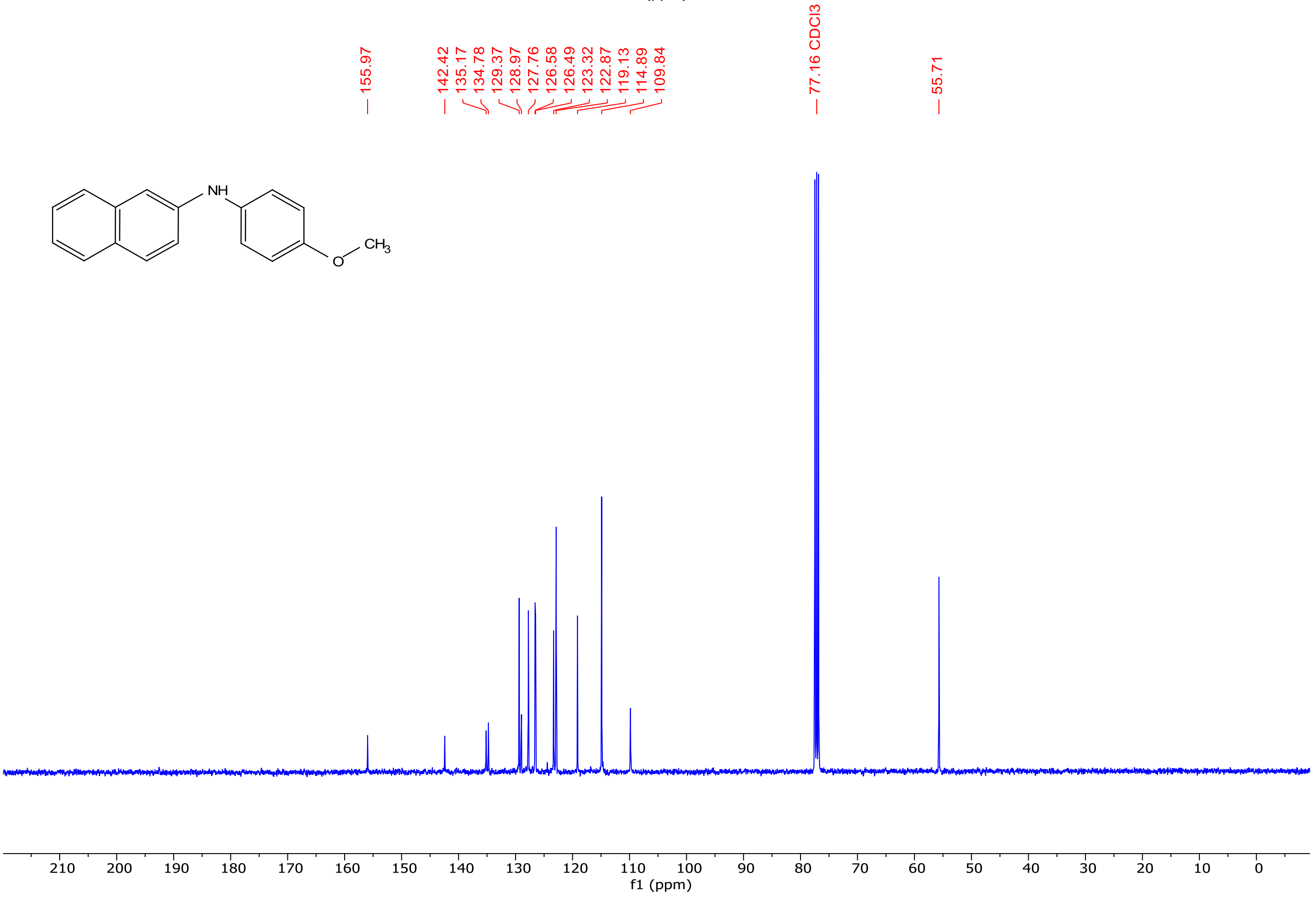

***N*-phenyl-4-(trifluoromethyl)aniline (44)**

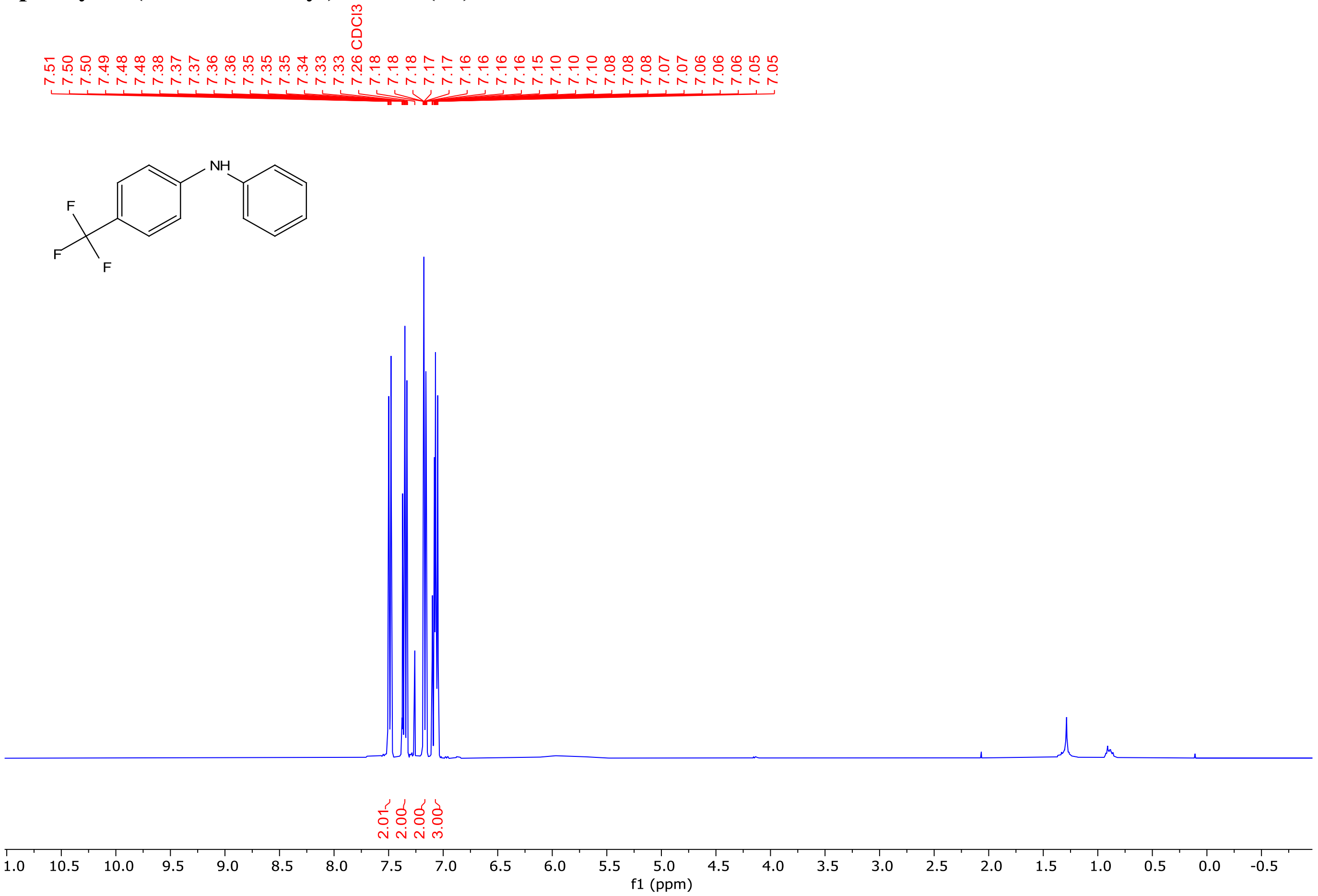

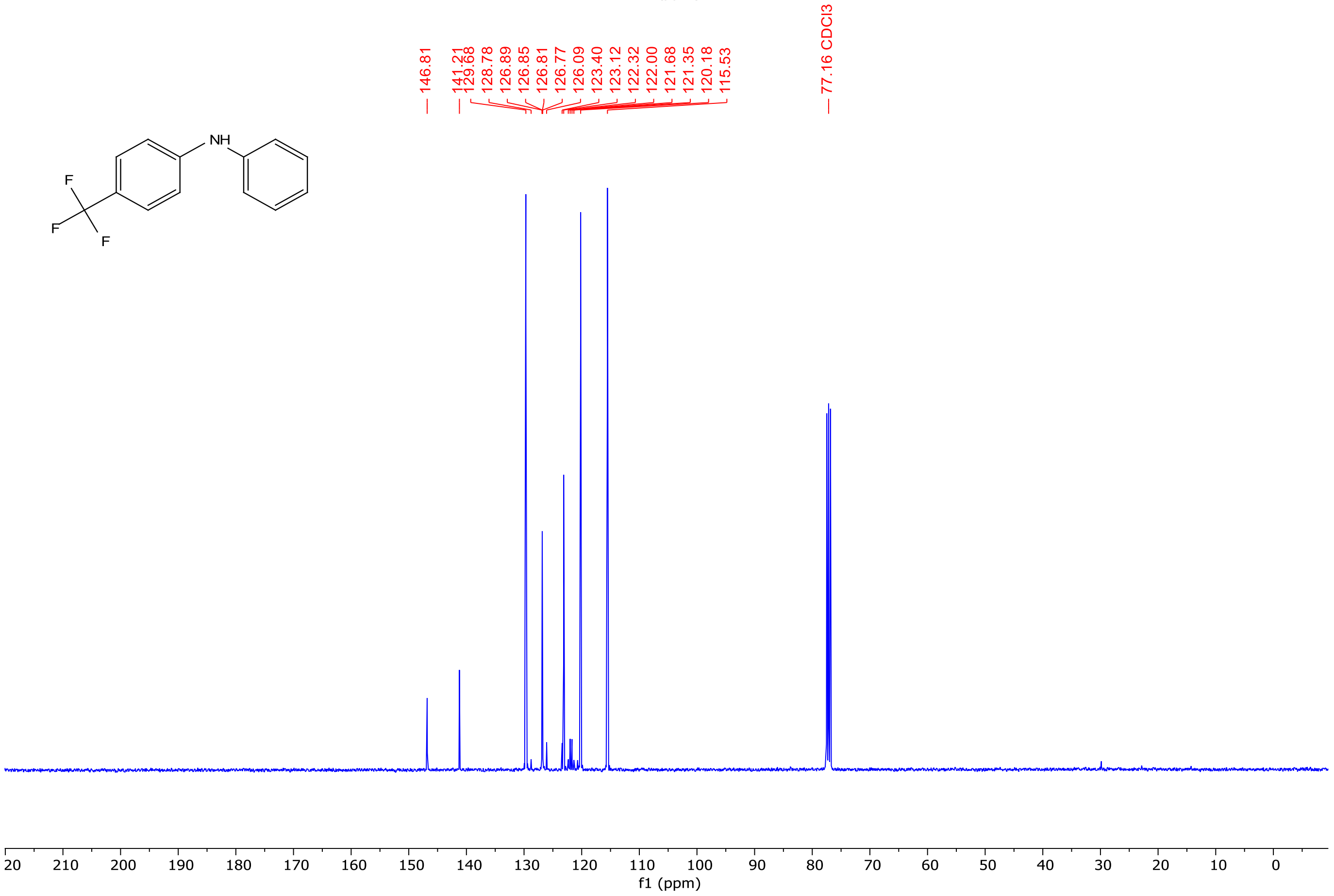

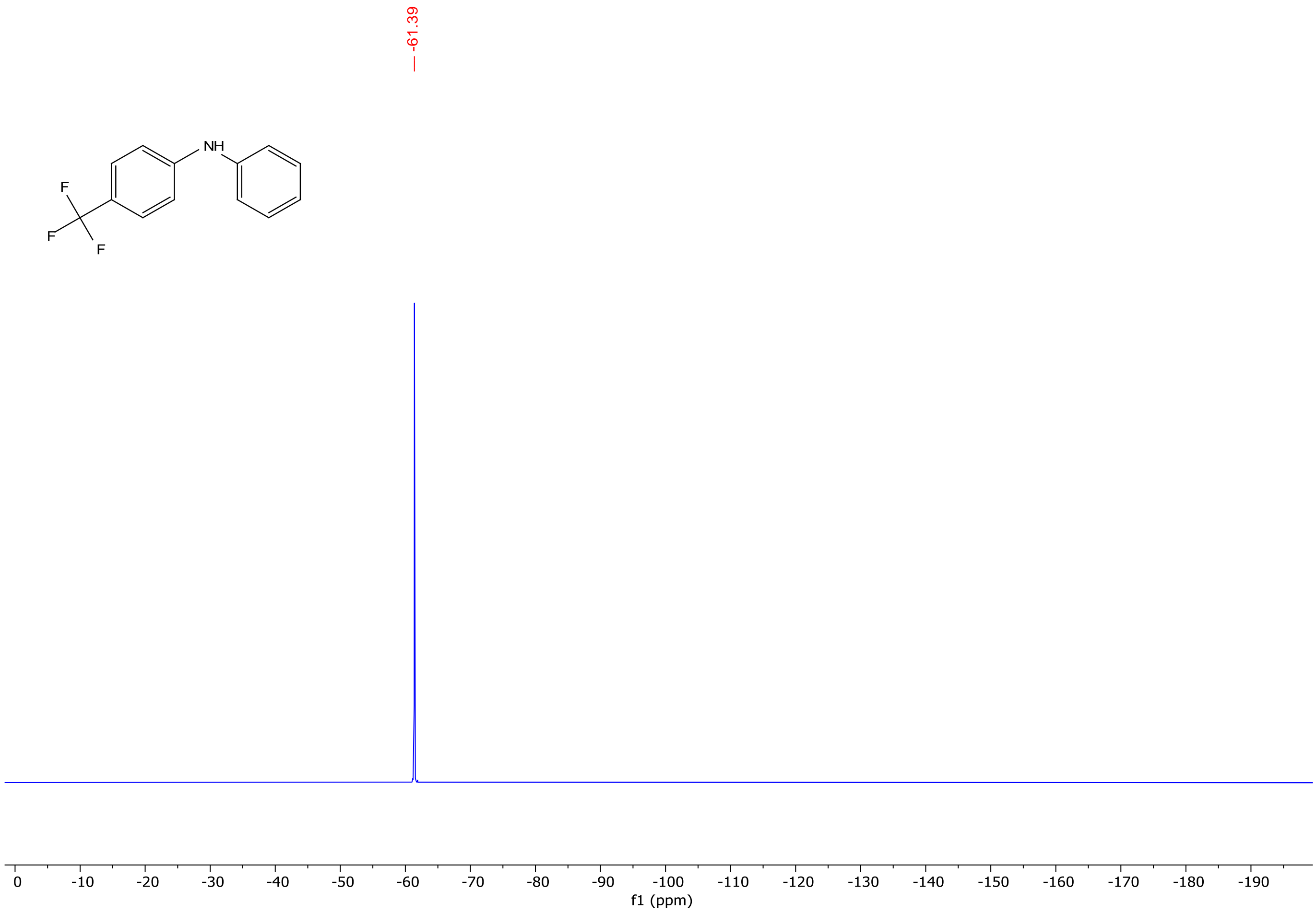
-61.39
NH
F
F
F
0 -10 -20 -30 -40 -50 -60 -70 -80 -90 -100 -110 -120 -130 -140 -150 -160 -170 -180 -190
f1 (ppm)

4-chloro-*N*-(4-(trifluoromethyl)phenyl)aniline (45)

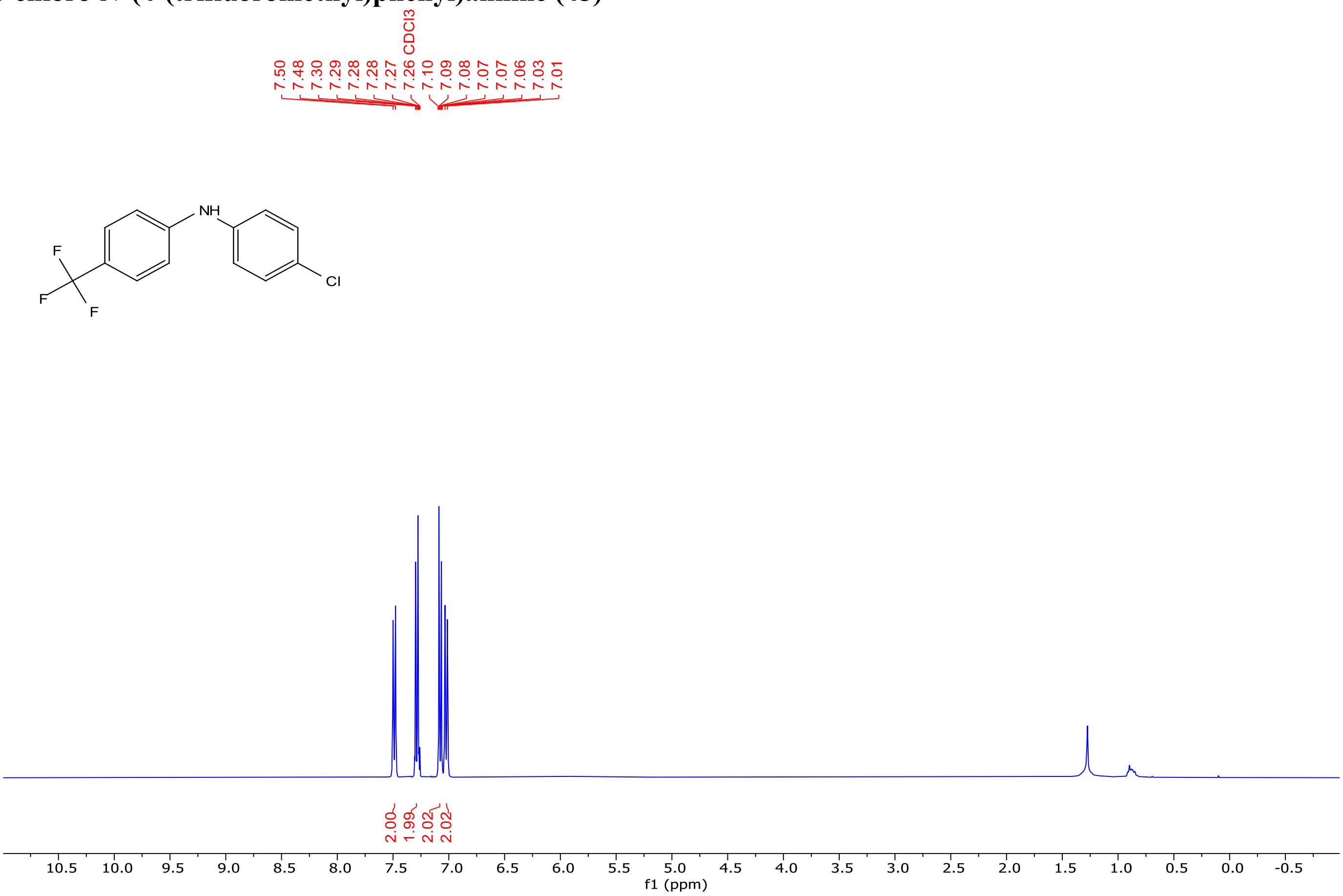

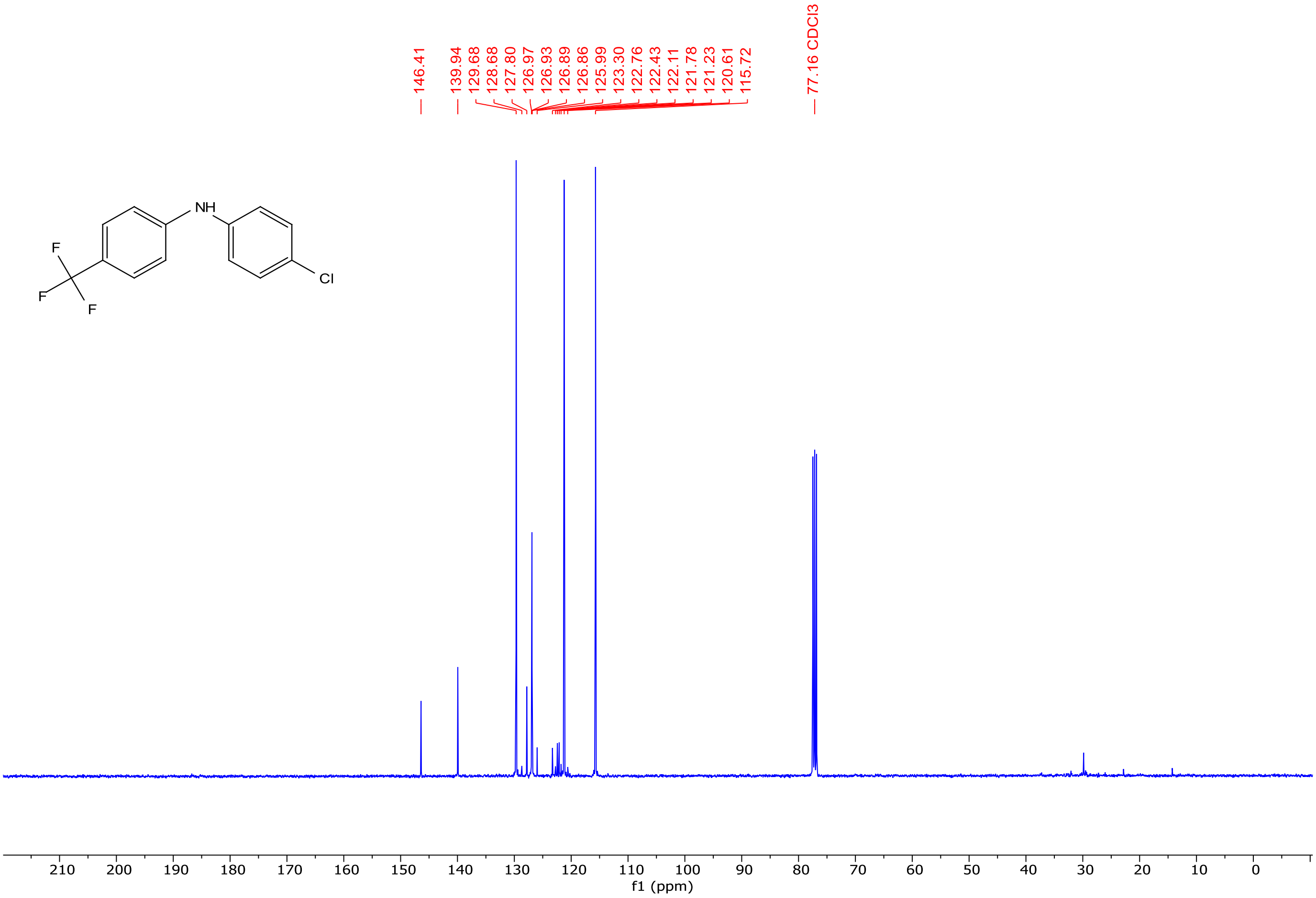

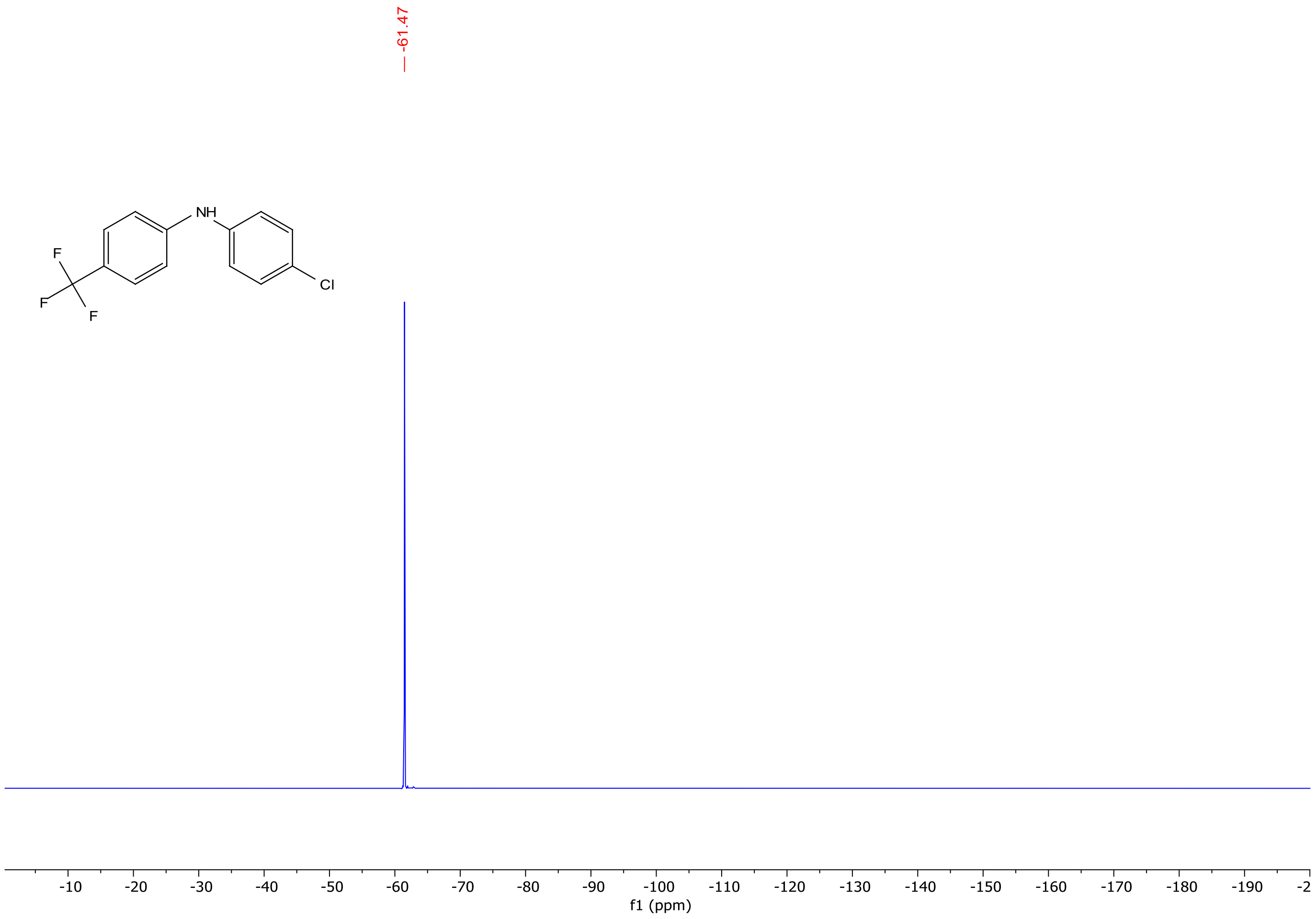
-61.47
NH
F
F
F
Cl
-10 -20 -30 -40 -50 -60 -70 -80 -90 -100 -110 -120 -130 -140 -150 -160 -170 -180 -190 -2
f1 (ppm)

bis(4-(trifluoromethyl)phenyl)amine (46)

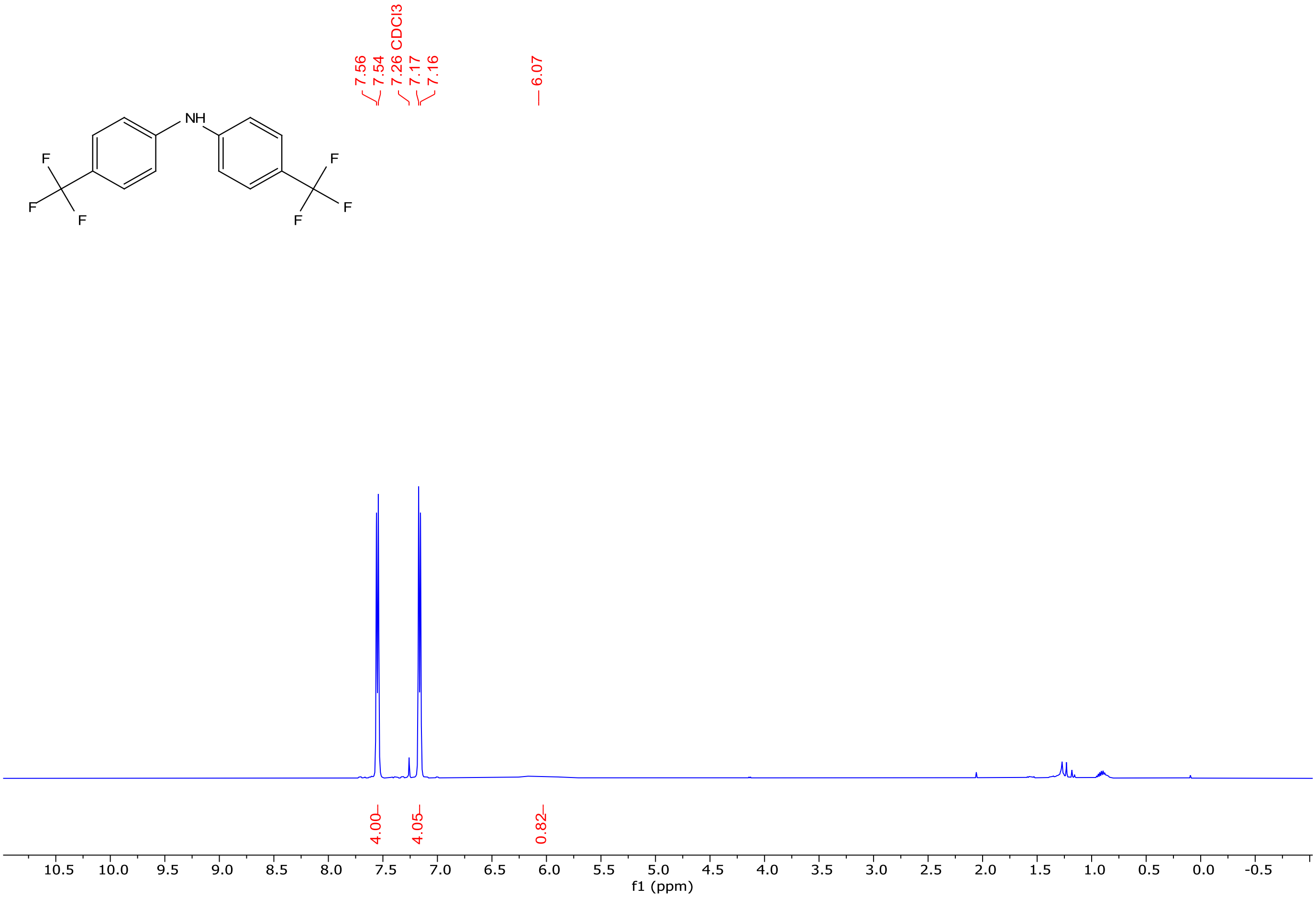

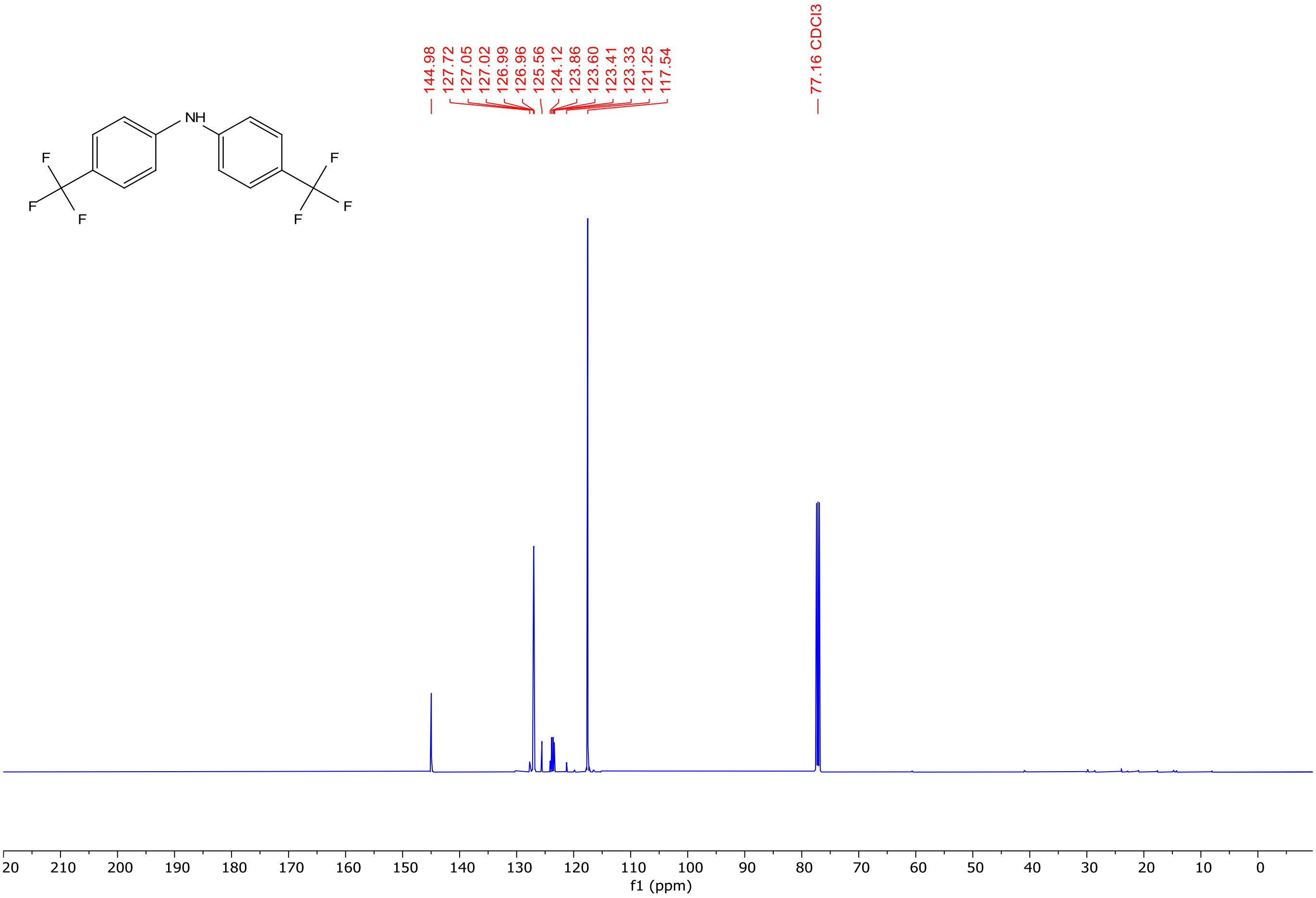

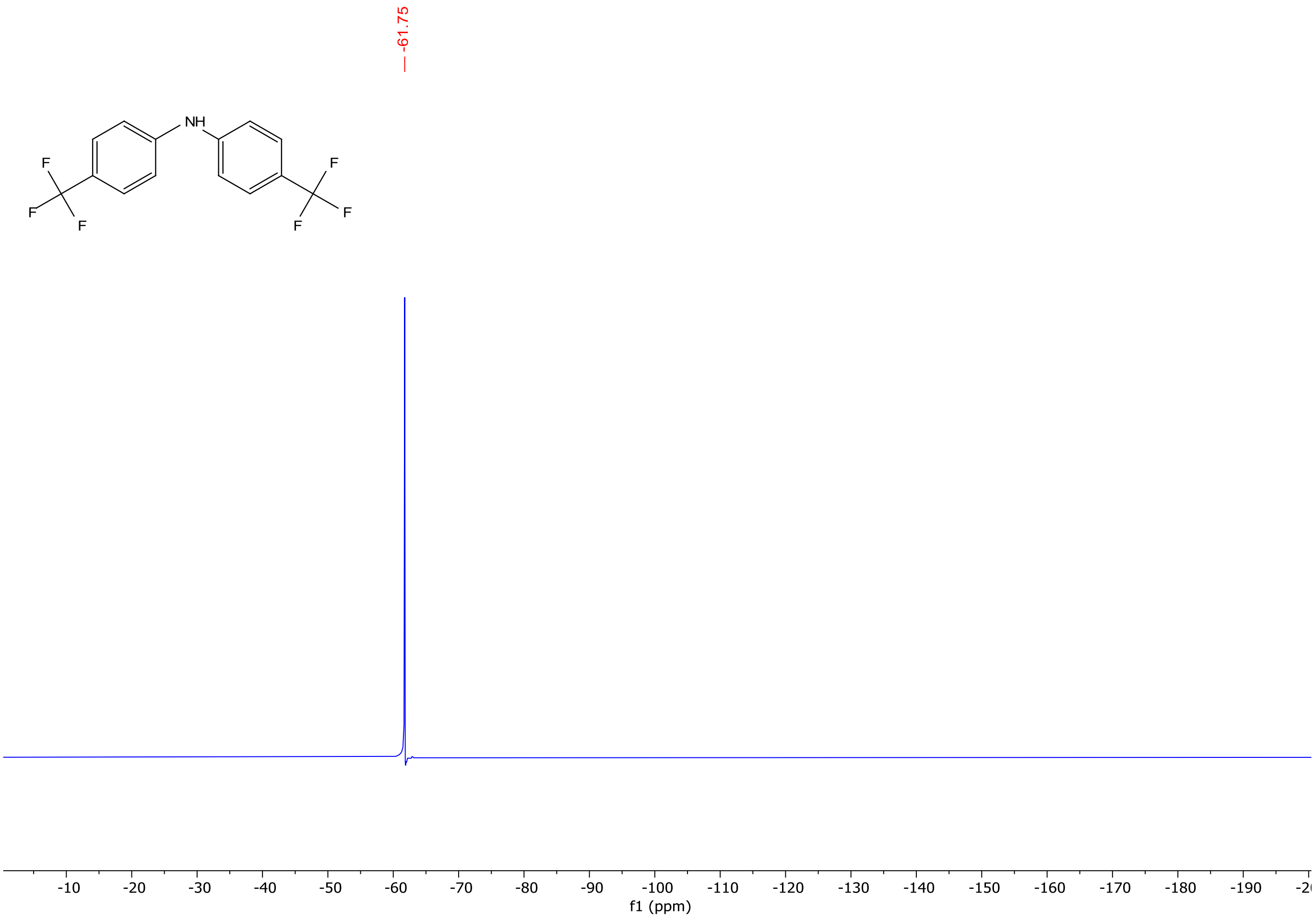
-61.75
NH
F
F
F
F
F
F
-10
-20
-30
-40
-50
-60
-70
-80
-90
-100
-110
-120
-130
-140
-150
-160
-170
-180
-190
f1 (ppm)

1-(3-((4-(trifluoromethyl)phenyl)amino)phenyl)ethan-1-one (47)

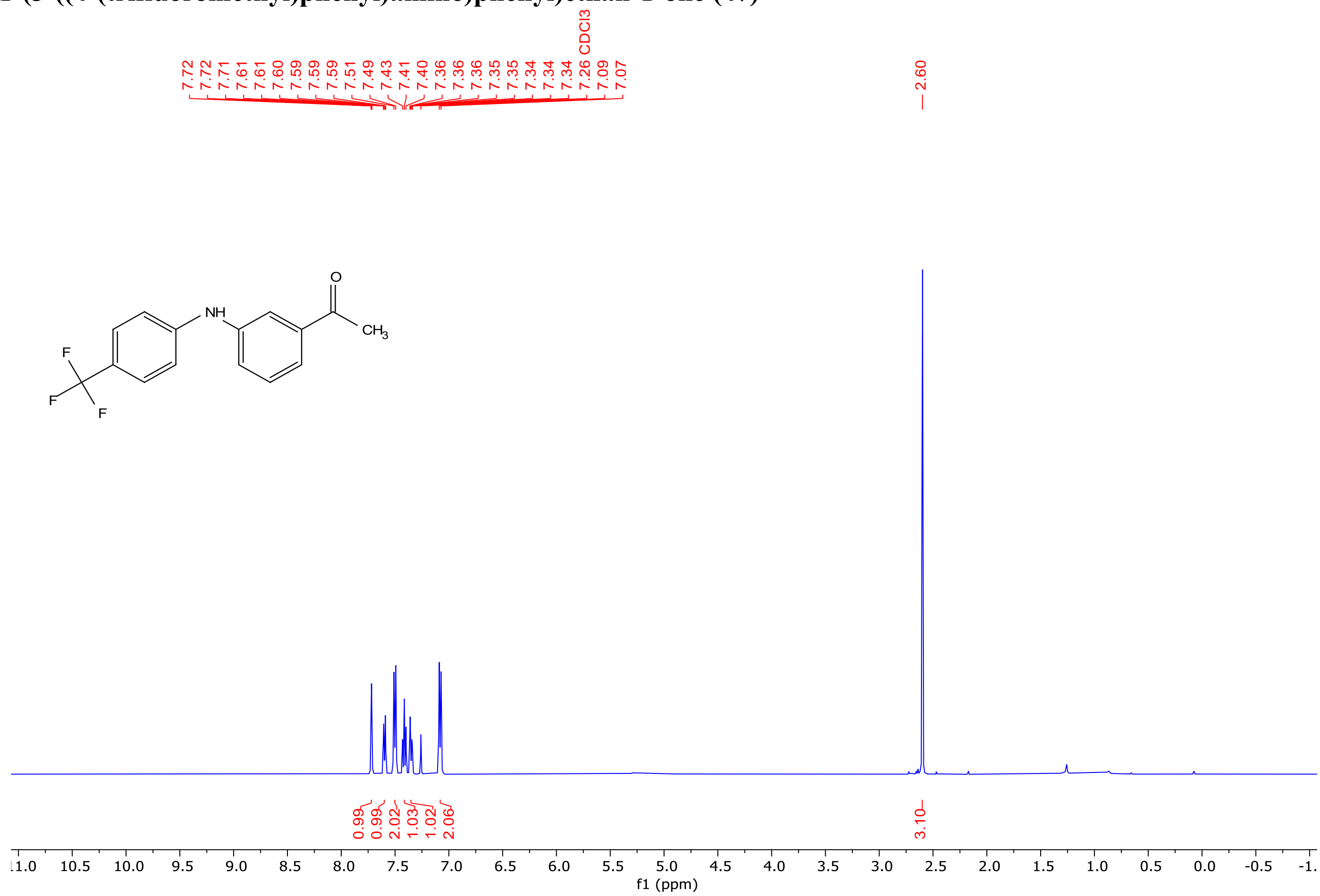

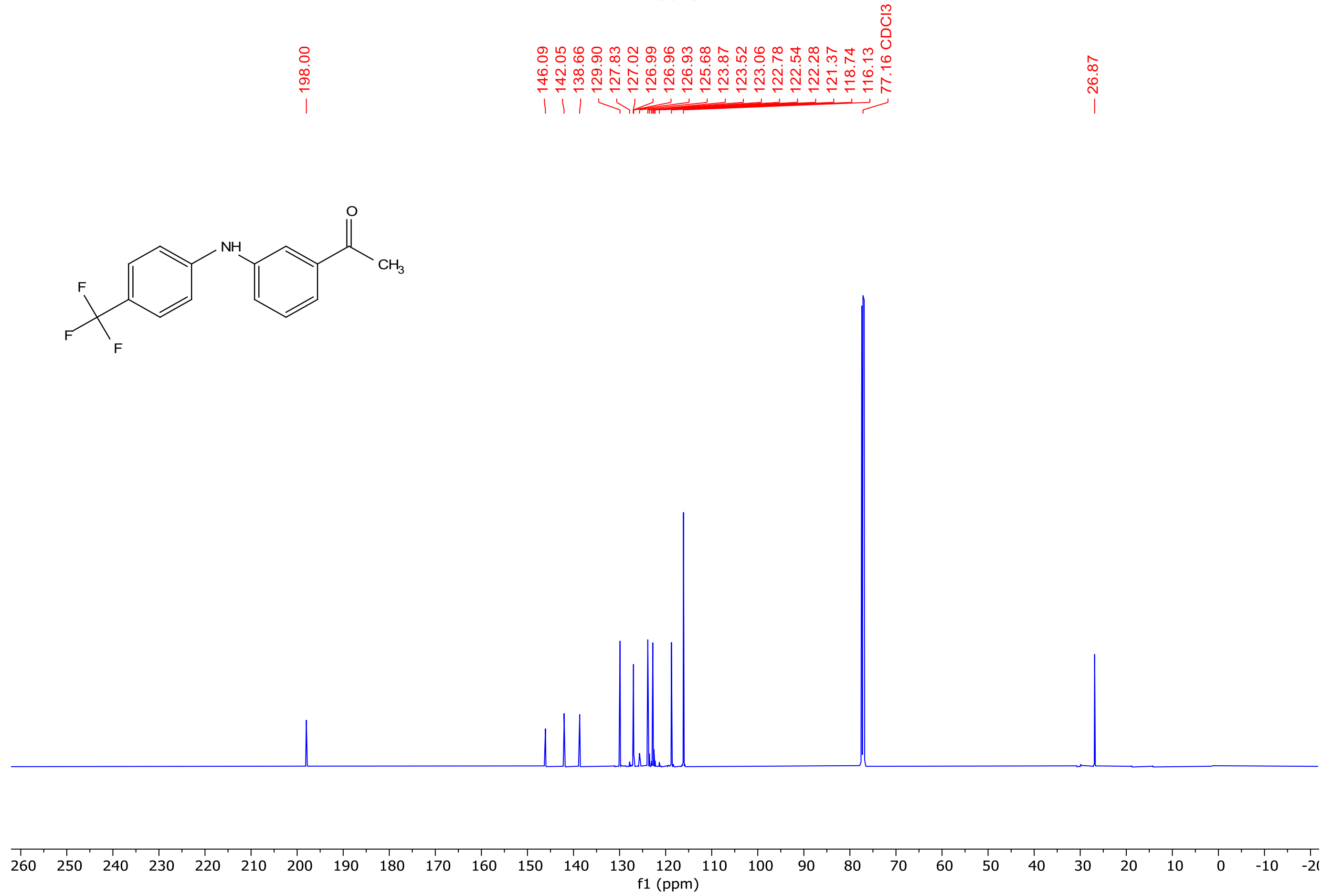

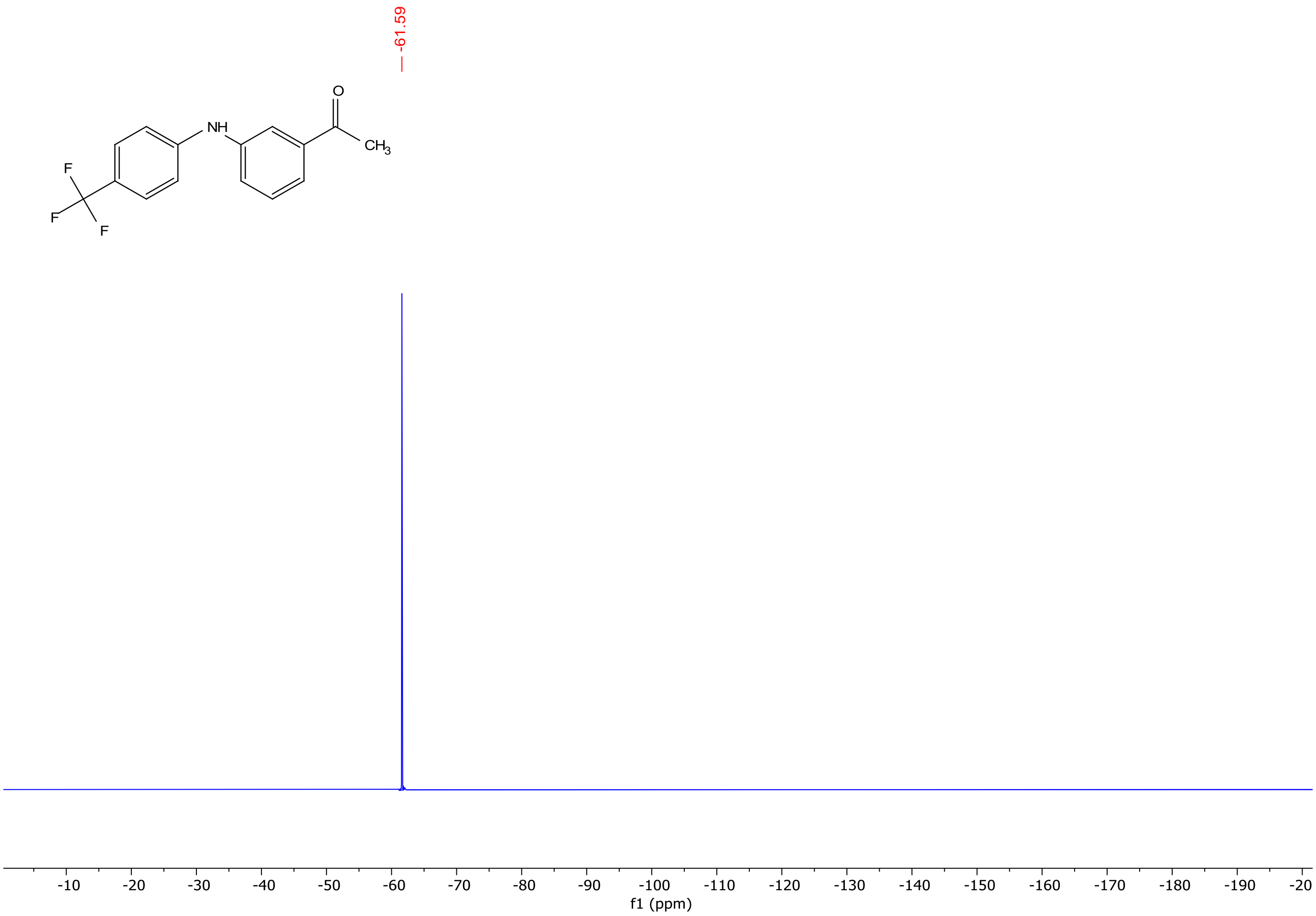
-61.59
O
NH
CH3
F
F
F
-10
-20
-30
-40
-50
-60
-70
-80
-90
-100
-110
-120
-130
-140
-150
-160
-170
-180
-190
-20
f1 (ppm)

N-phenyl-4-(trifluoromethyl)aniline (48)

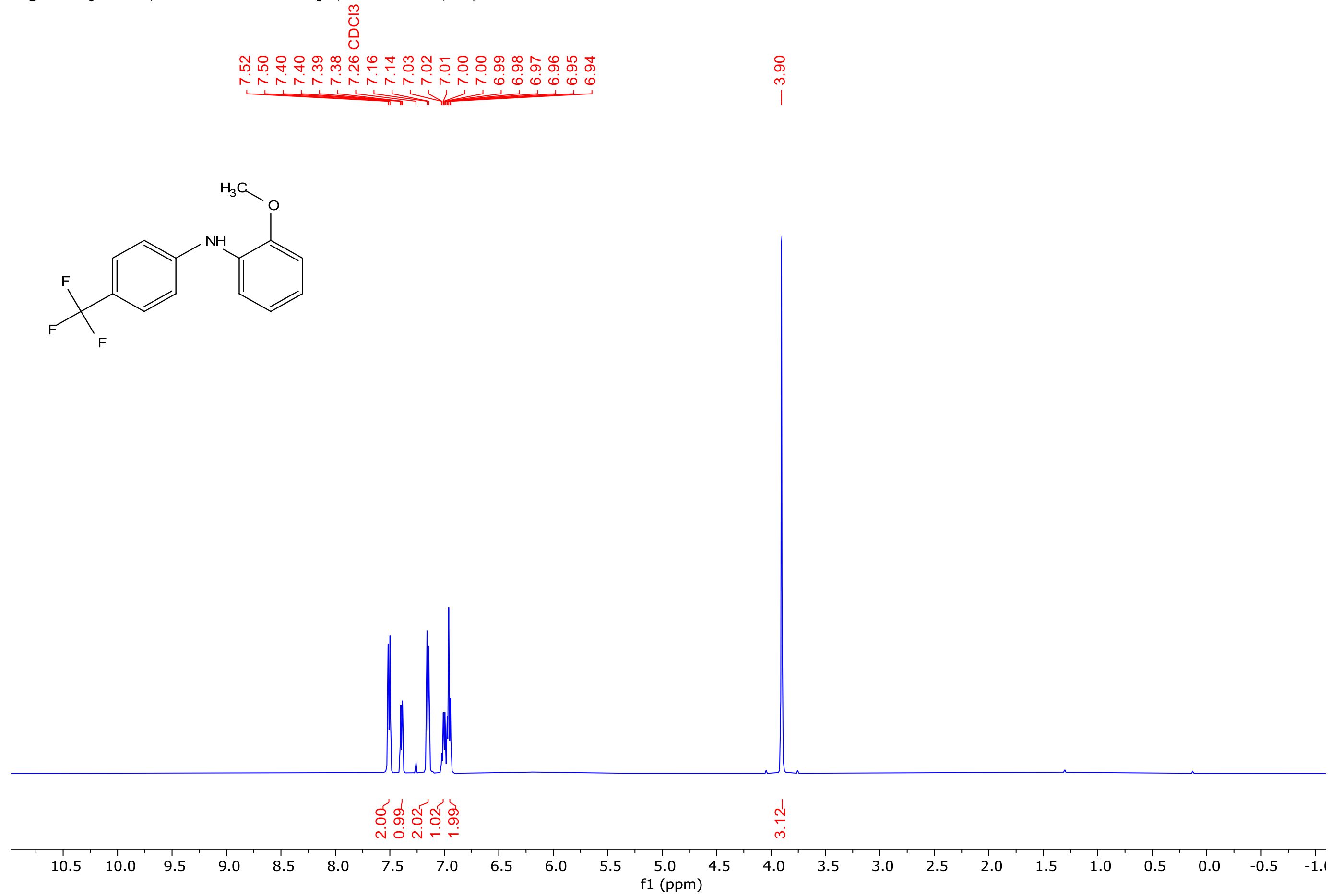

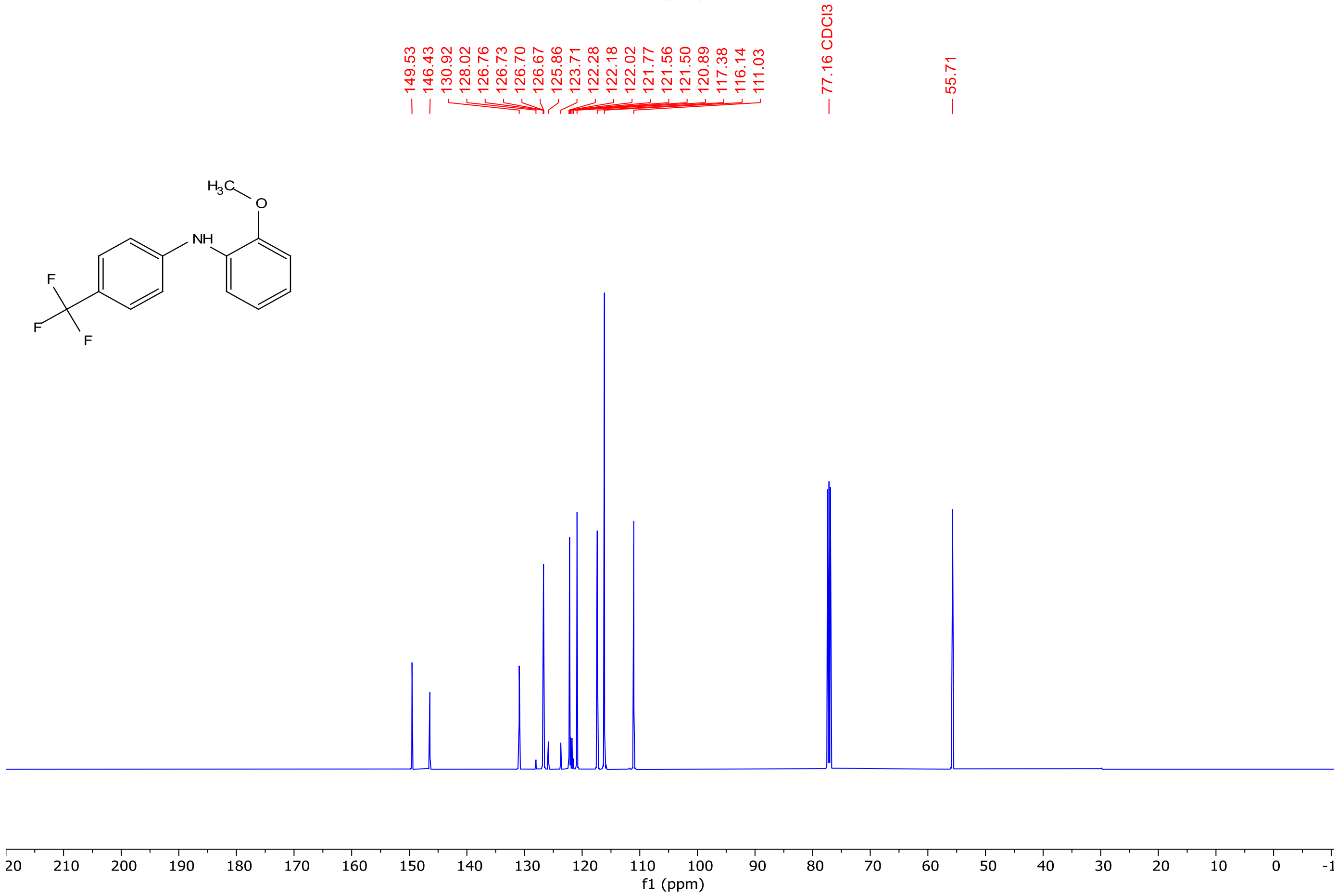

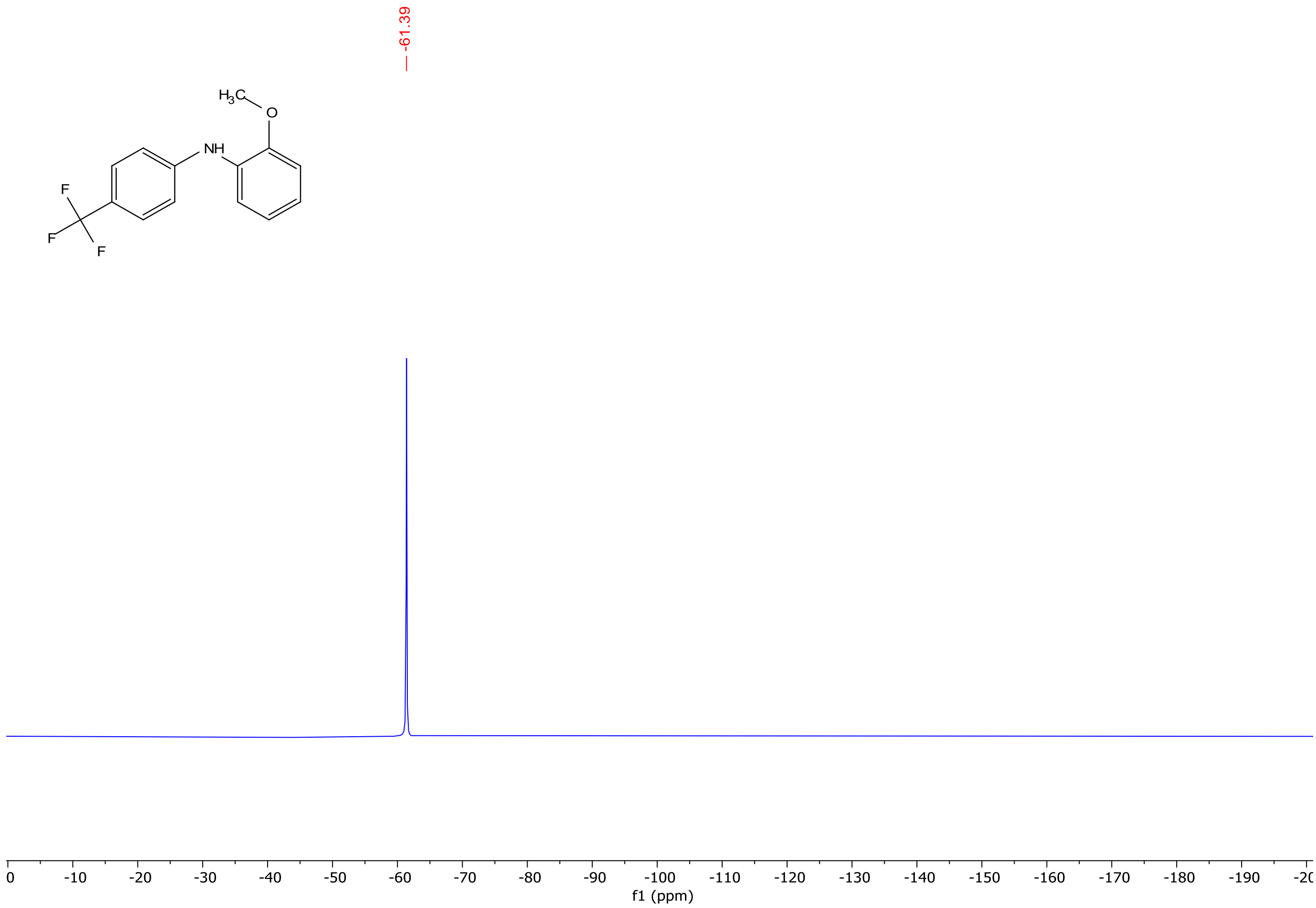

H3C
O
NH
F
F
F
-61.39
0
-10
-20
-30
-40
-50
-60
-70
-80
-90
-100
-110
-120
-130
-140
-150
-160
-170
-180
-190
f1 (ppm)

N-(4-(trifluoromethyl)phenyl)pyridin-3-amine (49)

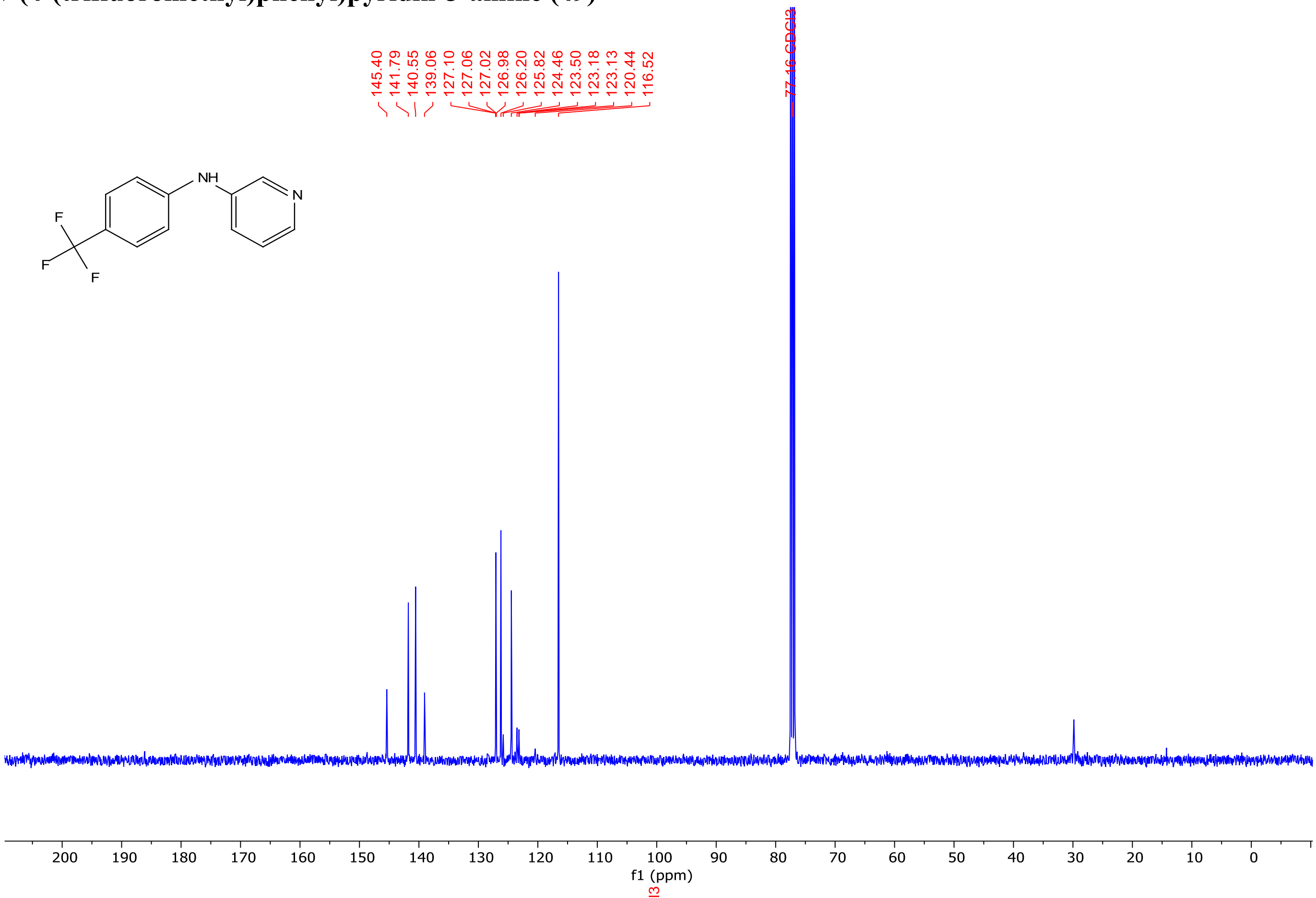

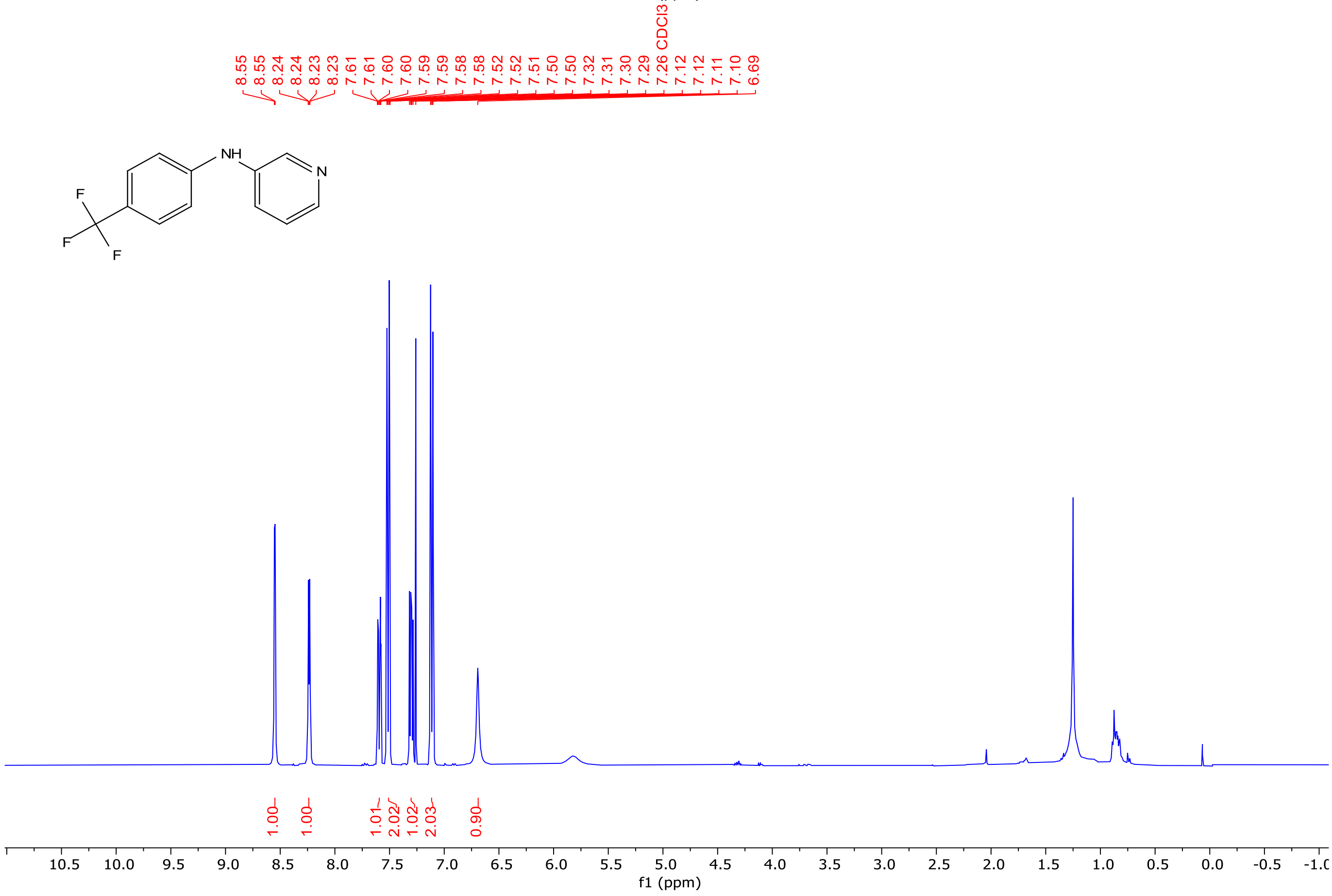

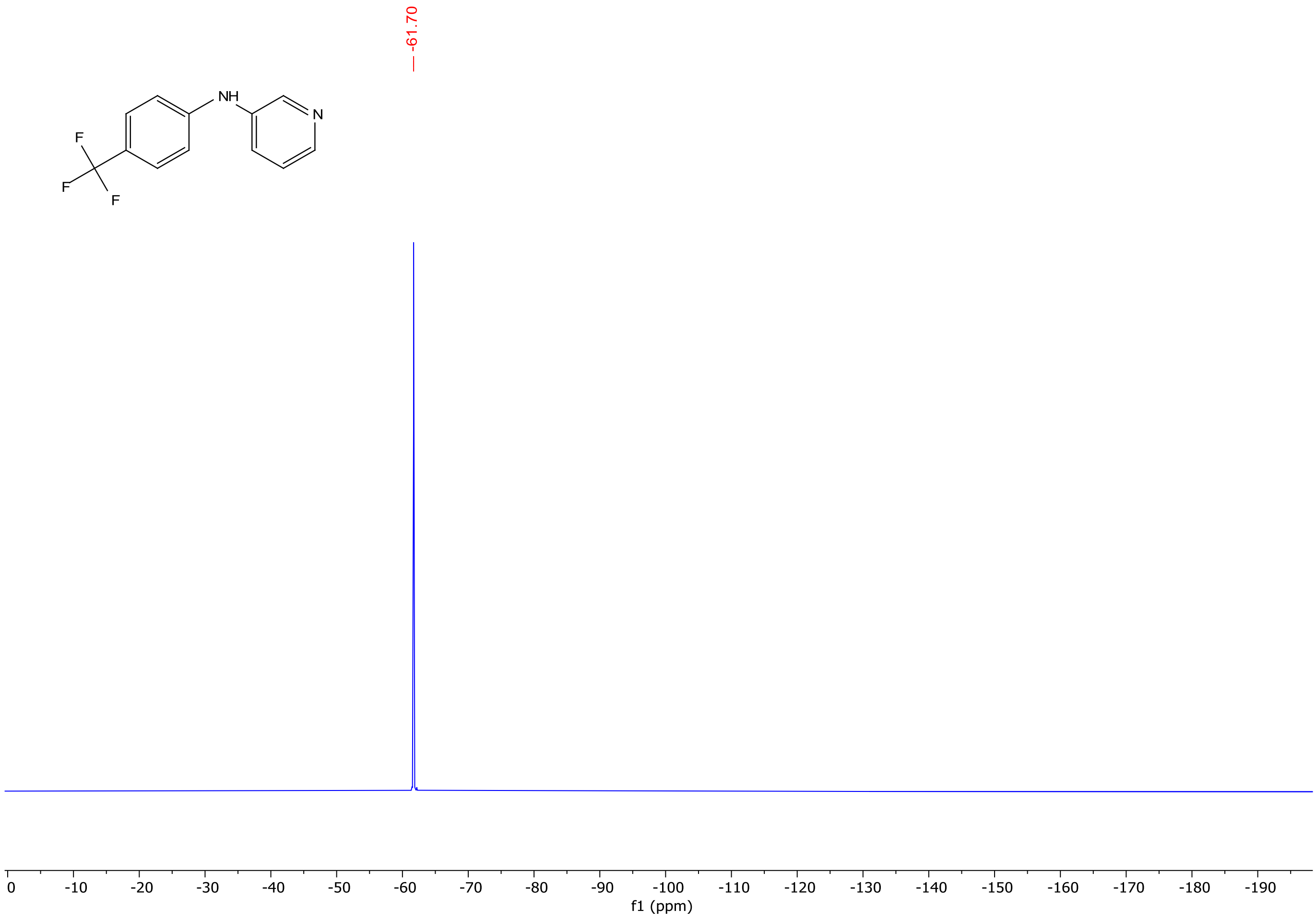
NH
N
F
F
F
-61.70
0
-10
-20
-30
-40
-50
-60
-70
-80
-90
-100
-110
-120
-130
-140
-150
-160
-170
-180
-190
f1 (ppm)

methyl 4-(3-(*tert*-butyl)ureido)benzoate (50)

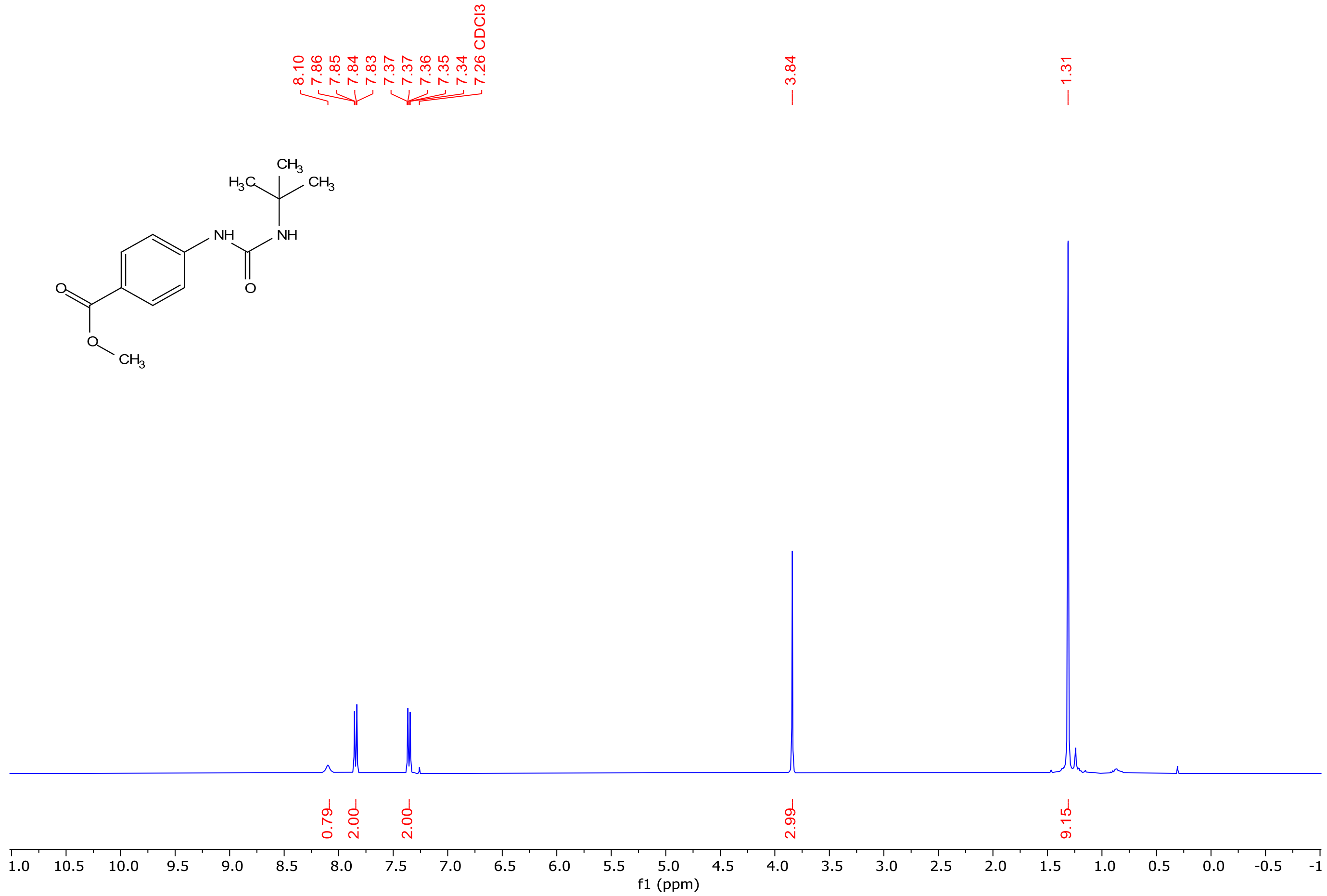

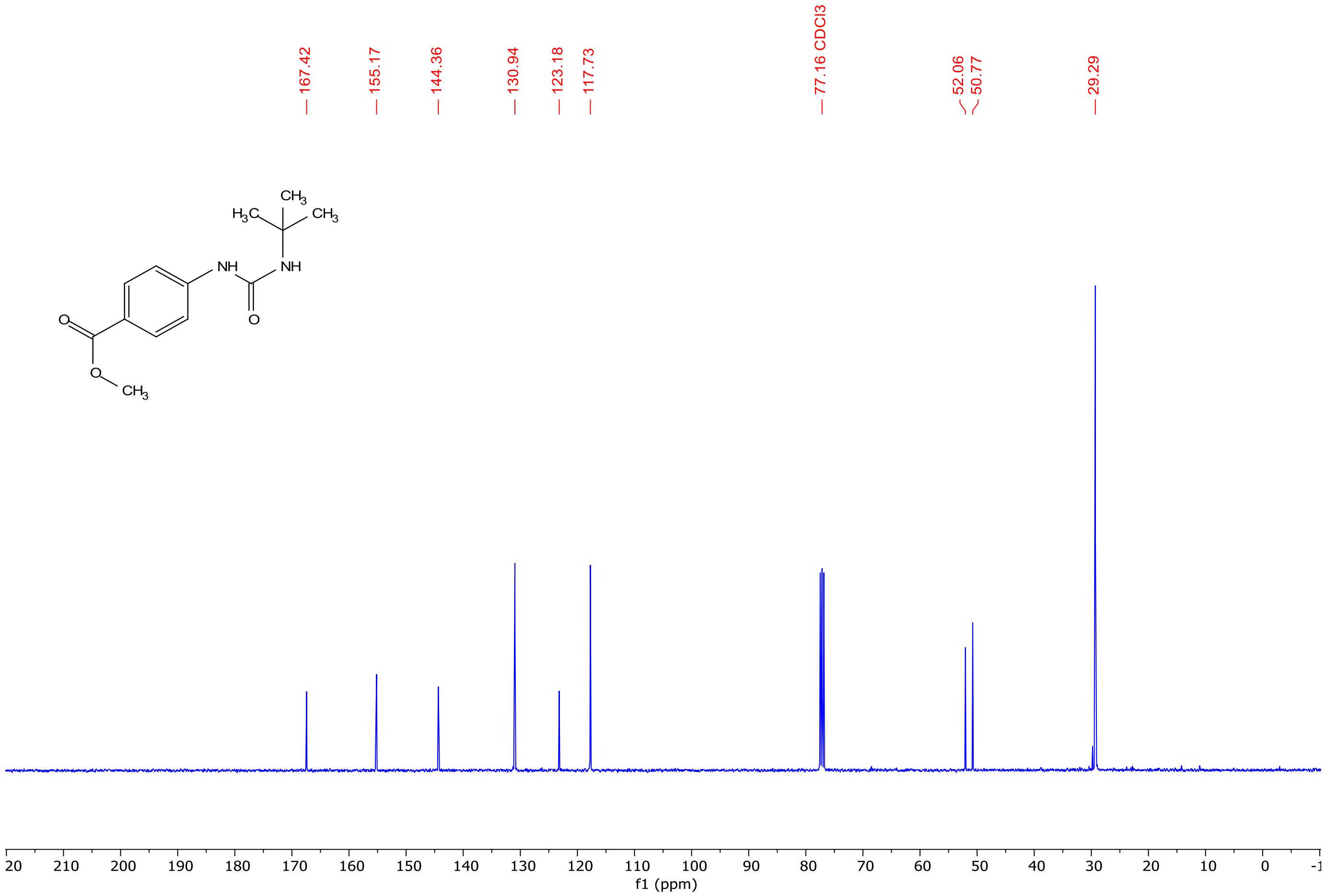

methyl 4-(((benzyloxy)carbonyl)amino)benzoate (51)

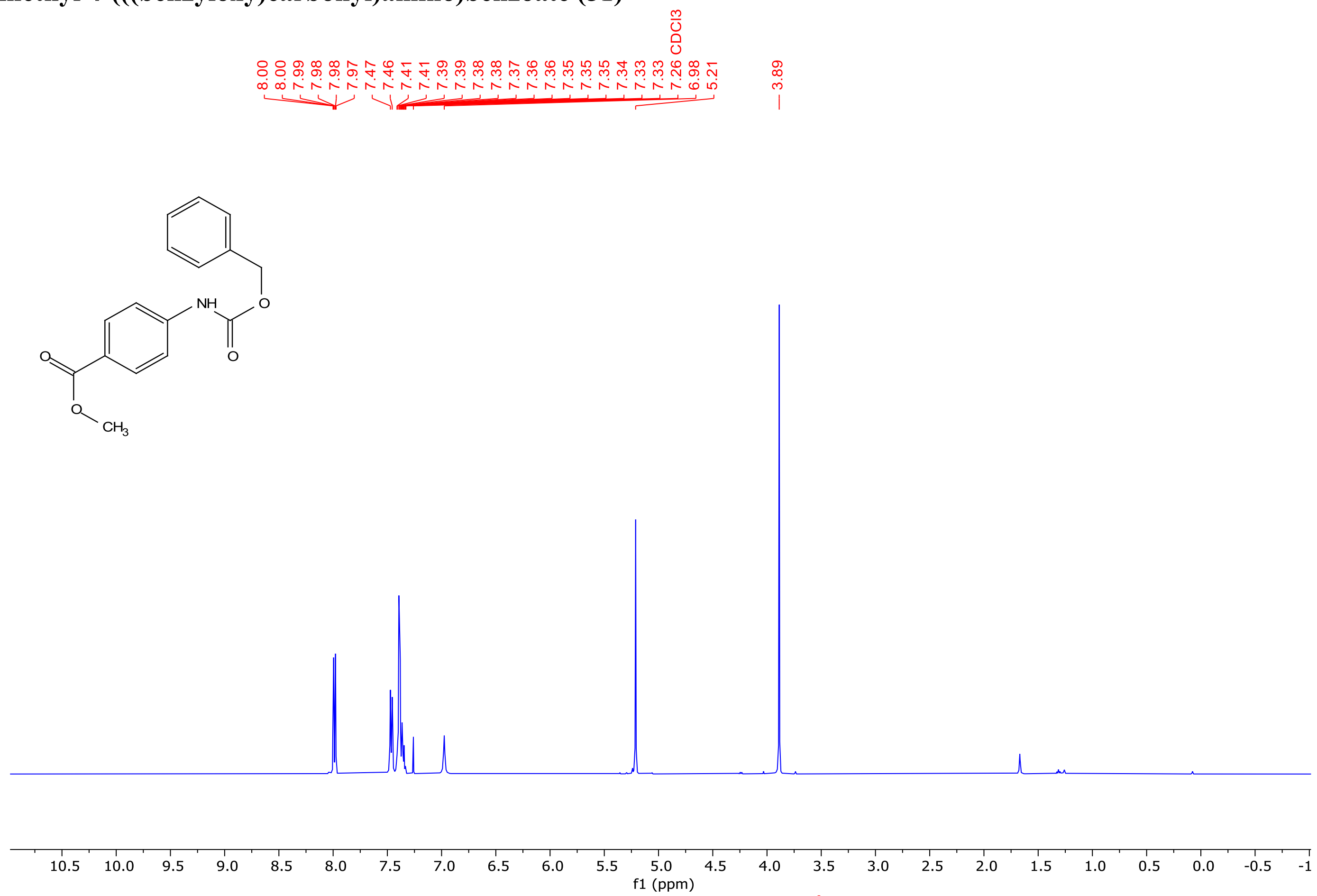

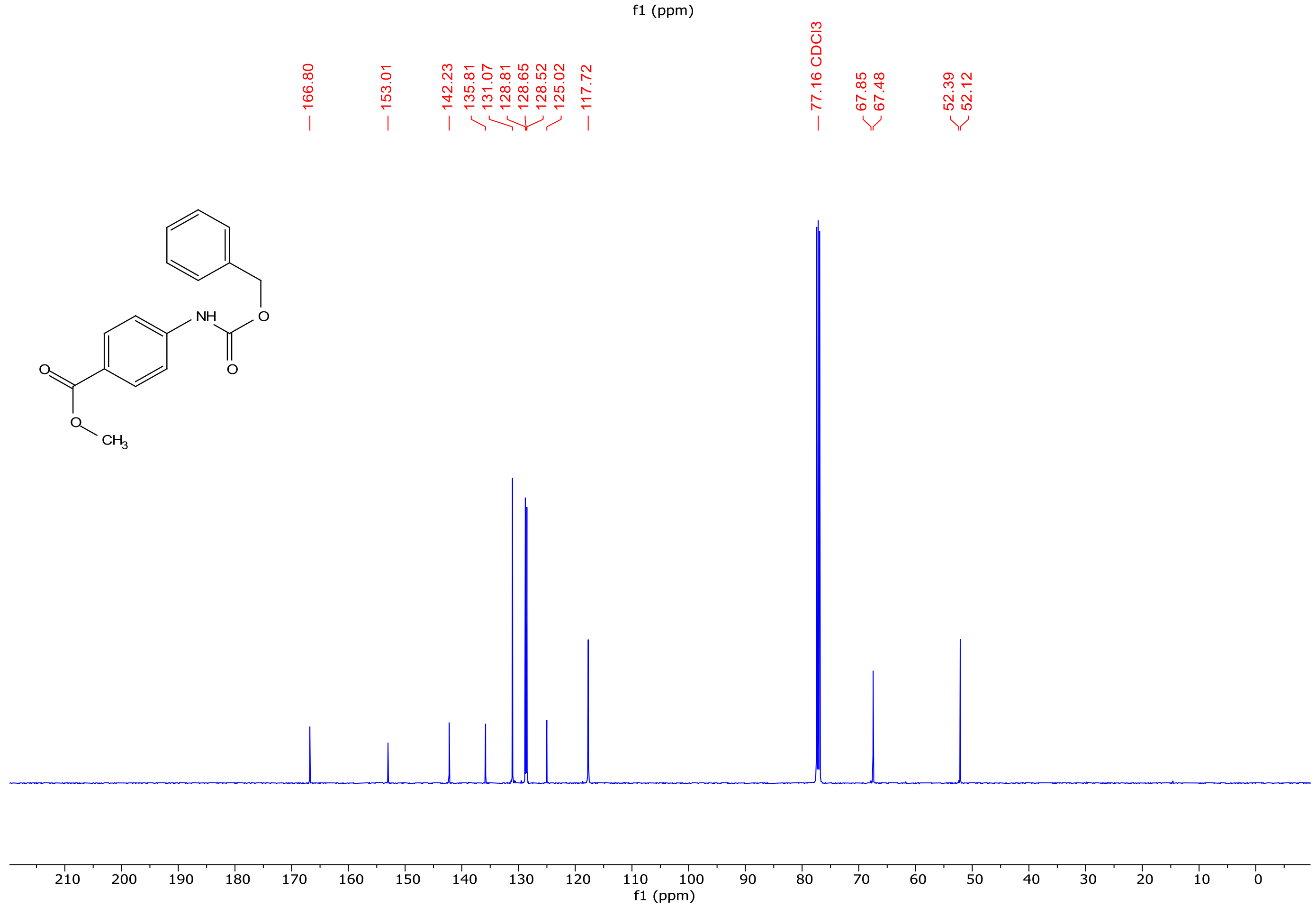

methyl 4-((oxodiphenyl-l6-sulfaneylidene)amino)benzoate (52)

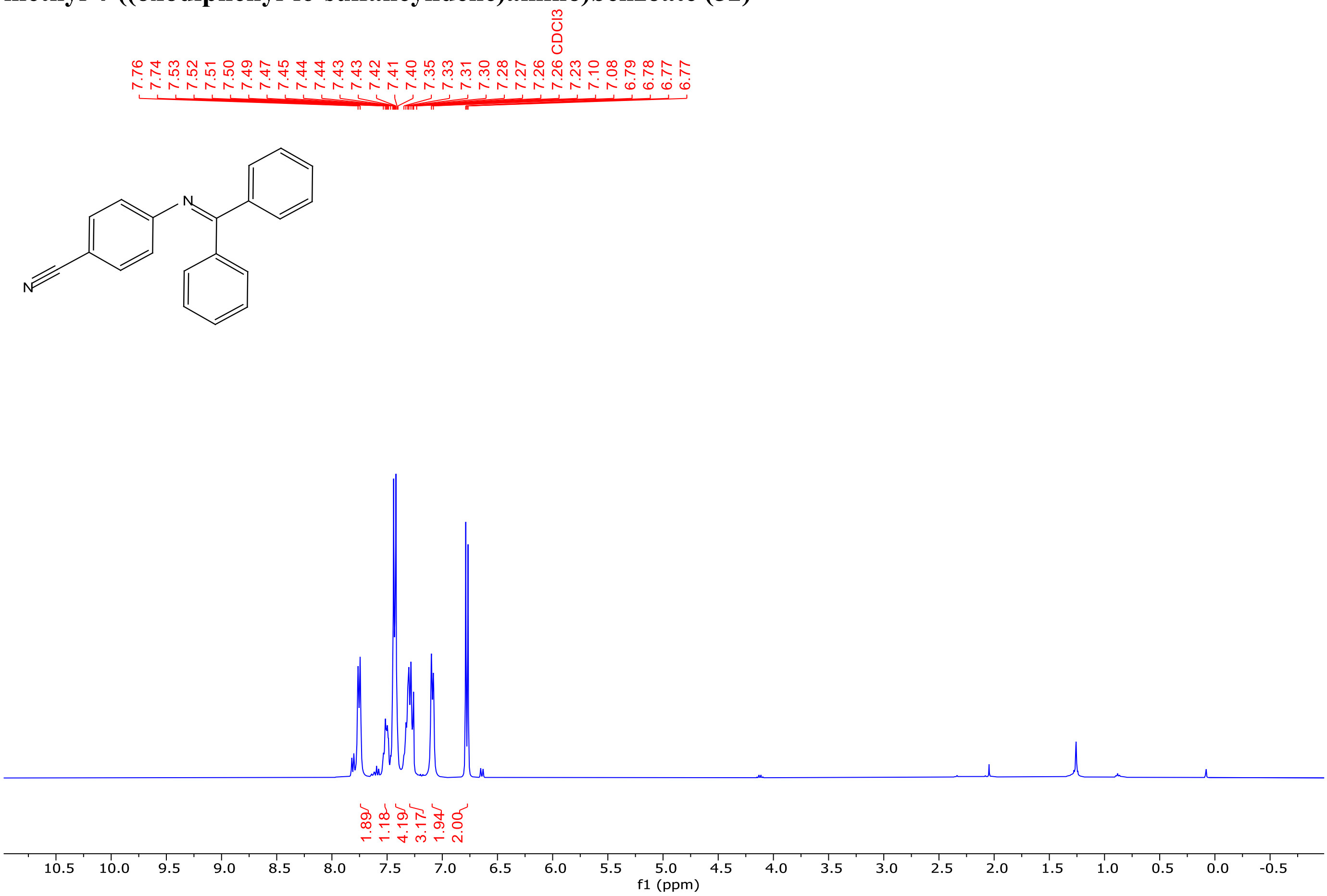

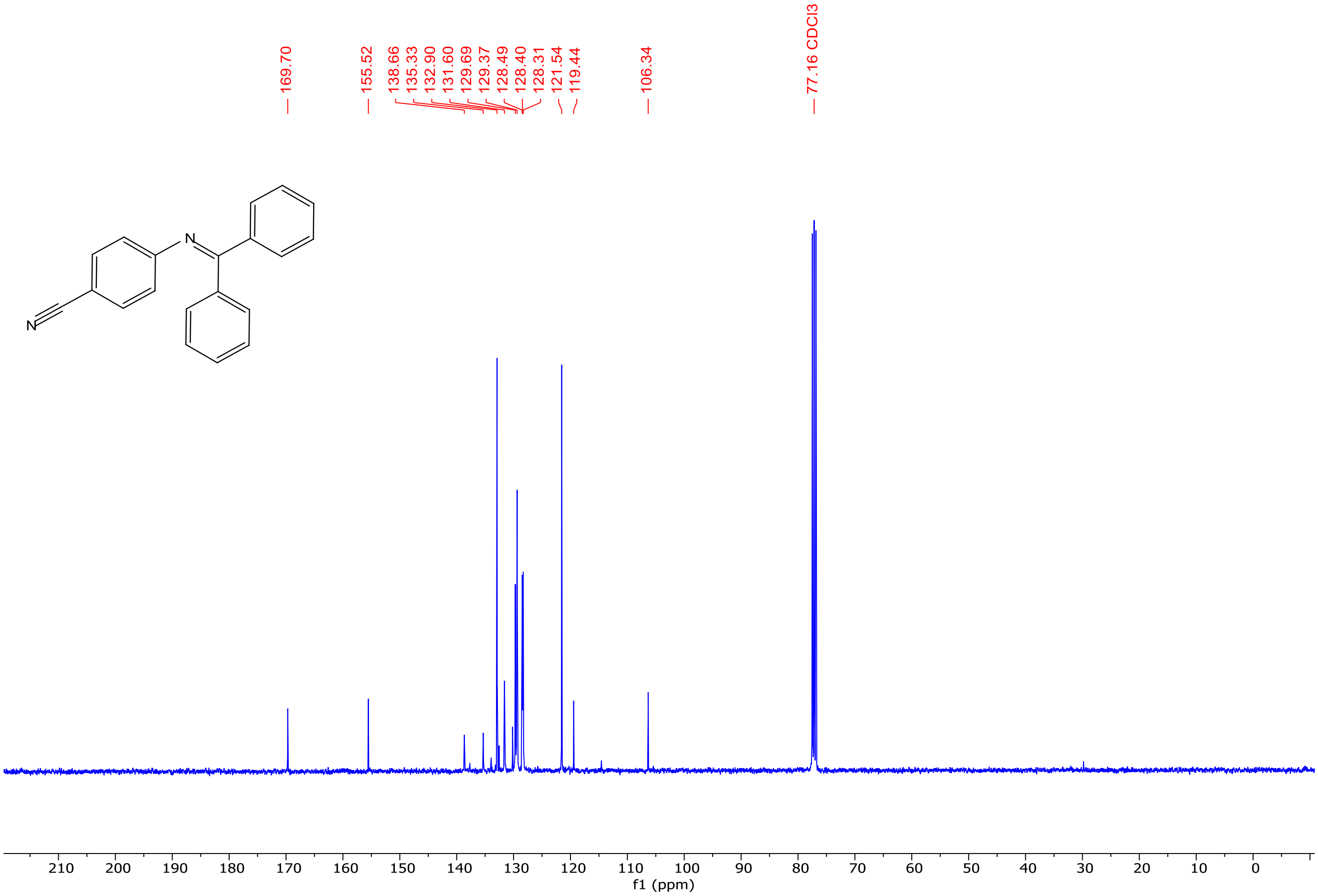

(53)

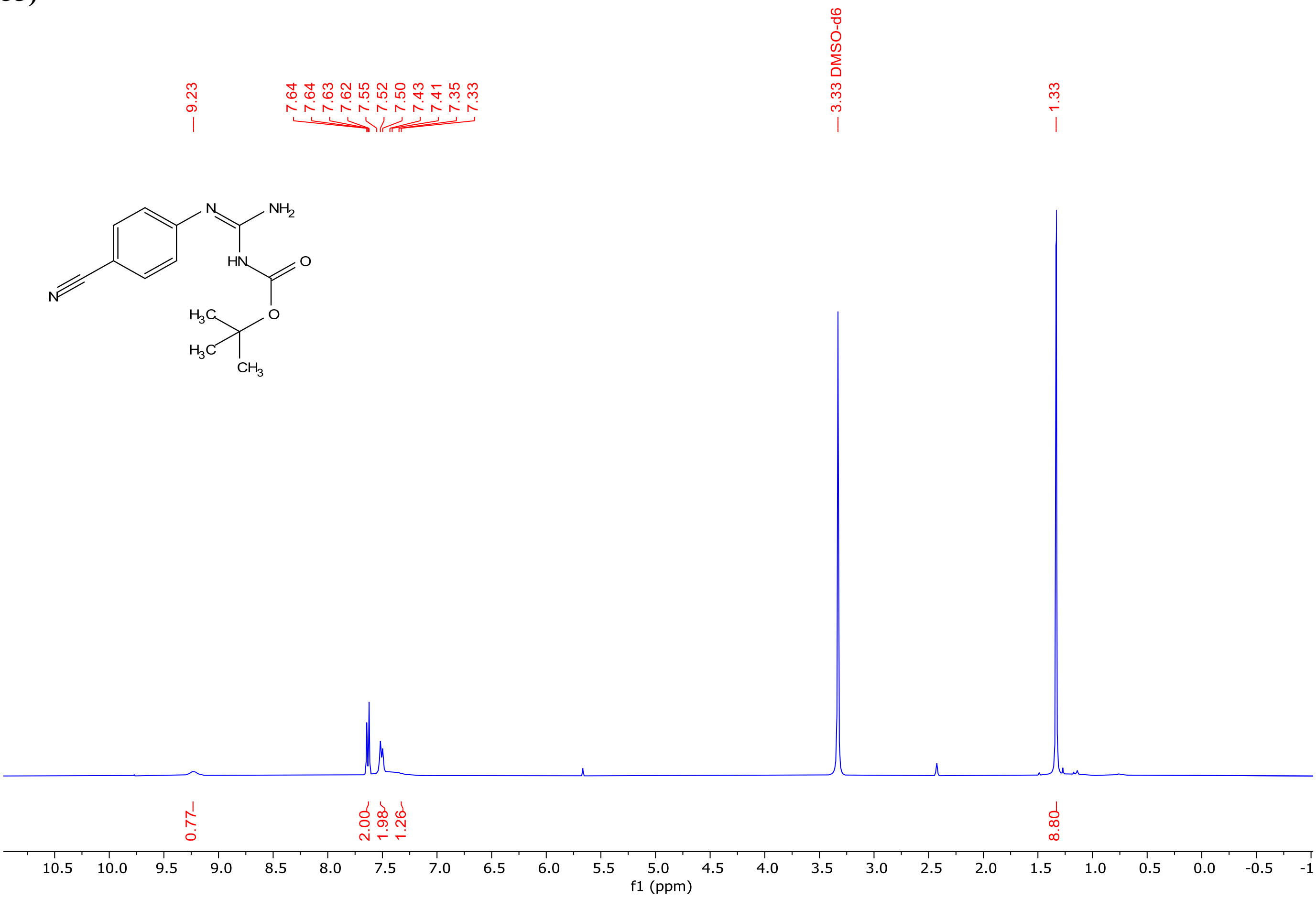
9.23
7.64
7.64
7.63
7.62
7.55
7.52
7.50
7.43
7.41
7.35
7.33
3.33 DMSO-d6
1.33
NH2
N
HN
O
O
N
H3C
H3C
CH3
0.77
2.00
1.98
1.26
8.80
10.5 10.0 9.5 9.0 8.5 8.0 7.5 7.0 6.5 6.0 5.5 5.0 4.5 4.0 3.5 3.0 2.5 2.0 1.5 1.0 0.5 0.0 -0.5 -1
f1 (ppm)

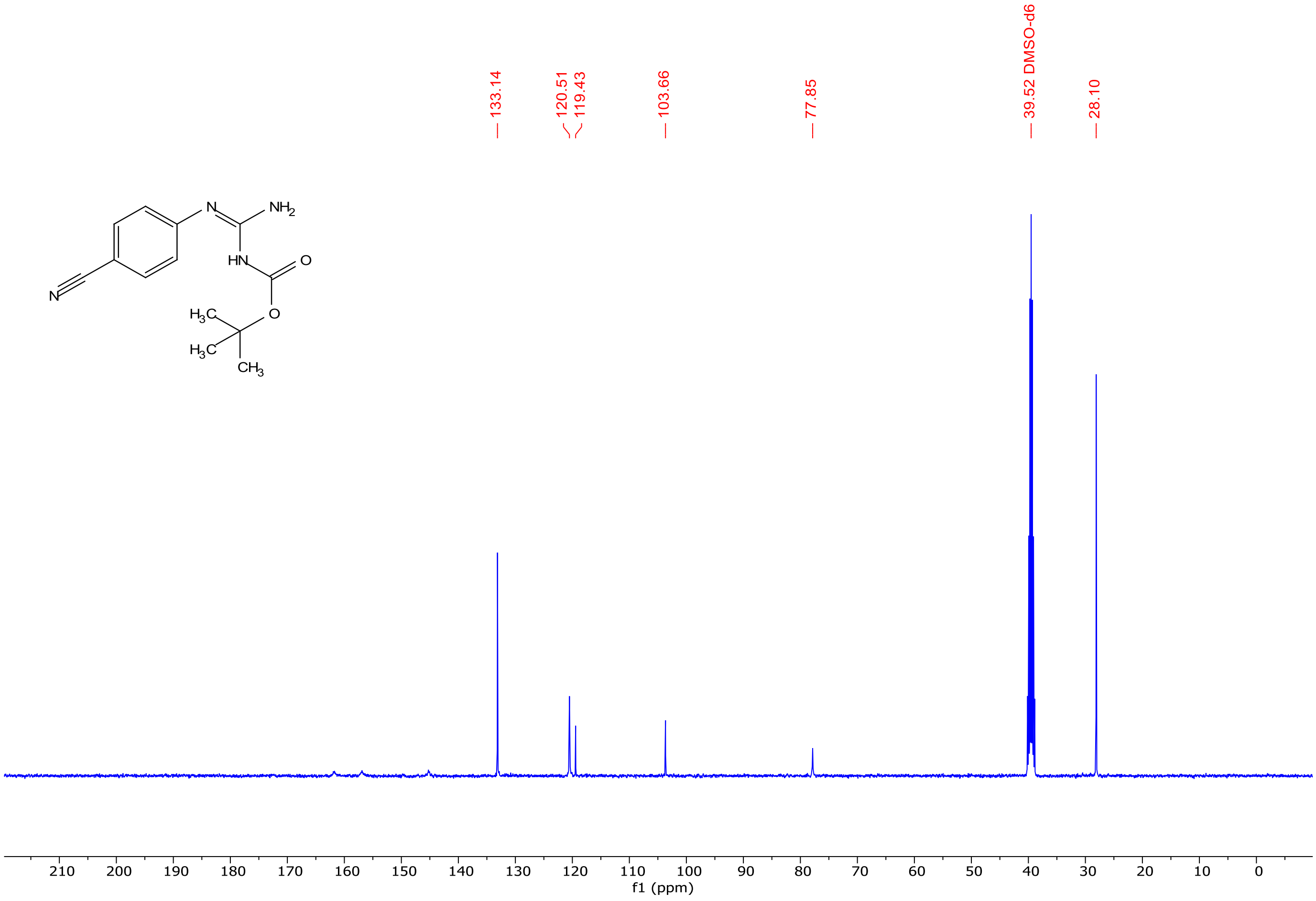
133.14
120.51
119.43
103.66
77.85
39.52 DMSO-d6
28.10
NH2
N
HN
O
O
N
H3C
H3C
CH3
210 200 190 180 170 160 150 140 130 120 110 100 90 80 70 60 50 40 30 20 10 0
f1 (ppm)

methyl 4-((dimethyl(oxo)-l6-sulfaneylidene)amino)benzoate (54)

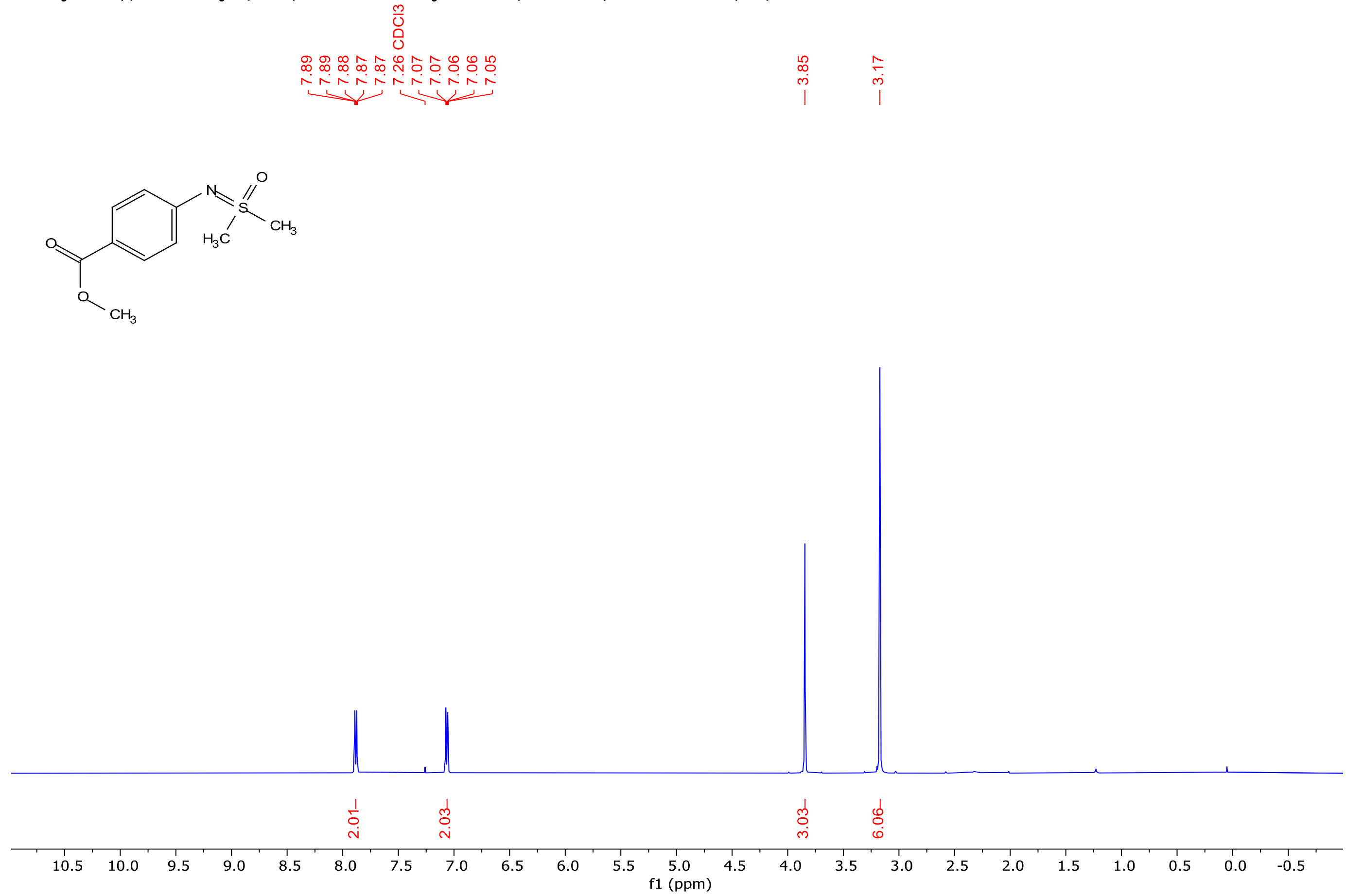

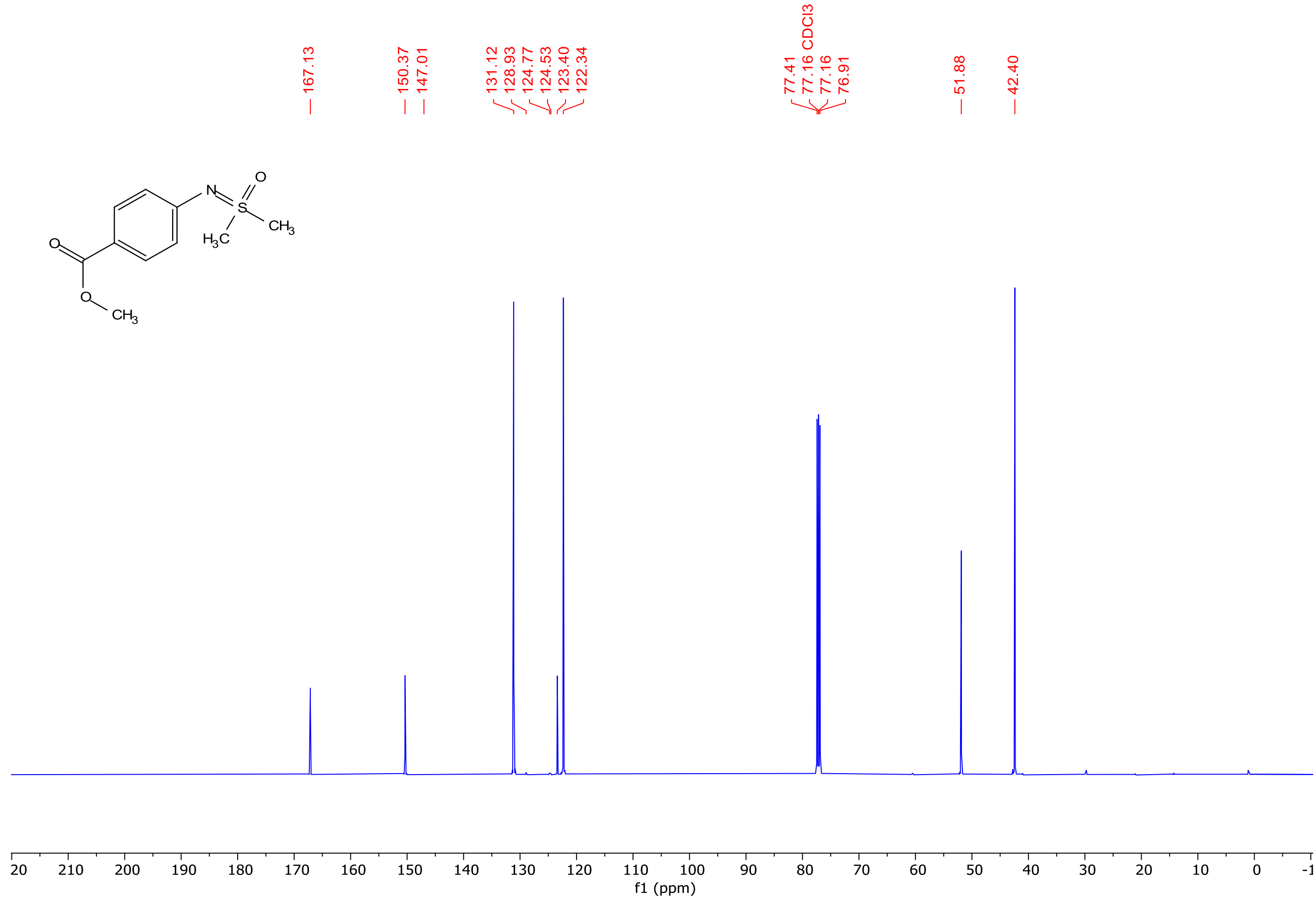

((4-benzoylphenyl)imino)dimethyl-l6-sulfanone (55)

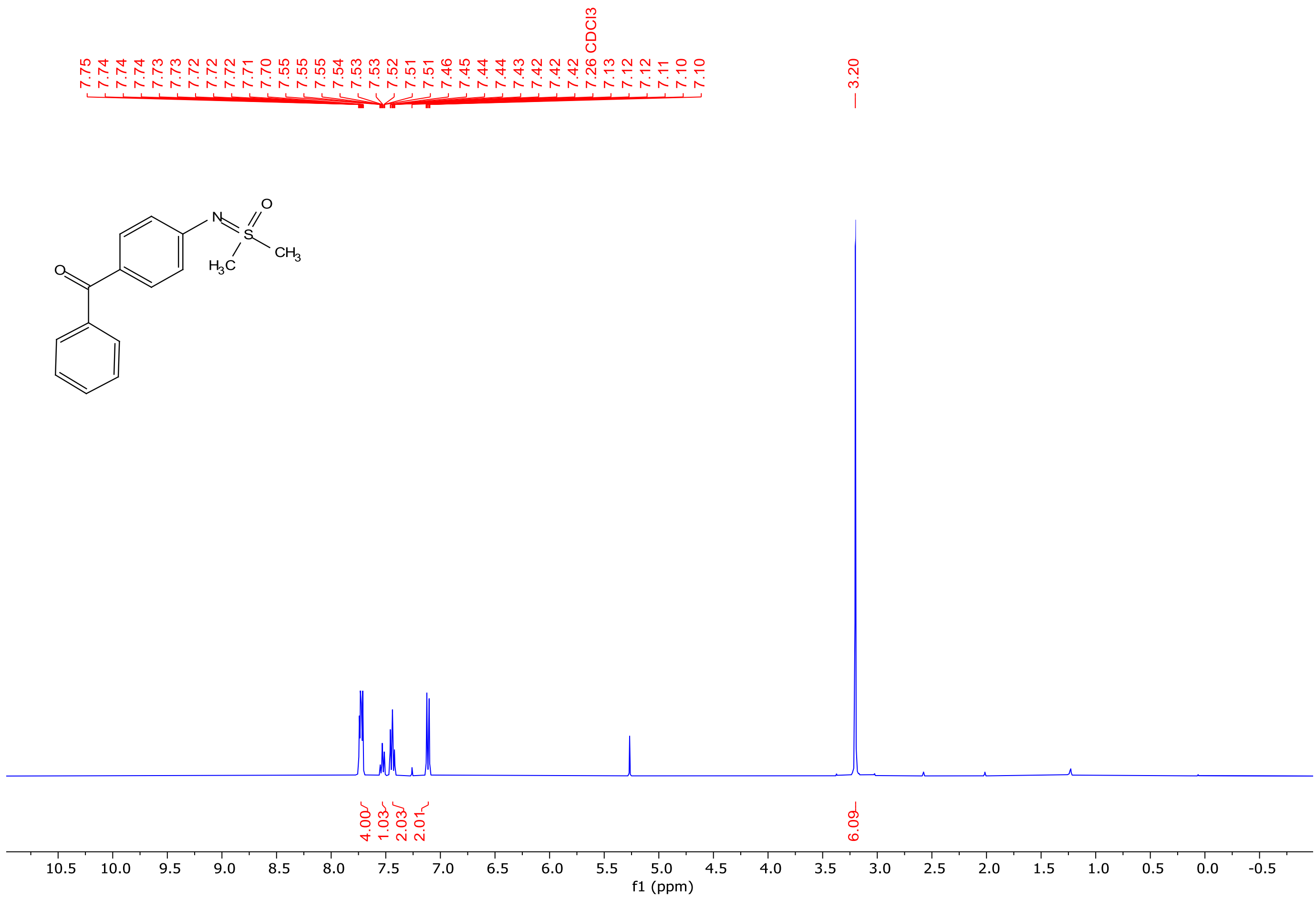

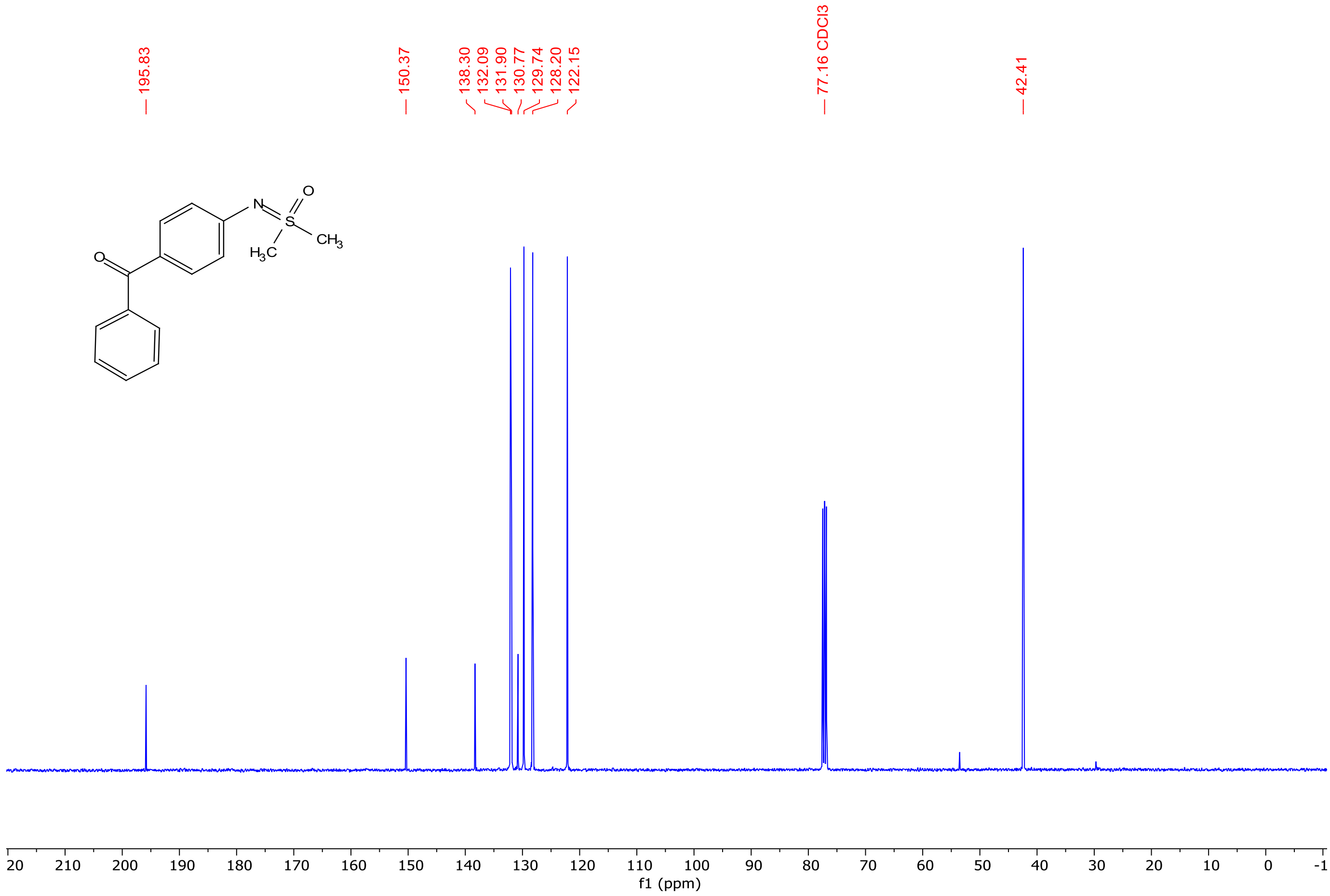

methyl 4-((oxodiphenyl-l6-sulfaneylidene)amino)benzoate (56)

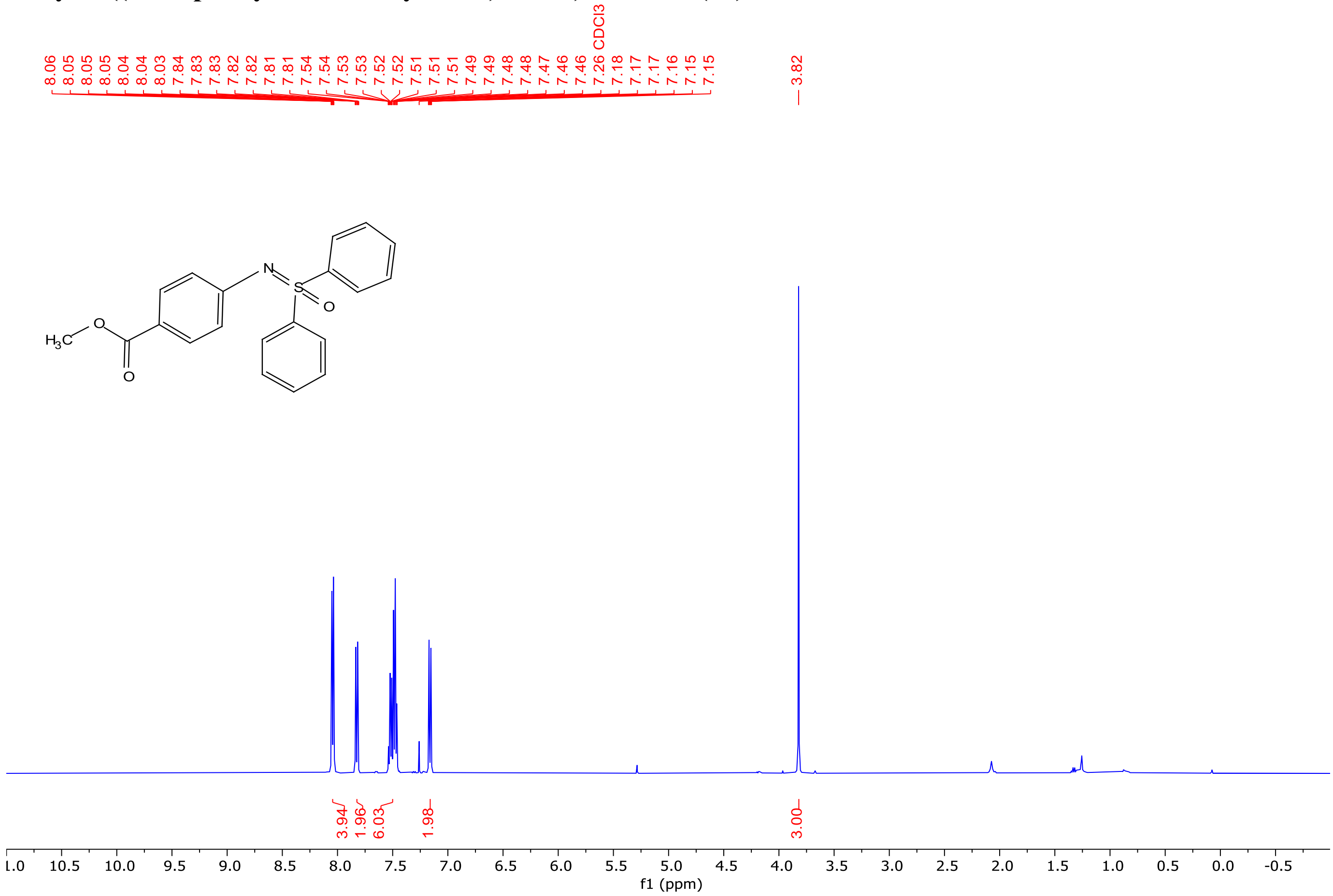

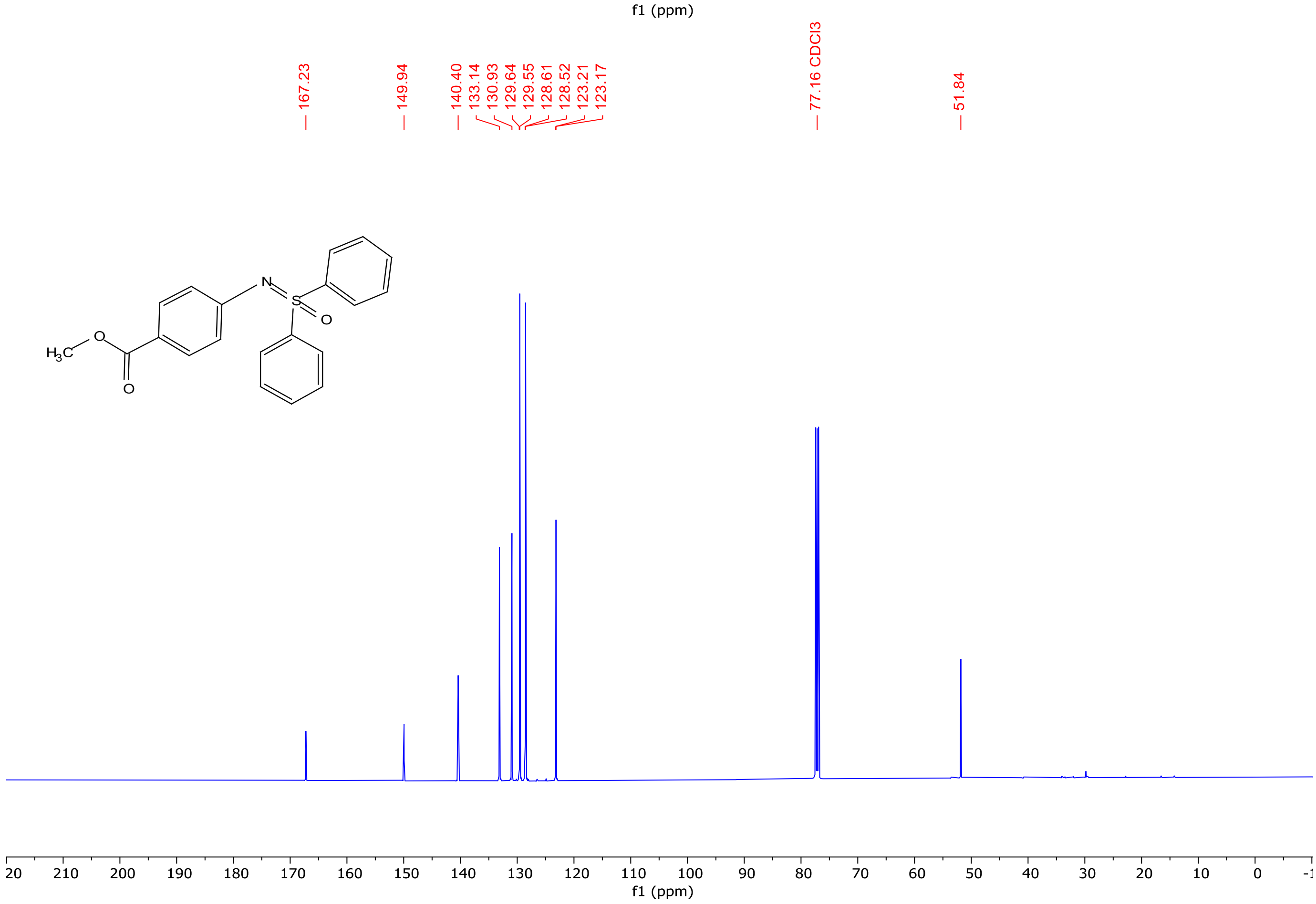

4-methyl-N-(4-(trifluoromethyl)phenyl)benzenesulfonamide (57)

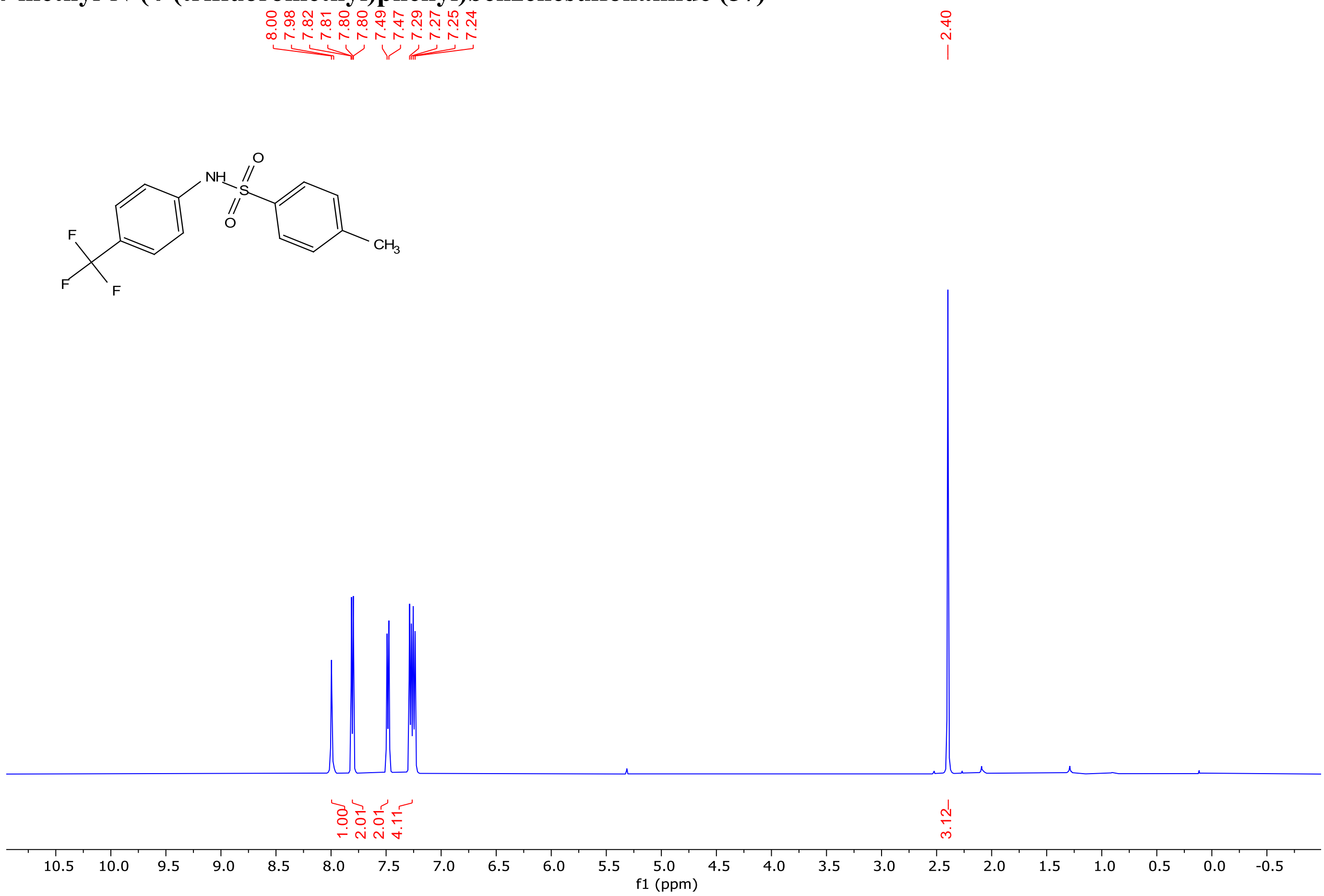

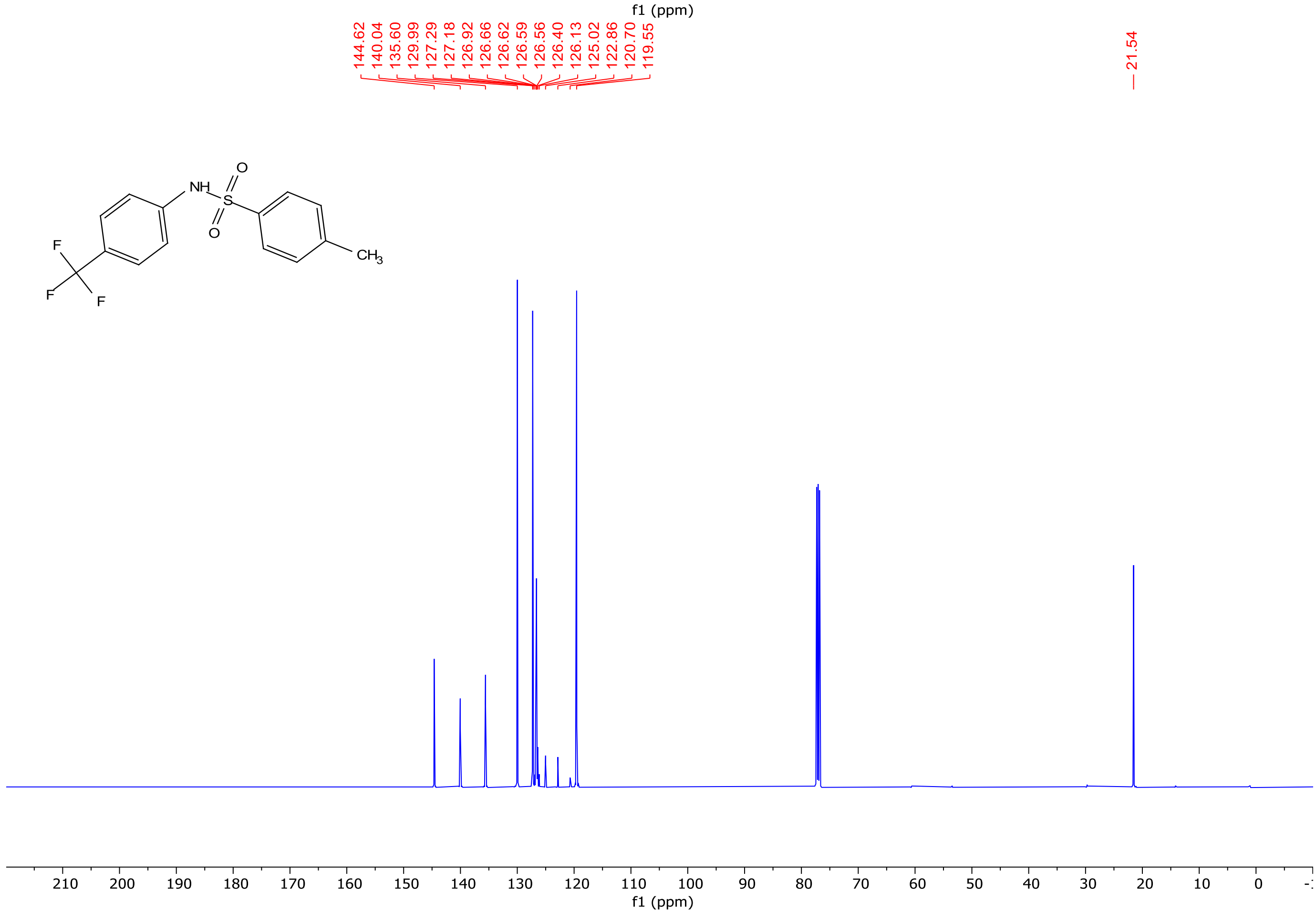

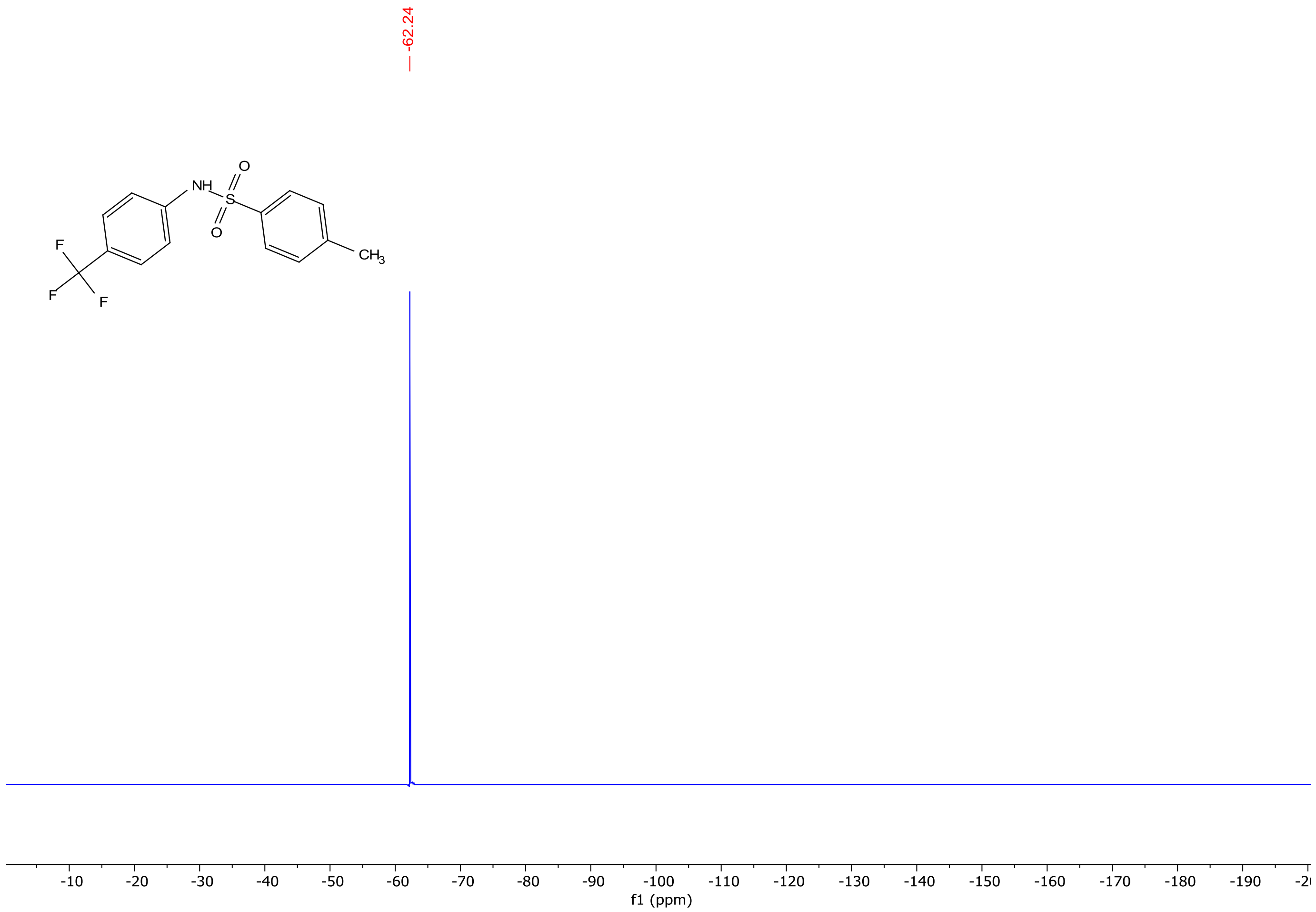
-62.24
NH
S
O
O
CH3
F
F
F
-10
-20
-30
-40
-50
-60
-70
-80
-90
-100
-110
-120
-130
-140
-150
-160
-170
-180
-190
f1 (ppm)

N-(4-benzoylphenyl)-4-methylbenzenesulfonamide (58)

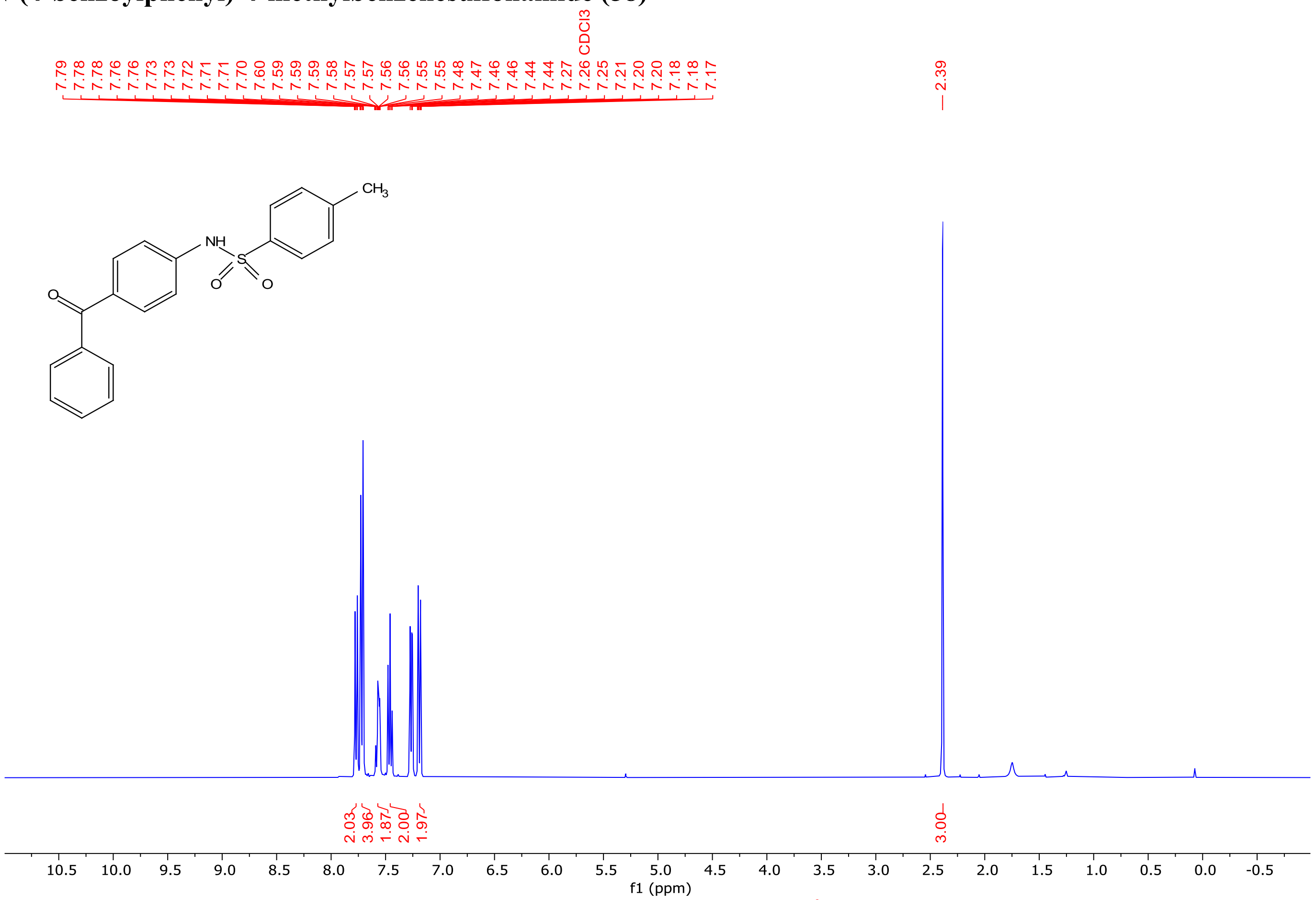

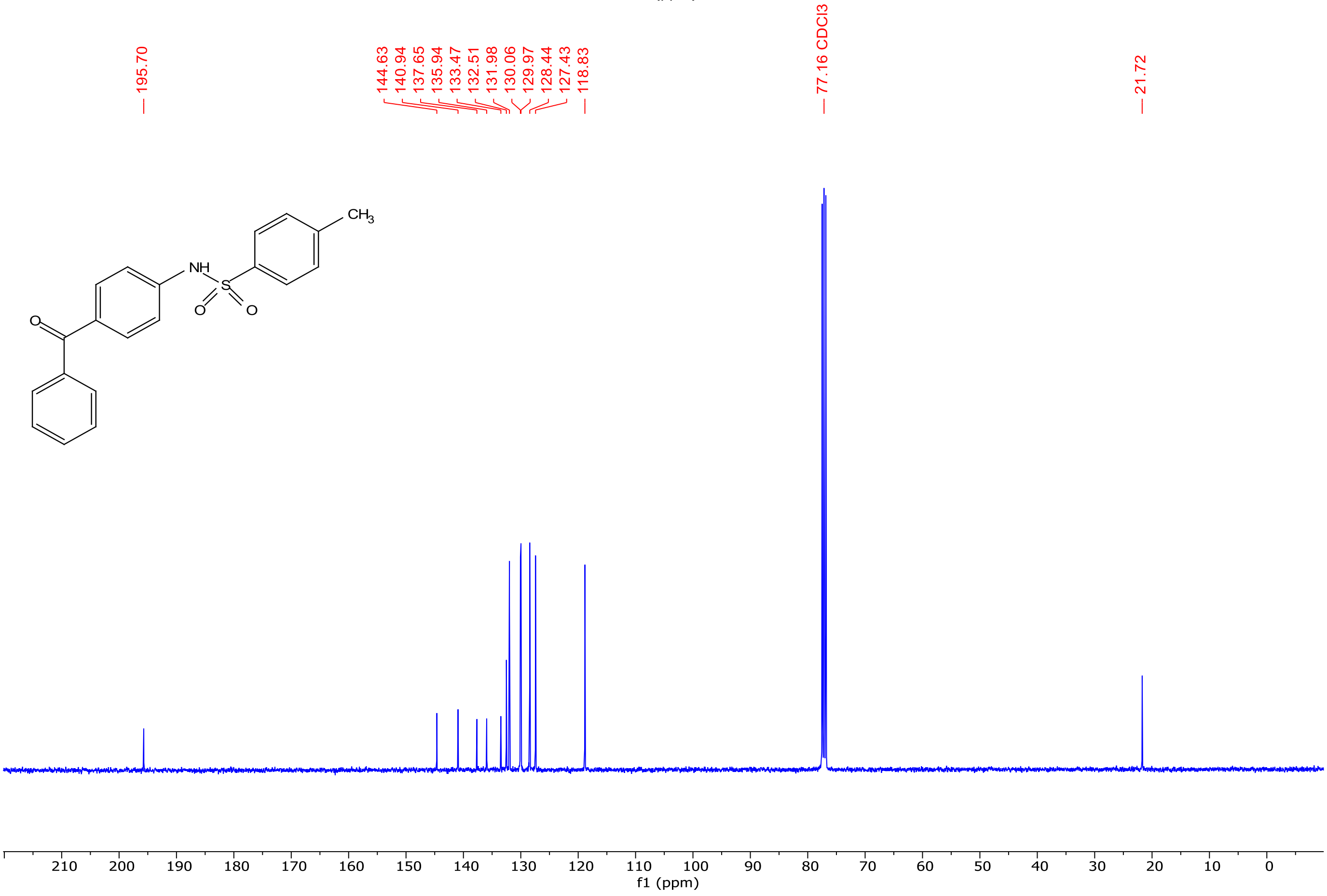

N-(4-(trifluoromethyl)phenyl)methanesulfonamide (59)

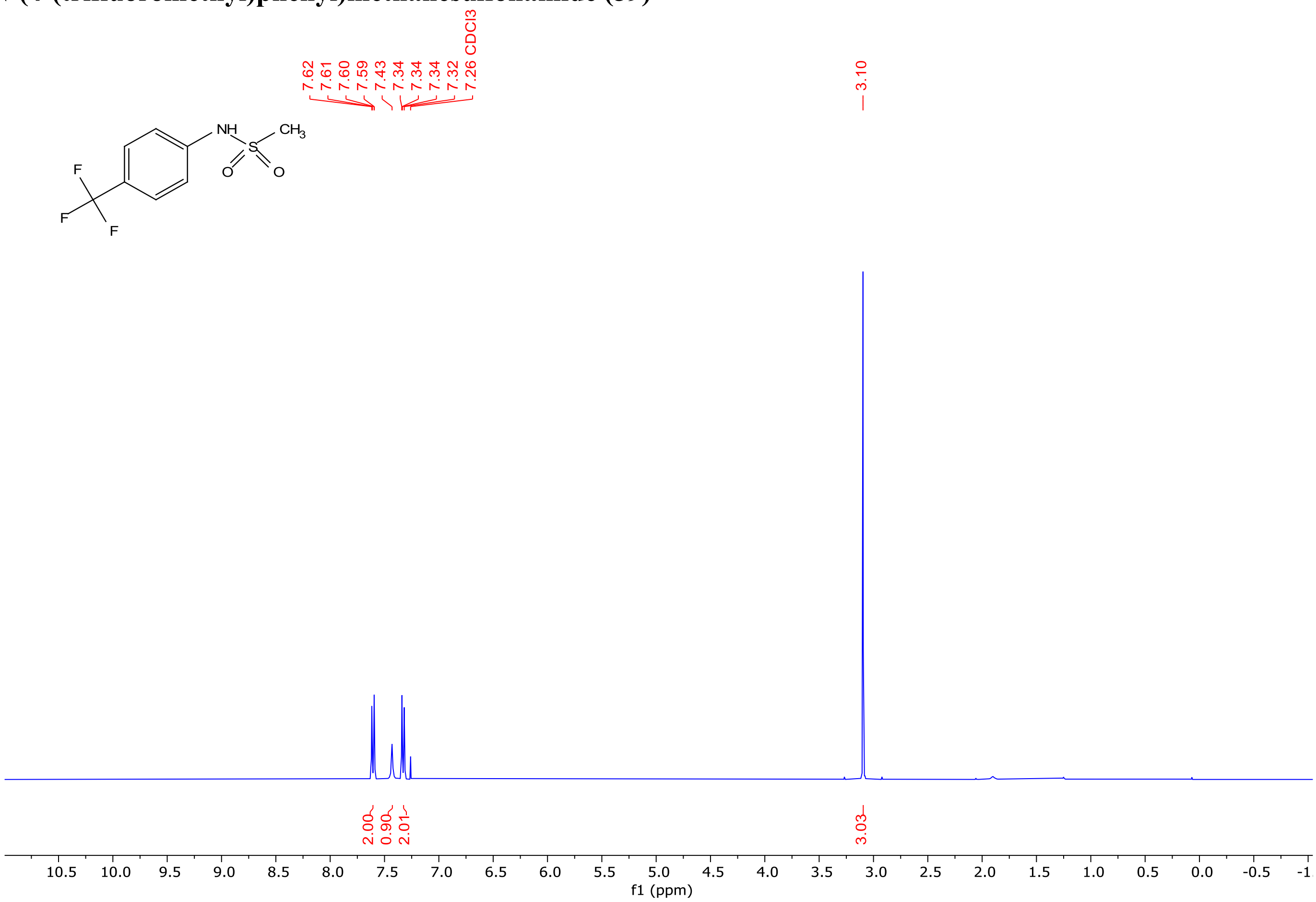

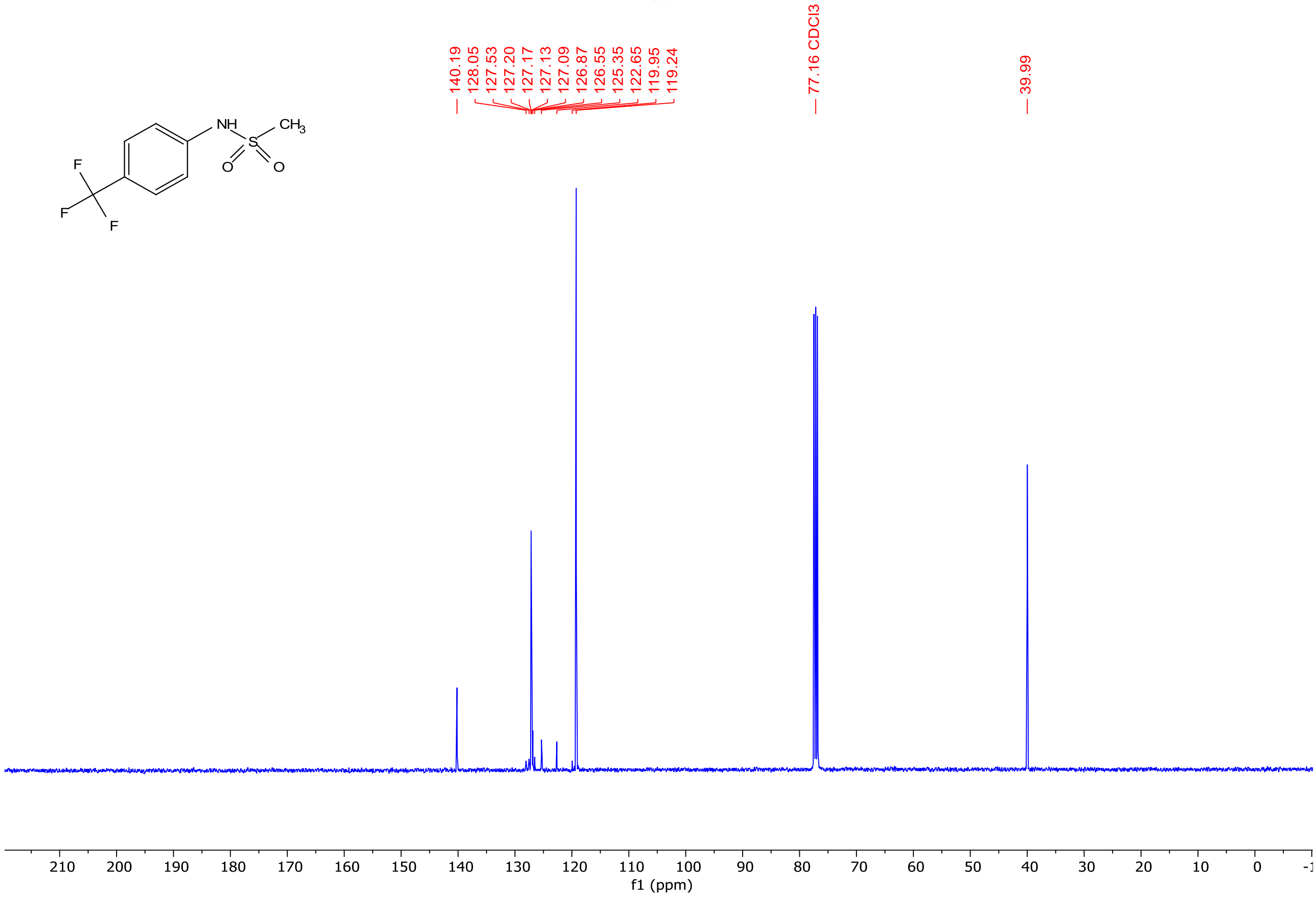

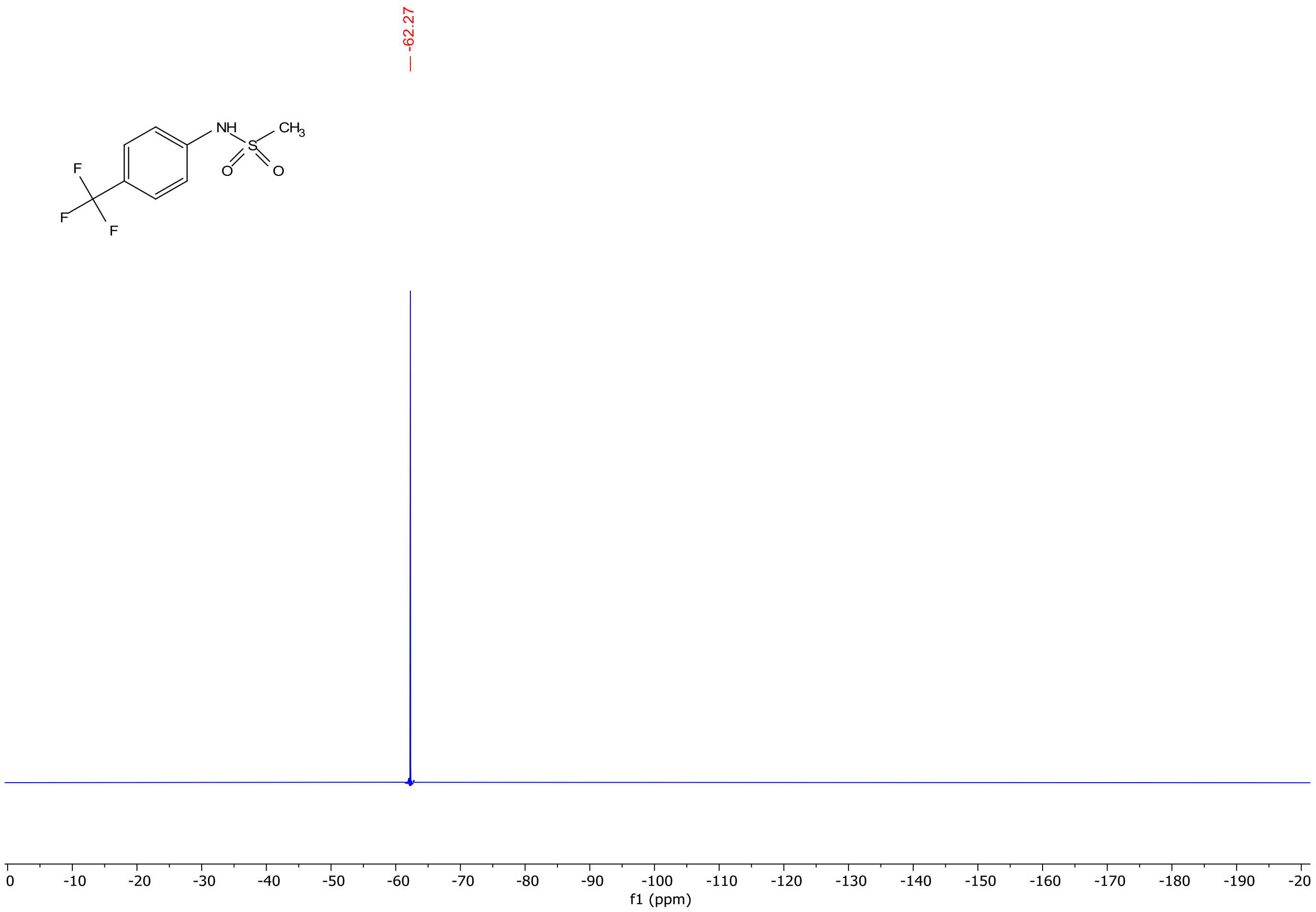

-62.27
NH
CH3
S
O
O
F
F
F
0
-10
-20
-30
-40
-50
-60
-70
-80
-90
-100
-110
-120
-130
-140
-150
-160
-170
-180
-190
-20
f1 (ppm)

benzyl 2-(4-(methoxycarbonyl)phenyl)hydrazine-1-carboxylate (60)

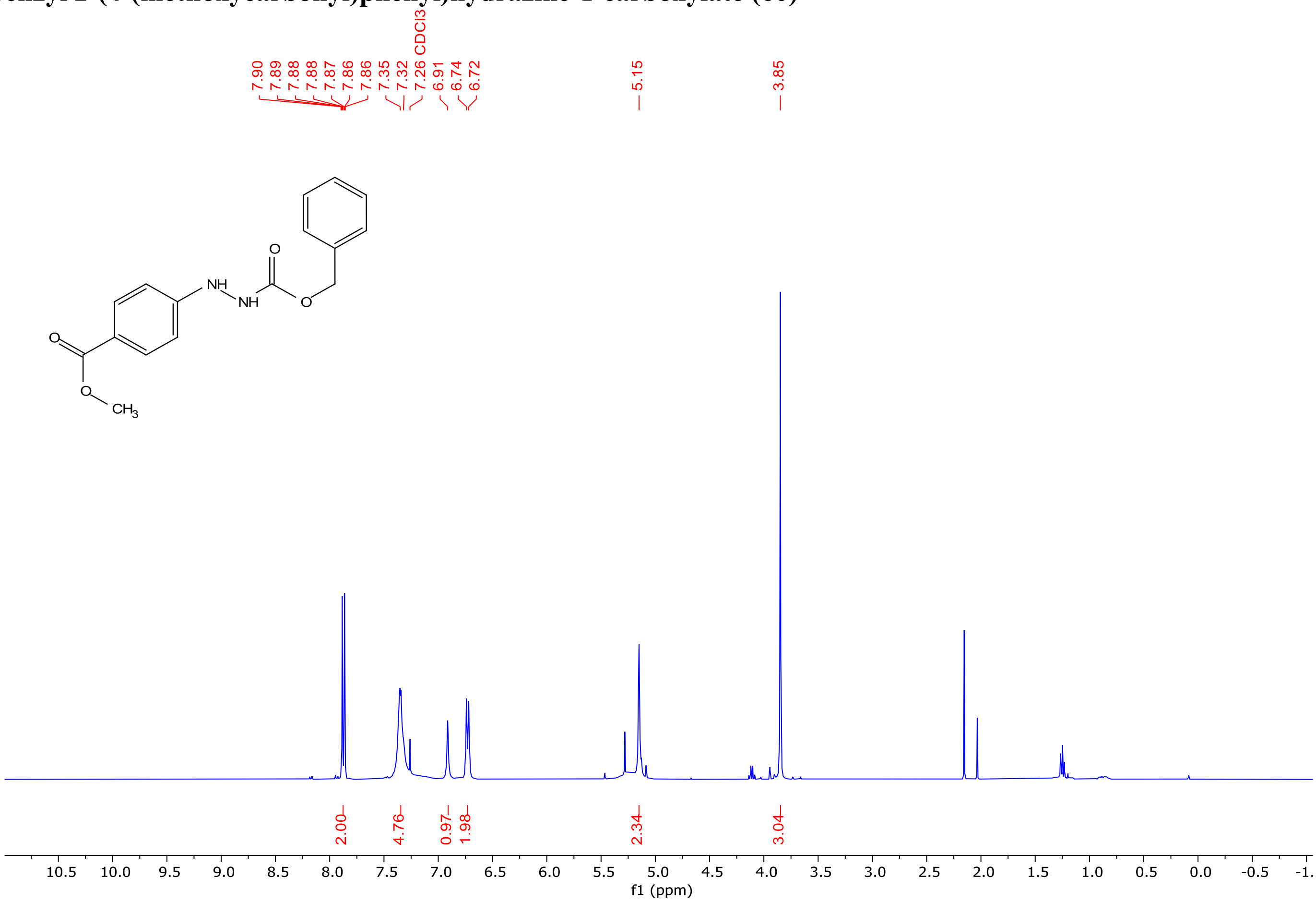

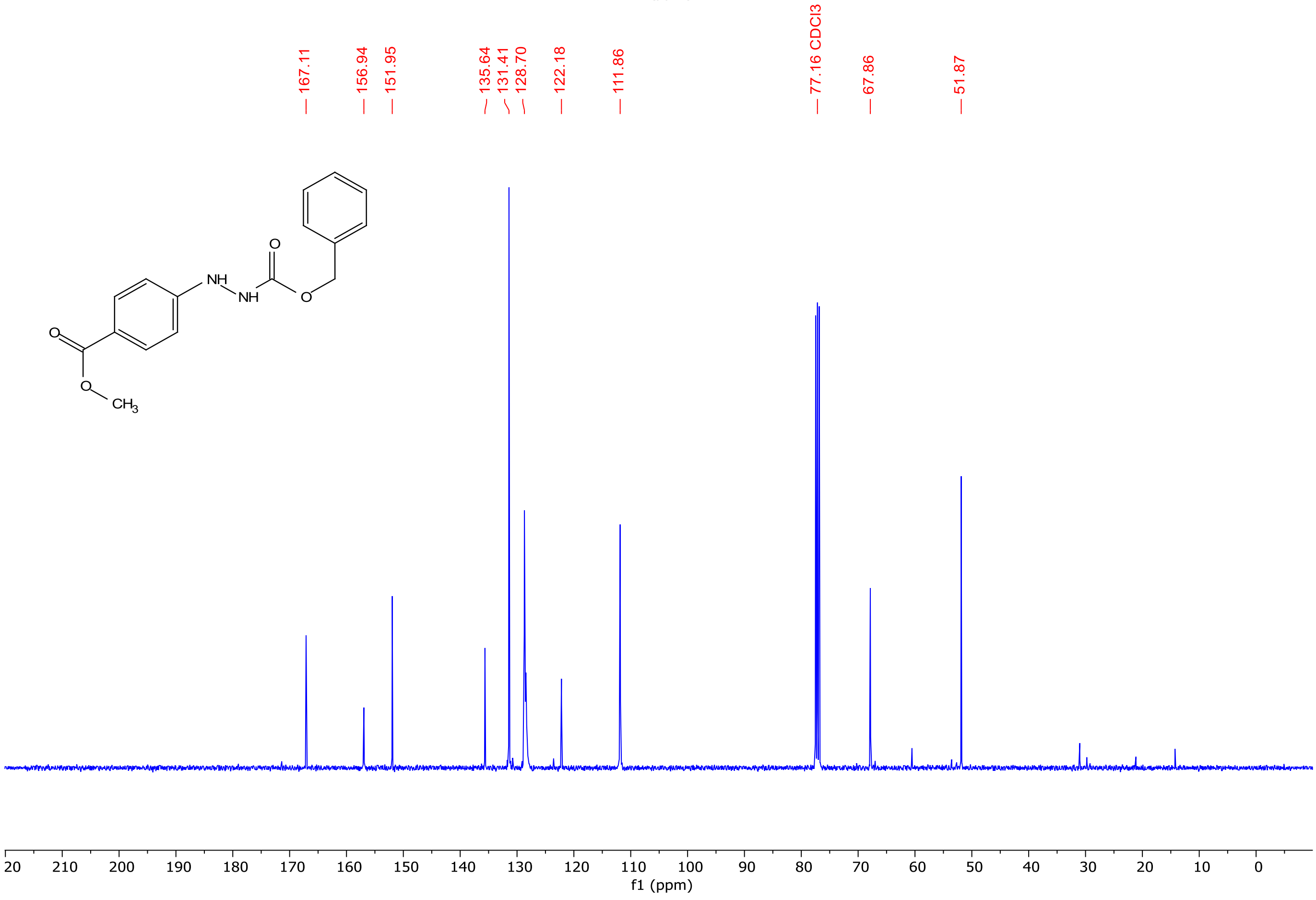

***tert*-butyl 2-(4-(methoxycarbonyl)phenyl)hydrazine-1-carboxylate (61)**

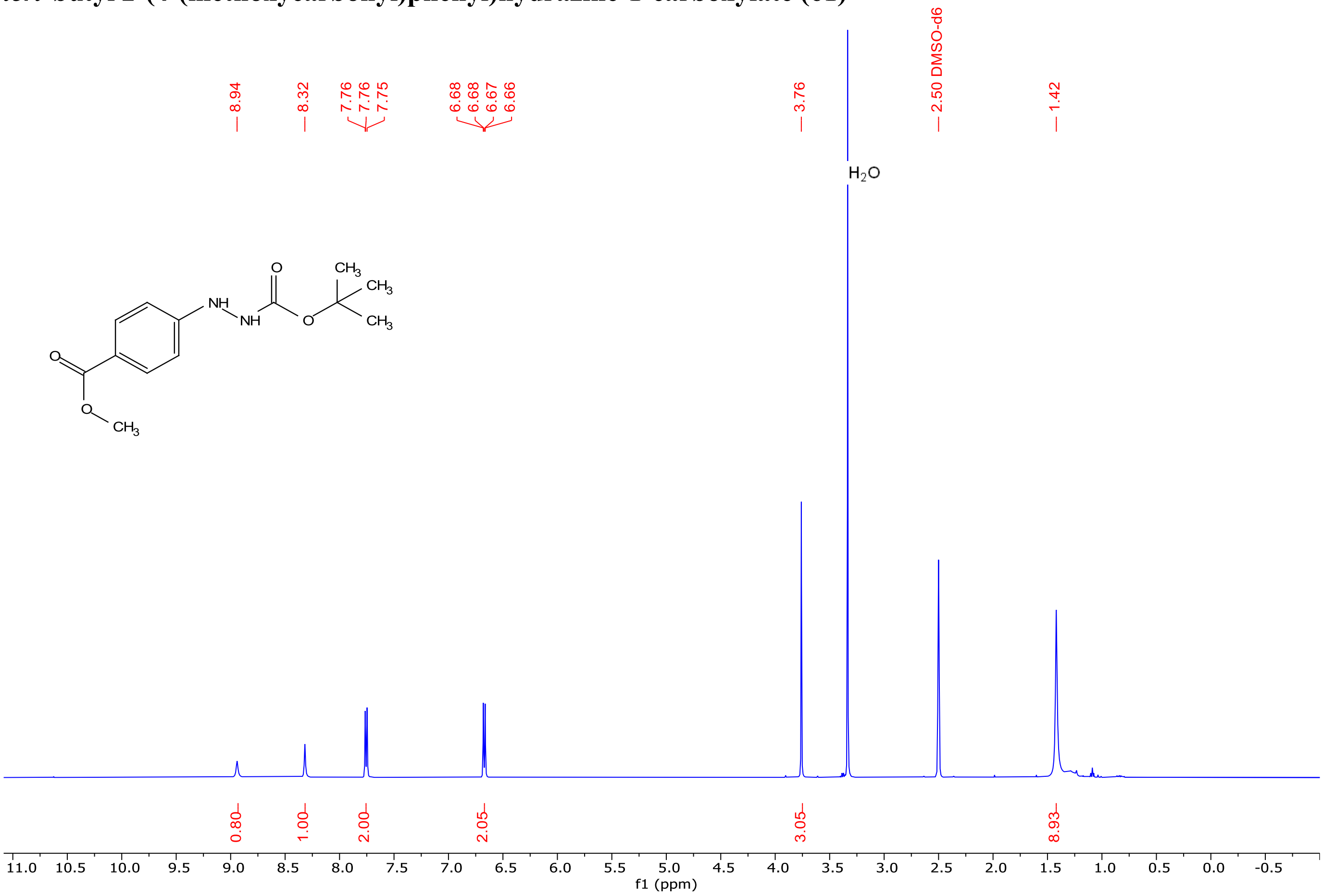

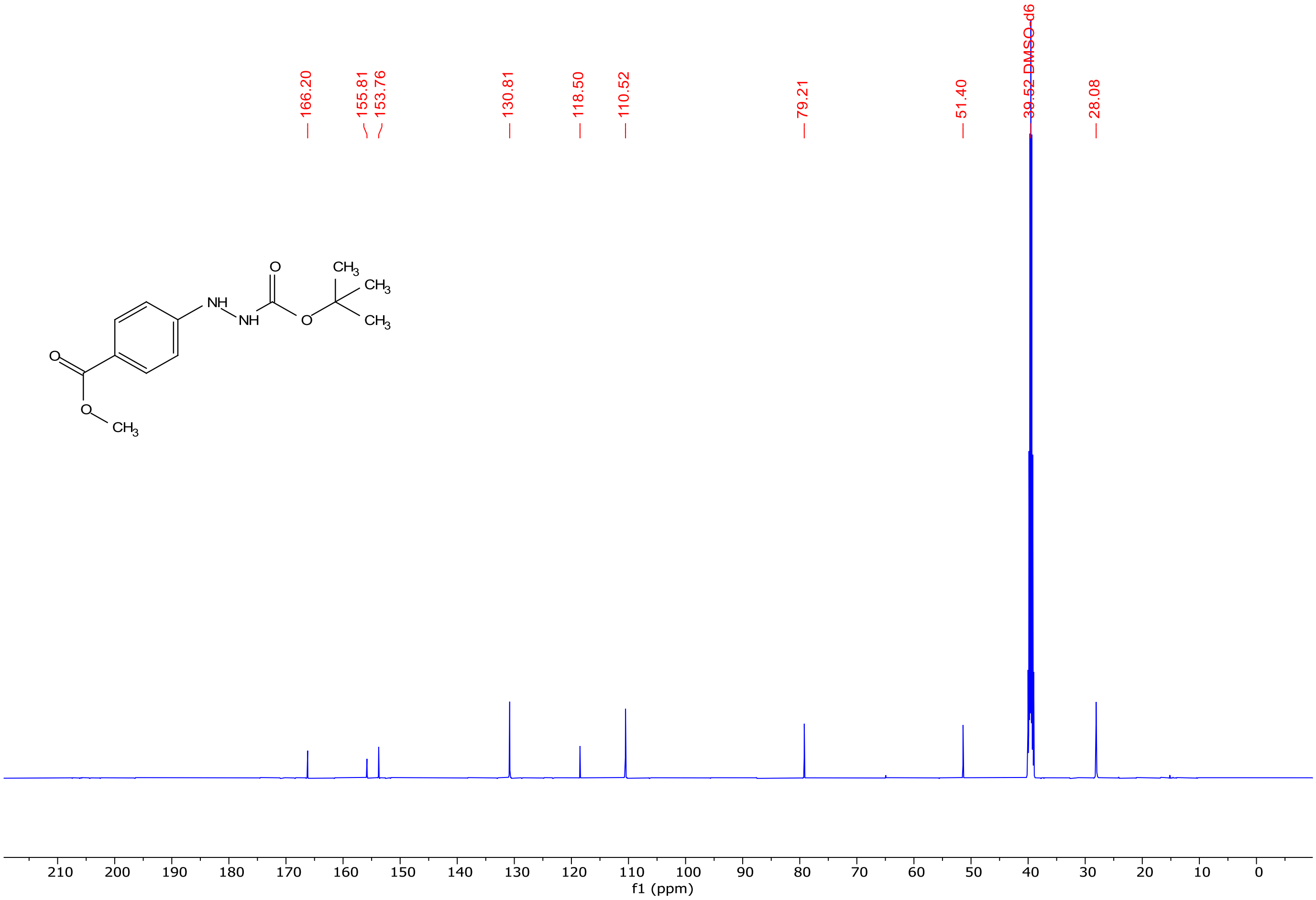

N'-(4-cyanophenyl)benzohydrazide (62)

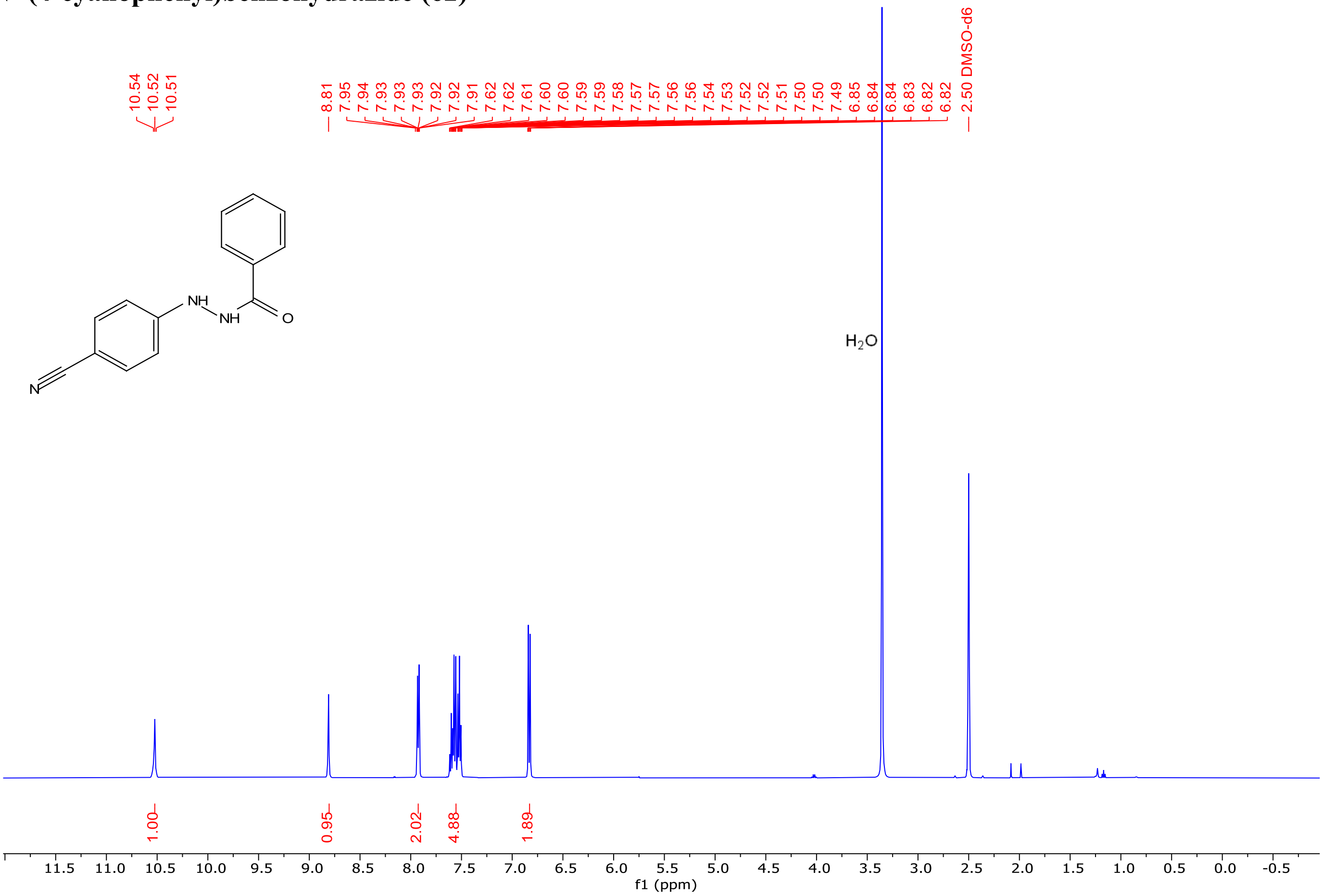

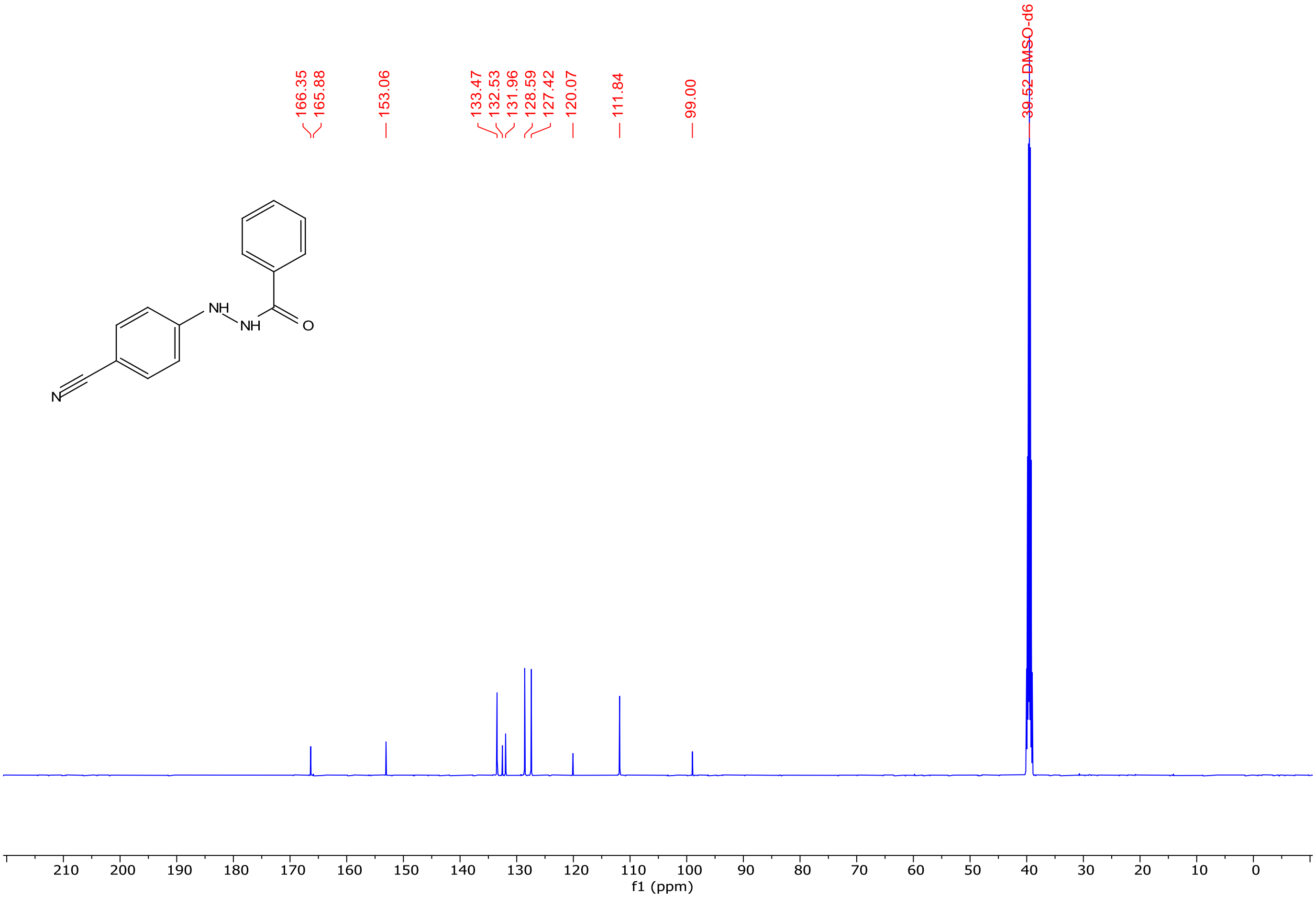

1-(diphenylmethylene)-2-(4-(trifluoromethyl)phenyl)hydrazine (63)

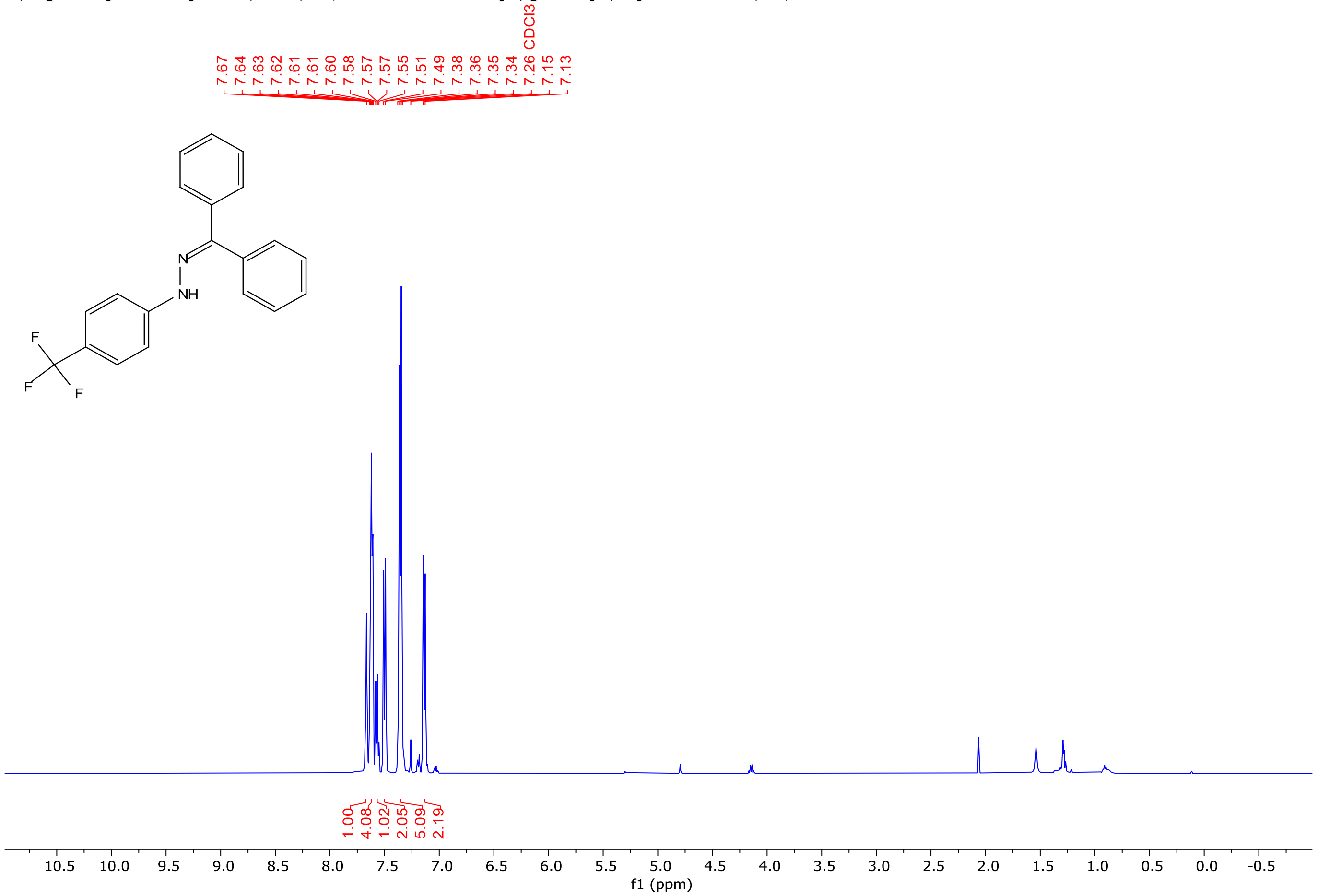

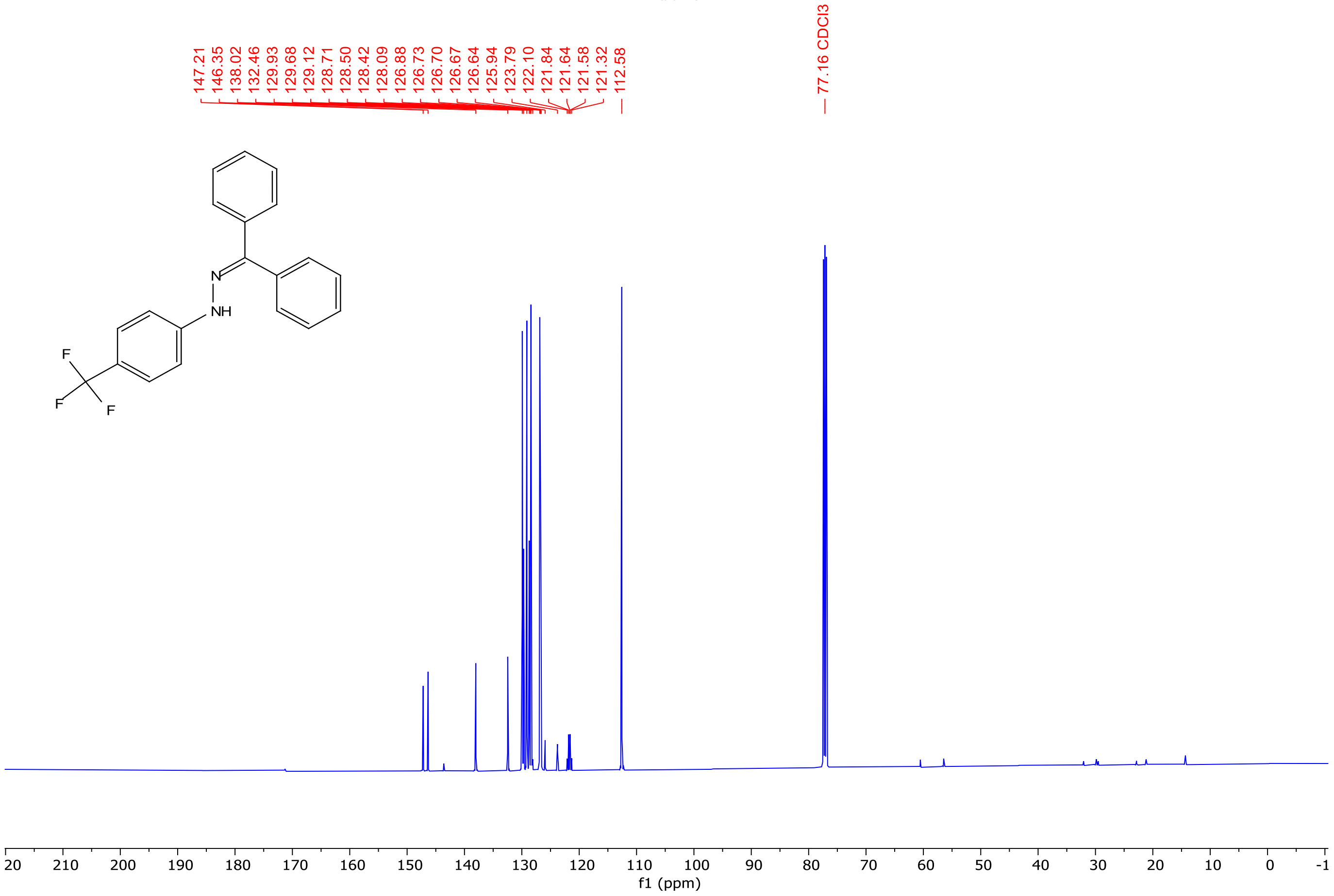

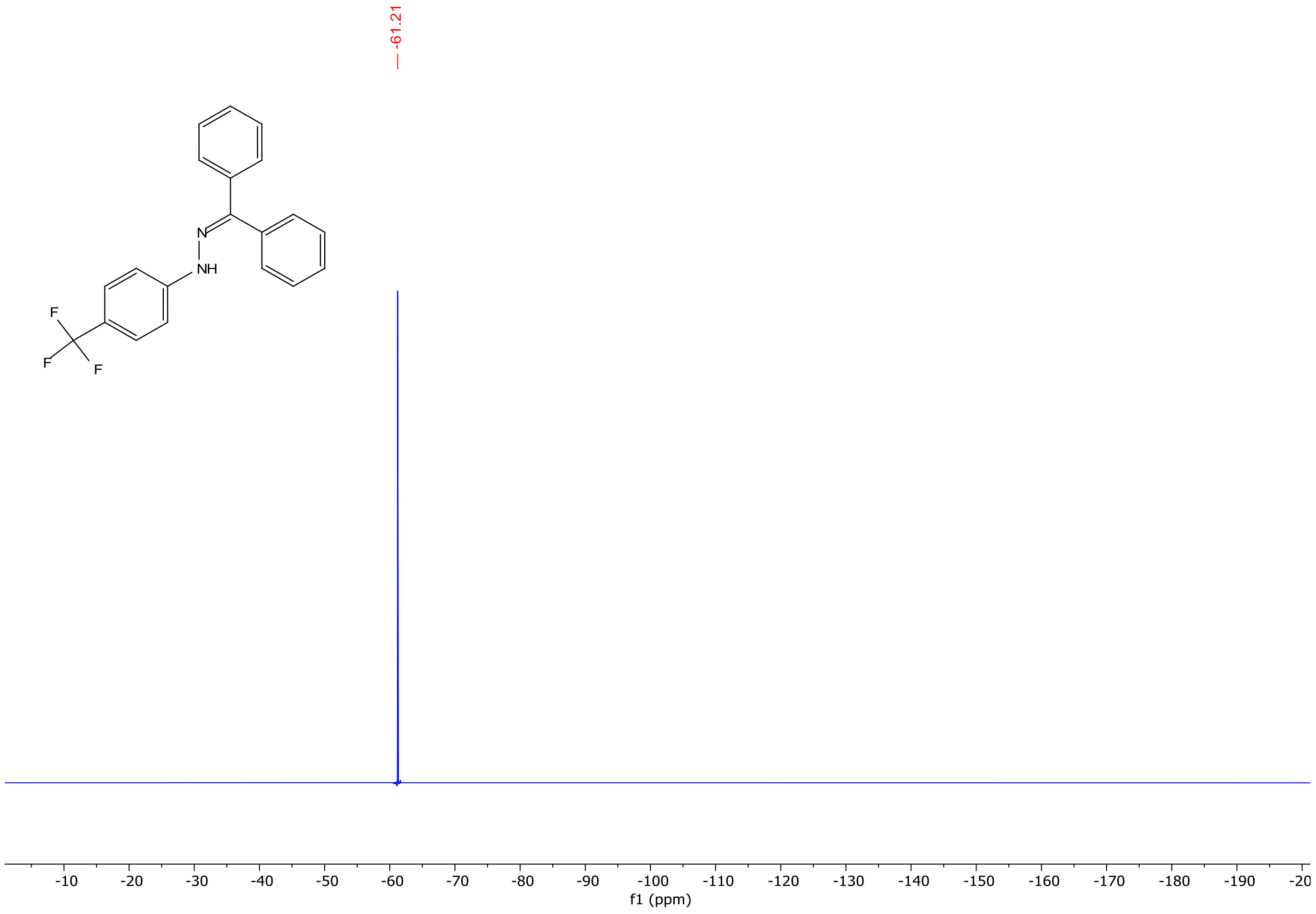
-61.21
F
F
F
N
NH
-10 -20 -30 -40 -50 -60 -70 -80 -90 -100 -110 -120 -130 -140 -150 -160 -170 -180 -190 -20
f1 (ppm)

N-(4-(trifluoromethyl)phenyl)benzamide (64)

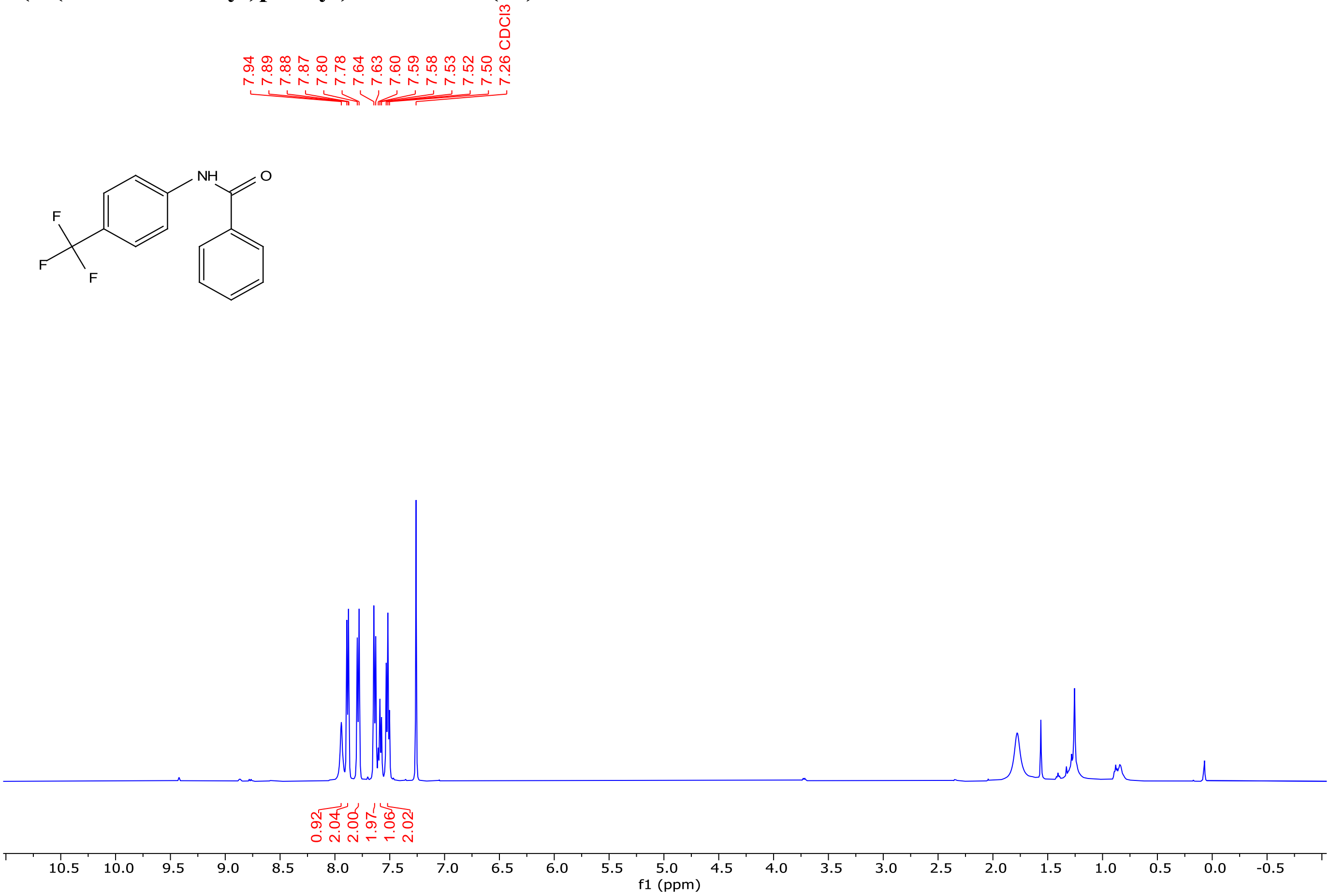

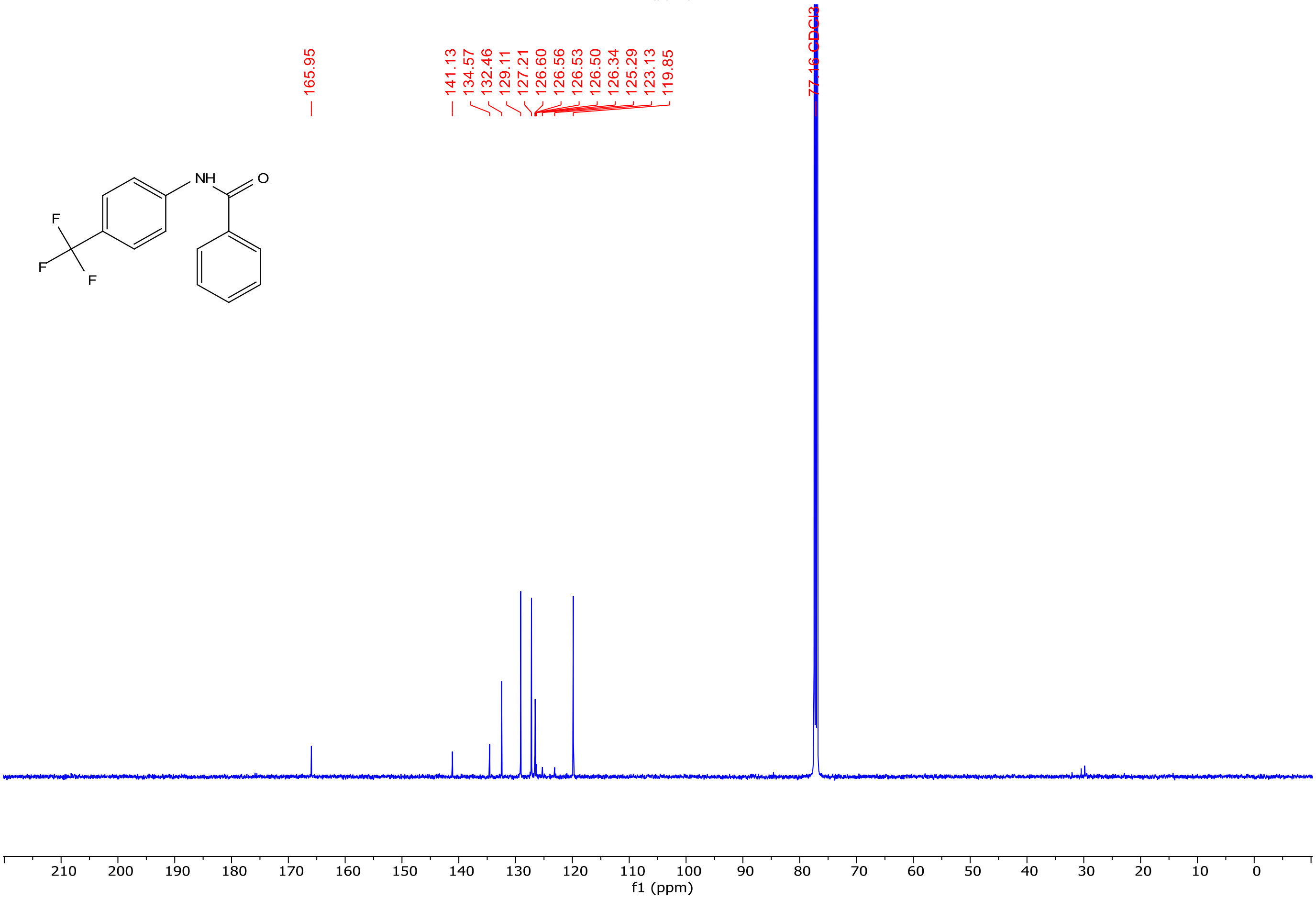

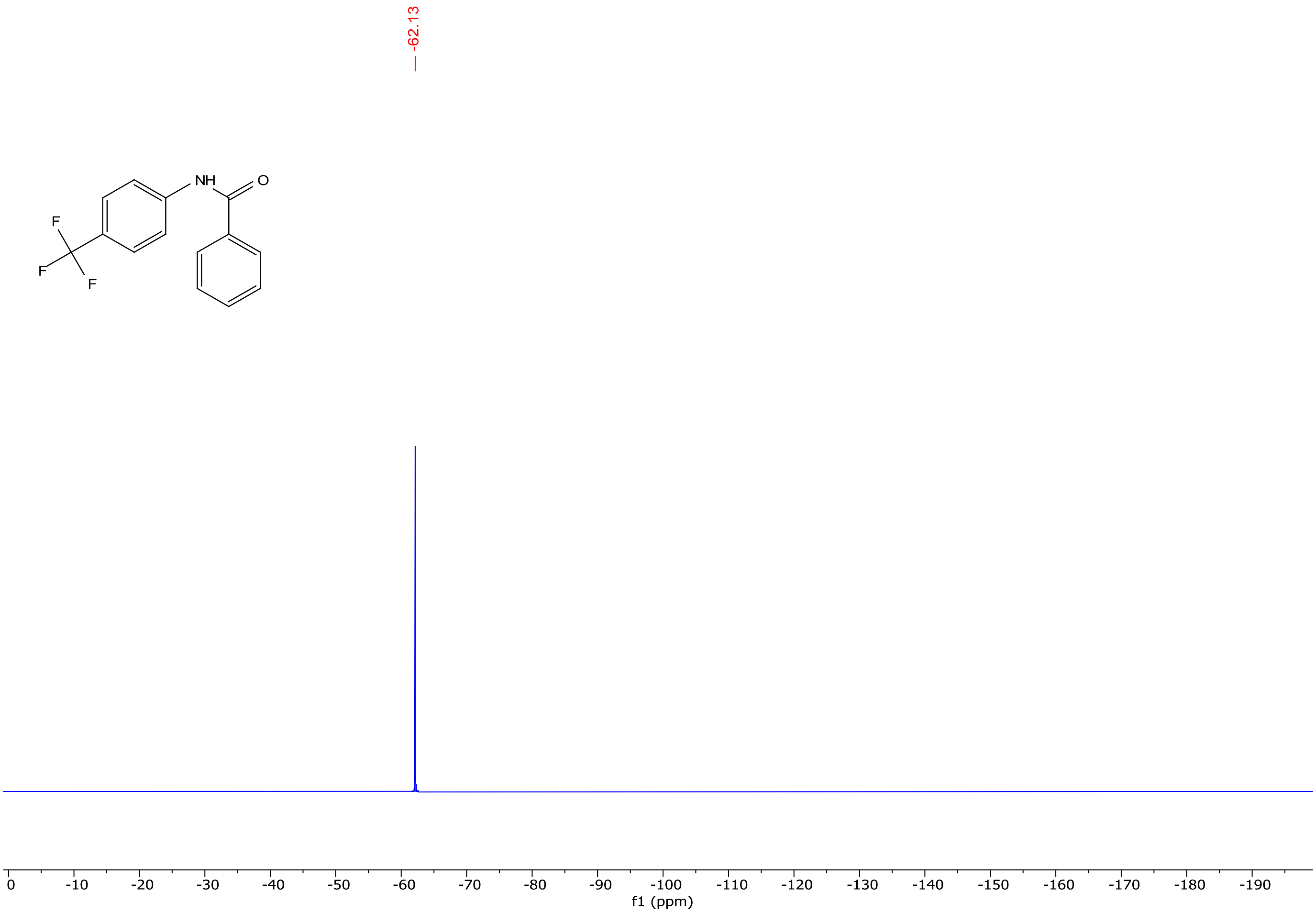
-62.13
NH
O
F
F
F
0
-10
-20
-30
-40
-50
-60
-70
-80
-90
-100
-110
-120
-130
-140
-150
-160
-170
-180
-190
f1 (ppm)

1-(4-(trifluoromethyl)phenyl)pyrrolidin-2-one (65)

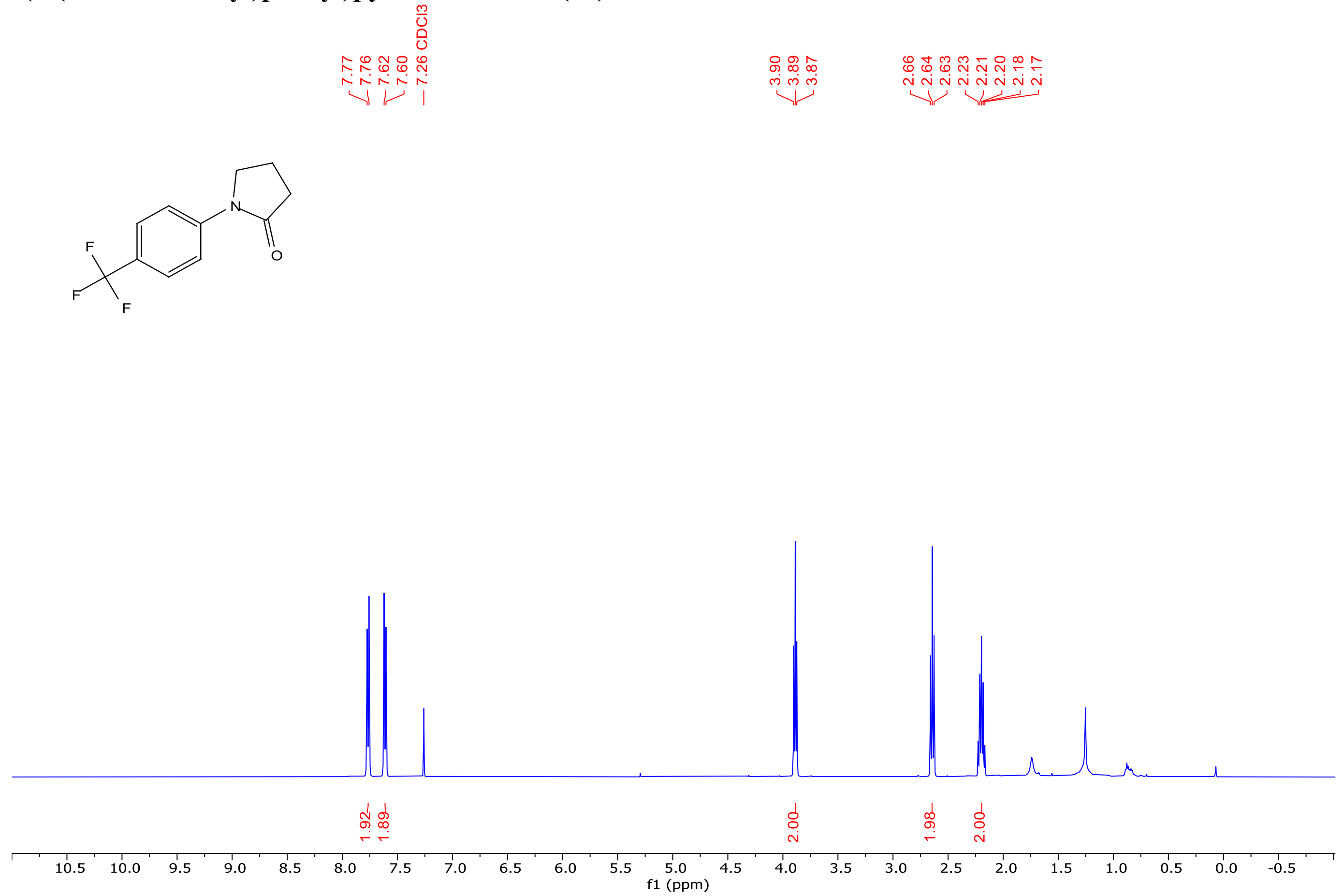

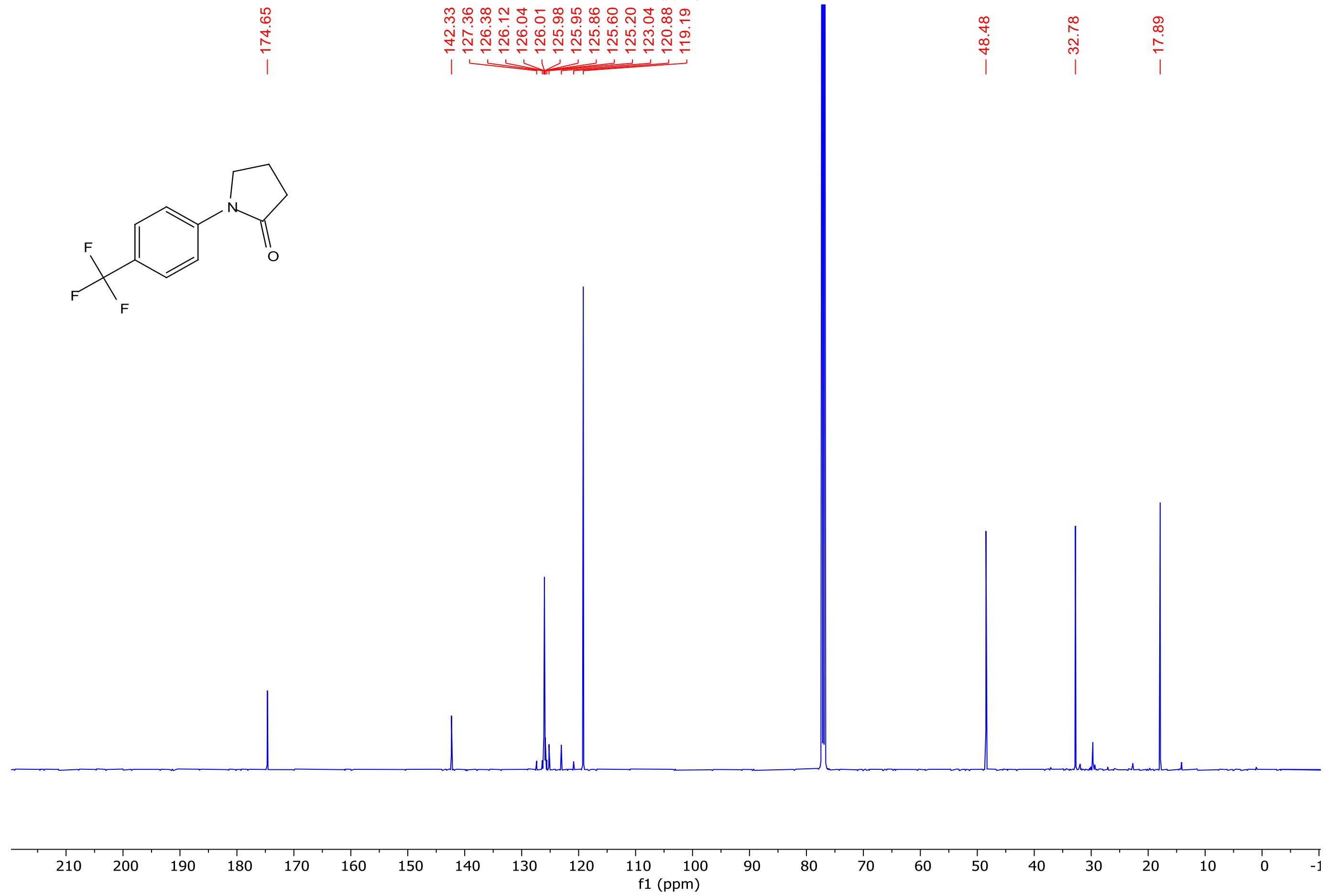

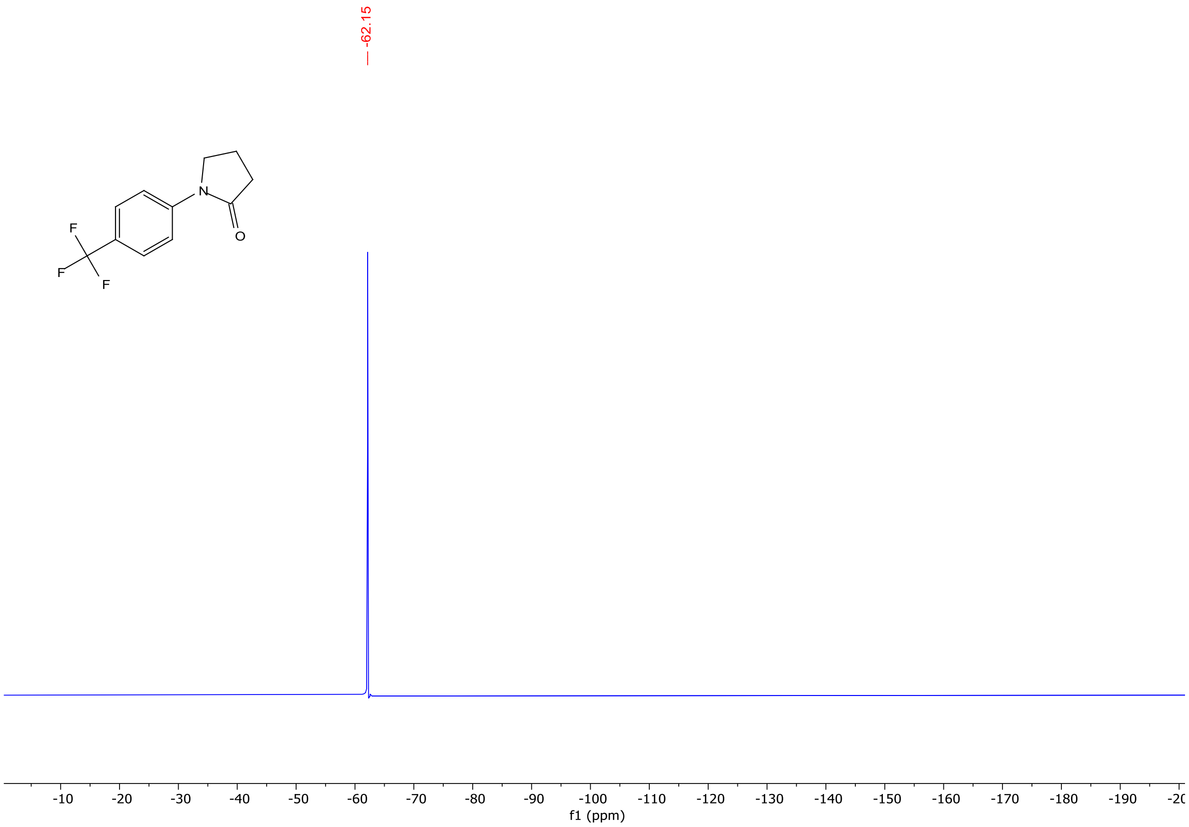
-62.15
F
F
F
N
O
-10
-20
-30
-40
-50
-60
-70
-80
-90
-100
-110
-120
-130
-140
-150
-160
-170
-180
-190
f1 (ppm)

methyl 4-(2,2,2-trifluoroacetamido)benzoate (66)

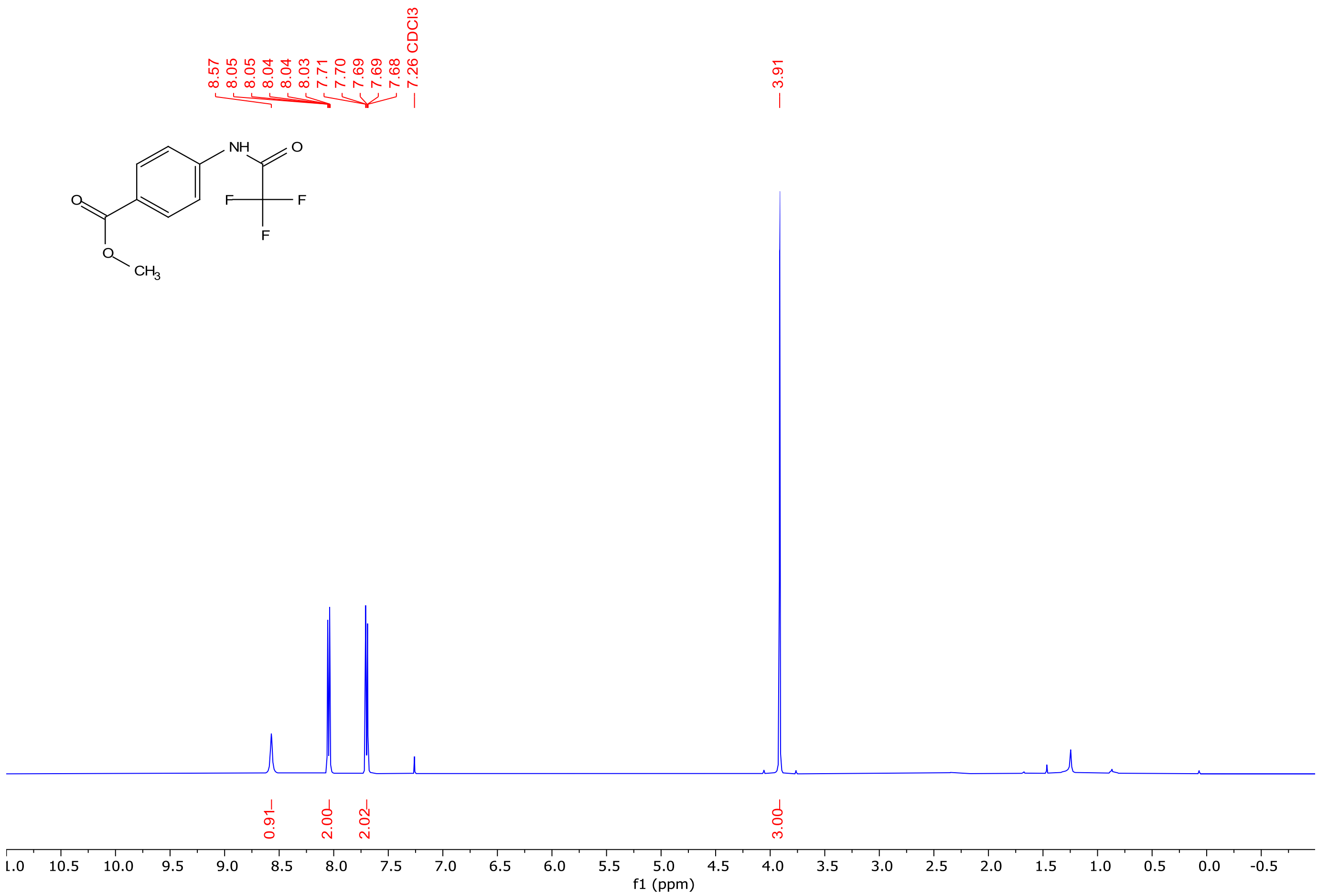

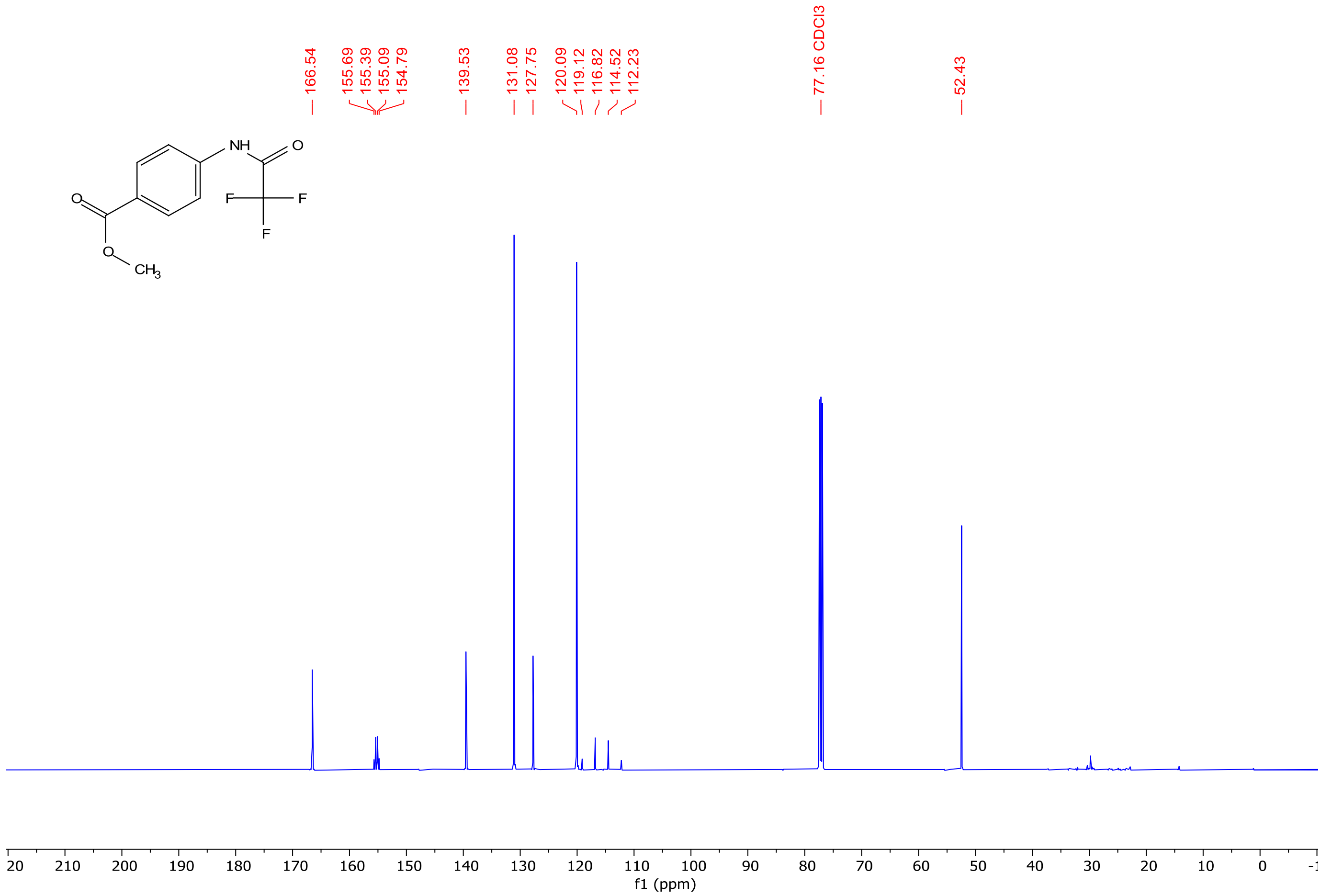

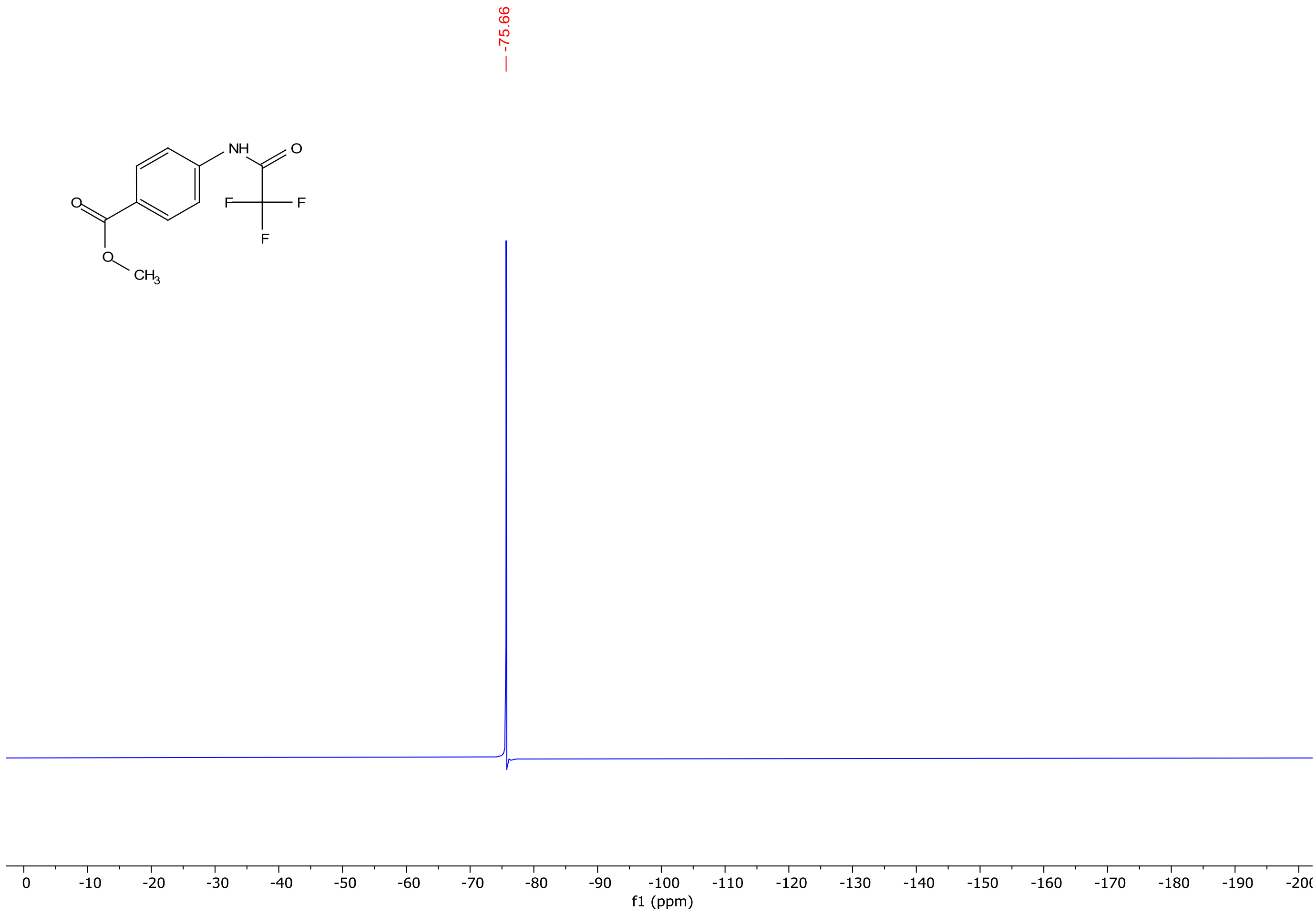
-75.66
NH
O
F
F
F
O
O
CH3
0
-10
-20
-30
-40
-50
-60
-70
-80
-90
-100
-110
-120
-130
-140
-150
-160
-170
-180
-190
f1 (ppm)

N-(4-(trifluoromethyl)phenyl)butyramide (67)

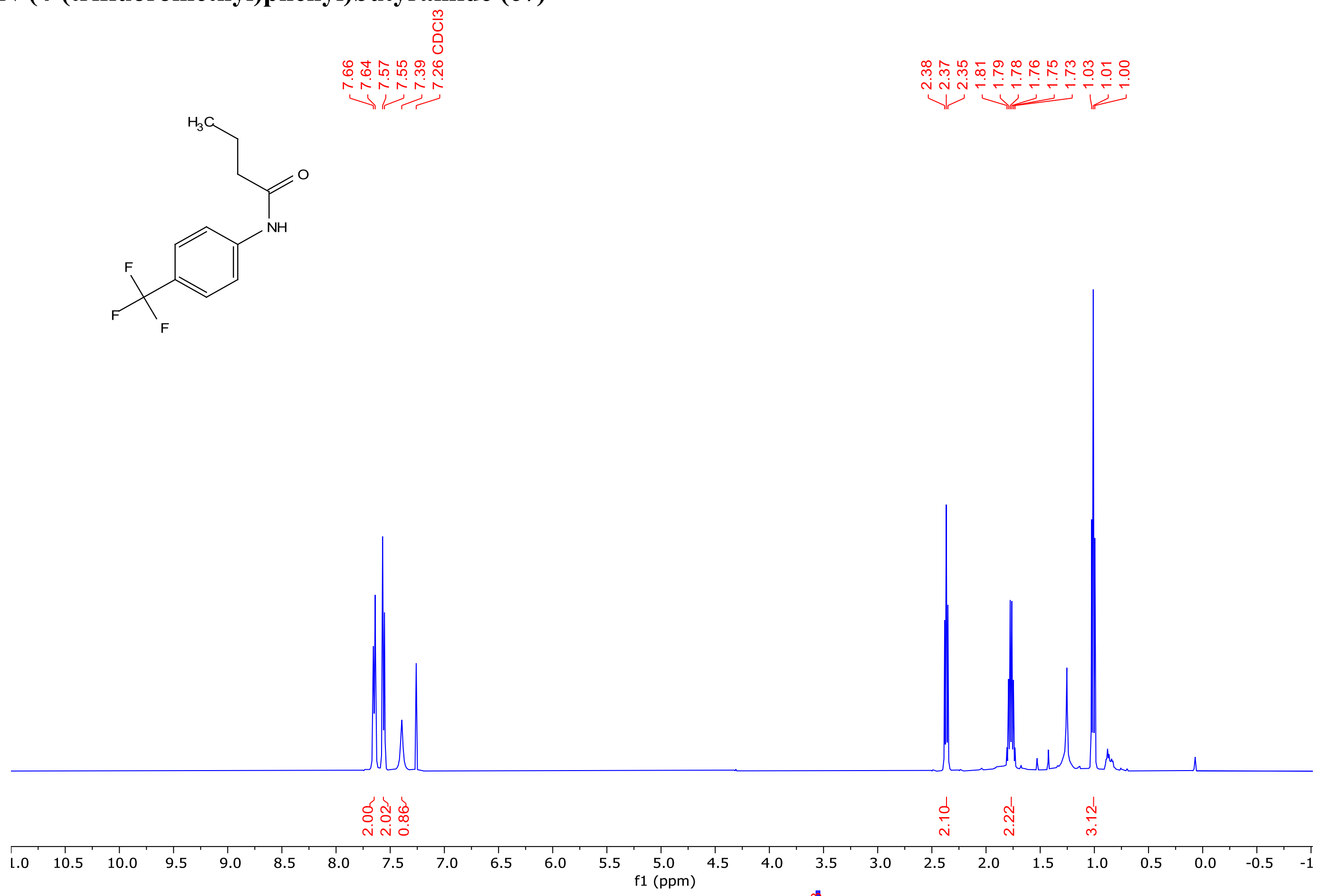

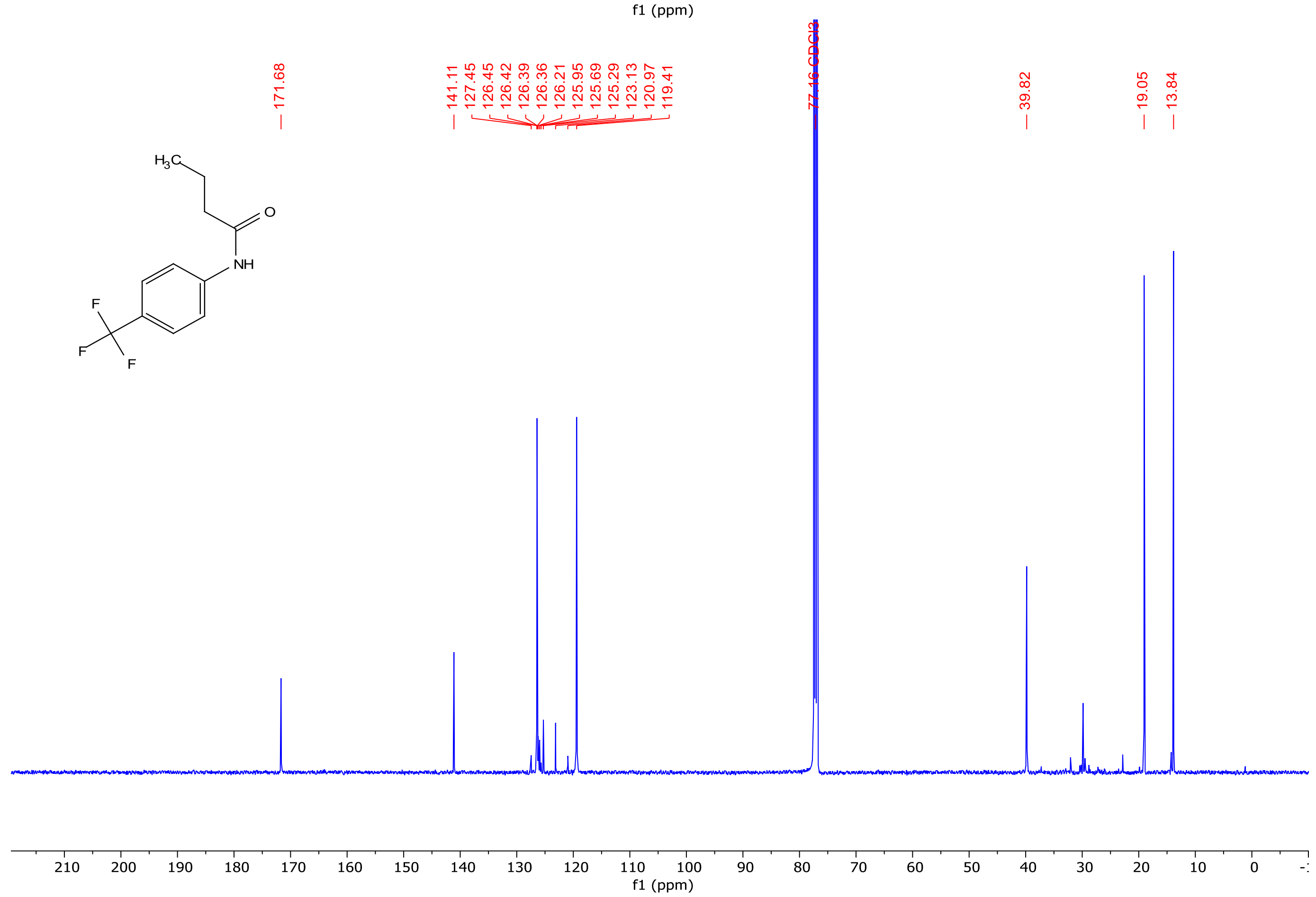

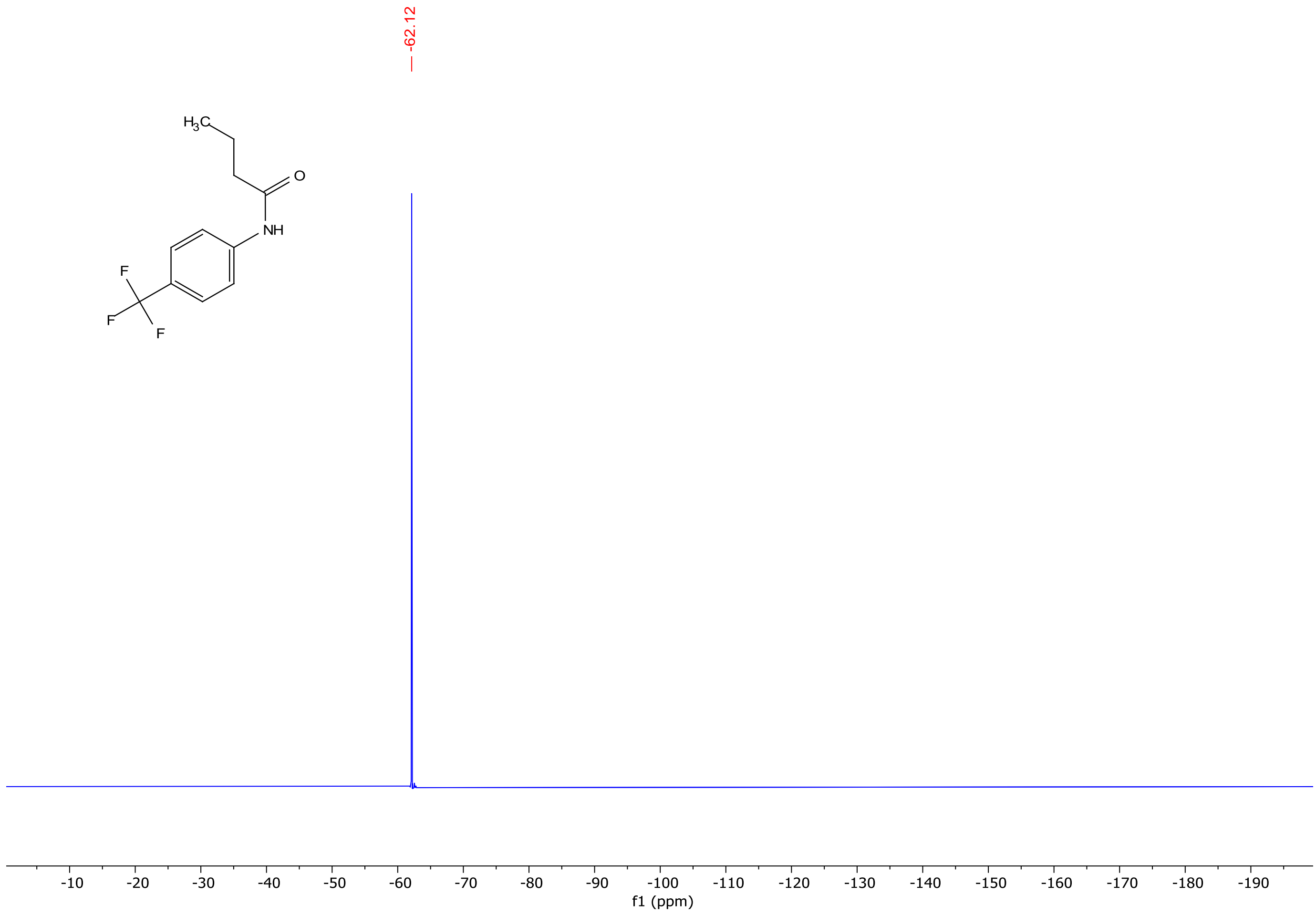
-62.12
H3C
O
NH
F
F
F
-10 -20 -30 -40 -50 -60 -70 -80 -90 -100 -110 -120 -130 -140 -150 -160 -170 -180 -190
f1 (ppm)

benzyl (4-(trifluoromethyl)phenyl)carbamate (68)

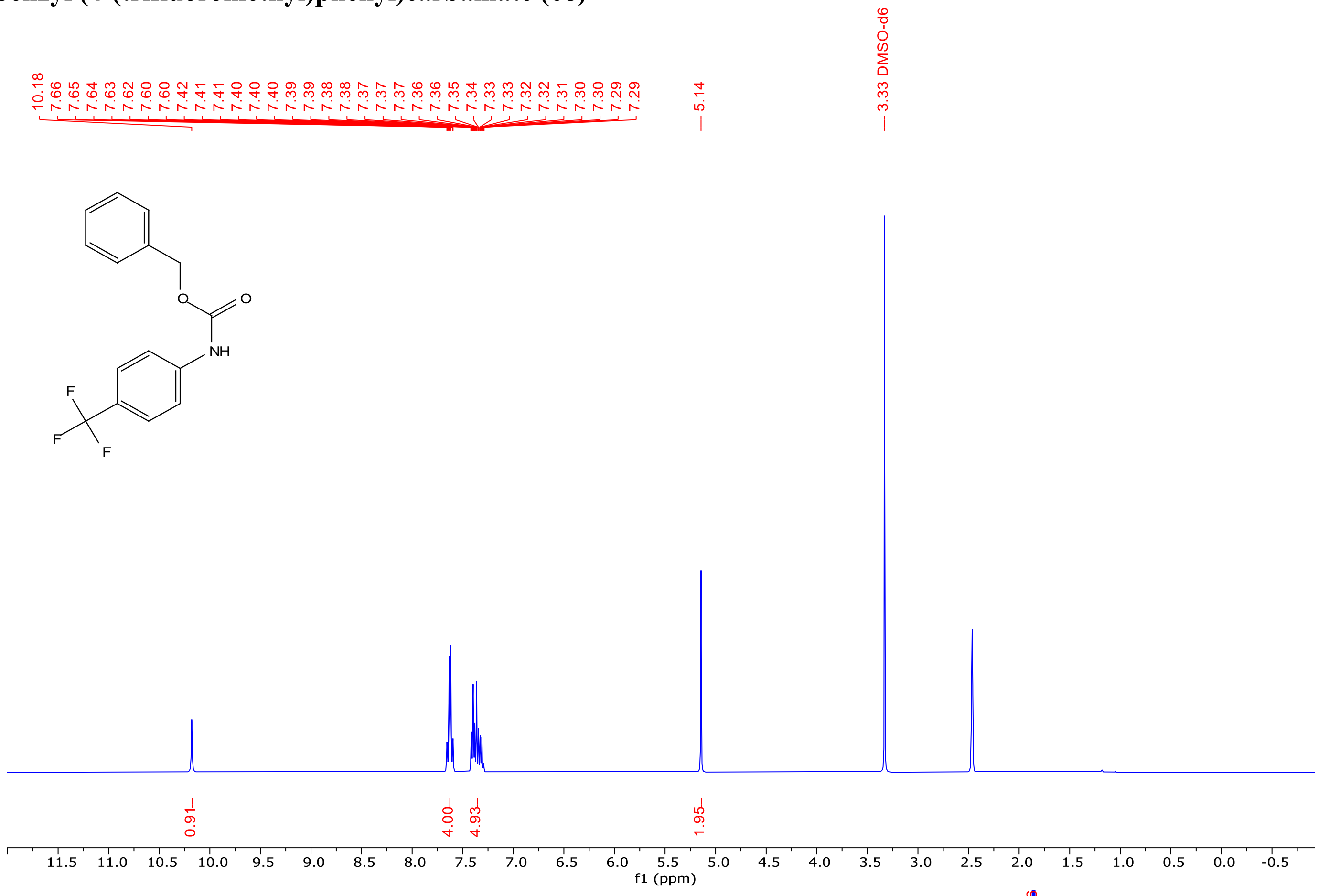

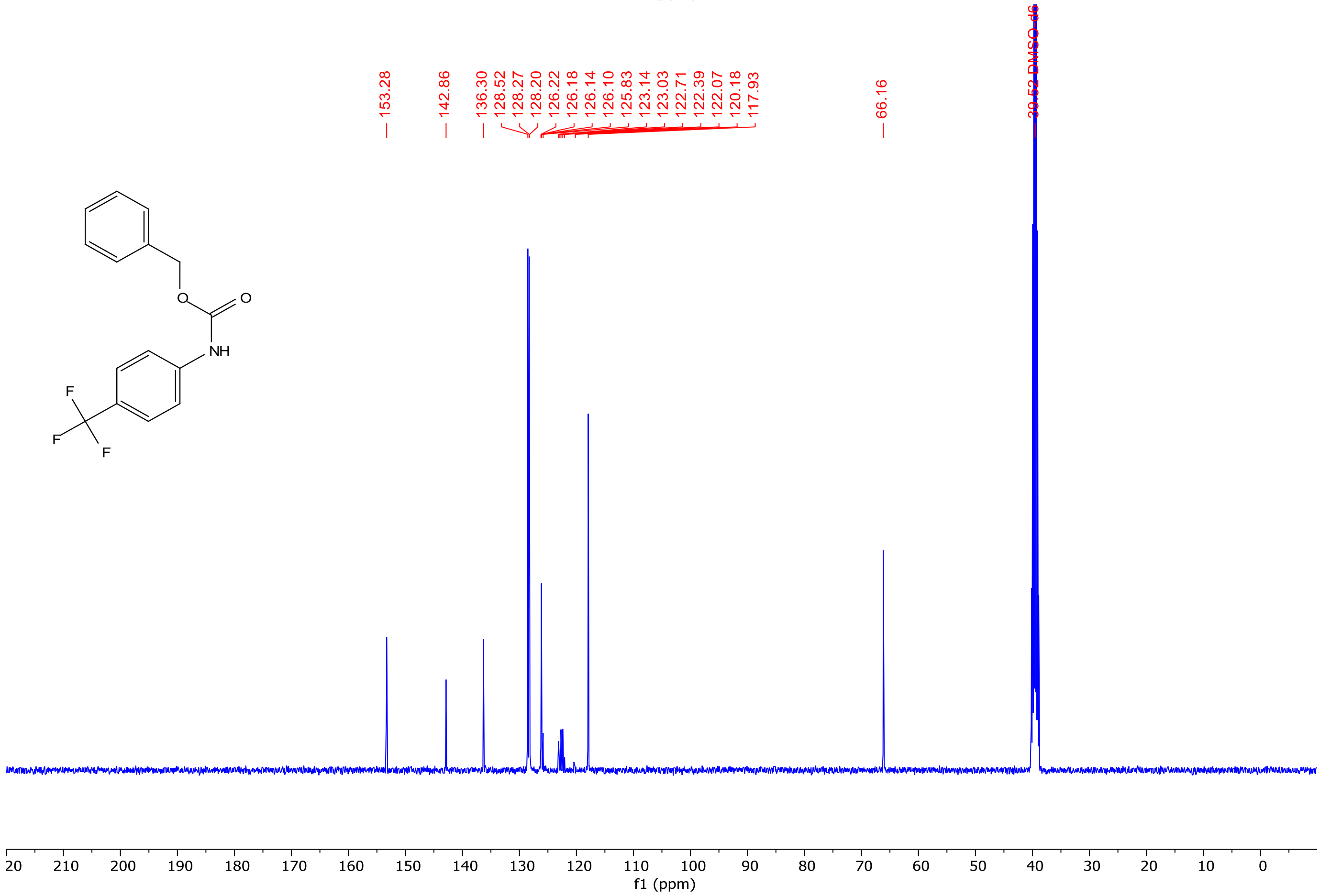

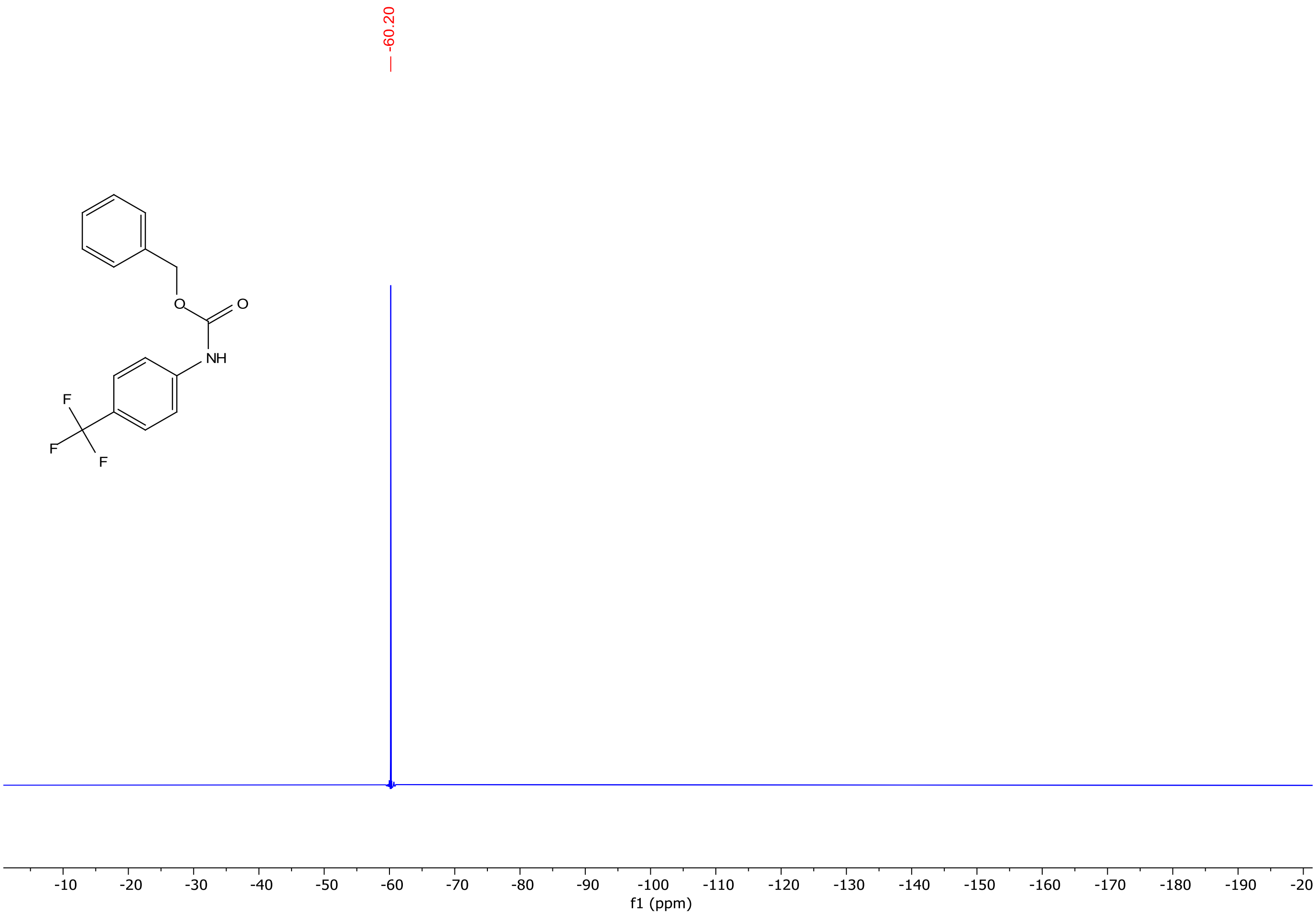
-60.20
O
O
NH
F
F
F
-10
-20
-30
-40
-50
-60
-70
-80
-90
-100
-110
-120
-130
-140
-150
-160
-170
-180
-190
-20
f1 (ppm)

methyl 4-((methoxycarbonyl)amino)benzoate (69)

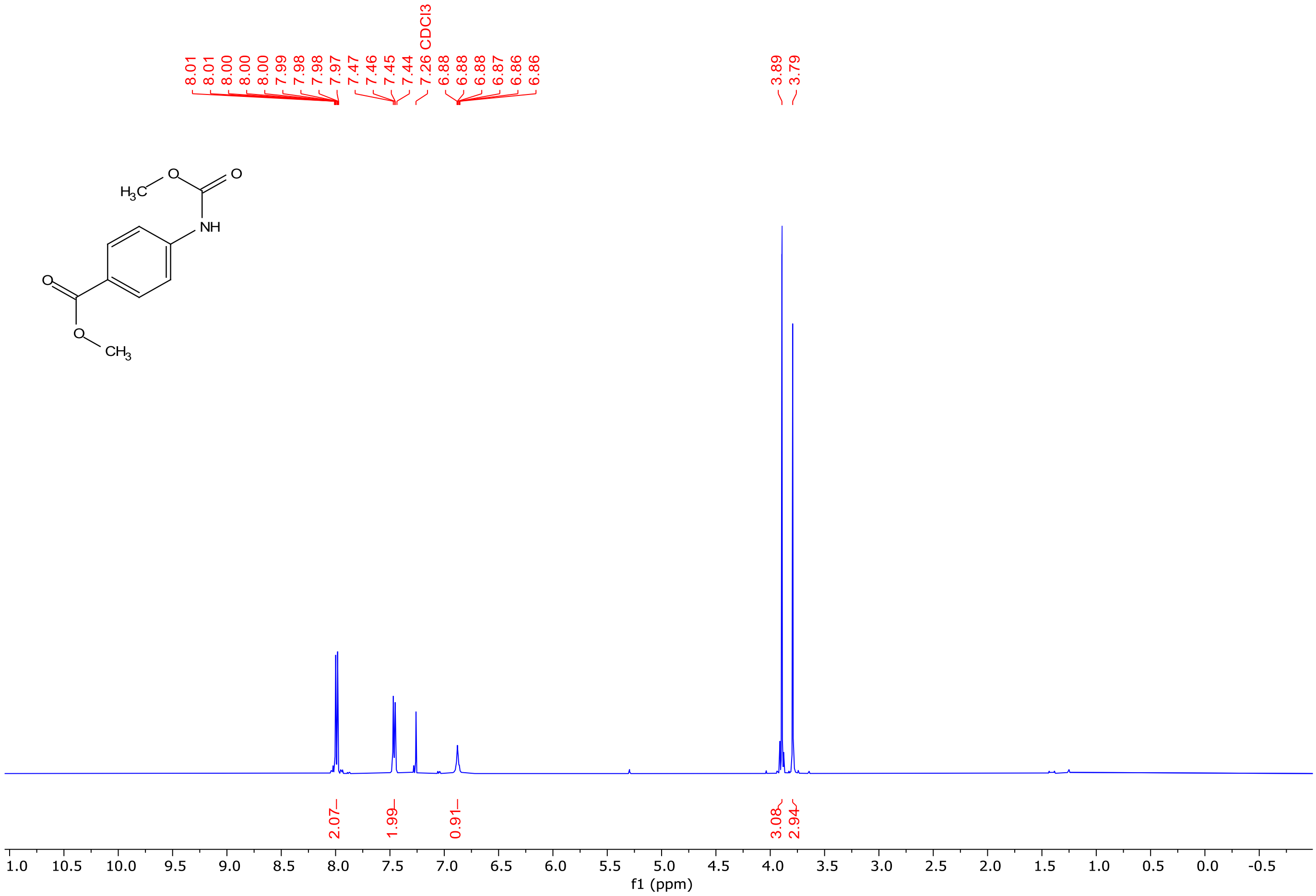

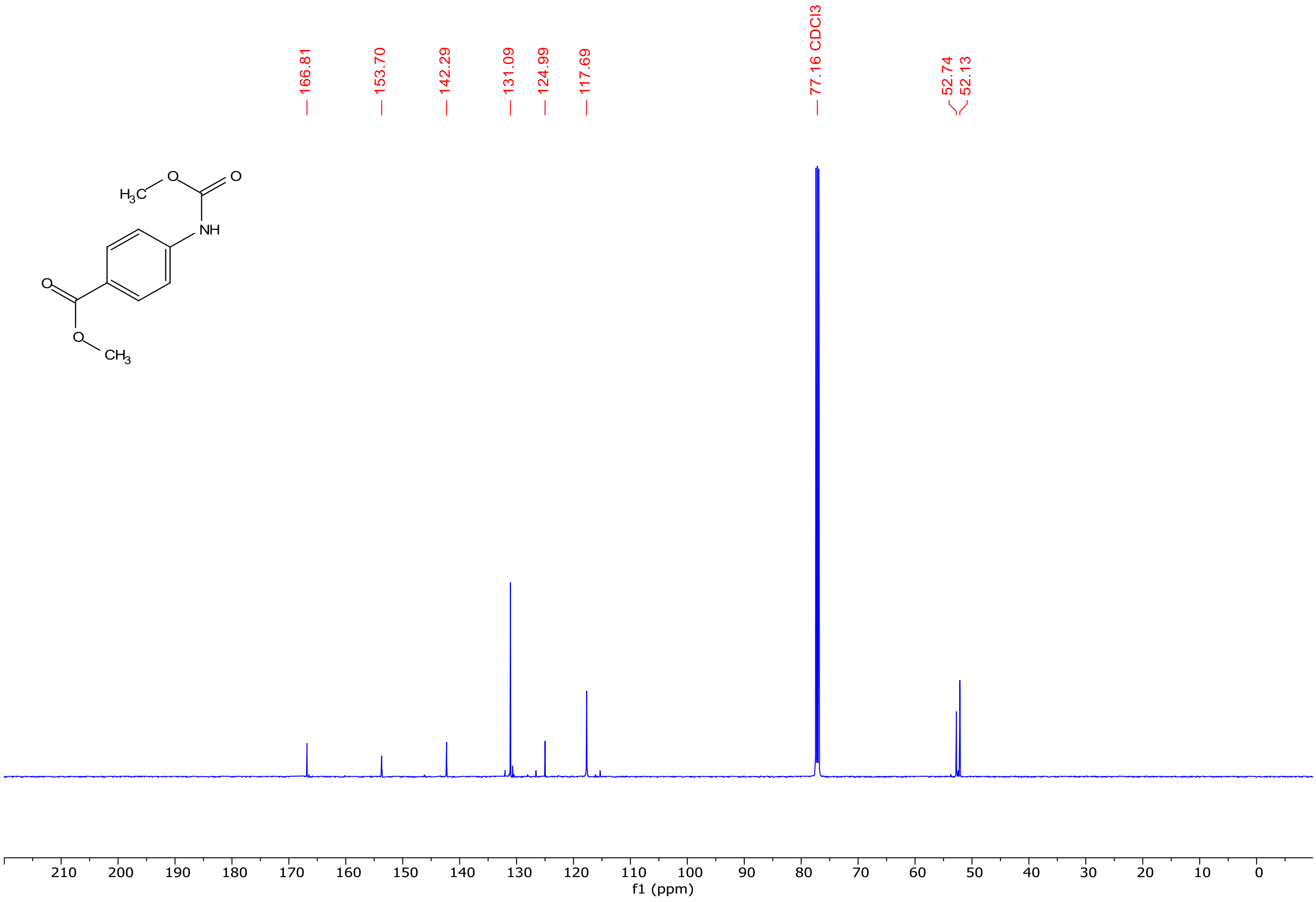

methyl 4-(2-oxooxazolidin-3-yl)benzoate (70)

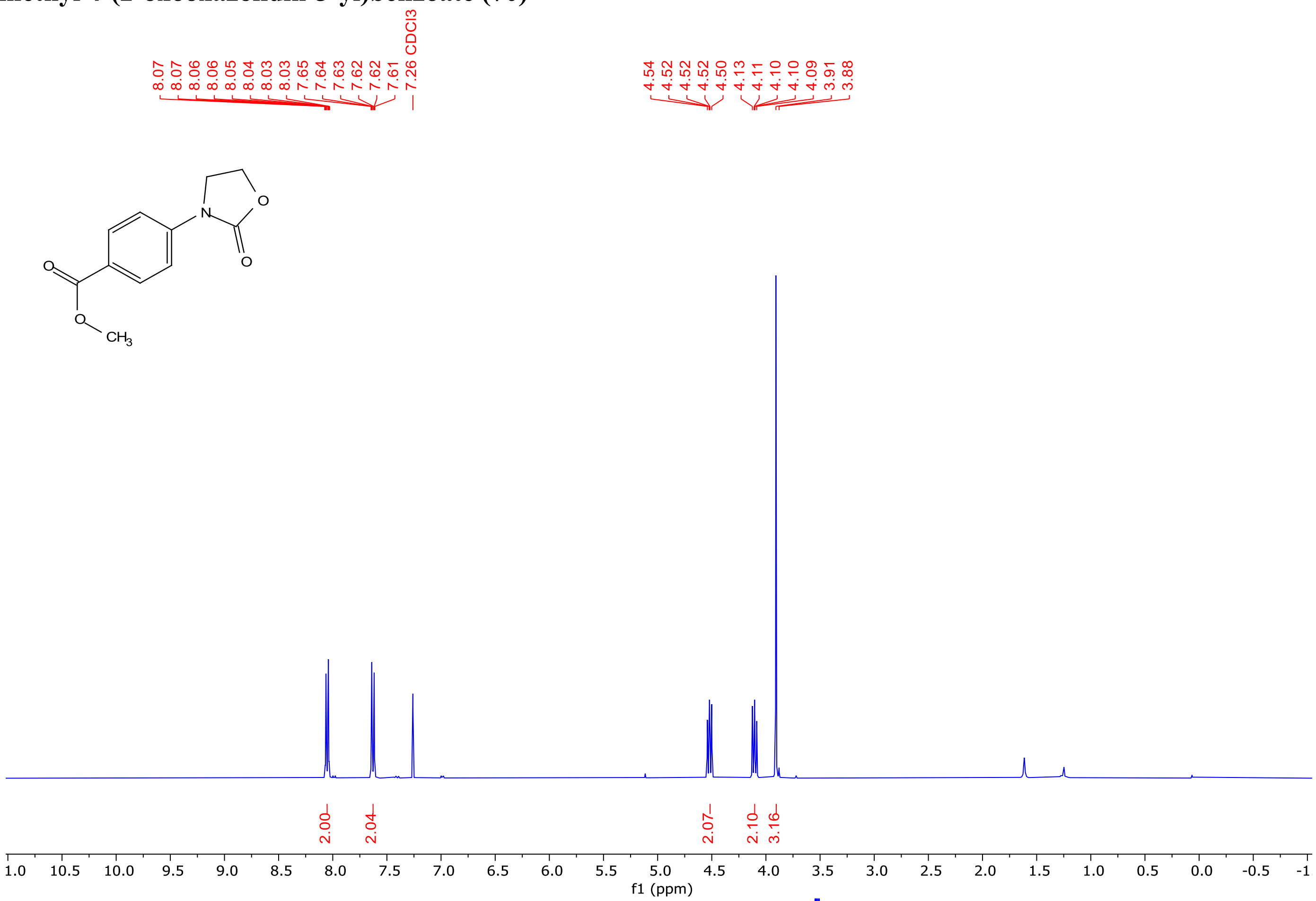

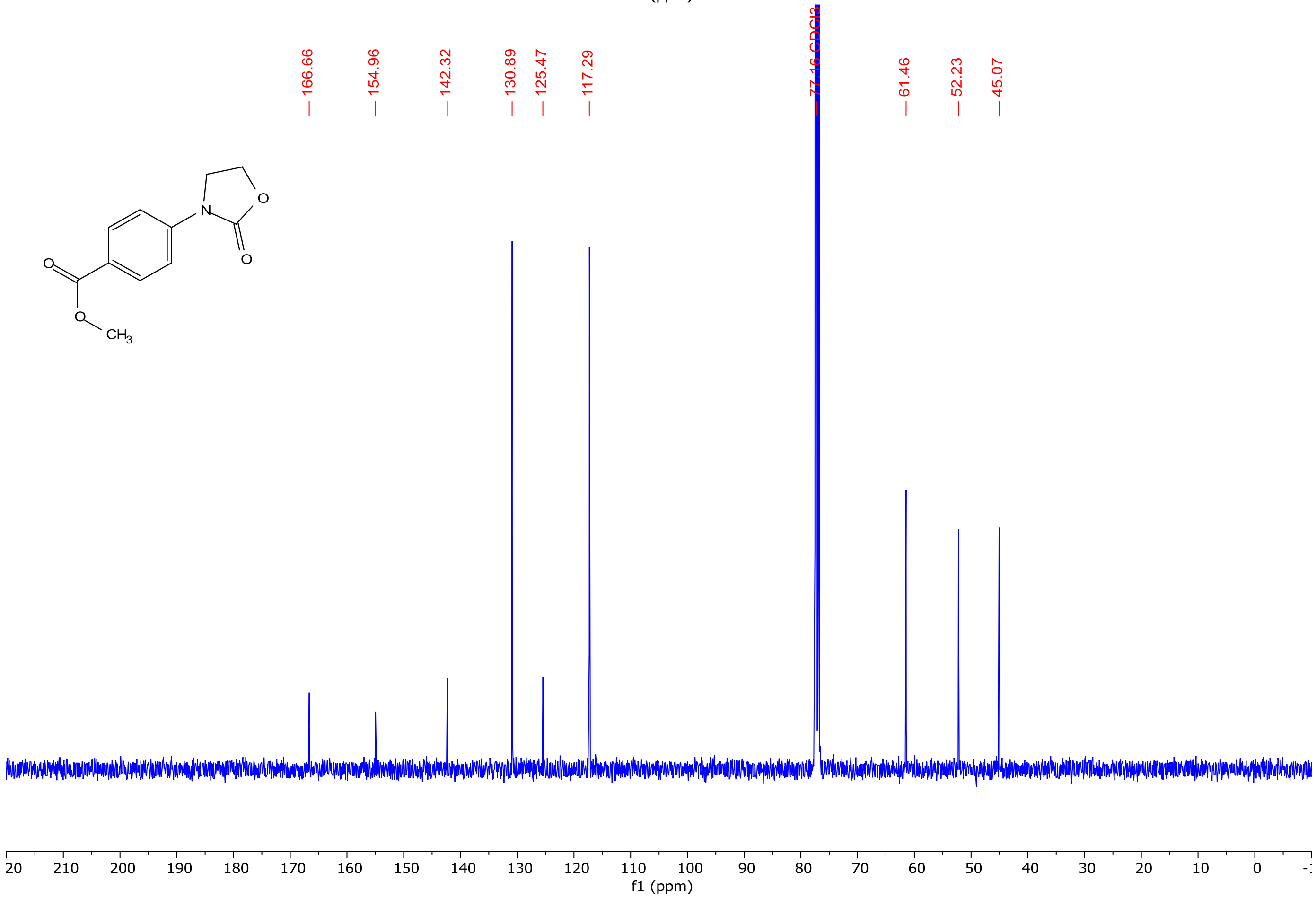

P,P-diphenyl-N-(4-(trifluoromethyl)phenyl)phosphinic amide (71)

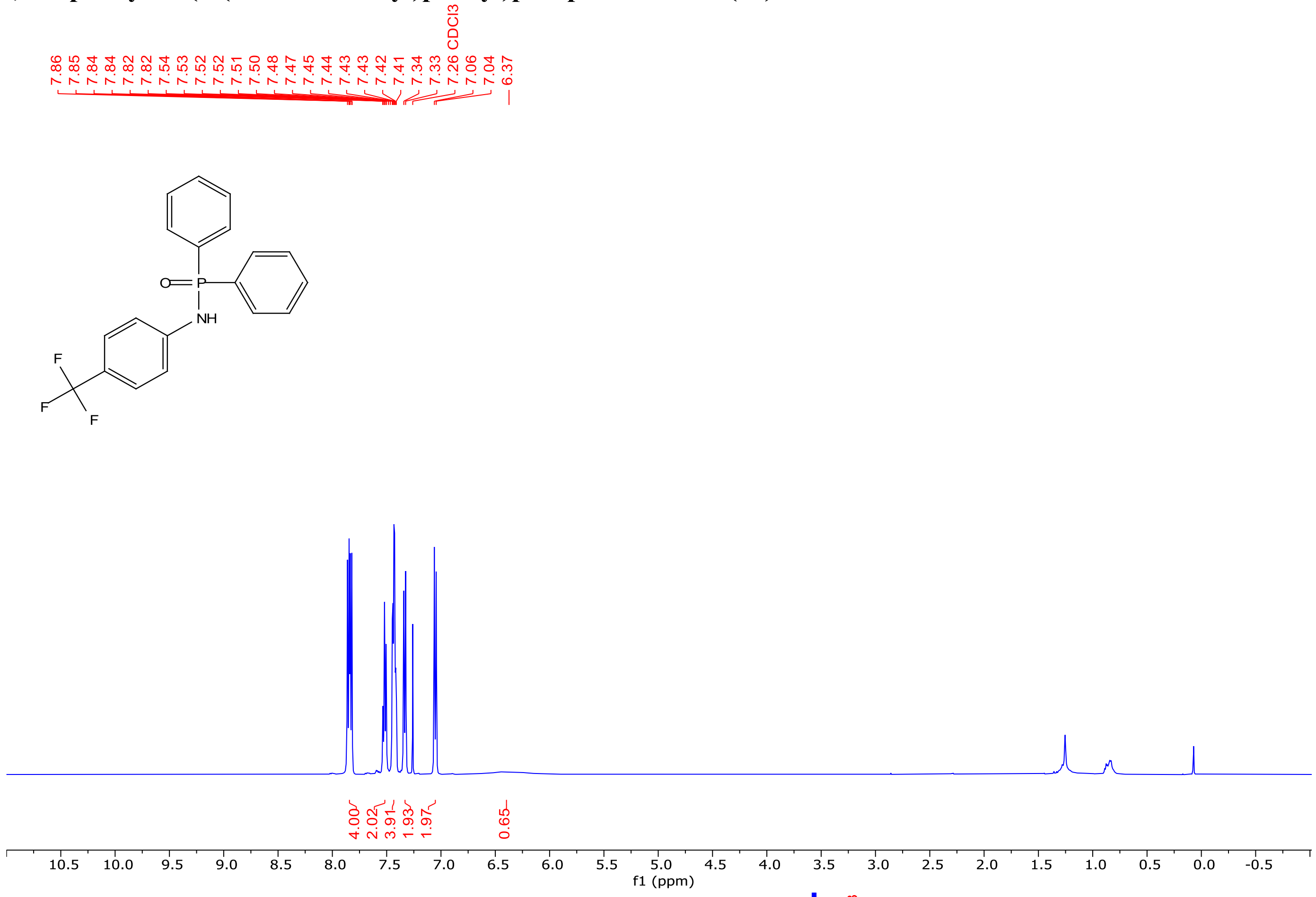

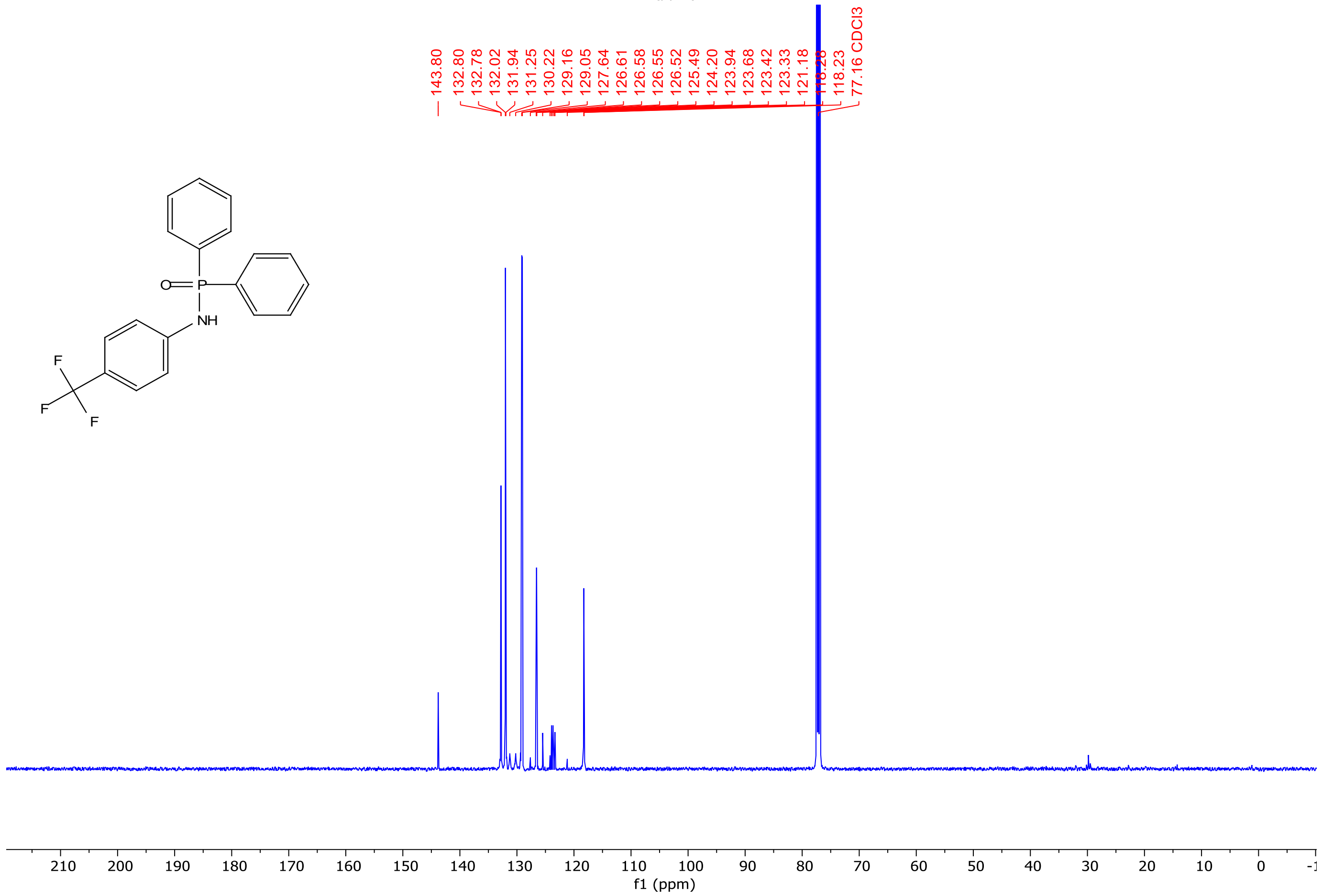

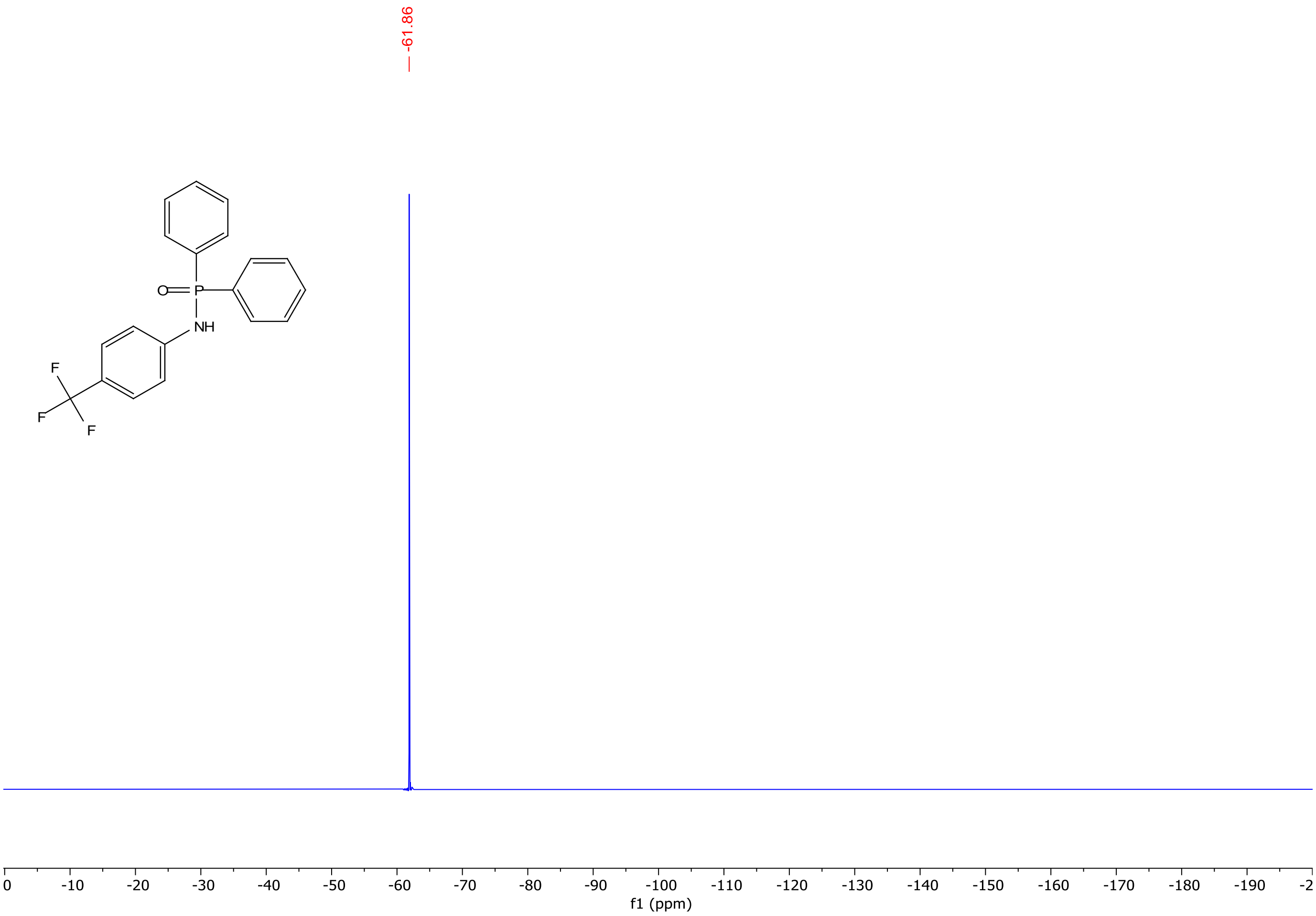
-61.86
O
P
NH
F
F
F
0
-10
-20
-30
-40
-50
-60
-70
-80
-90
-100
-110
-120
-130
-140
-150
-160
-170
-180
-190
f1 (ppm)

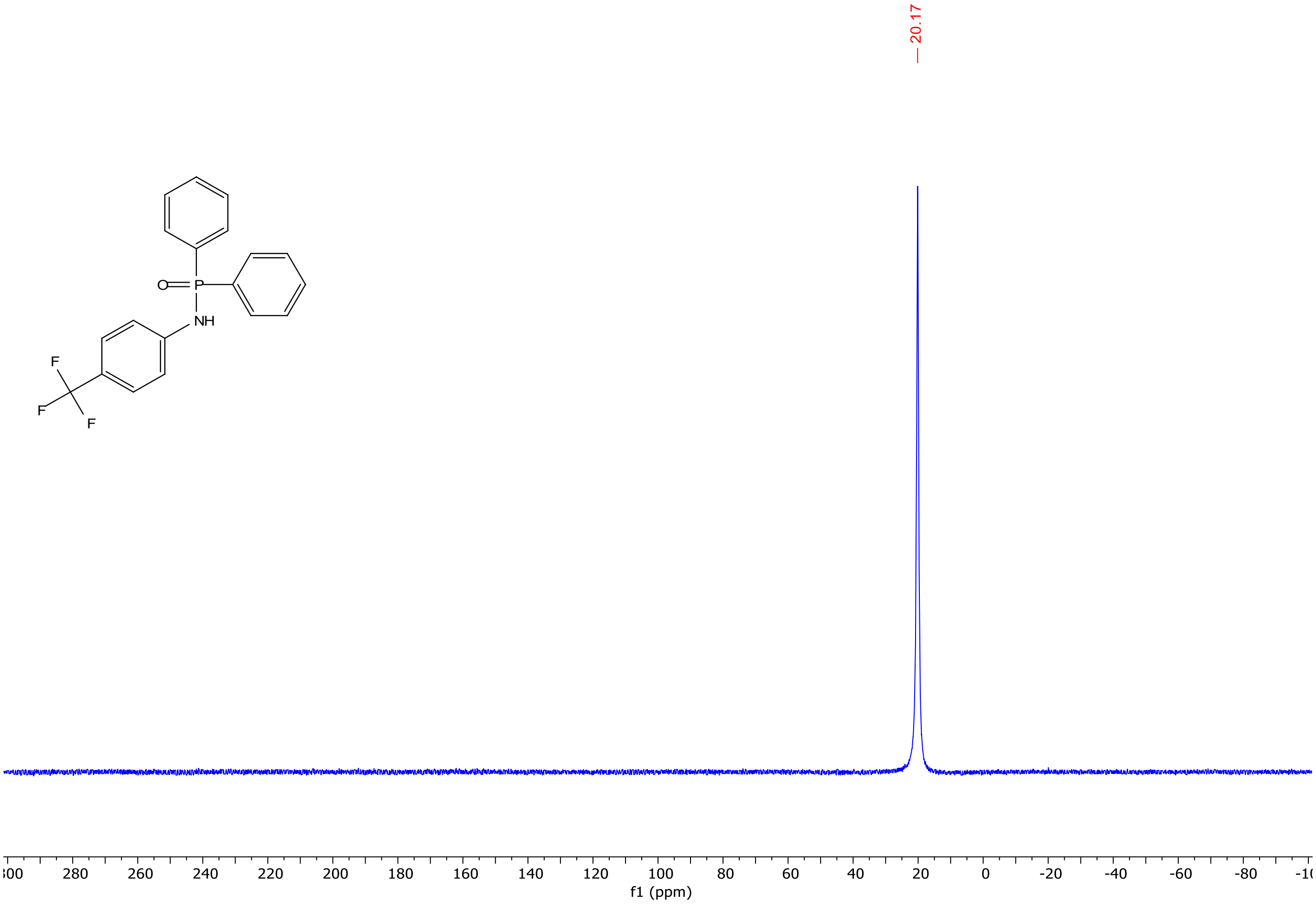
20.17
O
P
NH
F
F
F
280
260
240
220
200
180
160
140
120
100
80
60
40
20
0
-20
-40
-60
-80
f1 (ppm)

methyl 4-((diphenylphosphoryl)amino)benzoate (72)

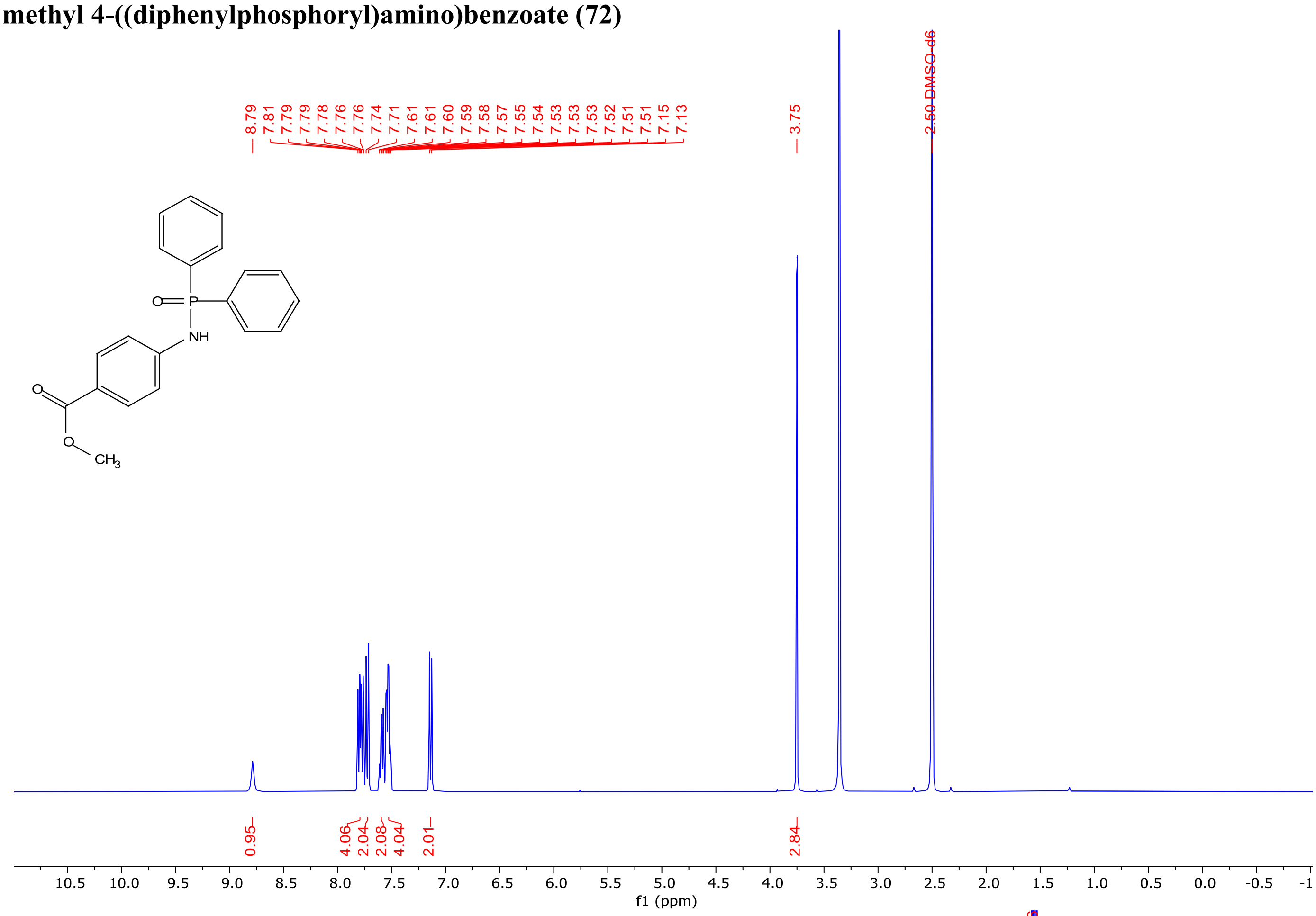

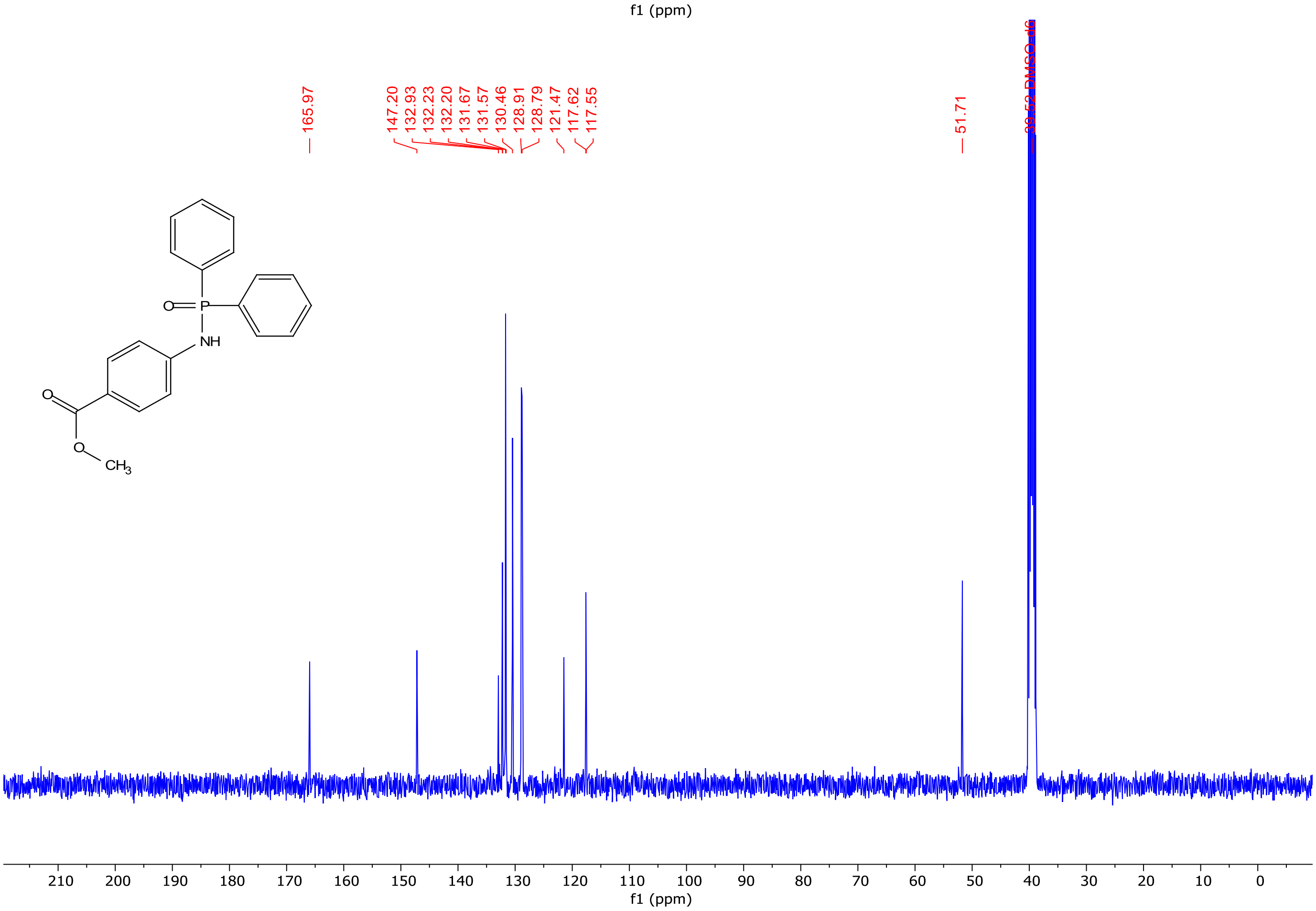

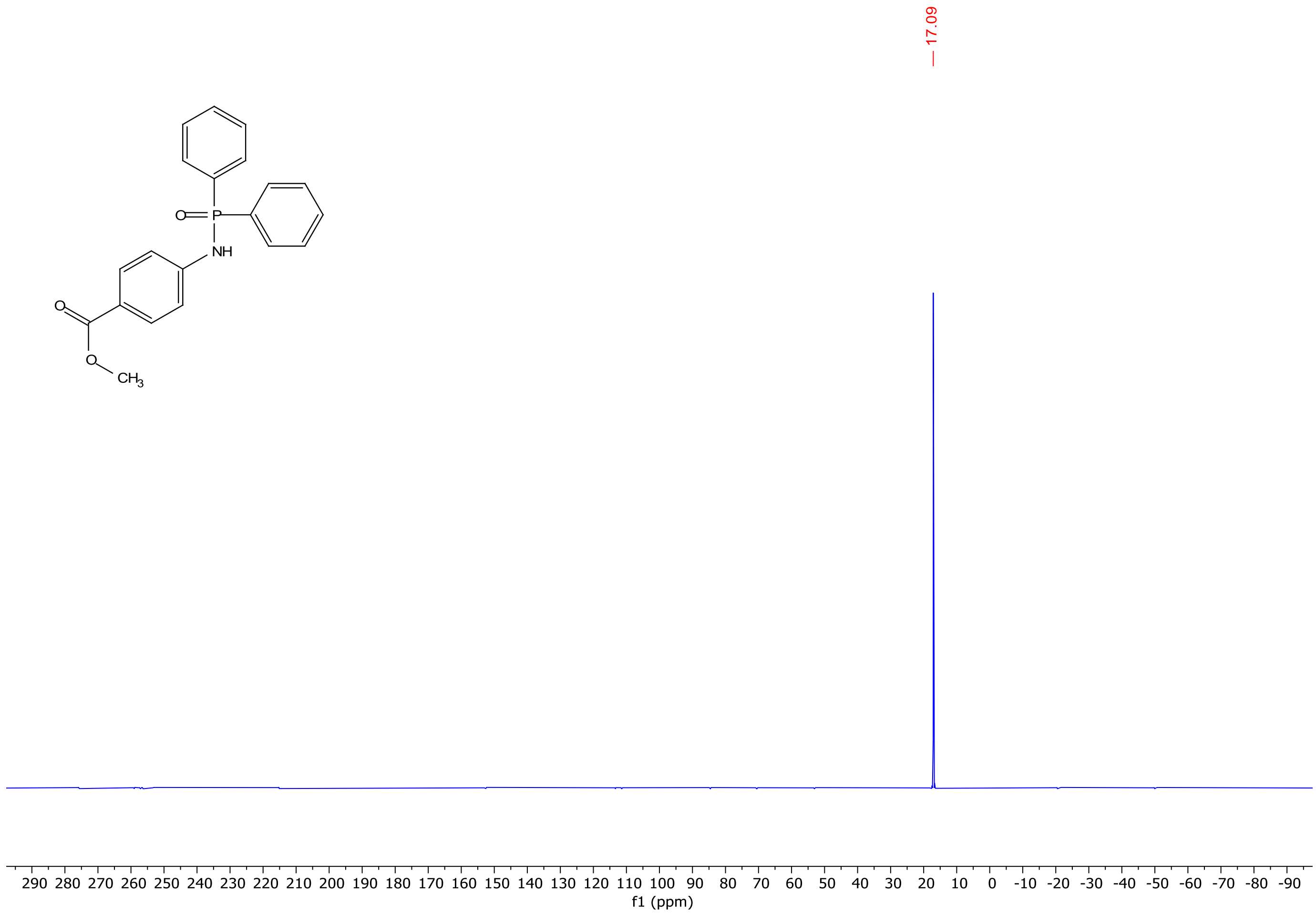
17.09
O=P
NH
O
O
CH3
290 280 270 260 250 240 230 220 210 200 190 180 170 160 150 140 130 120 110 100 90 80 70 60 50 40 30 20 10 0 -10 -20 -30 -40 -50 -60 -70 -80 -90
f1 (ppm)

1-(4-(trifluoromethyl)phenyl)indoline (73)

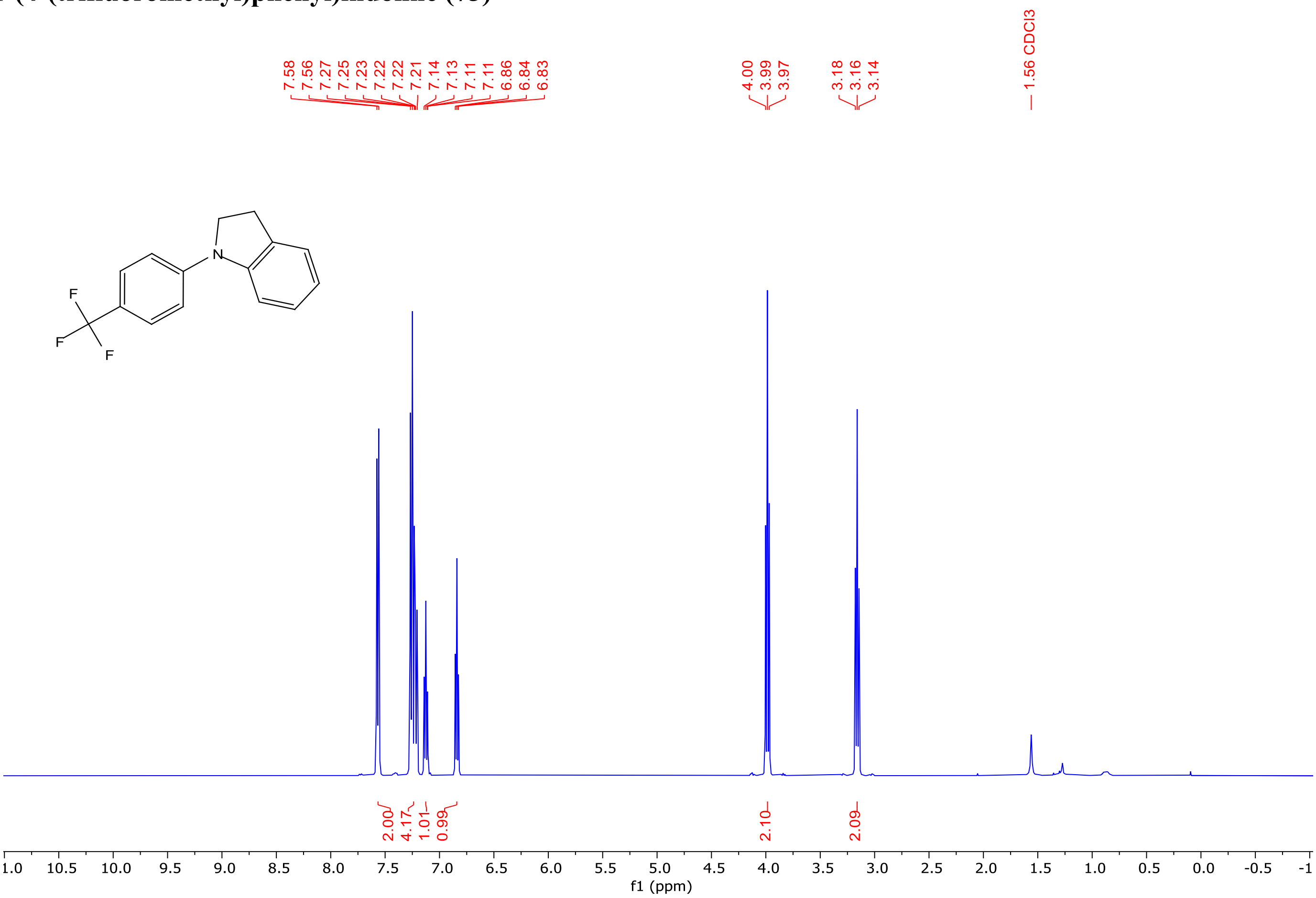

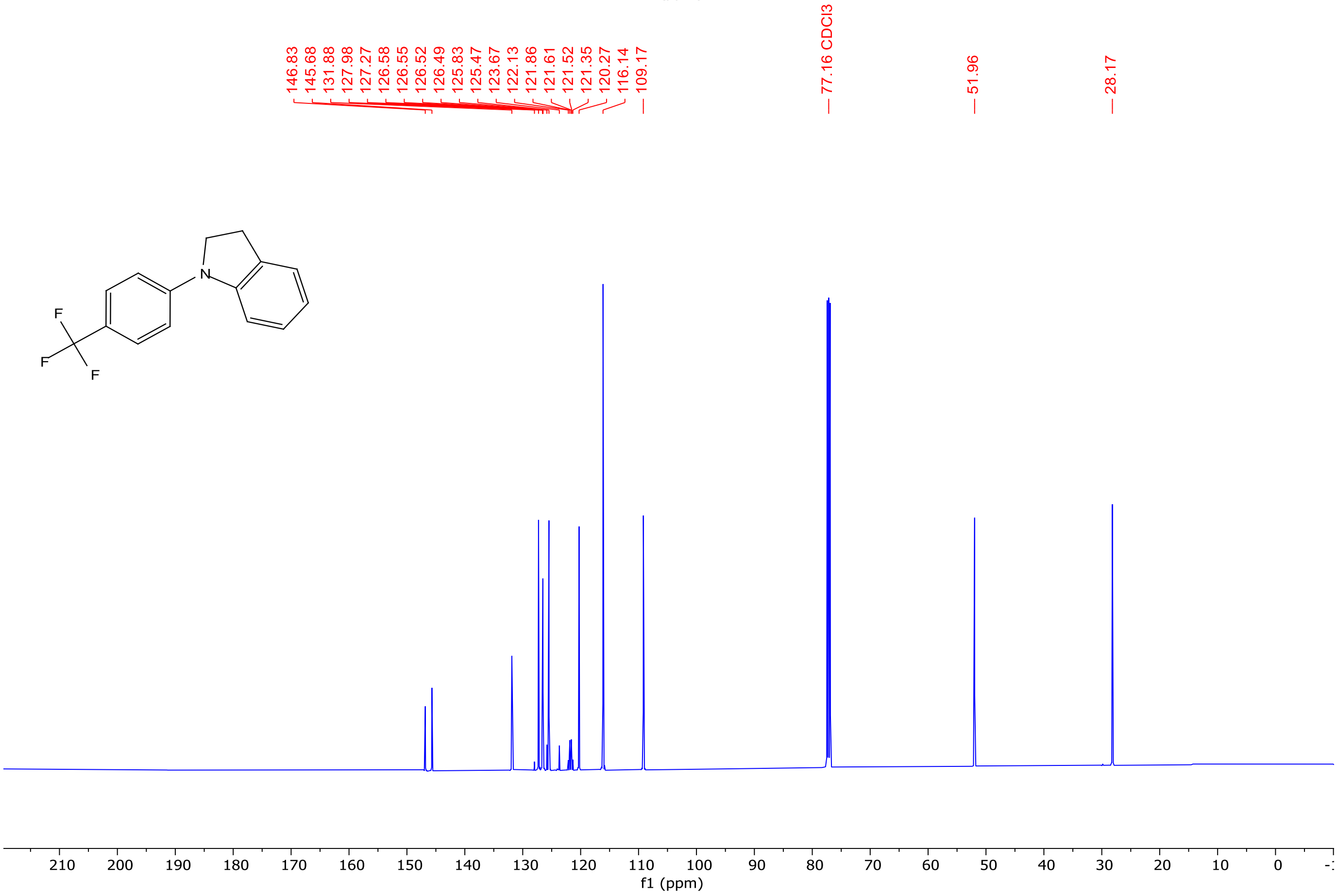

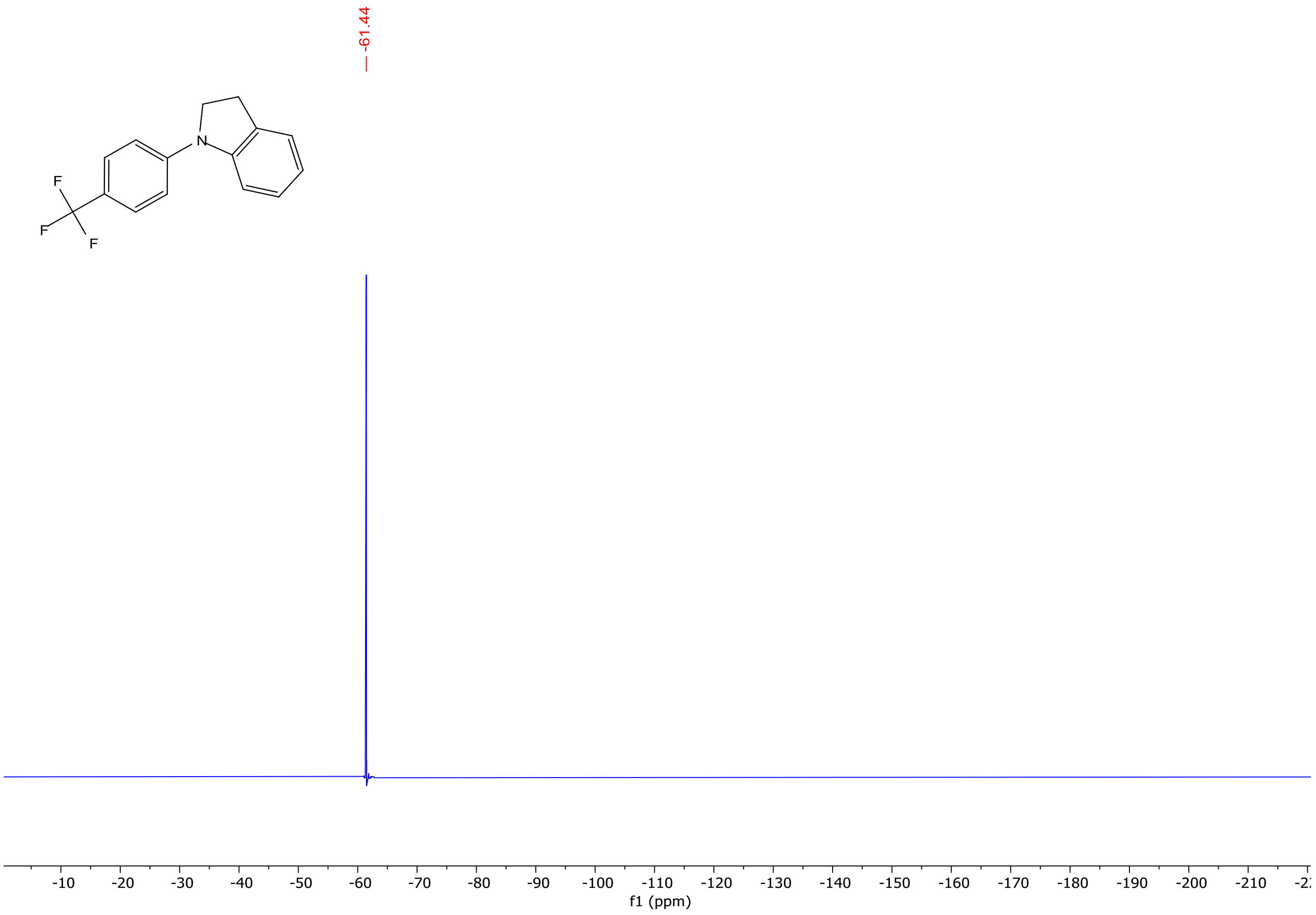
-61.44
F
F
F
N
-10 -20 -30 -40 -50 -60 -70 -80 -90 -100 -110 -120 -130 -140 -150 -160 -170 -180 -190 -200 -210
f1 (ppm)

4-(9*H*-carbazol-9-yl)benzonitrile (74)

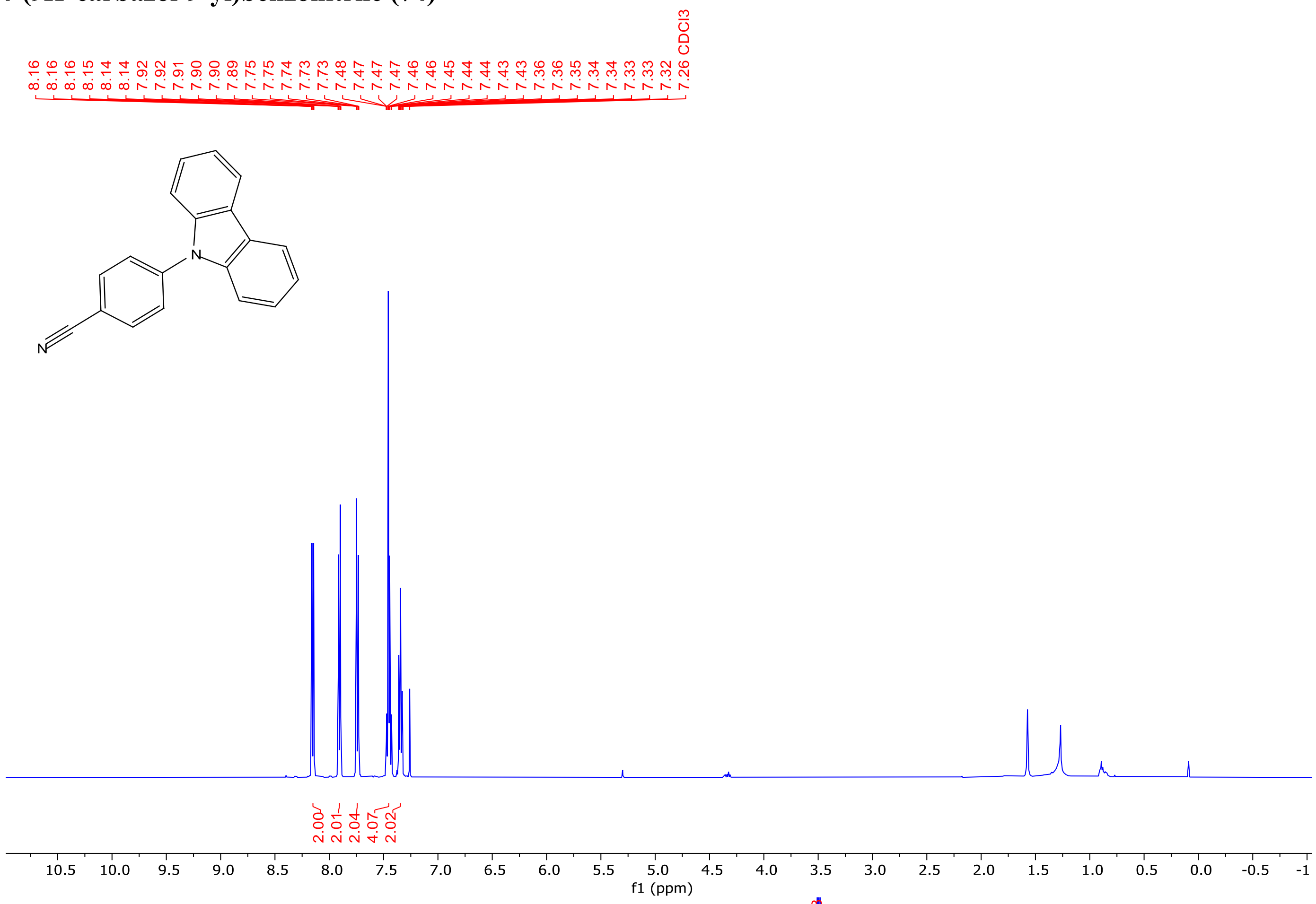

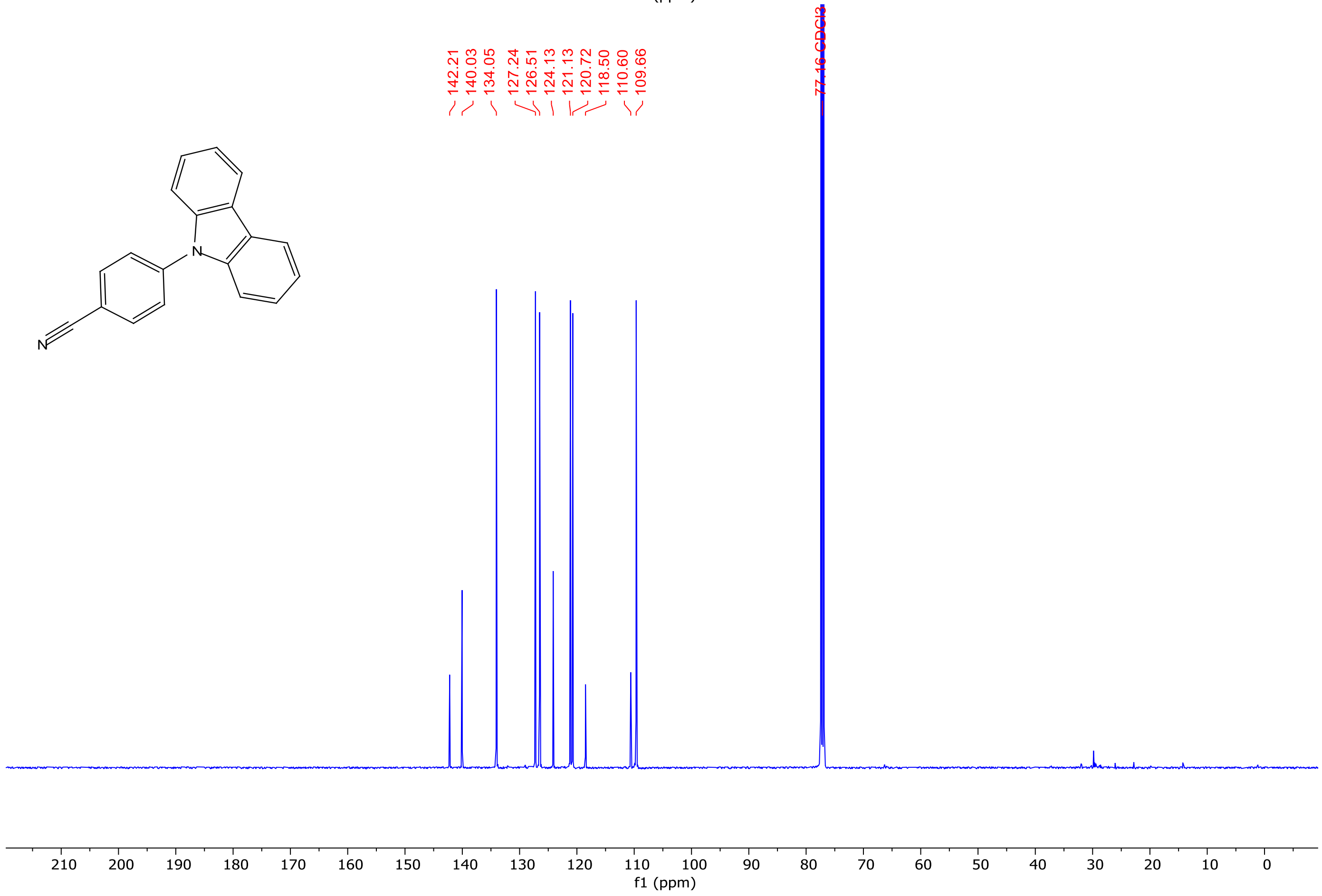

4-(5-methoxy-1H-indol-1-yl)benzonitrile (75)

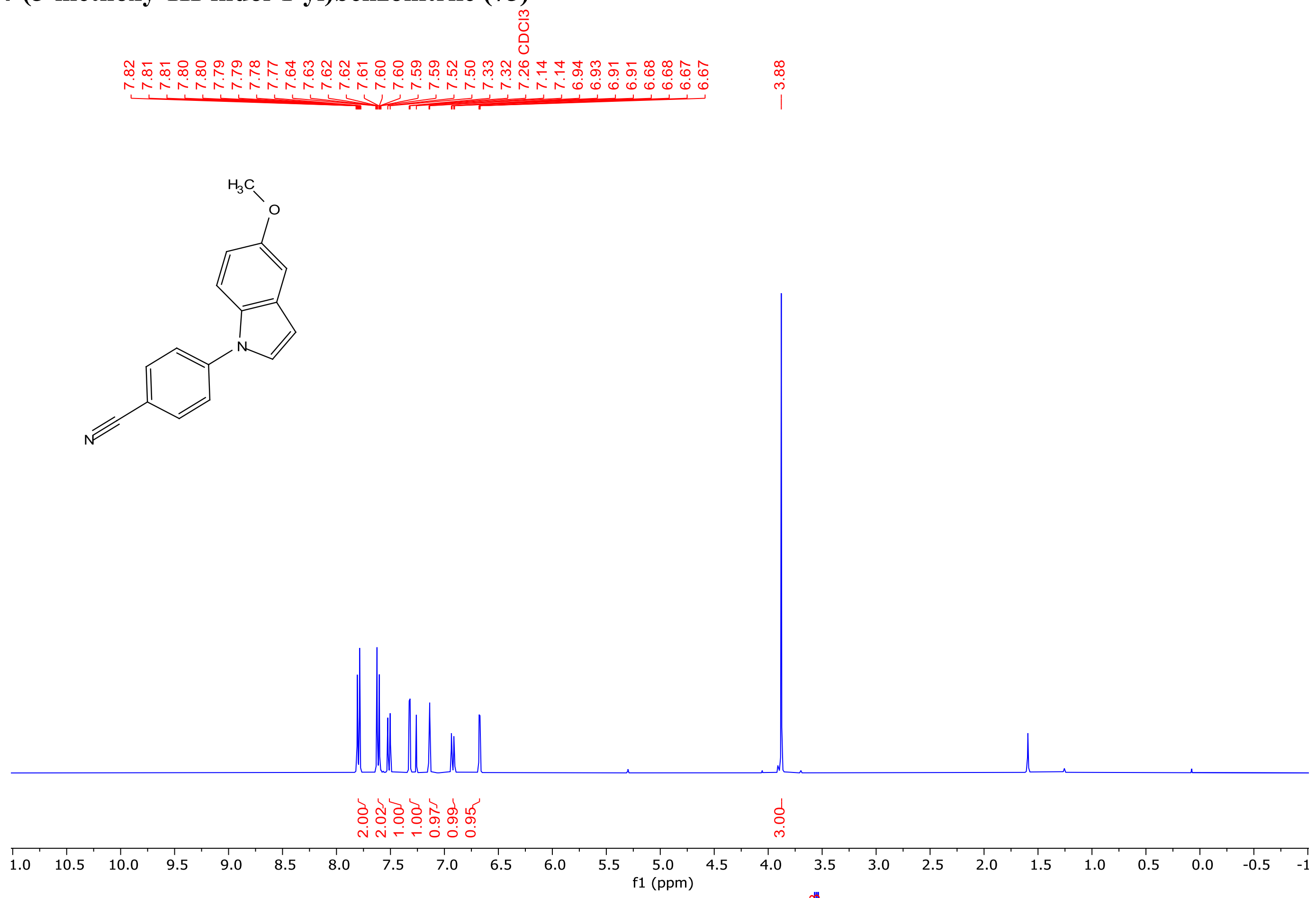

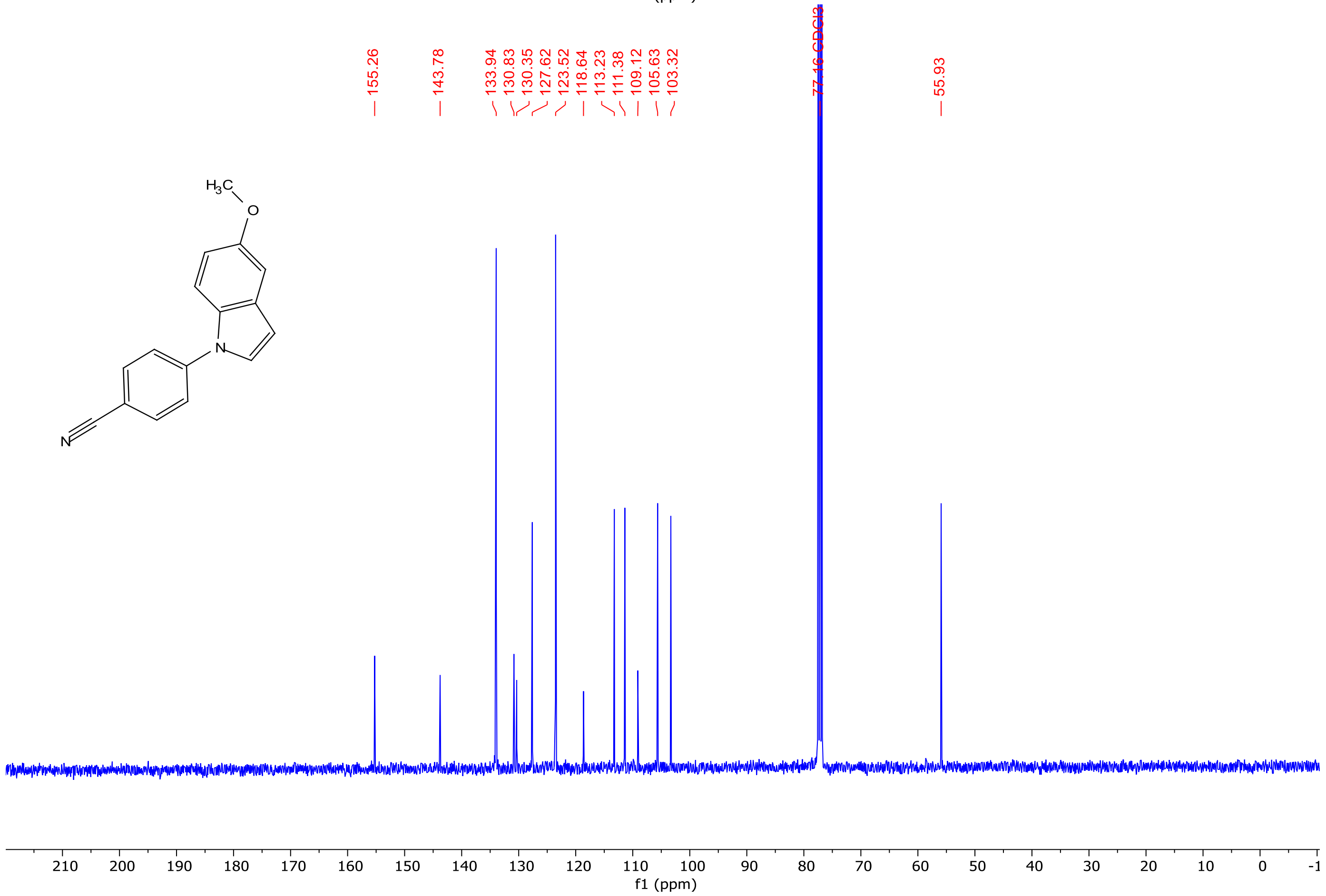

4-(1*H*-benzo[d]imidazol-1-yl)benzonitrile (76)

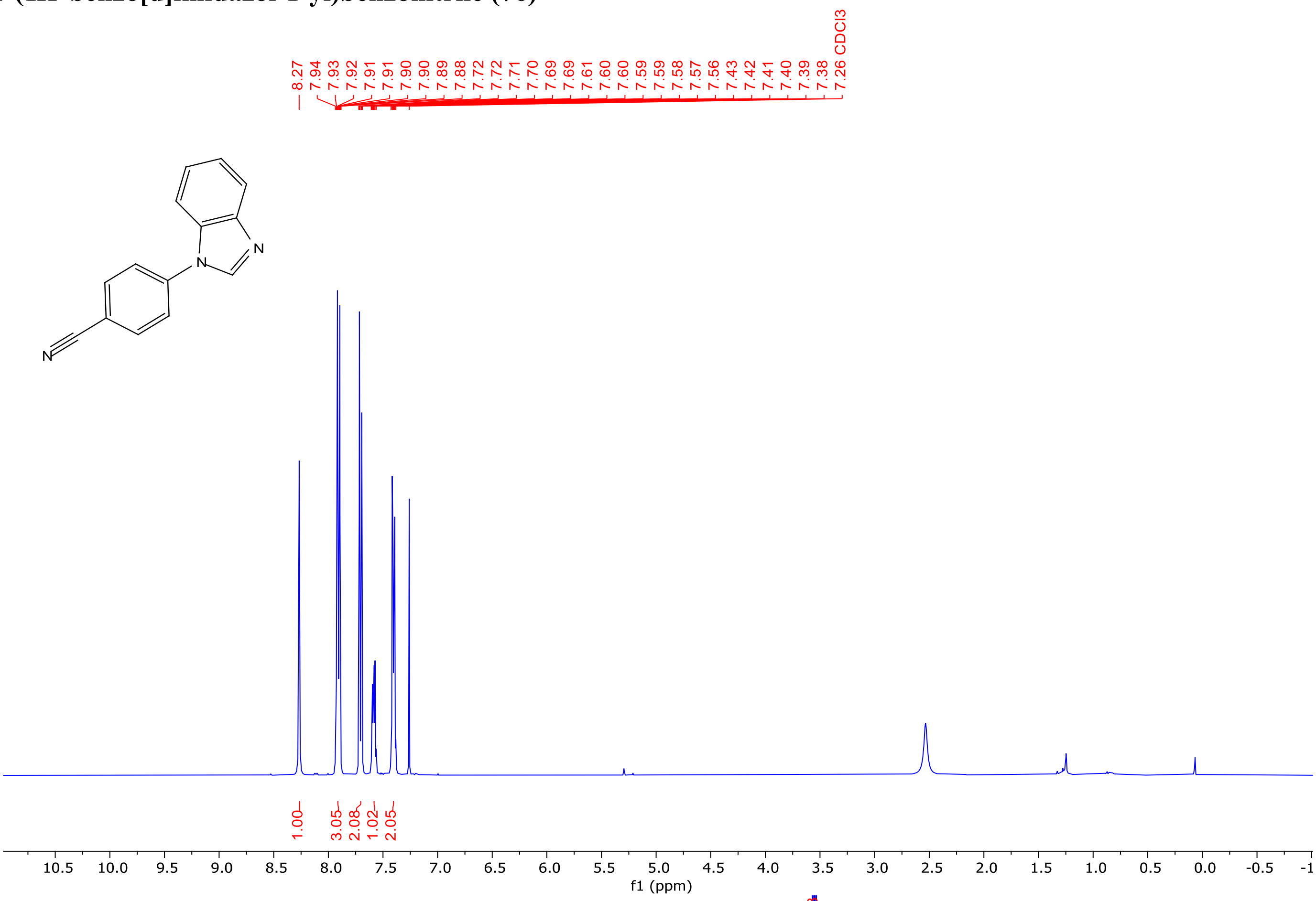

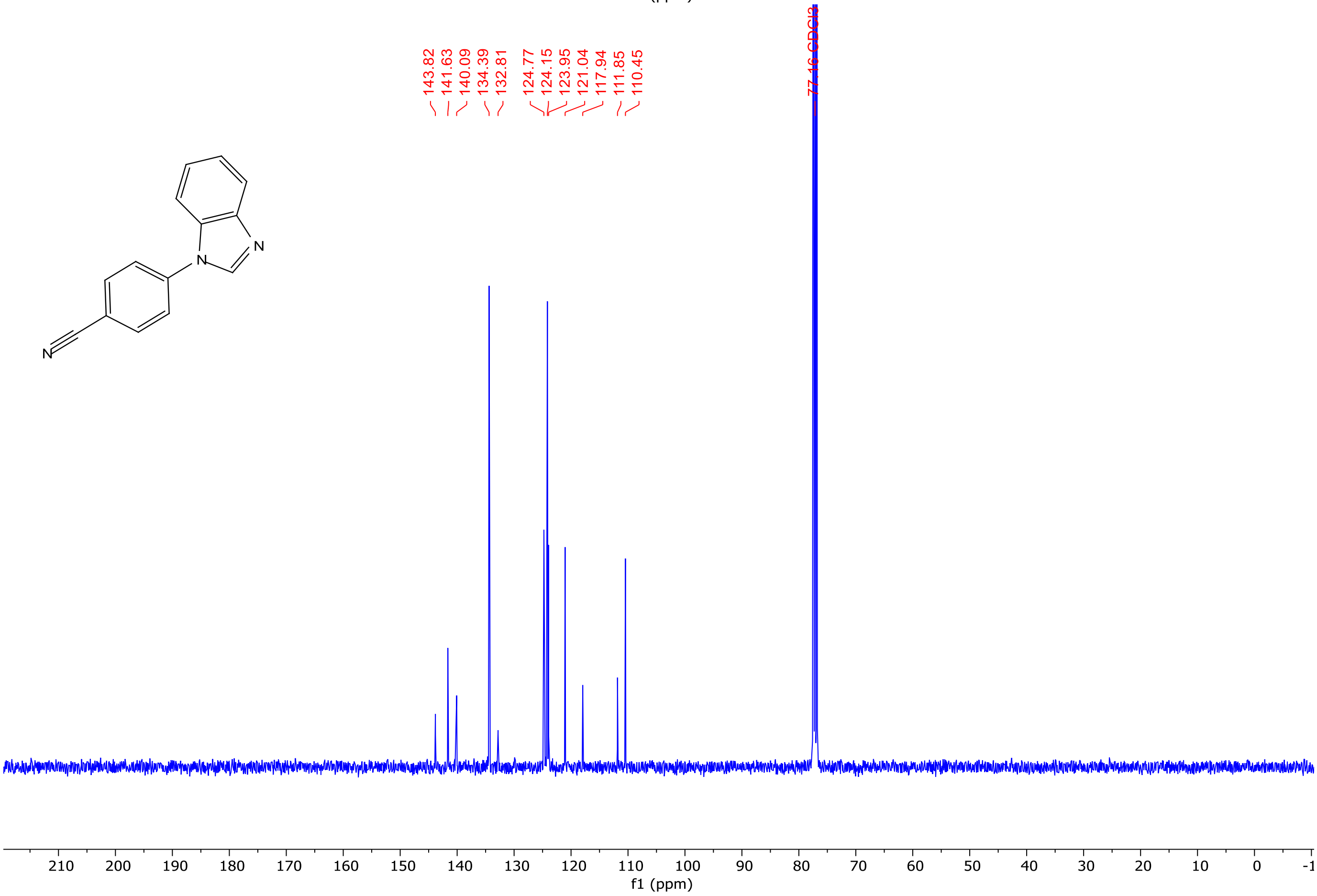

4-(((4-methoxyphenyl)(methyl)amino)methyl)benzonitrile (77)

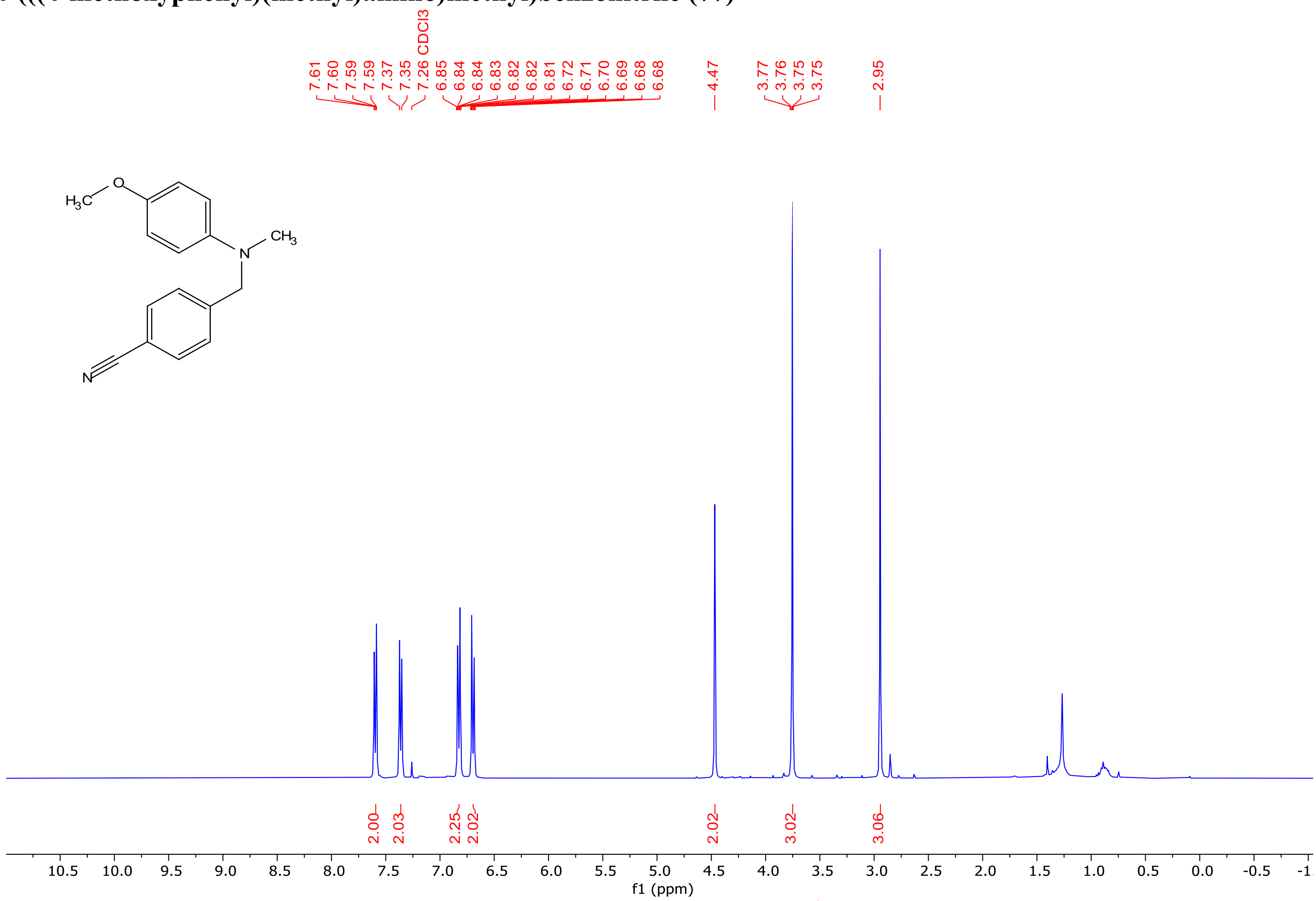

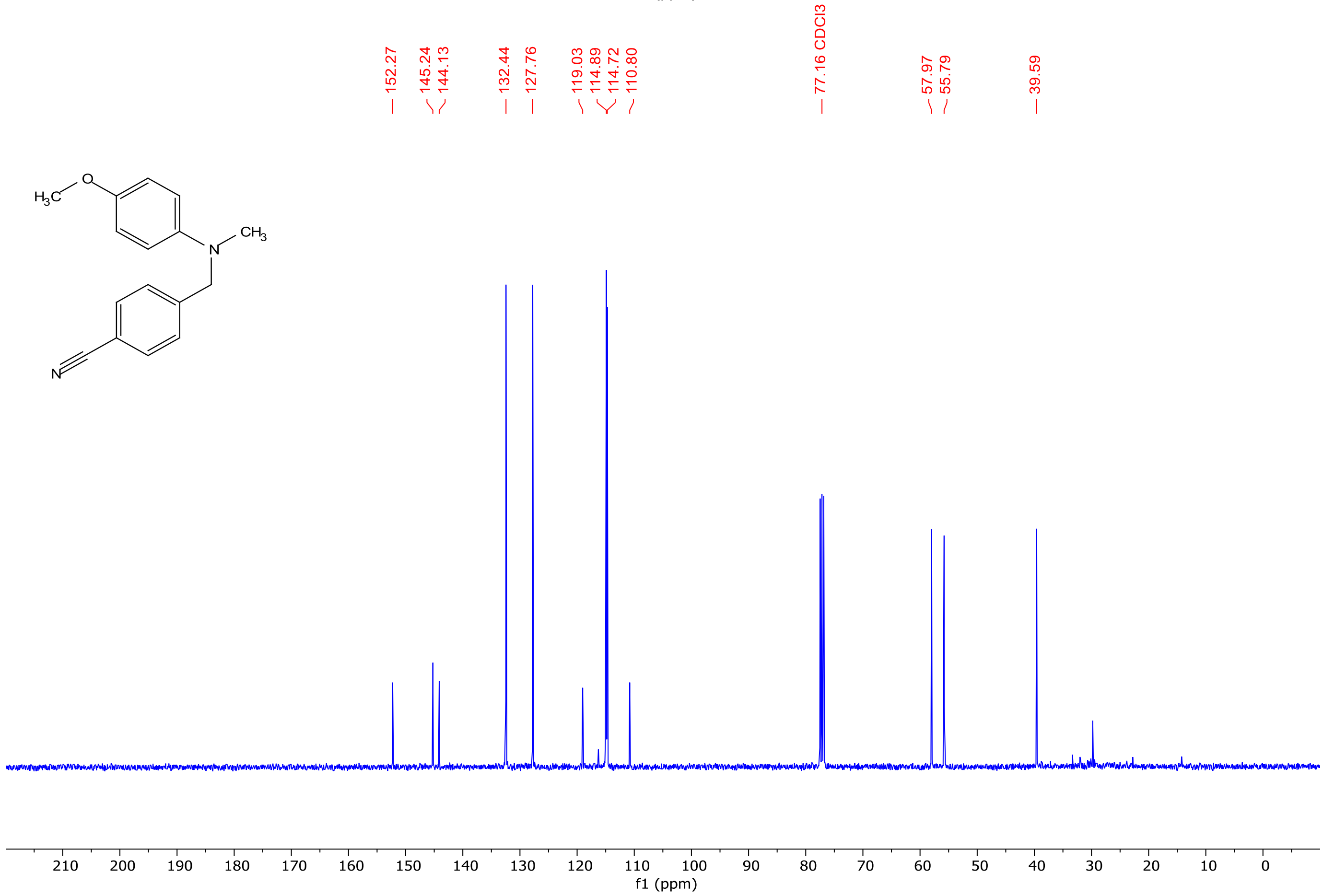

methyl [1,1'-biphenyl]-4-carboxylate (78)

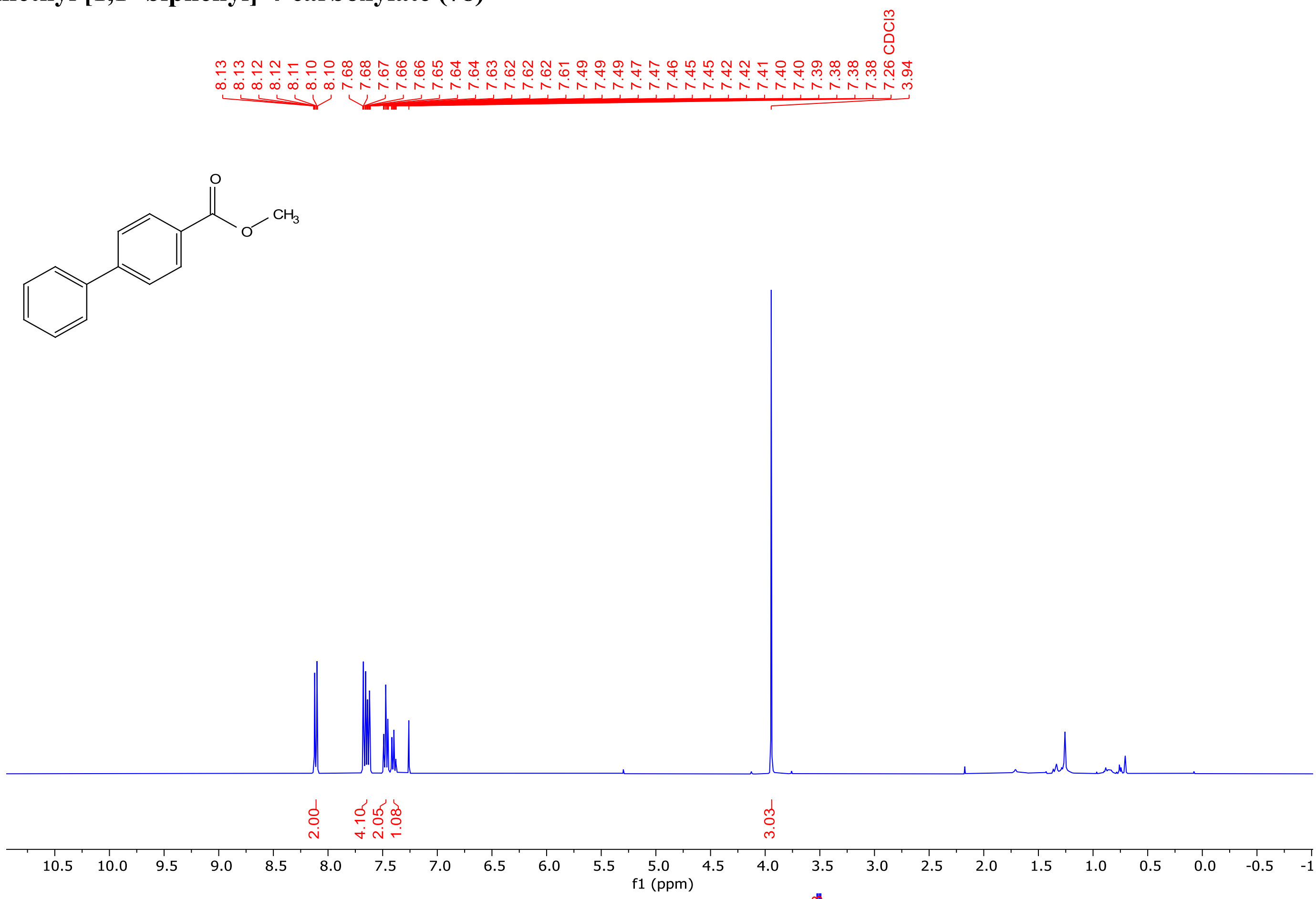

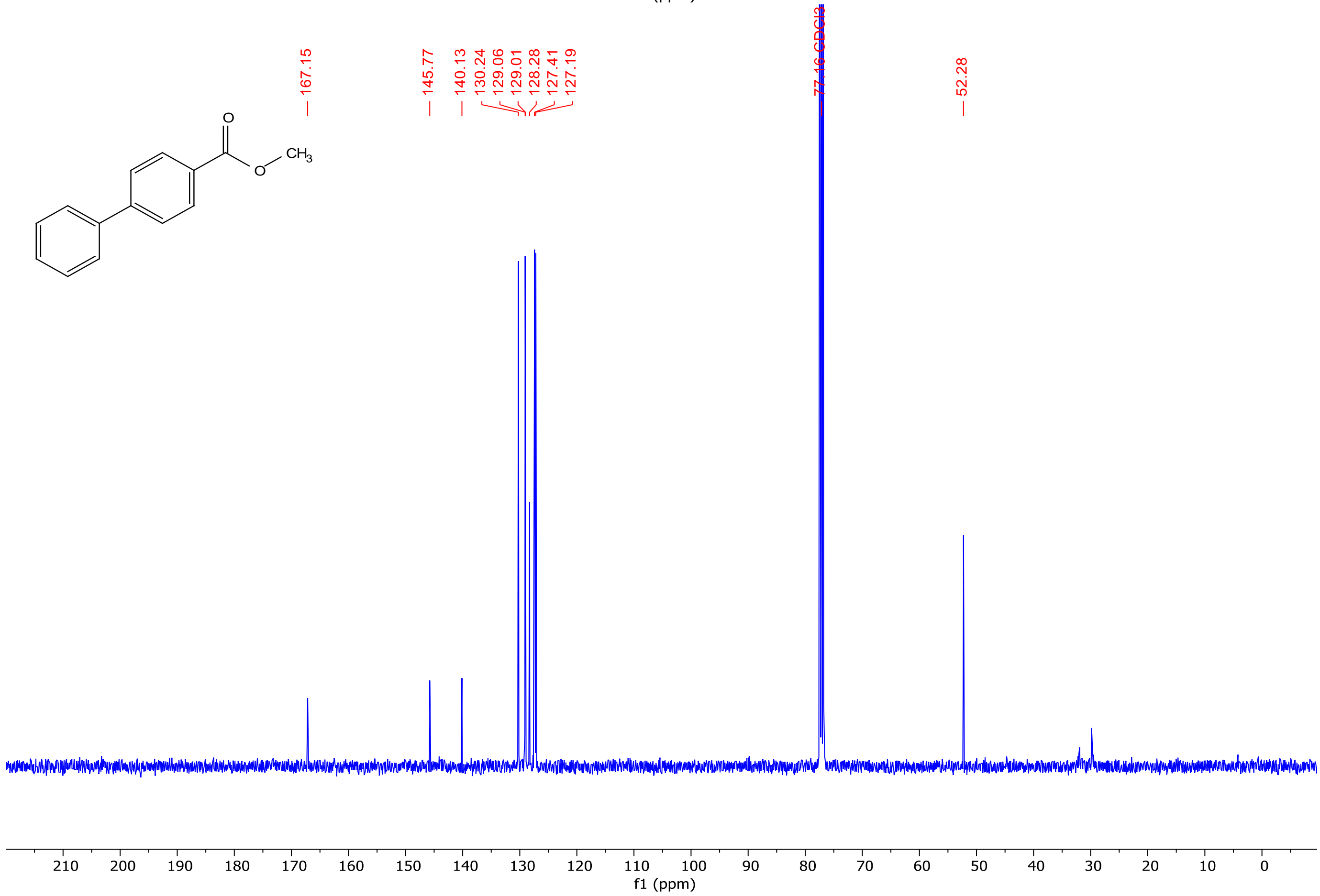

4-cyanophenyl 4-methylbenzoate (79)

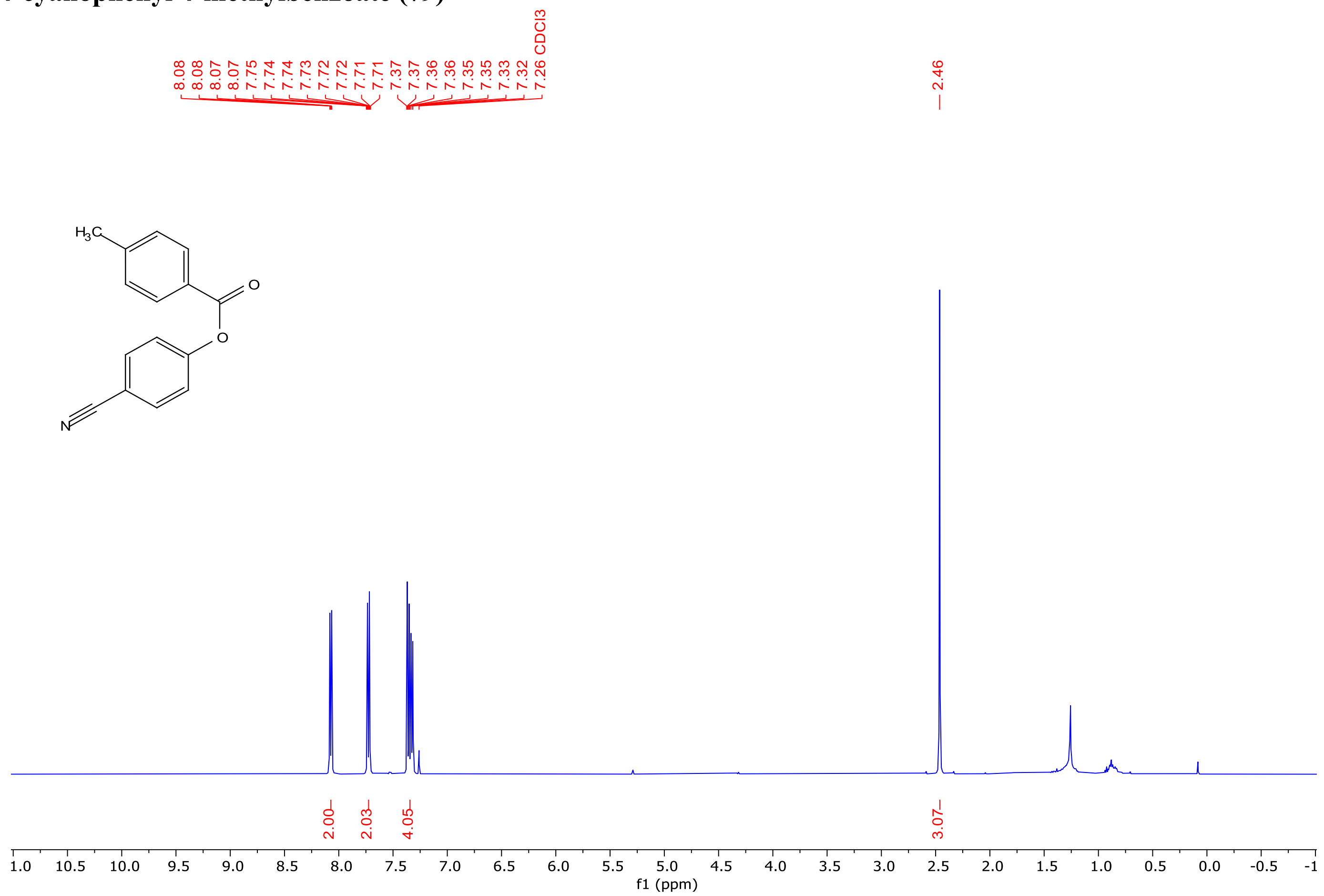

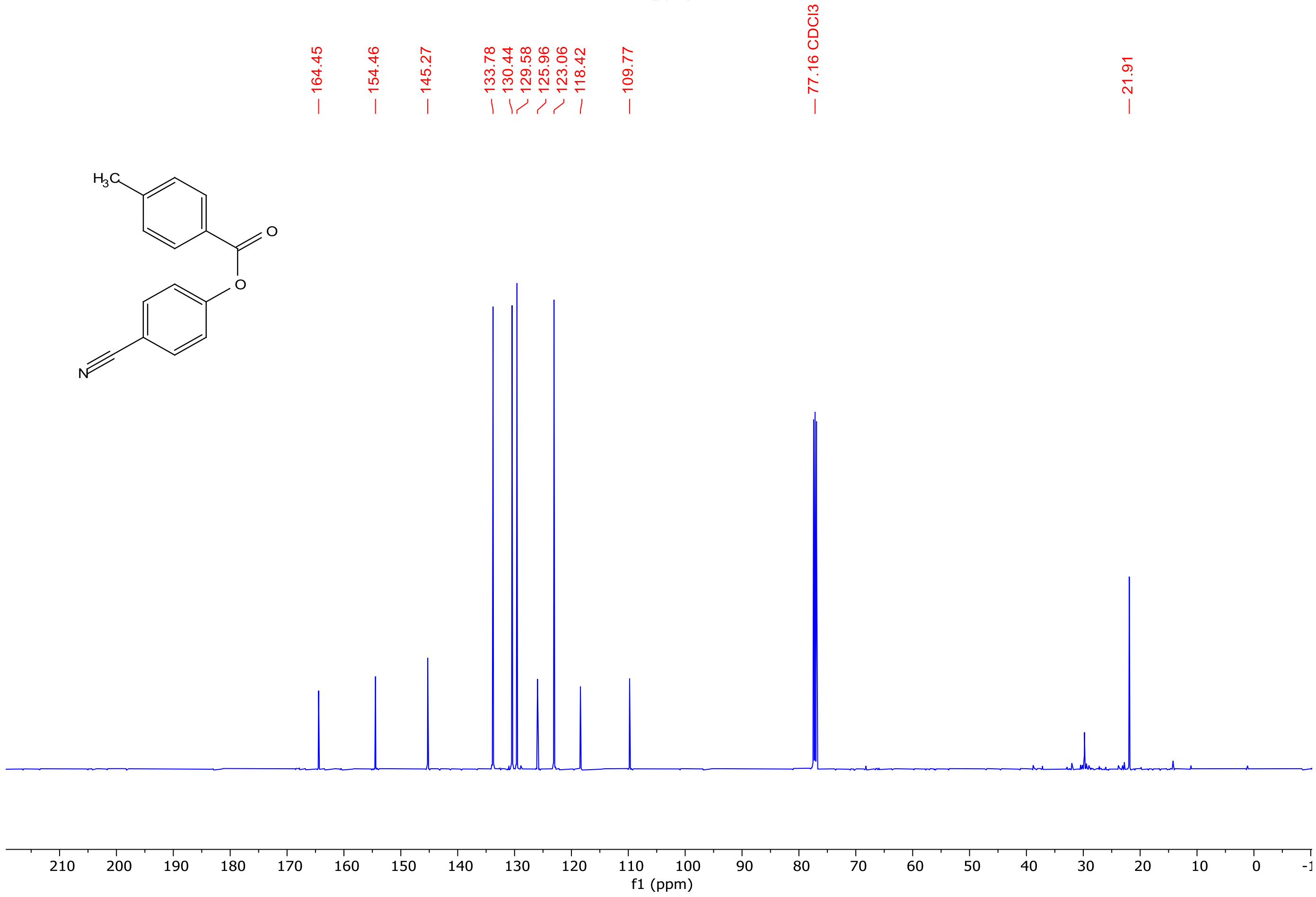

4-cyanophenyl 4-methylbenzoate (80)

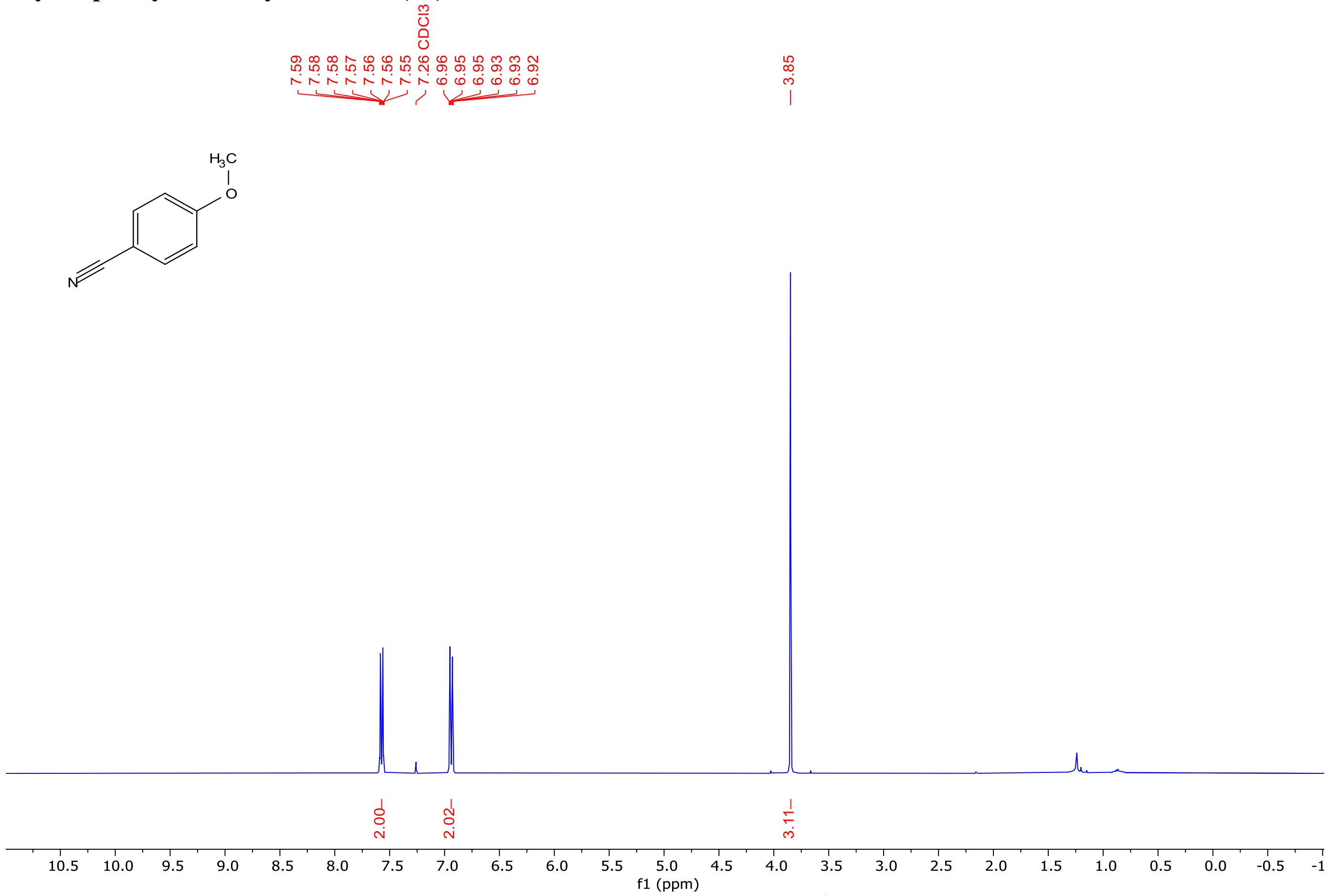

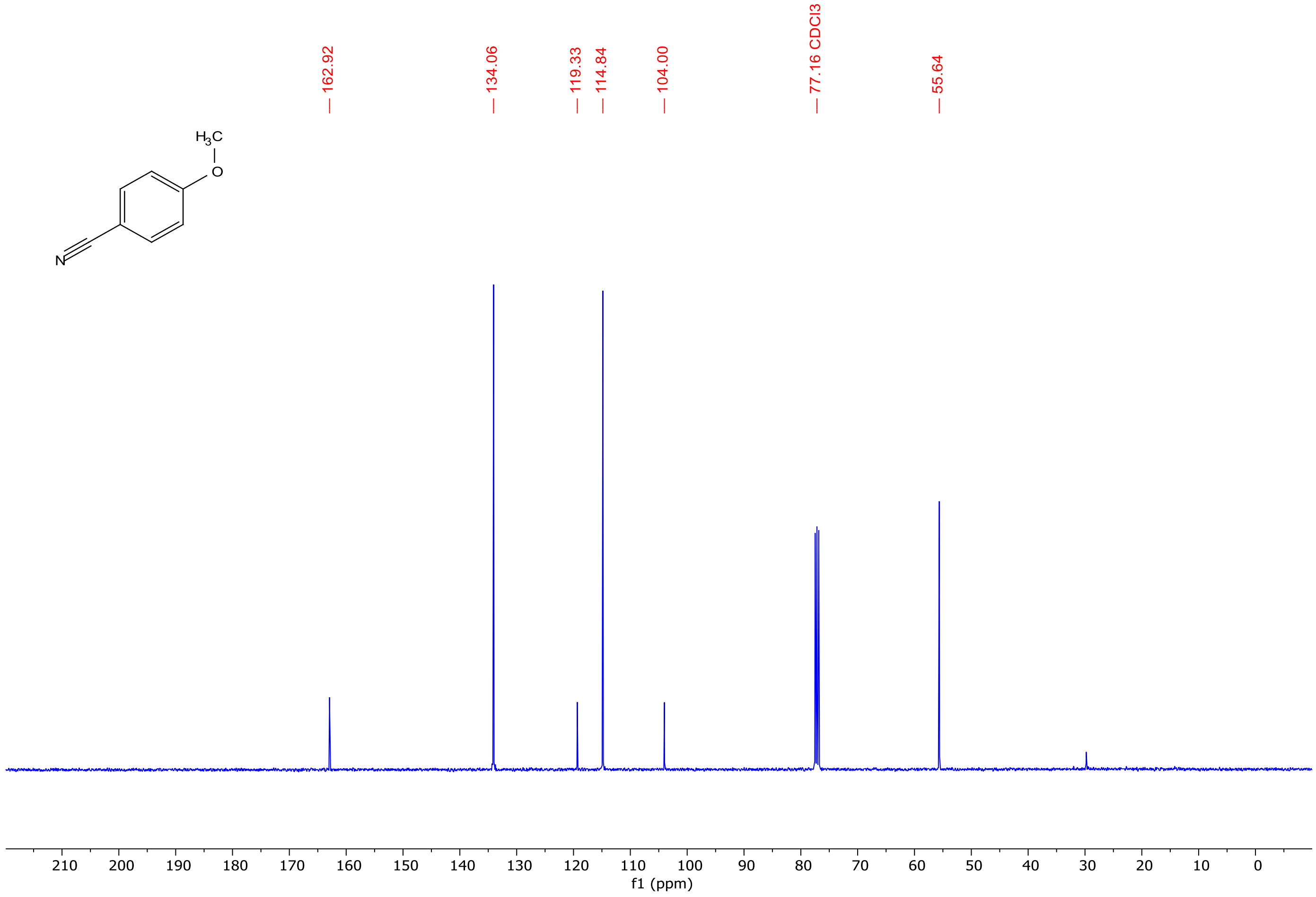

4-(hexyloxy)benzonitrile (81)

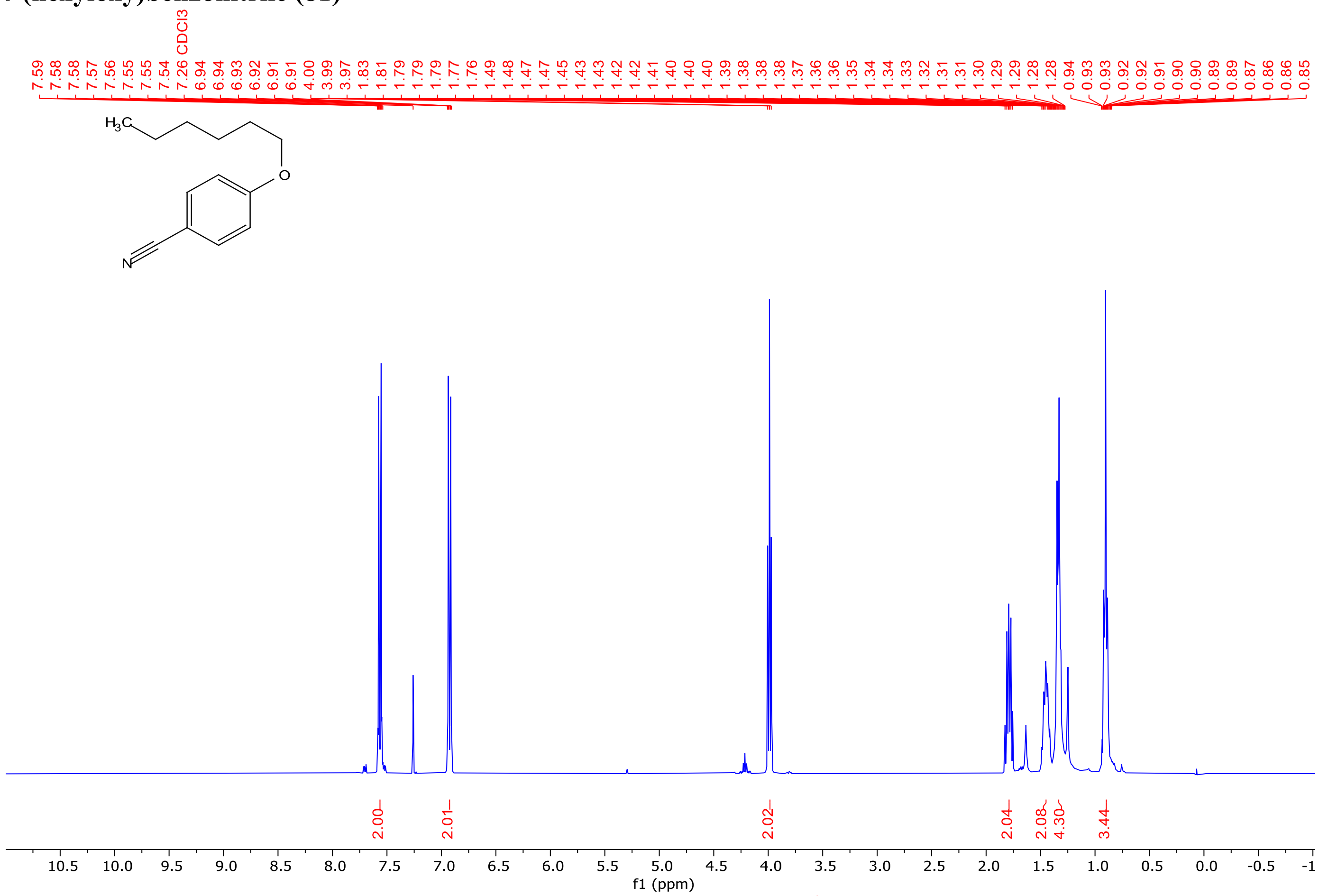

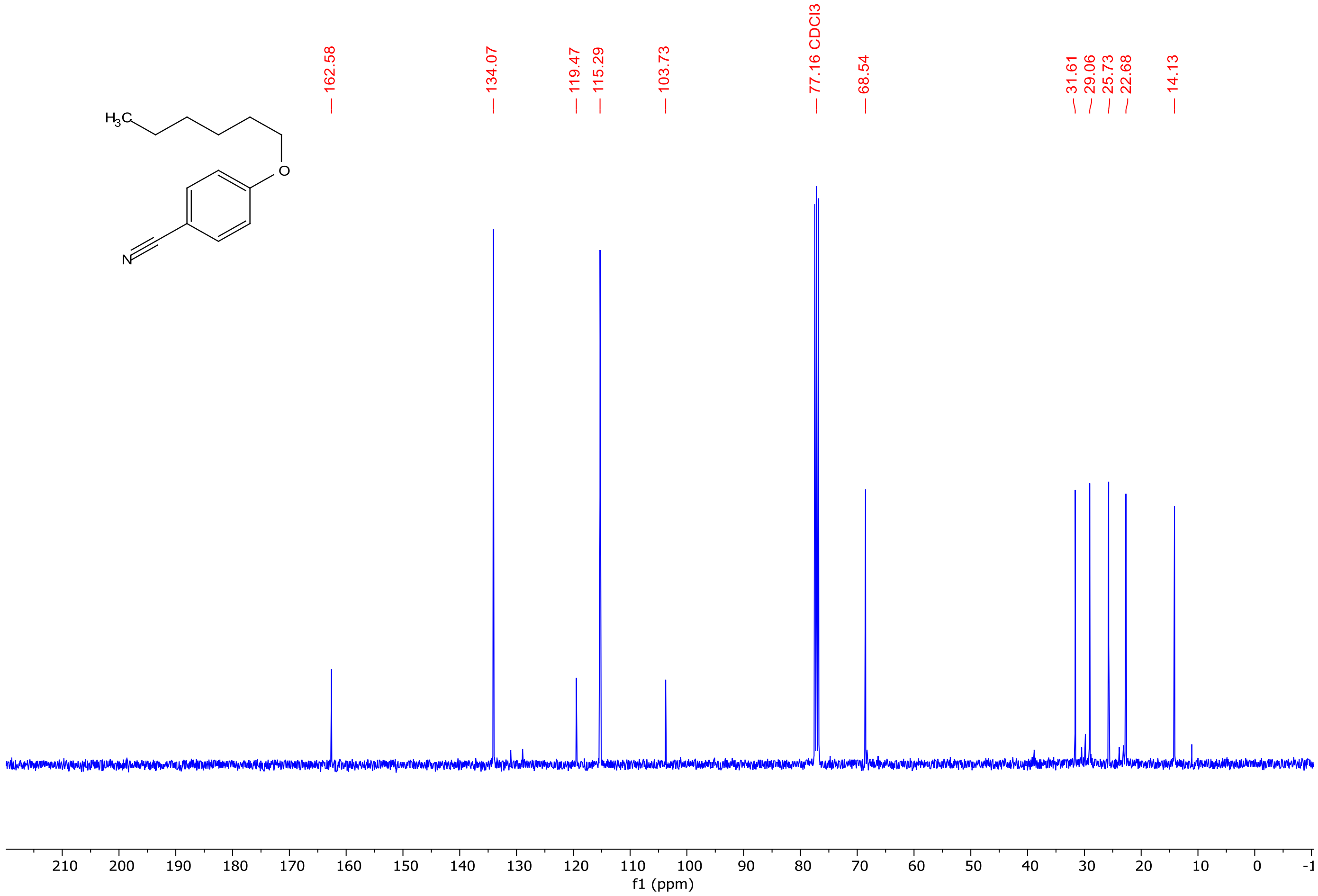

methyl 4-(4-methoxyphenoxy)benzoate (82)

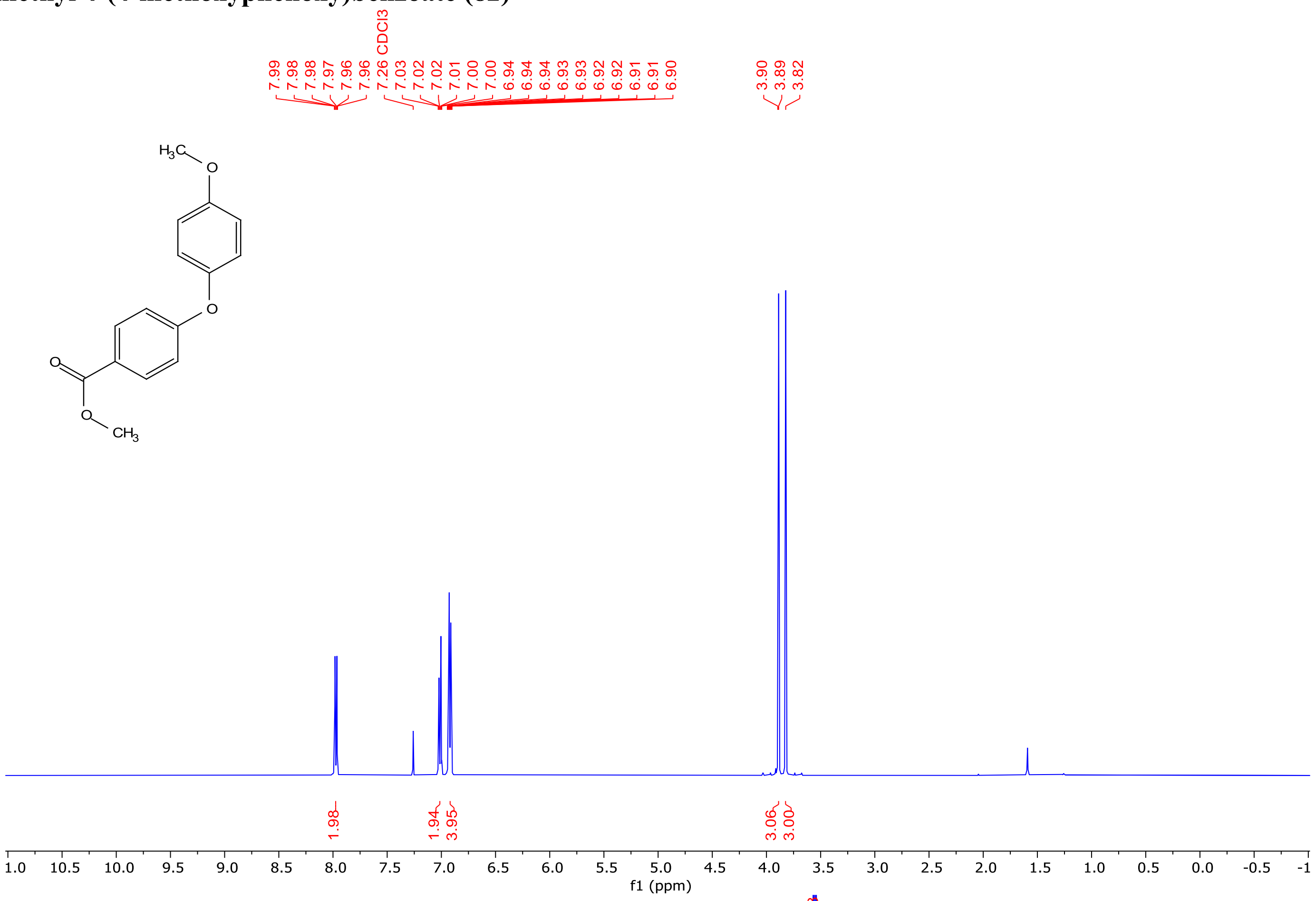

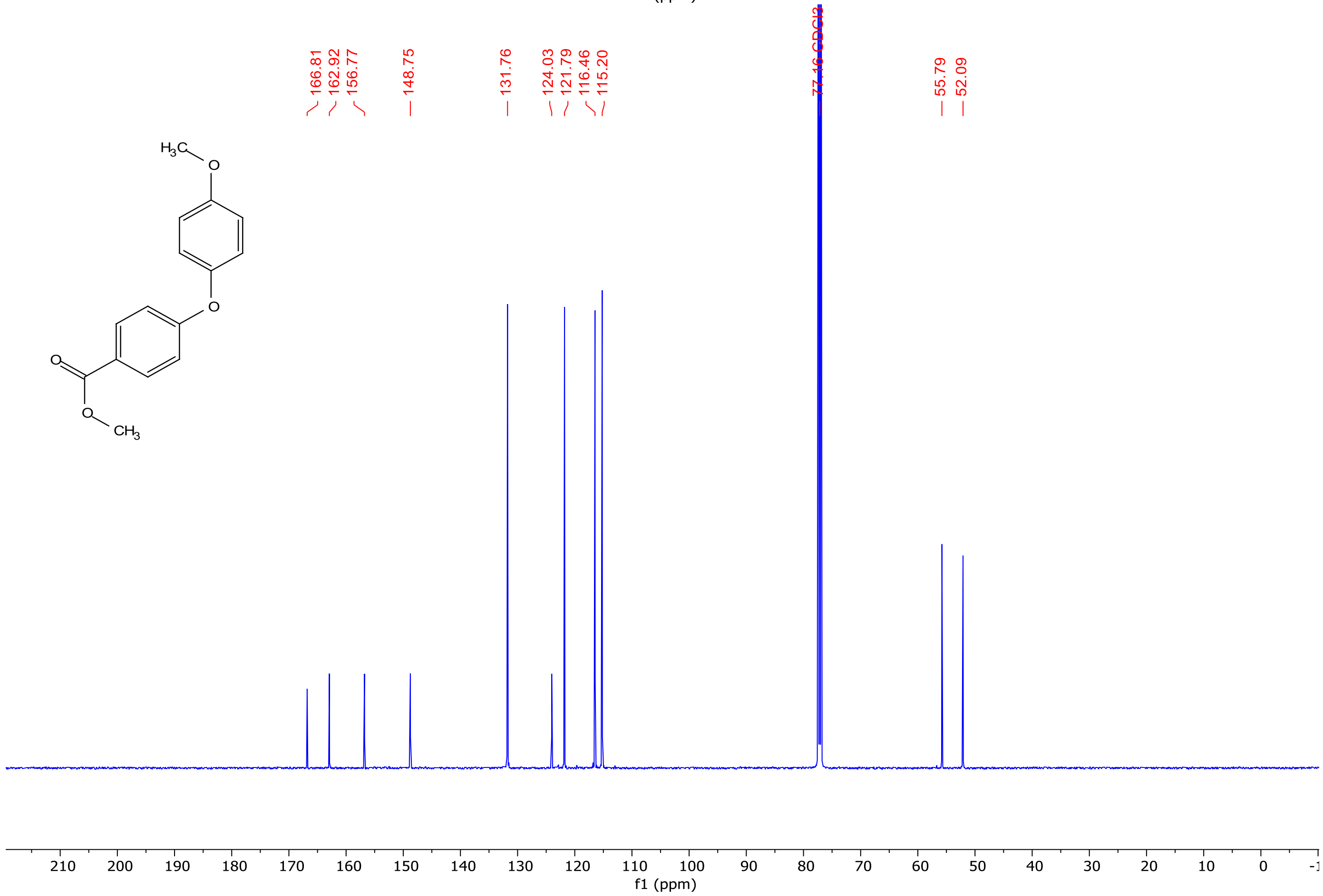

4-hydroxybenzonitrile (83)

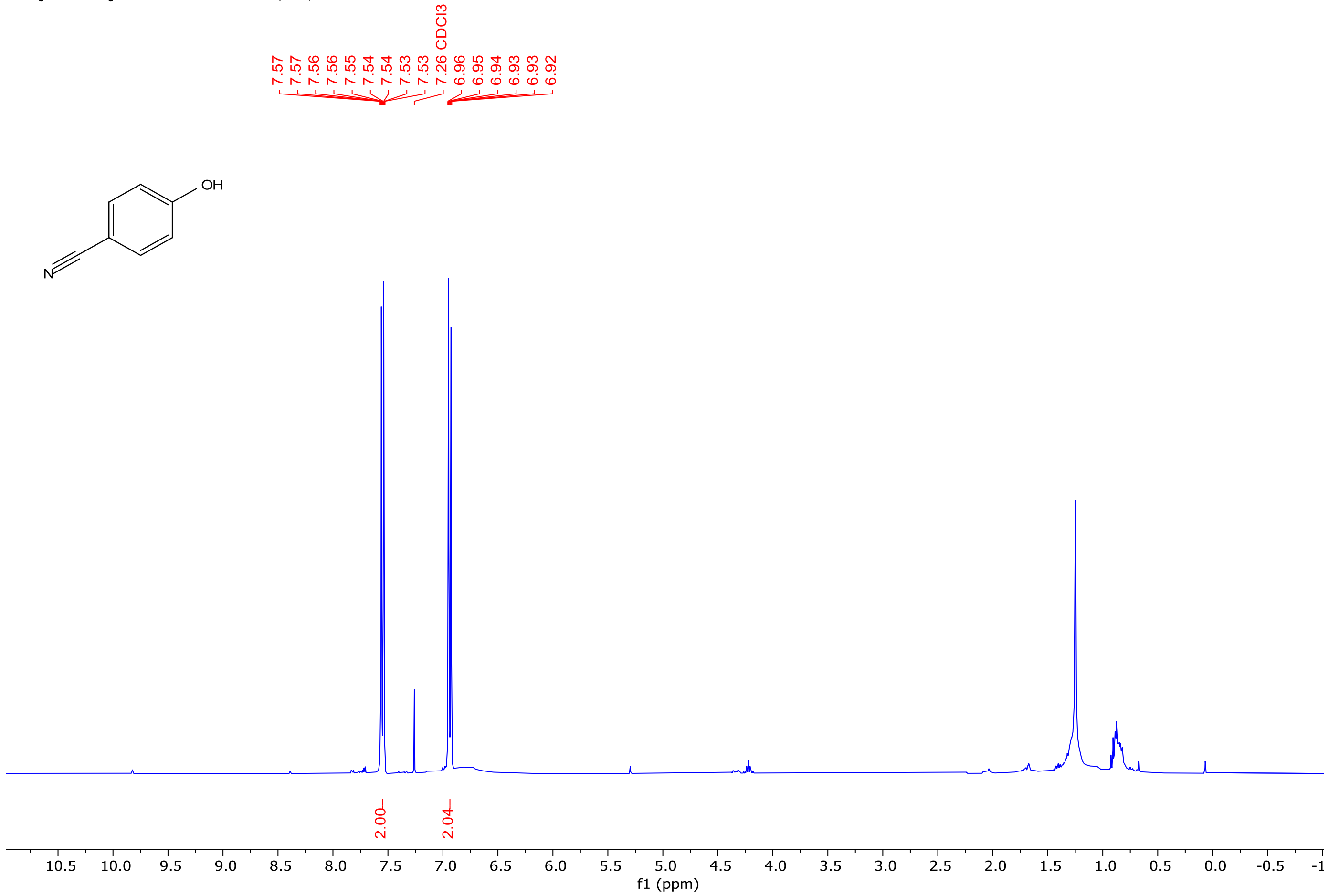

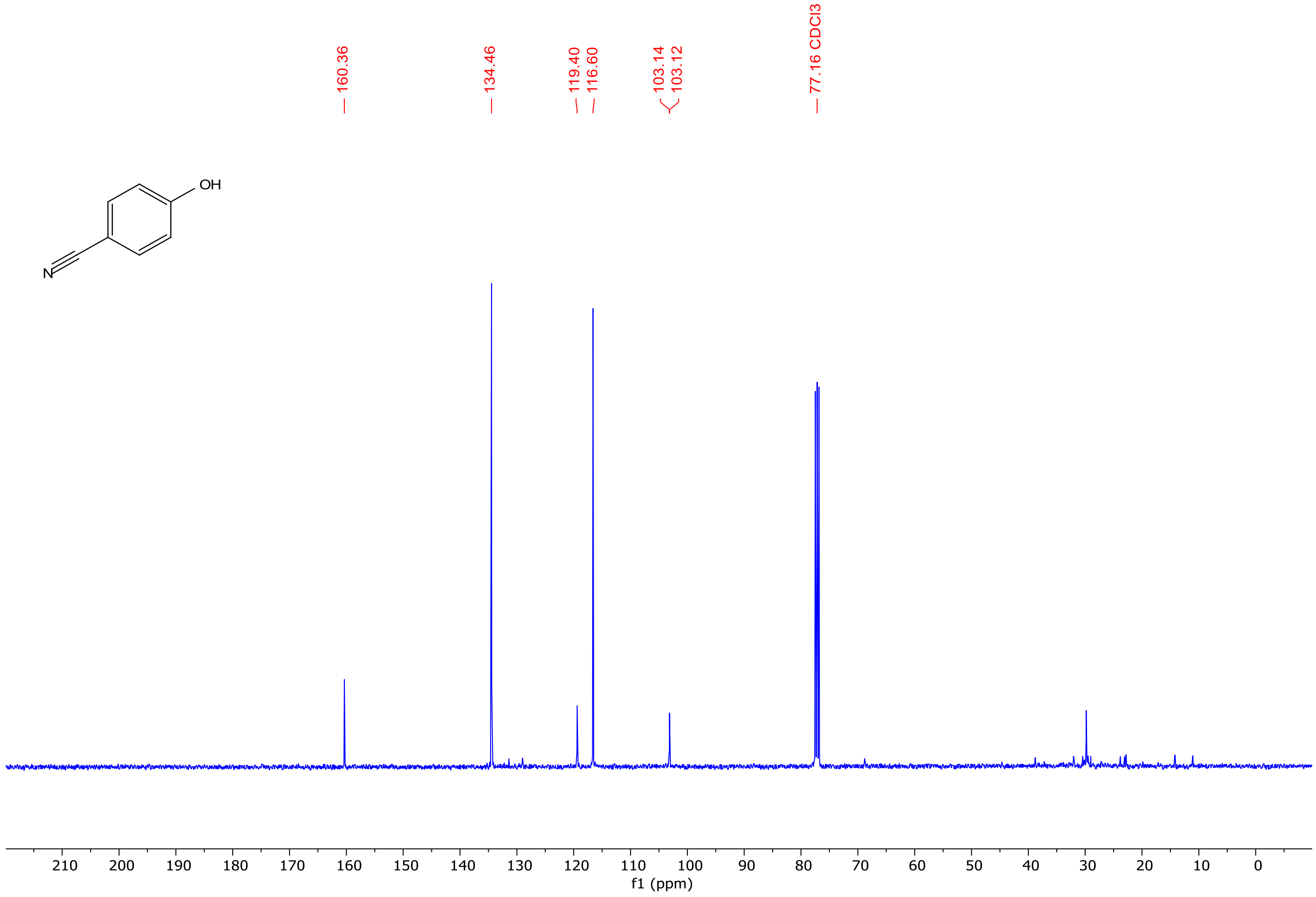

4-chlorobenzonitrile (84)

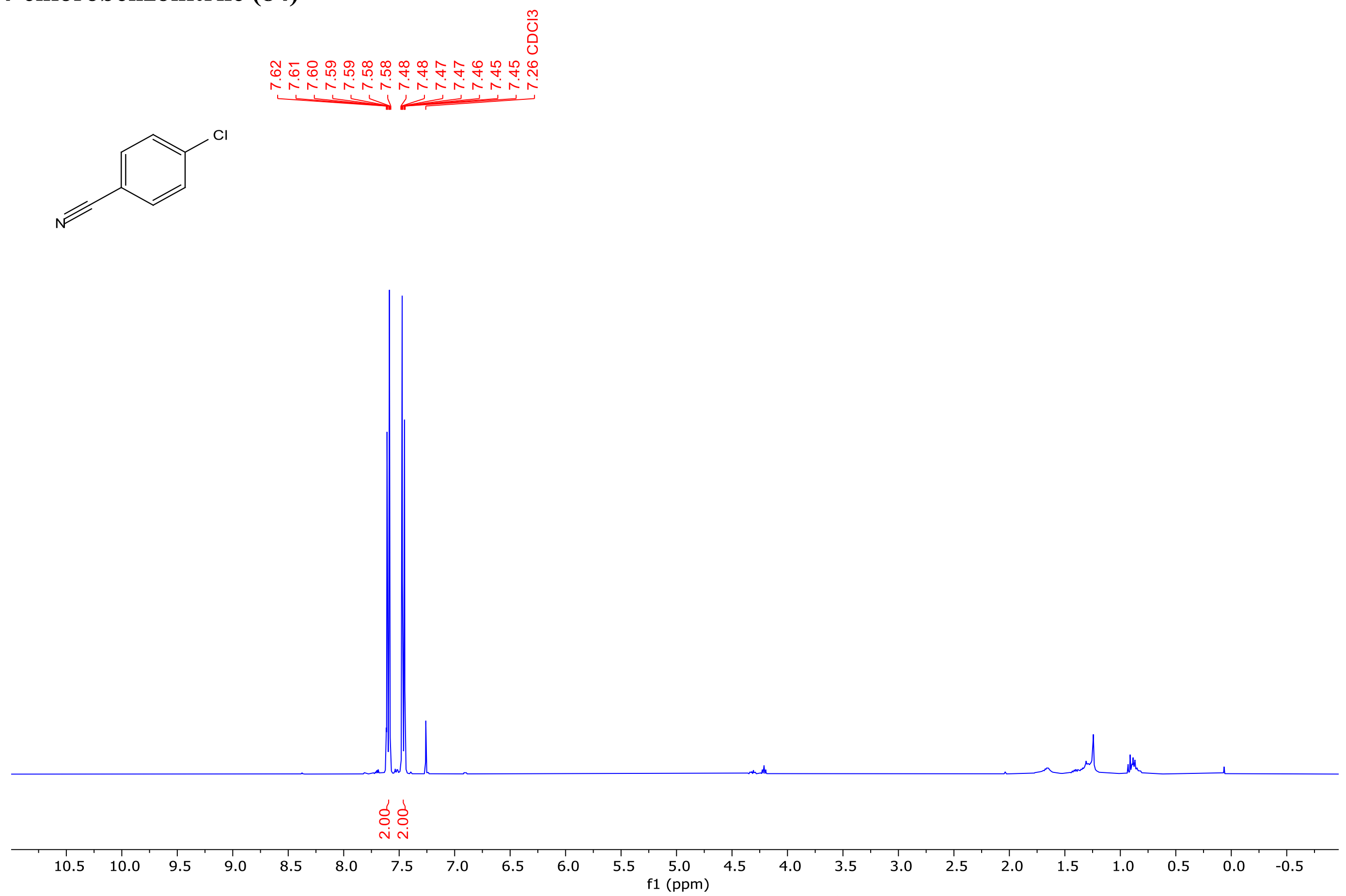

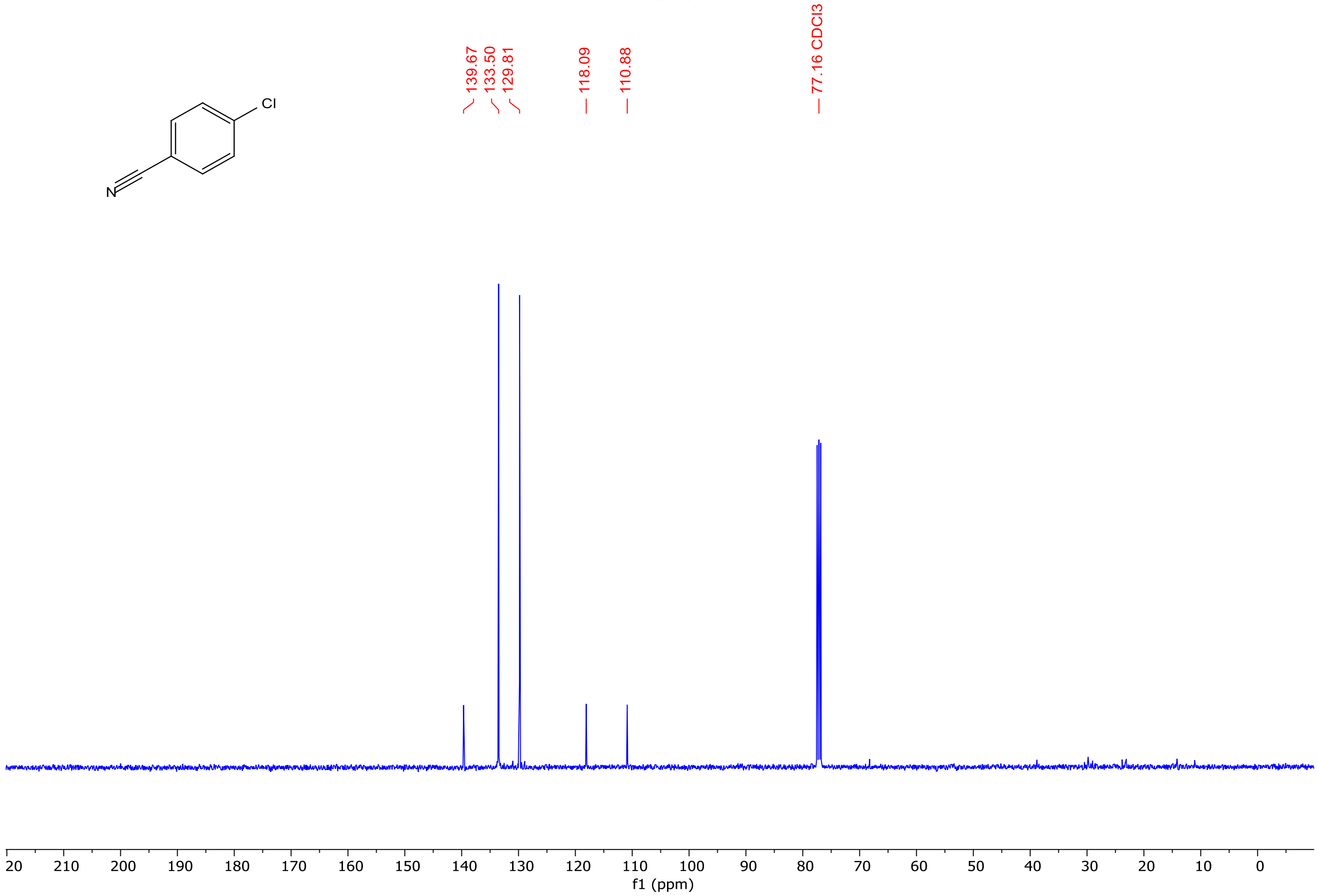

dimethyl 4,4'-thiodibenzoate (85)

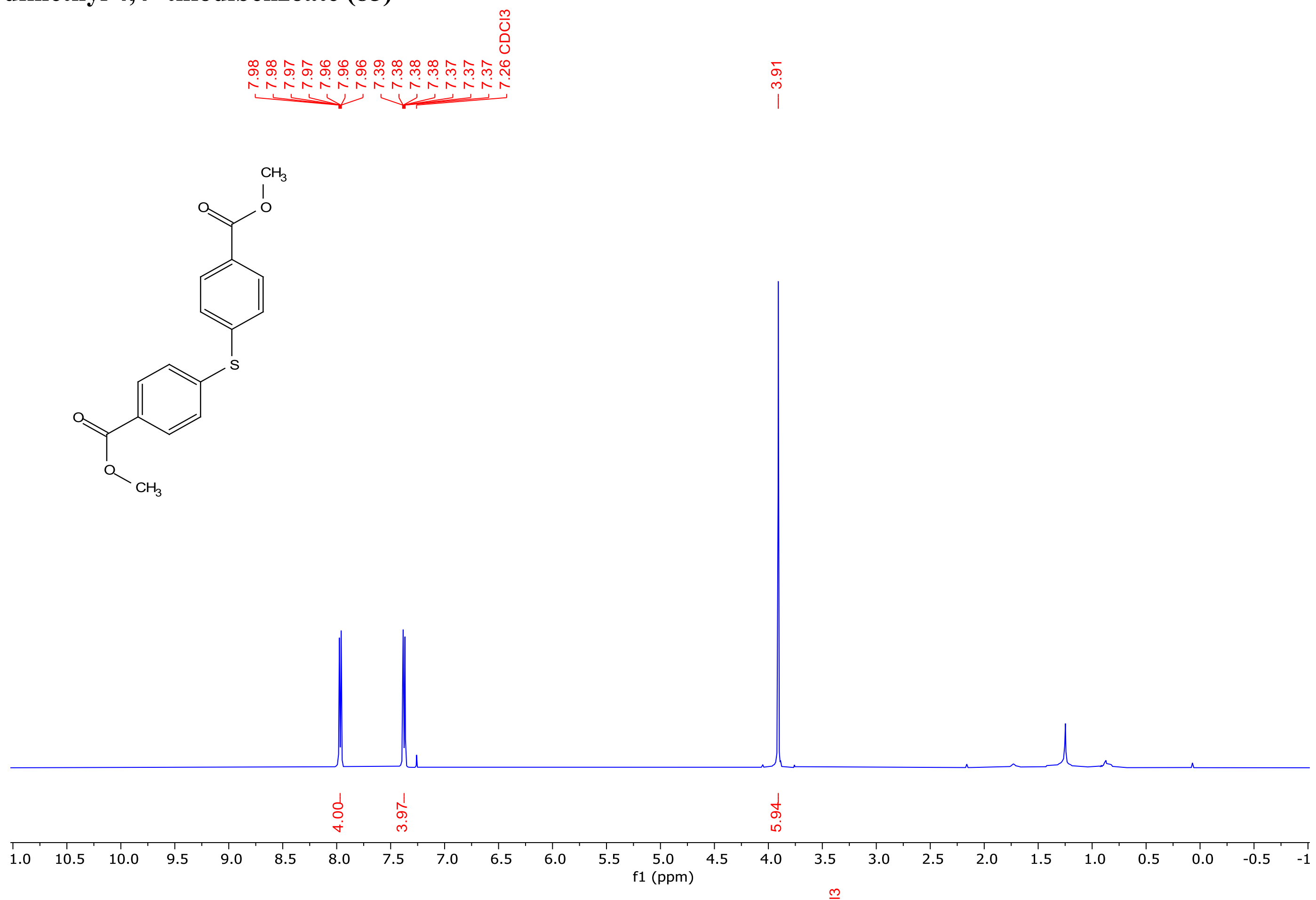

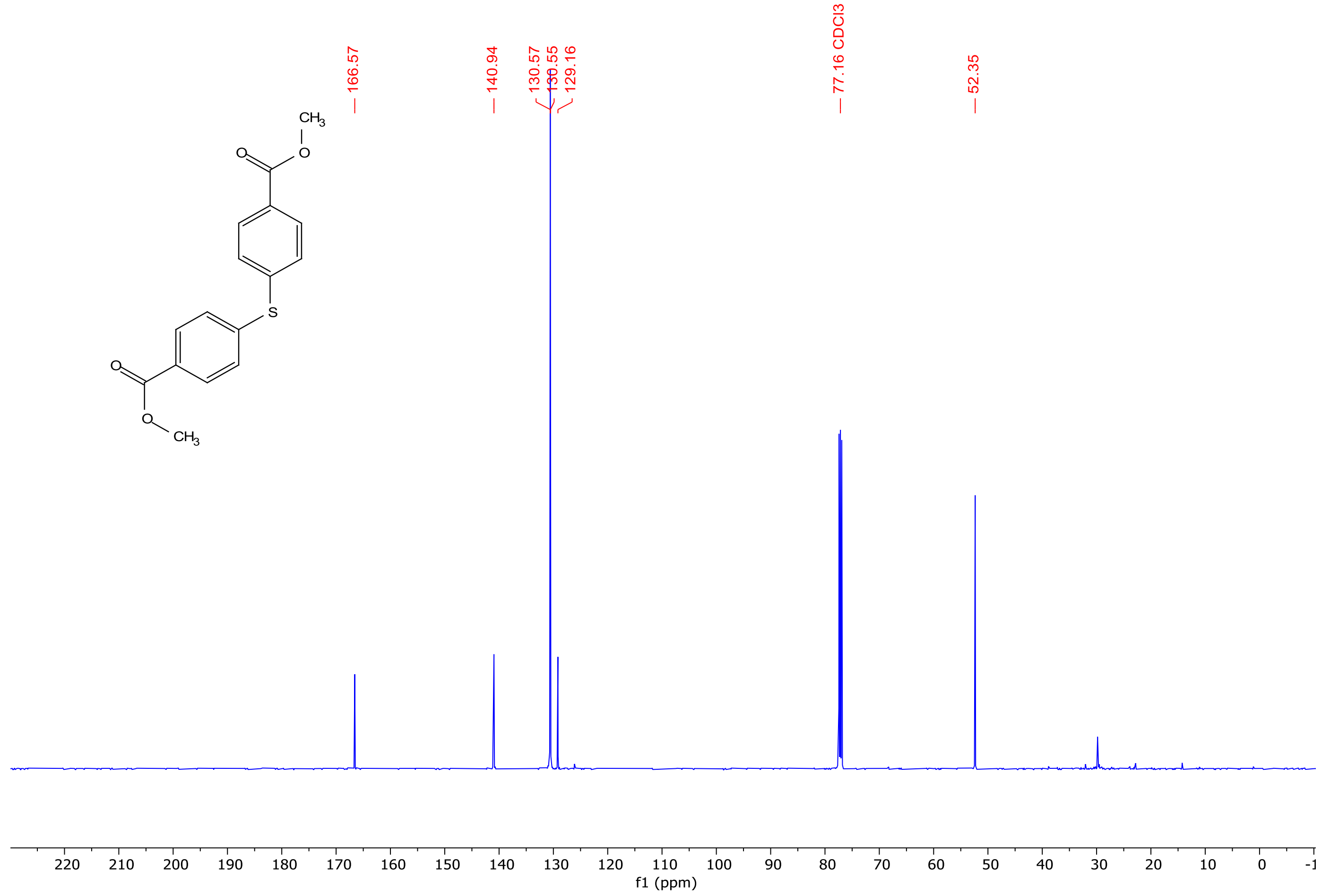

4-(decylthio)benzonitrile (86)

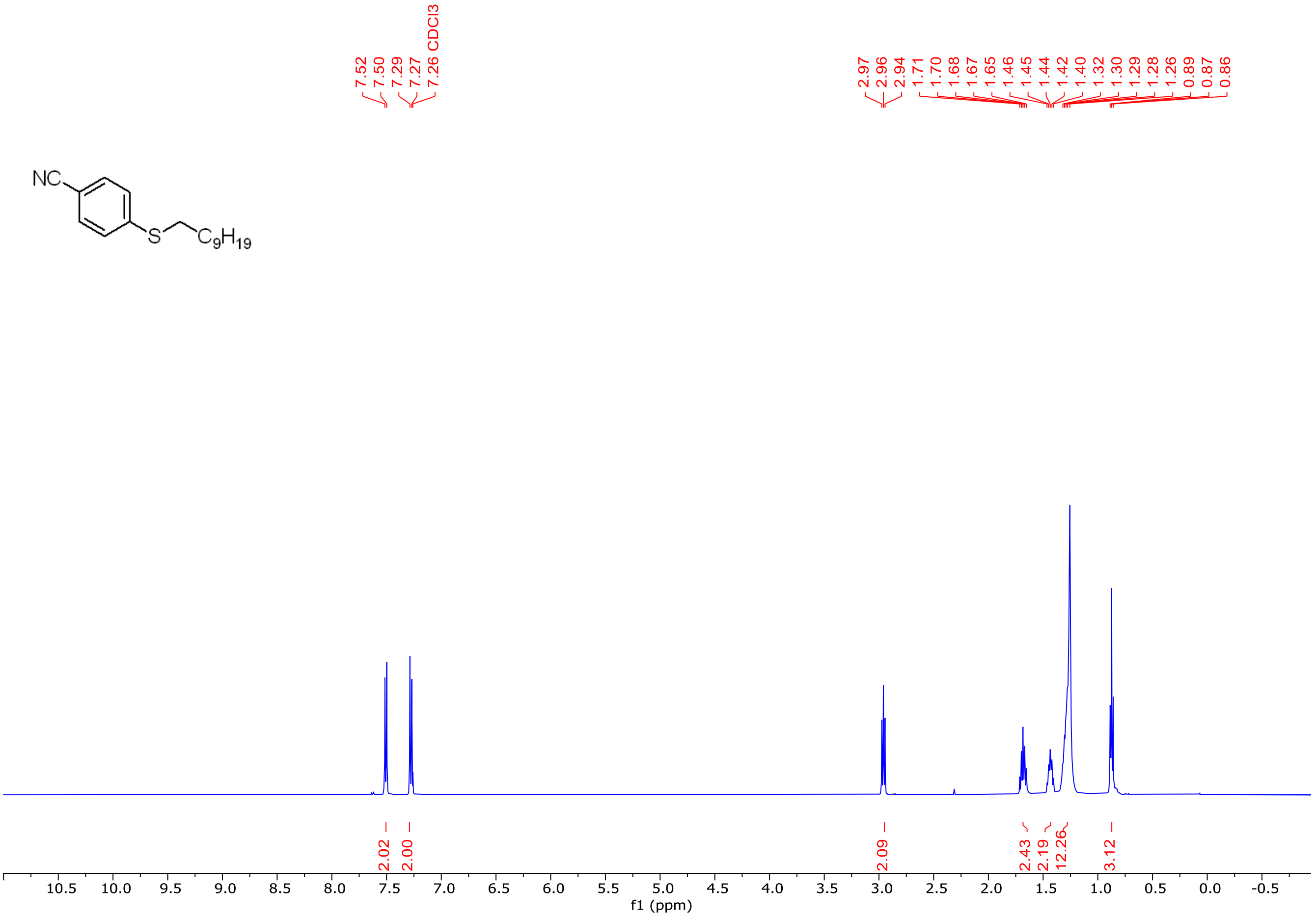

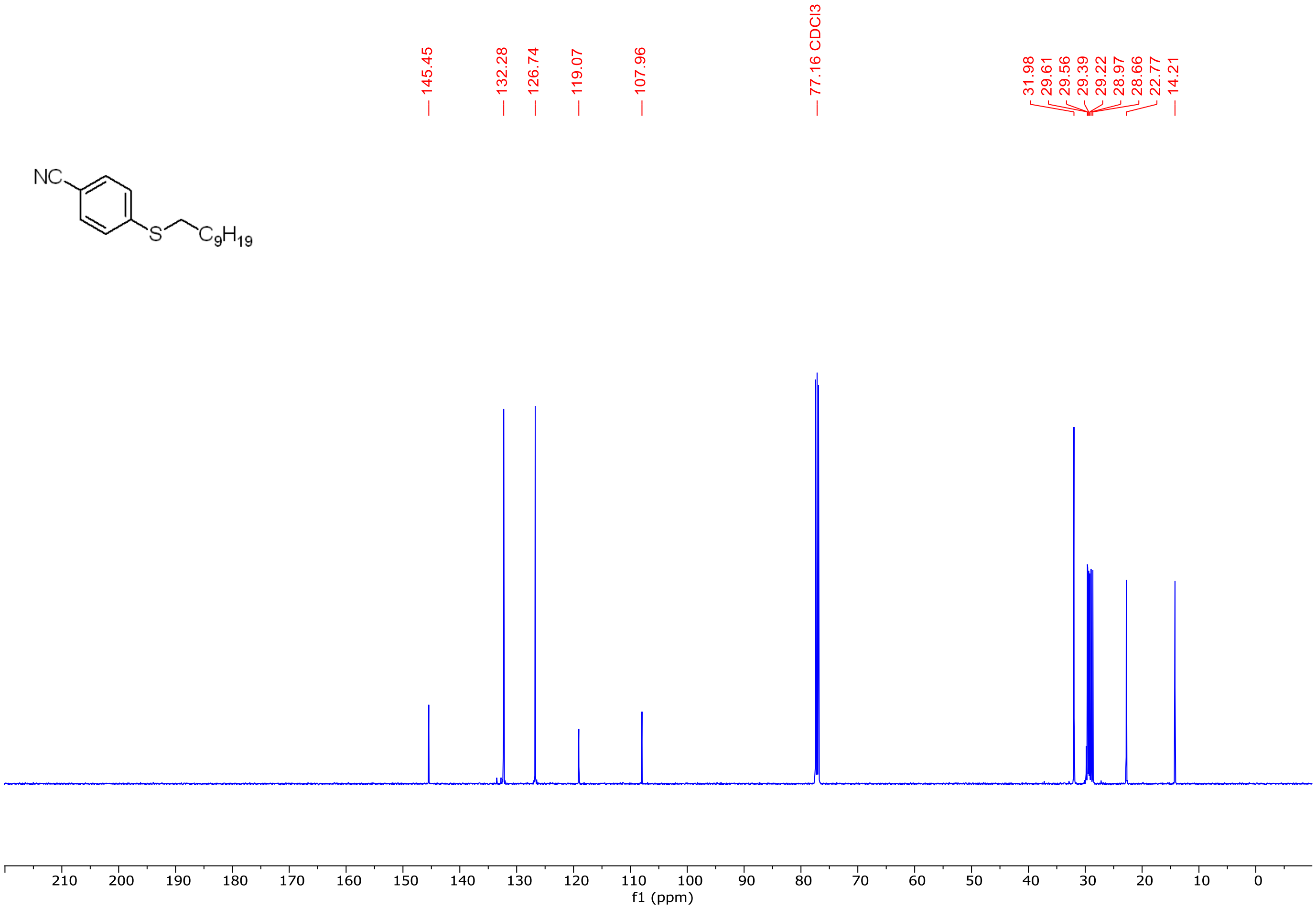

4-(cyclohexylthio)benzonitrile (87)

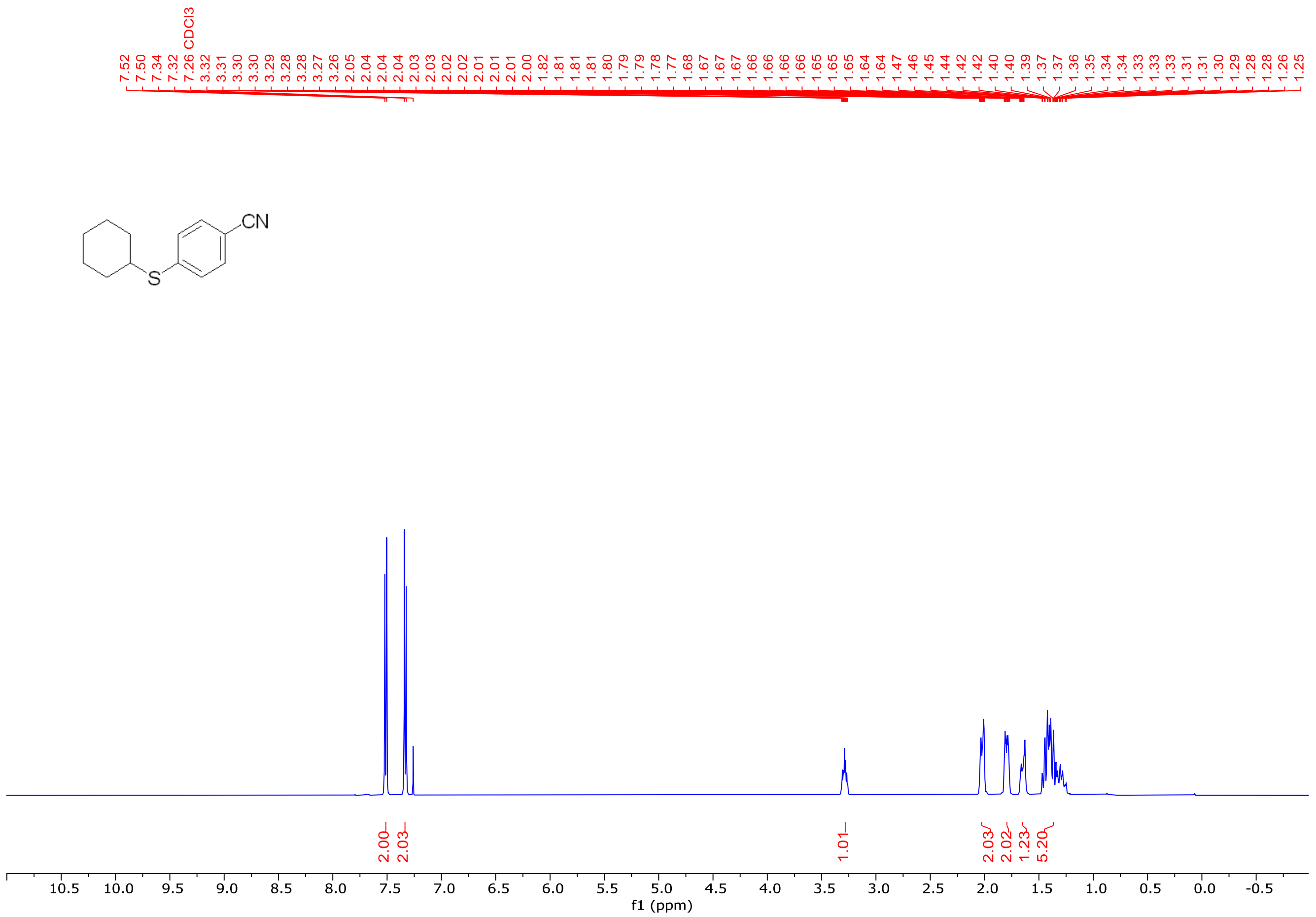

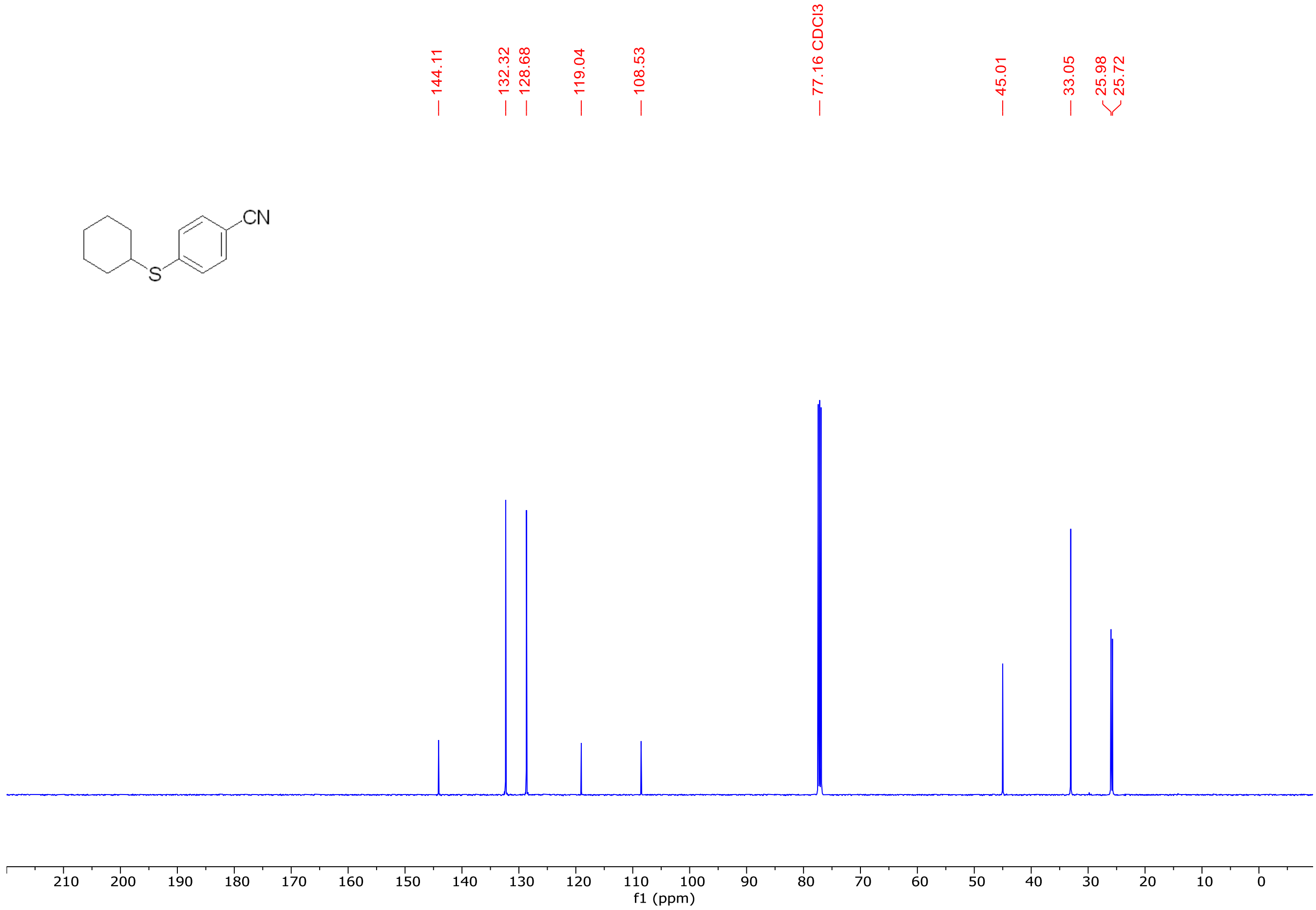

methyl(4-(phenylthio)phenyl)sulfane(88)

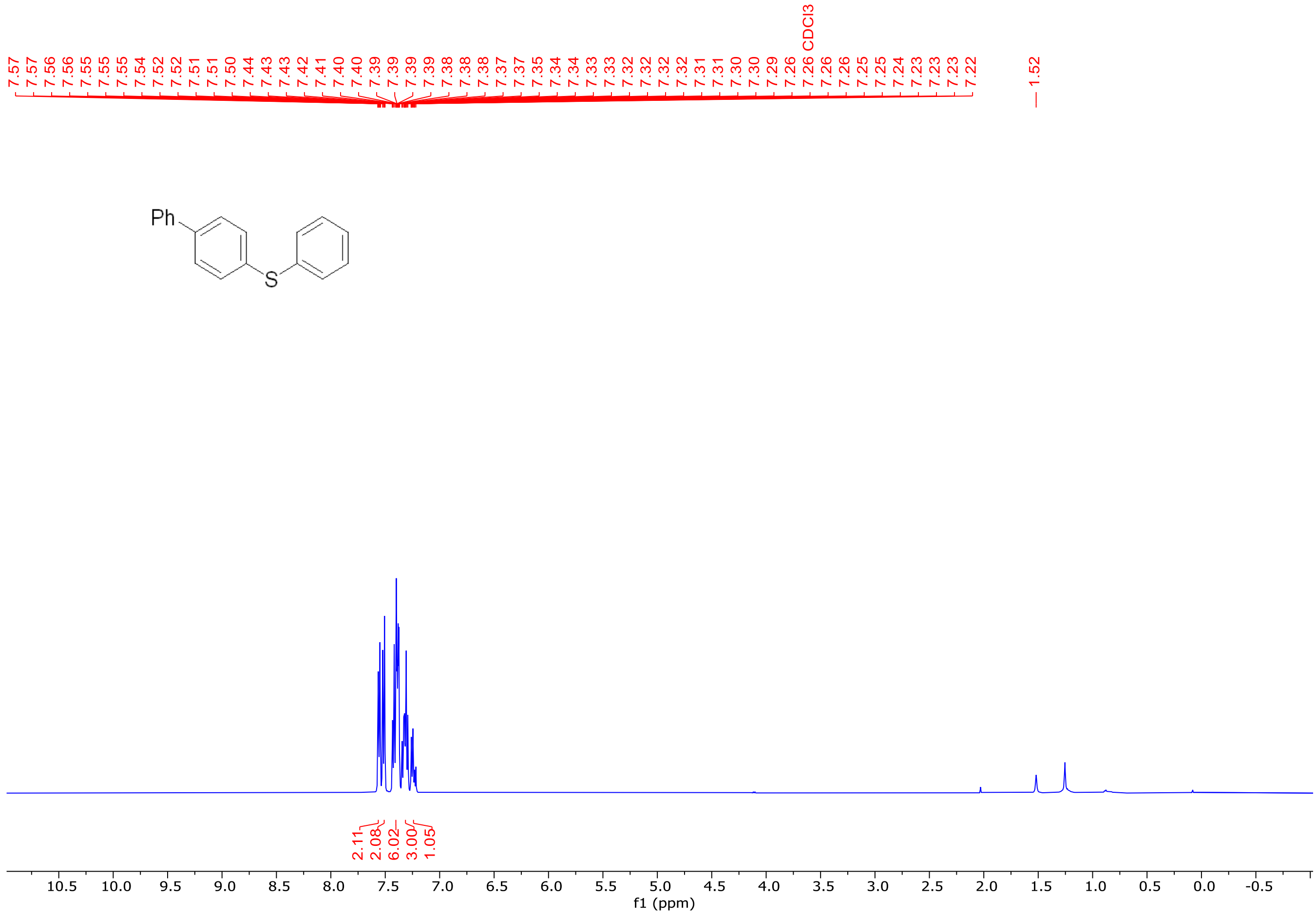

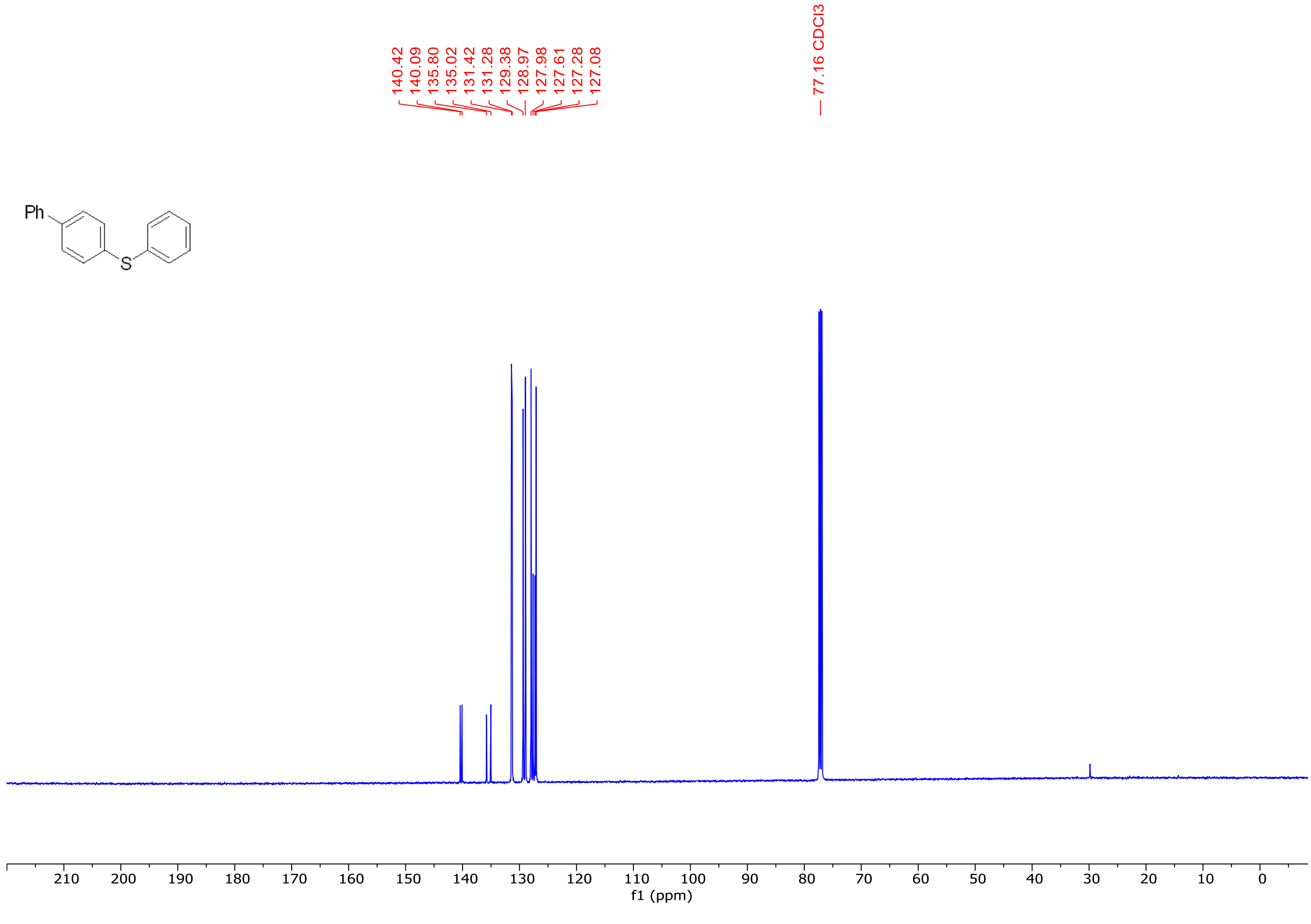

[1,1'-biphenyl]-4-yl(phenyl)sulfane(89)

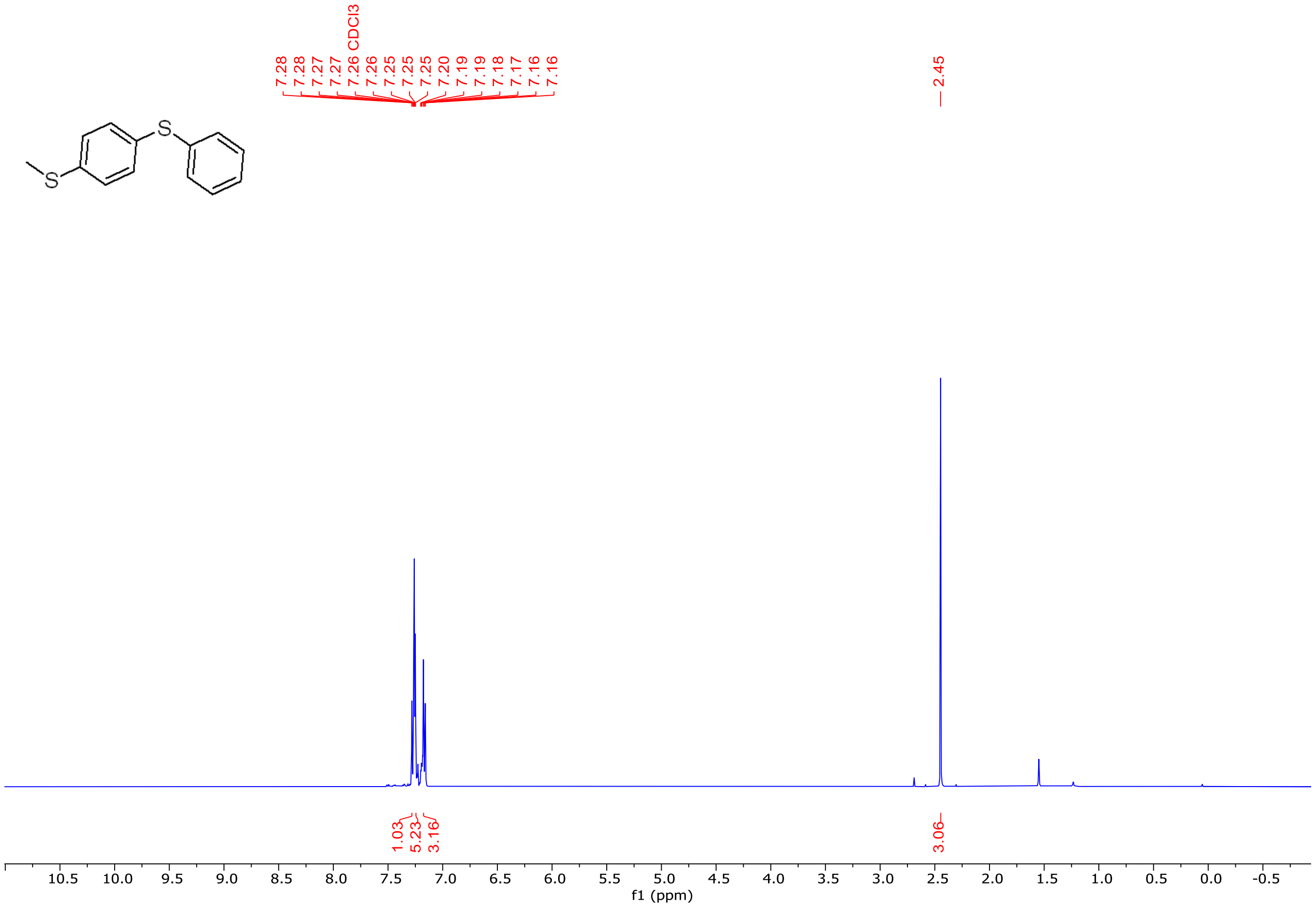

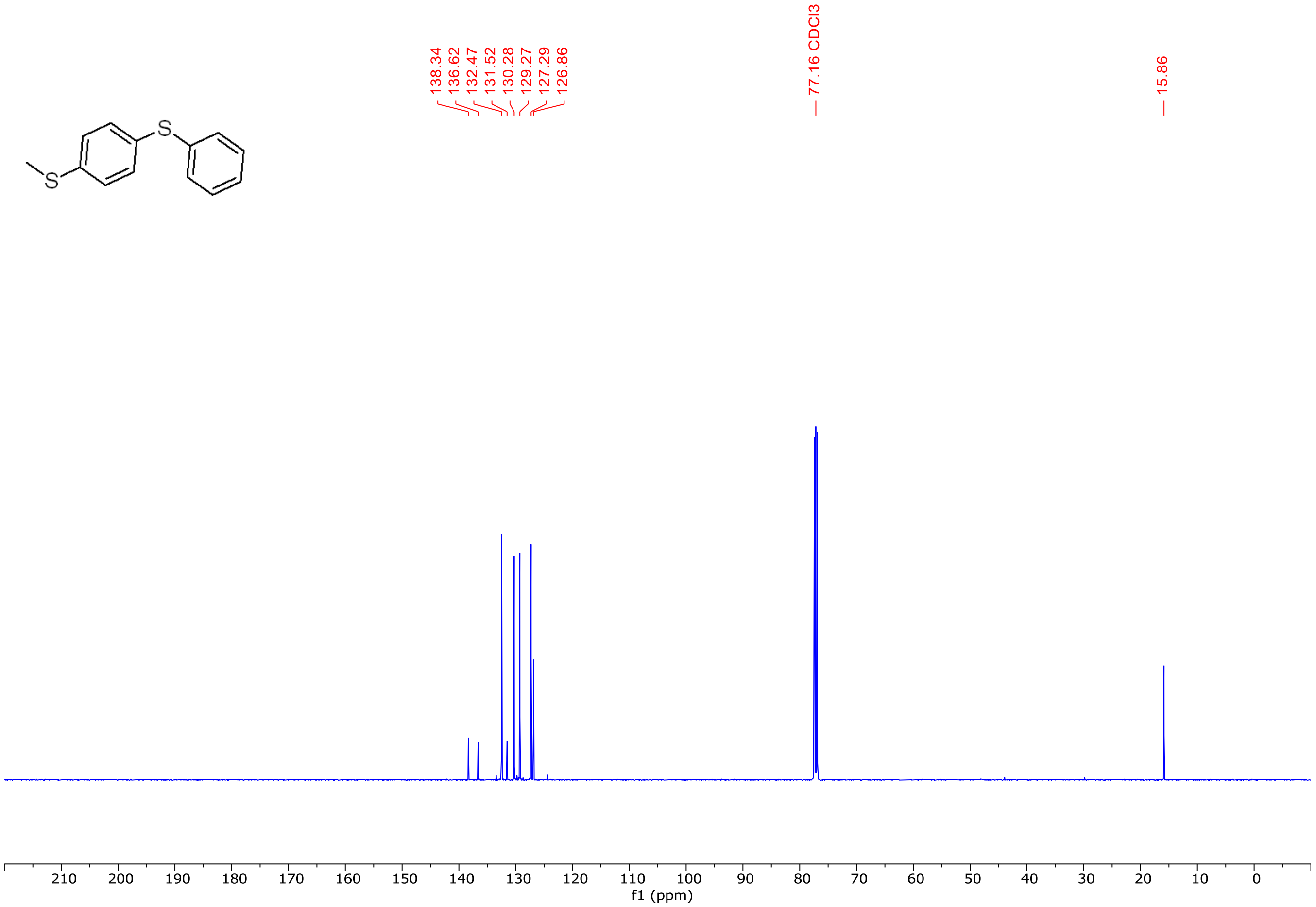

1-(4-(phenylthio)phenyl)ethan-1-one (90)

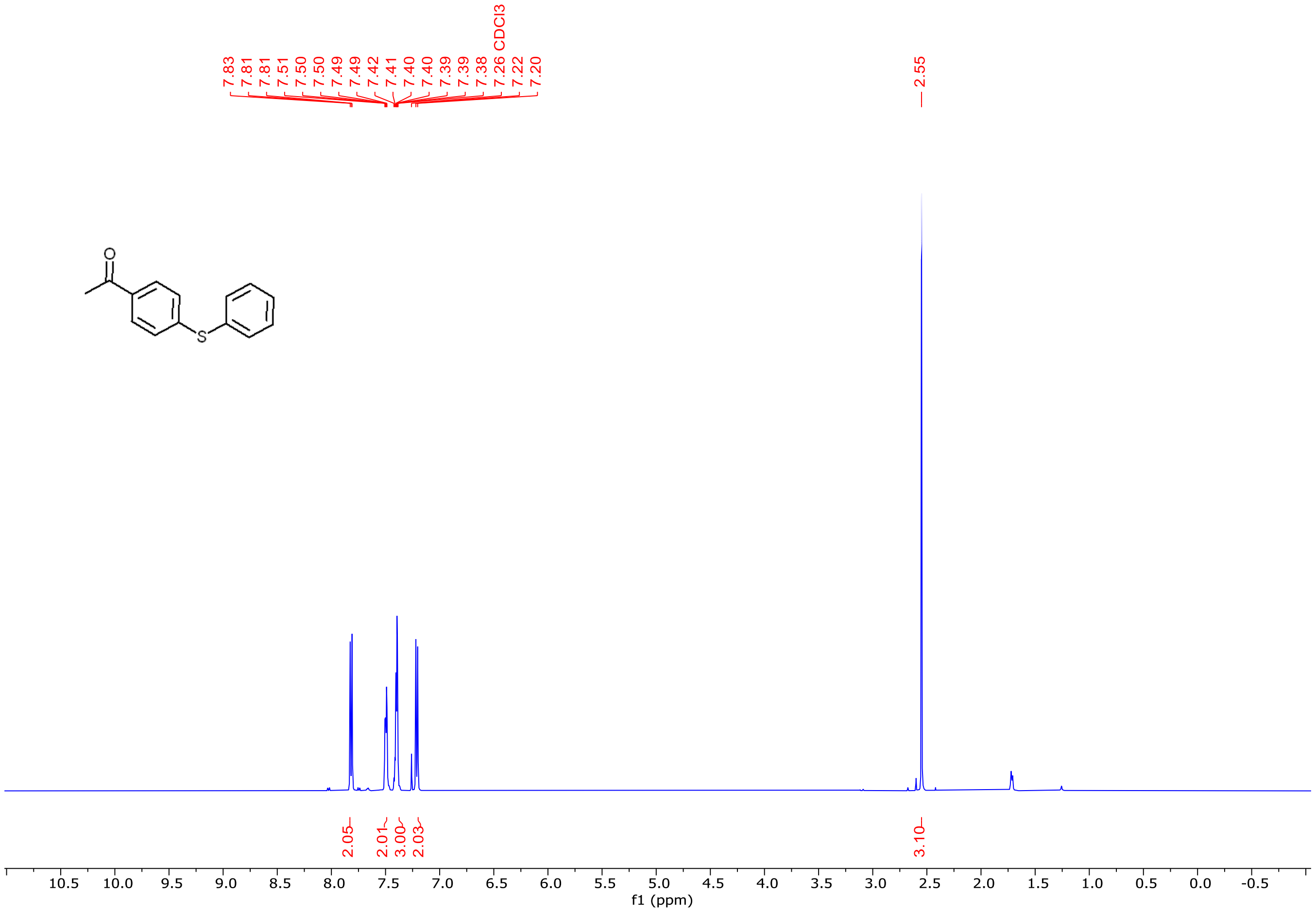

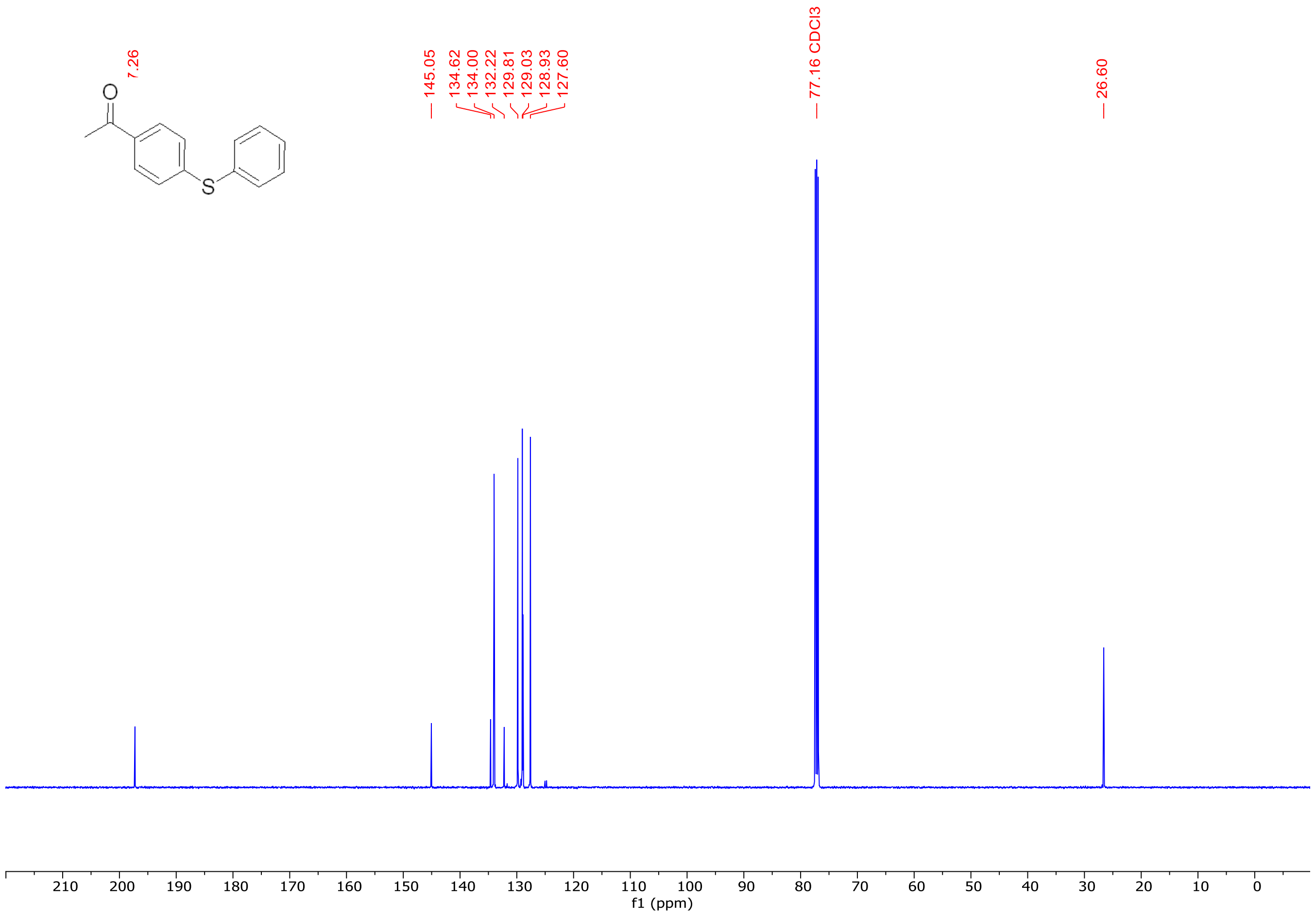

4-(phenylthio)benzonitrile (91)

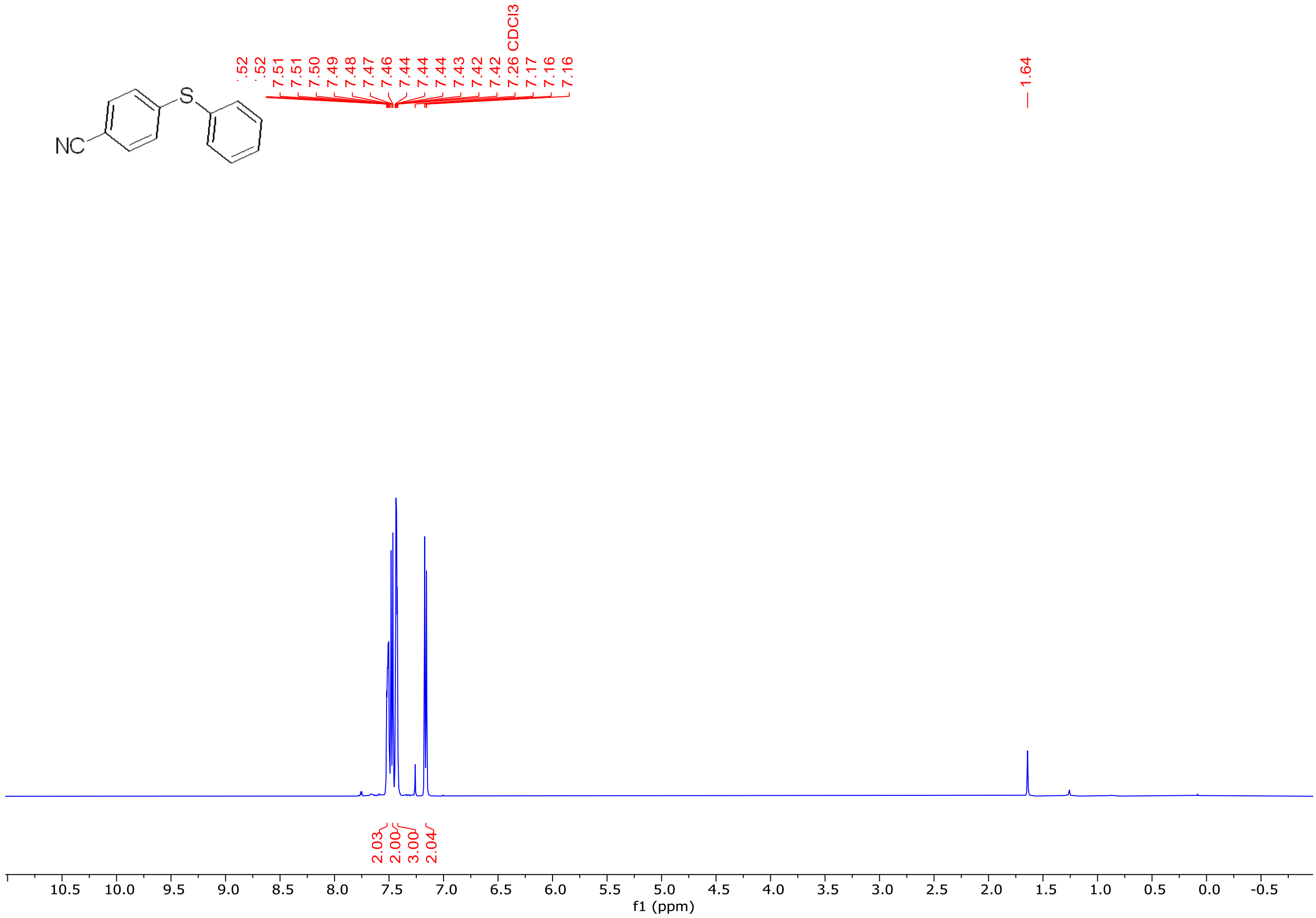

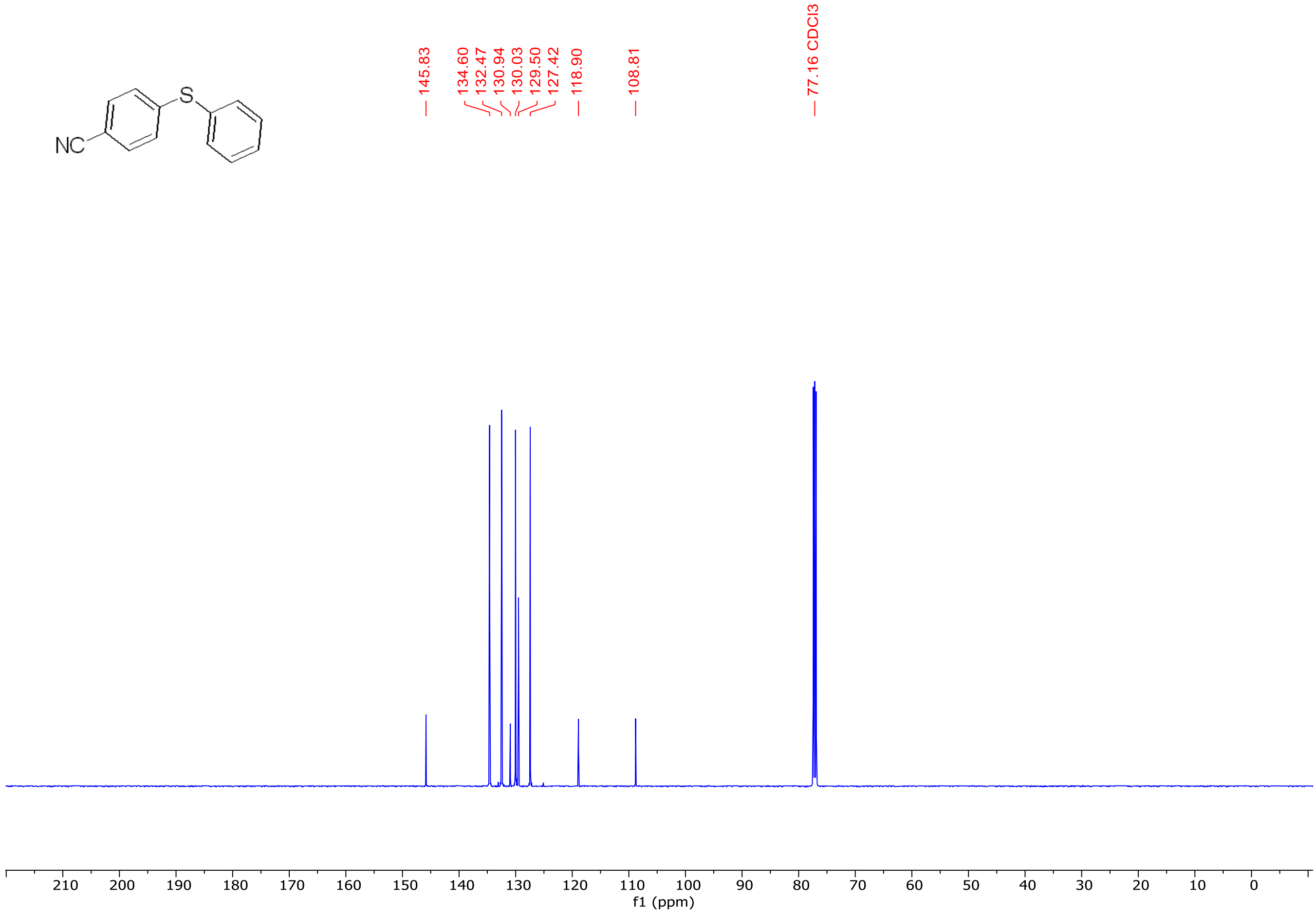

4,4,5,5-tetramethyl-2-(4-(phenylthio)phenyl)-1,3,2-dioxaborolane (92)

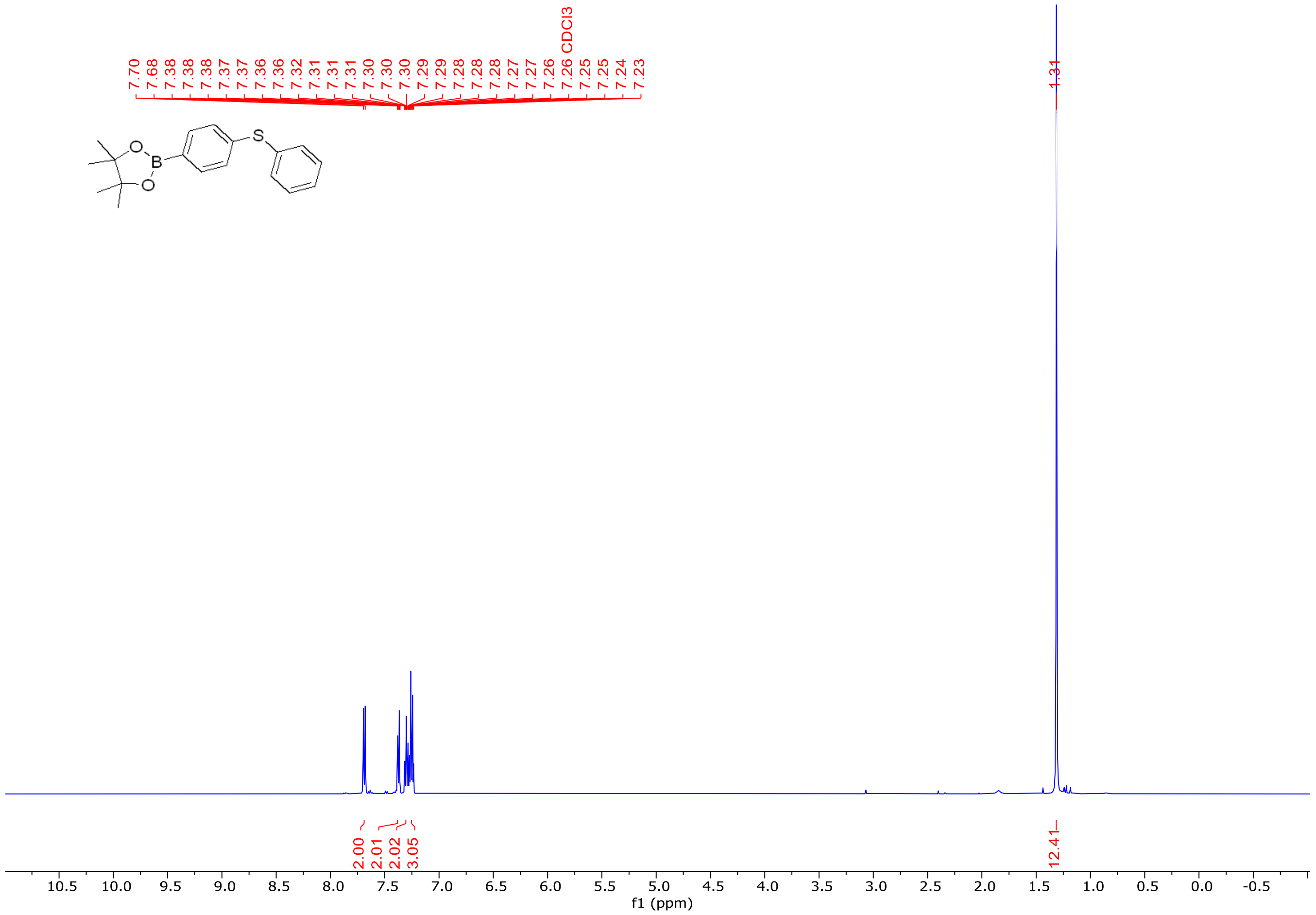

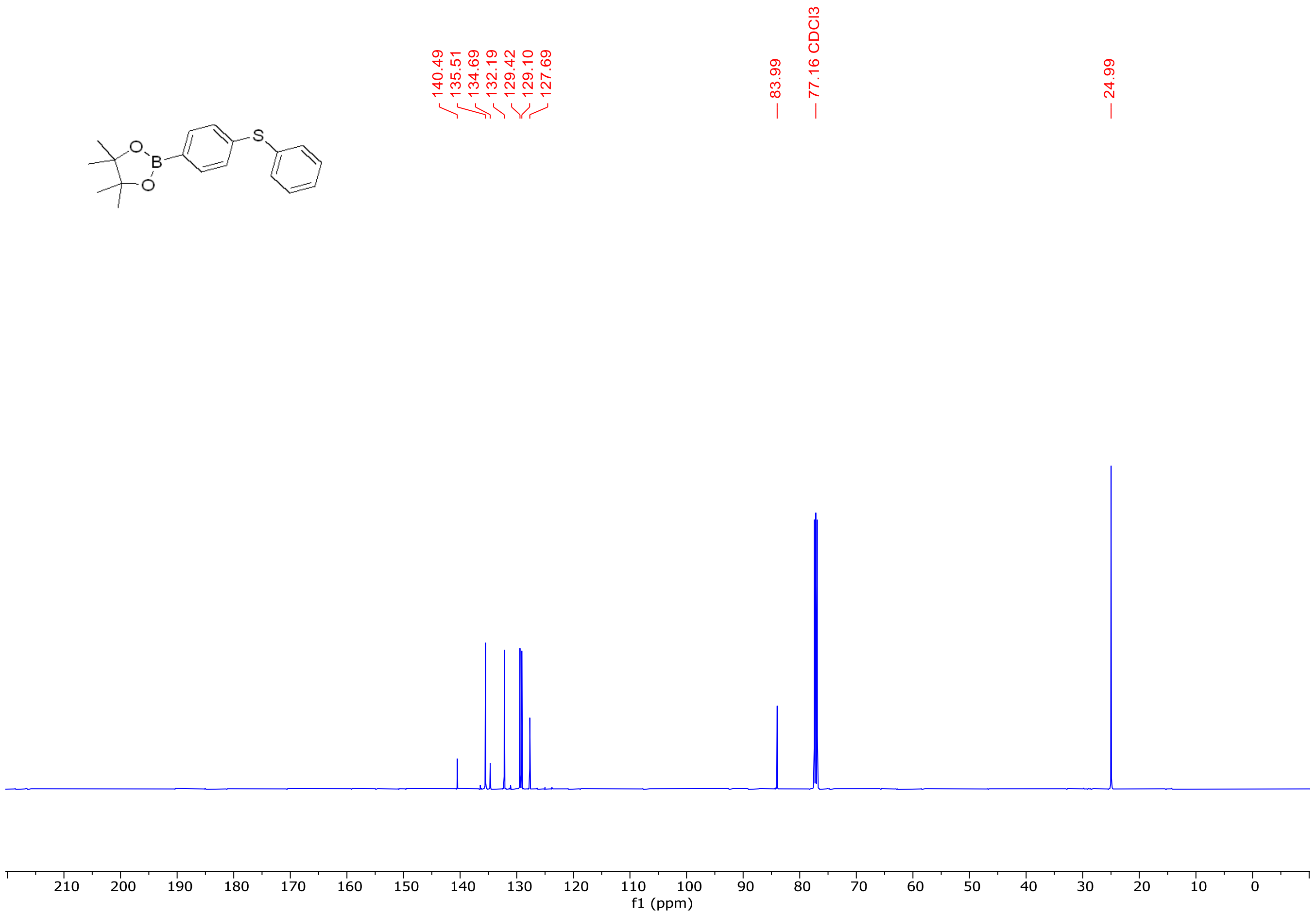

naphthalen-2-yl(phenyl)sulfane (93)

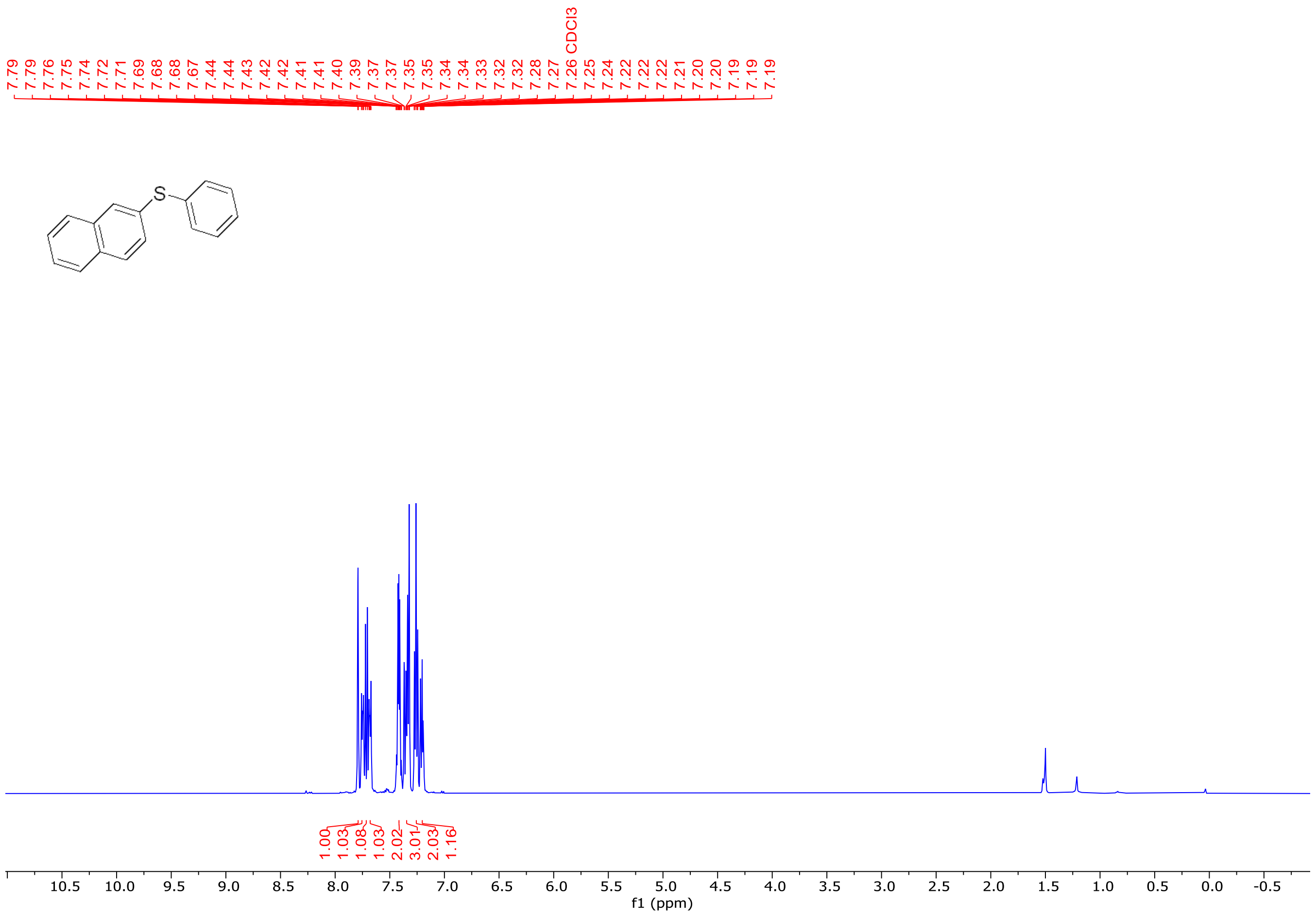

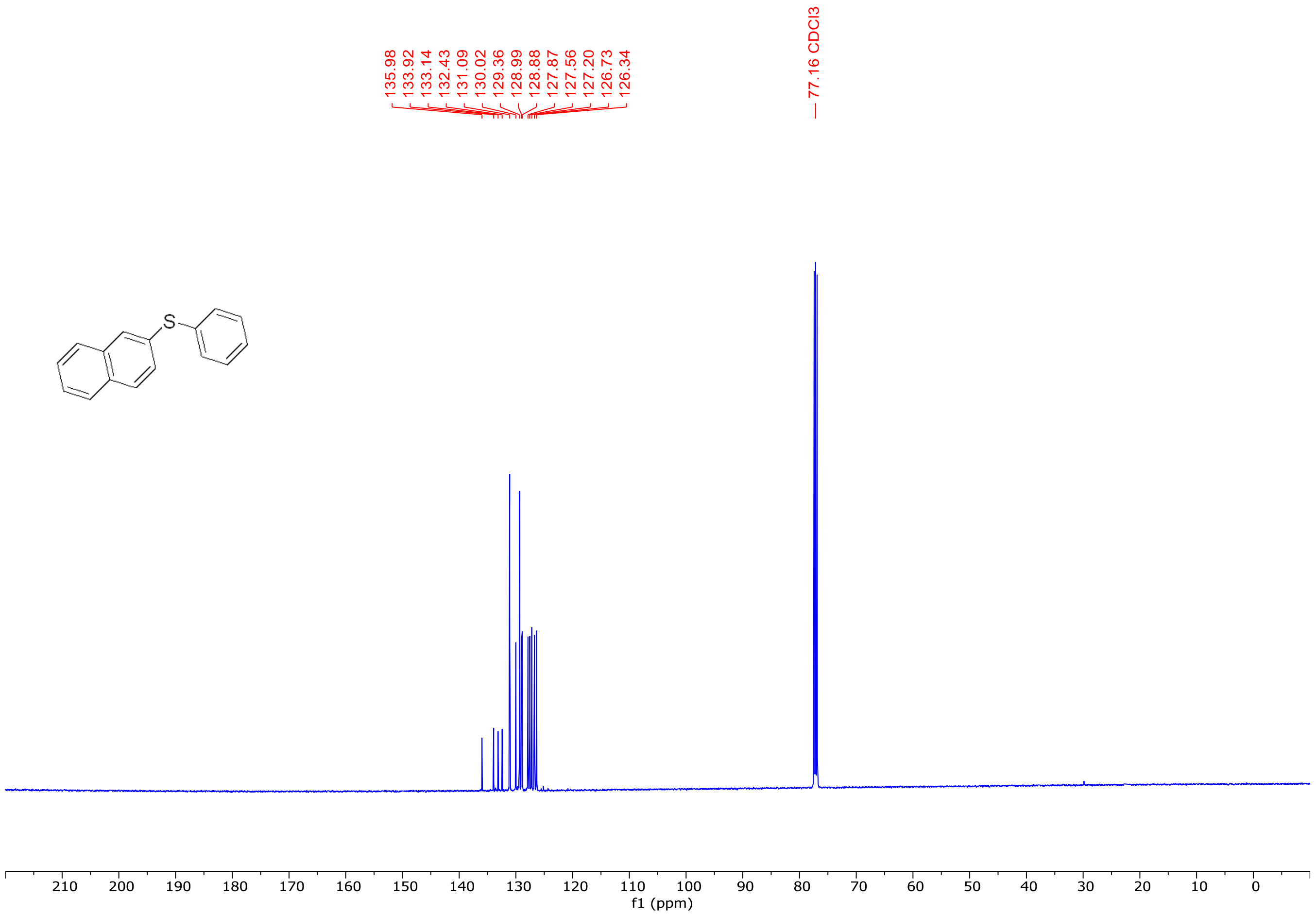

3-(phenylthio)quinoline (94)

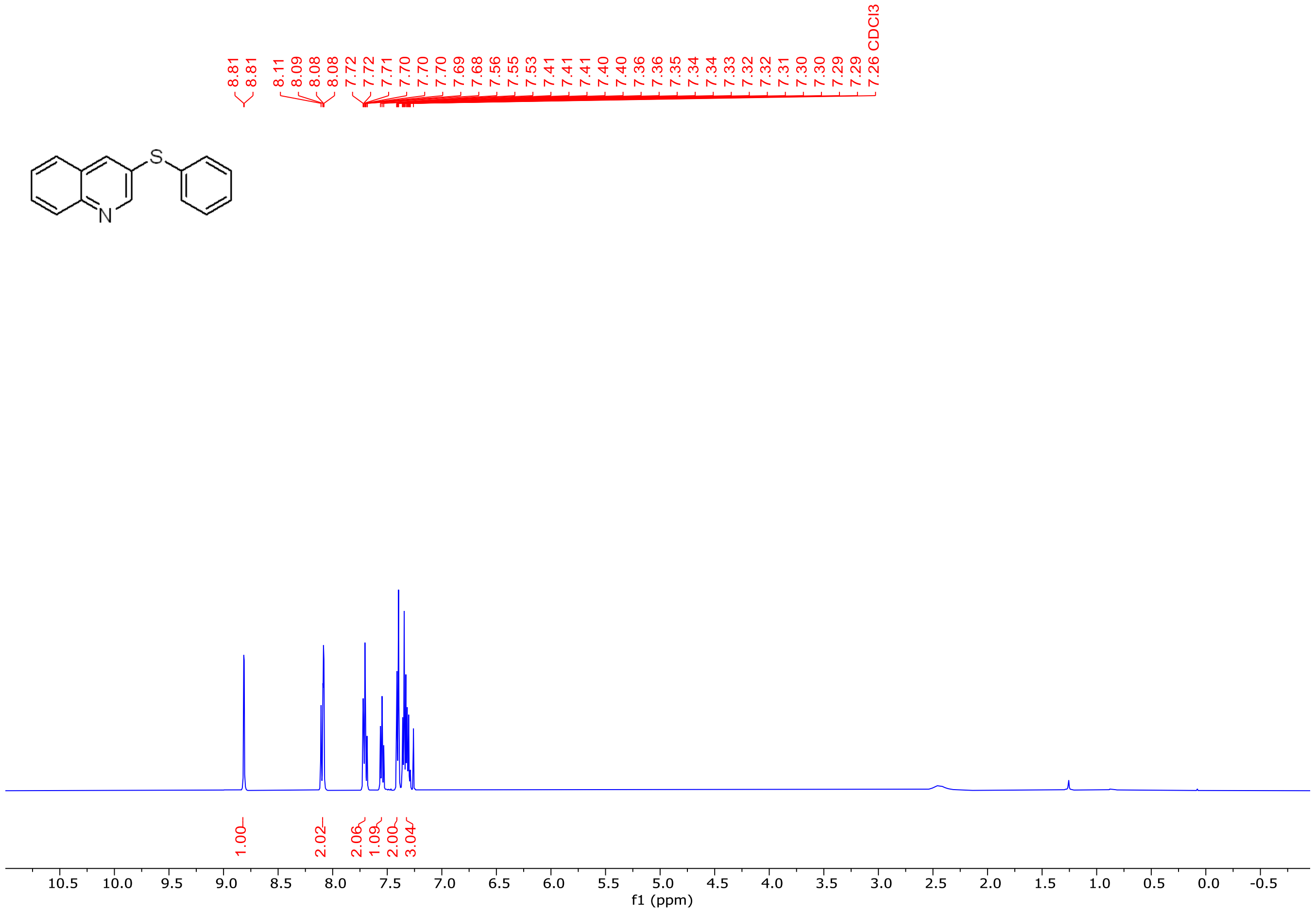

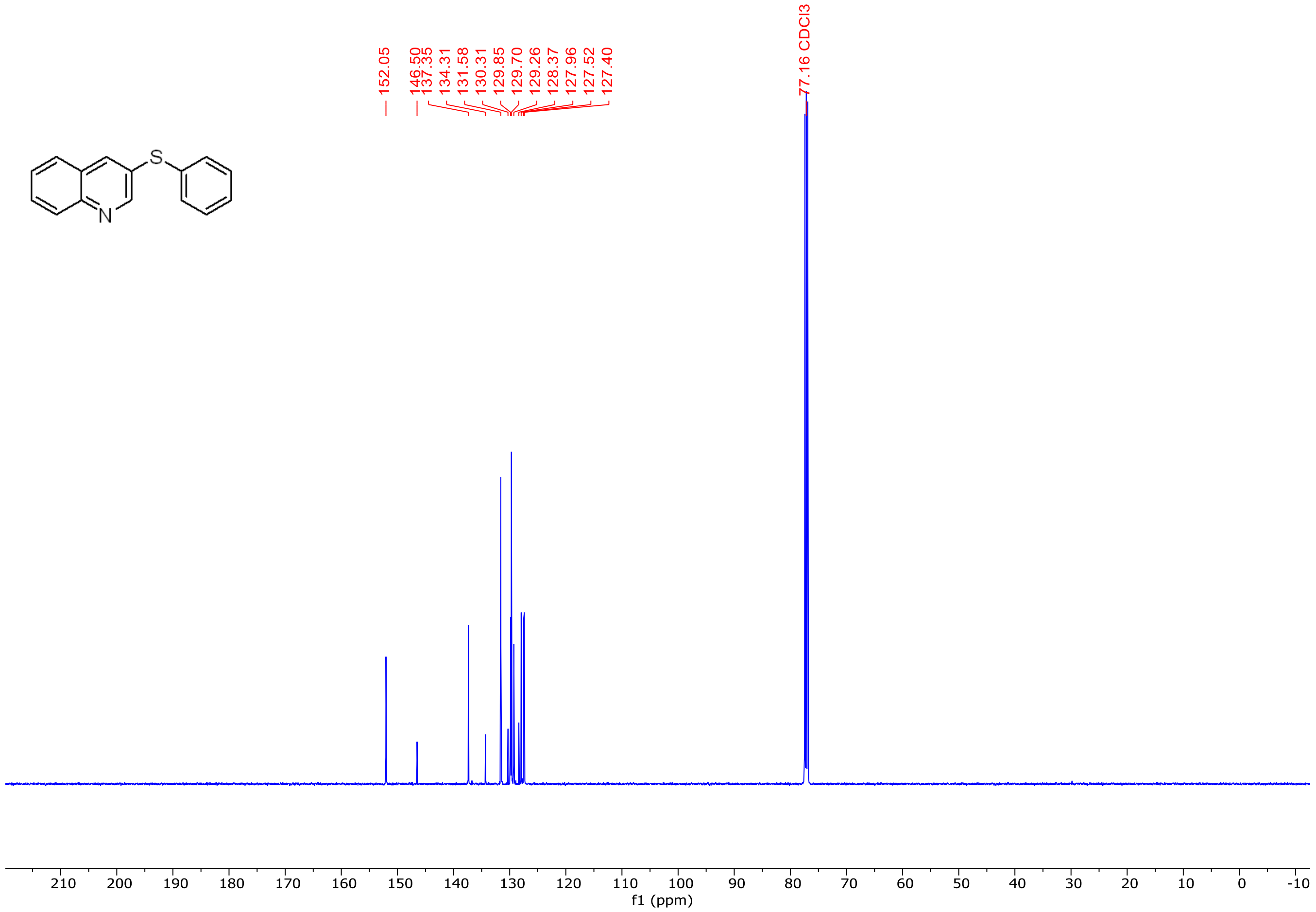

4-((3-methoxyphenyl)thio)benzonitrile (95)

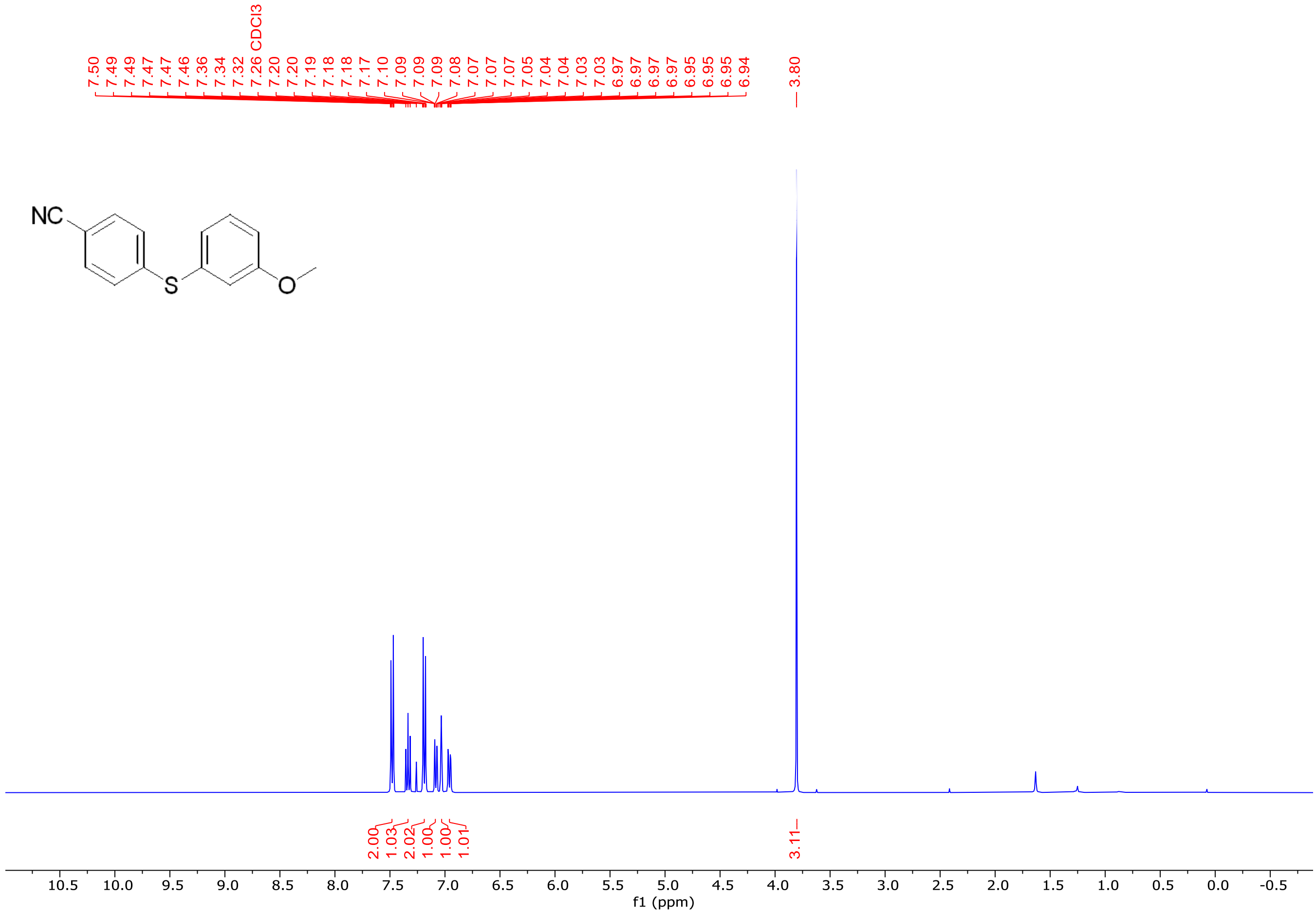

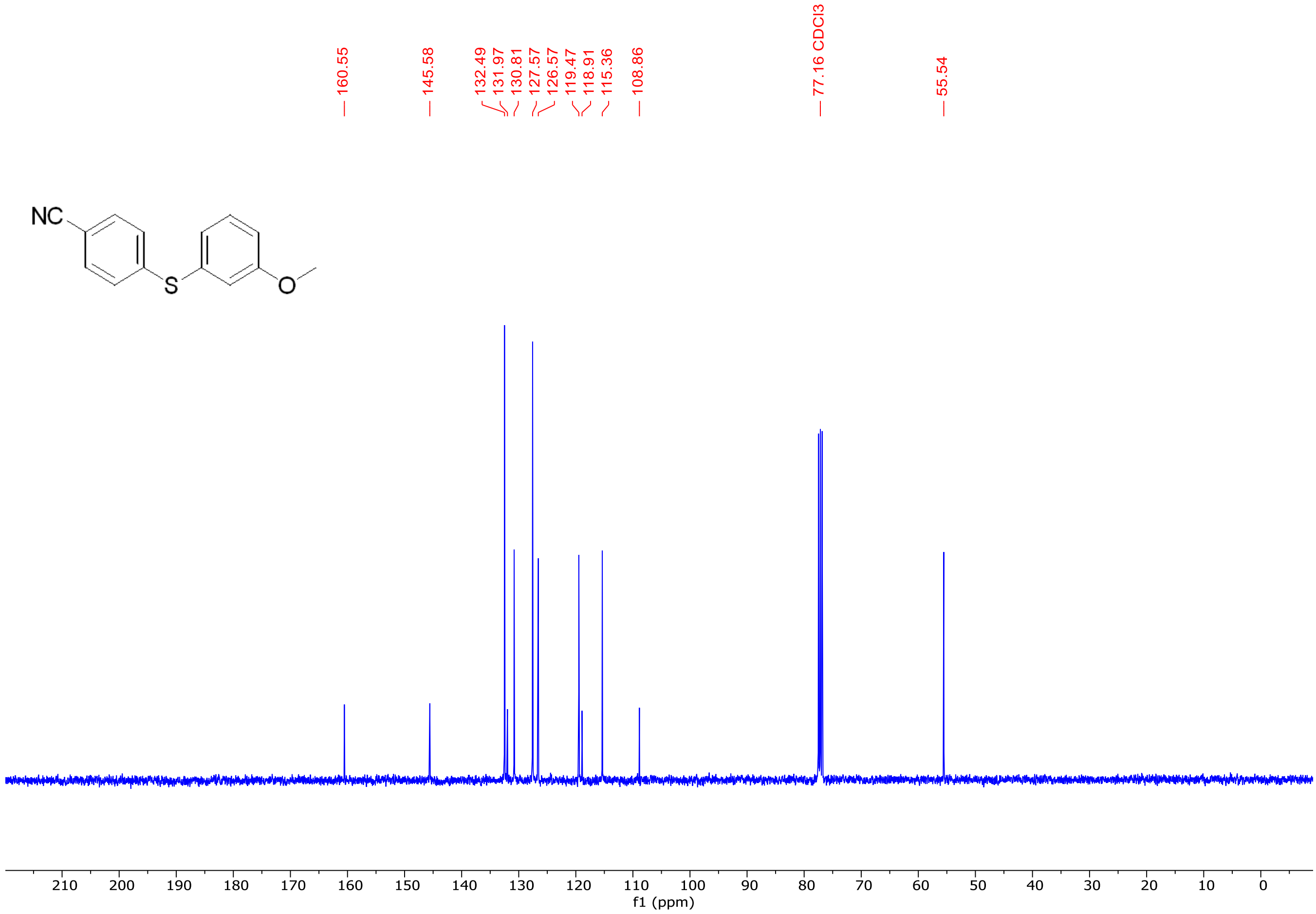

4-phenyl-2-(phenylthio)thiazole (96)

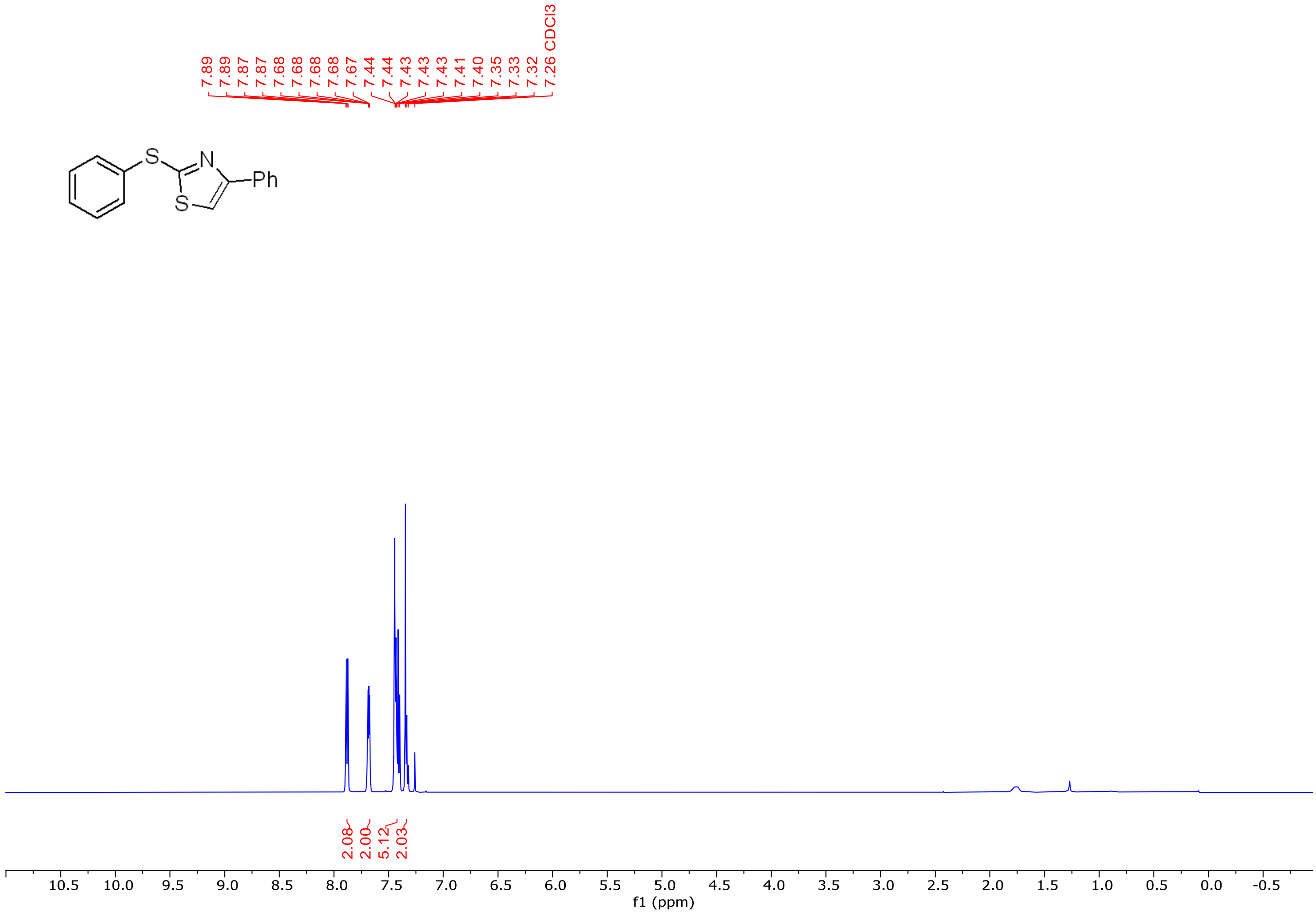

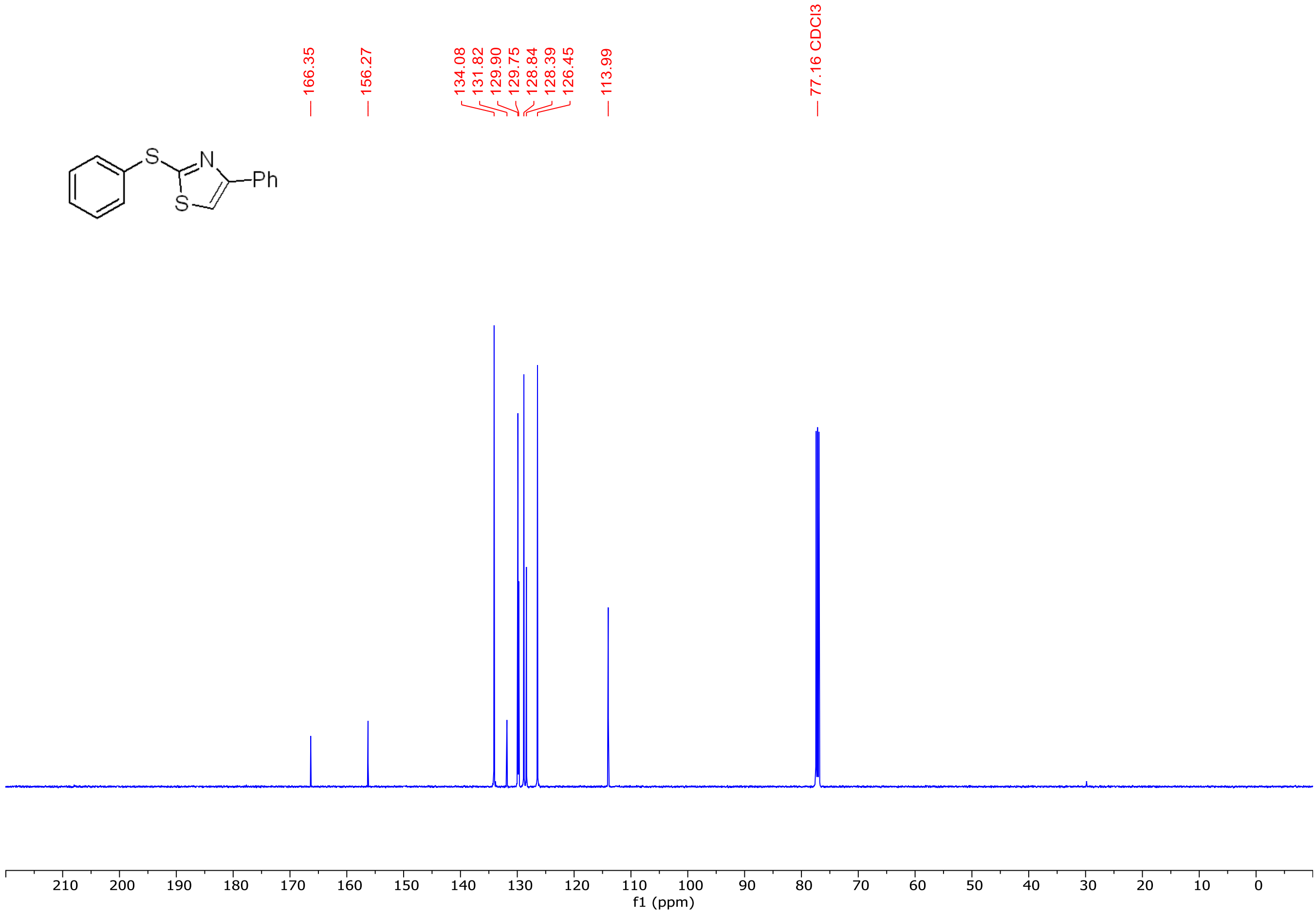

4-tosyl-1,1'-biphenyl (97)

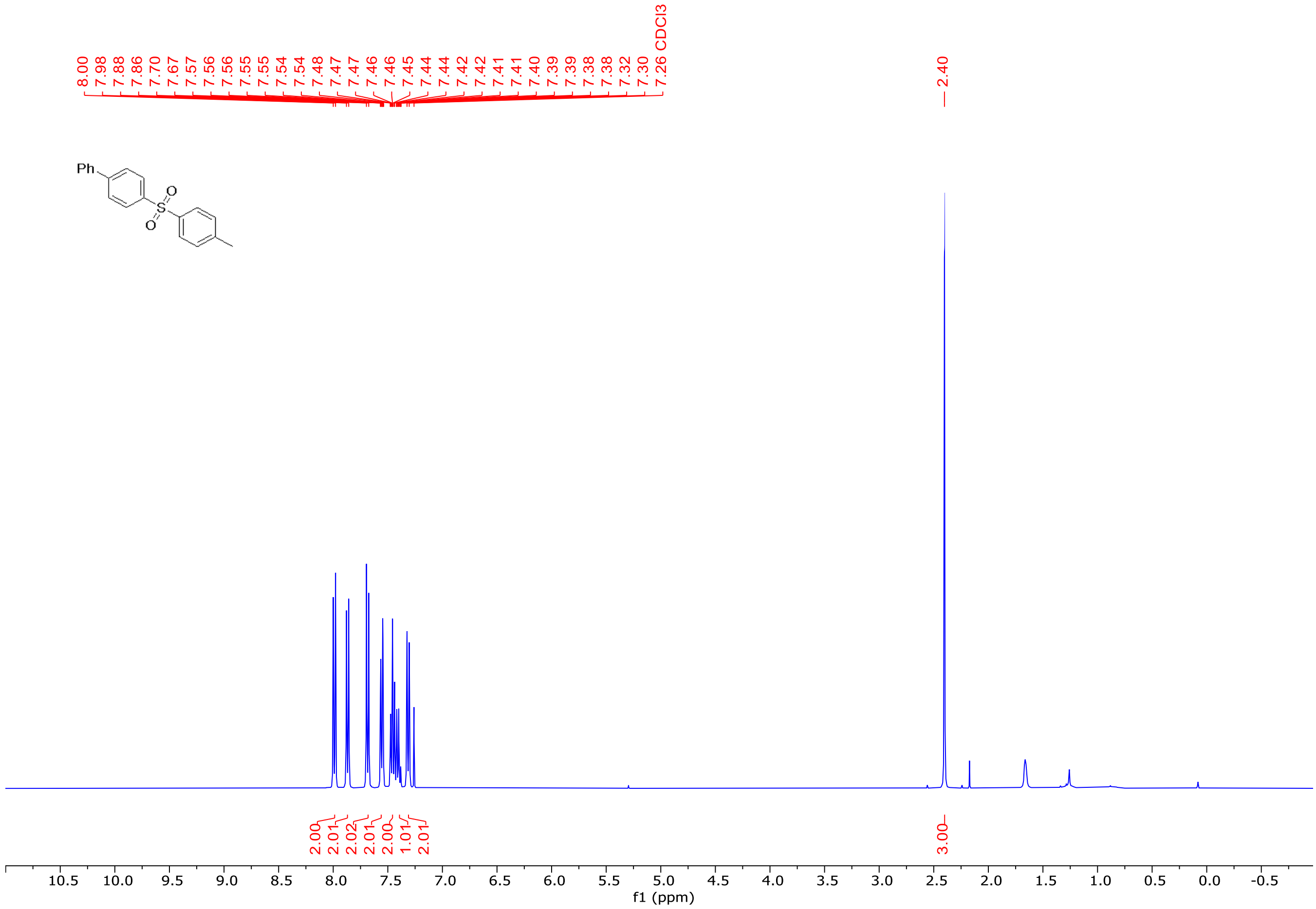

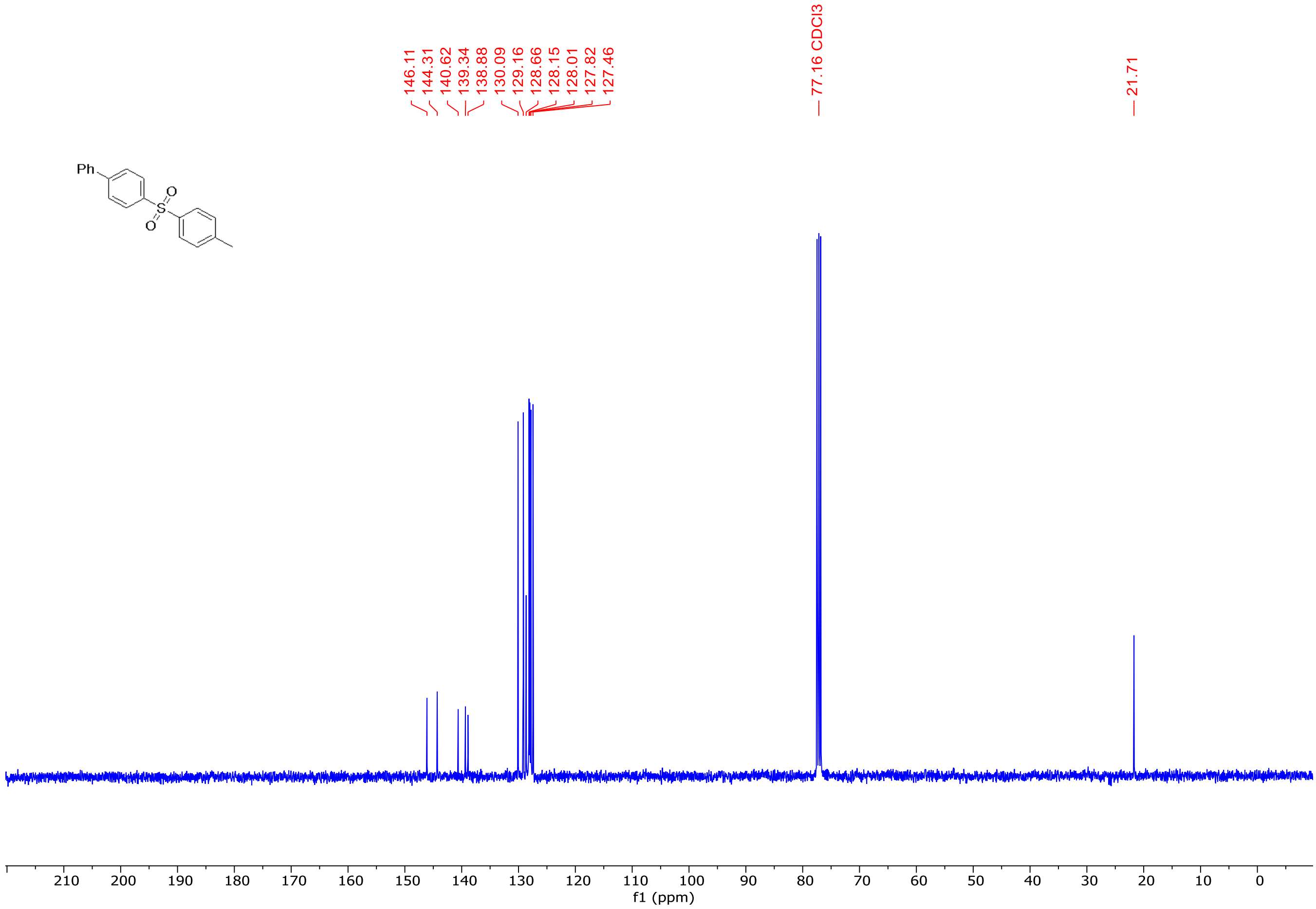

1-methoxy-4-tosylbenzene (98)

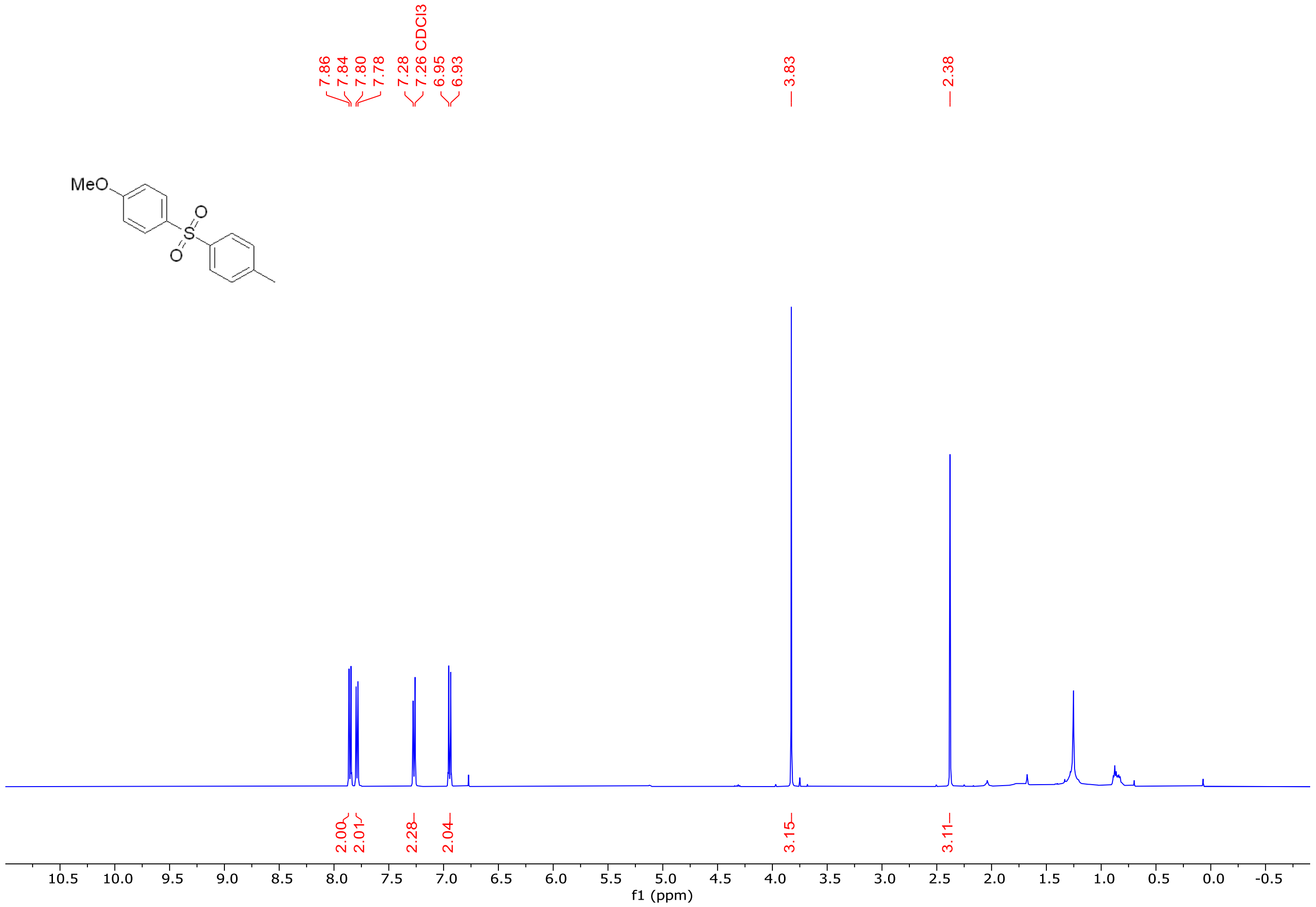

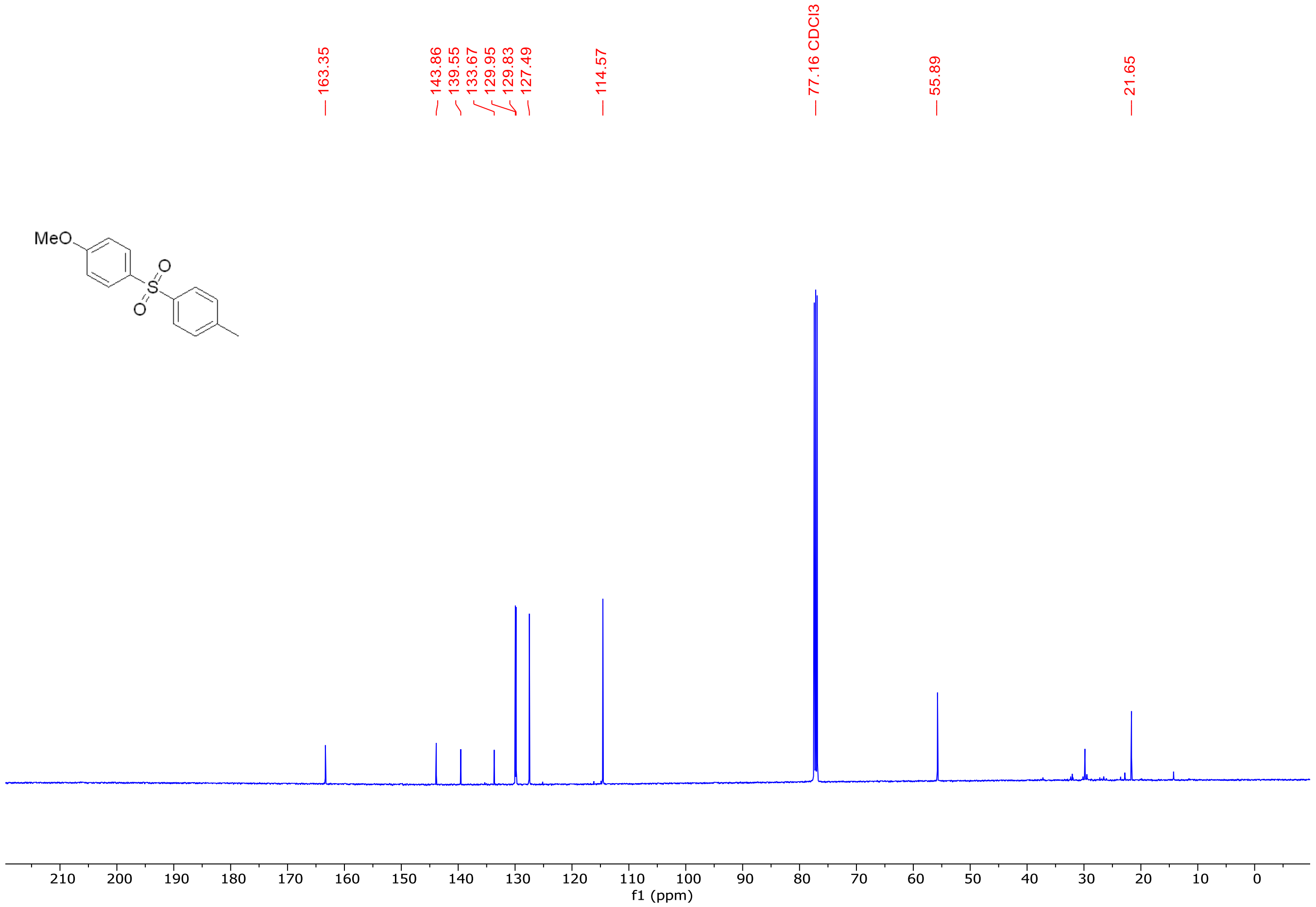

methyl(4-tosylphenyl)sulfane(99)

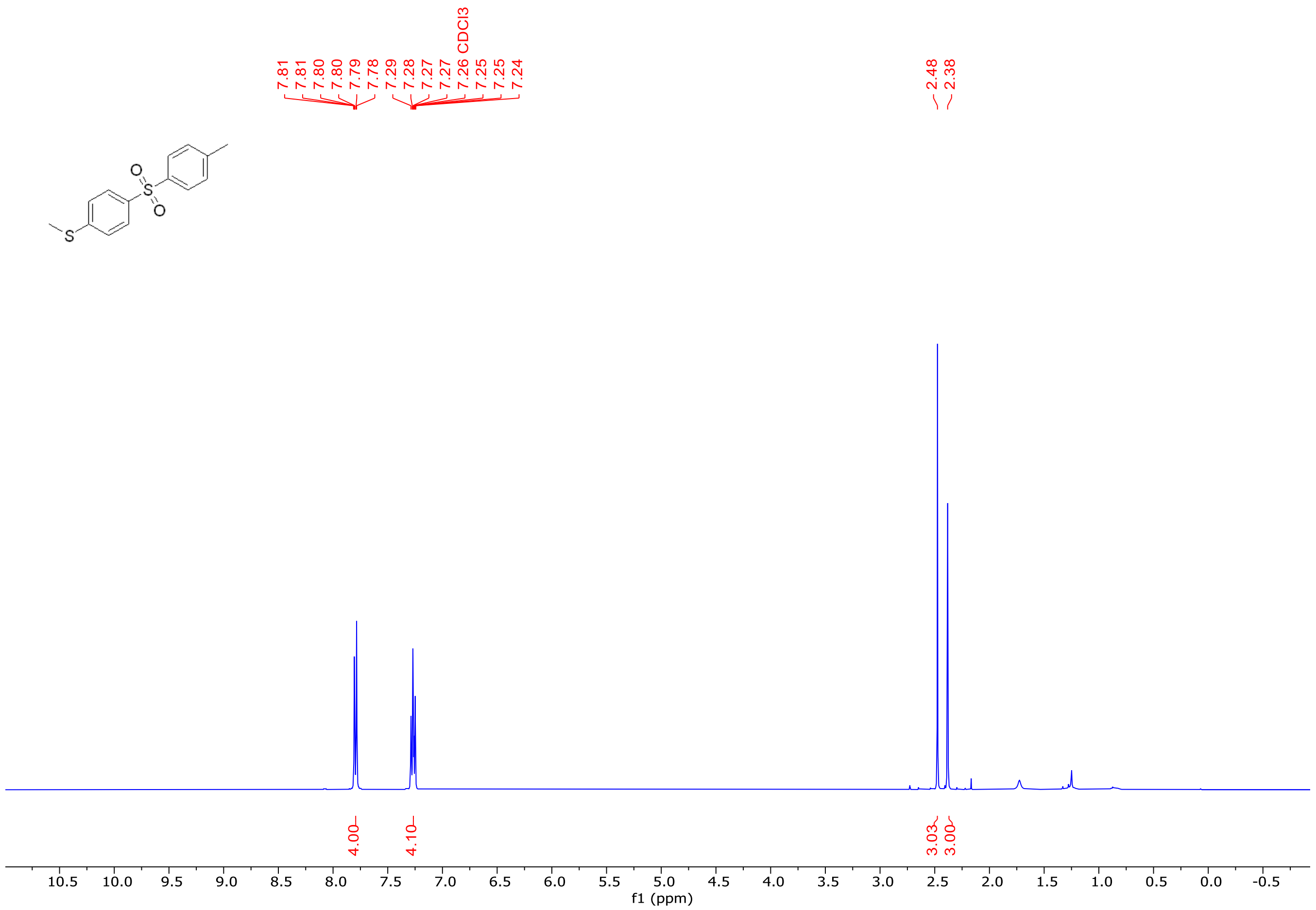

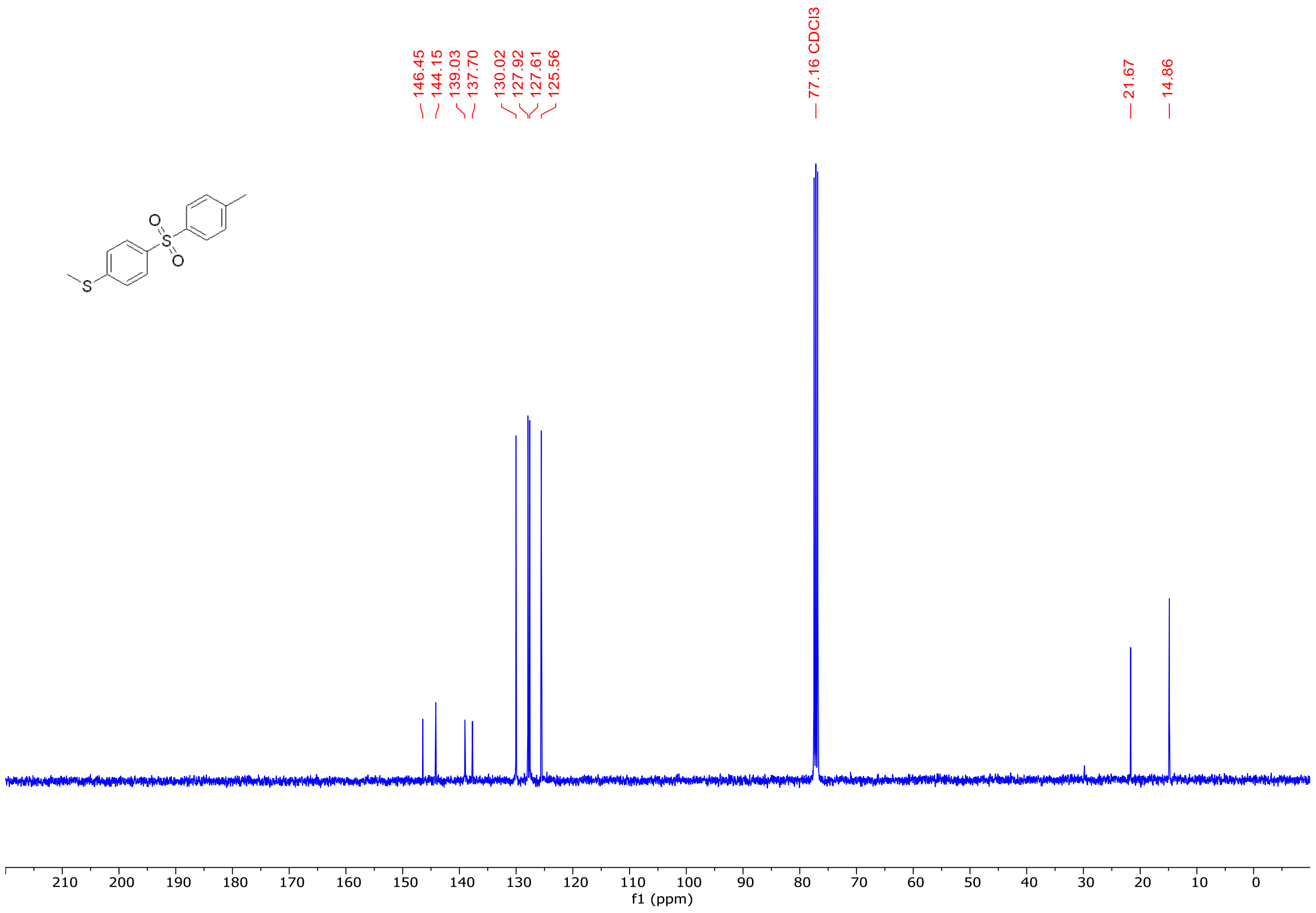

trimethyl(4-tosylphenyl)silane(100)

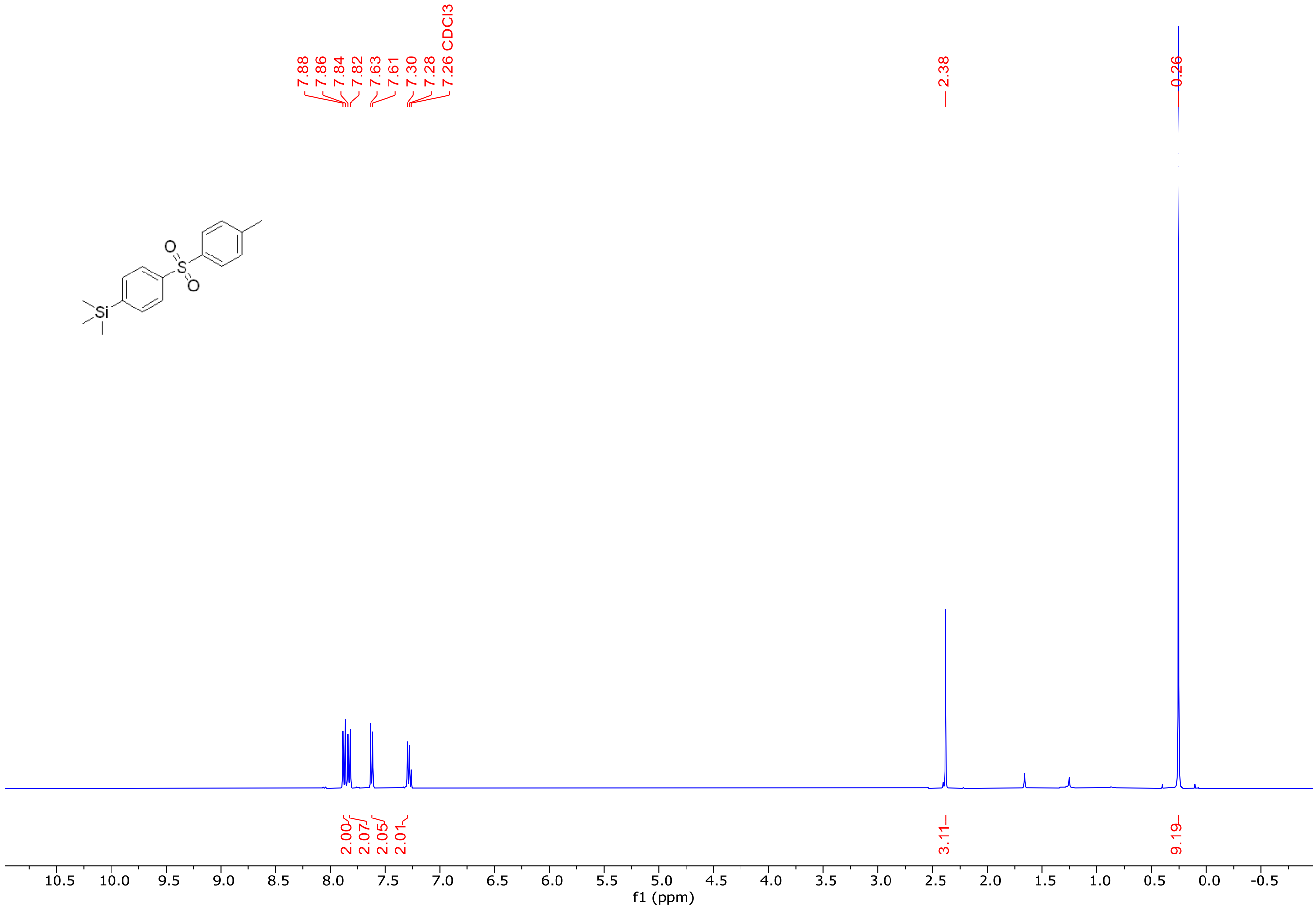

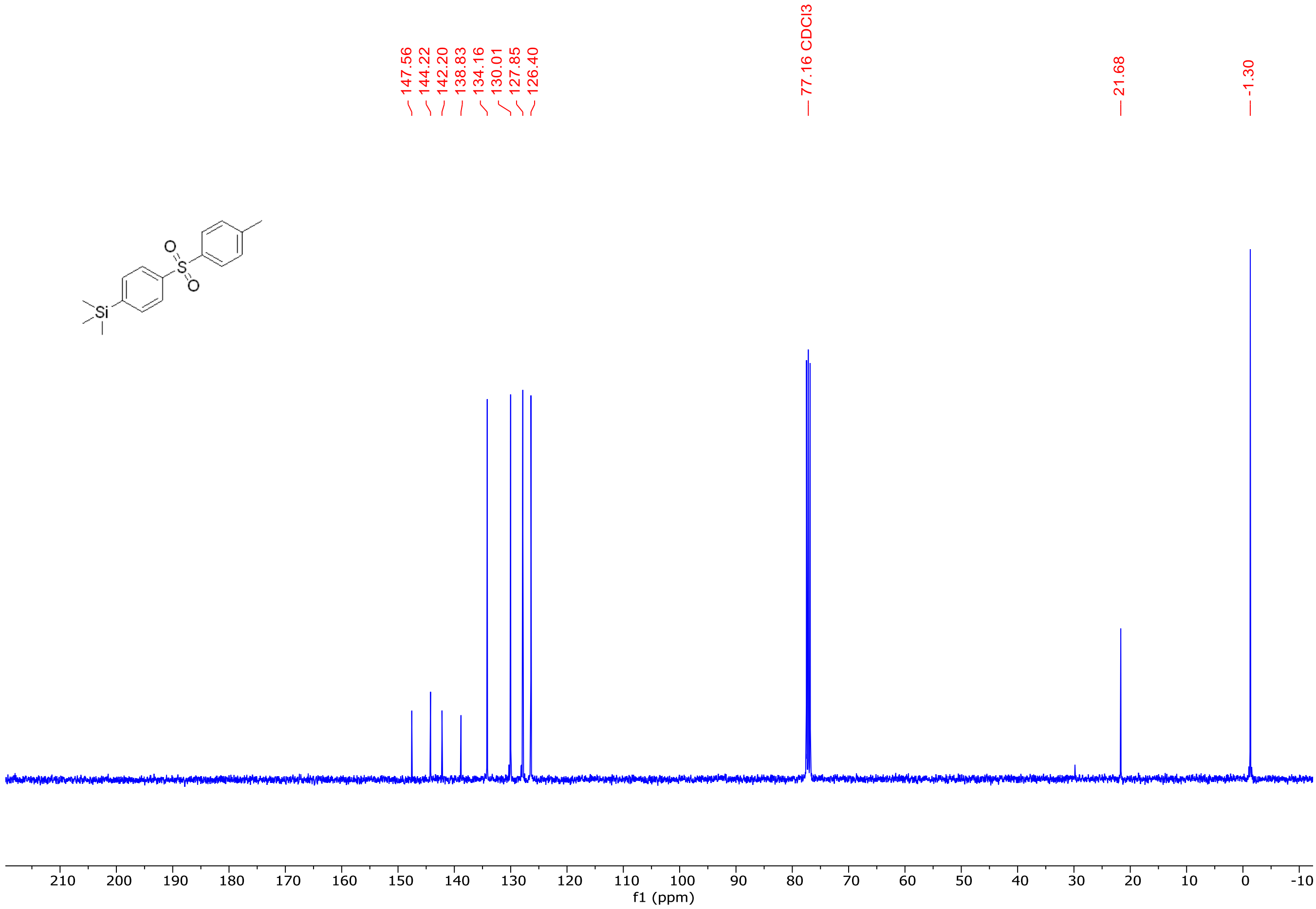

4-tosylbenzonitrile(101)

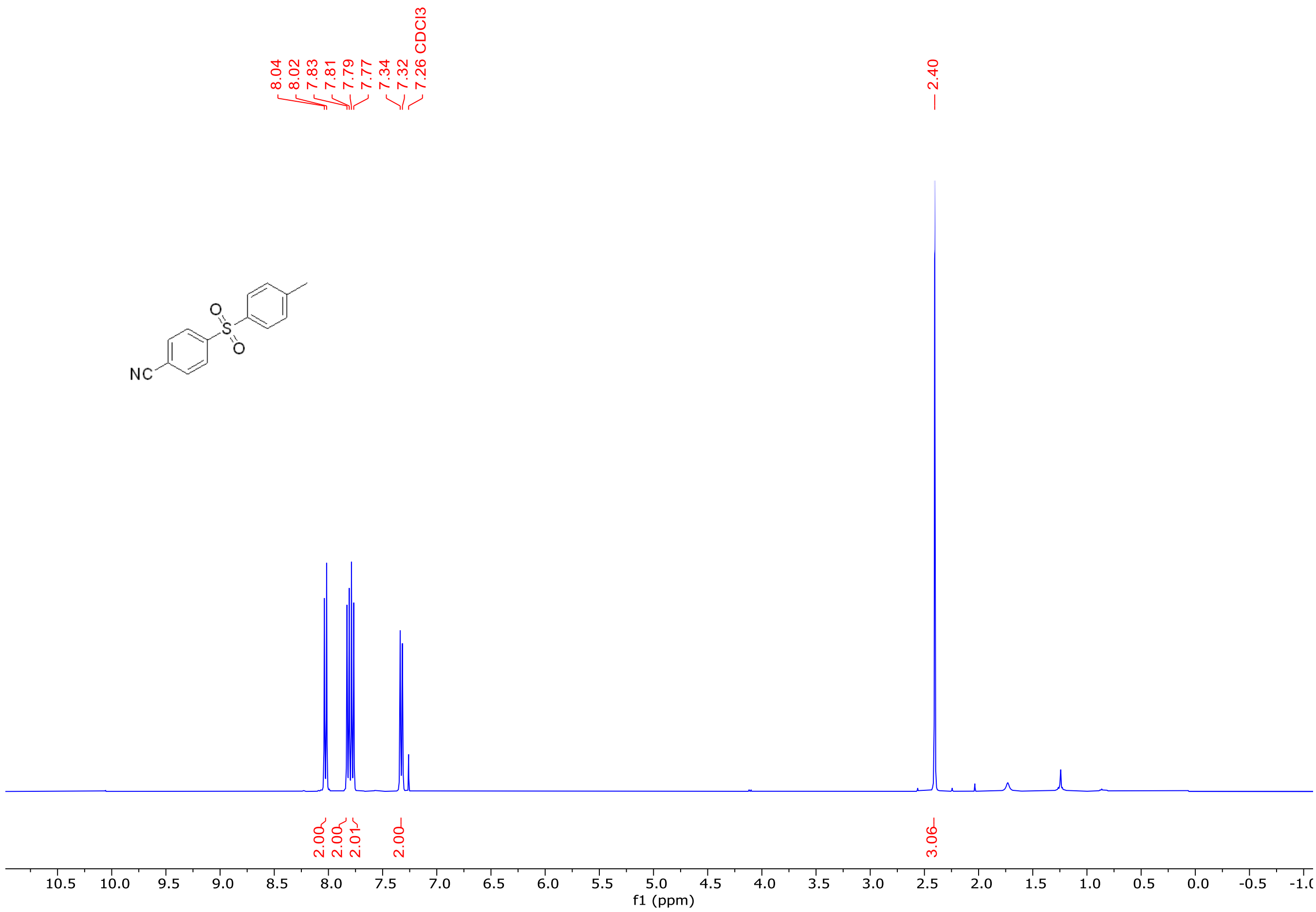

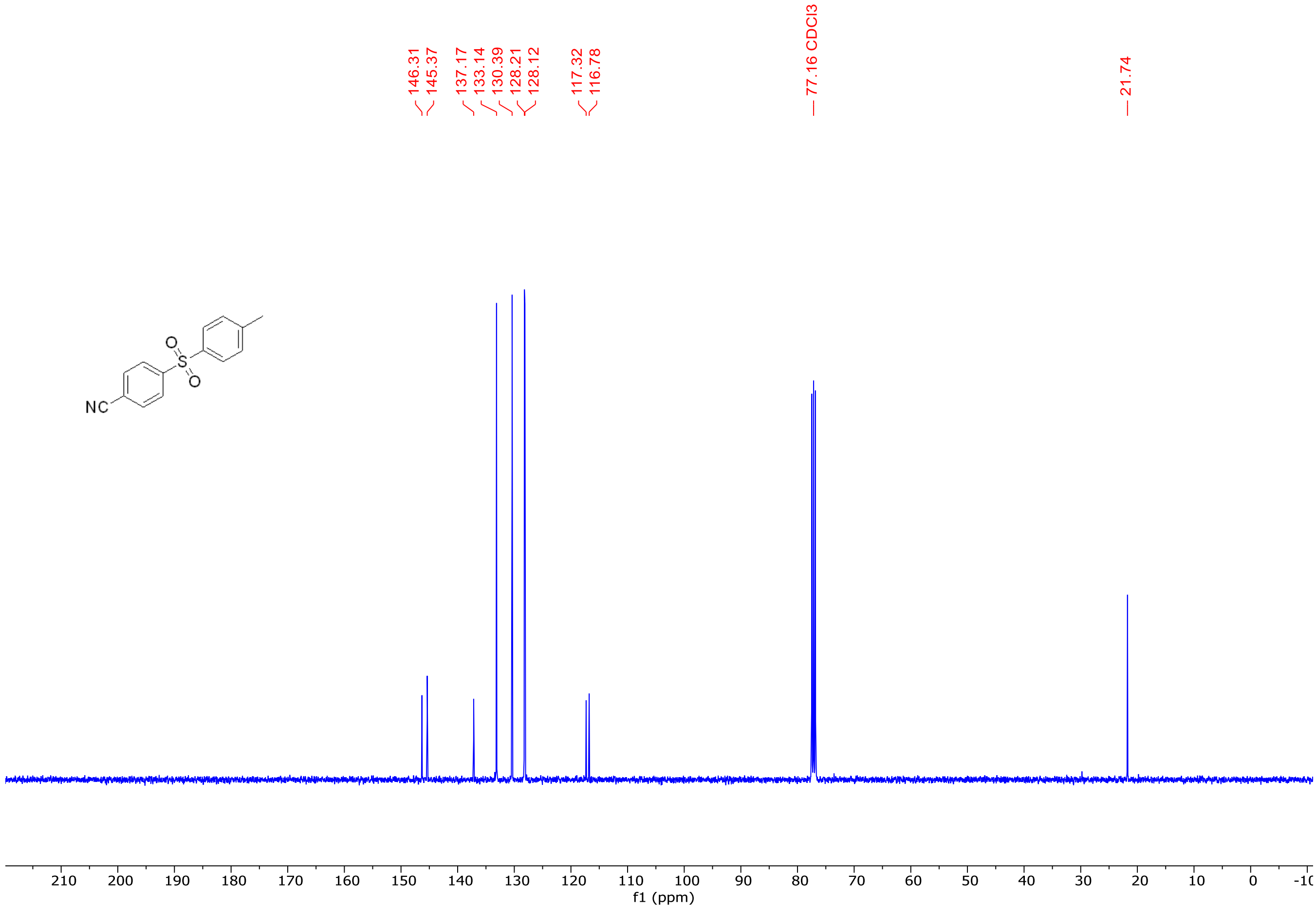

1-methyl-4-((4-(trifluoromethyl)phenyl)sulfonyl)benzene (102)

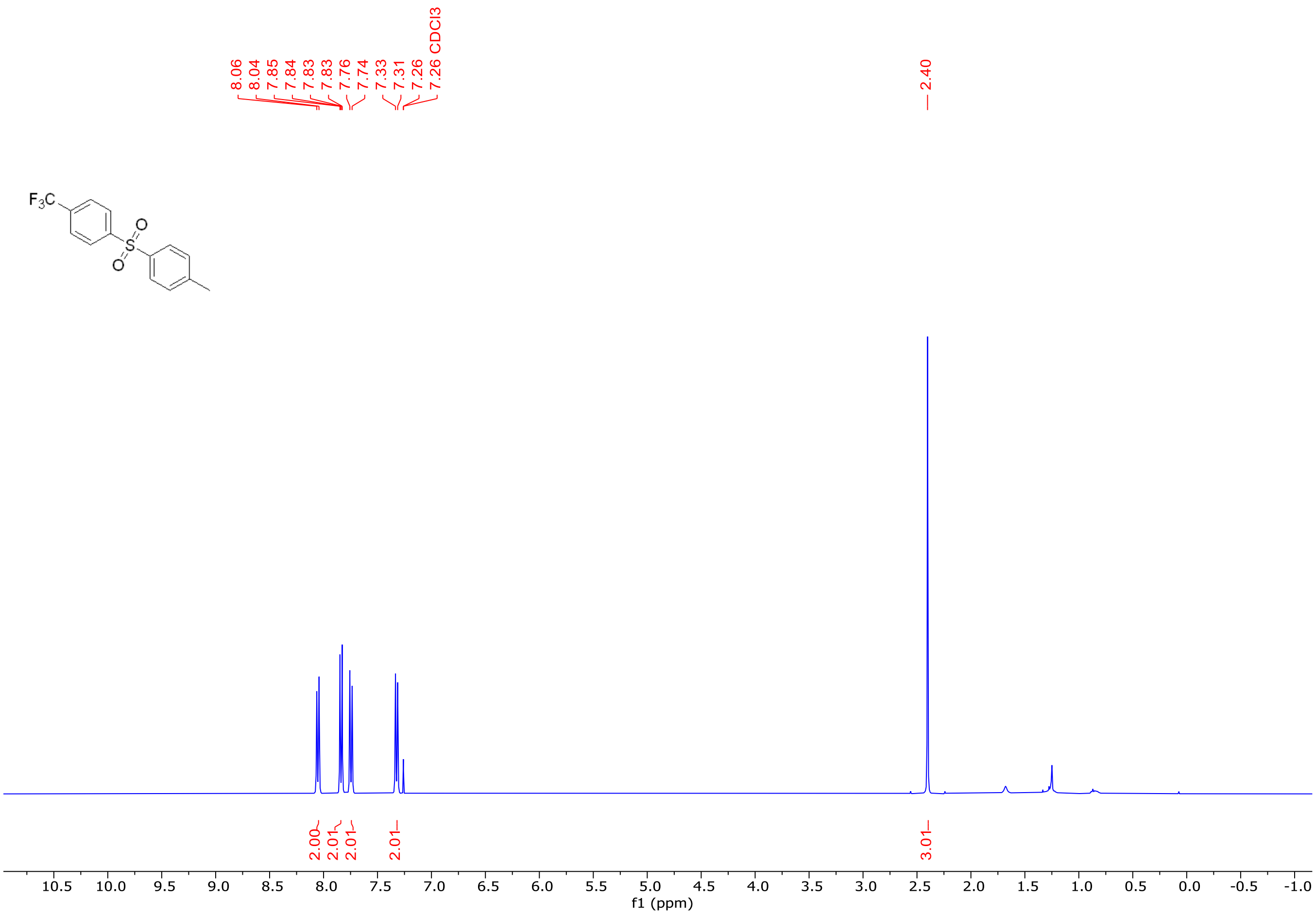

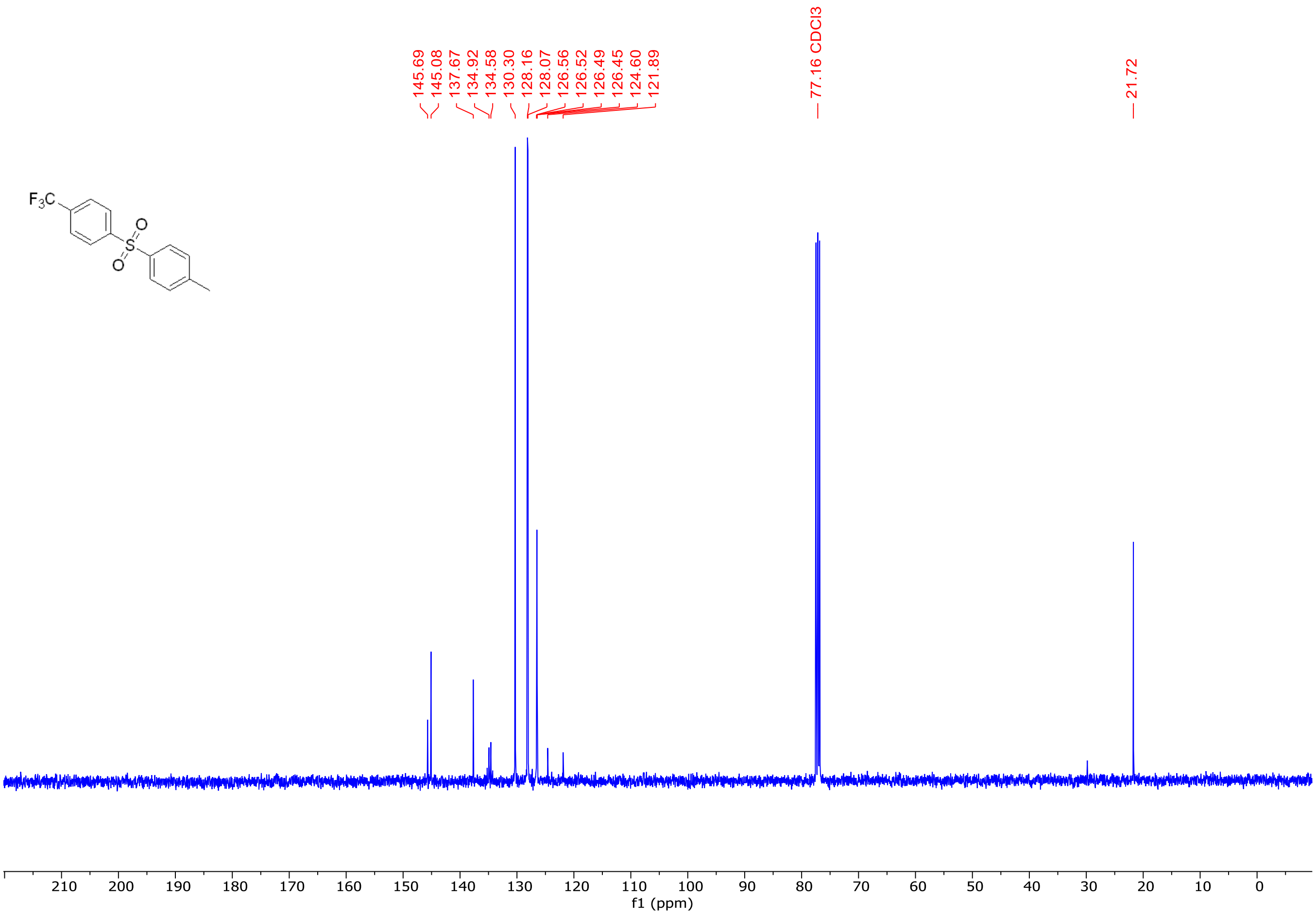

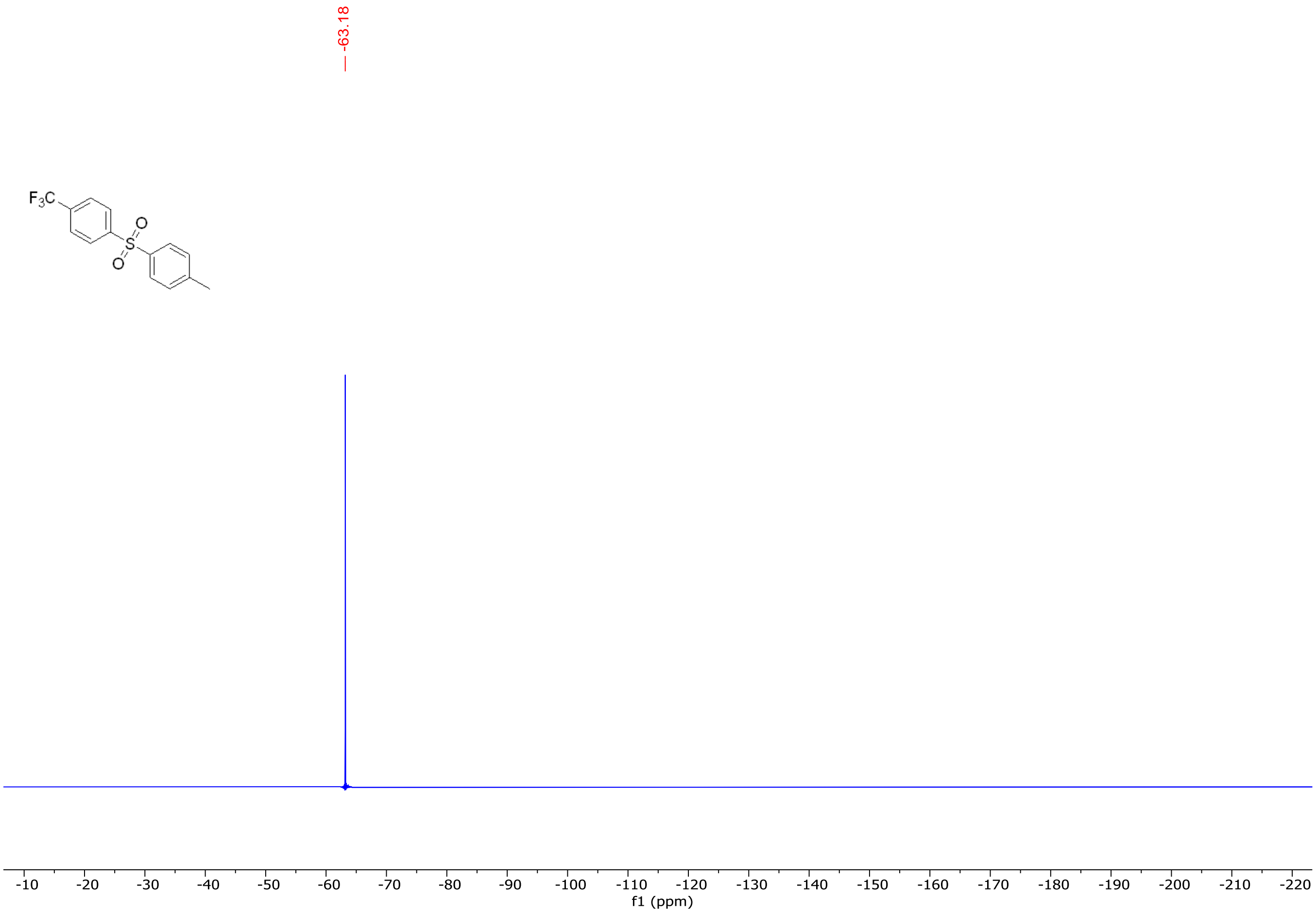
-63.18
F3C
S
O
O
-10 -20 -30 -40 -50 -60 -70 -80 -90 -100 -110 -120 -130 -140 -150 -160 -170 -180 -190 -200 -210 -220
f1 (ppm)

1-methyl-4-((4-(methylsulfonyl)phenyl)sulfonyl)benzene (103)

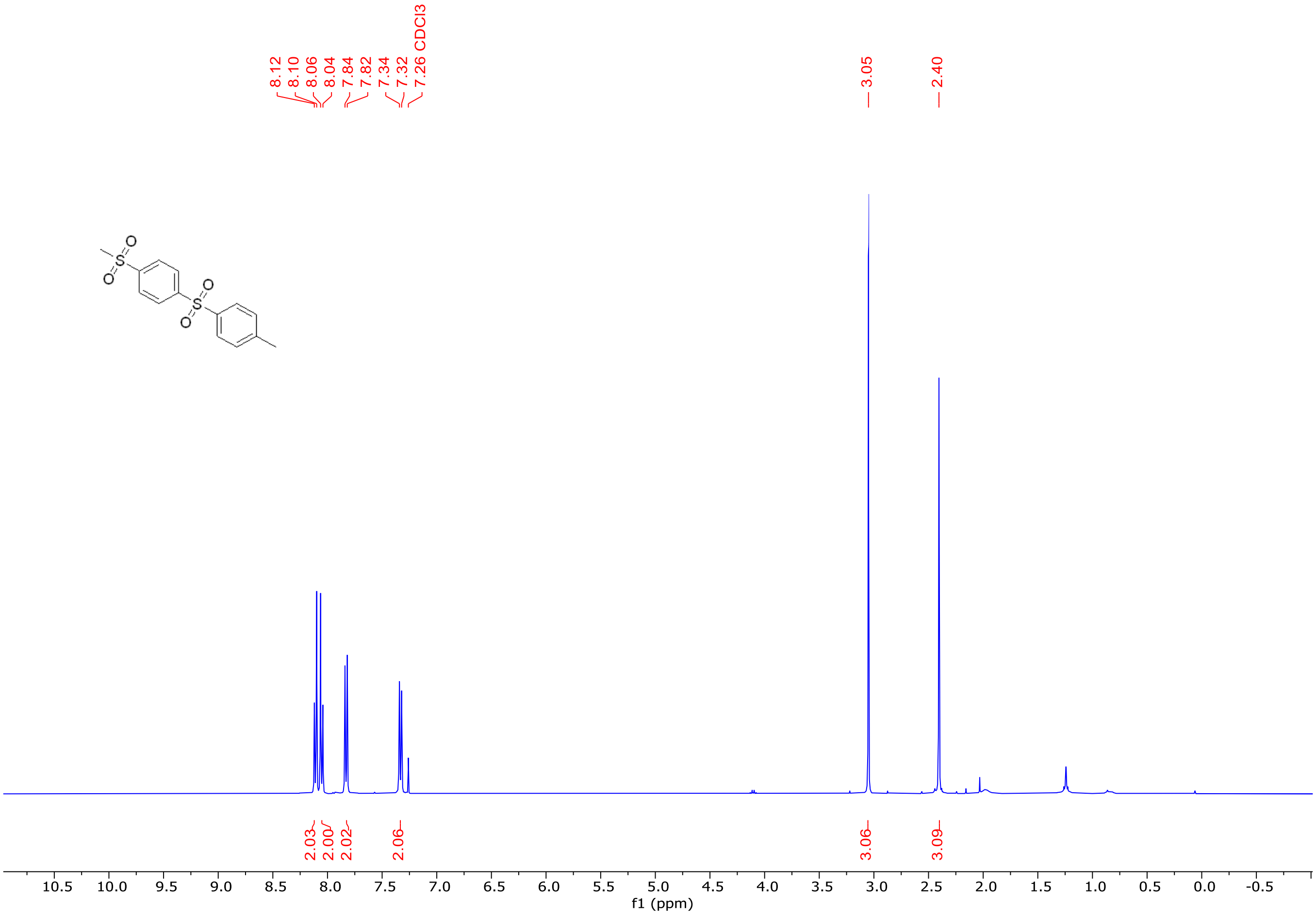

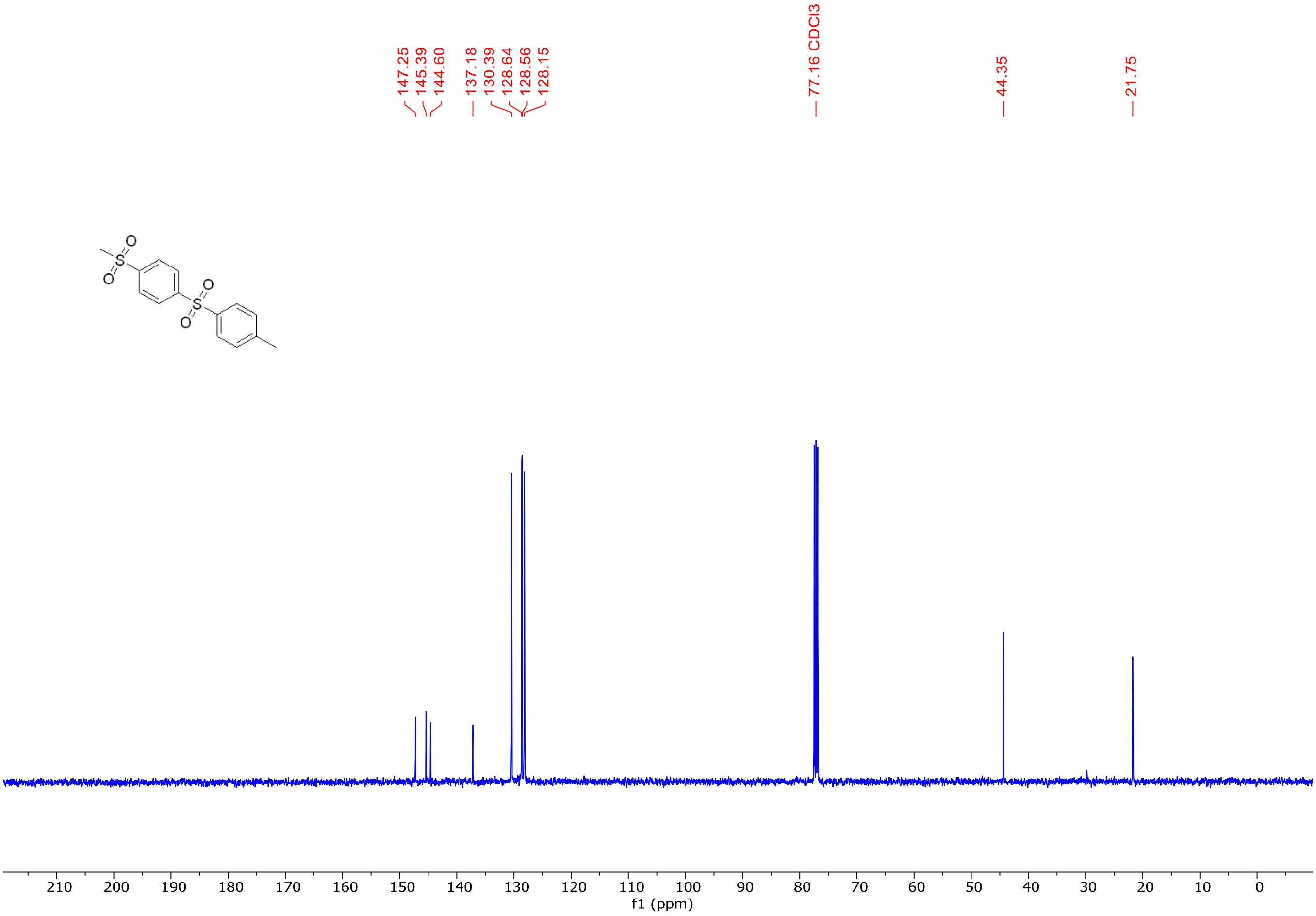

1-(4-tosylphenyl)ethan-1-one (104)

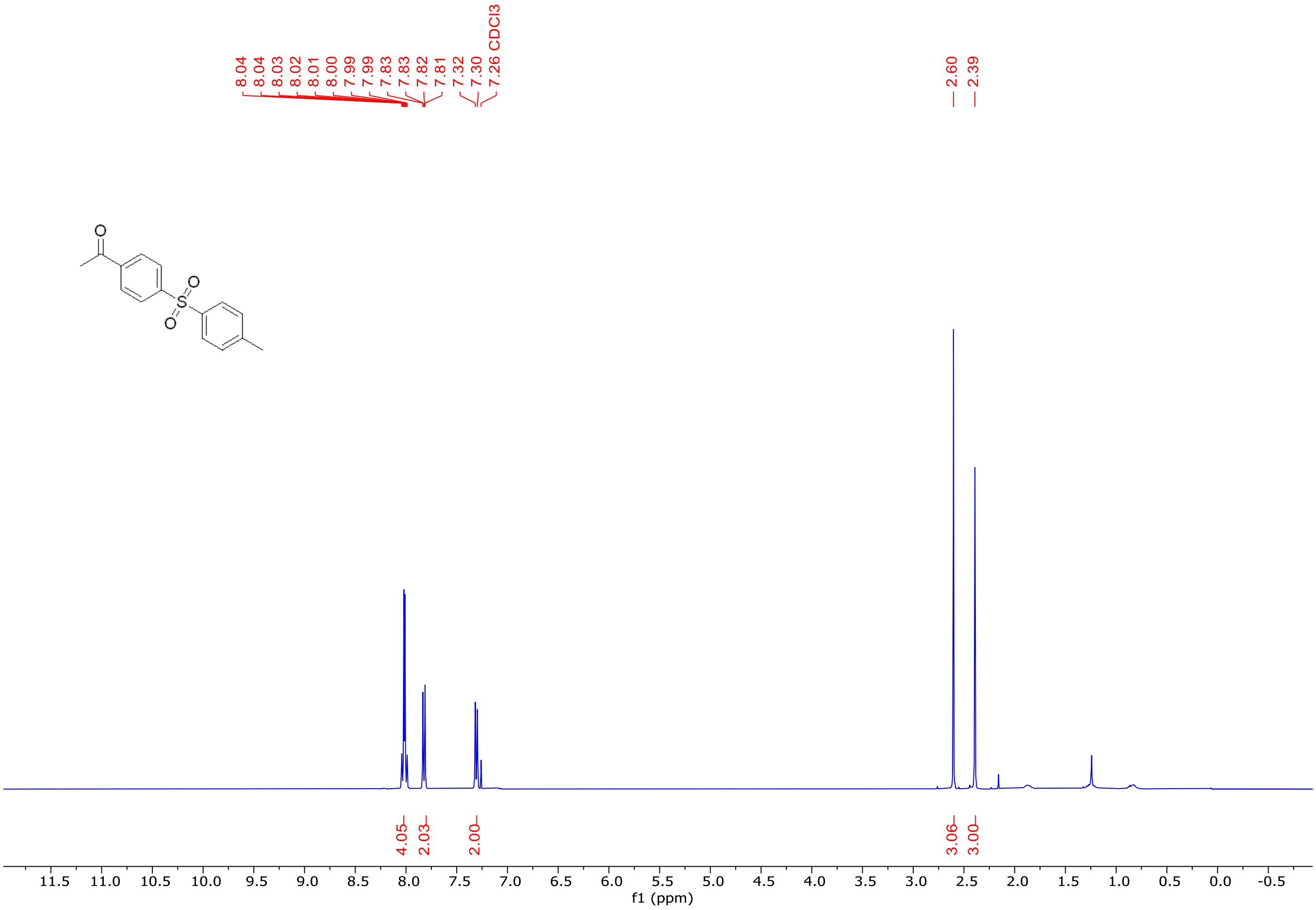

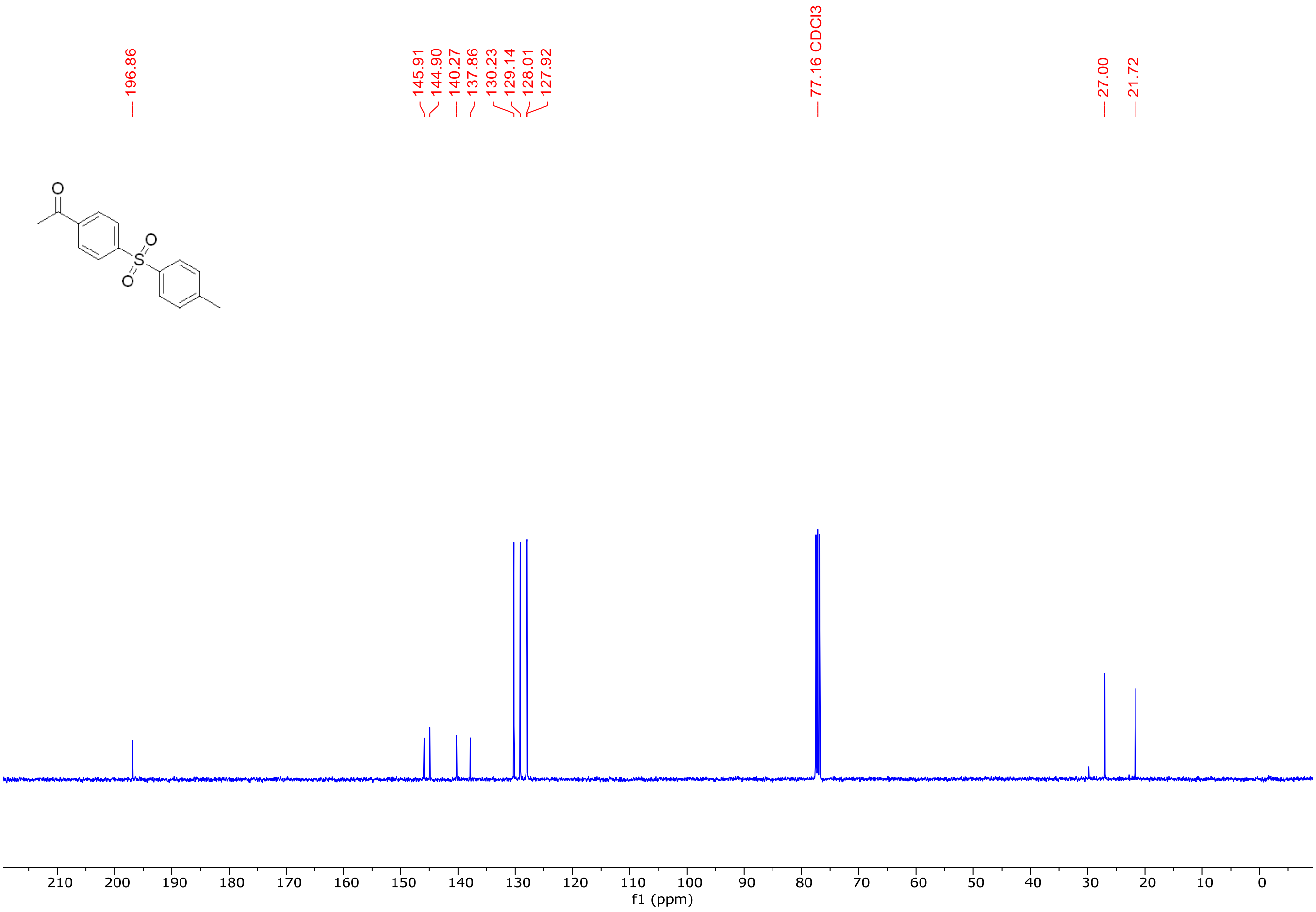

5-tosylisobenzofuran-1(3H)-one (105)

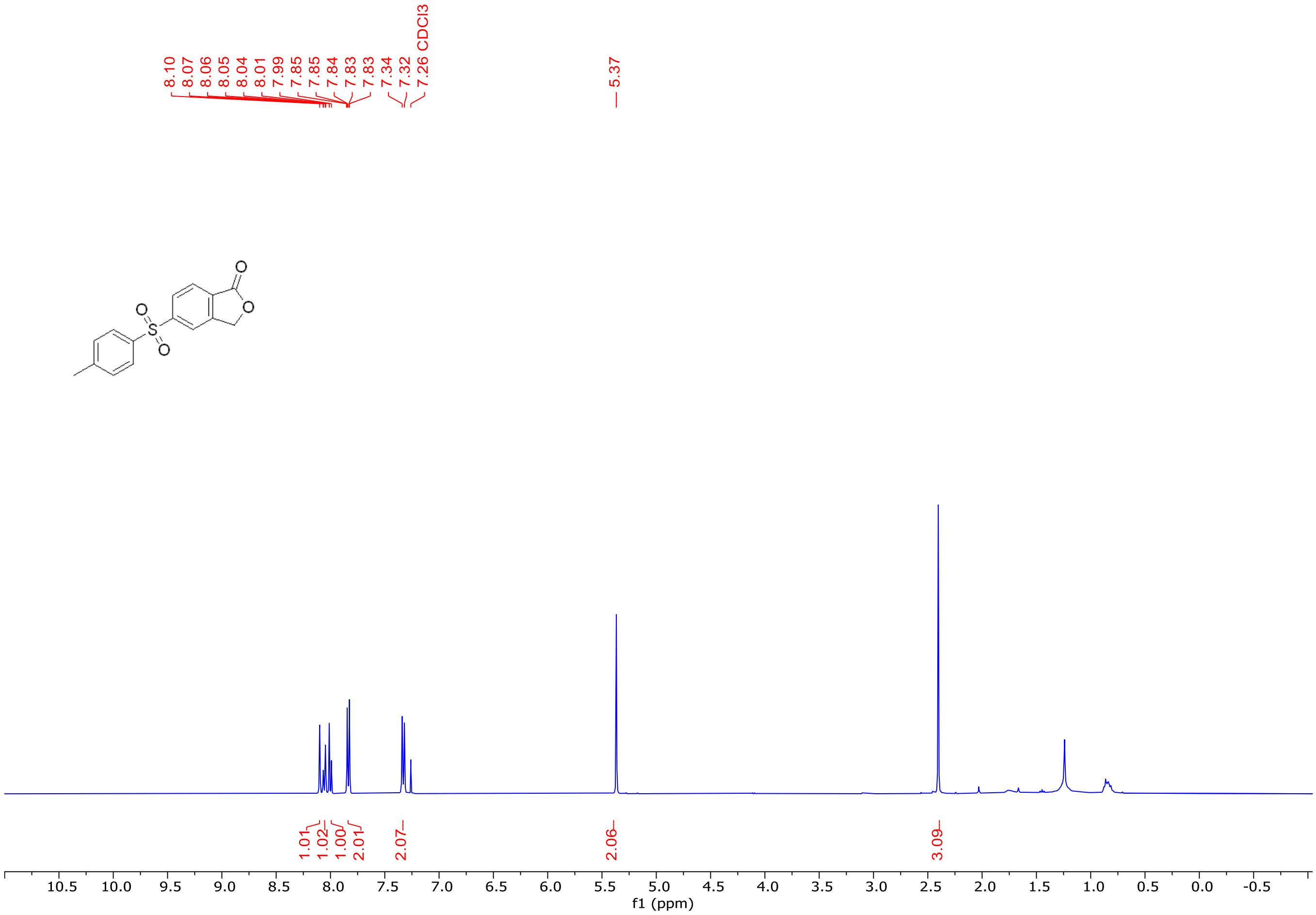

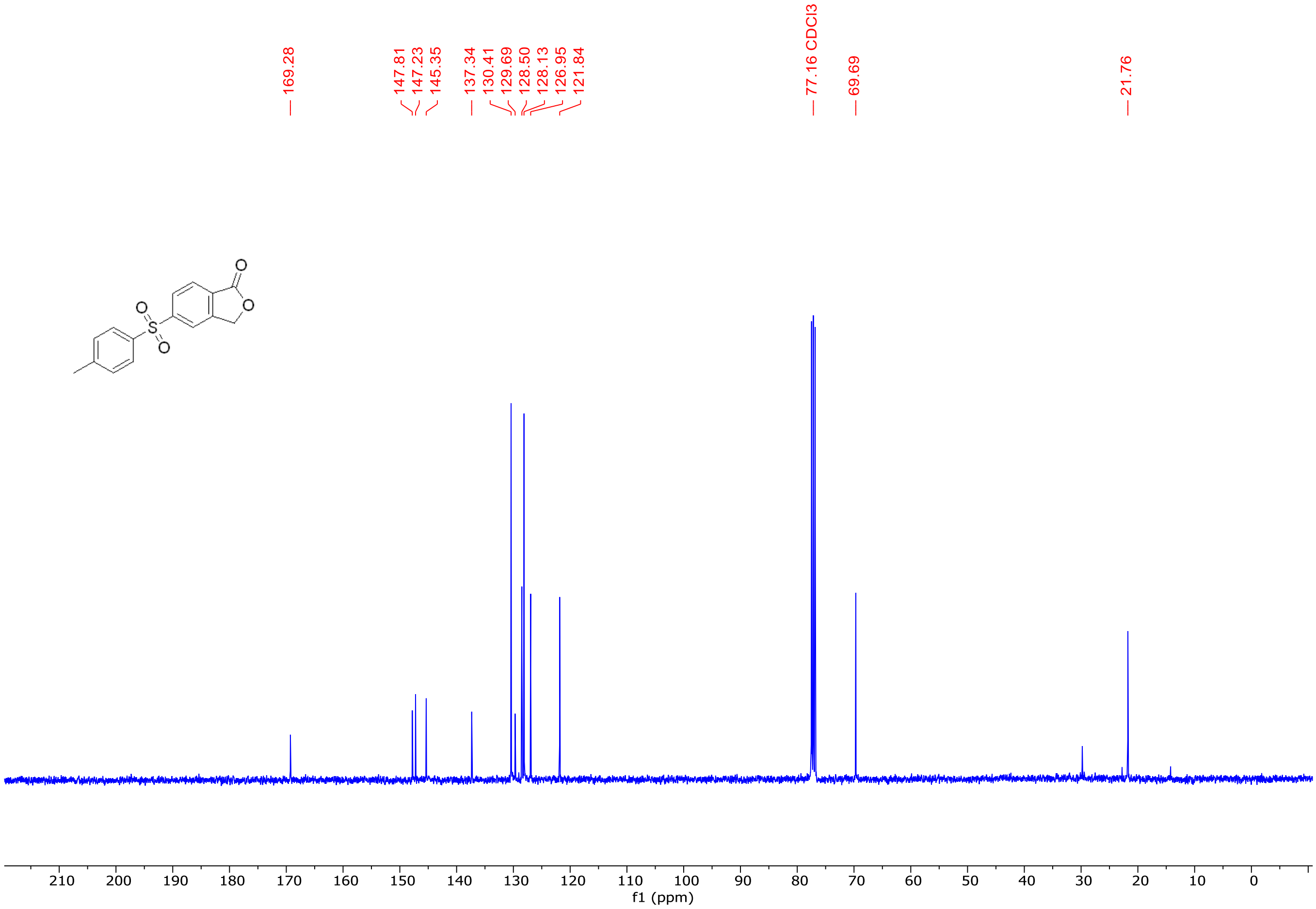

2-methyl-5-tosylisoindoline-1,3-dione (106)

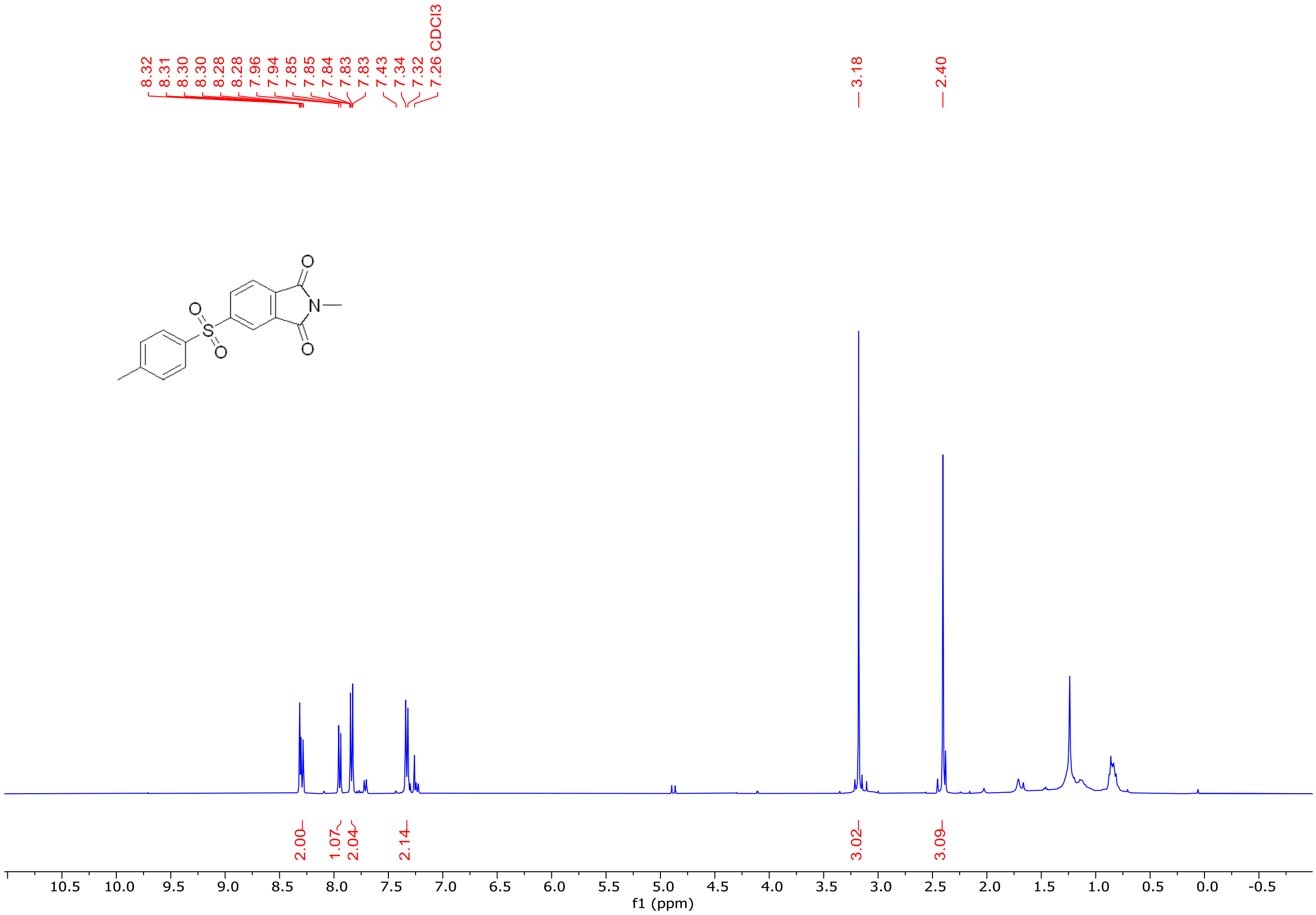

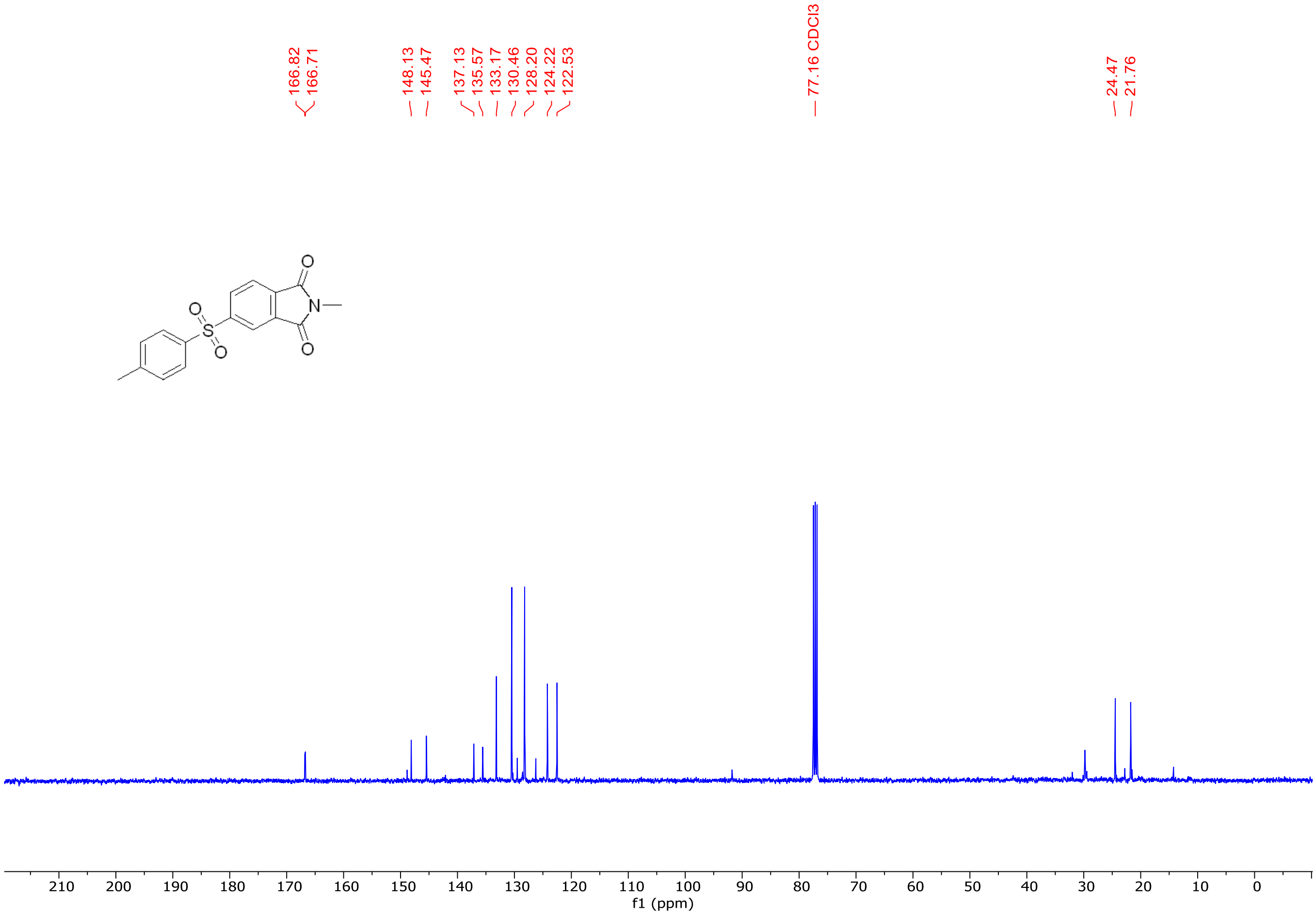

5-tosylpyrimidine (107)

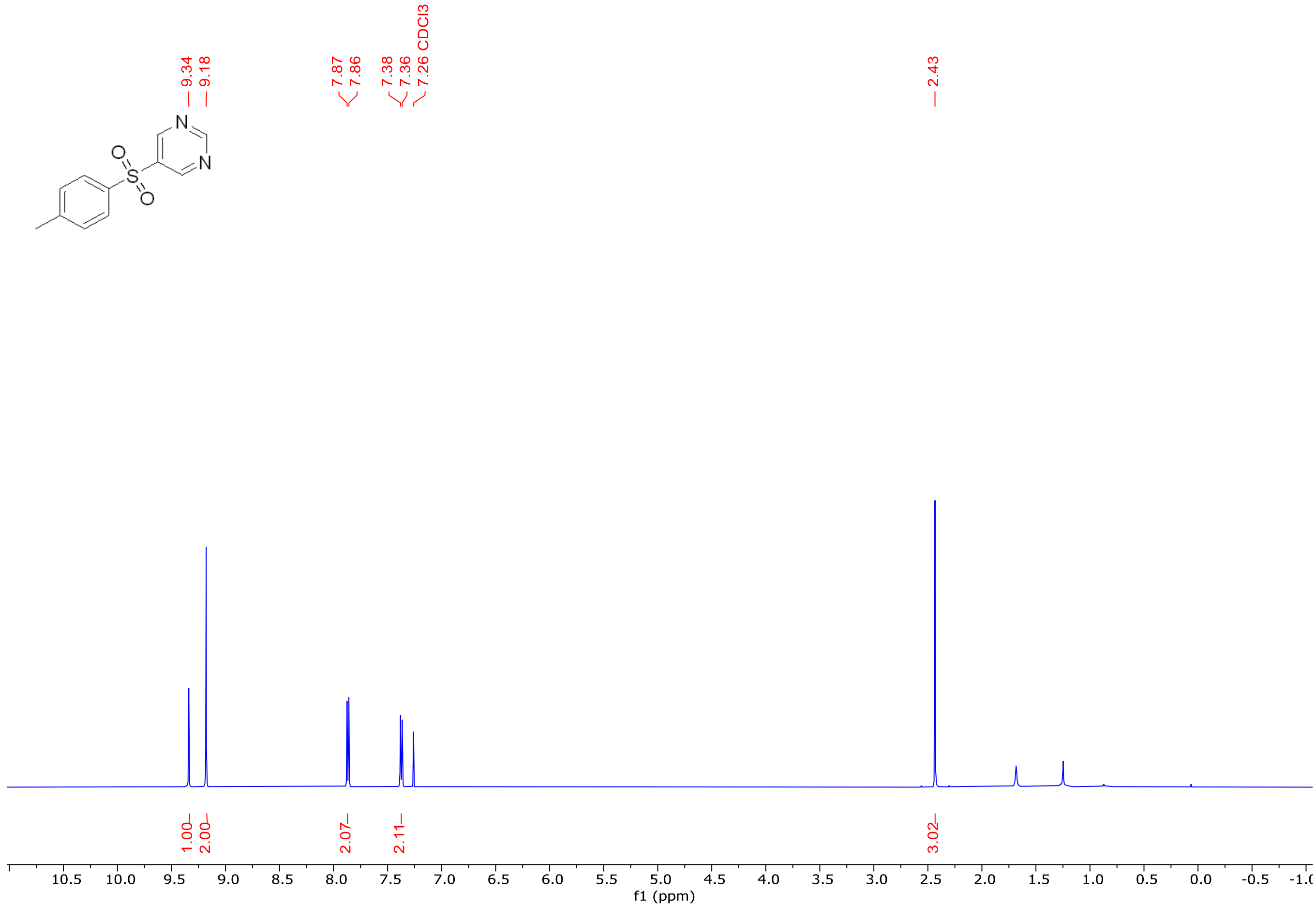

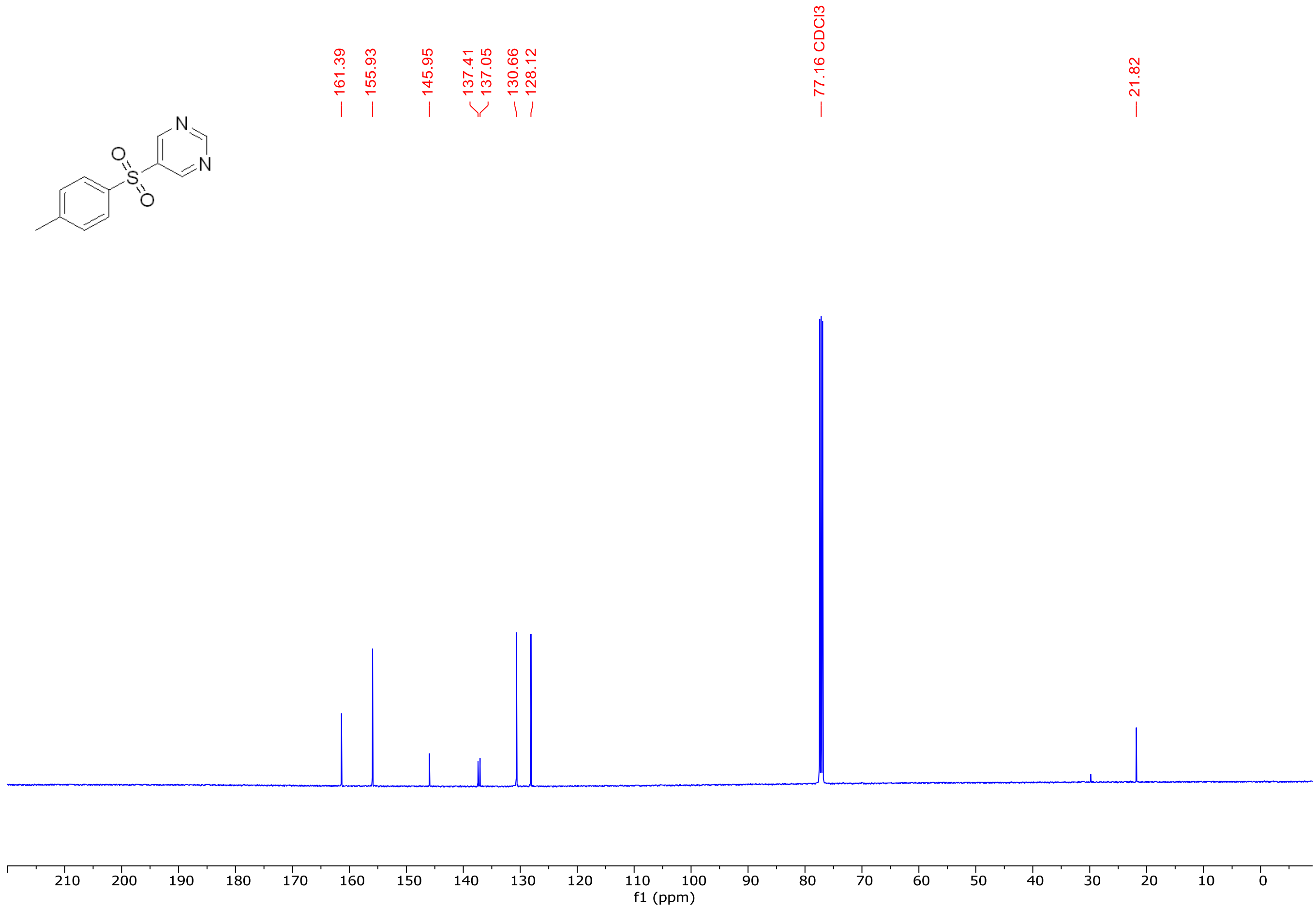

3-tosylquinoline (108)

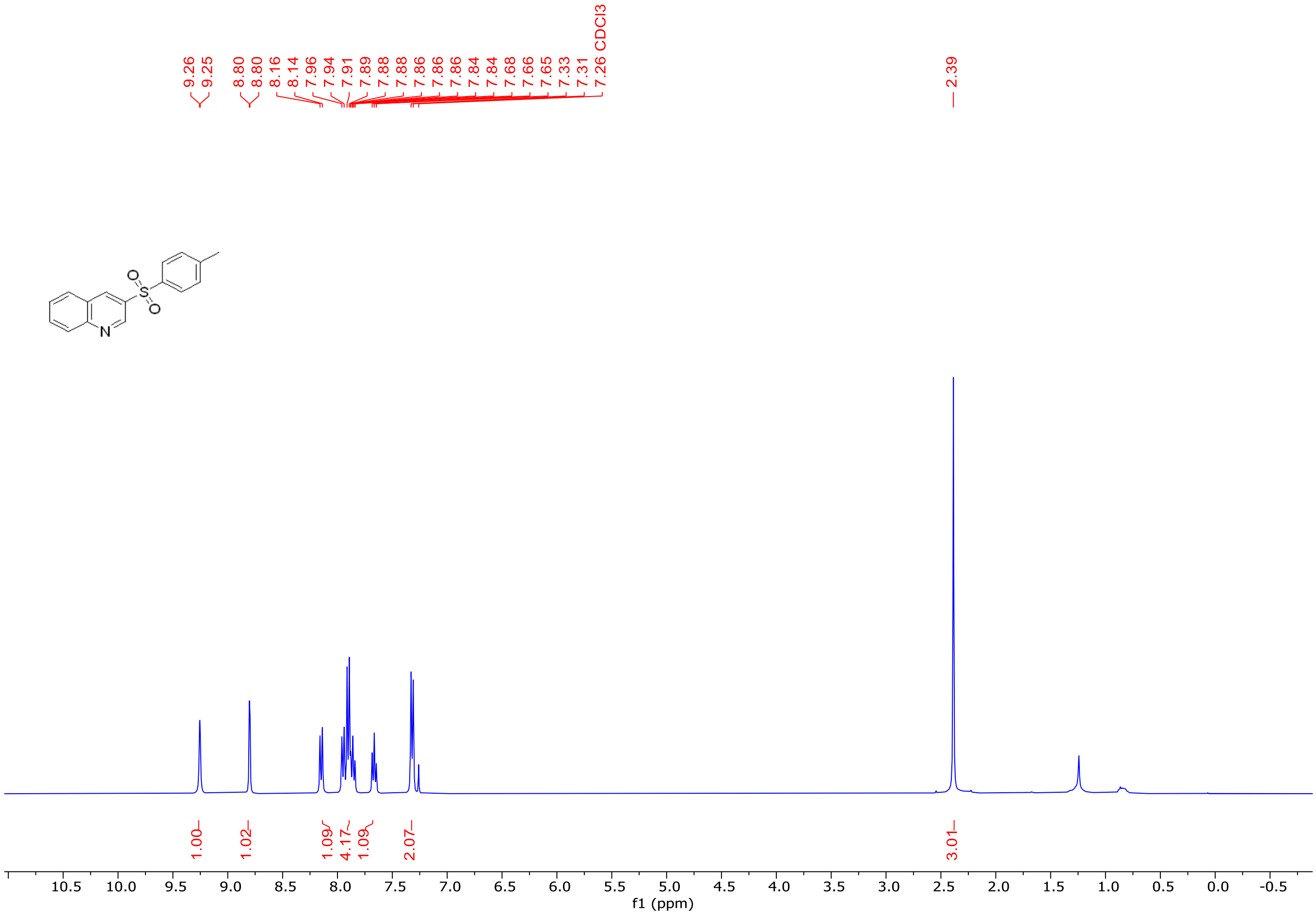

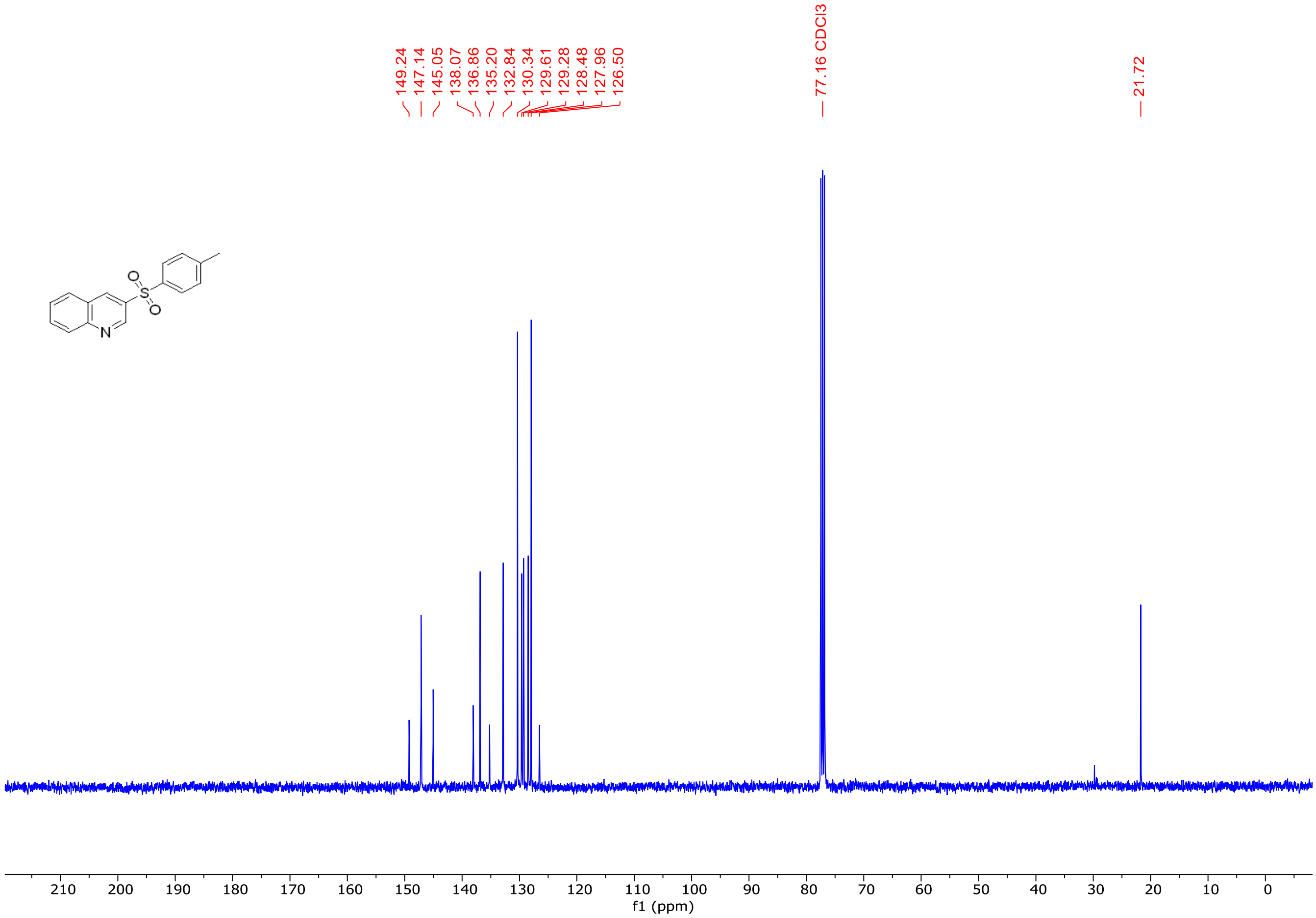

3-tosylthiophene (109)

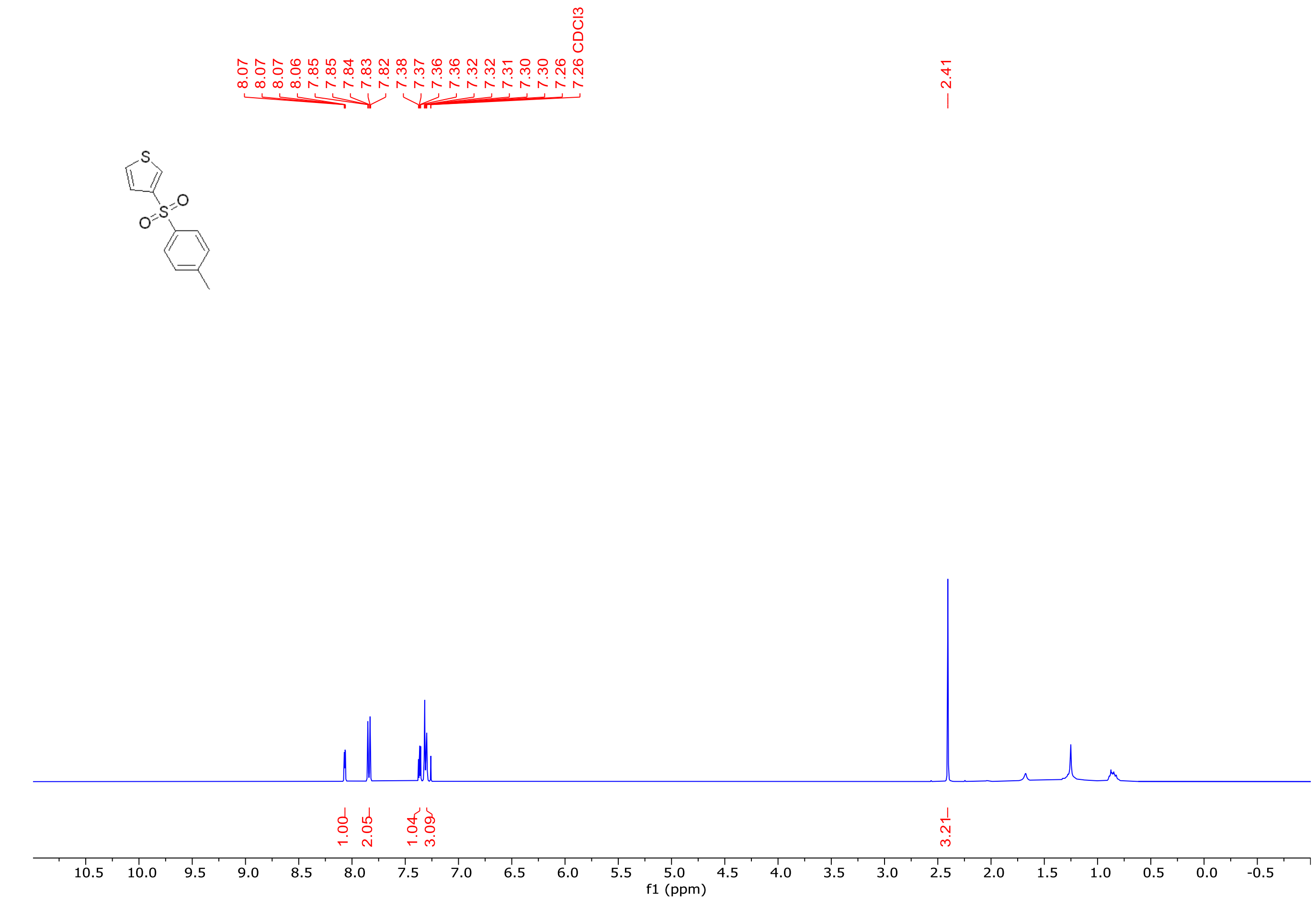

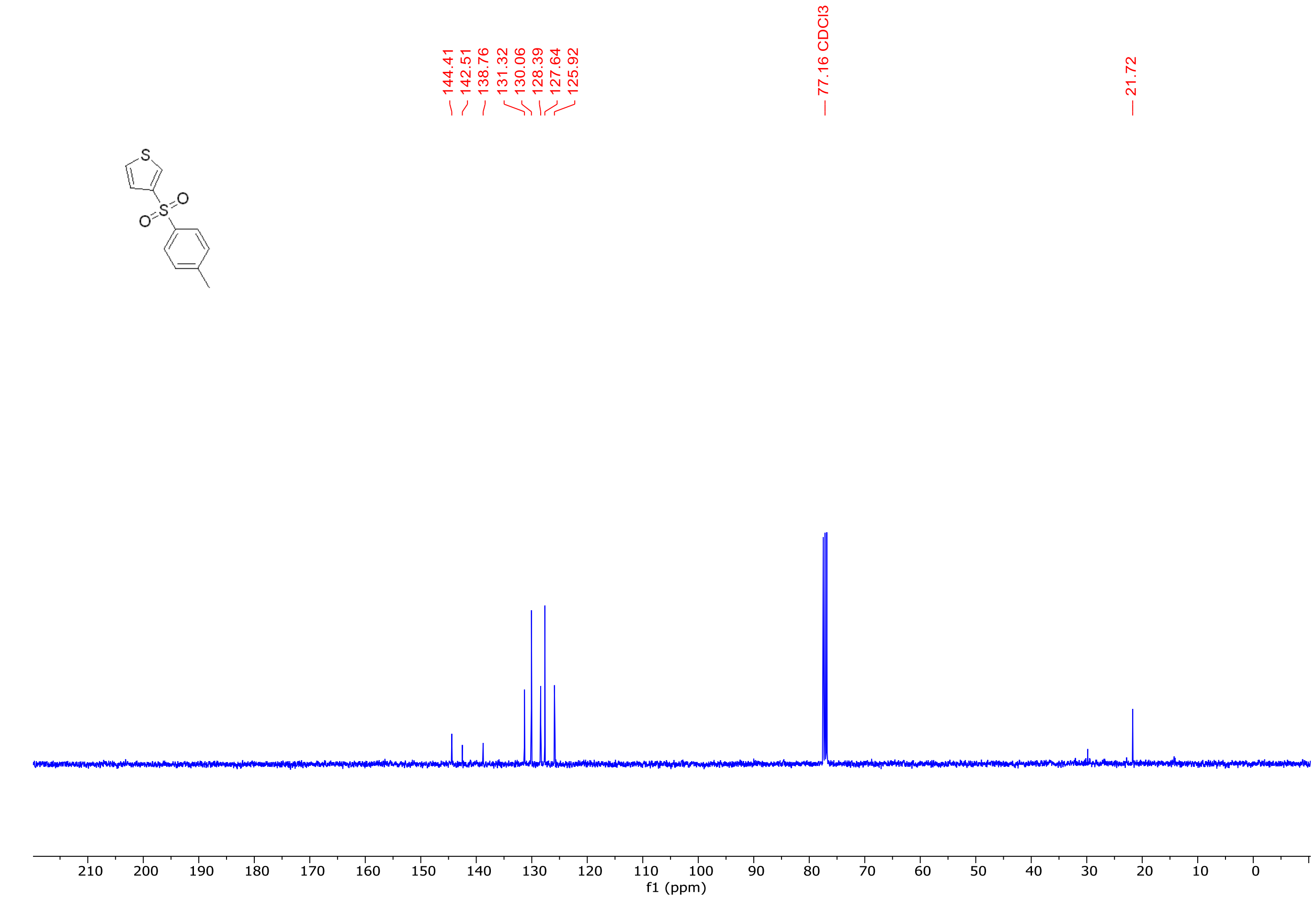

4-((4-fluorophenyl)sulfonyl)-1,1'-biphenyl (110)

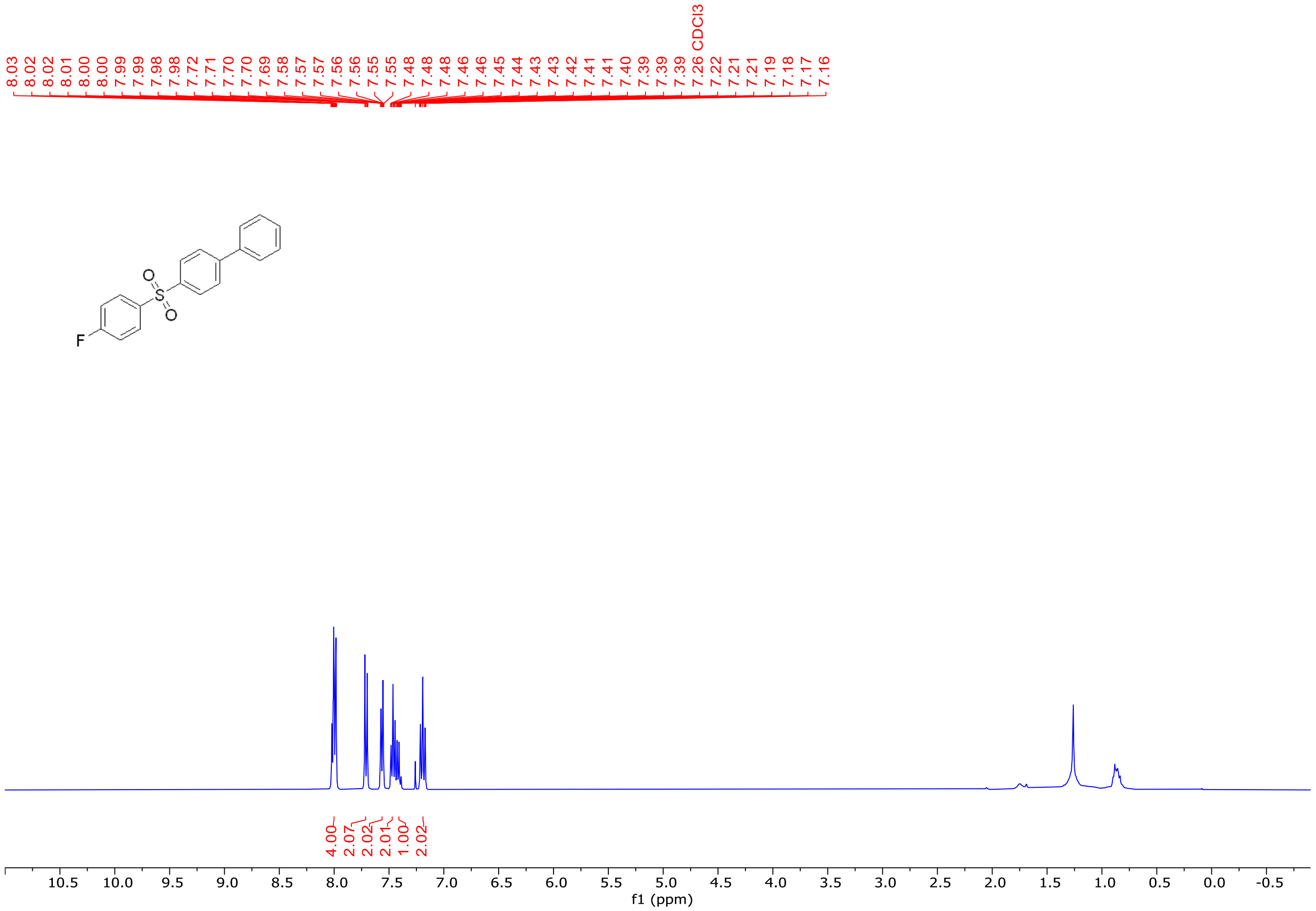

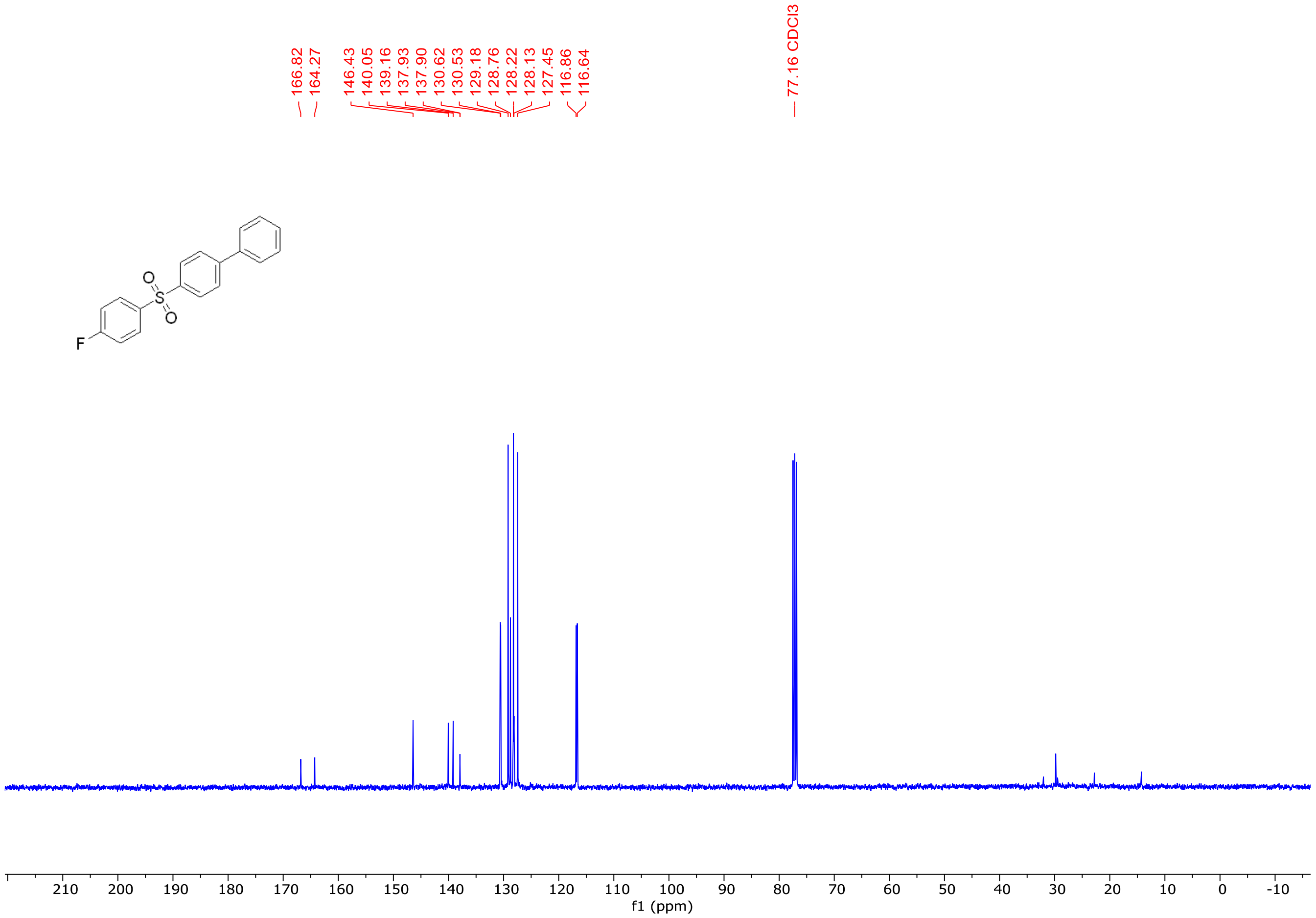

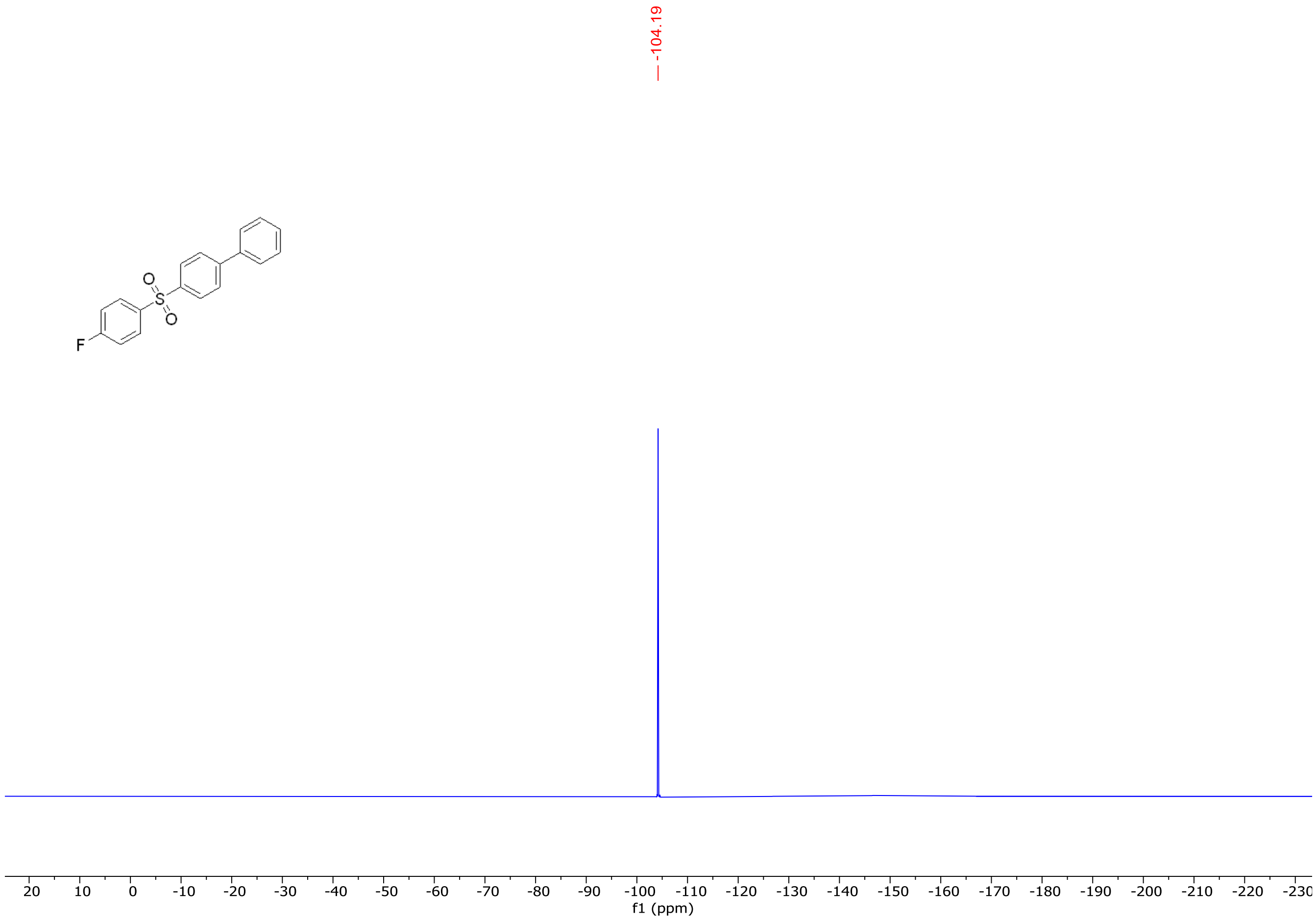
-104.19
F
O
S
O
20
10
0
-10
-20
-30
-40
-50
-60
-70
-80
-90
-100
-110
-120
-130
-140
-150
-160
-170
-180
-190
-200
-210
-220
-230
f1 (ppm)

4-((4-methoxyphenyl)sulfonyl)-1,1'-biphenyl (111)

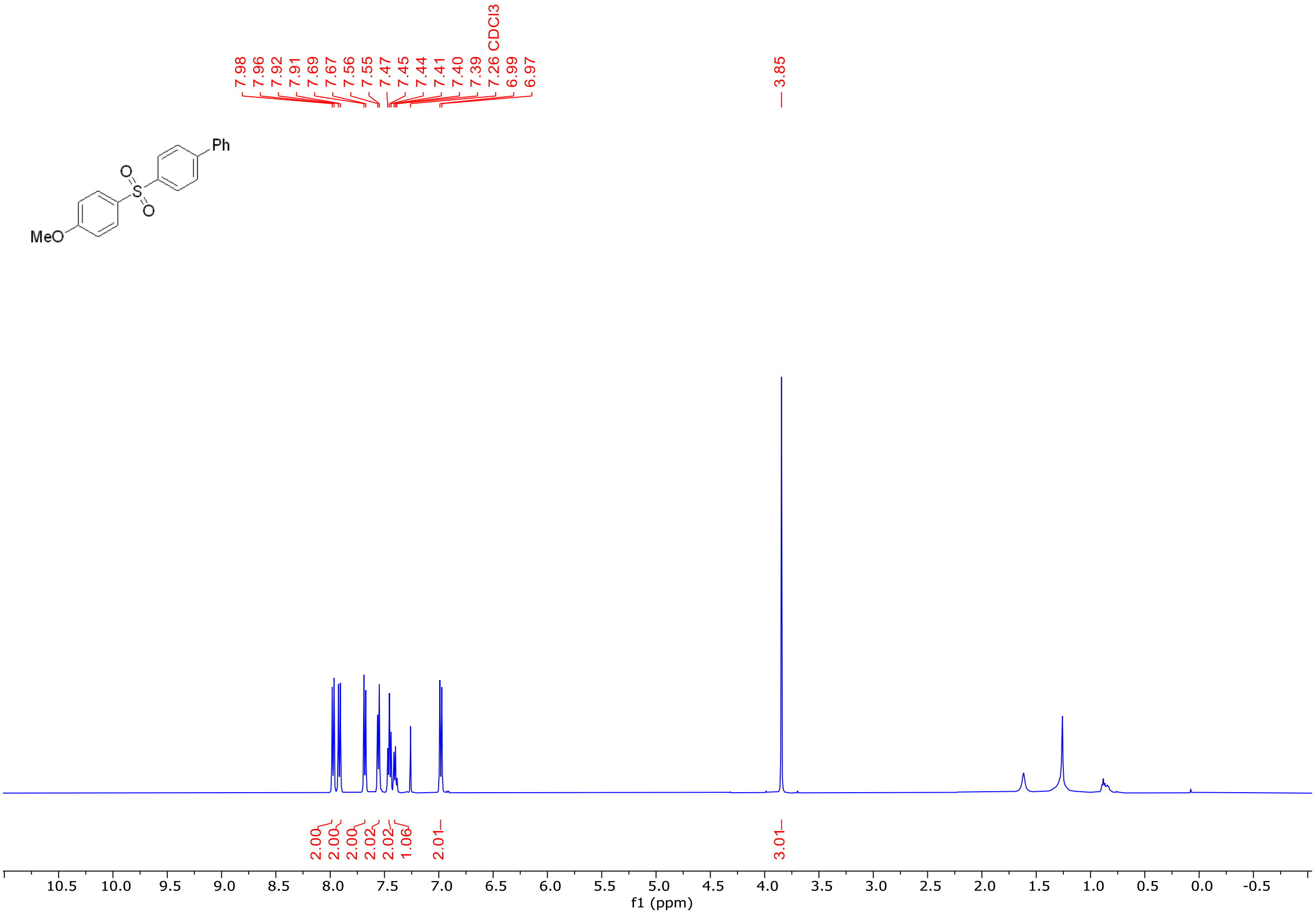

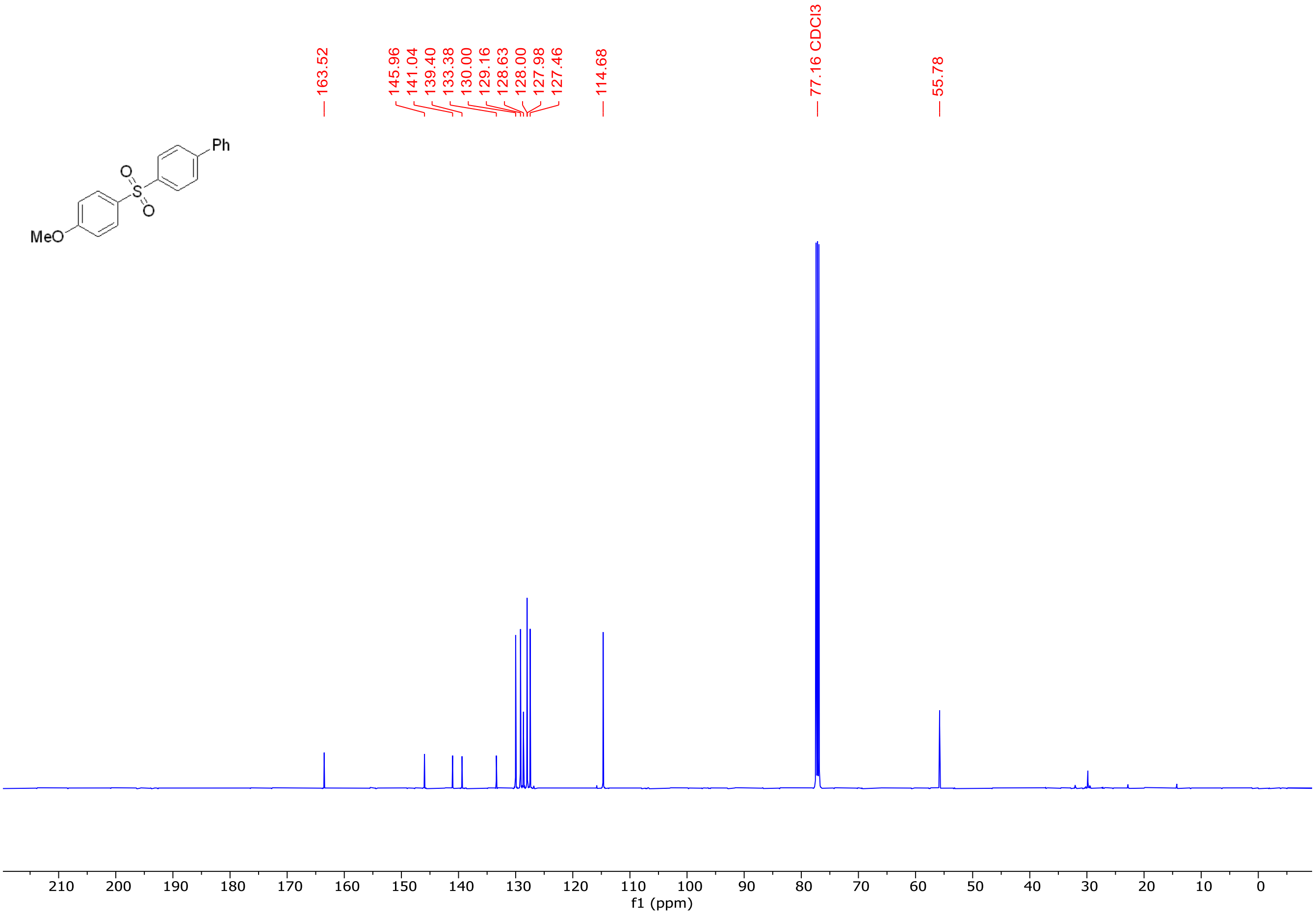

4-((4-(trifluoromethyl)phenyl)sulfonyl)-1,1'-bipheny (112)

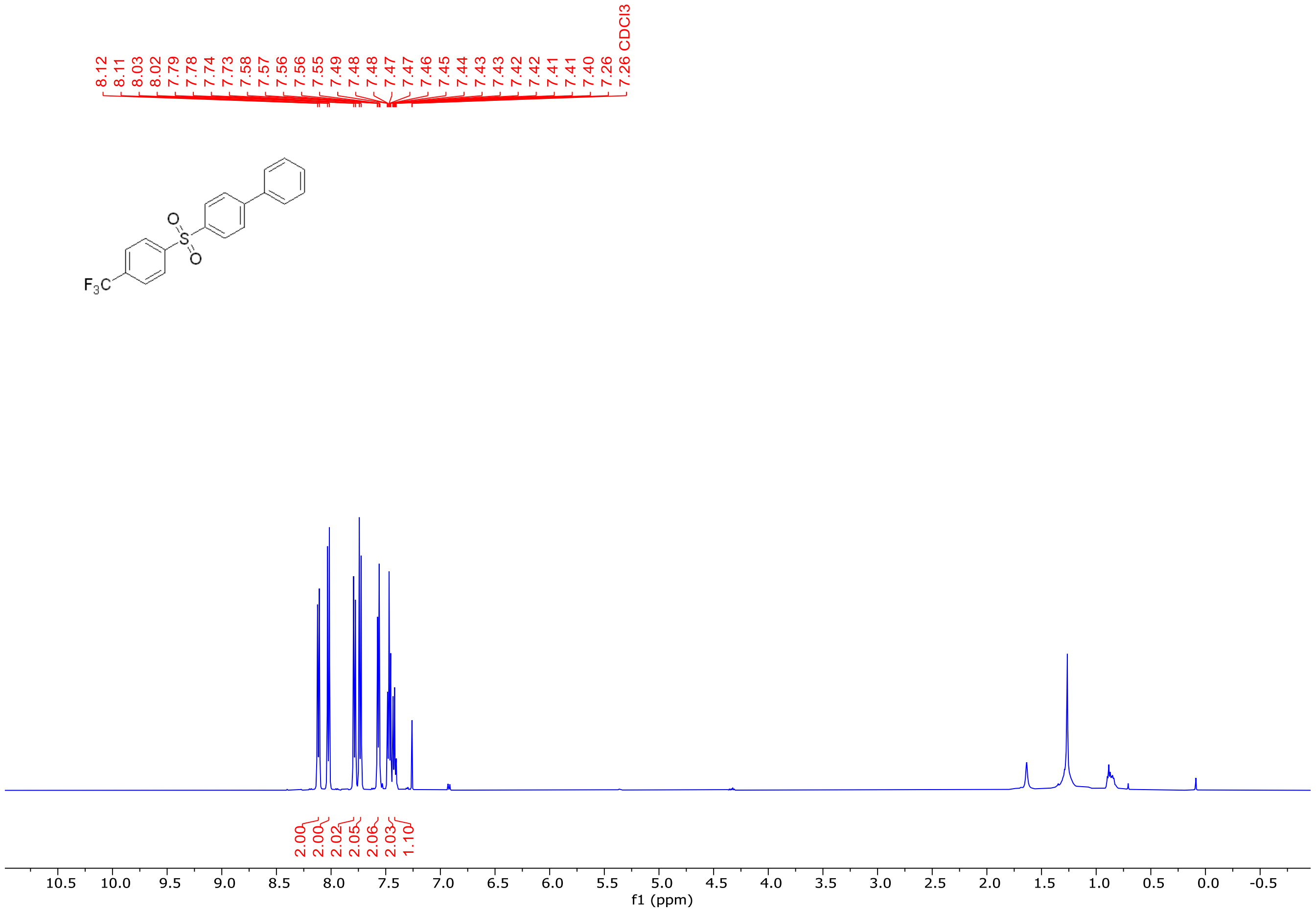

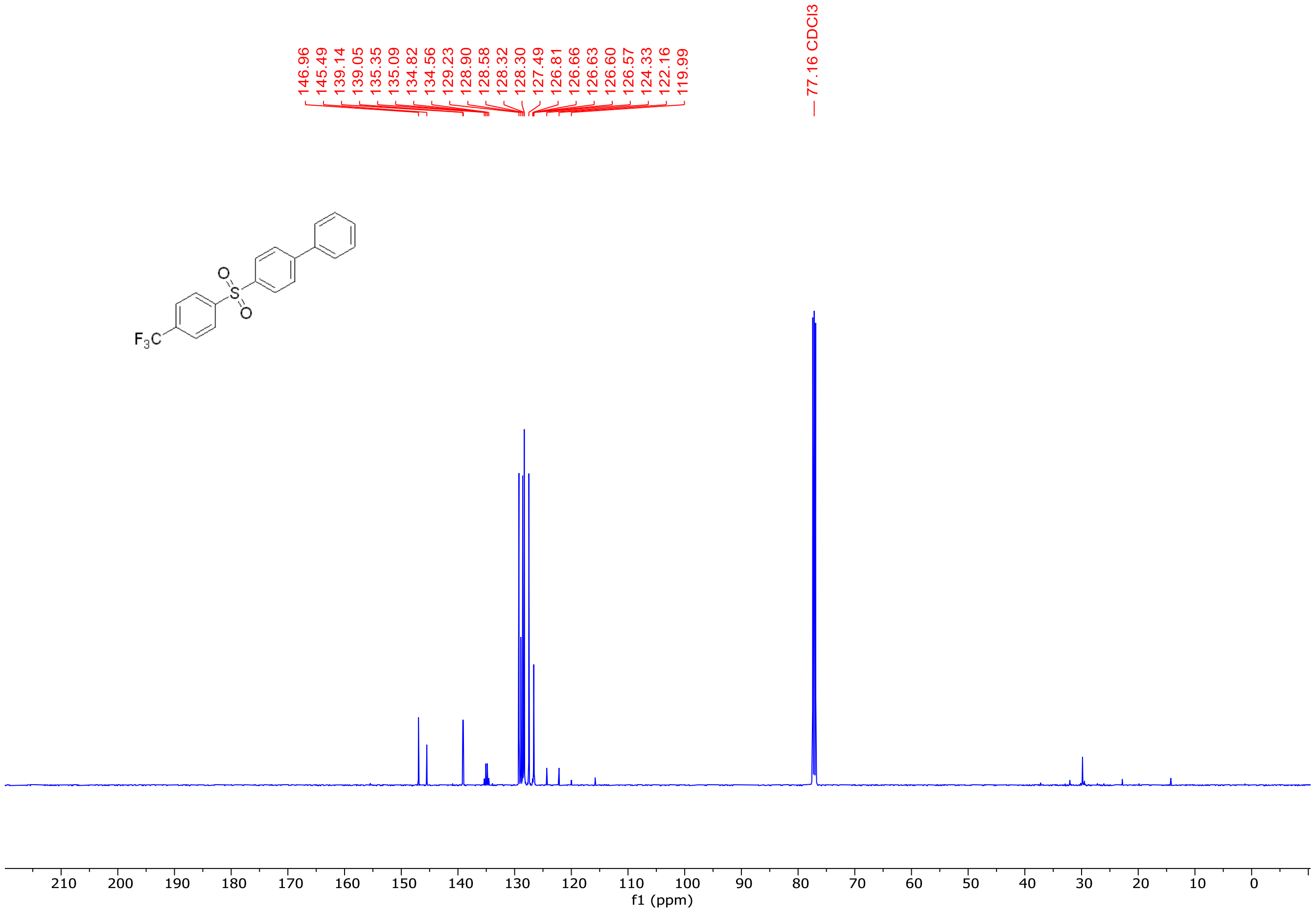

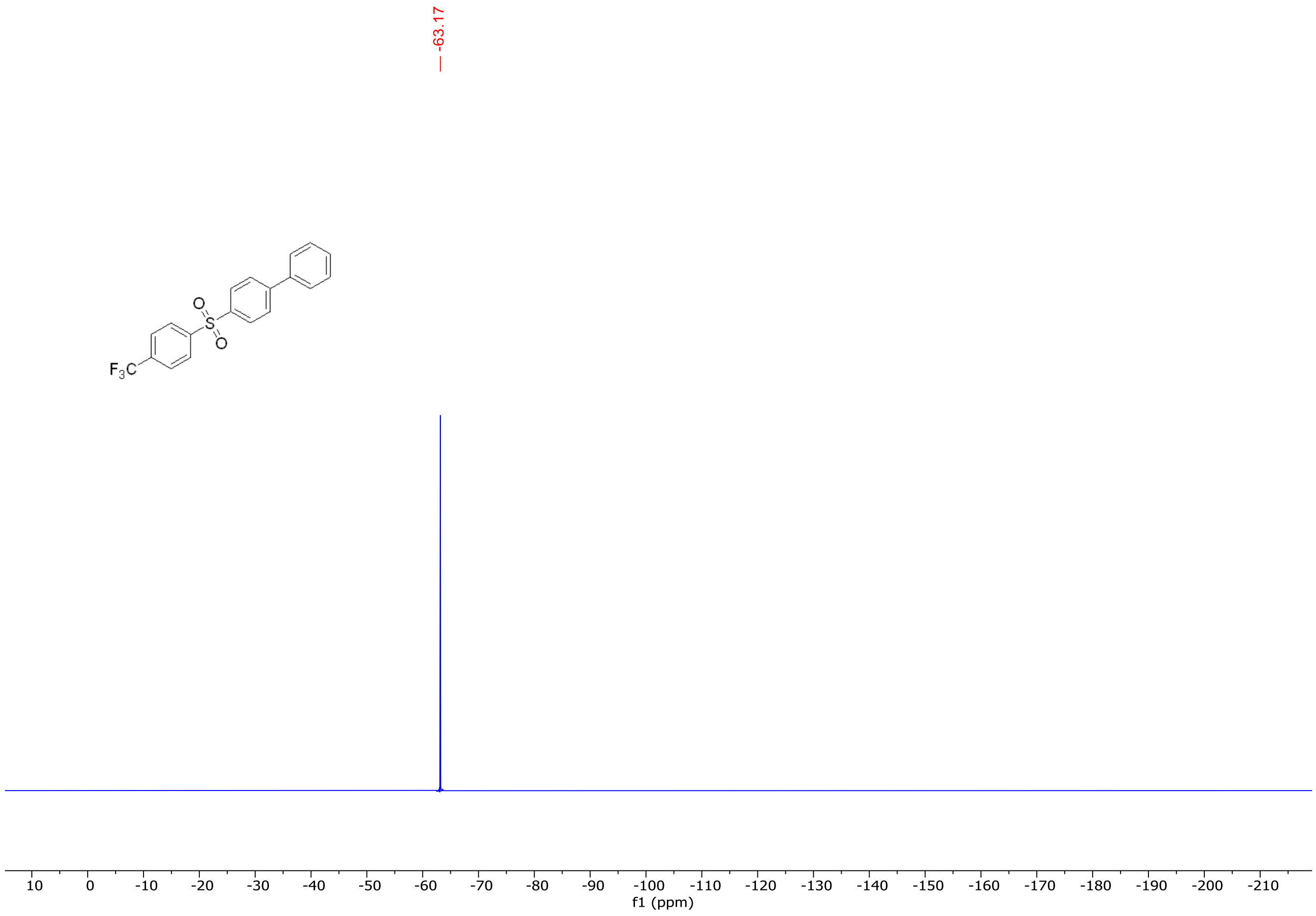

-63.17
F3C
O
S
O
10
0
-10
-20
-30
-40
-50
-60
-70
-80
-90
-100
-110
-120
-130
-140
-150
-160
-170
-180
-190
-200
-210
f1 (ppm)

N-(4-([1,1'-biphenyl]-4-ylsulfonyl)phenyl)acetamide (113)

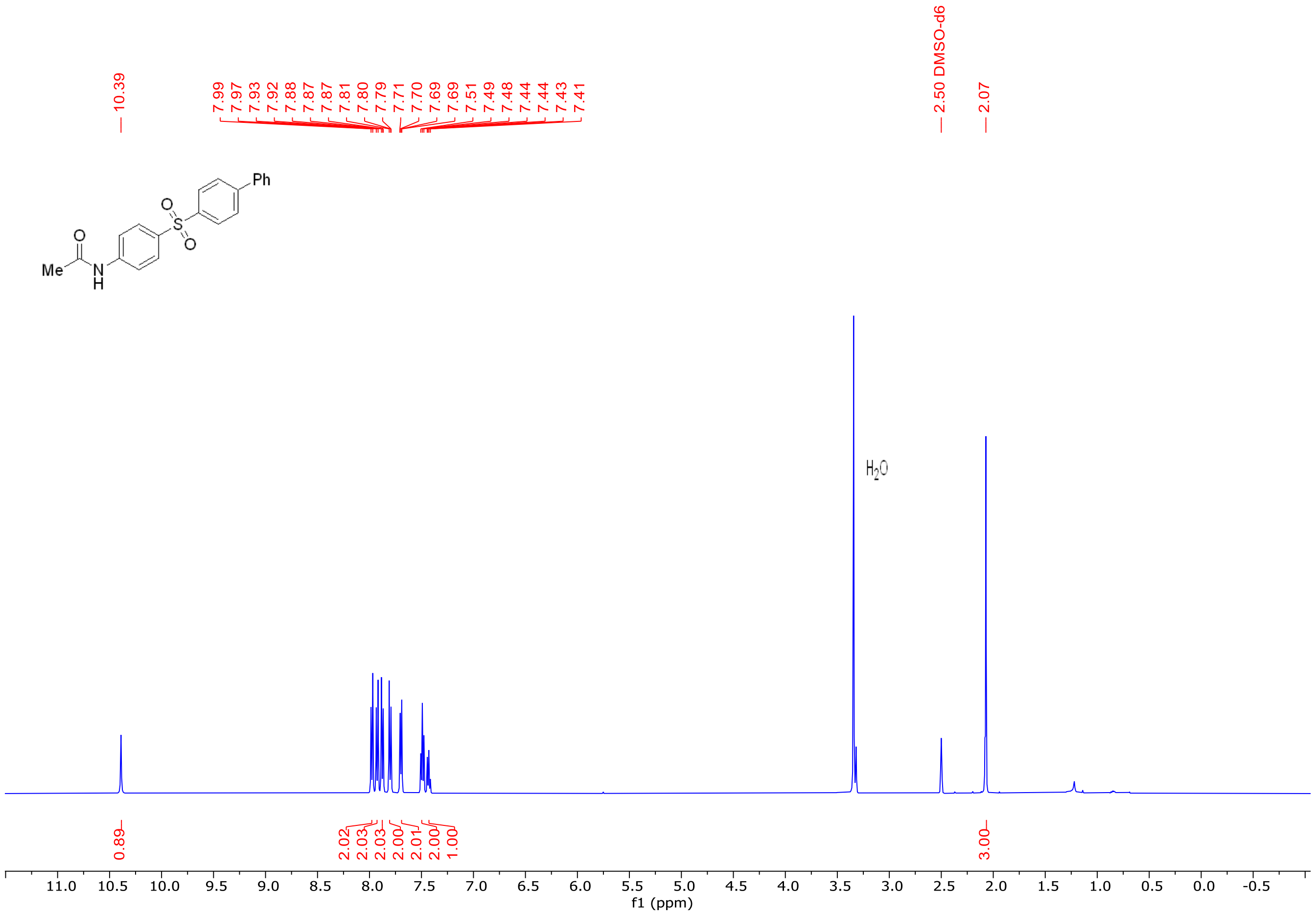

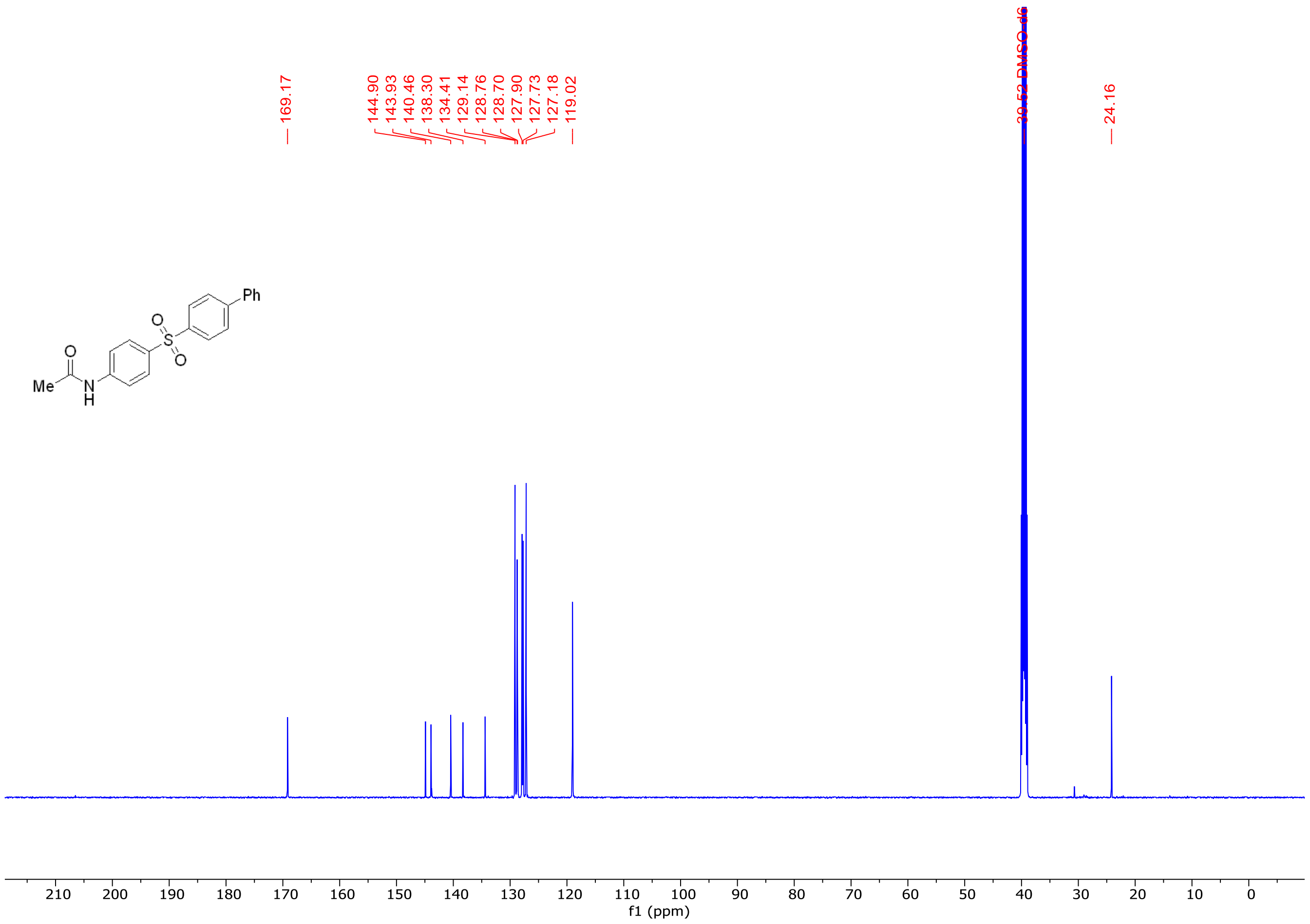

3-([1,1'-biphenyl]-4-ylsulfonyl)pyridine (114)

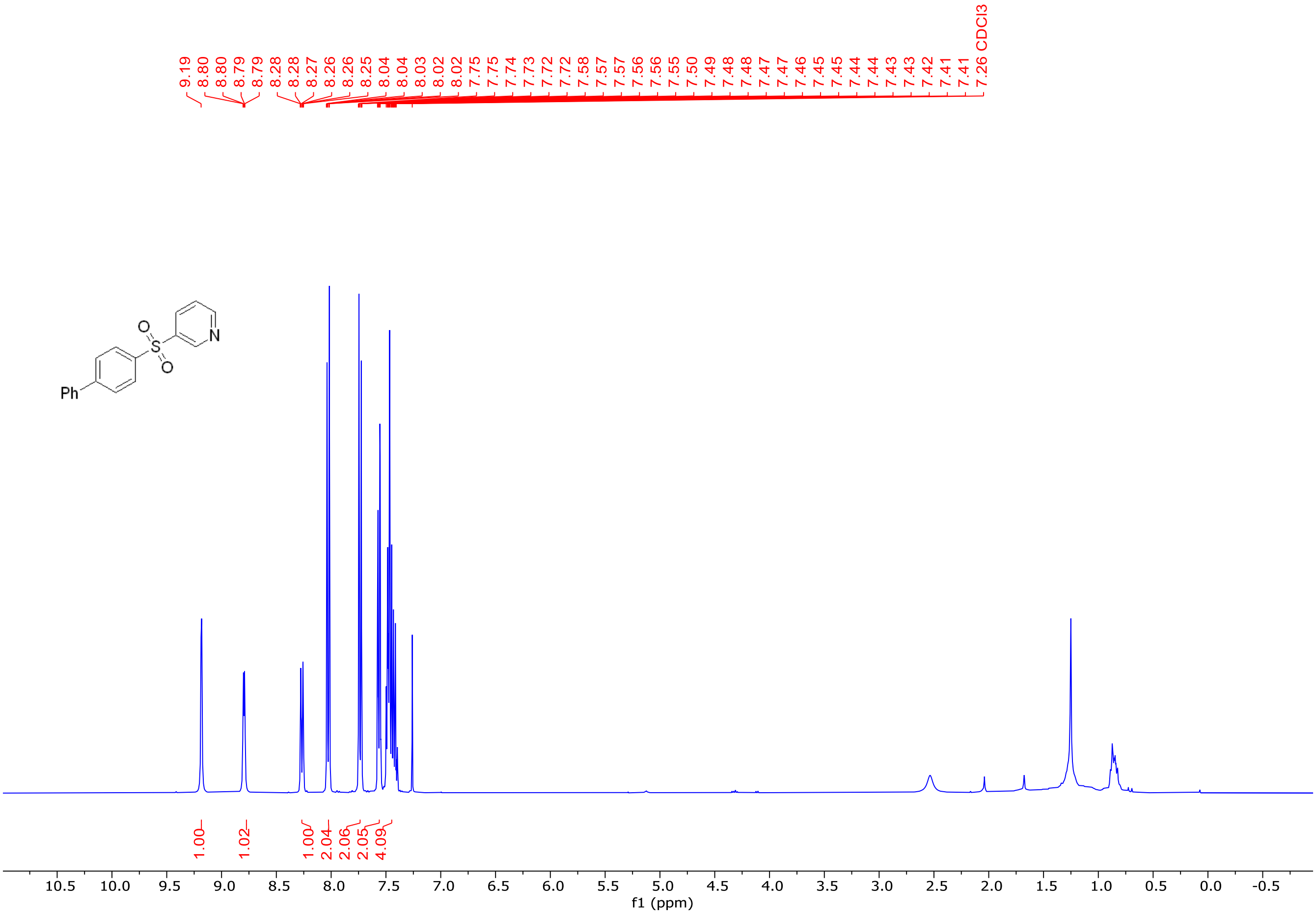

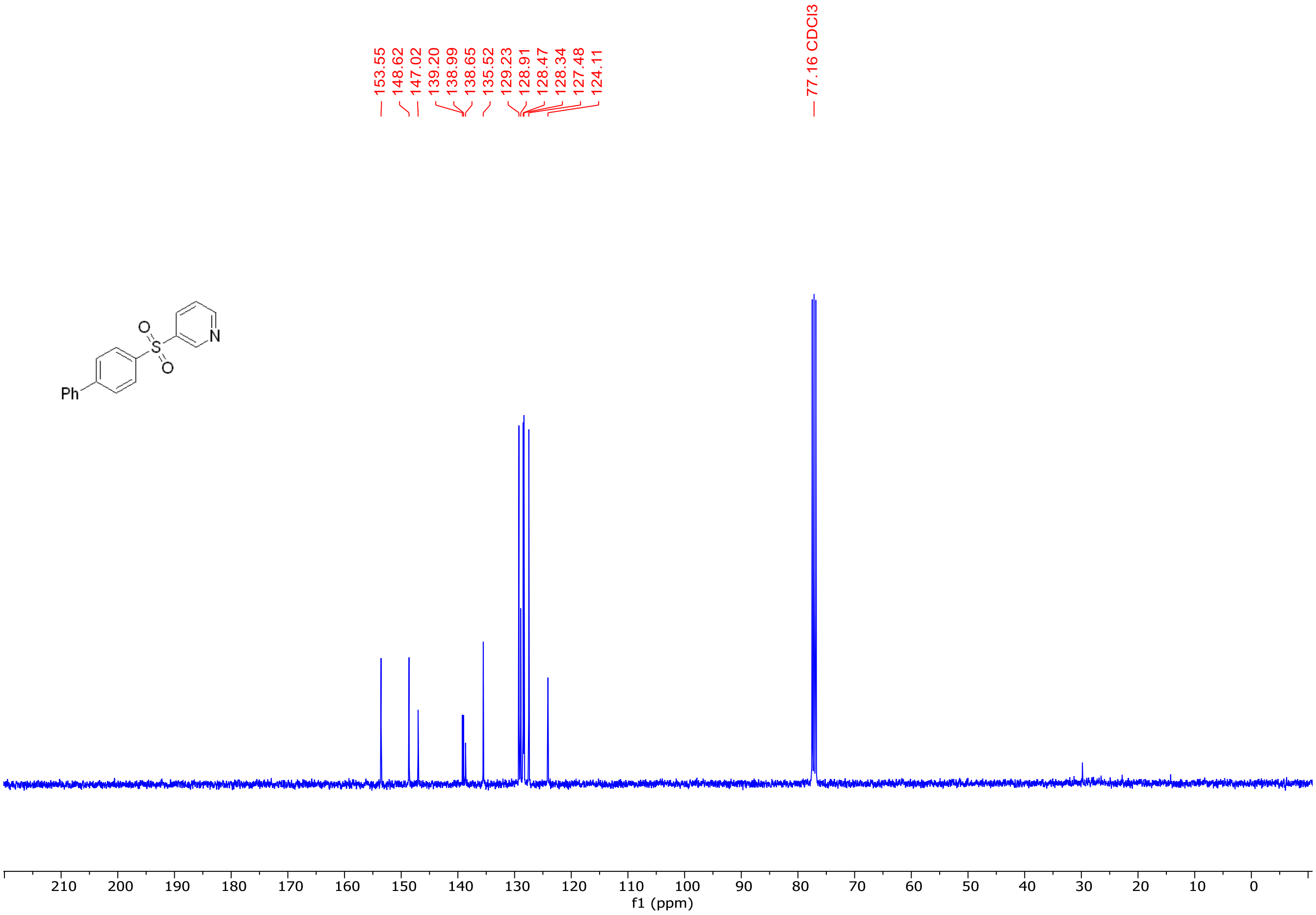

4-(cyclopropylsulfonyl)-1,1'-biphenyl (115)

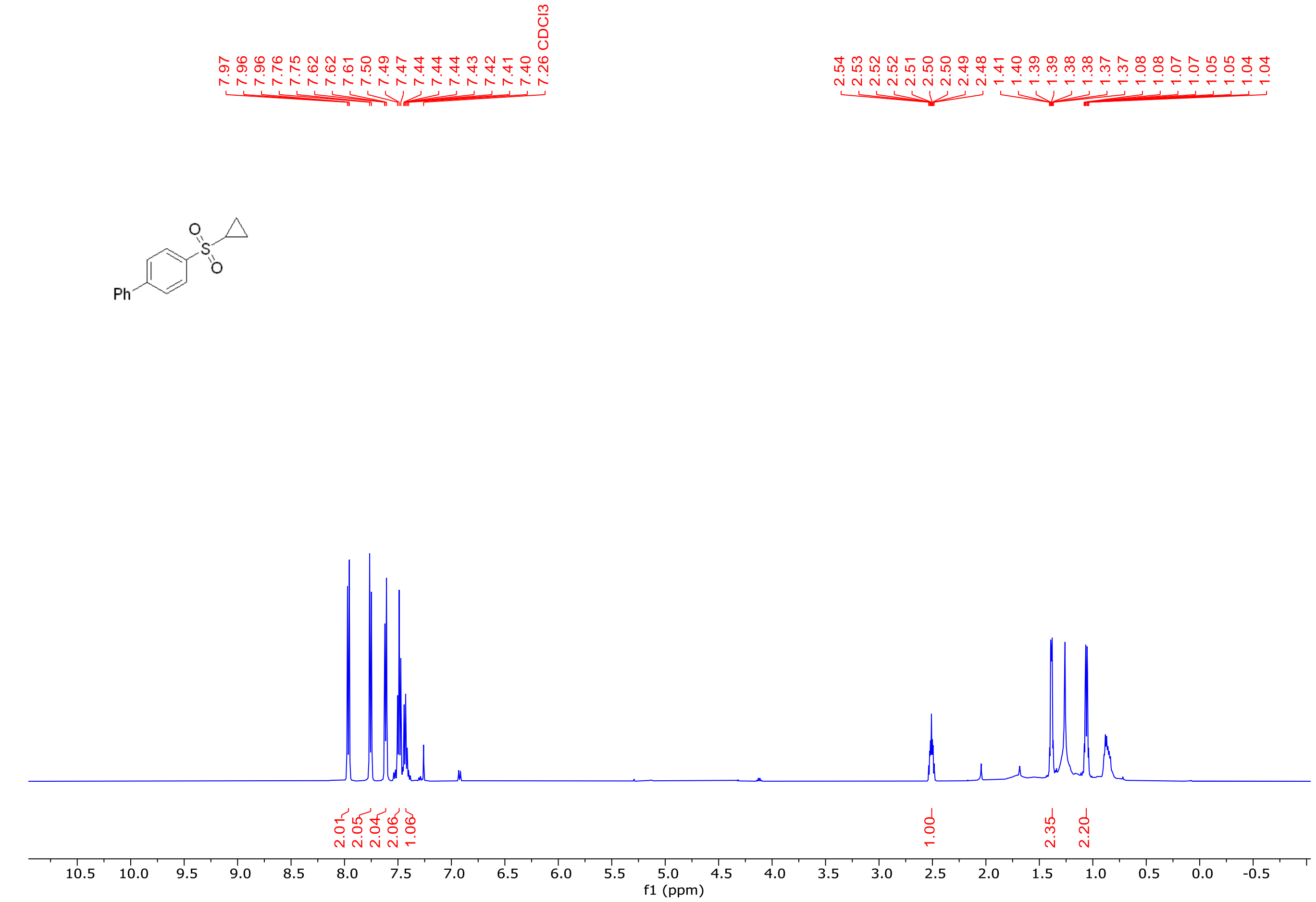

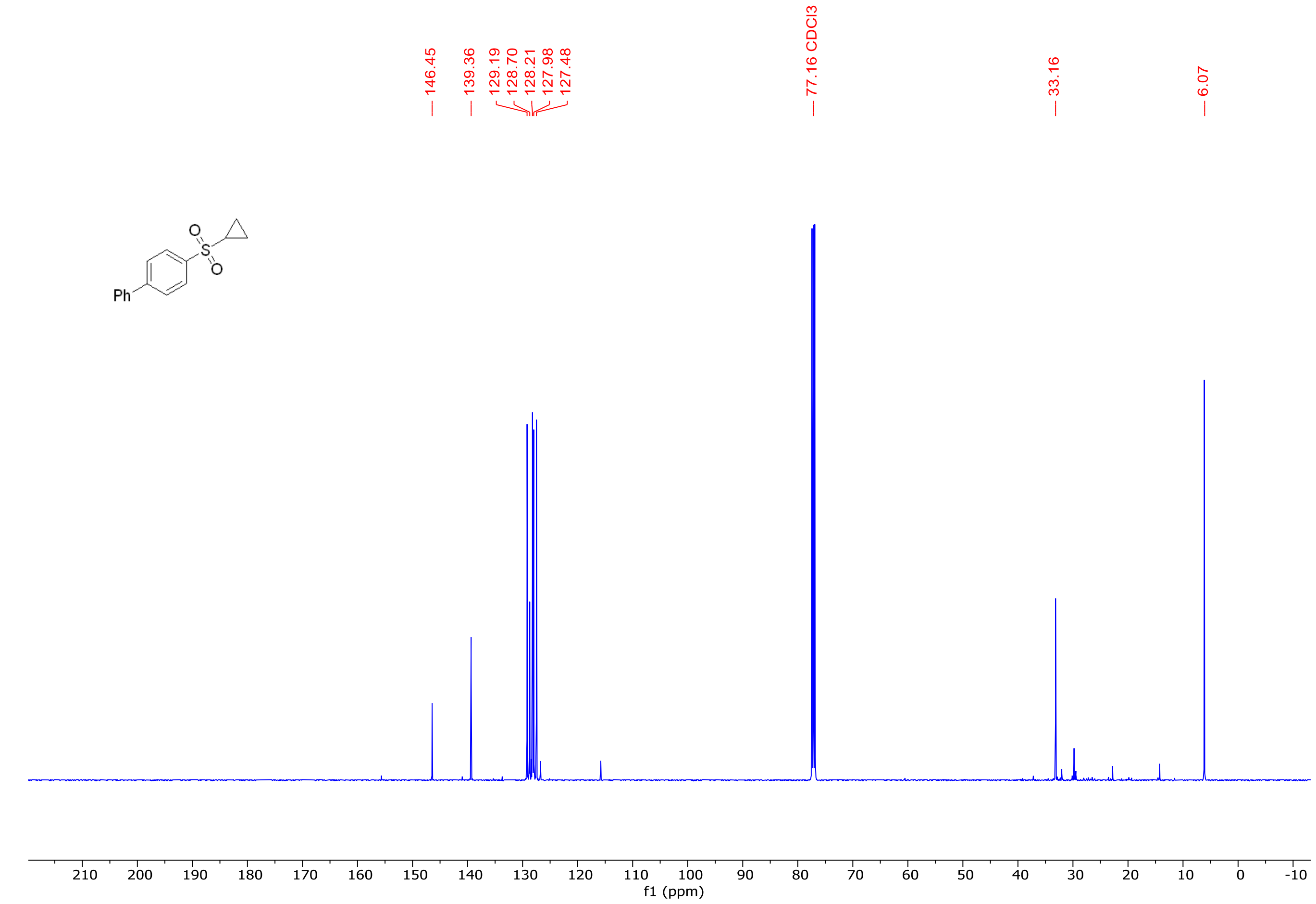

1-(4-(methylsulfonyl)phenyl)ethan-1-one (116)

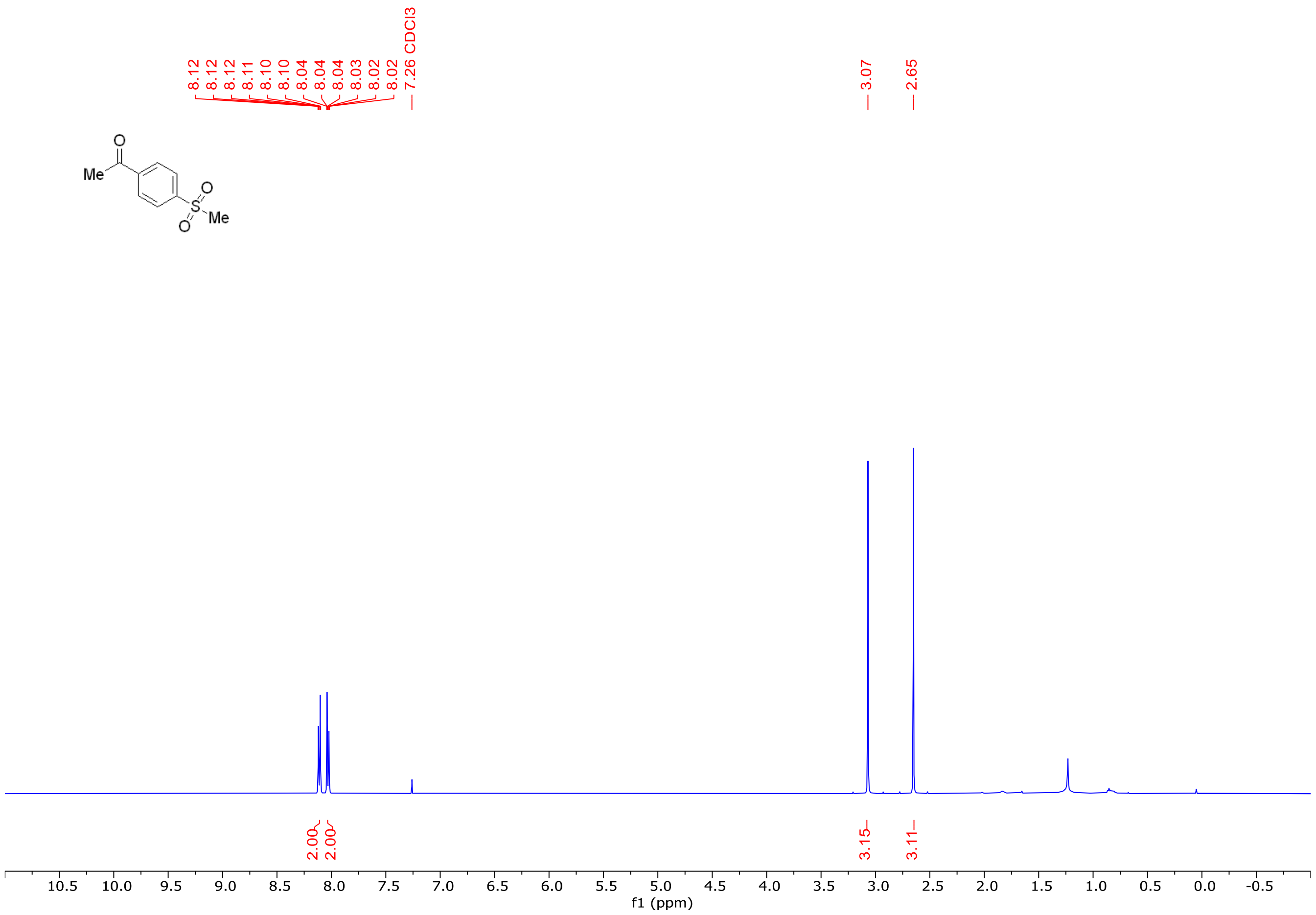

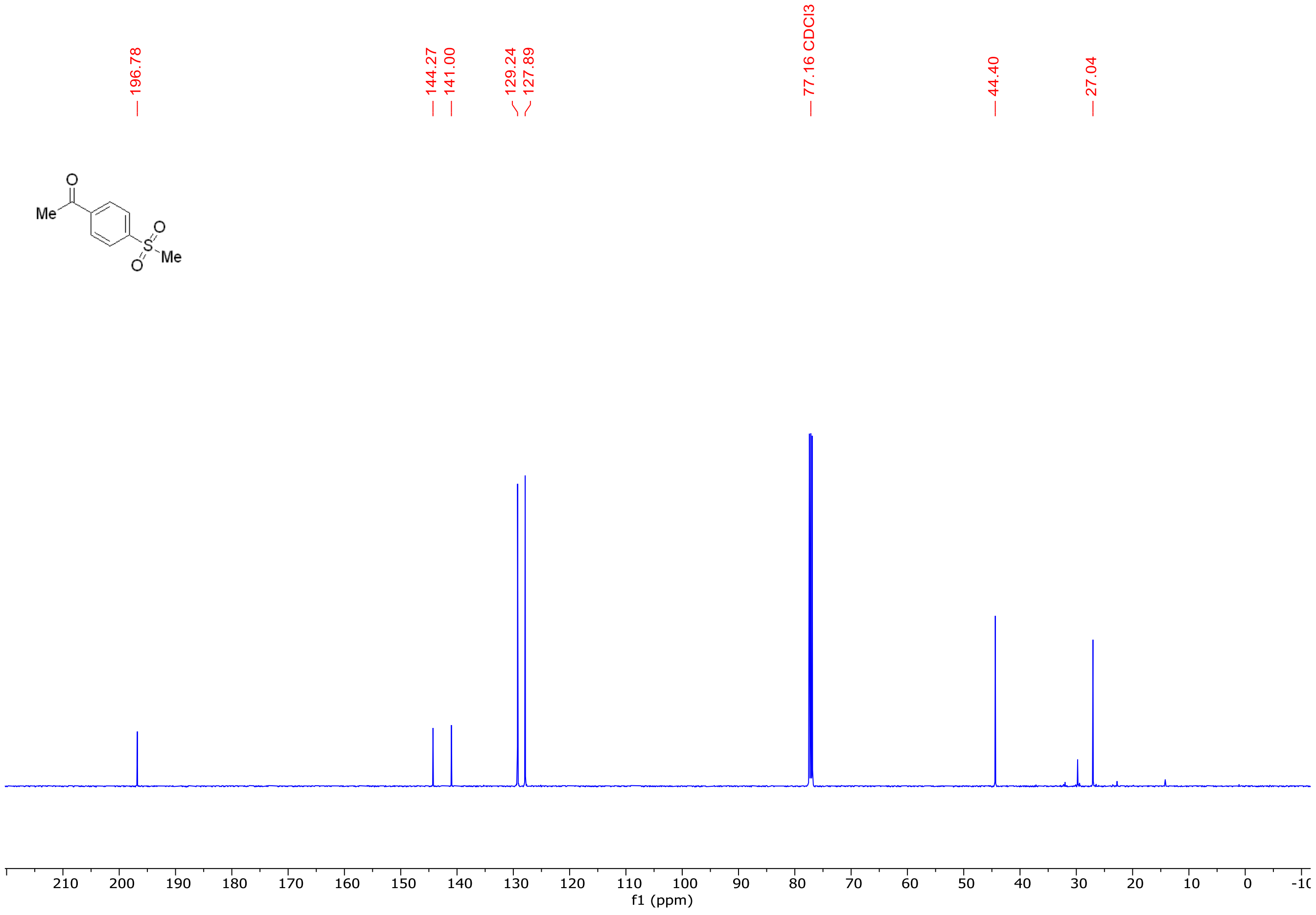

[1,1'-biphenyl]-4-yldiphenylphosphine oxide (117)

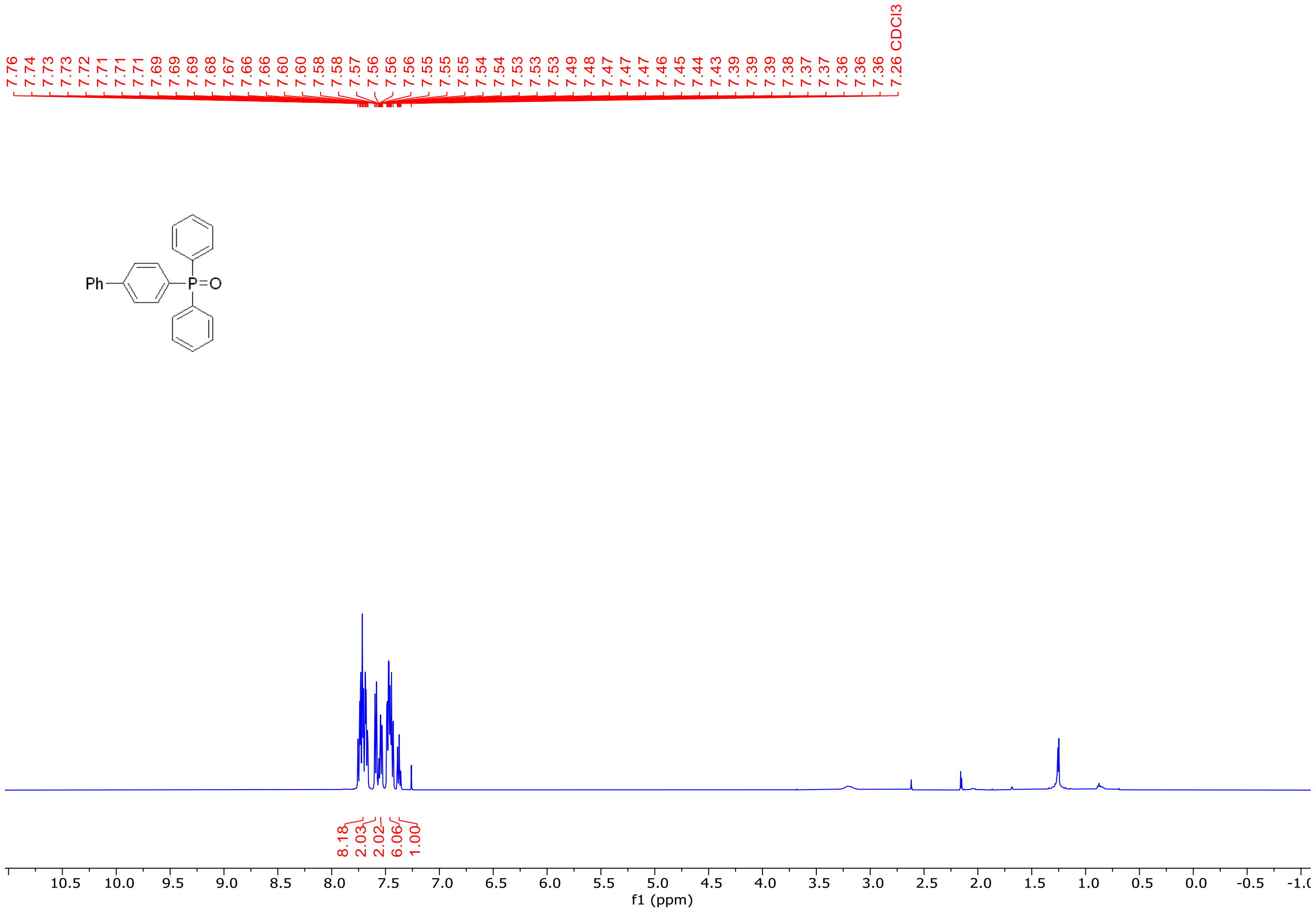

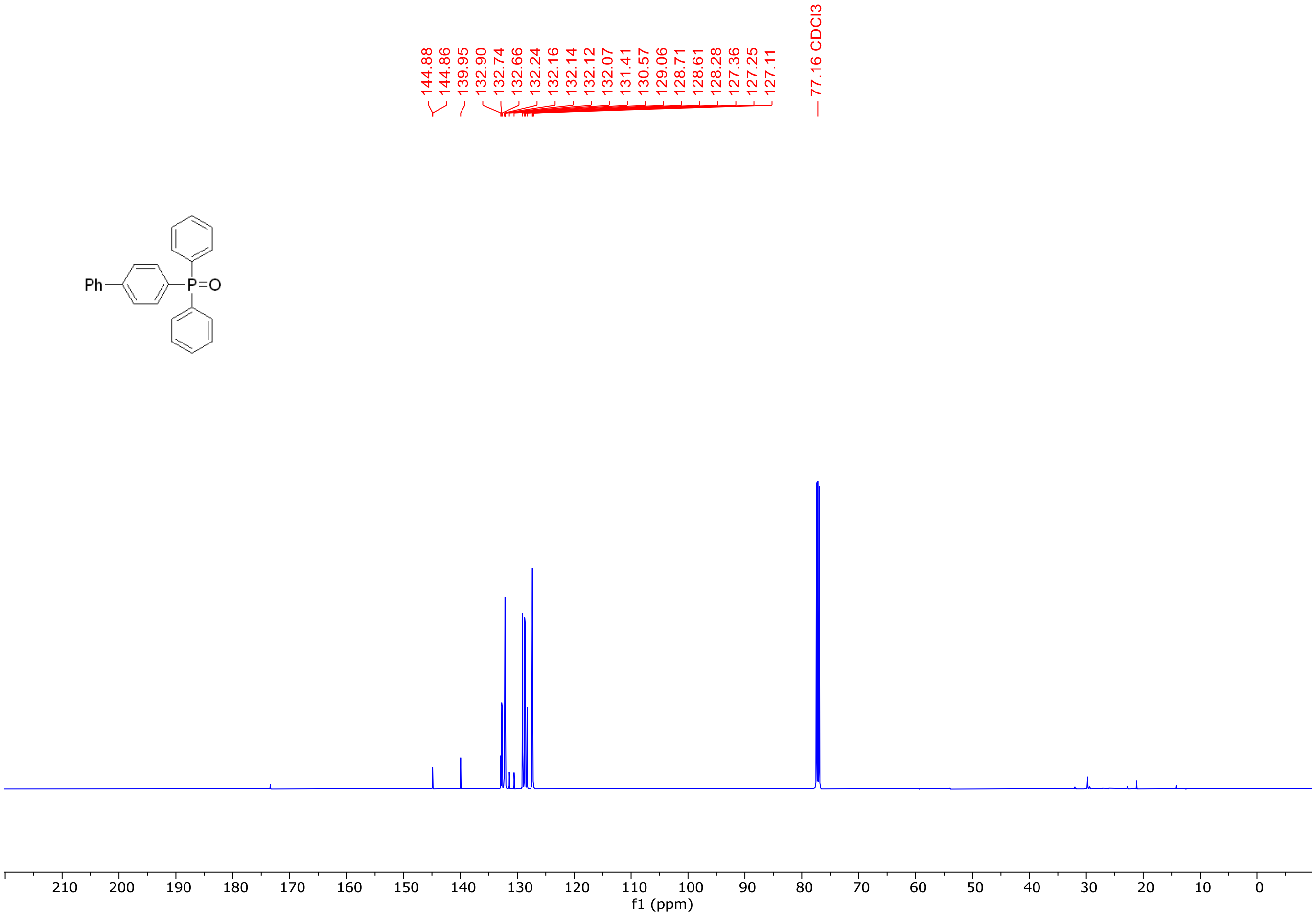

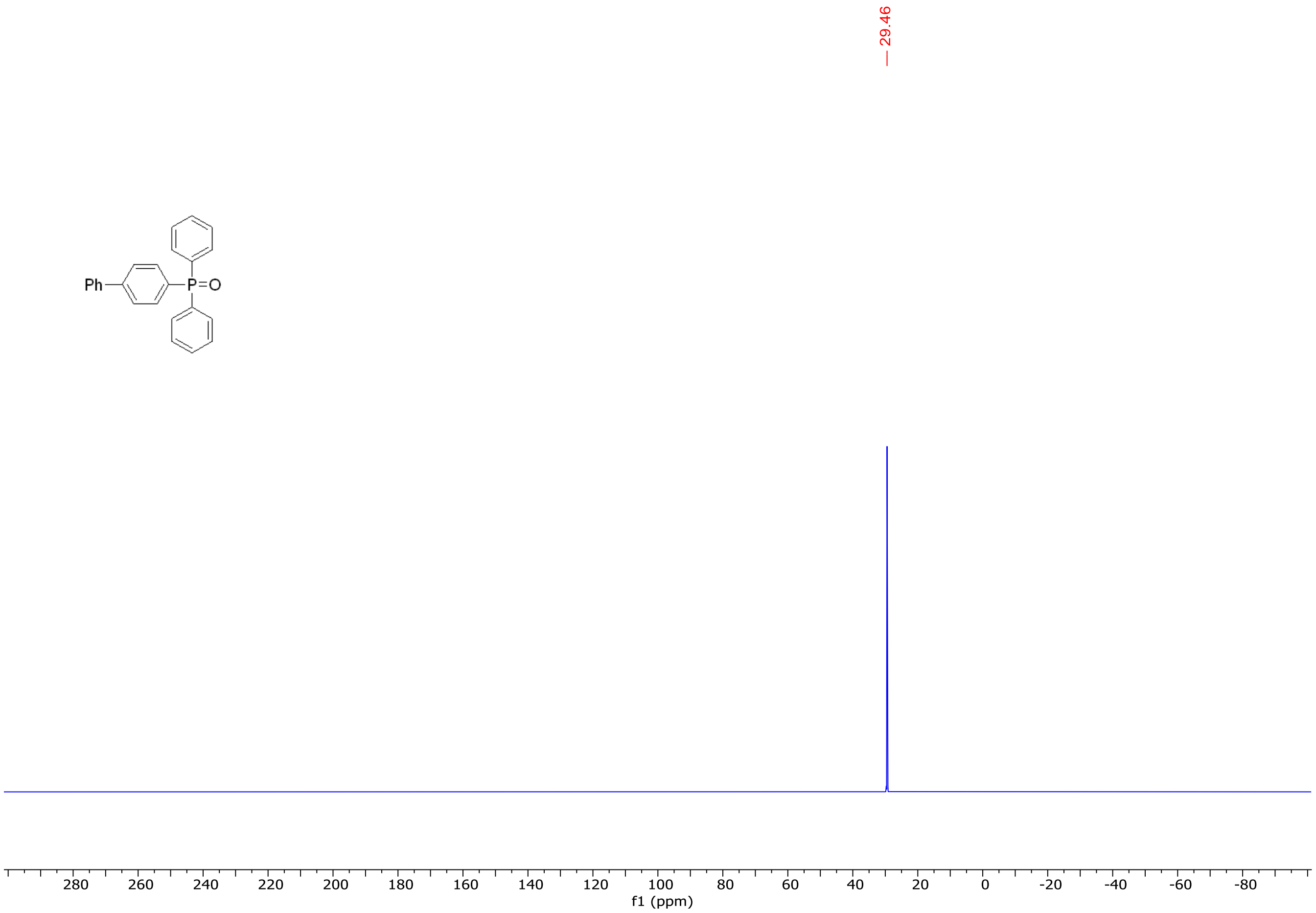
Ph
P=O
29.46
280
260
240
220
200
180
160
140
120
100
80
60
40
20
0
-20
-40
-60
-80
f1 (ppm)

diphenyl(4-(trifluoromethyl)phenyl)phosphine oxide (118)

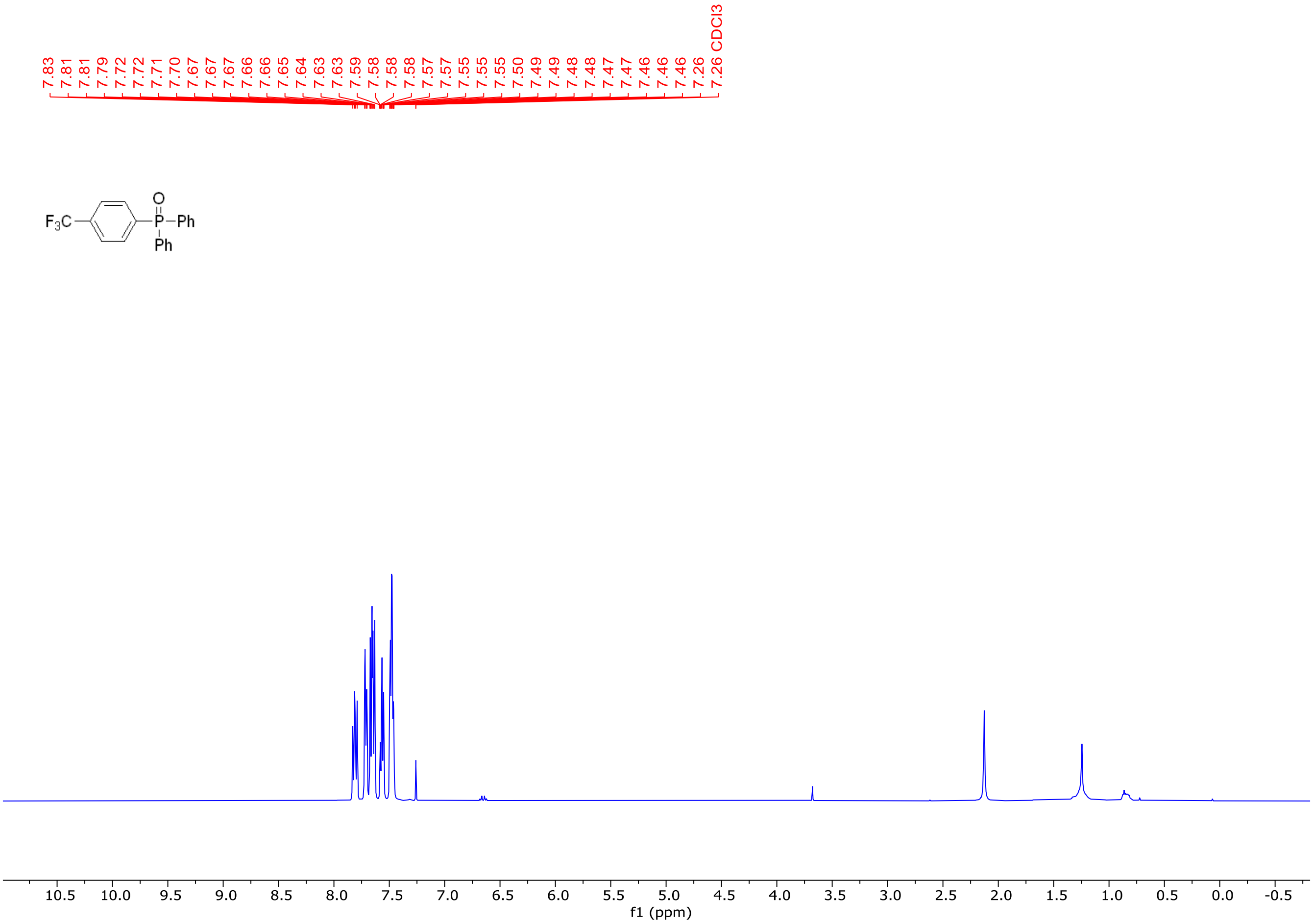

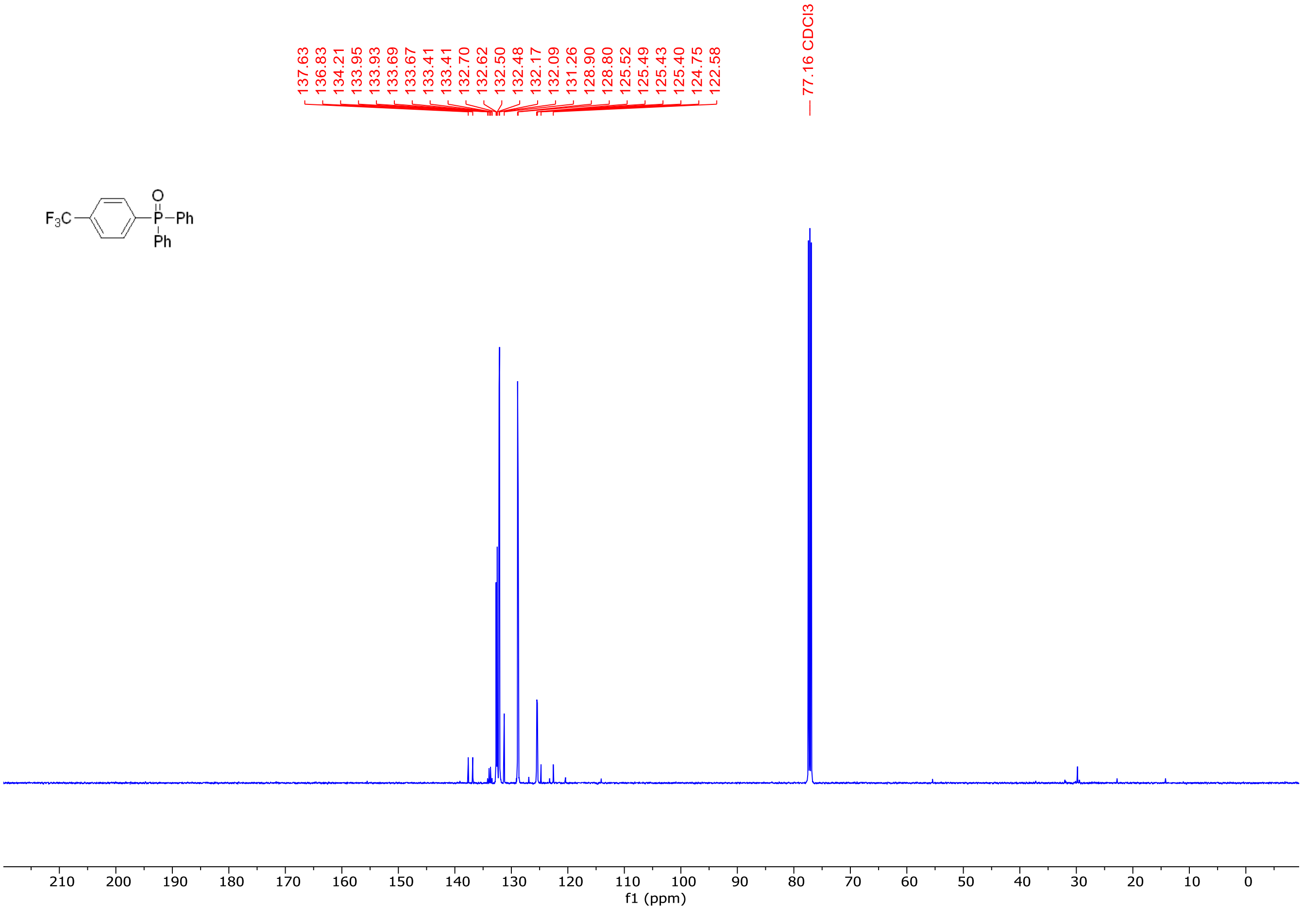

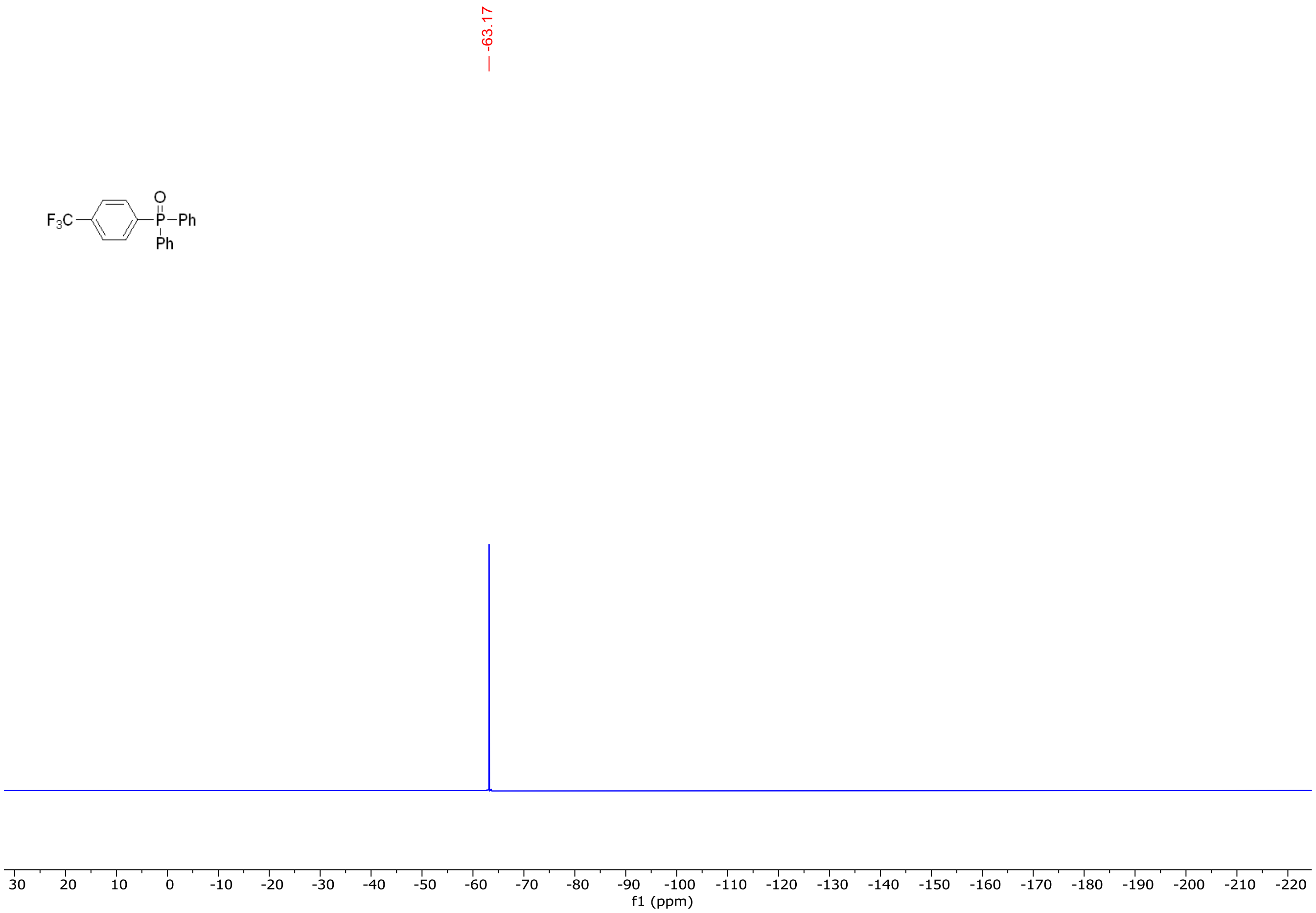
-63.17
F3C
O
P
Ph
Ph
30 20 10 0 -10 -20 -30 -40 -50 -60 -70 -80 -90 -100 -110 -120 -130 -140 -150 -160 -170 -180 -190 -200 -210 -220
f1 (ppm)

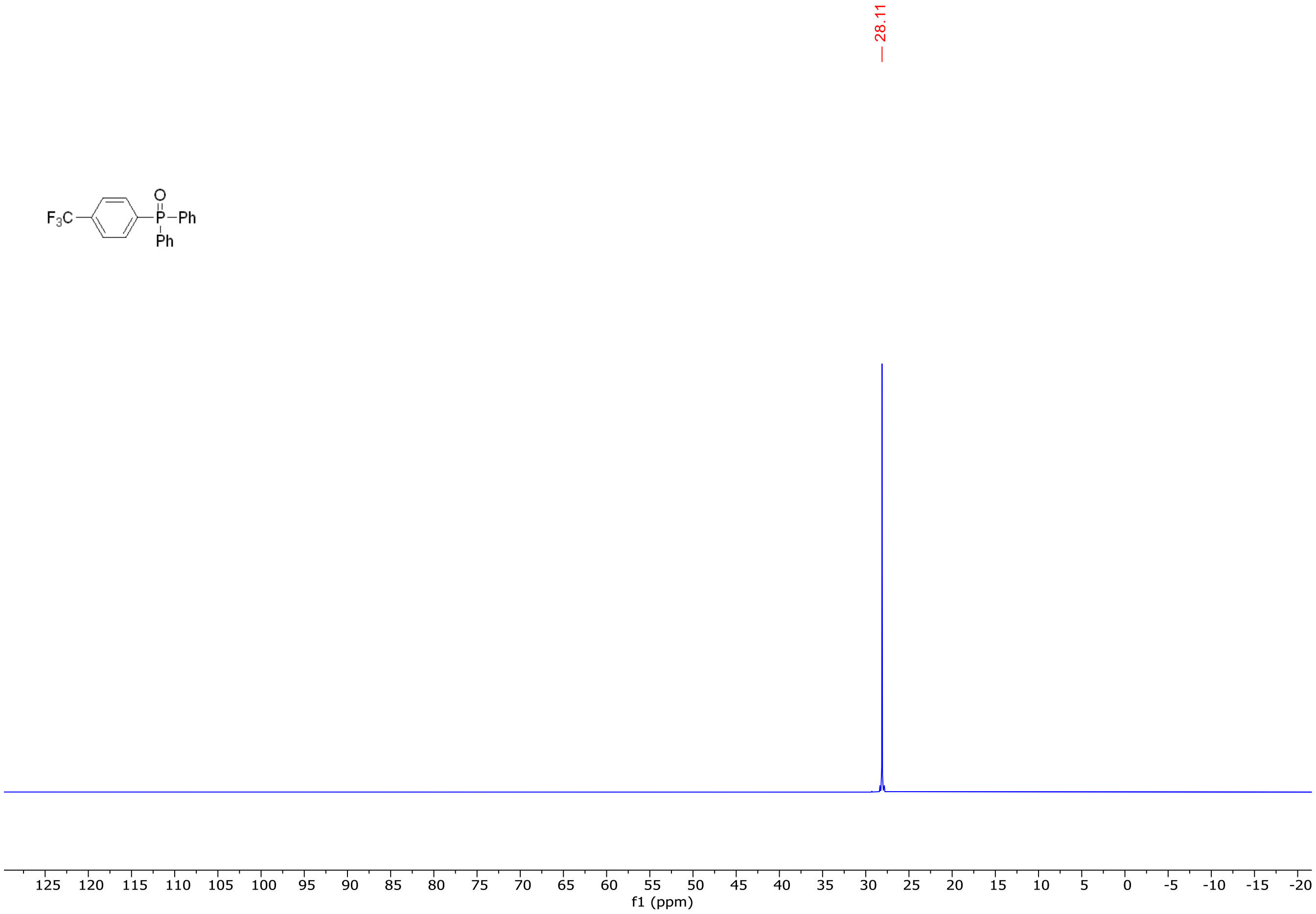
28.11
F3C
O
P
Ph
Ph
125 120 115 110 105 100 95 90 85 80 75 70 65 60 55 50 45 40 35 30 25 20 15 10 5 0 -5 -10 -15 -20
f1 (ppm)

methyl 4-(diphenylphosphoryl)benzoate (119)

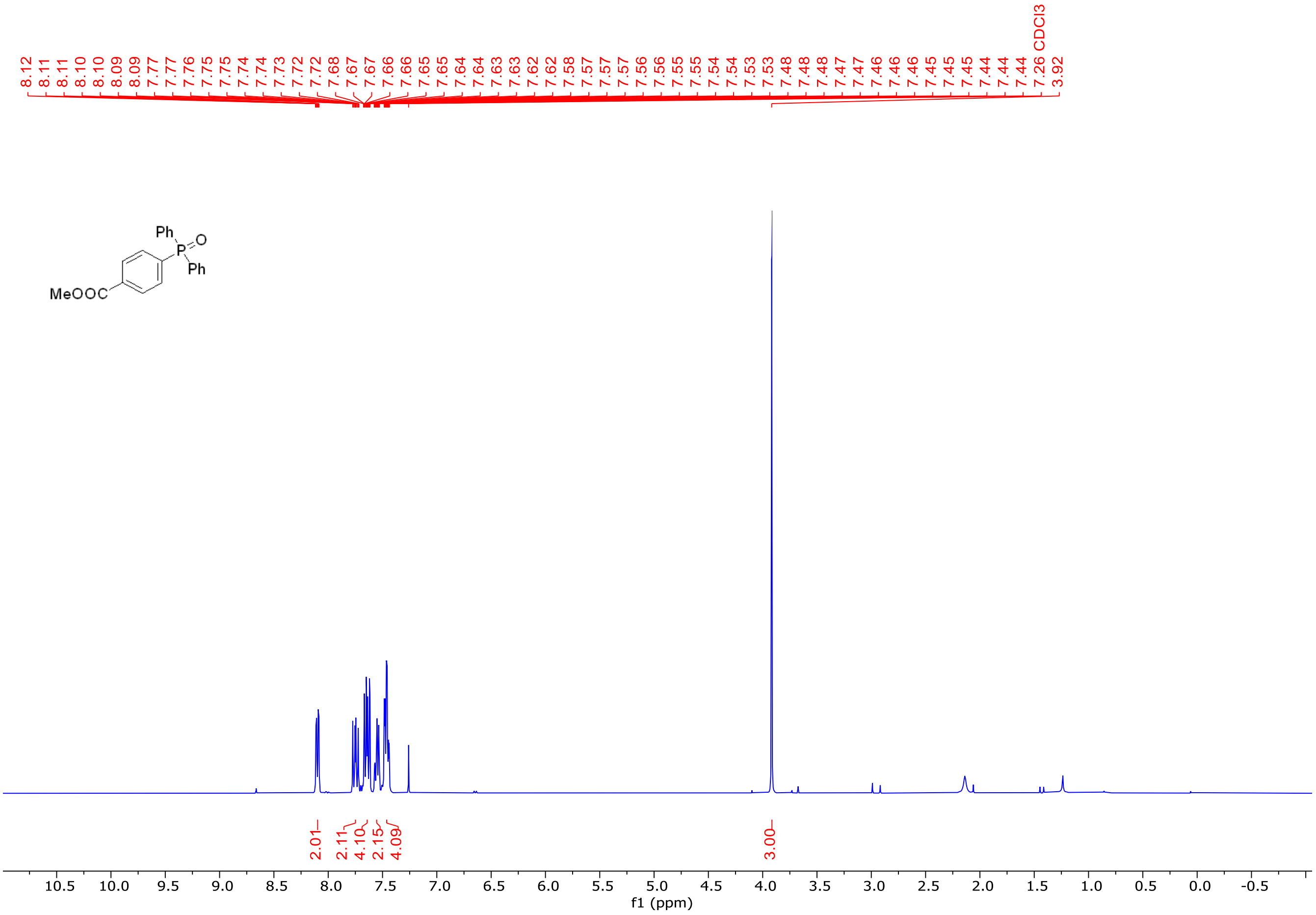

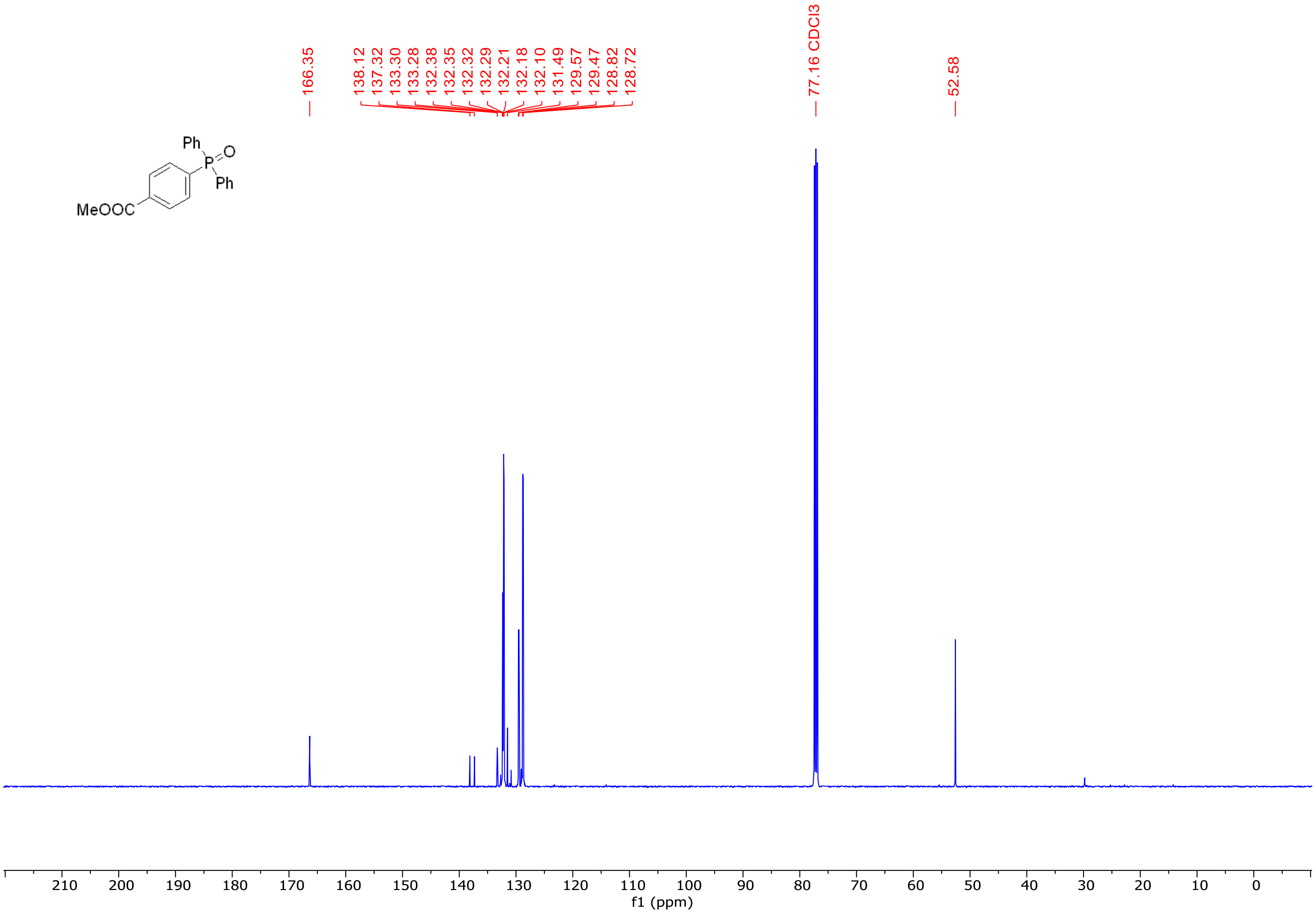

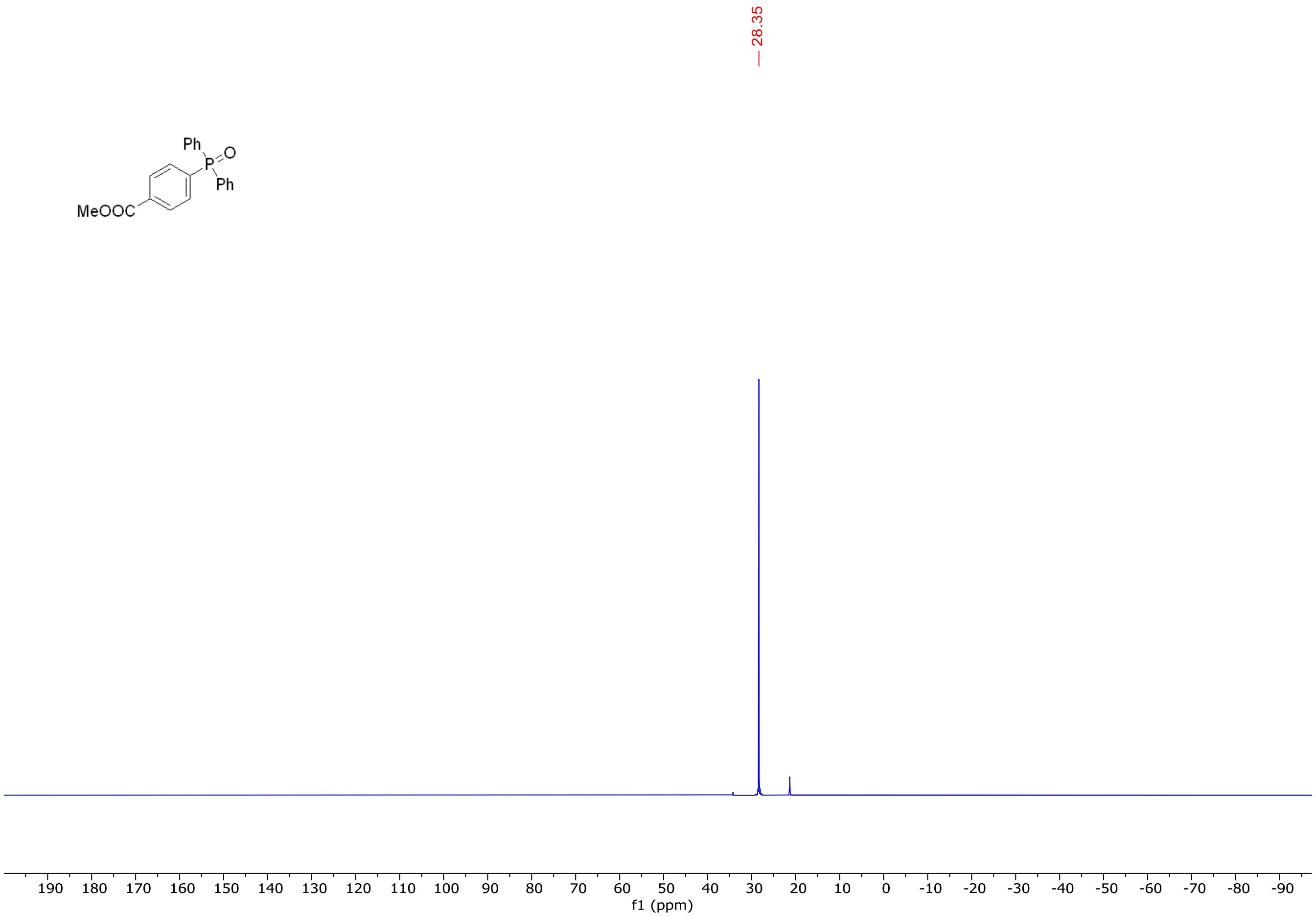

28.35
MeOOC
Ph
P
O
Ph
190 180 170 160 150 140 130 120 110 100 90 80 70 60 50 40 30 20 10 0 -10 -20 -30 -40 -50 -60 -70 -80 -90
f1 (ppm)

isopropyl 2-methyl-2-(4-(4-(piperidin-1-yl)benzoyl)phenoxy)propanoate (120)

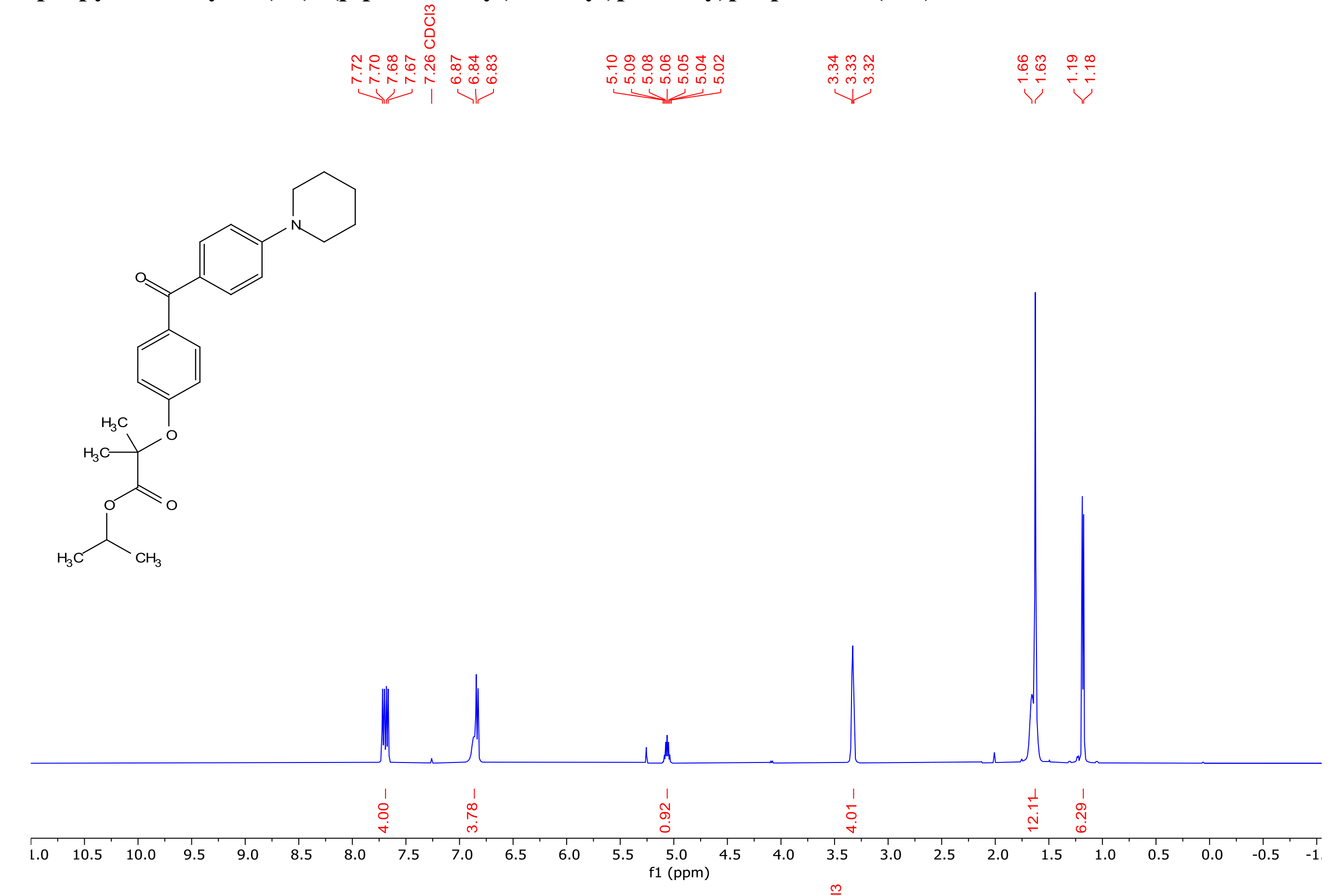

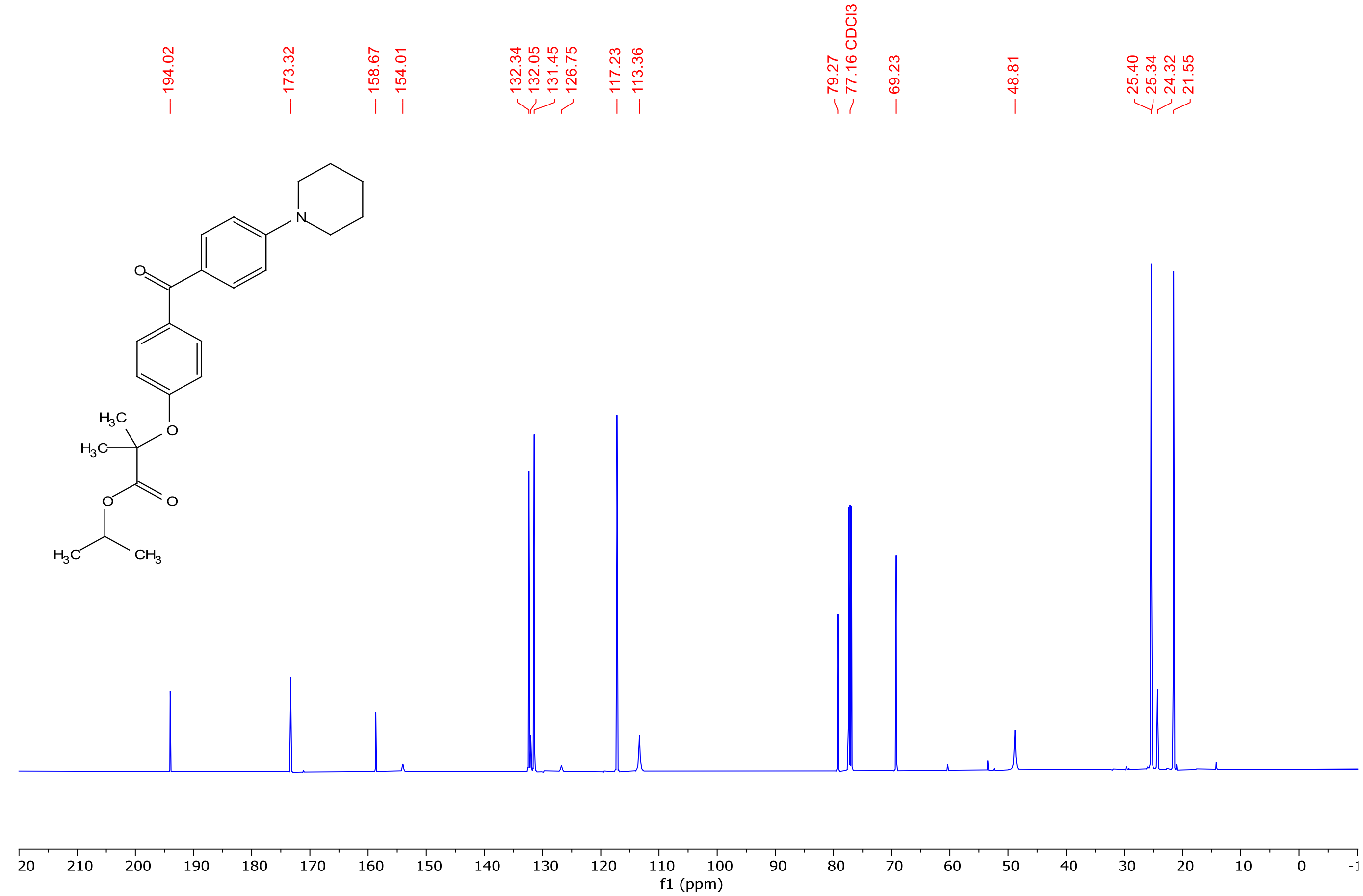

4-(piperidin-1-yl)phenyl adamantane-1-carboxylate (121)

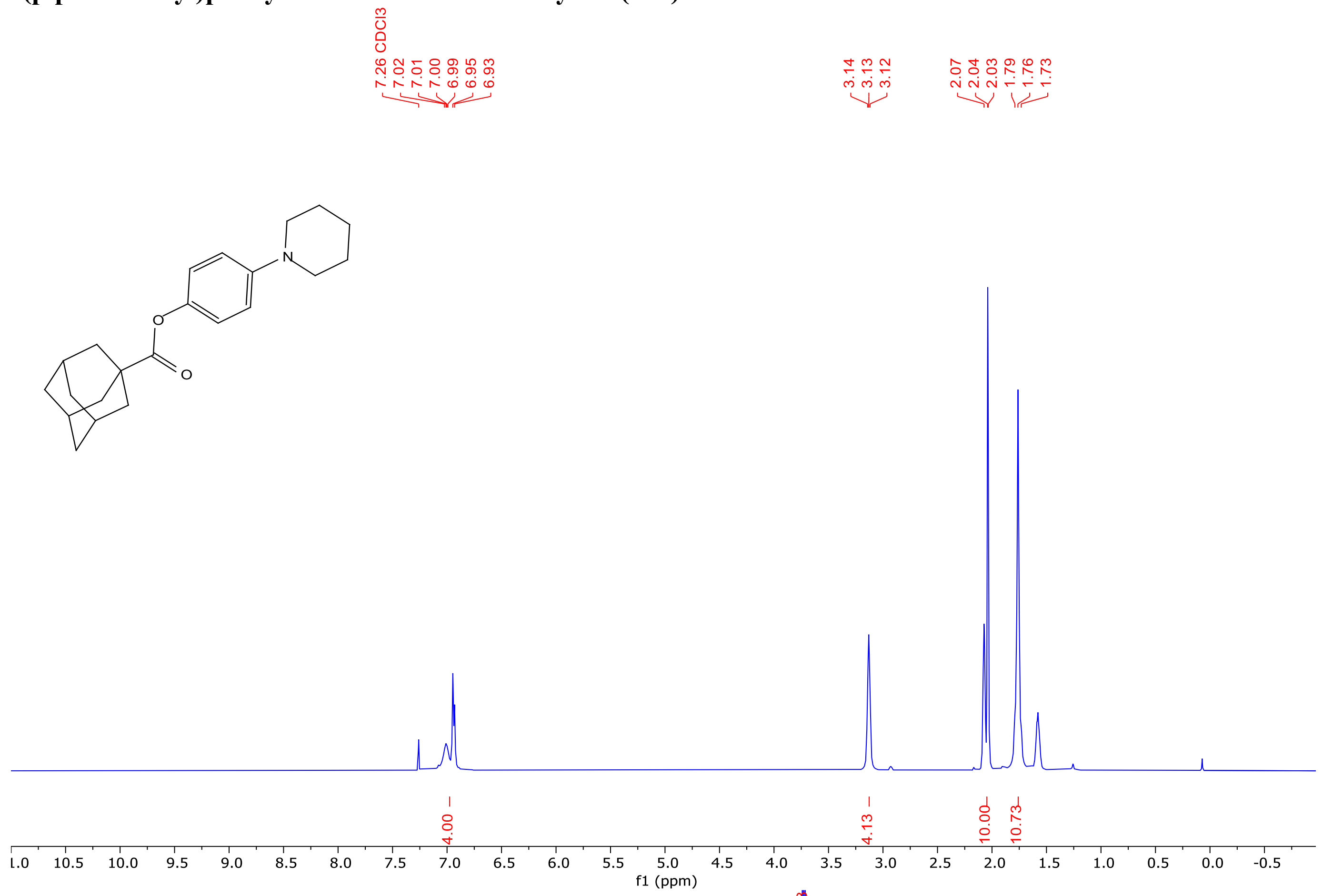

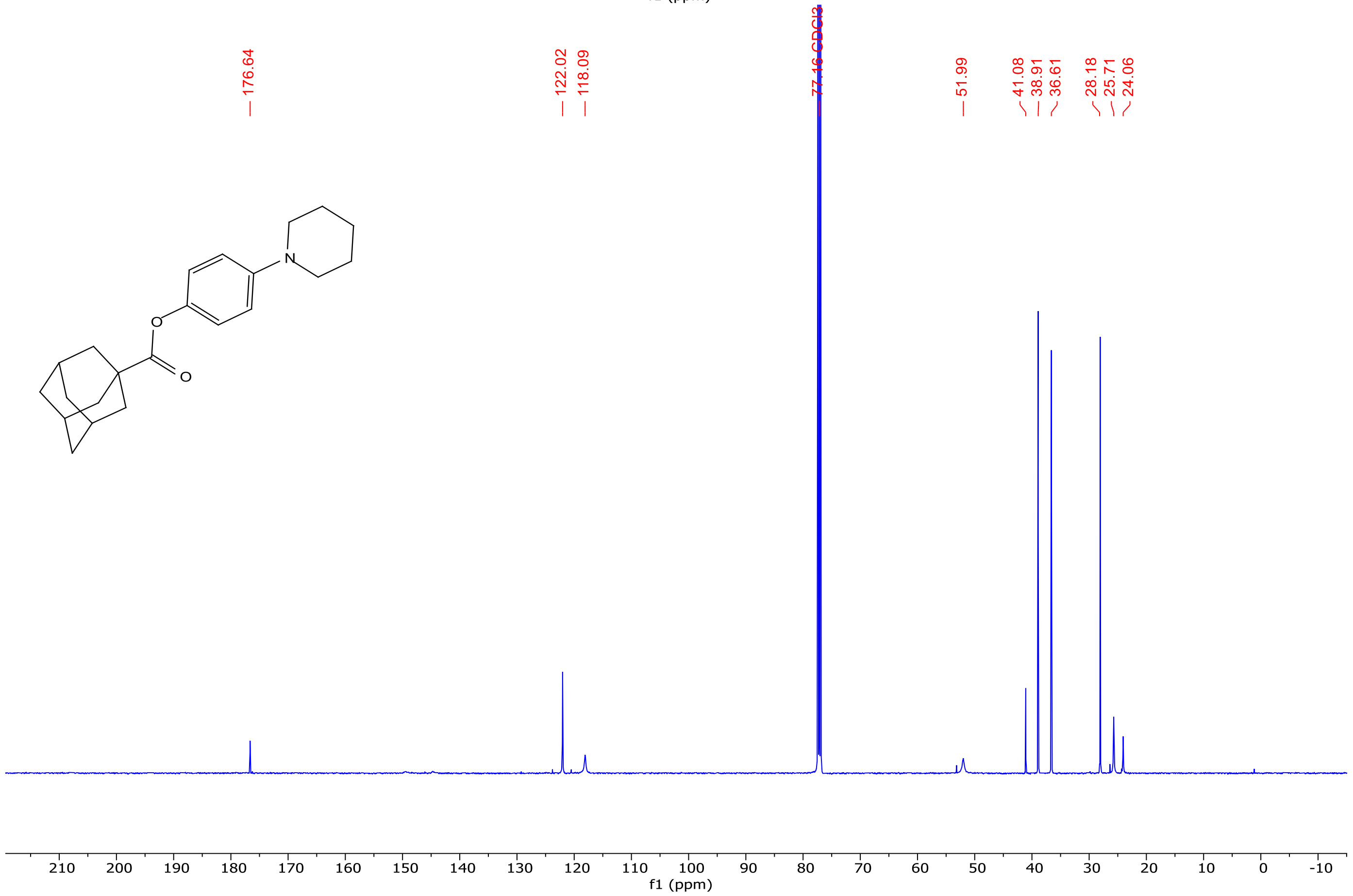

((5R,5aS,8aS,8bR)-2,2,7,7-tetramethyltetrahydro-5*H*-bis([1,3]dioxolo)[4,5-b:4',5'-d]pyran-5-yl)methyl 4-(piperidin-1-yl)benzoate (122)

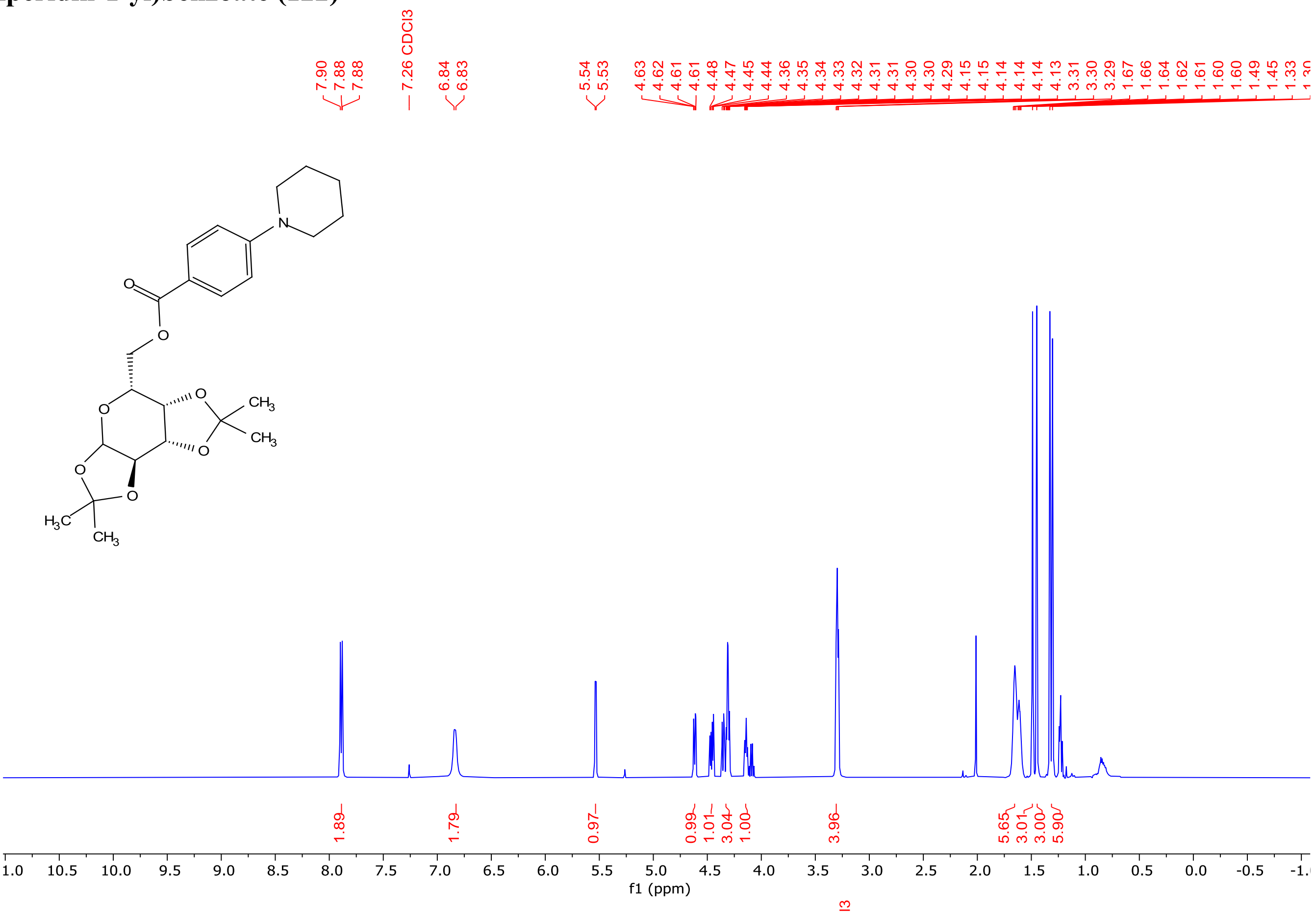

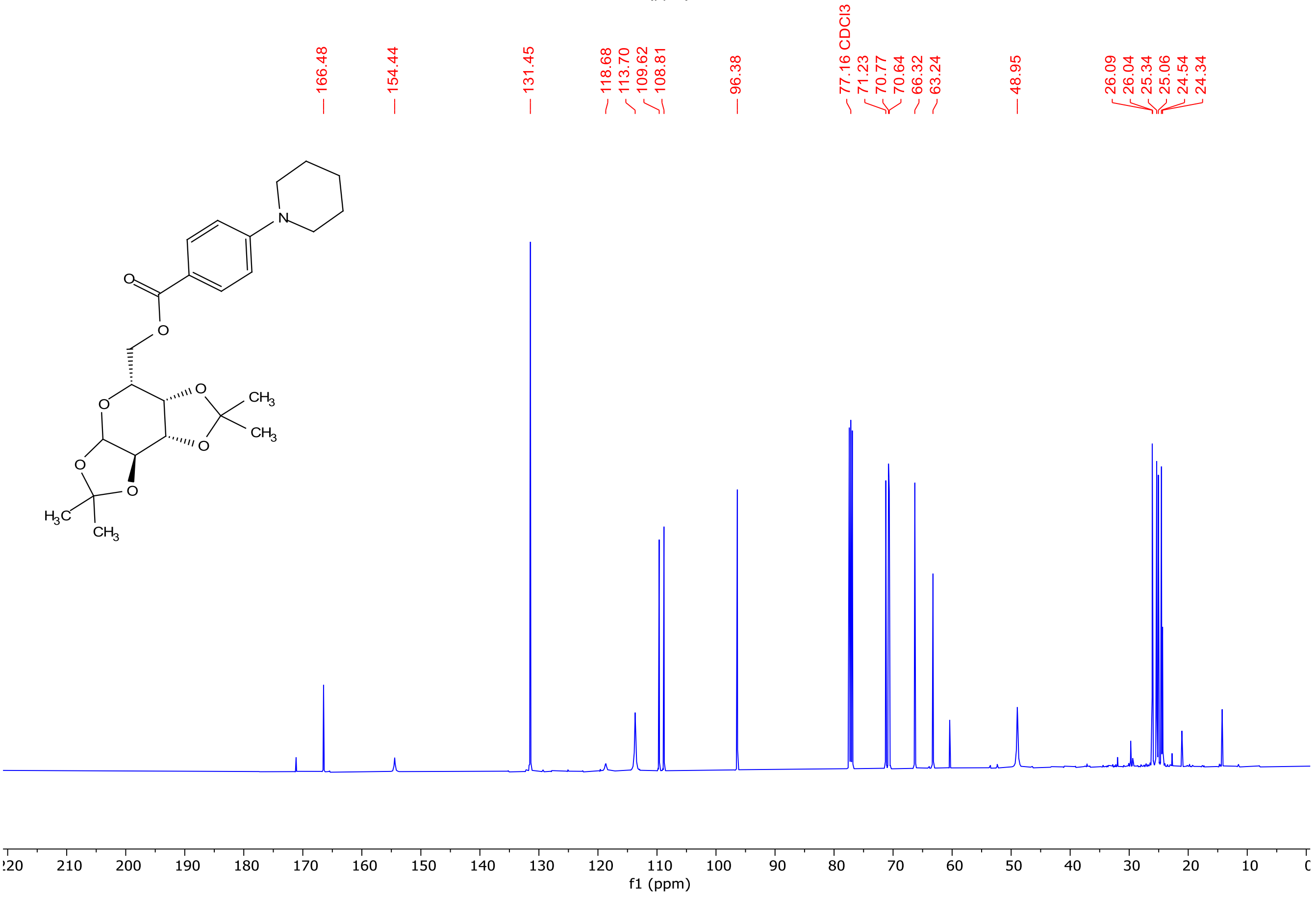

((5R,5aS,8aS,8bR)-2,2,7,7-tetramethyltetrahydro-5*H*-bis([1,3]dioxolo)[4,5-b:4',5'-d]pyran-5-yl)methyl 4-(piperidin-1-yl)benzoate (123)

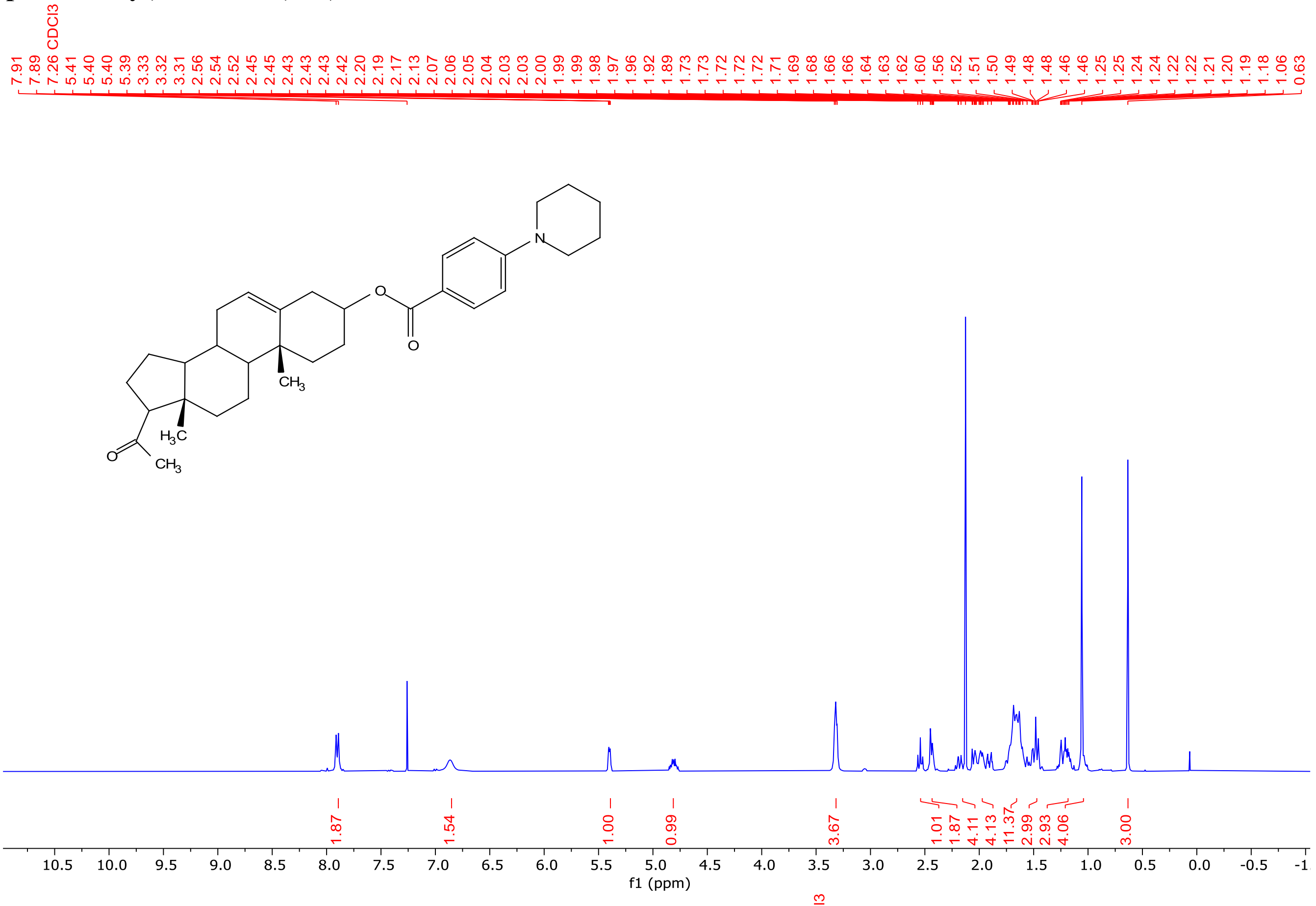

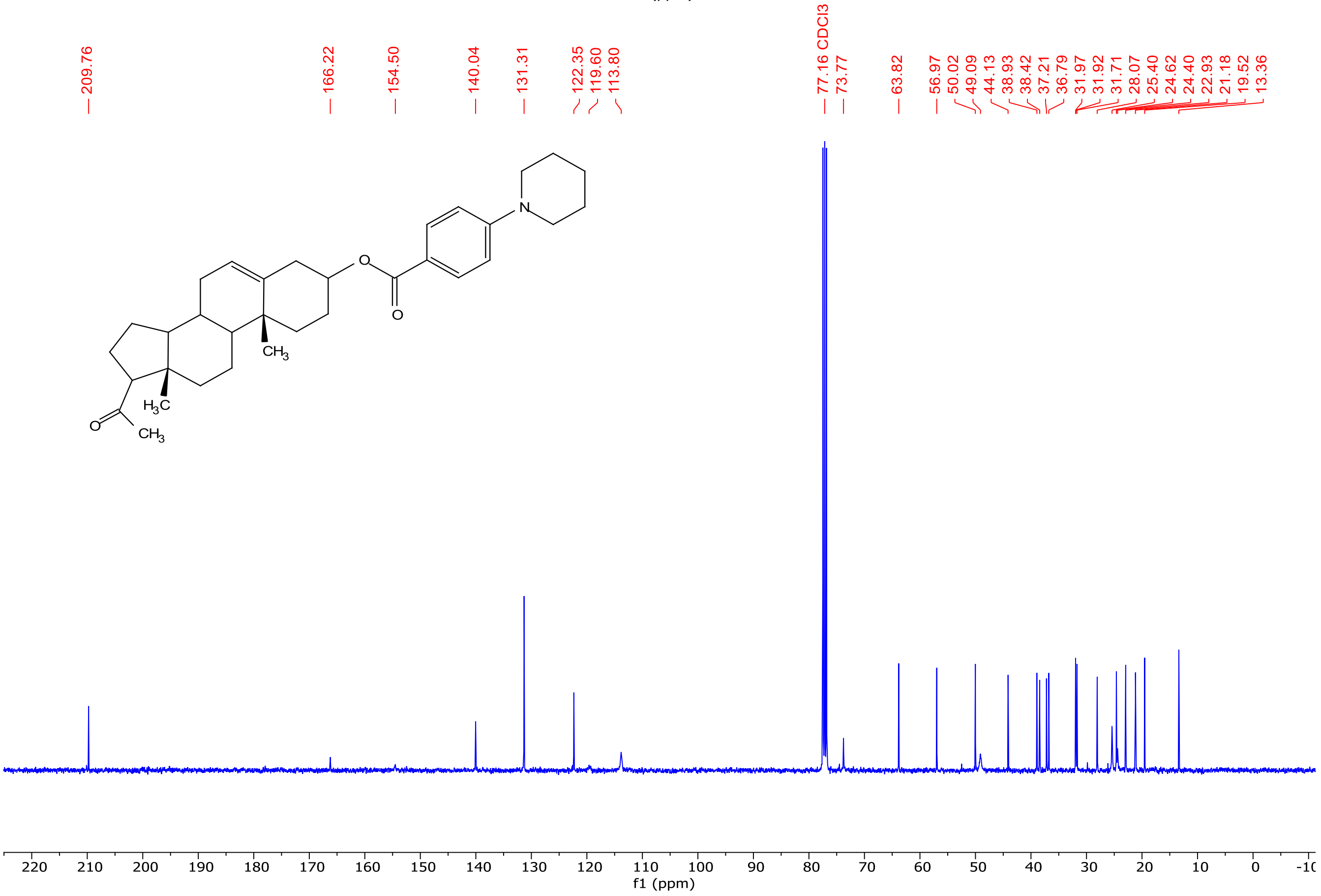

(4-(4-(2-chlorodibenzo[b,f][1,4]oxazepin-11-yl)piperazin-1-yl)phenyl)(phenyl)methanone (124)

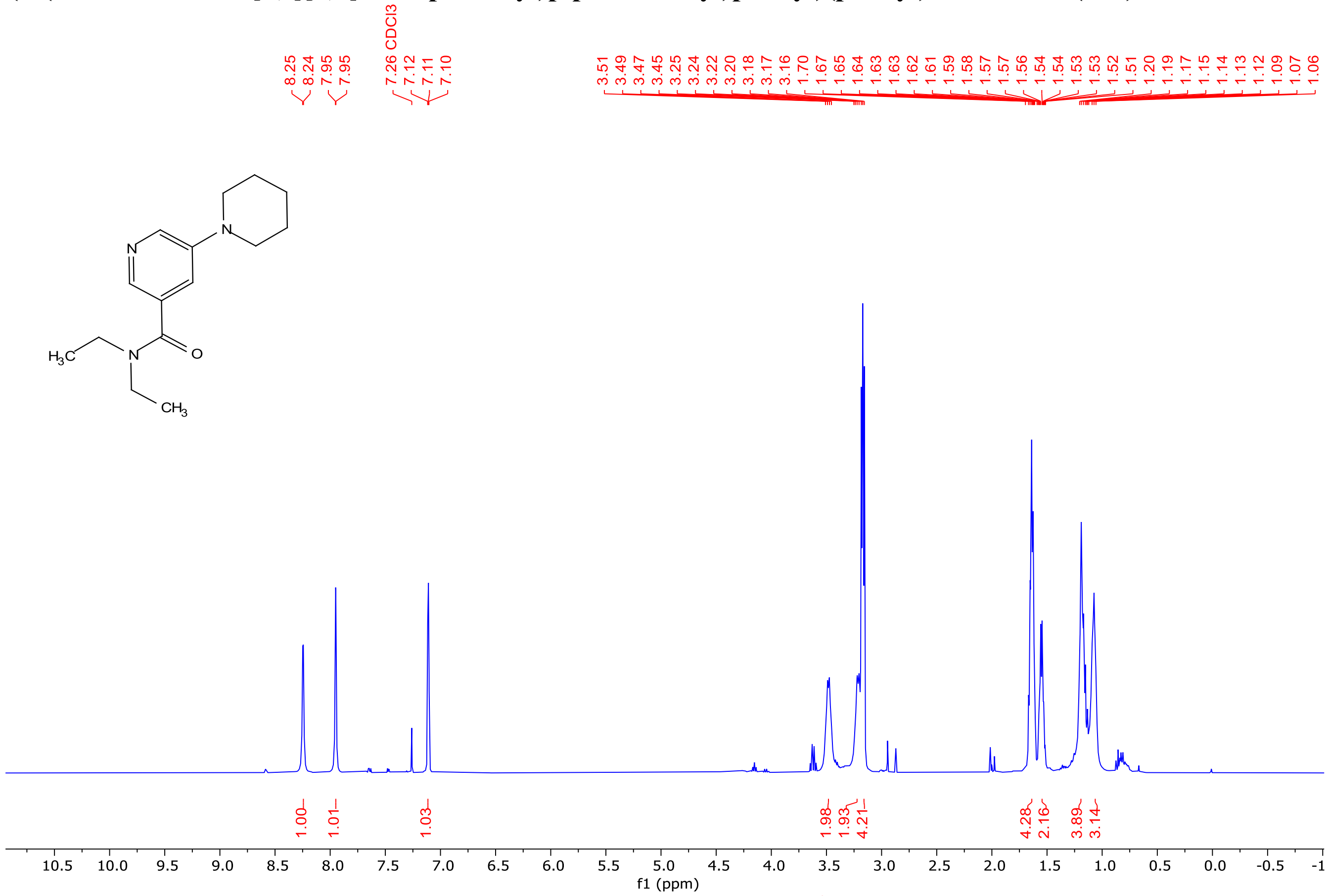

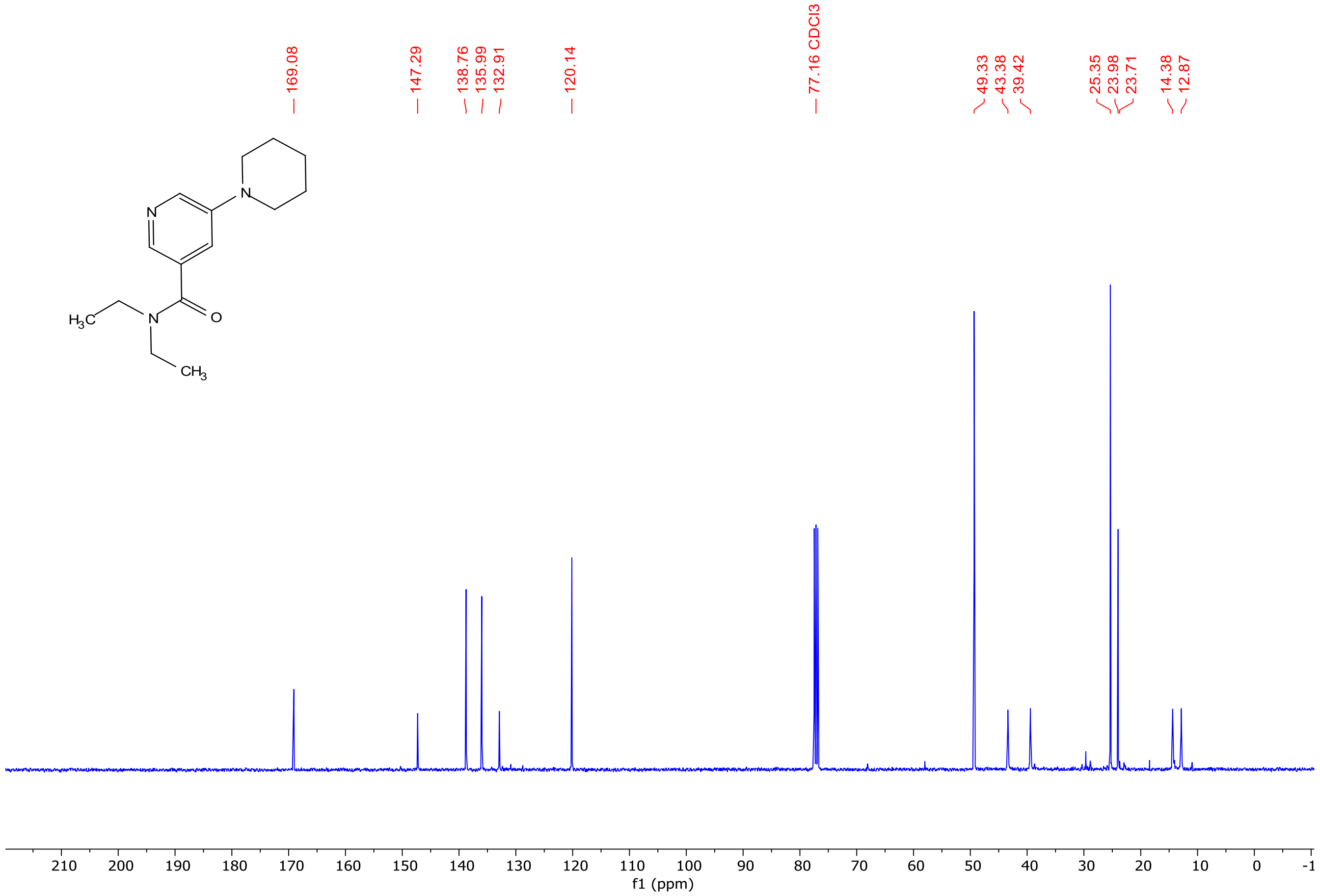

(4-((2-(benzo[d][1,3]dioxol-5-yl)ethyl)amino)phenyl)(phenyl)methanone (125)

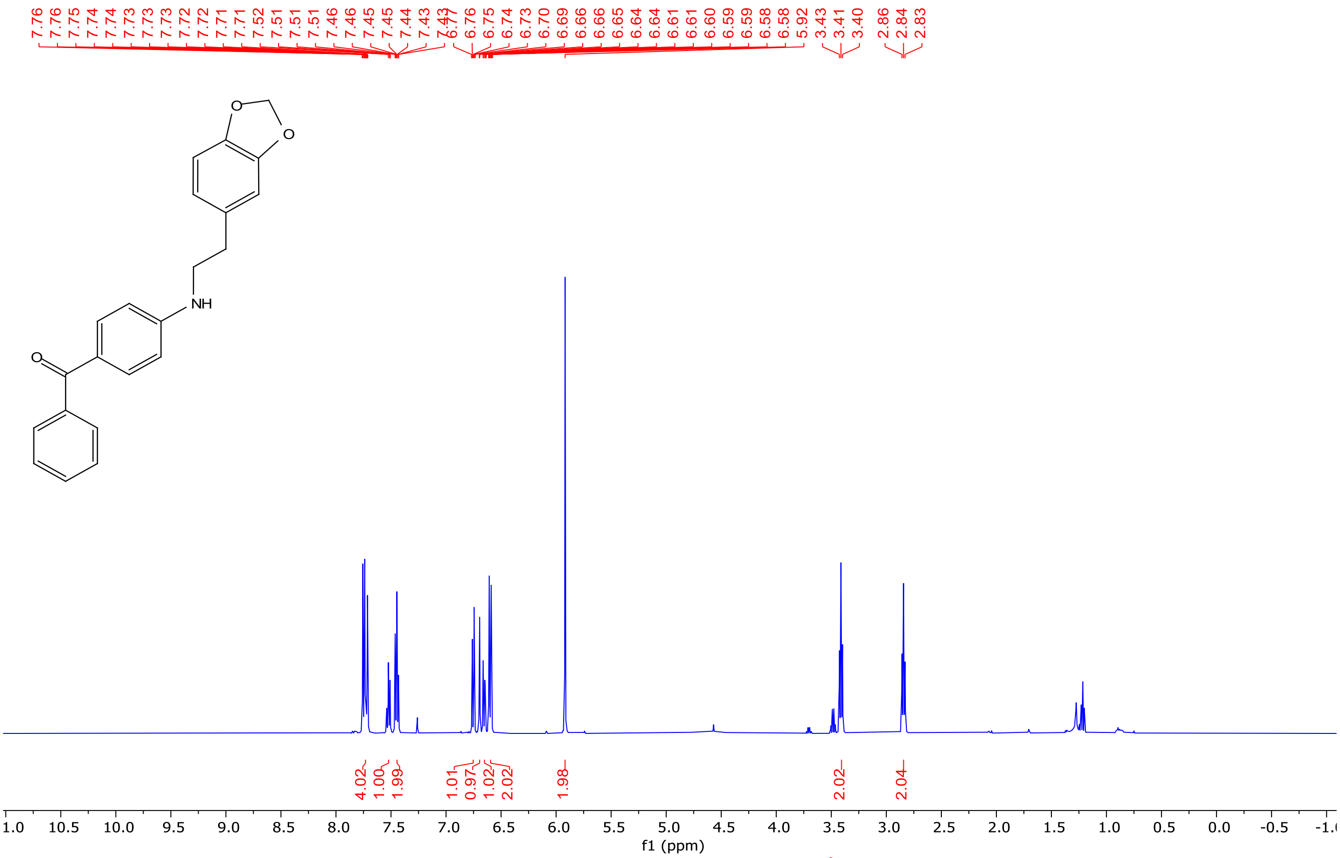

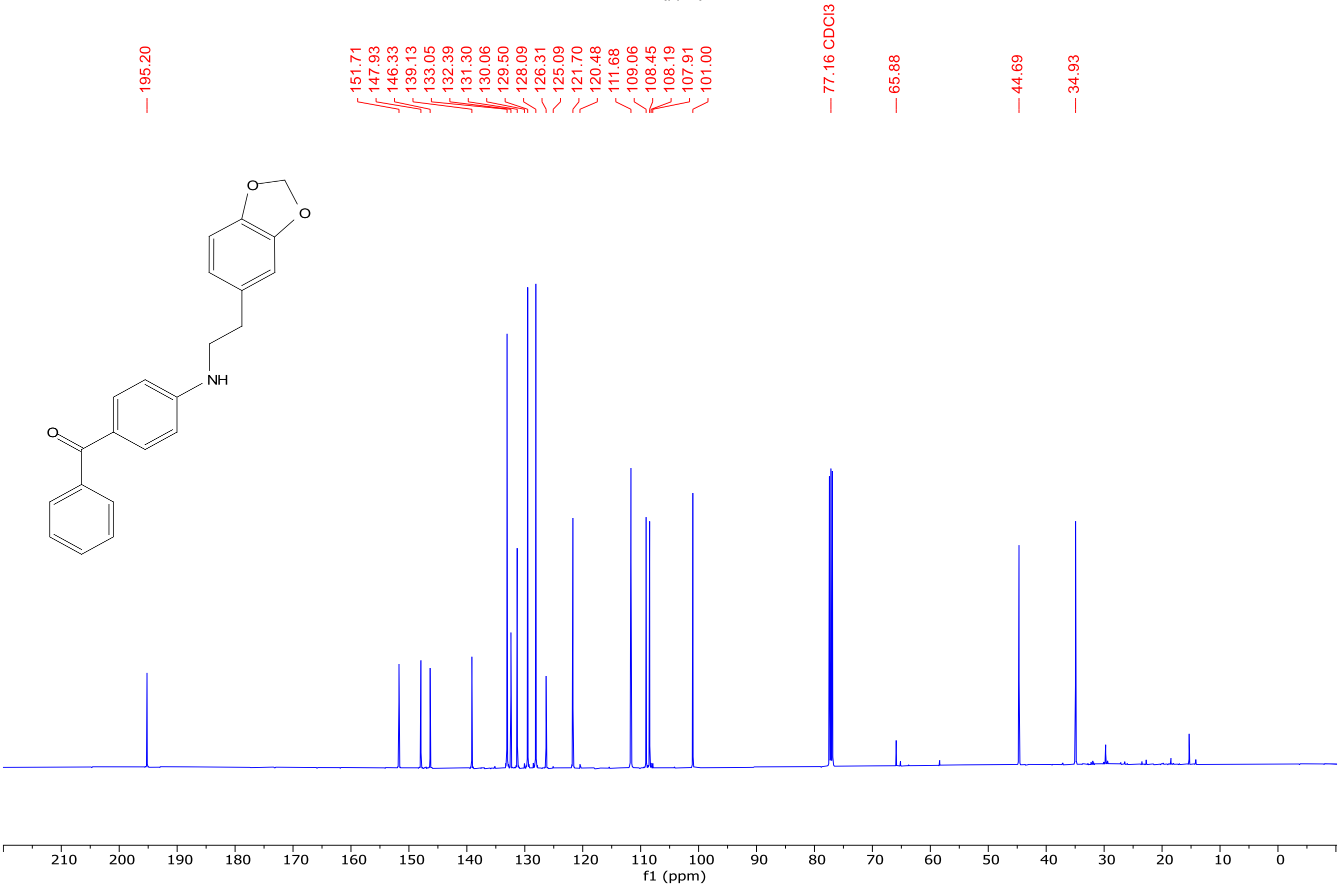

(4-(4-(8-chloro-5,6-dihydro-11*H*-benzo[5,6]cyclohepta[1,2-b]pyridin-11-ylidene)piperidin-1-yl)phenyl)(phenyl)methanone (126)

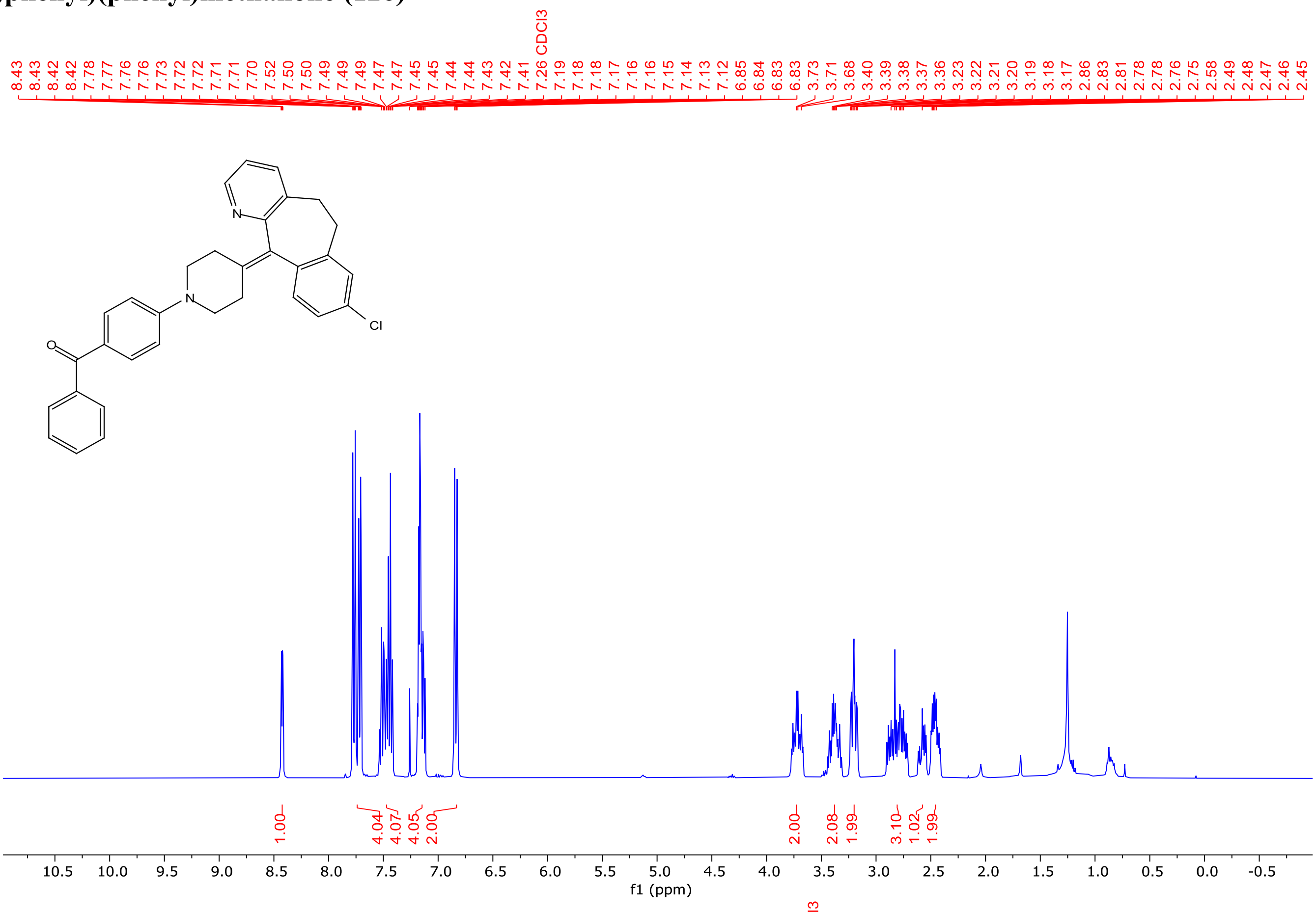

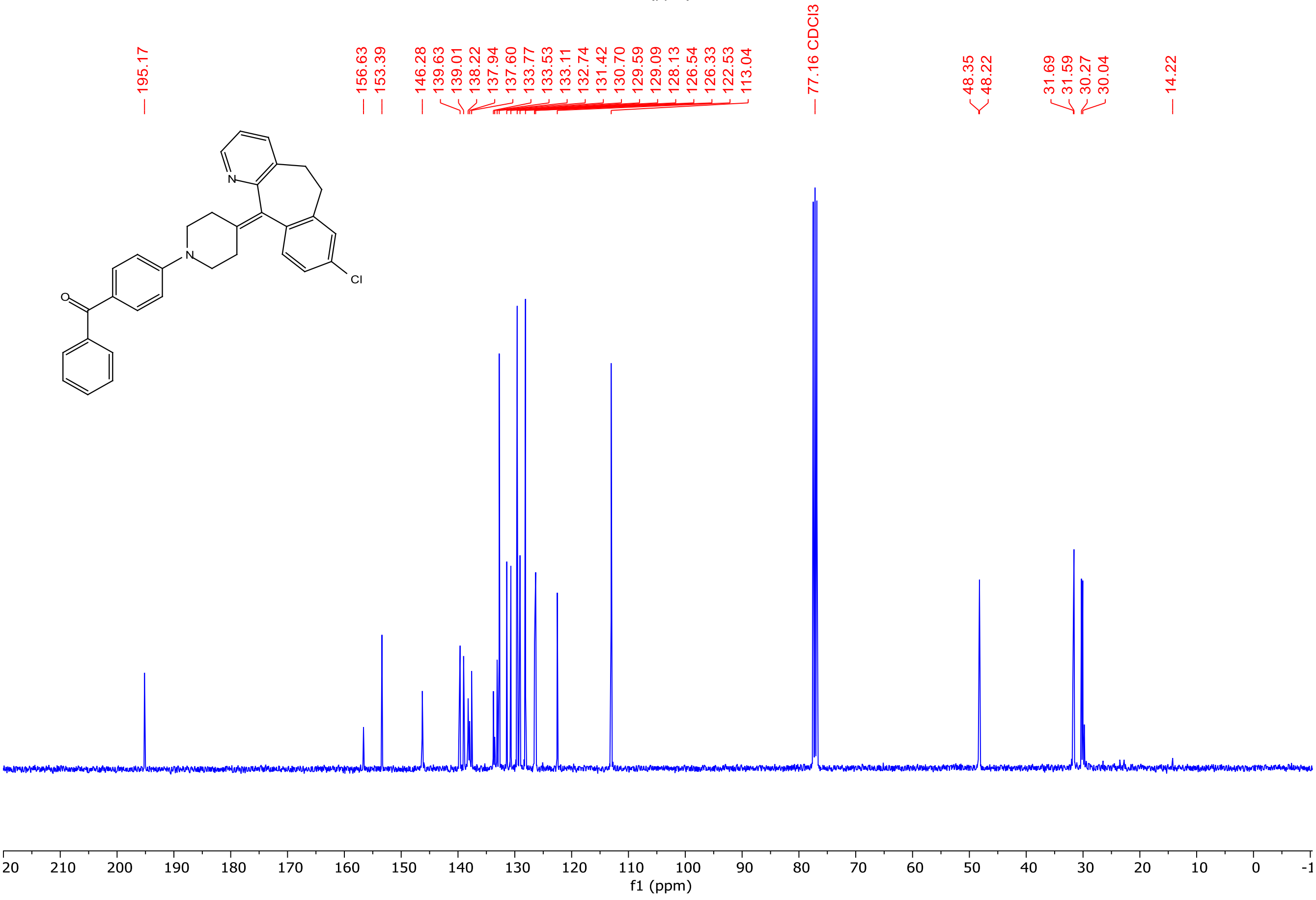

(4-(4-(2-chlorodibenzo[b,f][1,4]oxazepin-11-yl)piperazin-1-yl)phenyl)(phenyl)methanone (127)

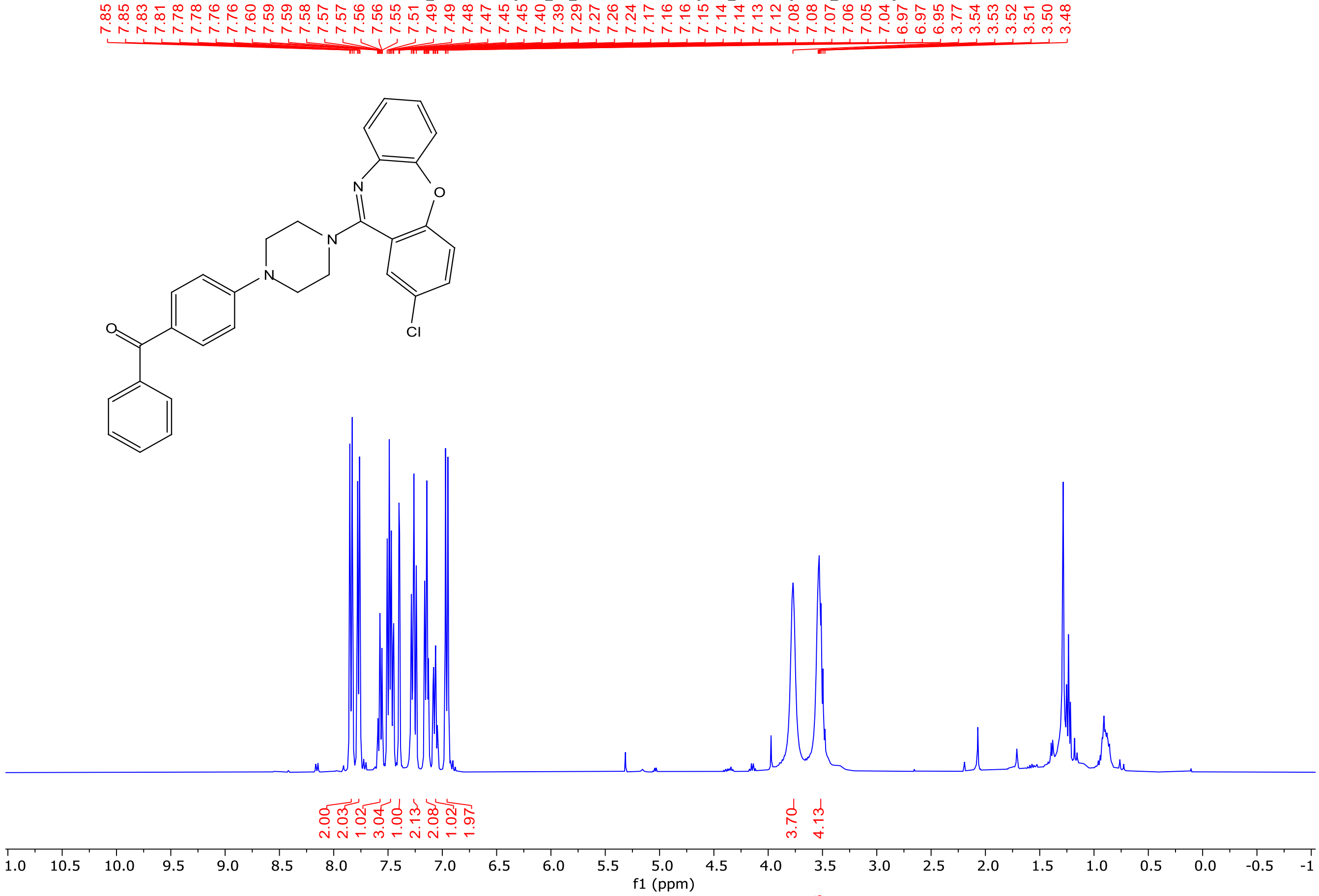

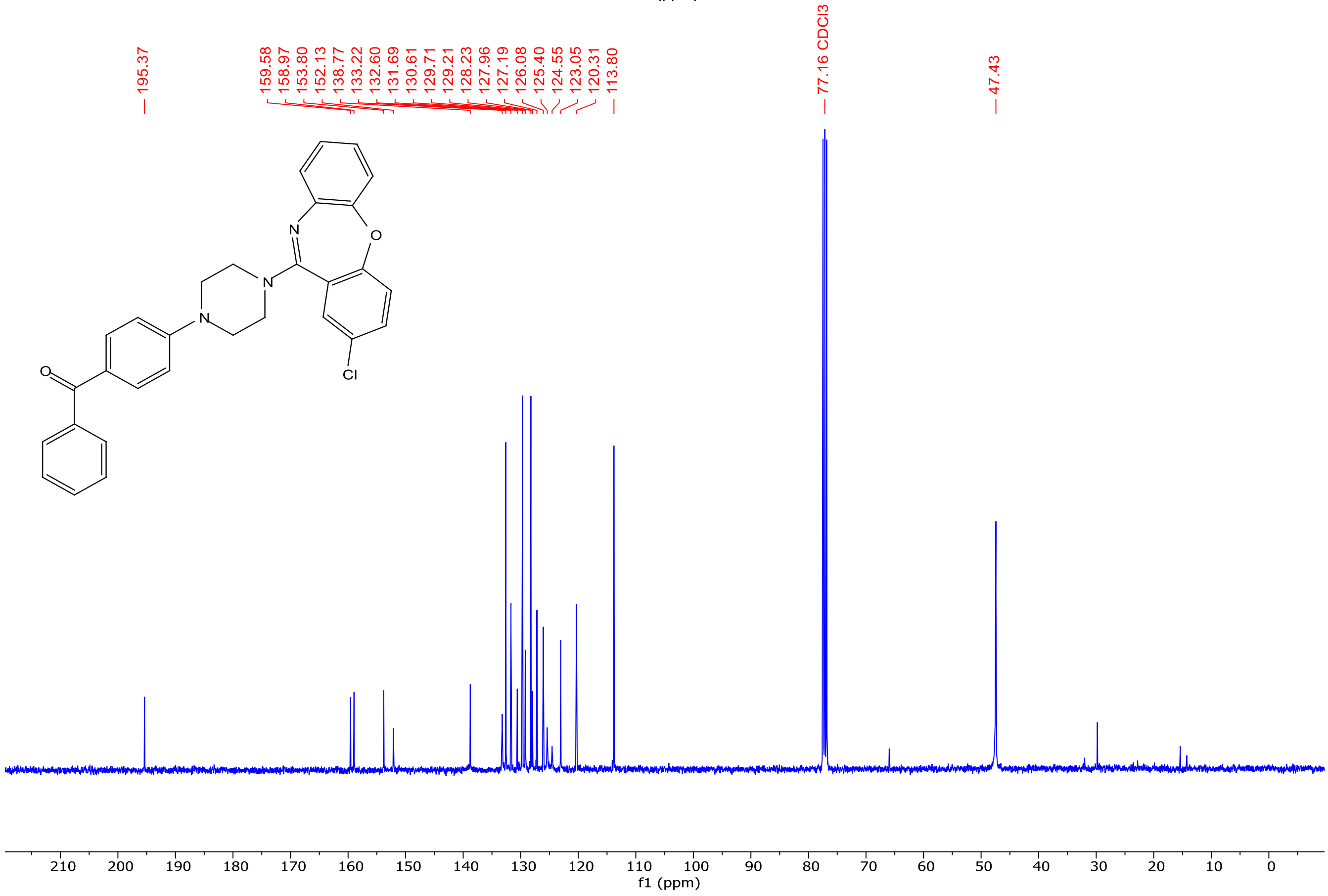

((5R,5aS,8aS,8bR)-2,2,7,7-tetramethyltetrahydro-5*H*-bis([1,3]dioxolo)[4,5-b:4',5'-d]pyran-5-yl)methyl 4-((4-methoxyphenyl)amino)benzoate (128)

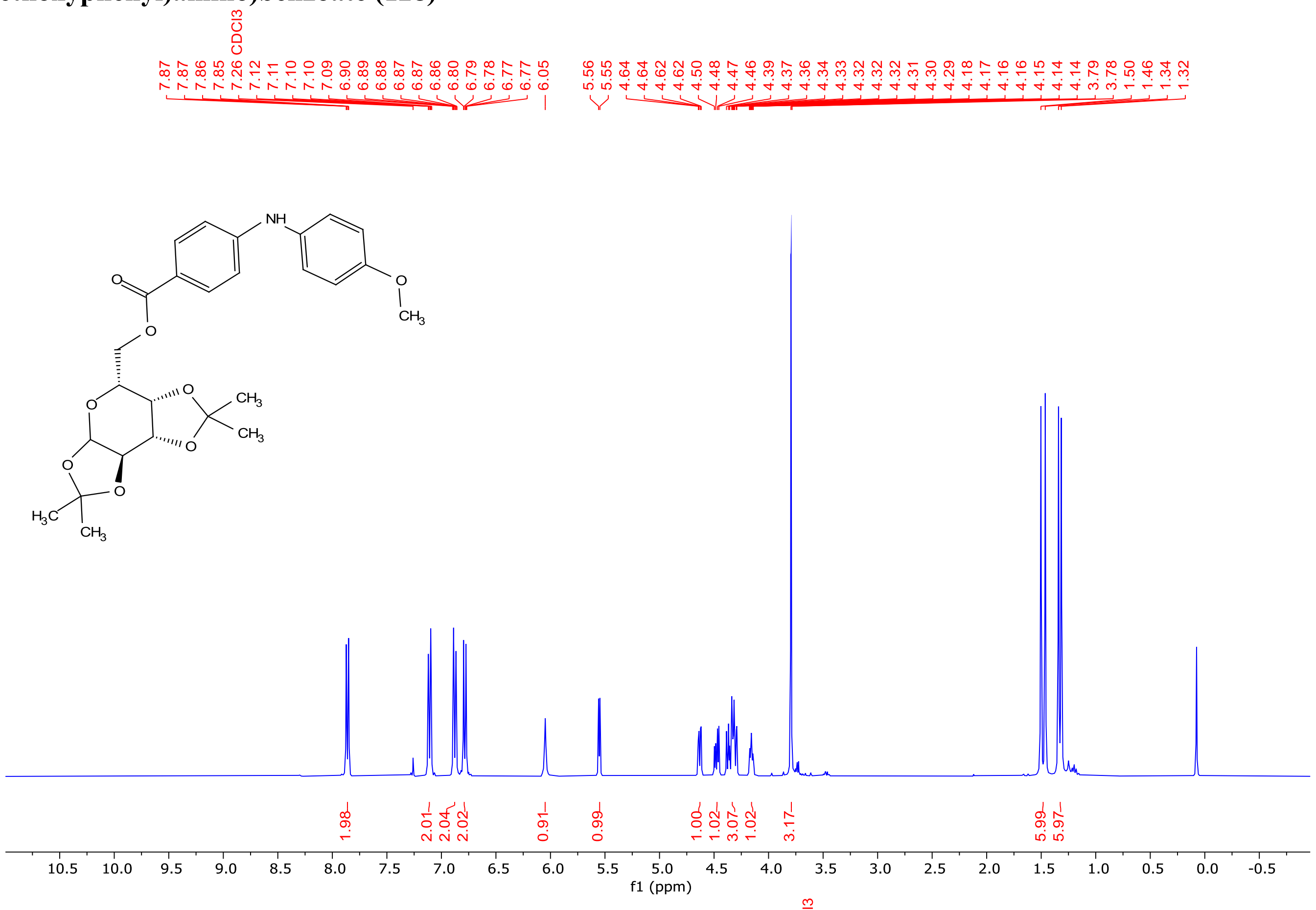

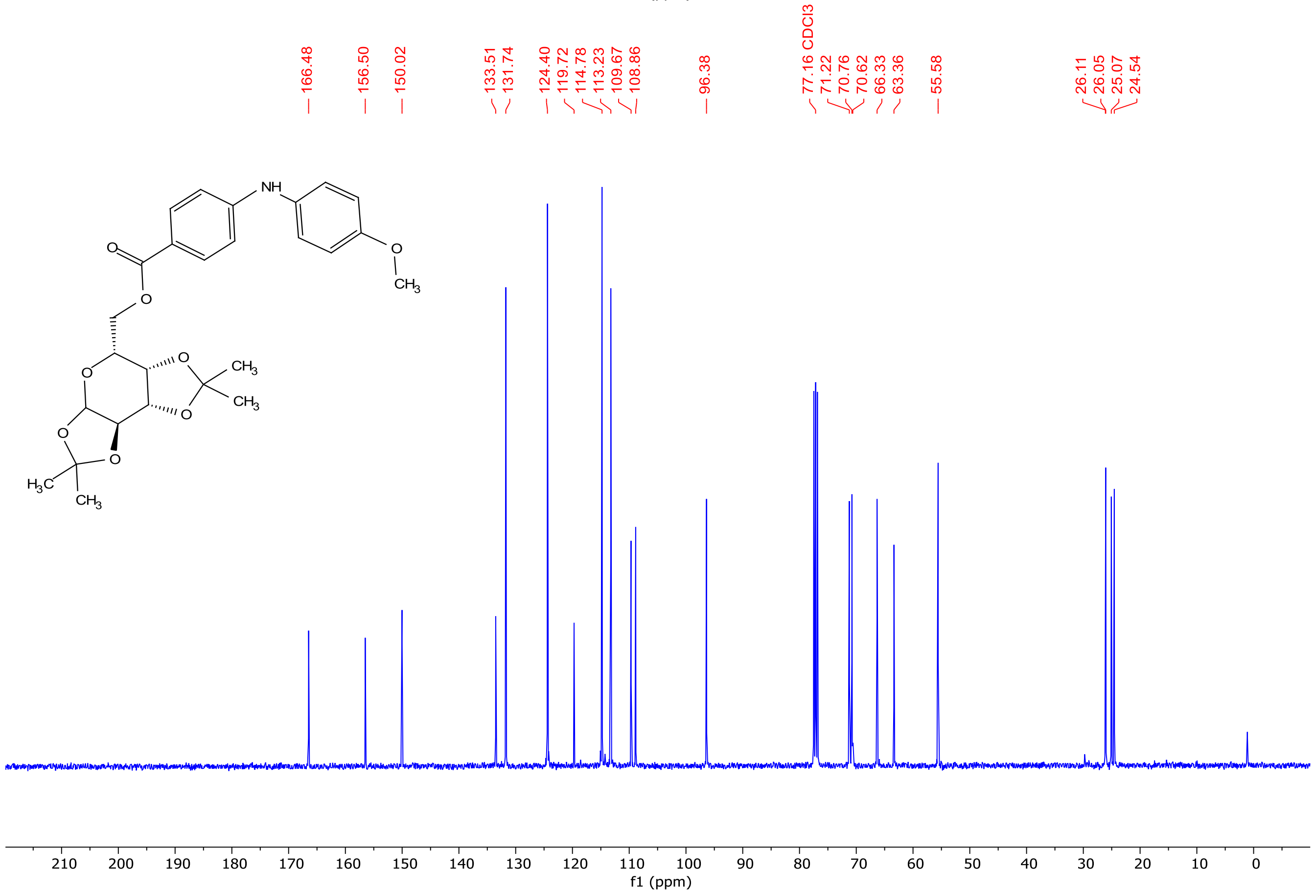

2-(diethylamino)ethyl 4-((4-(trifluoromethyl)phenyl)amino)benzoate (129)

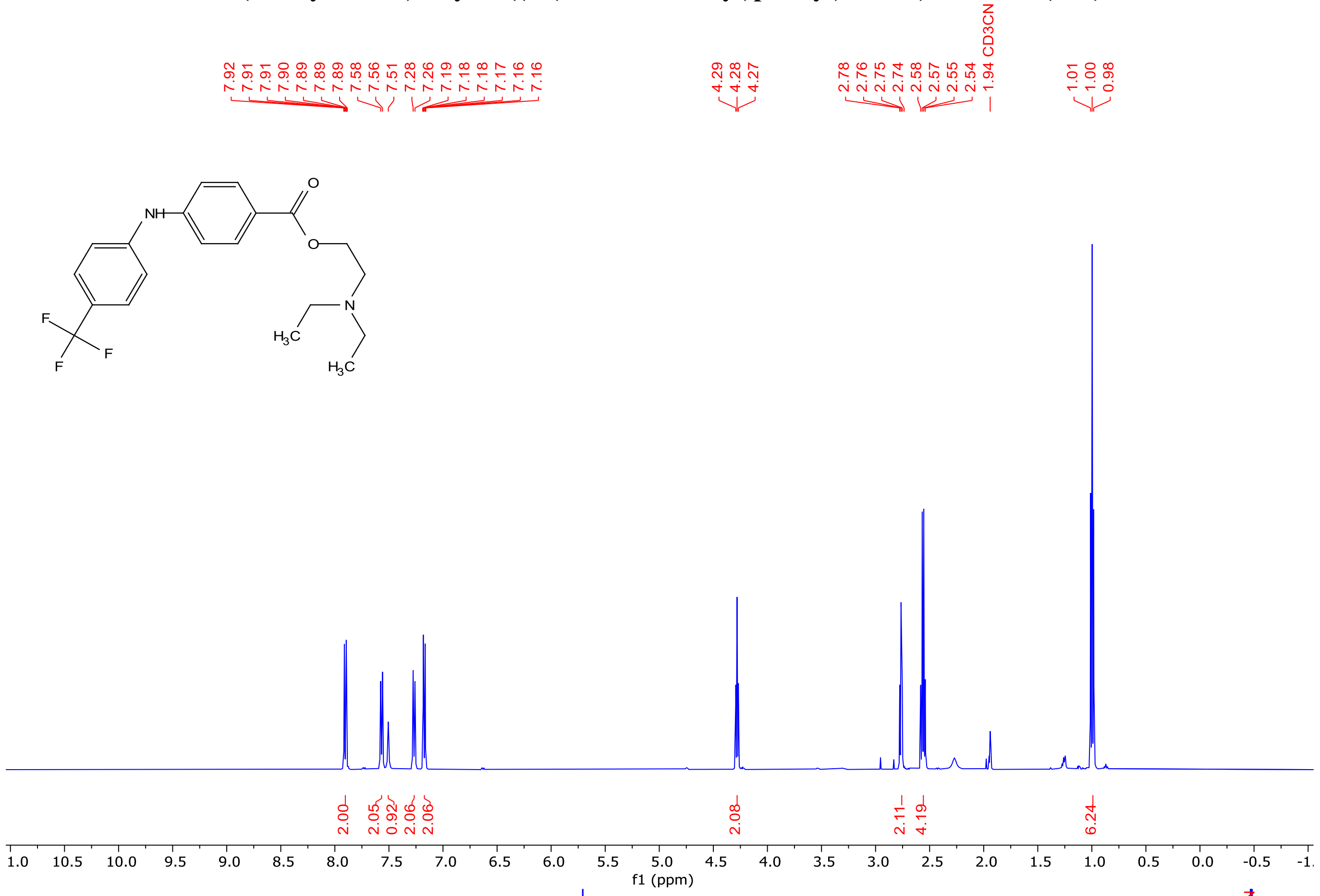

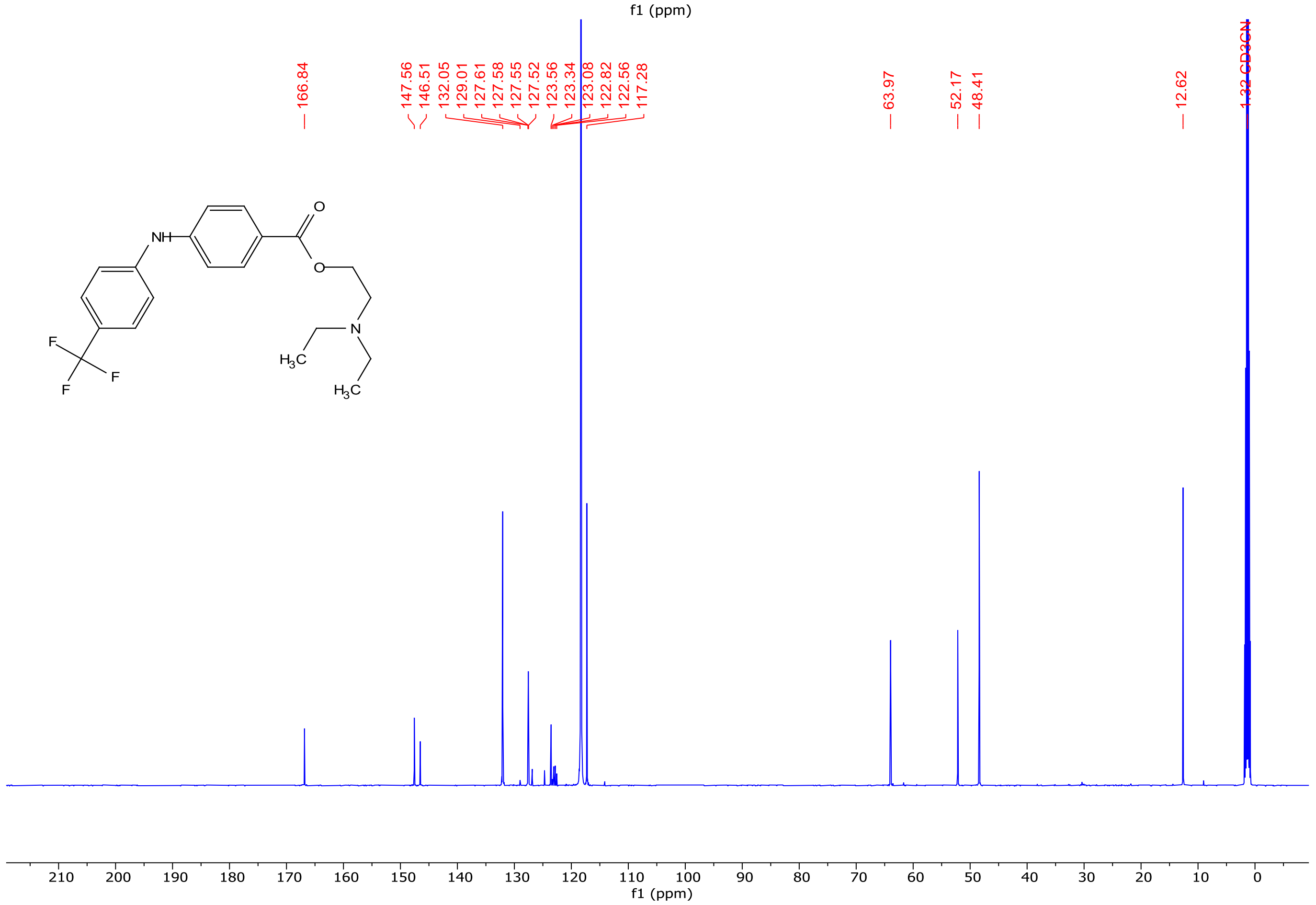

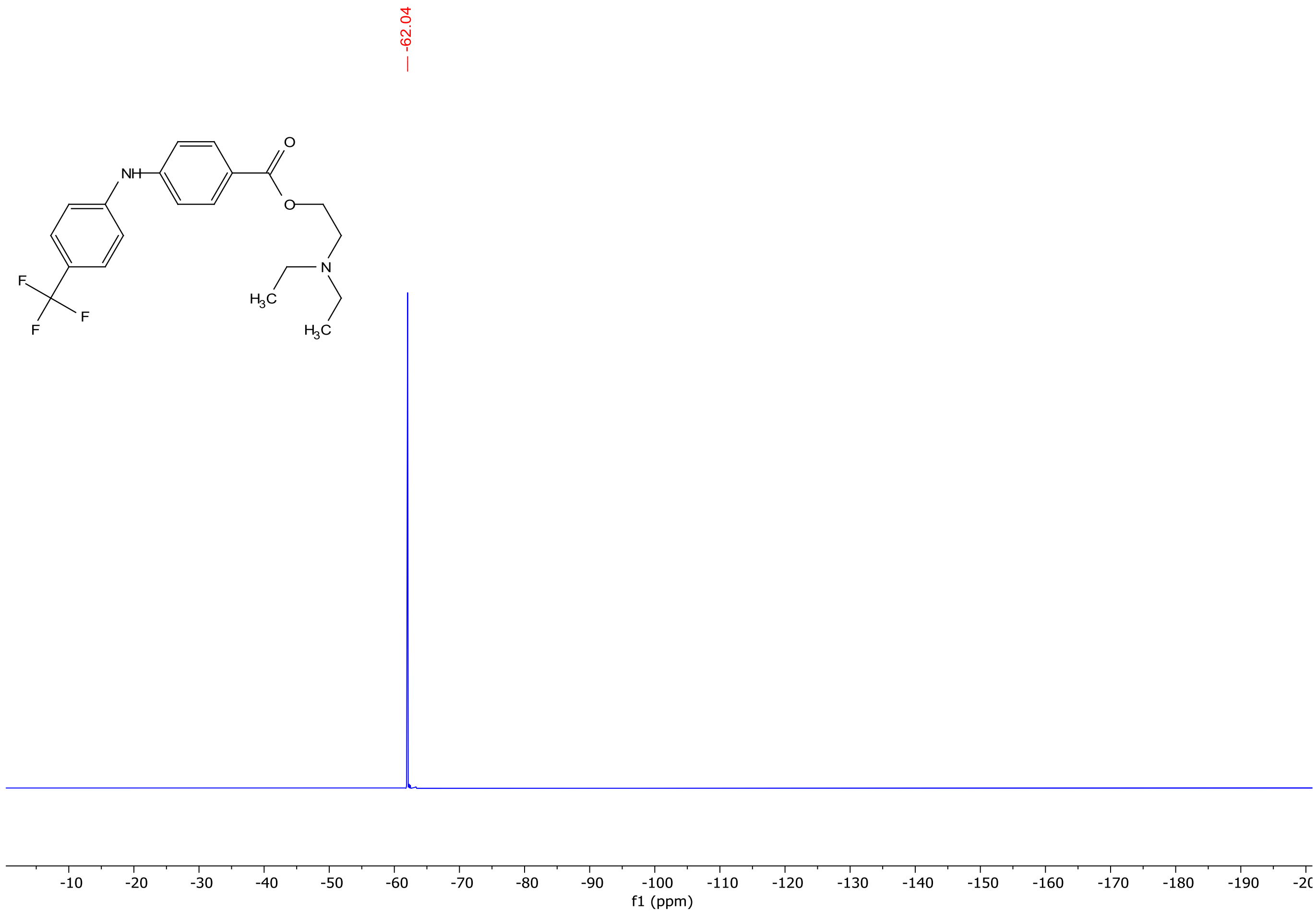
-62.04
NH
O
O
N
F
F
F
H3C
H3C
-10
-20
-30
-40
-50
-60
-70
-80
-90
-100
-110
-120
-130
-140
-150
-160
-170
-180
-190
f1 (ppm)

methyl 4-(2-(4-isobutylphenyl)propanamido)benzoate (130)

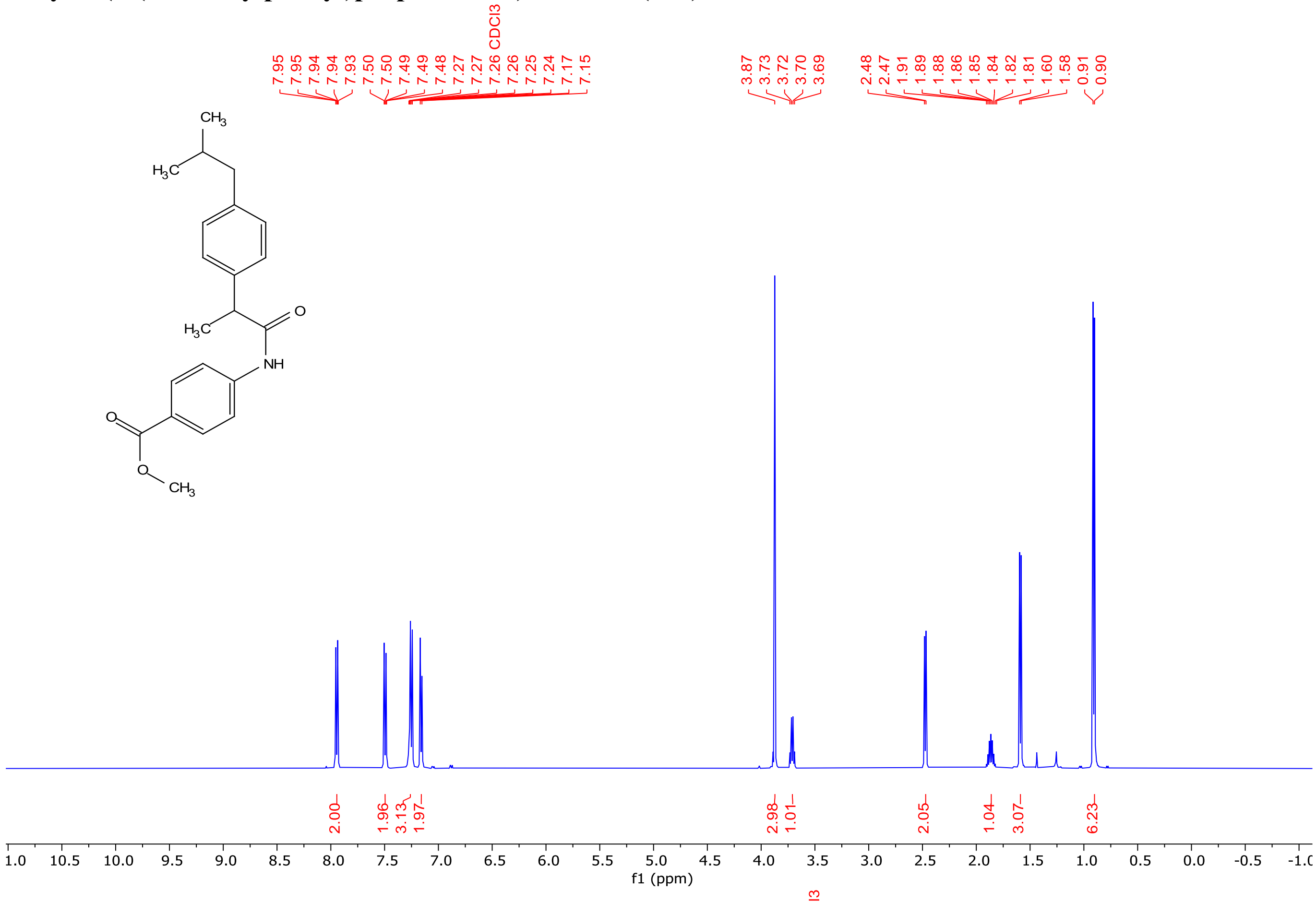

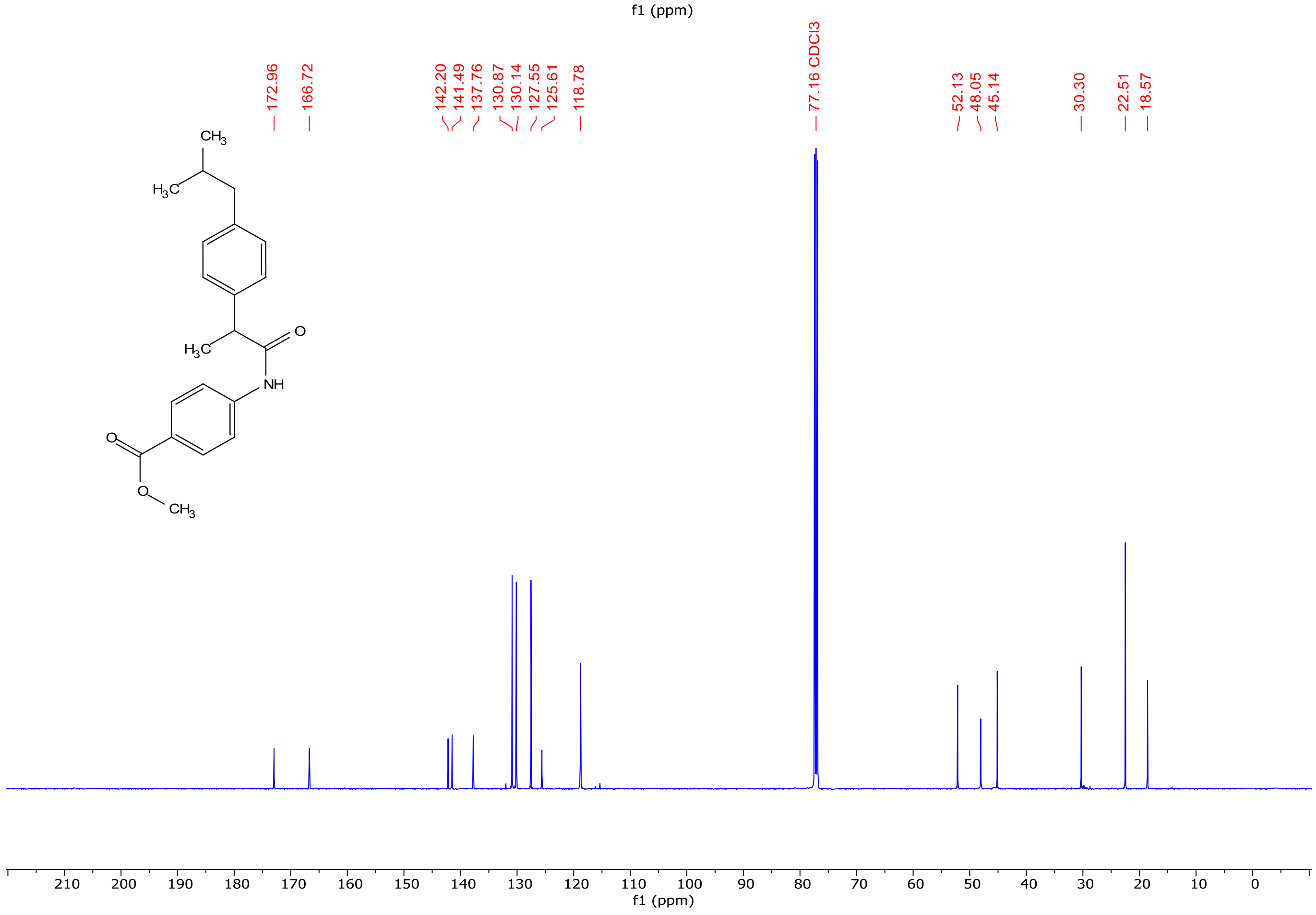

methyl 4-(4-(N,N-dipropylsulfamoyl)benzamido)benzoate (131)

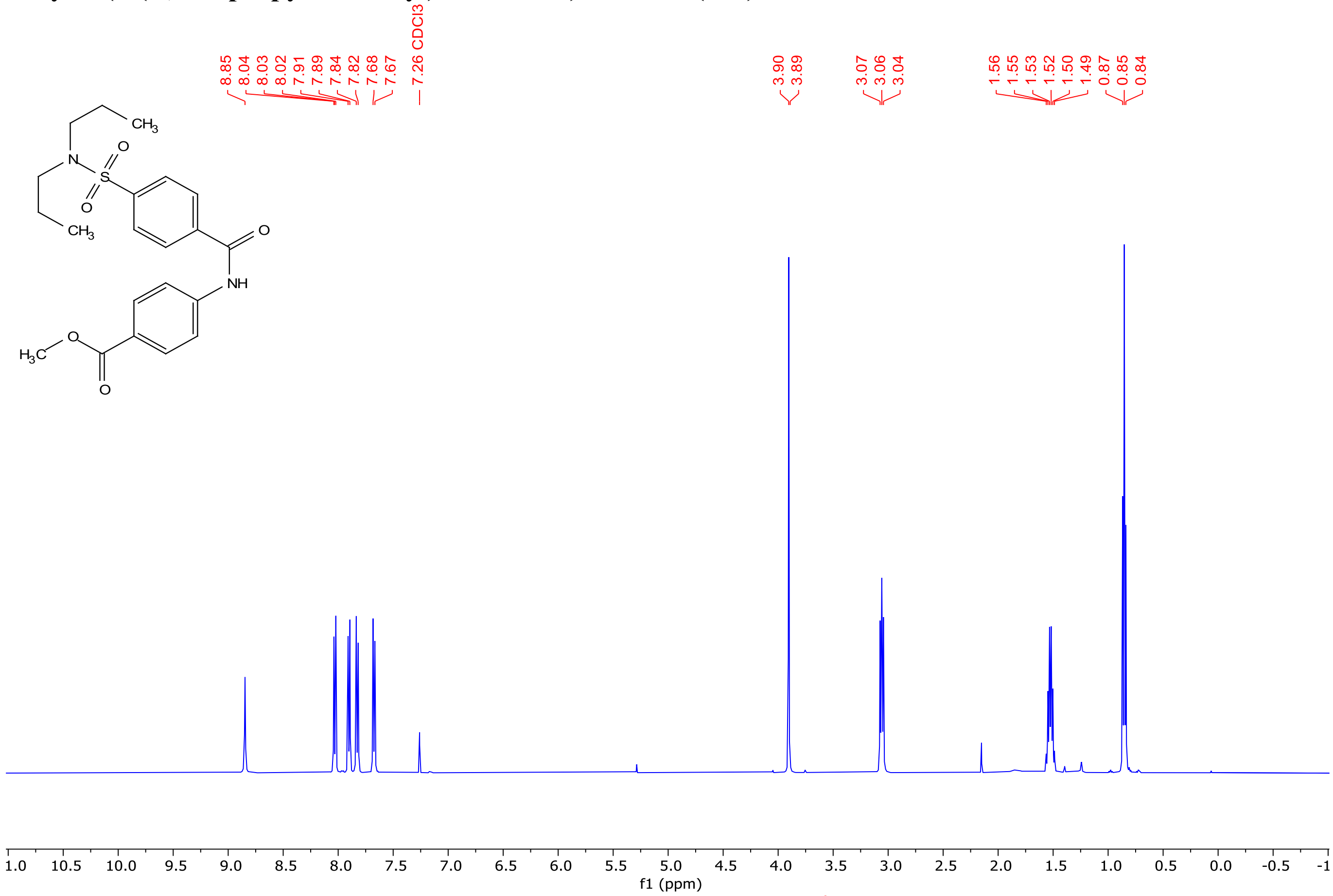

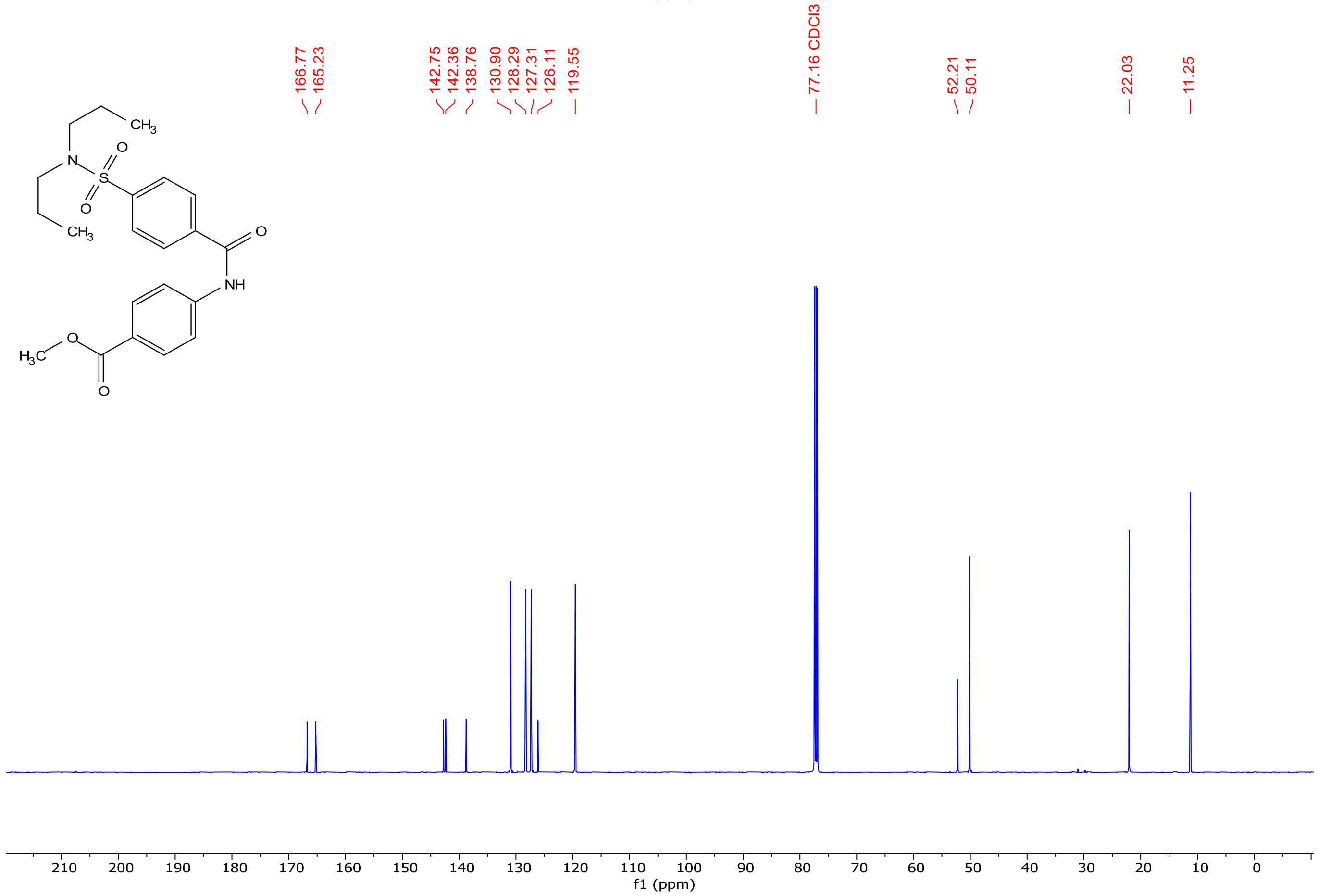

methyl 4-((4-(5-(p-tolyl)-3-(trifluoromethyl)-1*H*-pyrazol-1-yl)phenyl)sulfonamido)benzoate (132)

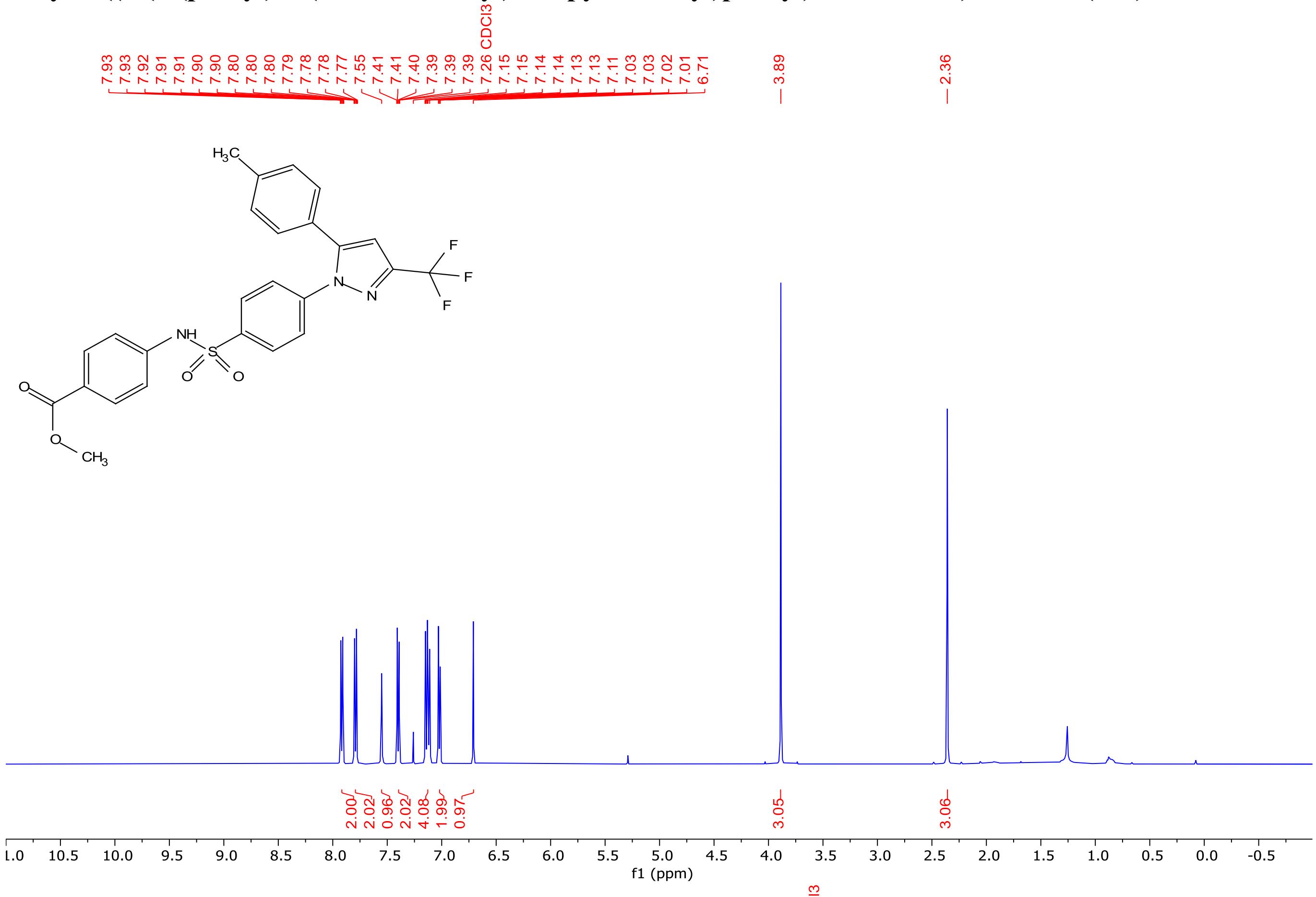

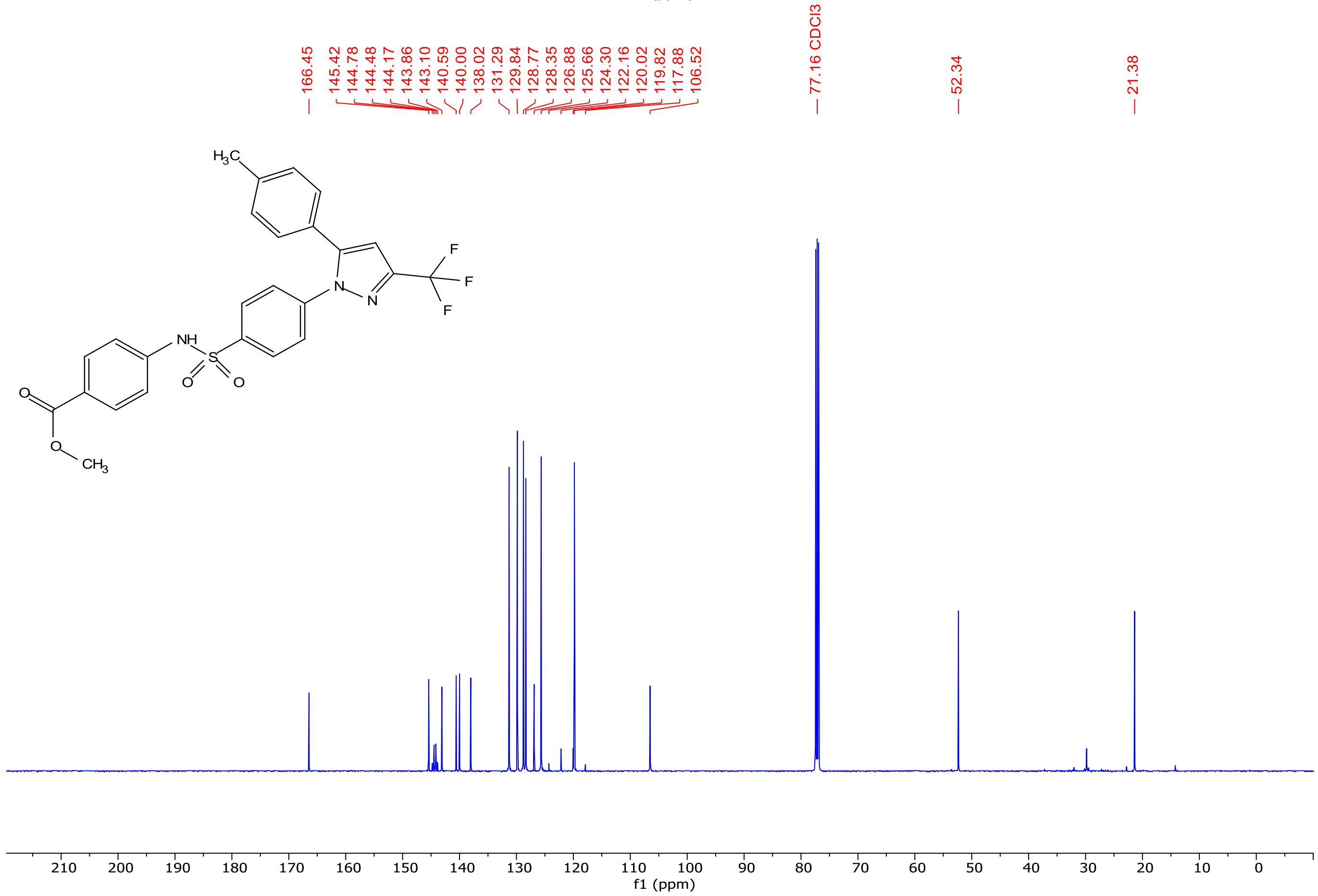

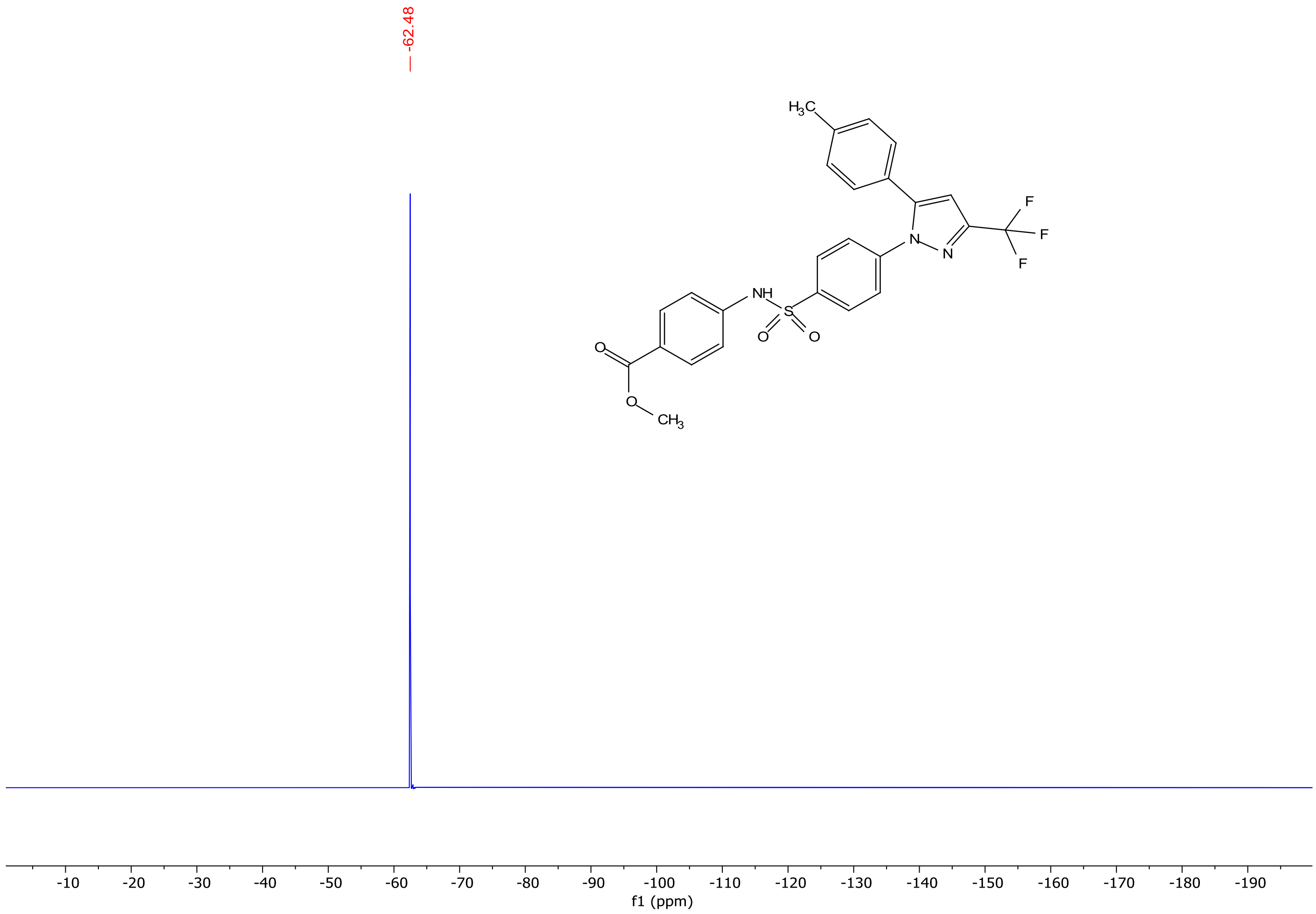
-62.48
H3C
F
F
F
N
N
NH
S
O
O
O
O
CH3
-10
-20
-30
-40
-50
-60
-70
-80
-90
-100
-110
-120
-130
-140
-150
-160
-170
-180
-190
f1 (ppm)

((5S,5aR,8aR,8bS)-2,2,7,7-tetramethyltetrahydro-5*H*-bis([1,3]dioxolo)[4,5-b:4',5'-d]pyran-5-yl)methyl 4-((4-methylphenyl)sulfonamido)benzoate (133)

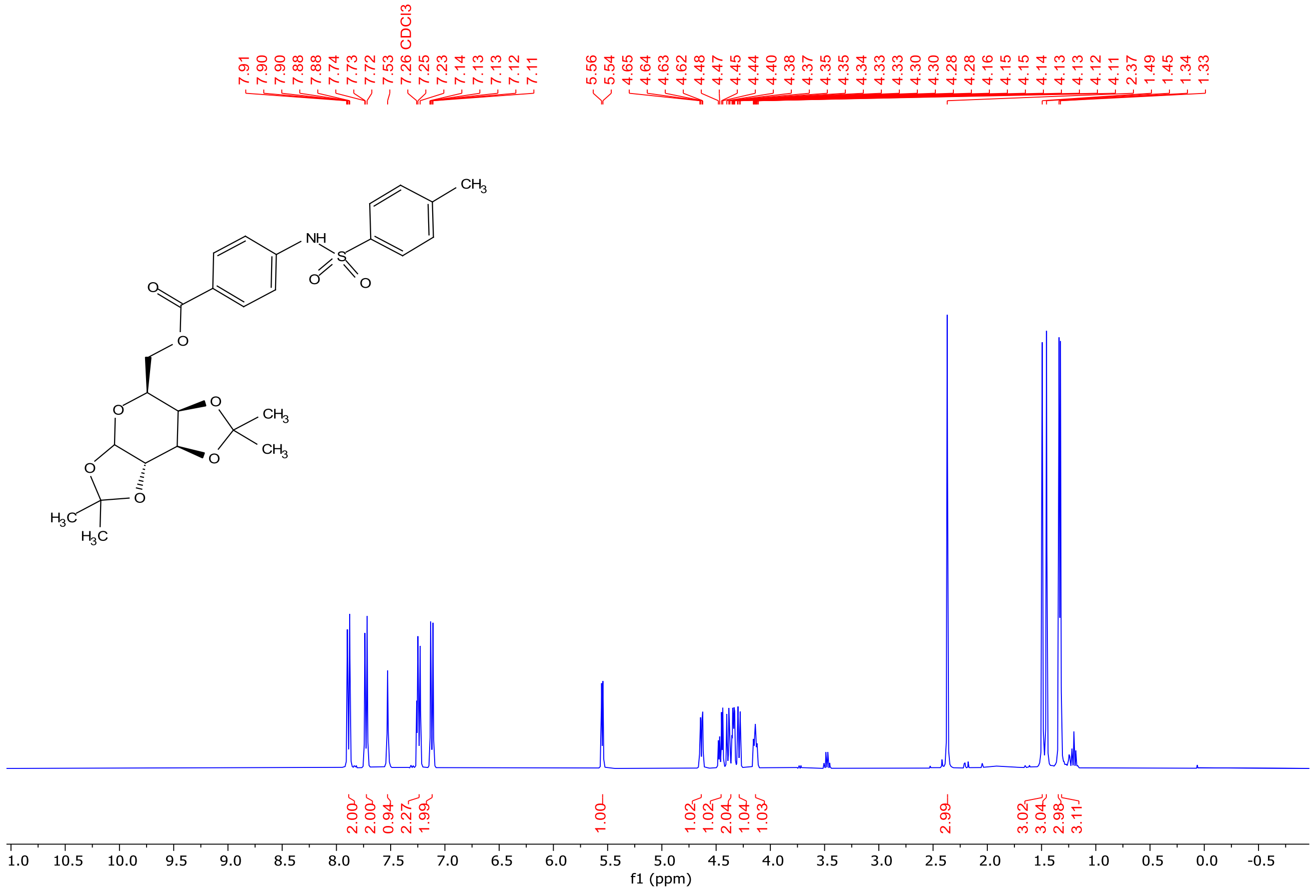

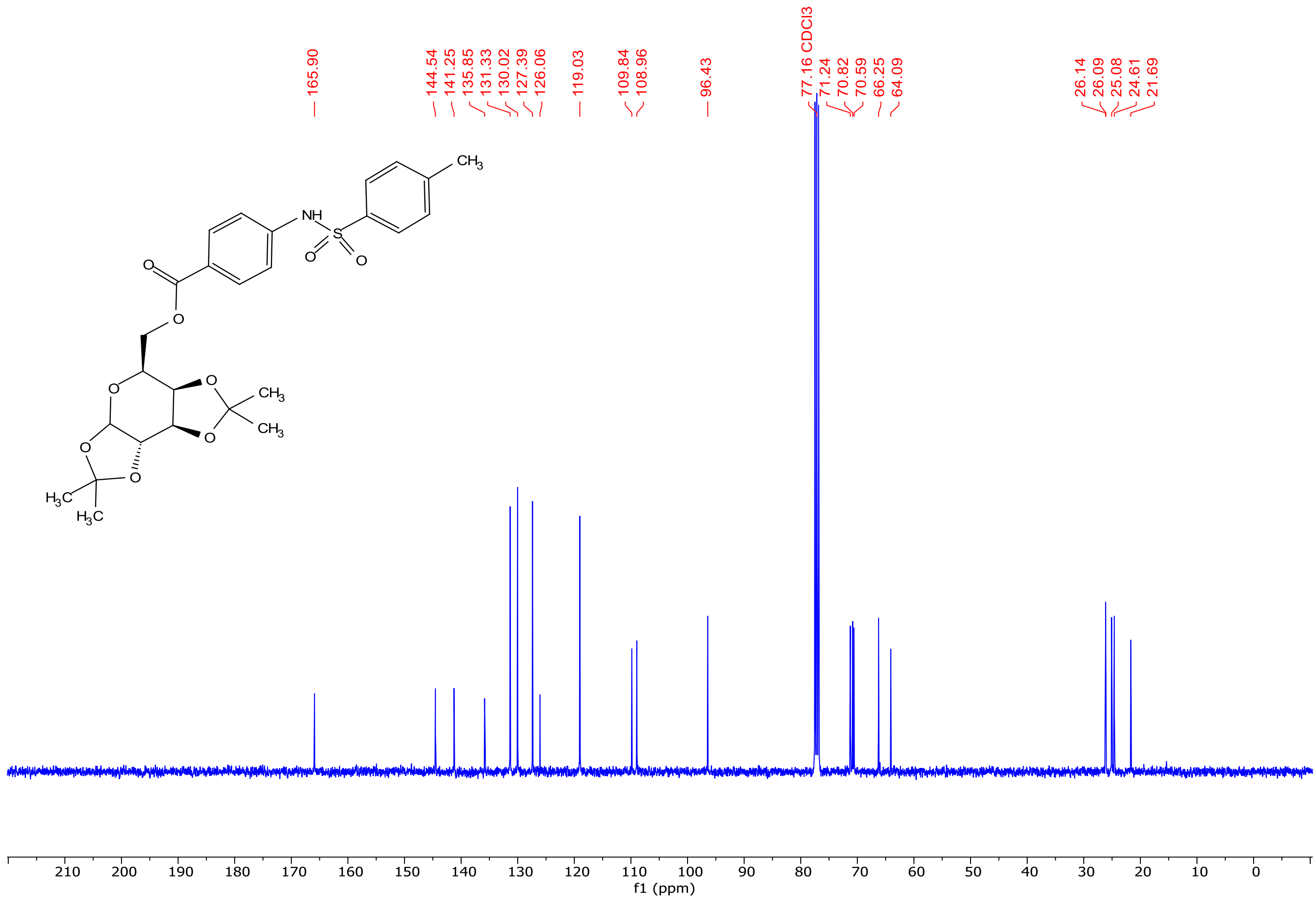

4-(((5R,5aS,8aS,8bR)-2,2,7,7-tetramethyltetrahydro-5*H*-bis([1,3]dioxolo)[4,5-b:4',5'-d]pyran-5-yl)methoxy)benzonitrile (134)

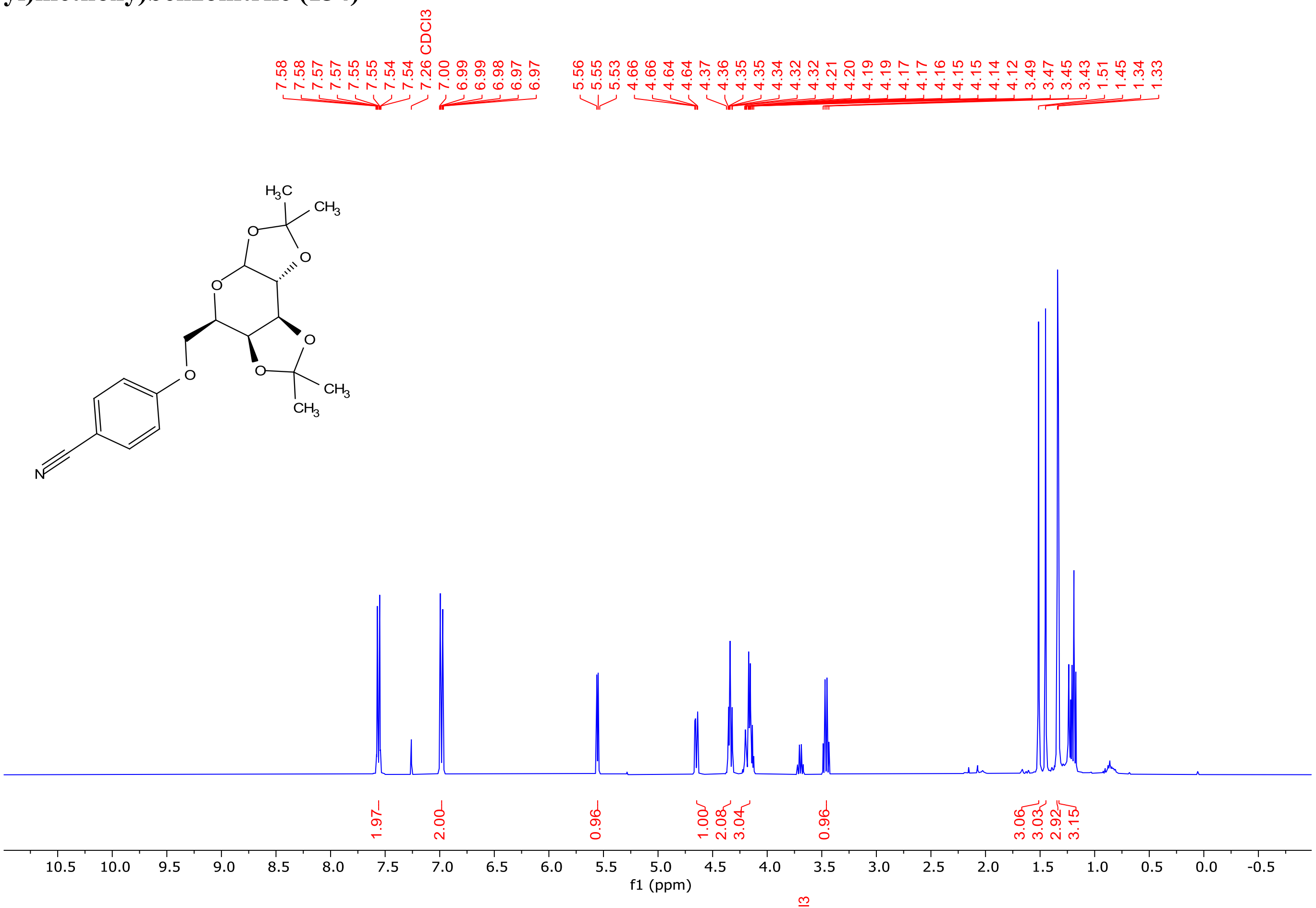

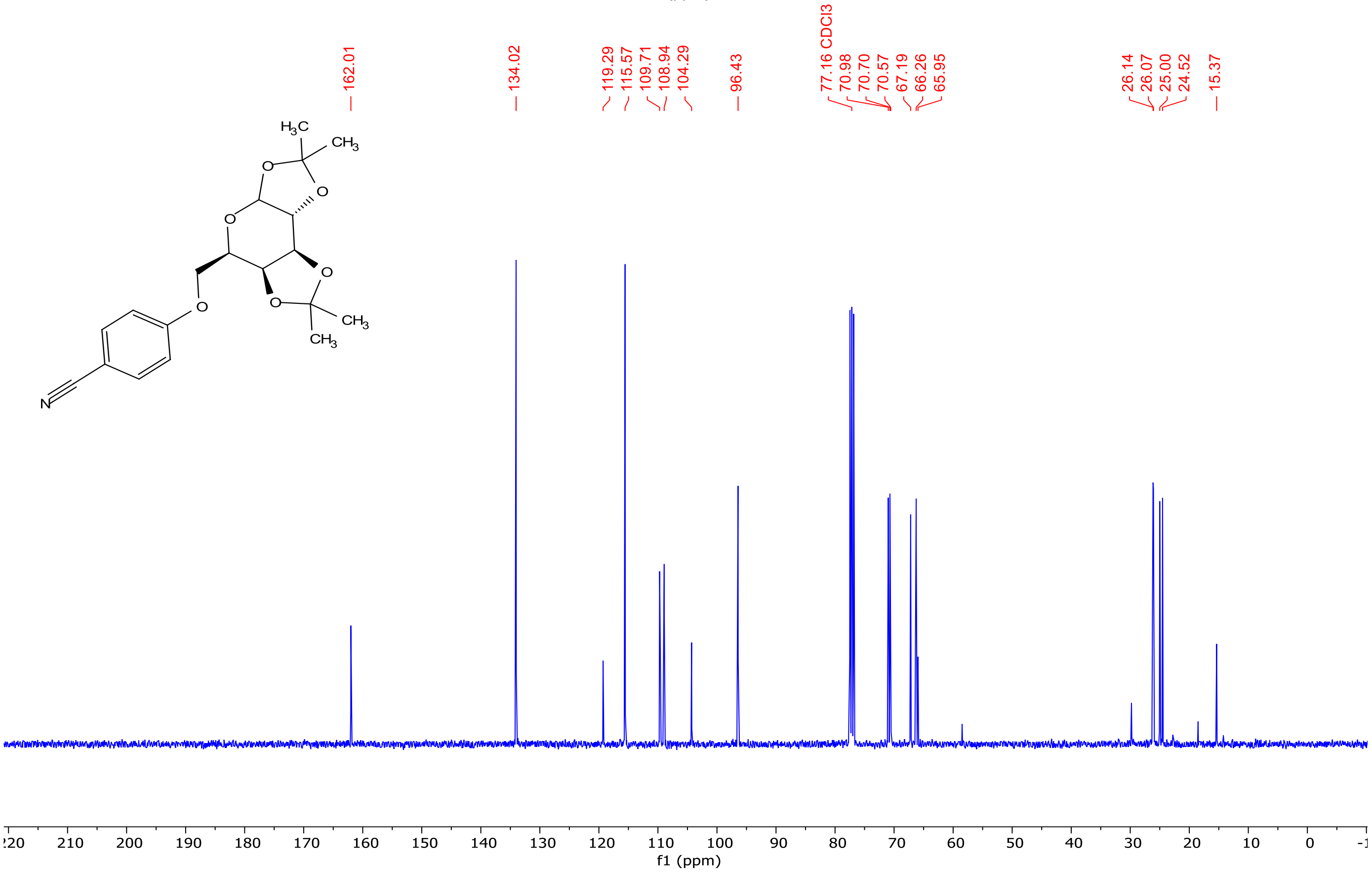

4-cyanophenyl (S)-2-(6-methoxynaphthalen-2-yl)propanoate (135)

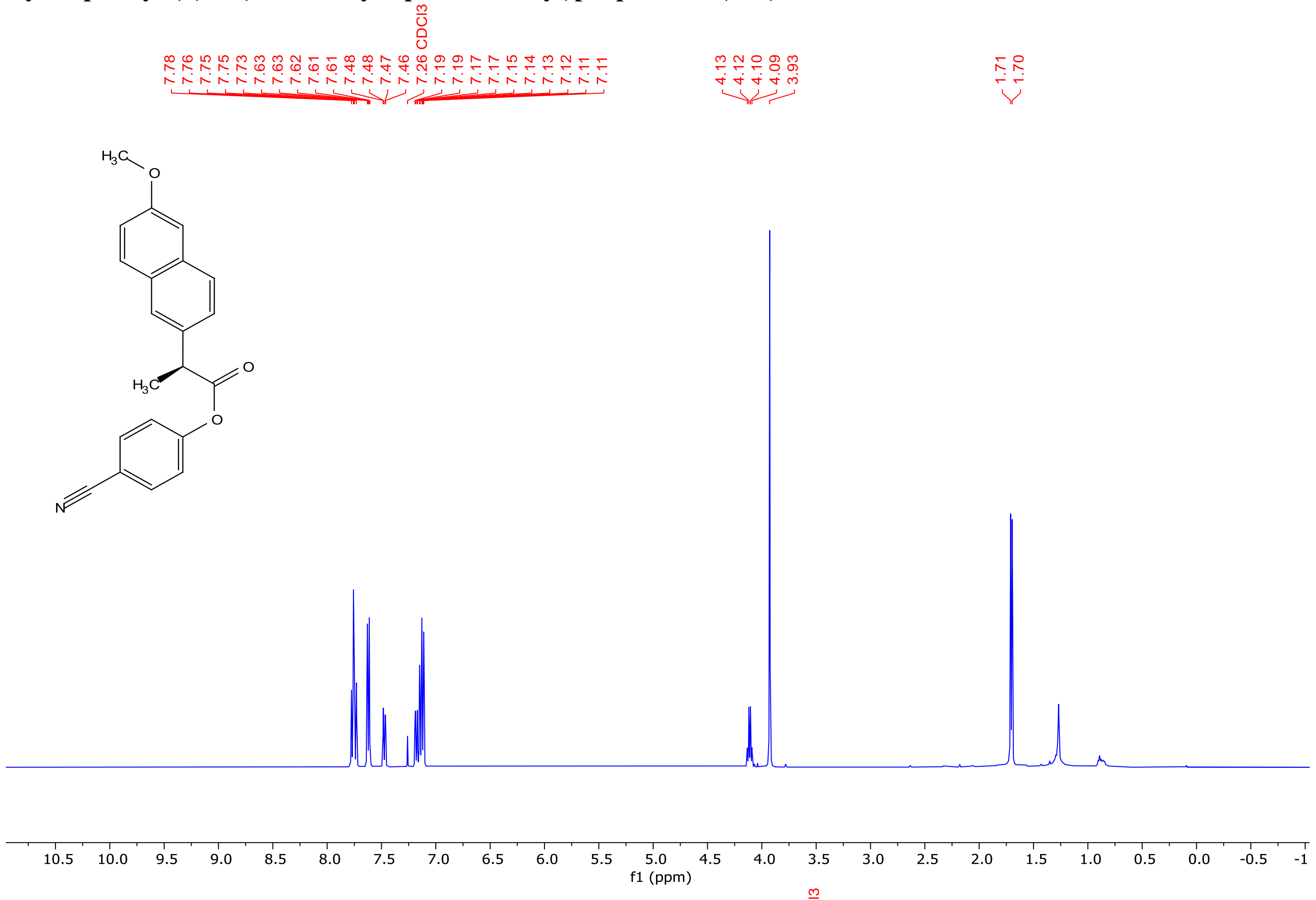

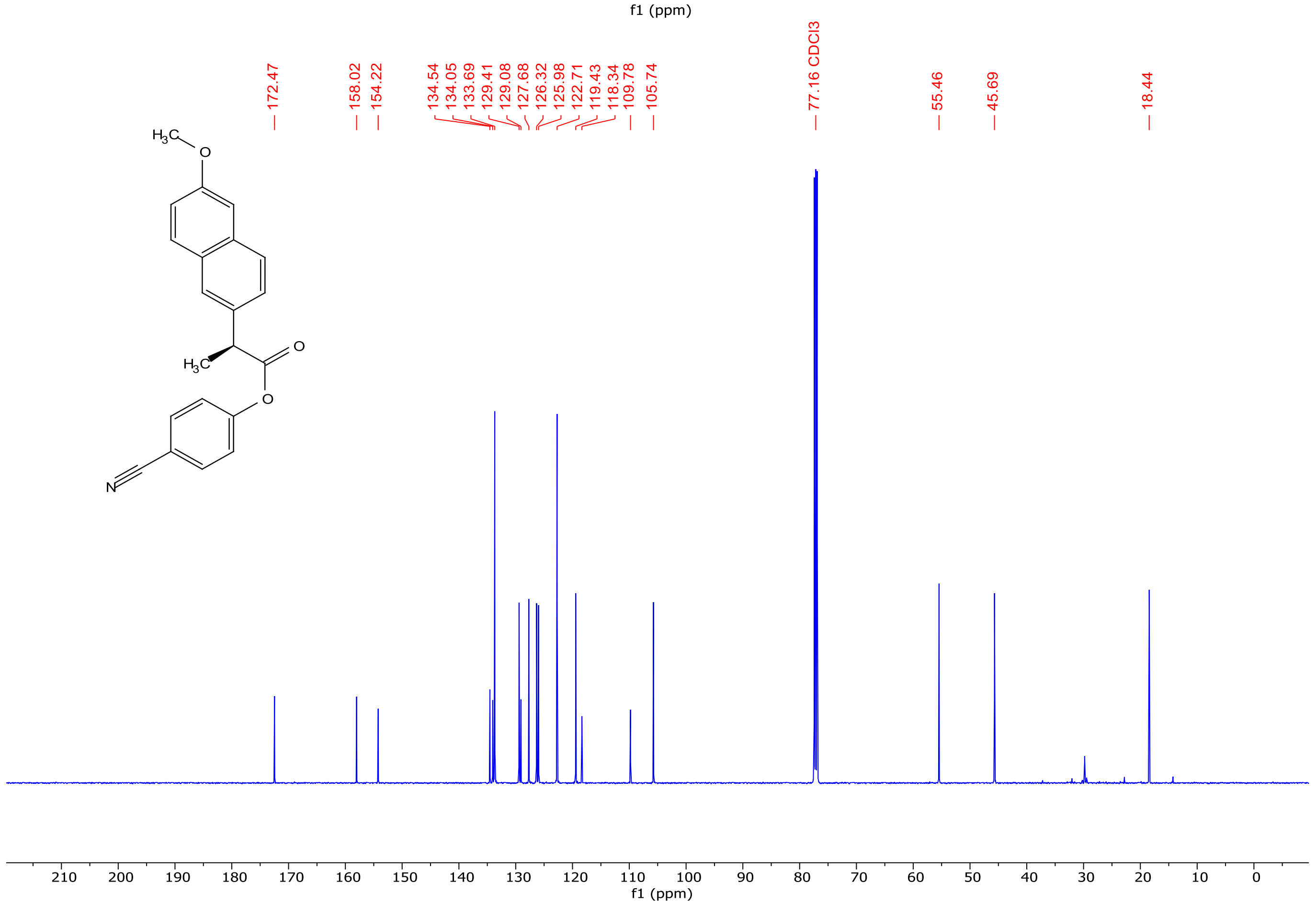

4-cyanophenyl 2-(4-chlorophenoxy)-2-methylpropanoate (136)

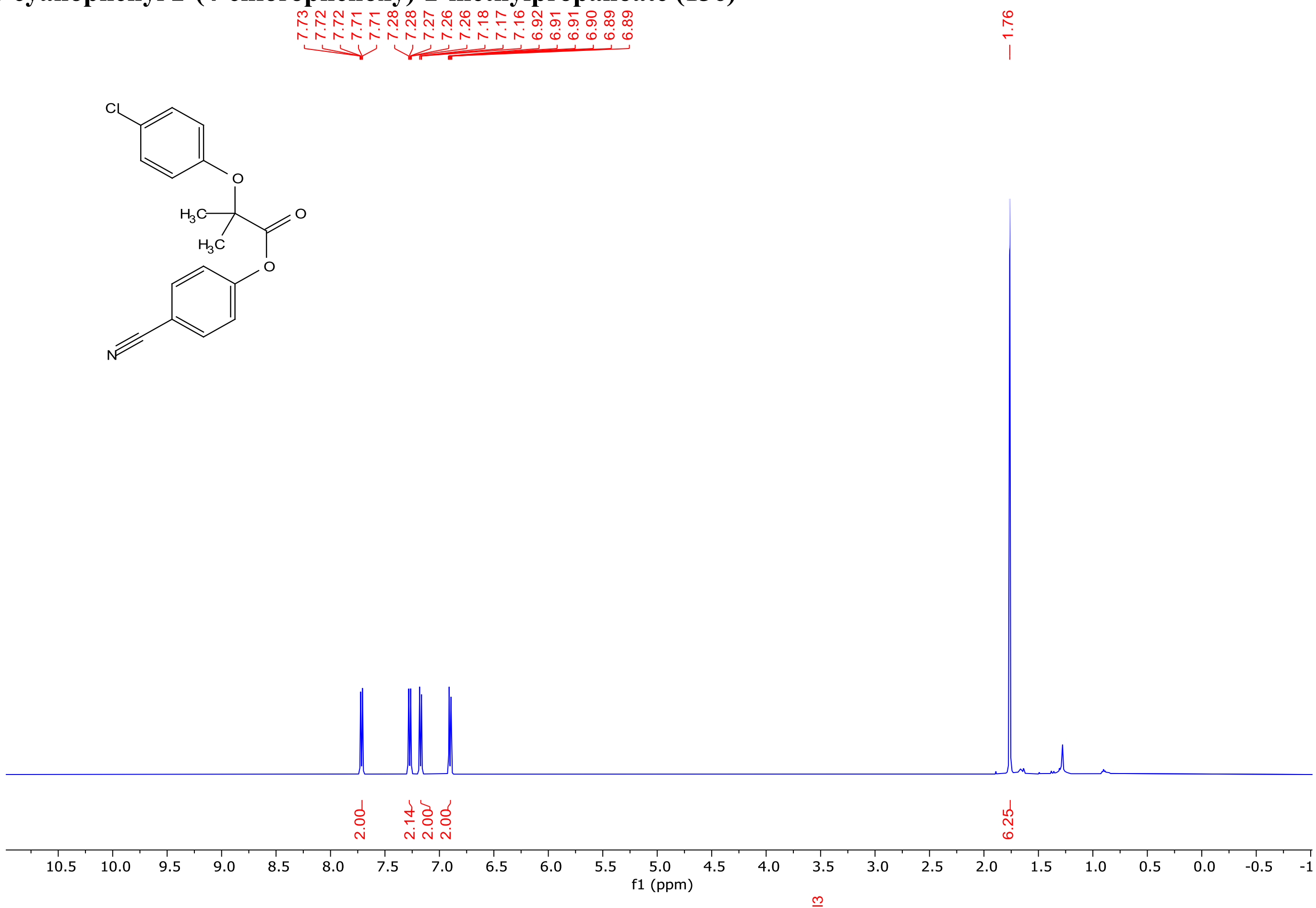

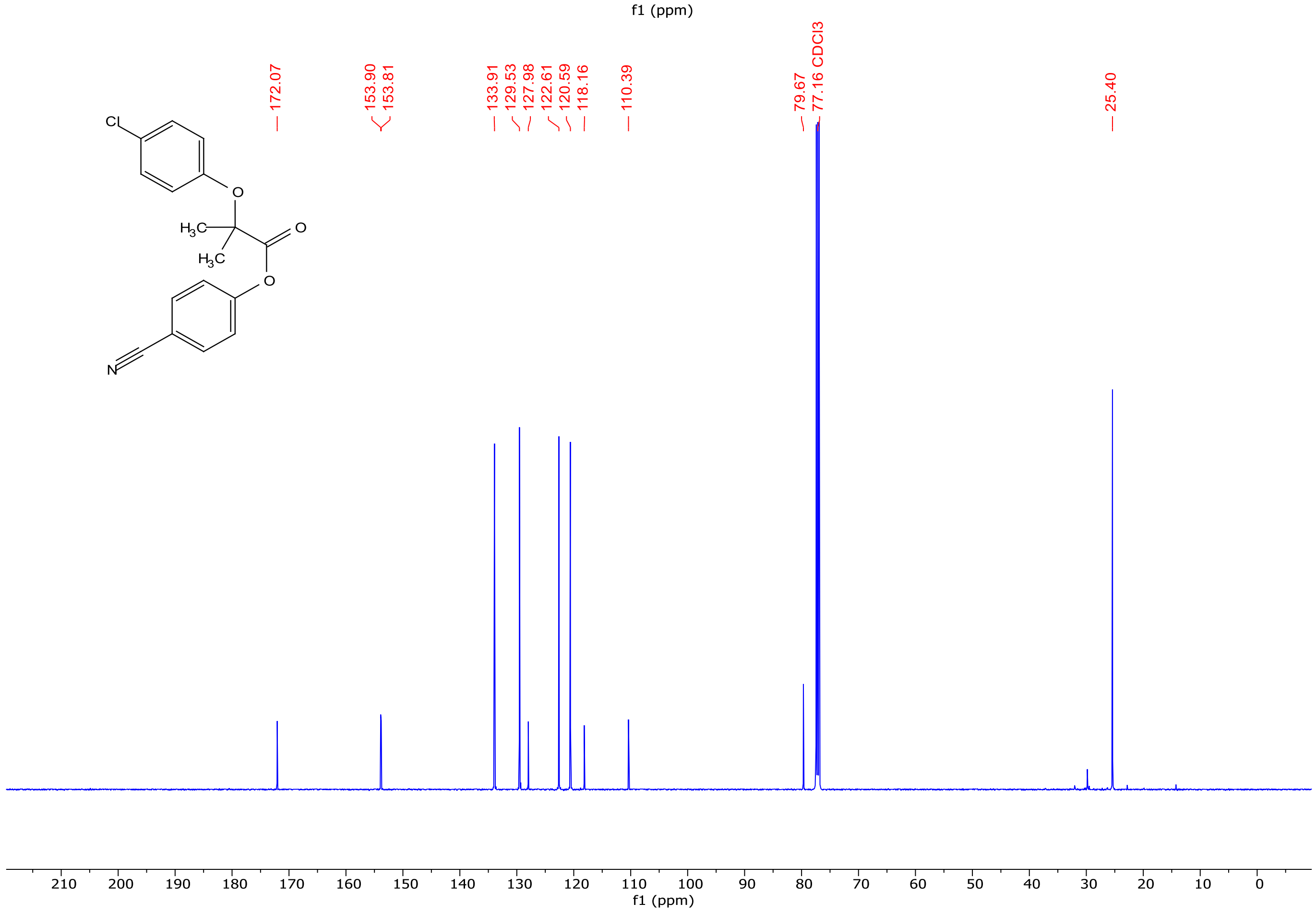

1,3,7-trimethyl-8-(phenylthio)-3,7-dihydro-1*H*-purine-2,6-dione (137)

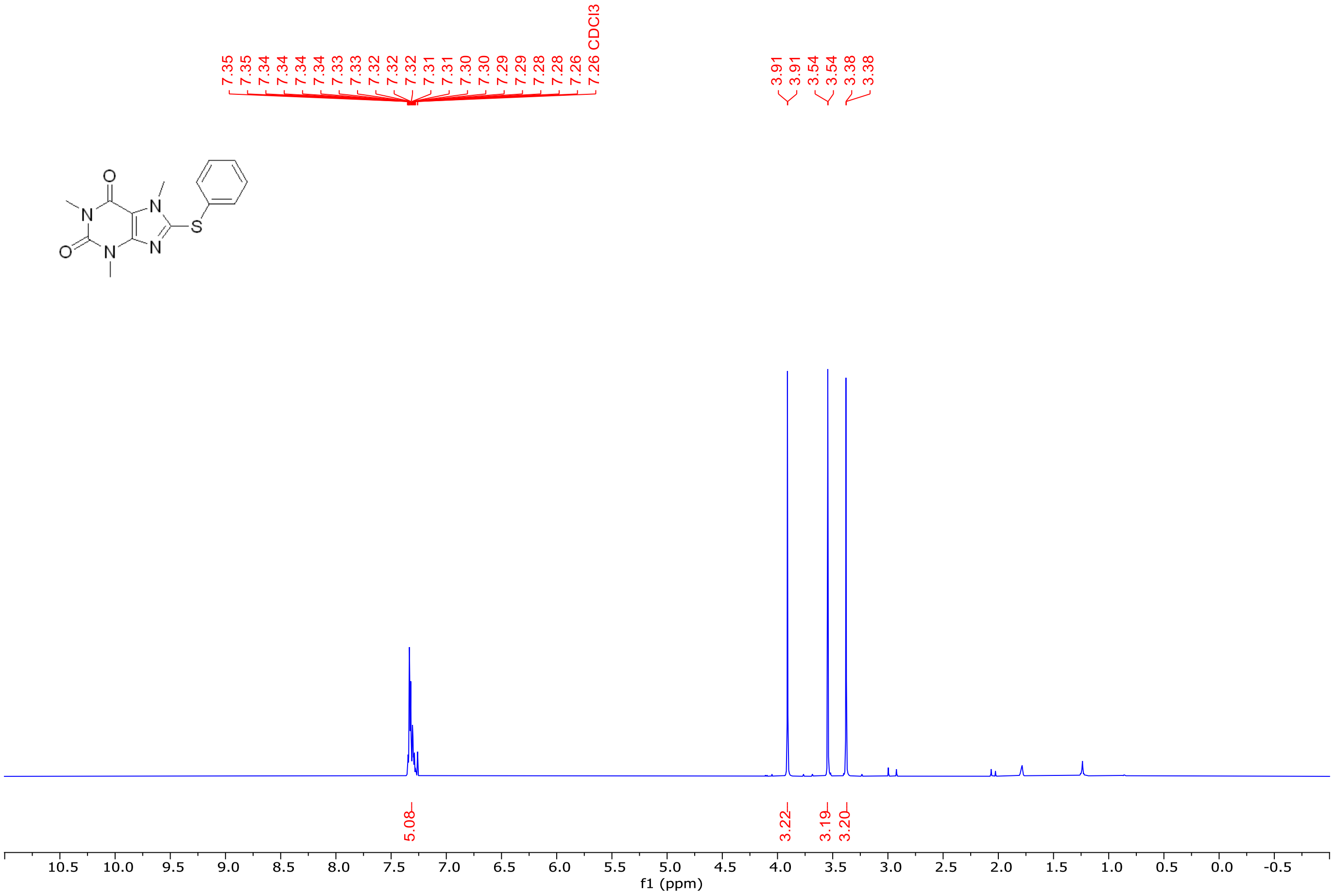

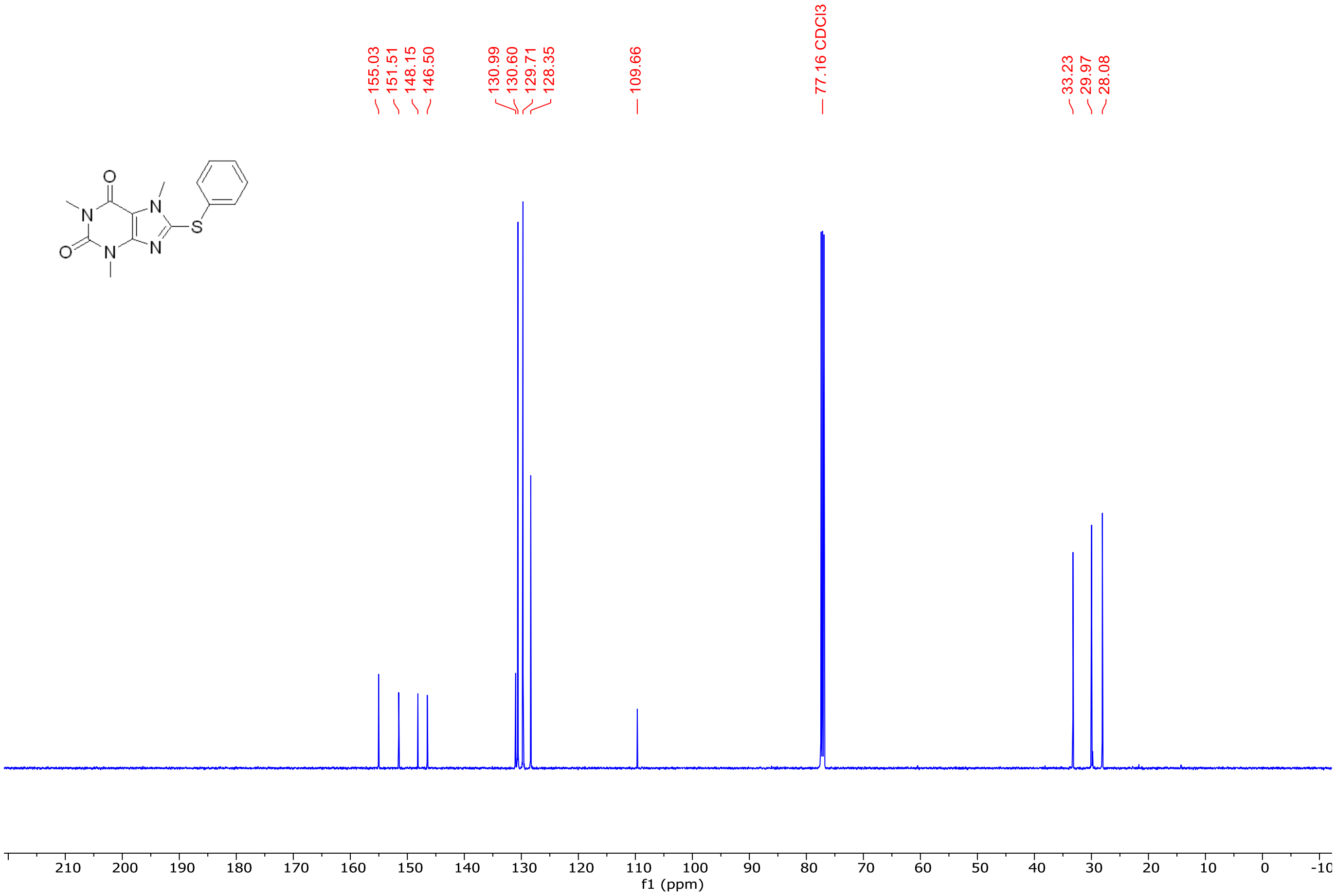

((5R,5aS,8aS,8bR)-2,2,7,7-tetramethyltetrahydro-5*H*-bis([1,3]dioxolo)[4,5-b:4',5'-d]pyran-5-yl)methyl 4-(phenylthio)benzoate (138)

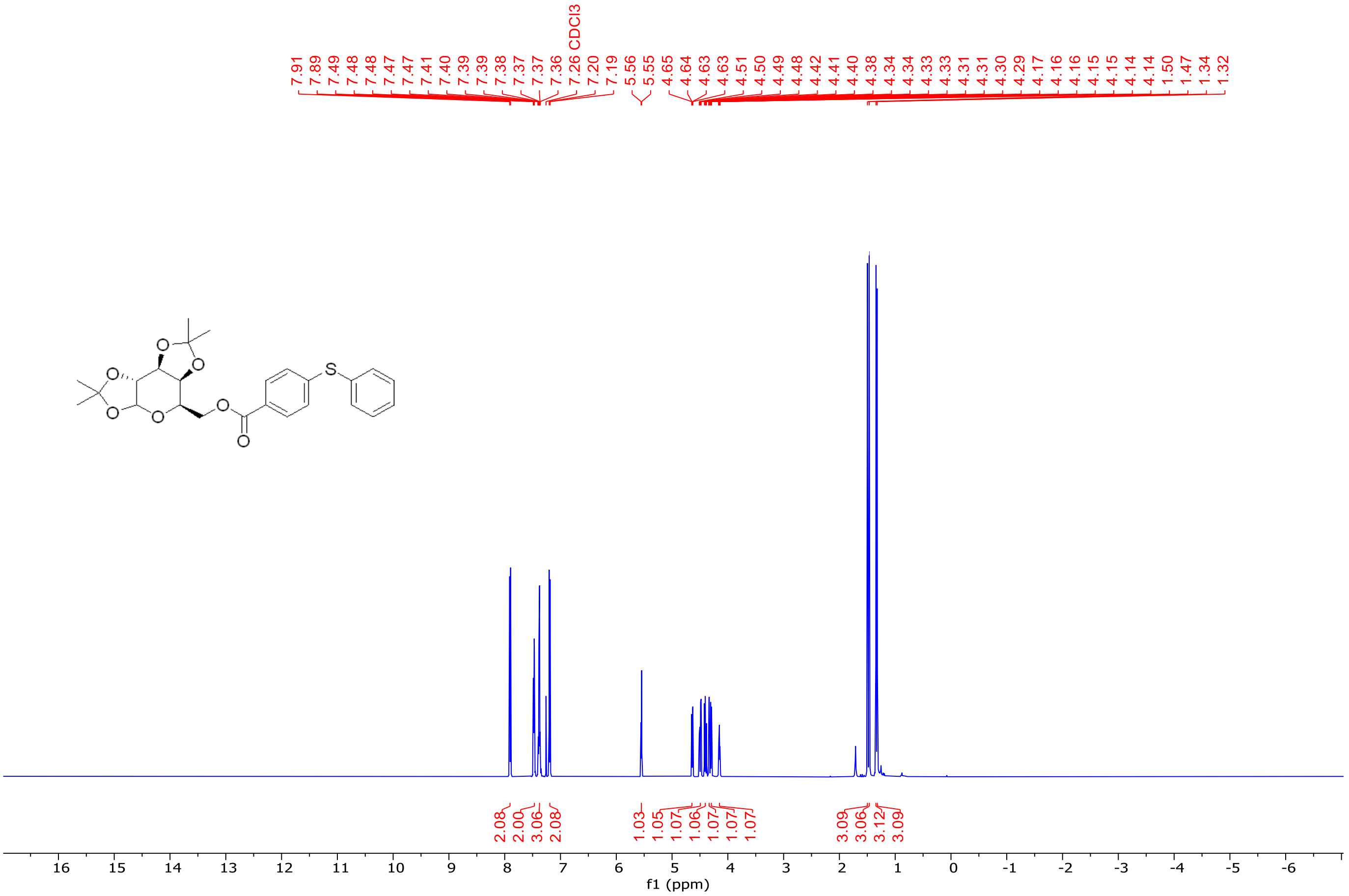

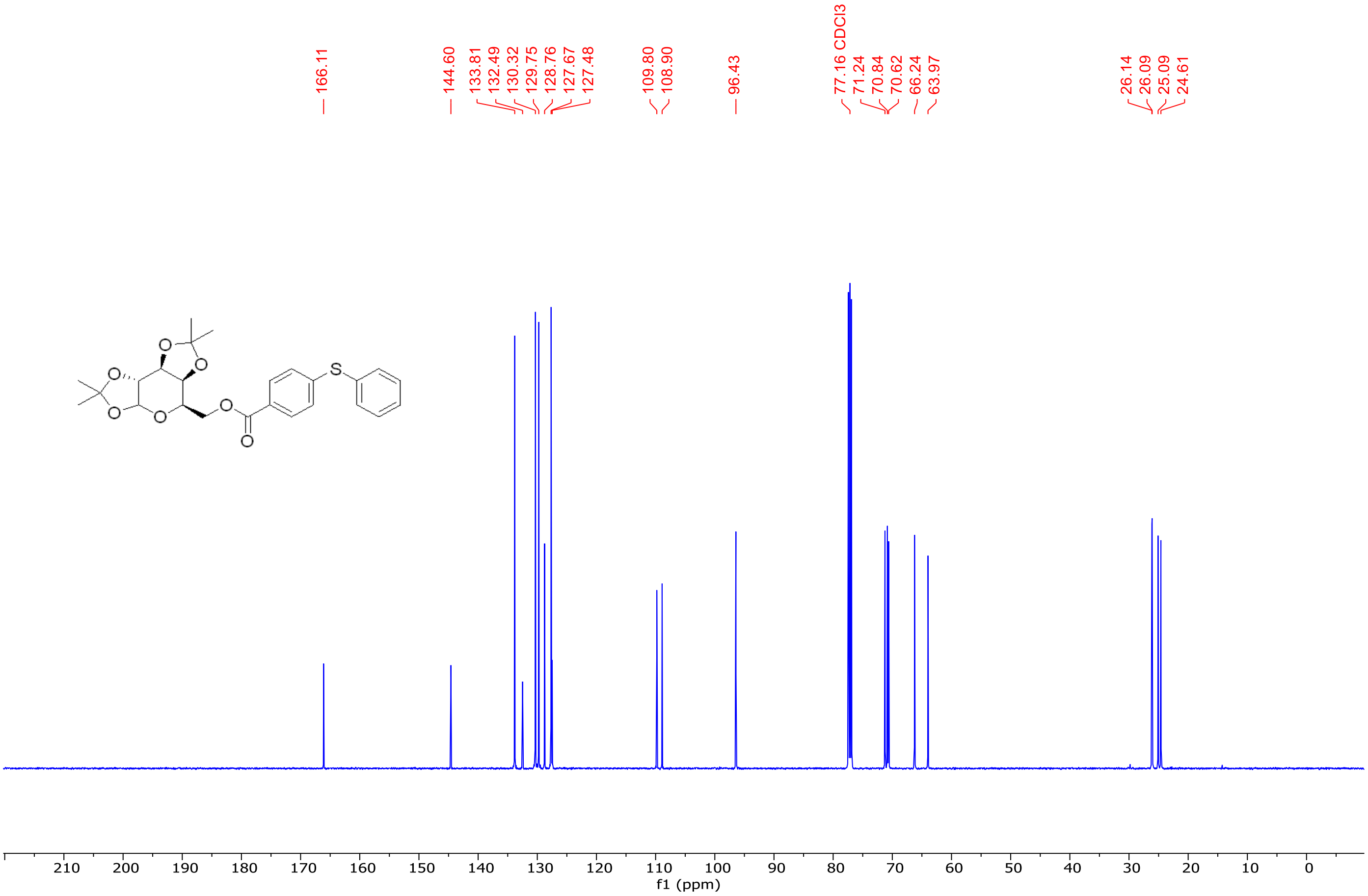

4-((3-methoxyphenyl)thio)phenyl adamantane-1-carboxylate(139)

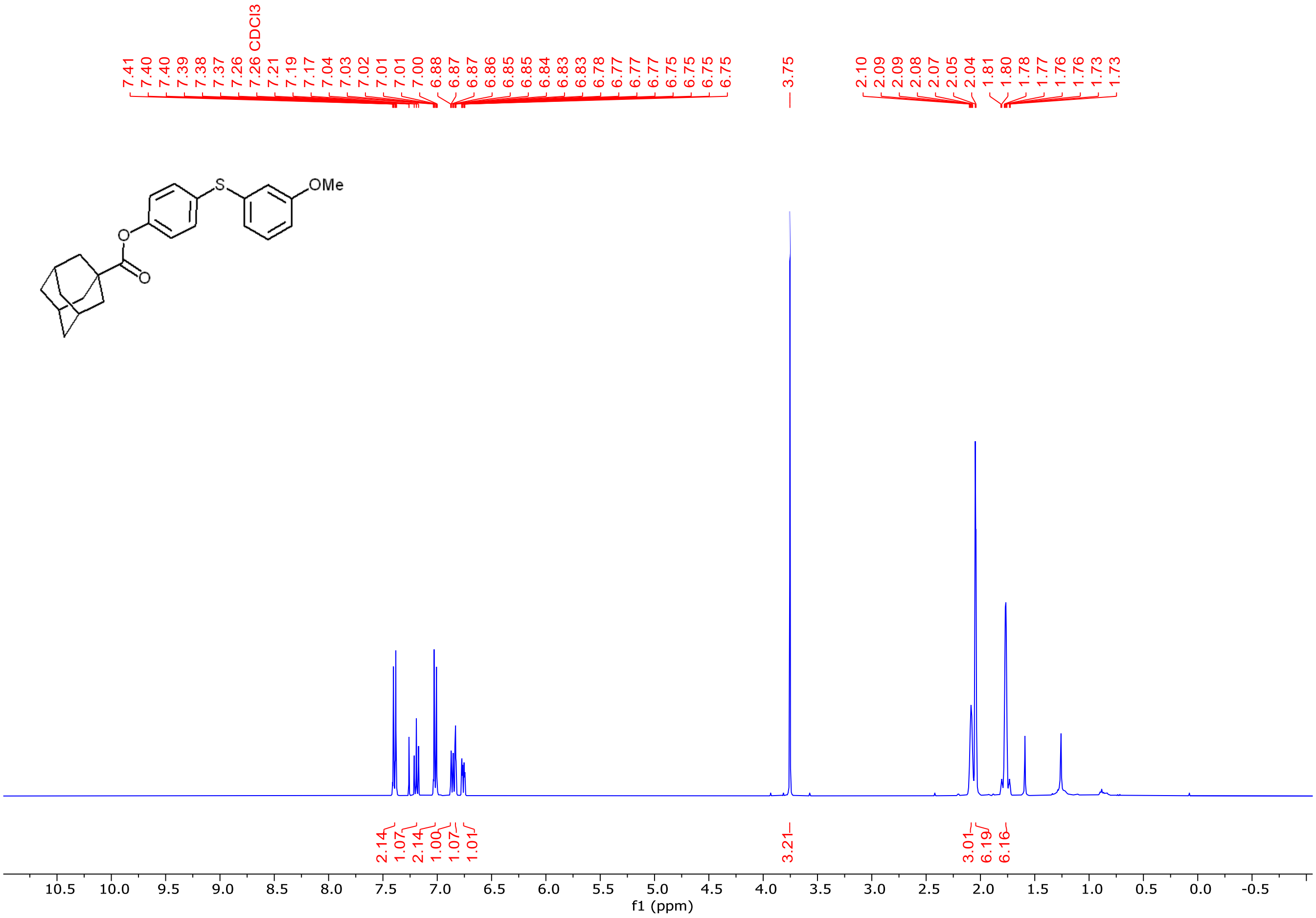

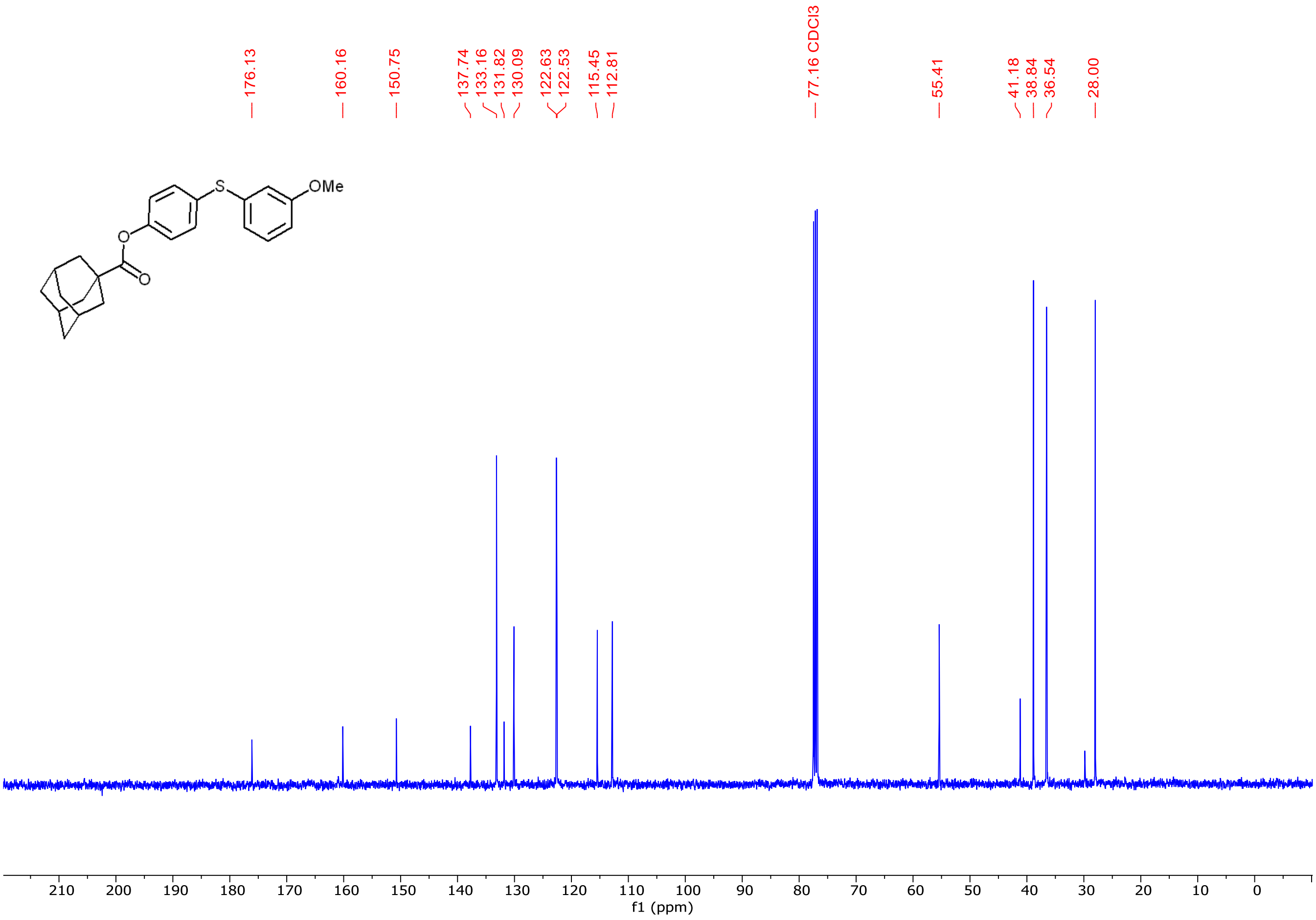

methyl N-(*tert*-butoxycarbonyl)-S-(4-cyanophenyl)-L-cysteinate(140)

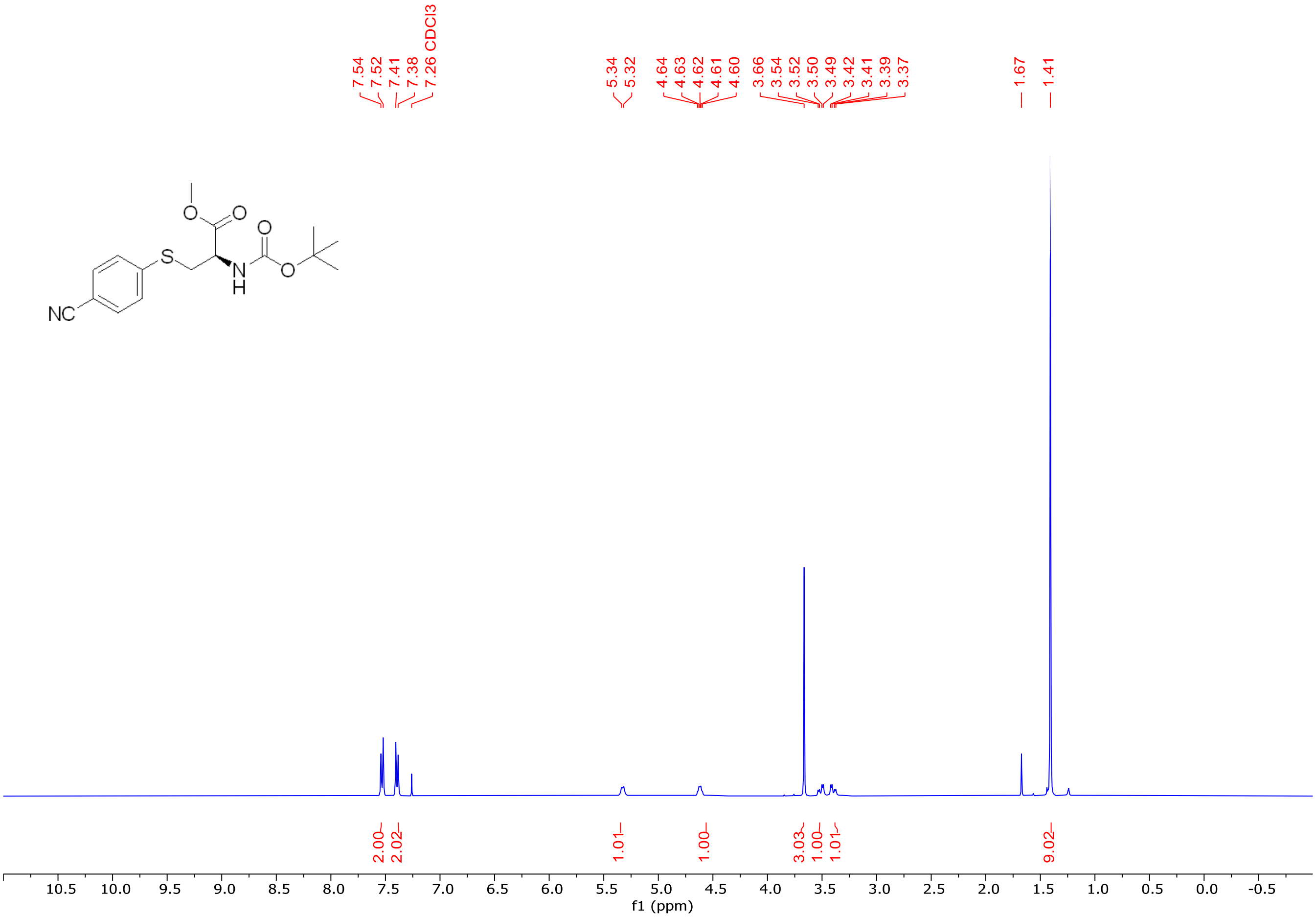

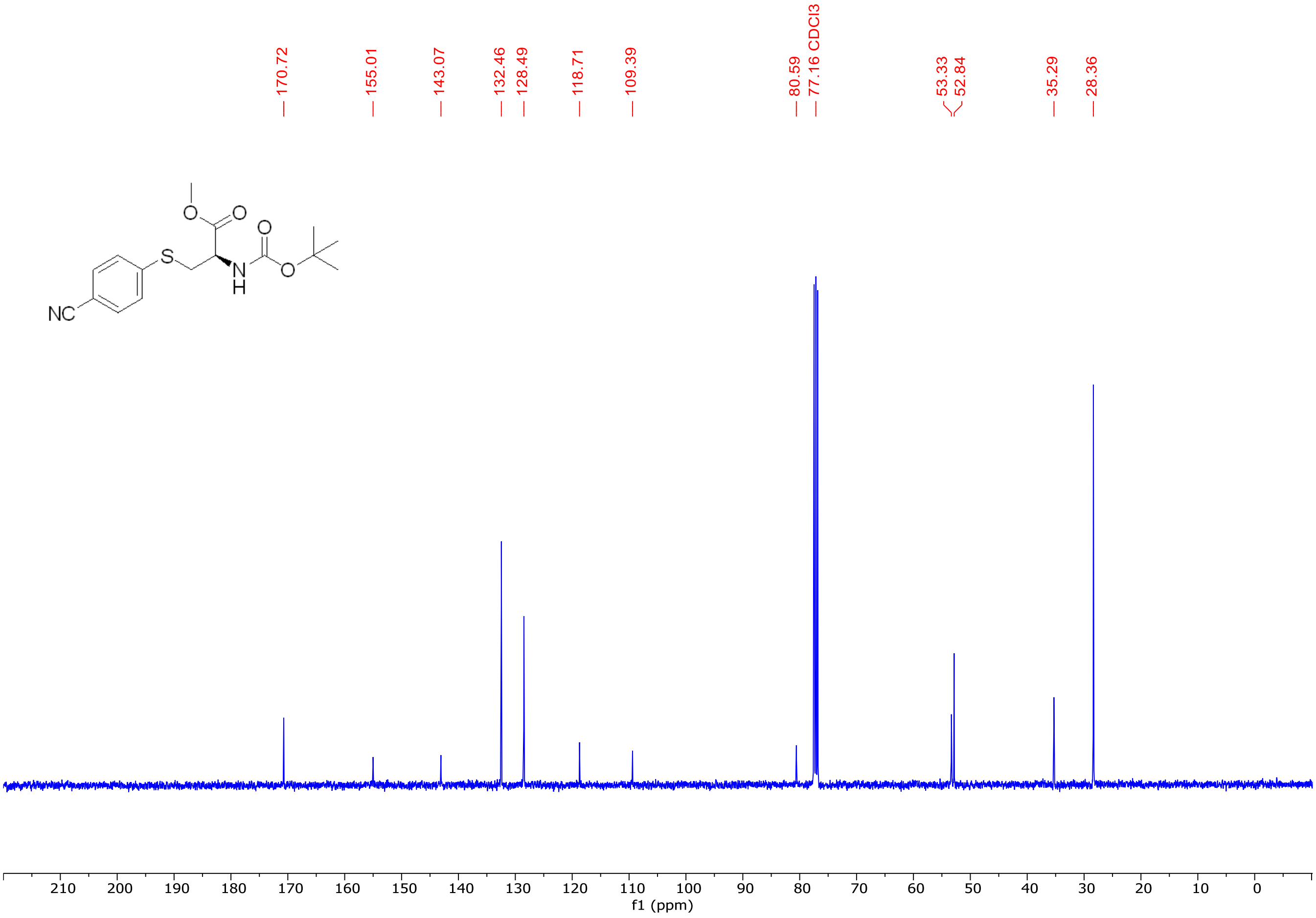

methyl N-(*tert*-butoxycarbonyl)-S-(4-(trifluoromethyl)phenyl)-L-cysteinate (141)

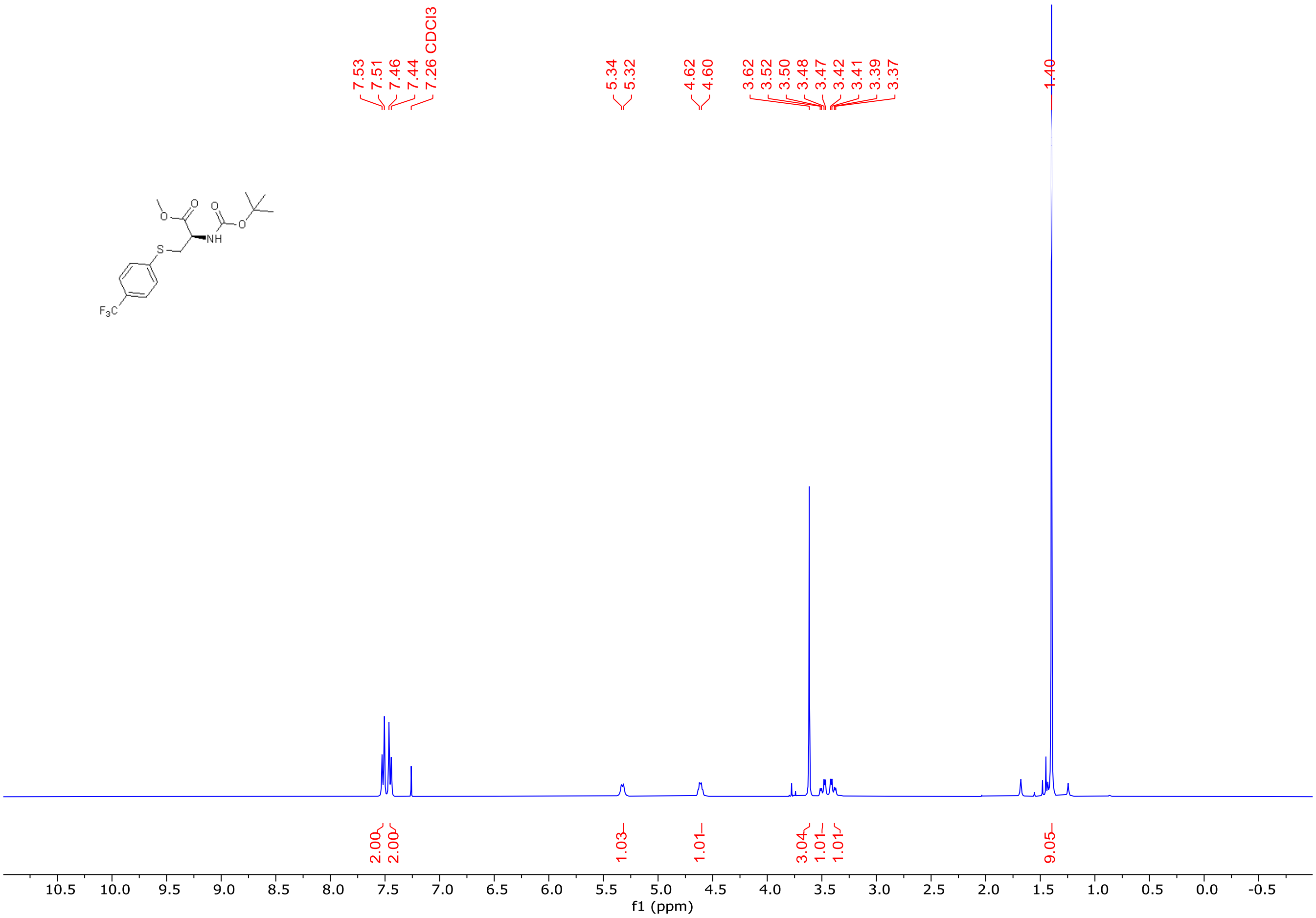

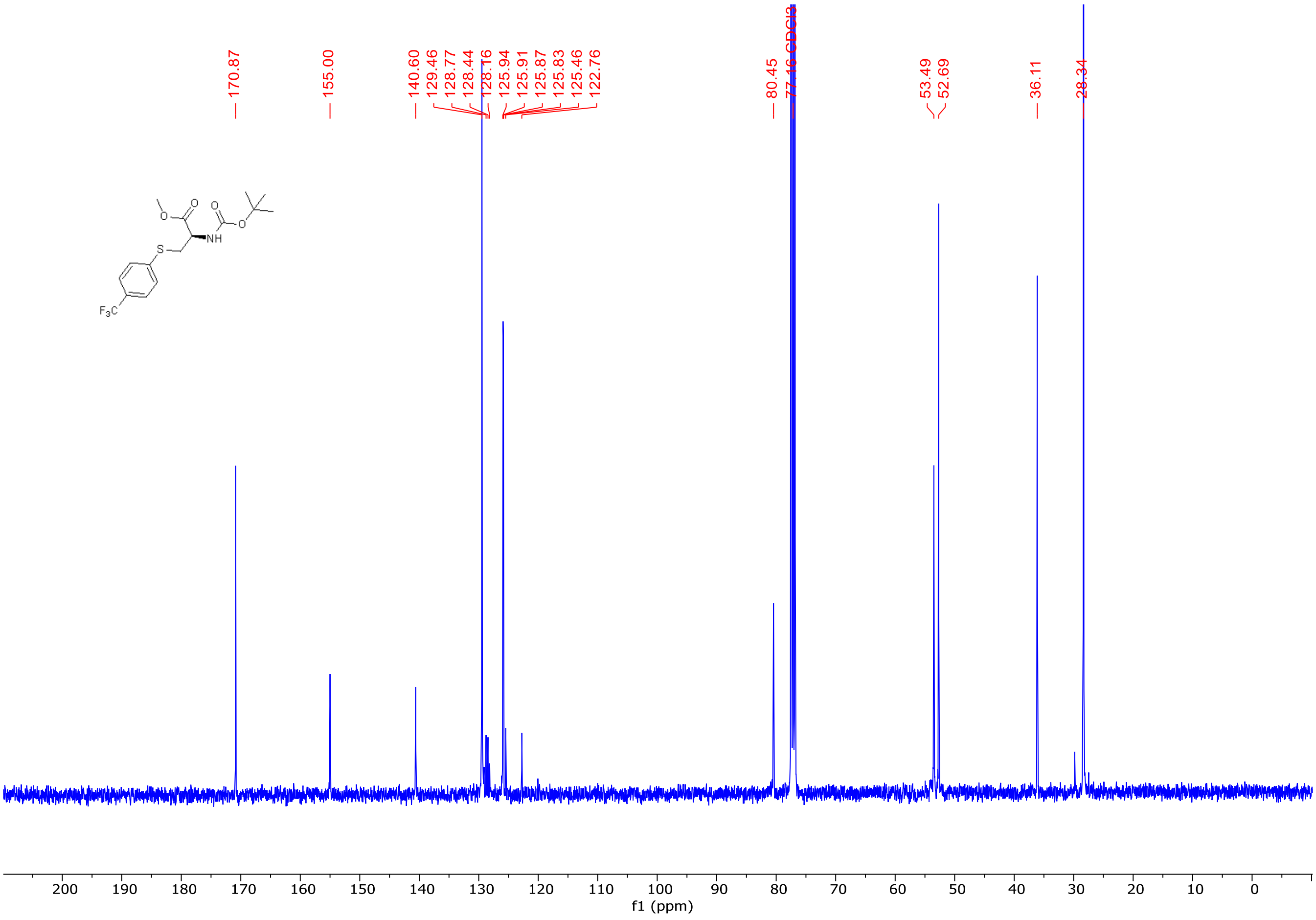

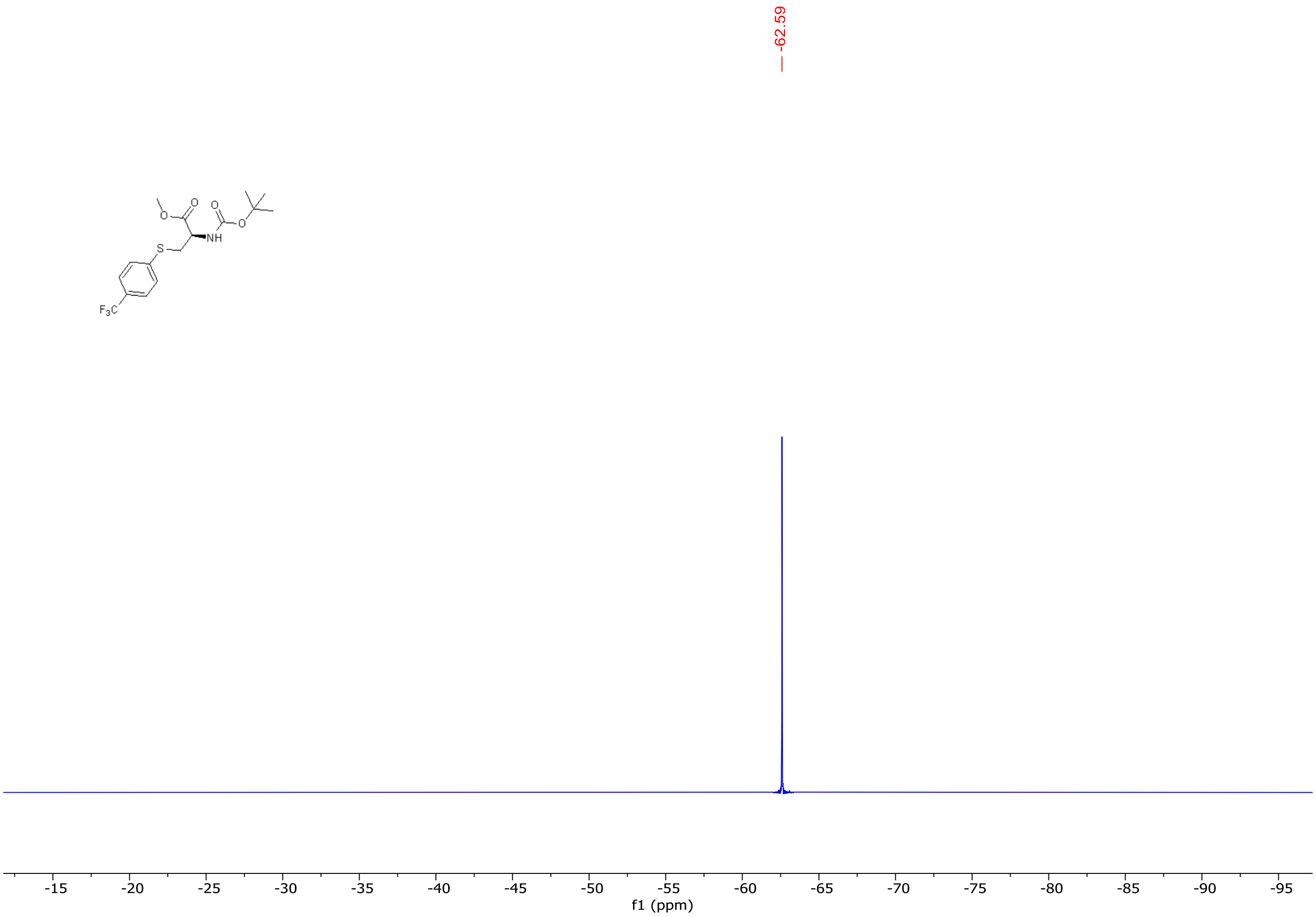
-62.59
f1 (ppm)
-15 -20 -25 -30 -35 -40 -45 -50 -55 -60 -65 -70 -75 -80 -85 -90 -95

methyl (R)-4-((2-((*tert*-butoxycarbonyl)amino)-3-methoxy-3-oxopropyl)thio)benzoate(142)

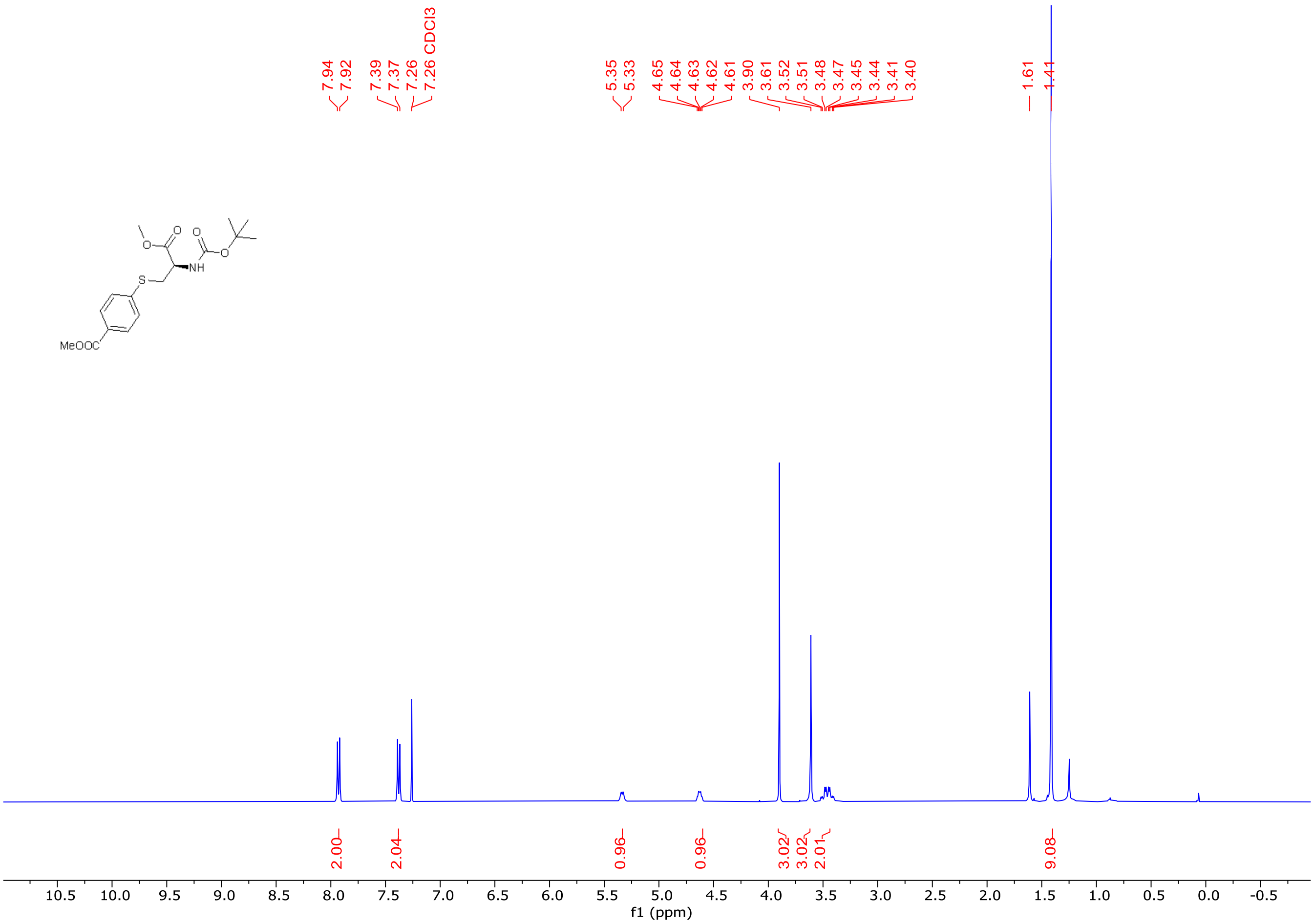

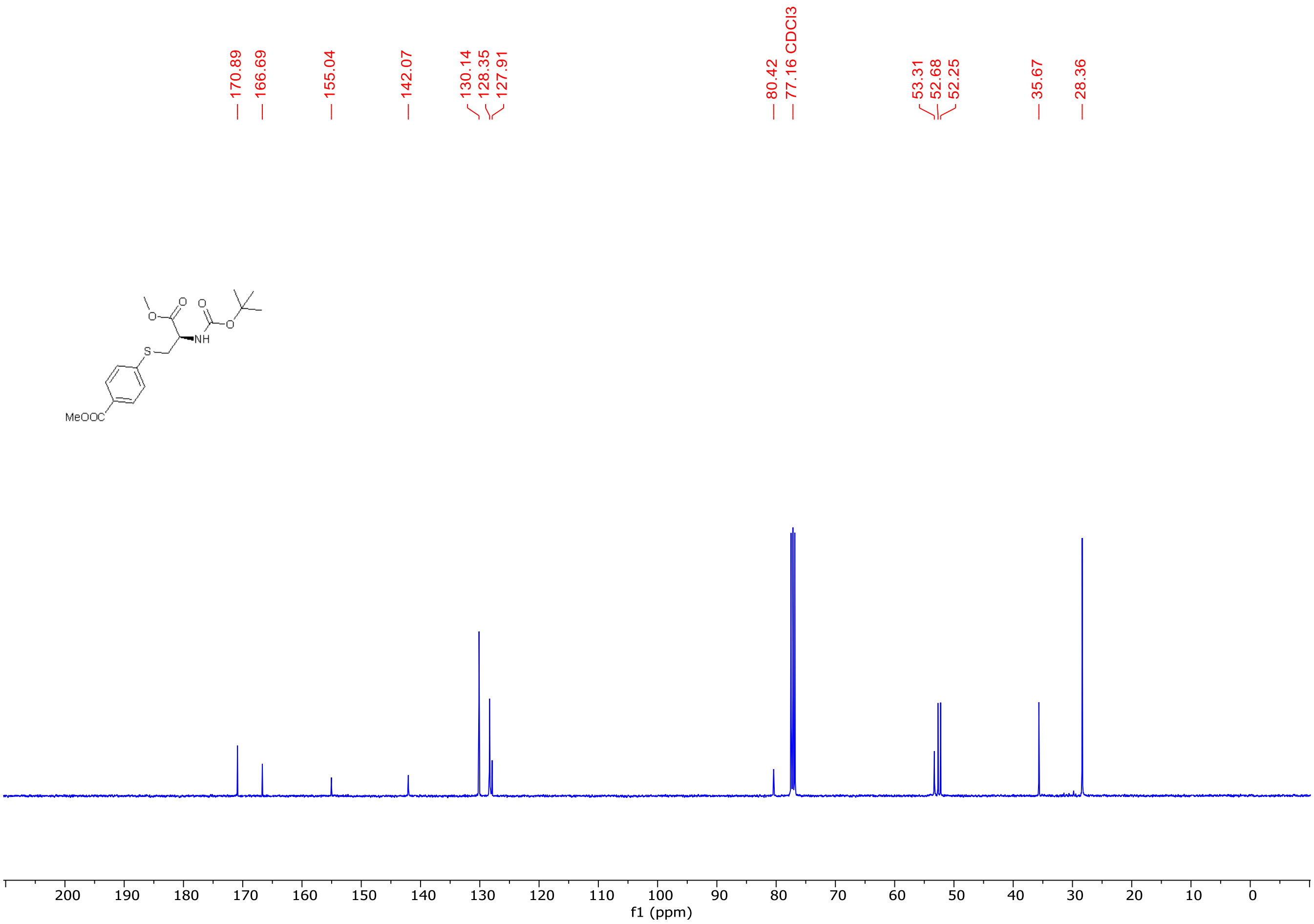

((5R,5aS,8aS,8bR)-2,2,7,7-tetramethyltetrahydro-5*H*-bis([1,3]dioxolo)[4,5-b:4',5'-d]pyran-5-yl)methyl 4-bromobenzoate (143)

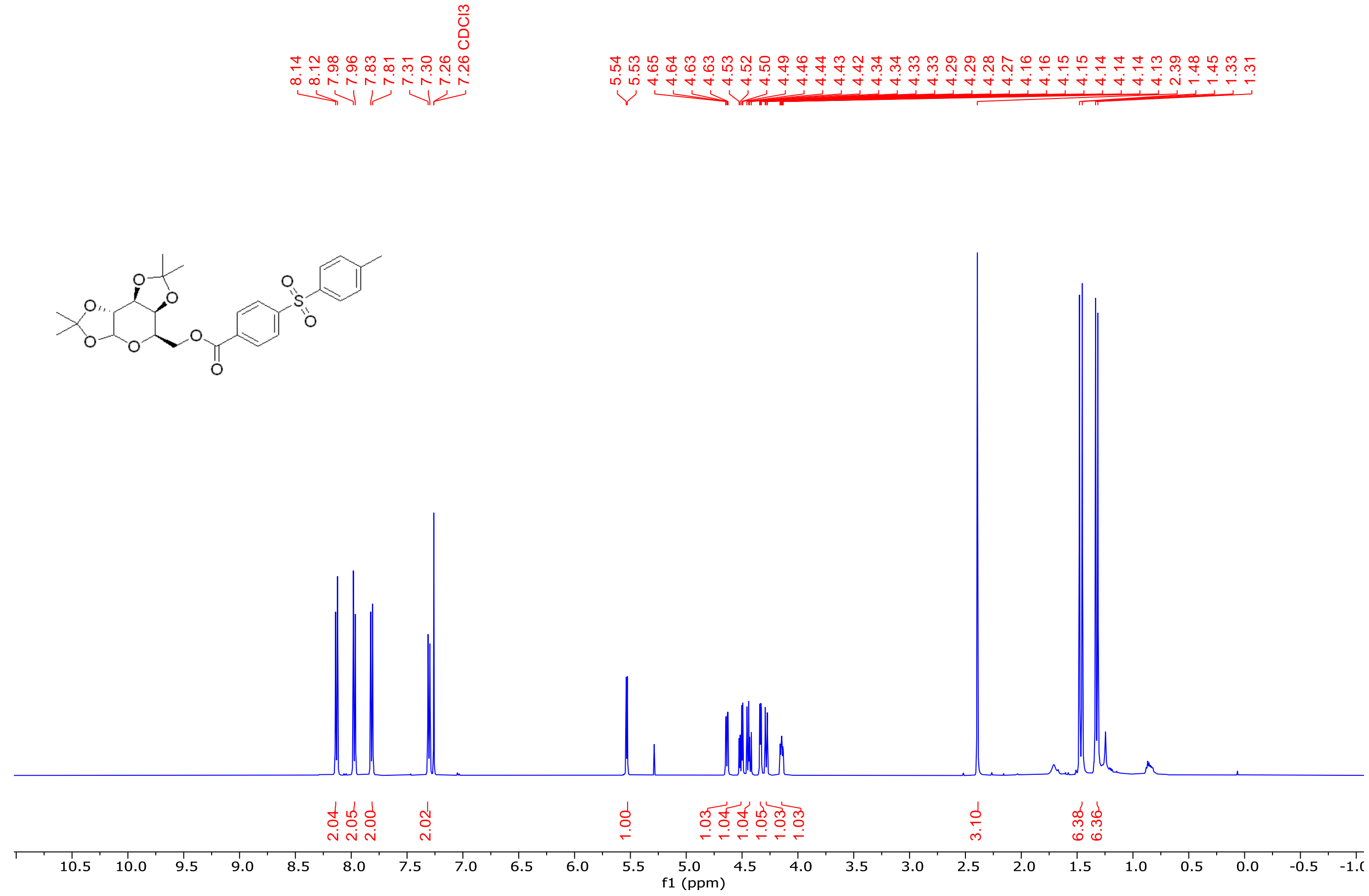

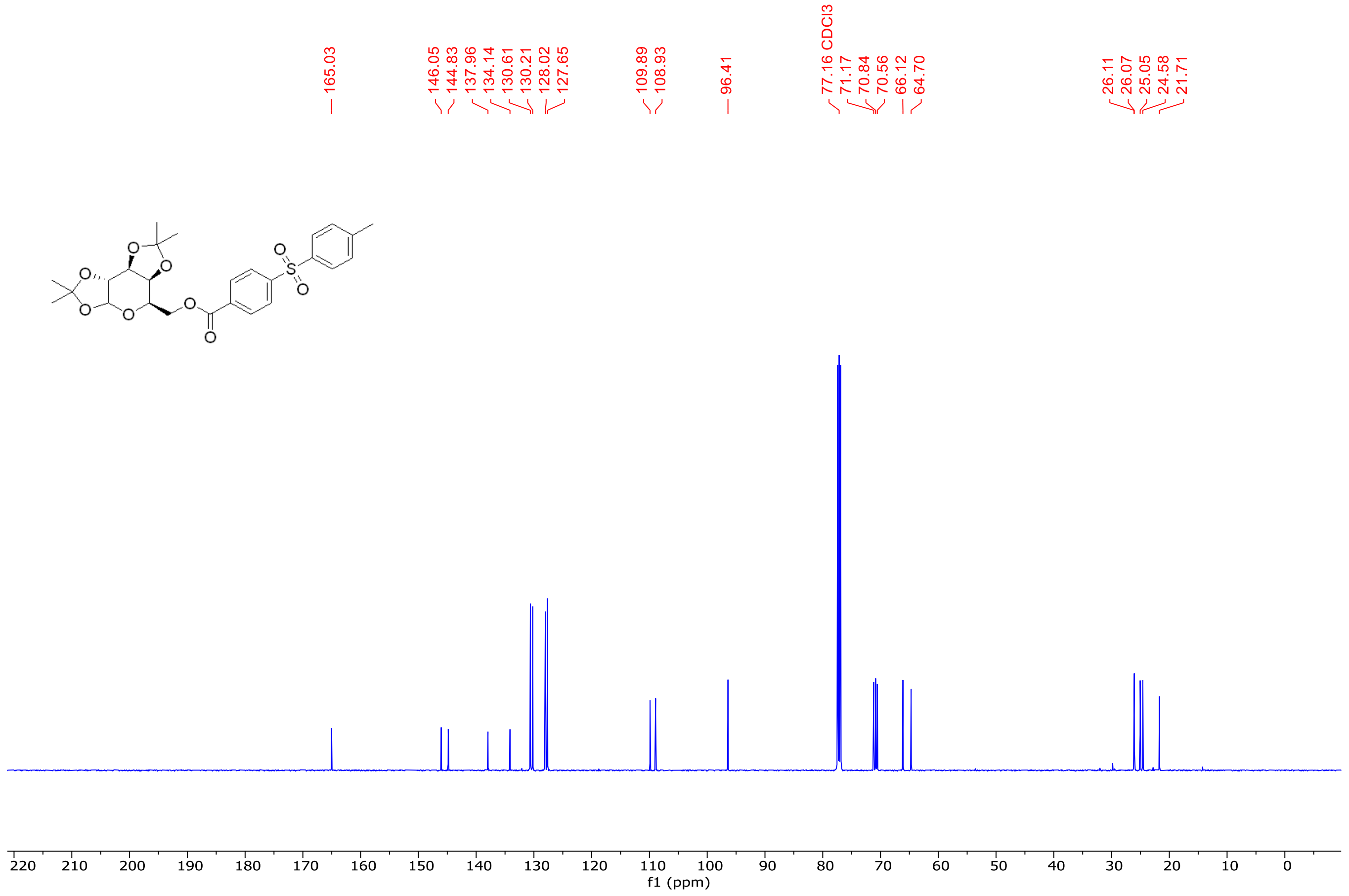

isopropyl 2-(4-(4-bromobenzoyl)phenoxy)-2-methylpropanoate (144)

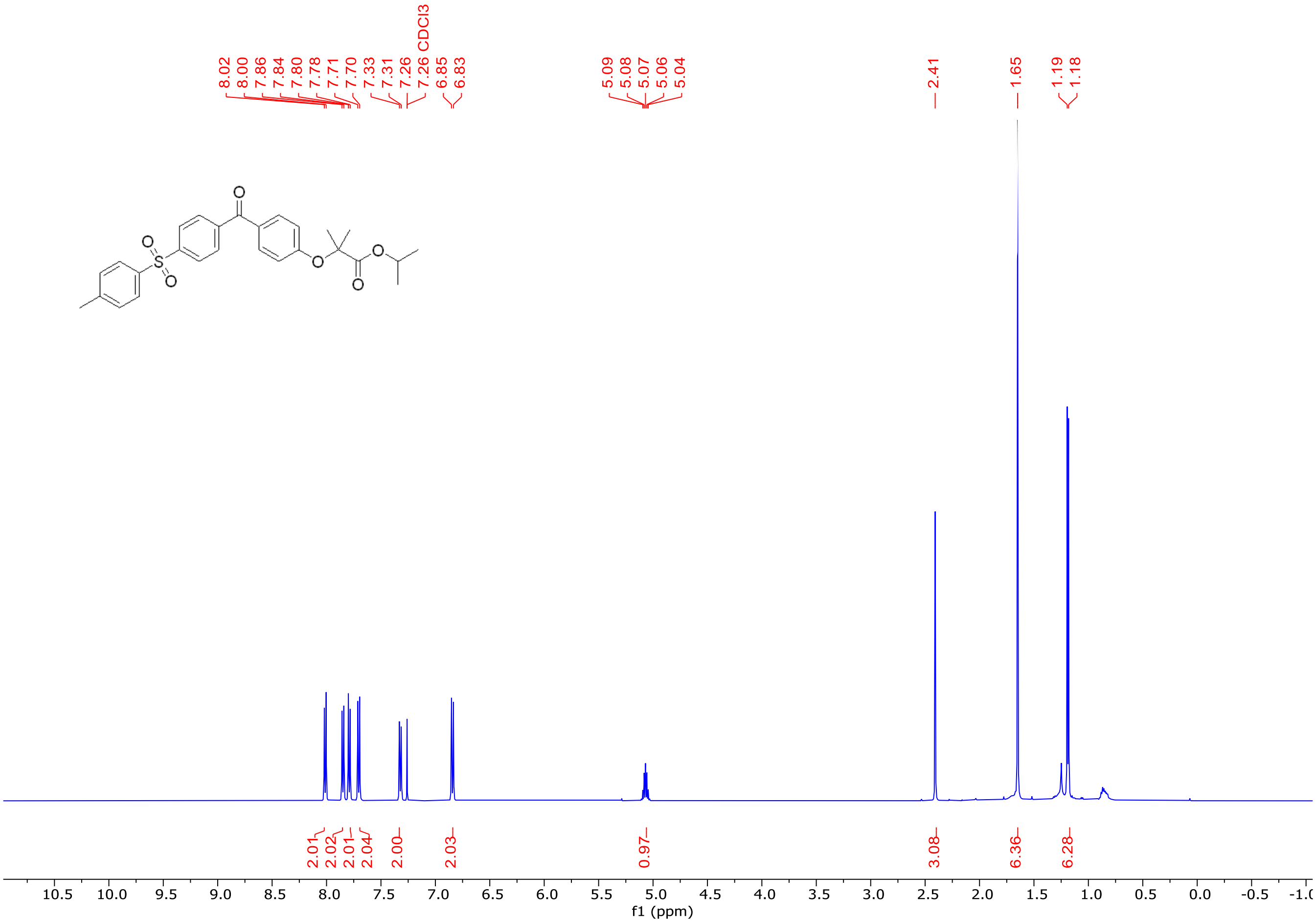

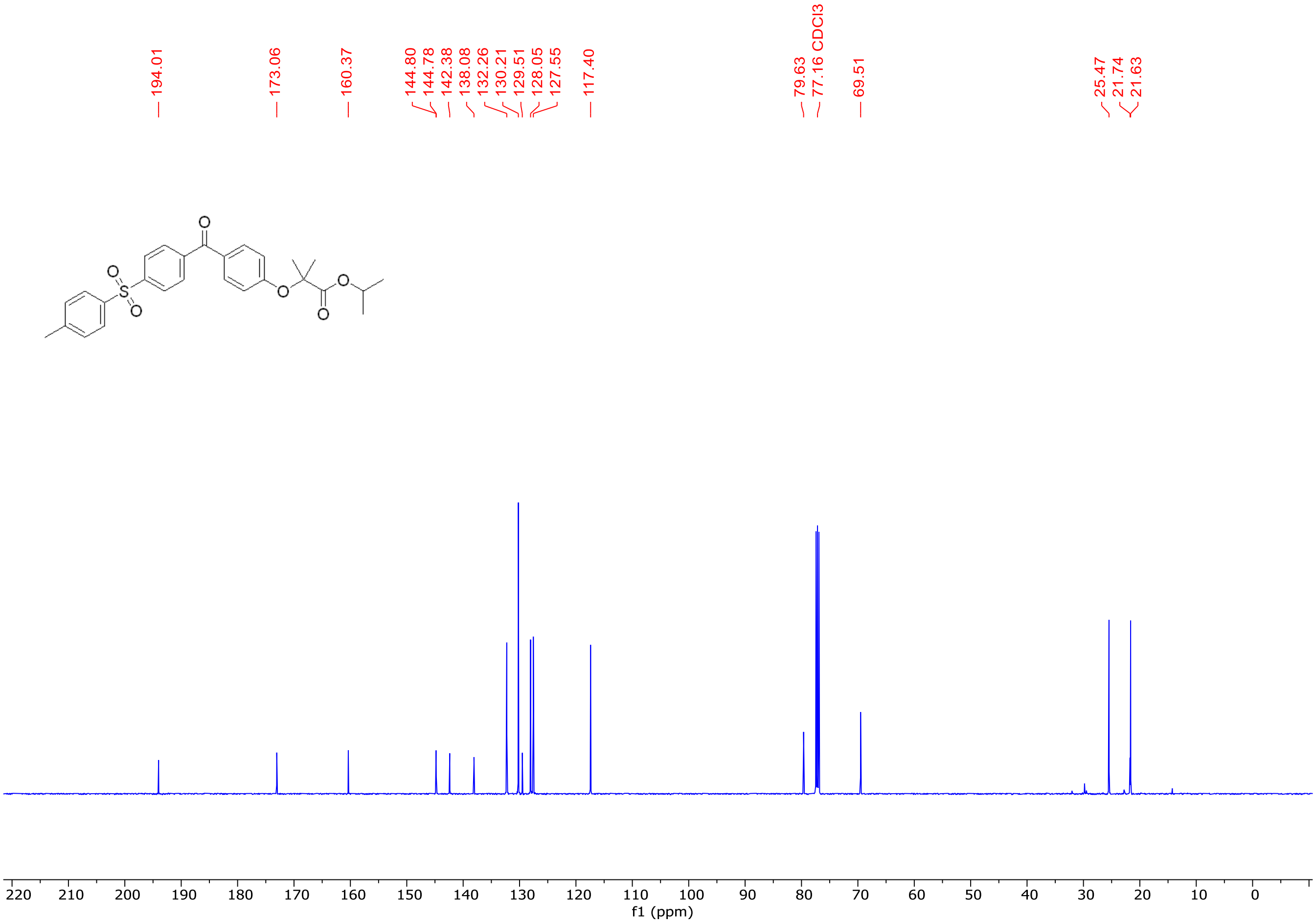

(10R,13S)-17-acetyl-10,13-dimethyl-2,3,4,7,8,9,10,11,12,13,14,15,16,17-tetradecahydro-1*H*-cyclopenta[a]phenanthren-3-yl 4-bromobenzoate (145)

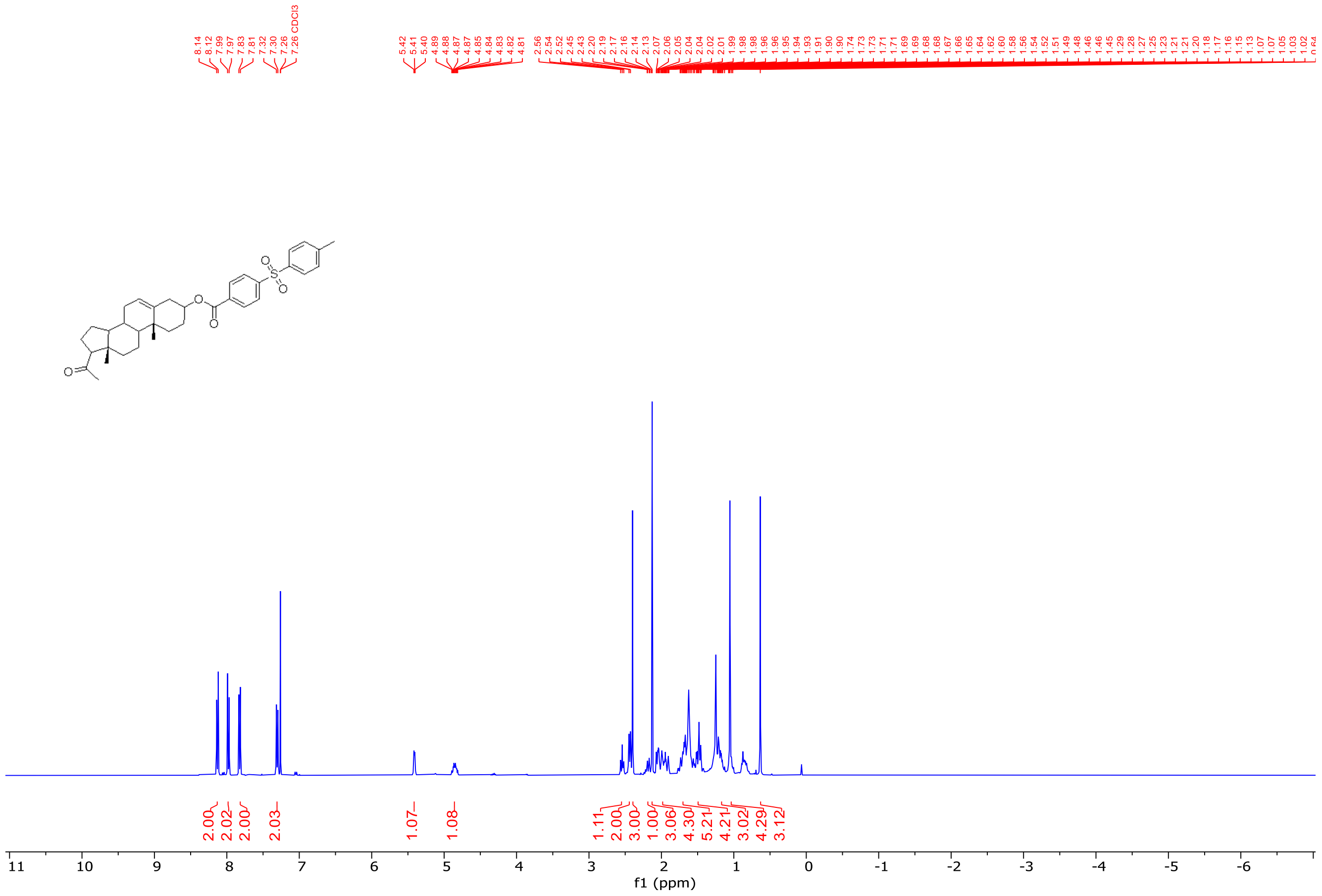

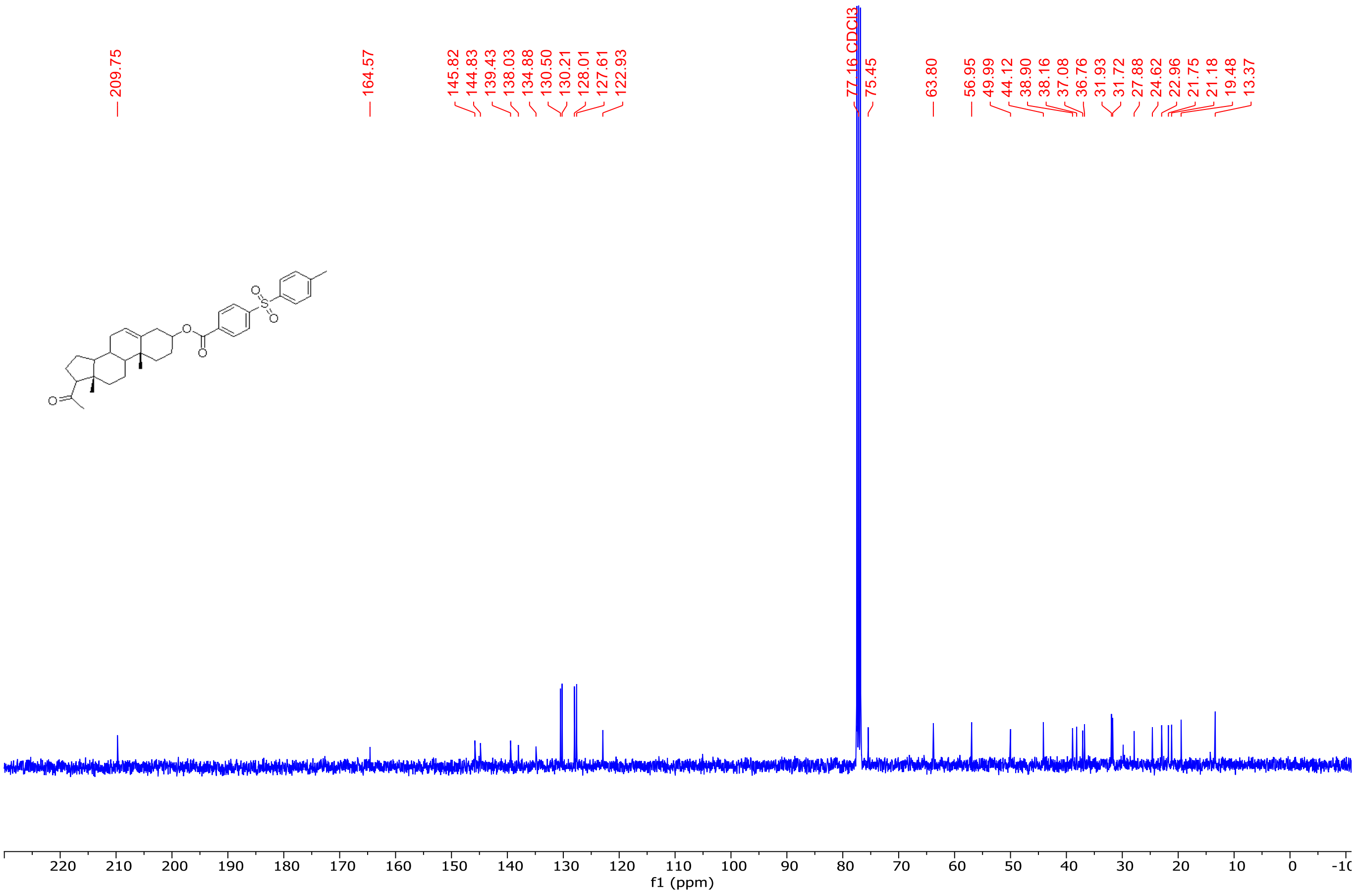

2-(piperidin-1-yl)-9*H*-fluoren-9-one (146)

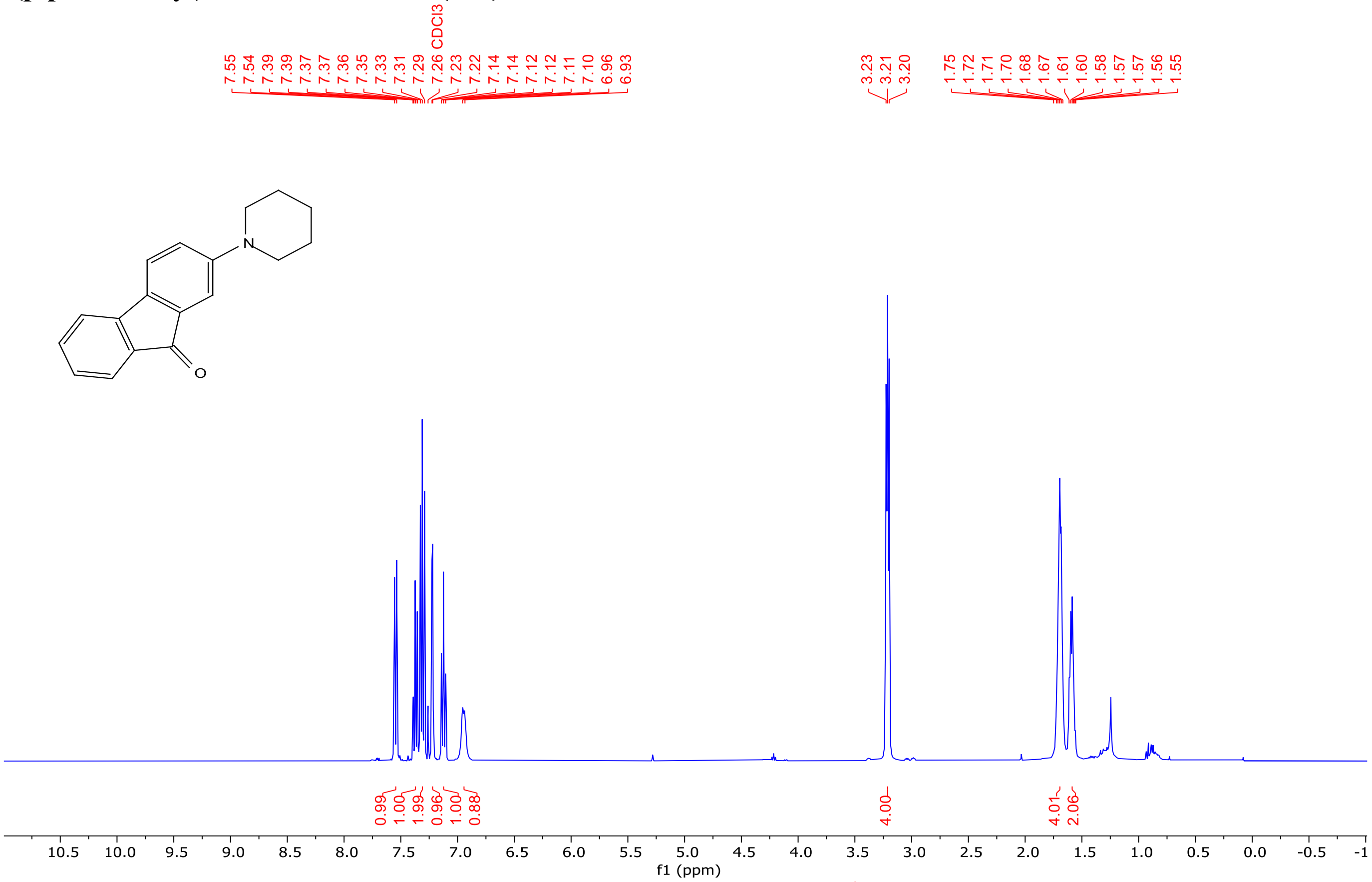

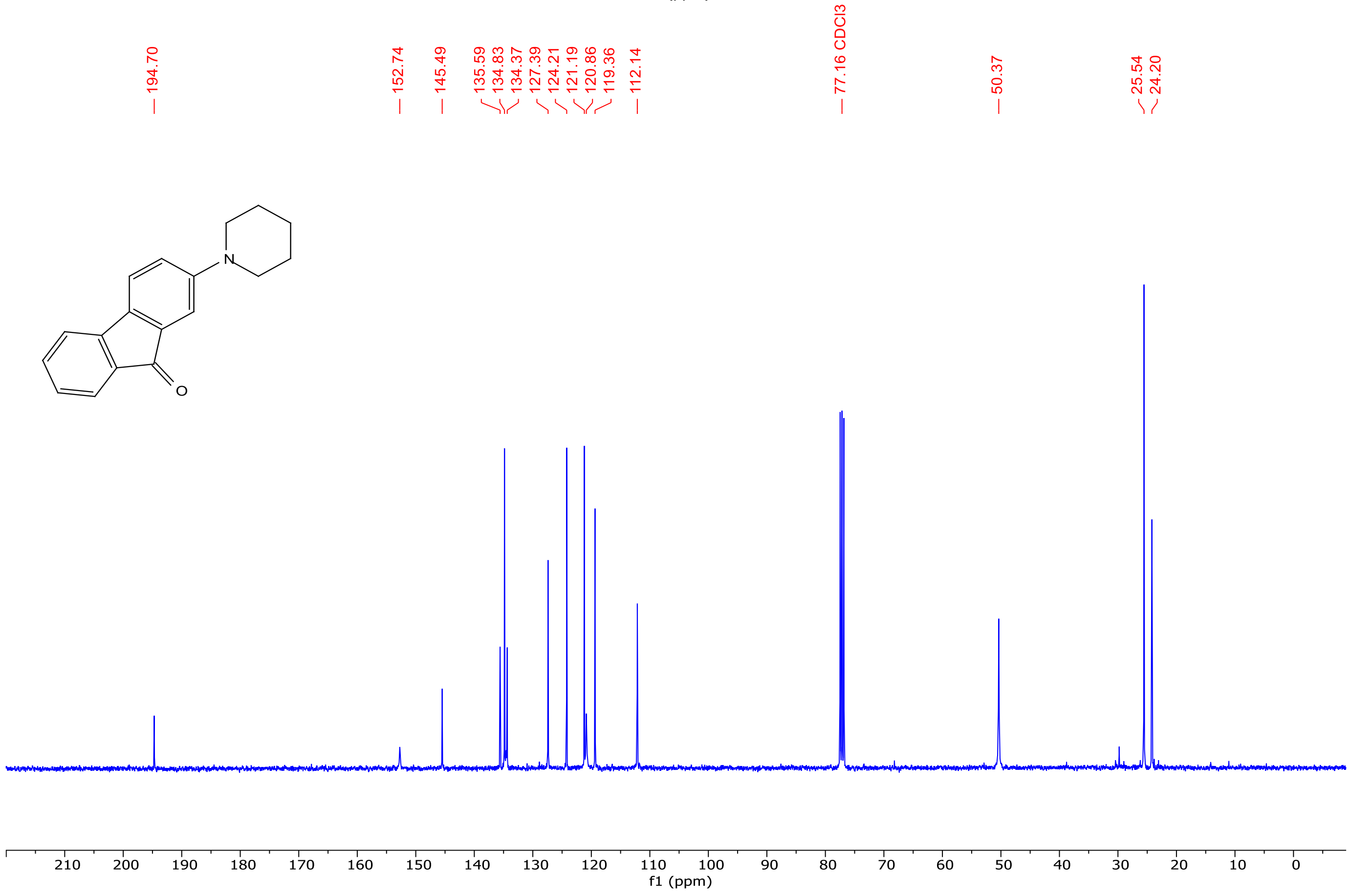

phenyl(4-(piperidin-1-yl)phenyl)methanone (147)

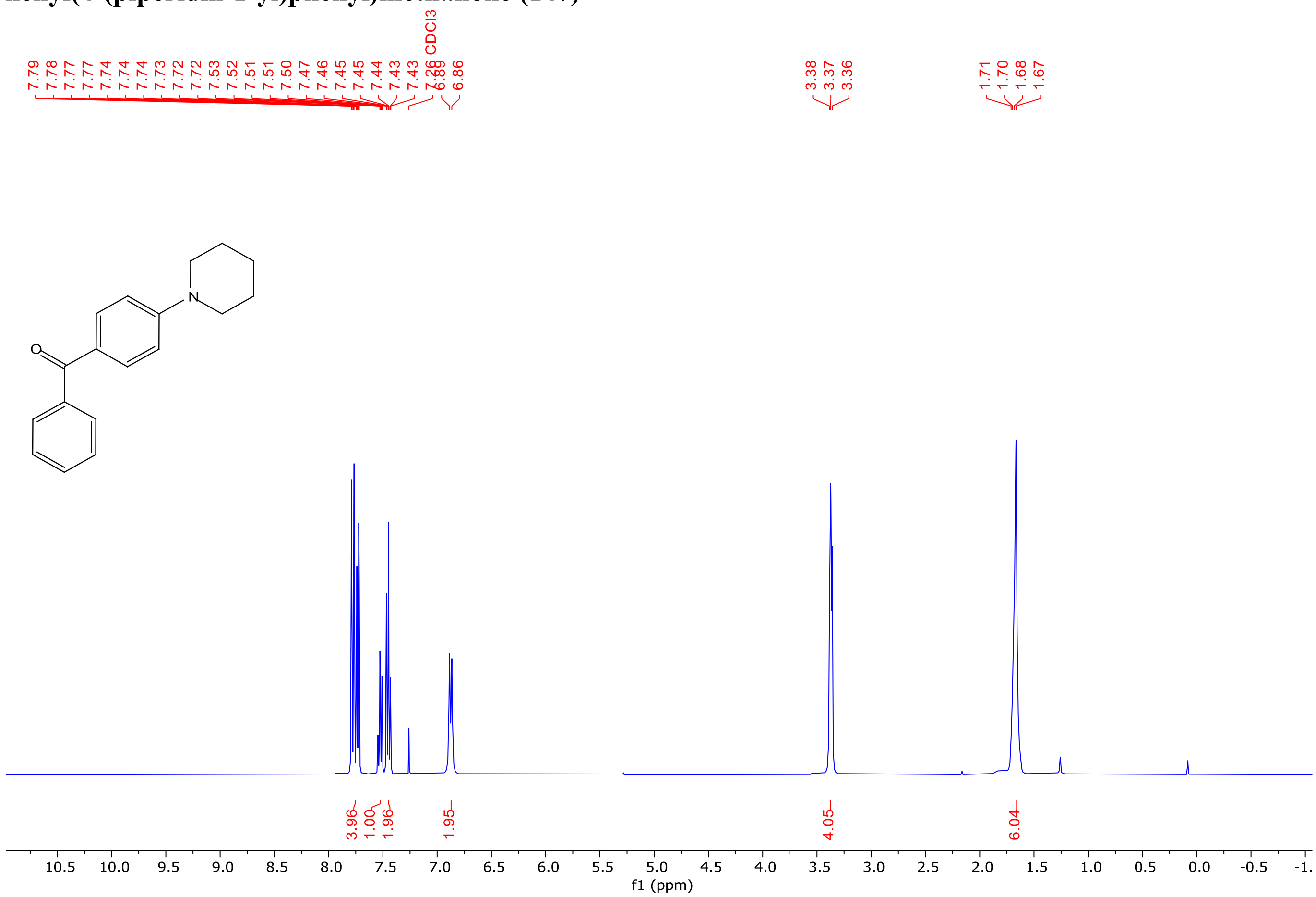

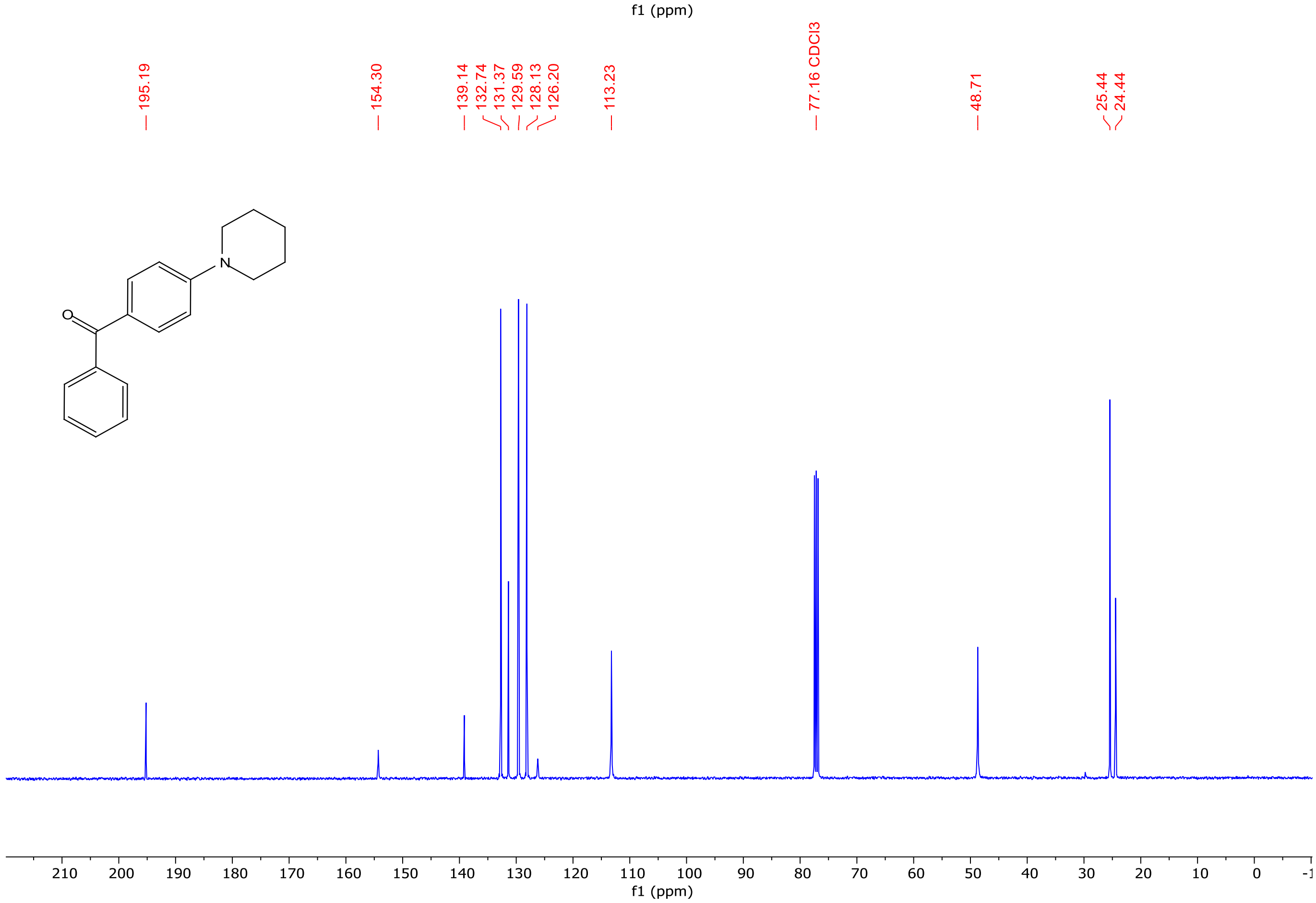

1-(4-nitrophenyl)piperidine (148)

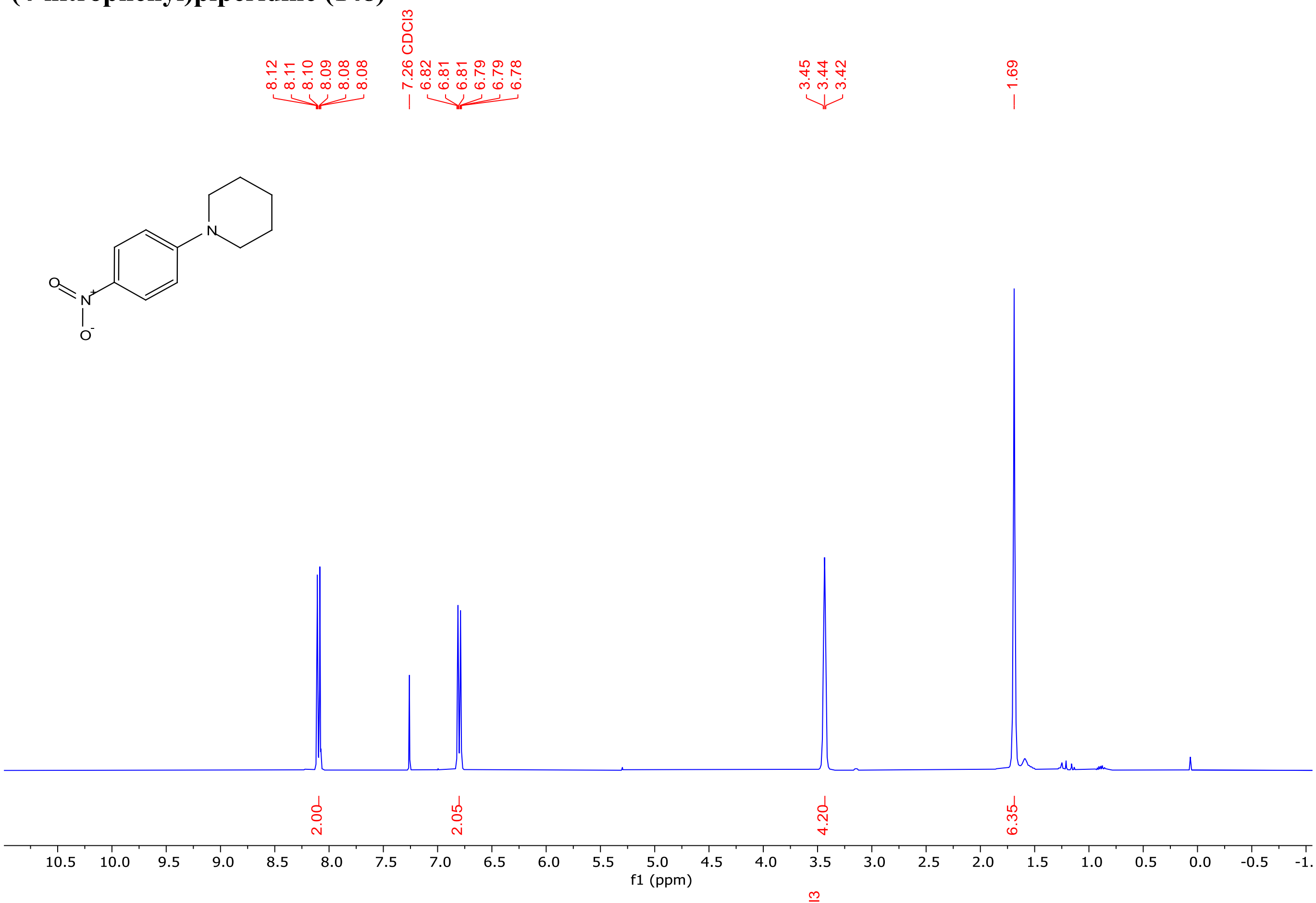

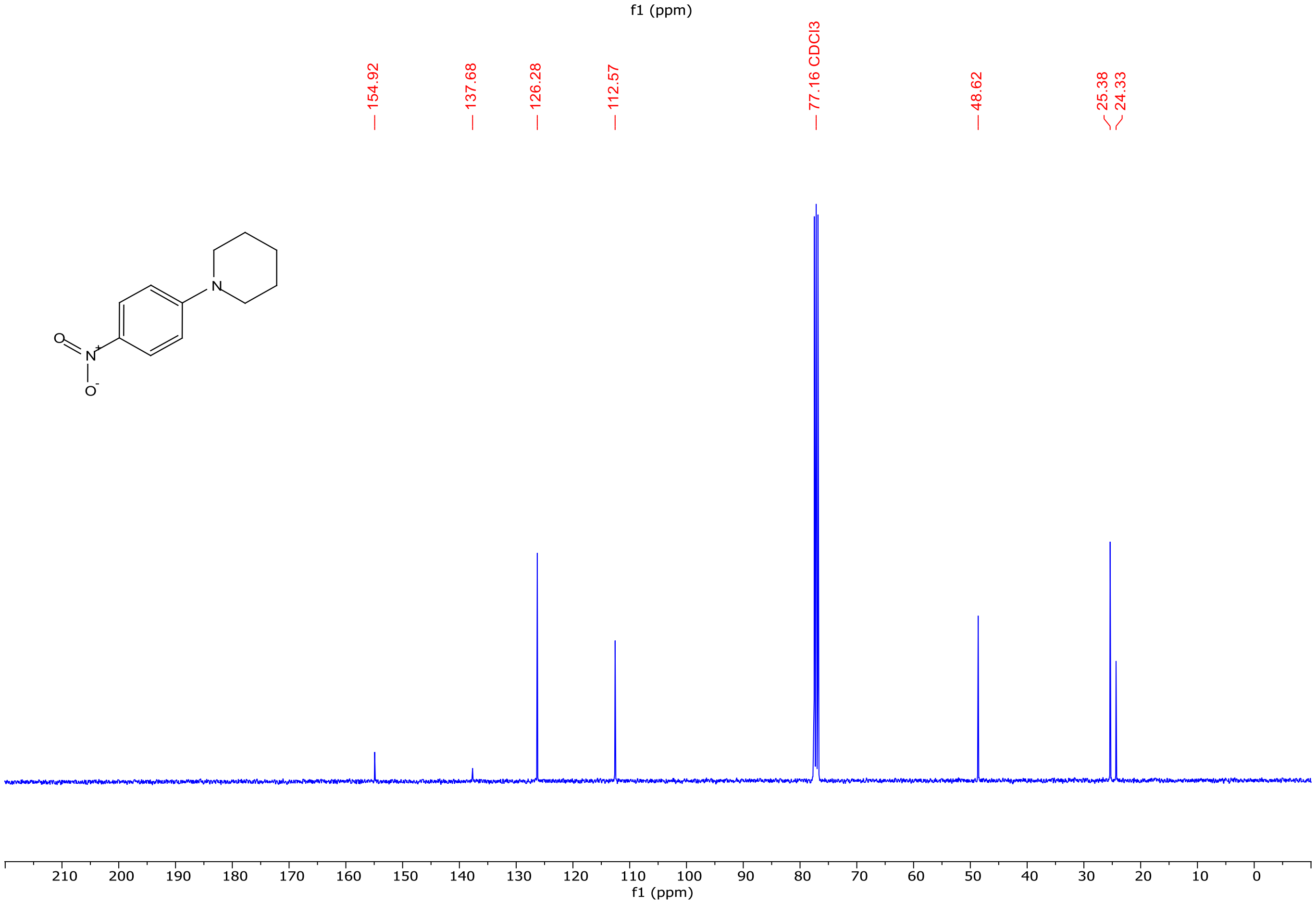

1-(phenanthren-3-yl)piperidine (149)

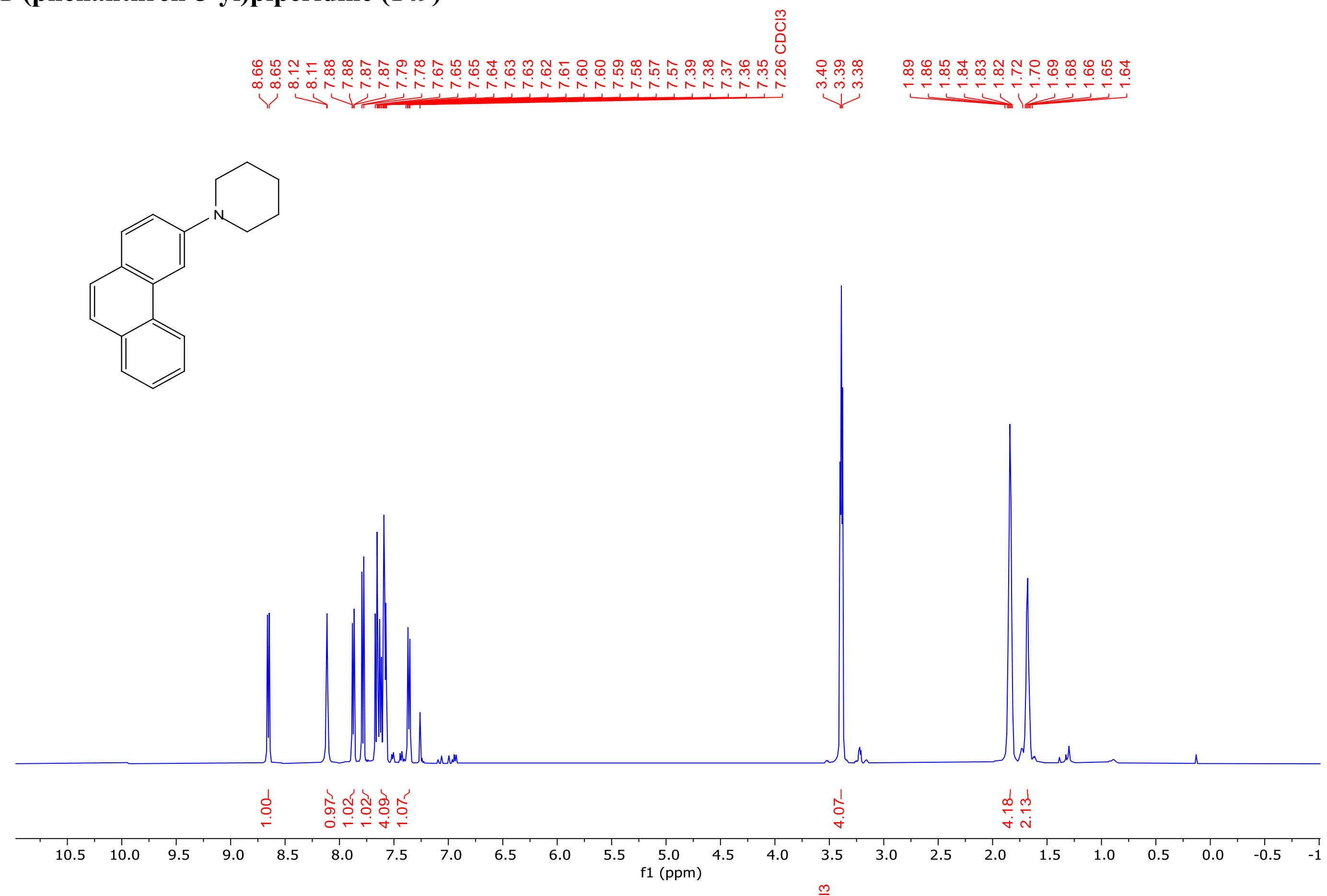

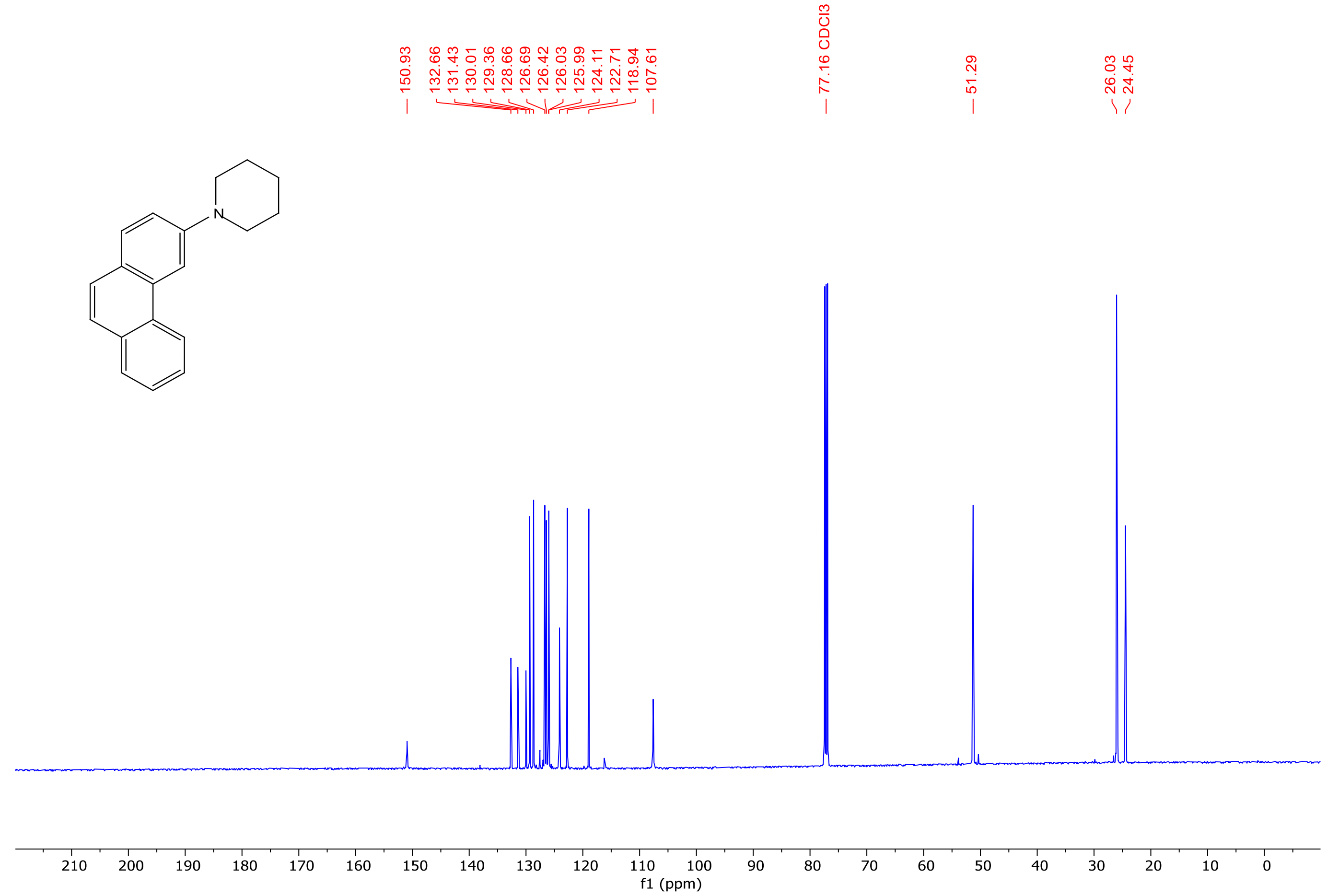

1-(pyren-1-yl)piperidine (150)

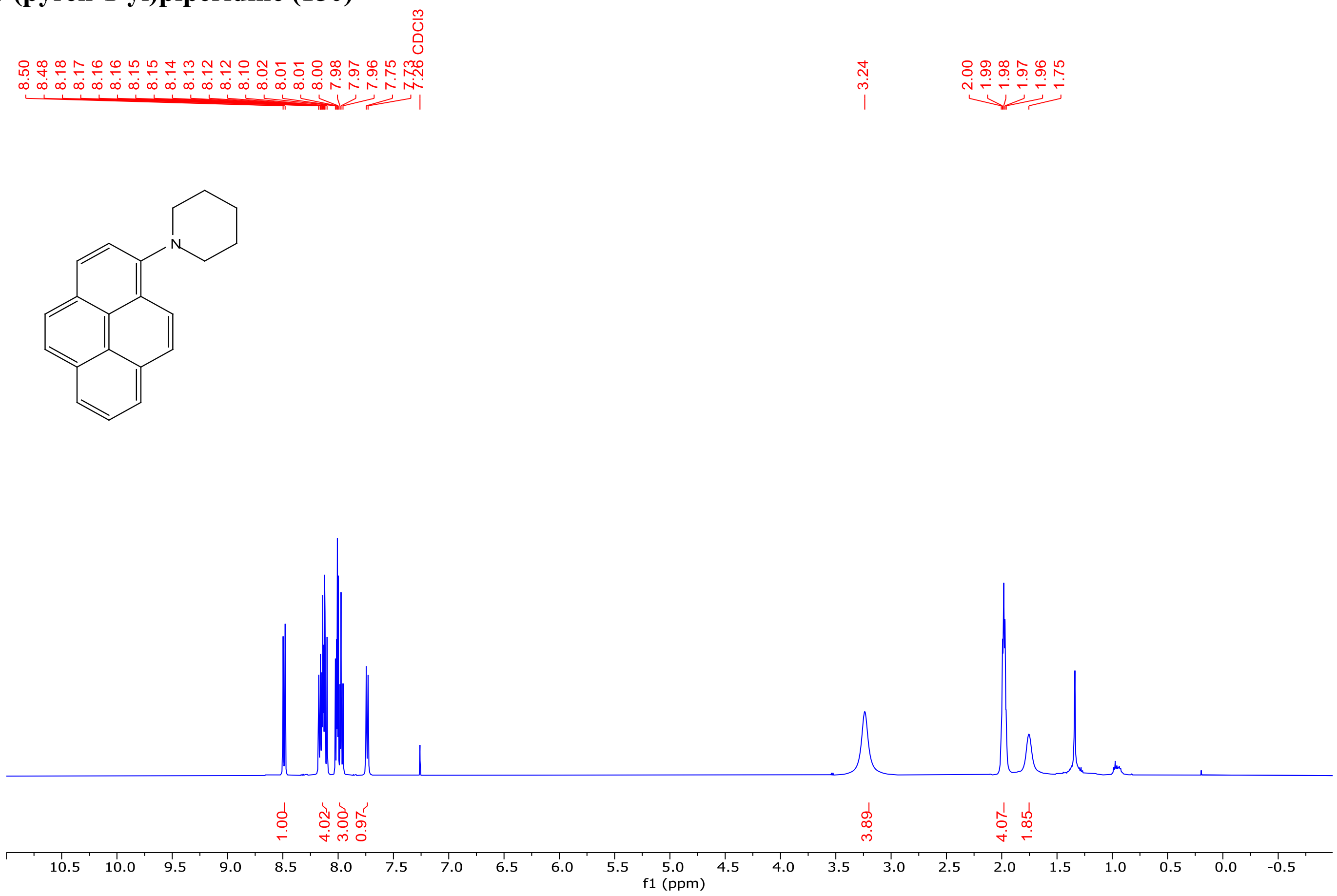

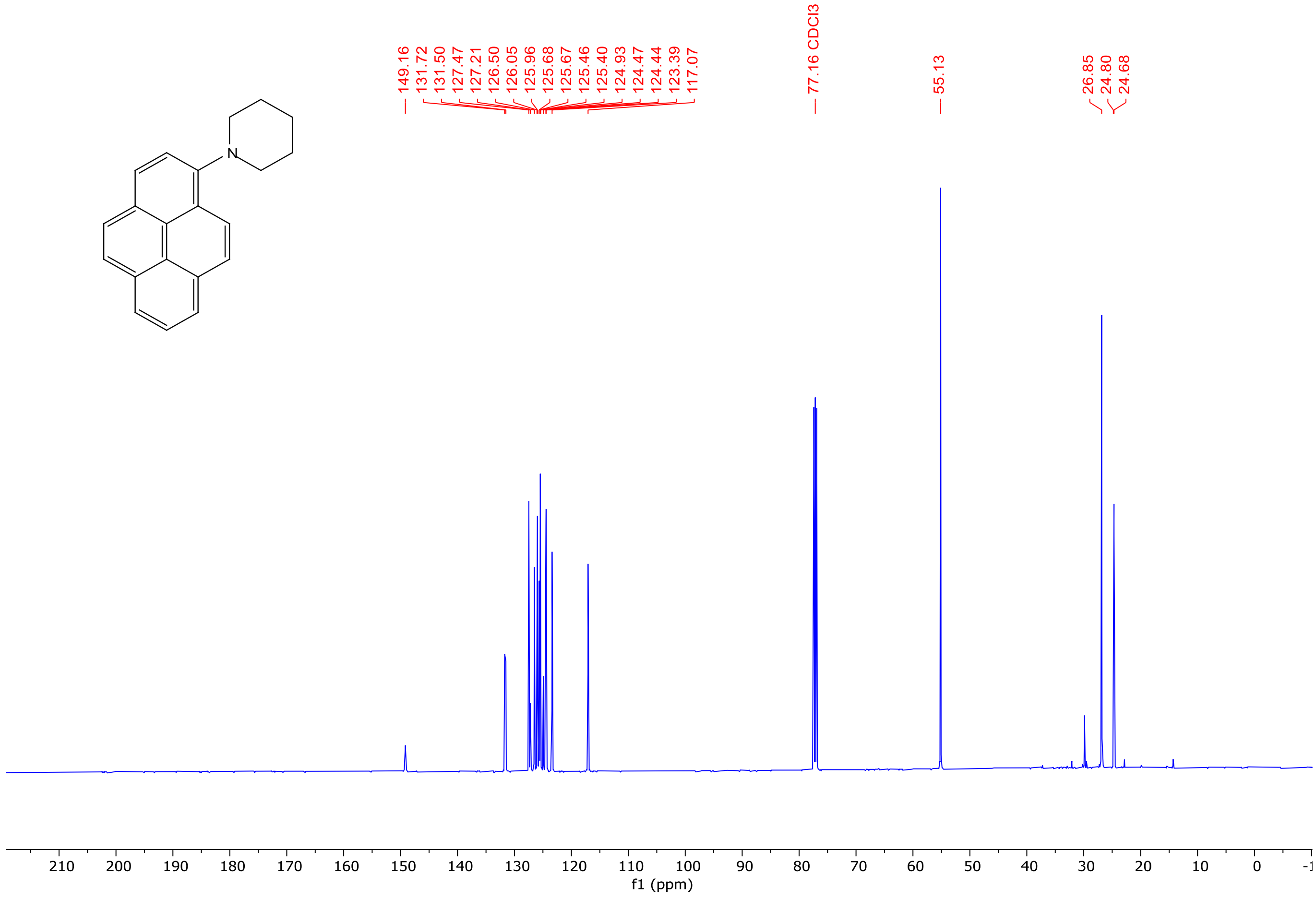

5-(piperidin-1-yl)quinoline-8-carbonitrile (151)

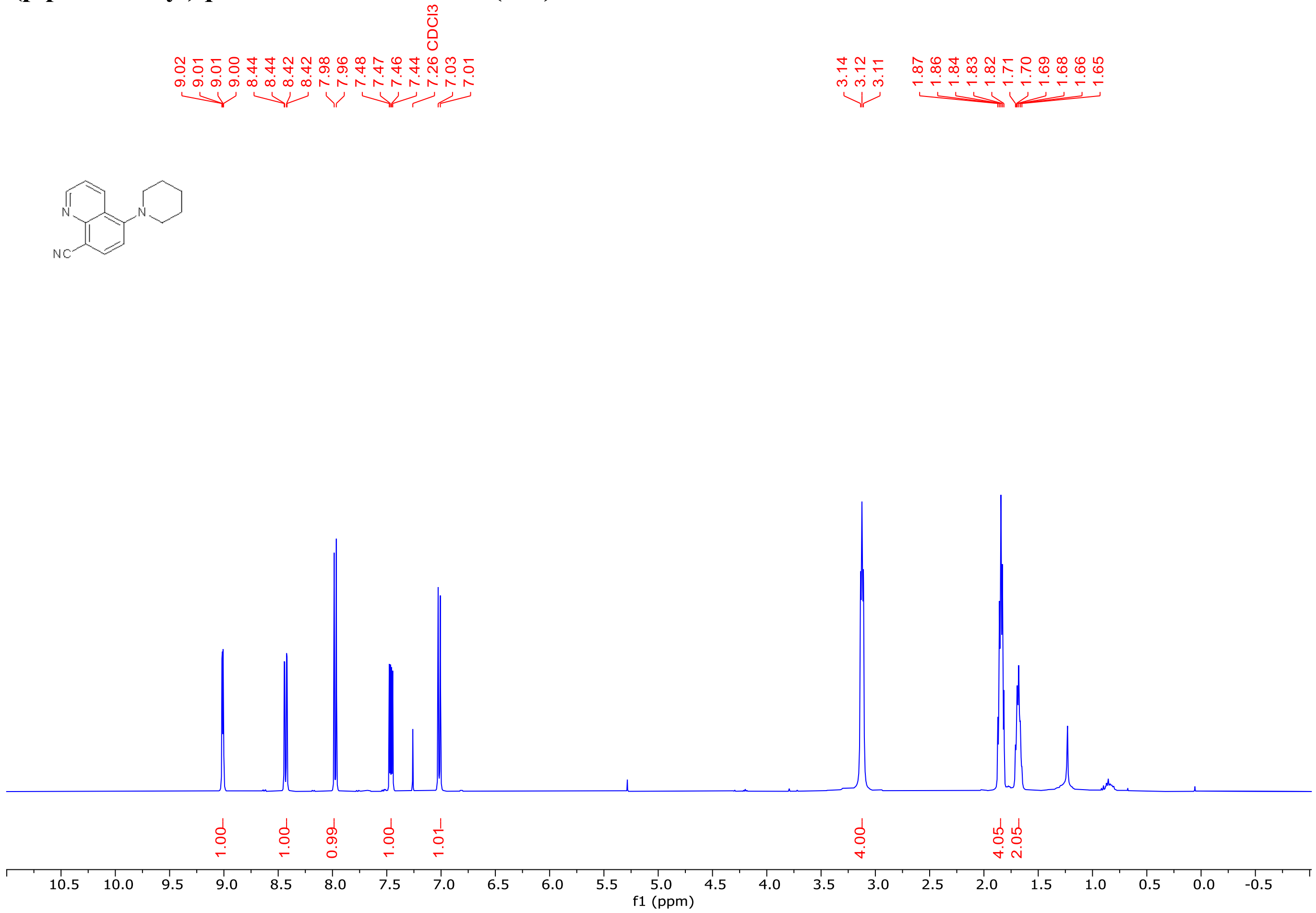

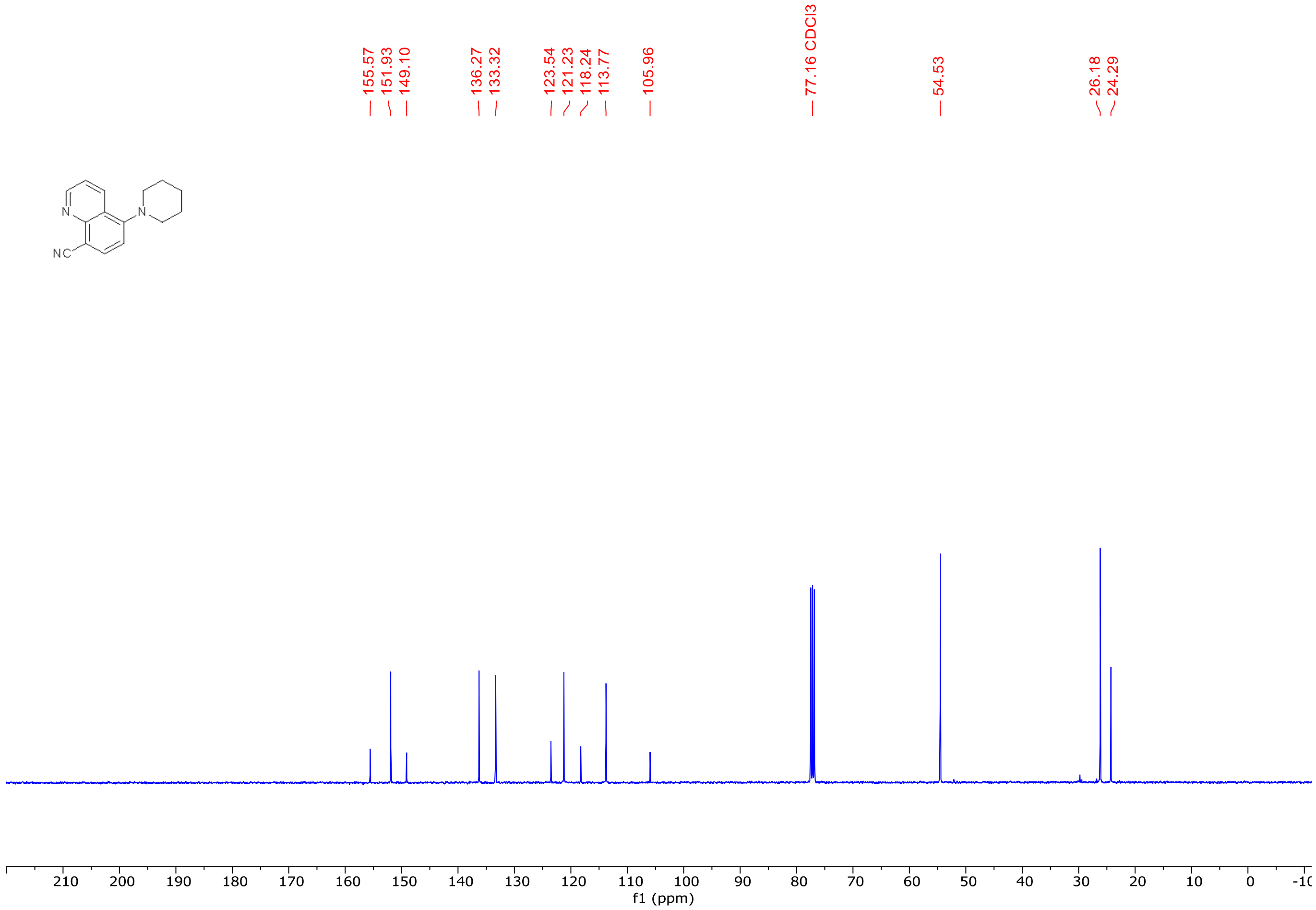

4-(piperidin-1-yl)benzo[c][1,2,5]thiadiazole (152)

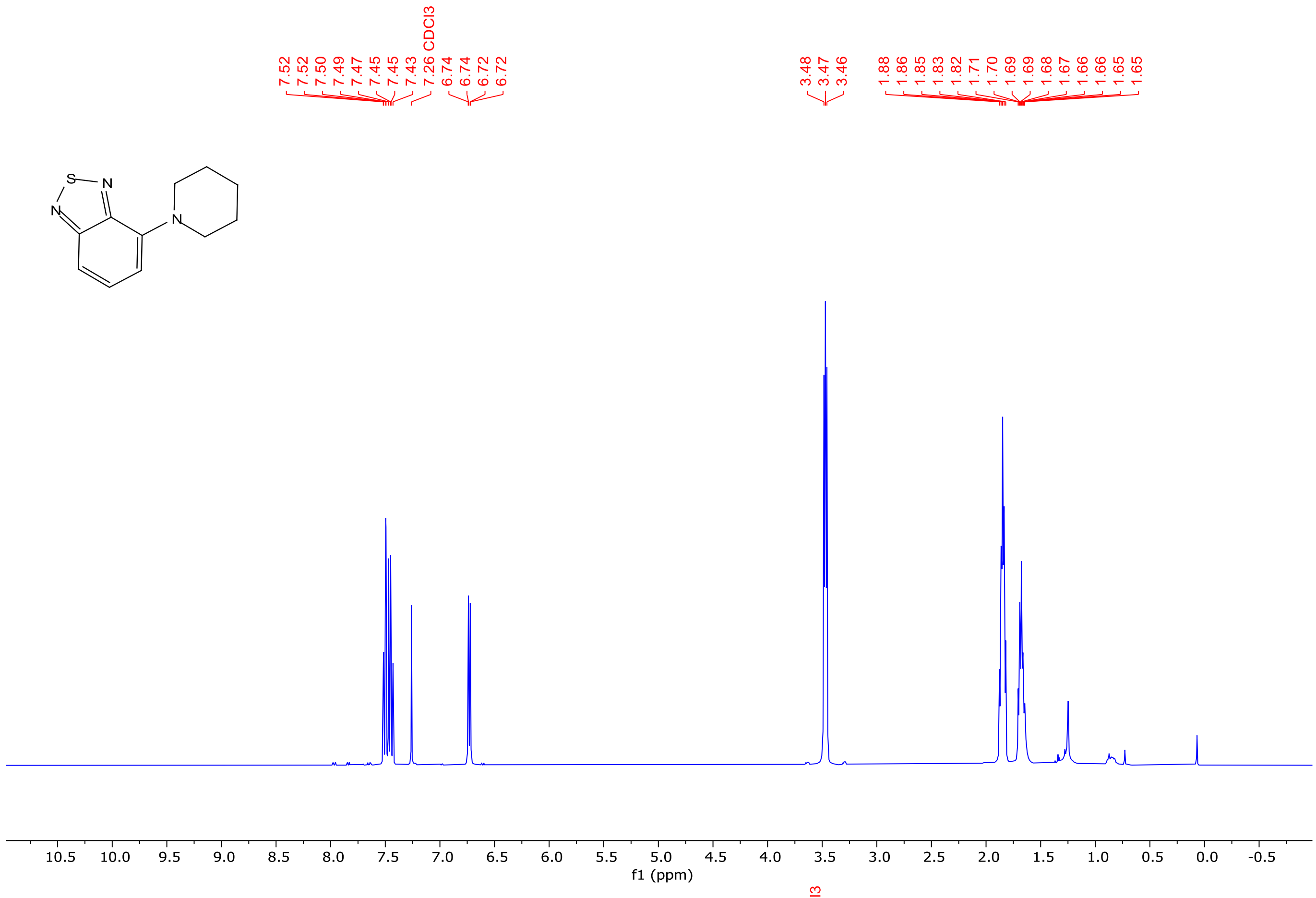
7.52
7.52
7.50
7.49
7.47
7.45
7.45
7.43
7.26 CDCl3
6.74
6.74
6.72
6.72
3.48
3.47
3.46
1.88
1.86
1.85
1.83
1.82
1.71
1.70
1.69
1.69
1.68
1.67
1.66
1.66
1.65
1.65
S
N
N
N
10.5 10.0 9.5 9.0 8.5 8.0 7.5 7.0 6.5 6.0 5.5 5.0 4.5 4.0 3.5 3.0 2.5 2.0 1.5 1.0 0.5 0.0 -0.5
f1 (ppm)

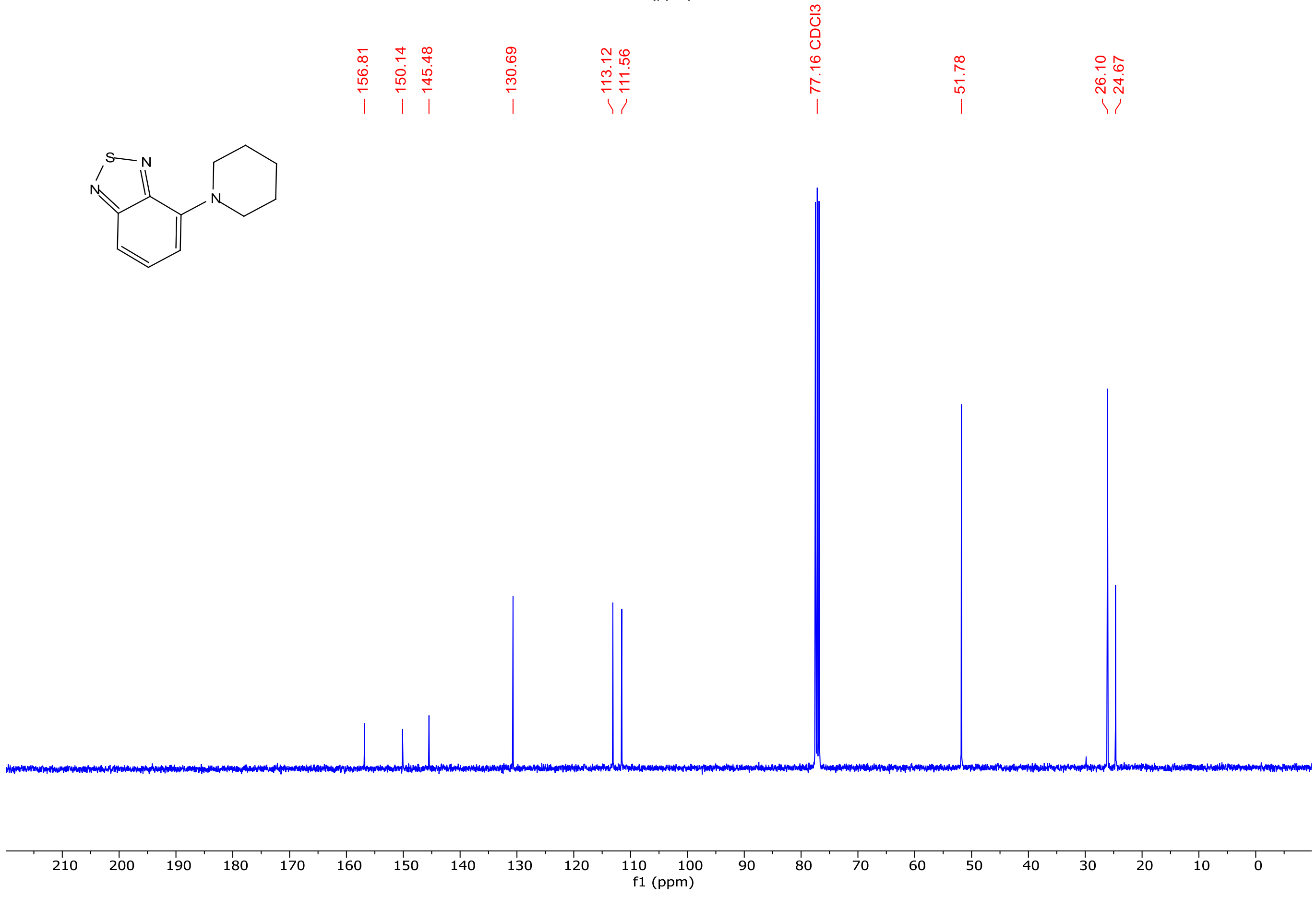
156.81
150.14
145.48
130.69
113.12
111.56
77.16 CDCl3
51.78
26.10
24.67
S
N
N
N
210 200 190 180 170 160 150 140 130 120 110 100 90 80 70 60 50 40 30 20 10 0
f1 (ppm)

5-(piperidin-1-yl)benzo[c][1,2,5]thiadiazole (153)

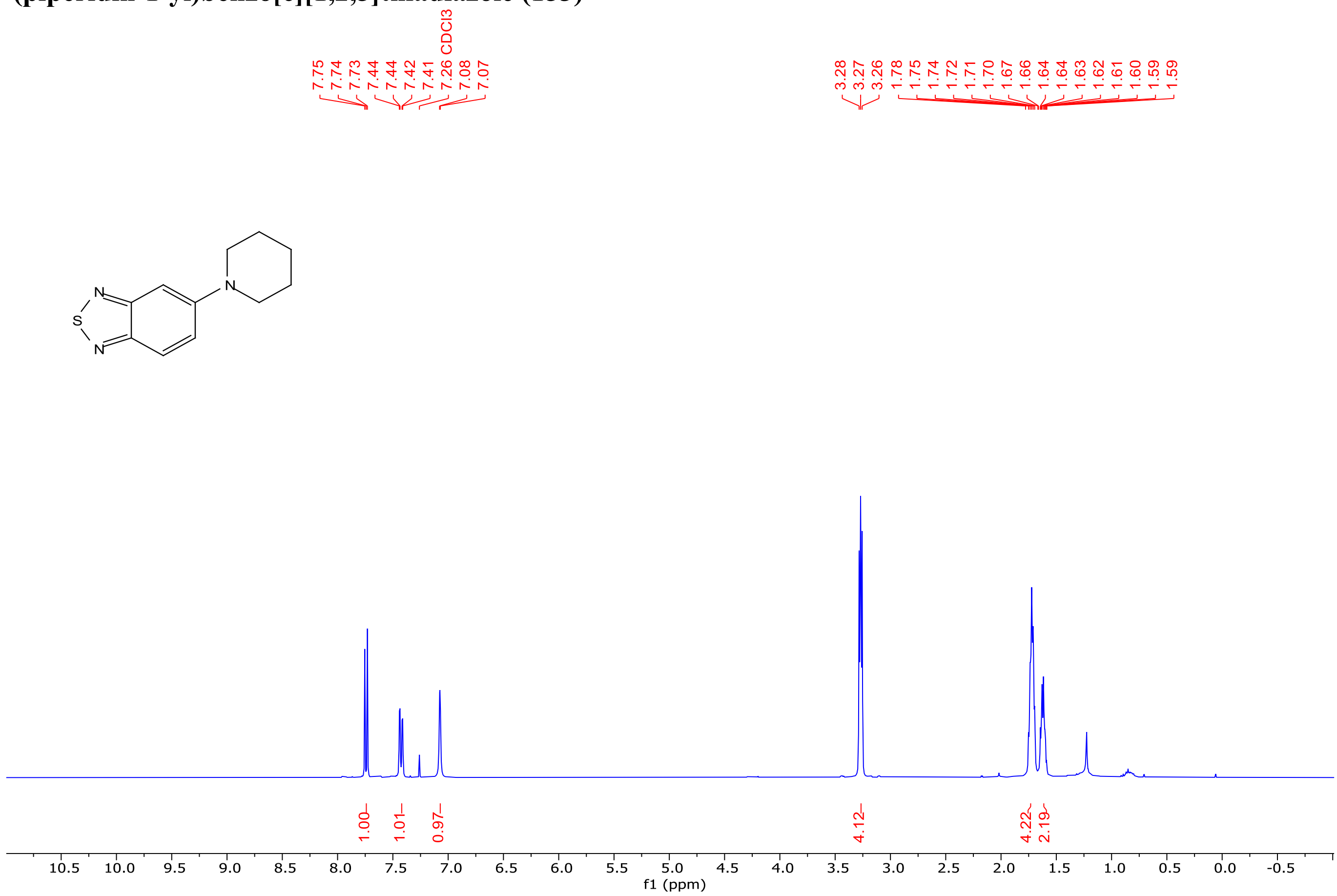

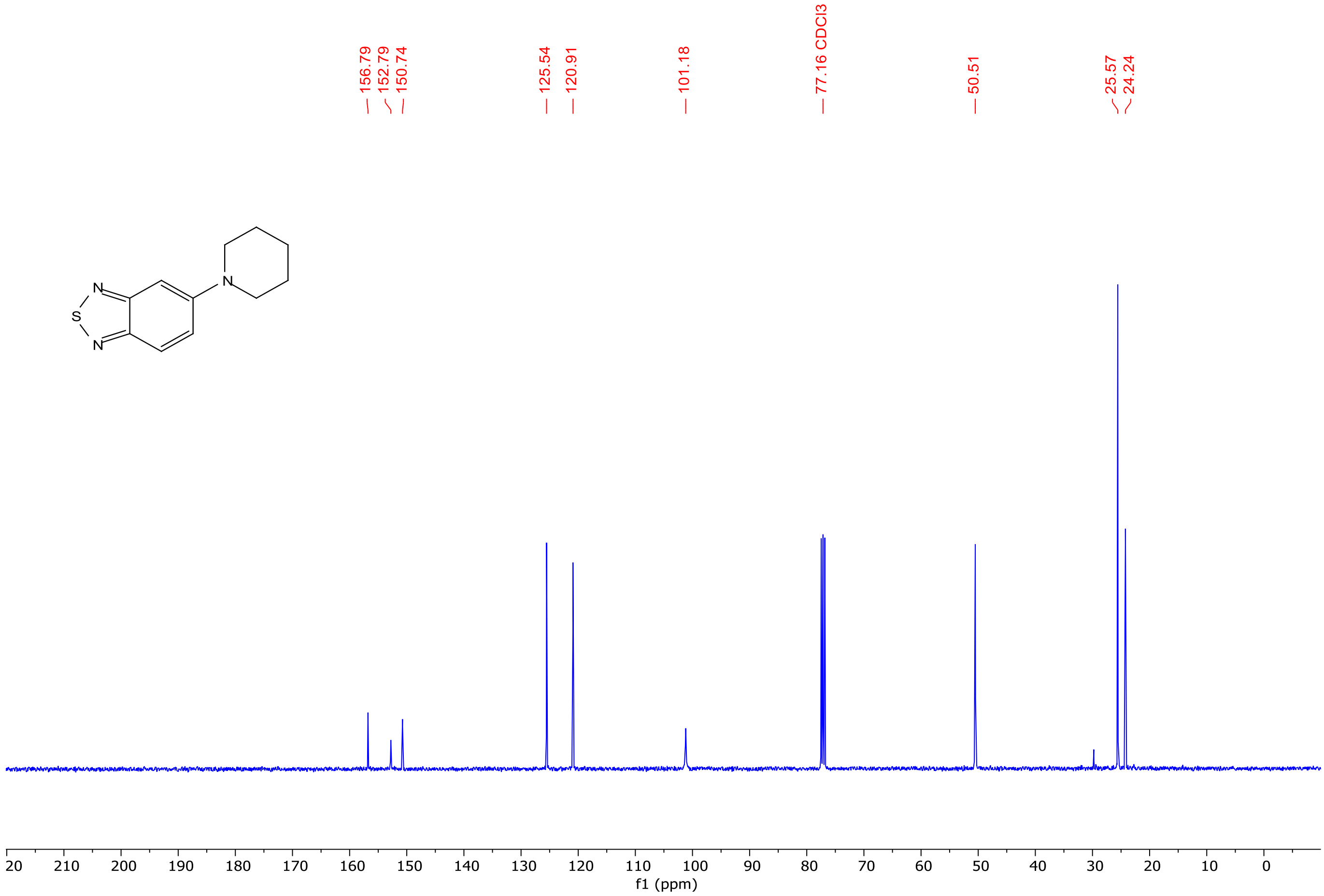

2-(piperidin-1-yl)quinoxaline (154)

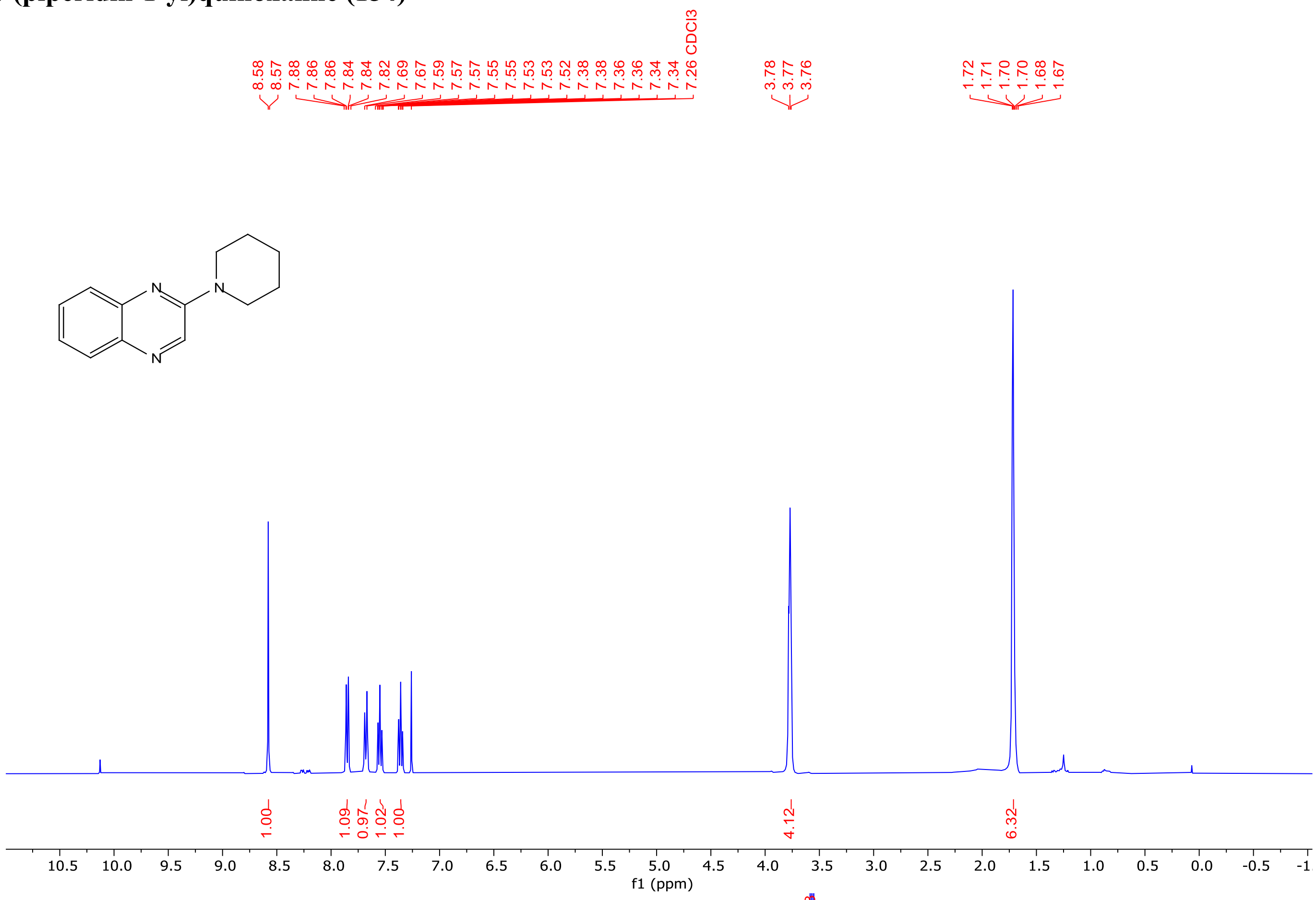

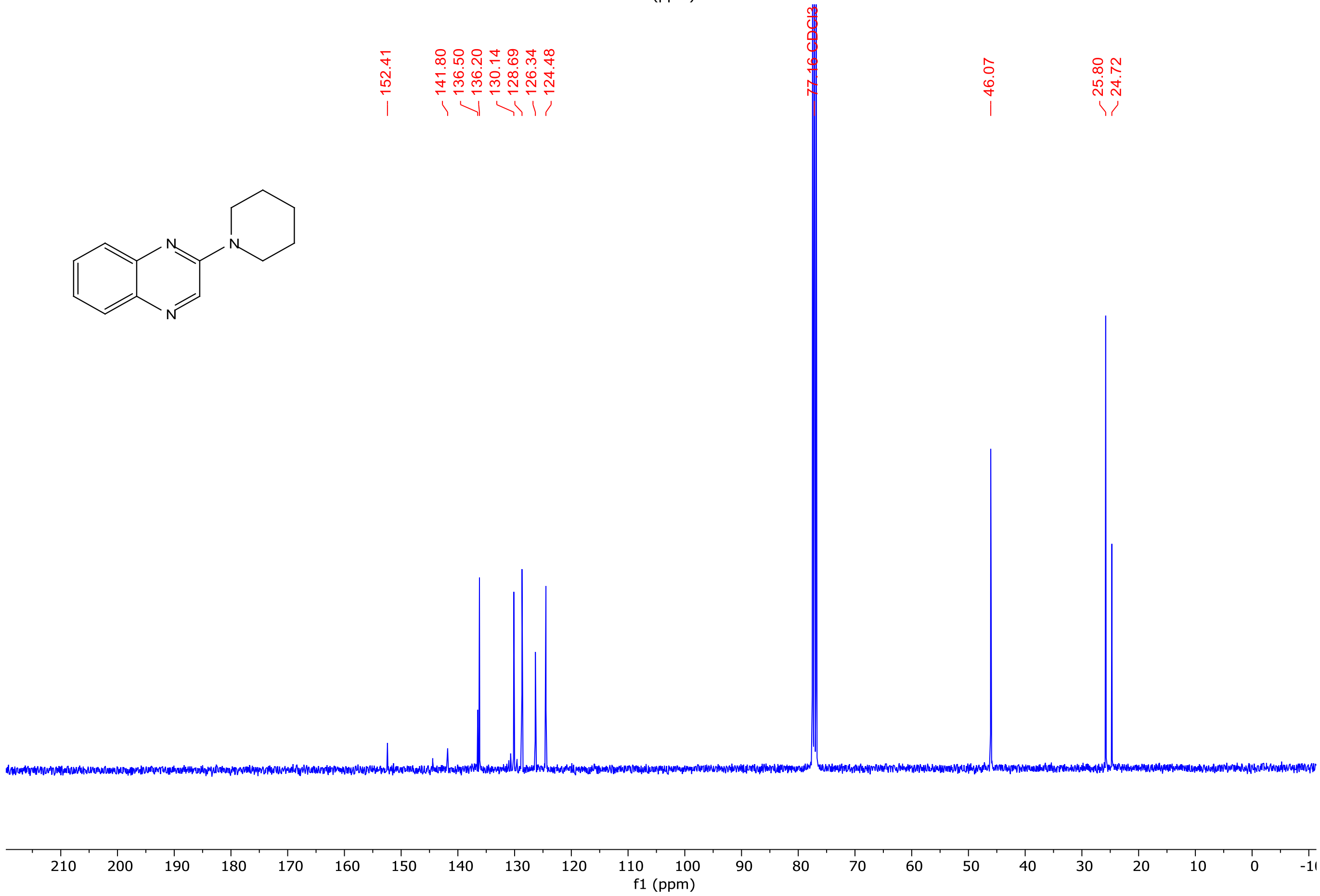

2-(piperidin-1-yl)pyrazine (155)

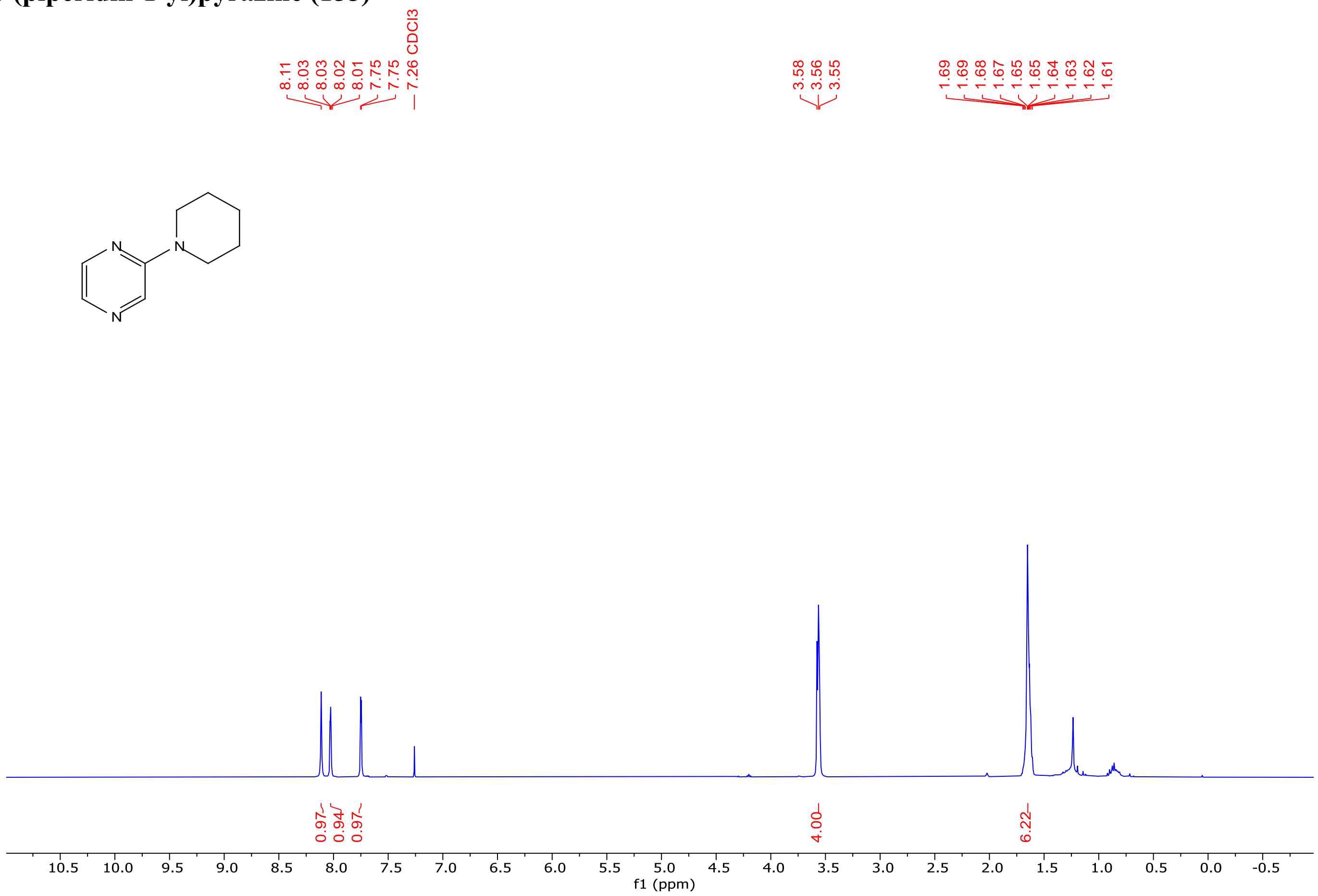

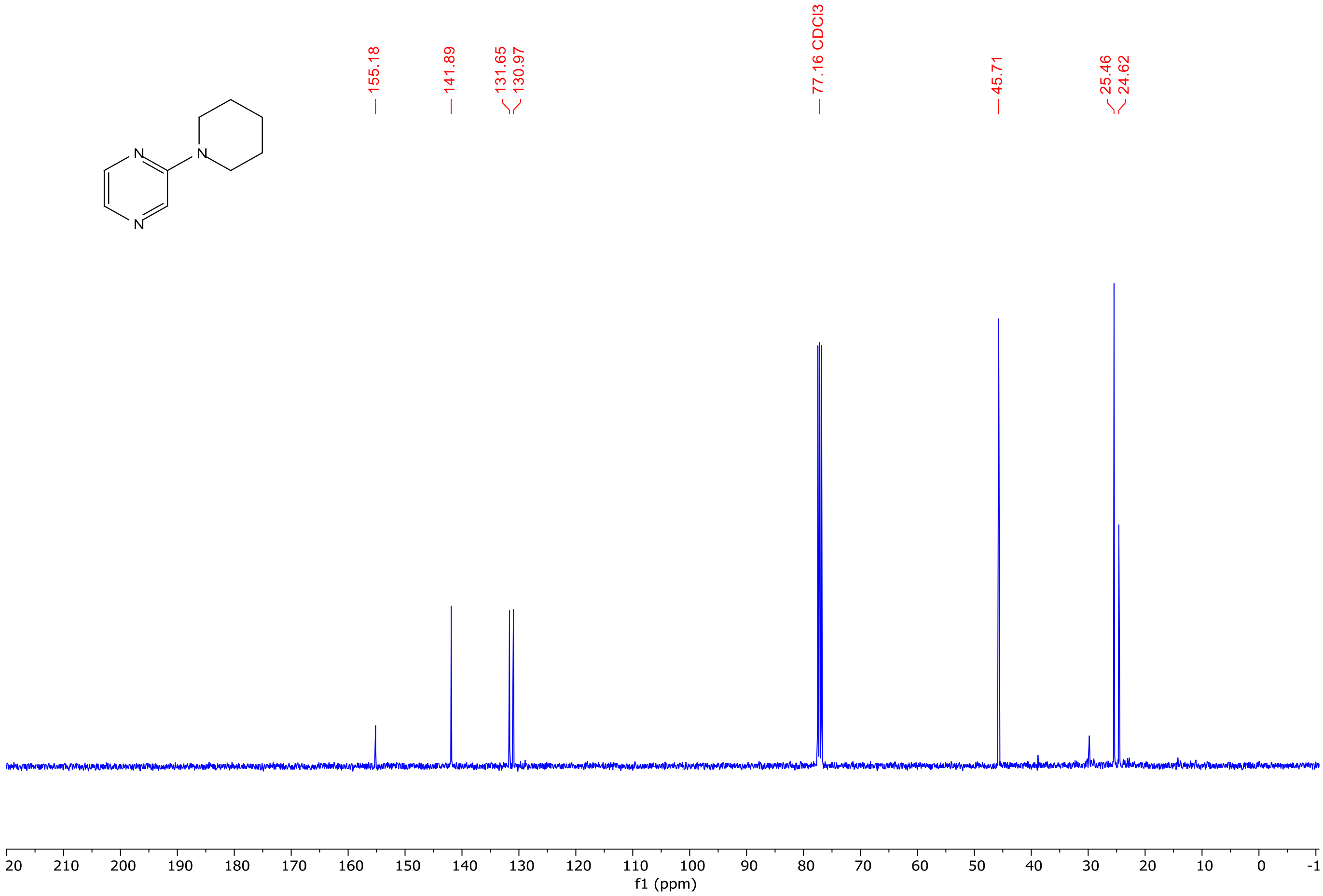

3,6-di(piperidin-1-yl)-9*H*-fluoren-9-one (156)

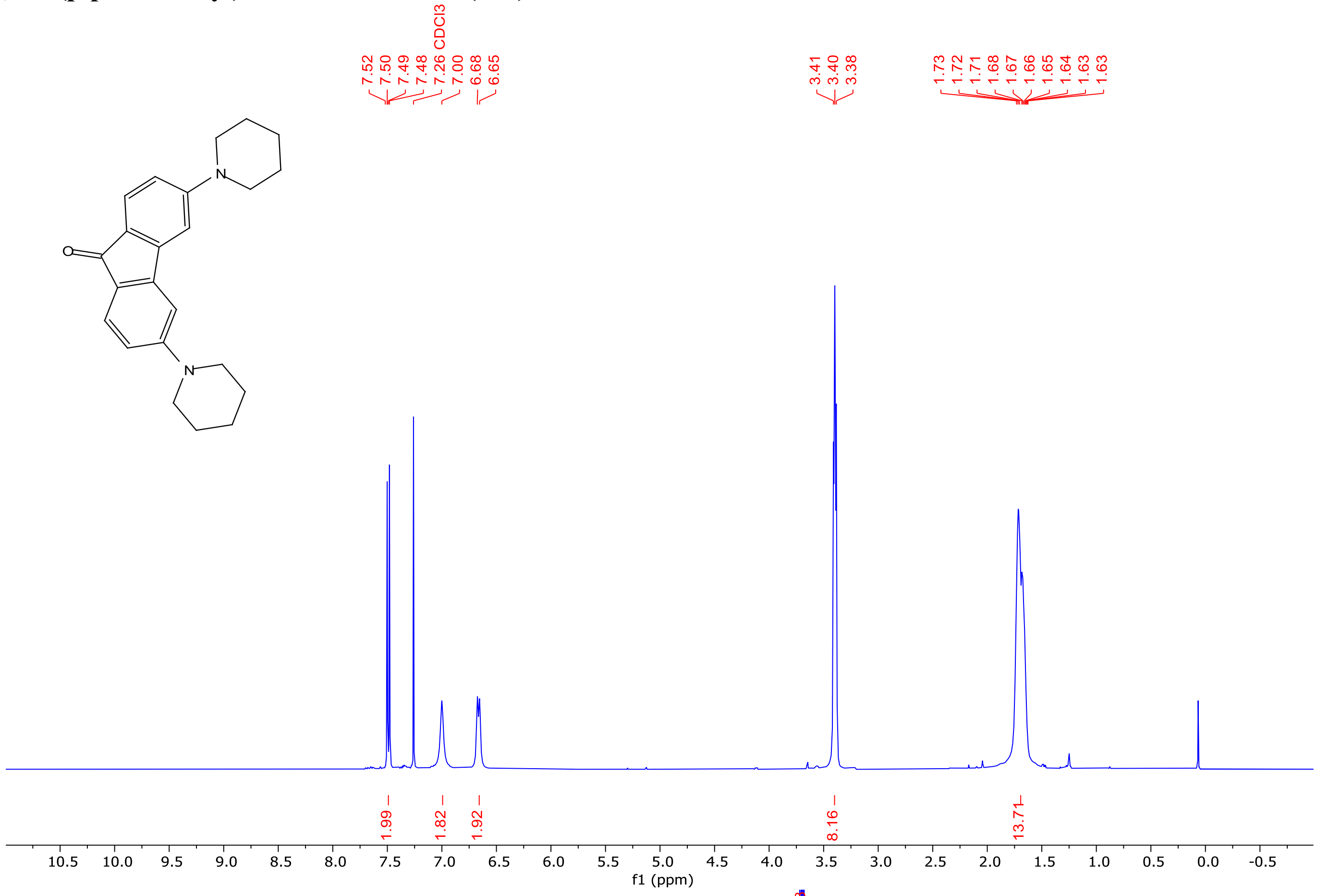

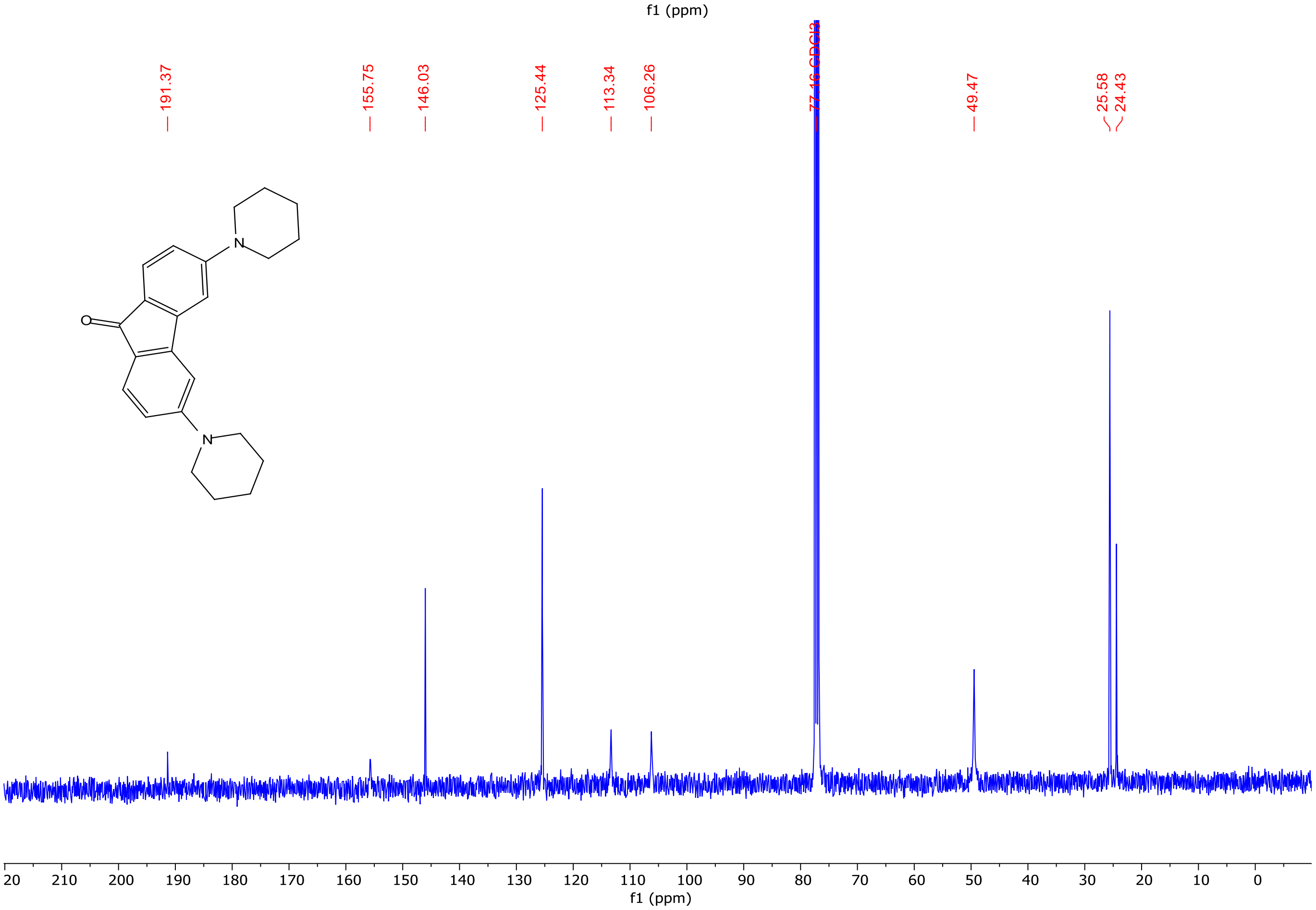

1-(4-methoxyphenyl)piperidine (157)

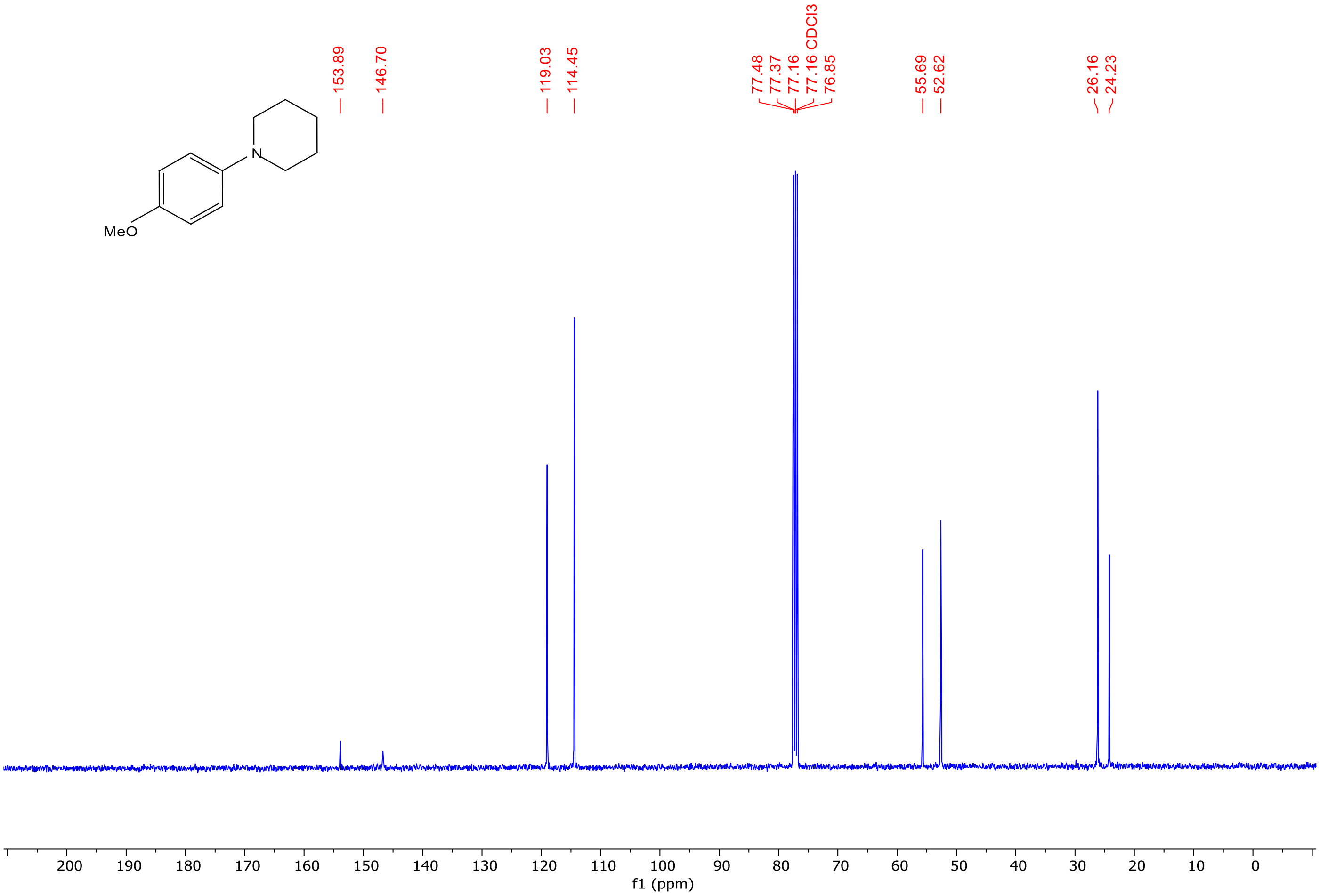

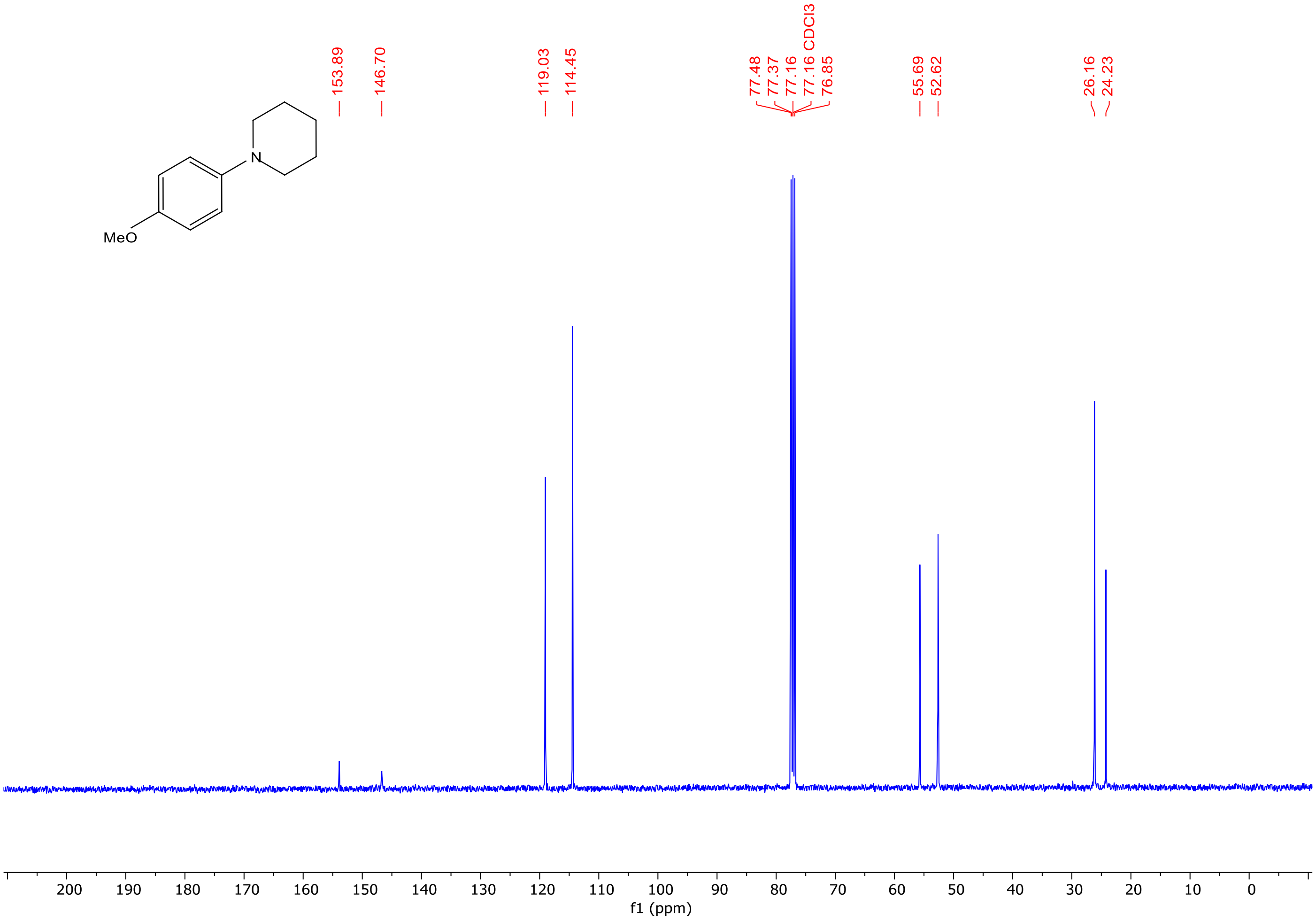

2-(2-fluoro-[1,1'-biphenyl]-4-yl)propanenitrile (158)

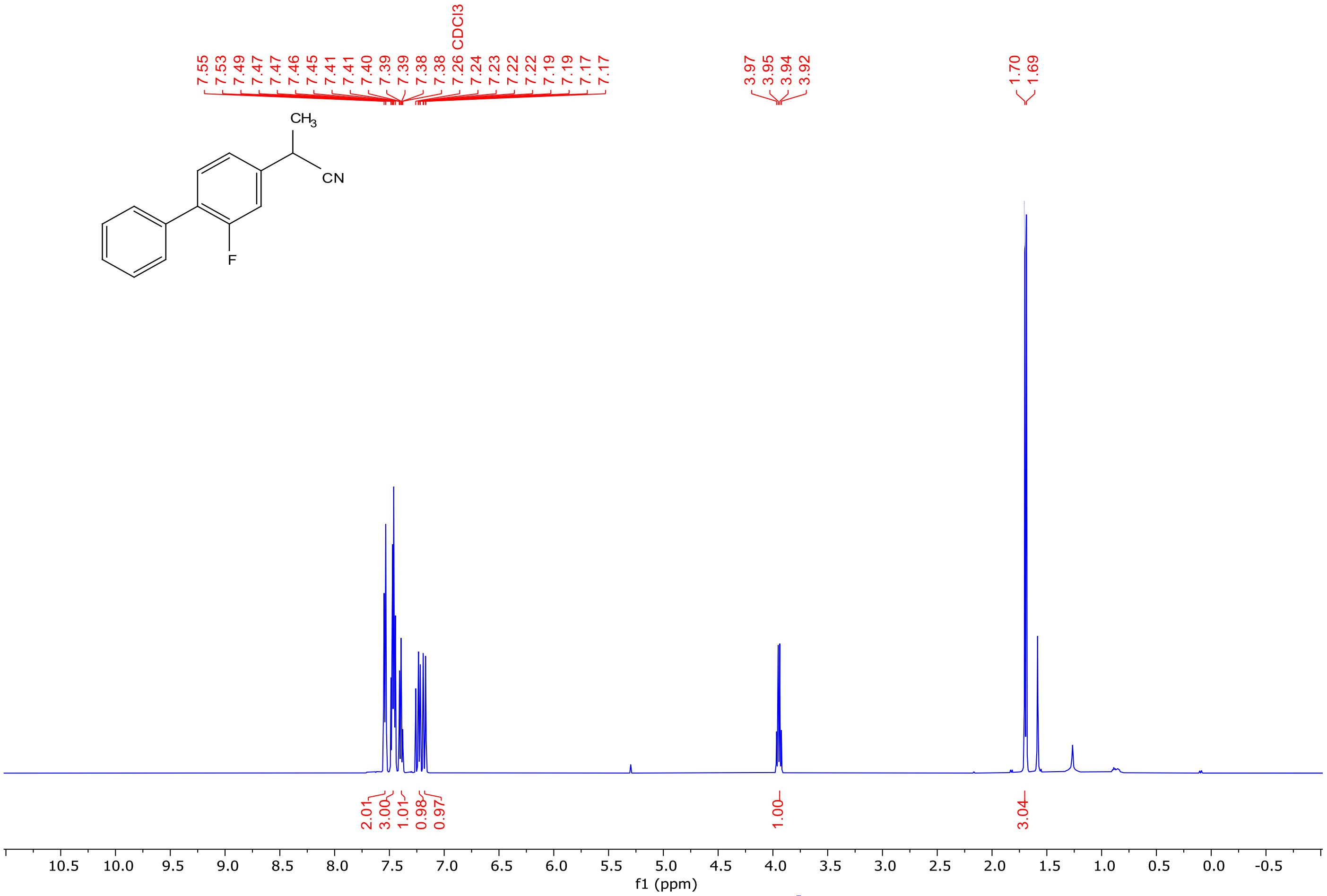

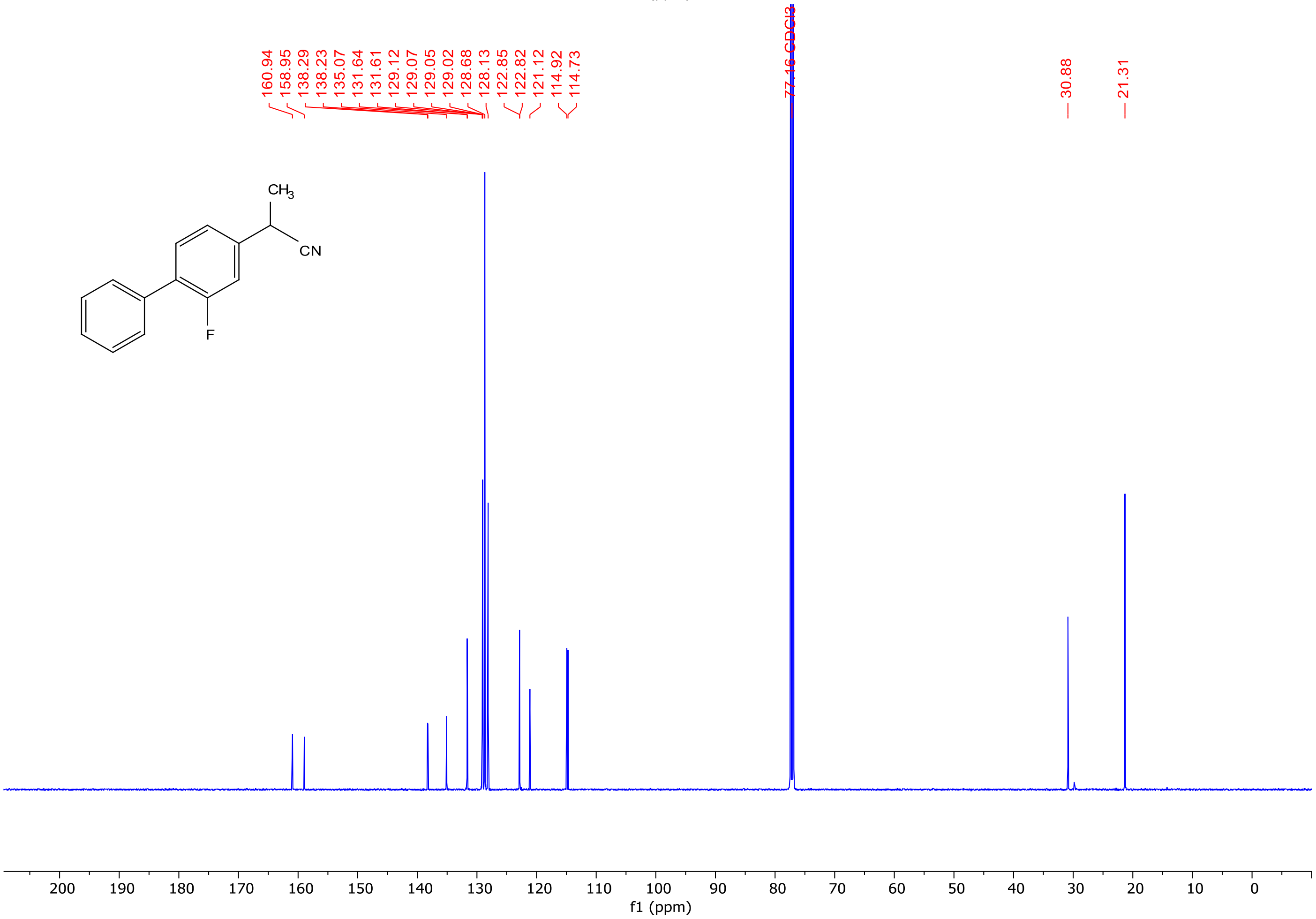

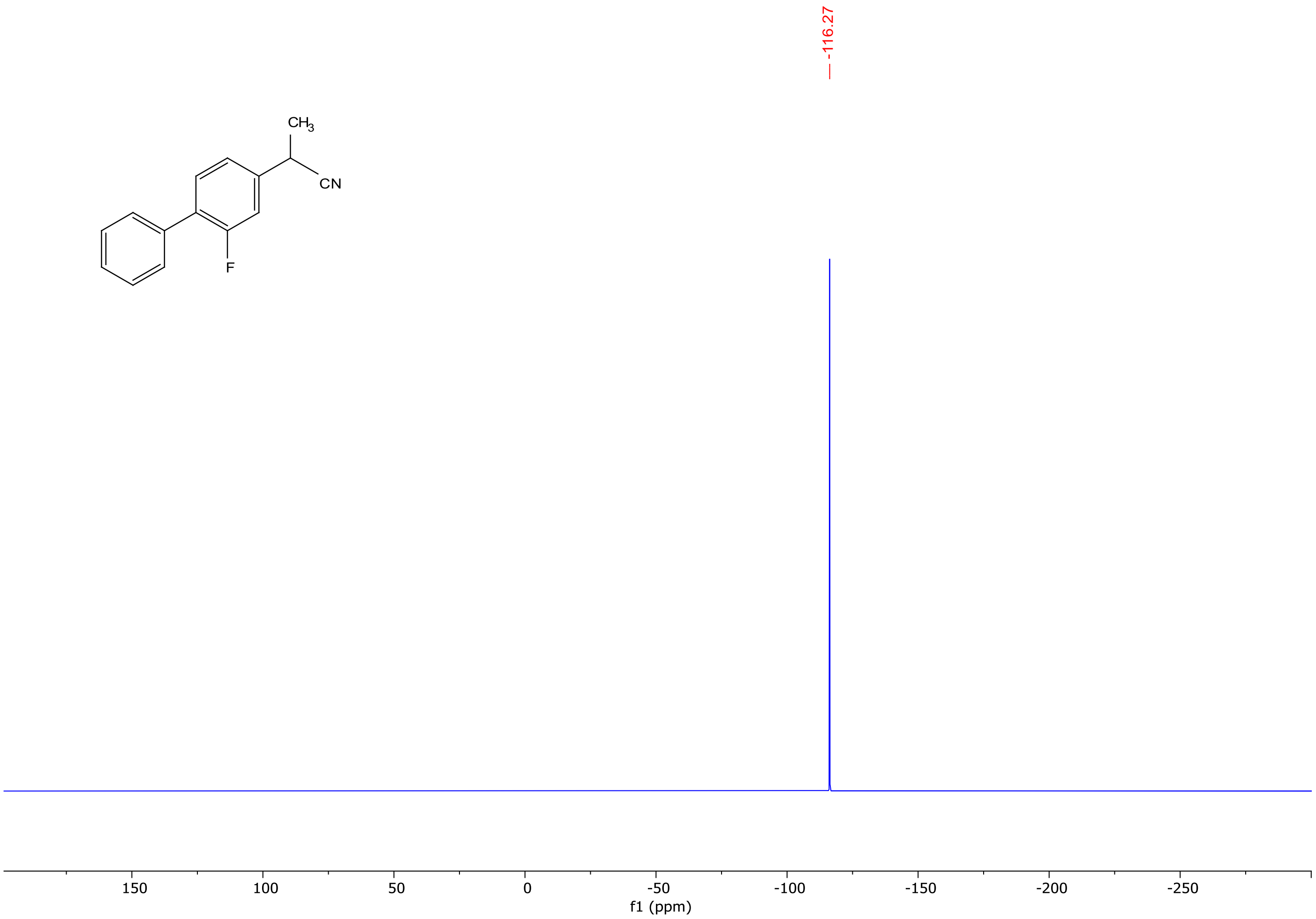
-116.27
CH3
CN
F
150
100
50
0
-50
-100
-150
-200
-250
f1 (ppm)

9. Supplementary references

[1] J. Luo, J. Zhang, *ACS Catal.* **2016**, *6*, 873-877.
[2] S. Grotjahn, B. Konig, *Org. Lett.* **2021**, *23*, 3146-3150.
[3] A. Gevorgyan, K. H. Hopmann, A. Bayer, *Organometallics* **2021**, *41*, 1777-1785.
[4] K. Manna, T. Ganguly, S. Baitalik, R. Jana, *Org. Lett.* **2021**, *23*, 8634-8639.
[5] J. Wei, N. Yang, F. Li, S. Cai, B. Zhang, Z. Cai, *Chemistry* **2024**, *30*, e202401036.
[6] D. Zhang, M. Cai, Z. Bin, Y. Zhang, D. Zhang, L. Duan, *Chem Sci* **2016**, *7*, 3355-3363.
[7] Y. J. Cho, K. S. Yook, J. Y. Lee, *Sci Rep* **2015**, *5*, 7859.
[8] A. B. Rolka, B. Koenig, *Org. Lett.* **2020**, *22*, 5035-5040.
[9] S. Ni, R. Halder, D. Ahmadli, E. J. Reijerse, J. Cornella, T. Ritter, *Nature Catalysis* **2024**, *7*, 733-741.
[10] H. Yue, C. Zhu, M. Rueping, *Angew. Chem. Int. Ed.* **2018**, *57*, 1371-1375.
[11] Y. Kwon, J. Lee, Y. Noh, D. Kim, Y. Lee, C. Yu, J. C. Roldao, S. Feng, J. Gierschner, R. Wannemacher, M. S. Kwon, *Nat. Commun.* **2023**, *14*, 92.
[12] J. Xu, J. Cao, X. Wu, H. Wang, X. Yang, X. Tang, R. W. Toh, R. Zhou, E. K. L. Yeow, J. Wu, *J. Am. Chem. Soc.* **2021**, *143*, 13266-13273.
[13] C. P. Chernowsky, A. F. Chmiel, Z. K. Wickens, *Angew. Chem. Int. Ed* **2021**, *60*, 21418-21425.
[14] H. M. Ko, C. W. Lee, M. S. Kwon, *ChemCatChem* **2023**, *15*, e202300661.
[15] S. Grotjahn, B. Konig, *Org. Lett.* **2021**, *23*, 3146-3150.
[16] K. Donabauer, M. Maity, A. L. Berger, G. S. Huff, S. Crespi, B. Konig, *Chem. Sci.* **2019**, *10*, 5162-5166.
[17] M. Czyz, T. Horngren, A. Kondopoulos, L. Franov, J. Forni, L. N. Pham, M. Coote, A. Polyzos, *10.26434/chemrxiv-2023-qrkjs* **2023**.
[18] E. Pinosa, E. Bassan, S. Cetin, M. Villa, S. Potenti, F. Calogero, A. Gualandi, A. Fermi, P. Ceroni, P. G. Cozzi, *J. Org. Chem.* **2023**, *88*, 6390-6400.
[19] S. J. Horsewill, G. Hierlmeier, Z. Farasat, J. P. Barham, D. J. Scott, *ACS Catal.* **2023**, *13*, 9392-9403.
[20] Bruker, AXS Inc., Madison, Wisconsin, USA, **2012**.
[21] G. M. Sheldrick, *Acta Cryst.* **2015**, *71*, 3-8.
[22] G. M. Sheldrick, *Acta Cryst.* **2015**, *71*, 3-8.
[23] O. V. Dolomanov, L. J. Bourhis, R. J. Gildea, J. A. K. Howard, H. Puschmann, *J. Appl. Cryst.* **2009**, *42*, 339-341.
[24] P. van der Sluis, A. L. Spek, *Acta Crystallographica Section A* **1990**, *46*, 194-201.
[25] N. Kataoka, Q. Shelby, J. P. Stambuli, H. J. F., *J. Org. Chem.* **2002**, *67*, 5553-5566.
[26] S. L. Goldschmid, N. E. Soon Tay, C. L. Joe, B. C. Lainhart, T. C. Sherwood, E. M. Simmons, M. Sezen-Edmonds, T. Rovis, *J. Am. Chem. Soc.* **2022**, *144*, 22409-22415.
[27] X. G., W. Y.-G., *Org. Lett.* **2004**, *6*, 985-987.
[28] T. D. Svejstrup, A. Ruffoni, F. Julia, V. M. Aubert, D. Leonori, *Angew. Chem. Int. Ed* **2017**, *56*, 14948-14952.
[29] J. Yao, L. Yu, W. Duan, C.-J. Li, *Org. Chem. Front.* **2023**, *10*, 524-530.
[30] K. Berg, P. Hegde, V. Pujari, M. Brinkmann, D. Z. Wilkins, T. Parish, D. C. Crick, C. C. Aldrich, *Eur. J. Med. Chem.* **2023**, *249*, 115125.
[31] R. Sun, Y. Qin, D. G. Nocera, *Angew. Chem. Int. Ed.* **2020**, *59*, 9527-9533.
[32] S. Wang, H. Wang, B. König, *Chem* **2021**, *7*, 1653-1665.
[33] B. P. Fors, S. L. Buchwald, *J. Am. Chem. Soc.* **2010**, *132*, 15914-15917.
[34] S. L. Rossler, B. J. Jelier, P. F. Tripet, A. Shemet, G. Jeschke, A. Togni, E. M. Carreira, *Angew. Chem. Int. Ed.* **2019**, *58*, 526-531.
[35] A. Gomtsyan, E. K. Bayburt, R. G. Schmidt, C. S. Surowy, P. Honore, K. C. Marsh, S. M. Hannick, H. A. McDonald, J. M. Wetter, J. P. Sullivan, M. F. Jarvis, C. R. Faltynek, L. C.-H., *J. Med. Chem.* **2008**, *51*, 392-395.
[36] Z. Lu, R. J. Twieg, *Tetrahedron* **2005**, *61*, 903-918.
[37] V. KlimesÏova, M. Svoboda, K. Waisser, M. Pour, J. Kaustova, *Farmaco* **1999**, *54*, 666-672.
[38] O. Navarro, N. Marion, J. Mei, S. P. Nolan, *Chem. Eur. J* **2006**, *12*, 5142-5148.
[39] H. Kim, S. Park, Y. Baek, K. Um, G. U. Han, D. H. Jeon, S. H. Han, P. H. Lee, *J. Org. Chem.* **2018**, *83*, 3486-3496.
[40] C. A. Malapit, M. Borrell, M. W. Milbauer, C. E. Brigham, M. S. Sanford, *J. Am. Chem. Soc.* **2020**, *142*, 5918-5923.
[41] M. S. Oderinde, N. H. Jones, A. Juneau, M. Frenette, B. Aquila, S. Tentarelli, D. W. Robbins, J. W. Johannes, *Angew. Chem. Int. Ed.* **2016**, *55*, 13219-13223.
[42] T. Cao, Y. P. Luo, L. Cheng, J. L. Zhao, Q. S. Jia, S. Zhang, X. W. Liu, *Eur. J. Org. Chem.* **2023**, *26*, e202300494.
[43] J. Li, Z. X. Wang, *Org. Lett.* **2017**, *19*, 3723-3726.
[44] G. Chakraborti, S. Paladhi, T. Mandal, J. Dash, *J. Org. Chem.* **2018**, *83*, 7347-7359.

[45] M. Shigeno, K. Hayashi, K. Nozawa-Kumada, Y. Kondo, *Org. Lett.* **2019**, *21*, 5505-5508.
[46] P. Zamani, J. Ozdemir, Y. Ha, M. Benamara, A. V. Kuchuk, T. Wang, J. Chen, R. Khosropour, M. H. Beyzavi, *Adv. Synth. Catal.* **2018**, *360*, 4372-4380.
[47] R. P. Wexler, P. Nuhant, T. J. Senter, Z. J. Gale-Day, *Org. Lett.* **2019**, *21*, 4540-4543.
[48] F. Glaser, O. S. Wenger, *JACS Au* **2022**, *2*, 1488-1503.
[49] C. H. Lim, M. Kudisch, B. Liu, G. M. Miyake, *J. Am. Chem. Soc.* **2018**, *140*, 7667-7673.
[50] W. P., J. P. Maciejewsk, *Org. Lett.* **2008**, *10*, 4383-4386.
[51] C. Zhu, A. P. Kale, H. Yue, M. Rueping, *JACS Au* **2021**, *1*, 1057-1065.
[52] I. Ghosh, N. Shlapakov, T. A. Karl, J. Duker, M. Nikitin, J. V. Burykina, V. P. Ananikov, B. Konig, *Nature* **2023**, *619*, 87-93.
[53] A. Wimmer, B. Konig, *Org. Lett.* **2019**, *21*, 2740-2744.
[54] F. Ye, F. Berger, H. Jia, J. Ford, A. Wortman, J. Borgel, C. Genicot, T. Ritter, *Angew. Chem. Int. Ed.* **2019**, *58*, 14615-14619.
[55] G. Dannhardt, B. L. Fiebich, J. Schweppenhaueser, *Eur. J. Med. Chem.* **2002**, *37*, 147-161.
[56] K. Kondo, E. Sekimoto, J. Nakao, Y. Murakami, *Tetrahedron* **2000**, *56*, 5843-5856.
[57] M. A. Graham, P. A. Bethel, J. Burgess, G. Fairley, S. C. Glossop, R. D. R. Greenwood, C. D. Jones, S. Lovell, S. Swallow, *Org. Lett.* **2013**, *15*, 6078-6081.
[58] A. A. Berezin, G. Zissimou, C. P. Constantinides, Y. Beldjoudi, J. M. Rawson, P. A. Koutentis, *J. Org. Chem.* **2014**, *79*, 314-327.
[59] X. Li, L. He, H. Chen, W. Wu, H. Jiang, *J. Org. Chem.* **2013**, *78*, 3636-3646.
[60] R. D. Bradley, A. Bahamonde, *Org. Lett.* **2022**, *24*, 7134-7139.
[61] Y.-C. Teo, B. Tan, *Synlett* **2015**, *26*, 1697-1701.
[62] M. Baumann, A. Leslie, T. S. Moody, M. Smyth, S. Wharry, *Org. Process Res. Dev.* **2020**, *25*, 452-456.
[63] L. Li, M. Xue, X. Yan, W. Liu, K. Xu, S. Zhang, *Org. Biomol. Chem.* **2018**, *16*, 4615-4618.
[64] W. Mahy, P. K. Plucinski, C. G. Frost, *Org. Lett.* **2014**, *16*, 5020-5023.
[65] Q. Zhu, S. Che, Z. Luo, Z. Zhao, *Synth. Commun.* **2020**, *50*, 947-957.
[66] M. Kudisch, C. H. Lim, P. Thordarson, G. M. Miyake, *J. Am. Chem. Soc.* **2019**, *141*, 19479-19486.
[67] S. Xu, B. Huang, G. Qiao, Z. Huang, Z. Zhang, Z. Li, P. Wang, Z. Zhang, *Org. Lett.* **2018**, *20*, 5578-5582.
[68] G. L. Frayne, G. M. Green, *Tetrahedron Letters* **2008**, *49*, 7328-7329.
[69] J. Q. Chen, X. Liu, J. Guo, Z. B. Dong, *Eur. J. Org. Chem.* **2020**, *2020*, 2414-2424.
[70] A. Wang, J. Huang, C. Zhao, Y. Fan, J. Qian, Q. Chen, M. He, W. Zhou, *Green Chem.* **2024**, *26*, 353-361.
[71] L. L. Chai, Y. H. Zhao, D. J. Young, X. Lu, H. X. Li, *Org. Lett.* **2022**, *24*, 6908-6913.
[72] Y. Zhu, L. Li, Z. Shen, *Chem. Eur. J* **2015**, *21*, 13246-13252.
[73] L. Liu, C. Nevado, *Organometallics* **2021**, *40*, 2188-2193.
[74] C. A. Malapit, N. Ichiishi, M. S. Sanford, *Org. Lett.* **2017**, *19*, 4142-4145.
[75] L. Yang, Z. Huang, G. Li, W. Zhang, R. Cao, C. Wang, J. Xiao, D. Xue, *Angew. Chem. Int. Ed.* **2018**, *57*, 1968-1972.
[76] P. Zhao, H. Yin, H. Gao, C. Xi, *J. Org. Chem.* **2013**, *78*, 5001-5006.
[77] M. M. Talukder, J. T. Miller, J. M. O. Cue, C. M. Udamulle, A. Bhadran, M. C. Biewer, M. C. Stefan, *Organometallics* **2020**, *40*, 83-94.
[78] M. Jouffroy, C. B. Kelly, G. A. Molander, *Org. Lett.* **2016**, *18*, 876-879.
[79] L. Y. Lam, C. Ma, *Org. Lett.* **2021**, *23*, 6164-6168.
[80] K. D. Jones, D. J. Power, D. Bierer, K. M. Gericke, S. G. Stewart, *Org. Lett.* **2018**, *20*, 208-211.
[81] M. Jiang, H. Li, H. Yang, H. Fu, *Angew. Chem. Int. Ed.* **2017**, *56*, 874-879.
[82] A. Nandy, I. Kazi, S. Guha, G. Sekar, *J. Org. Chem.* **2021**, *86*, 2570-2581.
[83] X. Wang, G. D. Cuny, T. Noel, *Angew. Chem. Int. Ed.* **2013**, *52*, 7860-7864.
[84] Y. Zhang, S. Xia, W. X. Shi, B. Lin, X. C. Su, W. Lu, X. Wu, X. Wang, X. Lu, M. Yan, X. J. Zhang, *Org. Lett.* **2022**, *24*, 7961-7966.
[85] L. Chen, J. Liang, Z. y. Chen, J. Chen, M. Yan, X. j. Zhang, *Adv. Synth. Catal.* **2019**, *361*, 956-960.
[86] K. Gulbe, M. R. Turks, *J. Org. Chem.* **2020**, *85*, 5660-5669.
[87] J. C. Hethcox, H. C. Johnson, J. Kim, X. Wang, L. Cheng, Y. Cao, M. Tan, D. A. DiRocco, Y. Ji, *Angew. Chem. Int. Ed.* **2023**, *62*, e202217623.
[88] C. Zhu, H. Yue, P. Nikolaienko, M. Rueping, *CCS Chem.* **2020**, *2*, 179-190.
[89] A. Jati, K. Dey, M. Nurhuda, M. A. Addicoat, R. Banerjee, B. Maji, *J. Am. Chem. Soc.* **2022**, *144*, 7822-7833.
[90] M. Shigeno, K. Hayashi, K. Nozawa-Kumada, Y. Kondo, *Chem. Eur. J* **2019**, *25*, 6077-6081.
[91] P. H. Gehrtz, V. Geiger, T. Schmidt, L. Srsan, I. Fleischer, *Org. Lett.* **2019**, *21*, 50-55.
[92] M. S. S. Bandaru, S. Bhilare, N. Chrysochos, V. Gayakhe, I. Trentin, C. Schulzke, A. R. Kapdi, *Org. Lett.* **2018**, *20*, 473-476.

[93] J. C. Vantourout, R. P. Law, A. Isidro-Llobet, S. J. Atkinson, A. J. B. Watson, *The Journal of Organic Chemistry* **2016**, *81*, 3942-3950.
[94] E. Papaplioura, J. Templ, N. Wildhack, M. Schnürch, *Eur. J. Org. Chem.* **2024**, *27*.
