# Supplementary material for: Predictive, Data‐Driven Design of Red‐Light Photoredox Catalysts for C─Heteroatom Bond Formation
Source: Angew Chem Int Ed Engl. 2026 Jan 19;65(9):e26086. doi: 10.1002/anie.202526086 (PMC12929924; doi:10.1002/anie.202526086)

checkCIF/PLATON report

Structure factors have been supplied for datablock(s) gia705_0m_corr

THIS REPORT IS FOR GUIDANCE ONLY. IF USED AS PART OF A REVIEW PROCEDURE FOR PUBLICATION, IT SHOULD NOT REPLACE THE EXPERTISE OF AN EXPERIENCED CRYSTALLOGRAPHIC REFEREE.

No syntax errors found. CIF dictionary Interpreting this report

Datablock: gia705_0m_corr

Bond precision: C-C = 0.0019 A Wavelength=1.34138

Cell:	a=13.2915(16)	b=12.2807(15)	c=34.425(4)
	alpha=90	beta=100.043(3)	gamma=90
Temperature:	100 K		

	Calculated	Reported
Volume	5533.1(11)	5533.0(12)
Space group	P 21/c	P 1 21/c 1
Hall group	-P 2ybc	-P 2ybc
Moiety formula	C62 H56 N6 O8, C H2 Cl2	C H2 Cl2, C62 H56 N6 O8
Sum formula	C63 H58 Cl2 N6 O8	C63 H58 Cl2 N6 O8
Mr	1098.05	1098.05
Dx,g cm-3	1.318	1.318
Z	4	4
Mu (mm-1)	1.024	1.024
F000	2304.0	2304.0
F000'	2311.55	
h,k,lmax	19,17,49	18,17,48
Nref	16980	15438
Tmin,Tmax	0.903,0.903	0.559,0.753
Tmin'	0.903	

Correction method= # Reported T Limits: Tmin=0.559 Tmax=0.753
AbsCorr = MULTI-SCAN

Data completeness= 0.909 Theta(max)= 73.766

R(reflections)= 0.0625(14275) wR2(reflections)= 0.1675(15438)

S = 1.124 Npar= 720

The following ALERTS were generated. Each ALERT has the format
test-name_ALERT_alert-type_alert-level.
Click on the hyperlinks for more details of the test.

Alert level C

```
PLAT029_ALERT_3_C _diffrn_measured_fraction_theta_full value Low .      0.963 Why?
PLAT042_ALERT_1_C Calc. and Reported MoietyFormula Strings  Differ     Please Check
              Calc: C62 H56 N6 O8, C H2 Cl2
              Rep.: C H2 Cl2, C62 H56 N6 O8
PLAT767_ALERT_4_C INS Embedded LIST 6 Instruction Should be LIST 4     Please Check
PLAT906_ALERT_3_C Large K Value in the Analysis of Variance ......      4.325 Check
PLAT911_ALERT_3_C Missing FCF Refl Between Thmin & STh/L=    0.600        373 Report
                0  4  0,   0 10  0,   1  3  0,   1  7  0,   1  9  0,   1 10  0,
                2  0  0,   4  4  0,   5  5  0,   5  6  0,   5  7  0,   5  9  0,
                6  7  0,   6  8  0,   8  9  0,   9 11  0, -12  4  1,  -8  7  1,
               -8 12  1,  -5  9  1,  -4  8  1,  -4 12  1,  -3  3  1,  -3  7  1,
               -3 10  1,  -2 14  1,  -1  8  1,   0  6  1,   1  8  1,   1 10  1,
                2 10  1,   3 10  1,   4  4  1,   5  5  1,   5  7  1,   6  5  1,
                6  7  1,   6 10  1,   6 12  1,   7  9  1,   8  9  1,  -9  5  2,
               -9  6  2,  -8  4  2,  -8  6  2,  -8  7  2,  -7 12  2,  -7 13  2,
               -6  9  2,  -5 10  2,  -5 11  2,  -4  4  2,  -4  8  2,  -3  3  2,
               -1  8  2,   0  6  2,   0 11  2,   1  8  2,   1 11  2,   2  2  2,
                3  2  2,   3  4  2,   3 10  2,   4  2  2,   4 10  2,   4 14  2,
                5  5  2,   5  6  2,   5  7  2,   6 13  2,   7  6  2,   7 13  2,
                9  5  2,   9  6  2, -11  3  3, -10  4  3, -10  7  3,  -8  8  3,
               -7 11  3,  -6 10  3,  -4 14  3,  -1  3  3,  -1  4  3,   1  2  3,
                1  8  3,   3  1  3,   4  8  3,   5  7  3,   7 10  3,   8  7  3,
                8 11  3,   9  5  3,   9  6  3,  10  5  3, -10  6  4,  -9  7  4,
PLAT934_ALERT_3_C Number of (Iobs-Icalc)/Sigma(W) > 10 Outliers ..          1 Check
               -6  9 13,
PLAT977_ALERT_2_C Check Negative Difference Density on H51       .      -0.32 eA-3
PLAT977_ALERT_2_C Check Negative Difference Density on H80B      .      -0.31 eA-3
```

Alert level G

```
ABSMU01_ALERT_1_G  Calculation of _exptl_absorpt_correction_mu
                not performed for this radiation type.
PLAT230_ALERT_2_G Hirshfeld Test Diff for    C4       --C7       .        5.1 s.u.
PLAT432_ALERT_2_G Short Inter X...Y Contact  O70      ..C45      .       2.94 Ang.
                                           1-x,-1/2+y,3/2-z  =      2_646 Check
PLAT883_ALERT_1_G Absent Datum for _atom_sites_solution_primary ..     Please Do !
PLAT912_ALERT_4_G Missing # of FCF Reflections Above STh/L=  0.600       1163 Note
PLAT913_ALERT_3_G Missing # of Very Strong Reflections in FCF ....          2 Note
               -1  3  3,  -3  0  4,
PLAT933_ALERT_2_G Number of HKL-OMIT Records in Embedded .res File         62 Note
              -10  7  3, -10  8  7,  -9  7  4,  -8  6 44,  -8  8 13,  -8  9 11,
               -8  9 12,  -8 10 37,  -7  7 13,  -7  8 11,  -7 12  9,  -6  8  4,
               -6  8  7,  -6  9  2,  -6 10  3,  -5  8  9,  -5  9  8,  -5  9 10,
               -4  7  7,  -4  8  8,  -4  8 20,  -4  9  6,  -4 10 12,  -4 10 16,
               -4 11 10,  -4 12  4,  -3  8 11,  -2  1  6,  -1  2 42,  -1  3  3,
               -1  9 12,  -1 10  5,   1  0  4,   1  2  3,   1 10 20,   1 11  6,
                2  0  0,   2  4 40,   3  1  3,   4  7  5,   4  7  6,   4  8  3,
                5  5  0,   5  8  7,   5  8 10,   6  5  4,   6  7  0,   6  7  5,
                6  8 11,   6  9  7,
PLAT969_ALERT_5_G The 'Henn et al.' R-Factor-gap value ..........      5.386 Note
```

```
             Predicted wR2: Based on SigI**2  3.11 or SHELX Weight 14.90
PLAT978_ALERT_2_G Number C-C Bonds with Positive Residual Density.         8 Info
PLAT992_ALERT_5_G Repd & Actual _reflns_number_gt Values Differ by         2 Check
```

```
   0 ALERT level A = Most likely a serious problem - resolve or explain
   0 ALERT level B = A potentially serious problem, consider carefully
   8 ALERT level C = Check. Ensure it is not caused by an omission or oversight
  10 ALERT level G = General information/check it is not something unexpected

   3 ALERT type 1 CIF construction/syntax error, inconsistent or missing data
   6 ALERT type 2 Indicator that the structure model may be wrong or deficient
   5 ALERT type 3 Indicator that the structure quality may be low
   2 ALERT type 4 Improvement, methodology, query or suggestion
   2 ALERT type 5 Informative message, check
```

It is advisable to attempt to resolve as many as possible of the alerts in all categories. Often the minor alerts point to easily fixed oversights, errors and omissions in your CIF or refinement strategy, so attention to these fine details can be worthwhile. In order to resolve some of the more serious problems it may be necessary to carry out additional measurements or structure refinements. However, the purpose of your study may justify the reported deviations and the more serious of these should normally be commented upon in the discussion or experimental section of a paper or in the "special_details" fields of the CIF. checkCIF was carefully designed to identify outliers and unusual parameters, but every test has its limitations and alerts that are not important in a particular case may appear. Conversely, the absence of alerts does not guarantee there are no aspects of the results needing attention. It is up to the individual to critically assess their own results and, if necessary, seek expert advice.

Publication of your CIF in IUCr journals

A basic structural check has been run on your CIF. These basic checks will be run on all CIFs submitted for publication in IUCr journals (*Acta Crystallographica*, *Journal of Applied Crystallography*, *Journal of Synchrotron Radiation*); however, if you intend to submit to *Acta Crystallographica Section C* or *E* or *IUCrData*, you should make sure that full publication checks are run on the final version of your CIF prior to submission.

Publication of your CIF in other journals

Please refer to the *Notes for Authors* of the relevant journal for any special instructions relating to CIF submission.

PLATON version of 02/02/2025; check.def file version of 02/02/2025

Datablock gia705_0m_corr - ellipsoid plot

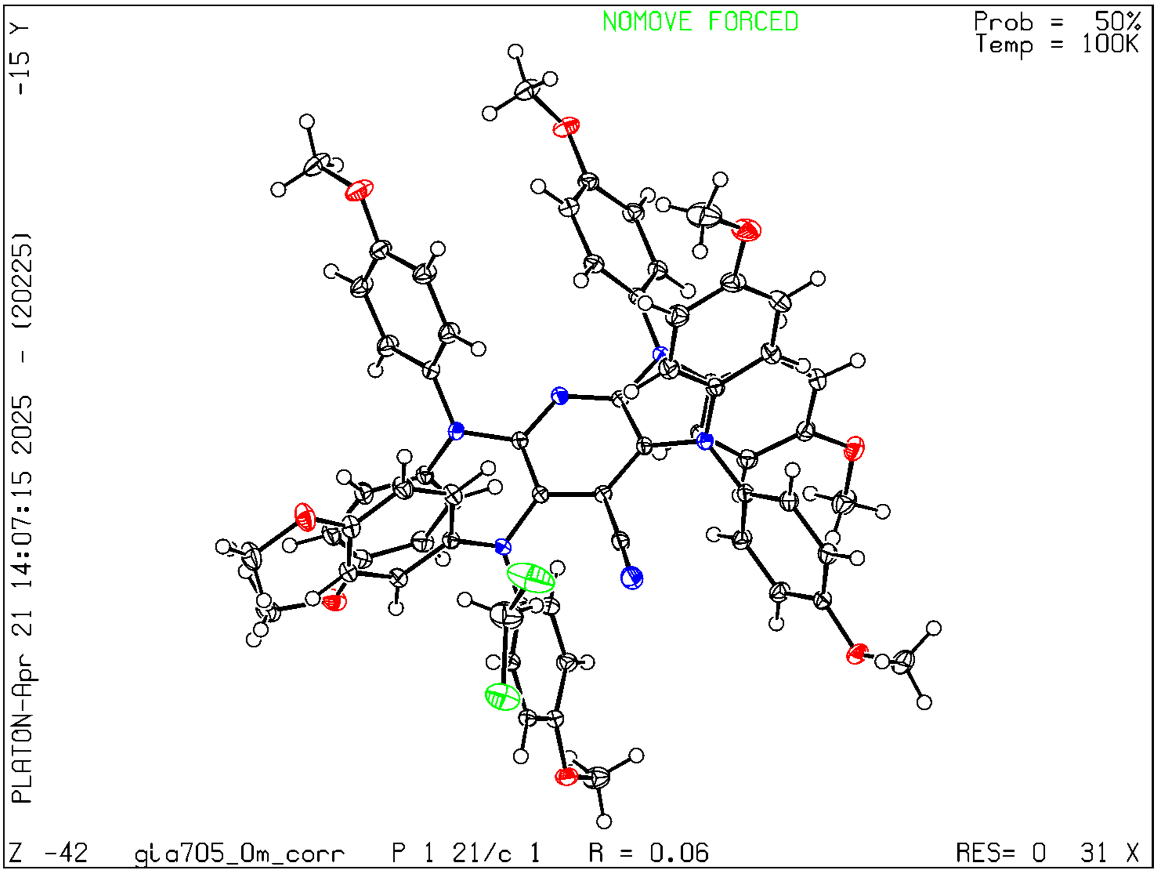

Supplement: Supplementary file 2 — Supporting Information [file ANIE-65-e26086-s001.zip › 2370576.pdf]
