# Supplementary material for: Predictive, Data‐Driven Design of Red‐Light Photoredox Catalysts for C─Heteroatom Bond Formation
Source: Angew Chem Int Ed Engl. 2026 Jan 19;65(9):e26086. doi: 10.1002/anie.202526086 (PMC12929924; doi:10.1002/anie.202526086)

checkCIF/PLATON report

Structure factors have been supplied for datablock(s) amir717_fin

THIS REPORT IS FOR GUIDANCE ONLY. IF USED AS PART OF A REVIEW PROCEDURE FOR PUBLICATION, IT SHOULD NOT REPLACE THE EXPERTISE OF AN EXPERIENCED CRYSTALLOGRAPHIC REFEREE.

No syntax errors found. CIF dictionary Interpreting this report

Datablock: amir717_fin

```
Bond precision:    C-C = 0.0039 A            Wavelength=1.34139

Cell:              a=11.9545(10)      b=21.2188(19)       c=24.139(2)
                   alpha=90           beta=93.819(4)      gamma=90
Temperature:       100 K

                  Calculated                     Reported
Volume            6109.5(9)                      6109.5(9)
Space group       P 21/c                         P 1 21/c 1
Hall group        -P 2ybc                        -P 2ybc
Moiety formula    C63 H57 N5 O8 [+ solvent]      C63 H57 N5 O8, 1.5[C4H8O2]
Sum formula       C63 H57 N5 O8 [+ solvent]      C69 H69 N5 O11
Mr                1012.14                        1144.29
Dx,g cm-3         1.100                          1.244
Z                 4                              4
Mu (mm-1)         0.375                          0.440
F000              2136.0                         2424.0
F000'             2140.84
h,k,lmax          16,30,34                       16,30,34
Nref              18355                          18104
Tmin,Tmax         0.900,0.916                    0.593,0.754
Tmin'             0.876

Correction method= # Reported T Limits: Tmin=0.593 Tmax=0.754
AbsCorr = MULTI-SCAN

Data completeness= 0.986            Theta(max)= 72.351

                                                   wR2(reflections)=
R(reflections)= 0.1138( 15118)                     0.3289( 18104)

S = 1.034                    Npar= 712
```

The following ALERTS were generated. Each ALERT has the format
test-name_ALERT_alert-type_alert-level.
Click on the hyperlinks for more details of the test.

Alert level B

```
PLAT097_ALERT_2_B Large Reported Max.  (Positive) Residual Density       1.48 eA-3
```

Author Response: Probably, residual peak of the solvent not covered my solvent mask

Alert level C

```
DIFMN02_ALERT_2_C  The minimum difference density is < -0.1*ZMAX*0.75
            _refine_diff_density_min given =     -0.699
            Test value =     -0.600
DIFMN03_ALERT_1_C  The minimum difference density is < -0.1*ZMAX*0.75
            The relevant atom site should be identified.
DIFMX02_ALERT_1_C  The maximum difference density is > 0.1*ZMAX*0.75
            The relevant atom site should be identified.
PLAT082_ALERT_2_C High R1 Value .................................        0.11 Report
PLAT084_ALERT_3_C High wR2 Value (i.e. > 0.25) ..................        0.33 Report
PLAT094_ALERT_2_C Ratio of Maximum / Minimum Residual Density ....       2.12 Report
PLAT098_ALERT_2_C Large Reported Min.  (Negative) Residual Density      -0.70 eA-3
PLAT250_ALERT_2_C Large U3/U1 Ratio for <U(i,j)> Tensor(Resd    1)        2.1 Note
PLAT906_ALERT_3_C Large K Value in the Analysis of Variance ......      5.044 Check
PLAT906_ALERT_3_C Large K Value in the Analysis of Variance ......      2.590 Check
PLAT911_ALERT_3_C Missing FCF Refl Between Thmin & STh/L=    0.600         40 Report
                2  0  0,    4  0  0,    6  0  0,    5  2  0,    0  4  0,    0  6  0,
                0  8  0,    0 10  0,    1 11  0,    0 14  0,    2 15  0,    0 20  0,
                0  7  1,    0  8  1,    0  9  1,    1 11  1,    0 13  1,    2 13  1,
               -1 14  1,   -2  0  2,    0 11  2,   -1 12  2,    0 14  2,   -8 11  3,
                0 12  3,    0 13  3,    1 15  3,    0 18  3,    1 20  3,   -2 11  4,
                1 12  4,    0 16  4,   -7 10  5,    0 18  5,    1 20  5,    8  0  6,
               -1  4  6,   -1 17  6,    1  1  7,    1 15 11,
PLAT918_ALERT_3_C Reflection(s) with I(obs) much Smaller I(calc) .         12 Check
PLAT977_ALERT_2_C Check Negative Difference Density on H1        .      -0.32 eA-3
```

Alert level G

```
FORMU01_ALERT_2_G  There is a discrepancy between the atom counts in the
            _chemical_formula_sum and the formula from the _atom_site* data.
            Atom count from _chemical_formula_sum:C69 H69 N5 O11
            Atom count from the _atom_site data:  C63 H57 N5 O8
ABSMU01_ALERT_1_G  Calculation of _exptl_absorpt_correction_mu
                not performed for this radiation type.
CELLZ01_ALERT_1_G Difference between formula and atom_site contents detected.
CELLZ01_ALERT_1_G ALERT: Large difference may be due to a
            symmetry error - see SYMMG tests
           From the CIF: _cell_formula_units_Z     4
           From the CIF: _chemical_formula_sum  C69 H69 N5 O11
           TEST: Compare cell contents of formula and atom_site data

           atom    Z*formula  cif sites diff
```

```
          C        276.00    252.00   24.00
          H        276.00    228.00   48.00
          N         20.00     20.00    0.00
          O         44.00     32.00   12.00
PLAT002_ALERT_2_G Number of Distance or Angle Restraints on AtSite          6 Note
PLAT003_ALERT_2_G Number of Uiso or U(i,j)  Restrained non-H-Atoms          2 Report
PLAT041_ALERT_1_G Calc. and Reported SumFormula    Strings  Differ     Please Check
              Calc: C63 H57 N5 O8
              Rep.: C69 H69 N5 O11
PLAT042_ALERT_1_G Calc. and Reported MoietyFormula Strings  Differ     Please Check
              Calc: C63 H57 N5 O8
              Rep.: C63 H57 N5 O8, 1.5[C4H8O2]
PLAT051_ALERT_1_G Mu(calc) and Mu(cif) Ratio Differs from 1.0 by .      14.76 %
PLAT072_ALERT_2_G SHELXL First  Parameter in WGHT  Unusually Large       0.15 Report
PLAT083_ALERT_2_G SHELXL Second Parameter in WGHT  Unusually Large      10.55 Why ?
PLAT176_ALERT_4_G The CIF-Embedded .res File Contains SADI Records          2 Report
PLAT177_ALERT_4_G The CIF-Embedded .res File Contains DELU Records          1 Report
PLAT191_ALERT_3_G A Non-default SADI Restraint Value has been used     0.0010 Report
PLAT191_ALERT_3_G A Non-default SADI Restraint Value has been used     0.0010 Report
PLAT192_ALERT_3_G A Non-default DELU Restraint Value for First Par     0.0010 Report
PLAT192_ALERT_3_G A Non-default DELU Restraint Value for SecondPar     0.0010 Report
PLAT230_ALERT_2_G Hirshfeld Test Diff for    C1       --C7A      .        5.2 s.u.
PLAT301_ALERT_3_G Main Residue  Disorder ..............(Resd    1)         3% Note
PLAT606_ALERT_4_G Solvent Accessible VOID(S) in Structure ........          ! Info
PLAT860_ALERT_3_G Number of Least-Squares Restraints .............          3 Note
PLAT868_ALERT_4_G ALERTS Due to the Use of _smtbx_masks Suppressed          ! Info
PLAT883_ALERT_1_G Absent Datum for _atom_sites_solution_primary ..     Please Do !
PLAT912_ALERT_4_G Missing # of FCF Reflections Above STh/L=  0.600        209 Note
PLAT933_ALERT_2_G Number of HKL-OMIT Records in Embedded .res File          2 Note
               -1  4  6,  -1 17  6,
PLAT969_ALERT_5_G The 'Henn et al.' R-Factor-gap value ..........      9.128 Note
              Predicted wR2: Based on SigI**2  3.60 or SHELX Weight 31.81
PLAT978_ALERT_2_G Number C-C Bonds with Positive Residual Density.          8 Info
PLAT984_ALERT_1_G The  C-f'=    0.0148 Deviates from the B&C-Value     0.0137 Check
PLAT984_ALERT_1_G The  N-f'=    0.0253 Deviates from the B&C-Value     0.0241 Check
PLAT984_ALERT_1_G The  O-f'=    0.0412 Deviates from the B&C-Value     0.0389 Check
PLAT992_ALERT_5_G Repd & Actual _reflns_number_gt Values Differ by          3 Check
```

---

0 **ALERT level A** = Most likely a serious problem - resolve or explain
1 **ALERT level B** = A potentially serious problem, consider carefully
13 **ALERT level C** = Check. Ensure it is not caused by an omission or oversight
31 **ALERT level G** = General information/check it is not something unexpected

12 ALERT type 1 CIF construction/syntax error, inconsistent or missing data
15 ALERT type 2 Indicator that the structure model may be wrong or deficient
11 ALERT type 3 Indicator that the structure quality may be low
5 ALERT type 4 Improvement, methodology, query or suggestion
2 ALERT type 5 Informative message, check

It is advisable to attempt to resolve as many as possible of the alerts in all categories. Often the minor alerts point to easily fixed oversights, errors and omissions in your CIF or refinement strategy, so attention to these fine details can be worthwhile. In order to resolve some of the more serious problems it may be necessary to carry out additional measurements or structure refinements. However, the purpose of your study may justify the reported deviations and the more serious of these should normally be commented upon in the discussion or experimental section of a paper or in the "special_details" fields of the CIF. checkCIF was carefully designed to identify outliers and unusual parameters, but every test has its limitations and alerts that are not important in a particular case may appear. Conversely, the absence of alerts does not guarantee there are no aspects of the results needing attention. It is up to the individual to critically assess their own results and, if necessary, seek expert advice.

Publication of your CIF in IUCr journals

A basic structural check has been run on your CIF. These basic checks will be run on all CIFs submitted for publication in IUCr journals (*Acta Crystallographica*, *Journal of Applied Crystallography*, *Journal of Synchrotron Radiation*); however, if you intend to submit to *Acta Crystallographica Section C* or *E* or *IUCrData*, you should make sure that full publication checks are run on the final version of your CIF prior to submission.

Publication of your CIF in other journals

Please refer to the *Notes for Authors* of the relevant journal for any special instructions relating to CIF submission.

PLATON version of 02/02/2025; check.def file version of 02/02/2025

Datablock amir717_fin - ellipsoid plot

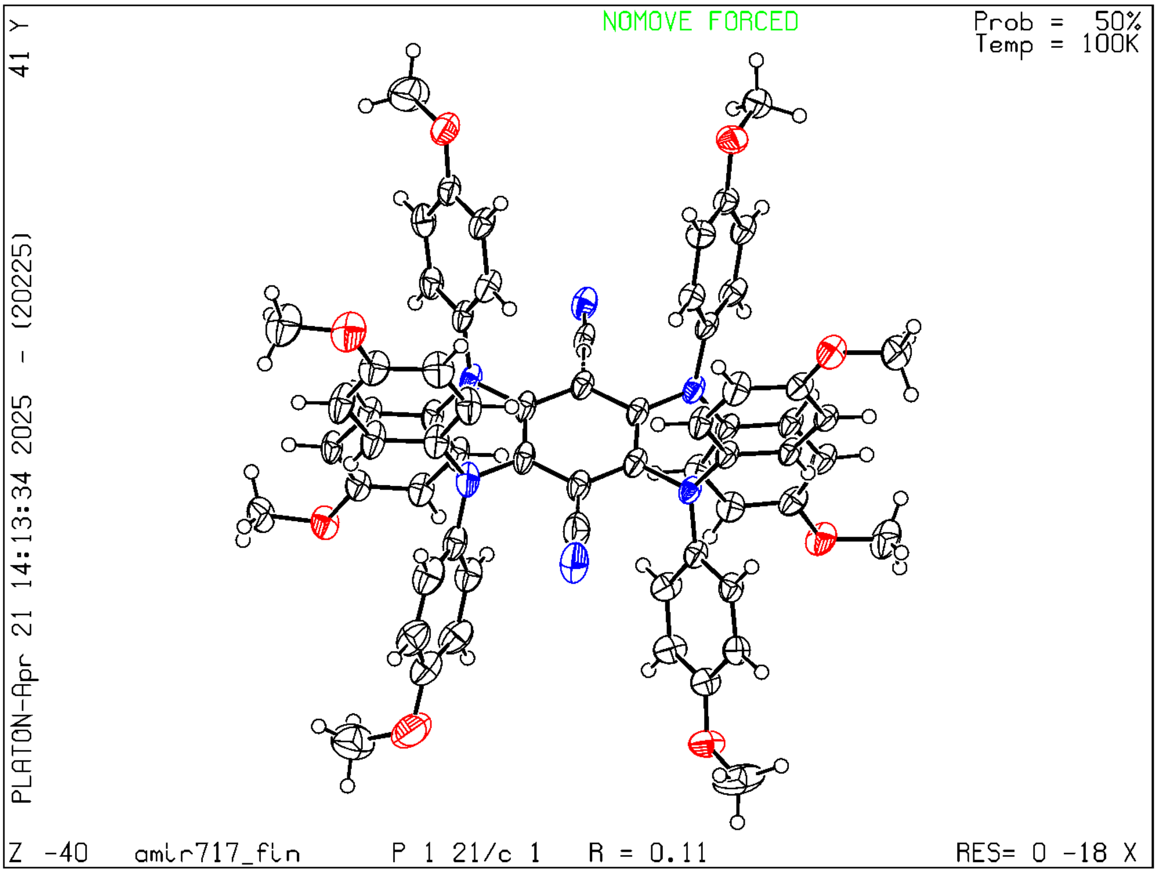

Supplement: Supplementary file 2 — Supporting Information [file ANIE-65-e26086-s001.zip › 2370594.pdf]
