# Supplementary material for: Predictive, Data‐Driven Design of Red‐Light Photoredox Catalysts for C─Heteroatom Bond Formation
Source: Angew Chem Int Ed Engl. 2026 Jan 19;65(9):e26086. doi: 10.1002/anie.202526086 (PMC12929924; doi:10.1002/anie.202526086)

checkCIF/PLATON report

Structure factors have been supplied for datablock(s) sgk19_0m

THIS REPORT IS FOR GUIDANCE ONLY. IF USED AS PART OF A REVIEW PROCEDURE FOR PUBLICATION, IT SHOULD NOT REPLACE THE EXPERTISE OF AN EXPERIENCED CRYSTALLOGRAPHIC REFEREE.

No syntax errors found. CIF dictionary Interpreting this report

Datablock: sgk19_0m

Bond precision:	C-C = 0.0032 A		Wavelength=1.34138
Cell:	a=12.9384(10)	b=13.5693(10)	c=17.7293(14)
	alpha=72.955(4)	beta=73.052(3)	gamma=72.394(3)
Temperature:	100 K		

	Calculated	Reported
Volume	2765.4(4)	2765.4(4)
Space group	P -1	P -1
Hall group	-P 1	-P 1
Moiety formula	C67 H64 N6 O9	C67 H64 N6 O9
Sum formula	C67 H64 N6 O9	C67 H64 N6 O9
Mr	1097.25	1097.24
Dx,g cm-3	1.318	1.318
Z	2	2
Mu (mm-1)	0.453	0.453
F000	1160.0	1160.0
F000'	1162.64	
h,k,lmax	16,16,22	16,16,22
Nref	11319	11275
Tmin,Tmax	0.886,0.942	0.664,0.752
Tmin'	0.886	

Correction method= # Reported T Limits: Tmin=0.664 Tmax=0.752
AbsCorr = MULTI-SCAN

Data completeness= 0.996 Theta(max)= 56.999

R(reflections)= 0.0568(8441) wR2(reflections)= 0.1557(11275)

S = 1.112 Npar= 823

The following ALERTS were generated. Each ALERT has the format

test-name_ALERT_alert-type_alert-level.

Click on the hyperlinks for more details of the test.

Alert level B

```
PLAT097_ALERT_2_B Large Reported Max.  (Positive) Residual Density       0.88 eA-3
```

Author Response: The reason for the presence of this peak may be due to insufficiently good quality of the crystal. It cannot be modeled as methoxy group disorder - too close to the oxygen

Alert level C

```
DIFMX02_ALERT_1_C  The maximum difference density is > 0.1*ZMAX*0.75
            The relevant atom site should be identified.
RINTA01_ALERT_3_C  The value of Rint is greater than 0.12
            Rint given   0.139
PLAT094_ALERT_2_C Ratio of Maximum / Minimum Residual Density ....       2.27 Report
PLAT220_ALERT_2_C NonSolvent   Resd 1  C   Ueq(max)/Ueq(min) Range        3.2 Ratio
PLAT906_ALERT_3_C Large K Value in the Analysis of Variance ......     16.569 Check
PLAT906_ALERT_3_C Large K Value in the Analysis of Variance ......      4.700 Check
PLAT911_ALERT_3_C Missing FCF Refl Between Thmin & STh/L=    0.600         25 Report
                0 -2  1,   2  1  1,  -1 13  1,   6  1  2,  -1-10  3,   1 -3  4,
               -3 14  5,   2  2  6,   2  3  6,   6  3  6,  -3-13  7,   2-10 11,
               13  9 12,  13 10 13,  -4 -6 14,  13  9 14,   2 -6 15,  -1  2 16,
                0 10 16,  12 10 16,   1  0 17,   3 -4 18,   0  3 18,   1  6 19,
                1  4 20,
```

Alert level G

```
ABSMU01_ALERT_1_G  Calculation of _exptl_absorpt_correction_mu
                not performed for this radiation type.
PLAT002_ALERT_2_G Number of Distance or Angle Restraints on AtSite         16 Note
PLAT003_ALERT_2_G Number of Uiso or U(i,j)  Restrained non-H-Atoms         16 Report
PLAT020_ALERT_3_G The Value of Rint is Greater Than 0.12 .........      0.139 Report
PLAT176_ALERT_4_G The CIF-Embedded .res File Contains SADI Records         10 Report
PLAT178_ALERT_4_G The CIF-Embedded .res File Contains SIMU Records          8 Report
PLAT301_ALERT_3_G Main Residue  Disorder .............(Resd    1)        10% Note
PLAT860_ALERT_3_G Number of Least-Squares Restraints .............         58 Note
PLAT883_ALERT_1_G Absent Datum for _atom_sites_solution_primary ..     Please Do !
PLAT912_ALERT_4_G Missing # of FCF Reflections Above STh/L=  0.600         19 Note
PLAT933_ALERT_2_G Number of HKL-OMIT Records in Embedded .res File         33 Note
               -6 -1 17,  -5-11 10,  -5  3 18,  -5 12 12,  -4-10 12,  -4 -6 14,
               -3-13  7,  -3-13  8,  -3 14  5,  -1-10  3,  -1  2 16,  -1 13  1,
                0  3 18,   0 10 16,   1 -3  4,   1  0 17,   1  4 20,   1  4 21,
                1  5 21,   1  6 19,   2-10 11,   2 -6 15,   2  5 21,   2 12 18,
                2 16  2,   3-12  9,   3 -4 18,   3  7 21,   4  2 21,   4 13 18,
                5-12  7,   5 11 21,   6 14 16,
PLAT967_ALERT_5_G Note: Two-Theta Cutoff Value in Embedded .res ..      114.0 Degree
PLAT969_ALERT_5_G The 'Henn et al.' R-Factor-gap value ..........      3.589 Note
              Predicted wR2: Based on SigI**2  4.34 or SHELX Weight 13.99
PLAT978_ALERT_2_G Number C-C Bonds with Positive Residual Density.          1 Info
```

```
  0 ALERT level A = Most likely a serious problem - resolve or explain
  1 ALERT level B = A potentially serious problem, consider carefully
  7 ALERT level C = Check. Ensure it is not caused by an omission or oversight
 14 ALERT level G = General information/check it is not something unexpected

  3 ALERT type 1 CIF construction/syntax error, inconsistent or missing data
  7 ALERT type 2 Indicator that the structure model may be wrong or deficient
  7 ALERT type 3 Indicator that the structure quality may be low
  3 ALERT type 4 Improvement, methodology, query or suggestion
  2 ALERT type 5 Informative message, check
```

It is advisable to attempt to resolve as many as possible of the alerts in all categories. Often the minor alerts point to easily fixed oversights, errors and omissions in your CIF or refinement strategy, so attention to these fine details can be worthwhile. In order to resolve some of the more serious problems it may be necessary to carry out additional measurements or structure refinements. However, the purpose of your study may justify the reported deviations and the more serious of these should normally be commented upon in the discussion or experimental section of a paper or in the "special_details" fields of the CIF. checkCIF was carefully designed to identify outliers and unusual parameters, but every test has its limitations and alerts that are not important in a particular case may appear. Conversely, the absence of alerts does not guarantee there are no aspects of the results needing attention. It is up to the individual to critically assess their own results and, if necessary, seek expert advice.

Publication of your CIF in IUCr journals

A basic structural check has been run on your CIF. These basic checks will be run on all CIFs submitted for publication in IUCr journals (*Acta Crystallographica*, *Journal of Applied Crystallography*, *Journal of Synchrotron Radiation*); however, if you intend to submit to *Acta Crystallographica Section C* or *E* or *IUCrData*, you should make sure that full publication checks are run on the final version of your CIF prior to submission.

Publication of your CIF in other journals

Please refer to the *Notes for Authors* of the relevant journal for any special instructions relating to CIF submission.

PLATON version of 02/02/2025; check.def file version of 02/02/2025

Datablock sgk19_0m - ellipsoid plot

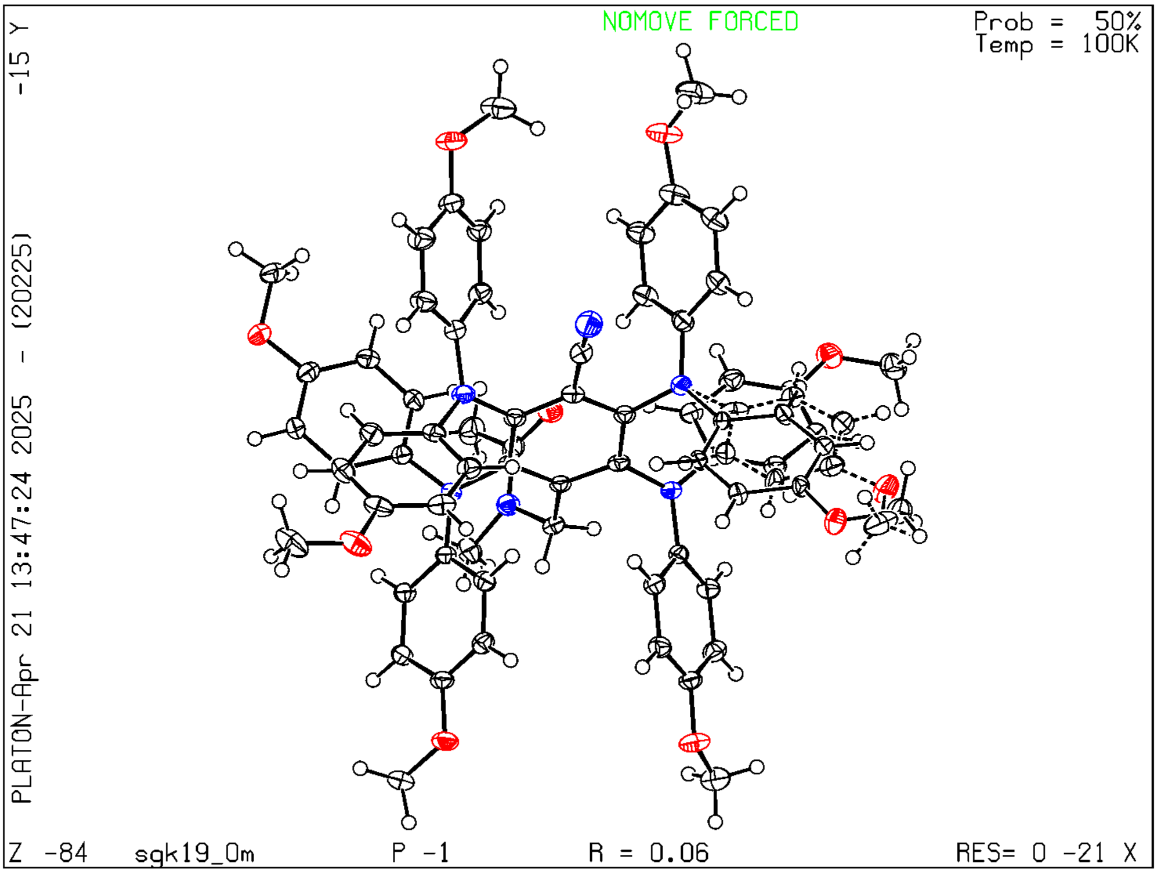

Supplement: Supplementary file 2 — Supporting Information [file 2370570.pdf › 2370570.pdf]
